# Supplementary material for: A Time-Course Study of the Expression Level of Synaptic Plasticity-Associated Genes in Un-Lesioned Spinal Cord and Brain Areas in a Rat Model of Spinal Cord Injury: A Bioinformatic Approach
Source: Int J Mol Sci. 2021 Aug 10;22(16):8606. doi: 10.3390/ijms22168606 (PMC8395345; doi:10.3390/ijms22168606)
Supplement: Supplementary file 1 [file ijms-22-08606-s001.zip › ijms-1289563-supplementary.pdf]

Supplementary Materials:

A Time-Course Study of the Expression Level of Synaptic Plasticity-Associated Genes in Un-Lesioned Spinal Cord and Brain Areas in a Rat Model of Spinal Cord Injury: A Bioinformatic Approach.

Baldassaro V.A.[1, 2], Sanna M.[3, 2],
Bighinati A.[1], Sannia M.[3], Gusciglio M.[4], Giardino L.[1, 3, 4],
Lorenzini L.[1, 3, 5], Calzà L.[3, 6, 7, 5]

Version Typeset August 2021

[1]Department of Veterinary Medical Science, University of Bologna, 40064 Ozzano Emilia, Bologna, Italy
[2]These authors equally contributed to the study
[3]Interdepartmental Center for Industrial Research in Life Sciences and Technologies, University of Bologna, 40064 Ozzano Emilia, Bologna, Italy
[4]IRET Foundation, 40064 Ozzano Emilia, Bologna, Italy
[5]These authors share senior authorship
[6]Montecatone Rehabilitation Institute, 40026 Imola, Bologna, Italy
[7]Department of Pharmacy and BioTechnology, University of Bologna, 40127 Bologna, Italy

Contents

1 Supplementary Tables **3**
1.1 RT^2 Profiler PCR Array . . . 3
1.2 GSEA Results Tables . . . 6

2 Supplementary Figures **310**
2.1 Pearson Correlation Plots . . . 310
2.2 NES vs Significance Plots . . . 312
2.3 Leading Edge Analysis . . . 315
2.4 Deregulated Pathways EN Plots and Heatmaps . . . 317
2.4.1 SC rostral 1 DPL Peak Pathways . . . 317
2.4.2 SC rostral 8 DPL Peak Pathways . . . 321
2.4.3 SC rostral 45 DPL Peak Pathways . . . 322
2.4.4 SC caudal 1 DPL Peak Pathways . . . 327
2.4.5 SC caudal 8 DPL Peak Pathways . . . 331
2.4.6 CTX-M 1 DPL Peak Pathways . . . 336
2.4.7 CTX-M 8 DPL Peak Pathways . . . 340
2.4.8 BG 45 DPL Peak Pathways . . . 342
2.4.9 CB 8 DPL Pathways . . . 344
2.4.10 CB 45 DPL Peak Pathways . . . 345

1 Supplementary Tables

1.1 RT^2 Profiler PCR Array

Supplementary Table S1: PARN-126ZA Genes Array Table.

	Position	Unigene	Refseq	Symbol	Description
0	A01	Rn.42924	NM_019254	Adam10	ADAM metallopeptidase domain 10
1	A02	Rn.214145	NM_001107239	Adcy1	Adenylate cyclase 1 (brain)
2	A03	Rn.10382	NM_017142	Adcy8	Adenylate cyclase 8 (brain)
3	A04	Rn.11422	NM_033230	Akt1	V-akt murine thymoma viral oncogene homolog 1
4	A05	Rn.10086	NM_019361	Arc	Activity-regulated cytoskeleton-associated pro...
5	A06	Rn.11266	NM_012513	Bdnf	Brain-derived neurotrophic factor
6	A07	Rn.107499	NM_012920	Camk2a	Calcium/calmodulin-dependent protein kinase II...
7	A08	Rn.10961	NM_133605	Camk2g	Calcium/calmodulin-dependent protein kinase II...
8	A09	Rn.23200	NM_031333	Cdh2	Cadherin 2
9	A10	Rn.6479	NM_024125	Cebpb	CCAAT/enhancer binding protein (C/EBP), beta
10	A11	Rn.6975	NM_013154	Cebpd	CCAAT/enhancer binding protein (C/EBP), delta
11	A12	Rn.89774	NM_012784	Cnr1	Cannabinoid receptor 1 (brain)
12	B01	Rn.90061	NM_031017	Creb1	CAMP responsive element binding protein 1
13	B02	Rn.10251	NM_001110860	Crem	CAMP responsive element modulator
14	B03	Rn.9765	NM_019621	Dlg4	Discs, large homolog 4 (Drosophila)
15	B04	Rn.9096	NM_012551	Egr1	Early growth response 1
16	B05	Rn.89235	NM_053633	Egr2	Early growth response 2
17	B06	Rn.44371	NM_017086	Egr3	Early growth response 3
18	B07	Rn.31998	NM_019137	Egr4	Early growth response 4
19	B08	Rn.229866	NM_001127319	Ephb2	Eph receptor B2
20	B09	Rn.103750	NM_022197	Fos	FBJ osteosarcoma oncogene
21	B10	Rn.10368	NM_017295	Gabra5	Gamma-aminobutyric acid (GABA) A receptor, alp...
22	B11	Rn.11391	NM_013145	Gnai1	Guanine nucleotide binding protein (G protein)...
23	B12	Rn.29971	NM_031608	Gria1	Glutamate receptor, ionotropic, AMPA 1
24	C01	Rn.91361	NM_017261	Gria2	Glutamate receptor, ionotropic, AMPA 2
25	C02	Rn.74049	NM_032990	Gria3	Glutamate receptor, ionotrophic, AMPA 3
26	C03	Rn.10938	NM_017263	Gria4	Glutamate receptor, ionotrophic, AMPA 4
27	C04	Rn.9840	NM_017010	Grin1	Glutamate receptor, ionotropic, N-methyl D-asp...
28	C05	Rn.9710	NM_012573	Grin2a	Glutamate receptor, ionotropic, N-methyl D-asp...
29	C06	Rn.9711	NM_012574	Grin2b	Glutamate receptor, ionotropic, N-methyl D-asp...
30	C07	Rn.9709	NM_012575	Grin2c	Glutamate receptor, ionotropic, N-methyl D-asp...
31	C08	Rn.91209	NM_022797	Grin2d	Glutamate receptor, ionotropic, N-methyl D-asp...

Continuation of Table **S1**

	Position	Unigene	Refseq	Symbol	Description
32	C09	Rn.74240	NM_032069	Grip1	Glutamate receptor interacting protein 1
33	C10	Rn.87787	NM_017011	Grm1	Glutamate receptor, metabotropic 1
34	C11	Rn.9681	NM_001105711	Grm2	Glutamate receptor, metabotropic 2
35	C12	Rn.41715	NM_001105712	Grm3	Glutamate receptor, metabotropic 3
36	D01	Rn.89046	NM_022666	Grm4	Glutamate receptor, metabotropic 4
37	D02	Rn.29972	NM_017012	Grm5	Glutamate receptor, metabotropic 5
38	D03	Rn.10409	NM_031040	Grm7	Glutamate receptor, metabotropic 7
39	D04	Rn.44420	NM_022202	Grm8	Glutamate receptor, metabotropic 8
40	D05	Rn.37500	NM_031707	Homer1	Homer homolog 1 (Drosophila)
41	D06	Rn.228346	NM_178866	Igf1	Insulin-like growth factor 1
42	D07	Rn.9874	NM_017128	Inhba	Inhibin beta-A
43	D08	Rn.93714	NM_021835	Jun	Jun oncogene
44	D09	Rn.15806	NM_021836	Junb	Jun B proto-oncogene
45	D10	Rn.2398	NM_031135	Klf10	Kruppel-like factor 10
46	D11	Rn.34914	NM_053842	Mapk1	Mitogen activated protein kinase 1
47	D12	Rn.10209	NM_031055	Mmp9	Matrix metallopeptidase 9
48	E01	Rn.11283	NM_031521	Ncam1	Neural cell adhesion molecule 1
49	E02	Rn.2411	NM_001276711	Nfkb1	Nuclear factor of kappa light polypeptide gene...
50	E03	Rn.8395	NM_030867	Nfkbib	Nuclear factor of kappa light polypeptide gene...
51	E04	Rn.22168	NM_001277055	Ngf	Nerve growth factor (beta polypeptide)
52	E05	Rn.10980	NM_012610	Ngfr	Nerve growth factor receptor (TNFR superfamily...
53	E06	Rn.10573	NM_052799	Nos1	Nitric oxide synthase 1, neuronal
54	E07	Rn.162101	NM_001034199	Nptx2	Neuronal pentraxin 2
55	E08	Rn.10000	NM_024388	Nr4a1	Nuclear receptor subfamily 4, group A, member 1
56	E09	Rn.9715	NM_031073	Ntf3	Neurotrophin 3
57	E10	Rn.44225	NM_013184	Ntf4	Neurotrophin 4
58	E11	Rn.11246	NM_012731	Ntrk2	Neurotrophic tyrosine kinase, receptor, type 2
59	E12	Rn.23337	NM_022868	Pcdh8	Protocadherin 8
60	F01	Rn.24750	NM_053460	Pick1	Protein interacting with PRKCA 1
61	F02	Rn.34888	NM_017034	Pim1	Pim-1 oncogene
62	F03	Rn.107102	NM_013151	Plat	Plasminogen activator, tissue
63	F04	Rn.11243	NM_013187	Plcg1	Phospholipase C, gamma 1
64	F05	Rn.2024	NM_031527	Ppp1ca	Protein phosphatase 1, catalytic subunit, alph...
65	F06	Rn.1495	NM_022498	Ppp1cc	Protein phosphatase 1, catalytic subunit, gamm...
66	F07	Rn.73852	NM_130403	Ppp1r14a	Protein phosphatase 1, regulatory (inhibitor) ...
67	F08	Rn.1271	NM_017039	Ppp2ca	Protein phosphatase 2, catalytic subunit, alph...
68	F09	Rn.6866	NM_017041	Ppp3ca	Protein phosphatase 3, catalytic subunit, alph...

Continuation of Table **S1**

	Position	Unigene	Refseq	Symbol	Description
69	F10	Rn.86669	NM_001105713	Prkca	Protein kinase C, alpha
70	F11	Rn.9747	NM_012628	Prkcg	Protein kinase C, gamma
71	F12	Rn.204724	NM_001105731	Prkg1	Protein kinase, cGMP-dependent, type 1
72	G01	Rn.44409	NM_013018	Rab3a	RAB3A, member RAS oncogene family
73	G02	Rn.19480	NM_199267	Rela	V-rel reticuloendotheliosis viral oncogene hom...
74	G03	Rn.98353	NM_080394	Reln	Reelin
75	G04	NaN	XM_003750065	Kif17	Kinesin family member 17
76	G05	Rn.1892	NM_053453	Rgs2	Regulator of G-protein signaling 2
77	G06	Rn.859	NM_013216	Rheb	Ras homolog enriched in brain
78	G07	NaN	NM_001107627	Sirt1	Sirtuin (silent mating type information regula...
79	G08	Rn.1501	NM_001109302	Srf	Serum response factor (c-fos serum response el...
80	G09	Rn.42910	NM_021695	Synpo	Synaptopodin
81	G10	Rn.25754	NM_053819	Timp1	TIMP metallopeptidase inhibitor 1
82	G11	Rn.2275	NM_012675	Tnf	Tumor necrosis factor (TNF superfamily, member 2)
83	G12	Rn.2502	NM_013053	Ywhaq	Tyrosine 3-monooxygenase/tryptophan 5-monooxyg...
84	H01	Rn.94978	NM_031144	Actb	Actin, beta
85	H02	Rn.1868	NM_012512	B2m	Beta-2 microglobulin
86	H03	Rn.47	NM_012583	Hprt1	Hypoxanthine phosphoribosyltransferase 1
87	H04	Rn.107896	NM_017025	Ldha	Lactate dehydrogenase A
88	H05	Rn.973	NM_001007604	Rplp1	Ribosomal protein, large, P1
89	H06	NaN	U26919	RGDC	Rat Genomic DNA Contamination
90	H07	NaN	SA_00104	RTC	Reverse Transcription Control
91	H08	NaN	SA_00104	RTC	Reverse Transcription Control
92	H09	NaN	SA_00104	RTC	Reverse Transcription Control
93	H10	NaN	SA_00103	PPC	Positive PCR Control
94	H11	NaN	SA_00103	PPC	Positive PCR Control
95	H12	NaN	SA_00103	PPC	Positive PCR Control

1.2 GSEA Results Tables

Supplementary Table S2: SC rostral segment early (1 DPL peak) profile GSEA results.

Begin of Table **S2**

	NAME	ID	SIZE	ES	NES	NOM.p.val	FDR.q.val	FWER.p.val
0	REACTOME_INTERLEUKIN_4_AND_INTERLEUKIN_13_SIGN...	R-RNO-6785807	8	0.922751	2.960325	0.000000	0.001343	0.001
1	REACTOME_SIGNALING_BY_INTERLEUKINS	R-RNO-449147	15	0.590450	2.482950	0.000000	0.022870	0.036
2	HALLMARK_TNFA_SIGNALING_VIA_NFKB	M5890	14	0.591473	2.428519	0.000000	0.020713	0.048
3	GOBP_NEGATIVE_REGULATION_OF_PEPTIDASE_ACTIVITY	GO:0010466	6	0.832010	2.357697	0.000000	0.026939	0.082
4	GOBP_OSTEOCLAST_DIFFERENTIATION	GO:0030316	6	0.807556	2.354578	0.000000	0.021834	0.082
5	REACTOME_TRANSCRIPTIONAL_REGULATION_OF_WHITE_A...	R-RNO-381340	6	0.807932	2.300164	0.000000	0.027823	0.125
6	GOBP_OSSIFICATION	GO:0001503	9	0.624718	2.155755	0.003165	0.069799	0.320
7	GOBP_NEGATIVE_REGULATION_OF_CYSTEINE_TYPE_ENDO...	GO:2000117	5	0.793688	2.100962	0.002740	0.090920	0.439
8	GOBP_MYELOID_LEUKOCYTE_DIFFERENTIATION	GO:0002573	11	0.594451	2.088907	0.003676	0.086556	0.455
9	GOBP_MYELOID_CELL_DIFFERENTIATION	GO:0030099	13	0.502879	2.049668	0.000000	0.097413	0.537
10	GOBP_CYTOKINE_MEDIATED_SIGNALING_PATHWAY	GO:0019221	16	0.477029	2.027812	0.000000	0.101125	0.582
11	GOBP_REGULATION_OF_OSTEOCLAST_DIFFERENTIATION	GO:0045670	5	0.768624	1.968335	0.011869	0.129783	0.705
12	GOBP_REGULATION_OF_MYELOID_LEUKOCYTE_DIFFERENT...	GO:0002761	8	0.575237	1.918722	0.003067	0.159148	0.815
13	GOBP_SKIN_DEVELOPMENT	GO:0043588	5	0.734940	1.912148	0.011905	0.152929	0.824
14	GOBP_RESPONSE_TO_MECHANICAL_STIMULUS	GO:0009612	6	0.682927	1.906121	0.006329	0.146871	0.829
15	REACTOME_DEATH_RECEPTOR_SIGNALLING	R-RNO-73887	5	0.737565	1.901219	0.017143	0.141962	0.840
16	GOBP_RESPONSE_TO_ENDOGENOUS_STIMULUS	GO:0009719	34	0.344841	1.889355	0.000000	0.142666	0.855
17	GOBP_POSITIVE_REGULATION_OF_CELL_DIFFERENTIATION	GO:0045597	27	0.368842	1.875748	0.000000	0.145758	0.875
18	GOBP_NEGATIVE_REGULATION_OF_CELL_POPULATION_PR...	GO:0008285	9	0.534148	1.871449	0.007042	0.141389	0.879
19	GOBP_POSITIVE_REGULATION_OF_DEVELOPMENTAL_PROCESS	GO:0051094	32	0.350193	1.864965	0.000000	0.139355	0.882
20	GOBP_IMMUNE_SYSTEM_DEVELOPMENT	GO:0002520	17	0.432172	1.854994	0.004219	0.139355	0.894
21	GOBP_REGULATION_OF_CELL_DIFFERENTIATION	GO:0045595	36	0.329660	1.825157	0.000000	0.156730	0.930
22	GOBP_RESPONSE_TO_CYTOKINE	GO:0034097	23	0.378370	1.813332	0.004484	0.161851	0.948
23	REACTOME_CYTOKINE_SIGNALING_IN_IMMUNE_SYSTEM	R-RNO-1280215	21	0.379680	1.812935	0.012195	0.155586	0.948
24	GOBP_INFLAMMATORY_RESPONSE	GO:0006954	12	0.471001	1.808953	0.018939	0.151975	0.950
25	GOBP_NEGATIVE_REGULATION_OF_PROTEOLYSIS	GO:0045861	8	0.548667	1.755278	0.028269	0.194117	0.983
26	GOBP_LEUKOCYTE_DIFFERENTIATION	GO:0002521	15	0.423575	1.754041	0.003953	0.188277	0.984
27	REACTOME_NGF_STIMULATED_TRANSCRIPTION	R-RNO-9031628	9	0.518987	1.738173	0.023649	0.197365	0.992
28	GOBP_OSTEOBLAST_DIFFERENTIATION	GO:0001649	5	0.664689	1.726725	0.011050	0.201508	0.993
29	GOBP_CELL_POPULATION_PROLIFERATION	GO:0008283	32	0.316965	1.716461	0.005464	0.205741	0.993
30	GOBP_REGULATION_OF_LEUKOCYTE_DIFFERENTIATION	GO:1902105	9	0.491450	1.685974	0.036545	0.233448	1.000
31	GOBP_REGULATION_OF_MYELOID_CELL_DIFFERENTIATION	GO:0045637	9	0.488989	1.680686	0.032609	0.232190	1.000
32	HALLMARK_EPITHELIAL_MESENCHYMAL_TRANSITION	M5930	5	0.650602	1.679643	0.021277	0.226711	1.000
33	GOBP_PRI_MIRNA_TRANSCRIPTION_BY_RNA_POLYMERASE_II	GO:0061614	7	0.543210	1.663227	0.036585	0.238343	1.000
34	GOBP_POSITIVE_REGULATION_OF_BIOSYNTHETIC_PROCESS	GO:0009891	27	0.323689	1.642793	0.017094	0.256950	1.000
35	GOBP_POSITIVE_REGULATION_OF_NUCLEOBASE_CONTAIN...	GO:0045935	28	0.318819	1.635624	0.015000	0.258728	1.000
36	GOBP_POSITIVE_REGULATION_OF_PRI_MIRNA_TRANSCRI...	GO:1902895	7	0.543210	1.631699	0.024390	0.257242	1.000
37	GOBP_ACTIN_FILAMENT_BASED_PROCESS	GO:0030029	7	0.541508	1.617816	0.022876	0.266744	1.000
38	GOBP_REGULATION_OF_DEFENSE_RESPONSE	GO:0031347	7	0.509083	1.574683	0.042980	0.318328	1.000
39	HALLMARK_HYPOXIA	M5891	5	0.612647	1.565726	0.056886	0.324307	1.000
40	GOBP_NEGATIVE_REGULATION_OF_CELL_DEATH	GO:0060548	22	0.331603	1.557555	0.035176	0.328949	1.000

Continuation of Table **S2**

	NAME	ID	SIZE	ES	NES	NOM.p.val	FDR.q.val	FWER.p.val
41	GOBP_INTRINSIC_APOPTOTIC_SIGNALING_PATHWAY	GO:0097193	5	0.595854	1.543189	0.045872	0.343915	1.000
42	GOBP_POSITIVE_REGULATION_OF_AXONOGENESIS	GO:0050772	5	0.602410	1.536202	0.065527	0.346841	1.000
43	GOBP_REGULATION_OF_HEMOPOIESIS	GO:1903706	10	0.424029	1.535530	0.052117	0.340095	1.000
44	GOBP_MULTI_MULTICELLULAR_ORGANISM_PROCESS	GO:0044706	8	0.471437	1.531031	0.043210	0.339680	1.000
45	GOBP_REGULATION_OF_CELL_SIZE	GO:0008361	5	0.590361	1.530751	0.054795	0.332738	1.000
46	GOBP_POSITIVE_REGULATION_OF_TRANSCRIPTION_BY_R...	GO:0045944	24	0.310715	1.511869	0.030973	0.355268	1.000
47	GOBP_NEURON_APOPTOTIC_PROCESS	GO:0051402	12	0.394737	1.490223	0.060000	0.383582	1.000
48	GOBP_REGULATION_OF_INFLAMMATORY_RESPONSE	GO:0050727	7	0.509083	1.488370	0.084112	0.378639	1.000
49	GOBP_REGULATION_OF_CELL_DEATH	GO:0010941	34	0.276107	1.488344	0.031963	0.371139	1.000
50	GOBP_POSITIVE_REGULATION_OF_MYELOID_CELL_DIFFE...	GO:0045639	7	0.490488	1.482751	0.070968	0.372727	1.000
51	GOBP_POSITIVE_REGULATION_OF_SMOOTH_MUSCLE_CELL...	GO:0048661	5	0.566265	1.474395	0.081159	0.379650	1.000
52	HALLMARK_ALLOGRAFT_REJECTION	M5950	7	0.497605	1.468273	0.069079	0.383234	1.000
53	GOBP_POSITIVE_REGULATION_OF_PEPTIDYL_TYROSINE_...	GO:0050731	8	0.462500	1.465458	0.080645	0.381109	1.000
54	GOBP_POSITIVE_REGULATION_OF_CYTOKINE_PRODUCTION	GO:0001819	7	0.481481	1.459276	0.066667	0.383954	1.000
55	GOBP_MONONUCLEAR_CELL_DIFFERENTIATION	GO:1903131	7	0.481482	1.454565	0.085044	0.384875	1.000
56	GOBP_REGULATION_OF_FAT_CELL_DIFFERENTIATION	GO:0045598	6	0.498263	1.425424	0.111765	0.429799	1.000
57	GOBP_LEUKOCYTE_CELL_CELL_ADHESION	GO:0007159	6	0.500000	1.408764	0.090634	0.454834	1.000
58	GOBP_RESPONSE_TO_NUTRIENT	GO:0007584	5	0.536494	1.405000	0.139535	0.454337	1.000
59	GOBP_EPITHELIAL_CELL_DIFFERENTIATION	GO:0030855	7	0.453790	1.400421	0.115385	0.455335	1.000
60	GOBP_POSITIVE_REGULATION_OF_MYELOID_LEUKOCYTE_...	GO:0002763	6	0.503944	1.393833	0.120370	0.460239	1.000
61	GOBP_RESPONSE_TO_REACTIVE_OXYGEN_SPECIES	GO:0000302	8	0.423541	1.392840	0.101045	0.454990	1.000
62	GOBP_REGULATION_OF_PEPTIDYL_SERINE_PHOSPHORYLA...	GO:0033135	8	0.434007	1.371879	0.110769	0.488968	1.000
63	GOBP_POSITIVE_REGULATION_OF_PEPTIDYL_SERINE_PH...	GO:0033138	8	0.434007	1.369715	0.125767	0.486112	1.000
64	GOBP_EMBRYONIC_ORGAN_DEVELOPMENT	GO:0048568	7	0.466098	1.369663	0.132911	0.478670	1.000
65	REACTOME_CELLULAR_SENESCENCE	R-RNO-2559583	6	0.482887	1.369502	0.125000	0.471748	1.000
66	GOBP_RESPONSE_TO_OXIDATIVE_STRESS	GO:0006979	8	0.423541	1.360988	0.102564	0.482503	1.000
67	GOBP_RESPONSE_TO_EXTRACELLULAR_STIMULUS	GO:0009991	13	0.339525	1.358283	0.093525	0.480482	1.000
68	GOBP_EMBRYO_DEVELOPMENT	GO:0009790	14	0.334556	1.356936	0.106061	0.476183	1.000
69	GOBP_POSITIVE_REGULATION_OF_DEFENSE_RESPONSE	GO:0031349	5	0.523801	1.356082	0.119403	0.471085	1.000
70	GOBP_CELLULAR_RESPONSE_TO_EXTERNAL_STIMULUS	GO:0071496	10	0.384817	1.346553	0.131673	0.482679	1.000
71	GOBP_NEGATIVE_REGULATION_OF_TRANSCRIPTION_BY_R...	GO:0000122	10	0.387147	1.345786	0.136364	0.477689	1.000
72	REACTOME_SENESCENCE_ASSOCIATED_SECRETORY_PHENO...	R-RNO-2559582	6	0.482887	1.335211	0.145161	0.490903	1.000
73	GOBP_RHYTHMIC_PROCESS	GO:0048511	15	0.322410	1.332421	0.120536	0.489551	1.000
74	GOBP_NEGATIVE_REGULATION_OF_IMMUNE_SYSTEM_PROCESS	GO:0002683	5	0.501871	1.329278	0.147929	0.488887	1.000
75	GOBP_RESPONSE_TO_UV	GO:0009411	5	0.511987	1.326654	0.165680	0.487851	1.000
76	GOBP_PERIPHERAL_NERVOUS_SYSTEM_DEVELOPMENT	GO:0007422	9	0.405063	1.320125	0.127517	0.494279	1.000
77	GOBP_NEGATIVE_REGULATION_OF_HYDROLASE_ACTIVITY	GO:0051346	11	0.348567	1.317281	0.115672	0.493901	1.000
78	GOBP_TISSUE_MORPHOGENESIS	GO:0048729	7	0.431526	1.317193	0.161491	0.487898	1.000
79	GOBP_POSITIVE_REGULATION_OF_CELL_CELL_ADHESION	GO:0022409	5	0.493976	1.309491	0.140056	0.496777	1.000
80	GOBP_NERVE_DEVELOPMENT	GO:0021675	9	0.392405	1.304619	0.154605	0.500199	1.000
81	GOBP_REGULATION_OF_PEPTIDASE_ACTIVITY	GO:0052547	10	0.364257	1.298780	0.142415	0.506125	1.000
82	GOBP_NEGATIVE_REGULATION_OF_LOCOMOTION	GO:0040013	5	0.496870	1.292414	0.174286	0.512766	1.000
83	GOBP_CELLULAR_RESPONSE_TO_EXTRACELLULAR_STIMULUS	GO:0031668	8	0.405901	1.292190	0.160883	0.506961	1.000

Continuation of Table **S2**

	NAME	ID	SIZE	ES	NES	NOM.p.val	FDR.q.val	FWER.p.val
84	GOBP_POSITIVE_REGULATION_OF_LEUKOCYTE_CELL_CEL...	GO:1903039	5	0.493976	1.290738	0.183976	0.503834	1.000
85	GOBP_NEGATIVE_REGULATION_OF_NEURON_APOPTOTIC_P...	GO:0043524	8	0.387500	1.288778	0.151515	0.502360	1.000
86	GOBP_REPRODUCTIVE_SYSTEM_DEVELOPMENT	GO:0061458	6	0.450016	1.273961	0.166667	0.526424	1.000
87	GOBP_REGULATION_OF_MULTICELLULAR_ORGANISMAL_DE...	GO:2000026	31	0.233392	1.270299	0.135678	0.528107	1.000
88	GOBP_POSITIVE_REGULATION_OF_TRANSMEMBRANE_TRAN...	GO:0034764	7	0.432099	1.264628	0.179191	0.533287	1.000
89	GOBP_REGULATION_OF_AXONOGENESIS	GO:0050770	7	0.432099	1.261935	0.147059	0.533245	1.000
90	GOBP_MEMBRANE_PROTEIN_PROTEOLYSIS	GO:0033619	6	0.455071	1.258937	0.179331	0.533337	1.000
91	GOBP_REGULATION_OF_LYMPHOCYTE_ACTIVATION	GO:0051249	5	0.493976	1.258292	0.189441	0.529065	1.000
92	GOBP_POSITIVE_REGULATION_OF_HEMOPOIESIS	GO:1903708	7	0.412196	1.251139	0.179487	0.538978	1.000
93	GOBP_DEFENSE_RESPONSE	GO:0006952	19	0.268609	1.228177	0.200913	0.581761	1.000
94	GOBP_PROTEIN_AUTOPHOSPHORYLATION	GO:0046777	5	0.457831	1.225862	0.204748	0.580963	1.000
95	GOBP_HOMEOSTASIS_OF_NUMBER_OF_CELLS	GO:0048872	5	0.469880	1.215091	0.229508	0.597441	1.000
96	HALLMARK_UV_RESPONSE_UP	M5941	5	0.474630	1.214075	0.218182	0.593256	1.000
97	GOBP_POSITIVE_REGULATION_OF_DEVELOPMENTAL_GROWTH	GO:0048639	8	0.382169	1.211029	0.229102	0.594144	1.000
98	GOBP_CELLULAR_RESPONSE_TO_CADMIUM_ION	GO:0071276	5	0.470236	1.209200	0.219585	0.591583	1.000
99	GOBP_EPITHELIUM_DEVELOPMENT	GO:0060429	15	0.286735	1.201919	0.198606	0.601240	1.000
100	GOBP_APOPTOTIC_SIGNALING_PATHWAY	GO:0097190	15	0.291399	1.199184	0.215909	0.600855	1.000
101	GOBP_RESPONSE_TO_NERVE_GROWTH_FACTOR	GO:1990089	7	0.395062	1.195291	0.246875	0.604257	1.000
102	GOBP_POSITIVE_REGULATION_OF_PROTEIN_TYROSINE_K...	GO:0061098	5	0.445783	1.164471	0.245714	0.669243	1.000
103	GOBP_MUSCLE_CELL_PROLIFERATION	GO:0033002	8	0.358376	1.159048	0.273312	0.677999	1.000
104	GOBP_RESPONSE_TO_OXYGEN_CONTAINING_COMPOUND	GO:1901700	39	0.204577	1.151828	0.228723	0.688505	1.000
105	GOBP_REGULATION_OF_RECEPTOR_MEDIATED_ENDOCYTOSIS	GO:0048259	5	0.445783	1.150438	0.277620	0.685419	1.000
106	GOBP_MORPHOGENESIS_OF_AN_EPITHELIUM	GO:0002009	6	0.417965	1.145050	0.286585	0.692153	1.000
107	GOBP_POSITIVE_REGULATION_OF_NERVOUS_SYSTEM_DEV...	GO:0051962	12	0.294021	1.139077	0.275735	0.700049	1.000
108	GOBP_APOPTOTIC_PROCESS	GO:0006915	35	0.207530	1.134091	0.262136	0.706272	1.000
109	GOBP_REGULATION_OF_PROTEIN_TYROSINE_KINASE_ACT...	GO:0061097	5	0.445783	1.132447	0.287791	0.703785	1.000
110	GOBP_RECEPTOR_MEDIATED_ENDOCYTOSIS	GO:0006898	6	0.402439	1.130893	0.274510	0.701172	1.000
111	GOBP_REGIONALIZATION	GO:0003002	6	0.390244	1.129050	0.285276	0.699698	1.000
112	GOBP_ANIMAL_ORGAN_MORPHOGENESIS	GO:0009887	12	0.283743	1.123749	0.271429	0.706669	1.000
113	GOBP_REGULATION_OF_CELLULAR_COMPONENT_SIZE	GO:0032535	7	0.367891	1.122414	0.277419	0.704135	1.000
114	GOBP_GLAND_DEVELOPMENT	GO:0048732	7	0.372799	1.119691	0.287926	0.705097	1.000
115	GOBP_RESPONSE_TO_GROWTH_FACTOR	GO:0070848	18	0.242386	1.117483	0.288793	0.704157	1.000
116	GOBP_CYTOKINE_PRODUCTION	GO:0001816	10	0.316989	1.115137	0.310105	0.704038	1.000
117	GOBP_NEUROTROPHIN_SIGNALING_PATHWAY	GO:0038179	6	0.390244	1.110732	0.323625	0.708926	1.000
118	GOBP_SPROUTING_ANGIOGENESIS	GO:0002040	5	0.433735	1.110017	0.298551	0.704725	1.000
119	GOBP_NEURON_DEATH	GO:0070997	18	0.244104	1.105382	0.292079	0.710156	1.000
120	GOBP_POSITIVE_REGULATION_OF_CELL_DEVELOPMENT	GO:0010720	12	0.294021	1.104732	0.316406	0.706032	1.000
121	GOBP_POSITIVE_REGULATION_OF_GENE_EXPRESSION	GO:0010628	16	0.253048	1.103683	0.297297	0.702819	1.000
122	GOBP_REPRODUCTION	GO:0000003	18	0.249701	1.102133	0.311403	0.700433	1.000
123	GOBP_RAS_PROTEIN_SIGNAL_TRANSDUCTION	GO:0007265	5	0.421687	1.100958	0.320856	0.697892	1.000
124	GOBP_NEUROTROPHIN_TRK_RECEPTOR_SIGNALING_PATHWAY	GO:0048011	6	0.390244	1.100617	0.372781	0.693060	1.000
125	GOBP_POSITIVE_REGULATION_OF_CELL_DEATH	GO:0010942	19	0.238123	1.099045	0.275000	0.691025	1.000
126	GOBP_POSITIVE_REGULATION_OF_CATION_TRANSMEMBRA...	GO:1904064	5	0.421687	1.094632	0.326870	0.695733	1.000

Continuation of Table **S2**

	NAME	ID	SIZE	ES	NES	NOM.p.val	FDR.q.val	FWER.p.val
127	GOBP_PATTERN_SPECIFICATION_PROCESS	GO:0007389	6	0.390244	1.093824	0.308176	0.692185	1.000
128	GOBP_POSITIVE_REGULATION_OF_NEUROGENESIS	GO:0050769	12	0.294021	1.081507	0.371025	0.716750	1.000
129	GOBP_CELL_CELL_JUNCTION_ASSEMBLY	GO:0007043	5	0.420669	1.078416	0.364742	0.718598	1.000
130	GOBP_RESPONSE_TO_ORGANIC_CYCLIC_COMPOUND	GO:0014070	20	0.233652	1.075103	0.328302	0.720624	1.000
131	GOBP_EMBRYO_DEVELOPMENT_ENDING_IN_BIRTH_OR_EGG...	GO:0009792	9	0.312711	1.073013	0.358209	0.720005	1.000
132	GOBP_AMEBOIDAL_TYPE_CELL_MIGRATION	GO:0001667	11	0.292994	1.070459	0.350000	0.720276	1.000
133	GOBP_POSITIVE_REGULATION_OF_MULTICELLULAR_ORGA...	GO:0051240	32	0.206577	1.069789	0.352381	0.716592	1.000
134	GOBP_IN_UTERO_EMBRYONIC_DEVELOPMENT	GO:0001701	8	0.327652	1.067714	0.334356	0.716376	1.000
135	GOBP_CELLULAR_RESPONSE_TO_OXYGEN_CONTAINING_CO...	GO:1901701	29	0.204257	1.061491	0.378238	0.726085	1.000
136	GOBP_FAT_CELL_DIFFERENTIATION	GO:0045444	9	0.312171	1.054375	0.348387	0.737559	1.000
137	GOBP_EXTRINSIC_APOPTOTIC_SIGNALING_PATHWAY	GO:0097191	7	0.350570	1.053130	0.346154	0.734964	1.000
138	GOBP_DEFENSE_RESPONSE_TO_OTHER_ORGANISM	GO:0098542	9	0.303833	1.046014	0.382838	0.746608	1.000
139	GOBP_REGULATION_OF_CIRCADIAN_RHYTHM	GO:0042752	7	0.339959	1.044631	0.362805	0.744492	1.000
140	GOBP_REGULATION_OF_PROTEOLYSIS	GO:0030162	12	0.280405	1.040987	0.380952	0.747798	1.000
141	GOBP_CELLULAR_COMPONENT_DISASSEMBLY	GO:0022411	6	0.373501	1.038960	0.394659	0.746773	1.000
142	GOBP_RESPONSE_TO_OXYGEN_LEVELS	GO:0070482	8	0.332405	1.034402	0.416938	0.752905	1.000
143	GOBP_RECEPTOR_METABOLIC_PROCESS	GO:0043112	5	0.397590	1.030348	0.413295	0.757794	1.000
144	GOBP_PEPTIDYL_TYROSINE_MODIFICATION	GO:0018212	10	0.284546	1.030200	0.369403	0.752961	1.000
145	GOBP_TUBE_MORPHOGENESIS	GO:0035239	15	0.246575	1.028567	0.382470	0.751475	1.000
146	GOBP_POSITIVE_REGULATION_OF_LIPASE_ACTIVITY	GO:0060193	5	0.385542	1.024295	0.432353	0.756088	1.000
147	HALLMARK_KRAS_SIGNALING_UP	M5953	6	0.366578	1.018665	0.406940	0.763755	1.000
148	GOBP_CELLULAR_RESPONSE_TO_NITROGEN_COMPOUND	GO:1901699	20	0.211044	1.012479	0.389830	0.772444	1.000
149	GOBP_RESPONSE_TO_PEPTIDE	GO:1901652	18	0.228572	1.010733	0.400844	0.771572	1.000
150	GOBP_NEGATIVE_REGULATION_OF_APOPTOTIC_SIGNALIN...	GO:2001234	7	0.324333	1.008630	0.431677	0.771550	1.000
151	GOBP_EPIDERMIS_DEVELOPMENT	GO:0008544	5	0.385542	1.005432	0.415954	0.774573	1.000
152	GOBP_RESPONSE_TO_ABIOTIC_STIMULUS	GO:0009628	27	0.197061	1.004904	0.463415	0.770655	1.000
153	GOBP_RESPONSE_TO_CADMIUM_ION	GO:0046686	6	0.350496	1.003856	0.427350	0.768192	1.000
154	GOBP_REGULATION_OF_NEURON_DIFFERENTIATION	GO:0045664	5	0.385542	0.997101	0.419098	0.779806	1.000
155	GOBP_RECEPTOR_INTERNALIZATION	GO:0031623	5	0.397590	0.995580	0.434426	0.778740	1.000
156	GOBP_INOSITOL_LIPID_MEDIATED_SIGNALING	GO:0048017	8	0.308742	0.993783	0.432990	0.777463	1.000
157	GOBP_NEGATIVE_REGULATION_OF_NEURON_DEATH	GO:1901215	11	0.272727	0.992546	0.455149	0.775832	1.000
158	GOBP_COGNITION	GO:0050890	28	0.188427	0.992432	0.476190	0.771195	1.000
159	GOBP_RESPONSE_TO_ACID_CHEMICAL	GO:0001101	6	0.348650	0.991421	0.441176	0.768658	1.000
160	GOBP_MAINTENANCE_OF_LOCATION	GO:0051235	7	0.323984	0.985761	0.435435	0.776903	1.000
161	GOBP_REGULATION_OF_PEPTIDYL_TYROSINE_PHOSPHORY...	GO:0050730	9	0.282733	0.976512	0.447099	0.792714	1.000
162	HALLMARK_APOPTOSIS	M5902	6	0.346976	0.974344	0.431818	0.792977	1.000
163	GOBP_REGULATION_OF_PHOSPHOLIPASE_C_ACTIVITY	GO:1900274	5	0.385542	0.973536	0.445748	0.790025	1.000
164	GOBP_CELLULAR_RESPONSE_TO_ABIOTIC_STIMULUS	GO:0071214	7	0.322147	0.970832	0.492537	0.791073	1.000
165	GOBP_SMOOTH_MUSCLE_CELL_PROLIFERATION	GO:0048659	6	0.343646	0.966993	0.444785	0.794966	1.000
166	GOBP_BLOOD_VESSEL_MORPHOGENESIS	GO:0048514	14	0.243243	0.965973	0.471483	0.792525	1.000
167	GOBP_REGULATION_OF_APOPTOTIC_SIGNALING_PATHWAY	GO:2001233	10	0.269231	0.946946	0.483180	0.831579	1.000
168	GOBP_REGULATION_OF_VASCULAR_ASSOCIATED_SMOOTH_...	GO:1904705	5	0.367759	0.944831	0.493113	0.831490	1.000
169	GOBP_POSITIVE_REGULATION_OF_CELL_POPULATION_PR...	GO:0008284	21	0.202991	0.944168	0.551724	0.827934	1.000

Continuation of Table **S2**

	NAME	ID	SIZE	ES	NES	NOM.p.val	FDR.q.val	FWER.p.val
170	GOBP_NEGATIVE_REGULATION_OF_PROTEIN_METABOLIC_...	GO:0051248	20	0.206828	0.943682	0.556098	0.824334	1.000
171	GOBP_REGULATION_OF_CELL_DEVELOPMENT	GO:0060284	15	0.229179	0.942961	0.526531	0.821382	1.000
172	GOBP_T_CELL_ACTIVATION	GO:0042110	8	0.296043	0.942421	0.550769	0.817904	1.000
173	GOBP_POSITIVE_REGULATION_OF_CELL_PROJECTION_OR...	GO:0031346	10	0.263454	0.939127	0.545752	0.820297	1.000
174	GOBP_RESPONSE_TO_NITROGEN_COMPOUND	GO:1901698	35	0.173399	0.938346	0.541667	0.817325	1.000
175	GOBP_POSITIVE_REGULATION_OF_ORGANELLE_ORGANIZA...	GO:0010638	10	0.264193	0.938133	0.511475	0.813289	1.000
176	GOBP_RESPONSE_TO_MOLECULE_OF_BACTERIAL_ORIGIN	GO:0002237	13	0.237073	0.938083	0.542636	0.808791	1.000
177	REACTOME_SIGNALING_BY_NTRKS	R-RNO-166520	18	0.206398	0.937190	0.566406	0.806319	1.000
178	GOBP_GLIOGENESIS	GO:0042063	12	0.244691	0.937020	0.515464	0.802196	1.000
179	GOBP_RESPONSE_TO_BACTERIUM	GO:0009617	13	0.237073	0.928220	0.564000	0.817219	1.000
180	GOBP_CELLULAR_RESPONSE_TO_REACTIVE_OXYGEN_SPECIES	GO:0034614	7	0.312426	0.926720	0.544025	0.816003	1.000
181	GOBP_REGULATION_OF_PROTEIN_CATABOLIC_PROCESS	GO:0042176	7	0.310043	0.925115	0.538206	0.815394	1.000
182	GOBP_CELLULAR_RESPONSE_TO_BIOTIC_STIMULUS	GO:0071216	8	0.282123	0.921191	0.560897	0.819238	1.000
183	GOBP_POSITIVE_REGULATION_OF_CELLULAR_COMPONENT...	GO:0044089	9	0.275348	0.909557	0.576369	0.840716	1.000
184	GOBP_CELLULAR_RESPONSE_TO_INORGANIC_SUBSTANCE	GO:0071241	11	0.246107	0.905116	0.578740	0.846360	1.000
185	GOBP_CELLULAR_RESPONSE_TO_MOLECULE_OF_BACTERIA...	GO:0071219	8	0.282123	0.904349	0.574324	0.843317	1.000
186	GOBP_CARBOHYDRATE_METABOLIC_PROCESS	GO:0005975	9	0.265823	0.902198	0.561644	0.843851	1.000
187	GOBP_REGULATION_OF_EXTRINSIC_APOPTOTIC_SIGNALI...	GO:2001236	6	0.329268	0.900480	0.534535	0.843186	1.000
188	GOBP_POSITIVE_REGULATION_OF_BINDING	GO:0051099	6	0.314323	0.899743	0.593567	0.840011	1.000
189	GOBP_CELLULAR_RESPONSE_TO_CHEMICAL_STRESS	GO:0062197	7	0.312426	0.899205	0.586103	0.836582	1.000
190	GOBP_REGULATION_OF_SMALL_MOLECULE_METABOLIC_PR...	GO:0062012	9	0.265823	0.897791	0.590476	0.835583	1.000
191	GOBP_CELLULAR_RESPONSE_TO_PEPTIDE	GO:1901653	13	0.226667	0.894479	0.581227	0.838232	1.000
192	GOBP_REGULATION_OF_NERVOUS_SYSTEM_DEVELOPMENT	GO:0051960	15	0.215999	0.893635	0.624535	0.835723	1.000
193	GOBP_POSITIVE_REGULATION_OF_RESPONSE_TO_EXTERN...	GO:0032103	9	0.265249	0.886115	0.610000	0.846711	1.000
194	GOBP_POSITIVE_REGULATION_OF_NEURON_DEATH	GO:1901216	5	0.350374	0.881613	0.598214	0.851963	1.000
195	GOBP_REGULATION_OF_EPITHELIAL_CELL_MIGRATION	GO:0010632	6	0.301974	0.871433	0.603279	0.869243	1.000
196	GOBP_NEGATIVE_REGULATION_OF_BIOSYNTHETIC_PROCESS	GO:0009890	13	0.218566	0.867179	0.616438	0.873859	1.000
197	GOBP_RESPONSE_TO_LIGHT_STIMULUS	GO:0009416	11	0.235352	0.861565	0.617857	0.880746	1.000
198	REACTOME_ESR_MEDIATED_SIGNALING	R-RNO-8939211	8	0.268343	0.860883	0.637255	0.877862	1.000
199	GOBP_NEGATIVE_REGULATION_OF_NUCLEOBASE_CONTAIN...	GO:0045934	13	0.218307	0.860778	0.617761	0.873783	1.000
200	GOBP_ANATOMICAL_STRUCTURE_FORMATION_INVOLVED_I...	GO:0048646	20	0.188820	0.859068	0.671296	0.872804	1.000
201	REACTOME_DEVELOPMENTAL_BIOLOGY	R-RNO-1266738	22	0.179148	0.858213	0.665158	0.869801	1.000
202	GOBP_NEGATIVE_REGULATION_OF_MOLECULAR_FUNCTION	GO:0044092	24	0.174591	0.854855	0.663507	0.872167	1.000
203	REACTOME_EXTRA_NUCLEAR_ESTROGEN_SIGNALING	R-RNO-9009391	7	0.280583	0.851443	0.675241	0.875037	1.000
204	REACTOME_SIGNALING_BY_NUCLEAR_RECEPTORS	R-RNO-9006931	8	0.268343	0.846029	0.652997	0.881479	1.000
205	GOBP_REGULATION_OF_DEVELOPMENTAL_GROWTH	GO:0048638	9	0.247509	0.845564	0.652459	0.878132	1.000
206	GOBP_RESPONSE_TO_LIPID	GO:0033993	19	0.186565	0.844920	0.681034	0.875227	1.000
207	GOBP_REGULATION_OF_CELLULAR_COMPONENT_BIOGENESIS	GO:0044087	10	0.230769	0.835975	0.686709	0.888405	1.000
208	GOBP_PROTEIN_LOCALIZATION_TO_PLASMA_MEMBRANE	GO:0072659	7	0.271605	0.834631	0.681115	0.886700	1.000
209	GOBP_NEGATIVE_REGULATION_OF_CATALYTIC_ACTIVITY	GO:0043086	17	0.197492	0.833706	0.691450	0.884383	1.000
210	GOBP_REGULATION_OF_NEUROGENESIS	GO:0050767	14	0.204986	0.832092	0.681992	0.883483	1.000
211	GOBP_LEARNING	GO:0007612	13	0.214610	0.831109	0.684397	0.881126	1.000
212	REACTOME_NUCLEAR_EVENTS_KINASE_AND_TRANSCRIPTI...	R-RNO-198725	11	0.227933	0.829574	0.682848	0.880018	1.000

Continuation of Table **S2**

	NAME	ID	SIZE	ES	NES	NOM.p.val	FDR.q.val	FWER.p.val
213	GOBP_PHAGOCYTOSIS	GO:0006909	5	0.313984	0.823634	0.652661	0.887231	1.000
214	GOBP_GENERATION_OF_PRECURSOR_METABOLITES_AND_E...	GO:0006091	5	0.325301	0.819119	0.666667	0.891227	1.000
215	GOBP_APOPTOTIC_MITOCHONDRIAL_CHANGES	GO:0008637	6	0.280488	0.812946	0.700288	0.898686	1.000
216	GOBP_POSITIVE_REGULATION_OF_CELL_GROWTH	GO:0030307	6	0.289468	0.812719	0.730104	0.894956	1.000
217	GOBP_MITOCHONDRION_ORGANIZATION	GO:0007005	7	0.271605	0.809367	0.739938	0.897279	1.000
218	GOBP_POSITIVE_REGULATION_OF_CELLULAR_COMPONENT...	GO:0051130	24	0.161920	0.804547	0.719298	0.901754	1.000
219	GOBP_RESPONSE_TO_RADIATION	GO:0009314	13	0.202164	0.801178	0.753906	0.903580	1.000
220	GOBP_PROTEIN_MODIFICATION_BY_SMALL_PROTEIN_CON...	GO:0070647	7	0.259259	0.798782	0.723684	0.903603	1.000
221	GOBP_MEMORY	GO:0007613	13	0.197345	0.789150	0.753968	0.916462	1.000
222	GOBP_REGULATION_OF_DNA_BINDING_TRANSCRIPTION_F...	GO:0051090	16	0.182921	0.784759	0.778571	0.920099	1.000
223	GOBP_RESPONSE_TO_INTERLEUKIN_1	GO:0070555	5	0.313845	0.783294	0.744382	0.918634	1.000
224	GOBP_ORGANIC_ACID_METABOLIC_PROCESS	GO:0006082	7	0.259259	0.775646	0.747475	0.927767	1.000
225	GOBP_TISSUE_MIGRATION	GO:0090130	9	0.227848	0.774783	0.778912	0.925098	1.000
226	GOBP_CARBOHYDRATE_DERIVATIVE_METABOLIC_PROCESS	GO:1901135	8	0.238102	0.772809	0.753086	0.924056	1.000
227	GOBP_NEUROINFLAMMATORY_RESPONSE	GO:0150076	5	0.295576	0.766714	0.748447	0.930125	1.000
228	GOBP_TEMPERATURE_HOMEOSTASIS	GO:0001659	5	0.293284	0.765820	0.736842	0.927558	1.000
229	GOBP_NEGATIVE_REGULATION_OF_CATABOLIC_PROCESS	GO:0009895	8	0.234119	0.764973	0.786885	0.924896	1.000
230	GOBP_REGULATION_OF_LIPID_METABOLIC_PROCESS	GO:0019216	7	0.259259	0.763299	0.758278	0.923715	1.000
231	GOBP_PROTEIN_KINASE_B_SIGNALING	GO:0043491	6	0.264584	0.756993	0.776025	0.929676	1.000
232	GOBP_RESPONSE_TO_HORMONE	GO:0009725	20	0.166165	0.753226	0.813853	0.931376	1.000
233	GOBP_REGULATION_OF_IMMUNE_SYSTEM_PROCESS	GO:0002682	21	0.155676	0.744707	0.831818	0.941272	1.000
234	GOBP_PROTEIN_CATABOLIC_PROCESS	GO:0030163	8	0.224278	0.737895	0.778846	0.947455	1.000
235	GOBP_MONOCARBOXYLIC_ACID_METABOLIC_PROCESS	GO:0032787	6	0.256098	0.737115	0.817365	0.944680	1.000
236	GOBP_MONOSACCHARIDE_METABOLIC_PROCESS	GO:0005996	6	0.256098	0.734007	0.782991	0.945105	1.000
237	GOBP_POSITIVE_REGULATION_OF_PHOSPHATIDYLINOSIT...	GO:0014068	5	0.286111	0.732071	0.790055	0.944128	1.000
238	GOBP_TELENCEPHALON_DEVELOPMENT	GO:0021537	7	0.234568	0.726567	0.815534	0.947992	1.000
239	GOBP_BLOOD_VESSEL_ENDOTHELIAL_CELL_MIGRATION	GO:0043534	8	0.225000	0.724806	0.858621	0.946391	1.000
240	GOBP_CELL_JUNCTION_ASSEMBLY	GO:0034329	8	0.225818	0.720379	0.845455	0.948791	1.000
241	GOBP_ENDOTHELIAL_CELL_MIGRATION	GO:0043542	8	0.225000	0.718164	0.869186	0.947988	1.000
242	GOBP_RESPONSE_TO_BIOTIC_STIMULUS	GO:0009607	17	0.157862	0.717363	0.871698	0.945447	1.000
243	GOBP_REGULATION_OF_LIPID_BIOSYNTHETIC_PROCESS	GO:0046890	6	0.256098	0.716666	0.811966	0.942465	1.000
244	GOBP_TRANSMEMBRANE_RECEPTOR_PROTEIN_SERINE_THR...	GO:0007178	6	0.256098	0.716244	0.775000	0.939345	1.000
245	GOBP_GLUCOSE_METABOLIC_PROCESS	GO:0006006	6	0.256098	0.712291	0.833828	0.941003	1.000
246	GOBP_ACTIVATION_OF_PROTEIN_KINASE_ACTIVITY	GO:0032147	10	0.196757	0.711940	0.884615	0.937639	1.000
247	GOBP_TUBE_DEVELOPMENT	GO:0035295	18	0.157401	0.708326	0.889344	0.939202	1.000
248	GOBP_DEVELOPMENTAL_CELL_GROWTH	GO:0048588	5	0.272833	0.701043	0.812680	0.944836	1.000
249	GOBP_NEGATIVE_REGULATION_OF_PROTEIN_CATABOLIC_...	GO:0042177	5	0.264669	0.696241	0.865270	0.946683	1.000
250	GOBP_MONOAMINE_TRANSPORT	GO:0015844	5	0.265060	0.694773	0.862259	0.944704	1.000
251	GOBP_NEGATIVE_REGULATION_OF_TRANSMEMBRANE_TRAN...	GO:0034763	6	0.246064	0.694754	0.862928	0.940960	1.000
252	GOBP_AXON_DEVELOPMENT	GO:0061564	18	0.153389	0.693965	0.920930	0.938242	1.000
253	GOBP_LIPID_BIOSYNTHETIC_PROCESS	GO:0008610	6	0.256098	0.677422	0.853211	0.955373	1.000
254	REACTOME_ESTROGEN_DEPENDENT_NUCLEAR_EVENTS_DOW...	R-RNO-9634638	5	0.256086	0.673036	0.857143	0.956675	1.000
255	GOBP_INTRACELLULAR_RECEPTOR_SIGNALING_PATHWAY	GO:0030522	5	0.253012	0.669349	0.870523	0.956832	1.000

Continuation of Table **S2**

	NAME	ID	SIZE	ES	NES	NOM.p.val	FDR.q.val	FWER.p.val
256	GOBP_PEPTIDYL_SERINE_MODIFICATION	GO:0018209	12	0.172444	0.653322	0.940000	0.970175	1.000
257	GOBP_POSITIVE_REGULATION_OF_SMALL_MOLECULE_MET...	GO:0062013	5	0.253012	0.633607	0.904110	0.985770	1.000
258	GOBP_REGULATION_OF_BINDING	GO:0051098	11	0.167505	0.628958	0.936396	0.986075	1.000
259	GOBP_REGULATION_OF_CELL_CELL_ADHESION	GO:0022407	9	0.185989	0.620166	0.958599	0.989946	1.000
260	GOBP_CELL_CELL_ADHESION	GO:0098609	14	0.150142	0.612462	0.962963	0.991996	1.000
261	GOBP_ASSOCIATIVE_LEARNING	GO:0008306	7	0.200243	0.603305	0.948640	0.994696	1.000
262	GOBP_MUSCLE_STRUCTURE_DEVELOPMENT	GO:0061061	16	0.138889	0.602117	0.953488	0.991787	1.000
263	GOBP_EMBRYONIC_MORPHOGENESIS	GO:0048598	6	0.215922	0.598940	0.933526	0.990320	1.000
264	GOBP_REGULATION_OF_ANION_TRANSMEMBRANE_TRANSPORT	GO:1903959	5	0.229882	0.586988	0.945860	0.995026	1.000
265	HALLMARK_PI3K_AKT_MTOR_SIGNALING	M5923	6	0.206315	0.586469	0.960000	0.991653	1.000
266	GOBP_PHOSPHATIDYLINOSITOL_3_KINASE_SIGNALING	GO:0014065	7	0.184026	0.575130	0.969789	0.994187	1.000
267	GOBP_SMALL_GTPASE_MEDIATED_SIGNAL_TRANSDUCTION	GO:0007264	8	0.175000	0.562528	0.969492	0.996730	1.000
268	GOBP_REGULATION_OF_PHOSPHATIDYLINOSITOL_3_KINA...	GO:0014066	6	0.196888	0.561923	0.968116	0.993294	1.000
269	GOBP_REGULATION_OF_PHOSPHOLIPASE_ACTIVITY	GO:0010517	6	0.182927	0.523021	0.997167	1.000000	1.000
270	GOBP_REGULATION_OF_LIPASE_ACTIVITY	GO:0060191	6	0.182927	0.513614	0.989933	1.000000	1.000
271	GOBP_POSITIVE_REGULATION_OF_IMMUNE_SYSTEM_PROCESS	GO:0002684	18	0.116068	0.507689	0.996212	0.999431	1.000
272	GOBP_REGULATION_OF_ANATOMICAL_STRUCTURE_SIZE	GO:0090066	10	0.142915	0.500134	0.977860	0.997011	1.000
273	GOBP_REGULATION_OF_PROTEIN_BINDING	GO:0043393	7	0.137979	0.422481	1.000000	0.999375	1.000
274	GOBP_REGULATION_OF_ANATOMICAL_STRUCTURE_MORPHO...	GO:0022603	17	-0.105904	-0.367504	0.998652	0.999539	1.000
275	GOBP_AGING	GO:0007568	13	-0.118336	-0.377863	0.998698	1.000000	1.000
276	GOBP_ENZYME_LINKED_RECEPTOR_PROTEIN_SIGNALING_...	GO:0007167	24	-0.116853	-0.434821	0.997409	1.000000	1.000
277	GOBP_VASCULATURE_DEVELOPMENT	GO:0001944	17	-0.126150	-0.436741	1.000000	1.000000	1.000
278	GOBP_CIRCULATORY_SYSTEM_DEVELOPMENT	GO:0072359	21	-0.117975	-0.437028	0.996139	1.000000	1.000
279	GOBP_POSITIVE_REGULATION_OF_DNA_BINDING_TRANSC...	GO:0051091	10	-0.154353	-0.450621	0.995575	1.000000	1.000
280	GOBP_POSITIVE_REGULATION_OF_GLIOGENESIS	GO:0014015	5	-0.196521	-0.455318	0.993893	1.000000	1.000
281	GOBP_REGULATION_OF_GLIOGENESIS	GO:0014013	5	-0.196521	-0.459066	0.994169	1.000000	1.000
282	GOBP_NEURON_MIGRATION	GO:0001764	5	-0.202327	-0.469725	0.998440	1.000000	1.000
283	GOBP_REGULATION_OF_NEURON_PROJECTION_DEVELOPMENT	GO:0010975	16	-0.146887	-0.489104	0.986486	1.000000	1.000
284	REACTOME_CELLULAR_RESPONSES_TO_EXTERNAL_STIMULI	R-RNO-8953897	11	-0.165255	-0.499314	0.983027	1.000000	1.000
285	GOBP_CELL_JUNCTION_ORGANIZATION	GO:0034330	19	-0.144928	-0.504704	0.977690	1.000000	1.000
286	GOBP_POSITIVE_REGULATION_OF_PROTEIN_PHOSPHORYL...	GO:0001934	22	-0.136159	-0.505624	0.979513	1.000000	1.000
287	GOBP_REGULATION_OF_IMMUNE_RESPONSE	GO:0050776	12	-0.164030	-0.506634	0.977654	1.000000	1.000
288	GOBP_SYNAPSE_ORGANIZATION	GO:0050808	17	-0.146905	-0.508554	0.979973	1.000000	1.000
289	GOBP_POSITIVE_REGULATION_OF_PROTEIN_MODIFICATI...	GO:0031401	22	-0.136159	-0.508561	0.983051	1.000000	1.000
290	GOBP_LONG_TERM_SYNAPTIC_DEPRESSION	GO:0060292	5	-0.217440	-0.509775	0.989264	1.000000	1.000
291	GOBP_REGULATION_OF_ENDOCYTOSIS	GO:0030100	6	-0.205081	-0.510696	0.973602	1.000000	1.000
292	GOBP_REGULATION_OF_PROTEIN_LOCALIZATION_TO_MEM...	GO:1905475	7	-0.204596	-0.519918	0.984849	1.000000	1.000
293	GOBP_EPITHELIAL_CELL_PROLIFERATION	GO:0050673	12	-0.173928	-0.533413	0.968320	1.000000	1.000
294	GOBP_IMPORT_INTO_CELL	GO:0098657	6	-0.216523	-0.536393	0.964602	1.000000	1.000
295	GOBP_REGULATION_OF_PROTEIN_PHOSPHORYLATION	GO:0001932	25	-0.143490	-0.545921	0.955186	1.000000	1.000
296	GOBP_REGULATION_OF_PROTEIN_STABILITY	GO:0031647	5	-0.236877	-0.549042	0.976958	1.000000	1.000
297	GOBP_POSITIVE_REGULATION_OF_MAP_KINASE_ACTIVITY	GO:0043406	6	-0.222406	-0.549139	0.963415	1.000000	1.000
298	REACTOME_POST_TRANSLATIONAL_PROTEIN_MODIFICATION	R-RNO-597592	7	-0.208639	-0.550696	0.952239	1.000000	1.000

Continuation of Table **S2**

	NAME	ID	SIZE	ES	NES	NOM.p.val	FDR.q.val	FWER.p.val
299	GOBP_ACTIVATION_OF_MAPK_ACTIVITY	GO:0000187	6	-0.222406	-0.551998	0.967742	1.000000	1.000
300	GOBP_PURINE_CONTAINING_COMPOUND_METABOLIC_PROCESS	GO:0072521	6	-0.223342	-0.553979	0.958209	1.000000	1.000
301	GOBP_RESPONSE_TO_NICOTINE	GO:0035094	7	-0.210901	-0.556822	0.959538	1.000000	1.000
302	GOBP_ANION_TRANSMEMBRANE_TRANSPORT	GO:0098656	7	-0.215154	-0.557472	0.947368	1.000000	1.000
303	GOBP_RESPONSE_TO_TEMPERATURE_STIMULUS	GO:0009266	9	-0.197220	-0.567293	0.959479	1.000000	1.000
304	GOBP_DNA_METABOLIC_PROCESS	GO:0006259	6	-0.234467	-0.580471	0.952527	1.000000	1.000
305	GOBP_POSITIVE_REGULATION_OF_PHOSPHORUS_METABOL...	GO:0010562	23	-0.155306	-0.586168	0.953488	1.000000	1.000
306	GOBP_MUSCLE_TISSUE_DEVELOPMENT	GO:0060537	11	-0.193900	-0.587470	0.942544	1.000000	1.000
307	GOBP_CELLULAR_COMPONENT_MORPHOGENESIS	GO:0032989	25	-0.157655	-0.590751	0.920615	1.000000	1.000
308	GOBP_POSITIVE_REGULATION_OF_PROTEIN_KINASE_ACT...	GO:0045860	18	-0.170318	-0.592071	0.944675	1.000000	1.000
309	GOBP_POSITIVE_REGULATION_OF_IMMUNE_RESPONSE	GO:0050778	9	-0.207695	-0.593196	0.947826	1.000000	1.000
310	GOBP_SKELETAL_MUSCLE_ORGAN_DEVELOPMENT	GO:0060538	5	-0.250940	-0.593414	0.955590	1.000000	1.000
311	GOBP_POSITIVE_REGULATION_OF_NEURON_PROJECTION_...	GO:0010976	5	-0.256647	-0.593931	0.935385	1.000000	1.000
312	GOBP_FOREBRAIN_DEVELOPMENT	GO:0030900	10	-0.205447	-0.596298	0.935664	1.000000	1.000
313	GOBP_CELL_PART_MORPHOGENESIS	GO:0032990	25	-0.157655	-0.596462	0.923761	1.000000	1.000
314	GOBP_NEGATIVE_REGULATION_OF_LIPID_METABOLIC_PR...	GO:0045833	5	-0.260425	-0.602233	0.933628	1.000000	1.000
315	GOBP_TRANSMEMBRANE_RECEPTOR_PROTEIN_TYROSINE_K...	GO:0007169	19	-0.173498	-0.604406	0.920000	1.000000	1.000
316	GOBP_LYMPHOCYTE_ACTIVATION	GO:0046649	10	-0.202958	-0.605912	0.924177	1.000000	1.000
317	GOBP_REGULATION_OF_CELLULAR_AMIDE_METABOLIC_PR...	GO:0034248	9	-0.213704	-0.606204	0.935574	1.000000	1.000
318	GOBP_REGULATION_OF_PHOSPHORUS_METABOLIC_PROCESS	GO:0051174	26	-0.162771	-0.608043	0.920984	1.000000	1.000
319	REACTOME_SIGNALING_BY_NTRK2_TRKB	R-RNO-9006115	6	-0.246116	-0.608051	0.908815	1.000000	1.000
320	GOBP_CELL_MORPHOGENESIS	GO:0000902	25	-0.157655	-0.608692	0.935317	1.000000	1.000
321	GOBP_POSITIVE_REGULATION_OF_CELL_ADHESION	GO:0045785	7	-0.231565	-0.610231	0.923195	1.000000	1.000
322	GOBP_POSITIVE_REGULATION_OF_MAPK_CASCADE	GO:0043410	9	-0.216395	-0.610797	0.926934	1.000000	1.000
323	GOBP_NEGATIVE_REGULATION_OF_CELL_DEVELOPMENT	GO:0010721	6	-0.247819	-0.614045	0.925595	1.000000	1.000
324	GOBP_ORGANONITROGEN_COMPOUND_CATABOLIC_PROCESS	GO:1901565	10	-0.210649	-0.619006	0.940278	1.000000	1.000
325	GOBP_REGULATION_OF_HORMONE_LEVELS	GO:0010817	12	-0.200696	-0.621727	0.916103	1.000000	1.000
326	GOBP_RESPONSE_TO_CALCIUM_ION	GO:0051592	6	-0.255934	-0.623693	0.911337	1.000000	1.000
327	GOBP_REGULATION_OF_PROTEIN_MODIFICATION_PROCESS	GO:0031399	28	-0.162277	-0.623810	0.917092	1.000000	1.000
328	GOBP_POSITIVE_REGULATION_OF_ION_TRANSPORT	GO:0043270	17	-0.180748	-0.623969	0.898667	1.000000	1.000
329	GOBP_REGULATION_OF_PROTEIN_KINASE_ACTIVITY	GO:0045859	21	-0.174151	-0.625182	0.909574	1.000000	1.000
330	GOBP_POSITIVE_REGULATION_OF_TRANSPORT	GO:0051050	26	-0.160936	-0.626176	0.919643	1.000000	1.000
331	GOBP_PEPTIDYL_AMINO_ACID_MODIFICATION	GO:0018193	22	-0.168541	-0.627797	0.884566	1.000000	1.000
332	REACTOME_SIGNALING_BY_RECEPTOR_TYROSINE_KINASES	R-RNO-9006934	26	-0.164835	-0.635886	0.890703	1.000000	1.000
333	GOBP_POSITIVE_REGULATION_OF_PROTEIN_METABOLIC_...	GO:0051247	27	-0.164636	-0.636082	0.893484	1.000000	1.000
334	GOBP_EPHRIN_RECEPTOR_SIGNALING_PATHWAY	GO:0048013	5	-0.267085	-0.637361	0.913767	1.000000	1.000
335	GOBP_REGULATION_OF_TRANSFERASE_ACTIVITY	GO:0051338	21	-0.174151	-0.638531	0.897503	1.000000	1.000
336	GOBP_REGULATION_OF_CELL_ACTIVATION	GO:0050865	8	-0.239731	-0.643675	0.890351	1.000000	1.000
337	GOBP_CARDIAC_MUSCLE_TISSUE_DEVELOPMENT	GO:0048738	6	-0.257535	-0.644616	0.879822	1.000000	1.000
338	GOBP_MULTICELLULAR_ORGANISMAL_RESPONSE_TO_STRESS	GO:0033555	5	-0.279031	-0.647185	0.888719	1.000000	1.000
339	GOBP_RESPONSE_TO_INSULIN	GO:0032868	5	-0.280960	-0.647424	0.892097	1.000000	1.000
340	GOBP_AMYLOID_PRECURSOR_PROTEIN_CATABOLIC_PROCESS	GO:0042987	5	-0.277078	-0.650088	0.894172	1.000000	1.000
341	GOBP_HEART_DEVELOPMENT	GO:0007507	6	-0.257535	-0.651779	0.885587	1.000000	1.000

Continuation of Table **S2**

	NAME	ID	SIZE	ES	NES	NOM.p.val	FDR.q.val	FWER.p.val
342	GOBP_AMYLOID_BETA_METABOLIC_PROCESS	GO:0050435	5	-0.277078	-0.651960	0.887879	1.000000	1.000
343	GOBP_AMYLOID_PRECURSOR_PROTEIN_METABOLIC_PROCESS	GO:0042982	5	-0.277078	-0.654382	0.875188	1.000000	1.000
344	GOBP_PEPTIDYL_LYSINE_MODIFICATION	GO:0018205	5	-0.279765	-0.655253	0.891850	1.000000	1.000
345	GOBP_PROTEIN_LOCALIZATION_TO_MEMBRANE	GO:0072657	13	-0.204681	-0.655336	0.882845	1.000000	1.000
346	GOBP_CELLULAR_LIPID_METABOLIC_PROCESS	GO:0044255	7	-0.249394	-0.655593	0.880775	1.000000	1.000
347	GOBP_ENDOTHELIAL_CELL_PROLIFERATION	GO:0001935	8	-0.233929	-0.655861	0.873381	1.000000	1.000
348	GOBP_LIPID_METABOLIC_PROCESS	GO:0006629	10	-0.223034	-0.657325	0.886657	1.000000	1.000
349	GOBP_TISSUE_HOMEOSTASIS	GO:0001894	5	-0.280632	-0.659432	0.883686	1.000000	1.000
350	GOBP_REGULATION_OF_MUSCLE_CELL_DIFFERENTIATION	GO:0051147	6	-0.264711	-0.665116	0.880060	1.000000	1.000
351	GOBP_CELL_ACTIVATION	GO:0001775	22	-0.184767	-0.671771	0.864474	1.000000	1.000
352	GOBP_CELLULAR_RESPONSE_TO_ORGANIC_CYCLIC_COMPOUND	GO:0071407	13	-0.208569	-0.671955	0.849007	1.000000	1.000
353	GOBP_POSITIVE_REGULATION_OF_TRANSFERASE_ACTIVITY	GO:0051347	19	-0.189698	-0.675121	0.847074	1.000000	1.000
354	REACTOME_INFECTIOUS_DISEASE	R-HSA-5663205	12	-0.216988	-0.677693	0.872951	1.000000	1.000
355	GOBP_CELLULAR_RESPONSE_TO_HORMONE_STIMULUS	GO:0032870	11	-0.227220	-0.683277	0.850350	1.000000	1.000
356	GOBP_REGULATION_OF_LONG_TERM_SYNAPTIC_POTENTIA...	GO:1900271	6	-0.275500	-0.686393	0.857364	1.000000	1.000
357	GOBP_POSITIVE_REGULATION_OF_GROWTH	GO:0045927	9	-0.247927	-0.688416	0.853623	1.000000	1.000
358	GOBP_DEVELOPMENTAL_MATURATION	GO:0021700	5	-0.299141	-0.689233	0.858034	1.000000	1.000
359	GOBP_ERK1_AND_ERK2_CASCADE	GO:0070371	5	-0.291635	-0.691633	0.867669	1.000000	1.000
360	GOBP_CELL_CELL_JUNCTION_ORGANIZATION	GO:0045216	6	-0.280364	-0.693507	0.838710	1.000000	1.000
361	GOBP_PEPTIDE_METABOLIC_PROCESS	GO:0006518	11	-0.227051	-0.695085	0.858956	1.000000	1.000
362	GOBP_RESPONSE_TO_MONOSACCHARIDE	GO:0034284	5	-0.297300	-0.695934	0.861538	1.000000	1.000
363	GOBP_REGULATION_OF_PROTEIN_LOCALIZATION_TO_CEL...	GO:1904375	6	-0.284474	-0.696862	0.859216	1.000000	1.000
364	GOBP_REGULATION_OF_CATABOLIC_PROCESS	GO:0009894	13	-0.215512	-0.698101	0.828104	1.000000	1.000
365	GOBP_STRIATED_MUSCLE_CELL_DIFFERENTIATION	GO:0051146	9	-0.244568	-0.698942	0.839525	1.000000	1.000
366	GOBP_REGULATION_OF_CELLULAR_CATABOLIC_PROCESS	GO:0031329	10	-0.237344	-0.702082	0.850000	1.000000	1.000
367	GOBP_NEGATIVE_REGULATION_OF_NERVOUS_SYSTEM_DEV...	GO:0051961	5	-0.297474	-0.702912	0.864742	1.000000	1.000
368	GOBP_GLIAL_CELL_DIFFERENTIATION	GO:0010001	10	-0.241437	-0.703706	0.813044	1.000000	1.000
369	GOBP_RESPONSE_TO_CARBOHYDRATE	GO:0009743	5	-0.297300	-0.704735	0.840601	1.000000	1.000
370	GOBP_MULTICELLULAR_ORGANISMAL_HOMEOSTASIS	GO:0048871	12	-0.232006	-0.707349	0.811554	1.000000	1.000
371	REACTOME_DISEASES_OF_SIGNAL_TRANSDUCTION_BY_GR...	R-HSA-5663202	12	-0.227756	-0.707473	0.836364	1.000000	1.000
372	GOBP_REGULATION_OF_MITOTIC_CELL_CYCLE	GO:0007346	5	-0.310461	-0.713380	0.811679	1.000000	1.000
373	GOBP_RESPONSE_TO_METAL_ION	GO:0010038	15	-0.217060	-0.713842	0.841678	1.000000	1.000
374	GOBP_CELLULAR_RESPONSE_TO_DNA_DAMAGE_STIMULUS	GO:0006974	5	-0.308476	-0.714549	0.814590	1.000000	1.000
375	GOBP_RESPONSE_TO_STARVATION	GO:0042594	5	-0.311379	-0.717850	0.813814	1.000000	1.000
376	GOBP_DEVELOPMENTAL_GROWTH	GO:0048589	10	-0.249504	-0.719140	0.840112	1.000000	1.000
377	GOBP_TAXIS	GO:0042330	14	-0.222258	-0.720601	0.817321	1.000000	1.000
378	GOBP_DEVELOPMENTAL_PROCESS_INVOLVED_IN_REPRODU...	GO:0003006	11	-0.238661	-0.722136	0.834294	1.000000	1.000
379	GOBP_NEUROGENESIS	GO:0022008	41	-0.177027	-0.723212	0.796818	1.000000	1.000
380	GOBP_NEGATIVE_REGULATION_OF_SYNAPTIC_TRANSMISSION	GO:0050805	6	-0.293251	-0.725119	0.824060	1.000000	1.000
381	GOBP_CELLULAR_RESPONSE_TO_STARVATION	GO:0009267	5	-0.311379	-0.728551	0.822400	1.000000	1.000
382	GOBP_POSITIVE_REGULATION_OF_INTRACELLULAR_SIGN...	GO:1902533	14	-0.223275	-0.729047	0.800813	1.000000	1.000
383	GOBP_REGULATION_OF_VESICLE_MEDIATED_TRANSPORT	GO:0060627	12	-0.233658	-0.730291	0.813423	1.000000	1.000
384	GOBP_RESPONSE_TO_INORGANIC_SUBSTANCE	GO:0010035	19	-0.208714	-0.731355	0.824011	1.000000	1.000

Continuation of Table **S2**

	NAME	ID	SIZE	ES	NES	NOM.p.val	FDR.q.val	FWER.p.val
385	GOBP_REGULATION_OF_HYDROLASE_ACTIVITY	GO:0051336	22	-0.202739	-0.740649	0.791403	1.000000	1.000
386	GOBP_POSITIVE_REGULATION_OF_MOLECULAR_FUNCTION	GO:0044093	37	-0.187327	-0.750831	0.764852	1.000000	1.000
387	GOBP_NEGATIVE_REGULATION_OF_SIGNALING	GO:0023057	21	-0.208100	-0.751349	0.797635	1.000000	1.000
388	GOBP_RESPONSE_TO_DRUG	GO:0042493	11	-0.251497	-0.753562	0.770515	1.000000	1.000
389	GOBP_LONG_TERM_MEMORY	GO:0007616	6	-0.303552	-0.754048	0.786687	1.000000	1.000
390	GOBP_SMALL_MOLECULE_BIOSYNTHETIC_PROCESS	GO:0044283	5	-0.326053	-0.756275	0.793510	1.000000	1.000
391	GOBP_PROTEIN_MODIFICATION_BY_SMALL_PROTEIN_CON...	GO:0032446	6	-0.311944	-0.760384	0.767442	1.000000	1.000
392	GOBP_ORGANOPHOSPHATE_METABOLIC_PROCESS	GO:0019637	8	-0.276638	-0.763487	0.756914	1.000000	1.000
393	GOBP_RESPIRATORY_SYSTEM_DEVELOPMENT	GO:0060541	6	-0.301638	-0.763542	0.770393	1.000000	1.000
394	GOBP_POSTSYNAPSE_ORGANIZATION	GO:0099173	10	-0.262365	-0.765231	0.765739	1.000000	1.000
395	GOBP_PROTEIN_LOCALIZATION_TO_CELL_PERIPHERY	GO:1990778	12	-0.247152	-0.768112	0.745179	1.000000	1.000
396	GOBP_POSITIVE_REGULATION_OF_LONG_TERM_SYNAPTIC...	GO:1900273	5	-0.331576	-0.771117	0.768049	1.000000	1.000
397	GOBP_REGULATION_OF_ORGANELLE_ORGANIZATION	GO:0033043	14	-0.236347	-0.771456	0.756393	1.000000	1.000
398	REACTOME_ANTI_INFLAMMATORY_RESPONSE_FAVOURING_...	R-HSA-9662851	5	-0.342290	-0.773489	0.769953	1.000000	1.000
399	GOBP_LOCOMOTION	GO:0040011	31	-0.198729	-0.773979	0.747112	1.000000	1.000
400	GOBP_MATERNAL_PROCESS_INVOLVED_IN_FEMALE_PREGN...	GO:0060135	5	-0.334101	-0.778829	0.735614	1.000000	1.000
401	GOBP_POSITIVE_REGULATION_OF_EPITHELIAL_CELL_MI...	GO:0010634	5	-0.338305	-0.781744	0.789474	1.000000	1.000
402	GOBP_BIOLOGICAL_PROCESS_INVOLVED_IN_SYMBIOTIC_...	GO:004440	10	-0.271201	-0.782893	0.744681	1.000000	1.000
403	GOBP_NEURON_DIFFERENTIATION	GO:0030182	37	-0.195949	-0.784380	0.740050	1.000000	1.000
404	GOBP_REGULATION_OF_SYNAPSE_STRUCTURE_OR_ACTIVITY	GO:0050803	11	-0.255691	-0.785471	0.732606	1.000000	1.000
405	GOBP_NEGATIVE_REGULATION_OF_BINDING	GO:0051100	6	-0.316559	-0.786286	0.736215	1.000000	1.000
406	GOBP_REGULATION_OF_CELL_PROJECTION_ORGANIZATION	GO:0031344	20	-0.220944	-0.788056	0.768725	1.000000	1.000
407	GOBP_EXOCYTOSIS	GO:0006887	11	-0.265448	-0.788260	0.758133	1.000000	1.000
408	GOBP_REGULATION_OF_CYSTEINE_TYPE_ENDOPEPTIDASE...	GO:2000116	9	-0.284756	-0.795820	0.730994	1.000000	1.000
409	GOBP_ORGANIC_HYDROXY_COMPOUND_METABOLIC_PROCESS	GO:1901615	8	-0.289379	-0.798825	0.719168	1.000000	1.000
410	GOBP_BEHAVIOR	GO:0007610	33	-0.204091	-0.800811	0.729193	1.000000	1.000
411	GOBP_POSITIVE_REGULATION_OF_APOPTOTIC_SIGNALIN...	GO:2001235	5	-0.341606	-0.802040	0.721386	1.000000	1.000
412	GOBP_SMALL_MOLECULE_METABOLIC_PROCESS	GO:0044281	16	-0.237436	-0.803595	0.738526	1.000000	1.000
413	GOBP_CYTOSKELETON_ORGANIZATION	GO:0007010	11	-0.266359	-0.803693	0.735537	1.000000	1.000
414	GOBP_NUCLEOBASE_CONTAINING_SMALL_MOLECULE_META...	GO:0055086	7	-0.312294	-0.804345	0.700590	1.000000	1.000
415	GOBP_ENDOCYTOSIS	GO:0006897	8	-0.296944	-0.805204	0.714497	1.000000	1.000
416	GOBP_NEGATIVE_REGULATION_OF_PROTEIN_MODIFICATI...	GO:0031400	10	-0.271705	-0.806007	0.719323	1.000000	1.000
417	GOBP_REGULATION_OF_CYTOSKELETON_ORGANIZATION	GO:0051493	5	-0.347092	-0.807242	0.729231	1.000000	1.000
418	GOBP_REGULATION_OF_CELLULAR_PROTEIN_LOCALIZATION	GO:1903827	12	-0.263226	-0.809202	0.708049	1.000000	1.000
419	GOBP_CELL_MIGRATION	GO:0016477	22	-0.221668	-0.810406	0.718147	1.000000	1.000
420	GOBP_DENDRITIC_SPINE_MORPHOGENESIS	GO:0060997	5	-0.342626	-0.812545	0.729521	1.000000	1.000
421	GOBP_MULTI_ORGANISM_PROCESS	GO:0051704	16	-0.237337	-0.812756	0.712925	1.000000	1.000
422	GOBP_BIOLOGICAL_ADHESION	GO:0022610	19	-0.236320	-0.814362	0.717131	1.000000	1.000
423	GOBP_REGULATION_OF_CELLULAR_LOCALIZATION	GO:0060341	17	-0.238145	-0.820208	0.686934	1.000000	1.000
424	REACTOME_ONCOGENIC_MAPK_SIGNALING	R-HSA-680295	5	-0.356466	-0.820738	0.713202	1.000000	1.000
425	GOBP_MYELOID_LEUKOCYTE_ACTIVATION	GO:0002274	8	-0.299911	-0.821491	0.701318	1.000000	1.000
426	GOBP_PROTEIN_PHOSPHORYLATION	GO:0006468	33	-0.212075	-0.826240	0.711223	1.000000	1.000
427	GOBP_REGULATION_OF_ORGAN_GROWTH	GO:0046620	5	-0.367800	-0.836898	0.690402	1.000000	1.000

Continuation of Table **S2**

	NAME	ID	SIZE	ES	NES	NOM.p.val	FDR.q.val	FWER.p.val
428	GOBP_REGULATION_OF_PEPTIDE_SECRETION	GO:0002791	7	-0.323610	-0.838777	0.687407	1.000000	1.000
429	GOBP_REGULATION_OF_ENDOTHELIAL_CELL_MIGRATION	GO:0010594	5	-0.358628	-0.839037	0.687500	1.000000	1.000
430	GOBP_RESPONSE_TO_PEPTIDE_HORMONE	GO:0043434	13	-0.266388	-0.846000	0.662873	1.000000	1.000
431	GOBP_NERVOUS_SYSTEM_PROCESS	GO:0050877	38	-0.208365	-0.846851	0.665446	1.000000	1.000
432	GOBP_HORMONE_TRANSPORT	GO:0009914	9	-0.300527	-0.853920	0.657593	1.000000	1.000
433	GOBP_CELL_MORPHOGENESIS_INVOLVED_IN_DIFFERENTI...	GO:0000904	22	-0.232577	-0.858501	0.654568	1.000000	1.000
434	GOBP_REGULATION_OF_METAL_ION_TRANSPORT	GO:0010959	6	-0.350443	-0.863581	0.665667	1.000000	1.000
435	GOBP_NEGATIVE_REGULATION_OF_CELLULAR_COMPONENT...	GO:0051129	11	-0.287791	-0.863801	0.644476	1.000000	1.000
436	GOBP_ORGAN_GROWTH	GO:0035265	5	-0.367800	-0.864454	0.666667	1.000000	1.000
437	GOBP_CELL_MORPHOGENESIS_INVOLVED_IN_NEURON_DIF...	GO:0048667	22	-0.232577	-0.867671	0.617725	1.000000	1.000
438	GOBP_POSITIVE_REGULATION_OF_HYDROLASE_ACTIVITY	GO:0051345	16	-0.256692	-0.867713	0.613281	1.000000	1.000
439	GOBP_IMMUNE_EFFECTOR_PROCESS	GO:0002252	11	-0.285153	-0.873340	0.632737	0.997849	1.000
440	GOBP_REGULATION_OF_HORMONE_SECRETION	GO:0046883	8	-0.318326	-0.876584	0.625731	0.994141	1.000
441	REACTOME_EPH_EPHRIN_SIGNALING	R-RNO-2682334	6	-0.354703	-0.877481	0.628571	0.995784	1.000
442	GOBP_ORGANIC_HYDROXY_COMPOUND_TRANSPORT	GO:0015850	6	-0.344268	-0.878627	0.622388	0.996941	1.000
443	GOBP_NEGATIVE_REGULATION_OF_RESPONSE_TO_STIMULUS	GO:0048585	16	-0.259419	-0.879063	0.646053	0.999581	1.000
444	GOBP_ERBB_SIGNALING_PATHWAY	GO:0038127	5	-0.377641	-0.884023	0.623234	0.992558	1.000
445	GOBP_ORGANONITROGEN_COMPOUND_BIOSYNTHETIC_PROCESS	GO:1901566	13	-0.283564	-0.891715	0.600801	0.979697	1.000
446	GOBP_POLYSACCHARIDE_METABOLIC_PROCESS	GO:0005976	5	-0.382944	-0.892400	0.615262	0.981657	1.000
447	GOBP_REGULATION_OF_INTRACELLULAR_SIGNAL_TRANSD...	GO:1902531	21	-0.242174	-0.893651	0.590793	0.982581	1.000
448	GOBP_POSITIVE_REGULATION_OF_ANION_TRANSPORT	GO:1903793	9	-0.307096	-0.894321	0.577201	0.984660	1.000
449	GOBP_REGULATION_OF_WNT_SIGNALING_PATHWAY	GO:0030111	5	-0.382454	-0.895705	0.597015	0.985260	1.000
450	GOBP_REGULATION_OF_RESPONSE_TO_EXTERNAL_STIMULUS	GO:0032101	15	-0.266988	-0.898297	0.588549	0.983143	1.000
451	GOBP_INSULIN_SECRETION	GO:0030073	7	-0.344258	-0.899369	0.618128	0.984361	1.000
452	GOBP_AMIDE_BIOSYNTHETIC_PROCESS	GO:0043604	6	-0.370029	-0.899386	0.607784	0.987944	1.000
453	REACTOME_PLATELET_ACTIVATION_SIGNALING_AND_AGG...	R-HSA-76002	7	-0.346693	-0.901057	0.597365	0.987858	1.000
454	GOBP_PEPTIDE_HORMONE_SECRETION	GO:0030072	7	-0.344258	-0.901147	0.608504	0.991295	1.000
455	GOBP_REGULATION_OF_CARBOHYDRATE_METABOLIC_PROCESS	GO:0006109	5	-0.390162	-0.901954	0.605016	0.993180	1.000
456	GOBP_CHROMATIN_ORGANIZATION	GO:0006325	5	-0.391354	-0.904599	0.582953	0.990804	1.000
457	GOBP_CARBOHYDRATE_BIOSYNTHETIC_PROCESS	GO:0016051	5	-0.390162	-0.905407	0.570776	0.992856	1.000
458	GOBP_MULTICELLULAR_ORGANISM_REPRODUCTION	GO:0032504	12	-0.292300	-0.909353	0.555710	0.987683	1.000
459	GOBP_CELLULAR_RESPONSE_TO_LIPID	GO:0071396	15	-0.280883	-0.909715	0.584131	0.990526	1.000
460	GOBP_MYOTUBE_DIFFERENTIATION	GO:0014902	5	-0.397590	-0.914068	0.578370	0.984461	1.000
461	GOBP_MUSCLE_ORGAN_DEVELOPMENT	GO:0007517	9	-0.315704	-0.914258	0.576487	0.987756	1.000
462	GOBP_POSITIVE_REGULATION_OF_EPITHELIAL_CELL_PR...	GO:0050679	7	-0.352755	-0.914427	0.570381	0.991064	1.000
463	GOBP_REGULATION_OF_CARBOHYDRATE_BIOSYNTHETIC_P...	GO:0043255	5	-0.390162	-0.918038	0.569733	0.986280	1.000
464	GOBP_NEGATIVE_REGULATION_OF_CELL_CYCLE	GO:0045786	5	-0.388723	-0.919240	0.587025	0.987321	1.000
465	GOBP_REGULATION_OF_MAPK_CASCADE	GO:0043408	13	-0.289289	-0.922039	0.553521	0.984860	1.000
466	GOBP_REGULATION_OF_TRANSMEMBRANE_TRANSPORT	GO:0034762	23	-0.252831	-0.923919	0.560468	0.984233	1.000
467	GOBP_PEPTIDE_BIOSYNTHETIC_PROCESS	GO:0043043	6	-0.370029	-0.924773	0.549777	0.986246	1.000
468	REACTOME_EXTRACELLULAR_MATRIX_ORGANIZATION	R-RNO-1474244	5	-0.402623	-0.924862	0.568421	0.989861	1.000
469	GOBP_POSITIVE_REGULATION_OF_SIGNALING	GO:0023056	34	-0.233837	-0.928207	0.554020	0.985836	1.000
470	GOBP_ACTIVATION_OF_IMMUNE_RESPONSE	GO:0002253	5	-0.398977	-0.931272	0.562021	0.982758	1.000

Continuation of Table **S2**

	NAME	ID	SIZE	ES	NES	NOM.p.val	FDR.q.val	FWER.p.val
471	GOBP_MACROMOLECULE_CATABOLIC_PROCESS	GO:0009057	11	-0.312774	-0.933947	0.543759	0.980494	1.000
472	GOBP_NEGATIVE_REGULATION_OF_DEVELOPMENTAL_PROCESS	GO:0051093	13	-0.294654	-0.939314	0.545076	0.972055	1.000
473	GOBP_DENDRITIC_SPINE_DEVELOPMENT	GO:0060996	6	-0.381641	-0.942065	0.538117	0.969385	1.000
474	GOBP_POSITIVE_REGULATION_OF_SYNAPTIC_TRANSMISSION	GO:0050806	15	-0.278759	-0.945032	0.503365	0.966285	1.000
475	GOBP_NEGATIVE_REGULATION_OF_PHOSPHORUS_METABOL...	GO:0010563	9	-0.338728	-0.947657	0.524854	0.964151	1.000
476	GOBP_NEGATIVE_REGULATION_OF_CELL_DIFFERENTIATION	GO:0045596	13	-0.294654	-0.948097	0.515646	0.966989	1.000
477	GOBP_CENTRAL_NERVOUS_SYSTEM_NEURON_DIFFERENTIA...	GO:0021953	5	-0.405736	-0.949488	0.541411	0.967592	1.000
478	GOBP_NEURON_DEVELOPMENT	GO:0048666	34	-0.241040	-0.952251	0.532746	0.964906	1.000
479	GOBP_CELLULAR_AMIDE_METABOLIC_PROCESS	GO:0043603	12	-0.308177	-0.954238	0.510578	0.964061	1.000
480	GOBP_POSITIVE_REGULATION_OF_CATALYTIC_ACTIVITY	GO:0043085	29	-0.248073	-0.956300	0.518566	0.963177	1.000
481	GOBP_REGULATION_OF_CELLULAR_COMPONENT_MOVEMENT	GO:0051270	15	-0.286914	-0.957512	0.533153	0.964175	1.000
482	GOBP_REGULATION_OF_NEURONAL_SYNAPTIC_PLASTICITY	GO:0048168	7	-0.358404	-0.958139	0.523169	0.966546	1.000
483	GOBP_NEGATIVE_REGULATION_OF_ION_TRANSPORT	GO:0043271	11	-0.321600	-0.960395	0.514936	0.964994	1.000
484	GOBP_NEGATIVE_REGULATION_OF_INTRACELLULAR_SIGN...	GO:1902532	7	-0.365602	-0.960469	0.512012	0.968770	1.000
485	GOBP_MUSCLE_CELL_DIFFERENTIATION	GO:0042692	10	-0.323984	-0.961032	0.513630	0.971468	1.000
486	GOBP_REGULATION_OF_MAP_KINASE_ACTIVITY	GO:0043405	9	-0.342459	-0.961560	0.530410	0.974087	1.000
487	GOBP_CIRCADIAN_RHYTHM	GO:0007623	13	-0.305956	-0.963432	0.502688	0.973612	1.000
488	GOBP_ENDOCRINE_SYSTEM_DEVELOPMENT	GO:0035270	6	-0.396638	-0.963598	0.541353	0.977309	1.000
489	GOBP_CALCIUM_MEDIATED_SIGNALING	GO:0019722	10	-0.325941	-0.966247	0.491620	0.974826	1.000
490	GOBP_PEPTIDE_SECRETION	GO:0002790	9	-0.341594	-0.966577	0.502140	0.978166	1.000
491	GOBP_NEURON_PROJECTION_GUIDANCE	GO:0097485	9	-0.337170	-0.970827	0.509573	0.971536	1.000
492	GOBP_POSITIVE_REGULATION_OF_PROTEIN_SERINE_THR...	GO:0071902	9	-0.342700	-0.972668	0.490701	0.971198	1.000
493	GOBP_RESPONSE_TO_KETONE	GO:1901654	7	-0.375973	-0.972820	0.512195	0.975011	1.000
494	REACTOME_INNATE_IMMUNE_SYSTEM	R-RNO-168249	16	-0.288357	-0.972842	0.496680	0.979170	1.000
495	GOBP_REGULATION_OF_PEPTIDE_HORMONE_SECRETION	GO:0090276	6	-0.395451	-0.974551	0.484756	0.979196	1.000
496	REACTOME_NEUTROPHIL_DEGRANULATION	R-RNO-6798695	6	-0.396667	-0.976684	0.508246	0.978031	1.000
497	GOBP_CELLULAR_CARBOHYDRATE_METABOLIC_PROCESS	GO:0044262	5	-0.419927	-0.978041	0.496979	0.978885	1.000
498	GOBP_RESPONSE_TO_HEAT	GO:0009408	7	-0.372100	-0.978883	0.506667	0.981178	1.000
499	GOBP_LONG_TERM_SYNAPTIC_POTENTIATION	GO:0060291	12	-0.312859	-0.979371	0.481638	0.984255	1.000
500	GOBP_REGULATION_OF_PROTEIN_SERINE_THREONINE_KI...	GO:0071900	12	-0.316089	-0.979436	0.492997	0.988442	1.000
501	REACTOME_VESICLE_MEDIATED_TRANSPORT	R-RNO-5653656	5	-0.425419	-0.987458	0.467669	0.971921	1.000
502	GOBP_CELL_ACTIVATION_INVOLVED_IN_IMMUNE_RESPONSE	GO:0002263	6	-0.396667	-0.991190	0.488722	0.966459	1.000
503	GOBP_POSITIVE_REGULATION_OF_ESTABLISHMENT_OF_P...	GO:1904951	5	-0.420890	-0.994911	0.482759	0.961393	1.000
504	GOBP_VESICLE_MEDIATED_TRANSPORT_IN_SYNAPSE	GO:0099003	7	-0.382716	-0.994981	0.483533	0.965526	1.000
505	REACTOME_TOLL_LIKE_RECEPTOR_TLR1_TLR2_CASCADE	R-RNO-168179	8	-0.372989	-0.997163	0.484581	0.964256	1.000
506	GOBP_POSITIVE_REGULATION_OF_ENDOTHELIAL_CELL_P...	GO:0001938	6	-0.401205	-0.997302	0.480858	0.968206	1.000
507	GOBP_CIRCULATORY_SYSTEM_PROCESS	GO:0003013	5	-0.430008	-0.997573	0.474654	0.971899	1.000
508	REACTOME_TOLL_LIKE_RECEPTOR_9_TLR9_CASCADE	R-RNO-168138	8	-0.372989	-0.998367	0.464888	0.974219	1.000
509	GOBP_REGULATION_OF_GTPASE_ACTIVITY	GO:0043087	5	-0.425761	-1.001446	0.485075	0.970625	1.000
510	GOBP_REGULATION_OF_GROWTH	GO:0040008	13	-0.310538	-1.002497	0.473538	0.972289	1.000
511	GOBP_MUSCLE_CELL_DEVELOPMENT	GO:0055001	5	-0.437716	-1.003272	0.471212	0.974670	1.000
512	REACTOME_MEMBRANE_TRAFFICKING	R-RNO-199991	5	-0.425419	-1.003789	0.466563	0.977891	1.000
513	GOBP_ANATOMICAL_STRUCTURE_HOMEOSTASIS	GO:0060249	7	-0.384362	-1.004579	0.467262	0.980449	1.000

Continuation of Table **S2**

	NAME	ID	SIZE	ES	NES	NOM.p.val	FDR.q.val	FWER.p.val
514	GOBP_RESPONSE_TO_ALKALOID	GO:0043279	5	-0.440285	-1.007550	0.465224	0.977129	1.000
515	GOBP_CELLULAR_MACROMOLECULE_LOCALIZATION	GO:0070727	20	-0.287657	-1.009095	0.456209	0.977579	1.000
516	GOBP_MYELOID_LEUKOCYTE_MEDIATED_IMMUNITY	GO:0002444	6	-0.396667	-1.009402	0.444795	0.981374	1.000
517	REACTOME_TOLL_LIKE_RECEPTOR_CASCADES	R-RNO-168898	8	-0.372989	-1.009612	0.470085	0.985555	1.000
518	GOBP_LEUKOCYTE_MIGRATION	GO:0050900	6	-0.406479	-1.009729	0.431085	0.990005	1.000
519	GOBP_POSITIVE_REGULATION_OF_CELLULAR_PROTEIN_L...	GO:1903829	7	-0.383383	-1.010994	0.449405	0.991259	1.000
520	GOBP_POSITIVE_REGULATION_OF_PROTEOLYSIS	GO:0045862	8	-0.372698	-1.011490	0.463557	0.994639	1.000
521	GOBP_NEURON_PROJECTION_ORGANIZATION	GO:0106027	8	-0.368945	-1.014573	0.441595	0.990987	1.000
522	GOBP_PROTEIN_LOCALIZATION_TO_CELL_JUNCTION	GO:1902414	6	-0.413131	-1.014950	0.476762	0.994597	1.000
523	GOBP_PROTEIN_DEPHOSPHORYLATION	GO:0006470	7	-0.382179	-1.015843	0.442363	0.997053	1.000
524	GOBP_REGULATION_OF_CELL_CYCLE	GO:0051726	13	-0.319186	-1.016073	0.444598	1.000000	1.000
525	GOBP_DEPHOSPHORYLATION	GO:0016311	7	-0.382179	-1.017675	0.433048	1.000000	1.000
526	GOBP_REGULATION_OF_CELLULAR_RESPONSE_TO_STRESS	GO:0080135	9	-0.360298	-1.019560	0.450216	1.000000	1.000
527	GOBP_CELL_PROJECTION_ORGANIZATION	GO:0030030	35	-0.256067	-1.019747	0.442211	1.000000	1.000
528	GOBP_REGULATION_OF_RESPONSE_TO_STRESS	GO:0080134	19	-0.288207	-1.021829	0.430446	1.000000	1.000
529	GOBP_POSITIVE_REGULATION_OF_PEPTIDASE_ACTIVITY	GO:0010952	7	-0.395871	-1.024942	0.430124	1.000000	1.000
530	GOBP_REGULATION_OF_LIPID_LOCALIZATION	GO:1905952	5	-0.437572	-1.025840	0.442249	1.000000	1.000
531	REACTOME_MYD88_INDEPENDENT_TLR4_CASCADE	R-RNO-166166	8	-0.372989	-1.027367	0.415473	1.000000	1.000
532	GOBP_ESTABLISHMENT_OF_PROTEIN_LOCALIZATION	GO:0045184	17	-0.301025	-1.027400	0.428571	1.000000	1.000
533	GOBP_NEGATIVE_REGULATION_OF_TRANSPORT	GO:0051051	13	-0.317179	-1.028373	0.435967	1.000000	1.000
534	GOBP_CELL_GROWTH	GO:0016049	10	-0.356386	-1.029903	0.432773	1.000000	1.000
535	GOBP_POSITIVE_REGULATION_OF_CYSTEINE_TYPE_ENDO...	GO:2001056	7	-0.395871	-1.032287	0.452489	1.000000	1.000
536	GOBP_REGULATION_OF_NEUROTRANSMITTER_TRANSPORT	GO:0051588	6	-0.414634	-1.032350	0.406907	1.000000	1.000
537	REACTOME_LEISHMANIA_INFECTION	R-HSA-9658195	10	-0.347032	-1.033476	0.425034	1.000000	1.000
538	REACTOME_NERVOUS_SYSTEM_DEVELOPMENT	R-RNO-9675108	14	-0.316682	-1.034492	0.423288	1.000000	1.000
539	REACTOME_MAPK_TARGETS_NUCLEAR_EVENTS_MEDIATED_...	R-HSA-450282	5	-0.443061	-1.034742	0.418182	1.000000	1.000
540	GOBP_LEUKOCYTE_MEDIATED_IMMUNITY	GO:0002443	8	-0.382515	-1.035226	0.419971	1.000000	1.000
541	GOBP_INTRACELLULAR_TRANSPORT	GO:0046907	10	-0.352990	-1.041010	0.437946	1.000000	1.000
542	GOBP_WOUND_HEALING	GO:0042060	13	-0.320977	-1.043331	0.402579	1.000000	1.000
543	GOBP_NEGATIVE_REGULATION_OF_PHOSPHORYLATION	GO:0042326	7	-0.398240	-1.045222	0.429395	1.000000	1.000
544	GOBP_REGULATION_OF_NERVOUS_SYSTEM_PROCESS	GO:0031644	8	-0.386354	-1.045414	0.407143	1.000000	1.000
545	GOBP_IMMUNE_RESPONSE_REGULATING_SIGNALING_PATHWAY	GO:0002764	8	-0.386391	-1.050992	0.400875	1.000000	1.000
546	GOBP_CIRCADIAN_REGULATION_OF_GENE_EXPRESSION	GO:0032922	5	-0.451813	-1.053837	0.390840	1.000000	1.000
547	GOBP_PROTEOLYSIS	GO:0006508	17	-0.306120	-1.056996	0.397919	1.000000	1.000
548	GOBP_REGULATION_OF_TRANSPORTER_ACTIVITY	GO:0032409	17	-0.304602	-1.058924	0.400268	1.000000	1.000
549	GOBP_FC_RECEPTOR_SIGNALING_PATHWAY	GO:0038093	8	-0.386391	-1.059190	0.402878	1.000000	1.000
550	GOBP_LIPID_LOCALIZATION	GO:0010876	10	-0.366699	-1.061920	0.372024	1.000000	1.000
551	GOBP_INTRACELLULAR_PROTEIN_TRANSPORT	GO:0006886	8	-0.394335	-1.062952	0.396226	1.000000	1.000
552	GOBP_LIPID_EXPORT_FROM_CELL	GO:0140353	5	-0.456163	-1.066032	0.412873	1.000000	1.000
553	REACTOME_INTERFERON_SIGNALING	R-RNO-913531	6	-0.425840	-1.067031	0.391705	1.000000	1.000
554	REACTOME_TRANSCRIPTIONAL_REGULATION_BY_MECP2	R-RNO-8986944	7	-0.407407	-1.068152	0.375546	1.000000	1.000
555	GOBP_POSITIVE_REGULATION_OF_SECRETION	GO:0051047	6	-0.429704	-1.071394	0.377001	1.000000	1.000
556	REACTOME_CIRCADIAN_CLOCK	R-HSA-400253	5	-0.457831	-1.074296	0.379464	1.000000	1.000

Continuation of Table **S2**

	NAME	ID	SIZE	ES	NES	NOM.p.val	FDR.q.val	FWER.p.val
557	GOBP_CELLULAR_RESPONSE_TO_PEPTIDE_HORMONE_STIM...	GO:0071375	9	-0.379408	-1.076934	0.357771	0.998661	1.000
558	GOBP_REGULATION_OF_PROTEIN_LOCALIZATION	GO:0032880	18	-0.313043	-1.077578	0.377238	1.000000	1.000
559	GOBP_CELLULAR_RESPONSE_TO_ALCOHOL	GO:0097306	5	-0.470464	-1.078167	0.399096	1.000000	1.000
560	GOBP_REGULATION_OF_PEPTIDE_TRANSPORT	GO:0090087	10	-0.367655	-1.080792	0.361149	1.000000	1.000
561	GOBP_REGULATION_OF_SYSTEM_PROCESS	GO:0044057	18	-0.310188	-1.083597	0.358768	1.000000	1.000
562	REACTOME_DAG_AND_IP3_SIGNALING	R-RNO-1489509	8	-0.398245	-1.084336	0.355958	1.000000	1.000
563	GOBP_MUSCLE_CONTRACTION	GO:0006936	5	-0.475086	-1.084432	0.360182	1.000000	1.000
564	GOBP_PLATELET_ACTIVATION	GO:0030168	6	-0.439645	-1.085277	0.362952	1.000000	1.000
565	GOBP_SYNAPTIC_SIGNALING	GO:0099536	47	-0.268839	-1.085597	0.375154	1.000000	1.000
566	GOBP_POSITIVE_REGULATION_OF_LOCOMOTION	GO:0040017	11	-0.360755	-1.085955	0.370319	1.000000	1.000
567	REACTOME_L1CAM_INTERACTIONS	R-RNO-373760	5	-0.463606	-1.087414	0.368657	1.000000	1.000
568	GOBP_CARBOHYDRATE_DERIVATIVE_BIOSYNTHETIC_PROCESS	GO:1901137	5	-0.465896	-1.088899	0.350388	1.000000	1.000
569	REACTOME_INTERLEUKIN_17_SIGNALING	R-RNO-448424	6	-0.439454	-1.088975	0.347181	1.000000	1.000
570	GOBP_REGULATION_OF_NEUROTRANSMITTER_LEVELS	GO:0001505	7	-0.419753	-1.092002	0.350725	1.000000	1.000
571	GOBP_FATTY_ACID_TRANSPORT	GO:0015908	6	-0.445646	-1.105253	0.335294	0.992244	1.000
572	GOBP_NEUROTRANSMITTER_TRANSPORT	GO:0006836	7	-0.419753	-1.105305	0.330484	0.998572	1.000
573	GOBP_GAMETE_GENERATION	GO:0007276	9	-0.386609	-1.107766	0.336662	0.996697	1.000
574	GOBP_REGULATION_OF_MUSCLE_ADAPTATION	GO:0043502	5	-0.481928	-1.109226	0.330769	0.998466	1.000
575	GOBP_HOMEOSTATIC_PROCESS	GO:0042592	31	-0.279663	-1.110197	0.339552	1.000000	1.000
576	REACTOME_RNA_POLYMERASE_II_TRANSCRIPTION	R-RNO-73857	20	-0.310016	-1.111509	0.328125	1.000000	1.000
577	GOBP_MUSCLE_ADAPTATION	GO:0043500	5	-0.481928	-1.115123	0.333866	0.998563	1.000
578	GOBP_PROTEIN_LOCALIZATION_TO_SYNAPSE	GO:0035418	5	-0.476798	-1.116602	0.320669	1.000000	1.000
579	GOBP_NEGATIVE_REGULATION_OF_RESPONSE_TO_EXTERN...	GO:0032102	5	-0.485600	-1.117970	0.324409	1.000000	1.000
580	GOBP_MONOCARBOXYLIC_ACID_TRANSPORT	GO:0015718	5	-0.485881	-1.119902	0.312694	1.000000	1.000
581	GOBP_NEGATIVE_REGULATION_OF_ANION_TRANSPORT	GO:1903792	8	-0.409779	-1.120853	0.338663	1.000000	1.000
582	GOBP_ACIDIC_AMINO_ACID_TRANSPORT	GO:0015800	5	-0.485881	-1.124610	0.302290	1.000000	1.000
583	GOBP_PROTEIN_LOCALIZATION_TO_POSTSYNAPSE	GO:0062237	5	-0.476798	-1.125301	0.302067	1.000000	1.000
584	GOBP_SECRETION	GO:0046903	24	-0.304839	-1.129857	0.301405	0.996688	1.000
585	GOBP_DICARBOXYLIC_ACID_TRANSPORT	GO:0006835	5	-0.485881	-1.130764	0.293395	1.000000	1.000
586	GOBP_REGULATION_OF_CELL_ADHESION	GO:0030155	13	-0.354813	-1.131292	0.293706	1.000000	1.000
587	GOBP_REGULATION_OF_TRANSPORT	GO:0051049	44	-0.278761	-1.131678	0.321770	1.000000	1.000
588	GOBP_INORGANIC_ION_TRANSMEMBRANE_TRANSPORT	GO:0098660	9	-0.394516	-1.134350	0.302726	1.000000	1.000
589	REACTOME_BETA_CATENIN_INDEPENDENT_WNT_SIGNALING	R-RNO-3858494	5	-0.493976	-1.137589	0.287591	1.000000	1.000
590	GOBP_CALCIUM_ION_TRANSPORT_INTO_CYTOSOL	GO:0060402	7	-0.438199	-1.138242	0.293413	1.000000	1.000
591	GOBP_REGULATION_OF_VASCULATURE_DEVELOPMENT	GO:1901342	5	-0.493884	-1.139157	0.303266	1.000000	1.000
592	GOBP_SENSORY_ORGAN_DEVELOPMENT	GO:0007423	8	-0.418031	-1.139854	0.307359	1.000000	1.000
593	REACTOME_RHO_GTPASE_EFFECTORS	R-RNO-195258	9	-0.404701	-1.146367	0.271041	1.000000	1.000
594	GOBP_CALCIUM_ION_TRANSMEMBRANE_TRANSPORT	GO:0070588	7	-0.438199	-1.146976	0.296407	1.000000	1.000
595	GOBP_REGULATION_OF_POSTSYNAPSE_ORGANIZATION	GO:0099175	6	-0.457235	-1.147920	0.295666	1.000000	1.000
596	GOBP_DENDRITE_MORPHOGENESIS	GO:0048813	7	-0.442763	-1.149962	0.295154	1.000000	1.000
597	GOBP_CYTOSOLIC_CALCIUM_ION_TRANSPORT	GO:0060401	7	-0.438199	-1.150805	0.300310	1.000000	1.000
598	GOBP_MUSCLE_SYSTEM_PROCESS	GO:0003012	9	-0.405094	-1.153083	0.287749	1.000000	1.000
599	GOBP_REGULATION_OF_BODY_FLUID_LEVELS	GO:0050878	11	-0.379200	-1.156773	0.297965	1.000000	1.000

Continuation of Table **S2**

	NAME	ID	SIZE	ES	NES	NOM.p.val	FDR.q.val	FWER.p.val
600	GOBP_CENTRAL_NERVOUS_SYSTEM_DEVELOPMENT	GO:0007417	27	-0.301034	-1.157940	0.303371	1.000000	1.000
601	GOBP_CARBOHYDRATE_HOMEOSTASIS	GO:0033500	7	-0.440064	-1.162099	0.263868	1.000000	1.000
602	REACTOME_G_ALPHA_Z_SIGNALLING_EVENTS	R-RNO-418597	5	-0.493976	-1.162235	0.267405	1.000000	1.000
603	REACTOME_TRANSCRIPTIONAL_REGULATION_BY_TP53	R-RNO-3700989	6	-0.470448	-1.164079	0.276596	1.000000	1.000
604	REACTOME_SIGNALING_BY_RHO_GTPASES_MIRO_GTPASES...	R-RNO-9716542	9	-0.404701	-1.166495	0.264012	1.000000	1.000
605	GOBP_CELL_CELL_SIGNALING_BY_WNT	GO:0198738	7	-0.436764	-1.168311	0.251841	1.000000	1.000
606	GOBP_COAGULATION	GO:0050817	8	-0.423395	-1.169896	0.279494	1.000000	1.000
607	GOBP_RESPONSE_TO_WOUNDING	GO:0009611	15	-0.349273	-1.171171	0.272849	1.000000	1.000
608	GOBP_ORGANOPHOSPHATE_BIOSYNTHETIC_PROCESS	GO:0090407	5	-0.505945	-1.175941	0.258970	1.000000	1.000
609	GOBP_REGULATION_OF_MUSCLE_SYSTEM_PROCESS	GO:0090257	8	-0.434877	-1.175997	0.237313	1.000000	1.000
610	REACTOME_PTEN_REGULATION	R-RNO-6807070	5	-0.506090	-1.177432	0.254290	1.000000	1.000
611	GOBP_REGULATION_OF_SYNAPTIC_PLASTICITY	GO:0048167	21	-0.325921	-1.177620	0.282638	1.000000	1.000
612	GOBP_SEXUAL_REPRODUCTION	GO:0019953	10	-0.398252	-1.179922	0.283804	1.000000	1.000
613	GOBP_CELL_CELL_SIGNALING	GO:0007267	56	-0.298321	-1.183036	0.266586	1.000000	1.000
614	GOBP_GROWTH	GO:0040007	14	-0.365982	-1.183077	0.272727	1.000000	1.000
615	GOBP_TRANSMEMBRANE_TRANSPORT	GO:0055085	27	-0.309359	-1.188971	0.259823	1.000000	1.000
616	GOBP_REGULATION_OF_SYNAPTIC_TRANSMISSION_GLUTA...	GO:0051966	12	-0.385135	-1.190906	0.267568	1.000000	1.000
617	GOBP_NUCLEOSIDE_PHOSPHATE_BIOSYNTHETIC_PROCESS	GO:1901293	5	-0.505945	-1.192798	0.241538	1.000000	1.000
618	REACTOME_FC_EPSILON_RECEPTOR_FCERI_SIGNALING	R-RNO-2454202	7	-0.462802	-1.197427	0.215152	1.000000	1.000
619	REACTOME_G_ALPHA_Q_SIGNALLING_EVENTS	R-RNO-416476	6	-0.477054	-1.199992	0.234824	1.000000	1.000
620	GOBP_REGULATION_OF_TRANS_SYNAPTIC_SIGNALING	GO:0099177	40	-0.293992	-1.203099	0.240903	1.000000	1.000
621	GOBP_DENDRITE_DEVELOPMENT	GO:0016358	10	-0.403987	-1.207640	0.218077	1.000000	1.000
622	GOBP_CHROMOSOME_ORGANIZATION	GO:0051276	6	-0.490371	-1.210312	0.232456	1.000000	1.000
623	GOBP_INNATE_IMMUNE_RESPONSE	GO:0045087	7	-0.467437	-1.215455	0.232170	1.000000	1.000
624	GOBP_FC_EPSILON_RECEPTOR_SIGNALING_PATHWAY	GO:0038095	7	-0.462802	-1.221193	0.243478	0.988504	1.000
625	GOBP_NEGATIVE_REGULATION_OF_CELL_PROJECTION_OR...	GO:0031345	5	-0.527500	-1.223418	0.207317	0.989456	1.000
626	GOBP_LOCOMOTORY_BEHAVIOR	GO:0007626	8	-0.454188	-1.225827	0.191176	0.989916	1.000
627	REACTOME_TRAFFICKING_OF_GLUR2_CONTAINING_AMPA_...	R-RNO-416993	8	-0.450000	-1.227917	0.176642	0.991887	1.000
628	GOBP_HEAD_DEVELOPMENT	GO:0060322	23	-0.330315	-1.227920	0.208333	1.000000	1.000
629	GOBP_SIGNAL_RELEASE	GO:0023061	14	-0.379726	-1.236594	0.193681	0.977540	1.000
630	GOBP_REGULATION_OF_SECRETION	GO:0051046	14	-0.379856	-1.240428	0.191432	0.972704	1.000
631	GOBP_NEUROTRANSMITTER_SECRETION	GO:0007269	6	-0.500000	-1.241094	0.207831	0.980484	1.000
632	GOBP_MALE_GAMETE_GENERATION	GO:0048232	5	-0.542169	-1.246496	0.203101	0.969499	1.000
633	GOBP_CELLULAR_MACROMOLECULE_CATABOLIC_PROCESS	GO:0044265	5	-0.544343	-1.252644	0.209231	0.955066	1.000
634	GOBP_REGULATION_OF_CELL_CYCLE_PROCESS	GO:0010564	5	-0.550414	-1.252782	0.206231	0.965091	1.000
635	GOBP_CELLULAR_RESPONSE_TO_KETONE	GO:1901655	5	-0.534818	-1.255053	0.170404	0.966587	1.000
636	GOBP_CHEMICAL_SYNAPTIC_TRANSMISSION_POSTSYNAPTIC	GO:0099565	9	-0.451440	-1.267849	0.174962	0.924492	1.000
637	GOBP_RESPONSE_TO_ALCOHOL	GO:0097305	11	-0.423741	-1.297619	0.173554	0.819952	1.000
638	GOBP_CELL_CYCLE	GO:0007049	21	-0.352648	-1.297864	0.158365	0.828658	1.000
639	GOBP_CALCIUM_ION_TRANSPORT	GO:0006816	11	-0.429318	-1.298588	0.150000	0.835324	1.000
640	GOBP_CELL_SURFACE_RECEPTOR_SIGNALING_PATHWAY_I...	GO:1905114	15	-0.397237	-1.300695	0.160265	0.837640	1.000
641	GOBP_PROTEIN_LOCALIZATION_TO_ORGANELLE	GO:0033365	8	-0.471388	-1.301144	0.167135	0.845867	1.000
642	GOBP_MITOTIC_CELL_CYCLE	GO:0000278	10	-0.447847	-1.308137	0.156780	0.829737	1.000

Continuation of Table **S2**

	NAME	ID	SIZE	ES	NES	NOM.p.val	FDR.q.val	FWER.p.val
643	REACTOME_HEMOSTASIS	R-RNO-109582	14	-0.403680	-1.309821	0.156000	0.833214	1.000
644	REACTOME_CA_DEPENDENT_EVENTS	R-RNO-111996	8	-0.475000	-1.311256	0.165906	0.838129	1.000
645	GOBP_RESPONSE_TO_TUMOR_NECROSIS_FACTOR	GO:0034612	7	-0.497987	-1.313266	0.152518	0.841031	1.000
646	GOBP_MAPK_CASCADE	GO:0000165	18	-0.379171	-1.316737	0.141145	0.838148	1.000
647	REACTOME_SIGNALING_BY_WNT	R-RNO-195721	7	-0.520046	-1.320924	0.140719	0.831779	1.000
648	GOBP_NUCLEAR_TRANSPORT	GO:0051169	5	-0.560217	-1.323734	0.122807	0.831657	1.000
649	GOBP_MEMBRANE_ORGANIZATION	GO:0061024	12	-0.420784	-1.328571	0.151344	0.823309	1.000
650	GOBP_NEGATIVE_REGULATION_OF_TRANSFERASE_ACTIVITY	GO:0051348	5	-0.571128	-1.330146	0.125937	0.827970	1.000
651	GOBP_ORGANIC_ACID_TRANSPORT	GO:0015849	7	-0.503540	-1.330361	0.141369	0.838356	1.000
652	GOBP_NEGATIVE_REGULATION_OF_KINASE_ACTIVITY	GO:0033673	5	-0.571128	-1.331859	0.120980	0.843636	1.000
653	GOBP_SYNAPTIC_TRANSMISSION_GLUTAMATERGIC	GO:0035249	13	-0.418705	-1.332131	0.160221	0.854343	1.000
654	GOBP_ORGANIC_ANION_TRANSPORT	GO:0015711	6	-0.537694	-1.334938	0.135053	0.854107	1.000
655	GOBP_METAL_ION_TRANSPORT	GO:0030001	11	-0.429318	-1.336007	0.148966	0.861508	1.000
656	GOBP_CARBOXYLIC_ACID_TRANSPORT	GO:0046942	6	-0.537694	-1.338010	0.131148	0.865587	1.000
657	REACTOME_CREB1_PHOSPHORYLATION_THROUGH_NMDA_RE...	R-RNO-442742	8	-0.485388	-1.341865	0.127566	0.862523	1.000
658	GOBP_PROTEIN_TETRAMERIZATION	GO:0051262	5	-0.578313	-1.342770	0.111635	0.871855	1.000
659	REACTOME_CELL_CYCLE_MITOTIC	R-RNO-69278	5	-0.578650	-1.343701	0.134441	0.881371	1.000
660	GOBP_POSTTRANSCRIPTIONAL_REGULATION_OF_GENE_EX...	GO:0010608	8	-0.490146	-1.344707	0.130435	0.890775	1.000
661	GOBP_AMINO_ACID_TRANSPORT	GO:0006865	6	-0.537694	-1.355343	0.102326	0.860527	1.000
662	GOBP_CELLULAR_GLUCOSE_HOMEOSTASIS	GO:0001678	5	-0.592096	-1.362074	0.115152	0.845778	1.000
663	GOBP_ESTABLISHMENT_OF_PROTEIN_LOCALIZATION_TO_...	GO:0072594	5	-0.583592	-1.368286	0.090352	0.833278	1.000
664	GOBP_PROTEIN_COMPLEX_OLIGOMERIZATION	GO:0051259	7	-0.530725	-1.378353	0.127193	0.806843	1.000
665	GOBP_SECOND_MESSENGER_MEDIATED_SIGNALING	GO:0019932	16	-0.413385	-1.381192	0.105820	0.808792	1.000
666	REACTOME_INTRACELLULAR_SIGNALING_BY_SECOND_MES...	R-RNO-9006925	15	-0.417131	-1.381440	0.111842	0.821499	1.000
667	REACTOME_G_PROTEIN_MEDIATED_EVENTS	R-RNO-112040	9	-0.481013	-1.382837	0.100730	0.830294	1.000
668	GOBP_DIVALENT_INORGANIC_CATION_HOMEOSTASIS	GO:0072507	13	-0.442679	-1.387292	0.114441	0.826272	1.000
669	GOBP_REGULATION_OF_ANION_TRANSPORT	GO:0044070	20	-0.386788	-1.396394	0.100000	0.802443	1.000
670	GOBP_REGULATION_OF_CYTOSOLIC_CALCIUM_ION_CONCE...	GO:0051480	13	-0.442679	-1.400049	0.108871	0.800699	1.000
671	GOBP_PROTEIN_CONTAINING_COMPLEX_SUBUNIT_ORGANI...	GO:0043933	15	-0.421908	-1.401436	0.096317	0.810104	1.000
672	REACTOME_NEUREXINS_AND_NEUROLIGINS	R-RNO-6794361	9	-0.493462	-1.402112	0.103448	0.822604	1.000
673	GOBP_METAL_ION_HOMEOSTASIS	GO:0055065	14	-0.436559	-1.406730	0.099329	0.819380	1.000
674	REACTOME_CELL_CYCLE	R-RNO-1640170	6	-0.572810	-1.408553	0.082171	0.827412	1.000
675	GOBP_EXCITATORY_CHEMICAL_SYNAPTIC_TRANSMISSION	GO:0098976	5	-0.599664	-1.408622	0.104596	0.843729	1.000
676	GOBP_NEGATIVE_REGULATION_OF_MULTICELLULAR_ORGA...	GO:0051241	15	-0.420879	-1.409943	0.100271	0.854781	1.000
677	REACTOME_RAS_ACTIVATION_UPON_CA2_INFLUX_THROUG...	R-HSA-442982	6	-0.573171	-1.410257	0.094595	0.871169	1.000
678	GOBP_REGULATION_OF_SIGNALING_RECEPTOR_ACTIVITY	GO:0010469	14	-0.432647	-1.420007	0.098274	0.846374	1.000
679	REACTOME_TRAFFICKING_OF_AMPA_RECEPTORS	R-RNO-399719	11	-0.467532	-1.420179	0.079498	0.863845	1.000
680	REACTOME_CLASS_C_3_METABOTROPIC_GLUTAMATE_PHER...	R-RNO-420499	7	-0.555922	-1.426068	0.084746	0.855601	1.000
681	REACTOME_ASSEMBLY_AND_CELL_SURFACE_PRESENTATIO...	R-RNO-9609736	8	-0.519388	-1.430865	0.074561	0.854270	1.000
682	REACTOME_MAPK_FAMILY_SIGNALING_CASCADES	R-RNO-5683057	12	-0.459035	-1.432120	0.070442	0.868940	1.000
683	GOBP_REGULATION_OF_CATION_CHANNEL_ACTIVITY	GO:2001257	14	-0.434545	-1.433049	0.083333	0.885145	1.000
684	REACTOME_GPCR_LIGAND_BINDING	R-RNO-500792	8	-0.530619	-1.438779	0.077037	0.880155	1.000
685	REACTOME_NEGATIVE_REGULATION_OF_NMDA_RECEPTOR_...	R-HSA-9617324	8	-0.519388	-1.440190	0.089443	0.895385	1.000

Continuation of Table **S2**

	NAME	ID	SIZE	ES	NES	NOM.p.val	FDR.q.val	FWER.p.val
686	GOBP_CHEMICAL_HOMEOSTASIS	GO:0048878	23	-0.388955	-1.444721	0.078844	0.896240	1.000
687	GOBP_REGULATION_OF_CATION_TRANSMEMBRANE_TRANSPORT	GO:1904062	16	-0.428081	-1.460395	0.071146	0.843066	1.000
688	REACTOME_ADAPTIVE_IMMUNE_SYSTEM	R-RNO-1280218	9	-0.509115	-1.463810	0.068376	0.850692	1.000
689	GOBP_CATION_TRANSMEMBRANE_TRANSPORT	GO:0098655	18	-0.423251	-1.465961	0.058589	0.864340	1.000
690	GOBP_ION_HOMEOSTASIS	GO:0050801	16	-0.437862	-1.473055	0.072776	0.854910	1.000
691	GOBP_CELLULAR_COMPONENT_MAINTENANCE	GO:0043954	5	-0.627201	-1.477436	0.055046	0.856431	1.000
692	GOBP_SENSORY_PERCEPTION_OF_PAIN	GO:0019233	5	-0.638554	-1.487454	0.050671	0.832943	1.000
693	GOBP_ENDOMEMBRANE_SYSTEM_ORGANIZATION	GO:0010256	6	-0.603065	-1.493016	0.046990	0.831064	1.000
694	GOBP_RESPONSE_TO_ETHANOL	GO:0045471	5	-0.638554	-1.494116	0.048601	0.852698	1.000
695	GOBP_CELLULAR_ION_HOMEOSTASIS	GO:0006873	14	-0.466689	-1.521191	0.061026	0.750830	1.000
696	REACTOME_INTEGRATION_OF_ENERGY_METABOLISM	R-RNO-163685	5	-0.661504	-1.539973	0.045741	0.694127	1.000
697	GOBP_REGULATION_OF_MEMBRANE_POTENTIAL	GO:0042391	17	-0.450870	-1.544169	0.042497	0.700519	1.000
698	GOBP_G_PROTEIN_COUPLED_GLUTAMATE_RECEPTOR_SIGN...	GO:0007216	8	-0.582439	-1.570704	0.026746	0.615723	1.000
699	REACTOME_OPIOID_SIGNALLING	R-RNO-111885	12	-0.500000	-1.571680	0.039945	0.636165	1.000
700	GOBP_REGULATION_OF_NEUROTRANSMITTER_RECEPTOR_A...	GO:0099601	13	-0.494573	-1.577922	0.040166	0.635698	1.000
701	GOBP_REGULATION_OF_POSTSYNAPTIC_MEMBRANE_POTEN...	GO:0060078	14	-0.488935	-1.592668	0.046113	0.606039	1.000
702	REACTOME_LONG_TERM_POTENTIATION	R-HSA-9620244	10	-0.546232	-1.608867	0.020576	0.567000	1.000
703	GOBP_CELLULAR_HOMEOSTASIS	GO:0019725	18	-0.455897	-1.619541	0.035573	0.551034	1.000
704	REACTOME_SYNAPTIC_ADHESION_LIKE_MOLECULES	R-RNO-8849932	9	-0.573582	-1.634053	0.029070	0.521470	1.000
705	GOBP_REGULATION_OF_NMDA_RECEPTOR_ACTIVITY	GO:2000310	10	-0.581937	-1.678013	0.011348	0.402719	1.000
706	GOBP_ADENYLATE_CYCLASE_INHIBITING_G_PROTEIN_CO...	GO:0007196	5	-0.723347	-1.682956	0.015699	0.410429	1.000
707	GOBP_REGULATION_OF_ION_TRANSPORT	GO:0043269	36	-0.419160	-1.686929	0.030265	0.420669	1.000
708	GOBP_G_PROTEIN_COUPLED_RECEPTOR_SIGNALING_PATHWAY	GO:0007186	18	-0.484188	-1.705699	0.015831	0.388496	1.000
709	REACTOME_PROTEIN_PROTEIN_INTERACTIONS_AT_SYNAPSES	R-RNO-6794362	12	-0.549302	-1.712471	0.011299	0.392093	1.000
710	GOBP_ADENYLATE_CYCLASE_MODULATING_G_PROTEIN_CO...	GO:0007188	11	-0.567980	-1.724652	0.014306	0.380440	1.000
711	GOBP_NEGATIVE_REGULATION_OF_GENE_EXPRESSION	GO:0010629	11	-0.570191	-1.742220	0.008511	0.357916	0.995
712	REACTOME_ACTIVATION_OF_NMDA_RECEPTORS_AND_POST...	R-RNO-442755	16	-0.512241	-1.755589	0.011984	0.344880	0.993
713	GOBP_ADENYLATE_CYCLASE_INHIBITING_G_PROTEIN_CO...	GO:0007193	9	-0.614078	-1.767819	0.007143	0.338171	0.992
714	REACTOME_SIGNALING_BY_GPCR	R-RNO-372790	22	-0.483142	-1.772512	0.003916	0.355531	0.991
715	REACTOME_UNBLOCKING_OF_NMDA_RECEPTORS_GLUTAMAT...	R-RNO-438066	12	-0.570093	-1.779374	0.011080	0.368191	0.986
716	REACTOME_G_ALPHA_I_SIGNALLING_EVENTS	R-RNO-418594	18	-0.515174	-1.786708	0.010652	0.386627	0.979
717	GOBP_CATION_TRANSPORT	GO:0006812	25	-0.477040	-1.795458	0.003876	0.404711	0.972
718	GOBP_IONOTROPIC_GLUTAMATE_RECEPTOR_SIGNALING_P...	GO:0035235	9	-0.625297	-1.801502	0.008596	0.440991	0.966
719	GOBP_CELL_CYCLE_PROCESS	GO:0022402	11	-0.606056	-1.874702	0.002729	0.267976	0.848
720	REACTOME_NEUROTRANSMITTER_RECEPTORS_AND_POSTSY...	R-RNO-112314	22	-0.510420	-1.905832	0.003802	0.239683	0.748
721	GOBP_SENSORY_PERCEPTION	GO:0007600	9	-0.670886	-1.909889	0.001422	0.289396	0.735
722	REACTOME_TRANSMISSION_ACROSS_CHEMICAL_SYNAPSES	R-RNO-112315	23	-0.519574	-1.926571	0.003856	0.325049	0.667
723	REACTOME_NEURONAL_SYSTEM	R-RNO-112316	26	-0.531507	-2.014244	0.001255	0.211187	0.374
724	GOBP_GLUTAMATE_RECEPTOR_SIGNALING_PATHWAY	GO:0007215	17	-0.646270	-2.239019	0.000000	0.031969	0.036

End of Table

Supplementary Table S3: SC rostral segment early (8 DPL peak) profile GSEA results.

Begin of Table **S3**

	NAME	ID	SIZE	ES	NES	NOM.p.val	FDR.q.val	FWER.p.val
0	REACTOME_INTERLEUKIN_4_AND_INTERLEUKIN_13_SIGN...	R-RNO-6785807	8	0.784993	2.518360	0.000000	0.021501	0.015
1	GOBP_NEGATIVE_REGULATION_OF_PEPTIDASE_ACTIVITY	GO:0010466	6	0.736709	2.130500	0.000000	0.257322	0.298
2	HALLMARK_TNFA_SIGNALING_VIA_NFKB	M5890	14	0.459459	1.908470	0.004831	0.688131	0.748
3	GOBP_POSITIVE_REGULATION_OF_CELL_DIFFERENTIATION	GO:0045597	27	0.353574	1.854106	0.000000	0.684561	0.836
4	GOBP_RESPONSE_TO_ENDOGENOUS_STIMULUS	GO:0009719	34	0.335154	1.838417	0.000000	0.588722	0.856
5	GOBP_NEGATIVE_REGULATION_OF_CYSTEINE_TYPE_ENDO...	GO:2000117	5	0.699344	1.801695	0.016892	0.588662	0.902
6	GOBP_OSTEOCLAST_DIFFERENTIATION	GO:0030316	6	0.622961	1.775699	0.013937	0.575180	0.930
7	GOBP_LEUKOCYTE_DIFFERENTIATION	GO:0002521	15	0.410959	1.771396	0.021622	0.513138	0.935
8	GOBP_POSITIVE_REGULATION_OF_SMOOTH_MUSCLE_CELL...	GO:0048661	5	0.674699	1.761438	0.022801	0.479749	0.948
9	GOBP_CYTOKINE_MEDIATED_SIGNALING_PATHWAY	GO:0019221	16	0.402778	1.742551	0.000000	0.472665	0.961
10	GOBP_OSTEOBLAST_DIFFERENTIATION	GO:0001649	5	0.674699	1.720435	0.026578	0.478233	0.979
11	GOBP_DEFENSE_RESPONSE	GO:0006952	19	0.376812	1.704846	0.012579	0.474040	0.984
12	REACTOME_SIGNALING_BY_INTERLEUKINS	R-RNO-449147	15	0.396585	1.683649	0.023474	0.484422	0.990
13	GOBP_BLOOD_VESSEL_MORPHOGENESIS	GO:0048514	14	0.405405	1.679468	0.021390	0.455829	0.991
14	REACTOME_CYTOKINE_SIGNALING_IN_IMMUNE_SYSTEM	R-RNO-1280215	21	0.338604	1.648508	0.018405	0.495027	0.996
15	GOBP_REGULATION_OF_CELL_DIFFERENTIATION	GO:0045595	36	0.308537	1.641018	0.000000	0.481683	0.998
16	GOBP_POSITIVE_REGULATION_OF_DEVELOPMENTAL_PROCESS	GO:0051094	32	0.300962	1.635074	0.008264	0.468198	0.998
17	GOBP_REGULATION_OF_HEMOPOIESIS	GO:1903706	10	0.461538	1.626675	0.037975	0.459145	1.000
18	GOBP_OSSIFICATION	GO:0001503	9	0.481444	1.623824	0.053571	0.439820	1.000
19	GOBP_CELLULAR_RESPONSE_TO_NITROGEN_COMPOUND	GO:1901699	20	0.333804	1.595932	0.017857	0.476835	1.000
20	GOBP_MYELOID_CELL_DIFFERENTIATION	GO:0030099	13	0.400000	1.595797	0.051282	0.454274	1.000
21	GOBP_ANATOMICAL_STRUCTURE_FORMATION_INVOLVED_I...	GO:0048646	20	0.336803	1.587974	0.023952	0.449877	1.000
22	GOBP_CELLULAR_RESPONSE_TO_PEPTIDE	GO:1901653	13	0.386667	1.565440	0.035398	0.481665	1.000
23	GOBP_PRI_MIRNA_TRANSCRIPTION_BY_RNA_POLYMERASE_II	GO:0061614	7	0.506173	1.561183	0.067568	0.470515	1.000
24	GOBP_POSITIVE_REGULATION_OF_CYTOKINE_PRODUCTION	GO:0001819	7	0.506173	1.561106	0.045627	0.451695	1.000
25	GOBP_NEGATIVE_REGULATION_OF_CELL_DEATH	GO:0060548	22	0.318182	1.557852	0.013158	0.441954	1.000
26	GOBP_REGULATION_OF_MYELOID_CELL_DIFFERENTIATION	GO:0045637	9	0.455696	1.555968	0.051724	0.429516	1.000
27	GOBP_REGULATION_OF_LEUKOCYTE_DIFFERENTIATION	GO:1902105	9	0.455696	1.545615	0.029787	0.434428	1.000
28	GOBP_POSITIVE_REGULATION_OF_PRI_MIRNA_TRANSCRI...	GO:1902895	7	0.506173	1.535649	0.054054	0.440023	1.000
29	GOBP_MONONUCLEAR_CELL_DIFFERENTIATION	GO:1903131	7	0.493827	1.520827	0.040161	0.455997	1.000
30	GOBP_CELL_POPULATION_PROLIFERATION	GO:0008283	32	0.265636	1.491342	0.042017	0.505450	1.000
31	HALLMARK_ALLOGRAFT_REJECTION	M5950	7	0.493827	1.488915	0.067857	0.495542	1.000
32	GOBP_REGULATION_OF_OSTEOCLAST_DIFFERENTIATION	GO:0045670	5	0.564178	1.474750	0.083333	0.512239	1.000
33	GOBP_REGULATION_OF_MYELOID_LEUKOCYTE_DIFFERENT...	GO:0002761	8	0.450000	1.466358	0.092308	0.516134	1.000
34	GOBP_AMEBOIDAL_TYPE_CELL_MIGRATION	GO:0001667	11	0.389610	1.464363	0.070423	0.505857	1.000
35	GOBP_RAS_PROTEIN_SIGNAL_TRANSDUCTION	GO:0007265	5	0.578313	1.460845	0.076923	0.499188	1.000
36	GOBP_NEGATIVE_REGULATION_OF_CELL_POPULATION_PR...	GO:0008285	9	0.431287	1.460354	0.073684	0.487369	1.000
37	GOBP_MYELOID_LEUKOCYTE_DIFFERENTIATION	GO:0002573	11	0.389610	1.443364	0.074890	0.514811	1.000
38	GOBP_CELLULAR_RESPONSE_TO_OXYGEN_CONTAINING_CO...	GO:1901701	29	0.267083	1.424809	0.043103	0.541622	1.000
39	REACTOME_TRANSCRIPTIONAL_REGULATION_OF_WHITE_A...	R-RNO-381340	6	0.500000	1.424755	0.073171	0.528197	1.000
40	GOBP_RESPONSE_TO_MECHANICAL_STIMULUS	GO:0009612	6	0.500000	1.417183	0.103679	0.532377	1.000

Continuation of Table **S3**

	NAME	ID	SIZE	ES	NES	NOM.p.val	FDR.q.val	FWER.p.val
41	GOBP_POSITIVE_REGULATION_OF_TRANSCRIPTION_BY_R...	GO:0045944	24	0.282914	1.412818	0.073826	0.530247	1.000
42	GOBP_RESPONSE_TO_PEPTIDE	GO:1901652	18	0.309449	1.411098	0.089385	0.521702	1.000
43	GOBP_RESPONSE_TO_CYTOKINE	GO:0034097	23	0.279196	1.404905	0.061728	0.523425	1.000
44	REACTOME_DEATH_RECEPTOR_SIGNALLING	R-RNO-73887	5	0.534155	1.403333	0.125000	0.515704	1.000
45	GOBP_POSITIVE_REGULATION_OF_MULTICELLULAR_ORGA...	GO:0051240	32	0.251094	1.398258	0.076271	0.515165	1.000
46	GOBP_POSITIVE_REGULATION_OF_NUCLEOBASE_CONTAIN...	GO:0045935	28	0.261989	1.383227	0.053846	0.538803	1.000
47	GOBP_LEUKOCYTE_CELL_CELL_ADHESION	GO:0007159	6	0.500000	1.380752	0.099678	0.533505	1.000
48	GOBP_CELLULAR_RESPONSE_TO_HORMONE_STIMULUS	GO:0032870	11	0.376623	1.374529	0.106667	0.536641	1.000
49	GOBP_IMMUNE_SYSTEM_DEVELOPMENT	GO:0002520	17	0.316293	1.369192	0.064103	0.538479	1.000
50	GOBP_CYTOKINE_PRODUCTION	GO:0001816	10	0.384615	1.363603	0.093220	0.541880	1.000
51	GOBP_NEGATIVE_REGULATION_OF_PROTEOLYSIS	GO:0045861	8	0.416564	1.360107	0.109929	0.538666	1.000
52	GOBP_INFLAMMATORY_RESPONSE	GO:0006954	12	0.355263	1.345273	0.146667	0.561388	1.000
53	GOBP_POSITIVE_REGULATION_OF_GENE_EXPRESSION	GO:0010628	16	0.308092	1.337342	0.080214	0.568983	1.000
54	GOBP_POSITIVE_REGULATION_OF_BIOSYNTHETIC_PROCESS	GO:0009891	27	0.247471	1.331844	0.084507	0.572267	1.000
55	GOBP_POSITIVE_REGULATION_OF_MYELOID_CELL_DIFFE...	GO:0045639	7	0.444444	1.327102	0.118081	0.573523	1.000
56	GOBP_POSITIVE_REGULATION_OF_HEMOPOIESIS	GO:1903708	7	0.444444	1.325982	0.132353	0.566240	1.000
57	GOBP_ANIMAL_ORGAN_MORPHOGENESIS	GO:0009887	12	0.343374	1.325744	0.112613	0.557122	1.000
58	GOBP_TISSUE_MIGRATION	GO:0090130	9	0.379747	1.315879	0.124464	0.570732	1.000
59	GOBP_CELLULAR_RESPONSE_TO_EXTRACELLULAR_STIMULUS	GO:0031668	8	0.418845	1.312738	0.170040	0.568363	1.000
60	HALLMARK_HYPOXIA	M5891	5	0.506880	1.307240	0.125828	0.572002	1.000
61	GOBP_CELLULAR_RESPONSE_TO_EXTERNAL_STIMULUS	GO:0071496	10	0.360491	1.305539	0.125561	0.566353	1.000
62	GOBP_FAT_CELL_DIFFERENTIATION	GO:0045444	9	0.379747	1.285547	0.162500	0.605189	1.000
63	GOBP_POSITIVE_REGULATION_OF_CELL_POPULATION_PR...	GO:0008284	21	0.267349	1.284313	0.143713	0.598937	1.000
64	GOBP_REGULATION_OF_ANION_TRANSMEMBRANE_TRANSPORT	GO:1903959	5	0.506024	1.282164	0.165079	0.594726	1.000
65	GOBP_POSITIVE_REGULATION_OF_CELL_CELL_ADHESION	GO:0022409	5	0.493976	1.271073	0.168498	0.612620	1.000
66	GOBP_PROTEIN_PHOSPHORYLATION	GO:0006468	33	0.225391	1.267578	0.119266	0.612100	1.000
67	GOBP_HOMEOSTASIS_OF_NUMBER_OF_CELLS	GO:0048872	5	0.481928	1.246952	0.190789	0.655338	1.000
68	GOBP_ENDOTHELIAL_CELL_PROLIFERATION	GO:0001935	8	0.375000	1.242722	0.160338	0.656854	1.000
69	GOBP_POSITIVE_REGULATION_OF_LEUKOCYTE_CELL_CEL...	GO:1903039	5	0.493976	1.242314	0.207792	0.648520	1.000
70	GOBP_SKIN_DEVELOPMENT	GO:0043588	5	0.481928	1.241924	0.210000	0.640357	1.000
71	GOBP_POSITIVE_REGULATION_OF_MYELOID_LEUKOCYTE_...	GO:0002763	6	0.439024	1.240634	0.204620	0.635096	1.000
72	GOBP_ENDOTHELIAL_CELL_MIGRATION	GO:0043542	8	0.375000	1.232408	0.192453	0.646238	1.000
73	GOBP_DEFENSE_RESPONSE_TO_OTHER_ORGANISM	GO:0098542	9	0.367088	1.231780	0.184874	0.638975	1.000
74	GOBP_CIRCULATORY_SYSTEM_DEVELOPMENT	GO:0072359	21	0.255471	1.230069	0.168675	0.634345	1.000
75	GOBP_REGULATION_OF_LYMPHOCYTE_ACTIVATION	GO:0051249	5	0.481928	1.224121	0.215753	0.640098	1.000
76	GOBP_REGULATION_OF_MULTICELLULAR_ORGANISMAL_DE...	GO:2000026	31	0.222563	1.219608	0.156250	0.643005	1.000
77	GOBP_RESPONSE_TO_OXYGEN_LEVELS	GO:0070482	8	0.375000	1.213817	0.191571	0.649382	1.000
78	GOBP_NEGATIVE_REGULATION_OF_NEURON_DEATH	GO:1901215	11	0.324675	1.211510	0.187192	0.647465	1.000
79	REACTOME_DAG_AND_IP3_SIGNALING	R-RNO-1489509	8	0.362500	1.195847	0.219331	0.678175	1.000
80	GOBP_PEPTIDYL_AMINO_ACID_MODIFICATION	GO:0018193	22	0.242424	1.194104	0.191489	0.674729	1.000
81	GOBP_EPIDERMIS_DEVELOPMENT	GO:0008544	5	0.457831	1.194013	0.227891	0.666829	1.000
82	GOBP_BLOOD_VESSEL_ENDOTHELIAL_CELL_MIGRATION	GO:0043534	8	0.375000	1.182722	0.240964	0.687024	1.000
83	GOBP_NEUROTROPHIN_SIGNALING_PATHWAY	GO:0038179	6	0.406856	1.173142	0.254902	0.706098	1.000

Continuation of Table **S3**

	NAME	ID	SIZE	ES	NES	NOM.p.val	FDR.q.val	FWER.p.val
84	GOBP_MAINTENANCE_OF_LOCATION	GO:0051235	7	0.370370	1.155704	0.235294	0.742957	1.000
85	GOBP_NEUROTROPHIN_TRK_RECEPTOR_SIGNALING_PATHWAY	GO:0048011	6	0.406856	1.150246	0.270833	0.749556	1.000
86	GOBP_RESPONSE_TO_GROWTH_FACTOR	GO:0070848	18	0.252367	1.143544	0.245033	0.758093	1.000
87	GOBP_PROTEIN_MODIFICATION_BY_SMALL_PROTEIN_CON...	GO:0070647	7	0.370370	1.137912	0.256917	0.765820	1.000
88	GOBP_POSITIVE_REGULATION_OF_EPITHELIAL_CELL_PR...	GO:0050679	7	0.370370	1.137830	0.238636	0.757553	1.000
89	GOBP_RESPONSE_TO_BIOTIC_STIMULUS	GO:0009607	17	0.260590	1.133773	0.245714	0.759584	1.000
90	GOBP_RESPONSE_TO_INTERLEUKIN_1	GO:0070555	5	0.433735	1.132552	0.318493	0.754552	1.000
91	GOBP_RESPONSE_TO_HORMONE	GO:0009725	20	0.234854	1.123023	0.266304	0.772827	1.000
92	GOBP_ACTIN_FILAMENT_BASED_PROCESS	GO:0030029	7	0.362602	1.117692	0.286275	0.779794	1.000
93	GOBP_PERIPHERAL_NERVOUS_SYSTEM_DEVELOPMENT	GO:0007422	9	0.316456	1.115001	0.229885	0.778519	1.000
94	GOBP_CELLULAR_RESPONSE_TO_ABIOTIC_STIMULUS	GO:0071214	7	0.370370	1.114226	0.300752	0.772426	1.000
95	GOBP_CELL_CELL_JUNCTION_ASSEMBLY	GO:0007043	5	0.433735	1.111734	0.291096	0.771955	1.000
96	HALLMARK_KRAS_SIGNALING_UP	M5953	6	0.378049	1.104313	0.300366	0.783924	1.000
97	GOBP_NEGATIVE_REGULATION_OF_TRANSCRIPTION_BY_R...	GO:0000122	10	0.307692	1.103511	0.287611	0.778414	1.000
98	GOBP_TUBE_MORPHOGENESIS	GO:0035239	15	0.264046	1.100737	0.322917	0.778284	1.000
99	GOBP_REGULATION_OF_CELL_DEATH	GO:0010941	34	0.200167	1.098826	0.307018	0.775653	1.000
100	GOBP_NEGATIVE_REGULATION_OF_PROTEIN_METABOLIC_...	GO:0051248	20	0.234273	1.095817	0.328859	0.776192	1.000
101	GOBP_NERVE_DEVELOPMENT	GO:0021675	9	0.316456	1.083592	0.333333	0.802992	1.000
102	GOBP_MEMBRANE_PROTEIN_PROTEOLYSIS	GO:0033619	6	0.376527	1.081807	0.356115	0.799513	1.000
103	GOBP_POSITIVE_REGULATION_OF_CELLULAR_COMPONENT...	GO:0044089	9	0.316456	1.080978	0.313953	0.794155	1.000
104	GOBP_EMBRYO_DEVELOPMENT_ENDING_IN_BIRTH_OR_EGG...	GO:0009792	9	0.310423	1.079028	0.333333	0.791668	1.000
105	GOBP_INNATE_IMMUNE_RESPONSE	GO:0045087	7	0.358025	1.078314	0.373239	0.786562	1.000
106	GOBP_RESPONSE_TO_EXTRACELLULAR_STIMULUS	GO:0009991	13	0.257865	1.072498	0.356436	0.795439	1.000
107	HALLMARK_APOPTOSIS	M5902	6	0.378049	1.070394	0.336957	0.794524	1.000
108	GOBP_SPROUTING_ANGIOGENESIS	GO:0002040	5	0.409639	1.070221	0.353147	0.787765	1.000
109	GOBP_NEGATIVE_REGULATION_OF_MOLECULAR_FUNCTION	GO:0044092	24	0.205784	1.063543	0.373418	0.798017	1.000
110	GOBP_POSITIVE_REGULATION_OF_IMMUNE_SYSTEM_PROCESS	GO:0002684	18	0.228571	1.043406	0.407407	0.844361	1.000
111	GOBP_PROTEOLYSIS	GO:0006508	17	0.241463	1.042306	0.386740	0.839725	1.000
112	GOBP_REGULATION_OF_EPITHELIAL_CELL_MIGRATION	GO:0010632	6	0.365854	1.038968	0.359589	0.842330	1.000
113	GOBP_CELL_JUNCTION_ASSEMBLY	GO:0034329	8	0.312500	1.034395	0.377510	0.847999	1.000
114	GOBP_LIPID_BIOSYNTHETIC_PROCESS	GO:0008610	6	0.365854	1.033883	0.430034	0.842132	1.000
115	GOBP_POSITIVE_REGULATION_OF_ENDOTHELIAL_CELL_P...	GO:0001938	6	0.365854	1.032511	0.362595	0.838798	1.000
116	GOBP_REGULATION_OF_LIPID_BIOSYNTHETIC_PROCESS	GO:0046890	6	0.365854	1.032212	0.391608	0.832297	1.000
117	GOBP_ACTIVATION_OF_IMMUNE_RESPONSE	GO:0002253	5	0.392890	1.026945	0.431095	0.839562	1.000
118	GOBP_REGULATION_OF_PEPTIDYL_SERINE_PHOSPHORYLA...	GO:0033135	8	0.312500	1.026524	0.409420	0.833669	1.000
119	GOBP_LIPID_METABOLIC_PROCESS	GO:0006629	10	0.294872	1.024577	0.375546	0.832026	1.000
120	GOBP_REGULATION_OF_FAT_CELL_DIFFERENTIATION	GO:0045598	6	0.373094	1.024209	0.394366	0.825967	1.000
121	GOBP_RESPONSE_TO_DRUG	GO:0042493	11	0.272727	1.024064	0.412281	0.819641	1.000
122	REACTOME_INTERFERON_SIGNALING	R-RNO-913531	6	0.353659	1.019389	0.449477	0.826180	1.000
123	GOBP_PROTEIN_MODIFICATION_BY_SMALL_PROTEIN_CON...	GO:0032446	6	0.365854	1.019260	0.433566	0.819832	1.000
124	GOBP_POSITIVE_REGULATION_OF_AXONOGENESIS	GO:0050772	5	0.401522	1.016773	0.409375	0.819567	1.000
125	GOBP_REGULATION_OF_LIPID_METABOLIC_PROCESS	GO:0019216	7	0.333333	1.016174	0.396364	0.814257	1.000
126	GOBP_IN_UTERO_EMBRYONIC_DEVELOPMENT	GO:0001701	8	0.323991	1.015139	0.419913	0.810562	1.000

Continuation of Table **S3**

	NAME	ID	SIZE	ES	NES	NOM.p.val	FDR.q.val	FWER.p.val
127	GOBP_TRANSMEMBRANE_RECEPTOR_PROTEIN_SERINE_THR...	GO:0007178	6	0.365854	1.009609	0.439024	0.817176	1.000
128	GOBP_POSITIVE_REGULATION_OF_PEPTIDYL_SERINE_PH...	GO:0033138	8	0.312500	1.005618	0.441509	0.821200	1.000
129	GOBP_PROTEIN_KINASE_B_SIGNALING	GO:0043491	6	0.365854	1.005044	0.414966	0.816132	1.000
130	GOBP_NEUROINFLAMMATORY_RESPONSE	GO:0150076	5	0.385542	1.002477	0.460481	0.816751	1.000
131	GOBP_REGULATION_OF_DEFENSE_RESPONSE	GO:0031347	7	0.333333	0.998121	0.393939	0.821904	1.000
132	GOBP_VASCULATURE_DEVELOPMENT	GO:0001944	17	0.225352	0.994420	0.434066	0.825485	1.000
133	GOBP_NEGATIVE_REGULATION_OF_NEURON_APOPTOTIC_P...	GO:0043524	8	0.312500	0.993219	0.421456	0.822188	1.000
134	GOBP_REGULATION_OF_INFLAMMATORY_RESPONSE	GO:0050727	7	0.333333	0.988091	0.438931	0.828629	1.000
135	GOBP_EMBRYO_DEVELOPMENT	GO:0009790	14	0.232034	0.974494	0.463918	0.857637	1.000
136	GOBP_NEURON_APOPTOTIC_PROCESS	GO:0051402	12	0.252780	0.971368	0.440191	0.859370	1.000
137	REACTOME_SIGNALING_BY_RECEPTOR_TYROSINE_KINASES	R-RNO-9006934	26	0.193548	0.965053	0.532787	0.869320	1.000
138	GOBP_ANION_TRANSMEMBRANE_TRANSPORT	GO:0098656	7	0.320988	0.965035	0.473485	0.863223	1.000
139	GOBP_REGULATION_OF_PROTEIN_CATABOLIC_PROCESS	GO:0042176	7	0.316559	0.964800	0.482490	0.857961	1.000
140	GOBP_PROTEIN_AUTOPHOSPHORYLATION	GO:0046777	5	0.375668	0.953968	0.477273	0.879314	1.000
141	GOBP_INTRINSIC_APOPTOTIC_SIGNALING_PATHWAY	GO:0097193	5	0.361446	0.950899	0.521127	0.880277	1.000
142	GOBP_POSITIVE_REGULATION_OF_SMALL_MOLECULE_MET...	GO:0062013	5	0.361446	0.944578	0.490066	0.888962	1.000
143	GOBP_EMBRYONIC_ORGAN_DEVELOPMENT	GO:0048568	7	0.306738	0.943732	0.516484	0.885192	1.000
144	GOBP_RESPONSE_TO_NERVE_GROWTH_FACTOR	GO:1990089	7	0.308642	0.939577	0.482759	0.890515	1.000
145	GOBP_MORPHOGENESIS_OF_AN_EPITHELIUM	GO:0002009	6	0.339022	0.938117	0.534546	0.887964	1.000
146	GOBP_NEGATIVE_REGULATION_OF_PROTEIN_CATABOLIC_...	GO:0042177	5	0.360499	0.937439	0.519573	0.883901	1.000
147	GOBP_POSITIVE_REGULATION_OF_LONG_TERM_SYNAPTIC...	GO:1900273	5	0.385542	0.937122	0.477663	0.878737	1.000
148	GOBP_POSITIVE_REGULATION_OF_PHOSPHATIDYLINOSIT...	GO:0014068	5	0.361446	0.936824	0.526667	0.873723	1.000
149	GOBP_REGULATION_OF_MITOTIC_CELL_CYCLE	GO:0007346	5	0.361446	0.932008	0.514469	0.878739	1.000
150	GOBP_CHROMATIN_ORGANIZATION	GO:0006325	5	0.361446	0.924508	0.513793	0.891461	1.000
151	GOBP_POSITIVE_REGULATION_OF_EPITHELIAL_CELL_MI...	GO:0010634	5	0.361446	0.923452	0.562091	0.888111	1.000
152	GOBP_NEGATIVE_REGULATION_OF_APOPTOTIC_SIGNALIN...	GO:2001234	7	0.308642	0.922864	0.540540	0.883827	1.000
153	GOBP_RESPONSE_TO_UV	GO:0009411	5	0.361446	0.922388	0.539216	0.878943	1.000
154	GOBP_PROTEIN_CATABOLIC_PROCESS	GO:0030163	8	0.275000	0.919328	0.544444	0.879721	1.000
155	GOBP_REGULATION_OF_AXONOGENESIS	GO:0050770	7	0.308642	0.917311	0.567164	0.878771	1.000
156	GOBP_RESPONSE_TO_LIPID	GO:0033993	19	0.194570	0.912739	0.655405	0.883259	1.000
157	GOBP_EPITHELIAL_CELL_PROLIFERATION	GO:0050673	12	0.240703	0.910004	0.537118	0.884419	1.000
158	GOBP_REGULATION_OF_ENDOTHELIAL_CELL_MIGRATION	GO:0010594	5	0.361446	0.907882	0.578778	0.884174	1.000
159	GOBP_NEGATIVE_REGULATION_OF_CATALYTIC_ACTIVITY	GO:0043086	17	0.197183	0.906013	0.603352	0.882979	1.000
160	GOBP_POSITIVE_REGULATION_OF_DEVELOPMENTAL_GROWTH	GO:0048639	8	0.289397	0.903093	0.547085	0.884324	1.000
161	GOBP_INOSITOL_LIPID_MEDIATED_SIGNALING	GO:0048017	8	0.280327	0.894555	0.614407	0.898875	1.000
162	GOBP_POSITIVE_REGULATION_OF_CELL_PROJECTION_OR...	GO:0031346	10	0.243590	0.891642	0.622407	0.900556	1.000
163	GOBP_NEGATIVE_REGULATION_OF_CATABOLIC_PROCESS	GO:0009895	8	0.275000	0.887684	0.598485	0.904326	1.000
164	GOBP_REGULATION_OF_PHOSPHOLIPASE_ACTIVITY	GO:0010517	6	0.304878	0.886693	0.620438	0.900842	1.000
165	GOBP_ORGAN_GROWTH	GO:0035265	5	0.344442	0.875389	0.593443	0.921439	1.000
166	GOBP_CELLULAR_RESPONSE_TO_ORGANIC_CYCLIC_COMPOUND	GO:0071407	13	0.221033	0.873040	0.609524	0.921276	1.000
167	GOBP_TAXIS	GO:0042330	14	0.216216	0.869511	0.616580	0.923763	1.000
168	GOBP_REGULATION_OF_NEURON_DIFFERENTIATION	GO:0045664	5	0.330682	0.865395	0.639498	0.927348	1.000
169	GOBP_REGULATION_OF_ORGAN_GROWTH	GO:0046620	5	0.344442	0.864631	0.610738	0.923899	1.000

Continuation of Table **S3**

	NAME	ID	SIZE	ES	NES	NOM.p.val	FDR.q.val	FWER.p.val
170	GOBP_POSITIVE_REGULATION_OF_DEFENSE_RESPONSE	GO:0031349	5	0.325301	0.863089	0.618557	0.921913	1.000
171	GOBP_PEPTIDYL_TYROSINE_MODIFICATION	GO:0018212	10	0.243590	0.856718	0.681159	0.930263	1.000
172	GOBP_RESPONSE_TO_NUTRIENT	GO:0007584	5	0.326175	0.851056	0.634304	0.937139	1.000
173	GOBP_NEGATIVE_REGULATION_OF_LIPID_METABOLIC_PR...	GO:0045833	5	0.325301	0.847292	0.648387	0.940458	1.000
174	GOBP_REGULATION_OF_LIPASE_ACTIVITY	GO:0060191	6	0.304878	0.846746	0.646643	0.936306	1.000
175	GOBP_T_CELL_ACTIVATION	GO:0042110	8	0.259647	0.844126	0.646586	0.937171	1.000
176	GOBP_MULTI_MULTICELLULAR_ORGANISM_PROCESS	GO:0044706	8	0.258645	0.841847	0.665272	0.937014	1.000
177	GOBP_POSITIVE_REGULATION_OF_TRANSMEMBRANE_TRAN...	GO:0034764	7	0.279034	0.839617	0.653226	0.935960	1.000
178	GOBP_NEGATIVE_REGULATION_OF_PHOSPHORYLATION	GO:0042326	7	0.283951	0.838554	0.681992	0.932837	1.000
179	GOBP_IMMUNE_RESPONSE_REGULATING_SIGNALING_PATHWAY	GO:000276	8	0.258109	0.834987	0.677656	0.934908	1.000
180	REACTOME_CELLULAR_SENESCENCE	R-RNO-2559583	6	0.292310	0.831575	0.680000	0.936837	1.000
181	GOBP_RESPONSE_TO_BACTERIUM	GO:0009617	13	0.213333	0.826976	0.732394	0.941478	1.000
182	GOBP_CARDIAC_MUSCLE_TISSUE_DEVELOPMENT	GO:0048738	6	0.290025	0.822716	0.696864	0.944831	1.000
183	GOBP_NEGATIVE_REGULATION_OF_IMMUNE_SYSTEM_PROCESS	GO:0002683	5	0.325301	0.821208	0.654804	0.942627	1.000
184	GOBP_RESPONSE_TO_MOLECULE_OF_BACTERIAL_ORIGIN	GO:0002237	13	0.213333	0.820523	0.713568	0.938834	1.000
185	GOBP_REGULATION_OF_DEVELOPMENTAL_GROWTH	GO:0048638	9	0.238398	0.817479	0.705628	0.939867	1.000
186	GOBP_FC_RECEPTOR_SIGNALING_PATHWAY	GO:0038093	8	0.258109	0.816242	0.697417	0.937375	1.000
187	GOBP_HEART_DEVELOPMENT	GO:0007507	6	0.290025	0.812509	0.683453	0.940164	1.000
188	GOBP_POSITIVE_REGULATION_OF_PEPTIDYL_TYROSINE_...	GO:0050731	8	0.247860	0.811331	0.710317	0.937216	1.000
189	GOBP_PEPTIDYL_SERINE_MODIFICATION	GO:0018209	12	0.210526	0.802020	0.772926	0.950699	1.000
190	REACTOME_DISEASES_OF_SIGNAL_TRANSDUCTION_BY_GR...	R-HSA-5663202	12	0.210526	0.801492	0.744186	0.946708	1.000
191	GOBP_LOCOMOTION	GO:0040011	31	0.141032	0.800225	0.725000	0.943952	1.000
192	REACTOME_SENESCENCE_ASSOCIATED_SECRETORY_PHENO...	R-RNO-2559582	6	0.292310	0.799141	0.732143	0.941095	1.000
193	GOBP_CARBOHYDRATE_DERIVATIVE_METABOLIC_PROCESS	GO:1901135	8	0.247846	0.798453	0.726531	0.937532	1.000
194	GOBP_POSITIVE_REGULATION_OF_BINDING	GO:0051099	6	0.280488	0.797442	0.665480	0.934412	1.000
195	GOBP_EPITHELIAL_CELL_DIFFERENTIATION	GO:0030855	7	0.259573	0.797056	0.704280	0.930468	1.000
196	GOBP_POSITIVE_REGULATION_OF_CELL_DEVELOPMENT	GO:0010720	12	0.203559	0.793231	0.719626	0.932833	1.000
197	GOBP_REGULATION_OF_CELL_CELL_ADHESION	GO:0022407	9	0.229812	0.790152	0.750000	0.933940	1.000
198	GOBP_POSITIVE_REGULATION_OF_NEUROGENESIS	GO:0050769	12	0.203559	0.787728	0.815385	0.933471	1.000
199	HALLMARK_EPITHELIAL_MESENCHYMAL_TRANSITION	M5930	5	0.301205	0.785078	0.773026	0.933847	1.000
200	GOBP_POSITIVE_REGULATION_OF_NERVOUS_SYSTEM_DEV...	GO:0051962	12	0.203559	0.784173	0.764103	0.930806	1.000
201	GOBP_POSITIVE_REGULATION_OF_LIPASE_ACTIVITY	GO:0060193	5	0.301205	0.781320	0.739583	0.931049	1.000
202	GOBP_POSITIVE_REGULATION_OF_NEURON_PROJECTION_...	GO:0010976	5	0.301205	0.779435	0.733766	0.930320	1.000
203	GOBP_NEGATIVE_REGULATION_OF_HYDROLASE_ACTIVITY	GO:0051346	11	0.212303	0.777924	0.787330	0.928315	1.000
204	GOBP_CELLULAR_RESPONSE_TO_INORGANIC_SUBSTANCE	GO:0071241	11	0.207792	0.776903	0.786364	0.925378	1.000
205	GOBP_REGULATION_OF_CELL_SIZE	GO:0008361	5	0.301205	0.770335	0.718654	0.932304	1.000
206	GOBP_REGULATION_OF_RESPONSE_TO_EXTERNAL_STIMULUS	GO:0032101	15	0.178082	0.763231	0.821429	0.939228	1.000
207	GOBP_DEVELOPMENTAL_CELL_GROWTH	GO:0048588	5	0.301205	0.763197	0.784195	0.934776	1.000
208	GOBP_NEGATIVE_REGULATION_OF_BIOSYNTHETIC_PROCESS	GO:0009890	13	0.185892	0.759040	0.781915	0.937079	1.000
209	REACTOME_NGF_STIMULATED_TRANSCRIPTION	R-RNO-9031628	9	0.222405	0.749540	0.809524	0.948400	1.000
210	GOBP_REGULATION_OF_PROTEIN_STABILITY	GO:0031647	5	0.289063	0.749381	0.784722	0.944166	1.000
211	GOBP_PHAGOCYTOSIS	GO:0006909	5	0.279304	0.748049	0.794212	0.941721	1.000
212	GOBP_POSITIVE_REGULATION_OF_GROWTH	GO:0045927	9	0.227577	0.746238	0.809917	0.940095	1.000

Continuation of Table **S3**

	NAME	ID	SIZE	ES	NES	NOM.p.val	FDR.q.val	FWER.p.val
213	GOBP_RESPONSE_TO_OXYGEN_CONTAINING_COMPOUND	GO:1901700	39	0.124374	0.739523	0.870690	0.946161	1.000
214	GOBP_REGULATION_OF_PHOSPHOLIPASE_C_ACTIVITY	GO:1900274	5	0.301205	0.738510	0.760656	0.943378	1.000
215	REACTOME_DEVELOPMENTAL_BIOLOGY	R-RNO-1266738	22	0.149855	0.731267	0.844595	0.949794	1.000
216	GOBP_AXON_DEVELOPMENT	GO:0061564	18	0.161514	0.721685	0.921466	0.959240	1.000
217	GOBP_REGULATION_OF_DNA_BINDING_TRANSCRIPTION_F...	GO:0051090	16	0.165910	0.721324	0.854651	0.955211	1.000
218	GOBP_CELL_CELL_JUNCTION_ORGANIZATION	GO:0045216	6	0.256097	0.720020	0.841060	0.952751	1.000
219	GOBP_TISSUE_MORPHOGENESIS	GO:0048729	7	0.234323	0.719031	0.833333	0.949867	1.000
220	GOBP_CELLULAR_RESPONSE_TO_STARVATION	GO:0009267	5	0.275483	0.715769	0.827119	0.949851	1.000
221	GOBP_ORGANONITROGEN_COMPOUND_CATABOLIC_PROCESS	GO:1901565	10	0.199702	0.709744	0.844156	0.953560	1.000
222	GOBP_NEURON_PROJECTION_GUIDANCE	GO:0097485	9	0.202532	0.701773	0.892857	0.959841	1.000
223	GOBP_RESPONSE_TO_STARVATION	GO:0042594	5	0.275483	0.695740	0.840278	0.963060	1.000
224	GOBP_REPRODUCTIVE_SYSTEM_DEVELOPMENT	GO:0061458	6	0.242811	0.695259	0.894340	0.959418	1.000
225	GOBP_DEVELOPMENTAL_GROWTH	GO:0048589	10	0.187504	0.689377	0.907080	0.962404	1.000
226	GOBP_CELLULAR_RESPONSE_TO_MOLECULE_OF_BACTERIA...	GO:0071219	8	0.213298	0.686701	0.891566	0.961310	1.000
227	GOBP_CELLULAR_RESPONSE_TO_BIOTIC_STIMULUS	GO:0071216	8	0.213298	0.686408	0.893939	0.957451	1.000
228	GOBP_REGULATION_OF_IMMUNE_SYSTEM_PROCESS	GO:0002682	21	0.144975	0.685642	0.913333	0.954190	1.000
229	GOBP_POSITIVE_REGULATION_OF_IMMUNE_RESPONSE	GO:0050778	9	0.202532	0.684082	0.887931	0.952005	1.000
230	GOBP_POSITIVE_REGULATION_OF_RESPONSE_TO_EXTERN...	GO:0032103	9	0.202532	0.682362	0.864407	0.950325	1.000
231	GOBP_GLAND_DEVELOPMENT	GO:0048732	7	0.229132	0.680928	0.887641	0.947834	1.000
232	GOBP_NEURON_DEATH	GO:0070997	18	0.145466	0.671866	0.913793	0.954052	1.000
233	GOBP_RESPONSE_TO_ORGANIC_CYCLIC_COMPOUND	GO:0014070	20	0.144374	0.668537	0.944099	0.953708	1.000
234	REACTOME_POST_TRANSLATIONAL_PROTEIN_MODIFICATION	R-RNO-597592	7	0.222929	0.664100	0.914286	0.954125	1.000
235	GOBP_POSITIVE_REGULATION_OF_CATION_TRANSMEMBRA...	GO:1904064	5	0.266763	0.663717	0.898246	0.950521	1.000
236	GOBP_RESPONSE_TO_CADMIUM_ION	GO:0046686	6	0.236588	0.660065	0.887789	0.949980	1.000
237	GOBP_GLIOGENESIS	GO:0042063	12	0.172702	0.656612	0.955285	0.949652	1.000
238	GOBP_RESPONSE_TO_OXIDATIVE_STRESS	GO:0006979	8	0.200000	0.644542	0.911894	0.956871	1.000
239	REACTOME_EXTRACELLULAR_MATRIX_ORGANIZATION	R-RNO-1474244	5	0.253012	0.643903	0.914591	0.953475	1.000
240	GOBP_EXTRINSIC_APOPTOTIC_SIGNALING_PATHWAY	GO:0097191	7	0.213948	0.632606	0.914498	0.959308	1.000
241	GOBP_RESPONSE_TO_REACTIVE_OXYGEN_SPECIES	GO:0000302	8	0.200000	0.631614	0.932075	0.956010	1.000
242	HALLMARK_UV_RESPONSE_UP	M5941	5	0.228916	0.598158	0.954693	0.975470	1.000
243	REACTOME_INFECTIOUS_DISEASE	R-HSA-5663205	12	0.149135	0.566231	0.972973	0.988075	1.000
244	GOBP_NEGATIVE_REGULATION_OF_TRANSMEMBRANE_TRAN...	GO:0034763	6	0.195147	0.548072	0.977273	0.991367	1.000
245	GOBP_TISSUE_HOMEOSTASIS	GO:0001894	5	0.204819	0.536539	0.973077	0.990628	1.000
246	GOBP_ERBB_SIGNALING_PATHWAY	GO:0038127	5	0.192771	0.507811	0.990099	0.993334	1.000
247	GOBP_POSITIVE_REGULATION_OF_CELLULAR_COMPONENT...	GO:0051130	24	-0.109107	-0.384918	0.996499	0.999159	1.000
248	GOBP_RESPONSE_TO_NITROGEN_COMPOUND	GO:1901698	35	-0.114099	-0.425075	0.996652	0.999031	1.000
249	GOBP_REGULATION_OF_CELLULAR_COMPONENT_SIZE	GO:0032535	7	-0.184156	-0.473932	0.991968	0.994017	1.000
250	REACTOME_ESTROGEN_DEPENDENT_NUCLEAR_EVENTS_DOW...	R-RNO-9634638	5	-0.216867	-0.483219	0.986130	0.993909	1.000
251	GOBP_REGIONALIZATION	GO:0003002	6	-0.205458	-0.491284	0.988935	0.993932	1.000
252	GOBP_REGULATION_OF_IMMUNE_RESPONSE	GO:0050776	12	-0.161627	-0.491716	0.980892	0.995929	1.000
253	GOBP_PATTERN_SPECIFICATION_PROCESS	GO:0007389	6	-0.205458	-0.497765	0.992063	0.996338	1.000
254	GOBP_TELENCEPHALON_DEVELOPMENT	GO:0021537	7	-0.201269	-0.500782	0.989026	0.997547	1.000
255	GOBP_CELL_CELL_ADHESION	GO:0098609	14	-0.160845	-0.505760	0.977833	0.998052	1.000

Continuation of Table **S3**

	NAME	ID	SIZE	ES	NES	NOM.p.val	FDR.q.val	FWER.p.val
256	GOBP_RESPONSE_TO_ABIOTIC_STIMULUS	GO:0009628	27	-0.139435	-0.508388	0.981395	0.999309	1.000
257	GOBP_IMPORT_INTO_CELL	GO:0098657	6	-0.212604	-0.516838	0.986188	0.998349	1.000
258	GOBP_EPITHELIUM_DEVELOPMENT	GO:0060429	15	-0.164005	-0.518340	0.974296	0.999973	1.000
259	GOBP_POSITIVE_REGULATION_OF_NEURON_DEATH	GO:1901216	5	-0.230676	-0.519159	0.979856	1.000000	1.000
260	GOBP_REGULATION_OF_GTPASE_ACTIVITY	GO:0043087	5	-0.233002	-0.528485	0.971852	1.000000	1.000
261	GOBP_RESPONSE_TO_LIGHT_STIMULUS	GO:0009416	11	-0.181539	-0.530893	0.963589	1.000000	1.000
262	REACTOME_ONCOGENIC_MAPK_SIGNALING	R-HSA-6802957	5	-0.234317	-0.530914	0.976261	1.000000	1.000
263	GOBP_TRANSMEMBRANE_RECEPTOR_PROTEIN_TYROSINE_K...	GO:0007169	19	-0.158897	-0.531921	0.972941	1.000000	1.000
264	GOBP_EMBRYONIC_MORPHOGENESIS	GO:0048598	6	-0.217951	-0.532552	0.983075	1.000000	1.000
265	GOBP_NEGATIVE_REGULATION_OF_NUCLEOBASE_CONTAIN...	GO:0045934	13	-0.175333	-0.539493	0.964149	1.000000	1.000
266	GOBP_ENZYME_LINKED_RECEPTOR_PROTEIN_SIGNALING_...	GO:0007167	24	-0.150104	-0.540346	0.967442	1.000000	1.000
267	GOBP_RESPONSE_TO_NICOTINE	GO:0035094	7	-0.215253	-0.541431	0.972259	1.000000	1.000
268	GOBP_NEGATIVE_REGULATION_OF_MULTICELLULAR_ORGA...	GO:0051241	15	-0.171221	-0.543129	0.972500	1.000000	1.000
269	GOBP_PHOSPHATIDYLINOSITOL_3_KINASE_SIGNALING	GO:0014065	7	-0.219319	-0.557360	0.953652	1.000000	1.000
270	GOBP_POSITIVE_REGULATION_OF_ORGANELLE_ORGANIZA...	GO:0010638	10	-0.198840	-0.567053	0.951219	1.000000	1.000
271	GOBP_REGULATION_OF_ANATOMICAL_STRUCTURE_SIZE	GO:0090066	10	-0.200522	-0.569979	0.937904	1.000000	1.000
272	REACTOME_SIGNALING_BY_NTRK2_TRKB	R-RNO-9006115	6	-0.231638	-0.570158	0.957082	1.000000	1.000
273	GOBP_CELLULAR_RESPONSE_TO_REACTIVE_OXYGEN_SPECIES	GO:0034614	7	-0.225237	-0.570272	0.954794	1.000000	1.000
274	GOBP_CELLULAR_RESPONSE_TO_CHEMICAL_STRESS	GO:0062197	7	-0.225237	-0.572638	0.951743	1.000000	1.000
275	GOBP_RESPONSE_TO_TEMPERATURE_STIMULUS	GO:0009266	9	-0.209151	-0.576758	0.959239	1.000000	1.000
276	GOBP_REGULATION_OF_NERVOUS_SYSTEM_DEVELOPMENT	GO:0051960	15	-0.181021	-0.580747	0.941323	1.000000	1.000
277	GOBP_MATERNAL_PROCESS_INVOLVED_IN_FEMALE_PREGN...	GO:0060135	5	-0.250799	-0.581010	0.949438	1.000000	1.000
278	GOBP_AGING	GO:0007568	13	-0.190038	-0.582161	0.945980	1.000000	1.000
279	GOBP_TUBE_DEVELOPMENT	GO:0035295	18	-0.174921	-0.582584	0.932845	1.000000	1.000
280	GOBP_CARBOHYDRATE_METABOLIC_PROCESS	GO:0005975	9	-0.214767	-0.589052	0.935271	1.000000	1.000
281	GOBP_TEMPERATURE_HOMEOSTASIS	GO:0001659	5	-0.255721	-0.589346	0.943870	1.000000	1.000
282	REACTOME_MYD88_INDEPENDENT_TLR4_CASCADE	R-RNO-166166	8	-0.223096	-0.589475	0.952128	1.000000	1.000
283	GOBP_REGULATION_OF_CELL_DEVELOPMENT	GO:0060284	15	-0.185295	-0.590447	0.931204	1.000000	1.000
284	GOBP_CELL_ACTIVATION	GO:0001775	22	-0.169255	-0.591346	0.935096	1.000000	1.000
285	GOBP_MACROMOLECULE_CATABOLIC_PROCESS	GO:0009057	11	-0.207125	-0.592384	0.950943	1.000000	1.000
286	REACTOME_TOLL_LIKE_RECEPTOR_CASCADES	R-RNO-168898	8	-0.223096	-0.593698	0.943122	1.000000	1.000
287	GOBP_APOPTOTIC_PROCESS	GO:0006915	35	-0.157896	-0.593930	0.914124	1.000000	1.000
288	REACTOME_TOLL_LIKE_RECEPTOR_TLR1_TLR2_CASCADE	R-RNO-168179	8	-0.223096	-0.595746	0.940781	1.000000	1.000
289	GOBP_REGULATION_OF_NEURON_PROJECTION_DEVELOPMENT	GO:0010975	16	-0.183209	-0.597279	0.928922	1.000000	1.000
290	GOBP_DNA_METABOLIC_PROCESS	GO:0006259	6	-0.247793	-0.597850	0.933151	1.000000	1.000
291	REACTOME_TOLL_LIKE_RECEPTOR_9_TLR9_CASCADE	R-RNO-168138	8	-0.223096	-0.599521	0.934492	1.000000	1.000
292	GOBP_REGULATION_OF_NEUROGENESIS	GO:0050767	14	-0.189992	-0.604027	0.920854	1.000000	1.000
293	GOBP_REGULATION_OF_PROTEIN_SERINE_THREONINE_KI...	GO:0071900	12	-0.200390	-0.604047	0.920984	1.000000	1.000
294	GOBP_CELLULAR_COMPONENT_DISASSEMBLY	GO:0022411	6	-0.251663	-0.605072	0.935705	1.000000	1.000
295	GOBP_SMALL_MOLECULE_BIOSYNTHETIC_PROCESS	GO:0044283	5	-0.268927	-0.605481	0.930595	1.000000	1.000
296	GOBP_REGULATION_OF_SMALL_MOLECULE_METABOLIC_PR...	GO:0062012	9	-0.220724	-0.606682	0.919419	1.000000	1.000
297	GOBP_RESPONSE_TO_RADIATION	GO:0009314	13	-0.199618	-0.611987	0.925786	1.000000	1.000
298	REACTOME_LEISHMANIA_INFECTION	R-HSA-9658195	10	-0.213991	-0.612256	0.926509	1.000000	1.000

Continuation of Table **S3**

	NAME	ID	SIZE	ES	NES	NOM.p.val	FDR.q.val	FWER.p.val
299	REACTOME_HEMOSTASIS	R-RNO-109582	14	-0.202156	-0.619065	0.900990	1.000000	1.000
300	GOBP_FOREBRAIN_DEVELOPMENT	GO:0030900	10	-0.218096	-0.622794	0.911539	1.000000	1.000
301	REACTOME_INTERLEUKIN_17_SIGNALING	R-RNO-448424	6	-0.258818	-0.622892	0.923717	1.000000	1.000
302	GOBP_POSITIVE_REGULATION_OF_CELL_DEATH	GO:0010942	19	-0.185289	-0.624362	0.897714	1.000000	1.000
303	GOBP_CIRCADIAN_REGULATION_OF_GENE_EXPRESSION	GO:0032922	5	-0.274707	-0.626784	0.927660	1.000000	1.000
304	GOBP_NEGATIVE_REGULATION_OF_LOCOMOTION	GO:0040013	5	-0.277471	-0.626907	0.912676	1.000000	1.000
305	REACTOME_SIGNALING_BY_NTRKS	R-RNO-166520	18	-0.189979	-0.628316	0.917476	1.000000	1.000
306	GOBP_REGULATION_OF_INTRACELLULAR_SIGNAL_TRANSD...	GO:1902531	21	-0.181653	-0.629324	0.913404	1.000000	1.000
307	GOBP_CELL_PART_MORPHOGENESIS	GO:0032990	25	-0.176486	-0.629438	0.911661	1.000000	1.000
308	GOBP_CELL_MORPHOGENESIS	GO:0000902	25	-0.176486	-0.632011	0.911935	1.000000	1.000
309	GOBP_MULTICELLULAR_ORGANISMAL_RESPONSE_TO_STRESS	GO:0033555	5	-0.276209	-0.634017	0.903930	1.000000	1.000
310	GOBP_APOPTOTIC_SIGNALING_PATHWAY	GO:0097190	15	-0.202856	-0.637952	0.899875	1.000000	1.000
311	GOBP_CELLULAR_COMPONENT_MORPHOGENESIS	GO:0032989	25	-0.176486	-0.639343	0.893887	1.000000	1.000
312	GOBP_CELLULAR_RESPONSE_TO_CADMIUM_ION	GO:0071276	5	-0.279725	-0.642169	0.932867	1.000000	1.000
313	REACTOME_L1CAM_INTERACTIONS	R-RNO-373760	5	-0.281745	-0.642213	0.891971	1.000000	1.000
314	GOBP_REGULATION_OF_PROTEIN_LOCALIZATION_TO_MEM...	GO:1905475	7	-0.256270	-0.649900	0.893960	1.000000	1.000
315	GOBP_REGULATION_OF_LIPID_LOCALIZATION	GO:1905952	5	-0.287071	-0.655125	0.900990	1.000000	1.000
316	GOBP_CELLULAR_RESPONSE_TO_DNA_DAMAGE_STIMULUS	GO:0006974	5	-0.284945	-0.655841	0.881503	1.000000	1.000
317	GOBP_REGULATION_OF_ANATOMICAL_STRUCTURE_MORPHO...	GO:0022603	17	-0.199600	-0.656454	0.893285	1.000000	1.000
318	GOBP_POSITIVE_REGULATION_OF_DNA_BINDING_TRANSC...	GO:0051091	10	-0.234812	-0.659850	0.886719	1.000000	1.000
319	GOBP_REGULATION_OF_PHOSPHATIDYLINOSITOL_3_KINA...	GO:0014066	6	-0.267683	-0.659917	0.887943	1.000000	1.000
320	GOBP_ORGANIC_HYDROXY_COMPOUND_METABOLIC_PROCESS	GO:1901615	8	-0.245539	-0.663838	0.879730	1.000000	1.000
321	GOBP_MUSCLE_TISSUE_DEVELOPMENT	GO:0060537	11	-0.227915	-0.664168	0.902188	1.000000	1.000
322	REACTOME_PLATELET_ACTIVATION_SIGNALING_AND_AGG...	R-HSA-76002	7	-0.262887	-0.665760	0.881379	1.000000	1.000
323	GOBP_RECEPTOR_MEDIATED_ENDOCYTOSIS	GO:0006898	6	-0.273648	-0.666842	0.895863	1.000000	1.000
324	GOBP_POLYSACCHARIDE_METABOLIC_PROCESS	GO:0005976	5	-0.292553	-0.668184	0.888418	1.000000	1.000
325	GOBP_CELLULAR_RESPONSE_TO_LIPID	GO:0071396	15	-0.208988	-0.669561	0.865385	1.000000	1.000
326	GOBP_REGULATION_OF_PEPTIDASE_ACTIVITY	GO:0052547	10	-0.232725	-0.670308	0.876463	1.000000	1.000
327	GOBP_REGULATION_OF_GLIOGENESIS	GO:0014013	5	-0.295102	-0.673243	0.883357	1.000000	1.000
328	GOBP_GLIAL_CELL_DIFFERENTIATION	GO:0010001	10	-0.238077	-0.674546	0.858466	1.000000	1.000
329	GOBP_EXOCYTOSIS	GO:0006887	11	-0.230807	-0.676119	0.868020	1.000000	1.000
330	GOBP_REGULATION_OF_PROTEOLYSIS	GO:0030162	12	-0.225892	-0.678539	0.859395	1.000000	1.000
331	GOBP_NEGATIVE_REGULATION_OF_RESPONSE_TO_STIMULUS	GO:0048585	16	-0.210434	-0.678635	0.859069	1.000000	1.000
332	GOBP_REGULATION_OF_WNT_SIGNALING_PATHWAY	GO:0030111	5	-0.300503	-0.681691	0.876274	1.000000	1.000
333	GOBP_POSITIVE_REGULATION_OF_GLIOGENESIS	GO:0014015	5	-0.295102	-0.683679	0.881924	1.000000	1.000
334	GOBP_INTRACELLULAR_RECEPTOR_SIGNALING_PATHWAY	GO:0030522	5	-0.302027	-0.683743	0.881857	1.000000	1.000
335	GOBP_PLATELET_ACTIVATION	GO:0030168	6	-0.283390	-0.685073	0.875683	1.000000	1.000
336	GOBP_REGULATION_OF_CIRCADIAN_RHYTHM	GO:0042752	7	-0.268253	-0.687284	0.859551	1.000000	1.000
337	GOBP_REGULATION_OF_PROTEIN_LOCALIZATION_TO_CEL...	GO:1904375	6	-0.285480	-0.694618	0.857542	1.000000	1.000
338	GOBP_NEGATIVE_REGULATION_OF_RESPONSE_TO_EXTERN...	GO:0032102	5	-0.307113	-0.698584	0.849432	1.000000	1.000
339	GOBP_REGULATION_OF_PROTEIN_KINASE_ACTIVITY	GO:0045859	21	-0.204638	-0.706512	0.828070	1.000000	1.000
340	GOBP_POSITIVE_REGULATION_OF_APOPTOTIC_SIGNALIN...	GO:2001235	5	-0.314914	-0.713002	0.845494	1.000000	1.000
341	GOBP_REGULATION_OF_TRANSFERASE_ACTIVITY	GO:0051338	21	-0.204638	-0.713315	0.817527	1.000000	1.000

Continuation of Table **S3**

	NAME	ID	SIZE	ES	NES	NOM.p.val	FDR.q.val	FWER.p.val
342	GOBP_MEMORY	GO:0007613	13	-0.233673	-0.714260	0.835600	1.000000	1.000
343	GOBP_GENERATION_OF_PRECURSOR_METABOLITES_AND_E...	GO:0006091	5	-0.314718	-0.717186	0.831697	1.000000	1.000
344	GOBP_NEGATIVE_REGULATION_OF_PHOSPHORUS_METABOL...	GO:0010563	9	-0.262971	-0.718416	0.821935	1.000000	1.000
345	GOBP_COGNITION	GO:0050890	28	-0.198584	-0.720678	0.827546	1.000000	1.000
346	GOBP_FC_EPSILON_RECEPTOR_SIGNALING_PATHWAY	GO:0038095	7	-0.281963	-0.721384	0.834492	1.000000	1.000
347	REACTOME_FC_EPSILON_RECEPTOR_FCERI_SIGNALING	R-RNO-2454202	7	-0.281963	-0.721581	0.818801	1.000000	1.000
348	GOBP_CARBOHYDRATE_BIOSYNTHETIC_PROCESS	GO:0016051	5	-0.323869	-0.722697	0.829912	1.000000	1.000
349	GOBP_RESPONSE_TO_WOUNDING	GO:0009611	15	-0.227930	-0.725898	0.822864	1.000000	1.000
350	GOBP_ERK1_AND_ERK2_CASCADE	GO:0070371	5	-0.322805	-0.727958	0.825648	1.000000	1.000
351	GOBP_PEPTIDE_METABOLIC_PROCESS	GO:0006518	11	-0.246237	-0.728380	0.805447	1.000000	1.000
352	GOBP_REGULATION_OF_MAPK_CASCADE	GO:0043408	13	-0.239447	-0.728918	0.832723	1.000000	1.000
353	REACTOME_CELLULAR_RESPONSES_TO_EXTERNAL_STIMULI	R-RNO-8953897	11	-0.248237	-0.730666	0.825129	1.000000	1.000
354	GOBP_NEGATIVE_REGULATION_OF_ION_TRANSPORT	GO:0043271	11	-0.250357	-0.730796	0.819338	1.000000	1.000
355	GOBP_RESPONSE_TO_ACID_CHEMICAL	GO:0001101	6	-0.295827	-0.731132	0.820690	1.000000	1.000
356	GOBP_RECEPTOR_INTERNALIZATION	GO:0031623	5	-0.318608	-0.732266	0.819820	1.000000	1.000
357	GOBP_REGULATION_OF_CATABOLIC_PROCESS	GO:0009894	13	-0.236000	-0.732349	0.803198	1.000000	1.000
358	GOBP_REGULATION_OF_CARBOHYDRATE_BIOSYNTHETIC_P...	GO:0043255	5	-0.323869	-0.732467	0.800000	1.000000	1.000
359	GOBP_RECEPTOR_METABOLIC_PROCESS	GO:0043112	5	-0.318608	-0.735109	0.796243	1.000000	1.000
360	GOBP_NEGATIVE_REGULATION_OF_PROTEIN_MODIFICATI...	GO:0031400	10	-0.260258	-0.735891	0.807939	1.000000	1.000
361	GOBP_AMYLOID_PRECURSOR_PROTEIN_CATABOLIC_PROCESS	GO:0042987	5	-0.324460	-0.737094	0.810811	1.000000	1.000
362	GOBP_POSITIVE_REGULATION_OF_HYDROLASE_ACTIVITY	GO:0051345	16	-0.227415	-0.738750	0.807692	1.000000	1.000
363	GOBP_REGULATION_OF_PROTEIN_PHOSPHORYLATION	GO:0001932	25	-0.207804	-0.738757	0.791762	1.000000	1.000
364	GOBP_REGULATION_OF_CELLULAR_COMPONENT_BIOGENESIS	GO:0044087	10	-0.263280	-0.739517	0.799228	1.000000	1.000
365	GOBP_REGULATION_OF_APOPTOTIC_SIGNALING_PATHWAY	GO:2001233	10	-0.261930	-0.739953	0.805627	1.000000	1.000
366	GOBP_REGULATION_OF_GROWTH	GO:0040008	13	-0.240030	-0.742395	0.803896	1.000000	1.000
367	GOBP_AMYLOID_BETA_METABOLIC_PROCESS	GO:0050435	5	-0.324460	-0.746784	0.803596	1.000000	1.000
368	GOBP_AMYLOID_PRECURSOR_PROTEIN_METABOLIC_PROCESS	GO:0042982	5	-0.324460	-0.746821	0.802651	1.000000	1.000
369	GOBP_REGULATION_OF_CARBOHYDRATE_METABOLIC_PROCESS	GO:0006109	5	-0.323869	-0.750759	0.773371	1.000000	1.000
370	GOBP_REPRODUCTION	GO:0000003	18	-0.223628	-0.751264	0.790898	1.000000	1.000
371	GOBP_NEGATIVE_REGULATION_OF_NERVOUS_SYSTEM_DEV...	GO:0051961	5	-0.330984	-0.751568	0.787519	1.000000	1.000
372	GOBP_POSITIVE_REGULATION_OF_INTRACELLULAR_SIGN...	GO:1902533	14	-0.237562	-0.751615	0.796548	1.000000	1.000
373	GOBP_POSITIVE_REGULATION_OF_LOCOMOTION	GO:0040017	11	-0.258913	-0.754353	0.795165	1.000000	1.000
374	GOBP_RESPONSE_TO_MONOSACCHARIDE	GO:0034284	5	-0.329580	-0.756305	0.790801	1.000000	1.000
375	GOBP_LIPID_LOCALIZATION	GO:0010876	10	-0.272237	-0.756768	0.766753	1.000000	1.000
376	GOBP_NEGATIVE_REGULATION_OF_ANION_TRANSPORT	GO:1903792	8	-0.281373	-0.762099	0.785034	1.000000	1.000
377	REACTOME_ADAPTIVE_IMMUNE_SYSTEM	R-RNO-1280218	9	-0.278713	-0.764186	0.780355	1.000000	1.000
378	GOBP_REGULATION_OF_HORMONE_LEVELS	GO:0010817	12	-0.252688	-0.764320	0.777070	1.000000	1.000
379	GOBP_NEGATIVE_REGULATION_OF_CELL_DEVELOPMENT	GO:0010721	6	-0.316857	-0.765843	0.759184	1.000000	1.000
380	GOBP_RESPONSE_TO_CARBOHYDRATE	GO:0009743	5	-0.329580	-0.765861	0.796215	1.000000	1.000
381	GOBP_CIRCADIAN_RHYTHM	GO:0007623	13	-0.247202	-0.766651	0.766460	1.000000	1.000
382	GOBP_CELLULAR_CARBOHYDRATE_METABOLIC_PROCESS	GO:0044262	5	-0.342016	-0.773555	0.788489	1.000000	1.000
383	GOBP_MONOAMINE_TRANSPORT	GO:0015844	5	-0.337074	-0.775095	0.749646	1.000000	1.000
384	GOBP_LYMPHOCYTE_ACTIVATION	GO:0046649	10	-0.270374	-0.778554	0.755263	1.000000	1.000

Continuation of Table **S3**

	NAME	ID	SIZE	ES	NES	NOM.p.val	FDR.q.val	FWER.p.val
385	GOBP_ORGANIC_HYDROXY_COMPOUND_TRANSPORT	GO:0015850	6	-0.321598	-0.780777	0.761636	1.000000	1.000
386	GOBP_REGULATION_OF_CELL_ACTIVATION	GO:0050865	8	-0.298521	-0.781291	0.738606	1.000000	1.000
387	GOBP_NEURON_DIFFERENTIATION	GO:0030182	37	-0.206484	-0.781932	0.741379	1.000000	1.000
388	GOBP_REGULATION_OF_PEPTIDYL_TYROSINE_PHOSPHORY...	GO:0050730	9	-0.287594	-0.782675	0.749340	1.000000	1.000
389	GOBP_MUSCLE_CELL_PROLIFERATION	GO:0033002	8	-0.296209	-0.784862	0.765605	1.000000	1.000
390	GOBP_BIOLOGICAL_ADHESION	GO:0022610	19	-0.234473	-0.785669	0.736397	1.000000	1.000
391	GOBP_POSITIVE_REGULATION_OF_PHOSPHORUS_METABOL...	GO:0010562	23	-0.225402	-0.786456	0.752969	1.000000	1.000
392	GOBP_NEGATIVE_REGULATION_OF_CELLULAR_COMPONENT...	GO:0051129	11	-0.265278	-0.788297	0.739355	1.000000	1.000
393	GOBP_REGULATION_OF_RESPONSE_TO_STRESS	GO:0080134	19	-0.231922	-0.788348	0.756126	1.000000	1.000
394	GOBP_RESPONSE_TO_TUMOR_NECROSIS_FACTOR	GO:0034612	7	-0.303492	-0.788455	0.741287	1.000000	1.000
395	GOBP_CHROMOSOME_ORGANIZATION	GO:0051276	6	-0.333024	-0.789546	0.749638	1.000000	1.000
396	REACTOME_ANTI_INFLAMMATORY_RESPONSE_FAVOURING_...	R-HSA-9662851	5	-0.347893	-0.791148	0.736919	1.000000	1.000
397	GOBP_POSITIVE_REGULATION_OF_TRANSFERASE_ACTIVITY	GO:0051347	19	-0.237680	-0.791851	0.746199	1.000000	1.000
398	REACTOME_NERVOUS_SYSTEM_DEVELOPMENT	R-RNO-9675108	14	-0.250086	-0.792115	0.737624	1.000000	1.000
399	GOBP_RHYTHMIC_PROCESS	GO:0048511	15	-0.246349	-0.792344	0.757463	1.000000	1.000
400	GOBP_LEUKOCYTE_MIGRATION	GO:0050900	6	-0.327994	-0.792360	0.741703	1.000000	1.000
401	GOBP_REGULATION_OF_MAP_KINASE_ACTIVITY	GO:0043405	9	-0.286182	-0.792552	0.736220	1.000000	1.000
402	GOBP_COAGULATION	GO:0050817	8	-0.295169	-0.793318	0.733062	1.000000	1.000
403	GOBP_POSITIVE_REGULATION_OF_ANION_TRANSPORT	GO:1903793	9	-0.291891	-0.793514	0.738667	1.000000	1.000
404	GOBP_REGULATION_OF_LONG_TERM_SYNAPTIC_POTENTIA...	GO:1900271	6	-0.324315	-0.798614	0.739316	1.000000	1.000
405	GOBP_PROTEIN_LOCALIZATION_TO_PLASMA_MEMBRANE	GO:0072659	7	-0.317439	-0.799600	0.722772	1.000000	1.000
406	GOBP_ACTIVATION_OF_PROTEIN_KINASE_ACTIVITY	GO:0032147	10	-0.279399	-0.801079	0.738065	1.000000	1.000
407	GOBP_NEGATIVE_REGULATION_OF_CELL_DIFFERENTIATION	GO:0045596	13	-0.261246	-0.801478	0.724311	1.000000	1.000
408	GOBP_NEGATIVE_REGULATION_OF_INTRACELLULAR_SIGN...	GO:1902532	7	-0.312065	-0.803536	0.724462	1.000000	1.000
409	REACTOME_INNATE_IMMUNE_SYSTEM	R-RNO-168249	16	-0.251708	-0.806745	0.736646	1.000000	1.000
410	GOBP_NEGATIVE_REGULATION_OF_TRANSPORT	GO:0051051	13	-0.261116	-0.806812	0.714834	1.000000	1.000
411	GOBP_POSITIVE_REGULATION_OF_MAP_KINASE_ACTIVITY	GO:0043406	6	-0.335041	-0.807445	0.738714	1.000000	1.000
412	GOBP_NEGATIVE_REGULATION_OF_DEVELOPMENTAL_PROCESS	GO:0051093	13	-0.261246	-0.807637	0.700614	1.000000	1.000
413	GOBP_ACTIVATION_OF_MAPK_ACTIVITY	GO:0000187	6	-0.335041	-0.808041	0.697234	1.000000	1.000
414	GOBP_CYTOSOLIC_CALCIUM_ION_TRANSPORT	GO:0060401	7	-0.321188	-0.810720	0.722067	1.000000	1.000
415	GOBP_CENTRAL_NERVOUS_SYSTEM_NEURON_DIFFERENTIA...	GO:0021953	5	-0.359312	-0.812780	0.695846	1.000000	1.000
416	GOBP_MULTICELLULAR_ORGANISMAL_HOMEOSTASIS	GO:0048871	12	-0.267389	-0.812828	0.716113	1.000000	1.000
417	GOBP_LONG_TERM_SYNAPTIC_DEPRESSION	GO:0060292	5	-0.360830	-0.815619	0.712857	1.000000	1.000
418	GOBP_REGULATION_OF_EXTRINSIC_APOPTOTIC_SIGNALI...	GO:2001236	6	-0.341572	-0.815894	0.710227	1.000000	1.000
419	GOBP_MULTI_ORGANISM_PROCESS	GO:0051704	16	-0.256654	-0.818983	0.703202	1.000000	1.000
420	GOBP_CALCIUM_ION_TRANSMEMBRANE_TRANSPORT	GO:0070588	7	-0.321188	-0.819381	0.719346	1.000000	1.000
421	GOBP_NEURON_MIGRATION	GO:0001764	5	-0.356220	-0.819526	0.702742	1.000000	1.000
422	GOBP_REGULATION_OF_PROTEIN_MODIFICATION_PROCESS	GO:0031399	28	-0.222047	-0.819563	0.704307	1.000000	1.000
423	GOBP_REGULATION_OF_CELLULAR_AMIDE_METABOLIC_PR...	GO:0034248	9	-0.299826	-0.819862	0.706806	1.000000	1.000
424	GOBP_POSITIVE_REGULATION_OF_CELL_GROWTH	GO:0030307	6	-0.338542	-0.820012	0.695890	1.000000	1.000
425	REACTOME_MAPK_TARGETS_NUCLEAR_EVENTS_MEDIATED_...	R-HSA-450282	5	-0.364032	-0.820301	0.719054	1.000000	1.000
426	GOBP_REGULATION_OF_VASCULATURE_DEVELOPMENT	GO:1901342	5	-0.365528	-0.820559	0.709440	1.000000	1.000
427	GOBP_MEMBRANE_ORGANIZATION	GO:0061024	12	-0.280177	-0.824240	0.720099	1.000000	1.000

Continuation of Table **S3**

	NAME	ID	SIZE	ES	NES	NOM.p.val	FDR.q.val	FWER.p.val
428	GOBP_CALCIUM_ION_TRANSPORT_INTO_CYTOSOL	GO:0060402	7	-0.321188	-0.824258	0.694102	1.000000	1.000
429	GOBP_BIOLOGICAL_PROCESS_INVOLVED_IN_SYMBIOTIC_...	GO:004440	10	-0.296362	-0.826034	0.691275	1.000000	1.000
430	GOBP_REGULATION_OF_PHOSPHORUS_METABOLIC_PROCESS	GO:0051174	26	-0.233215	-0.828515	0.701493	1.000000	1.000
431	GOBP_REGULATION_OF_PROTEIN_TYROSINE_KINASE_ACT...	GO:0061097	5	-0.365047	-0.828805	0.697778	1.000000	1.000
432	GOBP_CELLULAR_MACROMOLECULE_CATABOLIC_PROCESS	GO:0044265	5	-0.373494	-0.829246	0.731563	1.000000	1.000
433	GOBP_SMOOTH_MUSCLE_CELL_PROLIFERATION	GO:0048659	6	-0.336269	-0.830615	0.689605	1.000000	1.000
434	GOBP_NEURON_DEVELOPMENT	GO:0048666	34	-0.220567	-0.833480	0.682682	1.000000	1.000
435	GOBP_REGULATION_OF_CYSTEINE_TYPE_ENDOPEPTIDASE...	GO:2000116	9	-0.306778	-0.835725	0.666667	1.000000	1.000
436	GOBP_ORGANIC_ACID_TRANSPORT	GO:0015849	7	-0.334449	-0.838143	0.697260	1.000000	1.000
437	GOBP_REGULATION_OF_RECEPTOR_MEDIATED_ENDOCYTOSIS	GO:0048259	5	-0.365528	-0.839147	0.681159	1.000000	1.000
438	GOBP_LIPID_EXPORT_FROM_CELL	GO:0140353	5	-0.372510	-0.839741	0.677843	1.000000	1.000
439	GOBP_POSITIVE_REGULATION_OF_PROTEIN_TYROSINE_K...	GO:0061098	5	-0.365047	-0.839977	0.666179	1.000000	1.000
440	GOBP_RESPONSE_TO_INSULIN	GO:0032868	5	-0.368516	-0.841053	0.666667	1.000000	1.000
441	REACTOME_NUCLEAR_EVENTS_KINASE_AND_TRANSCRIPTI...	R-RNO-198725	11	-0.289384	-0.843772	0.671018	1.000000	1.000
442	GOBP_SMALL_GTPASE_MEDIATED_SIGNAL_TRANSDUCTION	GO:0007264	8	-0.314311	-0.845060	0.672857	1.000000	1.000
443	REACTOME_EXTRA_NUCLEAR_ESTROGEN_SIGNALING	R-RNO-9009391	7	-0.330617	-0.846289	0.677241	1.000000	1.000
444	GOBP_REGULATION_OF_METAL_ION_TRANSPORT	GO:0010959	6	-0.351460	-0.847131	0.695291	1.000000	1.000
445	GOBP_FATTY_ACID_TRANSPORT	GO:0015908	6	-0.351436	-0.851608	0.684432	1.000000	1.000
446	GOBP_REGULATION_OF_CELLULAR_CATABOLIC_PROCESS	GO:0031329	10	-0.300297	-0.851658	0.670935	1.000000	1.000
447	GOBP_POSITIVE_REGULATION_OF_MAPK_CASCADE	GO:0043410	9	-0.312453	-0.852425	0.679296	1.000000	1.000
448	GOBP_POSITIVE_REGULATION_OF_CELL_ADHESION	GO:0045785	7	-0.337509	-0.853958	0.655714	1.000000	1.000
449	REACTOME_CELL_CYCLE_MITOTIC	R-RNO-69278	5	-0.377700	-0.854089	0.672012	1.000000	1.000
450	GOBP_PROTEIN_LOCALIZATION_TO_CELL_JUNCTION	GO:1902414	6	-0.353868	-0.857199	0.675714	1.000000	1.000
451	GOBP_RESPONSE_TO_INORGANIC_SUBSTANCE	GO:0010035	19	-0.253701	-0.857520	0.646635	1.000000	1.000
452	GOBP_REGULATION_OF_CELLULAR_COMPONENT_MOVEMENT	GO:0051270	15	-0.269215	-0.859562	0.635135	1.000000	1.000
453	REACTOME_ESR_MEDIATED_SIGNALING	R-RNO-8939211	8	-0.327527	-0.862059	0.663968	1.000000	1.000
454	GOBP_INORGANIC_ION_TRANSMEMBRANE_TRANSPORT	GO:0098660	9	-0.314256	-0.863061	0.661499	1.000000	1.000
455	GOBP_GROWTH	GO:0040007	14	-0.271291	-0.863402	0.647700	1.000000	1.000
456	GOBP_CELL_MORPHOGENESIS_INVOLVED_IN_NEURON_DIF...	GO:0048667	22	-0.248468	-0.865135	0.661593	1.000000	1.000
457	GOBP_EPHRIN_RECEPTOR_SIGNALING_PATHWAY	GO:0048013	5	-0.371429	-0.866126	0.652299	1.000000	1.000
458	GOBP_CIRCULATORY_SYSTEM_PROCESS	GO:0003013	5	-0.385791	-0.866263	0.632267	1.000000	1.000
459	GOBP_SECRETION	GO:0046903	24	-0.244187	-0.867058	0.647740	1.000000	1.000
460	GOBP_IMMUNE_EFFECTOR_PROCESS	GO:0002252	11	-0.296621	-0.870819	0.627937	1.000000	1.000
461	GOBP_CELL_MORPHOGENESIS_INVOLVED_IN_DIFFERENTI...	GO:0000904	22	-0.248468	-0.872073	0.648585	1.000000	1.000
462	GOBP_CELLULAR_AMIDE_METABOLIC_PROCESS	GO:0043603	12	-0.292983	-0.872282	0.625641	1.000000	1.000
463	GOBP_WOUND_HEALING	GO:0042060	13	-0.287996	-0.872979	0.629032	1.000000	1.000
464	REACTOME_SIGNALING_BY_NUCLEAR_RECEPTORS	R-RNO-9006931	8	-0.327527	-0.873797	0.659489	1.000000	1.000
465	GOBP_PEPTIDYL_LYSINE_MODIFICATION	GO:0018205	5	-0.384058	-0.874839	0.653793	1.000000	1.000
466	GOBP_MUSCLE_STRUCTURE_DEVELOPMENT	GO:0061061	16	-0.270165	-0.875478	0.631579	1.000000	1.000
467	GOBP_REGULATION_OF_PROTEIN_BINDING	GO:0043393	7	-0.344877	-0.876947	0.645333	1.000000	1.000
468	GOBP_NEGATIVE_REGULATION_OF_TRANSFERASE_ACTIVITY	GO:0051348	5	-0.391372	-0.877966	0.651685	1.000000	1.000
469	GOBP_APOPTOTIC_MITOCHONDRIAL_CHANGES	GO:0008637	6	-0.367975	-0.881390	0.615922	1.000000	1.000
470	GOBP_REGULATION_OF_CELL_ADHESION	GO:0030155	13	-0.291838	-0.888093	0.628644	1.000000	1.000

Continuation of Table **S3**

	NAME	ID	SIZE	ES	NES	NOM.p.val	FDR.q.val	FWER.p.val
471	GOBP_NEUROGENESIS	GO:0022008	41	-0.233345	-0.888388	0.602941	1.000000	1.000
472	GOBP_POSITIVE_REGULATION_OF_TRANSPORT	GO:0051050	26	-0.252016	-0.890140	0.620023	1.000000	1.000
473	GOBP_RESPONSE_TO_HEAT	GO:0009408	7	-0.354755	-0.893733	0.613423	1.000000	1.000
474	GOBP_NEGATIVE_REGULATION_OF_KINASE_ACTIVITY	GO:0033673	5	-0.391372	-0.900585	0.610795	1.000000	1.000
475	GOBP_CELL_MIGRATION	GO:0016477	22	-0.255407	-0.901862	0.587544	1.000000	1.000
476	GOBP_ANATOMICAL_STRUCTURE_HOMEOSTASIS	GO:0060249	7	-0.358301	-0.906050	0.609418	1.000000	1.000
477	GOBP_CARBOHYDRATE_DERIVATIVE_BIOSYNTHETIC_PROCESS	GO:1901137	5	-0.401259	-0.909092	0.591777	1.000000	1.000
478	GOBP_CELL_PROJECTION_ORGANIZATION	GO:0030030	35	-0.239652	-0.909930	0.586009	1.000000	1.000
479	GOBP_RESPONSE_TO_CALCIUM_ION	GO:0051592	6	-0.375190	-0.910040	0.608696	1.000000	1.000
480	GOBP_PURINE_CONTAINING_COMPOUND_METABOLIC_PROCESS	GO:0072521	6	-0.383959	-0.910331	0.616162	1.000000	1.000
481	GOBP_REGULATION_OF_HYDROLASE_ACTIVITY	GO:0051336	22	-0.264684	-0.911371	0.586493	1.000000	1.000
482	GOBP_INSULIN_SECRETION	GO:0030073	7	-0.359307	-0.911617	0.573351	1.000000	1.000
483	GOBP_NEGATIVE_REGULATION_OF_SYNAPTIC_TRANSMISSION	GO:0050805	6	-0.375991	-0.913455	0.604082	1.000000	1.000
484	GOBP_POSITIVE_REGULATION_OF_PROTEIN_MODIFICATI...	GO:0031401	22	-0.264057	-0.914785	0.589327	1.000000	1.000
485	GOBP_ORGANOPHOSPHATE_METABOLIC_PROCESS	GO:0019637	8	-0.343181	-0.916551	0.592593	1.000000	1.000
486	GOBP_AMINO_ACID_TRANSPORT	GO:0006865	6	-0.383968	-0.917194	0.598011	1.000000	1.000
487	GOBP_REGULATION_OF_TRANSPORT	GO:0051049	44	-0.241696	-0.920039	0.573184	1.000000	1.000
488	GOBP_PEPTIDE_HORMONE_SECRETION	GO:0030072	7	-0.359307	-0.920183	0.561570	1.000000	1.000
489	GOBP_DEVELOPMENTAL_PROCESS_INVOLVED_IN_REPRODU...	GO:0003006	11	-0.314398	-0.920385	0.602740	1.000000	1.000
490	GOBP_HOMEOSTATIC_PROCESS	GO:0042592	31	-0.251521	-0.922662	0.564825	1.000000	1.000
491	GOBP_MONOCARBOXYLIC_ACID_TRANSPORT	GO:0015718	5	-0.407285	-0.922815	0.603129	1.000000	1.000
492	GOBP_ACIDIC_AMINO_ACID_TRANSPORT	GO:0015800	5	-0.407285	-0.923388	0.577260	1.000000	1.000
493	GOBP_ORGANIC_ANION_TRANSPORT	GO:0015711	6	-0.383968	-0.924567	0.569801	1.000000	1.000
494	GOBP_MAPK_CASCADE	GO:0000165	18	-0.280777	-0.925441	0.574316	1.000000	1.000
495	GOBP_REGULATION_OF_VASCULAR_ASSOCIATED_SMOOTH_...	GO:1904705	5	-0.401350	-0.926727	0.571429	1.000000	1.000
496	GOBP_REGULATION_OF_CELLULAR_RESPONSE_TO_STRESS	GO:0080135	9	-0.337870	-0.927364	0.572549	1.000000	1.000
497	GOBP_NEGATIVE_REGULATION_OF_CELL_PROJECTION_OR...	GO:0031345	5	-0.410470	-0.928369	0.577746	1.000000	1.000
498	GOBP_POSITIVE_REGULATION_OF_PROTEIN_KINASE_ACT...	GO:0045860	18	-0.277517	-0.929140	0.543165	1.000000	1.000
499	GOBP_POSITIVE_REGULATION_OF_PROTEIN_PHOSPHORYL...	GO:0001934	22	-0.264057	-0.929198	0.569428	1.000000	1.000
500	GOBP_LONG_TERM_SYNAPTIC_POTENTIATION	GO:0060291	12	-0.309167	-0.930700	0.568354	1.000000	1.000
501	GOBP_DICARBOXYLIC_ACID_TRANSPORT	GO:0006835	5	-0.407285	-0.930833	0.558989	1.000000	1.000
502	GOBP_CARBOXYLIC_ACID_TRANSPORT	GO:0046942	6	-0.383968	-0.932373	0.566334	1.000000	1.000
503	GOBP_REGULATION_OF_CELL_CYCLE	GO:0051726	13	-0.306685	-0.933800	0.549875	1.000000	1.000
504	GOBP_REGULATION_OF_MUSCLE_CELL_DIFFERENTIATION	GO:0051147	6	-0.388416	-0.935434	0.542373	1.000000	1.000
505	GOBP_METAL_ION_HOMEOSTASIS	GO:0055065	14	-0.298810	-0.936538	0.561064	1.000000	1.000
506	HALLMARK_PI3K_AKT_MTOR_SIGNALING	M5923	6	-0.388454	-0.938234	0.570850	1.000000	1.000
507	GOBP_ION_HOMEOSTASIS	GO:0050801	16	-0.290152	-0.943794	0.547101	1.000000	1.000
508	GOBP_RESPONSE_TO_PEPTIDE_HORMONE	GO:0043434	13	-0.311954	-0.944205	0.526992	1.000000	1.000
509	GOBP_ASSOCIATIVE_LEARNING	GO:0008306	7	-0.372650	-0.945773	0.567227	1.000000	1.000
510	GOBP_AMIDE_BIOSYNTHETIC_PROCESS	GO:0043604	6	-0.386379	-0.948126	0.537517	1.000000	1.000
511	GOBP_PEPTIDE_BIOSYNTHETIC_PROCESS	GO:0043043	6	-0.386379	-0.952491	0.537827	1.000000	1.000
512	GOBP_POSITIVE_REGULATION_OF_PROTEOLYSIS	GO:0045862	8	-0.362631	-0.953400	0.545455	1.000000	1.000
513	GOBP_SKELETAL_MUSCLE_ORGAN_DEVELOPMENT	GO:0060538	5	-0.412002	-0.953945	0.538244	1.000000	1.000

Continuation of Table **S3**

	NAME	ID	SIZE	ES	NES	NOM.p.val	FDR.q.val	FWER.p.val
514	GOBP_INTRACELLULAR_TRANSPORT	GO:0046907	10	-0.334143	-0.956493	0.523377	1.000000	1.000
515	GOBP_NEGATIVE_REGULATION_OF_SIGNALING	GO:0023057	21	-0.281336	-0.961626	0.525822	1.000000	1.000
516	GOBP_EXCITATORY_CHEMICAL_SYNAPTIC_TRANSMISSION	GO:0098976	5	-0.416038	-0.967427	0.497890	1.000000	1.000
517	GOBP_DEVELOPMENTAL_MATURATION	GO:0021700	5	-0.429347	-0.968594	0.526761	1.000000	1.000
518	GOBP_REGULATION_OF_BINDING	GO:0051098	11	-0.328439	-0.968896	0.506953	1.000000	1.000
519	GOBP_ORGANONITROGEN_COMPOUND_BIOSYNTHETIC_PROCESS	GO:1901566	13	-0.315723	-0.969156	0.484549	1.000000	1.000
520	REACTOME_EPH_EPHRIN_SIGNALING	R-RNO-2682334	6	-0.400443	-0.971352	0.516039	1.000000	1.000
521	GOBP_REGULATION_OF_CYTOSKELETON_ORGANIZATION	GO:0051493	5	-0.427073	-0.977426	0.486448	1.000000	1.000
522	REACTOME_NEGATIVE_REGULATION_OF_NMDA_RECEPTOR_...	R-HSA-961732	8	-0.376003	-0.978435	0.536521	1.000000	1.000
523	GOBP_BEHAVIOR	GO:0007610	33	-0.264393	-0.980150	0.498294	1.000000	1.000
524	REACTOME_CIRCADIAN_CLOCK	R-HSA-400253	5	-0.433735	-0.987150	0.475728	1.000000	1.000
525	GOBP_ORGANIC_ACID_METABOLIC_PROCESS	GO:0006082	7	-0.390932	-0.988213	0.507483	1.000000	1.000
526	GOBP_VESICLE_MEDIATED_TRANSPORT_IN_SYNAPSE	GO:0099003	7	-0.389869	-0.989298	0.490566	1.000000	1.000
527	REACTOME_ASSEMBLY_AND_CELL_SURFACE_PRESENTATIO...	R-RNO-9609736	8	-0.376003	-0.990208	0.492773	1.000000	1.000
528	GOBP_MUSCLE_ORGAN_DEVELOPMENT	GO:0007517	9	-0.365246	-0.991137	0.494133	1.000000	1.000
529	GOBP_REGULATION_OF_NERVOUS_SYSTEM_PROCESS	GO:0031644	8	-0.380000	-0.992828	0.501971	1.000000	1.000
530	GOBP_STRIATED_MUSCLE_CELL_DIFFERENTIATION	GO:0051146	9	-0.366495	-0.992997	0.487248	1.000000	1.000
531	GOBP_REGULATION_OF_CYTOSOLIC_CALCIUM_ION_CONCE...	GO:0051480	13	-0.328197	-0.993102	0.480601	1.000000	1.000
532	GOBP_CELL_GROWTH	GO:0016049	10	-0.352177	-0.993878	0.484127	1.000000	1.000
533	GOBP_POSITIVE_REGULATION_OF_CYSTEINE_TYPE_ENDO...	GO:2001056	7	-0.396678	-0.996275	0.475661	1.000000	1.000
534	GOBP_CALCIUM_MEDIATED_SIGNALING	GO:0019722	10	-0.354972	-1.004981	0.470361	1.000000	1.000
535	GOBP_MUSCLE_ADAPTATION	GO:0043500	5	-0.439943	-1.005425	0.445714	1.000000	1.000
536	GOBP_REGULATION_OF_MUSCLE_SYSTEM_PROCESS	GO:0090257	8	-0.379538	-1.007587	0.451482	1.000000	1.000
537	GOBP_DIVALENT_INORGANIC_CATION_HOMEOSTASIS	GO:0072507	13	-0.328197	-1.007683	0.454545	1.000000	1.000
538	GOBP_POSITIVE_REGULATION_OF_PEPTIDASE_ACTIVITY	GO:0010952	7	-0.396678	-1.007808	0.454791	1.000000	1.000
539	GOBP_CELLULAR_RESPONSE_TO_PEPTIDE_HORMONE_STIM...	GO:0071375	9	-0.371106	-1.013165	0.485640	1.000000	1.000
540	GOBP_NERVOUS_SYSTEM_PROCESS	GO:0050877	38	-0.268652	-1.014937	0.461197	1.000000	1.000
541	GOBP_PEPTIDE_SECRETION	GO:0002790	9	-0.369285	-1.014989	0.445178	1.000000	1.000
542	GOBP_REGULATION_OF_MUSCLE_ADAPTATION	GO:0043502	5	-0.439943	-1.018549	0.421499	1.000000	1.000
543	REACTOME_G_ALPHA_Q_SIGNALLING_EVENTS	R-RNO-416476	6	-0.426040	-1.021899	0.442441	1.000000	1.000
544	GOBP_POSITIVE_REGULATION_OF_PROTEIN_SERINE_THR...	GO:0071902	9	-0.367061	-1.023684	0.435762	1.000000	1.000
545	GOBP_POSITIVE_REGULATION_OF_CATALYTIC_ACTIVITY	GO:0043085	29	-0.278673	-1.024653	0.447545	1.000000	1.000
546	GOBP_REGULATION_OF_HORMONE_SECRETION	GO:0046883	8	-0.380487	-1.024964	0.448461	1.000000	1.000
547	GOBP_POSTTRANSCRIPTIONAL_REGULATION_OF_GENE_EX...	GO:0010608	8	-0.389757	-1.025261	0.446721	1.000000	1.000
548	GOBP_CELLULAR_GLUCOSE_HOMEOSTASIS	GO:0001678	5	-0.449349	-1.025656	0.402878	1.000000	1.000
549	GOBP_METAL_ION_TRANSPORT	GO:0030001	11	-0.355398	-1.025877	0.455458	1.000000	1.000
550	GOBP_REGULATION_OF_SYNAPTIC_PLASTICITY	GO:0048167	21	-0.298522	-1.026806	0.453237	1.000000	1.000
551	GOBP_SMALL_MOLECULE_METABOLIC_PROCESS	GO:0044281	16	-0.317535	-1.028401	0.444578	1.000000	1.000
552	GOBP_RESPONSE_TO_ALKALOID	GO:0043279	5	-0.445783	-1.028599	0.410689	1.000000	1.000
553	GOBP_MUSCLE_CONTRACTION	GO:0006936	5	-0.459507	-1.030296	0.426346	1.000000	1.000
554	GOBP_CELL_CELL_SIGNALING_BY_WNT	GO:0198738	7	-0.401564	-1.030696	0.446575	1.000000	1.000
555	REACTOME_CELL_CYCLE	R-RNO-1640170	6	-0.432403	-1.032565	0.432285	1.000000	1.000
556	GOBP_POSITIVE_REGULATION_OF_ION_TRANSPORT	GO:0043270	17	-0.310225	-1.032943	0.443890	1.000000	1.000

Continuation of Table **S3**

	NAME	ID	SIZE	ES	NES	NOM.p.val	FDR.q.val	FWER.p.val
557	GOBP_LONG_TERM_MEMORY	GO:0007616	6	-0.425535	-1.035336	0.434599	1.000000	1.000
558	GOBP_REGULATION_OF_BODY_FLUID_LEVELS	GO:0050878	11	-0.354701	-1.036344	0.434395	1.000000	1.000
559	REACTOME_G_ALPHA_Z_SIGNALLING_EVENTS	R-RNO-418597	5	-0.458829	-1.036891	0.416431	1.000000	1.000
560	GOBP_REGULATION_OF_ANION_TRANSPORT	GO:0044070	20	-0.302923	-1.037633	0.421302	1.000000	1.000
561	GOBP_RESPONSE_TO_KETONE	GO:1901654	7	-0.416952	-1.038390	0.424242	1.000000	1.000
562	GOBP_PROTEIN_LOCALIZATION_TO_SYNAPSE	GO:0035418	5	-0.459578	-1.040562	0.423188	1.000000	1.000
563	REACTOME_VESICLE_MEDIATED_TRANSPORT	R-RNO-5653656	5	-0.456478	-1.041515	0.397882	1.000000	1.000
564	GOBP_PROTEIN_LOCALIZATION_TO_MEMBRANE	GO:0072657	13	-0.344106	-1.041710	0.407814	1.000000	1.000
565	GOBP_MUSCLE_CELL_DIFFERENTIATION	GO:0042692	10	-0.369268	-1.042064	0.410622	1.000000	1.000
566	GOBP_HEAD_DEVELOPMENT	GO:0060322	23	-0.296788	-1.042362	0.434579	1.000000	1.000
567	GOBP_ENDOCRINE_SYSTEM_DEVELOPMENT	GO:0035270	6	-0.441807	-1.043165	0.426982	1.000000	1.000
568	GOBP_POSITIVE_REGULATION_OF_SYNAPTIC_TRANSMISSION	GO:0050806	15	-0.324621	-1.043242	0.419512	1.000000	1.000
569	GOBP_TRANSMEMBRANE_TRANSPORT	GO:0055085	27	-0.293714	-1.044387	0.408884	1.000000	1.000
570	REACTOME_MEMBRANE_TRAFFICKING	R-RNO-199991	5	-0.456478	-1.046892	0.424242	1.000000	1.000
571	GOBP_PROTEIN_LOCALIZATION_TO_POSTSYNAPSE	GO:0062237	5	-0.459578	-1.050103	0.395480	1.000000	1.000
572	GOBP_MONOCARBOXYLIC_ACID_METABOLIC_PROCESS	GO:0032787	6	-0.432593	-1.050564	0.406944	1.000000	1.000
573	GOBP_CALCIUM_ION_TRANSPORT	GO:0006816	11	-0.355398	-1.051212	0.394602	1.000000	1.000
574	GOBP_POSITIVE_REGULATION_OF_MOLECULAR_FUNCTION	GO:0044093	37	-0.275335	-1.051850	0.390190	1.000000	1.000
575	GOBP_CENTRAL_NERVOUS_SYSTEM_DEVELOPMENT	GO:0007417	27	-0.293232	-1.054910	0.401837	1.000000	1.000
576	GOBP_REGULATION_OF_CELL_PROJECTION_ORGANIZATION	GO:0031344	20	-0.303989	-1.055785	0.403129	1.000000	1.000
577	GOBP_DENDRITIC_SPINE_DEVELOPMENT	GO:0060996	6	-0.433566	-1.059645	0.388418	1.000000	1.000
578	REACTOME_BETA_CATENIN_INDEPENDENT_WNT_SIGNALING	R-RNO-3858494	5	-0.464338	-1.059816	0.390244	1.000000	1.000
579	GOBP_DENDRITIC_SPINE_MORPHOGENESIS	GO:0060997	5	-0.468418	-1.060351	0.410587	1.000000	1.000
580	GOBP_HORMONE_TRANSPORT	GO:0009914	9	-0.385155	-1.061188	0.407507	1.000000	1.000
581	GOBP_REGULATION_OF_NEUROTRANSMITTER_TRANSPORT	GO:0051588	6	-0.437356	-1.061976	0.393602	1.000000	1.000
582	GOBP_REGULATION_OF_PEPTIDE_SECRETION	GO:0002791	7	-0.428562	-1.066200	0.383448	1.000000	1.000
583	GOBP_MULTICELLULAR_ORGANISM_REPRODUCTION	GO:0032504	12	-0.353999	-1.066530	0.395379	1.000000	1.000
584	GOBP_ENDOMEMBRANE_SYSTEM_ORGANIZATION	GO:0010256	6	-0.440212	-1.070988	0.386301	1.000000	1.000
585	GOBP_REGULATION_OF_SYSTEM_PROCESS	GO:0044057	18	-0.326021	-1.087235	0.349879	1.000000	1.000
586	GOBP_REGULATION_OF_PEPTIDE_HORMONE_SECRETION	GO:0090276	6	-0.453527	-1.089411	0.340845	1.000000	1.000
587	GOBP_CELL_CYCLE	GO:0007049	21	-0.320869	-1.090761	0.362884	1.000000	1.000
588	GOBP_MUSCLE_SYSTEM_PROCESS	GO:0003012	9	-0.399627	-1.095387	0.350785	1.000000	1.000
589	GOBP_MYELOID_LEUKOCYTE_ACTIVATION	GO:0002274	8	-0.413495	-1.096025	0.359211	1.000000	1.000
590	GOBP_REGULATION_OF_CELLULAR_LOCALIZATION	GO:0060341	17	-0.336435	-1.099643	0.354293	1.000000	1.000
591	GOBP_NEGATIVE_REGULATION_OF_GENE_EXPRESSION	GO:0010629	11	-0.375249	-1.102073	0.355082	1.000000	1.000
592	GOBP_RESPONSE_TO_METAL_ION	GO:0010038	15	-0.344591	-1.103536	0.341104	1.000000	1.000
593	GOBP_MUSCLE_CELL_DEVELOPMENT	GO:0055001	5	-0.482136	-1.104087	0.325037	1.000000	1.000
594	REACTOME_LONG_TERM_POTENTIATION	R-HSA-9620244	10	-0.383744	-1.104610	0.346053	1.000000	1.000
595	GOBP_REGULATION_OF_POSTSYNAPTIC_MEMBRANE_POTEN...	GO:0060078	14	-0.355357	-1.104734	0.336145	1.000000	1.000
596	REACTOME_MAPK_FAMILY_SIGNALING_CASCADES	R-RNO-5683057	12	-0.370644	-1.105365	0.338812	1.000000	1.000
597	REACTOME_PTEN_REGULATION	R-RNO-6807070	5	-0.487796	-1.105616	0.332394	1.000000	1.000
598	GOBP_NUCLEOBASE_CONTAINING_SMALL_MOLECULE_META...	GO:0055086	7	-0.437991	-1.106261	0.336634	1.000000	1.000
599	REACTOME_NEUTROPHIL_DEGRANULATION	R-RNO-6798695	6	-0.461177	-1.114084	0.339489	1.000000	1.000

Continuation of Table **S3**

	NAME	ID	SIZE	ES	NES	NOM.p.val	FDR.q.val	FWER.p.val
600	GOBP_MYELOID_LEUKOCYTE_MEDIATED_IMMUNITY	GO:0002444	6	-0.461177	-1.119506	0.331915	1.000000	1.000
601	GOBP_CELL_JUNCTION_ORGANIZATION	GO:0034330	19	-0.337812	-1.120207	0.320096	1.000000	1.000
602	GOBP_GAMETE_GENERATION	GO:0007276	9	-0.411991	-1.121207	0.345997	1.000000	1.000
603	GOBP_CELLULAR_ION_HOMEOSTASIS	GO:0006873	14	-0.358165	-1.123210	0.317897	1.000000	1.000
604	GOBP_REGULATION_OF_SYNAPTIC_TRANSMISSION_GLUTA...	GO:0051966	12	-0.381041	-1.124200	0.317132	1.000000	1.000
605	REACTOME_TRAFFICKING_OF_GLUR2_CONTAINING_AMPA_...	R-RNO-416993	8	-0.430031	-1.126362	0.332414	1.000000	1.000
606	GOBP_NEGATIVE_REGULATION_OF_CELL_CYCLE	GO:0045786	5	-0.492412	-1.126754	0.288406	1.000000	1.000
607	GOBP_CELLULAR_RESPONSE_TO_ALCOHOL	GO:0097306	5	-0.493793	-1.127890	0.309659	1.000000	1.000
608	GOBP_REGULATION_OF_VESICLE_MEDIATED_TRANSPORT	GO:0060627	12	-0.370861	-1.128881	0.316391	1.000000	1.000
609	GOBP_CYTOSKELETON_ORGANIZATION	GO:0007010	11	-0.384753	-1.129941	0.304562	1.000000	1.000
610	GOBP_PROTEIN_LOCALIZATION_TO_CELL_PERIPHERY	GO:1990778	12	-0.376633	-1.129960	0.319255	1.000000	1.000
611	GOBP_SENSORY_ORGAN_DEVELOPMENT	GO:0007423	8	-0.427594	-1.131100	0.311081	1.000000	1.000
612	GOBP_POSITIVE_REGULATION_OF_SECRETION	GO:0051047	6	-0.464879	-1.134765	0.314841	1.000000	1.000
613	REACTOME_RNA_POLYMERASE_II_TRANSCRIPTION	R-RNO-73857	20	-0.333792	-1.137549	0.301663	1.000000	1.000
614	REACTOME_SIGNALING_BY_WNT	R-RNO-195721	7	-0.454792	-1.144613	0.303867	1.000000	1.000
615	GOBP_POSITIVE_REGULATION_OF_PROTEIN_METABOLIC_...	GO:0051247	27	-0.319058	-1.145538	0.306620	1.000000	1.000
616	GOBP_CELLULAR_LIPID_METABOLIC_PROCESS	GO:0044255	7	-0.451062	-1.147858	0.296296	1.000000	1.000
617	GOBP_CELL_ACTIVATION_INVOLVED_IN_IMMUNE_RESPONSE	GO:0002263	6	-0.461177	-1.149319	0.289291	1.000000	1.000
618	GOBP_CARBOHYDRATE_HOMEOSTASIS	GO:0033500	7	-0.449946	-1.152053	0.306430	1.000000	1.000
619	REACTOME_CLASS_C_3_METABOTROPIC_GLUTAMATE_PHER...	R-RNO-420499	7	-0.448677	-1.152288	0.288952	1.000000	1.000
620	GOBP_REGULATION_OF_ORGANELLE_ORGANIZATION	GO:0033043	14	-0.363498	-1.152732	0.280247	1.000000	1.000
621	GOBP_CHEMICAL_HOMEOSTASIS	GO:0048878	23	-0.330853	-1.154995	0.288235	1.000000	1.000
622	GOBP_LEARNING	GO:0007612	13	-0.376278	-1.159820	0.276029	1.000000	1.000
623	GOBP_REGULATION_OF_ENDOCYTOSIS	GO:0030100	6	-0.473851	-1.160275	0.280537	1.000000	1.000
624	GOBP_CELL_SURFACE_RECEPTOR_SIGNALING_PATHWAY_I...	GO:1905114	15	-0.364596	-1.164498	0.276775	1.000000	1.000
625	GOBP_MITOCHONDRION_ORGANIZATION	GO:0007005	7	-0.460814	-1.170978	0.262948	1.000000	1.000
626	REACTOME_RAS_ACTIVATION_UPON_CA2_INFLUX_THROUG...	R-HSA-442982	6	-0.487805	-1.177339	0.251366	1.000000	1.000
627	GOBP_SYNAPTIC_SIGNALING	GO:0099536	47	-0.304911	-1.178939	0.258602	1.000000	1.000
628	GOBP_GLUCOSE_METABOLIC_PROCESS	GO:0006006	6	-0.490797	-1.180926	0.260388	1.000000	1.000
629	GOBP_NUCLEAR_TRANSPORT	GO:0051169	5	-0.511968	-1.188470	0.252427	1.000000	1.000
630	GOBP_CELLULAR_HOMEOSTASIS	GO:0019725	18	-0.356988	-1.190694	0.245983	1.000000	1.000
631	GOBP_MONOSACCHARIDE_METABOLIC_PROCESS	GO:0005996	6	-0.490797	-1.193563	0.248237	1.000000	1.000
632	GOBP_NEUROTRANSMITTER_TRANSPORT	GO:0006836	7	-0.477525	-1.198238	0.244475	1.000000	1.000
633	GOBP_REGULATION_OF_NEURONAL_SYNAPTIC_PLASTICITY	GO:0048168	7	-0.470940	-1.204600	0.221477	1.000000	1.000
634	GOBP_SECOND_MESSENGER_MEDIATED_SIGNALING	GO:0019932	16	-0.373197	-1.211378	0.233633	1.000000	1.000
635	GOBP_INTRACELLULAR_PROTEIN_TRANSPORT	GO:0006886	8	-0.460040	-1.211394	0.210598	1.000000	1.000
636	GOBP_POSITIVE_REGULATION_OF_ESTABLISHMENT_OF_P...	GO:1904951	5	-0.531804	-1.212494	0.198847	1.000000	1.000
637	GOBP_REGULATION_OF_NEUROTRANSMITTER_LEVELS	GO:0001505	7	-0.477525	-1.212632	0.236016	1.000000	1.000
638	GOBP_REGULATION_OF_MEMBRANE_POTENTIAL	GO:0042391	17	-0.367862	-1.214250	0.222089	1.000000	1.000
639	GOBP_CHEMICAL_SYNAPTIC_TRANSMISSION_POSTSYNAPTIC	GO:0099565	9	-0.444164	-1.215004	0.241470	1.000000	1.000
640	GOBP_POSITIVE_REGULATION_OF_CELLULAR_PROTEIN_L...	GO:1903829	7	-0.477558	-1.223132	0.225585	1.000000	1.000
641	GOBP_ENDOCYTOSIS	GO:0006897	8	-0.463492	-1.224305	0.221622	1.000000	1.000
642	REACTOME_NEUREXINS_AND_NEUROLIGINS	R-RNO-6794361	9	-0.442768	-1.225097	0.226978	1.000000	1.000

Continuation of Table **S3**

	NAME	ID	SIZE	ES	NES	NOM.p.val	FDR.q.val	FWER.p.val
643	GOBP_ESTABLISHMENT_OF_PROTEIN_LOCALIZATION	GO:0045184	17	-0.365777	-1.230078	0.207108	1.000000	1.000
644	GOBP_NEGATIVE_REGULATION_OF_BINDING	GO:0051100	6	-0.511318	-1.231544	0.199726	1.000000	1.000
645	GOBP_MALE_GAMETE_GENERATION	GO:0048232	5	-0.542169	-1.235283	0.207232	1.000000	1.000
646	REACTOME_CA_DEPENDENT_EVENTS	R-RNO-111996	8	-0.462500	-1.242206	0.198413	1.000000	1.000
647	GOBP_REGULATION_OF_SECRETION	GO:0051046	14	-0.399329	-1.244348	0.205000	1.000000	1.000
648	REACTOME_SIGNALING_BY_RHO_GTPASES_MIRO_GTPASES...	R-RNO-9716542	9	-0.452156	-1.247256	0.184000	1.000000	1.000
649	GOBP_POSITIVE_REGULATION_OF_SIGNALING	GO:0023056	34	-0.331306	-1.247854	0.210465	1.000000	1.000
650	GOBP_RESPONSE_TO_ETHANOL	GO:0045471	5	-0.548100	-1.250737	0.193687	1.000000	1.000
651	GOBP_NEUROTRANSMITTER_SECRETION	GO:0007269	6	-0.510490	-1.250904	0.180617	1.000000	1.000
652	GOBP_DEPHOSPHORYLATION	GO:0016311	7	-0.490047	-1.251874	0.210235	1.000000	1.000
653	GOBP_SEXUAL_REPRODUCTION	GO:0019953	10	-0.437601	-1.253531	0.199741	1.000000	1.000
654	REACTOME_RHO_GTPASE_EFFECTORS	R-RNO-195258	9	-0.452156	-1.257324	0.187332	1.000000	1.000
655	GOBP_REGULATION_OF_PROTEIN_LOCALIZATION	GO:0032880	18	-0.377804	-1.261681	0.191697	1.000000	1.000
656	GOBP_G_PROTEIN_COUPLED_GLUTAMATE_RECEPTOR_SIGN...	GO:0007216	8	-0.477630	-1.263065	0.165554	1.000000	1.000
657	REACTOME_INTRACELLULAR_SIGNALING_BY_SECOND_MES...	R-RNO-9006925	15	-0.393939	-1.264675	0.193827	1.000000	1.000
658	GOBP_CELLULAR_RESPONSE_TO_KETONE	GO:1901655	5	-0.555812	-1.268112	0.169034	1.000000	1.000
659	GOBP_POSTSYNAPSE_ORGANIZATION	GO:0099173	10	-0.443231	-1.270217	0.179183	1.000000	1.000
660	GOBP_PROTEIN_DEPHOSPHORYLATION	GO:0006470	7	-0.490047	-1.271305	0.162382	1.000000	1.000
661	REACTOME_TRAFFICKING_OF_AMPA_RECEPTORS	R-RNO-399719	11	-0.441558	-1.274518	0.175796	1.000000	1.000
662	REACTOME_GPCR_LIGAND_BINDING	R-RNO-500792	8	-0.476379	-1.275162	0.177333	1.000000	1.000
663	REACTOME_TRANSCRIPTIONAL_REGULATION_BY_MECP2	R-RNO-8986944	7	-0.493827	-1.276374	0.157895	1.000000	1.000
664	GOBP_NUCLEOSIDE_PHOSPHATE_BIOSYNTHETIC_PROCESS	GO:1901293	5	-0.568589	-1.290962	0.156204	1.000000	1.000
665	GOBP_SYNAPTIC_TRANSMISSION_GLUTAMATERGIC	GO:0035249	13	-0.424684	-1.291277	0.154135	1.000000	1.000
666	GOBP_REGULATION_OF_CELL_CYCLE_PROCESS	GO:0010564	5	-0.560271	-1.292221	0.179379	1.000000	1.000
667	REACTOME_G_PROTEIN_MEDIATED_EVENTS	R-RNO-112040	9	-0.468354	-1.298769	0.150063	1.000000	1.000
668	GOBP_ORGANOPHOSPHATE_BIOSYNTHETIC_PROCESS	GO:0090407	5	-0.568589	-1.303493	0.120235	1.000000	1.000
669	GOBP_REGULATION_OF_TRANS_SYNAPTIC_SIGNALING	GO:0099177	40	-0.347238	-1.307300	0.160398	1.000000	1.000
670	GOBP_SENSORY_PERCEPTION_OF_PAIN	GO:0019233	5	-0.578313	-1.313459	0.143917	1.000000	1.000
671	GOBP_REGULATION_OF_SYNAPSE_STRUCTURE_OR_ACTIVITY	GO:0050803	11	-0.450475	-1.317153	0.141582	1.000000	1.000
672	REACTOME_UNBLOCKING_OF_NMDA_RECEPTORS_GLUTAMAT...	R-RNO-438066	12	-0.446363	-1.319514	0.144686	1.000000	1.000
673	GOBP_LOCOMOTORY_BEHAVIOR	GO:0007626	8	-0.494504	-1.323714	0.128631	1.000000	1.000
674	GOBP_SIGNAL_RELEASE	GO:0023061	14	-0.424209	-1.325561	0.145038	1.000000	1.000
675	GOBP_SYNAPSE_ORGANIZATION	GO:0050808	17	-0.401657	-1.326474	0.150188	1.000000	1.000
676	GOBP_ADENYLATE_CYCLASE_INHIBITING_G_PROTEIN_CO...	GO:0007196	5	-0.580153	-1.327959	0.130127	1.000000	1.000
677	GOBP_CELLULAR_COMPONENT_MAINTENANCE	GO:0043954	5	-0.587260	-1.330923	0.128427	1.000000	1.000
678	GOBP_REGULATION_OF_CELLULAR_PROTEIN_LOCALIZATION	GO:1903827	12	-0.440894	-1.333195	0.137056	1.000000	1.000
679	GOBP_LEUKOCYTE_MEDIATED_IMMUNITY	GO:0002443	8	-0.494080	-1.334947	0.123457	1.000000	1.000
680	REACTOME_CREB1_PHOSPHORYLATION_THROUGH_NMDA_RE...	R-RNO-442742	8	-0.500000	-1.352929	0.128134	1.000000	1.000
681	GOBP_MITOTIC_CELL_CYCLE	GO:0000278	10	-0.482837	-1.360933	0.118100	1.000000	1.000
682	GOBP_ADENYLATE_CYCLASE_INHIBITING_G_PROTEIN_CO...	GO:0007193	9	-0.491023	-1.367327	0.109354	1.000000	1.000
683	REACTOME_TRANSCRIPTIONAL_REGULATION_BY_TP53	R-RNO-3700989	6	-0.565151	-1.375273	0.085135	1.000000	1.000
684	REACTOME_PROTEIN_PROTEIN_INTERACTIONS_AT_SYNAPSES	R-RNO-6794362	12	-0.469600	-1.379378	0.116834	1.000000	1.000
685	GOBP_RESPIRATORY_SYSTEM_DEVELOPMENT	GO:0060541	6	-0.574009	-1.388794	0.097855	1.000000	1.000

Continuation of Table **S3**

	NAME	ID	SIZE	ES	NES	NOM.p.val	FDR.q.val	FWER.p.val
686	REACTOME_INTEGRATION_OF_ENERGY_METABOLISM	R-RNO-163685	5	-0.614458	-1.389489	0.095791	1.000000	1.000
687	GOBP_CELL_CELL_SIGNALING	GO:0007267	56	-0.376284	-1.403726	0.091620	1.000000	1.000
688	GOBP_RESPONSE_TO_ALCOHOL	GO:0097305	11	-0.486403	-1.406502	0.063144	1.000000	1.000
689	REACTOME_SYNAPTIC_ADHESION_LIKE_MOLECULES	R-RNO-8849932	9	-0.518656	-1.408335	0.099596	1.000000	1.000
690	GOBP_DENDRITE_DEVELOPMENT	GO:0016358	10	-0.506783	-1.414846	0.091731	1.000000	1.000
691	GOBP_IONOTROPIC_GLUTAMATE_RECEPTOR_SIGNALING_P...	GO:0035235	9	-0.520588	-1.418908	0.095238	1.000000	1.000
692	GOBP_CATION_TRANSPORT	GO:0006812	25	-0.393409	-1.421790	0.068525	1.000000	1.000
693	GOBP_NEURON_PROJECTION_ORGANIZATION	GO:0106027	8	-0.535198	-1.424414	0.097754	1.000000	1.000
694	GOBP_CATION_TRANSMEMBRANE_TRANSPORT	GO:0098655	18	-0.426629	-1.427653	0.085612	1.000000	1.000
695	GOBP_MYOTUBE_DIFFERENTIATION	GO:0014902	5	-0.635713	-1.432647	0.093151	1.000000	1.000
696	GOBP_REGULATION_OF_ION_TRANSPORT	GO:0043269	36	-0.379300	-1.434439	0.094145	1.000000	1.000
697	GOBP_REGULATION_OF_TRANSMEMBRANE_TRANSPORT	GO:0034762	23	-0.409189	-1.434890	0.082854	1.000000	1.000
698	REACTOME_OPIOID_SIGNALLING	R-RNO-111885	12	-0.486842	-1.450133	0.080051	1.000000	1.000
699	GOBP_REGULATION_OF_PEPTIDE_TRANSPORT	GO:0090087	10	-0.517736	-1.458708	0.063211	1.000000	1.000
700	REACTOME_ACTIVATION_OF_NMDA_RECEPTORS_AND_POST...	R-RNO-442755	16	-0.458006	-1.474546	0.062726	1.000000	1.000
701	GOBP_REGULATION_OF_POSTSYNAPSE_ORGANIZATION	GO:0099175	6	-0.610022	-1.500633	0.057103	1.000000	1.000
702	GOBP_DENDRITE_MORPHOGENESIS	GO:0048813	7	-0.587215	-1.508011	0.042466	1.000000	1.000
703	GOBP_ADENYLATE_CYCLASE_MODULATING_G_PROTEIN_CO...	GO:0007188	11	-0.517337	-1.509981	0.048346	1.000000	1.000
704	REACTOME_NEUROTRANSMITTER_RECEPTORS_AND_POSTSY...	R-RNO-112314	22	-0.456908	-1.546301	0.025060	0.867925	1.000
705	GOBP_REGULATION_OF_TRANSPORTER_ACTIVITY	GO:0032409	17	-0.466937	-1.552568	0.032927	0.875311	1.000
706	GOBP_CELL_CYCLE_PROCESS	GO:0022402	11	-0.546612	-1.584895	0.027379	0.735448	1.000
707	REACTOME_SIGNALING_BY_GPCR	R-RNO-372790	22	-0.457918	-1.594328	0.033058	0.726802	1.000
708	GOBP_GLUTAMATE_RECEPTOR_SIGNALING_PATHWAY	GO:0007215	17	-0.497391	-1.611705	0.020910	0.679855	1.000
709	GOBP_CELLULAR_MACROMOLECULE_LOCALIZATION	GO:0070727	20	-0.466129	-1.617401	0.021557	0.693508	1.000
710	GOBP_G_PROTEIN_COUPLED_RECEPTOR_SIGNALING_PATHWAY	GO:0007186	18	-0.490504	-1.625658	0.023370	0.697881	1.000
711	GOBP_REGULATION_OF_CATION_TRANSMEMBRANE_TRANSPORT	GO:1904062	16	-0.508995	-1.629910	0.021066	0.724018	1.000
712	GOBP_PROTEIN_COMPLEX_OLIGOMERIZATION	GO:0051259	7	-0.649794	-1.652536	0.012195	0.656236	1.000
713	GOBP_SENSORY_PERCEPTION	GO:0007600	9	-0.607595	-1.660205	0.014286	0.670151	1.000
714	GOBP_REGULATION_OF_NMDA_RECEPTOR_ACTIVITY	GO:2000310	10	-0.584148	-1.664863	0.020970	0.706904	1.000
715	REACTOME_TRANSMISSION_ACROSS_CHEMICAL_SYNAPSES	R-RNO-112315	23	-0.467033	-1.664978	0.018957	0.777070	1.000
716	REACTOME_NEURONAL_SYSTEM	R-RNO-112316	26	-0.466845	-1.684079	0.010490	0.744275	1.000
717	REACTOME_G_ALPHA_I_SIGNALLING_EVENTS	R-RNO-418594	18	-0.512476	-1.706904	0.019656	0.699389	0.995
718	GOBP_PROTEIN_TETRAMERIZATION	GO:0051262	5	-0.759258	-1.739027	0.005690	0.613457	0.989
719	GOBP_PROTEIN_CONTAINING_COMPLEX_SUBUNIT_ORGANI...	GO:0043933	15	-0.566463	-1.801572	0.006242	0.417409	0.948
720	GOBP_ESTABLISHMENT_OF_PROTEIN_LOCALIZATION_TO_...	GO:0072594	5	-0.789795	-1.804116	0.004213	0.487702	0.941
721	GOBP_PROTEIN_LOCALIZATION_TO_ORGANELLE	GO:0033365	8	-0.685173	-1.823297	0.004104	0.519052	0.908
722	GOBP_REGULATION_OF_SIGNALING_RECEPTOR_ACTIVITY	GO:0010469	14	-0.604349	-1.873540	0.001211	0.431636	0.773
723	GOBP_REGULATION_OF_CATION_CHANNEL_ACTIVITY	GO:2001257	14	-0.593628	-1.873590	0.004950	0.647454	0.773
724	GOBP_REGULATION_OF_NEUROTRANSMITTER_RECEPTOR_A...	GO:0099601	13	-0.623822	-1.920449	0.000000	0.800690	0.602

End of Table

Supplementary Table S4: SC rostral segment late (45 DPL peak) profile GSEA results.

Begin of Table **S4**

	NAME	ID	SIZE	ES	NES	NOM.p.val	FDR.q.val	FWER.p.val
0	GOBP_SYNAPTIC_SIGNALING	GO:0099536	47	0.579961	2.018370	0.001142	0.110040	0.101
1	GOBP_CELL_CELL_SIGNALING	GO:0007267	56	0.573256	2.007903	0.000000	0.064290	0.117
2	REACTOME_NEURONAL_SYSTEM	R-RNO-112316	26	0.594129	1.945261	0.000000	0.104607	0.267
3	GOBP_REGULATION_OF_TRANS_SYNAPTIC_SIGNALING	GO:0099177	40	0.549315	1.942583	0.000000	0.081764	0.277
4	REACTOME_NEUROTRANSMITTER_RECEPTORS_AND_POSTSY...	R-RNO-112314	22	0.582464	1.874906	0.003788	0.152251	0.520
5	REACTOME_TRANSMISSION_ACROSS_CHEMICAL_SYNAPSES	R-RNO-112315	23	0.559181	1.809385	0.004878	0.268486	0.775
6	GOBP_G_PROTEIN_COUPLED_RECEPTOR_SIGNALING_PATHWAY	GO:0007186	18	0.584833	1.792612	0.000000	0.279094	0.825
7	GOBP_POSITIVE_REGULATION_OF_TRANSMEMBRANE_TRAN...	GO:0034764	7	0.742789	1.775269	0.001497	0.291396	0.877
8	GOBP_POSITIVE_REGULATION_OF_SIGNALING	GO:0023056	34	0.483082	1.706538	0.008140	0.532927	0.985
9	GOBP_REGULATION_OF_TRANSPORT	GO:0051049	44	0.481413	1.691242	0.013953	0.551299	0.989
10	GOBP_CHEMICAL_HOMEOSTASIS	GO:0048878	23	0.525372	1.685761	0.013580	0.527675	0.995
11	GOBP_GLIOGENESIS	GO:0042063	12	0.604378	1.678670	0.015805	0.512521	0.996
12	REACTOME_DAG_AND_IP3_SIGNALING	R-RNO-1489509	8	0.686209	1.657882	0.011645	0.575414	1.000
13	GOBP_CATION_TRANSPORT	GO:0006812	25	0.506346	1.648938	0.017348	0.573248	1.000
14	REACTOME_ACTIVATION_OF_NMDA_RECEPTORS_AND_POST...	R-RNO-442755	16	0.542285	1.647503	0.020888	0.541463	1.000
15	GOBP_NEURON_DIFFERENTIATION	GO:0030182	37	0.475443	1.645604	0.012926	0.515312	1.000
16	REACTOME_SIGNALING_BY_GPCR	R-RNO-372790	22	0.509412	1.644095	0.017789	0.491757	1.000
17	GOBP_METAL_ION_HOMEOSTASIS	GO:0055065	14	0.562136	1.635932	0.015625	0.499041	1.000
18	GOBP_REGULATION_OF_POSTSYNAPTIC_MEMBRANE_POTEN...	GO:0060078	14	0.570756	1.633557	0.028683	0.481558	1.000
19	GOBP_EPITHELIAL_CELL_DIFFERENTIATION	GO:0030855	7	0.678777	1.623725	0.011940	0.497586	1.000
20	GOBP_MAPK_CASCADE	GO:0000165	18	0.534074	1.622645	0.021330	0.478529	1.000
21	GOBP_VESICLE_MEDIATED_TRANSPORT_IN_SYNAPSE	GO:0099003	7	0.686091	1.611744	0.016035	0.500597	1.000
22	GOBP_NEURON_DEVELOPMENT	GO:0048666	34	0.464378	1.610901	0.024706	0.482076	1.000
23	GOBP_ION_HOMEOSTASIS	GO:0050801	16	0.549220	1.609969	0.019430	0.466643	1.000
24	GOBP_REGULATION_OF_NEUROTRANSMITTER_TRANSPORT	GO:0051588	6	0.703024	1.606855	0.017699	0.459377	1.000
25	GOBP_DIVALENT_INORGANIC_CATION_HOMEOSTASIS	GO:0072507	13	0.569745	1.604588	0.026144	0.449745	1.000
26	GOBP_REGULATION_OF_MEMBRANE_POTENTIAL	GO:0042391	17	0.537767	1.602527	0.022251	0.440312	1.000
27	GOBP_REGULATION_OF_CYTOSOLIC_CALCIUM_ION_CONCE...	GO:0051480	13	0.569745	1.590337	0.028340	0.468599	1.000
28	GOBP_CELLULAR_ION_HOMEOSTASIS	GO:0006873	14	0.546138	1.557727	0.036339	0.581443	1.000
29	GOBP_PROTEIN_PHOSPHORYLATION	GO:0006468	33	0.454898	1.555198	0.026253	0.572667	1.000
30	GOBP_REGULATION_OF_CIRCADIAN_RHYTHM	GO:0042752	7	0.641028	1.549596	0.041298	0.577128	1.000
31	GOBP_REGULATION_OF_SYNAPTIC_PLASTICITY	GO:0048167	21	0.491107	1.535360	0.039390	0.623613	1.000
32	GOBP_POSITIVE_REGULATION_OF_CATION_TRANSMEMBRA...	GO:1904064	5	0.712489	1.531388	0.042813	0.623568	1.000
33	REACTOME_TRAFFICKING_OF_AMPA_RECEPTORS	R-RNO-399719	11	0.567396	1.523031	0.051842	0.642349	1.000
34	GOBP_REGULATION_OF_ANION_TRANSMEMBRANE_TRANSPORT	GO:1903959	5	0.722378	1.520107	0.039557	0.635747	1.000
35	GOBP_REGULATION_OF_ION_TRANSPORT	GO:0043269	36	0.436332	1.503304	0.038005	0.696873	1.000
36	GOBP_SIGNAL_RELEASE	GO:0023061	14	0.529178	1.502856	0.043709	0.680147	1.000
37	GOBP_CELL_PROJECTION_ORGANIZATION	GO:0030030	35	0.428223	1.502620	0.042824	0.663185	1.000
38	GOBP_PROTEIN_AUTOPHOSPHORYLATION	GO:0046777	5	0.698243	1.502291	0.034226	0.647679	1.000
39	GOBP_GLUTAMATE_RECEPTOR_SIGNALING_PATHWAY	GO:0007215	17	0.492958	1.501925	0.050378	0.632919	1.000
40	GOBP_POSITIVE_REGULATION_OF_SYNAPTIC_TRANSMISSION	GO:0050806	15	0.524178	1.498120	0.047493	0.632802	1.000

Continuation of Table **S4**

	NAME	ID	SIZE	ES	NES	NOM.p.val	FDR.q.val	FWER.p.val
41	GOBP_NEUROGENESIS	GO:0022008	41	0.424696	1.497123	0.056977	0.622551	1.000
42	GOBP_POSITIVE_REGULATION_OF_LONG_TERM_SYNAPTIC...	GO:1900273	5	0.708574	1.495042	0.039334	0.616362	1.000
43	REACTOME_CA_DEPENDENT_EVENTS	R-RNO-111996	8	0.600000	1.490308	0.059972	0.622924	1.000
44	GOBP_FOREBRAIN_DEVELOPMENT	GO:0030900	10	0.559943	1.483596	0.055021	0.636809	1.000
45	GOBP_REGULATION_OF_NEURON_PROJECTION_DEVELOPMENT	GO:0010975	16	0.497489	1.477156	0.051813	0.650835	1.000
46	REACTOME_G_PROTEIN_MEDIATED_EVENTS	R-RNO-112040	9	0.583518	1.474614	0.058156	0.647549	1.000
47	GOBP_REGULATION_OF_SYNAPTIC_TRANSMISSION_GLUTA...	GO:0051966	12	0.540506	1.474359	0.069930	0.634990	1.000
48	REACTOME_G_ALPHA_I_SIGNALLING_EVENTS	R-RNO-418594	18	0.480973	1.470582	0.048114	0.638257	1.000
49	GOBP_REGULATION_OF_NEUROTRANSMITTER_LEVELS	GO:0001505	7	0.611760	1.459971	0.064706	0.669806	1.000
50	GOBP_CATION_TRANSMEMBRANE_TRANSPORT	GO:0098655	18	0.477400	1.455205	0.059585	0.676904	1.000
51	GOBP_REGULATION_OF_LONG_TERM_SYNAPTIC_POTENTIA...	GO:1900271	6	0.650541	1.454531	0.066971	0.666718	1.000
52	HALLMARK_PI3K_AKT_MTOR_SIGNALING	M5923	6	0.631691	1.448507	0.079595	0.679976	1.000
53	REACTOME_ANTI_INFLAMMATORY_RESPONSE_FAVOURING_...	R-HSA-9662851	5	0.674785	1.445246	0.056782	0.681858	1.000
54	GOBP_REGULATION_OF_MAPK_CASCADE	GO:0043408	13	0.508563	1.444475	0.081365	0.672951	1.000
55	GOBP_NEGATIVE_REGULATION_OF_NEURON_DEATH	GO:1901215	11	0.529470	1.441110	0.079167	0.674995	1.000
56	GOBP_REGULATION_OF_CELL_PROJECTION_ORGANIZATION	GO:0031344	20	0.462295	1.437908	0.063492	0.676172	1.000
57	GOBP_SECRETION	GO:0046903	24	0.443305	1.432622	0.067155	0.685987	1.000
58	GOBP_ENDOCRINE_SYSTEM_DEVELOPMENT	GO:0035270	6	0.634596	1.428083	0.069630	0.693336	1.000
59	GOBP_NEUROTRANSMITTER_TRANSPORT	GO:0006836	7	0.611760	1.426992	0.076923	0.685810	1.000
60	GOBP_LONG_TERM_SYNAPTIC_POTENTIATION	GO:0060291	12	0.501933	1.418136	0.099440	0.712403	1.000
61	GOBP_DENDRITIC_SPINE_DEVELOPMENT	GO:0060996	6	0.641713	1.417268	0.071429	0.704022	1.000
62	GOBP_POSITIVE_REGULATION_OF_TRANSFERASE_ACTIVITY	GO:0051347	19	0.456771	1.415556	0.082310	0.700072	1.000
63	GOBP_REGULATION_OF_NEUROTRANSMITTER_RECEPTOR_A...	GO:0099601	13	0.493333	1.411806	0.088235	0.704203	1.000
64	GOBP_SYNAPTIC_TRANSMISSION_GLUTAMATERGIC	GO:0035249	13	0.497764	1.408940	0.100134	0.704999	1.000
65	GOBP_EMBRYO_DEVELOPMENT	GO:0009790	14	0.493873	1.405106	0.094315	0.710393	1.000
66	REACTOME_PROTEIN_PROTEIN_INTERACTIONS_AT_SYNAPSES	R-RNO-6794362	12	0.503261	1.404700	0.088742	0.701685	1.000
67	GOBP_NEUROTRANSMITTER_SECRETION	GO:0007269	6	0.623782	1.398775	0.087021	0.715529	1.000
68	REACTOME_UNBLOCKING_OF_NMDA_RECEPTORS_GLUTAMAT...	R-RNO-438066	12	0.501524	1.397337	0.095041	0.710650	1.000
69	GOBP_REGULATION_OF_SYNAPSE_STRUCTURE_OR_ACTIVITY	GO:0050803	11	0.514527	1.395172	0.091160	0.709182	1.000
70	GOBP_PROTEIN_KINASE_B_SIGNALING	GO:0043491	6	0.608340	1.390999	0.072485	0.716416	1.000
71	GOBP_NEURON_PROJECTION_ORGANIZATION	GO:0106027	8	0.554762	1.385954	0.102962	0.727270	1.000
72	GOBP_HOMEOSTATIC_PROCESS	GO:0042592	31	0.407834	1.383536	0.109785	0.727190	1.000
73	GOBP_POSITIVE_REGULATION_OF_PHOSPHORUS_METABOL...	GO:0010562	23	0.428263	1.383436	0.087871	0.717900	1.000
74	GOBP_INTRACELLULAR_PROTEIN_TRANSPORT	GO:0006886	8	0.563900	1.383387	0.092725	0.708518	1.000
75	GOBP_DNA_METABOLIC_PROCESS	GO:0006259	6	0.603794	1.380114	0.106782	0.712272	1.000
76	GOBP_POSITIVE_REGULATION_OF_TRANSPORT	GO:0051050	26	0.418634	1.379564	0.092121	0.705358	1.000
77	GOBP_FATTY_ACID_TRANSPORT	GO:0015908	6	0.613403	1.375902	0.096296	0.710335	1.000
78	GOBP_REGULATION_OF_METAL_ION_TRANSPORT	GO:0010959	6	0.612046	1.375379	0.097701	0.703529	1.000
79	REACTOME_OPIOID_SIGNALLING	R-RNO-111885	12	0.505538	1.373717	0.117881	0.701432	1.000
80	GOBP_EMBRYO_DEVELOPMENT_ENDING_IN_BIRTH_OR_EGG...	GO:0009792	9	0.541671	1.373420	0.096206	0.693914	1.000
81	GOBP_MEMBRANE_ORGANIZATION	GO:0061024	12	0.497731	1.369614	0.115127	0.700429	1.000
82	GOBP_CELLULAR_HOMEOSTASIS	GO:0019725	18	0.446059	1.366585	0.108005	0.703142	1.000
83	GOBP_DEVELOPMENTAL_MATURATION	GO:0021700	5	0.639897	1.363756	0.088647	0.705808	1.000

Continuation of Table **S4**

	NAME	ID	SIZE	ES	NES	NOM.p.val	FDR.q.val	FWER.p.val
84	GOBP_REGULATION_OF_CATION_TRANSMEMBRANE_TRANSPORT	GO:1904062	16	0.459204	1.360986	0.108639	0.707809	1.000
85	GOBP_REGULATION_OF_CELLULAR_COMPONENT_SIZE	GO:0032535	7	0.582271	1.360645	0.108470	0.700857	1.000
86	GOBP_RECEPTOR_MEDIATED_ENDOCYTOSIS	GO:0006898	6	0.610298	1.360455	0.111278	0.693533	1.000
87	GOBP_NERVOUS_SYSTEM_PROCESS	GO:0050877	38	0.391443	1.354058	0.119952	0.709812	1.000
88	GOBP_INTRACELLULAR_TRANSPORT	GO:0046907	10	0.517764	1.345684	0.127778	0.733854	1.000
89	GOBP_GLIAL_CELL_DIFFERENTIATION	GO:0010001	10	0.512735	1.343954	0.128671	0.732435	1.000
90	GOBP_REGULATION_OF_SECRETION	GO:0051046	14	0.464158	1.343068	0.140000	0.727919	1.000
91	GOBP_PROTEIN_LOCALIZATION_TO_MEMBRANE	GO:0072657	13	0.475718	1.334250	0.131169	0.753535	1.000
92	GOBP_MAINTENANCE_OF_LOCATION	GO:0051235	7	0.564066	1.334043	0.147661	0.746349	1.000
93	REACTOME_DISEASES_OF_SIGNAL_TRANSDUCTION_BY_GR...	R-HSA-5663202	12	0.491715	1.330985	0.128205	0.750687	1.000
94	GOBP_BEHAVIOR	GO:0007610	33	0.389457	1.323750	0.132544	0.771390	1.000
95	REACTOME_LONG_TERM_POTENTIATION	R-HSA-9620244	10	0.508939	1.323673	0.148611	0.763622	1.000
96	GOBP_ANION_TRANSMEMBRANE_TRANSPORT	GO:0098656	7	0.568040	1.323154	0.127168	0.757683	1.000
97	GOBP_CELLULAR_RESPONSE_TO_PEPTIDE	GO:1901653	13	0.476672	1.323139	0.148344	0.750026	1.000
98	GOBP_ORGANIC_ACID_TRANSPORT	GO:0015849	7	0.558650	1.322334	0.140173	0.745305	1.000
99	GOBP_REGULATION_OF_INTRACELLULAR_SIGNAL_TRANSD...	GO:1902531	21	0.422217	1.321694	0.141772	0.740270	1.000
100	GOBP_RECEPTOR_INTERNALIZATION	GO:0031623	5	0.620759	1.321207	0.143082	0.734710	1.000
101	GOBP_AMIDE_BIOSYNTHETIC_PROCESS	GO:0043604	6	0.591841	1.318286	0.128130	0.738419	1.000
102	GOBP_POSITIVE_REGULATION_OF_PROTEIN_PHOSPHORYL...	GO:0001934	22	0.406778	1.318069	0.130923	0.731984	1.000
103	GOBP_POSITIVE_REGULATION_OF_PROTEIN_KINASE_ACT...	GO:0045860	18	0.435845	1.316417	0.149808	0.731421	1.000
104	GOBP_REGULATION_OF_ANATOMICAL_STRUCTURE_SIZE	GO:0090066	10	0.500570	1.316354	0.155525	0.724622	1.000
105	REACTOME_NEUREXINS_AND_NEUROLIGINS	R-RNO-6794361	9	0.520212	1.314834	0.152661	0.723479	1.000
106	GOBP_PEPTIDE_BIOSYNTHETIC_PROCESS	GO:0043043	6	0.591841	1.314536	0.151468	0.717611	1.000
107	GOBP_TELENCEPHALON_DEVELOPMENT	GO:0021537	7	0.560174	1.312849	0.136364	0.716742	1.000
108	GOBP_POSITIVE_REGULATION_OF_PROTEIN_MODIFICATI...	GO:0031401	22	0.406778	1.310951	0.132242	0.716793	1.000
109	GOBP_RESPONSE_TO_HEAT	GO:0009408	7	0.549858	1.310516	0.153509	0.711925	1.000
110	GOBP_LOCOMOTORY_BEHAVIOR	GO:0007626	8	0.516631	1.306170	0.152661	0.721032	1.000
111	GOBP_POSITIVE_REGULATION_OF_PROTEIN_TYROSINE_K...	GO:0061098	5	0.617877	1.304195	0.137255	0.721372	1.000
112	REACTOME_CREB1_PHOSPHORYLATION_THROUGH_NMDA_RE...	R-RNO-442742	8	0.539183	1.300960	0.146240	0.726313	1.000
113	GOBP_REGULATION_OF_PROTEIN_TYROSINE_KINASE_ACT...	GO:0061097	5	0.617877	1.300480	0.134884	0.721783	1.000
114	REACTOME_INTRACELLULAR_SIGNALING_BY_SECOND_MES...	R-RNO-9006925	15	0.454645	1.297759	0.174373	0.725626	1.000
115	GOBP_RECEPTOR_METABOLIC_PROCESS	GO:0043112	5	0.620759	1.296794	0.152239	0.722572	1.000
116	GOBP_CELLULAR_RESPONSE_TO_PEPTIDE_HORMONE_STIM...	GO:0071375	9	0.505660	1.295991	0.158996	0.719248	1.000
117	GOBP_CELLULAR_RESPONSE_TO_KETONE	GO:1901655	5	0.616481	1.291990	0.154671	0.727216	1.000
118	REACTOME_TRAFFICKING_OF_GLUR2_CONTAINING_AMPA_...	R-RNO-416993	8	0.525000	1.288915	0.156835	0.731842	1.000
119	GOBP_CELLULAR_CARBOHYDRATE_METABOLIC_PROCESS	GO:0044262	5	0.610153	1.284127	0.170659	0.742307	1.000
120	GOBP_MULTICELLULAR_ORGANISMAL_RESPONSE_TO_STRESS	GO:0033555	5	0.609690	1.283818	0.164274	0.737240	1.000
121	GOBP_INORGANIC_ION_TRANSMEMBRANE_TRANSPORT	GO:0098660	9	0.507767	1.283019	0.175732	0.733839	1.000
122	REACTOME_RAS_ACTIVATION_UPON_CA2_INFLUX_THROUG...	R-HSA-442982	6	0.559221	1.280167	0.183976	0.737928	1.000
123	GOBP_REGULATION_OF_CELLULAR_RESPONSE_TO_STRESS	GO:0080135	9	0.508009	1.277486	0.170270	0.741338	1.000
124	REACTOME_HEMOSTASIS	R-RNO-109582	14	0.437739	1.276463	0.188602	0.738982	1.000
125	GOBP_CELLULAR_MACROMOLECULE_CATABOLIC_PROCESS	GO:0044265	5	0.602410	1.275100	0.176012	0.738070	1.000
126	GOBP_DENDRITIC_SPINE_MORPHOGENESIS	GO:0060997	5	0.603090	1.275074	0.158879	0.732388	1.000

Continuation of Table **S4**

	NAME	ID	SIZE	ES	NES	NOM.p.val	FDR.q.val	FWER.p.val
127	GOBP_DENDRITE_DEVELOPMENT	GO:0016358	10	0.482082	1.272149	0.176634	0.736172	1.000
128	GOBP_POSITIVE_REGULATION_OF_MAPK_CASCADE	GO:0043410	9	0.497392	1.271583	0.180441	0.732556	1.000
129	GOBP_POSITIVE_REGULATION_OF_PEPTIDYL_TYROSINE_...	GO:0050731	8	0.514072	1.271141	0.182584	0.728341	1.000
130	GOBP_GENERATION_OF_PRECURSOR_METABOLITES_AND_E...	GO:0006091	5	0.589069	1.270828	0.189655	0.723808	1.000
131	GOBP_CELL_SURFACE_RECEPTOR_SIGNALING_PATHWAY_I...	GO:1905114	15	0.435118	1.269584	0.161125	0.722606	1.000
132	GOBP_PEPTIDYL_TYROSINE_MODIFICATION	GO:0018212	10	0.486489	1.269439	0.186080	0.717651	1.000
133	REACTOME_G_ALPHA_Z_SIGNALLING_EVENTS	R-RNO-418597	5	0.602859	1.267927	0.152568	0.717230	1.000
134	REACTOME_GPCR_LIGAND_BINDING	R-RNO-500792	8	0.510051	1.265641	0.187675	0.719279	1.000
135	GOBP_POLYSACCHARIDE_METABOLIC_PROCESS	GO:0005976	5	0.607272	1.264903	0.158209	0.716380	1.000
136	GOBP_RESPONSE_TO_OXYGEN_LEVELS	GO:0070482	8	0.515302	1.263274	0.172414	0.716545	1.000
137	GOBP_REGULATION_OF_CATION_CHANNEL_ACTIVITY	GO:2001257	14	0.441079	1.261669	0.170410	0.716605	1.000
138	GOBP_POSITIVE_REGULATION_OF_PROTEIN_SERINE_THR...	GO:0071902	9	0.494234	1.261106	0.196676	0.713260	1.000
139	GOBP_REGULATION_OF_CELL_CYCLE_PROCESS	GO:0010564	5	0.594825	1.253227	0.188940	0.733618	1.000
140	GOBP_REGULATION_OF_VESICLE_MEDIATED_TRANSPORT	GO:0060627	12	0.454150	1.252509	0.195448	0.730799	1.000
141	GOBP_METAL_ION_TRANSPORT	GO:0030001	11	0.464498	1.252133	0.182306	0.726764	1.000
142	GOBP_CALCIUM_ION_TRANSPORT	GO:0006816	11	0.464498	1.248122	0.195418	0.734911	1.000
143	GOBP_REGULATION_OF_NMDA_RECEPTOR_ACTIVITY	GO:2000310	10	0.477212	1.246781	0.195155	0.734046	1.000
144	GOBP_SYNAPSE_ORGANIZATION	GO:0050808	17	0.419689	1.245985	0.211068	0.731638	1.000
145	GOBP_G_PROTEIN_COUPLED_GLUTAMATE_RECEPTOR_SIGN...	GO:0007216	8	0.503567	1.243015	0.196078	0.736440	1.000
146	GOBP_CARBOHYDRATE_DERIVATIVE_BIOSYNTHETIC_PROCESS	GO:1901137	5	0.589478	1.238245	0.200949	0.746510	1.000
147	REACTOME_ASSEMBLY_AND_CELL_SURFACE_PRESENTATIO...	R-RNO-9609736	8	0.504520	1.237257	0.204986	0.744476	1.000
148	GOBP_HOMEOSTASIS_OF_NUMBER_OF_CELLS	GO:0048872	5	0.573973	1.237131	0.197819	0.739995	1.000
149	REACTOME_NEGATIVE_REGULATION_OF_NMDA_RECEPTOR_...	R-HSA-9617324	8	0.504520	1.236479	0.204578	0.736964	1.000
150	GOBP_PROTEIN_LOCALIZATION_TO_PLASMA_MEMBRANE	GO:0072659	7	0.529955	1.236350	0.235211	0.732559	1.000
151	GOBP_TRANSMEMBRANE_TRANSPORT	GO:0055085	27	0.368599	1.234274	0.219927	0.734088	1.000
152	GOBP_RESPONSE_TO_NERVE_GROWTH_FACTOR	GO:1990089	7	0.519358	1.233172	0.212950	0.732638	1.000
153	GOBP_REGULATION_OF_SIGNALING_RECEPTOR_ACTIVITY	GO:0010469	14	0.431773	1.233082	0.205094	0.728168	1.000
154	REACTOME_CELL_CYCLE_MITOTIC	R-RNO-69278	5	0.578313	1.221772	0.221169	0.759163	1.000
155	GOBP_LEUKOCYTE_MIGRATION	GO:0050900	6	0.539314	1.216719	0.217325	0.769892	1.000
156	REACTOME_CELL_CYCLE	R-RNO-1640170	6	0.539383	1.214278	0.213043	0.772589	1.000
157	GOBP_POSITIVE_REGULATION_OF_EPITHELIAL_CELL_MI...	GO:0010634	5	0.574039	1.207091	0.251149	0.791010	1.000
158	GOBP_REGULATION_OF_RECEPTOR_MEDIATED_ENDOCYTOSIS	GO:0048259	5	0.577510	1.206497	0.250789	0.788136	1.000
159	REACTOME_SIGNALING_BY_WNT	R-RNO-195721	7	0.506173	1.206333	0.243590	0.783780	1.000
160	GOBP_CHEMICAL_SYNAPTIC_TRANSMISSION_POSTSYNAPTIC	GO:0099565	9	0.471181	1.205457	0.235955	0.781809	1.000
161	GOBP_REGULATION_OF_CELL_CELL_ADHESION	GO:0022407	9	0.472497	1.205031	0.239456	0.778373	1.000
162	GOBP_ORGANOPHOSPHATE_BIOSYNTHETIC_PROCESS	GO:0090407	5	0.549784	1.204295	0.228788	0.776065	1.000
163	REACTOME_SYNAPTIC_ADHESION_LIKE_MOLECULES	R-RNO-8849932	9	0.468354	1.202595	0.246238	0.776761	1.000
164	GOBP_MONOCARBOXYLIC_ACID_TRANSPORT	GO:0015718	5	0.553897	1.195709	0.264970	0.794178	1.000
165	GOBP_REGULATION_OF_PHOSPHOLIPASE_C_ACTIVITY	GO:1900274	5	0.557106	1.195374	0.230061	0.790660	1.000
166	HALLMARK_ALLOGRAFT_REJECTION	M5950	7	0.513553	1.195126	0.250737	0.786618	1.000
167	GOBP_IONOTROPIC_GLUTAMATE_RECEPTOR_SIGNALING_P...	GO:0035235	9	0.468354	1.193073	0.235616	0.788203	1.000
168	GOBP_PROTEIN_LOCALIZATION_TO_CELL_PERIPHERY	GO:1990778	12	0.433173	1.189817	0.268358	0.793923	1.000
169	GOBP_REGULATION_OF_RESPONSE_TO_STRESS	GO:0080134	19	0.381756	1.188860	0.249354	0.792258	1.000

Continuation of Table **S4**

	NAME	ID	SIZE	ES	NES	NOM.p.val	FDR.q.val	FWER.p.val
170	GOBP_REGULATION_OF_TRANSFERASE_ACTIVITY	GO:0051338	21	0.367372	1.185726	0.249357	0.797990	1.000
171	GOBP_REGULATION_OF_CELLULAR_AMIDE_METABOLIC_PR...	GO:0034248	9	0.471808	1.184840	0.270604	0.796164	1.000
172	GOBP_POSTTRANSCRIPTIONAL_REGULATION_OF_GENE_EX...	GO:0010608	8	0.477916	1.183684	0.251788	0.795305	1.000
173	GOBP_ADENYLATE_CYCLASE_MODULATING_G_PROTEIN_CO...	GO:0007188	11	0.435822	1.183207	0.271117	0.792168	1.000
174	GOBP_HORMONE_TRANSPORT	GO:0009914	9	0.463124	1.181749	0.249645	0.792226	1.000
175	REACTOME_PLATELET_ACTIVATION_SIGNALING_AND_AGG...	R-HSA-76002	7	0.497009	1.179868	0.236417	0.793585	1.000
176	GOBP_POSTSYNAPSE_ORGANIZATION	GO:0099173	10	0.449413	1.178675	0.267930	0.792805	1.000
177	REACTOME_ADAPTIVE_IMMUNE_SYSTEM	R-RNO-1280218	9	0.460450	1.177702	0.263523	0.791374	1.000
178	GOBP_NEGATIVE_REGULATION_OF_CELLULAR_COMPONENT...	GO:0051129	11	0.428419	1.176470	0.279778	0.790660	1.000
179	REACTOME_INTEGRATION_OF_ENERGY_METABOLISM	R-RNO-163685	5	0.561380	1.172774	0.270016	0.797194	1.000
180	GOBP_NUCLEOSIDE_PHOSPHATE_BIOSYNTHETIC_PROCESS	GO:1901293	5	0.549784	1.171665	0.258114	0.796059	1.000
181	GOBP_EPITHELIUM_DEVELOPMENT	GO:0060429	15	0.396540	1.169844	0.272727	0.796939	1.000
182	GOBP_POSITIVE_REGULATION_OF_CATALYTIC_ACTIVITY	GO:0043085	29	0.342752	1.168338	0.280095	0.797170	1.000
183	GOBP_ACIDIC_AMINO_ACID_TRANSPORT	GO:0015800	5	0.553897	1.165902	0.266568	0.800249	1.000
184	GOBP_NEGATIVE_REGULATION_OF_NEURON_APOPTOTIC_P...	GO:0043524	8	0.478864	1.165851	0.284900	0.796056	1.000
185	GOBP_POSITIVE_REGULATION_OF_LIPASE_ACTIVITY	GO:0060193	5	0.557106	1.164667	0.267647	0.795199	1.000
186	GOBP_REGULATION_OF_PROTEIN_PHOSPHORYLATION	GO:0001932	25	0.353012	1.164341	0.297899	0.791923	1.000
187	REACTOME_INTERFERON_SIGNALING	R-RNO-913531	6	0.521953	1.163054	0.273121	0.791801	1.000
188	GOBP_SECOND_MESSENGER_MEDIATED_SIGNALING	GO:0019932	16	0.386272	1.162848	0.286632	0.788203	1.000
189	GOBP_PROTEIN_LOCALIZATION_TO_SYNAPSE	GO:0035418	5	0.547774	1.162813	0.275148	0.784141	1.000
190	GOBP_REGULATION_OF_PROTEIN_KINASE_ACTIVITY	GO:0045859	21	0.367372	1.161385	0.282828	0.784599	1.000
191	GOBP_LIPID_LOCALIZATION	GO:0010876	10	0.444690	1.160056	0.279673	0.784614	1.000
192	GOBP_ENDOMEMBRANE_SYSTEM_ORGANIZATION	GO:0010256	6	0.504932	1.158611	0.302256	0.784914	1.000
193	GOBP_DICARBOXYLIC_ACID_TRANSPORT	GO:0006835	5	0.553897	1.152390	0.273148	0.799763	1.000
194	GOBP_PROTEIN_LOCALIZATION_TO_POSTSYNAPSE	GO:0062237	5	0.547774	1.150931	0.292719	0.799718	1.000
195	GOBP_NEGATIVE_REGULATION_OF_LIPID_METABOLIC_PR...	GO:0045833	5	0.537670	1.145263	0.299410	0.812103	1.000
196	GOBP_ERBB_SIGNALING_PATHWAY	GO:0038127	5	0.542406	1.143138	0.293853	0.814441	1.000
197	GOBP_REGULATION_OF_PROTEIN_MODIFICATION_PROCESS	GO:0031399	28	0.343347	1.141716	0.306683	0.814348	1.000
198	GOBP_SENSORY_PERCEPTION	GO:0007600	9	0.443038	1.141515	0.295794	0.810846	1.000
199	GOBP_REGULATION_OF_PEPTIDYL_TYROSINE_PHOSPHORY...	GO:0050730	9	0.445379	1.141050	0.329496	0.808162	1.000
200	REACTOME_G_ALPHA_Q_SIGNALLING_EVENTS	R-RNO-416476	6	0.503150	1.140285	0.294479	0.806429	1.000
201	GOBP_NEGATIVE_REGULATION_OF_PROTEOLYSIS	GO:0045861	8	0.456454	1.138464	0.306003	0.807768	1.000
202	GOBP_REGULATION_OF_ANATOMICAL_STRUCTURE_MORPHO...	GO:0022603	17	0.378239	1.136317	0.318822	0.809834	1.000
203	GOBP_PROTEIN_CONTAINING_COMPLEX_SUBUNIT_ORGANI...	GO:0043933	15	0.387062	1.135811	0.307298	0.807366	1.000
204	GOBP_NEURON_MIGRATION	GO:0001764	5	0.541191	1.135138	0.304878	0.805346	1.000
205	GOBP_SENSORY_PERCEPTION_OF_PAIN	GO:0019233	5	0.542169	1.134866	0.320840	0.802285	1.000
206	REACTOME_SIGNALING_BY_NTRK2_TRKB	R-RNO-9006115	6	0.497214	1.134555	0.305263	0.799438	1.000
207	GOBP_CELL_JUNCTION_ORGANIZATION	GO:0034330	19	0.369685	1.133070	0.310038	0.799933	1.000
208	GOBP_REGULATION_OF_CARBOHYDRATE_METABOLIC_PROCESS	GO:0006109	5	0.532634	1.131260	0.314985	0.801172	1.000
209	GOBP_GAMETE_GENERATION	GO:0007276	9	0.445295	1.130425	0.324728	0.799742	1.000
210	GOBP_CELLULAR_RESPONSE_TO_ALCOHOL	GO:0097306	5	0.533158	1.126608	0.320127	0.806980	1.000
211	GOBP_CIRCADIAN_RHYTHM	GO:0007623	13	0.392686	1.123823	0.323722	0.811168	1.000
212	GOBP_REGULATION_OF_LIPASE_ACTIVITY	GO:0060191	6	0.494158	1.123699	0.337798	0.807673	1.000

Continuation of Table **S4**

	NAME	ID	SIZE	ES	NES	NOM.p.val	FDR.q.val	FWER.p.val
213	GOBP_INSULIN_SECRETION	GO:0030073	7	0.475216	1.122864	0.301860	0.806225	1.000
214	GOBP_REGULATION_OF_CARBOHYDRATE_BIOSYNTHETIC_P...	GO:0043255	5	0.532634	1.121811	0.326187	0.805766	1.000
215	GOBP_PEPTIDE_HORMONE_SECRETION	GO:0030072	7	0.475216	1.121725	0.339595	0.802278	1.000
216	GOBP_ENDOCYTOSIS	GO:0006897	8	0.456329	1.120751	0.306294	0.801415	1.000
217	GOBP_POSITIVE_REGULATION_OF_NEURON_PROJECTION_...	GO:0010976	5	0.529076	1.120034	0.305215	0.799736	1.000
218	GOBP_CELLULAR_RESPONSE_TO_DNA_DAMAGE_STIMULUS	GO:0006974	5	0.529417	1.119047	0.326594	0.798809	1.000
219	GOBP_AXON_DEVELOPMENT	GO:0061564	18	0.372379	1.118752	0.319948	0.795951	1.000
220	GOBP_POSITIVE_REGULATION_OF_CELL_PROJECTION_OR...	GO:0031346	10	0.430290	1.118138	0.338356	0.794043	1.000
221	GOBP_REGULATION_OF_PHOSPHOLIPASE_ACTIVITY	GO:0010517	6	0.494158	1.115957	0.313869	0.796594	1.000
222	GOBP_REGULATION_OF_FAT_CELL_DIFFERENTIATION	GO:0045598	6	0.496602	1.112642	0.323034	0.802203	1.000
223	REACTOME_CLASS_C_3_METABOTROPIC_GLUTAMATE_PHER...	R-RNO-420499	7	0.470785	1.111284	0.326440	0.802371	1.000
224	GOBP_REGULATION_OF_PHOSPHORUS_METABOLIC_PROCESS	GO:0051174	26	0.340835	1.111158	0.335793	0.799064	1.000
225	GOBP_SEXUAL_REPRODUCTION	GO:0019953	10	0.421247	1.110413	0.338398	0.797432	1.000
226	GOBP_POSITIVE_REGULATION_OF_SMALL_MOLECULE_MET...	GO:0062013	5	0.517755	1.105990	0.360414	0.806017	1.000
227	GOBP_CARBOHYDRATE_BIOSYNTHETIC_PROCESS	GO:0016051	5	0.532634	1.105962	0.362170	0.802527	1.000
228	GOBP_CELLULAR_RESPONSE_TO_BIOTIC_STIMULUS	GO:0071216	8	0.449604	1.103410	0.356421	0.806134	1.000
229	GOBP_ACTIVATION_OF_PROTEIN_KINASE_ACTIVITY	GO:0032147	10	0.420417	1.103163	0.363128	0.803325	1.000
230	GOBP_POSITIVE_REGULATION_OF_ION_TRANSPORT	GO:0043270	17	0.357175	1.102194	0.344828	0.802466	1.000
231	GOBP_PEPTIDE_METABOLIC_PROCESS	GO:0006518	11	0.404986	1.101216	0.333786	0.801723	1.000
232	REACTOME_EXTRA_NUCLEAR_ESTROGEN_SIGNALING	R-RNO-9009391	7	0.469816	1.099607	0.365639	0.802347	1.000
233	GOBP_PEPTIDYL_AMINO_ACID_MODIFICATION	GO:0018193	22	0.347168	1.098630	0.344516	0.801327	1.000
234	GOBP_PEPTIDYL_SERINE_MODIFICATION	GO:0018209	12	0.400815	1.098596	0.354620	0.798044	1.000
235	GOBP_REGULATION_OF_NERVOUS_SYSTEM_PROCESS	GO:0031644	8	0.448495	1.096209	0.359124	0.801025	1.000
236	GOBP_POSITIVE_REGULATION_OF_INTRACELLULAR_SIGN...	GO:1902533	14	0.386304	1.095641	0.367876	0.799246	1.000
237	GOBP_CARBOXYLIC_ACID_TRANSPORT	GO:0046942	6	0.486287	1.094817	0.364023	0.798153	1.000
238	GOBP_REGULATION_OF_ENDOCYTOSIS	GO:0030100	6	0.488403	1.091068	0.358273	0.804840	1.000
239	GOBP_REGULATION_OF_LYMPHOCYTE_ACTIVATION	GO:0051249	5	0.512694	1.090718	0.376662	0.802491	1.000
240	GOBP_REGULATION_OF_CELLULAR_LOCALIZATION	GO:0060341	17	0.360359	1.086984	0.358974	0.808917	1.000
241	GOBP_CELLULAR_RESPONSE_TO_NITROGEN_COMPOUND	GO:1901699	20	0.347124	1.085746	0.347066	0.808992	1.000
242	GOBP_LIPID_EXPORT_FROM_CELL	GO:0140353	5	0.514408	1.085536	0.377273	0.806267	1.000
243	GOBP_REGULATION_OF_CELL_SIZE	GO:0008361	5	0.507039	1.083736	0.373660	0.807708	1.000
244	GOBP_CELLULAR_RESPONSE_TO_MOLECULE_OF_BACTERIA...	GO:0071219	8	0.449604	1.083038	0.372832	0.806246	1.000
245	GOBP_REGULATION_OF_LIPID_METABOLIC_PROCESS	GO:0019216	7	0.456705	1.082406	0.366197	0.804784	1.000
246	GOBP_AMINO_ACID_TRANSPORT	GO:0006865	6	0.486287	1.080411	0.377644	0.806660	1.000
247	GOBP_POSITIVE_REGULATION_OF_PROTEIN_METABOLIC_...	GO:0051247	27	0.324883	1.080379	0.387057	0.803481	1.000
248	GOBP_RESPONSE_TO_TEMPERATURE_STIMULUS	GO:0009266	9	0.419237	1.078318	0.365922	0.805461	1.000
249	GOBP_SENSORY_ORGAN_DEVELOPMENT	GO:0007423	8	0.436187	1.078197	0.387955	0.802538	1.000
250	GOBP_ORGANIC_ANION_TRANSPORT	GO:0015711	6	0.486287	1.076716	0.398485	0.803101	1.000
251	GOBP_PHOSPHATIDYLINOSITOL_3_KINASE_SIGNALING	GO:0014065	7	0.460838	1.074921	0.385307	0.804358	1.000
252	REACTOME_ESTROGEN_DEPENDENT_NUCLEAR_EVENTS_DOW...	R-RNO-9634638	5	0.507116	1.072643	0.391097	0.807100	1.000
253	GOBP_NEGATIVE_REGULATION_OF_TRANSMEMBRANE_TRAN...	GO:0034763	6	0.474901	1.072587	0.381657	0.804087	1.000
254	GOBP_ESTABLISHMENT_OF_PROTEIN_LOCALIZATION_TO_...	GO:0072594	5	0.498861	1.066284	0.372727	0.816997	1.000
255	GOBP_REGULATION_OF_ENDOTHELIAL_CELL_MIGRATION	GO:0010594	5	0.512557	1.063341	0.387906	0.821107	1.000

Continuation of Table **S4**

	NAME	ID	SIZE	ES	NES	NOM.p.val	FDR.q.val	FWER.p.val
256	GOBP_CELL_PART_MORPHOGENESIS	GO:0032990	25	0.319443	1.062620	0.387704	0.819715	1.000
257	GOBP_NEGATIVE_REGULATION_OF_PHOSPHORYLATION	GO:0042326	7	0.455951	1.062284	0.398827	0.817361	1.000
258	GOBP_NEGATIVE_REGULATION_OF_TRANSFERASE_ACTIVITY	GO:0051348	5	0.503944	1.062028	0.377916	0.814830	1.000
259	GOBP_CYTOSOLIC_CALCIUM_ION_TRANSPORT	GO:0060401	7	0.446263	1.061413	0.395620	0.813276	1.000
260	GOBP_REGULATION_OF_PROTEIN_LOCALIZATION_TO_MEM...	GO:1905475	7	0.458088	1.061076	0.393275	0.810935	1.000
261	GOBP_NEGATIVE_REGULATION_OF_TRANSPORT	GO:0051051	13	0.373642	1.060524	0.405333	0.809225	1.000
262	GOBP_PEPTIDE_SECRETION	GO:0002790	9	0.415105	1.059109	0.408769	0.809604	1.000
263	GOBP_REGULATION_OF_POSTSYNAPSE_ORGANIZATION	GO:0099175	6	0.461052	1.058055	0.381381	0.809335	1.000
264	REACTOME_TRANSCRIPTIONAL_REGULATION_BY_MECP2	R-RNO-8986944	7	0.438468	1.057455	0.407895	0.807806	1.000
265	GOBP_REGULATION_OF_HORMONE_SECRETION	GO:0046883	8	0.423324	1.057053	0.409692	0.805710	1.000
266	GOBP_ADENYLATE_CYCLASE_INHIBITING_G_PROTEIN_CO...	GO:0007193	9	0.413450	1.056288	0.401934	0.804657	1.000
267	GOBP_CENTRAL_NERVOUS_SYSTEM_NEURON_DIFFERENTIA...	GO:0021953	5	0.500231	1.056038	0.416918	0.802214	1.000
268	GOBP_NEGATIVE_REGULATION_OF_KINASE_ACTIVITY	GO:0033673	5	0.503944	1.054626	0.423304	0.802997	1.000
269	REACTOME_CIRCADIAN_CLOCK	R-HSA-400253	5	0.492116	1.052682	0.407353	0.804726	1.000
270	GOBP_CELLULAR_RESPONSE_TO_HORMONE_STIMULUS	GO:0032870	11	0.394869	1.052319	0.429155	0.802596	1.000
271	GOBP_CELLULAR_COMPONENT_MORPHOGENESIS	GO:0032989	25	0.319443	1.050897	0.405868	0.803311	1.000
272	GOBP_OSTEOBLAST_DIFFERENTIATION	GO:0001649	5	0.507381	1.048911	0.420091	0.805052	1.000
273	GOBP_CALCIUM_ION_TRANSPORT_INTO_CYTOSOL	GO:0060402	7	0.446263	1.048042	0.417867	0.804162	1.000
274	GOBP_CALCIUM_ION_TRANSMEMBRANE_TRANSPORT	GO:0070588	7	0.446263	1.047815	0.418535	0.801829	1.000
275	GOBP_CELL_MORPHOGENESIS	GO:0000902	25	0.319443	1.047670	0.404551	0.799320	1.000
276	GOBP_GLAND_DEVELOPMENT	GO:0048732	7	0.439702	1.043832	0.413013	0.805554	1.000
277	GOBP_IN_UTERO_EMBRYONIC_DEVELOPMENT	GO:0001701	8	0.425462	1.043619	0.405564	0.803203	1.000
278	REACTOME_BETA_CATENIN_INDEPENDENT_WNT_SIGNALING	R-RNO-3858494	5	0.493976	1.043054	0.432229	0.801683	1.000
279	GOBP_REGULATION_OF_CELL_ADHESION	GO:0030155	13	0.367387	1.041175	0.435762	0.803407	1.000
280	GOBP_REGULATION_OF_SYSTEM_PROCESS	GO:0044057	18	0.338266	1.039680	0.431790	0.804120	1.000
281	GOBP_CELLULAR_RESPONSE_TO_LIPID	GO:0071396	15	0.358252	1.034645	0.415894	0.813123	1.000
282	GOBP_REGULATION_OF_NERVOUS_SYSTEM_DEVELOPMENT	GO:0051960	15	0.356778	1.033285	0.437018	0.813330	1.000
283	GOBP_CIRCULATORY_SYSTEM_PROCESS	GO:0003013	5	0.489964	1.032767	0.426035	0.811723	1.000
284	GOBP_REGULATION_OF_EPITHELIAL_CELL_MIGRATION	GO:0010632	6	0.466564	1.032077	0.444444	0.810513	1.000
285	GOBP_PROTEIN_COMPLEX_OLIGOMERIZATION	GO:0051259	7	0.434238	1.029524	0.435345	0.813413	1.000
286	GOBP_POSITIVE_REGULATION_OF_CELLULAR_PROTEIN_L...	GO:1903829	7	0.441007	1.029496	0.433824	0.810658	1.000
287	GOBP_REGULATION_OF_MAP_KINASE_ACTIVITY	GO:0043405	9	0.405286	1.028634	0.435211	0.809717	1.000
288	GOBP_REGULATION_OF_AXONOGENESIS	GO:0050770	7	0.427567	1.025789	0.422414	0.813690	1.000
289	GOBP_ORGANONITROGEN_COMPOUND_BIOSYNTHETIC_PROCESS	GO:1901566	13	0.360086	1.024842	0.438070	0.813031	1.000
290	GOBP_NEGATIVE_REGULATION_OF_CYSTEINE_TYPE_ENDO...	GO:2000117	5	0.481255	1.024445	0.462639	0.811197	1.000
291	GOBP_DENDRITE_MORPHOGENESIS	GO:0048813	7	0.430445	1.023566	0.459227	0.810268	1.000
292	GOBP_EXOCYTOSIS	GO:0006887	11	0.375334	1.018884	0.440771	0.818217	1.000
293	GOBP_RESPONSE_TO_ALKALOID	GO:0043279	5	0.481383	1.017365	0.462250	0.818853	1.000
294	GOBP_CELL_CYCLE_PROCESS	GO:0022402	11	0.378601	1.016066	0.443526	0.818965	1.000
295	GOBP_LIPID_METABOLIC_PROCESS	GO:0006629	10	0.385587	1.015369	0.455296	0.817715	1.000
296	GOBP_COGNITION	GO:0050890	28	0.299733	1.011169	0.477189	0.824596	1.000
297	GOBP_TUBE_DEVELOPMENT	GO:0035295	18	0.331817	1.010480	0.452320	0.823419	1.000
298	GOBP_POSITIVE_REGULATION_OF_PHOSPHATIDYLINOSIT...	GO:0014068	5	0.465494	1.008740	0.460366	0.824522	1.000

Continuation of Table **S4**

	NAME	ID	SIZE	ES	NES	NOM.p.val	FDR.q.val	FWER.p.val
299	GOBP_POSITIVE_REGULATION_OF_CELLULAR_COMPONENT...	GO:0044089	9	0.392426	1.008635	0.447405	0.822000	1.000
300	GOBP_REGULATION_OF_HORMONE_LEVELS	GO:0010817	12	0.356860	1.006754	0.459060	0.823659	1.000
301	REACTOME_ONCOGENIC_MAPK_SIGNALING	R-HSA-6802957	5	0.465580	1.000835	0.472180	0.834454	1.000
302	GOBP_POSITIVE_REGULATION_OF_CELLULAR_COMPONENT...	GO:0051130	24	0.302713	1.000670	0.452830	0.832009	1.000
303	GOBP_CELLULAR_AMIDE_METABOLIC_PROCESS	GO:0043603	12	0.361094	0.993424	0.480000	0.845442	1.000
304	GOBP_RESPONSE_TO_LIGHT_STIMULUS	GO:0009416	11	0.365761	0.990598	0.471523	0.849051	1.000
305	GOBP_CENTRAL_NERVOUS_SYSTEM_DEVELOPMENT	GO:0007417	27	0.292388	0.990030	0.492665	0.847575	1.000
306	GOBP_REGULATION_OF_NEURONAL_SYNAPTIC_PLASTICITY	GO:0048168	7	0.422088	0.988802	0.491379	0.847695	1.000
307	GOBP_INTRINSIC_APOPTOTIC_SIGNALING_PATHWAY	GO:0097193	5	0.463652	0.988014	0.508501	0.846763	1.000
308	HALLMARK_KRAS_SIGNALING_UP	M5953	6	0.433197	0.987981	0.487482	0.844079	1.000
309	GOBP_POSITIVE_REGULATION_OF_DEVELOPMENTAL_PROCESS	GO:0051094	32	0.284040	0.986006	0.491985	0.845678	1.000
310	GOBP_CALCIUM_MEDIATED_SIGNALING	GO:0019722	10	0.368191	0.982802	0.491429	0.850297	1.000
311	GOBP_REGULATION_OF_CELLULAR_CATABOLIC_PROCESS	GO:0031329	10	0.374031	0.982715	0.509040	0.847756	1.000
312	GOBP_REGULATION_OF_PEPTIDYL_SERINE_PHOSPHORYLA...	GO:0033135	8	0.392795	0.979701	0.480226	0.851731	1.000
313	GOBP_REGULATION_OF_CELLULAR_COMPONENT_BIOGENESIS	GO:0044087	10	0.369462	0.978096	0.499320	0.852398	1.000
314	GOBP_ORGAN_GROWTH	GO:0035265	5	0.466755	0.972143	0.503748	0.862698	1.000
315	GOBP_REGULATION_OF_PEPTIDE_HORMONE_SECRETION	GO:0090276	6	0.428131	0.971854	0.521870	0.860666	1.000
316	GOBP_NEGATIVE_REGULATION_OF_IMMUNE_SYSTEM_PROCESS	GO:0002683	5	0.458157	0.969304	0.527048	0.863470	1.000
317	GOBP_REGULATION_OF_ORGANELLE_ORGANIZATION	GO:0033043	14	0.335545	0.969083	0.512616	0.861247	1.000
318	GOBP_REGULATION_OF_PEPTIDE_SECRETION	GO:0002791	7	0.416168	0.967985	0.520567	0.860940	1.000
319	GOBP_NEGATIVE_REGULATION_OF_ION_TRANSPORT	GO:0043271	11	0.360122	0.967908	0.527778	0.858399	1.000
320	GOBP_CELL_MORPHOGENESIS_INVOLVED_IN_DIFFERENTI...	GO:0000904	22	0.301922	0.967426	0.513806	0.856782	1.000
321	GOBP_REGULATION_OF_ORGAN_GROWTH	GO:0046620	5	0.466755	0.967171	0.530211	0.854660	1.000
322	GOBP_POSITIVE_REGULATION_OF_PEPTIDYL_SERINE_PH...	GO:0033138	8	0.392795	0.966916	0.527536	0.852618	1.000
323	GOBP_POSITIVE_REGULATION_OF_DNA_BINDING_TRANSC...	GO:0051091	10	0.373826	0.963466	0.526316	0.857296	1.000
324	GOBP_REGULATION_OF_EXTRINSIC_APOPTOTIC_SIGNALI...	GO:2001236	6	0.419498	0.960981	0.507225	0.859924	1.000
325	GOBP_POSITIVE_REGULATION_OF_LOCOMOTION	GO:0040017	11	0.364375	0.960239	0.519890	0.858813	1.000
326	GOBP_ESTABLISHMENT_OF_PROTEIN_LOCALIZATION	GO:0045184	17	0.320294	0.958866	0.507833	0.859066	1.000
327	GOBP_LONG_TERM_MEMORY	GO:0007616	6	0.431231	0.957409	0.507983	0.859564	1.000
328	GOBP_REGULATION_OF_MUSCLE_ADAPTATION	GO:0043502	5	0.441541	0.956200	0.529687	0.859448	1.000
329	GOBP_REGULATION_OF_ANION_TRANSPORT	GO:0044070	20	0.307874	0.955094	0.539702	0.859214	1.000
330	GOBP_CELLULAR_COMPONENT_MAINTENANCE	GO:0043954	5	0.451391	0.954875	0.547945	0.856979	1.000
331	GOBP_CELL_MORPHOGENESIS_INVOLVED_IN_NEURON_DIF...	GO:0048667	22	0.301922	0.954275	0.522556	0.855636	1.000
332	GOBP_NEGATIVE_REGULATION_OF_PROTEIN_MODIFICATI...	GO:0031400	10	0.359998	0.950730	0.530497	0.860453	1.000
333	GOBP_ORGANOPHOSPHATE_METABOLIC_PROCESS	GO:0019637	8	0.381223	0.949478	0.544286	0.860540	1.000
334	GOBP_CELLULAR_LIPID_METABOLIC_PROCESS	GO:0044255	7	0.401564	0.948976	0.545052	0.859116	1.000
335	GOBP_POSITIVE_REGULATION_OF_DEVELOPMENTAL_GROWTH	GO:0048639	8	0.384694	0.946960	0.545953	0.860649	1.000
336	GOBP_REGULATION_OF_MITOTIC_CELL_CYCLE	GO:0007346	5	0.448373	0.946857	0.543511	0.858305	1.000
337	GOBP_MEMORY	GO:0007613	13	0.334937	0.946478	0.537415	0.856616	1.000
338	GOBP_POSITIVE_REGULATION_OF_GROWTH	GO:0045927	9	0.374630	0.941952	0.559829	0.863466	1.000
339	GOBP_DEPHOSPHORYLATION	GO:0016311	7	0.400243	0.940883	0.561848	0.863105	1.000
340	GOBP_POSITIVE_REGULATION_OF_MOLECULAR_FUNCTION	GO:0044093	37	0.273734	0.940527	0.530374	0.861387	1.000
341	GOBP_PROTEIN_DEPHOSPHORYLATION	GO:0006470	7	0.400243	0.940294	0.542222	0.859383	1.000

Continuation of Table **S4**

	NAME	ID	SIZE	ES	NES	NOM.p.val	FDR.q.val	FWER.p.val
342	GOBP_REGULATION_OF_PROTEIN_SERINE_THREONINE_KI...	GO:0071900	12	0.335194	0.939431	0.549795	0.858598	1.000
343	GOBP_EXCITATORY_CHEMICAL_SYNAPTIC_TRANSMISSION	GO:0098976	5	0.445783	0.939274	0.547904	0.856424	1.000
344	GOBP_REGULATION_OF_LIPID_BIOSYNTHETIC_PROCESS	GO:0046890	6	0.416809	0.938819	0.540179	0.854936	1.000
345	GOBP_RESPONSE_TO_ETHANOL	GO:0045471	5	0.445783	0.936446	0.554217	0.857401	1.000
346	GOBP_REGULATION_OF_PHOSPHATIDYLINOSITOL_3_KINA...	GO:0014066	6	0.411724	0.935935	0.562874	0.855933	1.000
347	GOBP_LIPID_BIOSYNTHETIC_PROCESS	GO:0008610	6	0.416809	0.932088	0.573082	0.861176	1.000
348	GOBP_POSITIVE_REGULATION_OF_SECRETION	GO:0051047	6	0.421156	0.931636	0.574156	0.859581	1.000
349	GOBP_MUSCLE_ADAPTATION	GO:0043500	5	0.441541	0.930644	0.567407	0.859074	1.000
350	GOBP_RHYTHMIC_PROCESS	GO:0048511	15	0.314720	0.926783	0.565274	0.864203	1.000
351	GOBP_CELL_MIGRATION	GO:0016477	22	0.289693	0.920951	0.591811	0.873314	1.000
352	GOBP_NUCLEAR_TRANSPORT	GO:0051169	5	0.430060	0.919275	0.581602	0.874196	1.000
353	GOBP_BLOOD_VESSEL_MORPHOGENESIS	GO:0048514	14	0.322944	0.917078	0.593540	0.876011	1.000
354	GOBP_MULTICELLULAR_ORGANISM_REPRODUCTION	GO:0032504	12	0.332259	0.915927	0.592748	0.875862	1.000
355	GOBP_NEGATIVE_REGULATION_OF_CATABOLIC_PROCESS	GO:0009895	8	0.374748	0.914927	0.581259	0.875263	1.000
356	GOBP_PROTEIN_TETRAMERIZATION	GO:0051262	5	0.429397	0.913940	0.579186	0.874627	1.000
357	GOBP_HEAD_DEVELOPMENT	GO:0060322	23	0.282286	0.911885	0.592040	0.876371	1.000
358	GOBP_ACTIVATION_OF_MAPK_ACTIVITY	GO:0000187	6	0.405143	0.910298	0.600587	0.877049	1.000
359	GOBP_POSITIVE_REGULATION_OF_MAP_KINASE_ACTIVITY	GO:0043406	6	0.405143	0.908853	0.600592	0.877434	1.000
360	GOBP_CARBOHYDRATE_HOMEOSTASIS	GO:0033500	7	0.382980	0.907313	0.610542	0.878056	1.000
361	GOBP_MITOTIC_CELL_CYCLE	GO:0000278	10	0.337309	0.905979	0.590723	0.878189	1.000
362	GOBP_REGULATION_OF_DEVELOPMENTAL_GROWTH	GO:0048638	9	0.357592	0.905179	0.596467	0.877258	1.000
363	GOBP_INOSITOL_LIPID_MEDIATED_SIGNALING	GO:0048017	8	0.362307	0.898399	0.626100	0.887651	1.000
364	GOBP_REGULATION_OF_BODY_FLUID_LEVELS	GO:0050878	11	0.339152	0.898375	0.604054	0.885267	1.000
365	GOBP_CIRCULATORY_SYSTEM_DEVELOPMENT	GO:0072359	21	0.281467	0.897876	0.628535	0.883741	1.000
366	GOBP_REGULATION_OF_RESPONSE_TO_EXTERNAL_STIMULUS	GO:0032101	15	0.314523	0.893867	0.601333	0.888847	1.000
367	GOBP_POSITIVE_REGULATION_OF_LEUKOCYTE_CELL_CEL...	GO:1903039	5	0.413723	0.891376	0.619120	0.891125	1.000
368	GOBP_ANIMAL_ORGAN_MORPHOGENESIS	GO:0009887	12	0.313849	0.889333	0.601361	0.892675	1.000
369	GOBP_CARBOHYDRATE_DERIVATIVE_METABOLIC_PROCESS	GO:1901135	8	0.352983	0.888846	0.598601	0.891260	1.000
370	GOBP_RESPONSE_TO_ACID_CHEMICAL	GO:0001101	6	0.392319	0.887978	0.620690	0.890401	1.000
371	GOBP_NEGATIVE_REGULATION_OF_PHOSPHORUS_METABOL...	GO:0010563	9	0.344996	0.887420	0.622563	0.889000	1.000
372	GOBP_NEGATIVE_REGULATION_OF_CATALYTIC_ACTIVITY	GO:0043086	17	0.297861	0.887089	0.633075	0.887234	1.000
373	GOBP_POSITIVE_REGULATION_OF_EPITHELIAL_CELL_PR...	GO:0050679	7	0.374510	0.886607	0.608392	0.885829	1.000
374	GOBP_NEGATIVE_REGULATION_OF_PEPTIDASE_ACTIVITY	GO:0010466	6	0.388424	0.884397	0.635240	0.887510	1.000
375	GOBP_ADENYLATE_CYCLASE_INHIBITING_G_PROTEIN_CO...	GO:0007196	5	0.421687	0.883349	0.634146	0.887167	1.000
376	GOBP_LEUKOCYTE_CELL_CELL_ADHESION	GO:0007159	6	0.391994	0.882676	0.626667	0.885989	1.000
377	GOBP_POSITIVE_REGULATION_OF_CELL_CELL_ADHESION	GO:0022409	5	0.413723	0.881171	0.640553	0.886386	1.000
378	GOBP_TUBE_MORPHOGENESIS	GO:0035239	15	0.307985	0.880239	0.633638	0.885622	1.000
379	GOBP_REGULATION_OF_TRANSMEMBRANE_TRANSPORT	GO:0034762	23	0.272304	0.879034	0.648855	0.885538	1.000
380	GOBP_EMBRYONIC_MORPHOGENESIS	GO:0048598	6	0.390355	0.878912	0.631741	0.883418	1.000
381	GOBP_POSITIVE_REGULATION_OF_CELL_GROWTH	GO:0030307	6	0.392173	0.878564	0.649331	0.881682	1.000
382	GOBP_LEARNING	GO:0007612	13	0.307485	0.875688	0.629679	0.884556	1.000
383	GOBP_NEGATIVE_REGULATION_OF_CELL_DEATH	GO:0060548	22	0.276307	0.873640	0.655779	0.886143	1.000
384	GOBP_GLUCOSE_METABOLIC_PROCESS	GO:0006006	6	0.384855	0.871868	0.637462	0.886835	1.000

Continuation of Table **S4**

	NAME	ID	SIZE	ES	NES	NOM.p.val	FDR.q.val	FWER.p.val
385	GOBP_DEVELOPMENTAL_GROWTH	GO:0048589	10	0.332129	0.871127	0.630986	0.885912	1.000
386	GOBP_REGULATION_OF_PROTEIN_CATABOLIC_PROCESS	GO:0042176	7	0.365255	0.869269	0.637725	0.887061	1.000
387	GOBP_REGULATION_OF_VASCULATURE_DEVELOPMENT	GO:1901342	5	0.413688	0.867758	0.654743	0.887240	1.000
388	GOBP_MONOSACCHARIDE_METABOLIC_PROCESS	GO:0005996	6	0.384855	0.867737	0.631954	0.884991	1.000
389	GOBP_NEURON_DEATH	GO:0070997	18	0.285434	0.866991	0.625160	0.883950	1.000
390	GOBP_AGING	GO:0007568	13	0.307029	0.866010	0.630102	0.883489	1.000
391	GOBP_REGULATION_OF_SMALL_MOLECULE_METABOLIC_PR...	GO:0062012	9	0.334503	0.862417	0.653740	0.887549	1.000
392	GOBP_NERVE_DEVELOPMENT	GO:0021675	9	0.336150	0.861798	0.652531	0.886371	1.000
393	GOBP_POSITIVE_REGULATION_OF_ENDOTHELIAL_CELL_P...	GO:0001938	6	0.379955	0.858675	0.655488	0.889613	1.000
394	GOBP_AMEBOIDAL_TYPE_CELL_MIGRATION	GO:0001667	11	0.317684	0.856580	0.651260	0.890976	1.000
395	GOBP_PROTEIN_LOCALIZATION_TO_ORGANELLE	GO:0033365	8	0.343437	0.855922	0.643478	0.889816	1.000
396	GOBP_SMALL_MOLECULE_METABOLIC_PROCESS	GO:0044281	16	0.287356	0.855607	0.660053	0.888114	1.000
397	GOBP_NEGATIVE_REGULATION_OF_ANION_TRANSPORT	GO:1903792	8	0.352655	0.855252	0.656077	0.886546	1.000
398	GOBP_NEGATIVE_REGULATION_OF_PROTEIN_METABOLIC_...	GO:0051248	20	0.271517	0.854286	0.657071	0.885896	1.000
399	GOBP_REGULATION_OF_PROTEIN_LOCALIZATION	GO:0032880	18	0.282815	0.853575	0.665786	0.884927	1.000
400	GOBP_DEFENSE_RESPONSE	GO:0006952	19	0.278056	0.850251	0.638821	0.888458	1.000
401	GOBP_PHAGOCYTOSIS	GO:0006909	5	0.396903	0.848987	0.666165	0.888355	1.000
402	GOBP_REGULATION_OF_PEPTIDE_TRANSPORT	GO:0090087	10	0.323636	0.842469	0.679894	0.896812	1.000
403	GOBP_PATTERN_SPECIFICATION_PROCESS	GO:0007389	6	0.373778	0.842069	0.670695	0.895243	1.000
404	GOBP_CELLULAR_MACROMOLECULE_LOCALIZATION	GO:0070727	20	0.267745	0.838759	0.678161	0.898730	1.000
405	REACTOME_MAPK_FAMILY_SIGNALING_CASCADES	R-RNO-5683057	12	0.302557	0.835967	0.674581	0.901451	1.000
406	GOBP_SPROUTING_ANGIOGENESIS	GO:0002040	5	0.388130	0.835963	0.697819	0.899238	1.000
407	GOBP_REGULATION_OF_LIPID_LOCALIZATION	GO:1905952	5	0.392034	0.834309	0.712166	0.899959	1.000
408	GOBP_REGULATION_OF_CATABOLIC_PROCESS	GO:0009894	13	0.295239	0.832904	0.684070	0.900172	1.000
409	GOBP_REPRODUCTIVE_SYSTEM_DEVELOPMENT	GO:0061458	6	0.368967	0.832896	0.686544	0.897986	1.000
410	GOBP_STRIATED_MUSCLE_CELL_DIFFERENTIATION	GO:0051146	9	0.331742	0.832790	0.680764	0.895986	1.000
411	GOBP_REGIONALIZATION	GO:0003002	6	0.373778	0.831932	0.700880	0.895105	1.000
412	GOBP_REGULATION_OF_TRANSPORTER_ACTIVITY	GO:0032409	17	0.275722	0.827846	0.684211	0.899625	1.000
413	GOBP_RESPONSE_TO_ABIOTIC_STIMULUS	GO:0009628	27	0.247811	0.826005	0.706897	0.900459	1.000
414	GOBP_RESPONSE_TO_KETONE	GO:1901654	7	0.345982	0.824696	0.694524	0.900459	1.000
415	GOBP_REGULATION_OF_CELLULAR_PROTEIN_LOCALIZATION	GO:1903827	12	0.292699	0.823316	0.682243	0.900554	1.000
416	GOBP_APOPTOTIC_MITOCHONDRIAL_CHANGES	GO:0008637	6	0.363557	0.822225	0.695965	0.900244	1.000
417	GOBP_RESPONSE_TO_MOLECULE_OF_BACTERIAL_ORIGIN	GO:0002237	13	0.287963	0.821991	0.693243	0.898432	1.000
418	GOBP_NEGATIVE_REGULATION_OF_SYNAPTIC_TRANSMISSION	GO:0050805	6	0.359240	0.821898	0.699708	0.896429	1.000
419	GOBP_RESPONSE_TO_BACTERIUM	GO:0009617	13	0.287963	0.819541	0.703655	0.898225	1.000
420	GOBP_REGULATION_OF_PROTEIN_LOCALIZATION_TO_CEL...	GO:1904375	6	0.363287	0.817400	0.708093	0.899403	1.000
421	GOBP_RESPONSE_TO_PEPTIDE	GO:1901652	18	0.266981	0.812472	0.695652	0.905108	1.000
422	GOBP_NEUROTROPHIN_TRK_RECEPTOR_SIGNALING_PATHWAY	GO:0048011	6	0.353369	0.811177	0.713650	0.905158	1.000
423	REACTOME_NERVOUS_SYSTEM_DEVELOPMENT	R-RNO-9675108	14	0.281196	0.808416	0.725389	0.907377	1.000
424	GOBP_TRANSMEMBRANE_RECEPTOR_PROTEIN_TYROSINE_K...	GO:0007169	19	0.258632	0.805103	0.719059	0.910339	1.000
425	GOBP_REGULATION_OF_PROTEOLYSIS	GO:0030162	12	0.289615	0.802187	0.717984	0.912543	1.000
426	GOBP_CELLULAR_RESPONSE_TO_ABIOTIC_STIMULUS	GO:0071214	7	0.342142	0.796397	0.736070	0.919340	1.000
427	GOBP_CELL_CELL_ADHESION	GO:0098609	14	0.275429	0.796215	0.735294	0.917527	1.000

Continuation of Table **S4**

	NAME	ID	SIZE	ES	NES	NOM.p.val	FDR.q.val	FWER.p.val
428	GOBP_NEGATIVE_REGULATION_OF_INTRACELLULAR_SIGN...	GO:1902532	7	0.330248	0.796038	0.723781	0.915656	1.000
429	GOBP_NEUROTROPHIN_SIGNALING_PATHWAY	GO:0038179	6	0.353369	0.795836	0.728083	0.913853	1.000
430	GOBP_LOCOMOTION	GO:0040011	31	0.228546	0.794926	0.710145	0.913170	1.000
431	GOBP_NEURON_APOPTOTIC_PROCESS	GO:0051402	12	0.291318	0.793906	0.749662	0.912670	1.000
432	GOBP_MATERNAL_PROCESS_INVOLVED_IN_FEMALE_PREGN...	GO:0060135	5	0.383593	0.793102	0.759690	0.911813	1.000
433	GOBP_LONG_TERM_SYNAPTIC_DEPRESSION	GO:0060292	5	0.374218	0.792976	0.733537	0.909886	1.000
434	GOBP_ENDOTHELIAL_CELL_PROLIFERATION	GO:0001935	8	0.319925	0.792224	0.733711	0.908772	1.000
435	GOBP_VASCULATURE_DEVELOPMENT	GO:0001944	17	0.263332	0.792031	0.766927	0.906950	1.000
436	GOBP_RESPONSE_TO_NICOTINE	GO:0035094	7	0.334449	0.790981	0.739568	0.906530	1.000
437	GOBP_NEGATIVE_REGULATION_OF_LOCOMOTION	GO:0040013	5	0.366410	0.789256	0.737313	0.907033	1.000
438	REACTOME_MEMBRANE_TRAFFICKING	R-RNO-199991	5	0.369231	0.789223	0.749254	0.905025	1.000
439	REACTOME_CYTOKINE_SIGNALING_IN_IMMUNE_SYSTEM	R-RNO-1280215	21	0.249841	0.788484	0.736709	0.903947	1.000
440	GOBP_RESPONSE_TO_LIPID	GO:0033993	19	0.254464	0.787381	0.754057	0.903593	1.000
441	GOBP_FAT_CELL_DIFFERENTIATION	GO:0045444	9	0.305997	0.787179	0.745739	0.901899	1.000
442	GOBP_RESPONSE_TO_UV	GO:0009411	5	0.377657	0.786718	0.762346	0.900570	1.000
443	GOBP_PROTEIN_CATABOLIC_PROCESS	GO:0030163	8	0.317545	0.786464	0.743920	0.898962	1.000
444	GOBP_CELLULAR_RESPONSE_TO_EXTERNAL_STIMULUS	GO:0071496	10	0.298457	0.780584	0.762689	0.905507	1.000
445	GOBP_MULTI_ORGANISM_PROCESS	GO:0051704	16	0.264181	0.779939	0.748988	0.904444	1.000
446	REACTOME_VESICLE_MEDIATED_TRANSPORT	R-RNO-5653656	5	0.369231	0.774592	0.806748	0.910367	1.000
447	GOBP_NEGATIVE_REGULATION_OF_APOPTOTIC_SIGNALIN...	GO:2001234	7	0.327114	0.773537	0.752608	0.909837	1.000
448	GOBP_ERK1_AND_ERK2_CASCADE	GO:0070371	5	0.353170	0.763179	0.777778	0.922311	1.000
449	GOBP_MITOCHONDRION_ORGANIZATION	GO:0007005	7	0.321620	0.761394	0.763348	0.922786	1.000
450	GOBP_MONOAMINE_TRANSPORT	GO:0015844	5	0.354985	0.758311	0.808670	0.925015	1.000
451	GOBP_NUCLEOBASE_CONTAINING_SMALL_MOLECULE_META...	GO:0055086	7	0.316438	0.751403	0.763554	0.932782	1.000
452	GOBP_CARBOHYDRATE_METABOLIC_PROCESS	GO:0005975	9	0.295179	0.750659	0.797737	0.931750	1.000
453	GOBP_POSITIVE_REGULATION_OF_CELL_ADHESION	GO:0045785	7	0.317306	0.747919	0.787143	0.933534	1.000
454	GOBP_RESPONSE_TO_PEPTIDE_HORMONE	GO:0043434	13	0.265210	0.745956	0.824866	0.934144	1.000
455	GOBP_NEGATIVE_REGULATION_OF_GENE_EXPRESSION	GO:0010629	11	0.280512	0.745796	0.786217	0.932307	1.000
456	GOBP_ORGANONITROGEN_COMPOUND_CATABOLIC_PROCESS	GO:1901565	10	0.280737	0.743383	0.776999	0.933534	1.000
457	GOBP_POSITIVE_REGULATION_OF_GENE_EXPRESSION	GO:0010628	16	0.248324	0.743059	0.800000	0.931896	1.000
458	GOBP_RESPONSE_TO_ALCOHOL	GO:0097305	11	0.271673	0.741108	0.806849	0.932421	1.000
459	GOBP_REGULATION_OF_CELLULAR_COMPONENT_MOVEMENT	GO:0051270	15	0.254244	0.740381	0.785252	0.931366	1.000
460	GOBP_PROTEIN_LOCALIZATION_TO_CELL_JUNCTION	GO:1902414	6	0.333014	0.738210	0.815267	0.932156	1.000
461	GOBP_MUSCLE_CELL_DIFFERENTIATION	GO:0042692	10	0.278407	0.737884	0.792208	0.930573	1.000
462	GOBP_NEGATIVE_REGULATION_OF_SIGNALING	GO:0023057	21	0.235383	0.734187	0.814910	0.933425	1.000
463	GOBP_REPRODUCTION	GO:0000003	18	0.238262	0.732177	0.817594	0.934133	1.000
464	GOBP_REGULATION_OF_DNA_BINDING_TRANSCRIPTION_F...	GO:0051090	16	0.244460	0.730460	0.814474	0.934281	1.000
465	GOBP_MACROMOLECULE_CATABOLIC_PROCESS	GO:0009057	11	0.265997	0.728587	0.819293	0.934585	1.000
466	GOBP_EPITHELIAL_CELL_PROLIFERATION	GO:0050673	12	0.262624	0.720056	0.804289	0.943584	1.000
467	GOBP_MUSCLE_SYSTEM_PROCESS	GO:0003012	9	0.283464	0.719148	0.814111	0.942765	1.000
468	GOBP_ENDOTHELIAL_CELL_MIGRATION	GO:0043542	8	0.283447	0.715921	0.812921	0.944675	1.000
469	GOBP_IMPORT_INTO_CELL	GO:0098657	6	0.315328	0.713980	0.832836	0.945155	1.000
470	GOBP_REGULATION_OF_GROWTH	GO:0040008	13	0.259023	0.713096	0.819135	0.944339	1.000

Continuation of Table **S4**

	NAME	ID	SIZE	ES	NES	NOM.p.val	FDR.q.val	FWER.p.val
471	GOBP_REGULATION_OF_CYSTEINE_TYPE_ENDOPEPTIDASE...	GO:2000116	9	0.274191	0.711609	0.807580	0.944224	1.000
472	GOBP_ORGANIC_ACID_METABOLIC_PROCESS	GO:0006082	7	0.300400	0.711585	0.842466	0.942252	1.000
473	GOBP_REGULATION_OF_OSTEOCLAST_DIFFERENTIATION	GO:0045670	5	0.329609	0.710791	0.858859	0.941170	1.000
474	GOBP_POSITIVE_REGULATION_OF_PROTEOLYSIS	GO:0045862	8	0.287312	0.704387	0.835443	0.947084	1.000
475	GOBP_INNATE_IMMUNE_RESPONSE	GO:0045087	7	0.292838	0.703039	0.831625	0.946687	1.000
476	GOBP_CELL_JUNCTION_ASSEMBLY	GO:0034329	8	0.285659	0.701408	0.839650	0.946582	1.000
477	GOBP_EMBRYONIC_ORGAN_DEVELOPMENT	GO:0048568	7	0.303164	0.701267	0.832593	0.944769	1.000
478	GOBP_NEGATIVE_REGULATION_OF_CELL_PROJECTION_OR...	GO:0031345	5	0.332383	0.701176	0.835616	0.942910	1.000
479	GOBP_CELLULAR_RESPONSE_TO_OXYGEN_CONTAINING_CO...	GO:1901701	29	0.210703	0.700410	0.853629	0.941793	1.000
480	GOBP_GROWTH	GO:0040007	14	0.242780	0.697460	0.833333	0.943233	1.000
481	GOBP_BLOOD_VESSEL_ENDOTHELIAL_CELL_MIGRATION	GO:0043534	8	0.283447	0.696388	0.854930	0.942522	1.000
482	GOBP_MONOCARBOXYLIC_ACID_METABOLIC_PROCESS	GO:0032787	6	0.311794	0.695576	0.860058	0.941508	1.000
483	GOBP_TAXIS	GO:0042330	14	0.241473	0.691020	0.853977	0.944647	1.000
484	GOBP_REGULATION_OF_PROTEIN_STABILITY	GO:0031647	5	0.329342	0.690058	0.874807	0.943817	1.000
485	GOBP_CELLULAR_RESPONSE_TO_INORGANIC_SUBSTANCE	GO:0071241	11	0.257611	0.689685	0.863319	0.942304	1.000
486	GOBP_RESPONSE_TO_TUMOR_NECROSIS_FACTOR	GO:0034612	7	0.289646	0.688587	0.865278	0.941703	1.000
487	GOBP_REGULATION_OF_CELL_ACTIVATION	GO:0050865	8	0.284631	0.686618	0.849928	0.941917	1.000
488	GOBP_RESPONSE_TO_BIOTIC_STIMULUS	GO:0009607	17	0.226659	0.686172	0.864474	0.940428	1.000
489	GOBP_CELL_CELL_SIGNALING_BY_WNT	GO:0198738	7	0.292317	0.684920	0.856515	0.939891	1.000
490	GOBP_POSITIVE_REGULATION_OF_RESPONSE_TO_EXTERN...	GO:0032103	9	0.273118	0.683220	0.858543	0.939803	1.000
491	GOBP_BIOLOGICAL_ADHESION	GO:0022610	19	0.219498	0.681910	0.863520	0.939247	1.000
492	GOBP_PURINE_CONTAINING_COMPOUND_METABOLIC_PROCESS	GO:0072521	6	0.308673	0.680787	0.884181	0.938582	1.000
493	GOBP_NEURON_PROJECTION_GUIDANCE	GO:0097485	9	0.273168	0.678164	0.860955	0.939404	1.000
494	GOBP_REGULATION_OF_CYTOSKELETON_ORGANIZATION	GO:0051493	5	0.318758	0.676363	0.883614	0.939333	1.000
495	GOBP_TISSUE_MIGRATION	GO:0090130	9	0.268503	0.672779	0.871191	0.941138	1.000
496	GOBP_INFLAMMATORY_RESPONSE	GO:0006954	12	0.239345	0.666354	0.864792	0.945876	1.000
497	GOBP_POSITIVE_REGULATION_OF_ANION_TRANSPORT	GO:1903793	9	0.254085	0.656929	0.892458	0.953309	1.000
498	GOBP_NEUROINFLAMMATORY_RESPONSE	GO:0150076	5	0.310007	0.654343	0.917169	0.953902	1.000
499	GOBP_CELL_GROWTH	GO:0016049	10	0.247424	0.654113	0.883657	0.952206	1.000
500	GOBP_ORGANIC_HYDROXY_COMPOUND_METABOLIC_PROCESS	GO:1901615	8	0.262385	0.641436	0.903819	0.961834	1.000
501	GOBP_RESPONSE_TO_METAL_ION	GO:0010038	15	0.219218	0.640004	0.921260	0.961229	1.000
502	GOBP_REGULATION_OF_PEPTIDASE_ACTIVITY	GO:0052547	10	0.240015	0.629484	0.899729	0.968847	1.000
503	GOBP_NEGATIVE_REGULATION_OF_HYDROLASE_ACTIVITY	GO:0051346	11	0.230874	0.626295	0.910714	0.969618	1.000
504	GOBP_NEGATIVE_REGULATION_OF_CELL_DEVELOPMENT	GO:0010721	6	0.277700	0.625701	0.917526	0.968202	1.000
505	GOBP_DEVELOPMENTAL_PROCESS_INVOLVED_IN_REPRODU...	GO:0003006	11	0.229732	0.625439	0.907104	0.966469	1.000
506	GOBP_AMYLOID_PRECURSOR_PROTEIN_METABOLIC_PROCESS	GO:0042982	5	0.289564	0.619642	0.938897	0.969280	1.000
507	GOBP_NEGATIVE_REGULATION_OF_NERVOUS_SYSTEM_DEV...	GO:0051961	5	0.289622	0.617231	0.922256	0.969320	1.000
508	GOBP_CARDIAC_MUSCLE_TISSUE_DEVELOPMENT	GO:0048738	6	0.272845	0.611507	0.919075	0.971732	1.000
509	GOBP_ASSOCIATIVE_LEARNING	GO:0008306	7	0.262356	0.608788	0.926362	0.971866	1.000
510	GOBP_MULTI_MULTICELLULAR_ORGANISM_PROCESS	GO:0044706	8	0.246643	0.607370	0.929972	0.970993	1.000
511	GOBP_AMYLOID_PRECURSOR_PROTEIN_CATABOLIC_PROCESS	GO:0042987	5	0.289564	0.605670	0.937134	0.970265	1.000
512	GOBP_HEART_DEVELOPMENT	GO:0007507	6	0.272845	0.604764	0.935578	0.969093	1.000
513	GOBP_RESPONSE_TO_INORGANIC_SUBSTANCE	GO:0010035	19	0.195394	0.604657	0.935849	0.967278	1.000

Continuation of Table **S4**

	NAME	ID	SIZE	ES	NES	NOM.p.val	FDR.q.val	FWER.p.val
514	GOBP_AMYLOID_BETA_METABOLIC_PROCESS	GO:0050435	5	0.289564	0.604244	0.940549	0.965700	1.000
515	GOBP_NEGATIVE_REGULATION_OF_MOLECULAR_FUNCTION	GO:0044092	24	0.184592	0.603997	0.941038	0.964005	1.000
516	GOBP_REGULATION_OF_MUSCLE_SYSTEM_PROCESS	GO:0090257	8	0.243215	0.603157	0.939042	0.962822	1.000
517	GOBP_REGULATION_OF_WNT_SIGNALING_PATHWAY	GO:0030111	5	0.281555	0.601814	0.936556	0.961920	1.000
518	GOBP_RESPIRATORY_SYSTEM_DEVELOPMENT	GO:0060541	6	0.265806	0.587063	0.957353	0.970165	1.000
519	GOBP_APOPTOTIC_PROCESS	GO:0006915	35	0.169560	0.578620	0.957882	0.973673	1.000
520	REACTOME_L1CAM_INTERACTIONS	R-RNO-373760	5	0.271377	0.570881	0.960396	0.976358	1.000
521	GOBP_BIOLOGICAL_PROCESS_INVOLVED_IN_SYMBIOTIC_...	GO:004440	10	0.214721	0.560502	0.946721	0.980197	1.000
522	GOBP_POSITIVE_REGULATION_OF_ORGANELLE_ORGANIZA...	GO:0010638	10	0.216285	0.559195	0.945637	0.978978	1.000
523	GOBP_CIRCADIAN_REGULATION_OF_GENE_EXPRESSION	GO:0032922	5	0.262878	0.559061	0.973643	0.977172	1.000
524	GOBP_POSITIVE_REGULATION_OF_DEFENSE_RESPONSE	GO:0031349	5	0.260620	0.548368	0.977612	0.980835	1.000
525	GOBP_DEFENSE_RESPONSE_TO_OTHER_ORGANISM	GO:0098542	9	0.213048	0.539704	0.974755	0.982898	1.000
526	GOBP_REGULATION_OF_NEURON_DIFFERENTIATION	GO:0045664	5	0.252541	0.536454	0.977811	0.982412	1.000
527	GOBP_POSITIVE_REGULATION_OF_HYDROLASE_ACTIVITY	GO:0051345	16	0.179520	0.531422	0.972863	0.982542	1.000
528	GOBP_REGULATION_OF_HYDROLASE_ACTIVITY	GO:0051336	22	0.160603	0.516395	0.973585	0.986325	1.000
529	GOBP_NEGATIVE_REGULATION_OF_PROTEIN_CATABOLIC_...	GO:0042177	5	0.239233	0.503173	0.986804	0.988626	1.000
530	GOBP_NEGATIVE_REGULATION_OF_RESPONSE_TO_STIMULUS	GO:0048585	16	0.166973	0.500414	0.982984	0.987598	1.000
531	GOBP_RESPONSE_TO_CALCIUM_ION	GO:0051592	6	0.210584	0.475114	0.995529	0.992004	1.000
532	GOBP_PROTEOLYSIS	GO:0006508	17	0.146049	0.437320	0.996134	0.995953	1.000
533	GOBP_POSITIVE_REGULATION_OF_IMMUNE_RESPONSE	GO:0050778	9	-0.188828	-0.575975	0.976510	0.972107	1.000
534	GOBP_RESPONSE_TO_WOUNDING	GO:0009611	15	-0.159585	-0.602349	0.981482	0.963180	1.000
535	GOBP_MALE_GAMETE_GENERATION	GO:0048232	5	-0.253012	-0.604289	0.949008	0.967070	1.000
536	GOBP_CELLULAR_RESPONSE_TO_ORGANIC_CYCLIC_COMPOUND	GO:0071407	13	-0.181751	-0.645069	0.946360	0.942108	1.000
537	GOBP_CYTOKINE_PRODUCTION	GO:0001816	10	-0.215426	-0.667603	0.894118	0.925952	1.000
538	GOBP_IMMUNE_EFFECTOR_PROCESS	GO:0002252	11	-0.207177	-0.667842	0.904412	0.930700	1.000
539	GOBP_REGULATION_OF_MUSCLE_CELL_DIFFERENTIATION	GO:0051147	6	-0.267445	-0.679499	0.869318	0.923287	1.000
540	GOBP_COAGULATION	GO:0050817	8	-0.239735	-0.683345	0.856164	0.924074	1.000
541	REACTOME_RNA_POLYMERASE_II_TRANSCRIPTION	R-RNO-73857	20	-0.165242	-0.688255	0.863208	0.923626	1.000
542	REACTOME_SIGNALING_BY_RHO_GTPASES_MIRO_GTPASES...	R-RNO-9716542	9	-0.229804	-0.693671	0.855634	0.922112	1.000
543	REACTOME_RHO_GTPASE_EFFECTORS	R-RNO-195258	9	-0.229804	-0.696166	0.876712	0.923845	1.000
544	GOBP_POSITIVE_REGULATION_OF_CYTOKINE_PRODUCTION	GO:0001819	7	-0.249042	-0.701150	0.880000	0.922560	1.000
545	GOBP_REGULATION_OF_DEFENSE_RESPONSE	GO:0031347	7	-0.259259	-0.705255	0.859060	0.922667	1.000
546	GOBP_ORGANIC_HYDROXY_COMPOUND_TRANSPORT	GO:0015850	6	-0.279376	-0.707951	0.854545	0.924173	1.000
547	GOBP_REGULATION_OF_INFLAMMATORY_RESPONSE	GO:0050727	7	-0.259259	-0.711584	0.865385	0.924286	1.000
548	GOBP_TEMPERATURE_HOMEOSTASIS	GO:0001659	5	-0.305944	-0.725896	0.869048	0.909589	1.000
549	GOBP_WOUND_HEALING	GO:0042060	13	-0.213421	-0.748554	0.816794	0.880911	1.000
550	GOBP_MYELOID_LEUKOCYTE_ACTIVATION	GO:0002274	8	-0.262500	-0.767898	0.760274	0.855074	1.000
551	GOBP_CELLULAR_RESPONSE_TO_STARVATION	GO:0009267	5	-0.331225	-0.770891	0.728022	0.855034	1.000
552	GOBP_NEGATIVE_REGULATION_OF_RESPONSE_TO_EXTERN...	GO:0032102	5	-0.325301	-0.773116	0.746988	0.856111	1.000
553	GOBP_NEGATIVE_REGULATION_OF_CELL_DIFFERENTIATION	GO:0045596	13	-0.226667	-0.782602	0.784000	0.845644	1.000
554	GOBP_SMALL_MOLECULE_BIOSYNTHETIC_PROCESS	GO:0044283	5	-0.325301	-0.784218	0.748538	0.847771	1.000
555	GOBP_RESPONSE_TO_STARVATION	GO:0042594	5	-0.331225	-0.791858	0.764205	0.839568	1.000
556	GOBP_EPHRIN_RECEPTOR_SIGNALING_PATHWAY	GO:0048013	5	-0.340670	-0.811947	0.704478	0.809923	1.000

Continuation of Table **S4**

	NAME	ID	SIZE	ES	NES	NOM.p.val	FDR.q.val	FWER.p.val
557	GOBP_REGULATION_OF_GTPASE_ACTIVITY	GO:0043087	5	-0.343709	-0.820182	0.678873	0.799053	1.000
558	REACTOME_EPH_EPHRIN_SIGNALING	R-RNO-2682334	6	-0.328244	-0.821177	0.625748	0.801913	1.000
559	GOBP_MUSCLE_CELL_PROLIFERATION	GO:0033002	8	-0.284043	-0.821714	0.678689	0.805852	1.000
560	GOBP_NEGATIVE_REGULATION_OF_DEVELOPMENTAL_PROCESS	GO:0051093	13	-0.226667	-0.825767	0.714286	0.803077	1.000
561	GOBP_ACTIVATION_OF_IMMUNE_RESPONSE	GO:0002253	5	-0.337878	-0.830658	0.682584	0.798982	1.000
562	REACTOME_CELLULAR_RESPONSES_TO_EXTERNAL_STIMULI	R-RNO-8953897	11	-0.245370	-0.831103	0.695971	0.803131	1.000
563	GOBP_MUSCLE_CONTRACTION	GO:0006936	5	-0.349398	-0.833371	0.657382	0.804169	1.000
564	GOBP_PLATELET_ACTIVATION	GO:0030168	6	-0.320200	-0.844788	0.647059	0.787952	1.000
565	GOBP_MUSCLE_CELL_DEVELOPMENT	GO:0055001	5	-0.349398	-0.845846	0.636905	0.790722	1.000
566	REACTOME_NEUTROPHIL_DEGRANULATION	R-RNO-6798695	6	-0.329268	-0.849573	0.639437	0.788395	1.000
567	GOBP_MYELOID_LEUKOCYTE_MEDIATED_IMMUNITY	GO:0002444	6	-0.329268	-0.853905	0.635220	0.784900	1.000
568	GOBP_EPIDERMIS_DEVELOPMENT	GO:0008544	5	-0.354236	-0.854984	0.659824	0.788173	1.000
569	GOBP_ANATOMICAL_STRUCTURE_HOMEOSTASIS	GO:0060249	7	-0.315769	-0.856115	0.652038	0.790838	1.000
570	GOBP_MYOTUBE_DIFFERENTIATION	GO:0014902	5	-0.349398	-0.861083	0.622159	0.786448	1.000
571	GOBP_CELL_ACTIVATION_INVOLVED_IN_IMMUNE_RESPONSE	GO:0002263	6	-0.329268	-0.861579	0.613293	0.790653	1.000
572	GOBP_NEGATIVE_REGULATION_OF_MULTICELLULAR_ORGA...	GO:0051241	15	-0.232877	-0.875430	0.626556	0.769383	1.000
573	REACTOME_TOLL_LIKE_RECEPTOR_TLR1_TLR2_CASCADE	R-RNO-168179	8	-0.299598	-0.877066	0.602007	0.771186	1.000
574	GOBP_RESPONSE_TO_INTERLEUKIN_1	GO:0070555	5	-0.375340	-0.879303	0.585987	0.771820	1.000
575	GOBP_REGULATION_OF_PROTEIN_BINDING	GO:0043393	7	-0.318666	-0.879999	0.605863	0.775453	1.000
576	REACTOME_MYD88_INDEPENDENT_TLR4_CASCADE	R-RNO-166166	8	-0.299598	-0.881266	0.600000	0.778104	1.000
577	REACTOME_TOLL_LIKE_RECEPTOR_CASCADES	R-RNO-168898	8	-0.299598	-0.884229	0.585034	0.777513	1.000
578	REACTOME_TOLL_LIKE_RECEPTOR_9_TLR9_CASCADE	R-RNO-168138	8	-0.299598	-0.887034	0.617857	0.776933	1.000
579	HALLMARK_UV_RESPONSE_UP	M5941	5	-0.364515	-0.891458	0.560976	0.773364	1.000
580	GOBP_CELL_ACTIVATION	GO:0001775	22	-0.211768	-0.893785	0.663507	0.774111	1.000
581	GOBP_CELL_CELL_JUNCTION_ASSEMBLY	GO:0007043	5	-0.375193	-0.898075	0.562327	0.771290	1.000
582	GOBP_SMOOTH_MUSCLE_CELL_PROLIFERATION	GO:0048659	6	-0.349740	-0.905414	0.528701	0.761602	1.000
583	REACTOME_INFECTIOUS_DISEASE	R-HSA-5663205	12	-0.269179	-0.906936	0.617021	0.763898	1.000
584	REACTOME_INTERLEUKIN_17_SIGNALING	R-RNO-448424	6	-0.350397	-0.907289	0.563518	0.768446	1.000
585	GOBP_CELL_CELL_JUNCTION_ORGANIZATION	GO:0045216	6	-0.361652	-0.916013	0.516923	0.755710	1.000
586	GOBP_CELL_CYCLE	GO:0007049	21	-0.224069	-0.916324	0.594059	0.760482	1.000
587	GOBP_CELLULAR_GLUCOSE_HOMEOSTASIS	GO:0001678	5	-0.385542	-0.929404	0.530726	0.739660	1.000
588	REACTOME_LEISHMANIA_INFECTION	R-HSA-9658195	10	-0.288306	-0.930093	0.523298	0.743646	1.000
589	GOBP_POSITIVE_REGULATION_OF_APOPTOTIC_SIGNALIN...	GO:2001235	5	-0.385542	-0.936396	0.498507	0.736357	1.000
590	GOBP_SMALL_GTPASE_MEDIATED_SIGNAL_TRANSDUCTION	GO:0007264	8	-0.314810	-0.944682	0.481967	0.724464	1.000
591	GOBP_PROTEIN_MODIFICATION_BY_SMALL_PROTEIN_CON...	GO:0032446	6	-0.372679	-0.958612	0.505119	0.701400	1.000
592	GOBP_POSITIVE_REGULATION_OF_SMOOTH_MUSCLE_CELL...	GO:0048661	5	-0.397089	-0.960690	0.485380	0.702536	1.000
593	GOBP_POSITIVE_REGULATION_OF_ESTABLISHMENT_OF_P...	GO:1904951	5	-0.397590	-0.963172	0.457865	0.702709	1.000
594	GOBP_POSITIVE_REGULATION_OF_CELL_DEATH	GO:0010942	19	-0.237932	-0.965271	0.487562	0.703746	1.000
595	GOBP_LEUKOCYTE_MEDIATED_IMMUNITY	GO:0002443	8	-0.337500	-0.967306	0.465409	0.704833	1.000
596	REACTOME_EXTRACELLULAR_MATRIX_ORGANIZATION	R-RNO-1474244	5	-0.409639	-0.972667	0.465181	0.699370	1.000
597	GOBP_MULTICELLULAR_ORGANISMAL_HOMEOSTASIS	GO:0048871	12	-0.280097	-0.974933	0.471631	0.700343	1.000
598	REACTOME_DEVELOPMENTAL_BIOLOGY	R-RNO-1266738	22	-0.229011	-0.994125	0.464646	0.666714	1.000
599	GOBP_REGULATION_OF_CELL_DEVELOPMENT	GO:0060284	15	-0.275972	-0.996611	0.442623	0.666626	1.000

Continuation of Table **S4**

	NAME	ID	SIZE	ES	NES	NOM.p.val	FDR.q.val	FWER.p.val
600	GOBP_RESPONSE_TO_MECHANICAL_STIMULUS	GO:0009612	6	-0.370349	-1.000958	0.401961	0.663296	1.000
601	REACTOME_MAPK_TARGETS_NUCLEAR_EVENTS_MEDIATED_...	R-HSA-450282	5	-0.427073	-1.010663	0.404984	0.648941	1.000
602	GOBP_ENZYME_LINKED_RECEPTOR_PROTEIN_SIGNALING_...	GO:0007167	24	-0.239507	-1.012965	0.430622	0.649880	1.000
603	GOBP_REGULATION_OF_APOPTOTIC_SIGNALING_PATHWAY	GO:2001233	10	-0.317053	-1.018732	0.400709	0.643467	1.000
604	GOBP_PERIPHERAL_NERVOUS_SYSTEM_DEVELOPMENT	GO:0007422	9	-0.330331	-1.026445	0.401460	0.633681	1.000
605	GOBP_POSITIVE_REGULATION_OF_NEUROGENESIS	GO:0050769	12	-0.306182	-1.031645	0.377863	0.628889	1.000
606	GOBP_POSITIVE_REGULATION_OF_MYELOID_LEUKOCYTE_...	GO:0002763	6	-0.400289	-1.033368	0.379009	0.630700	1.000
607	GOBP_REGULATION_OF_NEUROGENESIS	GO:0050767	14	-0.280566	-1.035793	0.404959	0.631054	1.000
608	GOBP_POSITIVE_REGULATION_OF_CELL_DEVELOPMENT	GO:0010720	12	-0.306182	-1.040562	0.374016	0.626372	1.000
609	GOBP_OSTEOCLAST_DIFFERENTIATION	GO:0030316	6	-0.398685	-1.041748	0.368580	0.629273	1.000
610	GOBP_REGULATION_OF_CELL_DEATH	GO:0010941	34	-0.221414	-1.044157	0.424051	0.629500	1.000
611	GOBP_POSITIVE_REGULATION_OF_AXONOGENESIS	GO:0050772	5	-0.432755	-1.044946	0.367953	0.633429	1.000
612	REACTOME_POST_TRANSLATIONAL_PROTEIN_MODIFICATION	R-RNO-597592	7	-0.387151	-1.045708	0.380503	0.637423	1.000
613	GOBP_POSITIVE_REGULATION_OF_NERVOUS_SYSTEM_DEV...	GO:0051962	12	-0.306182	-1.055396	0.384328	0.622745	1.000
614	GOBP_RAS_PROTEIN_SIGNAL_TRANSDUCTION	GO:0007265	5	-0.439391	-1.055553	0.329412	0.628025	1.000
615	REACTOME_DEATH_RECEPTOR_SIGNALLING	R-RNO-73887	5	-0.433693	-1.058698	0.345238	0.627034	1.000
616	GOBP_POSITIVE_REGULATION_OF_PEPTIDASE_ACTIVITY	GO:0010952	7	-0.395062	-1.061516	0.338870	0.627181	1.000
617	GOBP_CYTOSKELETON_ORGANIZATION	GO:0007010	11	-0.316087	-1.068483	0.338130	0.619281	1.000
618	GOBP_RESPONSE_TO_NITROGEN_COMPOUND	GO:1901698	35	-0.226016	-1.068676	0.366120	0.624685	1.000
619	GOBP_POSITIVE_REGULATION_OF_CYSTEINE_TYPE_ENDO...	GO:2001056	7	-0.395062	-1.072599	0.356209	0.622193	1.000
620	GOBP_CHROMOSOME_ORGANIZATION	GO:0051276	6	-0.414173	-1.072910	0.346386	0.627431	1.000
621	GOBP_LYMPHOCYTE_ACTIVATION	GO:0046649	10	-0.344968	-1.073802	0.353175	0.631459	1.000
622	GOBP_REGULATION_OF_IMMUNE_SYSTEM_PROCESS	GO:0002682	21	-0.265290	-1.074172	0.363636	0.636818	1.000
623	GOBP_MORPHOGENESIS_OF_AN_EPITHELIUM	GO:0002009	6	-0.400087	-1.075064	0.333333	0.641270	1.000
624	REACTOME_ESR_MEDIATED_SIGNALING	R-RNO-8939211	8	-0.378285	-1.077997	0.348592	0.641440	1.000
625	REACTOME_INNATE_IMMUNE_SYSTEM	R-RNO-168249	16	-0.293349	-1.086835	0.320175	0.628400	1.000
626	GOBP_MEMBRANE_PROTEIN_PROTEOLYSIS	GO:0033619	6	-0.415698	-1.094225	0.355311	0.619253	1.000
627	GOBP_CELLULAR_RESPONSE_TO_CADMIUM_ION	GO:0071276	5	-0.446324	-1.094698	0.312866	0.624410	1.000
628	GOBP_REGULATION_OF_GLIOGENESIS	GO:0014013	5	-0.466353	-1.106269	0.300000	0.606594	1.000
629	REACTOME_SIGNALING_BY_NUCLEAR_RECEPTORS	R-RNO-9006931	8	-0.378285	-1.107596	0.279863	0.610250	1.000
630	GOBP_EXTRINSIC_APOPTOTIC_SIGNALING_PATHWAY	GO:0097191	7	-0.400746	-1.121780	0.296667	0.587415	1.000
631	GOBP_ANATOMICAL_STRUCTURE_FORMATION_INVOLVED_I...	GO:0048646	20	-0.278690	-1.122635	0.290640	0.591693	1.000
632	GOBP_RESPONSE_TO_RADIATION	GO:0009314	13	-0.319042	-1.124602	0.280156	0.594011	1.000
633	GOBP_POSITIVE_REGULATION_OF_GLIOGENESIS	GO:0014015	5	-0.466353	-1.128993	0.277008	0.591486	1.000
634	GOBP_FC_EPSILON_RECEPTOR_SIGNALING_PATHWAY	GO:0038095	7	-0.413386	-1.131829	0.280000	0.592015	1.000
635	REACTOME_PTEN_REGULATION	R-RNO-6807070	5	-0.479616	-1.131897	0.263768	0.598427	1.000
636	GOBP_RESPONSE_TO_GROWTH_FACTOR	GO:0070848	18	-0.289606	-1.136306	0.232877	0.595350	1.000
637	GOBP_POSITIVE_REGULATION_OF_IMMUNE_SYSTEM_PROCESS	GO:0002684	18	-0.291698	-1.137879	0.280374	0.598674	1.000
638	GOBP_RESPONSE_TO_CADMIUM_ION	GO:0046686	6	-0.441754	-1.139080	0.262500	0.602763	1.000
639	REACTOME_TRANSCRIPTIONAL_REGULATION_BY_TP53	R-RNO-3700989	6	-0.439264	-1.142261	0.264151	0.603174	1.000
640	REACTOME_FC_EPSILON_RECEPTOR_FCERI_SIGNALING	R-RNO-2454202	7	-0.413386	-1.142427	0.267516	0.609959	1.000
641	GOBP_POSITIVE_REGULATION_OF_BINDING	GO:0051099	6	-0.451219	-1.147463	0.266862	0.607015	1.000
642	GOBP_OSSIFICATION	GO:0001503	9	-0.366621	-1.148862	0.282609	0.611302	1.000

Continuation of Table **S4**

	NAME	ID	SIZE	ES	NES	NOM.p.val	FDR.q.val	FWER.p.val
643	GOBP_CYTOKINE_MEDIATED_SIGNALING_PATHWAY	GO:0019221	16	-0.303124	-1.153110	0.230047	0.609363	1.000
644	GOBP_ACTIN_FILAMENT_BASED_PROCESS	GO:0030029	7	-0.421261	-1.168487	0.224684	0.583591	1.000
645	GOBP_CELLULAR_COMPONENT_DISASSEMBLY	GO:0022411	6	-0.455885	-1.169941	0.241477	0.587911	1.000
646	GOBP_CELLULAR_RESPONSE_TO_REACTIVE_OXYGEN_SPECIES	GO:0034614	7	-0.417888	-1.174072	0.237342	0.585748	1.000
647	GOBP_RESPONSE_TO_EXTRACELLULAR_STIMULUS	GO:0009991	13	-0.339753	-1.177354	0.269373	0.586415	1.000
648	GOBP_RESPONSE_TO_INSULIN	GO:0032868	5	-0.488647	-1.179358	0.246106	0.589561	1.000
649	GOBP_TISSUE_HOMEOSTASIS	GO:0001894	5	-0.497384	-1.179904	0.240223	0.596054	1.000
650	GOBP_CHROMATIN_ORGANIZATION	GO:0006325	5	-0.493709	-1.183317	0.244957	0.596025	1.000
651	GOBP_TISSUE_MORPHOGENESIS	GO:0048729	7	-0.436807	-1.187540	0.245734	0.594620	1.000
652	GOBP_CELLULAR_RESPONSE_TO_CHEMICAL_STRESS	GO:0062197	7	-0.417888	-1.189496	0.256944	0.598258	1.000
653	GOBP_REGULATION_OF_MULTICELLULAR_ORGANISMAL_DE...	GO:2000026	31	-0.255009	-1.198794	0.236559	0.585902	1.000
654	GOBP_POSITIVE_REGULATION_OF_CELL_POPULATION_PR...	GO:0008284	21	-0.287913	-1.199113	0.206731	0.593254	1.000
655	GOBP_REGULATION_OF_MYELOID_LEUKOCYTE_DIFFERENT...	GO:0002761	8	-0.412065	-1.204109	0.241830	0.590916	1.000
656	REACTOME_SIGNALING_BY_INTERLEUKINS	R-RNO-449147	15	-0.322242	-1.209148	0.181818	0.587908	1.000
657	GOBP_REGULATION_OF_CELL_DIFFERENTIATION	GO:0045595	36	-0.249838	-1.216149	0.140449	0.581392	1.000
658	GOBP_POSITIVE_REGULATION_OF_MYELOID_CELL_DIFFE...	GO:0045639	7	-0.436005	-1.230284	0.190635	0.559466	1.000
659	GOBP_POSITIVE_REGULATION_OF_MULTICELLULAR_ORGA...	GO:0051240	32	-0.271307	-1.248364	0.175325	0.530099	1.000
660	HALLMARK_EPITHELIAL_MESENCHYMAL_TRANSITION	M5930	5	-0.536923	-1.250161	0.236760	0.534576	1.000
661	GOBP_POSITIVE_REGULATION_OF_HEMOPOIESIS	GO:1903708	7	-0.458349	-1.264510	0.175258	0.512463	1.000
662	GOBP_REGULATION_OF_IMMUNE_RESPONSE	GO:0050776	12	-0.369298	-1.266751	0.170455	0.516138	1.000
663	GOBP_PROTEIN_MODIFICATION_BY_SMALL_PROTEIN_CON...	GO:0070647	7	-0.475190	-1.279417	0.185668	0.500196	1.000
664	GOBP_CELLULAR_RESPONSE_TO_EXTRACELLULAR_STIMULUS	GO:0031668	8	-0.437326	-1.280897	0.165644	0.505768	1.000
665	GOBP_RESPONSE_TO_OXYGEN_CONTAINING_COMPOUND	GO:1901700	39	-0.270379	-1.285677	0.099338	0.504316	1.000
666	GOBP_T_CELL_ACTIVATION	GO:0042110	8	-0.439503	-1.286534	0.182131	0.510840	1.000
667	GOBP_RESPONSE_TO_ORGANIC_CYCLIC_COMPOUND	GO:0014070	20	-0.318332	-1.296448	0.113122	0.498692	1.000
668	GOBP_REGULATION_OF_MYELOID_CELL_DIFFERENTIATION	GO:0045637	9	-0.405586	-1.298127	0.157718	0.503615	1.000
669	GOBP_REGULATION_OF_VASCULAR_ASSOCIATED_SMOOTH_...	GO:1904705	5	-0.549071	-1.306645	0.175385	0.496260	1.000
670	GOBP_MYELOID_LEUKOCYTE_DIFFERENTIATION	GO:0002573	11	-0.399629	-1.328862	0.131579	0.462058	1.000
671	GOBP_RESPONSE_TO_HORMONE	GO:0009725	20	-0.324517	-1.331026	0.111111	0.466799	1.000
672	GOBP_RESPONSE_TO_CYTOKINE	GO:0034097	23	-0.308332	-1.347236	0.091398	0.444507	1.000
673	GOBP_REGULATION_OF_BINDING	GO:0051098	11	-0.413449	-1.350367	0.128405	0.447349	1.000
674	GOBP_TRANSMEMBRANE_RECEPTOR_PROTEIN_SERINE_THR...	GO:0007178	6	-0.516218	-1.352945	0.109325	0.451276	1.000
675	GOBP_MUSCLE_STRUCTURE_DEVELOPMENT	GO:0061061	16	-0.361520	-1.363159	0.105932	0.441367	1.000
676	GOBP_NEGATIVE_REGULATION_OF_BIOSYNTHETIC_PROCESS	GO:0009890	13	-0.394169	-1.382312	0.072340	0.416233	1.000
677	GOBP_NEGATIVE_REGULATION_OF_BINDING	GO:0051100	6	-0.544758	-1.383101	0.111111	0.423661	1.000
678	GOBP_RESPONSE_TO_OXIDATIVE_STRESS	GO:0006979	8	-0.489727	-1.386331	0.126582	0.427093	1.000
679	GOBP_POSITIVE_REGULATION_OF_CELL_DIFFERENTIATION	GO:0045597	27	-0.315492	-1.389635	0.070270	0.430220	1.000
680	GOBP_NEGATIVE_REGULATION_OF_NUCLEOBASE_CONTAIN...	GO:0045934	13	-0.392884	-1.398318	0.083333	0.423330	1.000
681	REACTOME_SIGNALING_BY_RECEPTOR_TYROSINE_KINASES	R-RNO-9006934	26	-0.320548	-1.405331	0.057292	0.420120	1.000
682	GOBP_CELL_POPULATION_PROLIFERATION	GO:0008283	32	-0.296713	-1.405551	0.052632	0.429693	1.000
683	GOBP_REGULATION_OF_LEUKOCYTE_DIFFERENTIATION	GO:1902105	9	-0.465653	-1.435693	0.058419	0.385548	1.000
684	GOBP_RESPONSE_TO_REACTIVE_OXYGEN_SPECIES	GO:0000302	8	-0.489727	-1.443211	0.078550	0.382528	1.000
685	REACTOME_TRANSCRIPTIONAL_REGULATION_OF_WHITE_A...	R-RNO-381340	6	-0.559593	-1.447679	0.087379	0.384890	1.000

Continuation of Table **S4**

	NAME	ID	SIZE	ES	NES	NOM.p.val	FDR.q.val	FWER.p.val
686	GOBP_RESPONSE_TO_DRUG	GO:0042493	11	-0.441015	-1.449566	0.090909	0.391519	1.000
687	REACTOME_CELLULAR_SENESCENCE	R-RNO-2559583	6	-0.569835	-1.453079	0.092879	0.395006	1.000
688	REACTOME_SIGNALING_BY_NTRKS	R-RNO-166520	18	-0.375880	-1.453213	0.090129	0.405507	1.000
689	REACTOME_SENESCENCE_ASSOCIATED_SECRETORY_PHENO...	R-RNO-2559582	6	-0.569835	-1.457702	0.098765	0.408737	1.000
690	GOBP_IMMUNE_RESPONSE_REGULATING_SIGNALING_PATHWAY	GO:0002764	8	-0.514187	-1.458344	0.100000	0.419044	1.000
691	GOBP_REGULATION_OF_CELL_CYCLE	GO:0051726	13	-0.410605	-1.475922	0.058577	0.399975	1.000
692	GOBP_RESPONSE_TO_MONOSACCHARIDE	GO:0034284	5	-0.620622	-1.485674	0.073016	0.394405	1.000
693	GOBP_INTRACELLULAR_RECEPTOR_SIGNALING_PATHWAY	GO:0030522	5	-0.621166	-1.493768	0.066852	0.392506	1.000
694	GOBP_FC_RECEPTOR_SIGNALING_PATHWAY	GO:0038093	8	-0.514187	-1.493961	0.025078	0.404713	1.000
695	GOBP_MUSCLE_ORGAN_DEVELOPMENT	GO:0007517	9	-0.489522	-1.494685	0.073801	0.417114	1.000
696	GOBP_RESPONSE_TO_CARBOHYDRATE	GO:0009743	5	-0.620622	-1.495151	0.058140	0.430526	1.000
697	GOBP_REGULATION_OF_HEMOPOIESIS	GO:1903706	10	-0.459472	-1.500683	0.069536	0.435305	1.000
698	GOBP_MUSCLE_TISSUE_DEVELOPMENT	GO:0060537	11	-0.462851	-1.504660	0.040293	0.443553	1.000
699	REACTOME_INTERLEUKIN_4_AND_INTERLEUKIN_13_SIGN...	R-RNO-6785807	8	-0.522996	-1.522088	0.055172	0.425226	1.000
700	GOBP_RESPONSE_TO_NUTRIENT	GO:0007584	5	-0.631836	-1.545407	0.074850	0.396988	1.000
701	GOBP_APOPTOTIC_SIGNALING_PATHWAY	GO:0097190	15	-0.422598	-1.571189	0.035433	0.369499	1.000
702	GOBP_MYELOID_CELL_DIFFERENTIATION	GO:0030099	13	-0.436857	-1.576361	0.037190	0.376092	1.000
703	GOBP_DEVELOPMENTAL_CELL_GROWTH	GO:0048588	5	-0.663419	-1.590167	0.062130	0.369014	1.000
704	GOBP_NEGATIVE_REGULATION_OF_CELL_CYCLE	GO:0045786	5	-0.676551	-1.602648	0.050595	0.365528	1.000
705	GOBP_SKELETAL_MUSCLE_ORGAN_DEVELOPMENT	GO:0060538	5	-0.674408	-1.640210	0.043732	0.321373	0.999
706	GOBP_RESPONSE_TO_ENDOGENOUS_STIMULUS	GO:0009719	34	-0.365070	-1.694751	0.005650	0.259520	0.996
707	GOBP_PEPTIDYL_LYSINE_MODIFICATION	GO:0018205	5	-0.711289	-1.709858	0.022152	0.255432	0.994
708	GOBP_SKIN_DEVELOPMENT	GO:0043588	5	-0.734940	-1.711968	0.040816	0.267747	0.993
709	GOBP_POSITIVE_REGULATION_OF_NEURON_DEATH	GO:1901216	5	-0.733208	-1.735205	0.019417	0.250635	0.985
710	HALLMARK_HYPOXIA	M5891	5	-0.748829	-1.780366	0.012158	0.209527	0.967
711	GOBP_NEGATIVE_REGULATION_OF_CELL_POPULATION_PR...	GO:0008285	9	-0.589598	-1.819714	0.009836	0.178189	0.937
712	REACTOME_NUCLEAR_EVENTS_KINASE_AND_TRANSCRIPTI...	R-RNO-198725	11	-0.558108	-1.851278	0.006873	0.157446	0.896
713	GOBP_NEGATIVE_REGULATION_OF_TRANSCRIPTION_BY_R...	GO:0000122	10	-0.556441	-1.857109	0.007407	0.164463	0.893
714	GOBP_LEUKOCYTE_DIFFERENTIATION	GO:0002521	15	-0.512902	-1.880018	0.004310	0.154865	0.848
715	GOBP_POSITIVE_REGULATION_OF_NUCLEOBASE_CONTAIN...	GO:0045935	28	-0.432931	-1.977779	0.000000	0.082897	0.595
716	REACTOME_NGF_STIMULATED_TRANSCRIPTION	R-RNO-9031628	9	-0.661777	-2.011560	0.014235	0.069736	0.493
717	GOBP_IMMUNE_SYSTEM_DEVELOPMENT	GO:0002520	17	-0.504553	-2.013322	0.000000	0.077424	0.489
718	HALLMARK_APOPTOSIS	M5902	6	-0.792848	-2.064126	0.003185	0.057833	0.346
719	GOBP_POSITIVE_REGULATION_OF_BIOSYNTHETIC_PROCESS	GO:0009891	27	-0.472440	-2.094931	0.000000	0.052790	0.282
720	GOBP_MONONUCLEAR_CELL_DIFFERENTIATION	GO:1903131	7	-0.801532	-2.108012	0.003155	0.057605	0.261
721	GOBP_POSITIVE_REGULATION_OF_TRANSCRIPTION_BY_R...	GO:0045944	24	-0.516244	-2.263185	0.000000	0.013703	0.056
722	GOBP_POSITIVE_REGULATION_OF_PRI_MIRNA_TRANSCRI...	GO:1902895	7	-0.805455	-2.266591	0.000000	0.017004	0.052
723	GOBP_PRI_MIRNA_TRANSCRIPTION_BY_RNA_POLYMERASE_II	GO:0061614	7	-0.805455	-2.273288	0.000000	0.022732	0.046
724	HALLMARK_TNFA_SIGNALING_VIA_NFKB	M5890	14	-0.760351	-2.751684	0.000000	0.000000	0.000

End of Table

Supplementary Table S5: SC caudal segment early (1 DPL peak) profile GSEA results.

Begin of Table **S5**

	NAME	ID	SIZE	ES	NES	NOM.p.val	FDR.q.val	FWER.p.val
0	REACTOME_INTERLEUKIN_4_AND_INTERLEUKIN_13_SIGN...	R-RNO-6785807	8	0.900000	2.823968	0.000000	0.000000	0.000
1	REACTOME_SIGNALING_BY_INTERLEUKINS	R-RNO-449147	15	0.571289	2.286192	0.003788	0.090280	0.118
2	GOBP_CYTOKINE_MEDIATED_SIGNALING_PATHWAY	GO:0019221	16	0.555290	2.273639	0.000000	0.066719	0.129
3	GOBP_NEGATIVE_REGULATION_OF_PEPTIDASE_ACTIVITY	GO:0010466	6	0.816037	2.221286	0.000000	0.073200	0.185
4	REACTOME_TRANSCRIPTIONAL_REGULATION_OF_WHITE_A...	R-RNO-381340	6	0.764970	2.095562	0.006211	0.152611	0.419
5	GOBP_CELLULAR_RESPONSE_TO_EXTERNAL_STIMULUS	GO:0071496	10	0.593990	2.048613	0.000000	0.177395	0.537
6	GOBP_NEGATIVE_REGULATION_OF_CELL_POPULATION_PR...	GO:0008285	9	0.620253	2.026670	0.003247	0.176840	0.595
7	GOBP_ACTIN_FILAMENT_BASED_PROCESS	GO:0030029	7	0.675066	1.991510	0.005900	0.193944	0.679
8	GOBP_NEGATIVE_REGULATION_OF_CYSTEINE_TYPE_ENDO...	GO:2000117	5	0.789750	1.990752	0.005362	0.173334	0.682
9	GOBP_NEGATIVE_REGULATION_OF_LOCOMOTION	GO:0040013	5	0.723951	1.874701	0.008197	0.298478	0.877
10	REACTOME_DEATH_RECEPTOR_SIGNALLING	R-RNO-73887	5	0.739534	1.873302	0.005479	0.274196	0.880
11	GOBP_NEGATIVE_REGULATION_OF_PROTEOLYSIS	GO:0045861	8	0.612182	1.856347	0.006579	0.276496	0.906
12	GOBP_RESPONSE_TO_UV	GO:0009411	5	0.727498	1.797517	0.022989	0.348180	0.961
13	GOBP_MYELOID_CELL_DIFFERENTIATION	GO:0030099	13	0.475357	1.794794	0.015385	0.328435	0.964
14	GOBP_RESPONSE_TO_CYTOKINE	GO:0034097	23	0.392093	1.771658	0.008230	0.344624	0.978
15	GOBP_RESPONSE_TO_REACTIVE_OXYGEN_SPECIES	GO:0000302	8	0.550213	1.762561	0.018519	0.340836	0.982
16	GOBP_RESPONSE_TO_OXIDATIVE_STRESS	GO:0006979	8	0.550213	1.754301	0.018750	0.335511	0.984
17	REACTOME_CYTOKINE_SIGNALING_IN_IMMUNE_SYSTEM	R-RNO-1280215	21	0.391720	1.739955	0.008403	0.341276	0.991
18	GOBP_REGULATION_OF_APOPTOTIC_SIGNALING_PATHWAY	GO:2001233	10	0.500000	1.708110	0.019355	0.381782	0.996
19	GOBP_SKIN_DEVELOPMENT	GO:0043588	5	0.686747	1.705049	0.025352	0.367950	0.997
20	HALLMARK_ALLOGRAFT_REJECTION	M5950	7	0.566357	1.680803	0.030960	0.396453	0.999
21	GOBP_INTRINSIC_APOPTOTIC_SIGNALING_PATHWAY	GO:0097193	5	0.674699	1.648582	0.033846	0.445877	0.999
22	GOBP_APOPTOTIC_SIGNALING_PATHWAY	GO:0097190	15	0.403995	1.641462	0.028455	0.441697	0.999
23	GOBP_CELLULAR_RESPONSE_TO_EXTRACELLULAR_STIMULUS	GO:0031668	8	0.526805	1.628601	0.035714	0.450763	0.999
24	GOBP_OSSIFICATION	GO:0001503	9	0.482868	1.605176	0.047170	0.484874	0.999
25	GOBP_RESPONSE_TO_EXTRACELLULAR_STIMULUS	GO:0009991	13	0.418596	1.598865	0.036232	0.480784	0.999
26	HALLMARK_TNFA_SIGNALING_VIA_NFKB	M5890	14	0.413644	1.595161	0.029091	0.471631	0.999
27	GOBP_POSITIVE_REGULATION_OF_PRI_MIRNA_TRANSCRI...	GO:1902895	7	0.530135	1.593852	0.043605	0.456894	0.999
28	GOBP_MYELOID_LEUKOCYTE_DIFFERENTIATION	GO:0002573	11	0.435931	1.570542	0.058824	0.493213	1.000
29	GOBP_RESPONSE_TO_NUTRIENT	GO:0007584	5	0.612976	1.557176	0.060694	0.507076	1.000
30	GOBP_NEGATIVE_REGULATION_OF_MOLECULAR_FUNCTION	GO:0044092	24	0.343750	1.554876	0.015625	0.496320	1.000
31	GOBP_PRI_MIRNA_TRANSCRIPTION_BY_RNA_POLYMERASE_II	GO:0061614	7	0.530135	1.546847	0.061350	0.498314	1.000
32	GOBP_MULTI_MULTICELLULAR_ORGANISM_PROCESS	GO:0044706	8	0.487356	1.534747	0.055556	0.512907	1.000
33	GOBP_IMMUNE_SYSTEM_DEVELOPMENT	GO:0002520	17	0.355993	1.527235	0.055118	0.515177	1.000
34	GOBP_POSITIVE_REGULATION_OF_APOPTOTIC_SIGNALIN...	GO:2001235	5	0.602410	1.505491	0.058011	0.551057	1.000
35	GOBP_EMBRYONIC_ORGAN_DEVELOPMENT	GO:0048568	7	0.504034	1.501317	0.063063	0.545638	1.000
36	GOBP_NEGATIVE_REGULATION_OF_TRANSMEMBRANE_TRAN...	GO:0034763	6	0.536585	1.498759	0.076923	0.536515	1.000
37	GOBP_MORPHOGENESIS_OF_AN_EPITHELIUM	GO:0002009	6	0.530992	1.492660	0.062500	0.536384	1.000
38	GOBP_POSITIVE_REGULATION_OF_SMOOTH_MUSCLE_CELL...	GO:0048661	5	0.592984	1.488849	0.083102	0.531884	1.000
39	GOBP_OSTEOCLAST_DIFFERENTIATION	GO:0030316	6	0.549064	1.484741	0.071207	0.529290	1.000
40	GOBP_INFLAMMATORY_RESPONSE	GO:0006954	12	0.411237	1.479026	0.061151	0.530103	1.000

Continuation of Table **S5**

	NAME	ID	SIZE	ES	NES	NOM.p.val	FDR.q.val	FWER.p.val
41	GOBP_NEGATIVE_REGULATION_OF_HYDROLASE_ACTIVITY	GO:0051346	11	0.409301	1.471043	0.047273	0.534891	1.000
42	GOBP_CYTOSKELETON_ORGANIZATION	GO:0007010	11	0.409713	1.466986	0.056106	0.532329	1.000
43	GOBP_NEGATIVE_REGULATION_OF_APOPTOTIC_SIGNALIN...	GO:2001234	7	0.505028	1.461049	0.087097	0.534633	1.000
44	GOBP_OSTEOBLAST_DIFFERENTIATION	GO:0001649	5	0.567340	1.452899	0.090361	0.541441	1.000
45	GOBP_RESPONSE_TO_CADMIUM_ION	GO:0046686	6	0.523728	1.451341	0.083601	0.534130	1.000
46	GOBP_TISSUE_MORPHOGENESIS	GO:0048729	7	0.495947	1.449420	0.097701	0.526783	1.000
47	HALLMARK_HYPOXIA	M5891	5	0.560713	1.430652	0.096210	0.560242	1.000
48	GOBP_CELLULAR_RESPONSE_TO_CHEMICAL_STRESS	GO:0062197	7	0.480599	1.424055	0.088889	0.566466	1.000
49	HALLMARK_APOPTOSIS	M5902	6	0.516166	1.407320	0.078947	0.595930	1.000
50	GOBP_NEGATIVE_REGULATION_OF_PHOSPHORUS_METABOL...	GO:0010563	9	0.417722	1.405140	0.114187	0.590142	1.000
51	GOBP_CELLULAR_RESPONSE_TO_REACTIVE_OXYGEN_SPECIES	GO:0034614	7	0.480599	1.403380	0.113043	0.583102	1.000
52	GOBP_EXTRINSIC_APOPTOTIC_SIGNALING_PATHWAY	GO:0097191	7	0.481481	1.400552	0.103152	0.579550	1.000
53	GOBP_POSITIVE_REGULATION_OF_AXONOGENESIS	GO:0050772	5	0.566265	1.399012	0.128527	0.572659	1.000
54	GOBP_REGULATION_OF_PEPTIDASE_ACTIVITY	GO:0052547	10	0.410726	1.391193	0.104377	0.582379	1.000
55	GOBP_REGULATION_OF_LIPASE_ACTIVITY	GO:0060191	6	0.500000	1.389767	0.109145	0.575741	1.000
56	GOBP_CELLULAR_RESPONSE_TO_CADMIUM_ION	GO:0071276	5	0.545887	1.387220	0.123711	0.570731	1.000
57	GOBP_SMOOTH_MUSCLE_CELL_PROLIFERATION	GO:0048659	6	0.491024	1.378732	0.112903	0.581253	1.000
58	GOBP_NEUROTROPHIN_SIGNALING_PATHWAY	GO:0038179	6	0.500000	1.369896	0.097561	0.592883	1.000
59	GOBP_NEUROTROPHIN_TRK_RECEPTOR_SIGNALING_PATHWAY	GO:0048011	6	0.500000	1.367410	0.114114	0.588689	1.000
60	GOBP_NEGATIVE_REGULATION_OF_PROTEIN_METABOLIC_...	GO:0051248	20	0.299452	1.356166	0.111111	0.610093	1.000
61	GOBP_REGULATION_OF_GTPASE_ACTIVITY	GO:0043087	5	0.530120	1.355659	0.109290	0.601652	1.000
62	GOBP_LEUKOCYTE_DIFFERENTIATION	GO:0002521	15	0.344040	1.354841	0.100000	0.594512	1.000
63	GOBP_MUSCLE_CELL_PROLIFERATION	GO:0033002	8	0.448356	1.349043	0.129412	0.598568	1.000
64	GOBP_REGULATION_OF_PROTEIN_BINDING	GO:0043393	7	0.453202	1.345939	0.123123	0.597514	1.000
65	GOBP_NEGATIVE_REGULATION_OF_TRANSCRIPTION_BY_R...	GO:0000122	10	0.392040	1.340041	0.128028	0.601577	1.000
66	GOBP_REGULATION_OF_PHOSPHOLIPASE_ACTIVITY	GO:0010517	6	0.500000	1.330905	0.162500	0.614208	1.000
67	GOBP_MEMBRANE_PROTEIN_PROTEOLYSIS	GO:0033619	6	0.471322	1.325873	0.128743	0.617713	1.000
68	GOBP_REGULATION_OF_EXTRINSIC_APOPTOTIC_SIGNALI...	GO:2001236	6	0.475610	1.325399	0.142424	0.610106	1.000
69	GOBP_MONOSACCHARIDE_METABOLIC_PROCESS	GO:0005996	6	0.475610	1.317874	0.137026	0.620150	1.000
70	GOBP_PROTEOLYSIS	GO:0006508	17	0.318077	1.316853	0.127273	0.613628	1.000
71	GOBP_POSITIVE_REGULATION_OF_PEPTIDYL_SERINE_PH...	GO:0033138	8	0.425000	1.316799	0.136519	0.605202	1.000
72	GOBP_REGULATION_OF_PEPTIDYL_SERINE_PHOSPHORYLA...	GO:0033135	8	0.425000	1.313251	0.155172	0.605491	1.000
73	GOBP_GLUCOSE_METABOLIC_PROCESS	GO:0006006	6	0.475610	1.306552	0.144809	0.613936	1.000
74	GOBP_NEGATIVE_REGULATION_OF_CATALYTIC_ACTIVITY	GO:0043086	17	0.317780	1.301923	0.113281	0.616830	1.000
75	GOBP_REGULATION_OF_CELL_SIZE	GO:0008361	5	0.502893	1.300239	0.158470	0.613308	1.000
76	GOBP_CARBOHYDRATE_METABOLIC_PROCESS	GO:0005975	9	0.392405	1.292990	0.166667	0.625012	1.000
77	GOBP_PROTEIN_KINASE_B_SIGNALING	GO:0043491	6	0.475610	1.292642	0.173780	0.617938	1.000
78	GOBP_REGULATION_OF_BINDING	GO:0051098	11	0.356158	1.285623	0.134100	0.626709	1.000
79	GOBP_POSITIVE_REGULATION_OF_CELL_DIFFERENTIATION	GO:0045597	27	0.261939	1.278710	0.147982	0.634482	1.000
80	GOBP_REGULATION_OF_NEURON_DIFFERENTIATION	GO:0045664	5	0.493976	1.272196	0.171254	0.642338	1.000
81	GOBP_RESPONSE_TO_MECHANICAL_STIMULUS	GO:0009612	6	0.460386	1.267286	0.214057	0.646325	1.000
82	GOBP_REGULATION_OF_PHOSPHOLIPASE_C_ACTIVITY	GO:1900274	5	0.493976	1.266700	0.170520	0.639971	1.000
83	GOBP_EPIDERMIS_DEVELOPMENT	GO:0008544	5	0.493976	1.263688	0.161290	0.640731	1.000

Continuation of Table **S5**

	NAME	ID	SIZE	ES	NES	NOM.p.val	FDR.q.val	FWER.p.val
84	GOBP_REGULATION_OF_FAT_CELL_DIFFERENTIATION	GO:0045598	6	0.457851	1.257120	0.152047	0.649315	1.000
85	GOBP_IMMUNE_RESPONSE_REGULATING_SIGNALING_PATHWAY	GO:0002764	8	0.396416	1.251042	0.195286	0.657148	1.000
86	GOBP_POSITIVE_REGULATION_OF_BIOSYNTHETIC_PROCESS	GO:0009891	27	0.257470	1.239395	0.187500	0.678835	1.000
87	GOBP_REGULATION_OF_CELL_DIFFERENTIATION	GO:0045595	36	0.241848	1.228878	0.195556	0.696463	1.000
88	GOBP_REGULATION_OF_EPITHELIAL_CELL_MIGRATION	GO:0010632	6	0.436126	1.224879	0.207207	0.699072	1.000
89	GOBP_REGULATION_OF_CARBOHYDRATE_METABOLIC_PROCESS	GO:0006109	5	0.469880	1.220266	0.188679	0.703539	1.000
90	GOBP_REGULATION_OF_ANION_TRANSMEMBRANE_TRANSPORT	GO:1903959	5	0.469880	1.219432	0.229692	0.698241	1.000
91	GOBP_POSITIVE_REGULATION_OF_LIPASE_ACTIVITY	GO:0060193	5	0.493976	1.214590	0.204204	0.703439	1.000
92	GOBP_FC_RECEPTOR_SIGNALING_PATHWAY	GO:0038093	8	0.396416	1.211555	0.220056	0.703966	1.000
93	GOBP_REGULATION_OF_IMMUNE_RESPONSE	GO:0050776	12	0.337391	1.209553	0.194030	0.701548	1.000
94	GOBP_REGULATION_OF_VASCULAR_ASSOCIATED_SMOOTH_...	GO:1904705	5	0.469880	1.204525	0.218329	0.708345	1.000
95	GOBP_CARBOHYDRATE_BIOSYNTHETIC_PROCESS	GO:0016051	5	0.469880	1.201346	0.223242	0.709644	1.000
96	GOBP_MAINTENANCE_OF_LOCATION	GO:0051235	7	0.419753	1.199787	0.222561	0.706569	1.000
97	GOBP_NEGATIVE_REGULATION_OF_INTRACELLULAR_SIGN...	GO:1902532	7	0.407407	1.197808	0.206061	0.704423	1.000
98	GOBP_POSITIVE_REGULATION_OF_DEVELOPMENTAL_GROWTH	GO:0048639	8	0.382299	1.194797	0.211480	0.704186	1.000
99	GOBP_POSITIVE_REGULATION_OF_TRANSCRIPTION_BY_R...	GO:0045944	24	0.249141	1.191710	0.235808	0.704965	1.000
100	GOBP_DEVELOPMENTAL_CELL_GROWTH	GO:0048588	5	0.469880	1.190766	0.229851	0.700547	1.000
101	GOBP_RAS_PROTEIN_SIGNAL_TRANSDUCTION	GO:0007265	5	0.469880	1.186071	0.256614	0.705562	1.000
102	GOBP_REGULATION_OF_CARBOHYDRATE_BIOSYNTHETIC_P...	GO:0043255	5	0.469880	1.182099	0.241176	0.710046	1.000
103	GOBP_NEGATIVE_REGULATION_OF_PHOSPHORYLATION	GO:0042326	7	0.407407	1.178586	0.253049	0.712188	1.000
104	GOBP_RESPONSE_TO_ORGANIC_CYCLIC_COMPOUND	GO:0014070	20	0.265083	1.172416	0.232143	0.722585	1.000
105	GOBP_POSITIVE_REGULATION_OF_PHOSPHATIDYLINOSIT...	GO:0014068	5	0.469880	1.170439	0.245810	0.721199	1.000
106	GOBP_POSITIVE_REGULATION_OF_NUCLEOBASE_CONTAIN...	GO:0045935	28	0.233193	1.167646	0.235577	0.721509	1.000
107	GOBP_CELLULAR_RESPONSE_TO_ABIOTIC_STIMULUS	GO:0071214	7	0.411025	1.165801	0.246334	0.719263	1.000
108	GOBP_POSITIVE_REGULATION_OF_IMMUNE_RESPONSE	GO:0050778	9	0.357136	1.164353	0.239057	0.716059	1.000
109	GOBP_NEGATIVE_REGULATION_OF_DEVELOPMENTAL_PROCESS	GO:0051093	13	0.306667	1.164231	0.242424	0.709890	1.000
110	GOBP_REGULATION_OF_OSTEOCLAST_DIFFERENTIATION	GO:0045670	5	0.458364	1.158893	0.271084	0.716530	1.000
111	GOBP_IMPORT_INTO_CELL	GO:0098657	6	0.414634	1.156733	0.251429	0.715842	1.000
112	GOBP_REGULATION_OF_PROTEOLYSIS	GO:0030162	12	0.330956	1.154072	0.251634	0.716379	1.000
113	GOBP_REGULATION_OF_CELLULAR_COMPONENT_SIZE	GO:0032535	7	0.388229	1.151861	0.295385	0.715934	1.000
114	GOBP_NEGATIVE_REGULATION_OF_RESPONSE_TO_EXTERN...	GO:0032102	5	0.469880	1.150375	0.306630	0.713777	1.000
115	GOBP_POSITIVE_REGULATION_OF_DEVELOPMENTAL_PROCESS	GO:0051094	32	0.222729	1.143297	0.226601	0.726102	1.000
116	GOBP_NEGATIVE_REGULATION_OF_CELL_DIFFERENTIATION	GO:0045596	13	0.306667	1.126890	0.292857	0.763295	1.000
117	REACTOME_SENESCENCE_ASSOCIATED_SECRETORY_PHENO...	R-RNO-2559582	6	0.405944	1.125761	0.283582	0.759810	1.000
118	GOBP_RESPONSE_TO_ENDOGENOUS_STIMULUS	GO:0009719	34	0.224328	1.125598	0.298507	0.753967	1.000
119	GOBP_CELLULAR_COMPONENT_DISASSEMBLY	GO:0022411	6	0.417599	1.125341	0.326347	0.748121	1.000
120	GOBP_EMBRYO_DEVELOPMENT	GO:0009790	14	0.294001	1.122695	0.300000	0.749103	1.000
121	REACTOME_CELLULAR_SENESCENCE	R-RNO-2559583	6	0.405944	1.109476	0.320475	0.777557	1.000
122	REACTOME_ESR_MEDIATED_SIGNALING	R-RNO-8939211	8	0.352913	1.107232	0.276873	0.777310	1.000
123	GOBP_CELL_CELL_JUNCTION_ASSEMBLY	GO:0007043	5	0.443995	1.106311	0.319149	0.773251	1.000
124	GOBP_POSITIVE_REGULATION_OF_MULTICELLULAR_ORGA...	GO:0051240	32	0.214286	1.099257	0.349057	0.785209	1.000
125	GOBP_PHAGOCYTOSIS	GO:0006909	5	0.434803	1.095823	0.310541	0.787844	1.000
126	GOBP_POSITIVE_REGULATION_OF_BINDING	GO:0051099	6	0.402439	1.085716	0.355685	0.808114	1.000

Continuation of Table **S5**

	NAME	ID	SIZE	ES	NES	NOM.p.val	FDR.q.val	FWER.p.val
127	GOBP_REGULATION_OF_DEVELOPMENTAL_GROWTH	GO:0048638	9	0.328853	1.084204	0.329114	0.806226	1.000
128	GOBP_CELLULAR_RESPONSE_TO_ORGANIC_CYCLIC_COMPOUND	GO:0071407	13	0.282516	1.082350	0.366667	0.804528	1.000
129	GOBP_REGULATION_OF_INFLAMMATORY_RESPONSE	GO:0050727	7	0.366960	1.080222	0.334375	0.803819	1.000
130	GOBP_NEGATIVE_REGULATION_OF_LIPID_METABOLIC_PR...	GO:0045833	5	0.428219	1.070865	0.352273	0.823432	1.000
131	GOBP_REGULATION_OF_DEFENSE_RESPONSE	GO:0031347	7	0.366960	1.070584	0.359621	0.817805	1.000
132	GOBP_REGULATION_OF_CYSTEINE_TYPE_ENDOPEPTIDASE...	GO:2000116	9	0.326767	1.070419	0.373737	0.812002	1.000
133	GOBP_ACTIVATION_OF_IMMUNE_RESPONSE	GO:0002253	5	0.422002	1.069992	0.337176	0.806952	1.000
134	GOBP_POSITIVE_REGULATION_OF_ORGANELLE_ORGANIZA...	GO:0010638	10	0.320296	1.068884	0.393333	0.803905	1.000
135	REACTOME_SIGNALING_BY_NUCLEAR_RECEPTORS	R-RNO-9006931	8	0.352913	1.064179	0.345196	0.809842	1.000
136	REACTOME_ADAPTIVE_IMMUNE_SYSTEM	R-RNO-1280218	9	0.329114	1.063166	0.363636	0.806794	1.000
137	GOBP_REGULATION_OF_DNA_BINDING_TRANSCRIPTION_F...	GO:0051090	16	0.264118	1.060510	0.376923	0.808698	1.000
138	GOBP_REGULATION_OF_LIPID_LOCALIZATION	GO:1905952	5	0.416976	1.056801	0.357143	0.812501	1.000
139	REACTOME_EXTRA_NUCLEAR_ESTROGEN_SIGNALING	R-RNO-9009391	7	0.351146	1.042955	0.387302	0.843996	1.000
140	GOBP_NEGATIVE_REGULATION_OF_CELL_DEATH	GO:0060548	22	0.227819	1.036211	0.385965	0.855551	1.000
141	GOBP_REGULATION_OF_CELLULAR_AMIDE_METABOLIC_PR...	GO:0034248	9	0.305619	1.010975	0.387960	0.917540	1.000
142	GOBP_NEGATIVE_REGULATION_OF_KINASE_ACTIVITY	GO:0033673	5	0.397590	1.010869	0.421687	0.911566	1.000
143	GOBP_NEGATIVE_REGULATION_OF_BINDING	GO:0051100	6	0.370341	1.010733	0.440341	0.905626	1.000
144	GOBP_ANATOMICAL_STRUCTURE_FORMATION_INVOLVED_I...	GO:0048646	20	0.223707	1.009728	0.406250	0.901989	1.000
145	GOBP_POSITIVE_REGULATION_OF_CELL_GROWTH	GO:0030307	6	0.365675	1.007472	0.407625	0.902265	1.000
146	GOBP_NEGATIVE_REGULATION_OF_TRANSFERASE_ACTIVITY	GO:0051348	5	0.397590	1.007256	0.422222	0.896887	1.000
147	GOBP_MUSCLE_CONTRACTION	GO:0006936	5	0.409639	1.007237	0.404070	0.890948	1.000
148	GOBP_CELL_JUNCTION_ASSEMBLY	GO:0034329	8	0.327923	1.004022	0.411371	0.893360	1.000
149	GOBP_POSITIVE_REGULATION_OF_SMALL_MOLECULE_MET...	GO:0062013	5	0.409639	1.000059	0.422840	0.898774	1.000
150	GOBP_GLAND_DEVELOPMENT	GO:0048732	7	0.342857	0.996080	0.450311	0.902788	1.000
151	GOBP_RESPONSE_TO_HORMONE	GO:0009725	20	0.221500	0.993601	0.440000	0.904089	1.000
152	GOBP_REGULATION_OF_PROTEIN_CATABOLIC_PROCESS	GO:0042176	7	0.334790	0.989675	0.456395	0.908979	1.000
153	HALLMARK_UV_RESPONSE_UP	M5941	5	0.386524	0.989075	0.447222	0.904569	1.000
154	GOBP_POSITIVE_REGULATION_OF_MYELOID_LEUKOCYTE_...	GO:0002763	6	0.359850	0.981348	0.433048	0.919333	1.000
155	HALLMARK_KRAS_SIGNALING_UP	M5953	6	0.357785	0.980052	0.433243	0.916812	1.000
156	GOBP_REPRODUCTIVE_SYSTEM_DEVELOPMENT	GO:0061458	6	0.366578	0.978555	0.467422	0.915147	1.000
157	GOBP_EPITHELIUM_DEVELOPMENT	GO:0060429	15	0.254098	0.978217	0.495968	0.910075	1.000
158	GOBP_POSITIVE_REGULATION_OF_MYELOID_CELL_DIFFE...	GO:0045639	7	0.331437	0.978092	0.464968	0.904734	1.000
159	GOBP_CELLULAR_RESPONSE_TO_OXYGEN_CONTAINING_CO...	GO:1901701	29	0.203915	0.977922	0.482143	0.899679	1.000
160	GOBP_POSITIVE_REGULATION_OF_CELLULAR_COMPONENT...	GO:0044089	9	0.303320	0.972882	0.490506	0.907061	1.000
161	GOBP_APOPTOTIC_MITOCHONDRIAL_CHANGES	GO:0008637	6	0.353659	0.971863	0.463014	0.904296	1.000
162	GOBP_CELLULAR_CARBOHYDRATE_METABOLIC_PROCESS	GO:0044262	5	0.373494	0.965481	0.487730	0.916117	1.000
163	GOBP_CELL_POPULATION_PROLIFERATION	GO:0008283	32	0.194601	0.957612	0.511521	0.930712	1.000
164	GOBP_REGULATION_OF_MYELOID_CELL_DIFFERENTIATION	GO:0045637	9	0.301445	0.956828	0.469055	0.927085	1.000
165	GOBP_REGULATION_OF_MYELOID_LEUKOCYTE_DIFFERENT...	GO:0002761	8	0.313217	0.948939	0.518634	0.942771	1.000
166	GOBP_PROTEIN_CATABOLIC_PROCESS	GO:0030163	8	0.312081	0.946981	0.520376	0.942308	1.000
167	GOBP_PROTEIN_LOCALIZATION_TO_PLASMA_MEMBRANE	GO:0072659	7	0.319858	0.945721	0.505952	0.940621	1.000
168	GOBP_POLYSACCHARIDE_METABOLIC_PROCESS	GO:0005976	5	0.373494	0.943741	0.495441	0.940204	1.000
169	GOBP_POSITIVE_REGULATION_OF_CELL_DEATH	GO:0010942	19	0.218035	0.941406	0.508130	0.940476	1.000

Continuation of Table **S5**

	NAME	ID	SIZE	ES	NES	NOM.p.val	FDR.q.val	FWER.p.val
170	GOBP_INOSITOL_LIPID_MEDIATED_SIGNALING	GO:0048017	8	0.298970	0.939988	0.516820	0.938858	1.000
171	GOBP_POSITIVE_REGULATION_OF_GROWTH	GO:0045927	9	0.294208	0.937856	0.530945	0.938532	1.000
172	GOBP_POSITIVE_REGULATION_OF_DEFENSE_RESPONSE	GO:0031349	5	0.372859	0.933018	0.533333	0.945280	1.000
173	GOBP_LEUKOCYTE_MIGRATION	GO:0050900	6	0.338581	0.932096	0.503030	0.941748	1.000
174	GOBP_POSITIVE_REGULATION_OF_TRANSMEMBRANE_TRAN...	GO:0034764	7	0.308642	0.927854	0.516820	0.946815	1.000
175	GOBP_NEGATIVE_REGULATION_OF_ION_TRANSPORT	GO:0043271	11	0.259740	0.924335	0.527778	0.950346	1.000
176	GOBP_FAT_CELL_DIFFERENTIATION	GO:0045444	9	0.277650	0.921036	0.576433	0.953188	1.000
177	GOBP_REGULATION_OF_PROTEIN_LOCALIZATION_TO_CEL...	GO:1904375	6	0.336702	0.920154	0.533537	0.949859	1.000
178	GOBP_NEGATIVE_REGULATION_OF_MULTICELLULAR_ORGA...	GO:0051241	15	0.233143	0.919272	0.574713	0.946497	1.000
179	GOBP_GENERATION_OF_PRECURSOR_METABOLITES_AND_E...	GO:0006091	5	0.373494	0.917577	0.578082	0.945694	1.000
180	GOBP_REGULATION_OF_SMALL_MOLECULE_METABOLIC_PR...	GO:0062012	9	0.278481	0.917488	0.518033	0.940662	1.000
181	GOBP_POSITIVE_REGULATION_OF_CELL_DEVELOPMENT	GO:0010720	12	0.243084	0.910497	0.565056	0.952963	1.000
182	GOBP_DEVELOPMENTAL_GROWTH	GO:0048589	10	0.269419	0.909041	0.537367	0.951648	1.000
183	GOBP_RESPONSE_TO_OXYGEN_CONTAINING_COMPOUND	GO:1901700	39	0.176114	0.907330	0.634518	0.950838	1.000
184	GOBP_RESPONSE_TO_LIGHT_STIMULUS	GO:0009416	11	0.255317	0.907178	0.580071	0.946049	1.000
185	GOBP_INTRACELLULAR_PROTEIN_TRANSPORT	GO:0006886	8	0.287500	0.901398	0.580060	0.955180	1.000
186	HALLMARK_EPITHELIAL_MESENCHYMAL_TRANSITION	M5930	5	0.355732	0.901075	0.582656	0.951018	1.000
187	REACTOME_NGF_STIMULATED_TRANSCRIPTION	R-RNO-9031628	9	0.275645	0.899694	0.616129	0.949714	1.000
188	GOBP_TISSUE_HOMEOSTASIS	GO:0001894	5	0.356290	0.897363	0.557185	0.950637	1.000
189	GOBP_POSITIVE_REGULATION_OF_ION_TRANSPORT	GO:0043270	17	0.210952	0.894015	0.607930	0.954024	1.000
190	GOBP_REGULATION_OF_CELL_CYCLE	GO:0051726	13	0.241245	0.891463	0.584559	0.954875	1.000
191	GOBP_WOUND_HEALING	GO:0042060	13	0.239113	0.891129	0.588679	0.950775	1.000
192	GOBP_ANIMAL_ORGAN_MORPHOGENESIS	GO:0009887	12	0.244699	0.890917	0.576779	0.946445	1.000
193	GOBP_POSITIVE_REGULATION_OF_NERVOUS_SYSTEM_DEV...	GO:0051962	12	0.243084	0.890763	0.603448	0.941994	1.000
194	GOBP_AGING	GO:0007568	13	0.239576	0.888160	0.575290	0.943376	1.000
195	GOBP_POSITIVE_REGULATION_OF_NEUROGENESIS	GO:0050769	12	0.243084	0.887733	0.622776	0.939708	1.000
196	REACTOME_ESTROGEN_DEPENDENT_NUCLEAR_EVENTS_DOW...	R-RNO-9634638	5	0.352186	0.887601	0.573913	0.935153	1.000
197	GOBP_POSITIVE_REGULATION_OF_ANION_TRANSPORT	GO:1903793	9	0.272770	0.886947	0.621212	0.931925	1.000
198	GOBP_POSITIVE_REGULATION_OF_GENE_EXPRESSION	GO:0010628	16	0.216239	0.885409	0.593407	0.931099	1.000
199	GOBP_NEGATIVE_REGULATION_OF_IMMUNE_SYSTEM_PROCESS	GO:0002683	5	0.350482	0.877843	0.589970	0.943670	1.000
200	GOBP_REGULATION_OF_NERVOUS_SYSTEM_DEVELOPMENT	GO:0051960	15	0.219178	0.867559	0.611111	0.963499	1.000
201	GOBP_REGULATION_OF_CELLULAR_COMPONENT_MOVEMENT	GO:0051270	15	0.217715	0.866720	0.666667	0.960601	1.000
202	GOBP_REGULATION_OF_CELL_DEVELOPMENT	GO:0060284	15	0.219178	0.863752	0.654762	0.962928	1.000
203	GOBP_NEGATIVE_REGULATION_OF_CELL_DEVELOPMENT	GO:0010721	6	0.317073	0.862027	0.608955	0.962257	1.000
204	GOBP_POSITIVE_REGULATION_OF_CELLULAR_PROTEIN_L...	GO:1903829	7	0.283951	0.853302	0.640244	0.976893	1.000
205	GOBP_DEFENSE_RESPONSE	GO:0006952	19	0.194489	0.851084	0.678392	0.976831	1.000
206	GOBP_PATTERN_SPECIFICATION_PROCESS	GO:0007389	6	0.304878	0.849903	0.677741	0.974819	1.000
207	GOBP_MULTI_ORGANISM_PROCESS	GO:0051704	16	0.208726	0.849902	0.690476	0.970160	1.000
208	GOBP_PERIPHERAL_NERVOUS_SYSTEM_DEVELOPMENT	GO:0007422	9	0.260110	0.849528	0.660377	0.966426	1.000
209	REACTOME_INFECTIOUS_DISEASE	R-HSA-5663205	12	0.227134	0.842919	0.671378	0.976145	1.000
210	GOBP_POSITIVE_REGULATION_OF_NEURON_DEATH	GO:1901216	5	0.335224	0.838113	0.634921	0.982171	1.000
211	GOBP_REGIONALIZATION	GO:0003002	6	0.304878	0.837841	0.643750	0.978282	1.000
212	GOBP_PROTEIN_MODIFICATION_BY_SMALL_PROTEIN_CON...	GO:0070647	7	0.278118	0.835961	0.658892	0.977817	1.000

Continuation of Table **S5**

	NAME	ID	SIZE	ES	NES	NOM.p.val	FDR.q.val	FWER.p.val
213	GOBP_PHOSPHATIDYLINOSITOL_3_KINASE_SIGNALING	GO:0014065	7	0.283951	0.835399	0.625369	0.974362	1.000
214	GOBP_NEGATIVE_REGULATION_OF_CATABOLIC_PROCESS	GO:0009895	8	0.273310	0.834456	0.714715	0.972301	1.000
215	GOBP_REGULATION_OF_ANATOMICAL_STRUCTURE_SIZE	GO:0090066	10	0.244660	0.833528	0.676768	0.969872	1.000
216	GOBP_CHROMATIN_ORGANIZATION	GO:0006325	5	0.331573	0.831907	0.654054	0.968753	1.000
217	REACTOME_FC_EPSILON_RECEPTOR_FCERI_SIGNALING	R-RNO-2454202	7	0.283951	0.829759	0.682779	0.968704	1.000
218	GOBP_FC_EPSILON_RECEPTOR_SIGNALING_PATHWAY	GO:0038095	7	0.283951	0.823928	0.706061	0.976878	1.000
219	GOBP_EPHRIN_RECEPTOR_SIGNALING_PATHWAY	GO:0048013	5	0.320222	0.823600	0.659459	0.973131	1.000
220	GOBP_REGULATION_OF_CELLULAR_COMPONENT_BIOGENESIS	GO:0044087	10	0.242218	0.820828	0.687296	0.974718	1.000
221	GOBP_REGULATION_OF_NEUROGENESIS	GO:0050767	14	0.216216	0.820302	0.685121	0.971337	1.000
222	GOBP_NEGATIVE_REGULATION_OF_BIOSYNTHETIC_PROCESS	GO:0009890	13	0.222495	0.819644	0.697318	0.968264	1.000
223	GOBP_NEGATIVE_REGULATION_OF_PROTEIN_MODIFICATI...	GO:0031400	10	0.235815	0.817842	0.693333	0.967258	1.000
224	GOBP_REGULATION_OF_AXONOGENESIS	GO:0050770	7	0.280137	0.817223	0.684848	0.964172	1.000
225	GOBP_REGULATION_OF_HYDROLASE_ACTIVITY	GO:0051336	22	0.181878	0.815166	0.737052	0.964345	1.000
226	GOBP_RESPONSE_TO_WOUNDING	GO:0009611	15	0.196574	0.815110	0.725564	0.960153	1.000
227	GOBP_NEGATIVE_REGULATION_OF_RESPONSE_TO_STIMULUS	GO:0048585	16	0.199906	0.814649	0.710623	0.956822	1.000
228	GOBP_POSITIVE_REGULATION_OF_PEPTIDASE_ACTIVITY	GO:0010952	7	0.267741	0.810088	0.695522	0.961275	1.000
229	GOBP_REGULATION_OF_MULTICELLULAR_ORGANISMAL_DE...	GO:2000026	31	0.161659	0.807526	0.738095	0.962193	1.000
230	HALLMARK_PI3K_AKT_MTOR_SIGNALING	M5923	6	0.289532	0.804435	0.696486	0.964217	1.000
231	GOBP_CELLULAR_LIPID_METABOLIC_PROCESS	GO:0044255	7	0.271605	0.797163	0.752443	0.973852	1.000
232	GOBP_NEGATIVE_REGULATION_OF_NERVOUS_SYSTEM_DEV...	GO:0051961	5	0.313253	0.796881	0.692547	0.970271	1.000
233	GOBP_RESPONSE_TO_MOLECULE_OF_BACTERIAL_ORIGIN	GO:0002237	13	0.207770	0.796052	0.749129	0.967679	1.000
234	GOBP_POSITIVE_REGULATION_OF_CELLULAR_COMPONENT...	GO:0051130	24	0.168940	0.795447	0.785088	0.964975	1.000
235	GOBP_RESPONSE_TO_BACTERIUM	GO:0009617	13	0.207770	0.794840	0.796491	0.961974	1.000
236	REACTOME_HEMOSTASIS	R-RNO-109582	14	0.202594	0.793949	0.772908	0.959309	1.000
237	GOBP_CARBOHYDRATE_DERIVATIVE_METABOLIC_PROCESS	GO:1901135	8	0.261345	0.793505	0.736508	0.956158	1.000
238	GOBP_REGULATION_OF_ENDOTHELIAL_CELL_MIGRATION	GO:0010594	5	0.314056	0.791663	0.709677	0.955811	1.000
239	GOBP_ENZYME_LINKED_RECEPTOR_PROTEIN_SIGNALING_...	GO:0007167	24	0.171875	0.788210	0.767857	0.958480	1.000
240	GOBP_ORGANIC_ACID_METABOLIC_PROCESS	GO:0006082	7	0.271605	0.785408	0.723032	0.959606	1.000
241	GOBP_PEPTIDE_BIOSYNTHETIC_PROCESS	GO:0043043	6	0.289471	0.783609	0.716714	0.958802	1.000
242	GOBP_RESPONSE_TO_RADIATION	GO:0009314	13	0.205345	0.783098	0.768683	0.955835	1.000
243	GOBP_MUSCLE_CELL_DEVELOPMENT	GO:0055001	5	0.313253	0.777775	0.729107	0.961726	1.000
244	GOBP_POSITIVE_REGULATION_OF_CYSTEINE_TYPE_ENDO...	GO:2001056	7	0.267741	0.775864	0.783172	0.961099	1.000
245	GOBP_AMIDE_BIOSYNTHETIC_PROCESS	GO:0043604	6	0.289471	0.775495	0.717579	0.957720	1.000
246	GOBP_NEGATIVE_REGULATION_OF_NUCLEOBASE_CONTAIN...	GO:0045934	13	0.204303	0.775257	0.761092	0.954186	1.000
247	GOBP_NEGATIVE_REGULATION_OF_ANION_TRANSPORT	GO:1903792	8	0.250000	0.774839	0.750000	0.951223	1.000
248	GOBP_REPRODUCTION	GO:0000003	18	0.184778	0.770583	0.825203	0.954853	1.000
249	GOBP_EMBRYONIC_MORPHOGENESIS	GO:0048598	6	0.280488	0.764235	0.742690	0.961830	1.000
250	GOBP_LIPID_LOCALIZATION	GO:0010876	10	0.230769	0.760356	0.800725	0.964581	1.000
251	GOBP_NEUROINFLAMMATORY_RESPONSE	GO:0150076	5	0.301644	0.759708	0.760000	0.961775	1.000
252	GOBP_LIPID_BIOSYNTHETIC_PROCESS	GO:0008610	6	0.272642	0.759200	0.787710	0.959081	1.000
253	GOBP_RESPONSE_TO_TUMOR_NECROSIS_FACTOR	GO:0034612	7	0.259082	0.756691	0.800000	0.959142	1.000
254	GOBP_REGULATION_OF_LIPID_BIOSYNTHETIC_PROCESS	GO:0046890	6	0.272642	0.751940	0.760684	0.963120	1.000
255	GOBP_IN_UTERO_EMBRYONIC_DEVELOPMENT	GO:0001701	8	0.246363	0.748104	0.800000	0.965331	1.000

Continuation of Table **S5**

	NAME	ID	SIZE	ES	NES	NOM.p.val	FDR.q.val	FWER.p.val
256	GOBP_CELL_GROWTH	GO:0016049	10	0.223149	0.747839	0.765151	0.961920	1.000
257	GOBP_INTRACELLULAR_RECEPTOR_SIGNALING_PATHWAY	GO:0030522	5	0.294181	0.745583	0.784367	0.961607	1.000
258	GOBP_POSITIVE_REGULATION_OF_PROTEOLYSIS	GO:0045862	8	0.243425	0.744545	0.823129	0.959882	1.000
259	GOBP_REGULATION_OF_PHOSPHATIDYLINOSITOL_3_KINA...	GO:0014066	6	0.280488	0.743752	0.793313	0.957540	1.000
260	GOBP_MONOCARBOXYLIC_ACID_METABOLIC_PROCESS	GO:0032787	6	0.268293	0.739179	0.800000	0.961443	1.000
261	GOBP_NEURON_MIGRATION	GO:0001764	5	0.301205	0.738954	0.778393	0.958103	1.000
262	GOBP_POSITIVE_REGULATION_OF_EPITHELIAL_CELL_MI...	GO:0010634	5	0.290324	0.736111	0.782967	0.958939	1.000
263	GOBP_REGULATION_OF_GROWTH	GO:0040008	13	0.186866	0.731119	0.835125	0.963151	1.000
264	GOBP_RESPONSE_TO_INSULIN	GO:0032868	5	0.289157	0.729405	0.788462	0.961919	1.000
265	GOBP_POSITIVE_REGULATION_OF_CELL_POPULATION_PR...	GO:0008284	21	0.163806	0.723396	0.881356	0.967522	1.000
266	GOBP_CELLULAR_RESPONSE_TO_BIOTIC_STIMULUS	GO:0071216	8	0.225937	0.717785	0.853896	0.971754	1.000
267	GOBP_NERVE_DEVELOPMENT	GO:0021675	9	0.215190	0.711452	0.885714	0.976626	1.000
268	GOBP_ORGANONITROGEN_COMPOUND_CATABOLIC_PROCESS	GO:1901565	10	0.210715	0.710265	0.856643	0.974580	1.000
269	GOBP_CELLULAR_RESPONSE_TO_STARVATION	GO:0009267	5	0.277108	0.704063	0.818444	0.980015	1.000
270	GOBP_CELLULAR_RESPONSE_TO_MOLECULE_OF_BACTERIA...	GO:0071219	8	0.225937	0.700387	0.851064	0.981361	1.000
271	GOBP_RESPONSE_TO_STARVATION	GO:0042594	5	0.277108	0.697356	0.853731	0.981609	1.000
272	GOBP_PEPTIDE_METABOLIC_PROCESS	GO:0006518	11	0.198234	0.697073	0.851711	0.978430	1.000
273	GOBP_RESPONSE_TO_NERVE_GROWTH_FACTOR	GO:1990089	7	0.236549	0.696125	0.849398	0.976045	1.000
274	GOBP_MACROMOLECULE_CATABOLIC_PROCESS	GO:0009057	11	0.195173	0.694871	0.875912	0.974343	1.000
275	GOBP_REGULATION_OF_ORGAN_GROWTH	GO:0046620	5	0.277108	0.694240	0.841945	0.971570	1.000
276	GOBP_ORGAN_GROWTH	GO:0035265	5	0.277108	0.692755	0.848315	0.970178	1.000
277	GOBP_POSITIVE_REGULATION_OF_LEUKOCYTE_CELL_CEL...	GO:1903039	5	0.269115	0.692685	0.856716	0.966785	1.000
278	GOBP_REGULATION_OF_CYTOSKELETON_ORGANIZATION	GO:0051493	5	0.277109	0.688593	0.857143	0.968662	1.000
279	GOBP_ORGANIC_HYDROXY_COMPOUND_METABOLIC_PROCESS	GO:1901615	8	0.218368	0.688284	0.854985	0.965549	1.000
280	GOBP_LEUKOCYTE_CELL_CELL_ADHESION	GO:0007159	6	0.247227	0.687334	0.870769	0.963218	1.000
281	GOBP_PEPTIDYL_SERINE_MODIFICATION	GO:0018209	12	0.184211	0.683352	0.892857	0.964531	1.000
282	GOBP_ANION_TRANSMEMBRANE_TRANSPORT	GO:0098656	7	0.234568	0.682120	0.884849	0.962667	1.000
283	GOBP_MATERNAL_PROCESS_INVOLVED_IN_FEMALE_PREGN...	GO:0060135	5	0.265060	0.681374	0.859838	0.960348	1.000
284	GOBP_NEGATIVE_REGULATION_OF_NEURON_APOPTOTIC_P...	GO:0043524	8	0.212500	0.680021	0.879880	0.958400	1.000
285	REACTOME_SIGNALING_BY_NTRKS	R-RNO-166520	18	0.162836	0.675012	0.917012	0.960637	1.000
286	GOBP_RESPONSE_TO_CALCIUM_ION	GO:0051592	6	0.242185	0.671145	0.900621	0.961645	1.000
287	GOBP_RESPONSE_TO_OXYGEN_LEVELS	GO:0070482	8	0.215319	0.669724	0.881789	0.959815	1.000
288	GOBP_REGULATION_OF_PROTEIN_SERINE_THREONINE_KI...	GO:0071900	12	0.180421	0.669579	0.885714	0.956583	1.000
289	REACTOME_L1CAM_INTERACTIONS	R-RNO-373760	5	0.265481	0.668440	0.865285	0.954435	1.000
290	GOBP_POSITIVE_REGULATION_OF_CELL_CELL_ADHESION	GO:0022409	5	0.269115	0.666356	0.886179	0.953760	1.000
291	GOBP_TRANSMEMBRANE_RECEPTOR_PROTEIN_TYROSINE_K...	GO:0007169	19	0.159420	0.666124	0.907258	0.950716	1.000
292	GOBP_ORGANIC_HYDROXY_COMPOUND_TRANSPORT	GO:0015850	6	0.240146	0.663121	0.883495	0.950676	1.000
293	GOBP_MITOCHONDRION_ORGANIZATION	GO:0007005	7	0.222222	0.661316	0.883436	0.949491	1.000
294	GOBP_BIOLOGICAL_PROCESS_INVOLVED_IN_SYMBIOTIC_...	GO:004440	10	0.194383	0.647319	0.919192	0.960717	1.000
295	GOBP_REGULATION_OF_ORGANELLE_ORGANIZATION	GO:0033043	14	0.166005	0.645548	0.933566	0.959363	1.000
296	REACTOME_TRANSCRIPTIONAL_REGULATION_BY_TP53	R-RNO-3700989	6	0.232742	0.642498	0.921136	0.958962	1.000
297	GOBP_POSITIVE_REGULATION_OF_HYDROLASE_ACTIVITY	GO:0051345	16	0.152778	0.633777	0.965780	0.963561	1.000
298	GOBP_SMALL_GTPASE_MEDIATED_SIGNAL_TRANSDUCTION	GO:0007264	8	0.200000	0.625020	0.930314	0.967567	1.000

Continuation of Table **S5**

	NAME	ID	SIZE	ES	NES	NOM.p.val	FDR.q.val	FWER.p.val
299	GOBP_EMBRYO_DEVELOPMENT_ENDING_IN_BIRTH_OR_EGG...	GO:0009792	9	0.191396	0.618590	0.951890	0.969345	1.000
300	GOBP_FATTY_ACID_TRANSPORT	GO:0015908	6	0.219512	0.610001	0.949843	0.972896	1.000
301	GOBP_HEART_DEVELOPMENT	GO:0007507	6	0.210200	0.604137	0.969040	0.974289	1.000
302	GOBP_GROWTH	GO:0040007	14	0.153747	0.598282	0.989437	0.974933	1.000
303	GOBP_REGULATION_OF_MITOTIC_CELL_CYCLE	GO:0007346	5	0.234588	0.582728	0.947059	0.980996	1.000
304	GOBP_CELLULAR_AMIDE_METABOLIC_PROCESS	GO:0043603	12	0.155332	0.578263	0.982014	0.980063	1.000
305	GOBP_PROTEIN_AUTOPHOSPHORYLATION	GO:0046777	5	0.221961	0.570884	0.955182	0.980769	1.000
306	GOBP_CARDIAC_MUSCLE_TISSUE_DEVELOPMENT	GO:0048738	6	0.210200	0.569538	0.937500	0.978238	1.000
307	GOBP_CYTOKINE_PRODUCTION	GO:0001816	10	0.153846	0.534602	0.990000	0.988565	1.000
308	GOBP_RESPONSE_TO_ACID_CHEMICAL	GO:0001101	6	0.189960	0.515294	0.989041	0.990339	1.000
309	GOBP_NEGATIVE_REGULATION_OF_TRANSPORT	GO:0051051	13	-0.135637	-0.427250	0.994467	0.996896	1.000
310	GOBP_RESPONSE_TO_KETONE	GO:1901654	7	-0.178454	-0.454553	0.994021	0.996023	1.000
311	GOBP_RESPONSE_TO_LIPID	GO:0033993	19	-0.134449	-0.464214	0.986505	0.996853	1.000
312	REACTOME_TOLL_LIKE_RECEPTOR_TLR1_TLR2_CASCADE	R-RNO-168179	8	-0.180490	-0.480922	0.989474	0.995826	1.000
313	REACTOME_MYD88_INDEPENDENT_TLR4_CASCADE	R-RNO-166166	8	-0.180490	-0.483036	0.988201	0.997781	1.000
314	REACTOME_TOLL_LIKE_RECEPTOR_CASCADES	R-RNO-168898	8	-0.180490	-0.483477	0.989899	1.000000	1.000
315	REACTOME_TOLL_LIKE_RECEPTOR_9_TLR9_CASCADE	R-RNO-168138	8	-0.180490	-0.483734	0.991241	1.000000	1.000
316	GOBP_POSITIVE_REGULATION_OF_CELL_PROJECTION_OR...	GO:0031346	10	-0.177471	-0.502915	0.977974	0.999407	1.000
317	GOBP_POSTTRANSCRIPTIONAL_REGULATION_OF_GENE_EX...	GO:0010608	8	-0.187858	-0.507799	0.984127	1.000000	1.000
318	GOBP_REGULATION_OF_MUSCLE_CELL_DIFFERENTIATION	GO:0051147	6	-0.211709	-0.512063	0.986466	1.000000	1.000
319	GOBP_POSITIVE_REGULATION_OF_PEPTIDYL_TYROSINE_...	GO:0050731	8	-0.194058	-0.513289	0.965714	1.000000	1.000
320	GOBP_REGULATION_OF_PROTEIN_STABILITY	GO:0031647	5	-0.229634	-0.516532	0.987578	1.000000	1.000
321	GOBP_REGULATION_OF_LYMPHOCYTE_ACTIVATION	GO:0051249	5	-0.240510	-0.539624	0.966507	0.997355	1.000
322	GOBP_POSITIVE_REGULATION_OF_ESTABLISHMENT_OF_P...	GO:1904951	5	-0.242344	-0.548802	0.970543	0.995105	1.000
323	GOBP_REGULATION_OF_CELL_CELL_ADHESION	GO:0022407	9	-0.202706	-0.551777	0.958271	0.996081	1.000
324	GOBP_ACTIVATION_OF_PROTEIN_KINASE_ACTIVITY	GO:0032147	10	-0.192447	-0.553195	0.968526	0.997793	1.000
325	GOBP_NEGATIVE_REGULATION_OF_GENE_EXPRESSION	GO:0010629	11	-0.188793	-0.556301	0.961538	0.998610	1.000
326	GOBP_MUSCLE_TISSUE_DEVELOPMENT	GO:0060537	11	-0.190375	-0.561453	0.964865	0.998176	1.000
327	GOBP_RESPONSE_TO_PEPTIDE	GO:1901652	18	-0.166069	-0.561937	0.949797	1.000000	1.000
328	GOBP_LIPID_METABOLIC_PROCESS	GO:0006629	10	-0.201568	-0.565300	0.949852	1.000000	1.000
329	GOBP_RESPONSE_TO_METAL_ION	GO:0010038	15	-0.172842	-0.568456	0.953947	1.000000	1.000
330	GOBP_REGULATION_OF_CELLULAR_PROTEIN_LOCALIZATION	GO:1903827	12	-0.191124	-0.579049	0.949454	0.997400	1.000
331	GOBP_COAGULATION	GO:0050817	8	-0.218460	-0.588327	0.943262	0.993426	1.000
332	GOBP_DEVELOPMENTAL_PROCESS_INVOLVED_IN_REPRODU...	GO:0003006	11	-0.201896	-0.589013	0.938289	0.995472	1.000
333	GOBP_CELLULAR_RESPONSE_TO_LIPID	GO:0071396	15	-0.186212	-0.591105	0.938089	0.996469	1.000
334	GOBP_NEURON_APOPTOTIC_PROCESS	GO:0051402	12	-0.195200	-0.593506	0.934752	0.997232	1.000
335	GOBP_NEGATIVE_REGULATION_OF_SIGNALING	GO:0023057	21	-0.168633	-0.593890	0.938642	0.999484	1.000
336	GOBP_REGULATION_OF_GLIOGENESIS	GO:0014013	5	-0.263896	-0.594080	0.938931	1.000000	1.000
337	GOBP_RESPONSE_TO_GROWTH_FACTOR	GO:0070848	18	-0.175529	-0.597909	0.927152	1.000000	1.000
338	GOBP_CELLULAR_RESPONSE_TO_DNA_DAMAGE_STIMULUS	GO:0006974	5	-0.262447	-0.598897	0.945652	1.000000	1.000
339	GOBP_NEGATIVE_REGULATION_OF_CELLULAR_COMPONENT...	GO:0051129	11	-0.202792	-0.599578	0.922628	1.000000	1.000
340	GOBP_DNA_METABOLIC_PROCESS	GO:0006259	6	-0.249659	-0.600673	0.934884	1.000000	1.000
341	GOBP_POSITIVE_REGULATION_OF_GLIOGENESIS	GO:0014015	5	-0.263896	-0.602404	0.943164	1.000000	1.000

Continuation of Table **S5**

	NAME	ID	SIZE	ES	NES	NOM.p.val	FDR.q.val	FWER.p.val
342	GOBP_ERK1_AND_ERK2_CASCADE	GO:0070371	5	-0.271059	-0.603101	0.924471	1.000000	1.000
343	GOBP_POSITIVE_REGULATION_OF_DNA_BINDING_TRANSC...	GO:0051091	10	-0.209573	-0.606026	0.928258	1.000000	1.000
344	GOBP_REGULATION_OF_PROTEIN_PHOSPHORYLATION	GO:0001932	25	-0.165814	-0.607606	0.916991	1.000000	1.000
345	GOBP_RESPONSE_TO_DRUG	GO:0042493	11	-0.208202	-0.614552	0.915954	1.000000	1.000
346	GOBP_REGULATION_OF_PHOSPHORUS_METABOLIC_PROCESS	GO:0051174	26	-0.166113	-0.615450	0.921290	1.000000	1.000
347	GOBP_LEARNING	GO:0007612	13	-0.198676	-0.616478	0.915160	1.000000	1.000
348	GOBP_ORGANIC_ACID_TRANSPORT	GO:0015849	7	-0.241238	-0.619475	0.911544	1.000000	1.000
349	GOBP_ORGANONITROGEN_COMPOUND_BIOSYNTHETIC_PROCESS	GO:1901566	13	-0.200773	-0.619753	0.927966	1.000000	1.000
350	GOBP_TRANSMEMBRANE_RECEPTOR_PROTEIN_SERINE_THR...	GO:0007178	6	-0.255637	-0.623223	0.910906	1.000000	1.000
351	GOBP_SMALL_MOLECULE_BIOSYNTHETIC_PROCESS	GO:0044283	5	-0.273716	-0.625954	0.930269	1.000000	1.000
352	GOBP_REGULATION_OF_TRANSFERASE_ACTIVITY	GO:0051338	21	-0.182486	-0.629106	0.919481	1.000000	1.000
353	REACTOME_NUCLEAR_EVENTS_KINASE_AND_TRANSCRIPTI...	R-RNO-198725	11	-0.216813	-0.634729	0.898352	1.000000	1.000
354	GOBP_REGULATION_OF_PEPTIDYL_TYROSINE_PHOSPHORY...	GO:0050730	9	-0.226525	-0.635775	0.904286	1.000000	1.000
355	GOBP_REGULATION_OF_PROTEIN_KINASE_ACTIVITY	GO:0045859	21	-0.182486	-0.635993	0.909677	1.000000	1.000
356	GOBP_POSITIVE_REGULATION_OF_LOCOMOTION	GO:0040017	11	-0.215677	-0.640106	0.895714	1.000000	1.000
357	REACTOME_INTERLEUKIN_17_SIGNALING	R-RNO-448424	6	-0.265749	-0.641903	0.892012	1.000000	1.000
358	GOBP_MUSCLE_STRUCTURE_DEVELOPMENT	GO:0061061	16	-0.197464	-0.646219	0.886792	1.000000	1.000
359	GOBP_POSITIVE_REGULATION_OF_PROTEIN_SERINE_THR...	GO:0071902	9	-0.242535	-0.648824	0.878437	1.000000	1.000
360	GOBP_ANATOMICAL_STRUCTURE_HOMEOSTASIS	GO:0060249	7	-0.252779	-0.649021	0.885246	1.000000	1.000
361	GOBP_NEGATIVE_REGULATION_OF_PROTEIN_CATABOLIC_...	GO:0042177	5	-0.291270	-0.655322	0.899038	1.000000	1.000
362	GOBP_MONOCARBOXYLIC_ACID_TRANSPORT	GO:0015718	5	-0.293809	-0.655807	0.894495	1.000000	1.000
363	GOBP_CELLULAR_RESPONSE_TO_NITROGEN_COMPOUND	GO:1901699	20	-0.186747	-0.657272	0.900937	1.000000	1.000
364	GOBP_DICARBOXYLIC_ACID_TRANSPORT	GO:0006835	5	-0.293809	-0.666263	0.873684	1.000000	1.000
365	GOBP_REGULATION_OF_PROTEIN_MODIFICATION_PROCESS	GO:0031399	28	-0.179845	-0.667107	0.881013	1.000000	1.000
366	REACTOME_EPH_EPHRIN_SIGNALING	R-RNO-2682334	6	-0.276592	-0.669302	0.873457	1.000000	1.000
367	GOBP_REGULATION_OF_INTRACELLULAR_SIGNAL_TRANSD...	GO:1902531	21	-0.190509	-0.671656	0.879630	1.000000	1.000
368	GOBP_CELLULAR_RESPONSE_TO_KETONE	GO:1901655	5	-0.295317	-0.674412	0.875583	1.000000	1.000
369	GOBP_SKELETAL_MUSCLE_ORGAN_DEVELOPMENT	GO:0060538	5	-0.302378	-0.676887	0.866373	1.000000	1.000
370	GOBP_MONOAMINE_TRANSPORT	GO:0015844	5	-0.298117	-0.677362	0.868657	1.000000	1.000
371	GOBP_ACIDIC_AMINO_ACID_TRANSPORT	GO:0015800	5	-0.293809	-0.677600	0.898551	1.000000	1.000
372	GOBP_RESPONSE_TO_INORGANIC_SUBSTANCE	GO:0010035	19	-0.196844	-0.677900	0.875170	1.000000	1.000
373	GOBP_MONONUCLEAR_CELL_DIFFERENTIATION	GO:1903131	7	-0.270377	-0.678575	0.848921	1.000000	1.000
374	GOBP_POSITIVE_REGULATION_OF_CYTOKINE_PRODUCTION	GO:0001819	7	-0.267851	-0.679774	0.873039	1.000000	1.000
375	GOBP_LOCOMOTION	GO:0040011	31	-0.183327	-0.680395	0.873724	1.000000	1.000
376	GOBP_CHROMOSOME_ORGANIZATION	GO:0051276	6	-0.277617	-0.681208	0.844512	1.000000	1.000
377	GOBP_T_CELL_ACTIVATION	GO:0042110	8	-0.263519	-0.683695	0.851485	1.000000	1.000
378	GOBP_RESPONSE_TO_ABIOTIC_STIMULUS	GO:0009628	27	-0.184636	-0.683808	0.871665	1.000000	1.000
379	GOBP_RESPONSE_TO_NITROGEN_COMPOUND	GO:1901698	35	-0.181536	-0.689073	0.864365	1.000000	1.000
380	GOBP_ERBB_SIGNALING_PATHWAY	GO:0038127	5	-0.310468	-0.692087	0.855590	1.000000	1.000
381	GOBP_DEFENSE_RESPONSE_TO_OTHER_ORGANISM	GO:0098542	9	-0.257580	-0.699363	0.847645	1.000000	1.000
382	GOBP_CELLULAR_RESPONSE_TO_ALCOHOL	GO:0097306	5	-0.310764	-0.702464	0.844720	1.000000	1.000
383	GOBP_AMYLOID_PRECURSOR_PROTEIN_METABOLIC_PROCESS	GO:0042982	5	-0.311497	-0.703389	0.848485	1.000000	1.000
384	GOBP_AMYLOID_BETA_METABOLIC_PROCESS	GO:0050435	5	-0.311497	-0.705760	0.842795	1.000000	1.000

Continuation of Table **S5**

	NAME	ID	SIZE	ES	NES	NOM.p.val	FDR.q.val	FWER.p.val
385	GOBP_CELLULAR_GLUCOSE_HOMEOSTASIS	GO:0001678	5	-0.312020	-0.705921	0.858730	1.000000	1.000
386	GOBP_PEPTIDYL_TYROSINE_MODIFICATION	GO:0018212	10	-0.246353	-0.708059	0.829132	1.000000	1.000
387	GOBP_CIRCULATORY_SYSTEM_PROCESS	GO:0003013	5	-0.308379	-0.709256	0.831832	1.000000	1.000
388	GOBP_ASSOCIATIVE_LEARNING	GO:0008306	7	-0.276491	-0.710629	0.806735	1.000000	1.000
389	GOBP_POSITIVE_REGULATION_OF_CELL_ADHESION	GO:0045785	7	-0.282950	-0.711715	0.823009	1.000000	1.000
390	GOBP_AMYLOID_PRECURSOR_PROTEIN_CATABOLIC_PROCESS	GO:0042987	5	-0.311497	-0.712191	0.825000	1.000000	1.000
391	REACTOME_SIGNALING_BY_NTRK2_TRKB	R-RNO-9006115	6	-0.298219	-0.712497	0.818862	1.000000	1.000
392	GOBP_POSITIVE_REGULATION_OF_PHOSPHORUS_METABOL...	GO:0010562	23	-0.200352	-0.715360	0.832011	1.000000	1.000
393	GOBP_ACTIVATION_OF_MAPK_ACTIVITY	GO:0000187	6	-0.297942	-0.720217	0.827534	1.000000	1.000
394	GOBP_NEGATIVE_REGULATION_OF_NEURON_DEATH	GO:1901215	11	-0.243287	-0.720913	0.820225	1.000000	1.000
395	GOBP_DEPHOSPHORYLATION	GO:0016311	7	-0.289380	-0.721810	0.801161	1.000000	1.000
396	GOBP_REGULATION_OF_ANION_TRANSPORT	GO:0044070	20	-0.207736	-0.722120	0.822493	1.000000	1.000
397	GOBP_SMALL_MOLECULE_METABOLIC_PROCESS	GO:0044281	16	-0.219801	-0.723830	0.806011	1.000000	1.000
398	GOBP_REGULATION_OF_CELLULAR_CATABOLIC_PROCESS	GO:0031329	10	-0.253143	-0.724799	0.829545	1.000000	1.000
399	GOBP_POSITIVE_REGULATION_OF_MAP_KINASE_ACTIVITY	GO:0043406	6	-0.297942	-0.728063	0.808219	1.000000	1.000
400	GOBP_PROTEIN_DEPHOSPHORYLATION	GO:0006470	7	-0.289380	-0.728410	0.810089	1.000000	1.000
401	GOBP_CELL_CYCLE	GO:0007049	21	-0.210750	-0.728811	0.810667	1.000000	1.000
402	GOBP_RESPONSE_TO_BIOTIC_STIMULUS	GO:0009607	17	-0.220579	-0.730327	0.809652	1.000000	1.000
403	GOBP_PLATELET_ACTIVATION	GO:0030168	6	-0.301307	-0.731528	0.820000	1.000000	1.000
404	REACTOME_INNATE_IMMUNE_SYSTEM	R-RNO-168249	16	-0.219605	-0.731597	0.809079	1.000000	1.000
405	GOBP_POSITIVE_REGULATION_OF_PROTEIN_PHOSPHORYL...	GO:0001934	22	-0.207491	-0.733522	0.826087	1.000000	1.000
406	GOBP_MULTICELLULAR_ORGANISM_REPRODUCTION	GO:0032504	12	-0.238146	-0.733641	0.811921	1.000000	1.000
407	GOBP_POSITIVE_REGULATION_OF_PROTEIN_KINASE_ACT...	GO:0045860	18	-0.221264	-0.736282	0.798408	1.000000	1.000
408	GOBP_POSITIVE_REGULATION_OF_PROTEIN_MODIFICATI...	GO:0031401	22	-0.207491	-0.741944	0.809524	1.000000	1.000
409	GOBP_HOMEOSTASIS_OF_NUMBER_OF_CELLS	GO:0048872	5	-0.323521	-0.742061	0.800578	1.000000	1.000
410	GOBP_MUSCLE_CELL_DIFFERENTIATION	GO:0042692	10	-0.262719	-0.746703	0.768202	1.000000	1.000
411	GOBP_RESPONSE_TO_TEMPERATURE_STIMULUS	GO:0009266	9	-0.272981	-0.747637	0.792264	1.000000	1.000
412	REACTOME_MAPK_TARGETS_NUCLEAR_EVENTS_MEDIATED_...	R-HSA-450282	5	-0.333901	-0.748503	0.793003	1.000000	1.000
413	GOBP_CELLULAR_RESPONSE_TO_PEPTIDE	GO:1901653	13	-0.241345	-0.749480	0.763636	1.000000	1.000
414	GOBP_CENTRAL_NERVOUS_SYSTEM_NEURON_DIFFERENTIA...	GO:0021953	5	-0.331574	-0.750266	0.812312	1.000000	1.000
415	REACTOME_VESICLE_MEDIATED_TRANSPORT	R-RNO-5653656	5	-0.327005	-0.752737	0.788344	1.000000	1.000
416	REACTOME_EXTRACELLULAR_MATRIX_ORGANIZATION	R-RNO-1474244	5	-0.335918	-0.753219	0.803077	1.000000	1.000
417	REACTOME_SIGNALING_BY_RECEPTOR_TYROSINE_KINASES	R-RNO-9006934	26	-0.204665	-0.755485	0.774667	1.000000	1.000
418	REACTOME_MEMBRANE_TRAFFICKING	R-RNO-199991	5	-0.327005	-0.755781	0.770739	1.000000	1.000
419	REACTOME_SIGNALING_BY_RHO_GTPASES_MIRO_GTPASES...	R-RNO-9716542	9	-0.274540	-0.756500	0.782923	1.000000	1.000
420	GOBP_GAMETE_GENERATION	GO:0007276	9	-0.273948	-0.759876	0.762663	1.000000	1.000
421	GOBP_POSITIVE_REGULATION_OF_CATION_TRANSMEMBRA...	GO:1904064	5	-0.333826	-0.760241	0.781011	1.000000	1.000
422	REACTOME_RHO_GTPASE_EFFECTORS	R-RNO-195258	9	-0.274540	-0.761089	0.771852	1.000000	1.000
423	GOBP_LIPID_EXPORT_FROM_CELL	GO:0140353	5	-0.334415	-0.762467	0.780635	1.000000	1.000
424	GOBP_ORGANIC_ANION_TRANSPORT	GO:0015711	6	-0.323244	-0.765635	0.753304	1.000000	1.000
425	GOBP_SYNAPSE_ORGANIZATION	GO:0050808	17	-0.230488	-0.768532	0.769863	1.000000	1.000
426	GOBP_PEPTIDYL_AMINO_ACID_MODIFICATION	GO:0018193	22	-0.220449	-0.769306	0.791281	1.000000	1.000
427	GOBP_APOPTOTIC_PROCESS	GO:0006915	35	-0.200042	-0.771202	0.760870	1.000000	1.000

Continuation of Table **S5**

	NAME	ID	SIZE	ES	NES	NOM.p.val	FDR.q.val	FWER.p.val
428	GOBP_REGULATION_OF_CELL_CYCLE_PROCESS	GO:0010564	5	-0.339377	-0.771893	0.780089	1.000000	1.000
429	GOBP_POSITIVE_REGULATION_OF_TRANSFERASE_ACTIVITY	GO:0051347	19	-0.225122	-0.773648	0.768340	1.000000	1.000
430	GOBP_ESTABLISHMENT_OF_PROTEIN_LOCALIZATION_TO_...	GO:0072594	5	-0.338558	-0.776894	0.761610	1.000000	1.000
431	GOBP_AMINO_ACID_TRANSPORT	GO:0006865	6	-0.323244	-0.782134	0.766667	1.000000	1.000
432	GOBP_REGULATION_OF_LIPID_METABOLIC_PROCESS	GO:0019216	7	-0.307048	-0.782303	0.734540	1.000000	1.000
433	GOBP_RESPIRATORY_SYSTEM_DEVELOPMENT	GO:0060541	6	-0.320348	-0.782767	0.746334	1.000000	1.000
434	GOBP_CIRCADIAN_REGULATION_OF_GENE_EXPRESSION	GO:0032922	5	-0.349398	-0.784006	0.728614	1.000000	1.000
435	GOBP_CARBOXYLIC_ACID_TRANSPORT	GO:0046942	6	-0.323244	-0.793120	0.725637	1.000000	1.000
436	GOBP_CELLULAR_RESPONSE_TO_INORGANIC_SUBSTANCE	GO:0071241	11	-0.266601	-0.793193	0.747475	1.000000	1.000
437	GOBP_REGULATION_OF_TRANSMEMBRANE_TRANSPORT	GO:0034762	23	-0.217441	-0.793702	0.730220	1.000000	1.000
438	GOBP_REGULATION_OF_PROTEIN_LOCALIZATION_TO_MEM...	GO:1905475	7	-0.312449	-0.796260	0.731118	1.000000	1.000
439	GOBP_REGULATION_OF_RESPONSE_TO_STRESS	GO:0080134	19	-0.234215	-0.796425	0.733424	1.000000	1.000
440	GOBP_POSITIVE_REGULATION_OF_MOLECULAR_FUNCTION	GO:0044093	37	-0.206058	-0.797471	0.737288	1.000000	1.000
441	GOBP_BIOLOGICAL_ADHESION	GO:0022610	19	-0.236392	-0.803917	0.728972	1.000000	1.000
442	REACTOME_LEISHMANIA_INFECTION	R-HSA-9658195	10	-0.278933	-0.804883	0.691069	1.000000	1.000
443	GOBP_REGULATION_OF_CELL_ADHESION	GO:0030155	13	-0.258851	-0.805770	0.714882	1.000000	1.000
444	GOBP_SENSORY_ORGAN_DEVELOPMENT	GO:0007423	8	-0.307285	-0.807310	0.715318	1.000000	1.000
445	GOBP_MULTICELLULAR_ORGANISMAL_HOMEOSTASIS	GO:0048871	12	-0.264123	-0.808954	0.723202	1.000000	1.000
446	GOBP_REGULATION_OF_RESPONSE_TO_EXTERNAL_STIMULUS	GO:0032101	15	-0.254428	-0.821245	0.700134	1.000000	1.000
447	GOBP_IMMUNE_EFFECTOR_PROCESS	GO:0002252	11	-0.281436	-0.822938	0.686301	1.000000	1.000
448	GOBP_RESPONSE_TO_MONOSACCHARIDE	GO:0034284	5	-0.365476	-0.823316	0.705710	1.000000	1.000
449	GOBP_RHYTHMIC_PROCESS	GO:0048511	15	-0.257778	-0.826658	0.693570	1.000000	1.000
450	GOBP_TELENCEPHALON_DEVELOPMENT	GO:0021537	7	-0.321181	-0.827266	0.679882	1.000000	1.000
451	REACTOME_PLATELET_ACTIVATION_SIGNALING_AND_AGG...	R-HSA-76002	7	-0.317564	-0.827559	0.678211	1.000000	1.000
452	GOBP_REGULATION_OF_IMMUNE_SYSTEM_PROCESS	GO:0002682	21	-0.234910	-0.827626	0.684140	1.000000	1.000
453	GOBP_NEURON_DEATH	GO:0070997	18	-0.239845	-0.829061	0.687500	1.000000	1.000
454	GOBP_CELL_MIGRATION	GO:0016477	22	-0.238468	-0.829568	0.691250	1.000000	1.000
455	GOBP_REGULATION_OF_CELL_DEATH	GO:0010941	34	-0.215446	-0.829630	0.695980	1.000000	1.000
456	GOBP_STRIATED_MUSCLE_CELL_DIFFERENTIATION	GO:0051146	9	-0.304984	-0.830258	0.681223	1.000000	1.000
457	GOBP_RESPONSE_TO_CARBOHYDRATE	GO:0009743	5	-0.365476	-0.833841	0.708649	1.000000	1.000
458	GOBP_TUBE_MORPHOGENESIS	GO:0035239	15	-0.256775	-0.838581	0.658311	1.000000	1.000
459	GOBP_CARBOHYDRATE_HOMEOSTASIS	GO:0033500	7	-0.332355	-0.850939	0.660920	1.000000	1.000
460	GOBP_TISSUE_MIGRATION	GO:0090130	9	-0.309178	-0.851428	0.661383	1.000000	1.000
461	GOBP_MITOTIC_CELL_CYCLE	GO:0000278	10	-0.296632	-0.852302	0.649275	1.000000	1.000
462	REACTOME_CIRCADIAN_CLOCK	R-HSA-400253	5	-0.373494	-0.856573	0.635682	1.000000	1.000
463	GOBP_REGULATION_OF_CATABOLIC_PROCESS	GO:0009894	13	-0.274172	-0.856708	0.641407	1.000000	1.000
464	GOBP_CELL_JUNCTION_ORGANIZATION	GO:0034330	19	-0.249545	-0.856859	0.646277	1.000000	1.000
465	GOBP_REGULATION_OF_ANATOMICAL_STRUCTURE_MORPHO...	GO:0022603	17	-0.263149	-0.860661	0.643048	1.000000	1.000
466	GOBP_CELL_CELL_ADHESION	GO:0098609	14	-0.274765	-0.866736	0.635628	1.000000	1.000
467	GOBP_CELLULAR_RESPONSE_TO_HORMONE_STIMULUS	GO:0032870	11	-0.290648	-0.872792	0.627143	1.000000	1.000
468	GOBP_BLOOD_VESSEL_MORPHOGENESIS	GO:0048514	14	-0.279892	-0.872879	0.643855	1.000000	1.000
469	GOBP_REGULATION_OF_NEURONAL_SYNAPTIC_PLASTICITY	GO:0048168	7	-0.338662	-0.874521	0.615942	1.000000	1.000
470	GOBP_NEGATIVE_REGULATION_OF_CELL_PROJECTION_OR...	GO:0031345	5	-0.383003	-0.875758	0.629179	1.000000	1.000

Continuation of Table **S5**

	NAME	ID	SIZE	ES	NES	NOM.p.val	FDR.q.val	FWER.p.val
471	GOBP_POSITIVE_REGULATION_OF_IMMUNE_SYSTEM_PROCESS	GO:0002684	18	-0.258453	-0.879661	0.632708	1.000000	1.000
472	GOBP_REGULATION_OF_WNT_SIGNALING_PATHWAY	GO:0030111	5	-0.395471	-0.884154	0.610687	1.000000	1.000
473	GOBP_REGULATION_OF_MAP_KINASE_ACTIVITY	GO:0043405	9	-0.322160	-0.884190	0.598582	1.000000	1.000
474	GOBP_PEPTIDYL_LYSINE_MODIFICATION	GO:0018205	5	-0.391495	-0.888295	0.643312	1.000000	1.000
475	GOBP_SEXUAL_REPRODUCTION	GO:0019953	10	-0.314355	-0.891089	0.590071	1.000000	1.000
476	GOBP_CALCIUM_MEDIATED_SIGNALING	GO:0019722	10	-0.315934	-0.891198	0.611501	1.000000	1.000
477	GOBP_REGULATION_OF_CELLULAR_LOCALIZATION	GO:0060341	17	-0.274508	-0.894899	0.609986	1.000000	1.000
478	GOBP_CIRCADIAN_RHYTHM	GO:0007623	13	-0.295592	-0.905154	0.577291	0.996750	1.000
479	GOBP_POSITIVE_REGULATION_OF_MAPK_CASCADE	GO:0043410	9	-0.325948	-0.907033	0.587798	0.996614	1.000
480	GOBP_REGULATION_OF_CELL_ACTIVATION	GO:0050865	8	-0.340617	-0.908737	0.578717	0.996946	1.000
481	GOBP_EPITHELIAL_CELL_PROLIFERATION	GO:0050673	12	-0.301305	-0.909481	0.596369	0.999249	1.000
482	GOBP_POSITIVE_REGULATION_OF_PROTEIN_TYROSINE_K...	GO:0061098	5	-0.403548	-0.911582	0.591528	0.998459	1.000
483	GOBP_REGULATION_OF_PROTEIN_TYROSINE_KINASE_ACT...	GO:0061097	5	-0.403548	-0.915351	0.560676	0.993935	1.000
484	GOBP_REGULATION_OF_MUSCLE_SYSTEM_PROCESS	GO:0090257	8	-0.347726	-0.915399	0.568421	0.997964	1.000
485	GOBP_POSITIVE_REGULATION_OF_INTRACELLULAR_SIGN...	GO:1902533	14	-0.294460	-0.916403	0.574555	0.999835	1.000
486	GOBP_LYMPHOCYTE_ACTIVATION	GO:0046649	10	-0.323123	-0.930313	0.552478	0.972004	1.000
487	GOBP_CIRCULATORY_SYSTEM_DEVELOPMENT	GO:0072359	21	-0.262962	-0.933827	0.540880	0.967834	1.000
488	GOBP_REGULATION_OF_CIRCADIAN_RHYTHM	GO:0042752	7	-0.373184	-0.934106	0.554252	0.971330	1.000
489	GOBP_PROTEIN_PHOSPHORYLATION	GO:0006468	33	-0.242364	-0.935852	0.553506	0.971579	1.000
490	GOBP_CELLULAR_MACROMOLECULE_CATABOLIC_PROCESS	GO:0044265	5	-0.417827	-0.940497	0.559091	0.964810	1.000
491	GOBP_CELL_CELL_JUNCTION_ORGANIZATION	GO:0045216	6	-0.395485	-0.940940	0.538020	0.967831	1.000
492	GOBP_PROTEIN_LOCALIZATION_TO_CELL_PERIPHERY	GO:1990778	12	-0.314197	-0.941910	0.542974	0.969608	1.000
493	GOBP_PROTEIN_MODIFICATION_BY_SMALL_PROTEIN_CON...	GO:0032446	6	-0.389379	-0.943226	0.554723	0.970605	1.000
494	GOBP_REGULATION_OF_NEURON_PROJECTION_DEVELOPMENT	GO:0010975	16	-0.282208	-0.944588	0.521439	0.971559	1.000
495	GOBP_CENTRAL_NERVOUS_SYSTEM_DEVELOPMENT	GO:0007417	27	-0.256103	-0.944771	0.550576	0.975309	1.000
496	GOBP_REGULATION_OF_ENDOCYTOSIS	GO:0030100	6	-0.398807	-0.946638	0.568862	0.975089	1.000
497	GOBP_AXON_DEVELOPMENT	GO:0061564	18	-0.283046	-0.950842	0.543276	0.969301	1.000
498	GOBP_POSITIVE_REGULATION_OF_PROTEIN_METABOLIC_...	GO:0051247	27	-0.259447	-0.952026	0.543004	0.970694	1.000
499	GOBP_SPROUTING_ANGIOGENESIS	GO:0002040	5	-0.420122	-0.955045	0.542768	0.967700	1.000
500	GOBP_PURINE_CONTAINING_COMPOUND_METABOLIC_PROCESS	GO:0072521	6	-0.386103	-0.957643	0.537267	0.965856	1.000
501	GOBP_REGULATION_OF_BODY_FLUID_LEVELS	GO:0050878	11	-0.326068	-0.959936	0.520115	0.964429	1.000
502	GOBP_EXOCYTOSIS	GO:0006887	11	-0.321785	-0.961342	0.516173	0.965223	1.000
503	GOBP_PROTEIN_LOCALIZATION_TO_CELL_JUNCTION	GO:1902414	6	-0.402573	-0.971952	0.500768	0.943795	1.000
504	GOBP_REGULATION_OF_SYNAPSE_STRUCTURE_OR_ACTIVITY	GO:0050803	11	-0.330094	-0.972336	0.487004	0.947079	1.000
505	GOBP_MALE_GAMETE_GENERATION	GO:0048232	5	-0.426195	-0.972425	0.513057	0.951202	1.000
506	GOBP_AMEBOIDAL_TYPE_CELL_MIGRATION	GO:0001667	11	-0.327019	-0.972450	0.503516	0.955485	1.000
507	GOBP_FOREBRAIN_DEVELOPMENT	GO:0030900	10	-0.344674	-0.975063	0.516499	0.953487	1.000
508	GOBP_REGULATION_OF_MAPK_CASCADE	GO:0043408	13	-0.321373	-0.977187	0.480716	0.952786	1.000
509	GOBP_POSITIVE_REGULATION_OF_TRANSPORT	GO:0051050	26	-0.266585	-0.983980	0.498084	0.940118	1.000
510	GOBP_GLIAL_CELL_DIFFERENTIATION	GO:0010001	10	-0.346183	-0.986430	0.459459	0.938326	1.000
511	GOBP_RESPONSE_TO_ALCOHOL	GO:0097305	11	-0.343565	-0.989141	0.493671	0.936128	1.000
512	GOBP_NEURON_PROJECTION_GUIDANCE	GO:0097485	9	-0.353126	-0.990130	0.462774	0.938104	1.000
513	REACTOME_ONCOGENIC_MAPK_SIGNALING	R-HSA-6802957	5	-0.440195	-0.996555	0.476831	0.926206	1.000

Continuation of Table **S5**

	NAME	ID	SIZE	ES	NES	NOM.p.val	FDR.q.val	FWER.p.val
514	REACTOME_DEVELOPMENTAL_BIOLOGY	R-RNO-1266738	22	-0.281109	-0.998516	0.476821	0.925710	1.000
515	GOBP_ENDOTHELIAL_CELL_MIGRATION	GO:0043542	8	-0.377832	-1.004041	0.476744	0.916032	1.000
516	GOBP_REGULATION_OF_VASCULATURE_DEVELOPMENT	GO:1901342	5	-0.449891	-1.004316	0.425150	0.919721	1.000
517	GOBP_PROTEIN_LOCALIZATION_TO_MEMBRANE	GO:0072657	13	-0.323366	-1.004966	0.464888	0.922488	1.000
518	GOBP_INTRACELLULAR_TRANSPORT	GO:0046907	10	-0.358783	-1.007695	0.448611	0.920180	1.000
519	GOBP_CELLULAR_COMPONENT_MAINTENANCE	GO:0043954	5	-0.433462	-1.007782	0.446617	0.924432	1.000
520	GOBP_MUSCLE_ORGAN_DEVELOPMENT	GO:0007517	9	-0.378375	-1.009409	0.479315	0.924728	1.000
521	GOBP_MUSCLE_SYSTEM_PROCESS	GO:0003012	9	-0.366025	-1.011933	0.449422	0.922697	1.000
522	GOBP_REGULATION_OF_HEMOPOIESIS	GO:1903706	10	-0.360966	-1.019880	0.421127	0.906657	1.000
523	GOBP_BLOOD_VESSEL_ENDOTHELIAL_CELL_MIGRATION	GO:0043534	8	-0.377832	-1.024280	0.443299	0.900035	1.000
524	GOBP_REGULATION_OF_PEPTIDE_SECRETION	GO:0002791	7	-0.395011	-1.025774	0.433934	0.900871	1.000
525	GOBP_POSITIVE_REGULATION_OF_HEMOPOIESIS	GO:1903708	7	-0.410507	-1.030707	0.411504	0.892795	1.000
526	GOBP_EPITHELIAL_CELL_DIFFERENTIATION	GO:0030855	7	-0.400149	-1.035708	0.418262	0.884234	1.000
527	GOBP_NUCLEAR_TRANSPORT	GO:0051169	5	-0.460295	-1.035780	0.429688	0.888527	1.000
528	GOBP_REGULATION_OF_LEUKOCYTE_DIFFERENTIATION	GO:1902105	9	-0.371039	-1.036228	0.435821	0.891812	1.000
529	GOBP_LONG_TERM_MEMORY	GO:0007616	6	-0.422084	-1.036466	0.428788	0.895810	1.000
530	GOBP_POSITIVE_REGULATION_OF_NEURON_PROJECTION_...	GO:0010976	5	-0.451417	-1.036468	0.431962	0.900404	1.000
531	GOBP_ORGANOPHOSPHATE_METABOLIC_PROCESS	GO:0019637	8	-0.385531	-1.036598	0.442136	0.904717	1.000
532	GOBP_TEMPERATURE_HOMEOSTASIS	GO:0001659	5	-0.455909	-1.037162	0.429675	0.907901	1.000
533	GOBP_CELLULAR_RESPONSE_TO_PEPTIDE_HORMONE_STIM...	GO:0071375	9	-0.377563	-1.038800	0.418571	0.908253	1.000
534	GOBP_CARBOHYDRATE_DERIVATIVE_BIOSYNTHETIC_PROCESS	GO:1901137	5	-0.457765	-1.045399	0.426357	0.895250	1.000
535	GOBP_NUCLEOBASE_CONTAINING_SMALL_MOLECULE_META...	GO:0055086	7	-0.415064	-1.049634	0.425339	0.888533	1.000
536	GOBP_RESPONSE_TO_NICOTINE	GO:0035094	7	-0.407623	-1.049682	0.397644	0.893095	1.000
537	GOBP_TUBE_DEVELOPMENT	GO:0035295	18	-0.310344	-1.051992	0.390789	0.891598	1.000
538	GOBP_MYELOID_LEUKOCYTE_ACTIVATION	GO:0002274	8	-0.389150	-1.053754	0.403400	0.891538	1.000
539	GOBP_MYELOID_LEUKOCYTE_MEDIATED_IMMUNITY	GO:0002444	6	-0.445698	-1.054499	0.426829	0.894422	1.000
540	REACTOME_G_ALPHA_Q_SIGNALLING_EVENTS	R-RNO-416476	6	-0.439024	-1.056116	0.405286	0.894825	1.000
541	GOBP_CELLULAR_MACROMOLECULE_LOCALIZATION	GO:0070727	20	-0.305042	-1.056512	0.395532	0.898573	1.000
542	GOBP_MEMORY	GO:0007613	13	-0.338720	-1.057839	0.393243	0.899976	1.000
543	GOBP_TRANSMEMBRANE_TRANSPORT	GO:0055085	27	-0.286747	-1.060515	0.377432	0.897526	1.000
544	GOBP_RESPONSE_TO_PEPTIDE_HORMONE	GO:0043434	13	-0.338051	-1.061078	0.396957	0.900942	1.000
545	GOBP_POSITIVE_REGULATION_OF_SIGNALING	GO:0023056	34	-0.277169	-1.063749	0.384324	0.898211	1.000
546	GOBP_REGULATION_OF_LONG_TERM_SYNAPTIC_POTENTIA...	GO:1900271	6	-0.448094	-1.064461	0.391502	0.901144	1.000
547	REACTOME_CLASS_C_3_METABOTROPIC_GLUTAMATE_PHER...	R-RNO-420499	7	-0.422220	-1.069713	0.386724	0.891695	1.000
548	GOBP_LEUKOCYTE_MEDIATED_IMMUNITY	GO:0002443	8	-0.399168	-1.073443	0.377504	0.886323	1.000
549	GOBP_CELL_ACTIVATION_INVOLVED_IN_IMMUNE_RESPONSE	GO:0002263	6	-0.445698	-1.075784	0.395173	0.884776	1.000
550	GOBP_RESPONSE_TO_HEAT	GO:0009408	7	-0.424892	-1.077888	0.387240	0.883886	1.000
551	GOBP_MAPK_CASCADE	GO:0000165	18	-0.320411	-1.078210	0.383091	0.888087	1.000
552	GOBP_NEGATIVE_REGULATION_OF_SYNAPTIC_TRANSMISSION	GO:0050805	6	-0.451107	-1.079409	0.379573	0.889906	1.000
553	GOBP_COGNITION	GO:0050890	28	-0.292021	-1.079725	0.375813	0.894142	1.000
554	GOBP_VASCULATURE_DEVELOPMENT	GO:0001944	17	-0.321373	-1.080094	0.358090	0.898204	1.000
555	REACTOME_CELL_CYCLE	R-RNO-1640170	6	-0.458818	-1.093210	0.372488	0.866146	1.000
556	REACTOME_NEUTROPHIL_DEGRANULATION	R-RNO-6798695	6	-0.445698	-1.096194	0.373457	0.862775	1.000

Continuation of Table **S5**

	NAME	ID	SIZE	ES	NES	NOM.p.val	FDR.q.val	FWER.p.val
557	GOBP_REGULATION_OF_PROTEIN_LOCALIZATION	GO:0032880	18	-0.323209	-1.096852	0.344737	0.866107	1.000
558	GOBP_NEURON_DIFFERENTIATION	GO:0030182	37	-0.287265	-1.101198	0.342172	0.858856	1.000
559	REACTOME_ANTI_INFLAMMATORY_RESPONSE_FAVOURING_...	R-HSA-9662851	5	-0.485363	-1.102138	0.328149	0.861456	1.000
560	GOBP_POSITIVE_REGULATION_OF_CATALYTIC_ACTIVITY	GO:0043085	29	-0.302889	-1.110952	0.328680	0.841565	1.000
561	GOBP_REGULATION_OF_CELL_PROJECTION_ORGANIZATION	GO:0031344	20	-0.323391	-1.111686	0.326746	0.844505	1.000
562	GOBP_REGULATION_OF_SYSTEM_PROCESS	GO:0044057	18	-0.333316	-1.114271	0.322709	0.842303	1.000
563	GOBP_REGULATION_OF_PEPTIDE_TRANSPORT	GO:0090087	10	-0.385517	-1.116225	0.327485	0.842044	1.000
564	GOBP_GLIOGENESIS	GO:0042063	12	-0.373068	-1.116467	0.332402	0.846537	1.000
565	GOBP_INNATE_IMMUNE_RESPONSE	GO:0045087	7	-0.438375	-1.119579	0.325000	0.842647	1.000
566	GOBP_REGULATION_OF_TRANSPORTER_ACTIVITY	GO:0032409	17	-0.341909	-1.128813	0.322537	0.821601	1.000
567	GOBP_G_PROTEIN_COUPLED_GLUTAMATE_RECEPTOR_SIGN...	GO:0007216	8	-0.427473	-1.129670	0.321908	0.824357	1.000
568	GOBP_ENDOTHELIAL_CELL_PROLIFERATION	GO:0001935	8	-0.433202	-1.135442	0.313146	0.813446	1.000
569	GOBP_CELL_PROJECTION_ORGANIZATION	GO:0030030	35	-0.296628	-1.136528	0.319899	0.815514	1.000
570	GOBP_REGULATION_OF_CELLULAR_RESPONSE_TO_STRESS	GO:0080135	9	-0.421220	-1.137814	0.298172	0.817192	1.000
571	GOBP_CELL_ACTIVATION	GO:0001775	22	-0.321017	-1.139430	0.302597	0.817760	1.000
572	GOBP_REGULATION_OF_RECEPTOR_MEDIATED_ENDOCYTOSIS	GO:0048259	5	-0.510853	-1.146482	0.297297	0.802818	1.000
573	REACTOME_CELLULAR_RESPONSES_TO_EXTERNAL_STIMULI	R-RNO-8953897	11	-0.387303	-1.146555	0.283262	0.807904	1.000
574	GOBP_POSITIVE_REGULATION_OF_LONG_TERM_SYNAPTIC...	GO:1900273	5	-0.506024	-1.152335	0.281972	0.796545	1.000
575	GOBP_MEMBRANE_ORGANIZATION	GO:0061024	12	-0.374581	-1.153745	0.302452	0.797875	1.000
576	GOBP_RESPONSE_TO_INTERLEUKIN_1	GO:0070555	5	-0.500691	-1.157027	0.296012	0.793536	1.000
577	GOBP_NERVOUS_SYSTEM_PROCESS	GO:0050877	38	-0.301856	-1.158764	0.307039	0.793719	1.000
578	GOBP_NEURON_PROJECTION_ORGANIZATION	GO:0106027	8	-0.439719	-1.159113	0.285094	0.798021	1.000
579	GOBP_DENDRITIC_SPINE_MORPHOGENESIS	GO:0060997	5	-0.519606	-1.160395	0.254870	0.799598	1.000
580	REACTOME_PTEN_REGULATION	R-RNO-6807070	5	-0.513039	-1.163592	0.255952	0.795878	1.000
581	REACTOME_INTERFERON_SIGNALING	R-RNO-913531	6	-0.492042	-1.168554	0.274074	0.786909	1.000
582	GOBP_CELLULAR_COMPONENT_MORPHOGENESIS	GO:0032989	25	-0.326232	-1.175816	0.264474	0.771542	1.000
583	GOBP_MUSCLE_ADAPTATION	GO:0043500	5	-0.518072	-1.177493	0.248109	0.772226	1.000
584	GOBP_HEAD_DEVELOPMENT	GO:0060322	23	-0.335156	-1.178667	0.267097	0.774213	1.000
585	GOBP_REGULATION_OF_MUSCLE_ADAPTATION	GO:0043502	5	-0.518072	-1.185143	0.254237	0.760817	1.000
586	GOBP_POSITIVE_REGULATION_OF_RESPONSE_TO_EXTERN...	GO:0032103	9	-0.432910	-1.185663	0.261669	0.764520	1.000
587	GOBP_DEVELOPMENTAL_MATURATION	GO:0021700	5	-0.519427	-1.188422	0.224299	0.761963	1.000
588	GOBP_CELL_MORPHOGENESIS	GO:0000902	25	-0.326232	-1.189264	0.244246	0.765022	1.000
589	GOBP_NEUROGENESIS	GO:0022008	41	-0.307241	-1.195564	0.257538	0.751431	1.000
590	GOBP_RESPONSE_TO_ETHANOL	GO:0045471	5	-0.518072	-1.198313	0.229299	0.749010	1.000
591	GOBP_CELL_PART_MORPHOGENESIS	GO:0032990	25	-0.326232	-1.198929	0.235589	0.752741	1.000
592	GOBP_EXCITATORY_CHEMICAL_SYNAPTIC_TRANSMISSION	GO:0098976	5	-0.530120	-1.200816	0.240250	0.752915	1.000
593	GOBP_ADENYLATE_CYCLASE_INHIBITING_G_PROTEIN_CO...	GO:0007196	5	-0.524630	-1.205367	0.247774	0.745292	1.000
594	GOBP_REGULATION_OF_PEPTIDE_HORMONE_SECRETION	GO:0090276	6	-0.497989	-1.205796	0.251553	0.749738	1.000
595	REACTOME_NERVOUS_SYSTEM_DEVELOPMENT	R-RNO-9675108	14	-0.384974	-1.207922	0.230014	0.749408	1.000
596	REACTOME_DISEASES_OF_SIGNAL_TRANSDUCTION_BY_GR...	R-HSA-5663202	12	-0.400228	-1.208693	0.232782	0.752815	1.000
597	GOBP_BEHAVIOR	GO:0007610	33	-0.313897	-1.209318	0.237009	0.756673	1.000
598	GOBP_INORGANIC_ION_TRANSMEMBRANE_TRANSPORT	GO:0098660	9	-0.437255	-1.215031	0.233094	0.745281	1.000
599	REACTOME_INTEGRATION_OF_ENERGY_METABOLISM	R-RNO-163685	5	-0.542169	-1.215428	0.218126	0.749986	1.000

Continuation of Table **S5**

	NAME	ID	SIZE	ES	NES	NOM.p.val	FDR.q.val	FWER.p.val
600	REACTOME_SIGNALING_BY_WNT	R-RNO-195721	7	-0.472307	-1.217834	0.244344	0.748684	1.000
601	GOBP_ADENYLATE_CYCLASE_INHIBITING_G_PROTEIN_CO...	GO:0007193	9	-0.442603	-1.225106	0.239377	0.733041	1.000
602	GOBP_MYOTUBE_DIFFERENTIATION	GO:0014902	5	-0.549237	-1.225110	0.213636	0.738988	1.000
603	REACTOME_GPCR_LIGAND_BINDING	R-RNO-500792	8	-0.455445	-1.225461	0.215517	0.743886	1.000
604	GOBP_ORGANOPHOSPHATE_BIOSYNTHETIC_PROCESS	GO:0090407	5	-0.542169	-1.226186	0.198767	0.748003	1.000
605	REACTOME_POST_TRANSLATIONAL_PROTEIN_MODIFICATION	R-RNO-597592	7	-0.483870	-1.228279	0.213333	0.747932	1.000
606	GOBP_DENDRITIC_SPINE_DEVELOPMENT	GO:0060996	6	-0.514146	-1.230799	0.188073	0.746690	1.000
607	GOBP_CELL_CELL_SIGNALING_BY_WNT	GO:0198738	7	-0.485064	-1.233844	0.228826	0.743785	1.000
608	GOBP_HOMEOSTATIC_PROCESS	GO:0042592	31	-0.335645	-1.234088	0.209799	0.749493	1.000
609	REACTOME_CELL_CYCLE_MITOTIC	R-RNO-69278	5	-0.526924	-1.234144	0.211161	0.755827	1.000
610	GOBP_SECOND_MESSENGER_MEDIATED_SIGNALING	GO:0019932	16	-0.378673	-1.238355	0.192513	0.749221	1.000
611	GOBP_NEURON_DEVELOPMENT	GO:0048666	34	-0.320395	-1.241070	0.221656	0.746699	1.000
612	GOBP_NUCLEOSIDE_PHOSPHATE_BIOSYNTHETIC_PROCESS	GO:1901293	5	-0.542169	-1.246884	0.174962	0.734593	1.000
613	GOBP_POSITIVE_REGULATION_OF_EPITHELIAL_CELL_PR...	GO:0050679	7	-0.487329	-1.250135	0.193452	0.731198	1.000
614	GOBP_REGULATION_OF_NERVOUS_SYSTEM_PROCESS	GO:0031644	8	-0.467617	-1.250251	0.229412	0.737421	1.000
615	GOBP_POSTSYNAPSE_ORGANIZATION	GO:0099173	10	-0.437228	-1.256209	0.178470	0.725287	1.000
616	GOBP_REGULATION_OF_SYNAPTIC_TRANSMISSION_GLUTA...	GO:0051966	12	-0.420592	-1.265240	0.184319	0.703253	1.000
617	GOBP_ENDOCRINE_SYSTEM_DEVELOPMENT	GO:0035270	6	-0.526971	-1.268268	0.179941	0.701009	1.000
618	GOBP_NEGATIVE_REGULATION_OF_CELL_CYCLE	GO:0045786	5	-0.559949	-1.269146	0.172956	0.704732	1.000
619	GOBP_CYTOSOLIC_CALCIUM_ION_TRANSPORT	GO:0060401	7	-0.498614	-1.270322	0.185907	0.707914	1.000
620	GOBP_CALCIUM_ION_TRANSPORT_INTO_CYTOSOL	GO:0060402	7	-0.498614	-1.272628	0.179910	0.707091	1.000
621	REACTOME_RNA_POLYMERASE_II_TRANSCRIPTION	R-RNO-73857	20	-0.366685	-1.275771	0.174834	0.703971	1.000
622	REACTOME_BETA_CATENIN_INDEPENDENT_WNT_SIGNALING	R-RNO-3858494	5	-0.572590	-1.282820	0.163110	0.688473	1.000
623	REACTOME_G_ALPHA_Z_SIGNALLING_EVENTS	R-RNO-418597	5	-0.561037	-1.292813	0.170370	0.665265	1.000
624	GOBP_PROTEIN_TETRAMERIZATION	GO:0051262	5	-0.566225	-1.295932	0.165877	0.662187	1.000
625	GOBP_CALCIUM_ION_TRANSMEMBRANE_TRANSPORT	GO:0070588	7	-0.498614	-1.301219	0.163447	0.652376	1.000
626	GOBP_PROTEIN_LOCALIZATION_TO_ORGANELLE	GO:0033365	8	-0.480056	-1.301984	0.161337	0.656814	1.000
627	GOBP_REGULATION_OF_VESICLE_MEDIATED_TRANSPORT	GO:0060627	12	-0.428572	-1.304425	0.136620	0.656393	1.000
628	GOBP_PROTEIN_LOCALIZATION_TO_SYNAPSE	GO:0035418	5	-0.576570	-1.305158	0.144578	0.660929	1.000
629	GOBP_LOCOMOTORY_BEHAVIOR	GO:0007626	8	-0.495736	-1.305407	0.152367	0.667054	1.000
630	GOBP_CHEMICAL_HOMEOSTASIS	GO:0048878	23	-0.363951	-1.306546	0.158311	0.670547	1.000
631	GOBP_PROTEIN_LOCALIZATION_TO_POSTSYNAPSE	GO:0062237	5	-0.576570	-1.312490	0.149390	0.659328	1.000
632	GOBP_ENDOMEMBRANE_SYSTEM_ORGANIZATION	GO:0010256	6	-0.559319	-1.325217	0.124615	0.627902	1.000
633	GOBP_REGULATION_OF_NEUROTRANSMITTER_TRANSPORT	GO:0051588	6	-0.548780	-1.327145	0.119084	0.628942	1.000
634	GOBP_REGULATION_OF_POSTSYNAPSE_ORGANIZATION	GO:0099175	6	-0.552741	-1.330497	0.135417	0.626209	1.000
635	GOBP_CELL_MORPHOGENESIS_INVOLVED_IN_NEURON_DIF...	GO:0048667	22	-0.381574	-1.340243	0.136598	0.603951	1.000
636	GOBP_RESPONSE_TO_ALKALOID	GO:0043279	5	-0.588087	-1.347265	0.122388	0.590963	1.000
637	GOBP_REGULATION_OF_HORMONE_LEVELS	GO:0010817	12	-0.461609	-1.351869	0.118384	0.584170	1.000
638	GOBP_LONG_TERM_SYNAPTIC_DEPRESSION	GO:0060292	5	-0.586435	-1.352537	0.125186	0.589058	1.000
639	GOBP_CHEMICAL_SYNAPTIC_TRANSMISSION_POSTSYNAPTIC	GO:0099565	9	-0.484702	-1.352917	0.139130	0.594766	1.000
640	GOBP_CELL_MORPHOGENESIS_INVOLVED_IN_DIFFERENTI...	GO:0000904	22	-0.381574	-1.355580	0.120104	0.594086	1.000
641	GOBP_REGULATION_OF_SIGNALING_RECEPTOR_ACTIVITY	GO:0010469	14	-0.427688	-1.356903	0.132597	0.597431	1.000
642	GOBP_POSITIVE_REGULATION_OF_SYNAPTIC_TRANSMISSION	GO:0050806	15	-0.426335	-1.357025	0.131649	0.604332	1.000

Continuation of Table **S5**

	NAME	ID	SIZE	ES	NES	NOM.p.val	FDR.q.val	FWER.p.val
643	GOBP_PEPTIDE_SECRETION	GO:0002790	9	-0.498935	-1.362063	0.134307	0.596917	1.000
644	REACTOME_MAPK_FAMILY_SIGNALING_CASCADES	R-RNO-5683057	12	-0.457009	-1.364161	0.122392	0.597401	1.000
645	GOBP_REGULATION_OF_METAL_ION_TRANSPORT	GO:0010959	6	-0.560813	-1.364759	0.118280	0.602935	1.000
646	GOBP_PROTEIN_COMPLEX_OLIGOMERIZATION	GO:0051259	7	-0.529892	-1.365265	0.113235	0.608957	1.000
647	REACTOME_NEUREXINS_AND_NEUROLIGINS	R-RNO-6794361	9	-0.482228	-1.366907	0.129080	0.611609	1.000
648	GOBP_REGULATION_OF_HORMONE_SECRETION	GO:0046883	8	-0.511802	-1.372776	0.114202	0.601206	1.000
649	GOBP_SYNAPTIC_TRANSMISSION_GLUTAMATERGIC	GO:0035249	13	-0.443800	-1.376257	0.124823	0.598654	1.000
650	GOBP_SENSORY_PERCEPTION_OF_PAIN	GO:0019233	5	-0.614458	-1.396832	0.087719	0.545051	1.000
651	GOBP_REGULATION_OF_NEUROTRANSMITTER_LEVELS	GO:0001505	7	-0.555556	-1.396885	0.097451	0.552283	1.000
652	GOBP_POSITIVE_REGULATION_OF_SECRETION	GO:0051047	6	-0.574755	-1.403132	0.090504	0.541878	1.000
653	GOBP_NEUROTRANSMITTER_SECRETION	GO:0007269	6	-0.578100	-1.406550	0.098009	0.539330	1.000
654	GOBP_INSULIN_SECRETION	GO:0030073	7	-0.547623	-1.409727	0.086257	0.537210	1.000
655	GOBP_PEPTIDE_HORMONE_SECRETION	GO:0030072	7	-0.547623	-1.411287	0.098820	0.540121	1.000
656	GOBP_CELL_CYCLE_PROCESS	GO:0022402	11	-0.466999	-1.411327	0.090129	0.547817	1.000
657	GOBP_DENDRITE_DEVELOPMENT	GO:0016358	10	-0.497937	-1.412033	0.102011	0.553785	1.000
658	GOBP_VESICLE_MEDIATED_TRANSPORT_IN_SYNAPSE	GO:0099003	7	-0.561300	-1.422867	0.092486	0.529765	1.000
659	GOBP_MULTICELLULAR_ORGANISMAL_RESPONSE_TO_STRESS	GO:0033555	5	-0.638554	-1.427362	0.075806	0.524852	1.000
660	GOBP_NEUROTRANSMITTER_TRANSPORT	GO:0006836	7	-0.555556	-1.430385	0.077827	0.524476	1.000
661	GOBP_G_PROTEIN_COUPLED_RECEPTOR_SIGNALING_PATHWAY	GO:0007186	18	-0.431676	-1.440458	0.093915	0.503061	1.000
662	REACTOME_NEGATIVE_REGULATION_OF_NMDA_RECEPTOR_...	R-HSA-9617324	8	-0.550000	-1.443245	0.070225	0.503757	1.000
663	GOBP_ESTABLISHMENT_OF_PROTEIN_LOCALIZATION	GO:0045184	17	-0.436434	-1.447617	0.081944	0.500468	1.000
664	GOBP_ENDOCYTOSIS	GO:0006897	8	-0.539662	-1.449498	0.072180	0.503044	1.000
665	GOBP_LONG_TERM_SYNAPTIC_POTENTIATION	GO:0060291	12	-0.488332	-1.453689	0.087500	0.499265	1.000
666	GOBP_REGULATION_OF_SYNAPTIC_PLASTICITY	GO:0048167	21	-0.409889	-1.457786	0.077720	0.495812	1.000
667	GOBP_SECRETION	GO:0046903	24	-0.406812	-1.460529	0.072258	0.496319	1.000
668	REACTOME_ASSEMBLY_AND_CELL_SURFACE_PRESENTATIO...	R-RNO-9609736	8	-0.550000	-1.465975	0.065502	0.489039	1.000
669	GOBP_REGULATION_OF_CATION_TRANSMEMBRANE_TRANSPORT	GO:1904062	16	-0.450802	-1.467663	0.073041	0.492839	1.000
670	GOBP_ADENYLATE_CYCLASE_MODULATING_G_PROTEIN_CO...	GO:0007188	11	-0.502115	-1.474389	0.085795	0.482657	1.000
671	GOBP_POSITIVE_REGULATION_OF_ENDOTHELIAL_CELL_P...	GO:0001938	6	-0.609604	-1.475996	0.060278	0.486982	1.000
672	REACTOME_RAS_ACTIVATION_UPON_CA2_INFLUX_THROUG...	R-HSA-442982	6	-0.621951	-1.490594	0.060993	0.454215	1.000
673	GOBP_REGULATION_OF_SECRETION	GO:0051046	14	-0.478993	-1.496572	0.055556	0.446908	1.000
674	GOBP_REGULATION_OF_ION_TRANSPORT	GO:0043269	36	-0.385777	-1.508194	0.071960	0.424216	1.000
675	GOBP_CELL_SURFACE_RECEPTOR_SIGNALING_PATHWAY_I...	GO:1905114	15	-0.484886	-1.510771	0.068120	0.426012	1.000
676	GOBP_REGULATION_OF_NEUROTRANSMITTER_RECEPTOR_A...	GO:0099601	13	-0.498644	-1.512563	0.044568	0.430169	1.000
677	GOBP_HORMONE_TRANSPORT	GO:0009914	9	-0.553517	-1.515678	0.050370	0.430586	1.000
678	GOBP_DENDRITE_MORPHOGENESIS	GO:0048813	7	-0.590681	-1.516216	0.044577	0.438114	1.000
679	GOBP_REGULATION_OF_MEMBRANE_POTENTIAL	GO:0042391	17	-0.472290	-1.526963	0.042216	0.418871	1.000
680	GOBP_IONOTROPIC_GLUTAMATE_RECEPTOR_SIGNALING_P...	GO:0035235	9	-0.559075	-1.530844	0.039187	0.417844	1.000
681	GOBP_CELLULAR_HOMEOSTASIS	GO:0019725	18	-0.461519	-1.539590	0.045392	0.403882	1.000
682	REACTOME_SYNAPTIC_ADHESION_LIKE_MOLECULES	R-RNO-8849932	9	-0.561187	-1.549487	0.042433	0.386947	1.000
683	GOBP_ION_HOMEOSTASIS	GO:0050801	16	-0.474224	-1.568573	0.039630	0.348626	1.000
684	GOBP_CALCIUM_ION_TRANSPORT	GO:0006816	11	-0.539376	-1.581617	0.030683	0.326564	1.000
685	GOBP_RECEPTOR_INTERNALIZATION	GO:0031623	5	-0.701972	-1.584845	0.030792	0.327658	1.000

Continuation of Table **S5**

	NAME	ID	SIZE	ES	NES	NOM.p.val	FDR.q.val	FWER.p.val
686	GOBP_METAL_ION_TRANSPORT	GO:0030001	11	-0.539376	-1.586899	0.037143	0.331651	1.000
687	REACTOME_DAG_AND_IP3_SIGNALING	R-RNO-1489509	8	-0.596431	-1.592211	0.030435	0.327938	1.000
688	GOBP_CATION_TRANSMEMBRANE_TRANSPORT	GO:0098655	18	-0.475133	-1.596057	0.035620	0.327784	1.000
689	REACTOME_PROTEIN_PROTEIN_INTERACTIONS_AT_SYNAPSES	R-RNO-6794362	12	-0.530810	-1.599467	0.032967	0.329218	1.000
690	GOBP_REGULATION_OF_CATION_CHANNEL_ACTIVITY	GO:2001257	14	-0.505156	-1.602820	0.025572	0.330627	1.000
691	GOBP_DIVALENT_INORGANIC_CATION_HOMEOSTASIS	GO:0072507	13	-0.523379	-1.604957	0.031208	0.335585	1.000
692	GOBP_RECEPTOR_MEDIATED_ENDOCYTOSIS	GO:0006898	6	-0.678741	-1.616791	0.018154	0.318780	1.000
693	GOBP_RECEPTOR_METABOLIC_PROCESS	GO:0043112	5	-0.701972	-1.624230	0.022157	0.312061	1.000
694	GOBP_REGULATION_OF_TRANS_SYNAPTIC_SIGNALING	GO:0099177	40	-0.406844	-1.624587	0.019084	0.321569	1.000
695	REACTOME_LONG_TERM_POTENTIATION	R-HSA-9620244	10	-0.574014	-1.626804	0.031944	0.327191	1.000
696	GOBP_REGULATION_OF_CYTOSOLIC_CALCIUM_ION_CONCE...	GO:0051480	13	-0.523379	-1.627263	0.030769	0.337165	1.000
697	GOBP_REGULATION_OF_TRANSPORT	GO:0051049	44	-0.411581	-1.636704	0.018564	0.326304	1.000
698	GOBP_METAL_ION_HOMEOSTASIS	GO:0055065	14	-0.510986	-1.641666	0.025572	0.326857	1.000
699	GOBP_REGULATION_OF_POSTSYNAPTIC_MEMBRANE_POTEN...	GO:0060078	14	-0.521047	-1.648322	0.018031	0.323010	1.000
700	GOBP_TAXIS	GO:0042330	14	-0.530904	-1.664598	0.023546	0.298835	1.000
701	REACTOME_CA_DEPENDENT_EVENTS	R-RNO-111996	8	-0.629874	-1.707873	0.011396	0.223236	0.999
702	GOBP_CELLULAR_ION_HOMEOSTASIS	GO:0006873	14	-0.540987	-1.708884	0.009550	0.231082	0.999
703	REACTOME_CREB1_PHOSPHORYLATION_THROUGH_NMDA_RE...	R-RNO-442742	8	-0.637500	-1.715217	0.014124	0.229466	0.999
704	GOBP_SENSORY_PERCEPTION	GO:0007600	9	-0.645570	-1.746304	0.011765	0.188347	0.996
705	REACTOME_TRANSCRIPTIONAL_REGULATION_BY_MECP2	R-RNO-8986944	7	-0.694299	-1.751326	0.007474	0.190076	0.996
706	REACTOME_OPIOID_SIGNALLING	R-RNO-111885	12	-0.581809	-1.752075	0.008658	0.198705	0.996
707	REACTOME_G_PROTEIN_MEDIATED_EVENTS	R-RNO-112040	9	-0.647307	-1.757173	0.004478	0.200199	0.993
708	GOBP_SIGNAL_RELEASE	GO:0023061	14	-0.564258	-1.782423	0.004213	0.171940	0.975
709	GOBP_REGULATION_OF_NMDA_RECEPTOR_ACTIVITY	GO:2000310	10	-0.632363	-1.791026	0.011577	0.169973	0.969
710	REACTOME_INTRACELLULAR_SIGNALING_BY_SECOND_MES...	R-RNO-9006925	15	-0.555499	-1.805708	0.006739	0.160351	0.952
711	REACTOME_TRAFFICKING_OF_GLUR2_CONTAINING_AMPA_...	R-RNO-416993	8	-0.675187	-1.810510	0.005970	0.165983	0.946
712	GOBP_CATION_TRANSPORT	GO:0006812	25	-0.499575	-1.810595	0.005249	0.178624	0.946
713	GOBP_PROTEIN_CONTAINING_COMPLEX_SUBUNIT_ORGANI...	GO:0043933	15	-0.558366	-1.812063	0.006775	0.191245	0.945
714	GOBP_GLUTAMATE_RECEPTOR_SIGNALING_PATHWAY	GO:0007215	17	-0.544366	-1.819266	0.002677	0.195190	0.931
715	REACTOME_UNBLOCKING_OF_NMDA_RECEPTORS_GLUTAMAT...	R-RNO-438066	12	-0.603834	-1.824378	0.007022	0.203406	0.924
716	REACTOME_SIGNALING_BY_GPCR	R-RNO-372790	22	-0.517946	-1.829147	0.006502	0.215499	0.919
717	GOBP_SYNAPTIC_SIGNALING	GO:0099536	47	-0.482112	-1.862374	0.002436	0.175899	0.821
718	REACTOME_ACTIVATION_OF_NMDA_RECEPTORS_AND_POST...	R-RNO-442755	16	-0.611111	-1.969570	0.000000	0.066520	0.439
719	REACTOME_TRAFFICKING_OF_AMPA_RECEPTORS	R-RNO-399719	11	-0.693509	-2.030433	0.000000	0.038970	0.245
720	REACTOME_G_ALPHA_I_SIGNALLING_EVENTS	R-RNO-418594	18	-0.618072	-2.109749	0.000000	0.016060	0.092
721	GOBP_CELL_CELL_SIGNALING	GO:0007267	56	-0.562372	-2.172927	0.000000	0.009460	0.044
722	REACTOME_NEUROTRANSMITTER_RECEPTORS_AND_POSTSY...	R-RNO-112314	22	-0.635336	-2.238737	0.000000	0.006321	0.023
723	REACTOME_TRANSMISSION_ACROSS_CHEMICAL_SYNAPSES	R-RNO-112315	23	-0.652775	-2.334456	0.000000	0.002479	0.006
724	REACTOME_NEURONAL_SYSTEM	R-RNO-112316	26	-0.634596	-2.356828	0.000000	0.003253	0.004

End of Table

Supplementary Table S6: SC caudal segment early (8 DPL peak) profile GSEA results.

Begin of Table **S6**

	NAME	ID	SIZE	ES	NES	NOM.p.val	FDR.q.val	FWER.p.val
0	REACTOME_INTERLEUKIN_4_AND_INTERLEUKIN_13_SIGN...	R-RNO-6785807	8	0.850000	2.650963	0.000000	0.004133	0.003
1	GOBP_NEGATIVE_REGULATION_OF_PEPTIDASE_ACTIVITY	GO:0010466	6	0.829268	2.291362	0.000000	0.058247	0.082
2	GOBP_NEGATIVE_REGULATION_OF_CYSTEINE_TYPE_ENDO...	GO:2000117	5	0.819277	2.102695	0.003257	0.159675	0.289
3	REACTOME_DEATH_RECEPTOR_SIGNALLING	R-RNO-73887	5	0.843374	2.079119	0.003175	0.142639	0.329
4	GOBP_CYTOKINE_MEDIATED_SIGNALING_PATHWAY	GO:0019221	16	0.477821	2.072472	0.006757	0.121649	0.346
5	GOBP_NEGATIVE_REGULATION_OF_PROTEOLYSIS	GO:0045861	8	0.633652	2.050440	0.009132	0.120438	0.397
6	GOBP_OSTEOBLAST_DIFFERENTIATION	GO:0001649	5	0.798331	1.999637	0.013158	0.140692	0.502
7	GOBP_NEGATIVE_REGULATION_OF_CELL_POPULATION_PR...	GO:0008285	9	0.584956	1.952865	0.018605	0.163842	0.612
8	REACTOME_SIGNALING_BY_INTERLEUKINS	R-RNO-449147	15	0.475514	1.947926	0.000000	0.148248	0.621
9	REACTOME_TRANSCRIPTIONAL_REGULATION_OF_WHITE_A...	R-RNO-381340	6	0.709519	1.937768	0.003745	0.141376	0.645
10	GOBP_POSITIVE_REGULATION_OF_SMOOTH_MUSCLE_CELL...	GO:0048661	5	0.734940	1.884392	0.006431	0.168348	0.737
11	GOBP_REGULATION_OF_ANION_TRANSMEMBRANE_TRANSPORT	GO:1903959	5	0.722892	1.834365	0.014134	0.201620	0.827
12	GOBP_MAINTENANCE_OF_LOCATION	GO:0051235	7	0.604938	1.780696	0.019084	0.243920	0.901
13	GOBP_RESPONSE_TO_CYTOKINE	GO:0034097	23	0.357711	1.722216	0.027027	0.307254	0.952
14	GOBP_NEGATIVE_REGULATION_OF_MOLECULAR_FUNCTION	GO:0044092	24	0.339218	1.686259	0.015267	0.348070	0.973
15	GOBP_APOPTOTIC_SIGNALING_PATHWAY	GO:0097190	15	0.410959	1.684289	0.011696	0.329308	0.974
16	GOBP_PROTEIN_KINASE_B_SIGNALING	GO:0043491	6	0.597561	1.662809	0.032967	0.347823	0.979
17	GOBP_NEGATIVE_REGULATION_OF_APOPTOTIC_SIGNALIN...	GO:2001234	7	0.543210	1.611342	0.034351	0.428348	0.997
18	HALLMARK_APOPTOSIS	M5902	6	0.577499	1.608824	0.033210	0.410407	0.998
19	GOBP_MEMBRANE_PROTEIN_PROTEOLYSIS	GO:0033619	6	0.575726	1.577970	0.053957	0.451800	0.998
20	GOBP_NEGATIVE_REGULATION_OF_HYDROLASE_ACTIVITY	GO:0051346	11	0.433606	1.573022	0.053097	0.441651	0.998
21	GOBP_REGULATION_OF_APOPTOTIC_SIGNALING_PATHWAY	GO:2001233	10	0.448718	1.558977	0.025974	0.450405	0.999
22	GOBP_CARBOHYDRATE_METABOLIC_PROCESS	GO:0005975	9	0.455696	1.549957	0.047170	0.451299	1.000
23	GOBP_REGULATION_OF_PROTEIN_BINDING	GO:0043393	7	0.518518	1.542498	0.074074	0.447695	1.000
24	GOBP_LEUKOCYTE_CELL_CELL_ADHESION	GO:0007159	6	0.548780	1.538624	0.063433	0.436162	1.000
25	REACTOME_CYTOKINE_SIGNALING_IN_IMMUNE_SYSTEM	R-RNO-1280215	21	0.321845	1.531217	0.034014	0.434406	1.000
26	GOBP_INTRINSIC_APOPTOTIC_SIGNALING_PATHWAY	GO:0097193	5	0.590361	1.529637	0.060284	0.421536	1.000
27	GOBP_POSITIVE_REGULATION_OF_SMALL_MOLECULE_MET...	GO:0062013	5	0.590361	1.528533	0.050167	0.408780	1.000
28	GOBP_NEGATIVE_REGULATION_OF_RESPONSE_TO_EXTERN...	GO:0032102	5	0.598862	1.505771	0.062718	0.439577	1.000
29	GOBP_ACTIN_FILAMENT_BASED_PROCESS	GO:0030029	7	0.493827	1.504634	0.077220	0.427695	1.000
30	GOBP_RESPONSE_TO_UV	GO:0009411	5	0.590361	1.500342	0.082143	0.421445	1.000
31	GOBP_NEGATIVE_REGULATION_OF_MULTICELLULAR_ORGA...	GO:0051241	15	0.357820	1.483136	0.061111	0.442787	1.000
32	GOBP_NEGATIVE_REGULATION_OF_PROTEIN_METABOLIC_...	GO:0051248	20	0.304733	1.466783	0.054264	0.462201	1.000
33	GOBP_NEGATIVE_REGULATION_OF_LOCOMOTION	GO:0040013	5	0.583830	1.450100	0.081356	0.483046	1.000
34	GOBP_POSITIVE_REGULATION_OF_GENE_EXPRESSION	GO:0010628	16	0.324126	1.442255	0.055901	0.485561	1.000
35	GOBP_INFLAMMATORY_RESPONSE	GO:0006954	12	0.379522	1.441211	0.060606	0.474136	1.000
36	GOBP_PRI_MIRNA_TRANSCRIPTION_BY_RNA_POLYMERASE_II	GO:0061614	7	0.467450	1.439829	0.072202	0.464377	1.000
37	GOBP_POSITIVE_REGULATION_OF_CELL_CELL_ADHESION	GO:0022409	5	0.542169	1.425610	0.093023	0.484717	1.000
38	GOBP_REGULATION_OF_DEFENSE_RESPONSE	GO:0031347	7	0.468013	1.424753	0.082645	0.473571	1.000
39	GOBP_POSITIVE_REGULATION_OF_AXONOGENESIS	GO:0050772	5	0.555503	1.421564	0.097902	0.468022	1.000
40	GOBP_CELLULAR_RESPONSE_TO_EXTERNAL_STIMULUS	GO:0071496	10	0.396947	1.410026	0.069149	0.479794	1.000

Continuation of Table **S6**

	NAME	ID	SIZE	ES	NES	NOM.p.val	FDR.q.val	FWER.p.val
41	GOBP_POSITIVE_REGULATION_OF_PRI_MIRNA_TRANSCRI...	GO:1902895	7	0.467450	1.409578	0.092593	0.469189	1.000
42	GOBP_PROTEOLYSIS	GO:0006508	17	0.331716	1.409049	0.076923	0.459111	1.000
43	GOBP_POSITIVE_REGULATION_OF_LEUKOCYTE_CELL_CEL...	GO:1903039	5	0.542169	1.406760	0.115523	0.452326	1.000
44	GOBP_POSITIVE_REGULATION_OF_PHOSPHATIDYLINOSIT...	GO:0014068	5	0.554217	1.403875	0.120275	0.448688	1.000
45	GOBP_POSITIVE_REGULATION_OF_BINDING	GO:0051099	6	0.512195	1.388809	0.125000	0.472215	1.000
46	GOBP_REGULATION_OF_INFLAMMATORY_RESPONSE	GO:0050727	7	0.468013	1.387067	0.100746	0.465522	1.000
47	GOBP_REGULATION_OF_BINDING	GO:0051098	11	0.390865	1.366613	0.095000	0.496693	1.000
48	GOBP_REGULATION_OF_CELL_CELL_ADHESION	GO:0022407	9	0.405063	1.357406	0.102564	0.506228	1.000
49	GOBP_OSSIFICATION	GO:0001503	9	0.401897	1.350846	0.120172	0.510587	1.000
50	GOBP_CELLULAR_CARBOHYDRATE_METABOLIC_PROCESS	GO:0044262	5	0.518072	1.349536	0.112281	0.503712	1.000
51	GOBP_POSITIVE_REGULATION_OF_CYTOKINE_PRODUCTION	GO:0001819	7	0.447422	1.346138	0.116379	0.501258	1.000
52	GOBP_POSITIVE_REGULATION_OF_CELL_DIFFERENTIATION	GO:0045597	27	0.257454	1.342514	0.095652	0.498908	1.000
53	GOBP_REGULATION_OF_CARBOHYDRATE_METABOLIC_PROCESS	GO:0006109	5	0.518072	1.342408	0.106618	0.490098	1.000
54	GOBP_DEVELOPMENTAL_CELL_GROWTH	GO:0048588	5	0.530121	1.339137	0.121094	0.487526	1.000
55	GOBP_REGULATION_OF_CARBOHYDRATE_BIOSYNTHETIC_P...	GO:0043255	5	0.518072	1.337963	0.141379	0.481517	1.000
56	GOBP_SMOOTH_MUSCLE_CELL_PROLIFERATION	GO:0048659	6	0.487805	1.337529	0.112727	0.474146	1.000
57	GOBP_GENERATION_OF_PRECURSOR_METABOLITES_AND_E...	GO:0006091	5	0.518072	1.334865	0.127208	0.471398	1.000
58	GOBP_NEGATIVE_REGULATION_OF_CELL_DEATH	GO:0060548	22	0.266423	1.333492	0.098765	0.465792	1.000
59	GOBP_MORPHOGENESIS_OF_AN_EPITHELIUM	GO:0002009	6	0.490067	1.330398	0.136842	0.463996	1.000
60	GOBP_RESPONSE_TO_MOLECULE_OF_BACTERIAL_ORIGIN	GO:0002237	13	0.333675	1.314412	0.139896	0.489334	1.000
61	GOBP_EXTRINSIC_APOPTOTIC_SIGNALING_PATHWAY	GO:0097191	7	0.432099	1.312685	0.150000	0.484675	1.000
62	GOBP_CARBOHYDRATE_BIOSYNTHETIC_PROCESS	GO:0016051	5	0.518072	1.310175	0.169935	0.482389	1.000
63	GOBP_POLYSACCHARIDE_METABOLIC_PROCESS	GO:0005976	5	0.518072	1.292294	0.153846	0.511206	1.000
64	GOBP_RESPONSE_TO_BACTERIUM	GO:0009617	13	0.333675	1.285532	0.146739	0.517268	1.000
65	GOBP_REGULATION_OF_FAT_CELL_DIFFERENTIATION	GO:0045598	6	0.464218	1.285140	0.138182	0.510234	1.000
66	GOBP_REGULATION_OF_CELL_DIFFERENTIATION	GO:0045595	36	0.227301	1.265996	0.086957	0.543145	1.000
67	GOBP_POSITIVE_REGULATION_OF_DEVELOPMENTAL_PROCESS	GO:0051094	32	0.235071	1.263451	0.129032	0.540692	1.000
68	REACTOME_CELLULAR_SENESCENCE	R-RNO-2559583	6	0.453492	1.257207	0.198413	0.545614	1.000
69	REACTOME_SENESCENCE_ASSOCIATED_SECRETORY_PHENO...	R-RNO-2559582	6	0.453492	1.252723	0.172662	0.547414	1.000
70	GOBP_TISSUE_MORPHOGENESIS	GO:0048729	7	0.419753	1.247029	0.166667	0.553925	1.000
71	GOBP_REGULATION_OF_GTPASE_ACTIVITY	GO:0043087	5	0.481928	1.245655	0.194346	0.549151	1.000
72	GOBP_GLUCOSE_METABOLIC_PROCESS	GO:0006006	6	0.439024	1.240985	0.172269	0.551771	1.000
73	GOBP_POSITIVE_REGULATION_OF_DEFENSE_RESPONSE	GO:0031349	5	0.488575	1.238543	0.200000	0.549066	1.000
74	GOBP_MYELOID_CELL_DIFFERENTIATION	GO:0030099	13	0.300670	1.237936	0.149701	0.542896	1.000
75	GOBP_PERIPHERAL_NERVOUS_SYSTEM_DEVELOPMENT	GO:0007422	9	0.355801	1.224405	0.197368	0.564941	1.000
76	GOBP_REGULATION_OF_VASCULAR_ASSOCIATED_SMOOTH_...	GO:1904705	5	0.481928	1.223218	0.185965	0.560542	1.000
77	GOBP_SKIN_DEVELOPMENT	GO:0043588	5	0.491114	1.223002	0.212544	0.554107	1.000
78	GOBP_NEGATIVE_REGULATION_OF_TRANSCRIPTION_BY_R...	GO:0000122	10	0.343307	1.207723	0.188776	0.579909	1.000
79	GOBP_MONOSACCHARIDE_METABOLIC_PROCESS	GO:0005996	6	0.439024	1.205303	0.210526	0.578745	1.000
80	GOBP_POSITIVE_REGULATION_OF_IMMUNE_RESPONSE	GO:0050778	9	0.354107	1.200078	0.232068	0.582966	1.000
81	GOBP_REGULATION_OF_DEVELOPMENTAL_GROWTH	GO:0048638	9	0.352055	1.198242	0.247368	0.580520	1.000
82	GOBP_CELLULAR_RESPONSE_TO_MOLECULE_OF_BACTERIA...	GO:0071219	8	0.376415	1.195760	0.248927	0.577841	1.000
83	GOBP_CELL_POPULATION_PROLIFERATION	GO:0008283	32	0.220748	1.190303	0.157895	0.581657	1.000

Continuation of Table **S6**

	NAME	ID	SIZE	ES	NES	NOM.p.val	FDR.q.val	FWER.p.val
84	GOBP_ACTIVATION_OF_IMMUNE_RESPONSE	GO:0002253	5	0.473733	1.186214	0.237354	0.586076	1.000
85	GOBP_RESPONSE_TO_MECHANICAL_STIMULUS	GO:0009612	6	0.421899	1.180344	0.257143	0.593458	1.000
86	HALLMARK_HYPOXIA	M5891	5	0.464336	1.173914	0.239852	0.600206	1.000
87	GOBP_REGULATION_OF_EXTRINSIC_APOPTOTIC_SIGNALI...	GO:2001236	6	0.426829	1.166786	0.268000	0.609739	1.000
88	GOBP_CELLULAR_RESPONSE_TO_BIOTIC_STIMULUS	GO:0071216	8	0.376415	1.161446	0.259574	0.614536	1.000
89	GOBP_RAS_PROTEIN_SIGNAL_TRANSDUCTION	GO:0007265	5	0.463971	1.155653	0.278317	0.621031	1.000
90	GOBP_TISSUE_HOMEOSTASIS	GO:0001894	5	0.444430	1.154216	0.257840	0.618433	1.000
91	GOBP_REGULATION_OF_LIPID_LOCALIZATION	GO:1905952	5	0.469880	1.150313	0.273927	0.621030	1.000
92	GOBP_DEFENSE_RESPONSE_TO_OTHER_ORGANISM	GO:0098542	9	0.342538	1.144591	0.270642	0.626987	1.000
93	GOBP_POSITIVE_REGULATION_OF_DEVELOPMENTAL_GROWTH	GO:0048639	8	0.360775	1.143424	0.273543	0.622916	1.000
94	GOBP_POSITIVE_REGULATION_OF_BIOSYNTHETIC_PROCESS	GO:0009891	27	0.228326	1.140818	0.258064	0.622328	1.000
95	GOBP_IMMUNE_SYSTEM_DEVELOPMENT	GO:0002520	17	0.262523	1.140214	0.282209	0.617204	1.000
96	HALLMARK_TNFA_SIGNALING_VIA_NFKB	M5890	14	0.278203	1.134372	0.240964	0.624429	1.000
97	GOBP_CARBOHYDRATE_DERIVATIVE_METABOLIC_PROCESS	GO:1901135	8	0.355310	1.134310	0.284091	0.618086	1.000
98	HALLMARK_ALLOGRAFT_REJECTION	M5950	7	0.376773	1.134252	0.259414	0.611968	1.000
99	GOBP_FC_RECEPTOR_SIGNALING_PATHWAY	GO:0038093	8	0.355435	1.129107	0.278481	0.617619	1.000
100	GOBP_REGULATION_OF_IMMUNE_RESPONSE	GO:0050776	12	0.295254	1.121226	0.254902	0.631100	1.000
101	GOBP_NEGATIVE_REGULATION_OF_BINDING	GO:0051100	6	0.410171	1.120548	0.304833	0.626864	1.000
102	REACTOME_ADAPTIVE_IMMUNE_SYSTEM	R-RNO-1280218	9	0.329114	1.118723	0.301653	0.624580	1.000
103	GOBP_CELLULAR_RESPONSE_TO_ORGANIC_CYCLIC_COMPOUND	GO:0071407	13	0.285754	1.118335	0.295000	0.619514	1.000
104	GOBP_IMMUNE_RESPONSE_REGULATING_SIGNALING_PATHWAY	GO:000276	8	0.355435	1.112767	0.308300	0.625656	1.000
105	GOBP_REGULATION_OF_CELL_ADHESION	GO:0030155	13	0.280000	1.102162	0.302326	0.643152	1.000
106	GOBP_CYTOKINE_PRODUCTION	GO:0001816	10	0.312785	1.096172	0.305936	0.652902	1.000
107	GOBP_REGULATION_OF_MULTICELLULAR_ORGANISMAL_DE...	GO:2000026	31	0.202802	1.094705	0.246914	0.651199	1.000
108	GOBP_POSITIVE_REGULATION_OF_NUCLEOBASE_CONTAIN...	GO:0045935	28	0.205451	1.082979	0.327586	0.670642	1.000
109	GOBP_DEVELOPMENTAL_GROWTH	GO:0048589	10	0.305326	1.075151	0.331707	0.683175	1.000
110	GOBP_REGULATION_OF_EPITHELIAL_CELL_MIGRATION	GO:0010632	6	0.390244	1.074346	0.329710	0.678917	1.000
111	GOBP_RESPONSE_TO_NUTRIENT	GO:0007584	5	0.415962	1.068296	0.392593	0.686907	1.000
112	GOBP_REGULATION_OF_ANATOMICAL_STRUCTURE_SIZE	GO:0090066	10	0.310759	1.067914	0.299517	0.681634	1.000
113	GOBP_POSITIVE_REGULATION_OF_APOPTOTIC_SIGNALIN...	GO:2001235	5	0.421687	1.063617	0.389091	0.686629	1.000
114	GOBP_REGULATION_OF_LYMPHOCYTE_ACTIVATION	GO:0051249	5	0.421687	1.060266	0.372694	0.689044	1.000
115	GOBP_POSITIVE_REGULATION_OF_MULTICELLULAR_ORGA...	GO:0051240	32	0.196700	1.055915	0.357143	0.693212	1.000
116	GOBP_OSTEOCLAST_DIFFERENTIATION	GO:0030316	6	0.375883	1.055669	0.356863	0.687825	1.000
117	GOBP_POSITIVE_REGULATION_OF_GROWTH	GO:0045927	9	0.309418	1.052134	0.365546	0.690007	1.000
118	GOBP_REGULATION_OF_NERVOUS_SYSTEM_DEVELOPMENT	GO:0051960	15	0.249200	1.044461	0.412587	0.701368	1.000
119	GOBP_POSITIVE_REGULATION_OF_PEPTIDYL_SERINE_PH...	GO:0033138	8	0.324908	1.041986	0.390438	0.700461	1.000
120	GOBP_REGULATION_OF_PEPTIDYL_SERINE_PHOSPHORYLA...	GO:0033135	8	0.324908	1.040885	0.420601	0.696859	1.000
121	GOBP_CELLULAR_RESPONSE_TO_OXYGEN_CONTAINING_CO...	GO:1901701	29	0.199997	1.021361	0.387097	0.738433	1.000
122	GOBP_REGULATION_OF_MUSCLE_SYSTEM_PROCESS	GO:0090257	8	0.325000	1.021225	0.415638	0.732814	1.000
123	GOBP_GLAND_DEVELOPMENT	GO:0048732	7	0.334236	1.007483	0.422925	0.758781	1.000
124	GOBP_MONONUCLEAR_CELL_DIFFERENTIATION	GO:1903131	7	0.339976	1.005551	0.451883	0.757515	1.000
125	GOBP_INOSITOL_LIPID_MEDIATED_SIGNALING	GO:0048017	8	0.316406	1.002518	0.449393	0.759812	1.000
126	GOBP_REGULATION_OF_NEURON_DIFFERENTIATION	GO:0045664	5	0.392180	1.000727	0.425249	0.757867	1.000

Continuation of Table **S6**

	NAME	ID	SIZE	ES	NES	NOM.p.val	FDR.q.val	FWER.p.val
127	GOBP_REGULATION_OF_PEPTIDYL_TYROSINE_PHOSPHORY...	GO:0050730	9	0.299408	0.998255	0.443925	0.758750	1.000
128	GOBP_ORGAN_GROWTH	GO:0035265	5	0.381101	0.997915	0.474820	0.753571	1.000
129	GOBP_RESPONSE_TO_ENDOGENOUS_STIMULUS	GO:0009719	34	0.183113	0.994952	0.456522	0.754448	1.000
130	GOBP_CELLULAR_RESPONSE_TO_EXTRACELLULAR_STIMULUS	GO:0031668	8	0.310091	0.993695	0.453782	0.751206	1.000
131	GOBP_POSITIVE_REGULATION_OF_CELL_GROWTH	GO:0030307	6	0.356985	0.991770	0.423077	0.749596	1.000
132	GOBP_NEGATIVE_REGULATION_OF_GENE_EXPRESSION	GO:0010629	11	0.272727	0.990087	0.446078	0.748115	1.000
133	GOBP_REGULATION_OF_ENDOTHELIAL_CELL_MIGRATION	GO:0010594	5	0.385542	0.989230	0.474747	0.744613	1.000
134	GOBP_REGULATION_OF_MITOTIC_CELL_CYCLE	GO:0007346	5	0.385542	0.988099	0.469595	0.741881	1.000
135	GOBP_FAT_CELL_DIFFERENTIATION	GO:0045444	9	0.295664	0.986821	0.430380	0.738878	1.000
136	GOBP_REGULATION_OF_NEUROGENESIS	GO:0050767	14	0.245709	0.983176	0.480874	0.740571	1.000
137	REACTOME_INFECTIOUS_DISEASE	R-HSA-5663205	12	0.263626	0.979187	0.492754	0.743918	1.000
138	GOBP_REGULATION_OF_CELL_DEVELOPMENT	GO:0060284	15	0.239794	0.974873	0.479769	0.747772	1.000
139	GOBP_TISSUE_MIGRATION	GO:0090130	9	0.291139	0.973249	0.438819	0.746696	1.000
140	GOBP_CHROMATIN_ORGANIZATION	GO:0006325	5	0.385542	0.969988	0.468966	0.748869	1.000
141	GOBP_POSITIVE_REGULATION_OF_EPITHELIAL_CELL_MI...	GO:0010634	5	0.385542	0.969252	0.477352	0.745507	1.000
142	GOBP_POSITIVE_REGULATION_OF_TRANSCRIPTION_BY_R...	GO:0045944	24	0.203391	0.965913	0.504000	0.748150	1.000
143	GOBP_MUSCLE_CELL_PROLIFERATION	GO:0033002	8	0.303707	0.963409	0.483254	0.748472	1.000
144	GOBP_T_CELL_ACTIVATION	GO:0042110	8	0.309911	0.961854	0.497817	0.746658	1.000
145	GOBP_REGULATION_OF_ORGAN_GROWTH	GO:0046620	5	0.381101	0.953776	0.489796	0.759051	1.000
146	GOBP_PROTEIN_DEPHOSPHORYLATION	GO:0006470	7	0.320988	0.950271	0.488722	0.761234	1.000
147	GOBP_DEPHOSPHORYLATION	GO:0016311	7	0.320988	0.949025	0.502058	0.758605	1.000
148	GOBP_POSITIVE_REGULATION_OF_CELL_DEVELOPMENT	GO:0010720	12	0.245595	0.944240	0.479592	0.764618	1.000
149	GOBP_RESPONSE_TO_INTERLEUKIN_1	GO:0070555	5	0.368060	0.942450	0.526882	0.763726	1.000
150	GOBP_REGULATION_OF_CELL_SIZE	GO:0008361	5	0.369922	0.937617	0.503472	0.768768	1.000
151	GOBP_NEGATIVE_REGULATION_OF_CATALYTIC_ACTIVITY	GO:0043086	17	0.222880	0.937520	0.595376	0.763879	1.000
152	GOBP_POSITIVE_REGULATION_OF_CELL_ADHESION	GO:0045785	7	0.320988	0.936632	0.520161	0.760919	1.000
153	REACTOME_INNATE_IMMUNE_SYSTEM	R-RNO-168249	16	0.217631	0.936451	0.564706	0.756687	1.000
154	GOBP_POSITIVE_REGULATION_OF_NERVOUS_SYSTEM_DEV...	GO:0051962	12	0.245595	0.923073	0.572770	0.780449	1.000
155	GOBP_BLOOD_VESSEL_ENDOTHELIAL_CELL_MIGRATION	GO:0043534	8	0.287500	0.918606	0.557377	0.784430	1.000
156	GOBP_ENDOTHELIAL_CELL_MIGRATION	GO:0043542	8	0.287500	0.917486	0.533613	0.781576	1.000
157	GOBP_CELL_GROWTH	GO:0016049	10	0.269231	0.916657	0.555556	0.778443	1.000
158	GOBP_POSITIVE_REGULATION_OF_NEUROGENESIS	GO:0050769	12	0.245595	0.916609	0.583756	0.773714	1.000
159	HALLMARK_KRAS_SIGNALING_UP	M5953	6	0.330454	0.916605	0.549296	0.768887	1.000
160	GOBP_ENDOTHELIAL_CELL_PROLIFERATION	GO:0001935	8	0.287500	0.906630	0.611607	0.784658	1.000
161	GOBP_REGULATION_OF_GROWTH	GO:0040008	13	0.233078	0.904369	0.573770	0.784084	1.000
162	GOBP_POSITIVE_REGULATION_OF_CELL_POPULATION_PR...	GO:0008284	21	0.189309	0.902359	0.580645	0.783720	1.000
163	GOBP_LEUKOCYTE_DIFFERENTIATION	GO:0002521	15	0.219425	0.896418	0.618182	0.790412	1.000
164	GOBP_RESPONSE_TO_ORGANIC_CYCLIC_COMPOUND	GO:0014070	20	0.193349	0.893317	0.666667	0.791992	1.000
165	GOBP_NEGATIVE_REGULATION_OF_CELL_DEVELOPMENT	GO:0010721	6	0.320491	0.880101	0.580000	0.814249	1.000
166	GOBP_REGULATION_OF_IMMUNE_SYSTEM_PROCESS	GO:0002682	21	0.182149	0.877930	0.632000	0.814197	1.000
167	GOBP_POSITIVE_REGULATION_OF_PEPTIDYL_TYROSINE_...	GO:0050731	8	0.282237	0.876676	0.621622	0.811832	1.000
168	GOBP_NEGATIVE_REGULATION_OF_NERVOUS_SYSTEM_DEV...	GO:0051961	5	0.335724	0.873836	0.620253	0.812393	1.000
169	GOBP_NEGATIVE_REGULATION_OF_NUCLEOBASE_CONTAIN...	GO:0045934	13	0.213333	0.866599	0.679426	0.821534	1.000

Continuation of Table **S6**

	NAME	ID	SIZE	ES	NES	NOM.p.val	FDR.q.val	FWER.p.val
170	GOBP_INNATE_IMMUNE_RESPONSE	GO:0045087	7	0.289150	0.863453	0.600000	0.823158	1.000
171	GOBP_CELL_CELL_JUNCTION_ASSEMBLY	GO:0007043	5	0.352095	0.859633	0.631399	0.825969	1.000
172	GOBP_REGULATION_OF_DNA_BINDING_TRANSCRIPTION_F...	GO:0051090	16	0.198823	0.851069	0.684211	0.838673	1.000
173	GOBP_POSITIVE_REGULATION_OF_IMMUNE_SYSTEM_PROCESS	GO:0002684	18	0.189856	0.850890	0.685714	0.834365	1.000
174	GOBP_REGULATION_OF_CELLULAR_COMPONENT_SIZE	GO:0032535	7	0.282032	0.848047	0.688000	0.834641	1.000
175	GOBP_EPITHELIAL_CELL_PROLIFERATION	GO:0050673	12	0.215762	0.847740	0.682796	0.830333	1.000
176	GOBP_GROWTH	GO:0040007	14	0.211635	0.846630	0.703911	0.827850	1.000
177	GOBP_NEGATIVE_REGULATION_OF_BIOSYNTHETIC_PROCESS	GO:0009890	13	0.213914	0.846260	0.661290	0.823851	1.000
178	GOBP_IN_UTERO_EMBRYONIC_DEVELOPMENT	GO:0001701	8	0.268618	0.845298	0.669683	0.820888	1.000
179	GOBP_MYELOID_LEUKOCYTE_DIFFERENTIATION	GO:0002573	11	0.222245	0.844695	0.676056	0.817766	1.000
180	GOBP_REGULATION_OF_MYELOID_CELL_DIFFERENTIATION	GO:0045637	9	0.234587	0.837252	0.707424	0.825862	1.000
181	GOBP_PEPTIDE_METABOLIC_PROCESS	GO:0006518	11	0.223825	0.820233	0.730000	0.853486	1.000
182	GOBP_NEUROTROPHIN_TRK_RECEPTOR_SIGNALING_PATHWAY	GO:0048011	6	0.287919	0.811654	0.665427	0.863558	1.000
183	GOBP_POSITIVE_REGULATION_OF_ANION_TRANSPORT	GO:1903793	9	0.243544	0.810648	0.722222	0.860938	1.000
184	GOBP_NEUROTROPHIN_SIGNALING_PATHWAY	GO:0038179	6	0.287919	0.809990	0.691700	0.857589	1.000
185	GOBP_REGULATION_OF_HEMOPOIESIS	GO:1903706	10	0.222658	0.793840	0.781659	0.880720	1.000
186	GOBP_MUSCLE_CONTRACTION	GO:0006936	5	0.301205	0.784896	0.752508	0.891388	1.000
187	GOBP_CELLULAR_GLUCOSE_HOMEOSTASIS	GO:0001678	5	0.313253	0.781790	0.768212	0.891743	1.000
188	GOBP_NEURON_APOPTOTIC_PROCESS	GO:0051402	12	0.202582	0.777287	0.828125	0.894494	1.000
189	GOBP_INTRACELLULAR_RECEPTOR_SIGNALING_PATHWAY	GO:0030522	5	0.297755	0.751532	0.767361	0.932543	1.000
190	GOBP_SPROUTING_ANGIOGENESIS	GO:0002040	5	0.297211	0.751060	0.781250	0.928388	1.000
191	GOBP_POSITIVE_REGULATION_OF_TRANSMEMBRANE_TRAN...	GO:0034764	7	0.254916	0.749835	0.776515	0.925597	1.000
192	GOBP_REGULATION_OF_OSTEOCLAST_DIFFERENTIATION	GO:0045670	5	0.291396	0.748408	0.762590	0.922923	1.000
193	GOBP_NEGATIVE_REGULATION_OF_RESPONSE_TO_STIMULUS	GO:0048585	16	0.173441	0.743742	0.856287	0.924483	1.000
194	GOBP_CELLULAR_AMIDE_METABOLIC_PROCESS	GO:0043603	12	0.194177	0.741730	0.866337	0.922841	1.000
195	GOBP_CELLULAR_RESPONSE_TO_ABIOTIC_STIMULUS	GO:0071214	7	0.240240	0.735904	0.827586	0.926570	1.000
196	GOBP_CELLULAR_RESPONSE_TO_LIPID	GO:0071396	15	0.178087	0.734421	0.905556	0.924255	1.000
197	GOBP_HOMEOSTASIS_OF_NUMBER_OF_CELLS	GO:0048872	5	0.294654	0.725792	0.786260	0.932461	1.000
198	GOBP_PROTEIN_AUTOPHOSPHORYLATION	GO:0046777	5	0.277643	0.718271	0.843416	0.937942	1.000
199	GOBP_RESPONSE_TO_TUMOR_NECROSIS_FACTOR	GO:0034612	7	0.238818	0.710560	0.872587	0.943498	1.000
200	GOBP_CELLULAR_COMPONENT_DISASSEMBLY	GO:0022411	6	0.256098	0.705572	0.843972	0.945202	1.000
201	REACTOME_HEMOSTASIS	R-RNO-109582	14	0.170135	0.696012	0.920455	0.953242	1.000
202	GOBP_EMBRYO_DEVELOPMENT_ENDING_IN_BIRTH_OR_EGG...	GO:0009792	9	0.202628	0.687301	0.876068	0.958925	1.000
203	GOBP_POSITIVE_REGULATION_OF_CELL_PROJECTION_OR...	GO:0031346	10	0.193454	0.685449	0.892523	0.956550	1.000
204	GOBP_CYTOSKELETON_ORGANIZATION	GO:0007010	11	0.183404	0.683791	0.891892	0.953639	1.000
205	GOBP_REGULATION_OF_LEUKOCYTE_DIFFERENTIATION	GO:1902105	9	0.202532	0.677548	0.900000	0.956444	1.000
206	GOBP_PROTEIN_MODIFICATION_BY_SMALL_PROTEIN_CON...	GO:0070647	7	0.222222	0.674722	0.896414	0.955160	1.000
207	GOBP_POSITIVE_REGULATION_OF_DNA_BINDING_TRANSC...	GO:0051091	10	0.184408	0.671541	0.902703	0.954056	1.000
208	GOBP_CELL_CELL_ADHESION	GO:0098609	14	0.172325	0.668169	0.937500	0.953535	1.000
209	REACTOME_NGF_STIMULATED_TRANSCRIPTION	R-RNO-9031628	9	0.202532	0.664117	0.909091	0.953468	1.000
210	GOBP_LYMPHOCYTE_ACTIVATION	GO:0046649	10	0.179889	0.658021	0.935644	0.955209	1.000
211	GOBP_AMYLOID_PRECURSOR_PROTEIN_METABOLIC_PROCESS	GO:0042982	5	0.253012	0.652189	0.910714	0.956217	1.000
212	GOBP_AMYLOID_PRECURSOR_PROTEIN_CATABOLIC_PROCESS	GO:0042987	5	0.253012	0.646092	0.925267	0.957335	1.000

Continuation of Table **S6**

	NAME	ID	SIZE	ES	NES	NOM.p.val	FDR.q.val	FWER.p.val
213	GOBP_AMYLOID_BETA_METABOLIC_PROCESS	GO:0050435	5	0.253012	0.632551	0.928315	0.964982	1.000
214	GOBP_POSITIVE_REGULATION_OF_HEMOPOIESIS	GO:1903708	7	0.197531	0.592621	0.940520	0.988652	1.000
215	GOBP_LONG_TERM_SYNAPTIC_DEPRESSION	GO:0060292	5	0.228916	0.585973	0.962838	0.987705	1.000
216	GOBP_CIRCADIAN_REGULATION_OF_GENE_EXPRESSION	GO:0032922	5	0.216868	0.560846	0.987138	0.994252	1.000
217	GOBP_ANATOMICAL_STRUCTURE_FORMATION_INVOLVED_I...	GO:0048646	20	0.115483	0.553497	0.992537	0.992273	1.000
218	GOBP_RESPONSE_TO_INSULIN	GO:0032868	5	0.216868	0.540678	0.988000	0.991729	1.000
219	GOBP_AMEBOIDAL_TYPE_CELL_MIGRATION	GO:0001667	11	0.121030	0.452301	0.995327	0.999161	1.000
220	GOBP_TUBE_MORPHOGENESIS	GO:0035239	15	-0.132209	-0.405127	0.998767	0.998400	1.000
221	GOBP_REGULATION_OF_RECEPTOR_MEDIATED_ENDOCYTOSIS	GO:0048259	5	-0.194501	-0.430792	0.998615	0.998509	1.000
222	GOBP_PROTEIN_CATABOLIC_PROCESS	GO:0030163	8	-0.173185	-0.447153	0.998706	0.998680	1.000
223	GOBP_BLOOD_VESSEL_MORPHOGENESIS	GO:0048514	14	-0.153097	-0.469870	0.990315	0.997030	1.000
224	GOBP_REGULATION_OF_ENDOCYTOSIS	GO:0030100	6	-0.197535	-0.470647	0.997308	0.998870	1.000
225	GOBP_EPIDERMIS_DEVELOPMENT	GO:0008544	5	-0.209928	-0.470947	0.997319	1.000000	1.000
226	GOBP_RESPONSE_TO_NERVE_GROWTH_FACTOR	GO:1990089	7	-0.198299	-0.488053	0.989218	0.999018	1.000
227	GOBP_NEGATIVE_REGULATION_OF_DEVELOPMENTAL_PROCESS	GO:0051093	13	-0.172485	-0.508365	0.972739	0.995216	1.000
228	GOBP_NEGATIVE_REGULATION_OF_CELL_DIFFERENTIATION	GO:0045596	13	-0.172485	-0.508556	0.980296	0.997156	1.000
229	GOBP_POSITIVE_REGULATION_OF_CELLULAR_COMPONENT...	GO:0044089	9	-0.199390	-0.523379	0.966837	0.993886	1.000
230	GOBP_REGULATION_OF_MYELOID_LEUKOCYTE_DIFFERENT...	GO:0002761	8	-0.204775	-0.530390	0.973958	0.993193	1.000
231	GOBP_NEURON_MIGRATION	GO:0001764	5	-0.240105	-0.531848	0.968792	0.994584	1.000
232	GOBP_REGULATION_OF_CELLULAR_COMPONENT_MOVEMENT	GO:0051270	15	-0.173636	-0.532002	0.969448	0.996552	1.000
233	GOBP_MACROMOLECULE_CATABOLIC_PROCESS	GO:0009057	11	-0.190412	-0.537279	0.968314	0.996250	1.000
234	GOBP_NEGATIVE_REGULATION_OF_INTRACELLULAR_SIGN...	GO:1902532	7	-0.224298	-0.546244	0.969617	0.994337	1.000
235	REACTOME_SIGNALING_BY_RHO_GTPASES_MIRO_GTPASES...	R-RNO-9716542	9	-0.209149	-0.555692	0.969152	0.991834	1.000
236	GOBP_NEGATIVE_REGULATION_OF_NEURON_APOPTOTIC_P...	GO:0043524	8	-0.215662	-0.556955	0.953765	0.993163	1.000
237	REACTOME_RHO_GTPASE_EFFECTORS	R-RNO-195258	9	-0.209149	-0.560037	0.951571	0.993598	1.000
238	GOBP_EMBRYO_DEVELOPMENT	GO:0009790	14	-0.182742	-0.561944	0.963270	0.994562	1.000
239	GOBP_REGULATION_OF_SMALL_MOLECULE_METABOLIC_PR...	GO:0062012	9	-0.214866	-0.565652	0.951507	0.994541	1.000
240	GOBP_PEPTIDYL_LYSINE_MODIFICATION	GO:0018205	5	-0.250422	-0.567693	0.972337	0.995491	1.000
241	GOBP_BIOLOGICAL_PROCESS_INVOLVED_IN_SYMBIOTIC_...	GO:004440	10	-0.204958	-0.571216	0.944862	0.995532	1.000
242	GOBP_POSITIVE_REGULATION_OF_ORGANELLE_ORGANIZA...	GO:0010638	10	-0.209747	-0.574943	0.945083	0.995375	1.000
243	GOBP_RESPONSE_TO_LIPID	GO:0033993	19	-0.177198	-0.586073	0.948538	0.990529	1.000
244	GOBP_REPRODUCTIVE_SYSTEM_DEVELOPMENT	GO:0061458	6	-0.248605	-0.586994	0.962667	0.991971	1.000
245	GOBP_POSITIVE_REGULATION_OF_CELLULAR_COMPONENT...	GO:0051130	24	-0.172410	-0.587822	0.933486	0.993493	1.000
246	GOBP_REGULATION_OF_PROTEIN_CATABOLIC_PROCESS	GO:0042176	7	-0.238198	-0.588718	0.946309	0.995016	1.000
247	GOBP_CHROMOSOME_ORGANIZATION	GO:0051276	6	-0.255983	-0.589788	0.932065	0.996329	1.000
248	GOBP_BIOLOGICAL_ADHESION	GO:0022610	19	-0.183289	-0.592441	0.915677	0.996583	1.000
249	GOBP_PATTERN_SPECIFICATION_PROCESS	GO:0007389	6	-0.252871	-0.593127	0.938005	0.998234	1.000
250	GOBP_REGIONALIZATION	GO:0003002	6	-0.252871	-0.595063	0.938525	0.998943	1.000
251	REACTOME_SIGNALING_BY_RECEPTOR_TYROSINE_KINASES	R-RNO-9006934	26	-0.175447	-0.597356	0.914097	0.999403	1.000
252	GOBP_LIPID_METABOLIC_PROCESS	GO:0006629	10	-0.224734	-0.605889	0.932161	0.995443	1.000
253	GOBP_NEGATIVE_REGULATION_OF_KINASE_ACTIVITY	GO:0033673	5	-0.272896	-0.605905	0.950912	0.997531	1.000
254	GOBP_REGULATION_OF_LIPID_BIOSYNTHETIC_PROCESS	GO:0046890	6	-0.260571	-0.609618	0.936592	0.997014	1.000
255	GOBP_CARBOHYDRATE_HOMEOSTASIS	GO:0033500	7	-0.245852	-0.610074	0.924084	0.998793	1.000

Continuation of Table **S6**

	NAME	ID	SIZE	ES	NES	NOM.p.val	FDR.q.val	FWER.p.val
256	GOBP_NEGATIVE_REGULATION_OF_TRANSFERASE_ACTIVITY	GO:0051348	5	-0.272896	-0.613962	0.932489	0.997866	1.000
257	GOBP_REGULATION_OF_MUSCLE_CELL_DIFFERENTIATION	GO:0051147	6	-0.258661	-0.614355	0.929825	0.999670	1.000
258	GOBP_RESPONSE_TO_KETONE	GO:1901654	7	-0.246649	-0.614562	0.916329	1.000000	1.000
259	GOBP_CELL_CELL_JUNCTION_ORGANIZATION	GO:0045216	6	-0.259315	-0.616435	0.932705	1.000000	1.000
260	GOBP_NEGATIVE_REGULATION_OF_ANION_TRANSPORT	GO:1903792	8	-0.241572	-0.619709	0.915232	1.000000	1.000
261	GOBP_RESPONSE_TO_GROWTH_FACTOR	GO:0070848	18	-0.191183	-0.619863	0.914184	1.000000	1.000
262	GOBP_LOCOMOTION	GO:0040011	31	-0.175239	-0.621669	0.924208	1.000000	1.000
263	HALLMARK_PI3K_AKT_MTOR_SIGNALING	M5923	6	-0.262890	-0.624989	0.937330	1.000000	1.000
264	GOBP_MUSCLE_CELL_DIFFERENTIATION	GO:0042692	10	-0.227547	-0.625683	0.933333	1.000000	1.000
265	GOBP_LIPID_BIOSYNTHETIC_PROCESS	GO:0008610	6	-0.260571	-0.627147	0.917469	1.000000	1.000
266	GOBP_NEGATIVE_REGULATION_OF_PROTEIN_MODIFICATI...	GO:0031400	10	-0.226163	-0.628560	0.920573	1.000000	1.000
267	GOBP_APOPTOTIC_PROCESS	GO:0006915	35	-0.174447	-0.633373	0.908197	1.000000	1.000
268	REACTOME_FC_EPSILON_RECEPTOR_FCERI_SIGNALING	R-RNO-2454202	7	-0.257681	-0.636552	0.910526	1.000000	1.000
269	GOBP_NEUROINFLAMMATORY_RESPONSE	GO:0150076	5	-0.286797	-0.637802	0.925666	1.000000	1.000
270	GOBP_COAGULATION	GO:0050817	8	-0.249033	-0.639136	0.917323	1.000000	1.000
271	GOBP_CELLULAR_LIPID_METABOLIC_PROCESS	GO:0044255	7	-0.264238	-0.642952	0.902145	1.000000	1.000
272	GOBP_REGULATION_OF_VASCULATURE_DEVELOPMENT	GO:1901342	5	-0.287142	-0.643679	0.917630	1.000000	1.000
273	REACTOME_EXTRACELLULAR_MATRIX_ORGANIZATION	R-RNO-1474244	5	-0.287071	-0.645639	0.915849	1.000000	1.000
274	GOBP_NEGATIVE_REGULATION_OF_SIGNALING	GO:0023057	21	-0.193791	-0.648334	0.885514	1.000000	1.000
275	GOBP_REGULATION_OF_NEURON_PROJECTION_DEVELOPMENT	GO:0010975	16	-0.212260	-0.649270	0.885305	1.000000	1.000
276	GOBP_NEGATIVE_REGULATION_OF_NEURON_DEATH	GO:1901215	11	-0.231786	-0.650697	0.885101	1.000000	1.000
277	GOBP_FC_EPSILON_RECEPTOR_SIGNALING_PATHWAY	GO:0038095	7	-0.257681	-0.651753	0.884115	1.000000	1.000
278	GOBP_CELL_MIGRATION	GO:0016477	22	-0.197845	-0.653618	0.882217	1.000000	1.000
279	GOBP_NEGATIVE_REGULATION_OF_ION_TRANSPORT	GO:0043271	11	-0.229115	-0.654249	0.892269	1.000000	1.000
280	GOBP_PHAGOCYTOSIS	GO:0006909	5	-0.293677	-0.655286	0.922644	1.000000	1.000
281	GOBP_POSTTRANSCRIPTIONAL_REGULATION_OF_GENE_EX...	GO:0010608	8	-0.258709	-0.655638	0.887550	1.000000	1.000
282	GOBP_REGULATION_OF_PROTEIN_TYROSINE_KINASE_ACT...	GO:0061097	5	-0.298844	-0.659869	0.891929	1.000000	1.000
283	GOBP_POSITIVE_REGULATION_OF_PROTEIN_TYROSINE_K...	GO:0061098	5	-0.298844	-0.660444	0.891929	1.000000	1.000
284	GOBP_PLATELET_ACTIVATION	GO:0030168	6	-0.279646	-0.661764	0.903533	1.000000	1.000
285	GOBP_POSITIVE_REGULATION_OF_MYELOID_LEUKOCYTE_...	GO:0002763	6	-0.277683	-0.663726	0.897849	1.000000	1.000
286	GOBP_REGULATION_OF_ANATOMICAL_STRUCTURE_MORPHO...	GO:0022603	17	-0.212534	-0.665297	0.880756	1.000000	1.000
287	GOBP_REGULATION_OF_WNT_SIGNALING_PATHWAY	GO:0030111	5	-0.301835	-0.670719	0.882603	1.000000	1.000
288	REACTOME_NUCLEAR_EVENTS_KINASE_AND_TRANSCRIPTI...	R-RNO-198725	11	-0.238645	-0.673050	0.884712	1.000000	1.000
289	GOBP_PEPTIDYL_SERINE_MODIFICATION	GO:0018209	12	-0.227826	-0.673902	0.864634	1.000000	1.000
290	HALLMARK_EPITHELIAL_MESENCHYMAL_TRANSITION	M5930	5	-0.296845	-0.674608	0.879044	1.000000	1.000
291	GOBP_CELLULAR_RESPONSE_TO_PEPTIDE	GO:1901653	13	-0.229450	-0.681785	0.868712	1.000000	1.000
292	GOBP_RESPONSE_TO_PEPTIDE	GO:1901652	18	-0.214231	-0.684356	0.874118	1.000000	1.000
293	GOBP_NEGATIVE_REGULATION_OF_TRANSMEMBRANE_TRAN...	GO:0034763	6	-0.296125	-0.686373	0.864247	1.000000	1.000
294	REACTOME_LEISHMANIA_INFECTION	R-HSA-9658195	10	-0.250845	-0.686805	0.864428	1.000000	1.000
295	GOBP_CELL_JUNCTION_ASSEMBLY	GO:0034329	8	-0.269247	-0.687350	0.870249	1.000000	1.000
296	GOBP_RESPONSE_TO_BIOTIC_STIMULUS	GO:0009607	17	-0.217569	-0.689466	0.869880	1.000000	1.000
297	GOBP_CELL_CYCLE	GO:0007049	21	-0.207051	-0.690020	0.853714	1.000000	1.000
298	GOBP_SMALL_MOLECULE_BIOSYNTHETIC_PROCESS	GO:0044283	5	-0.311213	-0.691045	0.866109	1.000000	1.000

Continuation of Table **S6**

	NAME	ID	SIZE	ES	NES	NOM.p.val	FDR.q.val	FWER.p.val
299	GOBP_PHOSPHATIDYLINOSITOL_3_KINASE_SIGNALING	GO:0014065	7	-0.278522	-0.692001	0.857916	1.000000	1.000
300	GOBP_RECEPTOR_MEDIATED_ENDOCYTOSIS	GO:0006898	6	-0.291085	-0.693525	0.846462	1.000000	1.000
301	GOBP_REGULATION_OF_RESPONSE_TO_STRESS	GO:0080134	19	-0.213301	-0.693768	0.855803	1.000000	1.000
302	GOBP_NEGATIVE_REGULATION_OF_SYNAPTIC_TRANSMISSION	GO:0050805	6	-0.292428	-0.695632	0.857143	1.000000	1.000
303	GOBP_PEPTIDYL_TYROSINE_MODIFICATION	GO:0018212	10	-0.252436	-0.696569	0.840149	1.000000	1.000
304	REACTOME_CELL_CYCLE_MITOTIC	R-RNO-69278	5	-0.314616	-0.702142	0.857531	1.000000	1.000
305	GOBP_ORGANONITROGEN_COMPOUND_BIOSYNTHETIC_PROCESS	GO:1901566	13	-0.234257	-0.704440	0.844282	1.000000	1.000
306	GOBP_CELL_ACTIVATION	GO:0001775	22	-0.212307	-0.705937	0.846336	1.000000	1.000
307	GOBP_NEGATIVE_REGULATION_OF_PHOSPHORUS_METABOL...	GO:0010563	9	-0.260613	-0.706439	0.822165	1.000000	1.000
308	GOBP_REGULATION_OF_CELL_ACTIVATION	GO:0050865	8	-0.273410	-0.706843	0.864682	1.000000	1.000
309	GOBP_POSITIVE_REGULATION_OF_LOCOMOTION	GO:0040017	11	-0.247204	-0.708535	0.839196	1.000000	1.000
310	GOBP_PROTEIN_MODIFICATION_BY_SMALL_PROTEIN_CON...	GO:0032446	6	-0.303729	-0.717496	0.822342	1.000000	1.000
311	GOBP_IMPORT_INTO_CELL	GO:0098657	6	-0.303136	-0.718434	0.833333	1.000000	1.000
312	GOBP_ORGANONITROGEN_COMPOUND_CATABOLIC_PROCESS	GO:1901565	10	-0.262245	-0.719173	0.825255	1.000000	1.000
313	GOBP_REGULATION_OF_PHOSPHATIDYLINOSITOL_3_KINA...	GO:0014066	6	-0.311008	-0.723242	0.841611	1.000000	1.000
314	GOBP_REGULATION_OF_PROTEIN_MODIFICATION_PROCESS	GO:0031399	28	-0.208360	-0.725342	0.815107	1.000000	1.000
315	GOBP_DEFENSE_RESPONSE	GO:0006952	19	-0.222688	-0.725652	0.822562	1.000000	1.000
316	REACTOME_SIGNALING_BY_NTRKS	R-RNO-166520	18	-0.225937	-0.726817	0.803140	1.000000	1.000
317	GOBP_POSITIVE_REGULATION_OF_EPITHELIAL_CELL_PR...	GO:0050679	7	-0.293809	-0.729860	0.814136	1.000000	1.000
318	GOBP_MUSCLE_STRUCTURE_DEVELOPMENT	GO:0061061	16	-0.235574	-0.731154	0.817001	1.000000	1.000
319	GOBP_REGULATION_OF_CELL_DEATH	GO:0010941	34	-0.202377	-0.732338	0.822727	1.000000	1.000
320	GOBP_REGULATION_OF_CELL_CYCLE_PROCESS	GO:0010564	5	-0.329254	-0.733637	0.835022	1.000000	1.000
321	GOBP_REGULATION_OF_CELLULAR_AMIDE_METABOLIC_PR...	GO:0034248	9	-0.272273	-0.737085	0.828931	1.000000	1.000
322	GOBP_NEURON_PROJECTION_GUIDANCE	GO:0097485	9	-0.277235	-0.737589	0.832700	1.000000	1.000
323	GOBP_RESPONSE_TO_HORMONE	GO:0009725	20	-0.228754	-0.744769	0.819861	1.000000	1.000
324	GOBP_WOUND_HEALING	GO:0042060	13	-0.254044	-0.745919	0.795620	1.000000	1.000
325	GOBP_REGULATION_OF_CELLULAR_RESPONSE_TO_STRESS	GO:0080135	9	-0.281502	-0.749944	0.787565	1.000000	1.000
326	GOBP_ACTIVATION_OF_PROTEIN_KINASE_ACTIVITY	GO:0032147	10	-0.274616	-0.752277	0.786164	1.000000	1.000
327	GOBP_NEGATIVE_REGULATION_OF_LIPID_METABOLIC_PR...	GO:0045833	5	-0.340077	-0.754178	0.804469	1.000000	1.000
328	GOBP_REGULATION_OF_RESPONSE_TO_EXTERNAL_STIMULUS	GO:0032101	15	-0.249899	-0.758116	0.774699	1.000000	1.000
329	GOBP_EPITHELIUM_DEVELOPMENT	GO:0060429	15	-0.245869	-0.761841	0.761336	1.000000	1.000
330	GOBP_NEGATIVE_REGULATION_OF_IMMUNE_SYSTEM_PROCESS	GO:0002683	5	-0.342446	-0.763822	0.772480	1.000000	1.000
331	GOBP_CELLULAR_MACROMOLECULE_CATABOLIC_PROCESS	GO:0044265	5	-0.347808	-0.767212	0.803596	0.997591	1.000
332	GOBP_INTRACELLULAR_PROTEIN_TRANSPORT	GO:0006886	8	-0.295465	-0.767536	0.774366	0.999607	1.000
333	GOBP_RESPONSE_TO_OXYGEN_LEVELS	GO:0070482	8	-0.300775	-0.769724	0.770252	0.998719	1.000
334	REACTOME_TOLL_LIKE_RECEPTOR_CASCADES	R-RNO-168898	8	-0.304442	-0.774192	0.761255	0.994478	1.000
335	GOBP_REGULATION_OF_PHOSPHORUS_METABOLIC_PROCESS	GO:0051174	26	-0.228230	-0.774406	0.767361	0.996698	1.000
336	GOBP_POSITIVE_REGULATION_OF_ENDOTHELIAL_CELL_P...	GO:0001938	6	-0.327253	-0.777736	0.762640	0.994306	1.000
337	GOBP_CELLULAR_RESPONSE_TO_KETONE	GO:1901655	5	-0.352709	-0.778505	0.769441	0.995666	1.000
338	GOBP_REGULATION_OF_AXONOGENESIS	GO:0050770	7	-0.314004	-0.779999	0.760695	0.995861	1.000
339	GOBP_REGULATION_OF_CYTOSKELETON_ORGANIZATION	GO:0051493	5	-0.342908	-0.780034	0.780585	0.998379	1.000
340	GOBP_NUCLEAR_TRANSPORT	GO:0051169	5	-0.353719	-0.780372	0.773040	1.000000	1.000
341	GOBP_NEGATIVE_REGULATION_OF_PROTEIN_CATABOLIC_...	GO:0042177	5	-0.352341	-0.782753	0.773352	0.999267	1.000

Continuation of Table **S6**

	NAME	ID	SIZE	ES	NES	NOM.p.val	FDR.q.val	FWER.p.val
342	REACTOME_MYD88_INDEPENDENT_TLR4_CASCADE	R-RNO-166166	8	-0.304442	-0.784280	0.738562	0.999455	1.000
343	GOBP_POSITIVE_REGULATION_OF_CATION_TRANSMEMBRA...	GO:1904064	5	-0.349398	-0.787982	0.768377	0.996321	1.000
344	REACTOME_TOLL_LIKE_RECEPTOR_TLR1_TLR2_CASCADE	R-RNO-168179	8	-0.304442	-0.788548	0.753264	0.998053	1.000
345	GOBP_ERBB_SIGNALING_PATHWAY	GO:0038127	5	-0.347093	-0.789239	0.763984	0.999589	1.000
346	GOBP_REGULATION_OF_CELL_CYCLE	GO:0051726	13	-0.270568	-0.789240	0.735802	1.000000	1.000
347	GOBP_NEURON_DEATH	GO:0070997	18	-0.247553	-0.790334	0.752625	1.000000	1.000
348	GOBP_REGULATION_OF_TRANSFERASE_ACTIVITY	GO:0051338	21	-0.239325	-0.791387	0.744509	1.000000	1.000
349	REACTOME_CIRCADIAN_CLOCK	R-HSA-400253	5	-0.361446	-0.792476	0.750341	1.000000	1.000
350	GOBP_CELLULAR_RESPONSE_TO_DNA_DAMAGE_STIMULUS	GO:0006974	5	-0.360847	-0.792881	0.753156	1.000000	1.000
351	GOBP_STRIATED_MUSCLE_CELL_DIFFERENTIATION	GO:0051146	9	-0.296096	-0.794515	0.752282	1.000000	1.000
352	REACTOME_TOLL_LIKE_RECEPTOR_9_TLR9_CASCADE	R-RNO-168138	8	-0.304442	-0.797109	0.742466	1.000000	1.000
353	GOBP_REGULATION_OF_PROTEIN_LOCALIZATION_TO_CEL...	GO:1904375	6	-0.340436	-0.799511	0.756164	1.000000	1.000
354	GOBP_REGULATION_OF_PROTEIN_KINASE_ACTIVITY	GO:0045859	21	-0.239325	-0.800034	0.732329	1.000000	1.000
355	GOBP_MULTICELLULAR_ORGANISMAL_HOMEOSTASIS	GO:0048871	12	-0.275249	-0.808625	0.722153	0.993261	1.000
356	GOBP_POSITIVE_REGULATION_OF_MYELOID_CELL_DIFFE...	GO:0045639	7	-0.325086	-0.809200	0.730978	0.994966	1.000
357	GOBP_INTRACELLULAR_TRANSPORT	GO:0046907	10	-0.297707	-0.813515	0.725131	0.990314	1.000
358	GOBP_REGULATION_OF_PROTEIN_STABILITY	GO:0031647	5	-0.367587	-0.813727	0.746248	0.992622	1.000
359	REACTOME_TRANSCRIPTIONAL_REGULATION_BY_TP53	R-RNO-3700989	6	-0.344722	-0.814512	0.718291	0.993945	1.000
360	GOBP_REGULATION_OF_LIPASE_ACTIVITY	GO:0060191	6	-0.346693	-0.816252	0.728571	0.993708	1.000
361	GOBP_POSITIVE_REGULATION_OF_PROTEIN_KINASE_ACT...	GO:0045860	18	-0.254491	-0.817105	0.725537	0.994955	1.000
362	GOBP_REGULATION_OF_PHOSPHOLIPASE_ACTIVITY	GO:0010517	6	-0.346693	-0.817347	0.715223	0.997281	1.000
363	GOBP_SMALL_GTPASE_MEDIATED_SIGNAL_TRANSDUCTION	GO:0007264	8	-0.314748	-0.818300	0.722149	0.998404	1.000
364	GOBP_RESPONSE_TO_TEMPERATURE_STIMULUS	GO:0009266	9	-0.309381	-0.818673	0.703608	1.000000	1.000
365	GOBP_TRANSMEMBRANE_RECEPTOR_PROTEIN_TYROSINE_K...	GO:0007169	19	-0.254835	-0.819756	0.731536	1.000000	1.000
366	GOBP_POSITIVE_REGULATION_OF_RESPONSE_TO_EXTERN...	GO:0032103	9	-0.304854	-0.820379	0.730769	1.000000	1.000
367	GOBP_RESPONSE_TO_OXYGEN_CONTAINING_COMPOUND	GO:1901700	39	-0.224616	-0.821895	0.685430	1.000000	1.000
368	REACTOME_MEMBRANE_TRAFFICKING	R-RNO-199991	5	-0.376994	-0.826700	0.710345	0.996976	1.000
369	GOBP_AGING	GO:0007568	13	-0.274892	-0.828709	0.685961	0.996012	1.000
370	GOBP_POSITIVE_REGULATION_OF_ION_TRANSPORT	GO:0043270	17	-0.261551	-0.829262	0.689696	0.997813	1.000
371	GOBP_CARBOHYDRATE_DERIVATIVE_BIOSYNTHETIC_PROCESS	GO:1901137	5	-0.363253	-0.829414	0.709632	1.000000	1.000
372	GOBP_DEVELOPMENTAL_PROCESS_INVOLVED_IN_REPRODU...	GO:0003006	11	-0.295691	-0.833283	0.706186	0.995946	1.000
373	GOBP_NEGATIVE_REGULATION_OF_PHOSPHORYLATION	GO:0042326	7	-0.331776	-0.833539	0.689243	0.998305	1.000
374	GOBP_PEPTIDE_BIOSYNTHETIC_PROCESS	GO:0043043	6	-0.352211	-0.834422	0.701705	0.999518	1.000
375	GOBP_NERVE_DEVELOPMENT	GO:0021675	9	-0.317591	-0.834744	0.683444	1.000000	1.000
376	REACTOME_INTERLEUKIN_17_SIGNALING	R-RNO-448424	6	-0.358449	-0.837367	0.704762	0.999686	1.000
377	GOBP_HEART_DEVELOPMENT	GO:0007507	6	-0.361401	-0.837584	0.692617	1.000000	1.000
378	GOBP_AMIDE_BIOSYNTHETIC_PROCESS	GO:0043604	6	-0.352211	-0.838260	0.689655	1.000000	1.000
379	GOBP_REGULATION_OF_PROTEIN_PHOSPHORYLATION	GO:0001932	25	-0.244814	-0.840052	0.677602	1.000000	1.000
380	GOBP_CARDIAC_MUSCLE_TISSUE_DEVELOPMENT	GO:0048738	6	-0.361401	-0.840901	0.684573	1.000000	1.000
381	GOBP_CELLULAR_RESPONSE_TO_HORMONE_STIMULUS	GO:0032870	11	-0.296515	-0.842400	0.680958	1.000000	1.000
382	GOBP_PEPTIDYL_AMINO_ACID_MODIFICATION	GO:0018193	22	-0.253547	-0.842411	0.666278	1.000000	1.000
383	GOBP_LEUKOCYTE_MIGRATION	GO:0050900	6	-0.360346	-0.843995	0.699864	1.000000	1.000
384	GOBP_MYOTUBE_DIFFERENTIATION	GO:0014902	5	-0.370176	-0.844379	0.686798	1.000000	1.000

Continuation of Table **S6**

	NAME	ID	SIZE	ES	NES	NOM.p.val	FDR.q.val	FWER.p.val
385	REACTOME_VESICLE_MEDIATED_TRANSPORT	R-RNO-5653656	5	-0.376994	-0.844526	0.679063	1.000000	1.000
386	GOBP_REGULATION_OF_ANION_TRANSPORT	GO:0044070	20	-0.257561	-0.847894	0.662486	1.000000	1.000
387	GOBP_PROTEIN_LOCALIZATION_TO_CELL_JUNCTION	GO:1902414	6	-0.364239	-0.850727	0.692412	1.000000	1.000
388	GOBP_ANATOMICAL_STRUCTURE_HOMEOSTASIS	GO:0060249	7	-0.348364	-0.852897	0.682825	1.000000	1.000
389	GOBP_LIPID_LOCALIZATION	GO:0010876	10	-0.309666	-0.854643	0.636129	1.000000	1.000
390	REACTOME_INTERFERON_SIGNALING	R-RNO-913531	6	-0.363516	-0.855462	0.685950	1.000000	1.000
391	GOBP_POSITIVE_REGULATION_OF_PROTEIN_PHOSPHORYL...	GO:0001934	22	-0.254643	-0.857540	0.657955	1.000000	1.000
392	GOBP_POSITIVE_REGULATION_OF_PROTEIN_MODIFICATI...	GO:0031401	22	-0.254643	-0.859371	0.677419	1.000000	1.000
393	GOBP_ORGANIC_ACID_METABOLIC_PROCESS	GO:0006082	7	-0.345324	-0.863171	0.652174	0.999751	1.000
394	GOBP_REGULATION_OF_CELLULAR_COMPONENT_BIOGENESIS	GO:0044087	10	-0.318571	-0.863970	0.630295	1.000000	1.000
395	GOBP_IMMUNE_EFFECTOR_PROCESS	GO:0002252	11	-0.308999	-0.865282	0.677096	1.000000	1.000
396	GOBP_RESPONSE_TO_ACID_CHEMICAL	GO:0001101	6	-0.368223	-0.865822	0.649596	1.000000	1.000
397	GOBP_RESPONSE_TO_MONOSACCHARIDE	GO:0034284	5	-0.387760	-0.866815	0.656944	1.000000	1.000
398	GOBP_ANIMAL_ORGAN_MORPHOGENESIS	GO:0009887	12	-0.299789	-0.869346	0.634116	1.000000	1.000
399	GOBP_ORGANIC_HYDROXY_COMPOUND_METABOLIC_PROCESS	GO:1901615	8	-0.335682	-0.871615	0.637076	1.000000	1.000
400	REACTOME_CELL_CYCLE	R-RNO-1640170	6	-0.363415	-0.874535	0.655028	0.997995	1.000
401	GOBP_CELLULAR_RESPONSE_TO_NITROGEN_COMPOUND	GO:1901699	20	-0.274434	-0.877699	0.667845	0.994704	1.000
402	GOBP_DNA_METABOLIC_PROCESS	GO:0006259	6	-0.377127	-0.878980	0.635097	0.995133	1.000
403	GOBP_GAMETE_GENERATION	GO:0007276	9	-0.326947	-0.880208	0.640741	0.995537	1.000
404	GOBP_MONOCARBOXYLIC_ACID_METABOLIC_PROCESS	GO:0032787	6	-0.371294	-0.880307	0.625000	0.998390	1.000
405	GOBP_POSITIVE_REGULATION_OF_NEURON_PROJECTION_...	GO:0010976	5	-0.391478	-0.881422	0.629734	0.999122	1.000
406	GOBP_POSITIVE_REGULATION_OF_LIPASE_ACTIVITY	GO:0060193	5	-0.397590	-0.882524	0.628965	0.999964	1.000
407	GOBP_RESPONSE_TO_CARBOHYDRATE	GO:0009743	5	-0.387760	-0.882662	0.645429	1.000000	1.000
408	GOBP_REGULATION_OF_CELLULAR_LOCALIZATION	GO:0060341	17	-0.276841	-0.883352	0.614302	1.000000	1.000
409	GOBP_MITOTIC_CELL_CYCLE	GO:0000278	10	-0.321030	-0.885814	0.628750	1.000000	1.000
410	GOBP_REGULATION_OF_PHOSPHOLIPASE_C_ACTIVITY	GO:1900274	5	-0.397590	-0.887387	0.625683	1.000000	1.000
411	GOBP_TRANSMEMBRANE_RECEPTOR_PROTEIN_SERINE_THR...	GO:0007178	6	-0.380291	-0.893817	0.637097	0.991324	1.000
412	REACTOME_DISEASES_OF_SIGNAL_TRANSDUCTION_BY_GR...	R-HSA-5663202	12	-0.312370	-0.900611	0.601242	0.979409	1.000
413	GOBP_CELLULAR_RESPONSE_TO_PEPTIDE_HORMONE_STIM...	GO:0071375	9	-0.332458	-0.901989	0.607417	0.979582	1.000
414	GOBP_POSITIVE_REGULATION_OF_TRANSFERASE_ACTIVITY	GO:0051347	19	-0.277121	-0.902172	0.590698	0.982344	1.000
415	GOBP_LIPID_EXPORT_FROM_CELL	GO:0140353	5	-0.405131	-0.903079	0.594901	0.983592	1.000
416	GOBP_RECEPTOR_INTERNALIZATION	GO:0031623	5	-0.414102	-0.904008	0.620833	0.984813	1.000
417	GOBP_TUBE_DEVELOPMENT	GO:0035295	18	-0.279044	-0.904037	0.590047	0.987955	1.000
418	GOBP_NEGATIVE_REGULATION_OF_CATABOLIC_PROCESS	GO:0009895	8	-0.357945	-0.904177	0.614474	0.990871	1.000
419	GOBP_CENTRAL_NERVOUS_SYSTEM_NEURON_DIFFERENTIA...	GO:0021953	5	-0.408556	-0.905451	0.596045	0.991274	1.000
420	REACTOME_ONCOGENIC_MAPK_SIGNALING	R-HSA-6802957	5	-0.404445	-0.905885	0.583102	0.993542	1.000
421	GOBP_NEGATIVE_REGULATION_OF_TRANSPORT	GO:0051051	13	-0.307395	-0.906385	0.605875	0.995702	1.000
422	GOBP_CIRCULATORY_SYSTEM_DEVELOPMENT	GO:0072359	21	-0.272697	-0.907594	0.594373	0.996318	1.000
423	GOBP_MUSCLE_CELL_DEVELOPMENT	GO:0055001	5	-0.413976	-0.910006	0.592437	0.993923	1.000
424	GOBP_MALE_GAMETE_GENERATION	GO:0048232	5	-0.406309	-0.910148	0.591922	0.996858	1.000
425	GOBP_RESPONSE_TO_EXTRACELLULAR_STIMULUS	GO:0009991	13	-0.314332	-0.915547	0.602469	0.987841	1.000
426	GOBP_REGULATION_OF_CELLULAR_CATABOLIC_PROCESS	GO:0031329	10	-0.338927	-0.916162	0.609665	0.989733	1.000
427	GOBP_VASCULATURE_DEVELOPMENT	GO:0001944	17	-0.292637	-0.916398	0.580952	0.992532	1.000

Continuation of Table **S6**

	NAME	ID	SIZE	ES	NES	NOM.p.val	FDR.q.val	FWER.p.val
428	GOBP_RESPONSE_TO_LIGHT_STIMULUS	GO:0009416	11	-0.324988	-0.916913	0.572660	0.994704	1.000
429	GOBP_NEGATIVE_REGULATION_OF_CELLULAR_COMPONENT...	GO:0051129	11	-0.322351	-0.919128	0.575309	0.992715	1.000
430	GOBP_REGULATION_OF_PROTEOLYSIS	GO:0030162	12	-0.319656	-0.920068	0.577307	0.994136	1.000
431	GOBP_CIRCADIAN_RHYTHM	GO:0007623	13	-0.311067	-0.923193	0.563882	0.990152	1.000
432	GOBP_POSITIVE_REGULATION_OF_PROTEIN_SERINE_THR...	GO:0071902	9	-0.348141	-0.925260	0.581425	0.988661	1.000
433	GOBP_RESPONSE_TO_PEPTIDE_HORMONE	GO:0043434	13	-0.311667	-0.925649	0.591584	0.991215	1.000
434	GOBP_PROTEIN_PHOSPHORYLATION	GO:0006468	33	-0.258178	-0.927232	0.590508	0.991023	1.000
435	GOBP_SMALL_MOLECULE_METABOLIC_PROCESS	GO:0044281	16	-0.301653	-0.929089	0.572491	0.990244	1.000
436	GOBP_CELL_JUNCTION_ORGANIZATION	GO:0034330	19	-0.287178	-0.929359	0.571934	0.993020	1.000
437	GOBP_RESPONSE_TO_CADMIUM_ION	GO:0046686	6	-0.400175	-0.930835	0.566622	0.992912	1.000
438	GOBP_REGULATION_OF_CIRCADIAN_RHYTHM	GO:0042752	7	-0.378874	-0.932605	0.583663	0.992145	1.000
439	GOBP_RESPONSE_TO_WOUNDING	GO:0009611	15	-0.304270	-0.934759	0.559466	0.990324	1.000
440	GOBP_RECEPTOR_METABOLIC_PROCESS	GO:0043112	5	-0.414102	-0.936405	0.567455	0.989864	1.000
441	GOBP_ENZYME_LINKED_RECEPTOR_PROTEIN_SIGNALING_...	GO:0007167	24	-0.276629	-0.937168	0.553265	0.991593	1.000
442	GOBP_NEGATIVE_REGULATION_OF_CELL_PROJECTION_OR...	GO:0031345	5	-0.421065	-0.940762	0.554960	0.986027	1.000
443	GOBP_REGULATION_OF_CELLULAR_PROTEIN_LOCALIZATION	GO:1903827	12	-0.323978	-0.941481	0.567183	0.987688	1.000
444	GOBP_RESPONSE_TO_OXIDATIVE_STRESS	GO:0006979	8	-0.368810	-0.941561	0.559740	0.990994	1.000
445	GOBP_APOPTOTIC_MITOCHONDRIAL_CHANGES	GO:0008637	6	-0.400949	-0.941702	0.537212	0.994168	1.000
446	GOBP_POSITIVE_REGULATION_OF_PHOSPHORUS_METABOL...	GO:0010562	23	-0.278474	-0.944471	0.549943	0.991147	1.000
447	GOBP_REGULATION_OF_PROTEIN_SERINE_THREONINE_KI...	GO:0071900	12	-0.327308	-0.946735	0.538365	0.988921	1.000
448	GOBP_POSITIVE_REGULATION_OF_ESTABLISHMENT_OF_P...	GO:1904951	5	-0.422991	-0.947729	0.557065	0.990086	1.000
449	GOBP_REGULATION_OF_PROTEIN_LOCALIZATION_TO_MEM...	GO:1905475	7	-0.379983	-0.948756	0.555108	0.991186	1.000
450	GOBP_REGULATION_OF_LIPID_METABOLIC_PROCESS	GO:0019216	7	-0.384869	-0.949688	0.570470	0.992404	1.000
451	GOBP_REGULATION_OF_CATABOLIC_PROCESS	GO:0009894	13	-0.327352	-0.954400	0.523515	0.984090	1.000
452	GOBP_RESPONSE_TO_HEAT	GO:0009408	7	-0.388747	-0.960420	0.522788	0.972928	1.000
453	GOBP_CELL_CELL_SIGNALING_BY_WNT	GO:0198738	7	-0.382308	-0.961325	0.528571	0.974079	1.000
454	GOBP_RESPONSE_TO_REACTIVE_OXYGEN_SPECIES	GO:0000302	8	-0.368810	-0.961466	0.534301	0.977363	1.000
455	GOBP_REPRODUCTION	GO:0000003	18	-0.298492	-0.963504	0.525120	0.975734	1.000
456	GOBP_RESPONSE_TO_INORGANIC_SUBSTANCE	GO:0010035	19	-0.294584	-0.964646	0.515187	0.976577	1.000
457	GOBP_EMBRYONIC_ORGAN_DEVELOPMENT	GO:0048568	7	-0.387277	-0.966332	0.528121	0.975855	1.000
458	GOBP_MUSCLE_SYSTEM_PROCESS	GO:0003012	9	-0.359911	-0.968518	0.518325	0.973450	1.000
459	GOBP_CELLULAR_RESPONSE_TO_REACTIVE_OXYGEN_SPECIES	GO:0034614	7	-0.393268	-0.969891	0.533424	0.973708	1.000
460	REACTOME_SIGNALING_BY_NTRK2_TRKB	R-RNO-9006115	6	-0.402439	-0.974258	0.500000	0.966161	1.000
461	GOBP_REGULATION_OF_HYDROLASE_ACTIVITY	GO:0051336	22	-0.290844	-0.982034	0.497090	0.950193	1.000
462	GOBP_REGULATION_OF_GLIOGENESIS	GO:0014013	5	-0.447573	-0.987050	0.496393	0.940750	1.000
463	GOBP_CELLULAR_RESPONSE_TO_CHEMICAL_STRESS	GO:0062197	7	-0.393268	-0.990946	0.482260	0.934022	1.000
464	GOBP_ESTABLISHMENT_OF_PROTEIN_LOCALIZATION_TO_...	GO:0072594	5	-0.450095	-0.993776	0.488920	0.930425	1.000
465	GOBP_SECOND_MESSENGER_MEDIATED_SIGNALING	GO:0019932	16	-0.319290	-0.994408	0.467213	0.932373	1.000
466	GOBP_POSITIVE_REGULATION_OF_GLIOGENESIS	GO:0014015	5	-0.447573	-0.995198	0.473907	0.933761	1.000
467	REACTOME_NEUTROPHIL_DEGRANULATION	R-RNO-6798695	6	-0.423572	-0.995792	0.489160	0.935964	1.000
468	GOBP_CELL_ACTIVATION_INVOLVED_IN_IMMUNE_RESPONSE	GO:0002263	6	-0.423572	-0.997711	0.467480	0.934686	1.000
469	GOBP_RESPONSE_TO_METAL_ION	GO:0010038	15	-0.329158	-0.998627	0.476364	0.935914	1.000
470	GOBP_REGULATION_OF_BODY_FLUID_LEVELS	GO:0050878	11	-0.358285	-1.001766	0.459900	0.931155	1.000

Continuation of Table **S6**

	NAME	ID	SIZE	ES	NES	NOM.p.val	FDR.q.val	FWER.p.val
471	GOBP_REGULATION_OF_ORGANELLE_ORGANIZATION	GO:0033043	14	-0.337447	-1.009621	0.456368	0.914225	1.000
472	GOBP_FOREBRAIN_DEVELOPMENT	GO:0030900	10	-0.363096	-1.009999	0.443717	0.916850	1.000
473	GOBP_RESPONSE_TO_CALCIUM_ION	GO:0051592	6	-0.433439	-1.011688	0.462888	0.915767	1.000
474	GOBP_ENDOCYTOSIS	GO:0006897	8	-0.390066	-1.012205	0.437077	0.918035	1.000
475	GOBP_CIRCULATORY_SYSTEM_PROCESS	GO:0003013	5	-0.455657	-1.012989	0.448324	0.919609	1.000
476	GOBP_LONG_TERM_MEMORY	GO:0007616	6	-0.428579	-1.014406	0.460598	0.919684	1.000
477	GOBP_REGULATION_OF_CELL_PROJECTION_ORGANIZATION	GO:0031344	20	-0.306969	-1.015699	0.458810	0.919795	1.000
478	GOBP_MUSCLE_ORGAN_DEVELOPMENT	GO:0007517	9	-0.373736	-1.017243	0.448539	0.919383	1.000
479	GOBP_RHYTHMIC_PROCESS	GO:0048511	15	-0.329280	-1.017999	0.446321	0.921172	1.000
480	GOBP_MYELOID_LEUKOCYTE_MEDIATED_IMMUNITY	GO:0002444	6	-0.423572	-1.020413	0.418157	0.918178	1.000
481	REACTOME_EXTRA_NUCLEAR_ESTROGEN_SIGNALING	R-RNO-9009391	7	-0.412487	-1.029199	0.421265	0.898100	1.000
482	GOBP_RESPONSE_TO_NICOTINE	GO:0035094	7	-0.416515	-1.031457	0.438896	0.895573	1.000
483	GOBP_TAXIS	GO:0042330	14	-0.339898	-1.031632	0.431373	0.898782	1.000
484	GOBP_REGULATION_OF_HORMONE_LEVELS	GO:0010817	12	-0.355650	-1.034795	0.404177	0.893870	1.000
485	GOBP_SEXUAL_REPRODUCTION	GO:0019953	10	-0.382210	-1.040402	0.419562	0.882369	1.000
486	GOBP_REGULATION_OF_SYNAPSE_STRUCTURE_OR_ACTIVITY	GO:0050803	11	-0.368234	-1.041782	0.404337	0.882305	1.000
487	GOBP_SYNAPSE_ORGANIZATION	GO:0050808	17	-0.330055	-1.043274	0.426887	0.881915	1.000
488	REACTOME_ESR_MEDIATED_SIGNALING	R-RNO-8939211	8	-0.406189	-1.045433	0.425560	0.879882	1.000
489	GOBP_MULTICELLULAR_ORGANISM_REPRODUCTION	GO:0032504	12	-0.364483	-1.047045	0.435961	0.879185	1.000
490	REACTOME_ANTI_INFLAMMATORY_RESPONSE_FAVOURING_...	R-HSA-9662851	5	-0.476903	-1.047365	0.411189	0.882081	1.000
491	HALLMARK_UV_RESPONSE_UP	M5941	5	-0.471416	-1.049798	0.393162	0.878957	1.000
492	GOBP_PROTEIN_COMPLEX_OLIGOMERIZATION	GO:0051259	7	-0.431281	-1.056544	0.392438	0.864300	1.000
493	GOBP_REGULATION_OF_SYSTEM_PROCESS	GO:0044057	18	-0.335132	-1.060573	0.418632	0.856998	1.000
494	GOBP_POSITIVE_REGULATION_OF_CELLULAR_PROTEIN_L...	GO:1903829	7	-0.430259	-1.062730	0.387399	0.854927	1.000
495	GOBP_PURINE_CONTAINING_COMPOUND_METABOLIC_PROCESS	GO:0072521	6	-0.447672	-1.064219	0.372093	0.854615	1.000
496	GOBP_REGULATION_OF_LONG_TERM_SYNAPTIC_POTENTIA...	GO:1900271	6	-0.455590	-1.064681	0.401907	0.857136	1.000
497	GOBP_RESPONSE_TO_STARVATION	GO:0042594	5	-0.481524	-1.065815	0.359661	0.857811	1.000
498	REACTOME_SIGNALING_BY_NUCLEAR_RECEPTORS	R-RNO-9006931	8	-0.406189	-1.066692	0.404539	0.859151	1.000
499	REACTOME_PLATELET_ACTIVATION_SIGNALING_AND_AGG...	R-HSA-76002	7	-0.427030	-1.068083	0.389543	0.858916	1.000
500	GOBP_MULTICELLULAR_ORGANISMAL_RESPONSE_TO_STRESS	GO:0033555	5	-0.485190	-1.070182	0.385359	0.856954	1.000
501	GOBP_REGULATION_OF_PEPTIDE_SECRETION	GO:0002791	7	-0.437874	-1.070351	0.357616	0.860265	1.000
502	GOBP_CELLULAR_RESPONSE_TO_STARVATION	GO:0009267	5	-0.481524	-1.075573	0.373427	0.849126	1.000
503	GOBP_PROTEIN_LOCALIZATION_TO_POSTSYNAPSE	GO:0062237	5	-0.486396	-1.076414	0.375000	0.850703	1.000
504	GOBP_REGULATION_OF_PEPTIDE_HORMONE_SECRETION	GO:0090276	6	-0.466019	-1.082574	0.380223	0.837103	1.000
505	REACTOME_CELLULAR_RESPONSES_TO_EXTERNAL_STIMULI	R-RNO-8953897	11	-0.384844	-1.086205	0.361461	0.830562	1.000
506	GOBP_PROTEIN_LOCALIZATION_TO_SYNAPSE	GO:0035418	5	-0.486396	-1.086572	0.337838	0.833343	1.000
507	GOBP_ORGANIC_HYDROXY_COMPOUND_TRANSPORT	GO:0015850	6	-0.456118	-1.096272	0.348361	0.810120	1.000
508	GOBP_POSITIVE_REGULATION_OF_TRANSPORT	GO:0051050	26	-0.322156	-1.099127	0.356902	0.805588	1.000
509	GOBP_REGULATION_OF_PROTEIN_LOCALIZATION	GO:0032880	18	-0.340102	-1.100017	0.345828	0.806796	1.000
510	GOBP_RESPONSE_TO_RADIATION	GO:0009314	13	-0.372167	-1.100361	0.353960	0.809677	1.000
511	GOBP_REGULATION_OF_NERVOUS_SYSTEM_PROCESS	GO:0031644	8	-0.431106	-1.101068	0.364362	0.811578	1.000
512	GOBP_MUSCLE_TISSUE_DEVELOPMENT	GO:0060537	11	-0.388900	-1.101206	0.340768	0.814972	1.000
513	GOBP_EMBRYONIC_MORPHOGENESIS	GO:0048598	6	-0.466873	-1.106211	0.319120	0.804618	1.000

Continuation of Table **S6**

	NAME	ID	SIZE	ES	NES	NOM.p.val	FDR.q.val	FWER.p.val
514	GOBP_LEUKOCYTE_MEDIATED_IMMUNITY	GO:0002443	8	-0.430891	-1.108912	0.320704	0.800405	1.000
515	GOBP_MULTI_ORGANISM_PROCESS	GO:0051704	16	-0.346949	-1.110369	0.336574	0.800216	1.000
516	GOBP_ENDOMEMBRANE_SYSTEM_ORGANIZATION	GO:0010256	6	-0.471879	-1.117926	0.340483	0.783414	1.000
517	GOBP_REGULATION_OF_INTRACELLULAR_SIGNAL_TRANSD...	GO:1902531	21	-0.336651	-1.118902	0.336009	0.784286	1.000
518	REACTOME_ESTROGEN_DEPENDENT_NUCLEAR_EVENTS_DOW...	R-RNO-9634638	5	-0.500880	-1.120538	0.307153	0.783573	1.000
519	GOBP_RESPONSE_TO_DRUG	GO:0042493	11	-0.396257	-1.122134	0.311111	0.782897	1.000
520	GOBP_POSITIVE_REGULATION_OF_MAPK_CASCADE	GO:0043410	9	-0.428237	-1.126885	0.317889	0.773680	1.000
521	GOBP_CELLULAR_RESPONSE_TO_ALCOHOL	GO:0097306	5	-0.502900	-1.127052	0.310000	0.777029	1.000
522	GOBP_POSITIVE_REGULATION_OF_INTRACELLULAR_SIGN...	GO:1902533	14	-0.373160	-1.127839	0.303030	0.778727	1.000
523	GOBP_MEMORY	GO:0007613	13	-0.384815	-1.128180	0.321696	0.781634	1.000
524	GOBP_DENDRITIC_SPINE_MORPHOGENESIS	GO:0060997	5	-0.514852	-1.131873	0.296296	0.774994	1.000
525	GOBP_REGULATION_OF_PEPTIDASE_ACTIVITY	GO:0052547	10	-0.412740	-1.136852	0.309278	0.765023	1.000
526	GOBP_POSITIVE_REGULATION_OF_HYDROLASE_ACTIVITY	GO:0051345	16	-0.364479	-1.138746	0.296117	0.763032	1.000
527	GOBP_ACTIVATION_OF_MAPK_ACTIVITY	GO:0000187	6	-0.493880	-1.155529	0.247191	0.720409	1.000
528	GOBP_POSITIVE_REGULATION_OF_MAP_KINASE_ACTIVITY	GO:0043406	6	-0.493880	-1.157169	0.291287	0.719448	1.000
529	GOBP_ERK1_AND_ERK2_CASCADE	GO:0070371	5	-0.519507	-1.160664	0.270572	0.713768	1.000
530	GOBP_NEGATIVE_REGULATION_OF_CELL_CYCLE	GO:0045786	5	-0.520457	-1.160783	0.268428	0.717071	1.000
531	GOBP_ANION_TRANSMEMBRANE_TRANSPORT	GO:0098656	7	-0.467998	-1.166148	0.261905	0.705939	1.000
532	REACTOME_RNA_POLYMERASE_II_TRANSCRIPTION	R-RNO-73857	20	-0.359391	-1.171689	0.247059	0.694306	1.000
533	GOBP_POSITIVE_REGULATION_OF_LONG_TERM_SYNAPTIC...	GO:1900273	5	-0.522380	-1.173691	0.266667	0.692472	1.000
534	GOBP_CELLULAR_COMPONENT_MORPHOGENESIS	GO:0032989	25	-0.345047	-1.174357	0.265143	0.694275	1.000
535	REACTOME_MAPK_TARGETS_NUCLEAR_EVENTS_MEDIATED_...	R-HSA-450282	5	-0.528385	-1.176755	0.267908	0.691302	1.000
536	GOBP_CELL_PART_MORPHOGENESIS	GO:0032990	25	-0.345047	-1.179801	0.267717	0.686362	1.000
537	REACTOME_DEVELOPMENTAL_BIOLOGY	R-RNO-1266738	22	-0.354981	-1.183012	0.260718	0.681095	1.000
538	GOBP_CELL_MORPHOGENESIS	GO:0000902	25	-0.345047	-1.184609	0.251995	0.680333	1.000
539	GOBP_REGULATION_OF_PEPTIDE_TRANSPORT	GO:0090087	10	-0.431972	-1.188118	0.246787	0.674382	1.000
540	GOBP_CELL_CYCLE_PROCESS	GO:0022402	11	-0.422034	-1.189070	0.237611	0.675377	1.000
541	GOBP_CELL_MORPHOGENESIS_INVOLVED_IN_NEURON_DIF...	GO:0048667	22	-0.358398	-1.189309	0.254023	0.678480	1.000
542	GOBP_POSITIVE_REGULATION_OF_CELL_DEATH	GO:0010942	19	-0.363029	-1.190170	0.248235	0.679958	1.000
543	GOBP_PROTEIN_LOCALIZATION_TO_ORGANELLE	GO:0033365	8	-0.467929	-1.193822	0.236041	0.673191	1.000
544	GOBP_RESPIRATORY_SYSTEM_DEVELOPMENT	GO:0060541	6	-0.511778	-1.195323	0.221289	0.672861	1.000
545	REACTOME_SIGNALING_BY_WNT	R-RNO-195721	7	-0.482202	-1.196352	0.238411	0.673670	1.000
546	GOBP_CELL_MORPHOGENESIS_INVOLVED_IN_DIFFERENTI...	GO:0000904	22	-0.358398	-1.199990	0.244521	0.667177	1.000
547	GOBP_SKELETAL_MUSCLE_ORGAN_DEVELOPMENT	GO:0060538	5	-0.536128	-1.200111	0.226891	0.670538	1.000
548	GOBP_RESPONSE_TO_ALKALOID	GO:0043279	5	-0.538151	-1.200167	0.216066	0.674179	1.000
549	GOBP_CALCIUM_MEDIATED_SIGNALING	GO:0019722	10	-0.432882	-1.200591	0.243073	0.676715	1.000
550	GOBP_MITOCHONDRION_ORGANIZATION	GO:0007005	7	-0.484528	-1.201357	0.236000	0.678484	1.000
551	REACTOME_POST_TRANSLATIONAL_PROTEIN_MODIFICATION	R-RNO-597592	7	-0.489380	-1.201414	0.218543	0.682158	1.000
552	GOBP_REGULATION_OF_MAP_KINASE_ACTIVITY	GO:0043405	9	-0.453353	-1.202411	0.240602	0.683376	1.000
553	GOBP_CELLULAR_MACROMOLECULE_LOCALIZATION	GO:0070727	20	-0.365628	-1.204134	0.224277	0.682272	1.000
554	GOBP_POSITIVE_REGULATION_OF_PROTEIN_METABOLIC_...	GO:0051247	27	-0.345549	-1.205970	0.243820	0.681016	1.000
555	REACTOME_INTEGRATION_OF_ENERGY_METABOLISM	R-RNO-163685	5	-0.542169	-1.209384	0.240331	0.675202	1.000
556	GOBP_TELENCEPHALON_DEVELOPMENT	GO:0021537	7	-0.483254	-1.210240	0.220028	0.676509	1.000

Continuation of Table **S6**

	NAME	ID	SIZE	ES	NES	NOM.p.val	FDR.q.val	FWER.p.val
557	GOBP_PROTEIN_LOCALIZATION_TO_MEMBRANE	GO:0072657	13	-0.412578	-1.215876	0.232360	0.664307	1.000
558	GOBP_REGULATION_OF_TRANSMEMBRANE_TRANSPORT	GO:0034762	23	-0.357250	-1.218218	0.227950	0.661669	1.000
559	GOBP_ADENYLATE_CYCLASE_INHIBITING_G_PROTEIN_CO...	GO:0007196	5	-0.554022	-1.220394	0.220816	0.659065	1.000
560	GOBP_POSITIVE_REGULATION_OF_PROTEOLYSIS	GO:0045862	8	-0.475030	-1.220790	0.232558	0.661975	1.000
561	GOBP_PROTEIN_LOCALIZATION_TO_PLASMA_MEMBRANE	GO:0072659	7	-0.500420	-1.223316	0.207622	0.659066	1.000
562	GOBP_INSULIN_SECRETION	GO:0030073	7	-0.494120	-1.224552	0.221614	0.659684	1.000
563	GOBP_CELLULAR_COMPONENT_MAINTENANCE	GO:0043954	5	-0.544998	-1.224787	0.209497	0.663032	1.000
564	GOBP_EPITHELIAL_CELL_DIFFERENTIATION	GO:0030855	7	-0.497576	-1.228931	0.201847	0.655568	1.000
565	GOBP_POSITIVE_REGULATION_OF_PEPTIDASE_ACTIVITY	GO:0010952	7	-0.506670	-1.230211	0.194444	0.656014	1.000
566	GOBP_REGULATION_OF_MUSCLE_ADAPTATION	GO:0043502	5	-0.550733	-1.233226	0.196453	0.651259	1.000
567	GOBP_REGULATION_OF_POSTSYNAPSE_ORGANIZATION	GO:0099175	6	-0.524672	-1.233911	0.201072	0.653392	1.000
568	GOBP_REGULATION_OF_CYSTEINE_TYPE_ENDOPEPTIDASE...	GO:2000116	9	-0.459510	-1.234881	0.200782	0.654676	1.000
569	GOBP_CELLULAR_RESPONSE_TO_INORGANIC_SUBSTANCE	GO:0071241	11	-0.440201	-1.238110	0.191011	0.649599	1.000
570	GOBP_NEURON_DIFFERENTIATION	GO:0030182	37	-0.345350	-1.239698	0.214597	0.649013	1.000
571	GOBP_MUSCLE_ADAPTATION	GO:0043500	5	-0.550733	-1.241769	0.186969	0.647111	1.000
572	GOBP_ESTABLISHMENT_OF_PROTEIN_LOCALIZATION	GO:0045184	17	-0.396998	-1.242524	0.197430	0.649090	1.000
573	GOBP_CHEMICAL_SYNAPTIC_TRANSMISSION_POSTSYNAPTIC	GO:0099565	9	-0.462464	-1.244061	0.199224	0.649076	1.000
574	GOBP_REGULATION_OF_SYNAPTIC_TRANSMISSION_GLUTA...	GO:0051966	12	-0.436316	-1.247869	0.214724	0.642536	1.000
575	GOBP_ORGANOPHOSPHATE_METABOLIC_PROCESS	GO:0019637	8	-0.485329	-1.252971	0.182529	0.632291	1.000
576	GOBP_DENDRITE_DEVELOPMENT	GO:0016358	10	-0.453225	-1.253528	0.186352	0.634851	1.000
577	GOBP_REGULATION_OF_MAPK_CASCADE	GO:0043408	13	-0.418682	-1.254315	0.186585	0.636952	1.000
578	GOBP_PEPTIDE_HORMONE_SECRETION	GO:0030072	7	-0.494120	-1.256270	0.206494	0.635683	1.000
579	REACTOME_BETA_CATENIN_INDEPENDENT_WNT_SIGNALING	R-RNO-3858494	5	-0.566265	-1.256370	0.188406	0.639779	1.000
580	GOBP_TEMPERATURE_HOMEOSTASIS	GO:0001659	5	-0.560088	-1.259294	0.174221	0.635855	1.000
581	GOBP_GLIOGENESIS	GO:0042063	12	-0.437677	-1.262367	0.169118	0.631376	1.000
582	REACTOME_CLASS_C_3_METABOTROPIC_GLUTAMATE_PHER...	R-RNO-420499	7	-0.507326	-1.266290	0.177866	0.624270	1.000
583	GOBP_POSITIVE_REGULATION_OF_CYSTEINE_TYPE_ENDO...	GO:2001056	7	-0.506670	-1.271326	0.184426	0.614429	1.000
584	GOBP_TRANSMEMBRANE_TRANSPORT	GO:0055085	27	-0.361807	-1.275317	0.158427	0.607070	1.000
585	GOBP_PROTEIN_LOCALIZATION_TO_CELL_PERIPHERY	GO:1990778	12	-0.443422	-1.276364	0.171569	0.608608	1.000
586	GOBP_CELLULAR_RESPONSE_TO_CADMIUM_ION	GO:0071276	5	-0.577965	-1.279612	0.150815	0.603532	1.000
587	GOBP_REGULATION_OF_METAL_ION_TRANSPORT	GO:0010959	6	-0.544639	-1.280391	0.149254	0.605753	1.000
588	GOBP_AXON_DEVELOPMENT	GO:0061564	18	-0.399778	-1.282507	0.164269	0.603810	1.000
589	GOBP_MYELOID_LEUKOCYTE_ACTIVATION	GO:0002274	8	-0.504585	-1.286647	0.163030	0.596468	1.000
590	GOBP_POSITIVE_REGULATION_OF_NEURON_DEATH	GO:1901216	5	-0.567978	-1.293057	0.147383	0.582467	1.000
591	GOBP_DEVELOPMENTAL_MATURATION	GO:0021700	5	-0.585263	-1.299380	0.156425	0.569658	1.000
592	GOBP_PROTEIN_TETRAMERIZATION	GO:0051262	5	-0.582145	-1.307806	0.135246	0.550919	1.000
593	REACTOME_G_ALPHA_Q_SIGNALLING_EVENTS	R-RNO-416476	6	-0.557530	-1.310865	0.142259	0.546887	1.000
594	GOBP_CELL_SURFACE_RECEPTOR_SIGNALING_PATHWAY_I...	GO:1905114	15	-0.420965	-1.317332	0.142518	0.533561	1.000
595	GOBP_REGULATION_OF_HORMONE_SECRETION	GO:0046883	8	-0.516624	-1.335001	0.122876	0.491102	1.000
596	GOBP_REGULATION_OF_TRANSPORTER_ACTIVITY	GO:0032409	17	-0.422429	-1.335864	0.134118	0.492576	1.000
597	GOBP_POSITIVE_REGULATION_OF_MOLECULAR_FUNCTION	GO:0044093	37	-0.368582	-1.339329	0.138239	0.487289	1.000
598	GOBP_ORGANOPHOSPHATE_BIOSYNTHETIC_PROCESS	GO:0090407	5	-0.604715	-1.343017	0.105263	0.481612	1.000
599	GOBP_PEPTIDE_SECRETION	GO:0002790	9	-0.498265	-1.343548	0.120000	0.484215	1.000

Continuation of Table **S6**

	NAME	ID	SIZE	ES	NES	NOM.p.val	FDR.q.val	FWER.p.val
600	GOBP_FATTY_ACID_TRANSPORT	GO:0015908	6	-0.565687	-1.343981	0.120596	0.486915	1.000
601	GOBP_POSTSYNAPSE_ORGANIZATION	GO:0099173	10	-0.496171	-1.344516	0.121437	0.489366	1.000
602	GOBP_ORGANIC_ACID_TRANSPORT	GO:0015849	7	-0.543054	-1.347476	0.107143	0.485777	1.000
603	GOBP_NUCLEOSIDE_PHOSPHATE_BIOSYNTHETIC_PROCESS	GO:1901293	5	-0.604715	-1.353237	0.106145	0.474708	1.000
604	GOBP_G_PROTEIN_COUPLED_GLUTAMATE_RECEPTOR_SIGN...	GO:0007216	8	-0.523834	-1.353774	0.120823	0.477042	1.000
605	GOBP_NUCLEOBASE_CONTAINING_SMALL_MOLECULE_META...	GO:0055086	7	-0.555078	-1.354543	0.110067	0.479075	1.000
606	GOBP_MULTI_MULTICELLULAR_ORGANISM_PROCESS	GO:0044706	8	-0.522736	-1.356030	0.115681	0.479252	1.000
607	GOBP_SYNAPTIC_TRANSMISSION_GLUTAMATERGIC	GO:0035249	13	-0.456545	-1.362213	0.117429	0.467853	1.000
608	GOBP_CELL_PROJECTION_ORGANIZATION	GO:0030030	35	-0.373057	-1.362311	0.111849	0.471638	1.000
609	REACTOME_GPCR_LIGAND_BINDING	R-RNO-500792	8	-0.525887	-1.367952	0.094148	0.461421	1.000
610	REACTOME_G_ALPHA_Z_SIGNALLING_EVENTS	R-RNO-418597	5	-0.602410	-1.372797	0.091938	0.453698	1.000
611	GOBP_POSITIVE_REGULATION_OF_SECRETION	GO:0051047	6	-0.575026	-1.372858	0.094595	0.457443	1.000
612	GOBP_DICARBOXYLIC_ACID_TRANSPORT	GO:0006835	5	-0.624947	-1.373425	0.083333	0.460011	1.000
613	GOBP_MEMBRANE_ORGANIZATION	GO:0061024	12	-0.475936	-1.377446	0.088418	0.453678	1.000
614	GOBP_GLIAL_CELL_DIFFERENTIATION	GO:0010001	10	-0.501228	-1.380678	0.094994	0.449515	1.000
615	REACTOME_L1CAM_INTERACTIONS	R-RNO-373760	5	-0.628147	-1.382939	0.090909	0.447854	1.000
616	GOBP_DENDRITE_MORPHOGENESIS	GO:0048813	7	-0.554950	-1.383141	0.095176	0.451488	1.000
617	REACTOME_EPH_EPHRIN_SIGNALING	R-RNO-2682334	6	-0.582873	-1.383299	0.102180	0.455286	1.000
618	GOBP_SECRETION	GO:0046903	24	-0.409581	-1.383696	0.109070	0.458414	1.000
619	GOBP_ACIDIC_AMINO_ACID_TRANSPORT	GO:0015800	5	-0.624947	-1.384145	0.090014	0.461625	1.000
620	GOBP_DENDRITIC_SPINE_DEVELOPMENT	GO:0060996	6	-0.576691	-1.388516	0.106901	0.454838	1.000
621	GOBP_NEURON_DEVELOPMENT	GO:0048666	34	-0.385072	-1.388806	0.098324	0.458369	1.000
622	GOBP_AMINO_ACID_TRANSPORT	GO:0006865	6	-0.597876	-1.392111	0.083221	0.454392	1.000
623	GOBP_CARBOXYLIC_ACID_TRANSPORT	GO:0046942	6	-0.597876	-1.395719	0.093623	0.449473	1.000
624	GOBP_HORMONE_TRANSPORT	GO:0009914	9	-0.534973	-1.396604	0.088083	0.451522	1.000
625	GOBP_CALCIUM_ION_TRANSMEMBRANE_TRANSPORT	GO:0070588	7	-0.573322	-1.396796	0.084100	0.455509	1.000
626	GOBP_SENSORY_ORGAN_DEVELOPMENT	GO:0007423	8	-0.539262	-1.400108	0.101935	0.451501	1.000
627	GOBP_MONOCARBOXYLIC_ACID_TRANSPORT	GO:0015718	5	-0.624947	-1.400890	0.084469	0.454094	1.000
628	REACTOME_DAG_AND_IP3_SIGNALING	R-RNO-1489509	8	-0.545640	-1.401913	0.079470	0.456195	1.000
629	GOBP_EXOCYTOSIS	GO:0006887	11	-0.490628	-1.407056	0.082174	0.447395	1.000
630	GOBP_NEURON_PROJECTION_ORGANIZATION	GO:0106027	8	-0.551982	-1.407599	0.089032	0.450853	1.000
631	REACTOME_MAPK_FAMILY_SIGNALING_CASCADES	R-RNO-5683057	12	-0.482904	-1.411985	0.079807	0.444243	1.000
632	GOBP_CYTOSOLIC_CALCIUM_ION_TRANSPORT	GO:0060401	7	-0.573322	-1.414494	0.077446	0.443097	1.000
633	GOBP_REGULATION_OF_ION_TRANSPORT	GO:0043269	36	-0.389808	-1.417684	0.089305	0.440361	1.000
634	GOBP_CATION_TRANSMEMBRANE_TRANSPORT	GO:0098655	18	-0.439388	-1.419000	0.081395	0.441844	1.000
635	GOBP_ORGANIC_ANION_TRANSPORT	GO:0015711	6	-0.597876	-1.421787	0.073370	0.439285	1.000
636	GOBP_HOMEOSTATIC_PROCESS	GO:0042592	31	-0.398999	-1.422121	0.071429	0.443275	1.000
637	GOBP_INORGANIC_ION_TRANSMEMBRANE_TRANSPORT	GO:0098660	9	-0.527261	-1.425998	0.062262	0.438213	1.000
638	GOBP_CELLULAR_HOMEOSTASIS	GO:0019725	18	-0.449661	-1.426856	0.072941	0.440681	1.000
639	REACTOME_NERVOUS_SYSTEM_DEVELOPMENT	R-RNO-9675108	14	-0.475167	-1.428879	0.065296	0.440114	1.000
640	GOBP_CALCIUM_ION_TRANSPORT	GO:0006816	11	-0.502948	-1.429027	0.076826	0.445039	1.000
641	REACTOME_TRANSCRIPTIONAL_REGULATION_BY_MECP2	R-RNO-8986944	7	-0.582810	-1.429380	0.063325	0.449413	1.000
642	GOBP_MONOAMINE_TRANSPORT	GO:0015844	5	-0.645358	-1.434131	0.065278	0.443499	1.000

Continuation of Table **S6**

	NAME	ID	SIZE	ES	NES	NOM.p.val	FDR.q.val	FWER.p.val
643	GOBP_IONOTROPIC_GLUTAMATE_RECEPTOR_SIGNALING_P...	GO:0035235	9	-0.537666	-1.437174	0.070000	0.440229	1.000
644	GOBP_REGULATION_OF_VESICLE_MEDIATED_TRANSPORT	GO:0060627	12	-0.495908	-1.437770	0.078205	0.444267	1.000
645	GOBP_CALCIUM_ION_TRANSPORT_INTO_CYTOSOL	GO:0060402	7	-0.573322	-1.438866	0.080537	0.446581	1.000
646	GOBP_CHEMICAL_HOMEOSTASIS	GO:0048878	23	-0.427048	-1.439643	0.084296	0.450087	1.000
647	GOBP_METAL_ION_TRANSPORT	GO:0030001	11	-0.502948	-1.440051	0.069853	0.454836	1.000
648	GOBP_RESPONSE_TO_NITROGEN_COMPOUND	GO:1901698	35	-0.406253	-1.447275	0.076923	0.441411	1.000
649	REACTOME_RAS_ACTIVATION_UPON_CA2_INFLUX_THROUG...	R-HSA-442982	6	-0.612042	-1.457593	0.065246	0.420542	1.000
650	GOBP_POSITIVE_REGULATION_OF_SIGNALING	GO:0023056	34	-0.406980	-1.458933	0.062084	0.422360	1.000
651	GOBP_LONG_TERM_SYNAPTIC_POTENTIATION	GO:0060291	12	-0.505580	-1.469100	0.058096	0.402125	1.000
652	GOBP_NEUROGENESIS	GO:0022008	41	-0.408275	-1.473300	0.057047	0.396711	1.000
653	GOBP_RESPONSE_TO_ABIOTIC_STIMULUS	GO:0009628	27	-0.420002	-1.473553	0.055866	0.401478	1.000
654	GOBP_VESICLE_MEDIATED_TRANSPORT_IN_SYNAPSE	GO:0099003	7	-0.597739	-1.476885	0.058124	0.398696	1.000
655	GOBP_ADENYLATE_CYCLASE_INHIBITING_G_PROTEIN_CO...	GO:0007193	9	-0.553073	-1.477594	0.073383	0.402464	1.000
656	GOBP_EXCITATORY_CHEMICAL_SYNAPTIC_TRANSMISSION	GO:0098976	5	-0.674349	-1.482913	0.043539	0.394982	1.000
657	GOBP_REGULATION_OF_TRANSPORT	GO:0051049	44	-0.409308	-1.483467	0.060307	0.399382	1.000
658	REACTOME_SYNAPTIC_ADHESION_LIKE_MOLECULES	R-RNO-8849932	9	-0.565946	-1.493910	0.035202	0.378258	1.000
659	REACTOME_TRAFFICKING_OF_GLUR2_CONTAINING_AMPA_...	R-RNO-416993	8	-0.584130	-1.494439	0.066225	0.382469	1.000
660	GOBP_RESPONSE_TO_ALCOHOL	GO:0097305	11	-0.532213	-1.496157	0.055696	0.383915	1.000
661	GOBP_METAL_ION_HOMEOSTASIS	GO:0055065	14	-0.497848	-1.499174	0.043742	0.382033	1.000
662	GOBP_EPHRIN_RECEPTOR_SIGNALING_PATHWAY	GO:0048013	5	-0.674042	-1.500579	0.046008	0.384492	1.000
663	GOBP_POSITIVE_REGULATION_OF_CATALYTIC_ACTIVITY	GO:0043085	29	-0.441269	-1.512506	0.043624	0.361039	1.000
664	GOBP_REGULATION_OF_NEUROTRANSMITTER_LEVELS	GO:0001505	7	-0.617669	-1.515815	0.041444	0.358831	1.000
665	GOBP_NEUROTRANSMITTER_TRANSPORT	GO:0006836	7	-0.617669	-1.523413	0.037037	0.347095	1.000
666	GOBP_LOCOMOTORY_BEHAVIOR	GO:0007626	8	-0.578414	-1.531450	0.042581	0.334641	1.000
667	GOBP_MAPK_CASCADE	GO:0000165	18	-0.481307	-1.532718	0.037209	0.337364	1.000
668	GOBP_REGULATION_OF_POSTSYNAPTIC_MEMBRANE_POTEN...	GO:0060078	14	-0.510995	-1.541737	0.035496	0.322805	1.000
669	REACTOME_LONG_TERM_POTENTIATION	R-HSA-9620244	10	-0.564724	-1.545852	0.026718	0.319676	1.000
670	GOBP_MATERNAL_PROCESS_INVOLVED_IN_FEMALE_PREGN...	GO:0060135	5	-0.689626	-1.546167	0.033520	0.324773	1.000
671	REACTOME_NEUREXINS_AND_NEUROLIGINS	R-RNO-6794361	9	-0.583649	-1.553540	0.023622	0.314913	1.000
672	REACTOME_INTRACELLULAR_SIGNALING_BY_SECOND_MES...	R-RNO-9006925	15	-0.508988	-1.557504	0.033175	0.312030	1.000
673	GOBP_PROTEIN_CONTAINING_COMPLEX_SUBUNIT_ORGANI...	GO:0043933	15	-0.514730	-1.562909	0.029976	0.305645	1.000
674	GOBP_HEAD_DEVELOPMENT	GO:0060322	23	-0.459542	-1.562981	0.036047	0.311515	1.000
675	GOBP_NEUROTRANSMITTER_SECRETION	GO:0007269	6	-0.658537	-1.573744	0.031662	0.294132	1.000
676	REACTOME_NEGATIVE_REGULATION_OF_NMDA_RECEPTOR_...	R-HSA-961732	8	-0.609517	-1.581282	0.024485	0.283479	1.000
677	REACTOME_ASSEMBLY_AND_CELL_SURFACE_PRESENTATIO...	R-RNO-9609736	8	-0.609517	-1.585045	0.023316	0.281518	1.000
678	REACTOME_PTEN_REGULATION	R-RNO-6807070	5	-0.711373	-1.589450	0.025956	0.278012	1.000
679	GOBP_CENTRAL_NERVOUS_SYSTEM_DEVELOPMENT	GO:0007417	27	-0.459423	-1.595509	0.017026	0.271078	1.000
680	REACTOME_CA_DEPENDENT_EVENTS	R-RNO-111996	8	-0.625000	-1.605423	0.024235	0.256591	1.000
681	GOBP_REGULATION_OF_MEMBRANE_POTENTIAL	GO:0042391	17	-0.504949	-1.608057	0.025731	0.256983	1.000
682	GOBP_ENDOCRINE_SYSTEM_DEVELOPMENT	GO:0035270	6	-0.686349	-1.614611	0.023035	0.249867	1.000
683	GOBP_REGULATION_OF_CYTOSOLIC_CALCIUM_ION_CONCE...	GO:0051480	13	-0.548547	-1.624236	0.020631	0.236795	1.000
684	GOBP_DIVALENT_INORGANIC_CATION_HOMEOSTASIS	GO:0072507	13	-0.548547	-1.626426	0.016867	0.238239	1.000
685	GOBP_REGULATION_OF_CATION_TRANSMEMBRANE_TRANSPORT	GO:1904062	16	-0.522452	-1.630347	0.020457	0.237078	1.000

Continuation of Table **S6**

	NAME	ID	SIZE	ES	NES	NOM.p.val	FDR.q.val	FWER.p.val
686	GOBP_ADENYLATE_CYCLASE_MODULATING_G_PROTEIN_CO...	GO:0007188	11	-0.575197	-1.631949	0.016049	0.239970	1.000
687	GOBP_REGULATION_OF_NEURONAL_SYNAPTIC_PLASTICITY	GO:0048168	7	-0.671868	-1.674084	0.006757	0.174043	0.998
688	GOBP_ION_HOMEOSTASIS	GO:0050801	16	-0.534390	-1.674562	0.010883	0.177972	0.998
689	REACTOME_OPIOID_SIGNALLING	R-RNO-111885	12	-0.578947	-1.675804	0.016393	0.180725	0.998
690	GOBP_REGULATION_OF_SECRETION	GO:0051046	14	-0.552200	-1.683909	0.007168	0.174410	0.997
691	REACTOME_PROTEIN_PROTEIN_INTERACTIONS_AT_SYNAPSES	R-RNO-6794362	12	-0.587224	-1.700171	0.011307	0.155660	0.994
692	GOBP_COGNITION	GO:0050890	28	-0.488629	-1.713015	0.009091	0.144083	0.992
693	GOBP_REGULATION_OF_CATION_CHANNEL_ACTIVITY	GO:2001257	14	-0.574871	-1.714200	0.013018	0.147328	0.991
694	GOBP_ASSOCIATIVE_LEARNING	GO:0008306	7	-0.690872	-1.714581	0.006757	0.151709	0.991
695	GOBP_NERVOUS_SYSTEM_PROCESS	GO:0050877	38	-0.472666	-1.715893	0.006593	0.155510	0.990
696	REACTOME_G_PROTEIN_MEDIATED_EVENTS	R-RNO-112040	9	-0.632911	-1.717259	0.007595	0.159273	0.989
697	GOBP_G_PROTEIN_COUPLED_RECEPTOR_SIGNALING_PATHWAY	GO:0007186	18	-0.547868	-1.728097	0.014286	0.149059	0.981
698	REACTOME_CREB1_PHOSPHORYLATION_THROUGH_NMDA_RE...	R-RNO-442742	8	-0.667485	-1.730324	0.006579	0.151196	0.979
699	GOBP_CATION_TRANSPORT	GO:0006812	25	-0.507066	-1.732377	0.012687	0.154251	0.979
700	GOBP_REGULATION_OF_SIGNALING_RECEPTOR_ACTIVITY	GO:0010469	14	-0.570295	-1.734783	0.010856	0.156608	0.978
701	GOBP_REGULATION_OF_NMDA_RECEPTOR_ACTIVITY	GO:2000310	10	-0.643207	-1.738805	0.009988	0.157851	0.975
702	GOBP_CELLULAR_ION_HOMEOSTASIS	GO:0006873	14	-0.586959	-1.749861	0.008373	0.148060	0.967
703	GOBP_POSITIVE_REGULATION_OF_SYNAPTIC_TRANSMISSION	GO:0050806	15	-0.568107	-1.752610	0.006024	0.150662	0.961
704	GOBP_GLUTAMATE_RECEPTOR_SIGNALING_PATHWAY	GO:0007215	17	-0.547291	-1.760493	0.008304	0.146765	0.952
705	GOBP_REGULATION_OF_NEUROTRANSMITTER_TRANSPORT	GO:0051588	6	-0.738216	-1.764317	0.006729	0.149277	0.948
706	REACTOME_UNBLOCKING_OF_NMDA_RECEPTORS_GLUTAMAT...	R-RNO-438066	12	-0.603836	-1.772242	0.004944	0.146428	0.940
707	REACTOME_TRAFFICKING_OF_AMPA_RECEPTORS	R-RNO-399719	11	-0.612620	-1.772307	0.010204	0.154563	0.940
708	GOBP_REGULATION_OF_NEUROTRANSMITTER_RECEPTOR_A...	GO:0099601	13	-0.605535	-1.792867	0.002413	0.133359	0.903
709	GOBP_SYNAPTIC_SIGNALING	GO:0099536	47	-0.497587	-1.819590	0.005507	0.110527	0.848
710	GOBP_SIGNAL_RELEASE	GO:0023061	14	-0.606604	-1.826460	0.003745	0.110375	0.830
711	GOBP_SENSORY_PERCEPTION_OF_PAIN	GO:0019233	5	-0.821836	-1.827264	0.002869	0.117237	0.829
712	REACTOME_SIGNALING_BY_GPCR	R-RNO-372790	22	-0.561404	-1.850861	0.003501	0.098777	0.748
713	GOBP_LEARNING	GO:0007612	13	-0.623505	-1.856330	0.003676	0.100764	0.722
714	GOBP_BEHAVIOR	GO:0007610	33	-0.524783	-1.866342	0.002222	0.100165	0.687
715	REACTOME_ACTIVATION_OF_NMDA_RECEPTORS_AND_POST...	R-RNO-442755	16	-0.606031	-1.902437	0.002331	0.075281	0.550
716	GOBP_REGULATION_OF_SYNAPTIC_PLASTICITY	GO:0048167	21	-0.581217	-1.925688	0.001145	0.063086	0.455
717	REACTOME_G_ALPHA_I_SIGNALLING_EVENTS	R-RNO-418594	18	-0.611447	-1.954176	0.000000	0.051105	0.361
718	GOBP_CELL_CELL_SIGNALING	GO:0007267	56	-0.546936	-1.970412	0.000000	0.047453	0.304
719	GOBP_SENSORY_PERCEPTION	GO:0007600	9	-0.748452	-1.978668	0.001295	0.050751	0.278
720	GOBP_REGULATION_OF_TRANS_SYNAPTIC_SIGNALING	GO:0099177	40	-0.550881	-2.015969	0.000000	0.036091	0.183
721	GOBP_RESPONSE_TO_ETHANOL	GO:0045471	5	-0.920693	-2.053963	0.001368	0.029496	0.128
722	REACTOME_NEUROTRANSMITTER_RECEPTORS_AND_POSTSY...	R-RNO-112314	22	-0.635096	-2.107194	0.000000	0.019659	0.066
723	REACTOME_TRANSMISSION_ACROSS_CHEMICAL_SYNAPSES	R-RNO-112315	23	-0.647100	-2.226893	0.000000	0.004021	0.009
724	REACTOME_NEURONAL_SYSTEM	R-RNO-112316	26	-0.658596	-2.307014	0.000000	0.003537	0.004

End of Table

Supplementary Table S7: SC caudal segment late (45 DPL peak) profile GSEA results.

Begin of Table **S7**

	NAME	ID	SIZE	ES	NES	NOM.p.val	FDR.q.val	FWER.p.val
0	GOBP_REGULATION_OF_TRANSPORT	GO:0051049	44	0.474193	1.889698	0.003604	1.000000	0.789
1	GOBP_REGULATION_OF_NMDA_RECEPTOR_ACTIVITY	GO:2000310	10	0.663015	1.875735	0.003906	0.697381	0.829
2	REACTOME_BETA_CATENIN_INDEPENDENT_WNT_SIGNALING	R-RNO-3858494	5	0.831325	1.867099	0.009560	0.499257	0.853
3	REACTOME_TRAFFICKING_OF_AMPA_RECEPTORS	R-RNO-399719	11	0.586301	1.760962	0.015444	0.925434	0.990
4	GOBP_POSITIVE_REGULATION_OF_PHOSPHATIDYLINOSIT...	GO:0014068	5	0.760258	1.745467	0.009862	0.828830	0.998
5	GOBP_REGULATION_OF_SECRETION	GO:0051046	14	0.559599	1.735413	0.011450	0.740975	0.999
6	REACTOME_CA_DEPENDENT_EVENTS	R-RNO-111996	8	0.634228	1.682862	0.025591	0.927006	1.000
7	GOBP_REGULATION_OF_ION_TRANSPORT	GO:0043269	36	0.425834	1.674123	0.014440	0.861662	1.000
8	GOBP_MULTICELLULAR_ORGANISMAL_RESPONSE_TO_STRESS	GO:0033555	5	0.725926	1.662167	0.023438	0.835798	1.000
9	GOBP_REGULATION_OF_PHOSPHATIDYLINOSITOL_3_KINA...	GO:0014066	6	0.690108	1.649739	0.035055	0.817130	1.000
10	REACTOME_TRAFFICKING_OF_GLUR2_CONTAINING_AMPA_...	R-RNO-416993	8	0.622691	1.635950	0.022312	0.814072	1.000
11	GOBP_SIGNAL_RELEASE	GO:0023061	14	0.507187	1.593127	0.028736	0.977330	1.000
12	GOBP_NUCLEOSIDE_PHOSPHATE_BIOSYNTHETIC_PROCESS	GO:1901293	5	0.695524	1.581219	0.034682	0.969315	1.000
13	REACTOME_NEUROTRANSMITTER_RECEPTORS_AND_POSTSY...	R-RNO-112314	22	0.443452	1.577878	0.041353	0.916988	1.000
14	GOBP_REGULATION_OF_SYNAPTIC_PLASTICITY	GO:0048167	21	0.446175	1.565029	0.041293	0.922754	1.000
15	GOBP_ORGANOPHOSPHATE_BIOSYNTHETIC_PROCESS	GO:0090407	5	0.695524	1.564679	0.049020	0.866720	1.000
16	REACTOME_TRANSMISSION_ACROSS_CHEMICAL_SYNAPSES	R-RNO-112315	23	0.425414	1.555688	0.028302	0.857711	1.000
17	GOBP_REGULATION_OF_ENDOCYTOSIS	GO:0030100	6	0.625754	1.524679	0.072614	0.964385	1.000
18	GOBP_NEURON_DIFFERENTIATION	GO:0030182	37	0.380794	1.498858	0.039146	1.000000	1.000
19	GOBP_REGULATION_OF_VESICLE_MEDIATED_TRANSPORT	GO:0060627	12	0.491614	1.475037	0.095517	1.000000	1.000
20	GOBP_POSITIVE_REGULATION_OF_PEPTIDYL_TYROSINE_...	GO:0050731	8	0.568632	1.470524	0.091262	1.000000	1.000
21	REACTOME_ACTIVATION_OF_NMDA_RECEPTORS_AND_POST...	R-RNO-442755	16	0.445542	1.466909	0.077381	1.000000	1.000
22	GOBP_POSITIVE_REGULATION_OF_ORGANELLE_ORGANIZA...	GO:0010638	10	0.516408	1.456897	0.081633	1.000000	1.000
23	GOBP_ENDOCYTOSIS	GO:0006897	8	0.561568	1.456420	0.096457	1.000000	1.000
24	GOBP_TAXIS	GO:0042330	14	0.445904	1.450670	0.077710	1.000000	1.000
25	REACTOME_NEURONAL_SYSTEM	R-RNO-112316	26	0.388813	1.445383	0.062030	1.000000	1.000
26	GOBP_DEVELOPMENTAL_MATURATION	GO:0021700	5	0.644710	1.441076	0.101167	0.989905	1.000
27	GOBP_REGULATION_OF_NEUROTRANSMITTER_TRANSPORT	GO:0051588	6	0.607224	1.436559	0.108911	0.975692	1.000
28	GOBP_INSULIN_SECRETION	GO:0030073	7	0.563899	1.429114	0.085828	0.975825	1.000
29	GOBP_PEPTIDE_HORMONE_SECRETION	GO:0030072	7	0.563899	1.419962	0.116466	0.983538	1.000
30	GOBP_NEGATIVE_REGULATION_OF_CELL_PROJECTION_OR...	GO:0031345	5	0.638554	1.417892	0.112167	0.960695	1.000
31	REACTOME_DAG_AND_IP3_SIGNALING	R-RNO-1489509	8	0.542408	1.416519	0.089866	0.936074	1.000
32	REACTOME_G_PROTEIN_MEDIATED_EVENTS	R-RNO-112040	9	0.518577	1.415466	0.111111	0.912166	1.000
33	GOBP_CYTOSKELETON_ORGANIZATION	GO:0007010	11	0.469657	1.409864	0.099426	0.908896	1.000
34	GOBP_SECRETION	GO:0046903	24	0.388010	1.406475	0.095149	0.896980	1.000
35	GOBP_REGULATION_OF_PEPTIDE_SECRETION	GO:0002791	7	0.549880	1.405874	0.100386	0.874777	1.000
36	GOBP_NEUROTRANSMITTER_TRANSPORT	GO:0006836	7	0.544502	1.402483	0.105263	0.864331	1.000
37	GOBP_REGULATION_OF_NEUROTRANSMITTER_LEVELS	GO:0001505	7	0.544502	1.397612	0.130518	0.859994	1.000
38	GOBP_POSITIVE_REGULATION_OF_PROTEIN_TYROSINE_K...	GO:0061098	5	0.610675	1.392818	0.118321	0.856408	1.000
39	GOBP_POSITIVE_REGULATION_OF_INTRACELLULAR_SIGN...	GO:1902533	14	0.437784	1.384024	0.097606	0.869543	1.000
40	GOBP_REGULATION_OF_PEPTIDE_HORMONE_SECRETION	GO:0090276	6	0.583321	1.375093	0.130769	0.883623	1.000

Continuation of Table **S7**

	NAME	ID	SIZE	ES	NES	NOM.p.val	FDR.q.val	FWER.p.val
41	GOBP_REGULATION_OF_PEPTIDYL_TYROSINE_PHOSPHORY...	GO:0050730	9	0.491862	1.372482	0.123753	0.872647	1.000
42	GOBP_REGULATION_OF_RECEPTOR_MEDIATED_ENDOCYTOSIS	GO:0048259	5	0.607019	1.366538	0.135397	0.874096	1.000
43	GOBP_NEUROGENESIS	GO:0022008	41	0.339989	1.360091	0.108893	0.878318	1.000
44	REACTOME_G_ALPHA_Z_SIGNALLING_EVENTS	R-RNO-418597	5	0.599327	1.354861	0.131179	0.877576	1.000
45	GOBP_REGULATION_OF_PROTEIN_TYROSINE_KINASE_ACT...	GO:0061097	5	0.610675	1.347464	0.157993	0.887397	1.000
46	GOBP_REGULATION_OF_GTPASE_ACTIVITY	GO:0043087	5	0.598252	1.346646	0.150198	0.871155	1.000
47	GOBP_NEURON_MIGRATION	GO:0001764	5	0.598765	1.343552	0.139489	0.863954	1.000
48	GOBP_POSITIVE_REGULATION_OF_MAPK_CASCADE	GO:0043410	9	0.487103	1.343002	0.137363	0.847844	1.000
49	GOBP_NEGATIVE_REGULATION_OF_NEURON_APOPTOTIC_P...	GO:0043524	8	0.504369	1.322774	0.143426	0.904410	1.000
50	GOBP_NEUROTRANSMITTER_SECRETION	GO:0007269	6	0.548844	1.321194	0.156250	0.892574	1.000
51	GOBP_PEPTIDYL_TYROSINE_MODIFICATION	GO:0018212	10	0.467872	1.320888	0.153696	0.876654	1.000
52	GOBP_PHOSPHATIDYLINOSITOL_3_KINASE_SIGNALING	GO:0014065	7	0.528379	1.320437	0.143426	0.861658	1.000
53	GOBP_RECEPTOR_METABOLIC_PROCESS	GO:0043112	5	0.586000	1.316186	0.186180	0.860001	1.000
54	REACTOME_SIGNALING_BY_WNT	R-RNO-195721	7	0.517309	1.311882	0.165339	0.858872	1.000
55	GOBP_PEPTIDE_SECRETION	GO:0002790	9	0.477623	1.306499	0.179630	0.861483	1.000
56	REACTOME_TRANSCRIPTIONAL_REGULATION_BY_MECP2	R-RNO-8986944	7	0.519248	1.300339	0.170000	0.867880	1.000
57	GOBP_CELLULAR_COMPONENT_MAINTENANCE	GO:0043954	5	0.584374	1.299695	0.171598	0.855130	1.000
58	GOBP_POSITIVE_REGULATION_OF_CELLULAR_COMPONENT...	GO:0051130	24	0.365521	1.297990	0.167910	0.846191	1.000
59	GOBP_RECEPTOR_INTERNALIZATION	GO:0031623	5	0.586000	1.292789	0.182353	0.849365	1.000
60	GOBP_DENDRITE_DEVELOPMENT	GO:0016358	10	0.452142	1.292669	0.180608	0.835940	1.000
61	GOBP_REGULATION_OF_CYTOSKELETON_ORGANIZATION	GO:0051493	5	0.567797	1.291066	0.179283	0.827999	1.000
62	GOBP_NEURON_DEVELOPMENT	GO:0048666	34	0.327568	1.286555	0.136525	0.829158	1.000
63	GOBP_POSITIVE_REGULATION_OF_LONG_TERM_SYNAPTIC...	GO:1900273	5	0.579696	1.286023	0.192913	0.818163	1.000
64	GOBP_SYNAPSE_ORGANIZATION	GO:0050808	17	0.390541	1.285465	0.167897	0.807278	1.000
65	GOBP_NEURON_PROJECTION_GUIDANCE	GO:0097485	9	0.467153	1.284936	0.178908	0.796554	1.000
66	GOBP_INTRACELLULAR_TRANSPORT	GO:0046907	10	0.449779	1.279357	0.171429	0.802230	1.000
67	GOBP_POSITIVE_REGULATION_OF_NEURON_PROJECTION_...	GO:0010976	5	0.569975	1.273067	0.204159	0.810015	1.000
68	GOBP_REGULATION_OF_PEPTIDE_TRANSPORT	GO:0090087	10	0.447267	1.263074	0.197368	0.829689	1.000
69	GOBP_REGULATION_OF_SYNAPSE_STRUCTURE_OR_ACTIVITY	GO:0050803	11	0.431532	1.257060	0.183236	0.836068	1.000
70	GOBP_RECEPTOR_MEDIATED_ENDOCYTOSIS	GO:0006898	6	0.530409	1.251695	0.212598	0.841390	1.000
71	GOBP_ACTIN_FILAMENT_BASED_PROCESS	GO:0030029	7	0.496472	1.249611	0.213052	0.836102	1.000
72	GOBP_NEGATIVE_REGULATION_OF_NEURON_DEATH	GO:1901215	11	0.433861	1.246519	0.202756	0.833888	1.000
73	GOBP_RESPONSE_TO_STARVATION	GO:0042594	5	0.544196	1.244306	0.225141	0.828896	1.000
74	GOBP_CELL_MIGRATION	GO:0016477	22	0.348876	1.234305	0.197158	0.850003	1.000
75	GOBP_LOCOMOTION	GO:0040011	31	0.323832	1.231782	0.179584	0.846024	1.000
76	GOBP_INOSITOL_LIPID_MEDIATED_SIGNALING	GO:0048017	8	0.462398	1.230176	0.210421	0.839775	1.000
77	GOBP_REGULATION_OF_CATION_CHANNEL_ACTIVITY	GO:2001257	14	0.385089	1.228460	0.181474	0.834055	1.000
78	GOBP_HORMONE_TRANSPORT	GO:0009914	9	0.441635	1.226762	0.215686	0.828657	1.000
79	GOBP_POSITIVE_REGULATION_OF_LIPASE_ACTIVITY	GO:0060193	5	0.536025	1.213444	0.228102	0.859179	1.000
80	GOBP_CELL_PROJECTION_ORGANIZATION	GO:0030030	35	0.308541	1.210015	0.196364	0.859001	1.000
81	GOBP_DICARBOXYLIC_ACID_TRANSPORT	GO:0006835	5	0.540301	1.208936	0.231237	0.851651	1.000
82	REACTOME_UNBLOCKING_OF_NMDA_RECEPTORS_GLUTAMAT...	R-RNO-438066	12	0.399865	1.206617	0.262042	0.848212	1.000
83	GOBP_ACIDIC_AMINO_ACID_TRANSPORT	GO:0015800	5	0.540301	1.206126	0.241379	0.839595	1.000

Continuation of Table **S7**

	NAME	ID	SIZE	ES	NES	NOM.p.val	FDR.q.val	FWER.p.val
84	GOBP_MONOCARBOXYLIC_ACID_TRANSPORT	GO:0015718	5	0.540301	1.205875	0.239216	0.830418	1.000
85	GOBP_FOREBRAIN_DEVELOPMENT	GO:0030900	10	0.431473	1.205413	0.237383	0.822064	1.000
86	GOBP_REGULATION_OF_PHOSPHOLIPASE_C_ACTIVITY	GO:1900274	5	0.536025	1.203546	0.258449	0.818090	1.000
87	GOBP_CELLULAR_RESPONSE_TO_STARVATION	GO:0009267	5	0.544196	1.202045	0.232902	0.813012	1.000
88	GOBP_REGULATION_OF_NEURONAL_SYNAPTIC_PLASTICITY	GO:0048168	7	0.467012	1.194159	0.246602	0.826569	1.000
89	GOBP_REGULATION_OF_HORMONE_SECRETION	GO:0046883	8	0.456692	1.189201	0.272556	0.831670	1.000
90	GOBP_PEPTIDYL_AMINO_ACID_MODIFICATION	GO:0018193	22	0.337516	1.185001	0.246429	0.834955	1.000
91	GOBP_HEAD_DEVELOPMENT	GO:0060322	23	0.333965	1.178692	0.265306	0.843617	1.000
92	GOBP_DENDRITE_MORPHOGENESIS	GO:0048813	7	0.459567	1.174279	0.270059	0.847240	1.000
93	GOBP_NEUROTROPHIN_TRK_RECEPTOR_SIGNALING_PATHWAY	GO:0048011	6	0.482493	1.163980	0.270270	0.867239	1.000
94	GOBP_GLIOGENESIS	GO:0042063	12	0.385540	1.157432	0.282908	0.877059	1.000
95	REACTOME_OPIOID_SIGNALLING	R-RNO-111885	12	0.374912	1.152633	0.279383	0.882173	1.000
96	GOBP_MONOAMINE_TRANSPORT	GO:0015844	5	0.522261	1.147676	0.306391	0.887151	1.000
97	GOBP_REGULATION_OF_MUSCLE_SYSTEM_PROCESS	GO:0090257	8	0.432231	1.146412	0.283809	0.881638	1.000
98	GOBP_MUSCLE_ADAPTATION	GO:0043500	5	0.502323	1.144917	0.288136	0.877039	1.000
99	GOBP_REGULATION_OF_ORGANELLE_ORGANIZATION	GO:0033043	14	0.357174	1.140505	0.281536	0.880361	1.000
100	GOBP_NEUROTROPHIN_SIGNALING_PATHWAY	GO:0038179	6	0.482493	1.136239	0.293878	0.884652	1.000
101	GOBP_CELL_MORPHOGENESIS_INVOLVED_IN_DIFFERENTI...	GO:0000904	22	0.314961	1.130659	0.280443	0.892069	1.000
102	REACTOME_SIGNALING_BY_NTRK2_TRKB	R-RNO-9006115	6	0.475849	1.125405	0.317930	0.898172	1.000
103	GOBP_BIOLOGICAL_ADHESION	GO:0022610	19	0.330123	1.121403	0.314885	0.900991	1.000
104	GOBP_REGULATION_OF_MUSCLE_ADAPTATION	GO:0043502	5	0.502323	1.121282	0.321012	0.892711	1.000
105	GOBP_IONOTROPIC_GLUTAMATE_RECEPTOR_SIGNALING_P...	GO:0035235	9	0.404500	1.113204	0.318182	0.907753	1.000
106	GOBP_REGULATION_OF_PHOSPHOLIPASE_ACTIVITY	GO:0010517	6	0.451362	1.108617	0.332669	0.912161	1.000
107	GOBP_POSITIVE_REGULATION_OF_CELLULAR_COMPONENT...	GO:0044089	9	0.402070	1.108345	0.337786	0.904541	1.000
108	GOBP_CELL_MORPHOGENESIS_INVOLVED_IN_NEURON_DIF...	GO:0048667	22	0.314961	1.107255	0.323017	0.899540	1.000
109	GOBP_POSITIVE_REGULATION_OF_TRANSPORT	GO:0051050	26	0.303492	1.107022	0.309055	0.891967	1.000
110	REACTOME_NERVOUS_SYSTEM_DEVELOPMENT	R-RNO-9675108	14	0.351735	1.104813	0.336614	0.890215	1.000
111	GOBP_REGULATION_OF_NEUROTRANSMITTER_RECEPTOR_A...	GO:0099601	13	0.361988	1.101090	0.349515	0.892293	1.000
112	GOBP_REGULATION_OF_LIPASE_ACTIVITY	GO:0060191	6	0.451362	1.098449	0.353430	0.891697	1.000
113	GOBP_NEURON_PROJECTION_ORGANIZATION	GO:0106027	8	0.410714	1.095202	0.340741	0.892863	1.000
114	GOBP_CARBOHYDRATE_DERIVATIVE_BIOSYNTHETIC_PROCESS	GO:1901137	5	0.488884	1.093777	0.352490	0.888770	1.000
115	GOBP_REGULATION_OF_ANION_TRANSPORT	GO:0044070	20	0.311361	1.089931	0.363139	0.891604	1.000
116	GOBP_CELL_JUNCTION_ORGANIZATION	GO:0034330	19	0.320351	1.089017	0.318761	0.886327	1.000
117	GOBP_CELLULAR_COMPONENT_MORPHOGENESIS	GO:0032989	25	0.294572	1.087399	0.338374	0.883334	1.000
118	GOBP_LIPID_EXPORT_FROM_CELL	GO:0140353	5	0.486862	1.087141	0.343874	0.876610	1.000
119	GOBP_REGULATION_OF_ANATOMICAL_STRUCTURE_MORPHO...	GO:0022603	17	0.322232	1.085772	0.344828	0.872977	1.000
120	GOBP_PEPTIDYL_SERINE_MODIFICATION	GO:0018209	12	0.356140	1.080782	0.356061	0.878651	1.000
121	GOBP_REGULATION_OF_CELLULAR_COMPONENT_BIOGENESIS	GO:0044087	10	0.382421	1.071322	0.347092	0.896404	1.000
122	GOBP_CELL_MORPHOGENESIS	GO:0000902	25	0.294572	1.068843	0.373002	0.895596	1.000
123	GOBP_REGULATION_OF_RESPONSE_TO_EXTERNAL_STIMULUS	GO:0032101	15	0.329193	1.061694	0.379648	0.907761	1.000
124	GOBP_RHYTHMIC_PROCESS	GO:0048511	15	0.339938	1.059370	0.354167	0.907211	1.000
125	GOBP_REGULATION_OF_LONG_TERM_SYNAPTIC_POTENTIA...	GO:1900271	6	0.437012	1.057498	0.367387	0.905000	1.000
126	GOBP_NUCLEOBASE_CONTAINING_SMALL_MOLECULE_META...	GO:0055086	7	0.419288	1.056727	0.384466	0.899863	1.000

Continuation of Table **S7**

	NAME	ID	SIZE	ES	NES	NOM.p.val	FDR.q.val	FWER.p.val
127	REACTOME_LONG_TERM_POTENTIATION	R-HSA-9620244	10	0.368356	1.055905	0.390196	0.894971	1.000
128	GOBP_POSITIVE_REGULATION_OF_LOCOMOTION	GO:0040017	11	0.359513	1.055227	0.382524	0.889771	1.000
129	GOBP_REGULATION_OF_CELLULAR_COMPONENT_MOVEMENT	GO:0051270	15	0.330829	1.052534	0.387037	0.890358	1.000
130	GOBP_REGULATION_OF_TRANS_SYNAPTIC_SIGNALING	GO:0099177	40	0.261689	1.045476	0.392473	0.901945	1.000
131	GOBP_CELL_PART_MORPHOGENESIS	GO:0032990	25	0.294572	1.042650	0.415560	0.902186	1.000
132	GOBP_NEGATIVE_REGULATION_OF_CELLULAR_COMPONENT...	GO:0051129	11	0.353338	1.041928	0.428571	0.896953	1.000
133	GOBP_LONG_TERM_SYNAPTIC_POTENTIATION	GO:0060291	12	0.353374	1.038738	0.404494	0.898441	1.000
134	GOBP_POSITIVE_REGULATION_OF_PROTEIN_KINASE_ACT...	GO:0045860	18	0.303422	1.037906	0.426326	0.893967	1.000
135	REACTOME_EXTRACELLULAR_MATRIX_ORGANIZATION	R-RNO-1474244	5	0.465199	1.033787	0.419660	0.897985	1.000
136	GOBP_DNA_METABOLIC_PROCESS	GO:0006259	6	0.430250	1.032435	0.413255	0.895152	1.000
137	GOBP_CARBOXYLIC_ACID_TRANSPORT	GO:0046942	6	0.427394	1.030694	0.406910	0.893121	1.000
138	GOBP_AMINO_ACID_TRANSPORT	GO:0006865	6	0.427394	1.024442	0.403194	0.902651	1.000
139	GOBP_ORGANIC_ANION_TRANSPORT	GO:0015711	6	0.427394	1.024186	0.413442	0.896865	1.000
140	GOBP_AXON_DEVELOPMENT	GO:0061564	18	0.299339	1.020975	0.441860	0.898470	1.000
141	GOBP_PROTEIN_KINASE_B_SIGNALING	GO:0043491	6	0.426799	1.020850	0.421359	0.892488	1.000
142	GOBP_MUSCLE_SYSTEM_PROCESS	GO:0003012	9	0.363704	1.017603	0.431452	0.894329	1.000
143	GOBP_POSITIVE_REGULATION_OF_TRANSFERASE_ACTIVITY	GO:0051347	19	0.294287	1.017496	0.424408	0.888378	1.000
144	REACTOME_G_ALPHA_I_SIGNALLING_EVENTS	R-RNO-418594	18	0.311993	1.016865	0.418079	0.883828	1.000
145	GOBP_SYNAPTIC_SIGNALING	GO:0099536	47	0.251308	1.016087	0.439153	0.879959	1.000
146	GOBP_MUSCLE_ORGAN_DEVELOPMENT	GO:0007517	9	0.364206	1.015718	0.425094	0.874785	1.000
147	GOBP_PURINE_CONTAINING_COMPOUND_METABOLIC_PROCESS	GO:0072521	6	0.427055	1.012255	0.417457	0.877436	1.000
148	GOBP_CIRCADIAN_RHYTHM	GO:0007623	13	0.325966	1.010584	0.439338	0.875495	1.000
149	GOBP_GLIAL_CELL_DIFFERENTIATION	GO:0010001	10	0.350969	1.005991	0.431298	0.880504	1.000
150	GOBP_CENTRAL_NERVOUS_SYSTEM_DEVELOPMENT	GO:0007417	27	0.264750	0.995892	0.453382	0.899605	1.000
151	GOBP_CENTRAL_NERVOUS_SYSTEM_NEURON_DIFFERENTIA...	GO:0021953	5	0.442387	0.990993	0.443623	0.906285	1.000
152	GOBP_INTRACELLULAR_PROTEIN_TRANSPORT	GO:0006886	8	0.374951	0.987457	0.458647	0.909008	1.000
153	GOBP_DENDRITIC_SPINE_DEVELOPMENT	GO:0060996	6	0.407548	0.983436	0.479087	0.913247	1.000
154	GOBP_PROTEIN_PHOSPHORYLATION	GO:0006468	33	0.253179	0.982686	0.491228	0.909195	1.000
155	GOBP_REGULATION_OF_CELL_CELL_ADHESION	GO:0022407	9	0.358170	0.980487	0.482625	0.908682	1.000
156	GOBP_POSITIVE_REGULATION_OF_CELL_POPULATION_PR...	GO:0008284	21	0.279478	0.975358	0.509434	0.914859	1.000
157	GOBP_ESTABLISHMENT_OF_PROTEIN_LOCALIZATION	GO:0045184	17	0.290231	0.965119	0.481188	0.934053	1.000
158	REACTOME_GPCR_LIGAND_BINDING	R-RNO-500792	8	0.362500	0.960461	0.465070	0.939485	1.000
159	GOBP_REGULATION_OF_SIGNALING_RECEPTOR_ACTIVITY	GO:0010469	14	0.305984	0.958621	0.493230	0.938103	1.000
160	GOBP_GAMETE_GENERATION	GO:0007276	9	0.346877	0.957874	0.487903	0.934140	1.000
161	GOBP_CELLULAR_MACROMOLECULE_CATABOLIC_PROCESS	GO:0044265	5	0.425713	0.956446	0.484112	0.932287	1.000
162	GOBP_REGULATION_OF_VASCULATURE_DEVELOPMENT	GO:1901342	5	0.415410	0.951749	0.506692	0.937684	1.000
163	GOBP_REGULATION_OF_CELL_ACTIVATION	GO:0050865	8	0.370634	0.949812	0.528155	0.936704	1.000
164	GOBP_TELENCEPHALON_DEVELOPMENT	GO:0021537	7	0.373857	0.945290	0.517304	0.941689	1.000
165	GOBP_SYNAPTIC_TRANSMISSION_GLUTAMATERGIC	GO:0035249	13	0.315167	0.943129	0.533582	0.941137	1.000
166	GOBP_PLATELET_ACTIVATION	GO:0030168	6	0.392458	0.941227	0.513627	0.940095	1.000
167	GOBP_NERVOUS_SYSTEM_PROCESS	GO:0050877	38	0.239492	0.940421	0.527103	0.936426	1.000
168	GOBP_REGULATION_OF_NEUROGENESIS	GO:0050767	14	0.302020	0.940375	0.508772	0.931008	1.000
169	GOBP_REGULATION_OF_CELL_DEVELOPMENT	GO:0060284	15	0.291492	0.939479	0.548638	0.927713	1.000

Continuation of Table **S7**

	NAME	ID	SIZE	ES	NES	NOM.p.val	FDR.q.val	FWER.p.val
170	GOBP_POSITIVE_REGULATION_OF_PEPTIDASE_ACTIVITY	GO:0010952	7	0.372933	0.938042	0.533203	0.925641	1.000
171	GOBP_BEHAVIOR	GO:0007610	33	0.243044	0.936297	0.565455	0.924329	1.000
172	GOBP_MAINTENANCE_OF_LOCATION	GO:0051235	7	0.374341	0.932395	0.556886	0.928069	1.000
173	GOBP_POSITIVE_REGULATION_OF_CYSTEINE_TYPE_ENDO...	GO:2001056	7	0.372933	0.930391	0.556797	0.927483	1.000
174	GOBP_NUCLEAR_TRANSPORT	GO:0051169	5	0.411925	0.923876	0.529297	0.937375	1.000
175	GOBP_REGULATION_OF_TRANSPORTER_ACTIVITY	GO:0032409	17	0.277075	0.923842	0.563601	0.932135	1.000
176	GOBP_REGULATION_OF_CELLULAR_RESPONSE_TO_STRESS	GO:0080135	9	0.337636	0.922644	0.540385	0.929685	1.000
177	GOBP_REGULATION_OF_PEPTIDYL_SERINE_PHOSPHORYLA...	GO:0033135	8	0.334193	0.920737	0.540741	0.928779	1.000
178	REACTOME_CLASS_C_3_METABOTROPIC_GLUTAMATE_PHER...	R-RNO-420499	7	0.358025	0.920452	0.547273	0.924190	1.000
179	GOBP_POSTSYNAPSE_ORGANIZATION	GO:0099173	10	0.328984	0.919403	0.560311	0.921404	1.000
180	GOBP_RESPONSE_TO_NERVE_GROWTH_FACTOR	GO:1990089	7	0.364561	0.918029	0.548255	0.919283	1.000
181	GOBP_COGNITION	GO:0050890	28	0.243820	0.916349	0.596330	0.918117	1.000
182	GOBP_SEXUAL_REPRODUCTION	GO:0019953	10	0.325728	0.915149	0.579848	0.915766	1.000
183	GOBP_LIPID_LOCALIZATION	GO:0010876	10	0.326341	0.914022	0.544031	0.913386	1.000
184	GOBP_CELL_CYCLE_PROCESS	GO:0022402	11	0.309162	0.913205	0.543925	0.910305	1.000
185	GOBP_CELLULAR_HOMEOSTASIS	GO:0019725	18	0.269594	0.905614	0.583181	0.921848	1.000
186	GOBP_NEURON_APOPTOTIC_PROCESS	GO:0051402	12	0.301217	0.904719	0.577821	0.919101	1.000
187	GOBP_CELLULAR_GLUCOSE_HOMEOSTASIS	GO:0001678	5	0.401787	0.903289	0.564797	0.917454	1.000
188	GOBP_NEGATIVE_REGULATION_OF_CELL_DEATH	GO:0060548	22	0.251391	0.900058	0.613553	0.919627	1.000
189	GOBP_PROTEIN_TETRAMERIZATION	GO:0051262	5	0.413626	0.898465	0.586694	0.918128	1.000
190	GOBP_POSITIVE_REGULATION_OF_PROTEIN_PHOSPHORYL...	GO:0001934	22	0.248824	0.896823	0.613553	0.916808	1.000
191	GOBP_POSITIVE_REGULATION_OF_PEPTIDYL_SERINE_PH...	GO:0033138	8	0.334193	0.886341	0.599222	0.934460	1.000
192	GOBP_POSITIVE_REGULATION_OF_PROTEIN_MODIFICATI...	GO:0031401	22	0.248824	0.884370	0.630713	0.933784	1.000
193	REACTOME_ONCOGENIC_MAPK_SIGNALING	R-HSA-6802957	5	0.393206	0.881970	0.592814	0.934243	1.000
194	GOBP_REGULATION_OF_TRANSFERASE_ACTIVITY	GO:0051338	21	0.252137	0.881283	0.639405	0.930875	1.000
195	GOBP_REGULATION_OF_HORMONE_LEVELS	GO:0010817	12	0.288500	0.879485	0.618774	0.929808	1.000
196	GOBP_PROTEIN_AUTOPHOSPHORYLATION	GO:0046777	5	0.396215	0.879459	0.571705	0.925129	1.000
197	GOBP_PATTERN_SPECIFICATION_PROCESS	GO:0007389	6	0.367403	0.877743	0.604478	0.924122	1.000
198	GOBP_EXOCYTOSIS	GO:0006887	11	0.298701	0.874911	0.606589	0.925410	1.000
199	GOBP_REGIONALIZATION	GO:0003002	6	0.367403	0.873721	0.613208	0.923244	1.000
200	GOBP_REGULATION_OF_CATION_TRANSMEMBRANE_TRANSPORT	GO:1904062	16	0.267303	0.872567	0.600000	0.921057	1.000
201	GOBP_REGULATION_OF_HYDROLASE_ACTIVITY	GO:0051336	22	0.243886	0.871070	0.638298	0.919432	1.000
202	REACTOME_CREB1_PHOSPHORYLATION_THROUGH_NMDA_RE...	R-RNO-442742	8	0.324068	0.868445	0.630058	0.920438	1.000
203	GOBP_REGULATION_OF_RESPONSE_TO_STRESS	GO:0080134	19	0.250805	0.868033	0.620112	0.916752	1.000
204	GOBP_POSITIVE_REGULATION_OF_SYNAPTIC_TRANSMISSION	GO:0050806	15	0.272247	0.867334	0.626894	0.913684	1.000
205	GOBP_REGULATION_OF_PROTEIN_KINASE_ACTIVITY	GO:0045859	21	0.252137	0.866160	0.655303	0.911538	1.000
206	GOBP_POSITIVE_REGULATION_OF_PROTEIN_SERINE_THR...	GO:0071902	9	0.311954	0.859442	0.631263	0.920460	1.000
207	GOBP_NERVE_DEVELOPMENT	GO:0021675	9	0.314140	0.855395	0.642857	0.923722	1.000
208	GOBP_POSITIVE_REGULATION_OF_PHOSPHORUS_METABOL...	GO:0010562	23	0.241394	0.853030	0.687956	0.924206	1.000
209	GOBP_PROTEIN_COMPLEX_OLIGOMERIZATION	GO:0051259	7	0.340791	0.847107	0.642578	0.931357	1.000
210	GOBP_REGULATION_OF_PROTEIN_STABILITY	GO:0031647	5	0.371534	0.843930	0.626016	0.933335	1.000
211	GOBP_REGULATION_OF_BODY_FLUID_LEVELS	GO:0050878	11	0.288916	0.838760	0.696296	0.939037	1.000
212	GOBP_MAPK_CASCADE	GO:0000165	18	0.250087	0.836738	0.696589	0.938760	1.000

Continuation of Table **S7**

	NAME	ID	SIZE	ES	NES	NOM.p.val	FDR.q.val	FWER.p.val
213	GOBP_RESPONSE_TO_ETHANOL	GO:0045471	5	0.367444	0.836215	0.657143	0.935451	1.000
214	GOBP_NEGATIVE_REGULATION_OF_PHOSPHORYLATION	GO:0042326	7	0.330615	0.833684	0.627413	0.936264	1.000
215	REACTOME_INTEGRATION_OF_ENERGY_METABOLISM	R-RNO-163685	5	0.374706	0.830532	0.685981	0.938164	1.000
216	GOBP_CIRCADIAN_REGULATION_OF_GENE_EXPRESSION	GO:0032922	5	0.371419	0.826870	0.664032	0.940922	1.000
217	REACTOME_L1CAM_INTERACTIONS	R-RNO-373760	5	0.365191	0.826100	0.672549	0.938112	1.000
218	GOBP_REGULATION_OF_INTRACELLULAR_SIGNAL_TRANSD...	GO:1902531	21	0.235186	0.825809	0.692759	0.934416	1.000
219	GOBP_PROTEIN_CONTAINING_COMPLEX_SUBUNIT_ORGANI...	GO:0043933	15	0.260947	0.825183	0.680154	0.931297	1.000
220	GOBP_FATTY_ACID_TRANSPORT	GO:0015908	6	0.346687	0.824742	0.675048	0.927823	1.000
221	GOBP_AMYLOID_PRECURSOR_PROTEIN_METABOLIC_PROCESS	GO:0042982	5	0.365158	0.823020	0.676768	0.926959	1.000
222	GOBP_POSITIVE_REGULATION_OF_RESPONSE_TO_EXTERN...	GO:0032103	9	0.305171	0.822880	0.687379	0.923104	1.000
223	GOBP_POSITIVE_REGULATION_OF_SECRETION	GO:0051047	6	0.341464	0.822208	0.672691	0.920092	1.000
224	GOBP_NEGATIVE_REGULATION_OF_NERVOUS_SYSTEM_DEV...	GO:0051961	5	0.369760	0.821600	0.692022	0.917247	1.000
225	GOBP_WOUND_HEALING	GO:0042060	13	0.265244	0.820774	0.686424	0.914767	1.000
226	GOBP_AMYLOID_PRECURSOR_PROTEIN_CATABOLIC_PROCESS	GO:0042987	5	0.365158	0.820667	0.679842	0.910962	1.000
227	GOBP_AMYLOID_BETA_METABOLIC_PROCESS	GO:0050435	5	0.365158	0.815890	0.662362	0.915814	1.000
228	GOBP_REGULATION_OF_CIRCADIAN_RHYTHM	GO:0042752	7	0.323822	0.815228	0.695652	0.913084	1.000
229	GOBP_MITOTIC_CELL_CYCLE	GO:0000278	10	0.283057	0.814593	0.705653	0.910174	1.000
230	GOBP_ORGANIC_HYDROXY_COMPOUND_TRANSPORT	GO:0015850	6	0.342495	0.813897	0.683071	0.907495	1.000
231	REACTOME_RAS_ACTIVATION_UPON_CA2_INFLUX_THROUG...	R-HSA-442982	6	0.334668	0.809383	0.704415	0.911544	1.000
232	GOBP_MEMBRANE_ORGANIZATION	GO:0061024	12	0.265516	0.803861	0.720532	0.917439	1.000
233	GOBP_DENDRITIC_SPINE_MORPHOGENESIS	GO:0060997	5	0.356111	0.793899	0.714286	0.931159	1.000
234	GOBP_CELL_CELL_SIGNALING	GO:0007267	56	0.198110	0.790801	0.802158	0.932513	1.000
235	REACTOME_SIGNALING_BY_NTRKS	R-RNO-166520	18	0.235097	0.787114	0.781076	0.934896	1.000
236	GOBP_ADENYLATE_CYCLASE_INHIBITING_G_PROTEIN_CO...	GO:0007193	5	0.349398	0.786204	0.760700	0.932513	1.000
237	GOBP_POSITIVE_REGULATION_OF_NEUROGENESIS	GO:0050769	12	0.256342	0.782953	0.713755	0.934089	1.000
238	GOBP_POSITIVE_REGULATION_OF_NERVOUS_SYSTEM_DEV...	GO:0051962	12	0.256342	0.782487	0.730916	0.930966	1.000
239	GOBP_NEURON_DEATH	GO:0070997	18	0.233365	0.780036	0.766284	0.931243	1.000
240	GOBP_MULTICELLULAR_ORGANISM_REPRODUCTION	GO:0032504	12	0.262573	0.779268	0.746032	0.928669	1.000
241	GOBP_ADENYLATE_CYCLASE_MODULATING_G_PROTEIN_CO...	GO:0007188	11	0.263417	0.775778	0.759369	0.930842	1.000
242	GOBP_MEMORY	GO:0007613	13	0.257184	0.775553	0.759358	0.927329	1.000
243	GOBP_REGULATION_OF_PROTEIN_PHOSPHORYLATION	GO:0001932	25	0.207762	0.769296	0.809437	0.933750	1.000
244	GOBP_RESPONSE_TO_ACID_CHEMICAL	GO:0001101	6	0.322092	0.765735	0.787004	0.935766	1.000
245	GOBP_RESPONSE_TO_NITROGEN_COMPOUND	GO:1901698	35	0.195049	0.763210	0.793419	0.935845	1.000
246	GOBP_ACTIVATION_OF_MAPK_ACTIVITY	GO:0000187	6	0.321361	0.762326	0.752621	0.933605	1.000
247	GOBP_POSITIVE_REGULATION_OF_CELL_DEVELOPMENT	GO:0010720	12	0.256342	0.759465	0.753846	0.934435	1.000
248	GOBP_NEGATIVE_REGULATION_OF_CELL_DEVELOPMENT	GO:0010721	6	0.317622	0.759359	0.763303	0.930862	1.000
249	GOBP_G_PROTEIN_COUPLED_RECEPTOR_SIGNALING_PATHWAY	GO:0007186	18	0.222495	0.758584	0.805970	0.928319	1.000
250	GOBP_POSITIVE_REGULATION_OF_ION_TRANSPORT	GO:0043270	17	0.226321	0.755754	0.789963	0.928925	1.000
251	GOBP_MALE_GAMETE_GENERATION	GO:0048232	5	0.335701	0.755246	0.767442	0.925993	1.000
252	GOBP_POSITIVE_REGULATION_OF_MAP_KINASE_ACTIVITY	GO:0043406	6	0.321361	0.754392	0.764591	0.923579	1.000
253	GOBP_CHROMATIN_ORGANIZATION	GO:0006325	5	0.342757	0.754165	0.773513	0.920337	1.000
254	GOBP_COAGULATION	GO:0050817	8	0.288796	0.746755	0.770245	0.927997	1.000
255	GOBP_REGULATION_OF_MAPK_CASCADE	GO:0043408	13	0.246107	0.745048	0.788054	0.927042	1.000

Continuation of Table **S7**

	NAME	ID	SIZE	ES	NES	NOM.p.val	FDR.q.val	FWER.p.val
256	REACTOME_SYNAPTIC_ADHESION_LIKE_MOLECULES	R-RNO-8849932	9	0.275180	0.741833	0.779661	0.927974	1.000
257	GOBP_REGULATION_OF_SYNAPTIC_TRANSMISSION_GLUTA...	GO:0051966	12	0.250000	0.739406	0.825095	0.927710	1.000
258	GOBP_REGULATION_OF_LIPID_LOCALIZATION	GO:1905952	5	0.329511	0.739249	0.798058	0.924372	1.000
259	GOBP_GLUTAMATE_RECEPTOR_SIGNALING_PATHWAY	GO:0007215	17	0.222619	0.735311	0.811069	0.926493	1.000
260	GOBP_POSITIVE_REGULATION_OF_CATALYTIC_ACTIVITY	GO:0043085	29	0.195329	0.731969	0.846863	0.927774	1.000
261	GOBP_PEPTIDYL_LYSINE_MODIFICATION	GO:0018205	5	0.323311	0.730193	0.830000	0.926902	1.000
262	GOBP_LONG_TERM_SYNAPTIC_DEPRESSION	GO:0060292	5	0.331897	0.722831	0.804040	0.933872	1.000
263	REACTOME_INTERFERON_SIGNALING	R-RNO-913531	6	0.305091	0.722232	0.804388	0.931079	1.000
264	GOBP_ORGANIC_ACID_TRANSPORT	GO:0015849	7	0.285378	0.721443	0.772201	0.928647	1.000
265	GOBP_CELLULAR_RESPONSE_TO_LIPID	GO:0071396	15	0.227292	0.716275	0.844037	0.932069	1.000
266	GOBP_SENSORY_PERCEPTION	GO:0007600	9	0.266628	0.715119	0.812627	0.930006	1.000
267	GOBP_NEGATIVE_REGULATION_OF_CELL_DIFFERENTIATION	GO:0045596	13	0.226667	0.711885	0.832335	0.930893	1.000
268	GOBP_MYOTUBE_DIFFERENTIATION	GO:0014902	5	0.317712	0.711491	0.852273	0.927980	1.000
269	GOBP_CELLULAR_RESPONSE_TO_NITROGEN_COMPOUND	GO:1901699	20	0.206874	0.710506	0.838420	0.925779	1.000
270	GOBP_NEGATIVE_REGULATION_OF_RESPONSE_TO_EXTERN...	GO:0032102	5	0.312866	0.697960	0.848980	0.938651	1.000
271	GOBP_ORGANOPHOSPHATE_METABOLIC_PROCESS	GO:0019637	8	0.266495	0.695321	0.833992	0.938551	1.000
272	GOBP_REGULATION_OF_PHOSPHORUS_METABOLIC_PROCESS	GO:0051174	26	0.186420	0.692740	0.867993	0.938315	1.000
273	GOBP_POSITIVE_REGULATION_OF_ESTABLISHMENT_OF_P...	GO:1904951	5	0.306104	0.691079	0.880240	0.936872	1.000
274	GOBP_NEGATIVE_REGULATION_OF_DEVELOPMENTAL_PROCESS	GO:0051093	13	0.226667	0.690446	0.858527	0.934257	1.000
275	GOBP_CHROMOSOME_ORGANIZATION	GO:0051276	6	0.293658	0.689974	0.859345	0.931466	1.000
276	GOBP_SENSORY_ORGAN_DEVELOPMENT	GO:0007423	8	0.266230	0.689264	0.829114	0.928990	1.000
277	GOBP_POSITIVE_REGULATION_OF_PROTEIN_METABOLIC_...	GO:0051247	27	0.183729	0.687395	0.879121	0.927904	1.000
278	GOBP_NEGATIVE_REGULATION_OF_SYNAPTIC_TRANSMISSION	GO:0050805	6	0.289438	0.680039	0.851064	0.932858	1.000
279	GOBP_RESPONSE_TO_CALCIUM_ION	GO:0051592	6	0.279779	0.675474	0.867675	0.934761	1.000
280	REACTOME_SIGNALING_BY_GPCR	R-RNO-372790	22	0.189234	0.669443	0.891071	0.937887	1.000
281	GOBP_SKELETAL_MUSCLE_ORGAN_DEVELOPMENT	GO:0060538	5	0.302006	0.665249	0.846881	0.939023	1.000
282	GOBP_G_PROTEIN_COUPLED_GLUTAMATE_RECEPTOR_SIGN...	GO:0007216	8	0.250000	0.663957	0.882023	0.937046	1.000
283	GOBP_SMALL_MOLECULE_BIOSYNTHETIC_PROCESS	GO:0044283	5	0.292901	0.663176	0.901210	0.934713	1.000
284	GOBP_DEFENSE_RESPONSE_TO_OTHER_ORGANISM	GO:0098542	9	0.239715	0.652726	0.908549	0.942169	1.000
285	GOBP_CATION_TRANSPORT	GO:0006812	25	0.182629	0.652561	0.925573	0.939061	1.000
286	GOBP_POSITIVE_REGULATION_OF_CATION_TRANSMEMBRA...	GO:1904064	5	0.288685	0.633238	0.895050	0.954174	1.000
287	GOBP_REGULATION_OF_NEURON_DIFFERENTIATION	GO:0045664	5	0.282744	0.632942	0.906191	0.951125	1.000
288	GOBP_POSITIVE_REGULATION_OF_ANION_TRANSPORT	GO:1903793	9	0.229130	0.630228	0.916201	0.950156	1.000
289	GOBP_EPITHELIAL_CELL_PROLIFERATION	GO:0050673	12	0.204060	0.616150	0.933594	0.959030	1.000
290	GOBP_ADENYLATE_CYCLASE_INHIBITING_G_PROTEIN_CO...	GO:0007196	9	0.227848	0.608279	0.916058	0.961906	1.000
291	GOBP_REGULATION_OF_POSTSYNAPSE_ORGANIZATION	GO:0099175	6	0.245794	0.595094	0.940952	0.968330	1.000
292	GOBP_CELL_ACTIVATION	GO:0001775	22	0.167833	0.593614	0.937153	0.966003	1.000
293	GOBP_ERK1_AND_ERK2_CASCADE	GO:0070371	5	0.256109	0.569189	0.962887	0.978126	1.000
294	GOBP_VESICLE_MEDIATED_TRANSPORT_IN_SYNAPSE	GO:0099003	7	0.221735	0.562607	0.960452	0.978633	1.000
295	GOBP_REGULATION_OF_PROTEIN_LOCALIZATION	GO:0032880	18	0.165719	0.540815	0.973635	0.985748	1.000
296	GOBP_ORGANIC_HYDROXY_COMPOUND_METABOLIC_PROCESS	GO:1901615	8	0.196391	0.520707	0.976699	0.990253	1.000
297	REACTOME_G_ALPHA_Q_SIGNALLING_EVENTS	R-RNO-416476	6	0.221354	0.516616	0.986166	0.988301	1.000
298	GOBP_NEUROINFLAMMATORY_RESPONSE	GO:0150076	5	0.228916	0.511031	0.994434	0.986752	1.000

Continuation of Table **S7**

	NAME	ID	SIZE	ES	NES	NOM.p.val	FDR.q.val	FWER.p.val
299	GOBP_TEMPERATURE_HOMEOSTASIS	GO:0001659	5	0.228916	0.505619	0.980469	0.984958	1.000
300	REACTOME_ANTI_INFLAMMATORY_RESPONSE_FAVOURING_...	R-HSA-9662851	5	-0.228916	-0.507198	0.976331	0.984880	1.000
301	GOBP_CELL_CELL_SIGNALING_BY_WNT	GO:0198738	7	-0.197779	-0.510482	0.978947	0.986170	1.000
302	GOBP_CYTOKINE_PRODUCTION	GO:0001816	10	-0.179615	-0.527599	0.967532	0.982844	1.000
303	GOBP_REGULATION_OF_OSTEOCLAST_DIFFERENTIATION	GO:0045670	5	-0.246852	-0.543534	0.978088	0.978347	1.000
304	GOBP_SENSORY_PERCEPTION_OF_PAIN	GO:0019233	5	-0.240964	-0.546937	0.970833	0.979200	1.000
305	GOBP_EPHRIN_RECEPTOR_SIGNALING_PATHWAY	GO:0048013	5	-0.253012	-0.568383	0.954918	0.970312	1.000
306	GOBP_REGULATION_OF_NERVOUS_SYSTEM_PROCESS	GO:0031644	8	-0.222327	-0.607216	0.933619	0.946897	1.000
307	REACTOME_EPH_EPHRIN_SIGNALING	R-RNO-2682334	6	-0.256098	-0.620456	0.915289	0.939045	1.000
308	GOBP_EMBRYONIC_MORPHOGENESIS	GO:0048598	6	-0.258797	-0.626565	0.922764	0.936186	1.000
309	REACTOME_TRANSCRIPTIONAL_REGULATION_OF_WHITE_A...	R-RNO-381340	6	-0.261621	-0.627219	0.927835	0.937960	1.000
310	GOBP_RESPONSE_TO_MONOSACCHARIDE	GO:0034284	5	-0.278706	-0.631007	0.898925	0.936994	1.000
311	GOBP_RESPONSE_TO_CARBOHYDRATE	GO:0009743	5	-0.278706	-0.631744	0.906375	0.938600	1.000
312	GOBP_CELLULAR_MACROMOLECULE_LOCALIZATION	GO:0070727	20	-0.175390	-0.633743	0.937107	0.938994	1.000
313	GOBP_LONG_TERM_MEMORY	GO:0007616	6	-0.271592	-0.638189	0.904110	0.937157	1.000
314	GOBP_NEGATIVE_REGULATION_OF_CELL_POPULATION_PR...	GO:0008285	9	-0.232440	-0.638640	0.902490	0.939017	1.000
315	GOBP_POSITIVE_REGULATION_OF_APOPTOTIC_SIGNALIN...	GO:2001235	5	-0.285435	-0.638714	0.906582	0.941257	1.000
316	GOBP_REPRODUCTIVE_SYSTEM_DEVELOPMENT	GO:0061458	6	-0.271035	-0.641341	0.886905	0.941168	1.000
317	GOBP_HOMEOSTATIC_PROCESS	GO:0042592	31	-0.160737	-0.642270	0.918793	0.942579	1.000
318	GOBP_NEGATIVE_REGULATION_OF_TRANSPORT	GO:0051051	13	-0.201043	-0.645477	0.899782	0.941895	1.000
319	GOBP_REGULATION_OF_PEPTIDASE_ACTIVITY	GO:0052547	10	-0.228685	-0.653405	0.888438	0.936271	1.000
320	GOBP_REGULATION_OF_METAL_ION_TRANSPORT	GO:0010959	6	-0.277772	-0.653736	0.889130	0.938226	1.000
321	GOBP_INNATE_IMMUNE_RESPONSE	GO:0045087	7	-0.260760	-0.654917	0.866944	0.939337	1.000
322	GOBP_REGULATION_OF_MAP_KINASE_ACTIVITY	GO:0043405	9	-0.240464	-0.670469	0.837553	0.924839	1.000
323	GOBP_RESPONSE_TO_INTERLEUKIN_1	GO:0070555	5	-0.302904	-0.672631	0.832985	0.924760	1.000
324	GOBP_MULTICELLULAR_ORGANISMAL_HOMEOSTASIS	GO:0048871	12	-0.219280	-0.672693	0.870021	0.927020	1.000
325	GOBP_CELLULAR_RESPONSE_TO_DNA_DAMAGE_STIMULUS	GO:0006974	5	-0.305020	-0.676017	0.879142	0.925670	1.000
326	GOBP_ACTIVATION_OF_PROTEIN_KINASE_ACTIVITY	GO:0032147	10	-0.234035	-0.676804	0.865732	0.927011	1.000
327	REACTOME_NGF_STIMULATED_TRANSCRIPTION	R-RNO-9031628	9	-0.246881	-0.679442	0.866242	0.926420	1.000
328	REACTOME_NEGATIVE_REGULATION_OF_NMDA_RECEPTOR_...	R-HSA-961732	8	-0.264214	-0.684021	0.875510	0.923613	1.000
329	GOBP_RESPONSE_TO_WOUNDING	GO:0009611	15	-0.216045	-0.688021	0.871681	0.921289	1.000
330	GOBP_CELLULAR_RESPONSE_TO_KETONE	GO:1901655	5	-0.312339	-0.688531	0.859155	0.923084	1.000
331	GOBP_REGULATION_OF_HEMOPOIESIS	GO:1903706	10	-0.241346	-0.690281	0.865385	0.923202	1.000
332	GOBP_INTRINSIC_APOPTOTIC_SIGNALING_PATHWAY	GO:0097193	5	-0.319499	-0.698322	0.861635	0.915427	1.000
333	GOBP_REGULATION_OF_GLIOGENESIS	GO:0014013	5	-0.316372	-0.705483	0.834016	0.908766	1.000
334	GOBP_CATION_TRANSMEMBRANE_TRANSPORT	GO:0098655	18	-0.204204	-0.706553	0.870968	0.909637	1.000
335	GOBP_CELL_JUNCTION_ASSEMBLY	GO:0034329	8	-0.261227	-0.708840	0.835443	0.909183	1.000
336	GOBP_NEGATIVE_REGULATION_OF_GENE_EXPRESSION	GO:0010629	11	-0.239767	-0.709817	0.824242	0.910340	1.000
337	GOBP_POSITIVE_REGULATION_OF_PROTEOLYSIS	GO:0045862	8	-0.261047	-0.711468	0.842767	0.910585	1.000
338	REACTOME_ASSEMBLY_AND_CELL_SURFACE_PRESENTATIO...	R-RNO-9609736	8	-0.264214	-0.712507	0.820988	0.911463	1.000
339	REACTOME_CELL_CYCLE_MITOTIC	R-RNO-69278	5	-0.322857	-0.712548	0.842324	0.913771	1.000
340	GOBP_POSITIVE_REGULATION_OF_GLIOGENESIS	GO:0014015	5	-0.316372	-0.716416	0.813008	0.910955	1.000
341	GOBP_POSITIVE_REGULATION_OF_EPITHELIAL_CELL_MI...	GO:0010634	5	-0.323887	-0.719243	0.805118	0.909375	1.000

Continuation of Table **S7**

	NAME	ID	SIZE	ES	NES	NOM.p.val	FDR.q.val	FWER.p.val
342	REACTOME_CELL_CYCLE	R-RNO-1640170	6	-0.292499	-0.719399	0.840551	0.911584	1.000
343	GOBP_REGULATION_OF_MYELOID_CELL_DIFFERENTIATION	GO:0045637	9	-0.257888	-0.719618	0.819328	0.913613	1.000
344	GOBP_RESPONSE_TO_ALCOHOL	GO:0097305	11	-0.246753	-0.720492	0.814815	0.914861	1.000
345	GOBP_CELLULAR_RESPONSE_TO_ORGANIC_CYCLIC_COMPOUND	GO:0071407	13	-0.229440	-0.721552	0.833333	0.915816	1.000
346	GOBP_POSITIVE_REGULATION_OF_CYTOKINE_PRODUCTION	GO:0001819	7	-0.278564	-0.722817	0.789264	0.916528	1.000
347	GOBP_REGULATION_OF_MITOTIC_CELL_CYCLE	GO:0007346	5	-0.322142	-0.724359	0.802419	0.916838	1.000
348	REACTOME_PLATELET_ACTIVATION_SIGNALING_AND_AGG...	R-HSA-76002	7	-0.283210	-0.724800	0.798000	0.918615	1.000
349	GOBP_REGULATION_OF_MUSCLE_CELL_DIFFERENTIATION	GO:0051147	6	-0.303809	-0.725187	0.812775	0.920491	1.000
350	GOBP_REGULATION_OF_PROTEIN_MODIFICATION_PROCESS	GO:0031399	28	-0.192510	-0.733001	0.838636	0.912060	1.000
351	REACTOME_NEUREXINS_AND_NEUROLIGINS	R-RNO-6794361	9	-0.261142	-0.733115	0.809524	0.914344	1.000
352	GOBP_POSITIVE_REGULATION_OF_SIGNALING	GO:0023056	34	-0.182556	-0.735179	0.833333	0.913763	1.000
353	GOBP_LOCOMOTORY_BEHAVIOR	GO:0007626	8	-0.275000	-0.736751	0.804527	0.914065	1.000
354	GOBP_ENDOTHELIAL_CELL_PROLIFERATION	GO:0001935	8	-0.279074	-0.739076	0.795031	0.913033	1.000
355	GOBP_RESPONSE_TO_ALKALOID	GO:0043279	5	-0.337798	-0.740503	0.779070	0.913475	1.000
356	GOBP_TRANSMEMBRANE_RECEPTOR_PROTEIN_SERINE_THR...	GO:0007178	6	-0.312285	-0.741046	0.763043	0.915138	1.000
357	GOBP_LIPID_BIOSYNTHETIC_PROCESS	GO:0008610	6	-0.312603	-0.742144	0.774757	0.915978	1.000
358	GOBP_REGULATION_OF_LIPID_BIOSYNTHETIC_PROCESS	GO:0046890	6	-0.312603	-0.745215	0.803063	0.914160	1.000
359	GOBP_REGULATION_OF_PROTEOLYSIS	GO:0030162	12	-0.241193	-0.745667	0.783370	0.916085	1.000
360	GOBP_REGULATION_OF_CYSTEINE_TYPE_ENDOPEPTIDASE...	GO:2000116	9	-0.269447	-0.747341	0.773019	0.916188	1.000
361	GOBP_DEFENSE_RESPONSE	GO:0006952	19	-0.213259	-0.751748	0.808190	0.912229	1.000
362	GOBP_NEGATIVE_REGULATION_OF_ION_TRANSPORT	GO:0043271	11	-0.246828	-0.752545	0.775000	0.913558	1.000
363	GOBP_MULTI_ORGANISM_PROCESS	GO:0051704	16	-0.228735	-0.754064	0.776573	0.913702	1.000
364	GOBP_CARBOHYDRATE_DERIVATIVE_METABOLIC_PROCESS	GO:1901135	8	-0.279235	-0.754475	0.764831	0.915685	1.000
365	REACTOME_MAPK_FAMILY_SIGNALING_CASCADES	R-RNO-5683057	12	-0.253182	-0.756055	0.787629	0.915858	1.000
366	GOBP_FAT_CELL_DIFFERENTIATION	GO:0045444	9	-0.272323	-0.761995	0.761616	0.909097	1.000
367	GOBP_POSITIVE_REGULATION_OF_ENDOTHELIAL_CELL_P...	GO:0001938	6	-0.322667	-0.763973	0.761506	0.908547	1.000
368	GOBP_LEUKOCYTE_MEDIATED_IMMUNITY	GO:0002443	8	-0.287500	-0.764228	0.751073	0.910698	1.000
369	GOBP_REGULATION_OF_LIPID_METABOLIC_PROCESS	GO:0019216	7	-0.300315	-0.765164	0.754678	0.911893	1.000
370	GOBP_REGULATION_OF_FAT_CELL_DIFFERENTIATION	GO:0045598	6	-0.315394	-0.766871	0.755061	0.911597	1.000
371	GOBP_METAL_ION_TRANSPORT	GO:0030001	11	-0.262125	-0.768832	0.742204	0.910973	1.000
372	REACTOME_NUCLEAR_EVENTS_KINASE_AND_TRANSCRIPTI...	R-RNO-198725	11	-0.259394	-0.774536	0.731006	0.904595	1.000
373	GOBP_RESPONSE_TO_TEMPERATURE_STIMULUS	GO:0009266	9	-0.279007	-0.774571	0.721519	0.907112	1.000
374	GOBP_APOPTOTIC_PROCESS	GO:0006915	35	-0.192045	-0.781123	0.815618	0.898614	1.000
375	GOBP_REGULATION_OF_LEUKOCYTE_DIFFERENTIATION	GO:1902105	9	-0.284453	-0.781447	0.723493	0.900689	1.000
376	GOBP_POSITIVE_REGULATION_OF_EPITHELIAL_CELL_PR...	GO:0050679	7	-0.304020	-0.783213	0.740365	0.900515	1.000
377	GOBP_CALCIUM_ION_TRANSPORT	GO:0006816	11	-0.262125	-0.783848	0.701826	0.901949	1.000
378	GOBP_POSITIVE_REGULATION_OF_GENE_EXPRESSION	GO:0010628	16	-0.235521	-0.783966	0.744240	0.904309	1.000
379	REACTOME_HEMOSTASIS	R-RNO-109582	14	-0.250798	-0.791148	0.755940	0.894731	1.000
380	GOBP_REGULATION_OF_POSTSYNAPTIC_MEMBRANE_POTEN...	GO:0060078	14	-0.242890	-0.793276	0.722105	0.893882	1.000
381	GOBP_REGULATION_OF_NEURON_PROJECTION_DEVELOPMENT	GO:0010975	16	-0.235544	-0.793309	0.725055	0.896437	1.000
382	REACTOME_CIRCADIAN_CLOCK	R-HSA-400253	5	-0.354545	-0.795131	0.737705	0.895913	1.000
383	GOBP_ESTABLISHMENT_OF_PROTEIN_LOCALIZATION_TO_...	GO:0072594	5	-0.361555	-0.802688	0.706935	0.885710	1.000
384	GOBP_RESPONSE_TO_PEPTIDE	GO:1901652	18	-0.232673	-0.805931	0.737166	0.882776	1.000

Continuation of Table **S7**

	NAME	ID	SIZE	ES	NES	NOM.p.val	FDR.q.val	FWER.p.val
385	REACTOME_PROTEIN_PROTEIN_INTERACTIONS_AT_SYNAPSES	R-RNO-6794362	12	-0.263158	-0.806973	0.714575	0.883612	1.000
386	GOBP_NEGATIVE_REGULATION_OF_LIPID_METABOLIC_PR...	GO:0045833	5	-0.359331	-0.809689	0.705882	0.881469	1.000
387	GOBP_NEGATIVE_REGULATION_OF_ANION_TRANSPORT	GO:1903792	8	-0.302382	-0.810514	0.686235	0.882675	1.000
388	GOBP_REGULATION_OF_MYELOID_LEUKOCYTE_DIFFERENT...	GO:0002761	8	-0.302711	-0.813524	0.678350	0.879499	1.000
389	GOBP_REGULATION_OF_SYSTEM_PROCESS	GO:0044057	18	-0.238700	-0.813681	0.736739	0.881858	1.000
390	GOBP_POSITIVE_REGULATION_OF_DEFENSE_RESPONSE	GO:0031349	5	-0.358253	-0.814454	0.679513	0.883166	1.000
391	GOBP_PROTEIN_LOCALIZATION_TO_ORGANELLE	GO:0033365	8	-0.300993	-0.814635	0.704225	0.885508	1.000
392	GOBP_MUSCLE_TISSUE_DEVELOPMENT	GO:0060537	11	-0.281116	-0.818114	0.696842	0.882119	1.000
393	GOBP_RESPONSE_TO_OXYGEN_CONTAINING_COMPOUND	GO:1901700	39	-0.199484	-0.821008	0.736501	0.879639	1.000
394	GOBP_CELLULAR_RESPONSE_TO_ALCOHOL	GO:0097306	5	-0.361833	-0.822973	0.701903	0.878610	1.000
395	REACTOME_NEUTROPHIL_DEGRANULATION	R-RNO-6798695	6	-0.341463	-0.823651	0.660338	0.880154	1.000
396	GOBP_NEGATIVE_REGULATION_OF_MULTICELLULAR_ORGA...	GO:0051241	15	-0.250690	-0.824490	0.668860	0.881454	1.000
397	GOBP_REGULATION_OF_CELL_PROJECTION_ORGANIZATION	GO:0031344	20	-0.235060	-0.825317	0.692140	0.882725	1.000
398	GOBP_MYELOID_LEUKOCYTE_MEDIATED_IMMUNITY	GO:0002444	6	-0.341463	-0.827919	0.659919	0.880997	1.000
399	GOBP_DIVALENT_INORGANIC_CATION_HOMEOSTASIS	GO:0072507	13	-0.269552	-0.834821	0.662500	0.870895	1.000
400	GOBP_CELL_ACTIVATION_INVOLVED_IN_IMMUNE_RESPONSE	GO:0002263	6	-0.341463	-0.835395	0.655031	0.872581	1.000
401	GOBP_REGULATION_OF_CELL_DEATH	GO:0010941	34	-0.208777	-0.836920	0.731405	0.872578	1.000
402	GOBP_ENDOMEMBRANE_SYSTEM_ORGANIZATION	GO:0010256	6	-0.345614	-0.846190	0.639066	0.858012	1.000
403	GOBP_REGULATION_OF_CELLULAR_LOCALIZATION	GO:0060341	17	-0.245999	-0.848673	0.656613	0.855842	1.000
404	GOBP_REGULATION_OF_ENDOTHELIAL_CELL_MIGRATION	GO:0010594	5	-0.377361	-0.848914	0.652928	0.858065	1.000
405	GOBP_REGULATION_OF_CELLULAR_CATABOLIC_PROCESS	GO:0031329	10	-0.292225	-0.852262	0.642553	0.854766	1.000
406	GOBP_REGULATION_OF_CYTOSOLIC_CALCIUM_ION_CONCE...	GO:0051480	13	-0.269552	-0.855593	0.641791	0.851355	1.000
407	GOBP_SECOND_MESSENGER_MEDIATED_SIGNALING	GO:0019932	16	-0.259088	-0.858059	0.638393	0.849343	1.000
408	GOBP_REGULATION_OF_CELL_DIFFERENTIATION	GO:0045595	36	-0.212871	-0.858170	0.664269	0.851756	1.000
409	GOBP_CELLULAR_RESPONSE_TO_ABIOTIC_STIMULUS	GO:0071214	7	-0.341724	-0.859745	0.616601	0.851612	1.000
410	GOBP_POSITIVE_REGULATION_OF_CELL_PROJECTION_OR...	GO:0031346	10	-0.294934	-0.860325	0.639175	0.853199	1.000
411	GOBP_POSITIVE_REGULATION_OF_HYDROLASE_ACTIVITY	GO:0051345	16	-0.254525	-0.863409	0.640333	0.850267	1.000
412	GOBP_CALCIUM_MEDIATED_SIGNALING	GO:0019722	10	-0.301784	-0.867092	0.652977	0.845853	1.000
413	GOBP_MUSCLE_STRUCTURE_DEVELOPMENT	GO:0061061	16	-0.263450	-0.867893	0.633406	0.847142	1.000
414	GOBP_CIRCULATORY_SYSTEM_PROCESS	GO:0003013	5	-0.400419	-0.869143	0.613821	0.847502	1.000
415	GOBP_METAL_ION_HOMEOSTASIS	GO:0055065	14	-0.270270	-0.870285	0.610309	0.848077	1.000
416	GOBP_RESPONSE_TO_PEPTIDE_HORMONE	GO:0043434	13	-0.274055	-0.870441	0.637097	0.850428	1.000
417	GOBP_CELLULAR_ION_HOMEOSTASIS	GO:0006873	14	-0.270270	-0.871187	0.627409	0.851754	1.000
418	GOBP_MYELOID_LEUKOCYTE_DIFFERENTIATION	GO:0002573	11	-0.292851	-0.875138	0.595618	0.846775	1.000
419	GOBP_POSITIVE_REGULATION_OF_MOLECULAR_FUNCTION	GO:0044093	37	-0.214430	-0.876418	0.651376	0.847137	1.000
420	GOBP_POSITIVE_REGULATION_OF_AXONOGENESIS	GO:0050772	5	-0.388118	-0.877223	0.594080	0.848456	1.000
421	REACTOME_ADAPTIVE_IMMUNE_SYSTEM	R-RNO-1280218	9	-0.317988	-0.878575	0.596194	0.848596	1.000
422	GOBP_REGULATION_OF_AXONOGENESIS	GO:0050770	7	-0.342160	-0.883526	0.603696	0.842097	1.000
423	GOBP_OSTEOCLAST_DIFFERENTIATION	GO:0030316	6	-0.367724	-0.883716	0.575630	0.844514	1.000
424	GOBP_RESPONSE_TO_NICOTINE	GO:0035094	7	-0.345679	-0.885157	0.580499	0.844347	1.000
425	GOBP_REGULATION_OF_INFLAMMATORY_RESPONSE	GO:0050727	7	-0.353646	-0.887432	0.578000	0.842869	1.000
426	GOBP_RESPONSE_TO_HEAT	GO:0009408	7	-0.347222	-0.892237	0.580392	0.836169	1.000
427	GOBP_ANIMAL_ORGAN_MORPHOGENESIS	GO:0009887	12	-0.293925	-0.896242	0.588727	0.831408	1.000

Continuation of Table **S7**

	NAME	ID	SIZE	ES	NES	NOM.p.val	FDR.q.val	FWER.p.val
428	GOBP_EXCITATORY_CHEMICAL_SYNAPTIC_TRANSMISSION	GO:0098976	5	-0.398286	-0.896846	0.565591	0.832856	1.000
429	GOBP_POSITIVE_REGULATION_OF_SMALL_MOLECULE_MET...	GO:0062013	5	-0.403905	-0.900251	0.579710	0.829196	1.000
430	GOBP_REGULATION_OF_TRANSMEMBRANE_TRANSPORT	GO:0034762	23	-0.243276	-0.900782	0.597458	0.830935	1.000
431	GOBP_REGULATION_OF_CELL_CYCLE_PROCESS	GO:0010564	5	-0.404383	-0.904073	0.556263	0.827612	1.000
432	GOBP_REGULATION_OF_DEFENSE_RESPONSE	GO:0031347	7	-0.353646	-0.907198	0.565306	0.824377	1.000
433	GOBP_CHEMICAL_SYNAPTIC_TRANSMISSION_POSTSYNAPTIC	GO:0099565	9	-0.325642	-0.907492	0.576344	0.826677	1.000
434	GOBP_NEGATIVE_REGULATION_OF_IMMUNE_SYSTEM_PROCESS	GO:0002683	5	-0.405951	-0.910443	0.567901	0.823638	1.000
435	GOBP_LEUKOCYTE_DIFFERENTIATION	GO:0002521	15	-0.281485	-0.911164	0.553061	0.825066	1.000
436	GOBP_REGULATION_OF_VASCULAR_ASSOCIATED_SMOOTH_...	GO:1904705	5	-0.404269	-0.911264	0.557203	0.827691	1.000
437	GOBP_CHEMICAL_HOMEOSTASIS	GO:0048878	23	-0.240829	-0.913117	0.557447	0.826857	1.000
438	GOBP_CELL_POPULATION_PROLIFERATION	GO:0008283	32	-0.230967	-0.917377	0.581292	0.821145	1.000
439	GOBP_PROTEOLYSIS	GO:0006508	17	-0.273142	-0.917844	0.556769	0.823001	1.000
440	GOBP_ORGANONITROGEN_COMPOUND_BIOSYNTHETIC_PROCESS	GO:1901566	13	-0.293081	-0.919931	0.567329	0.821940	1.000
441	GOBP_EMBRYO_DEVELOPMENT	GO:0009790	14	-0.287247	-0.920467	0.540773	0.823744	1.000
442	GOBP_INTRACELLULAR_RECEPTOR_SIGNALING_PATHWAY	GO:0030522	5	-0.412425	-0.922779	0.547619	0.821917	1.000
443	HALLMARK_KRAS_SIGNALING_UP	M5953	6	-0.384706	-0.924107	0.543611	0.822284	1.000
444	GOBP_REPRODUCTION	GO:0000003	18	-0.265472	-0.925872	0.556745	0.821610	1.000
445	GOBP_ANATOMICAL_STRUCTURE_HOMEOSTASIS	GO:0060249	7	-0.366772	-0.926661	0.548035	0.823007	1.000
446	GOBP_SMALL_GTPASE_MEDIATED_SIGNAL_TRANSDUCTION	GO:0007264	8	-0.350114	-0.928278	0.520085	0.822898	1.000
447	REACTOME_INTRACELLULAR_SIGNALING_BY_SECOND_MES...	R-RNO-9006925	15	-0.277756	-0.929408	0.532225	0.823669	1.000
448	GOBP_CELL_SURFACE_RECEPTOR_SIGNALING_PATHWAY_I...	GO:1905114	15	-0.282610	-0.930717	0.548729	0.824308	1.000
449	GOBP_ION_HOMEOSTASIS	GO:0050801	16	-0.277778	-0.931811	0.528926	0.825120	1.000
450	GOBP_REGULATION_OF_EPITHELIAL_CELL_MIGRATION	GO:0010632	6	-0.385622	-0.932442	0.548035	0.826958	1.000
451	GOBP_MYELOID_LEUKOCYTE_ACTIVATION	GO:0002274	8	-0.350000	-0.932564	0.531697	0.829731	1.000
452	GOBP_REGULATION_OF_MEMBRANE_POTENTIAL	GO:0042391	17	-0.276998	-0.933258	0.560000	0.831254	1.000
453	GOBP_DEVELOPMENTAL_PROCESS_INVOLVED_IN_REPRODU...	GO:0003006	11	-0.322967	-0.935609	0.553377	0.829532	1.000
454	GOBP_CELLULAR_RESPONSE_TO_MOLECULE_OF_BACTERIA...	GO:0071219	8	-0.359270	-0.943030	0.528184	0.817404	1.000
455	GOBP_CELLULAR_RESPONSE_TO_EXTRACELLULAR_STIMULUS	GO:0031668	8	-0.349032	-0.945290	0.496774	0.815733	1.000
456	GOBP_RESPONSE_TO_ENDOGENOUS_STIMULUS	GO:0009719	34	-0.233717	-0.945859	0.517986	0.817606	1.000
457	GOBP_CELLULAR_RESPONSE_TO_HORMONE_STIMULUS	GO:0032870	11	-0.318423	-0.948209	0.520518	0.815549	1.000
458	GOBP_TRANSMEMBRANE_RECEPTOR_PROTEIN_TYROSINE_K...	GO:0007169	19	-0.270459	-0.948857	0.514768	0.817212	1.000
459	GOBP_PHAGOCYTOSIS	GO:0006909	5	-0.424990	-0.954268	0.495069	0.809423	1.000
460	REACTOME_DEVELOPMENTAL_BIOLOGY	R-RNO-1266738	22	-0.268255	-0.954517	0.517316	0.811991	1.000
461	GOBP_ERBB_SIGNALING_PATHWAY	GO:0038127	5	-0.428193	-0.958321	0.515924	0.807107	1.000
462	GOBP_CELLULAR_RESPONSE_TO_BIOTIC_STIMULUS	GO:0071216	8	-0.359270	-0.959955	0.508097	0.806921	1.000
463	GOBP_POSITIVE_REGULATION_OF_MULTICELLULAR_ORGA...	GO:0051240	32	-0.242568	-0.963246	0.516704	0.803043	1.000
464	GOBP_POSITIVE_REGULATION_OF_CELLULAR_PROTEIN_L...	GO:1903829	7	-0.370922	-0.965362	0.477551	0.802094	1.000
465	GOBP_POSITIVE_REGULATION_OF_TRANSMEMBRANE_TRAN...	GO:0034764	7	-0.372886	-0.967529	0.459459	0.800604	1.000
466	GOBP_CELLULAR_RESPONSE_TO_OXYGEN_CONTAINING_CO...	GO:1901701	29	-0.247816	-0.969591	0.507463	0.799452	1.000
467	GOBP_PERIPHERAL_NERVOUS_SYSTEM_DEVELOPMENT	GO:0007422	9	-0.346844	-0.969694	0.486081	0.802346	1.000
468	GOBP_MONOCARBOXYLIC_ACID_METABOLIC_PROCESS	GO:0032787	6	-0.410127	-0.970162	0.467836	0.804532	1.000
469	GOBP_PROTEIN_MODIFICATION_BY_SMALL_PROTEIN_CON...	GO:0070647	7	-0.385791	-0.978392	0.491453	0.790244	1.000
470	GOBP_RESPONSE_TO_OXYGEN_LEVELS	GO:0070482	8	-0.371359	-0.980746	0.481153	0.788432	1.000

Continuation of Table **S7**

	NAME	ID	SIZE	ES	NES	NOM.p.val	FDR.q.val	FWER.p.val
471	GOBP_POSITIVE_REGULATION_OF_HEMOPOIESIS	GO:1903708	7	-0.390164	-0.982112	0.453280	0.788581	1.000
472	GOBP_INFLAMMATORY_RESPONSE	GO:0006954	12	-0.321055	-0.983722	0.447257	0.788435	1.000
473	GOBP_REGULATION_OF_MULTICELLULAR_ORGANISMAL_DE...	GO:2000026	31	-0.247448	-0.986242	0.494279	0.786297	1.000
474	GOBP_OSSIFICATION	GO:0001503	9	-0.355999	-0.987187	0.463938	0.787511	1.000
475	GOBP_REGULATION_OF_LYMPHOCYTE_ACTIVATION	GO:0051249	5	-0.447016	-0.994596	0.460581	0.774988	1.000
476	GOBP_CELLULAR_COMPONENT_DISASSEMBLY	GO:0022411	6	-0.412176	-0.997021	0.450397	0.773021	1.000
477	GOBP_POSITIVE_REGULATION_OF_MYELOID_LEUKOCYTE_...	GO:0002763	6	-0.415664	-0.997227	0.470833	0.775777	1.000
478	GOBP_PROTEIN_LOCALIZATION_TO_POSTSYNAPSE	GO:0062237	5	-0.449699	-0.998643	0.443320	0.776118	1.000
479	GOBP_SMALL_MOLECULE_METABOLIC_PROCESS	GO:0044281	16	-0.299032	-1.001057	0.433333	0.774084	1.000
480	GOBP_RESPONSE_TO_LIPID	GO:0033993	19	-0.289528	-1.001403	0.464368	0.776527	1.000
481	REACTOME_CELLULAR_RESPONSES_TO_EXTERNAL_STIMULI	R-RNO-8953897	11	-0.344597	-1.001478	0.435146	0.779584	1.000
482	GOBP_IMMUNE_SYSTEM_DEVELOPMENT	GO:0002520	17	-0.289842	-1.001718	0.474946	0.782287	1.000
483	GOBP_ASSOCIATIVE_LEARNING	GO:0008306	7	-0.396731	-1.003805	0.434426	0.780959	1.000
484	REACTOME_POST_TRANSLATIONAL_PROTEIN_MODIFICATION	R-RNO-597592	7	-0.392535	-1.004320	0.443750	0.783140	1.000
485	GOBP_POSITIVE_REGULATION_OF_DEVELOPMENTAL_PROCESS	GO:0051094	32	-0.247034	-1.005577	0.435268	0.783442	1.000
486	GOBP_LEARNING	GO:0007612	13	-0.332276	-1.013174	0.427293	0.770430	1.000
487	GOBP_REGULATION_OF_NERVOUS_SYSTEM_DEVELOPMENT	GO:0051960	15	-0.312689	-1.014100	0.420719	0.771742	1.000
488	GOBP_NEGATIVE_REGULATION_OF_SIGNALING	GO:0023057	21	-0.283387	-1.016667	0.422566	0.768912	1.000
489	GOBP_PROTEIN_LOCALIZATION_TO_SYNAPSE	GO:0035418	5	-0.449699	-1.018342	0.446721	0.768462	1.000
490	REACTOME_VESICLE_MEDIATED_TRANSPORT	R-RNO-5653656	5	-0.454482	-1.022191	0.430584	0.763599	1.000
491	GOBP_MYELOID_CELL_DIFFERENTIATION	GO:0030099	13	-0.328562	-1.023580	0.396146	0.763811	1.000
492	REACTOME_MEMBRANE_TRAFFICKING	R-RNO-199991	5	-0.454482	-1.024754	0.418919	0.764517	1.000
493	GOBP_EPITHELIAL_CELL_DIFFERENTIATION	GO:0030855	7	-0.396753	-1.026972	0.422833	0.762951	1.000
494	GOBP_REGULATION_OF_PROTEIN_SERINE_THREONINE_KI...	GO:0071900	12	-0.338359	-1.028841	0.401806	0.762259	1.000
495	GOBP_NEGATIVE_REGULATION_OF_CELL_CYCLE	GO:0045786	5	-0.464469	-1.032478	0.380435	0.757881	1.000
496	GOBP_AMEBOIDAL_TYPE_CELL_MIGRATION	GO:0001667	11	-0.345952	-1.032926	0.417323	0.760135	1.000
497	GOBP_RESPONSE_TO_KETONE	GO:1901654	7	-0.410843	-1.035647	0.393075	0.757547	1.000
498	GOBP_MUSCLE_CELL_DIFFERENTIATION	GO:0042692	10	-0.366490	-1.036750	0.390351	0.758434	1.000
499	GOBP_POSITIVE_REGULATION_OF_IMMUNE_RESPONSE	GO:0050778	9	-0.373772	-1.037740	0.410148	0.759642	1.000
500	GOBP_MUSCLE_CONTRACTION	GO:0006936	5	-0.465952	-1.037835	0.406639	0.762860	1.000
501	GOBP_ORGANIC_ACID_METABOLIC_PROCESS	GO:0006082	7	-0.408090	-1.038076	0.387295	0.765730	1.000
502	GOBP_TISSUE_HOMEOSTASIS	GO:0001894	5	-0.463968	-1.038352	0.413793	0.768513	1.000
503	GOBP_REGULATION_OF_IMMUNE_RESPONSE	GO:0050776	12	-0.340732	-1.039730	0.415556	0.768865	1.000
504	GOBP_CELLULAR_RESPONSE_TO_PEPTIDE	GO:1901653	13	-0.329145	-1.040098	0.378788	0.771418	1.000
505	GOBP_POSTTRANSCRIPTIONAL_REGULATION_OF_GENE_EX...	GO:0010608	8	-0.392071	-1.040296	0.377119	0.774486	1.000
506	GOBP_POSITIVE_REGULATION_OF_NUCLEOBASE_CONTAIN...	GO:0045935	28	-0.270192	-1.046896	0.395745	0.763597	1.000
507	GOBP_ENZYME_LINKED_RECEPTOR_PROTEIN_SIGNALING_...	GO:0007167	24	-0.273872	-1.047268	0.381395	0.766180	1.000
508	GOBP_RESPONSE_TO_HORMONE	GO:0009725	20	-0.291206	-1.047363	0.387029	0.769450	1.000
509	GOBP_CELLULAR_RESPONSE_TO_PEPTIDE_HORMONE_STIM...	GO:0071375	9	-0.374362	-1.047844	0.392070	0.771915	1.000
510	GOBP_NEGATIVE_REGULATION_OF_PROTEIN_CATABOLIC_...	GO:0042177	5	-0.471549	-1.051579	0.385396	0.766584	1.000
511	GOBP_PROTEIN_MODIFICATION_BY_SMALL_PROTEIN_CON...	GO:0032446	6	-0.435950	-1.053848	0.398374	0.765293	1.000
512	GOBP_LYMPHOCYTE_ACTIVATION	GO:0046649	10	-0.363952	-1.055417	0.363636	0.765435	1.000
513	REACTOME_SIGNALING_BY_RHO_GTPASES_MIRO_GTPASES...	R-RNO-9716542	9	-0.382580	-1.056650	0.366667	0.766464	1.000

Continuation of Table **S7**

	NAME	ID	SIZE	ES	NES	NOM.p.val	FDR.q.val	FWER.p.val
514	GOBP_NEGATIVE_REGULATION_OF_KINASE_ACTIVITY	GO:0033673	5	-0.469344	-1.057049	0.388889	0.769239	1.000
515	REACTOME_TOLL_LIKE_RECEPTOR_TLR1_TLR2_CASCADE	R-RNO-168179	8	-0.404275	-1.057248	0.374494	0.772374	1.000
516	GOBP_INORGANIC_ION_TRANSMEMBRANE_TRANSPORT	GO:0098660	9	-0.392302	-1.058399	0.379913	0.773633	1.000
517	GOBP_CARBOHYDRATE_HOMEOSTASIS	GO:0033500	7	-0.420198	-1.059449	0.382845	0.774782	1.000
518	HALLMARK_ALLOGRAFT_REJECTION	M5950	7	-0.419098	-1.059857	0.398095	0.777519	1.000
519	REACTOME_TOLL_LIKE_RECEPTOR_CASCADES	R-RNO-168898	8	-0.404275	-1.061902	0.397119	0.776698	1.000
520	GOBP_NEGATIVE_REGULATION_OF_TRANSFERASE_ACTIVITY	GO:0051348	5	-0.469344	-1.064803	0.397059	0.774100	1.000
521	REACTOME_MYD88_INDEPENDENT_TLR4_CASCADE	R-RNO-166166	8	-0.404275	-1.065823	0.386364	0.775602	1.000
522	REACTOME_RHO_GTPASE_EFFECTORS	R-RNO-195258	9	-0.382580	-1.066570	0.379447	0.777547	1.000
523	REACTOME_INTERLEUKIN_17_SIGNALING	R-RNO-448424	6	-0.447398	-1.067316	0.374150	0.779940	1.000
524	GOBP_MUSCLE_CELL_DEVELOPMENT	GO:0055001	5	-0.471427	-1.069166	0.373757	0.779466	1.000
525	GOBP_CELLULAR_CARBOHYDRATE_METABOLIC_PROCESS	GO:0044262	5	-0.472915	-1.069227	0.346809	0.783131	1.000
526	GOBP_REGULATION_OF_SMALL_MOLECULE_METABOLIC_PR...	GO:0062012	9	-0.384896	-1.070763	0.344000	0.783572	1.000
527	REACTOME_RNA_POLYMERASE_II_TRANSCRIPTION	R-RNO-73857	20	-0.297121	-1.077177	0.332645	0.772374	1.000
528	GOBP_POSITIVE_REGULATION_OF_MYELOID_CELL_DIFFE...	GO:0045639	7	-0.426880	-1.089351	0.354379	0.748122	1.000
529	GOBP_ENDOTHELIAL_CELL_MIGRATION	GO:0043542	8	-0.413113	-1.093696	0.337278	0.742302	1.000
530	GOBP_POSITIVE_REGULATION_OF_BINDING	GO:0051099	6	-0.450264	-1.094518	0.346473	0.744404	1.000
531	REACTOME_SIGNALING_BY_RECEPTOR_TYROSINE_KINASES	R-RNO-9006934	26	-0.290839	-1.095382	0.326531	0.746264	1.000
532	GOBP_REGULATION_OF_CELL_ADHESION	GO:0030155	13	-0.352098	-1.096289	0.353319	0.747944	1.000
533	GOBP_POSITIVE_REGULATION_OF_CELL_DIFFERENTIATION	GO:0045597	27	-0.282151	-1.096442	0.326572	0.751471	1.000
534	REACTOME_INNATE_IMMUNE_SYSTEM	R-RNO-168249	16	-0.330559	-1.098612	0.331967	0.750533	1.000
535	REACTOME_TOLL_LIKE_RECEPTOR_9_TLR9_CASCADE	R-RNO-168138	8	-0.404275	-1.099496	0.330578	0.752523	1.000
536	GOBP_PROTEIN_DEPHOSPHORYLATION	GO:0006470	7	-0.443867	-1.102257	0.318182	0.750147	1.000
537	GOBP_POSITIVE_REGULATION_OF_BIOSYNTHETIC_PROCESS	GO:0009891	27	-0.282950	-1.102967	0.337963	0.752552	1.000
538	HALLMARK_UV_RESPONSE_UP	M5941	5	-0.493858	-1.104883	0.353783	0.752263	1.000
539	GOBP_REGULATION_OF_IMMUNE_SYSTEM_PROCESS	GO:0002682	21	-0.301268	-1.105639	0.300216	0.754703	1.000
540	GOBP_BLOOD_VESSEL_ENDOTHELIAL_CELL_MIGRATION	GO:0043534	8	-0.413113	-1.107592	0.325397	0.754224	1.000
541	GOBP_PROTEIN_LOCALIZATION_TO_MEMBRANE	GO:0072657	13	-0.357280	-1.111264	0.319743	0.749675	1.000
542	GOBP_CELLULAR_RESPONSE_TO_INORGANIC_SUBSTANCE	GO:0071241	11	-0.370174	-1.113550	0.331313	0.748361	1.000
543	GOBP_RESPONSE_TO_GROWTH_FACTOR	GO:0070848	18	-0.320566	-1.114251	0.313978	0.750833	1.000
544	GOBP_TISSUE_MORPHOGENESIS	GO:0048729	7	-0.448841	-1.115709	0.341202	0.751663	1.000
545	GOBP_TISSUE_MIGRATION	GO:0090130	9	-0.405878	-1.121775	0.314286	0.741900	1.000
546	REACTOME_INFECTIOUS_DISEASE	R-HSA-5663205	12	-0.359778	-1.122194	0.304255	0.745183	1.000
547	GOBP_DEPHOSPHORYLATION	GO:0016311	7	-0.443867	-1.128033	0.309237	0.736034	1.000
548	GOBP_MEMBRANE_PROTEIN_PROTEOLYSIS	GO:0033619	6	-0.479058	-1.129251	0.312227	0.737551	1.000
549	GOBP_NEGATIVE_REGULATION_OF_LOCOMOTION	GO:0040013	5	-0.496614	-1.130574	0.295833	0.738519	1.000
550	GOBP_RESPONSE_TO_LIGHT_STIMULUS	GO:0009416	11	-0.376255	-1.133790	0.317895	0.735093	1.000
551	GOBP_REGULATION_OF_CELLULAR_PROTEIN_LOCALIZATION	GO:1903827	12	-0.373706	-1.139122	0.293182	0.726784	1.000
552	GOBP_TRANSMEMBRANE_TRANSPORT	GO:0055085	27	-0.291497	-1.139126	0.289238	0.730977	1.000
553	GOBP_POSITIVE_REGULATION_OF_NEURON_DEATH	GO:1901216	5	-0.509087	-1.143212	0.314815	0.725663	1.000
554	GOBP_FC_EPSILON_RECEPTOR_SIGNALING_PATHWAY	GO:0038095	7	-0.457847	-1.145332	0.301518	0.724984	1.000
555	GOBP_NEGATIVE_REGULATION_OF_APOPTOTIC_SIGNALIN...	GO:2001234	7	-0.459021	-1.147099	0.292887	0.725161	1.000
556	GOBP_CARBOHYDRATE_METABOLIC_PROCESS	GO:0005975	9	-0.404643	-1.147320	0.299401	0.729039	1.000

Continuation of Table **S7**

	NAME	ID	SIZE	ES	NES	NOM.p.val	FDR.q.val	FWER.p.val
557	GOBP_NEGATIVE_REGULATION_OF_PHOSPHORUS_METABOL...	GO:0010563	9	-0.402895	-1.147775	0.290456	0.732394	1.000
558	GOBP_RESPONSE_TO_BIOTIC_STIMULUS	GO:0009607	17	-0.330958	-1.148909	0.263636	0.734046	1.000
559	GOBP_RESPONSE_TO_INSULIN	GO:0032868	5	-0.512939	-1.150783	0.301969	0.734051	1.000
560	REACTOME_MAPK_TARGETS_NUCLEAR_EVENTS_MEDIATED_...	R-HSA-450282	5	-0.517623	-1.153926	0.301010	0.731214	1.000
561	GOBP_VASCULATURE_DEVELOPMENT	GO:0001944	17	-0.336227	-1.155498	0.272917	0.732012	1.000
562	REACTOME_DISEASES_OF_SIGNAL_TRANSDUCTION_BY_GR...	R-HSA-5663202	12	-0.376228	-1.156413	0.269076	0.734648	1.000
563	GOBP_CELL_CELL_JUNCTION_ASSEMBLY	GO:0007043	5	-0.518789	-1.158427	0.289628	0.734235	1.000
564	GOBP_NEGATIVE_REGULATION_OF_RESPONSE_TO_STIMULUS	GO:0048585	16	-0.342970	-1.160829	0.261702	0.732601	1.000
565	GOBP_RESPONSE_TO_CYTOKINE	GO:0034097	23	-0.317811	-1.161548	0.273138	0.735465	1.000
566	GOBP_RESPONSE_TO_DRUG	GO:0042493	11	-0.394379	-1.162359	0.262821	0.738054	1.000
567	GOBP_PROTEIN_CATABOLIC_PROCESS	GO:0030163	8	-0.437013	-1.163100	0.286008	0.741101	1.000
568	GOBP_ORGANONITROGEN_COMPOUND_CATABOLIC_PROCESS	GO:1901565	10	-0.404112	-1.167916	0.278373	0.733660	1.000
569	HALLMARK_EPITHELIAL_MESENCHYMAL_TRANSITION	M5930	5	-0.530086	-1.169265	0.299413	0.734929	1.000
570	REACTOME_FC_EPSILON_RECEPTOR_FCERI_SIGNALING	R-RNO-2454202	7	-0.457847	-1.173043	0.260870	0.729951	1.000
571	GOBP_CYTOKINE_MEDIATED_SIGNALING_PATHWAY	GO:0019221	16	-0.350068	-1.177802	0.274678	0.722589	1.000
572	GOBP_REGULATION_OF_CARBOHYDRATE_METABOLIC_PROCESS	GO:0006109	5	-0.529298	-1.179125	0.295833	0.724295	1.000
573	GOBP_ANATOMICAL_STRUCTURE_FORMATION_INVOLVED_I...	GO:0048646	20	-0.331459	-1.181175	0.246888	0.723879	1.000
574	GOBP_OSTEOBLAST_DIFFERENTIATION	GO:0001649	5	-0.528953	-1.186429	0.288066	0.716058	1.000
575	GOBP_REGULATION_OF_CARBOHYDRATE_BIOSYNTHETIC_P...	GO:0043255	5	-0.529298	-1.188145	0.273453	0.716261	1.000
576	GOBP_POSITIVE_REGULATION_OF_DNA_BINDING_TRANSC...	GO:0051091	10	-0.417960	-1.190442	0.264192	0.715117	1.000
577	HALLMARK_HYPOXIA	M5891	5	-0.521764	-1.193107	0.268537	0.713096	1.000
578	GOBP_GROWTH	GO:0040007	14	-0.362667	-1.194045	0.217778	0.715498	1.000
579	GOBP_CARBOHYDRATE_BIOSYNTHETIC_PROCESS	GO:0016051	5	-0.529298	-1.194274	0.255274	0.719759	1.000
580	GOBP_T_CELL_ACTIVATION	GO:0042110	8	-0.439166	-1.194497	0.246781	0.724202	1.000
581	GOBP_BLOOD_VESSEL_MORPHOGENESIS	GO:0048514	14	-0.383743	-1.201805	0.228512	0.710959	1.000
582	GOBP_POSITIVE_REGULATION_OF_CELL_ADHESION	GO:0045785	7	-0.470971	-1.202342	0.230937	0.714346	1.000
583	GOBP_RESPONSE_TO_UV	GO:0009411	5	-0.524583	-1.203418	0.240938	0.716795	1.000
584	GOBP_CELL_CELL_JUNCTION_ORGANIZATION	GO:0045216	6	-0.503628	-1.204850	0.236253	0.718153	1.000
585	GOBP_RESPONSE_TO_TUMOR_NECROSIS_FACTOR	GO:0034612	7	-0.478162	-1.206102	0.253731	0.720159	1.000
586	GOBP_BIOLOGICAL_PROCESS_INVOLVED_IN_SYMBIOTIC_...	GO:004440	10	-0.414804	-1.210546	0.233546	0.714001	1.000
587	GOBP_CALCIUM_ION_TRANSPORT_INTO_CYTOSOL	GO:0060402	7	-0.482775	-1.213035	0.253138	0.712839	1.000
588	GOBP_REGULATION_OF_DNA_BINDING_TRANSCRIPTION_F...	GO:0051090	16	-0.360993	-1.214691	0.236287	0.713508	1.000
589	GOBP_POSITIVE_REGULATION_OF_TRANSCRIPTION_BY_R...	GO:0045944	24	-0.324072	-1.217251	0.188437	0.711888	1.000
590	GOBP_GENERATION_OF_PRECURSOR_METABOLITES_AND_E...	GO:0006091	5	-0.540143	-1.218163	0.247401	0.714738	1.000
591	GOBP_NEGATIVE_REGULATION_OF_NUCLEOBASE_CONTAIN...	GO:0045934	13	-0.388176	-1.220782	0.214912	0.713221	1.000
592	REACTOME_CELLULAR_SENESCENCE	R-RNO-2559583	6	-0.520283	-1.224770	0.250000	0.708187	1.000
593	GOBP_SMOOTH_MUSCLE_CELL_PROLIFERATION	GO:0048659	6	-0.502625	-1.225034	0.234216	0.712949	1.000
594	GOBP_CARDIAC_MUSCLE_TISSUE_DEVELOPMENT	GO:0048738	6	-0.517251	-1.225409	0.247951	0.717445	1.000
595	GOBP_CIRCULATORY_SYSTEM_DEVELOPMENT	GO:0072359	21	-0.342821	-1.226122	0.204167	0.721104	1.000
596	GOBP_ANION_TRANSMEMBRANE_TRANSPORT	GO:0098656	7	-0.494550	-1.229908	0.216769	0.716618	1.000
597	GOBP_IMMUNE_EFFECTOR_PROCESS	GO:0002252	11	-0.409554	-1.230782	0.210989	0.719990	1.000
598	GOBP_REGULATION_OF_CELL_CYCLE	GO:0051726	13	-0.387077	-1.234892	0.210412	0.714926	1.000
599	GOBP_CALCIUM_ION_TRANSMEMBRANE_TRANSPORT	GO:0070588	7	-0.482775	-1.238704	0.223762	0.710275	1.000

Continuation of Table **S7**

	NAME	ID	SIZE	ES	NES	NOM.p.val	FDR.q.val	FWER.p.val
600	GOBP_HEART_DEVELOPMENT	GO:0007507	6	-0.517251	-1.239939	0.209205	0.712925	1.000
601	REACTOME_SENESCENCE_ASSOCIATED_SECRETORY_PHENO...	R-RNO-2559582	6	-0.520283	-1.240168	0.210204	0.717999	1.000
602	GOBP_MORPHOGENESIS_OF_AN_EPITHELIUM	GO:0002009	6	-0.521096	-1.243535	0.223790	0.715126	1.000
603	GOBP_NEGATIVE_REGULATION_OF_PROTEOLYSIS	GO:0045861	8	-0.477530	-1.245738	0.213531	0.715072	1.000
604	GOBP_CYTOSOLIC_CALCIUM_ION_TRANSPORT	GO:0060401	7	-0.482775	-1.247522	0.210526	0.716020	1.000
605	GOBP_NEGATIVE_REGULATION_OF_HYDROLASE_ACTIVITY	GO:0051346	11	-0.420789	-1.248377	0.203947	0.719563	1.000
606	GOBP_LIPID_METABOLIC_PROCESS	GO:0006629	10	-0.441926	-1.249168	0.200855	0.723370	1.000
607	GOBP_REGULATION_OF_WNT_SIGNALING_PATHWAY	GO:0030111	5	-0.565248	-1.251328	0.226891	0.723427	1.000
608	REACTOME_LEISHMANIA_INFECTION	R-HSA-9658195	10	-0.427030	-1.251585	0.191176	0.728936	1.000
609	GOBP_MACROMOLECULE_CATABOLIC_PROCESS	GO:0009057	11	-0.425265	-1.253382	0.200413	0.730194	1.000
610	GOBP_FC_RECEPTOR_SIGNALING_PATHWAY	GO:0038093	8	-0.478834	-1.253430	0.185950	0.736469	1.000
611	GOBP_RESPONSE_TO_ORGANIC_CYCLIC_COMPOUND	GO:0014070	20	-0.353629	-1.254105	0.197368	0.740998	1.000
612	GOBP_CELLULAR_RESPONSE_TO_EXTERNAL_STIMULUS	GO:0071496	10	-0.442476	-1.256419	0.203285	0.740795	1.000
613	GOBP_MUSCLE_CELL_PROLIFERATION	GO:0033002	8	-0.484818	-1.261805	0.171843	0.731964	1.000
614	GOBP_POLYSACCHARIDE_METABOLIC_PROCESS	GO:0005976	5	-0.569708	-1.265789	0.185031	0.727453	1.000
615	GOBP_NEGATIVE_REGULATION_OF_PROTEIN_MODIFICATI...	GO:0031400	10	-0.449880	-1.281380	0.179654	0.690051	1.000
616	GOBP_REGULATION_OF_ANATOMICAL_STRUCTURE_SIZE	GO:0090066	10	-0.444547	-1.281516	0.190678	0.696015	1.000
617	GOBP_IMMUNE_RESPONSE_REGULATING_SIGNALING_PATHWAY	GO:000276	8	-0.478834	-1.282527	0.190377	0.699836	1.000
618	GOBP_RESPIRATORY_SYSTEM_DEVELOPMENT	GO:0060541	6	-0.533856	-1.284334	0.190476	0.701377	1.000
619	GOBP_CELL_CYCLE	GO:0007049	21	-0.349020	-1.286035	0.165198	0.703879	1.000
620	GOBP_PROTEIN_LOCALIZATION_TO_CELL_JUNCTION	GO:1902414	6	-0.539079	-1.286454	0.193548	0.709356	1.000
621	GOBP_RESPONSE_TO_ABIOTIC_STIMULUS	GO:0009628	27	-0.337552	-1.288025	0.142857	0.711664	1.000
622	GOBP_MONONUCLEAR_CELL_DIFFERENTIATION	GO:1903131	7	-0.491649	-1.295420	0.183299	0.697773	1.000
623	GOBP_EPIDERMIS_DEVELOPMENT	GO:0008544	5	-0.591297	-1.299997	0.172269	0.691564	1.000
624	REACTOME_DEATH_RECEPTOR_SIGNALLING	R-RNO-73887	5	-0.570238	-1.301921	0.184000	0.692282	1.000
625	GOBP_POSITIVE_REGULATION_OF_IMMUNE_SYSTEM_PROCESS	GO:0002684	18	-0.370201	-1.310298	0.155844	0.676022	1.000
626	GOBP_REGULATION_OF_APOPTOTIC_SIGNALING_PATHWAY	GO:2001233	10	-0.448022	-1.311844	0.168085	0.678786	1.000
627	GOBP_RESPONSE_TO_MOLECULE_OF_BACTERIAL_ORIGIN	GO:0002237	13	-0.414004	-1.311908	0.141962	0.685612	1.000
628	GOBP_EMBRYO_DEVELOPMENT_ENDING_IN_BIRTH_OR_EGG...	GO:0009792	9	-0.486696	-1.314993	0.162698	0.684204	1.000
629	GOBP_APOPTOTIC_SIGNALING_PATHWAY	GO:0097190	15	-0.408208	-1.315730	0.138075	0.689218	1.000
630	GOBP_GLUCOSE_METABOLIC_PROCESS	GO:0006006	6	-0.551188	-1.322651	0.154661	0.678137	1.000
631	GOBP_NEGATIVE_REGULATION_OF_PROTEIN_METABOLIC_...	GO:0051248	20	-0.370325	-1.323908	0.127835	0.682022	1.000
632	GOBP_MONOSACCHARIDE_METABOLIC_PROCESS	GO:0005996	6	-0.551188	-1.331230	0.172211	0.668270	1.000
633	GOBP_REGULATION_OF_ORGAN_GROWTH	GO:0046620	5	-0.589085	-1.335141	0.159509	0.663790	1.000
634	GOBP_DEVELOPMENTAL_GROWTH	GO:0048589	10	-0.466972	-1.338962	0.158730	0.659872	1.000
635	GOBP_RESPONSE_TO_BACTERIUM	GO:0009617	13	-0.414004	-1.339566	0.136364	0.665467	1.000
636	GOBP_ORGAN_GROWTH	GO:0035265	5	-0.589085	-1.344123	0.136170	0.659775	1.000
637	GOBP_NEGATIVE_REGULATION_OF_CATALYTIC_ACTIVITY	GO:0043086	17	-0.397651	-1.344544	0.122318	0.665965	1.000
638	GOBP_STRIATED_MUSCLE_CELL_DIFFERENTIATION	GO:0051146	9	-0.490181	-1.345129	0.136268	0.672057	1.000
639	GOBP_EMBRYONIC_ORGAN_DEVELOPMENT	GO:0048568	7	-0.529485	-1.345221	0.139442	0.679514	1.000
640	GOBP_MITOCHONDRION_ORGANIZATION	GO:0007005	7	-0.538929	-1.346241	0.160000	0.684583	1.000
641	GOBP_ACTIVATION_OF_IMMUNE_RESPONSE	GO:0002253	5	-0.609398	-1.347338	0.140787	0.689059	1.000
642	GOBP_SKIN_DEVELOPMENT	GO:0043588	5	-0.608173	-1.350117	0.151394	0.689062	1.000

Continuation of Table **S7**

	NAME	ID	SIZE	ES	NES	NOM.p.val	FDR.q.val	FWER.p.val
643	GOBP_NEGATIVE_REGULATION_OF_BIOSYNTHETIC_PROCESS	GO:0009890	13	-0.437255	-1.353876	0.125532	0.685923	1.000
644	GOBP_NEGATIVE_REGULATION_OF_CATABOLIC_PROCESS	GO:0009895	8	-0.510338	-1.355736	0.121535	0.688937	1.000
645	GOBP_REGULATION_OF_PROTEIN_CATABOLIC_PROCESS	GO:0042176	7	-0.526907	-1.357839	0.130802	0.691271	1.000
646	GOBP_REGULATION_OF_CATABOLIC_PROCESS	GO:0009894	13	-0.432226	-1.358588	0.127119	0.698141	1.000
647	GOBP_NEGATIVE_REGULATION_OF_INTRACELLULAR_SIGN...	GO:1902532	7	-0.533457	-1.363170	0.136264	0.692992	1.000
648	GOBP_APOPTOTIC_MITOCHONDRIAL_CHANGES	GO:0008637	6	-0.579419	-1.368273	0.152220	0.687266	1.000
649	GOBP_CELL_CELL_ADHESION	GO:0098609	14	-0.416022	-1.369775	0.111597	0.691535	1.000
650	GOBP_POSITIVE_REGULATION_OF_CELL_DEATH	GO:0010942	19	-0.385208	-1.369989	0.117914	0.700135	1.000
651	GOBP_RESPONSE_TO_METAL_ION	GO:0010038	15	-0.416804	-1.373779	0.111588	0.697334	1.000
652	HALLMARK_TNFA_SIGNALING_VIA_NFKB	M5890	14	-0.438501	-1.376416	0.128151	0.698010	1.000
653	GOBP_RESPONSE_TO_CADMIUM_ION	GO:0046686	6	-0.573837	-1.379685	0.135628	0.698165	1.000
654	GOBP_LEUKOCYTE_CELL_CELL_ADHESION	GO:0007159	6	-0.580612	-1.381186	0.126253	0.703336	1.000
655	GOBP_RAS_PROTEIN_SIGNAL_TRANSDUCTION	GO:0007265	5	-0.617564	-1.384816	0.125000	0.700912	1.000
656	GOBP_REGULATION_OF_GROWTH	GO:0040008	13	-0.433207	-1.392815	0.106522	0.685317	1.000
657	GOBP_TUBE_DEVELOPMENT	GO:0035295	18	-0.390908	-1.393722	0.095745	0.692562	1.000
658	GOBP_EPITHELIUM_DEVELOPMENT	GO:0060429	15	-0.427629	-1.401216	0.097458	0.679332	1.000
659	GOBP_NEGATIVE_REGULATION_OF_PEPTIDASE_ACTIVITY	GO:0010466	6	-0.585469	-1.401791	0.112245	0.687830	1.000
660	GOBP_REGULATION_OF_PROTEIN_LOCALIZATION_TO_MEM...	GO:1905475	7	-0.547828	-1.401816	0.116935	0.698316	1.000
661	GOBP_RESPONSE_TO_INORGANIC_SUBSTANCE	GO:0010035	19	-0.399930	-1.403198	0.076253	0.704564	1.000
662	GOBP_REGULATION_OF_ANION_TRANSMEMBRANE_TRANSPORT	GO:1903959	5	-0.638373	-1.413985	0.115914	0.680861	1.000
663	HALLMARK_PI3K_AKT_MTOR_SIGNALING	M5923	6	-0.595923	-1.417757	0.114053	0.680041	1.000
664	GOBP_DEVELOPMENTAL_CELL_GROWTH	GO:0048588	5	-0.646082	-1.417794	0.095918	0.691151	1.000
665	GOBP_REGULATION_OF_CELLULAR_AMIDE_METABOLIC_PR...	GO:0034248	9	-0.500525	-1.417989	0.086498	0.701975	1.000
666	GOBP_RESPONSE_TO_RADIATION	GO:0009314	13	-0.441499	-1.419133	0.103926	0.710117	1.000
667	GOBP_NEGATIVE_REGULATION_OF_TRANSMEMBRANE_TRAN...	GO:0034763	6	-0.585290	-1.422206	0.104167	0.712038	1.000
668	GOBP_MATERNAL_PROCESS_INVOLVED_IN_FEMALE_PREGN...	GO:0060135	5	-0.630643	-1.422640	0.095041	0.723020	1.000
669	GOBP_NEGATIVE_REGULATION_OF_TRANSCRIPTION_BY_R...	GO:0000122	10	-0.486197	-1.422709	0.085339	0.735710	1.000
670	REACTOME_CYTOKINE_SIGNALING_IN_IMMUNE_SYSTEM	R-RNO-1280215	21	-0.390135	-1.427272	0.067391	0.733226	1.000
671	REACTOME_TRANSCRIPTIONAL_REGULATION_BY_TP53	R-RNO-3700989	6	-0.598846	-1.432778	0.113734	0.726744	1.000
672	REACTOME_ESTROGEN_DEPENDENT_NUCLEAR_EVENTS_DOW...	R-RNO-9634638	5	-0.646414	-1.437984	0.083658	0.719940	1.000
673	GOBP_NEGATIVE_REGULATION_OF_MOLECULAR_FUNCTION	GO:0044092	24	-0.383490	-1.438915	0.064103	0.729941	1.000
674	GOBP_RESPONSE_TO_OXIDATIVE_STRESS	GO:0006979	8	-0.537564	-1.448055	0.094456	0.711033	1.000
675	GOBP_RESPONSE_TO_REACTIVE_OXYGEN_SPECIES	GO:0000302	8	-0.537564	-1.452429	0.096311	0.709326	1.000
676	GOBP_POSITIVE_REGULATION_OF_DEVELOPMENTAL_GROWTH	GO:0048639	8	-0.547288	-1.453682	0.082474	0.719063	1.000
677	GOBP_CELL_GROWTH	GO:0016049	10	-0.495758	-1.460859	0.082136	0.707137	1.000
678	GOBP_POSITIVE_REGULATION_OF_GROWTH	GO:0045927	9	-0.536625	-1.463954	0.090909	0.711907	1.000
679	GOBP_CELLULAR_RESPONSE_TO_CADMIUM_ION	GO:0071276	5	-0.637243	-1.465920	0.078556	0.720059	1.000
680	GOBP_TUBE_MORPHOGENESIS	GO:0035239	15	-0.439518	-1.478317	0.063291	0.689578	1.000
681	GOBP_CELLULAR_LIPID_METABOLIC_PROCESS	GO:0044255	7	-0.580407	-1.489710	0.072835	0.664708	1.000
682	GOBP_CELLULAR_RESPONSE_TO_CHEMICAL_STRESS	GO:0062197	7	-0.587323	-1.504229	0.073996	0.631122	1.000
683	GOBP_NEGATIVE_REGULATION_OF_CYSTEINE_TYPE_ENDO...	GO:2000117	5	-0.662376	-1.504634	0.057940	0.644852	1.000
684	GOBP_CELLULAR_RESPONSE_TO_REACTIVE_OXYGEN_SPECIES	GO:0034614	7	-0.587323	-1.507702	0.069530	0.649586	1.000
685	GOBP_LEUKOCYTE_MIGRATION	GO:0050900	6	-0.631875	-1.510705	0.069388	0.654676	1.000

Continuation of Table **S7**

	NAME	ID	SIZE	ES	NES	NOM.p.val	FDR.q.val	FWER.p.val
686	GOBP_NEGATIVE_REGULATION_OF_BINDING	GO:0051100	6	-0.622556	-1.512076	0.066000	0.666636	1.000
687	GOBP_RESPONSE_TO_EXTRACELLULAR_STIMULUS	GO:0009991	13	-0.467150	-1.519617	0.058065	0.656393	1.000
688	GOBP_PROTEIN_LOCALIZATION_TO_CELL_PERIPHERY	GO:1990778	12	-0.496423	-1.520347	0.055666	0.671493	1.000
689	REACTOME_EXTRA_NUCLEAR_ESTROGEN_SIGNALING	R-RNO-9009391	7	-0.607930	-1.523406	0.062745	0.679231	1.000
690	GOBP_MULTI_MULTICELLULAR_ORGANISM_PROCESS	GO:0044706	8	-0.557755	-1.525546	0.062366	0.690202	1.000
691	GOBP_HOMEOSTASIS_OF_NUMBER_OF_CELLS	GO:0048872	5	-0.685487	-1.536664	0.058943	0.666886	1.000
692	GOBP_REGULATION_OF_CELLULAR_COMPONENT_SIZE	GO:0032535	7	-0.603828	-1.545356	0.054167	0.654699	1.000
693	GOBP_PEPTIDE_BIOSYNTHETIC_PROCESS	GO:0043043	6	-0.639139	-1.553564	0.047414	0.643668	1.000
694	GOBP_AMIDE_BIOSYNTHETIC_PROCESS	GO:0043604	6	-0.639139	-1.554288	0.050201	0.661663	1.000
695	GOBP_GLAND_DEVELOPMENT	GO:0048732	7	-0.625519	-1.561354	0.055985	0.656445	1.000
696	GOBP_IMPORT_INTO_CELL	GO:0098657	6	-0.646326	-1.563310	0.048583	0.671175	1.000
697	GOBP_REGULATION_OF_PROTEIN_LOCALIZATION_TO_CEL...	GO:1904375	6	-0.659548	-1.569864	0.051181	0.668626	1.000
698	GOBP_PROTEIN_LOCALIZATION_TO_PLASMA_MEMBRANE	GO:0072659	7	-0.629465	-1.578859	0.036893	0.655763	1.000
699	GOBP_POSITIVE_REGULATION_OF_PRI_MIRNA_TRANSCRI...	GO:1902895	7	-0.628178	-1.584943	0.041754	0.655981	1.000
700	GOBP_PEPTIDE_METABOLIC_PROCESS	GO:0006518	11	-0.533437	-1.587809	0.033543	0.669259	1.000
701	GOBP_POSITIVE_REGULATION_OF_SMOOTH_MUSCLE_CELL...	GO:0048661	5	-0.715656	-1.593259	0.039014	0.673200	1.000
702	GOBP_POSITIVE_REGULATION_OF_CELL_CELL_ADHESION	GO:0022409	5	-0.717485	-1.601114	0.027837	0.670864	1.000
703	REACTOME_PTEN_REGULATION	R-RNO-6807070	5	-0.711920	-1.602902	0.036217	0.693613	1.000
704	GOBP_PRI_MIRNA_TRANSCRIPTION_BY_RNA_POLYMERASE_II	GO:0061614	7	-0.628178	-1.608056	0.041841	0.702654	1.000
705	GOBP_RESPONSE_TO_NUTRIENT	GO:0007584	5	-0.725790	-1.608398	0.036364	0.736059	1.000
706	GOBP_POSITIVE_REGULATION_OF_LEUKOCYTE_CELL_CEL...	GO:1903039	5	-0.717485	-1.609459	0.028112	0.770410	1.000
707	GOBP_REGULATION_OF_PROTEIN_BINDING	GO:0043393	7	-0.630009	-1.613016	0.032000	0.794032	1.000
708	GOBP_IN_UTERO_EMBRYONIC_DEVELOPMENT	GO:0001701	8	-0.606849	-1.613797	0.034632	0.835592	1.000
709	GOBP_REGULATION_OF_DEVELOPMENTAL_GROWTH	GO:0048638	9	-0.583251	-1.615060	0.037773	0.880631	1.000
710	HALLMARK_APOPTOSIS	M5902	6	-0.673505	-1.620250	0.030242	0.910137	1.000
711	GOBP_SPROUTING_ANGIOGENESIS	GO:0002040	5	-0.719135	-1.623936	0.026804	0.955039	1.000
712	GOBP_REGULATION_OF_EXTRINSIC_APOPTOTIC_SIGNALI...	GO:2001236	6	-0.686321	-1.624386	0.018557	1.000000	1.000
713	GOBP_AGING	GO:0007568	13	-0.519766	-1.636957	0.028630	1.000000	1.000
714	REACTOME_SIGNALING_BY_INTERLEUKINS	R-RNO-449147	15	-0.502410	-1.638089	0.035197	1.000000	1.000
715	GOBP_ENDOCRINE_SYSTEM_DEVELOPMENT	GO:0035270	6	-0.699680	-1.661875	0.028513	1.000000	1.000
716	GOBP_REGULATION_OF_CELL_SIZE	GO:0008361	5	-0.755216	-1.662006	0.026804	1.000000	1.000
717	GOBP_REGULATION_OF_BINDING	GO:0051098	11	-0.559021	-1.662269	0.028986	1.000000	1.000
718	REACTOME_ESR_MEDIATED_SIGNALING	R-RNO-8939211	8	-0.644575	-1.694483	0.026477	1.000000	1.000
719	REACTOME_SIGNALING_BY_NUCLEAR_RECEPTORS	R-RNO-9006931	8	-0.644575	-1.723517	0.014403	1.000000	0.995
720	GOBP_EXTRINSIC_APOPTOTIC_SIGNALING_PATHWAY	GO:0097191	7	-0.690277	-1.734438	0.014706	1.000000	0.989
721	GOBP_POSITIVE_REGULATION_OF_CELL_GROWTH	GO:0030307	6	-0.755612	-1.774435	0.002049	1.000000	0.972
722	GOBP_CELLULAR_AMIDE_METABOLIC_PROCESS	GO:0043603	12	-0.566680	-1.774934	0.006342	1.000000	0.972
723	REACTOME_INTERLEUKIN_4_AND_INTERLEUKIN_13_SIGN...	R-RNO-6785807	8	-0.687500	-1.818746	0.010121	1.000000	0.928
724	GOBP_RESPONSE_TO_MECHANICAL_STIMULUS	GO:0009612	6	-0.773717	-1.850761	0.000000	1.000000	0.862

End of Table

Supplementary Table S8: SC cervical segment early profile (1 DPL peak) GSEA results.

Begin of Table **S8**

	NAME	ID	SIZE	ES	NES	NOM.p.val	FDR.q.val	FWER.p.val
0	GOBP_RESPONSE_TO_LIGHT_STIMULUS	GO:0009416	11	0.731334	2.024651	0.000000	0.156407	0.142
1	GOBP_PROTEIN_CONTAINING_COMPLEX_SUBUNIT_ORGANI...	GO:0043933	15	0.663449	1.974649	0.003026	0.160386	0.267
2	GOBP_MEMORY	GO:0007613	13	0.673529	1.937396	0.000000	0.165605	0.381
3	GOBP_REGULATION_OF_BINDING	GO:0051098	11	0.705199	1.920197	0.000000	0.158111	0.456
4	GOBP_REGULATION_OF_PROTEIN_BINDING	GO:0043393	7	0.809960	1.910167	0.003284	0.141292	0.497
5	GOBP_INFLAMMATORY_RESPONSE	GO:0006954	12	0.654238	1.886026	0.000000	0.152717	0.584
6	GOBP_RESPONSE_TO_RADIATION	GO:0009314	13	0.639975	1.882968	0.001608	0.135849	0.595
7	GOBP_RESPONSE_TO_NERVE_GROWTH_FACTOR	GO:1990089	7	0.776983	1.854240	0.003241	0.161113	0.692
8	REACTOME_INTERLEUKIN_4_AND_INTERLEUKIN_13_SIGN...	R-RNO-6785807	8	0.719360	1.810831	0.003236	0.221923	0.853
9	GOBP_POSITIVE_REGULATION_OF_BINDING	GO:0051099	6	0.746774	1.714130	0.008264	0.504614	0.996
10	GOBP_CYTOKINE_MEDIATED_SIGNALING_PATHWAY	GO:0019221	16	0.553707	1.702685	0.013235	0.507436	0.996
11	GOBP_REGULATION_OF_NEURON_DIFFERENTIATION	GO:0045664	5	0.778648	1.693701	0.017974	0.499902	0.996
12	GOBP_NEGATIVE_REGULATION_OF_PEPTIDASE_ACTIVITY	GO:0010466	6	0.718984	1.653030	0.017857	0.649190	0.999
13	REACTOME_SIGNALING_BY_INTERLEUKINS	R-RNO-449147	15	0.546048	1.634720	0.020772	0.697544	0.999
14	REACTOME_CYTOKINE_SIGNALING_IN_IMMUNE_SYSTEM	R-RNO-1280215	21	0.487838	1.625963	0.030726	0.698163	1.000
15	GOBP_NEGATIVE_REGULATION_OF_MOLECULAR_FUNCTION	GO:0044092	24	0.481572	1.622320	0.025373	0.673187	1.000
16	GOBP_EPITHELIAL_CELL_DIFFERENTIATION	GO:0030855	7	0.679012	1.620021	0.024958	0.645223	1.000
17	GOBP_NEGATIVE_REGULATION_OF_BINDING	GO:0051100	6	0.709012	1.611042	0.021173	0.653762	1.000
18	GOBP_CELLULAR_COMPONENT_DISASSEMBLY	GO:0022411	6	0.706912	1.608042	0.025000	0.632488	1.000
19	GOBP_POSITIVE_REGULATION_OF_PEPTIDYL_SERINE_PH...	GO:0033138	8	0.639284	1.601790	0.030596	0.630937	1.000
20	GOBP_REGULATION_OF_LIPID_METABOLIC_PROCESS	GO:0019216	7	0.661304	1.593920	0.025907	0.639143	1.000
21	GOBP_REGULATION_OF_CELLULAR_COMPONENT_SIZE	GO:0032535	7	0.663806	1.593518	0.023659	0.612050	1.000
22	GOBP_REGULATION_OF_OSTEOCLAST_DIFFERENTIATION	GO:0045670	5	0.734940	1.589716	0.027350	0.601488	1.000
23	GOBP_RECEPTOR_MEDIATED_ENDOCYTOSIS	GO:0006898	6	0.686854	1.585166	0.026981	0.596329	1.000
24	GOBP_REGULATION_OF_PROTEIN_LOCALIZATION_TO_MEM...	GO:1905475	7	0.666355	1.584227	0.033708	0.576909	1.000
25	GOBP_REGULATION_OF_PEPTIDYL_SERINE_PHOSPHORYLA...	GO:0033135	8	0.639284	1.573723	0.035439	0.597143	1.000
26	GOBP_POSITIVE_REGULATION_OF_CELL_DEATH	GO:0010942	19	0.491058	1.568551	0.047407	0.597376	1.000
27	GOBP_OSTEOCLAST_DIFFERENTIATION	GO:0030316	6	0.682927	1.561724	0.038898	0.605323	1.000
28	GOBP_MULTI_MULTICELLULAR_ORGANISM_PROCESS	GO:0044706	8	0.609696	1.553119	0.035256	0.622344	1.000
29	GOBP_REGULATION_OF_PEPTIDASE_ACTIVITY	GO:0052547	10	0.573977	1.529197	0.034591	0.707040	1.000
30	GOBP_RESPONSE_TO_UV	GO:0009411	5	0.696333	1.496278	0.035000	0.848450	1.000
31	GOBP_RESPONSE_TO_METAL_ION	GO:0010038	15	0.493430	1.489503	0.060423	0.859173	1.000
32	GOBP_PROTEIN_COMPLEX_OLIGOMERIZATION	GO:0051259	7	0.623111	1.488133	0.062500	0.840821	1.000
33	GOBP_REGULATION_OF_PROTEIN_LOCALIZATION_TO_CEL...	GO:1904375	6	0.651848	1.485393	0.051282	0.830233	1.000
34	GOBP_AGING	GO:0007568	13	0.509943	1.484576	0.046828	0.810366	1.000
35	GOBP_LONG_TERM_SYNAPTIC_DEPRESSION	GO:0060292	5	0.690196	1.478621	0.041806	0.818449	1.000
36	GOBP_INTRINSIC_APOPTOTIC_SIGNALING_PATHWAY	GO:0097193	5	0.695569	1.475786	0.048093	0.809136	1.000
37	GOBP_REGULATION_OF_CELL_DEATH	GO:0010941	34	0.405518	1.475658	0.047757	0.788345	1.000
38	GOBP_POSITIVE_REGULATION_OF_TRANSPORT	GO:0051050	26	0.432714	1.475257	0.060172	0.769975	1.000
39	GOBP_NEGATIVE_REGULATION_OF_LIPID_METABOLIC_PR...	GO:0045833	5	0.680186	1.457372	0.056995	0.835328	1.000
40	GOBP_NEGATIVE_REGULATION_OF_TRANSMEMBRANE_TRAN...	GO:0034763	6	0.644586	1.452012	0.064620	0.841271	1.000

Continuation of Table **S8**

	NAME	ID	SIZE	ES	NES	NOM.p.val	FDR.q.val	FWER.p.val
41	GOBP_DEFENSE_RESPONSE	GO:0006952	19	0.448065	1.440478	0.078669	0.877577	1.000
42	REACTOME_SIGNALING_BY_RECEPTOR_TYROSINE_KINASES	R-RNO-9006934	26	0.423253	1.432083	0.071756	0.898062	1.000
43	GOBP_CELLULAR_RESPONSE_TO_ABIOTIC_STIMULUS	GO:0071214	7	0.607824	1.424489	0.076800	0.915661	1.000
44	GOBP_LONG_TERM_MEMORY	GO:0007616	6	0.626814	1.417558	0.090150	0.931756	1.000
45	GOBP_NEGATIVE_REGULATION_OF_CYSTEINE_TYPE_ENDO...	GO:2000117	5	0.666466	1.414575	0.060504	0.927070	1.000
46	GOBP_RESPONSE_TO_INORGANIC_SUBSTANCE	GO:0010035	19	0.442936	1.413843	0.075893	0.910832	1.000
47	GOBP_RECEPTOR_METABOLIC_PROCESS	GO:0043112	5	0.649864	1.413676	0.087329	0.892677	1.000
48	GOBP_POSITIVE_REGULATION_OF_IMMUNE_SYSTEM_PROCESS	GO:0002684	18	0.443000	1.412332	0.097561	0.881013	1.000
49	GOBP_RECEPTOR_INTERNALIZATION	GO:0031623	5	0.649864	1.411213	0.081037	0.868797	1.000
50	GOBP_NEGATIVE_REGULATION_OF_CELL_DEATH	GO:0060548	22	0.420897	1.410717	0.081203	0.853872	1.000
51	GOBP_REGULATION_OF_FAT_CELL_DIFFERENTIATION	GO:0045598	6	0.616379	1.408797	0.109299	0.847150	1.000
52	GOBP_REGULATION_OF_CELL_SIZE	GO:0008361	5	0.658172	1.405710	0.096186	0.845925	1.000
53	GOBP_RESPONSE_TO_CYTOKINE	GO:0034097	23	0.425425	1.399262	0.076487	0.860231	1.000
54	GOBP_REGULATION_OF_RECEPTOR_MEDIATED_ENDOCYTOSIS	GO:0048259	5	0.654508	1.395425	0.086957	0.863283	1.000
55	GOBP_POSITIVE_REGULATION_OF_SMOOTH_MUSCLE_CELL...	GO:0048661	5	0.642403	1.393229	0.097403	0.858735	1.000
56	GOBP_REGULATION_OF_NEURON_PROJECTION_DEVELOPMENT	GO:0010975	16	0.449841	1.391981	0.078635	0.849382	1.000
57	GOBP_NEGATIVE_REGULATION_OF_LOCOMOTION	GO:0040013	5	0.651213	1.391131	0.098993	0.838860	1.000
58	GOBP_PROTEOLYSIS	GO:0006508	17	0.451662	1.390741	0.082840	0.826433	1.000
59	GOBP_COGNITION	GO:0050890	28	0.394444	1.384548	0.103499	0.839553	1.000
60	GOBP_ACTIVATION_OF_PROTEIN_KINASE_ACTIVITY	GO:0032147	10	0.514590	1.383987	0.107023	0.828201	1.000
61	REACTOME_POST_TRANSLATIONAL_PROTEIN_MODIFICATION	R-RNO-597592	7	0.580050	1.381619	0.095390	0.825779	1.000
62	GOBP_REGULATION_OF_ENDOCYTOSIS	GO:0030100	6	0.605406	1.379007	0.100993	0.824332	1.000
63	GOBP_REGULATION_OF_INFLAMMATORY_RESPONSE	GO:0050727	7	0.575485	1.374290	0.115192	0.833324	1.000
64	GOBP_IMMUNE_EFFECTOR_PROCESS	GO:0002252	11	0.483391	1.374238	0.116208	0.820729	1.000
65	GOBP_MYELOID_LEUKOCYTE_ACTIVATION	GO:0002274	8	0.550409	1.369653	0.116613	0.827405	1.000
66	GOBP_REGULATION_OF_IMMUNE_RESPONSE	GO:0050776	12	0.472895	1.367241	0.130094	0.824992	1.000
67	GOBP_LIPID_BIOSYNTHETIC_PROCESS	GO:0008610	6	0.594932	1.366774	0.125620	0.814742	1.000
68	GOBP_REGULATION_OF_DEFENSE_RESPONSE	GO:0031347	7	0.575485	1.362293	0.118314	0.822737	1.000
69	GOBP_NEGATIVE_REGULATION_OF_NEURON_DEATH	GO:1901215	11	0.487270	1.359693	0.110769	0.822197	1.000
70	GOBP_LIPID_METABOLIC_PROCESS	GO:0006629	10	0.503782	1.350586	0.131045	0.850484	1.000
71	GOBP_RESPONSE_TO_CADMIUM_ION	GO:0046686	6	0.587893	1.343796	0.113238	0.867106	1.000
72	GOBP_ACTIN_FILAMENT_BASED_PROCESS	GO:0030029	7	0.561922	1.341075	0.132787	0.866825	1.000
73	GOBP_POSITIVE_REGULATION_OF_ANION_TRANSPORT	GO:1903793	9	0.501529	1.339773	0.150000	0.860802	1.000
74	GOBP_REGULATION_OF_CELLULAR_CATABOLIC_PROCESS	GO:0031329	10	0.494057	1.334099	0.135350	0.873703	1.000
75	GOBP_REGULATION_OF_SMALL_MOLECULE_METABOLIC_PR...	GO:0062012	9	0.507014	1.333124	0.131833	0.866269	1.000
76	HALLMARK_ALLOGRAFT_REJECTION	M5950	7	0.555051	1.327995	0.122642	0.877645	1.000
77	GOBP_INOSITOL_LIPID_MEDIATED_SIGNALING	GO:0048017	8	0.537108	1.325488	0.138315	0.876894	1.000
78	GOBP_REGULATION_OF_LIPID_BIOSYNTHETIC_PROCESS	GO:0046890	6	0.594932	1.324216	0.141046	0.870868	1.000
79	GOBP_NEGATIVE_REGULATION_OF_SYNAPTIC_TRANSMISSION	GO:0050805	6	0.594143	1.323987	0.134021	0.860930	1.000
80	GOBP_NEGATIVE_REGULATION_OF_HYDROLASE_ACTIVITY	GO:0051346	11	0.475929	1.323606	0.159875	0.852291	1.000
81	GOBP_NEUROINFLAMMATORY_RESPONSE	GO:0150076	5	0.613684	1.323420	0.134775	0.842669	1.000
82	GOBP_RESPONSE_TO_NICOTINE	GO:0035094	7	0.546881	1.322992	0.162205	0.834242	1.000
83	GOBP_EXTRINSIC_APOPTOTIC_SIGNALING_PATHWAY	GO:0097191	7	0.544851	1.321975	0.159052	0.828229	1.000

Continuation of Table **S8**

	NAME	ID	SIZE	ES	NES	NOM.p.val	FDR.q.val	FWER.p.val
84	GOBP_REGULATION_OF_CYSTEINE_TYPE_ENDOPEPTIDASE...	GO:2000116	9	0.500109	1.319724	0.141935	0.827449	1.000
85	GOBP_CELLULAR_RESPONSE_TO_INORGANIC_SUBSTANCE	GO:0071241	11	0.473490	1.316081	0.143951	0.831694	1.000
86	GOBP_CELLULAR_AMIDE_METABOLIC_PROCESS	GO:0043603	12	0.454528	1.313527	0.136508	0.832016	1.000
87	GOBP_RESPONSE_TO_ACID_CHEMICAL	GO:0001101	6	0.578391	1.309235	0.145614	0.839707	1.000
88	REACTOME_MEMBRANE_TRAFFICKING	R-RNO-199991	5	0.605869	1.305858	0.143836	0.843474	1.000
89	GOBP_NEGATIVE_REGULATION_OF_APOPTOTIC_SIGNALIN...	GO:2001234	7	0.548127	1.303325	0.158706	0.844108	1.000
90	REACTOME_EXTRA_NUCLEAR_ESTROGEN_SIGNALING	R-RNO-900939	7	0.538187	1.302169	0.155449	0.839367	1.000
91	GOBP_SMOOTH_MUSCLE_CELL_PROLIFERATION	GO:0048659	6	0.568056	1.300100	0.150820	0.838406	1.000
92	GOBP_POSITIVE_REGULATION_OF_CYTOKINE_PRODUCTION	GO:0001819	7	0.547419	1.299915	0.161840	0.830129	1.000
93	GOBP_REGULATION_OF_CELLULAR_COMPONENT_MOVEMENT	GO:0051270	15	0.424757	1.296439	0.159574	0.833624	1.000
94	GOBP_MONOSACCHARIDE_METABOLIC_PROCESS	GO:0005996	6	0.573451	1.295088	0.154093	0.830227	1.000
95	GOBP_GLUCOSE_METABOLIC_PROCESS	GO:0006006	6	0.573451	1.293002	0.170213	0.829205	1.000
96	GOBP_REGULATION_OF_HYDROLASE_ACTIVITY	GO:0051336	22	0.380764	1.290903	0.157353	0.828274	1.000
97	GOBP_REGULATION_OF_PROTEIN_LOCALIZATION	GO:0032880	18	0.409564	1.290876	0.152466	0.819921	1.000
98	GOBP_ASSOCIATIVE_LEARNING	GO:0008306	7	0.535321	1.290528	0.155116	0.812999	1.000
99	GOBP_NEURON_DEVELOPMENT	GO:0048666	34	0.357748	1.290232	0.147059	0.805755	1.000
100	GOBP_PERIPHERAL_NERVOUS_SYSTEM_DEVELOPMENT	GO:0007422	9	0.493287	1.288591	0.166667	0.803450	1.000
101	GOBP_OSTEOBLAST_DIFFERENTIATION	GO:0001649	5	0.601396	1.285479	0.164076	0.806703	1.000
102	GOBP_NEGATIVE_REGULATION_OF_CATABOLIC_PROCESS	GO:0009895	8	0.512299	1.284125	0.180952	0.804028	1.000
103	GOBP_NEGATIVE_REGULATION_OF_PROTEOLYSIS	GO:0045861	8	0.516302	1.283868	0.177711	0.797241	1.000
104	GOBP_CELL_PROJECTION_ORGANIZATION	GO:0030030	35	0.352332	1.282446	0.147937	0.795125	1.000
105	GOBP_NEGATIVE_REGULATION_OF_CATALYTIC_ACTIVITY	GO:0043086	17	0.413724	1.282438	0.171429	0.787633	1.000
106	GOBP_REGULATION_OF_LONG_TERM_SYNAPTIC_POTENTIA...	GO:1900271	6	0.569506	1.280955	0.166392	0.785526	1.000
107	REACTOME_VESICLE_MEDIATED_TRANSPORT	R-RNO-5653656	5	0.605869	1.277447	0.167513	0.790898	1.000
108	GOBP_PEPTIDE_METABOLIC_PROCESS	GO:0006518	11	0.462628	1.273735	0.188768	0.796637	1.000
109	GOBP_LEUKOCYTE_MEDIATED_IMMUNITY	GO:0002443	8	0.505884	1.272230	0.181970	0.794709	1.000
110	GOBP_POSITIVE_REGULATION_OF_CELL_GROWTH	GO:0030307	6	0.575523	1.271877	0.168285	0.788739	1.000
111	GOBP_NEURON_DEATH	GO:0070997	18	0.401328	1.269323	0.177465	0.790942	1.000
112	GOBP_APOPTOTIC_PROCESS	GO:0006915	35	0.350878	1.268243	0.188652	0.787606	1.000
113	GOBP_IMPORT_INTO_CELL	GO:0098657	6	0.543017	1.267392	0.188103	0.783394	1.000
114	GOBP_POSITIVE_REGULATION_OF_GENE_EXPRESSION	GO:0010628	16	0.403644	1.254086	0.195455	0.822239	1.000
115	GOBP_PROTEIN_LOCALIZATION_TO_MEMBRANE	GO:0072657	13	0.433387	1.250538	0.201210	0.827483	1.000
116	GOBP_REGULATION_OF_PEPTIDE_HORMONE_SECRETION	GO:0090276	6	0.551877	1.250516	0.205255	0.820483	1.000
117	GOBP_LEUKOCYTE_MIGRATION	GO:0050900	6	0.548798	1.250033	0.212418	0.815028	1.000
118	GOBP_ENDOCYTOSIS	GO:0006897	8	0.502853	1.245864	0.206785	0.822112	1.000
119	REACTOME_EPH_EPHRIN_SIGNALING	R-RNO-268233	6	0.539735	1.244645	0.217600	0.819516	1.000
120	GOBP_REGULATION_OF_PEPTIDE_SECRETION	GO:0002791	7	0.516817	1.244606	0.214286	0.812935	1.000
121	GOBP_MULTICELLULAR_ORGANISMAL_HOMEOSTASIS	GO:0048871	12	0.441585	1.239758	0.231884	0.822510	1.000
122	GOBP_POSITIVE_REGULATION_OF_IMMUNE_RESPONSE	GO:0050778	9	0.471559	1.238038	0.221865	0.821737	1.000
123	GOBP_REGULATION_OF_HORMONE_LEVELS	GO:0010817	12	0.431565	1.233018	0.191063	0.832008	1.000
124	REACTOME_INNATE_IMMUNE_SYSTEM	R-RNO-168249	16	0.400564	1.232559	0.216301	0.826874	1.000
125	GOBP_CELLULAR_LIPID_METABOLIC_PROCESS	GO:0044255	7	0.519120	1.222910	0.226984	0.853554	1.000
126	GOBP_REGULATION_OF_PROTEOLYSIS	GO:0030162	12	0.425739	1.220094	0.227964	0.856463	1.000

Continuation of Table **S8**

	NAME	ID	SIZE	ES	NES	NOM.p.val	FDR.q.val	FWER.p.val
127	GOBP_NERVOUS_SYSTEM_PROCESS	GO:0050877	38	0.336614	1.218500	0.218182	0.854986	1.000
128	GOBP_POSITIVE_REGULATION_OF_PHOSPHATIDYLINOSIT...	GO:0014068	5	0.568305	1.217272	0.227273	0.852496	1.000
129	REACTOME_SIGNALING_BY_NUCLEAR_RECEPTORS	R-RNO-9006931	8	0.481005	1.216913	0.231013	0.847074	1.000
130	GOBP_INSULIN_SECRETION	GO:0030073	7	0.510769	1.214703	0.223114	0.847977	1.000
131	GOBP_NEUROTROPHIN_TRK_RECEPTOR_SIGNALING_PATHWAY	GO:0048011	6	0.525482	1.214255	0.235489	0.843004	1.000
132	GOBP_REGULATION_OF_ANATOMICAL_STRUCTURE_SIZE	GO:0090066	10	0.462544	1.213797	0.232484	0.838243	1.000
133	GOBP_TEMPERATURE_HOMEOSTASIS	GO:0001659	5	0.555846	1.212463	0.233449	0.836545	1.000
134	GOBP_PROTEIN_LOCALIZATION_TO_PLASMA_MEMBRANE	GO:0072659	7	0.516984	1.211528	0.240196	0.833571	1.000
135	GOBP_REGULATION_OF_CARBOHYDRATE_BIOSYNTHETIC_P...	GO:0043255	5	0.563349	1.211510	0.233058	0.827503	1.000
136	GOBP_POSTSYNAPSE_ORGANIZATION	GO:0099173	10	0.459944	1.207936	0.230769	0.833425	1.000
137	GOBP_BEHAVIOR	GO:0007610	33	0.339927	1.206907	0.218571	0.830557	1.000
138	REACTOME_ESR_MEDIATED_SIGNALING	R-RNO-8939211	8	0.481005	1.206899	0.243200	0.824612	1.000
139	GOBP_POSITIVE_REGULATION_OF_LOCOMOTION	GO:0040017	11	0.434396	1.203674	0.234548	0.829002	1.000
140	GOBP_POSITIVE_REGULATION_OF_DEFENSE_RESPONSE	GO:0031349	5	0.562919	1.203225	0.243478	0.824646	1.000
141	GOBP_PEPTIDE_HORMONE_SECRETION	GO:0030072	7	0.510769	1.202905	0.256536	0.819926	1.000
142	GOBP_CARBOHYDRATE_BIOSYNTHETIC_PROCESS	GO:0016051	5	0.563349	1.202668	0.250853	0.815154	1.000
143	GOBP_REGULATION_OF_CATABOLIC_PROCESS	GO:0009894	13	0.417235	1.201575	0.240061	0.812810	1.000
144	GOBP_REGULATION_OF_CARBOHYDRATE_METABOLIC_PROCESS	GO:0006109	5	0.563349	1.199063	0.250000	0.814924	1.000
145	GOBP_CELLULAR_CARBOHYDRATE_METABOLIC_PROCESS	GO:0044262	5	0.545059	1.196572	0.245409	0.817851	1.000
146	GOBP_REGULATION_OF_CELL_PROJECTION_ORGANIZATION	GO:0031344	20	0.366652	1.194146	0.227273	0.820042	1.000
147	GOBP_REPRODUCTION	GO:0000003	18	0.373587	1.189810	0.243370	0.828380	1.000
148	GOBP_ENZYME_LINKED_RECEPTOR_PROTEIN_SIGNALING_...	GO:0007167	24	0.351248	1.189558	0.248547	0.823564	1.000
149	HALLMARK_PI3K_AKT_MTOR_SIGNALING	M5923	6	0.519843	1.185509	0.257240	0.830398	1.000
150	GOBP_NEUROTROPHIN_SIGNALING_PATHWAY	GO:0038179	6	0.525482	1.183780	0.264559	0.830417	1.000
151	GOBP_NEURON_DIFFERENTIATION	GO:0030182	37	0.322061	1.182687	0.254446	0.828345	1.000
152	GOBP_REGULATION_OF_PROTEIN_SERINE_THREONINE_KI...	GO:0071900	12	0.413839	1.175638	0.253414	0.845425	1.000
153	GOBP_CELL_ACTIVATION	GO:0001775	22	0.354860	1.173056	0.265537	0.847939	1.000
154	GOBP_REGULATION_OF_PEPTIDE_TRANSPORT	GO:0090087	10	0.447898	1.168514	0.274845	0.856496	1.000
155	GOBP_EXOCYTOSIS	GO:0006887	11	0.415843	1.167485	0.268817	0.854262	1.000
156	GOBP_NEUROGENESIS	GO:0022008	41	0.318144	1.165592	0.255072	0.854924	1.000
157	GOBP_REGULATION_OF_HORMONE_SECRETION	GO:0046883	8	0.449184	1.162006	0.283388	0.860624	1.000
158	GOBP_OSSIFICATION	GO:0001503	9	0.441324	1.160569	0.250814	0.859754	1.000
159	GOBP_REGULATION_OF_POSTSYNAPSE_ORGANIZATION	GO:0099175	6	0.503139	1.160194	0.274062	0.855621	1.000
160	GOBP_POLYSACCHARIDE_METABOLIC_PROCESS	GO:0005976	5	0.545024	1.155751	0.294218	0.863447	1.000
161	GOBP_RESPONSE_TO_NUTRIENT	GO:0007584	5	0.542169	1.155461	0.285240	0.858999	1.000
162	GOBP_AMYLOID_PRECURSOR_PROTEIN_CATABOLIC_PROCESS	GO:0042987	5	0.533908	1.152009	0.284192	0.864240	1.000
163	GOBP_CARBOHYDRATE_DERIVATIVE_METABOLIC_PROCESS	GO:1901135	8	0.468011	1.151732	0.296296	0.859863	1.000
164	GOBP_POSITIVE_REGULATION_OF_GLIOGENESIS	GO:0014015	5	0.542169	1.151447	0.298658	0.855512	1.000
165	GOBP_AMYLOID_PRECURSOR_PROTEIN_METABOLIC_PROCESS	GO:0042982	5	0.533908	1.150729	0.270548	0.852576	1.000
166	GOBP_REGULATION_OF_GLIOGENESIS	GO:0014013	5	0.542169	1.150039	0.312715	0.849707	1.000
167	GOBP_REGULATION_OF_IMMUNE_SYSTEM_PROCESS	GO:0002682	21	0.351613	1.146644	0.282318	0.855021	1.000
168	GOBP_POSITIVE_REGULATION_OF_SIGNALING	GO:0023056	34	0.317185	1.146341	0.281337	0.850756	1.000
169	GOBP_POSITIVE_REGULATION_OF_LIPASE_ACTIVITY	GO:0060193	5	0.526189	1.145270	0.316781	0.848899	1.000

Continuation of Table **S8**

	NAME	ID	SIZE	ES	NES	NOM.p.val	FDR.q.val	FWER.p.val
170	REACTOME_EXTRACELLULAR_MATRIX_ORGANIZATION	R-RNO-1474244	5	0.532405	1.144847	0.302521	0.845091	1.000
171	GOBP_REGULATION_OF_RESPONSE_TO_STRESS	GO:0080134	19	0.360851	1.144728	0.291176	0.840556	1.000
172	GOBP_REGULATION_OF_PROTEIN_PHOSPHORYLATION	GO:0001932	25	0.339528	1.143798	0.285916	0.838443	1.000
173	GOBP_AMYLOID_BETA_METABOLIC_PROCESS	GO:0050435	5	0.533908	1.142297	0.336752	0.837789	1.000
174	REACTOME_ESTROGEN_DEPENDENT_NUCLEAR_EVENTS_DOW...	R-RNO-963463	5	0.523405	1.141606	0.305419	0.835188	1.000
175	GOBP_DENDRITE_MORPHOGENESIS	GO:0048813	7	0.470710	1.138868	0.306818	0.838435	1.000
176	REACTOME_SIGNALING_BY_NTRKS	R-RNO-166520	18	0.361961	1.136217	0.301994	0.841481	1.000
177	GOBP_NEGATIVE_REGULATION_OF_PROTEIN_METABOLIC_...	GO:0051248	20	0.350078	1.134564	0.277698	0.841576	1.000
178	GOBP_CELLULAR_RESPONSE_TO_CADMIUM_ION	GO:0071276	5	0.515219	1.121235	0.345455	0.875689	1.000
179	REACTOME_INFECTIOUS_DISEASE	R-HSA-5663205	12	0.393573	1.121057	0.315866	0.871352	1.000
180	GOBP_REGULATION_OF_PHOSPHOLIPASE_C_ACTIVITY	GO:1900274	5	0.526189	1.116917	0.310051	0.878947	1.000
181	GOBP_NEGATIVE_REGULATION_OF_NEURON_APOPTOTIC_P...	GO:0043524	8	0.444330	1.116724	0.333858	0.874710	1.000
182	GOBP_CELL_MIGRATION	GO:0016477	22	0.333901	1.116014	0.303907	0.872159	1.000
183	GOBP_REGULATION_OF_EPITHELIAL_CELL_MIGRATION	GO:0010632	6	0.490485	1.111603	0.344123	0.879744	1.000
184	GOBP_MACROMOLECULE_CATABOLIC_PROCESS	GO:0009057	11	0.402012	1.109869	0.329835	0.879885	1.000
185	GOBP_CELL_CELL_JUNCTION_ORGANIZATION	GO:0045216	6	0.474992	1.108840	0.323680	0.878046	1.000
186	GOBP_POSITIVE_REGULATION_OF_CELLULAR_COMPONENT...	GO:0051130	24	0.328648	1.106696	0.327917	0.879389	1.000
187	GOBP_POSITIVE_REGULATION_OF_HYDROLASE_ACTIVITY	GO:0051345	16	0.364860	1.105460	0.317337	0.878308	1.000
188	GOBP_MONOCARBOXYLIC_ACID_METABOLIC_PROCESS	GO:0032787	6	0.494763	1.103949	0.349206	0.878078	1.000
189	GOBP_PROTEIN_KINASE_B_SIGNALING	GO:0043491	6	0.493972	1.103517	0.345719	0.874603	1.000
190	GOBP_NEGATIVE_REGULATION_OF_IMMUNE_SYSTEM_PROCESS	GO:0002683	5	0.507762	1.098553	0.341924	0.883987	1.000
191	GOBP_REGULATION_OF_PHOSPHORUS_METABOLIC_PROCESS	GO:0051174	26	0.321877	1.096215	0.328909	0.885936	1.000
192	GOBP_MEMBRANE_PROTEIN_PROTEOLYSIS	GO:0033619	6	0.479020	1.095820	0.352554	0.882529	1.000
193	GOBP_NEGATIVE_REGULATION_OF_ION_TRANSPORT	GO:0043271	11	0.403598	1.094968	0.345313	0.880552	1.000
194	GOBP_REGULATION_OF_CELLULAR_PROTEIN_LOCALIZATION	GO:1903827	12	0.376889	1.093240	0.349544	0.880917	1.000
195	GOBP_EPHRIN_RECEPTOR_SIGNALING_PATHWAY	GO:0048013	5	0.510420	1.083570	0.377104	0.903329	1.000
196	GOBP_REGULATION_OF_CELL_DIFFERENTIATION	GO:0045595	36	0.302323	1.083207	0.347280	0.899732	1.000
197	GOBP_POSITIVE_REGULATION_OF_GROWTH	GO:0045927	9	0.418443	1.081892	0.376361	0.898933	1.000
198	GOBP_GENERATION_OF_PRECURSOR_METABOLITES_AND_E...	GO:0006091	5	0.506024	1.080559	0.386288	0.898319	1.000
199	GOBP_HORMONE_TRANSPORT	GO:0009914	9	0.423765	1.080050	0.371069	0.895341	1.000
200	GOBP_BIOLOGICAL_PROCESS_INVOLVED_IN_SYMBIOTIC_...	GO:004440	10	0.404774	1.079636	0.341158	0.892087	1.000
201	GOBP_GLIOGENESIS	GO:0042063	12	0.379652	1.079543	0.368421	0.887909	1.000
202	GOBP_PROTEIN_PHOSPHORYLATION	GO:0006468	33	0.301978	1.077395	0.356742	0.889349	1.000
203	REACTOME_TRANSCRIPTIONAL_REGULATION_OF_WHITE_A...	R-RNO-381340	6	0.461663	1.069953	0.381877	0.905279	1.000
204	GOBP_POSITIVE_REGULATION_OF_ION_TRANSPORT	GO:0043270	17	0.346564	1.069327	0.379971	0.902581	1.000
205	GOBP_RESPONSE_TO_KETONE	GO:1901654	7	0.451195	1.068408	0.400000	0.900624	1.000
206	GOBP_APOPTOTIC_MITOCHONDRIAL_CHANGES	GO:0008637	6	0.473220	1.068247	0.382450	0.896725	1.000
207	GOBP_SECRETION	GO:0046903	24	0.318104	1.068228	0.361357	0.892465	1.000
208	GOBP_REGULATION_OF_TRANSFERASE_ACTIVITY	GO:0051338	21	0.322161	1.067402	0.365889	0.890366	1.000
209	GOBP_POSITIVE_REGULATION_OF_PROTEIN_MODIFICATI...	GO:0031401	22	0.320552	1.066035	0.359882	0.889894	1.000
210	GOBP_REGULATION_OF_PROTEIN_KINASE_ACTIVITY	GO:0045859	21	0.322161	1.064887	0.365926	0.888855	1.000
211	GOBP_REGULATION_OF_LIPASE_ACTIVITY	GO:0060191	6	0.456443	1.064859	0.390203	0.884750	1.000
212	GOBP_RESPONSE_TO_NITROGEN_COMPOUND	GO:1901698	35	0.298471	1.064434	0.380818	0.881945	1.000

Continuation of Table **S8**

	NAME	ID	SIZE	ES	NES	NOM.p.val	FDR.q.val	FWER.p.val
213	REACTOME_TRANSCRIPTIONAL_REGULATION_BY_TP53	R-RNO-3700989	6	0.466900	1.063381	0.382504	0.880746	1.000
214	GOBP_PHOSPHATIDYLINOSITOL_3_KINASE_SIGNALING	GO:0014065	7	0.445684	1.062397	0.396899	0.879243	1.000
215	GOBP_REGULATION_OF_VESICLE_MEDIATED_TRANSPORT	GO:0060627	12	0.376180	1.061960	0.378336	0.876380	1.000
216	GOBP_CYTOSKELETON_ORGANIZATION	GO:0007010	11	0.385993	1.058714	0.411498	0.881072	1.000
217	GOBP_CARBOHYDRATE_METABOLIC_PROCESS	GO:0005975	9	0.409679	1.056483	0.404651	0.883010	1.000
218	GOBP_POSITIVE_REGULATION_OF_PROTEIN_PHOSPHORYL...	GO:0001934	22	0.320552	1.054661	0.388809	0.883679	1.000
219	REACTOME_DEVELOPMENTAL_BIOLOGY	R-RNO-1266738	22	0.317886	1.054310	0.392012	0.880618	1.000
220	GOBP_REGULATION_OF_APOPTOTIC_SIGNALING_PATHWAY	GO:2001233	10	0.382745	1.054163	0.389058	0.877053	1.000
221	GOBP_REGULATION_OF_PROTEIN_CATABOLIC_PROCESS	GO:0042176	7	0.435516	1.053069	0.403846	0.876063	1.000
222	GOBP_CELLULAR_RESPONSE_TO_REACTIVE_OXYGEN_SPECIES	GO:0034614	7	0.431659	1.052146	0.397351	0.874440	1.000
223	GOBP_POSITIVE_REGULATION_OF_PROTEOLYSIS	GO:0045862	8	0.418493	1.050079	0.413183	0.875796	1.000
224	GOBP_ANATOMICAL_STRUCTURE_HOMEOSTASIS	GO:0060249	7	0.438851	1.049167	0.399676	0.874237	1.000
225	GOBP_LOCOMOTION	GO:0040011	31	0.298223	1.048894	0.397759	0.871042	1.000
226	GOBP_REGULATION_OF_PHOSPHOLIPASE_ACTIVITY	GO:0010517	6	0.456443	1.048768	0.403333	0.867570	1.000
227	GOBP_MULTI_ORGANISM_PROCESS	GO:0051704	16	0.346697	1.048652	0.400000	0.864015	1.000
228	GOBP_CELLULAR_RESPONSE_TO_CHEMICAL_STRESS	GO:0062197	7	0.431659	1.048611	0.410774	0.860330	1.000
229	GOBP_REGULATION_OF_MYELOID_CELL_DIFFERENTIATION	GO:0045637	9	0.403946	1.044423	0.401626	0.867293	1.000
230	GOBP_RESPONSE_TO_BACTERIUM	GO:0009617	13	0.358196	1.033900	0.424427	0.890035	1.000
231	GOBP_REGULATION_OF_EXTRINSIC_APOPTOTIC_SIGNALI...	GO:2001236	6	0.456893	1.031639	0.430213	0.891901	1.000
232	GOBP_TRANSMEMBRANE_RECEPTOR_PROTEIN_TYROSINE_K...	GO:0007169	19	0.329555	1.028616	0.410795	0.895648	1.000
233	GOBP_PATTERN_SPECIFICATION_PROCESS	GO:0007389	6	0.455501	1.028594	0.448445	0.891878	1.000
234	GOBP_PEPTIDE_SECRETION	GO:0002790	9	0.399662	1.026116	0.429245	0.893993	1.000
235	GOBP_POSITIVE_REGULATION_OF_RESPONSE_TO_EXTERN...	GO:0032103	9	0.400569	1.025470	0.436066	0.891625	1.000
236	GOBP_POSITIVE_REGULATION_OF_PROTEIN_SERINE_THR...	GO:0071902	9	0.400686	1.024748	0.429936	0.889623	1.000
237	GOBP_TISSUE_HOMEOSTASIS	GO:0001894	5	0.476300	1.021003	0.440735	0.895468	1.000
238	GOBP_REGULATION_OF_RESPONSE_TO_EXTERNAL_STIMULUS	GO:0032101	15	0.338700	1.018327	0.440184	0.898385	1.000
239	GOBP_PROTEIN_TETRAMERIZATION	GO:0051262	5	0.471601	1.017458	0.447712	0.896749	1.000
240	GOBP_PEPTIDYL_SERINE_MODIFICATION	GO:0018209	12	0.358200	1.017098	0.436275	0.893858	1.000
241	GOBP_RESPONSE_TO_ABIOTIC_STIMULUS	GO:0009628	27	0.290781	1.015575	0.452113	0.893915	1.000
242	GOBP_REGIONALIZATION	GO:0003002	6	0.455501	1.012335	0.436306	0.898193	1.000
243	GOBP_POSITIVE_REGULATION_OF_PHOSPHORUS_METABOL...	GO:0010562	23	0.305238	1.012302	0.446715	0.894581	1.000
244	GOBP_REGULATION_OF_VASCULAR_ASSOCIATED_SMOOTH_...	GO:1904705	5	0.473268	1.011766	0.474295	0.892266	1.000
245	GOBP_MYELOID_CELL_DIFFERENTIATION	GO:0030099	13	0.355072	1.010661	0.436137	0.891178	1.000
246	GOBP_REGULATION_OF_ORGANELLE_ORGANIZATION	GO:0033043	14	0.335259	1.010028	0.450663	0.889081	1.000
247	GOBP_RESPONSE_TO_MOLECULE_OF_BACTERIAL_ORIGIN	GO:0002237	13	0.358196	1.008369	0.447368	0.889595	1.000
248	GOBP_PROTEIN_LOCALIZATION_TO_CELL_PERIPHERY	GO:1990778	12	0.359051	1.007281	0.469630	0.888670	1.000
249	REACTOME_CIRCADIAN_CLOCK	R-HSA-400253	5	0.475206	1.006488	0.479542	0.887025	1.000
250	GOBP_PROTEIN_CATABOLIC_PROCESS	GO:0030163	8	0.402744	1.004216	0.460925	0.888738	1.000
251	GOBP_POSITIVE_REGULATION_OF_SYNAPTIC_TRANSMISSION	GO:0050806	15	0.328667	0.998818	0.451219	0.897765	1.000
252	GOBP_POSITIVE_REGULATION_OF_MYELOID_LEUKOCYTE_...	GO:0002763	6	0.440364	0.997449	0.475712	0.897611	1.000
253	GOBP_CELLULAR_COMPONENT_MORPHOGENESIS	GO:0032989	25	0.288621	0.996591	0.459538	0.896152	1.000
254	GOBP_NEGATIVE_REGULATION_OF_INTRACELLULAR_SIGN...	GO:1902532	7	0.422870	0.991085	0.483037	0.905509	1.000
255	GOBP_GLAND_DEVELOPMENT	GO:0048732	7	0.414551	0.988624	0.472222	0.907925	1.000

Continuation of Table **S8**

	NAME	ID	SIZE	ES	NES	NOM.p.val	FDR.q.val	FWER.p.val
256	GOBP_REGULATION_OF_CELLULAR_LOCALIZATION	GO:0060341	17	0.316803	0.988251	0.457778	0.905324	1.000
257	GOBP_CELL_PART_MORPHOGENESIS	GO:0032990	25	0.288621	0.986560	0.480905	0.905831	1.000
258	GOBP_RESPONSE_TO_MECHANICAL_STIMULUS	GO:0009612	6	0.432266	0.985964	0.472178	0.903748	1.000
259	GOBP_APOPTOTIC_SIGNALING_PATHWAY	GO:0097190	15	0.325573	0.984426	0.491499	0.904095	1.000
260	GOBP_RESPONSE_TO_GROWTH_FACTOR	GO:0070848	18	0.312488	0.984261	0.476401	0.901109	1.000
261	GOBP_REGULATION_OF_ANION_TRANSPORT	GO:0044070	20	0.301751	0.983709	0.486291	0.898826	1.000
262	GOBP_REGULATION_OF_CELL_CYCLE_PROCESS	GO:0010564	5	0.461303	0.980566	0.507563	0.902841	1.000
263	GOBP_PROTEIN_DEPHOSPHORYLATION	GO:0006470	7	0.412974	0.979473	0.485981	0.901979	1.000
264	GOBP_DENDRITIC_SPINE_MORPHOGENESIS	GO:0060997	5	0.468992	0.976442	0.517065	0.905765	1.000
265	GOBP_NEGATIVE_REGULATION_OF_SIGNALING	GO:0023057	21	0.303422	0.975465	0.494186	0.904661	1.000
266	GOBP_POSITIVE_REGULATION_OF_LONG_TERM_SYNAPTIC...	GO:1900273	5	0.457866	0.975078	0.497400	0.902203	1.000
267	GOBP_CELL_MORPHOGENESIS	GO:0000902	25	0.288621	0.974976	0.503526	0.899055	1.000
268	GOBP_SMALL_MOLECULE_METABOLIC_PROCESS	GO:0044281	16	0.313622	0.973041	0.495495	0.900077	1.000
269	GOBP_MYELOID_LEUKOCYTE_DIFFERENTIATION	GO:0002573	11	0.346629	0.971778	0.507200	0.899526	1.000
270	GOBP_DEPHOSPHORYLATION	GO:0016311	7	0.412974	0.966836	0.521382	0.907452	1.000
271	GOBP_REGULATION_OF_NERVOUS_SYSTEM_DEVELOPMENT	GO:0051960	15	0.317929	0.966623	0.500000	0.904624	1.000
272	GOBP_REGULATION_OF_WNT_SIGNALING_PATHWAY	GO:0030111	5	0.443648	0.964708	0.510101	0.905655	1.000
273	GOBP_FAT_CELL_DIFFERENTIATION	GO:0045444	9	0.372610	0.964204	0.511774	0.903354	1.000
274	GOBP_MEMBRANE_ORGANIZATION	GO:0061024	12	0.329276	0.961667	0.521875	0.905806	1.000
275	GOBP_CARBOHYDRATE_HOMEOSTASIS	GO:0033500	7	0.397142	0.959953	0.491311	0.906341	1.000
276	GOBP_CELL_POPULATION_PROLIFERATION	GO:0008283	32	0.266205	0.959076	0.506456	0.904994	1.000
277	GOBP_RESPONSE_TO_ENDOGENOUS_STIMULUS	GO:0009719	34	0.263566	0.958047	0.513699	0.903952	1.000
278	GOBP_CELL_ACTIVATION_INVOLVED_IN_IMMUNE_RESPONSE	GO:0002263	6	0.414807	0.953358	0.520525	0.911605	1.000
279	GOBP_ESTABLISHMENT_OF_PROTEIN_LOCALIZATION_TO_...	GO:0072594	5	0.443663	0.951015	0.545455	0.913773	1.000
280	GOBP_RESPIRATORY_SYSTEM_DEVELOPMENT	GO:0060541	6	0.420268	0.950815	0.524181	0.910889	1.000
281	GOBP_SKELETAL_MUSCLE_ORGAN_DEVELOPMENT	GO:0060538	5	0.443771	0.950749	0.559567	0.907798	1.000
282	GOBP_DENDRITE_DEVELOPMENT	GO:0016358	10	0.359931	0.950358	0.545171	0.905315	1.000
283	GOBP_NEURON_PROJECTION_ORGANIZATION	GO:0106027	8	0.381665	0.950020	0.536667	0.902844	1.000
284	GOBP_RESPONSE_TO_OXIDATIVE_STRESS	GO:0006979	8	0.369099	0.949218	0.524721	0.901545	1.000
285	GOBP_REGULATION_OF_PROTEIN_MODIFICATION_PROCESS	GO:0031399	28	0.275739	0.948441	0.517493	0.900070	1.000
286	GOBP_POSITIVE_REGULATION_OF_PROTEIN_KINASE_ACT...	GO:0045860	18	0.299996	0.947939	0.509119	0.897908	1.000
287	GOBP_RESPONSE_TO_TUMOR_NECROSIS_FACTOR	GO:0034612	7	0.390819	0.947186	0.536050	0.896383	1.000
288	GOBP_GLIAL_CELL_DIFFERENTIATION	GO:0010001	10	0.348164	0.942545	0.534056	0.903336	1.000
289	REACTOME_NGF_STIMULATED_TRANSCRIPTION	R-RNO-9031628	9	0.353930	0.942122	0.521880	0.901241	1.000
290	GOBP_REGULATION_OF_CELLULAR_RESPONSE_TO_STRESS	GO:0080135	9	0.359889	0.941958	0.545455	0.898481	1.000
291	GOBP_MYELOID_LEUKOCYTE_MEDIATED_IMMUNITY	GO:0002444	6	0.414807	0.941251	0.546798	0.896902	1.000
292	GOBP_POSITIVE_REGULATION_OF_DEVELOPMENTAL_GROWTH	GO:0048639	8	0.381139	0.939303	0.546178	0.898086	1.000
293	GOBP_RESPONSE_TO_CALCIUM_ION	GO:0051592	6	0.412950	0.937279	0.543253	0.899311	1.000
294	GOBP_DEVELOPMENTAL_CELL_GROWTH	GO:0048588	5	0.430309	0.935343	0.539823	0.900355	1.000
295	GOBP_MALE_GAMETE_GENERATION	GO:0048232	5	0.428139	0.933271	0.554593	0.901903	1.000
296	GOBP_RESPONSE_TO_ALCOHOL	GO:0097305	11	0.342062	0.931378	0.552335	0.902965	1.000
297	GOBP_AMIDE_BIOSYNTHETIC_PROCESS	GO:0043604	6	0.407264	0.927346	0.550580	0.908628	1.000
298	REACTOME_NEUTROPHIL_DEGRANULATION	R-RNO-6798695	6	0.414807	0.926485	0.585324	0.907362	1.000

Continuation of Table **S8**

	NAME	ID	SIZE	ES	NES	NOM.p.val	FDR.q.val	FWER.p.val
299	GOBP_PROTEIN_LOCALIZATION_TO_CELL_JUNCTION	GO:1902414	6	0.397833	0.921168	0.575563	0.915602	1.000
300	GOBP_GAMETE_GENERATION	GO:0007276	9	0.355826	0.920672	0.576983	0.913618	1.000
301	GOBP_POSITIVE_REGULATION_OF_MOLECULAR_FUNCTION	GO:0044093	37	0.253866	0.919332	0.568966	0.913434	1.000
302	GOBP_RESPONSE_TO_REACTIVE_OXYGEN_SPECIES	GO:0000302	8	0.369099	0.918985	0.571429	0.911168	1.000
303	GOBP_POSITIVE_REGULATION_OF_DNA_BINDING_TRANSC...	GO:0051091	10	0.344294	0.918932	0.573718	0.908280	1.000
304	GOBP_SMALL_MOLECULE_BIOSYNTHETIC_PROCESS	GO:0044283	5	0.431932	0.918490	0.591837	0.906158	1.000
305	GOBP_NEURON_APOPTOTIC_PROCESS	GO:0051402	12	0.321733	0.908973	0.583882	0.923634	1.000
306	GOBP_PEPTIDE_BIOSYNTHETIC_PROCESS	GO:0043043	6	0.407264	0.906961	0.594077	0.924892	1.000
307	GOBP_REGULATION_OF_ION_TRANSPORT	GO:0043269	36	0.248988	0.906657	0.583902	0.922581	1.000
308	GOBP_ESTABLISHMENT_OF_PROTEIN_LOCALIZATION	GO:0045184	17	0.290831	0.904299	0.599402	0.924406	1.000
309	GOBP_REGULATION_OF_DEVELOPMENTAL_GROWTH	GO:0048638	9	0.352029	0.904064	0.571429	0.921993	1.000
310	GOBP_AMEBOIDAL_TYPE_CELL_MIGRATION	GO:0001667	11	0.326439	0.903821	0.590980	0.919470	1.000
311	GOBP_REGULATION_OF_HEMOPOIESIS	GO:1903706	10	0.336200	0.901923	0.594427	0.920550	1.000
312	GOBP_LEUKOCYTE_CELL_CELL_ADHESION	GO:0007159	6	0.398548	0.899472	0.585209	0.922418	1.000
313	GOBP_MONOAMINE_TRANSPORT	GO:0015844	5	0.422960	0.898652	0.612211	0.921107	1.000
314	GOBP_DENDRITIC_SPINE_DEVELOPMENT	GO:0060996	6	0.397434	0.895675	0.609677	0.924132	1.000
315	GOBP_NEGATIVE_REGULATION_OF_ANION_TRANSPORT	GO:1903792	8	0.355244	0.891762	0.602761	0.929284	1.000
316	REACTOME_ADAPTIVE_IMMUNE_SYSTEM	R-RNO-1280218	9	0.348963	0.890813	0.594340	0.928382	1.000
317	GOBP_RESPONSE_TO_BIOTIC_STIMULUS	GO:0009607	17	0.288691	0.890720	0.604106	0.925672	1.000
318	GOBP_POSITIVE_REGULATION_OF_TRANSFERASE_ACTIVITY	GO:0051347	19	0.279005	0.880034	0.626292	0.944098	1.000
319	REACTOME_MAPK_TARGETS_NUCLEAR_EVENTS_MEDIATED_...	R-HSA-450282	5	0.407757	0.877732	0.639594	0.945867	1.000
320	GOBP_POSITIVE_REGULATION_OF_ORGANELLE_ORGANIZA...	GO:0010638	10	0.331060	0.877701	0.613354	0.942987	1.000
321	REACTOME_SIGNALING_BY_NTRK2_TRKB	R-RNO-9006115	6	0.385972	0.877293	0.610282	0.940881	1.000
322	GOBP_ORGANONITROGEN_COMPOUND_CATABOLIC_PROCESS	GO:1901565	10	0.327478	0.876730	0.617886	0.939075	1.000
323	GOBP_CELL_CELL_JUNCTION_ASSEMBLY	GO:0007043	5	0.407365	0.875615	0.613445	0.938195	1.000
324	GOBP_RESPONSE_TO_PEPTIDE_HORMONE	GO:0043434	13	0.304436	0.873939	0.637747	0.938616	1.000
325	GOBP_POSITIVE_REGULATION_OF_NEURON_PROJECTION_...	GO:0010976	5	0.417717	0.873912	0.646667	0.935776	1.000
326	GOBP_REGULATION_OF_MITOTIC_CELL_CYCLE	GO:0007346	5	0.405823	0.872542	0.635897	0.935650	1.000
327	GOBP_DEVELOPMENTAL_GROWTH	GO:0048589	10	0.330048	0.872371	0.621875	0.933110	1.000
328	GOBP_REGULATION_OF_MYELOID_LEUKOCYTE_DIFFERENT...	GO:0002761	8	0.349035	0.871666	0.610224	0.931570	1.000
329	GOBP_REGULATION_OF_CELLULAR_AMIDE_METABOLIC_PR...	GO:0034248	9	0.334759	0.870242	0.630915	0.931515	1.000
330	GOBP_CARBOHYDRATE_DERIVATIVE_BIOSYNTHETIC_PROCESS	GO:1901137	5	0.410411	0.865397	0.648557	0.938454	1.000
331	GOBP_REGULATION_OF_TRANSMEMBRANE_TRANSPORT	GO:0034762	23	0.259338	0.864858	0.630952	0.936731	1.000
332	GOBP_REGULATION_OF_INTRACELLULAR_SIGNAL_TRANSD...	GO:1902531	21	0.265874	0.864661	0.631420	0.934288	1.000
333	GOBP_MORPHOGENESIS_OF_AN_EPITHELIUM	GO:0002009	6	0.382120	0.863905	0.659933	0.932876	1.000
334	GOBP_MITOCHONDRION_ORGANIZATION	GO:0007005	7	0.363920	0.862446	0.661994	0.932936	1.000
335	GOBP_NEGATIVE_REGULATION_OF_RESPONSE_TO_EXTERN...	GO:0032102	5	0.397421	0.862220	0.629948	0.930630	1.000
336	GOBP_MUSCLE_CELL_PROLIFERATION	GO:0033002	8	0.344817	0.862010	0.623779	0.928261	1.000
337	GOBP_REGULATION_OF_ANION_TRANSMEMBRANE_TRANSPORT	GO:1903959	5	0.404242	0.858347	0.649919	0.932419	1.000
338	HALLMARK_KRAS_SIGNALING_UP	M5953	6	0.375118	0.857722	0.628617	0.930820	1.000
339	GOBP_DEFENSE_RESPONSE_TO_OTHER_ORGANISM	GO:0098542	9	0.333096	0.857665	0.653907	0.928176	1.000
340	GOBP_POSITIVE_REGULATION_OF_NEURON_DEATH	GO:1901216	5	0.398065	0.856996	0.654762	0.926676	1.000
341	GOBP_POSITIVE_REGULATION_OF_LEUKOCYTE_CELL_CEL...	GO:1903039	5	0.394961	0.855219	0.681818	0.927250	1.000

Continuation of Table **S8**

	NAME	ID	SIZE	ES	NES	NOM.p.val	FDR.q.val	FWER.p.val
342	GOBP_POSITIVE_REGULATION_OF_SMALL_MOLECULE_MET...	GO:0062013	5	0.394061	0.851529	0.674576	0.931659	1.000
343	HALLMARK_APOPTOSIS	M5902	6	0.371032	0.850826	0.649241	0.930326	1.000
344	GOBP_CELL_GROWTH	GO:0016049	10	0.315553	0.848633	0.653784	0.931572	1.000
345	GOBP_POSITIVE_REGULATION_OF_CELL_CELL_ADHESION	GO:0022409	5	0.394961	0.845794	0.659933	0.934217	1.000
346	REACTOME_DISEASES_OF_SIGNAL_TRANSDUCTION_BY_GR...	R-HSA-5663202	12	0.298357	0.844743	0.668760	0.933562	1.000
347	GOBP_POSITIVE_REGULATION_OF_SECRETION	GO:0051047	6	0.379653	0.844214	0.671642	0.931761	1.000
348	GOBP_CYTOKINE_PRODUCTION	GO:0001816	10	0.319721	0.843242	0.670330	0.931033	1.000
349	GOBP_LEUKOCYTE_DIFFERENTIATION	GO:0002521	15	0.280259	0.842037	0.679412	0.930546	1.000
350	GOBP_POSITIVE_REGULATION_OF_EPITHELIAL_CELL_MI...	GO:0010634	5	0.399720	0.841966	0.676768	0.928000	1.000
351	GOBP_CELLULAR_RESPONSE_TO_HORMONE_STIMULUS	GO:0032870	11	0.301490	0.841537	0.663158	0.926197	1.000
352	GOBP_REGULATION_OF_LIPID_LOCALIZATION	GO:1905952	5	0.386854	0.837652	0.659649	0.930629	1.000
353	GOBP_POSITIVE_REGULATION_OF_TRANSMEMBRANE_TRAN...	GO:0034764	7	0.350120	0.837253	0.659677	0.928694	1.000
354	REACTOME_DEATH_RECEPTOR_SIGNALLING	R-RNO-73887	5	0.394931	0.835480	0.683849	0.929264	1.000
355	GOBP_CIRCULATORY_SYSTEM_PROCESS	GO:0003013	5	0.393160	0.831678	0.708543	0.933569	1.000
356	GOBP_LOCOMOTORY_BEHAVIOR	GO:0007626	8	0.328581	0.831548	0.672727	0.931205	1.000
357	REACTOME_ANTI_INFLAMMATORY_RESPONSE_FAVOURING_...	R-HSA-9662851	5	0.394997	0.830515	0.699357	0.930542	1.000
358	GOBP_NEGATIVE_REGULATION_OF_CELL_POPULATION_PR...	GO:0008285	9	0.322386	0.828292	0.697248	0.931860	1.000
359	GOBP_NEGATIVE_REGULATION_OF_PHOSPHORUS_METABOL...	GO:0010563	9	0.319854	0.828122	0.675241	0.929548	1.000
360	GOBP_REGULATION_OF_TRANS_SYNAPTIC_SIGNALING	GO:0099177	40	0.230950	0.826860	0.705882	0.929204	1.000
361	REACTOME_INTERLEUKIN_17_SIGNALING	R-RNO-448424	6	0.366655	0.824684	0.704620	0.930432	1.000
362	GOBP_BIOLOGICAL_ADHESION	GO:0022610	19	0.259896	0.823198	0.696581	0.930703	1.000
363	GOBP_REGULATION_OF_SYNAPTIC_TRANSMISSION_GLUTA...	GO:0051966	12	0.288064	0.821896	0.688207	0.930397	1.000
364	GOBP_POSITIVE_REGULATION_OF_CELL_PROJECTION_OR...	GO:0031346	10	0.307494	0.821359	0.690513	0.928841	1.000
365	GOBP_RESPONSE_TO_EXTRACELLULAR_STIMULUS	GO:0009991	13	0.284228	0.820526	0.705279	0.927756	1.000
366	GOBP_POSITIVE_REGULATION_OF_HEMOPOIESIS	GO:1903708	7	0.339236	0.819816	0.676033	0.926405	1.000
367	GOBP_CELLULAR_RESPONSE_TO_KETONE	GO:1901655	5	0.385603	0.818484	0.713299	0.926331	1.000
368	GOBP_RESPONSE_TO_DRUG	GO:0042493	11	0.299335	0.816898	0.685215	0.926489	1.000
369	GOBP_NEGATIVE_REGULATION_OF_NERVOUS_SYSTEM_DEV...	GO:0051961	5	0.381633	0.815470	0.734219	0.926507	1.000
370	GOBP_SYNAPSE_ORGANIZATION	GO:0050808	17	0.258545	0.813414	0.719530	0.927444	1.000
371	GOBP_CELLULAR_MACROMOLECULE_LOCALIZATION	GO:0070727	20	0.247342	0.808786	0.720117	0.933050	1.000
372	GOBP_NEGATIVE_REGULATION_OF_CELLULAR_COMPONENT...	GO:0051129	11	0.289179	0.807754	0.712934	0.932376	1.000
373	GOBP_ORGANIC_HYDROXY_COMPOUND_TRANSPORT	GO:0015850	6	0.354964	0.807369	0.701131	0.930581	1.000
374	GOBP_FOREBRAIN_DEVELOPMENT	GO:0030900	10	0.303420	0.806909	0.719937	0.928831	1.000
375	GOBP_SYNAPTIC_TRANSMISSION_GLUTAMATERGIC	GO:0035249	13	0.281601	0.806858	0.706314	0.926470	1.000
376	GOBP_ORGANIC_HYDROXY_COMPOUND_METABOLIC_PROCESS	GO:1901615	8	0.312500	0.803844	0.709729	0.929198	1.000
377	GOBP_REGULATION_OF_SYNAPSE_STRUCTURE_OR_ACTIVITY	GO:0050803	11	0.288568	0.800278	0.708006	0.932485	1.000
378	GOBP_NEGATIVE_REGULATION_OF_PROTEIN_MODIFICATI...	GO:0031400	10	0.300574	0.799051	0.732628	0.931987	1.000
379	GOBP_NEGATIVE_REGULATION_OF_BIOSYNTHETIC_PROCESS	GO:0009890	13	0.275581	0.798030	0.736999	0.931280	1.000
380	GOBP_POSITIVE_REGULATION_OF_CELLULAR_COMPONENT...	GO:0044089	9	0.304809	0.796528	0.725522	0.931269	1.000
381	GOBP_CATION_TRANSPORT	GO:0006812	25	0.232578	0.796186	0.755304	0.929377	1.000
382	GOBP_REGULATION_OF_MULTICELLULAR_ORGANISMAL_DE...	GO:2000026	31	0.225423	0.796149	0.760962	0.927011	1.000
383	GOBP_MUSCLE_STRUCTURE_DEVELOPMENT	GO:0061061	16	0.261437	0.795901	0.729771	0.924943	1.000
384	GOBP_REGULATION_OF_ENDOTHELIAL_CELL_MIGRATION	GO:0010594	5	0.371565	0.793322	0.718910	0.926977	1.000

Continuation of Table **S8**

	NAME	ID	SIZE	ES	NES	NOM.p.val	FDR.q.val	FWER.p.val
385	GOBP_T_CELL_ACTIVATION	GO:0042110	8	0.311792	0.792435	0.714968	0.926005	1.000
386	GOBP_POSITIVE_REGULATION_OF_ESTABLISHMENT_OF_P...	GO:1904951	5	0.369980	0.792234	0.747856	0.923958	1.000
387	GOBP_LEARNING	GO:0007612	13	0.271170	0.792066	0.741486	0.921820	1.000
388	GOBP_CELLULAR_RESPONSE_TO_PEPTIDE_HORMONE_STIM...	GO:0071375	9	0.301261	0.790186	0.737785	0.922297	1.000
389	GOBP_NEGATIVE_REGULATION_OF_CELL_DEVELOPMENT	GO:0010721	6	0.349192	0.789736	0.739683	0.920643	1.000
390	GOBP_REGULATION_OF_GROWTH	GO:0040008	13	0.274262	0.789714	0.749221	0.918334	1.000
391	REACTOME_TOLL_LIKE_RECEPTOR_TLR1_TLR2_CASCADE	R-RNO-168179	8	0.310203	0.788535	0.754072	0.917797	1.000
392	GOBP_SEXUAL_REPRODUCTION	GO:0019953	10	0.300119	0.788021	0.755267	0.916332	1.000
393	GOBP_IN_UTERO_EMBRYONIC_DEVELOPMENT	GO:0001701	8	0.318600	0.786135	0.739550	0.917103	1.000
394	REACTOME_MYD88_INDEPENDENT_TLR4_CASCADE	R-RNO-166166	8	0.310203	0.786064	0.728896	0.914881	1.000
395	REACTOME_TOLL_LIKE_RECEPTOR_CASCADES	R-RNO-168898	8	0.310203	0.785346	0.735910	0.913672	1.000
396	REACTOME_TOLL_LIKE_RECEPTOR_9_TLR9_CASCADE	R-RNO-168138	8	0.310203	0.785136	0.736066	0.911697	1.000
397	GOBP_IMMUNE_SYSTEM_DEVELOPMENT	GO:0002520	17	0.250683	0.783089	0.770865	0.912517	1.000
398	GOBP_MUSCLE_ORGAN_DEVELOPMENT	GO:0007517	9	0.304404	0.782533	0.751926	0.911141	1.000
399	GOBP_CELLULAR_RESPONSE_TO_NITROGEN_COMPOUND	GO:1901699	20	0.242638	0.780204	0.762391	0.912438	1.000
400	GOBP_POSITIVE_REGULATION_OF_MAP_KINASE_ACTIVITY	GO:0043406	6	0.343175	0.778795	0.732323	0.912304	1.000
401	GOBP_NEGATIVE_REGULATION_OF_PROTEIN_CATABOLIC_...	GO:0042177	5	0.359196	0.778208	0.744538	0.910943	1.000
402	GOBP_ACTIVATION_OF_MAPK_ACTIVITY	GO:0000187	6	0.343175	0.777692	0.743265	0.909440	1.000
403	GOBP_CELL_CELL_ADHESION	GO:0098609	14	0.267576	0.777395	0.774390	0.907578	1.000
404	GOBP_POSITIVE_REGULATION_OF_PEPTIDASE_ACTIVITY	GO:0010952	7	0.317311	0.773866	0.734177	0.910555	1.000
405	REACTOME_NUCLEAR_EVENTS_KINASE_AND_TRANSCRIPTI...	R-RNO-198725	11	0.277204	0.771880	0.732198	0.911372	1.000
406	GOBP_ENDOCRINE_SYSTEM_DEVELOPMENT	GO:0035270	6	0.340687	0.770364	0.757009	0.911445	1.000
407	GGOBP_REGULATION_OF_PHOSPHATIDYLINOSITOL_3_KINA...	GO:0014066	6	0.335304	0.768954	0.750422	0.911330	1.000
408	GOBP_GROWTH	GO:0040007	14	0.258839	0.768222	0.751152	0.910227	1.000
409	GOBP_POSITIVE_REGULATION_OF_CYSTEINE_TYPE_ENDO...	GO:2001056	7	0.317311	0.762180	0.772655	0.916924	1.000
410	GOBP_RESPONSE_TO_LIPID	GO:0033993	19	0.233521	0.760477	0.787106	0.917257	1.000
411	GOBP_NEGATIVE_REGULATION_OF_RESPONSE_TO_STIMULUS	GO:0048585	16	0.247957	0.759614	0.765060	0.916298	1.000
412	GOBP_RESPONSE_TO_ALKALOID	GO:0043279	5	0.355800	0.759044	0.792208	0.914930	1.000
413	GOBP_CELLULAR_RESPONSE_TO_EXTERNAL_STIMULUS	GO:0071496	10	0.281551	0.756410	0.774351	0.916543	1.000
414	GOBP_NERVE_DEVELOPMENT	GO:0021675	9	0.292096	0.755192	0.788906	0.916172	1.000
415	GOBP_ORGANIC_ACID_METABOLIC_PROCESS	GO:0006082	7	0.312070	0.746952	0.780096	0.925727	1.000
416	GOBP_RESPONSE_TO_PEPTIDE	GO:1901652	18	0.231424	0.737830	0.818318	0.936087	1.000
417	GOBP_IMMUNE_RESPONSE_REGULATING_SIGNALING_PATHWAY	GO:000276	8	0.290419	0.735254	0.786859	0.937360	1.000
418	GOBP_POSITIVE_REGULATION_OF_AXONOGENESIS	GO:0050772	5	0.339464	0.732625	0.791946	0.938592	1.000
419	GOBP_RESPONSE_TO_OXYGEN_LEVELS	GO:0070482	8	0.293408	0.731985	0.801887	0.937252	1.000
420	GOBP_NEGATIVE_REGULATION_OF_NUCLEOBASE_CONTAIN...	GO:0045934	13	0.247796	0.730243	0.821981	0.937444	1.000
421	GOBP_LYMPHOCYTE_ACTIVATION	GO:0046649	10	0.267213	0.727710	0.815748	0.938572	1.000
422	GOBP_MAINTENANCE_OF_LOCATION	GO:0051235	7	0.302305	0.726826	0.798658	0.937597	1.000
423	GOBP_REGULATION_OF_VASCULATURE_DEVELOPMENT	GO:1901342	5	0.344698	0.726623	0.816225	0.935637	1.000
424	GOBP_REGULATION_OF_ANATOMICAL_STRUCTURE_MORPHO...	GO:0022603	17	0.233126	0.725730	0.817379	0.934705	1.000
425	GOBP_POSITIVE_REGULATION_OF_MYELOID_CELL_DIFFE...	GO:0045639	7	0.307659	0.722009	0.833608	0.937383	1.000
426	GOBP_REGULATION_OF_DNA_BINDING_TRANSCRIPTION_F...	GO:0051090	16	0.236485	0.721933	0.847534	0.935304	1.000
427	GOBP_HOMEOSTASIS_OF_NUMBER_OF_CELLS	GO:0048872	5	0.345987	0.721138	0.821429	0.934159	1.000

Continuation of Table **S8**

	NAME	ID	SIZE	ES	NES	NOM.p.val	FDR.q.val	FWER.p.val
428	GOBP_REGULATION_OF_NEUROGENESIS	GO:0050767	14	0.242998	0.716996	0.816923	0.937268	1.000
429	GOBP_FC_RECEPTOR_SIGNALING_PATHWAY	GO:0038093	8	0.290419	0.715695	0.822531	0.936764	1.000
430	GOBP_ANION_TRANSMEMBRANE_TRANSPORT	GO:0098656	7	0.289420	0.713922	0.851097	0.936807	1.000
431	GOBP_CELL_CELL_SIGNALING_BY_WNT	GO:0198738	7	0.296782	0.713176	0.846278	0.935548	1.000
432	REACTOME_NERVOUS_SYSTEM_DEVELOPMENT	R-RNO-9675108	14	0.242413	0.711681	0.850075	0.935417	1.000
433	GOBP_POSITIVE_REGULATION_OF_MULTICELLULAR_ORGA...	GO:0051240	32	0.201189	0.710331	0.863448	0.934995	1.000
434	GOBP_CELL_JUNCTION_ASSEMBLY	GO:0034329	8	0.279590	0.710168	0.831818	0.933059	1.000
435	GOBP_MATERNAL_PROCESS_INVOLVED_IN_FEMALE_PREGN...	GO:0060135	5	0.335518	0.709035	0.829431	0.932337	1.000
436	GOBP_TRANSMEMBRANE_RECEPTOR_PROTEIN_SERINE_THR...	GO:0007178	6	0.315859	0.702517	0.836120	0.938281	1.000
437	GOBP_RESPONSE_TO_ETHANOL	GO:0045471	5	0.323430	0.700264	0.856900	0.938924	1.000
438	GOBP_REGULATION_OF_CYTOSKELETON_ORGANIZATION	GO:0051493	5	0.329569	0.699766	0.831918	0.937445	1.000
439	GOBP_VESICLE_MEDIATED_TRANSPORT_IN_SYNAPSE	GO:0099003	7	0.294535	0.694658	0.829431	0.941487	1.000
440	GOBP_POSITIVE_REGULATION_OF_CATION_TRANSMEMBRA...	GO:1904064	5	0.320648	0.691599	0.873754	0.942878	1.000
441	GOBP_REGULATION_OF_CELL_DEVELOPMENT	GO:0060284	15	0.229648	0.690604	0.857143	0.941939	1.000
442	GOBP_CELLULAR_RESPONSE_TO_ORGANIC_CYCLIC_COMPOUND	GO:0071407	13	0.234275	0.683445	0.855639	0.947886	1.000
443	GOBP_METAL_ION_HOMEOSTASIS	GO:0055065	14	0.226595	0.680277	0.875576	0.949360	1.000
444	GOBP_EMBRYONIC_ORGAN_DEVELOPMENT	GO:0048568	7	0.283612	0.679925	0.853081	0.947612	1.000
445	GOBP_INNATE_IMMUNE_RESPONSE	GO:0045087	7	0.283951	0.678606	0.859016	0.946985	1.000
446	GOBP_RESPONSE_TO_CARBOHYDRATE	GO:0009743	5	0.316124	0.678533	0.867450	0.944925	1.000
447	GOBP_REGULATION_OF_SIGNALING_RECEPTOR_ACTIVITY	GO:0010469	14	0.223878	0.673337	0.882261	0.948363	1.000
448	GOBP_RESPONSE_TO_MONOSACCHARIDE	GO:0034284	5	0.316124	0.672474	0.885615	0.947135	1.000
449	REACTOME_INTERFERON_SIGNALING	R-RNO-913531	6	0.302405	0.672344	0.880131	0.945167	1.000
450	GOBP_NEGATIVE_REGULATION_OF_TRANSCRIPTION_BY_R...	GO:0000122	10	0.248792	0.671990	0.865325	0.943408	1.000
451	GOBP_POSITIVE_REGULATION_OF_PEPTIDYL_TYROSINE_...	GO:0050731	8	0.266278	0.670207	0.876161	0.943210	1.000
452	GOBP_REGULATION_OF_PEPTIDYL_TYROSINE_PHOSPHORY...	GO:0050730	9	0.255739	0.670059	0.888025	0.941313	1.000
453	GOBP_AXON_DEVELOPMENT	GO:0061564	18	0.215632	0.669048	0.870968	0.940311	1.000
454	GOBP_NEGATIVE_REGULATION_OF_KINASE_ACTIVITY	GO:0033673	5	0.299632	0.654671	0.900000	0.952772	1.000
455	GOBP_TRANSMEMBRANE_TRANSPORT	GO:0055085	27	0.188518	0.653054	0.905444	0.952259	1.000
456	GOBP_PRI_MIRNA_TRANSCRIPTION_BY_RNA_POLYMERASE_II	GO:0061614	7	0.268801	0.647041	0.902973	0.956156	1.000
457	GOBP_NEGATIVE_REGULATION_OF_TRANSFERASE_ACTIVITY	GO:0051348	5	0.299632	0.643248	0.914634	0.957674	1.000
458	GOBP_POSITIVE_REGULATION_OF_PRI_MIRNA_TRANSCRI...	GO:1902895	7	0.268801	0.640063	0.913540	0.958536	1.000
459	GOBP_REGULATION_OF_MAPK_CASCADE	GO:0043408	13	0.221274	0.639833	0.907353	0.956645	1.000
460	GOBP_CELLULAR_RESPONSE_TO_EXTRACELLULAR_STIMULUS	GO:0031668	8	0.249104	0.638959	0.900813	0.955340	1.000
461	REACTOME_LEISHMANIA_INFECTION	R-HSA-9658195	10	0.234170	0.638627	0.905956	0.953602	1.000
462	GOBP_CELL_MORPHOGENESIS_INVOLVED_IN_NEURON_DIF...	GO:0048667	22	0.194129	0.638581	0.912722	0.951583	1.000
463	GOBP_REGULATION_OF_CELLULAR_COMPONENT_BIOGENESIS	GO:0044087	10	0.237287	0.638313	0.926984	0.949765	1.000
464	GOBP_CELL_MORPHOGENESIS_INVOLVED_IN_DIFFERENTI...	GO:0000904	22	0.194129	0.633375	0.922401	0.952118	1.000
465	GOBP_POSITIVE_REGULATION_OF_NERVOUS_SYSTEM_DEV...	GO:0051962	12	0.219988	0.632319	0.902022	0.951022	1.000
466	GOBP_PROTEIN_MODIFICATION_BY_SMALL_PROTEIN_CON...	GO:0070647	7	0.267586	0.630601	0.929146	0.950468	1.000
467	HALLMARK_HYPOXIA	M5891	5	0.289757	0.628972	0.919672	0.949698	1.000
468	GOBP_POSITIVE_REGULATION_OF_CELL_DEVELOPMENT	GO:0010720	12	0.219988	0.627932	0.927100	0.948517	1.000
469	GOBP_POSITIVE_REGULATION_OF_NEUROGENESIS	GO:0050769	12	0.219988	0.625617	0.915873	0.948494	1.000
470	GOBP_REGULATION_OF_MAP_KINASE_ACTIVITY	GO:0043405	9	0.243214	0.621595	0.916129	0.949833	1.000

Continuation of Table **S8**

	NAME	ID	SIZE	ES	NES	NOM.p.val	FDR.q.val	FWER.p.val
471	GOBP_REGULATION_OF_CIRCADIAN_RHYTHM	GO:0042752	7	0.253986	0.614443	0.911717	0.953731	1.000
472	GOBP_POSITIVE_REGULATION_OF_CELL_DIFFERENTIATION	GO:0045597	27	0.175149	0.607805	0.944915	0.956731	1.000
473	GOBP_POSITIVE_REGULATION_OF_INTRACELLULAR_SIGN...	GO:1902533	14	0.201074	0.599680	0.950617	0.960341	1.000
474	GOBP_RESPONSE_TO_INSULIN	GO:0032868	5	0.277108	0.593571	0.946218	0.962472	1.000
475	GOBP_G_PROTEIN_COUPLED_RECEPTOR_SIGNALING_PATHWAY	GO:0007186	18	0.186823	0.582440	0.942222	0.967754	1.000
476	GOBP_PURINE_CONTAINING_COMPOUND_METABOLIC_PROCESS	GO:0072521	6	0.255271	0.568945	0.956882	0.973543	1.000
477	HALLMARK_UV_RESPONSE_UP	M5941	5	0.269483	0.565472	0.948387	0.973326	1.000
478	GOBP_REGULATION_OF_AXONOGENESIS	GO:0050770	7	0.233223	0.557679	0.954248	0.975508	1.000
479	GOBP_MUSCLE_TISSUE_DEVELOPMENT	GO:0060537	11	0.201726	0.556529	0.965723	0.974073	1.000
480	GOBP_PROTEIN_LOCALIZATION_TO_POSTSYNAPSE	GO:0062237	5	0.252372	0.543229	0.971235	0.978316	1.000
481	GOBP_PROTEIN_LOCALIZATION_TO_SYNAPSE	GO:0035418	5	0.252372	0.529382	0.971901	0.982193	1.000
482	GOBP_CELL_SURFACE_RECEPTOR_SIGNALING_PATHWAY_I...	GO:1905114	15	0.164384	0.494930	0.978788	0.991200	1.000
483	GOBP_REGULATION_OF_GTPASE_ACTIVITY	GO:0043087	5	0.215819	0.465571	0.989967	0.995189	1.000
484	REACTOME_CELLULAR_RESPONSES_TO_EXTERNAL_STIMULI	R-RNO-8953897	11	0.167175	0.464879	0.995482	0.993217	1.000
485	REACTOME_RNA_POLYMERASE_II_TRANSCRIPTION	R-RNO-73857	20	-0.123186	-0.465799	1.000000	0.996698	1.000
486	GOBP_STRIATED_MUSCLE_CELL_DIFFERENTIATION	GO:0051146	9	-0.164557	-0.479524	0.994350	0.999266	1.000
487	REACTOME_TRANSCRIPTIONAL_REGULATION_BY_MECP2	R-RNO-8986944	7	-0.191591	-0.504664	0.997348	0.999203	1.000
488	GOBP_MUSCLE_CELL_DIFFERENTIATION	GO:0042692	10	-0.166667	-0.514977	0.982906	1.000000	1.000
489	REACTOME_SENESCENCE_ASSOCIATED_SECRETORY_PHENO...	R-RNO-2559582	6	-0.222271	-0.556103	0.967568	0.991866	1.000
490	REACTOME_CELLULAR_SENESCENCE	R-RNO-2559583	6	-0.222271	-0.569412	0.964557	0.989928	1.000
491	GOBP_ADENYLATE_CYCLASE_MODULATING_G_PROTEIN_CO...	GO:0007188	11	-0.186940	-0.587283	0.955556	0.984723	1.000
492	REACTOME_FC_EPSILON_RECEPTOR_FCERI_SIGNALING	R-RNO-2454202	7	-0.239276	-0.609136	0.923077	0.974938	1.000
493	GOBP_HEAD_DEVELOPMENT	GO:0060322	23	-0.160242	-0.614755	0.947712	0.974944	1.000
494	GOBP_ADENYLATE_CYCLASE_INHIBITING_G_PROTEIN_CO...	GO:0007193	9	-0.215190	-0.628394	0.924282	0.968275	1.000
495	GOBP_FC_EPSILON_RECEPTOR_SIGNALING_PATHWAY	GO:0038095	7	-0.239276	-0.635113	0.938667	0.966634	1.000
496	GOBP_CIRCADIAN_REGULATION_OF_GENE_EXPRESSION	GO:0032922	5	-0.276140	-0.646065	0.927273	0.960601	1.000
497	GOBP_RESPONSE_TO_STARVATION	GO:0042594	5	-0.283142	-0.650781	0.903704	0.960487	1.000
498	GOBP_RESPONSE_TO_INTERLEUKIN_1	GO:0070555	5	-0.284838	-0.653714	0.901809	0.961677	1.000
499	GOBP_REGULATION_OF_NEUROTRANSMITTER_RECEPTOR_A...	GO:0099601	13	-0.195481	-0.654521	0.898551	0.965121	1.000
500	GOBP_CELLULAR_RESPONSE_TO_STARVATION	GO:0009267	5	-0.283142	-0.658682	0.904040	0.965534	1.000
501	HALLMARK_TNFA_SIGNALING_VIA_NFKB	M5890	14	-0.200888	-0.661839	0.921630	0.966886	1.000
502	GOBP_CARDIAC_MUSCLE_TISSUE_DEVELOPMENT	GO:0048738	6	-0.269587	-0.670334	0.911616	0.961863	1.000
503	GOBP_HEART_DEVELOPMENT	GO:0007507	6	-0.269587	-0.670944	0.874372	0.965380	1.000
504	GOBP_CALCIUM_MEDIATED_SIGNALING	GO:0019722	10	-0.227258	-0.680030	0.889785	0.958847	1.000
505	REACTOME_LONG_TERM_POTENTIATION	R-HSA-9620244	10	-0.230769	-0.681744	0.857143	0.961359	1.000
506	GOBP_POSITIVE_REGULATION_OF_DEVELOPMENTAL_PROCESS	GO:0051094	32	-0.162931	-0.689288	0.910394	0.956818	1.000
507	REACTOME_INTEGRATION_OF_ENERGY_METABOLISM	R-RNO-163685	5	-0.301928	-0.695354	0.829146	0.953271	1.000
508	HALLMARK_EPITHELIAL_MESENCHYMAL_TRANSITION	M5930	5	-0.300634	-0.701377	0.851759	0.949871	1.000
509	GOBP_CELLULAR_GLUCOSE_HOMEOSTASIS	GO:0001678	5	-0.301575	-0.705709	0.853659	0.948157	1.000
510	GOBP_SMALL_GTPASE_MEDIATED_SIGNAL_TRANSDUCTION	GO:0007264	8	-0.255561	-0.707676	0.864000	0.949793	1.000
511	GOBP_ACTIVATION_OF_IMMUNE_RESPONSE	GO:0002253	5	-0.310665	-0.712578	0.815789	0.947851	1.000
512	GOBP_PROTEIN_MODIFICATION_BY_SMALL_PROTEIN_CON...	GO:0032446	6	-0.292821	-0.712630	0.798578	0.952225	1.000
513	GOBP_NEGATIVE_REGULATION_OF_MULTICELLULAR_ORGA...	GO:0051241	15	-0.206393	-0.717870	0.851312	0.949263	1.000

Continuation of Table **S8**

	NAME	ID	SIZE	ES	NES	NOM.p.val	FDR.q.val	FWER.p.val
514	GOBP_REGULATION_OF_ORGAN_GROWTH	GO:0046620	5	-0.312659	-0.722558	0.816121	0.946963	1.000
515	GOBP_REGULATION_OF_PROTEIN_STABILITY	GO:0031647	5	-0.313842	-0.728050	0.802564	0.943638	1.000
516	GOBP_NUCLEAR_TRANSPORT	GO:0051169	5	-0.314507	-0.730524	0.823232	0.944683	1.000
517	GOBP_CENTRAL_NERVOUS_SYSTEM_DEVELOPMENT	GO:0007417	27	-0.179942	-0.731625	0.864780	0.947488	1.000
518	GOBP_ORGAN_GROWTH	GO:0035265	5	-0.312659	-0.738010	0.798077	0.941715	1.000
519	GOBP_CHEMICAL_SYNAPTIC_TRANSMISSION_POSTSYNAPTIC	GO:0099565	9	-0.252468	-0.739961	0.831551	0.943195	1.000
520	REACTOME_ONCOGENIC_MAPK_SIGNALING	R-HSA-6802957	5	-0.321002	-0.740085	0.805755	0.947628	1.000
521	REACTOME_PTEN_REGULATION	R-RNO-6807070	5	-0.318334	-0.741551	0.799032	0.949913	1.000
522	GOBP_CELLULAR_RESPONSE_TO_PEPTIDE	GO:1901653	13	-0.231209	-0.744935	0.817365	0.949101	1.000
523	GOBP_REGULATION_OF_MUSCLE_CELL_DIFFERENTIATION	GO:0051147	6	-0.292683	-0.747040	0.773196	0.950145	1.000
524	GOBP_PEPTIDYL_TYROSINE_MODIFICATION	GO:0018212	10	-0.252156	-0.756438	0.766017	0.939998	1.000
525	GOBP_IONOTROPIC_GLUTAMATE_RECEPTOR_SIGNALING_P...	GO:0035235	9	-0.260686	-0.757500	0.780899	0.943010	1.000
526	GOBP_MAPK_CASCADE	GO:0000165	18	-0.208081	-0.758138	0.804416	0.946671	1.000
527	GOBP_CELLULAR_RESPONSE_TO_ALCOHOL	GO:0097306	5	-0.320362	-0.761673	0.772947	0.945356	1.000
528	GOBP_NEGATIVE_REGULATION_OF_GENE_EXPRESSION	GO:0010629	11	-0.254639	-0.762293	0.763736	0.949217	1.000
529	GOBP_ADENYLATE_CYCLASE_INHIBITING_G_PROTEIN_CO...	GO:0007196	5	-0.319039	-0.762907	0.771290	0.952937	1.000
530	GOBP_CELLULAR_RESPONSE_TO_MOLECULE_OF_BACTERIA...	GO:0071219	8	-0.282041	-0.766307	0.744681	0.951783	1.000
531	GOBP_PROTEIN_AUTOPHOSPHORYLATION	GO:0046777	5	-0.333459	-0.771511	0.712737	0.947897	1.000
532	GOBP_LONG_TERM_SYNAPTIC_POTENTIATION	GO:0060291	12	-0.240740	-0.772275	0.752022	0.951451	1.000
533	REACTOME_DAG_AND_IP3_SIGNALING	R-RNO-1489509	8	-0.290713	-0.790385	0.737127	0.923639	1.000
534	GOBP_POSITIVE_REGULATION_OF_CELL_ADHESION	GO:0045785	7	-0.302610	-0.791134	0.694581	0.927062	1.000
535	GOBP_CELLULAR_RESPONSE_TO_BIOTIC_STIMULUS	GO:0071216	8	-0.282041	-0.800740	0.724311	0.914688	1.000
536	GOBP_NEGATIVE_REGULATION_OF_PHOSPHORYLATION	GO:0042326	7	-0.311726	-0.807121	0.682081	0.907340	1.000
537	GOBP_CELLULAR_RESPONSE_TO_LIPID	GO:0071396	15	-0.234555	-0.808222	0.741379	0.910224	1.000
538	REACTOME_GPCR_LIGAND_BINDING	R-RNO-500792	8	-0.288029	-0.808524	0.722222	0.914539	1.000
539	GOBP_MULTICELLULAR_ORGANISM_REPRODUCTION	GO:0032504	12	-0.258422	-0.808606	0.718391	0.919281	1.000
540	GOBP_CELL_CYCLE_PROCESS	GO:0022402	11	-0.257776	-0.811495	0.698864	0.918671	1.000
541	GOBP_CATION_TRANSMEMBRANE_TRANSPORT	GO:0098655	18	-0.218817	-0.816378	0.725490	0.914094	1.000
542	GOBP_POSITIVE_REGULATION_OF_MAPK_CASCADE	GO:0043410	9	-0.287201	-0.818418	0.705882	0.915203	1.000
543	GOBP_REGULATION_OF_CELL_CYCLE	GO:0051726	13	-0.245352	-0.818842	0.686813	0.919376	1.000
544	GOBP_RESPONSE_TO_WOUNDING	GO:0009611	15	-0.234839	-0.820998	0.708475	0.920329	1.000
545	GOBP_REGULATION_OF_CATION_TRANSMEMBRANE_TRANSPORT	GO:1904062	16	-0.235854	-0.827636	0.732558	0.912503	1.000
546	GOBP_CENTRAL_NERVOUS_SYSTEM_NEURON_DIFFERENTIA...	GO:0021953	5	-0.354529	-0.830699	0.669951	0.911536	1.000
547	GOBP_ORGANIC_ACID_TRANSPORT	GO:0015849	7	-0.312483	-0.831154	0.661247	0.915790	1.000
548	GOBP_SYNAPTIC_SIGNALING	GO:0099536	47	-0.189830	-0.831195	0.736111	0.920901	1.000
549	GOBP_REGULATION_OF_LEUKOCYTE_DIFFERENTIATION	GO:1902105	9	-0.287349	-0.834592	0.683924	0.919064	1.000
550	GOBP_INTRACELLULAR_RECEPTOR_SIGNALING_PATHWAY	GO:0030522	5	-0.361446	-0.835666	0.635697	0.922308	1.000
551	GOBP_DEVELOPMENTAL_PROCESS_INVOLVED_IN_REPRODU...	GO:0003006	11	-0.271687	-0.837441	0.678679	0.924002	1.000
552	GOBP_SENSORY_PERCEPTION_OF_PAIN	GO:0019233	5	-0.356824	-0.846704	0.647754	0.909909	1.000
553	REACTOME_L1CAM_INTERACTIONS	R-RNO-373760	5	-0.371938	-0.847899	0.641278	0.912885	1.000
554	GOBP_TELENCEPHALON_DEVELOPMENT	GO:0021537	7	-0.327266	-0.854563	0.620948	0.904387	1.000
555	GOBP_REGULATION_OF_NERVOUS_SYSTEM_PROCESS	GO:0031644	8	-0.312500	-0.854785	0.656250	0.909208	1.000
556	GOBP_RAS_PROTEIN_SIGNAL_TRANSDUCTION	GO:0007265	5	-0.369032	-0.861333	0.619403	0.900406	1.000

Continuation of Table **S8**

	NAME	ID	SIZE	ES	NES	NOM.p.val	FDR.q.val	FWER.p.val
557	GOBP_ERBB_SIGNALING_PATHWAY	GO:0038127	5	-0.376318	-0.862676	0.600000	0.902787	1.000
558	GOBP_DEVELOPMENTAL_MATURATION	GO:0021700	5	-0.376095	-0.871410	0.606138	0.889387	1.000
559	GOBP_RESPONSE_TO_ORGANIC_CYCLIC_COMPOUND	GO:0014070	20	-0.232127	-0.871425	0.692568	0.894716	1.000
560	GOBP_PROTEIN_LOCALIZATION_TO_ORGANELLE	GO:0033365	8	-0.316730	-0.872468	0.643052	0.898238	1.000
561	GOBP_MONONUCLEAR_CELL_DIFFERENTIATION	GO:1903131	7	-0.337335	-0.876423	0.587786	0.895279	1.000
562	GOBP_WOUND_HEALING	GO:0042060	13	-0.266552	-0.878041	0.619048	0.897100	1.000
563	GOBP_ORGANOPHOSPHATE_METABOLIC_PROCESS	GO:0019637	8	-0.321259	-0.878735	0.605744	0.901120	1.000
564	GOBP_MITOTIC_CELL_CYCLE	GO:0000278	10	-0.295074	-0.884707	0.584856	0.893021	1.000
565	GOBP_CELL_CYCLE	GO:0007049	21	-0.232014	-0.884755	0.616822	0.898536	1.000
566	GOBP_DIVALENT_INORGANIC_CATION_HOMEOSTASIS	GO:0072507	13	-0.268182	-0.884794	0.605042	0.904078	1.000
567	GOBP_REGULATION_OF_CYTOSOLIC_CALCIUM_ION_CONCE...	GO:0051480	13	-0.268182	-0.890004	0.606838	0.897193	1.000
568	GOBP_RESPONSE_TO_OXYGEN_CONTAINING_COMPOUND	GO:1901700	39	-0.207382	-0.896827	0.620301	0.887863	1.000
569	REACTOME_UNBLOCKING_OF_NMDA_RECEPTORS_GLUTAMAT...	R-RNO-438066	12	-0.272492	-0.899131	0.563050	0.888443	1.000
570	GOBP_REGULATION_OF_CELL_ADHESION	GO:0030155	13	-0.269920	-0.903591	0.570652	0.883910	1.000
571	GOBP_REGULATION_OF_NEURONAL_SYNAPTIC_PLASTICITY	GO:0048168	7	-0.347838	-0.903920	0.550532	0.888955	1.000
572	GOBP_REGULATION_OF_SYNAPTIC_PLASTICITY	GO:0048167	21	-0.239425	-0.911706	0.595819	0.876946	1.000
573	GOBP_TISSUE_MIGRATION	GO:0090130	9	-0.316349	-0.913044	0.563636	0.879695	1.000
574	GOBP_POSITIVE_REGULATION_OF_TRANSCRIPTION_BY_R...	GO:0045944	24	-0.235271	-0.914474	0.607029	0.882010	1.000
575	GOBP_NUCLEOBASE_CONTAINING_SMALL_MOLECULE_META...	GO:0055086	7	-0.352207	-0.918375	0.519231	0.878980	1.000
576	GOBP_SIGNAL_RELEASE	GO:0023061	14	-0.268200	-0.921973	0.573370	0.876616	1.000
577	GOBP_FATTY_ACID_TRANSPORT	GO:0015908	6	-0.361819	-0.926370	0.524155	0.872310	1.000
578	GOBP_REGULATION_OF_TRANSPORTER_ACTIVITY	GO:0032409	17	-0.266553	-0.928805	0.530547	0.872441	1.000
579	GOBP_CELLULAR_COMPONENT_MAINTENANCE	GO:0043954	5	-0.394519	-0.930372	0.509434	0.874593	1.000
580	GOBP_EMBRYO_DEVELOPMENT_ENDING_IN_BIRTH_OR_EGG...	GO:0009792	9	-0.324472	-0.937194	0.502645	0.864421	1.000
581	GOBP_EXCITATORY_CHEMICAL_SYNAPTIC_TRANSMISSION	GO:0098976	5	-0.409639	-0.937537	0.487245	0.869577	1.000
582	GOBP_REGULATION_OF_CATION_CHANNEL_ACTIVITY	GO:2001257	14	-0.279167	-0.939720	0.539157	0.870167	1.000
583	GOBP_REGULATION_OF_MEMBRANE_POTENTIAL	GO:0042391	17	-0.255253	-0.946615	0.529781	0.859462	1.000
584	GOBP_NEGATIVE_REGULATION_OF_TRANSPORT	GO:0051051	13	-0.288460	-0.947089	0.506631	0.864343	1.000
585	GOBP_INTRACELLULAR_TRANSPORT	GO:0046907	10	-0.316758	-0.947826	0.528875	0.868353	1.000
586	GOBP_TUBE_MORPHOGENESIS	GO:0035239	15	-0.279874	-0.948872	0.501520	0.872055	1.000
587	GOBP_REGULATION_OF_POSTSYNAPTIC_MEMBRANE_POTEN...	GO:0060078	14	-0.279329	-0.949256	0.488439	0.877298	1.000
588	GOBP_RESPONSE_TO_HORMONE	GO:0009725	20	-0.250457	-0.952379	0.524845	0.876172	1.000
589	GOBP_NEGATIVE_REGULATION_OF_CELL_PROJECTION_OR...	GO:0031345	5	-0.412094	-0.952807	0.497449	0.881728	1.000
590	GOBP_DNA_METABOLIC_PROCESS	GO:0006259	6	-0.389550	-0.964045	0.497561	0.860745	1.000
591	GOBP_REGULATION_OF_BODY_FLUID_LEVELS	GO:0050878	11	-0.313154	-0.969118	0.491071	0.855000	1.000
592	GOBP_CELLULAR_RESPONSE_TO_OXYGEN_CONTAINING_CO...	GO:1901701	29	-0.229191	-0.970686	0.501639	0.857879	1.000
593	GOBP_CELL_JUNCTION_ORGANIZATION	GO:0034330	19	-0.263086	-0.976853	0.512195	0.849215	1.000
594	GOBP_PEPTIDYL_LYSINE_MODIFICATION	GO:0018205	5	-0.421687	-0.978067	0.459135	0.852612	1.000
595	GOBP_POSTTRANSCRIPTIONAL_REGULATION_OF_GENE_EX...	GO:0010608	8	-0.355705	-0.983209	0.435530	0.846020	1.000
596	GOBP_CIRCADIAN_RHYTHM	GO:0007623	13	-0.301943	-0.986396	0.448179	0.844412	1.000
597	GOBP_NEGATIVE_REGULATION_OF_CELL_CYCLE	GO:0045786	5	-0.422990	-0.989018	0.439153	0.844602	1.000
598	GOBP_ION_HOMEOSTASIS	GO:0050801	16	-0.285720	-0.992548	0.498442	0.842587	1.000
599	GOBP_NUCLEOSIDE_PHOSPHATE_BIOSYNTHETIC_PROCESS	GO:1901293	5	-0.433867	-0.994340	0.402564	0.844711	1.000

Continuation of Table **S8**

	NAME	ID	SIZE	ES	NES	NOM.p.val	FDR.q.val	FWER.p.val
600	GOBP_POSITIVE_REGULATION_OF_CELL_POPULATION_PR...	GO:0008284	21	-0.257296	-0.994828	0.454248	0.850271	1.000
601	REACTOME_ACTIVATION_OF_NMDA_RECEPTORS_AND_POST...	R-RNO-442755	16	-0.285815	-0.997261	0.449686	0.850728	1.000
602	GOBP_ORGANOPHOSPHATE_BIOSYNTHETIC_PROCESS	GO:0090407	5	-0.433867	-1.000910	0.405941	0.848062	1.000
603	GOBP_REGULATION_OF_LYMPHOCYTE_ACTIVATION	GO:0051249	5	-0.432380	-1.002407	0.426316	0.851182	1.000
604	GOBP_CELL_CELL_SIGNALING	GO:0007267	56	-0.228109	-1.011132	0.488722	0.835959	1.000
605	GOBP_SENSORY_ORGAN_DEVELOPMENT	GO:0007423	8	-0.372816	-1.015803	0.442815	0.831057	1.000
606	GOBP_CHROMATIN_ORGANIZATION	GO:0006325	5	-0.440684	-1.017015	0.417874	0.835256	1.000
607	REACTOME_SYNAPTIC_ADHESION_LIKE_MOLECULES	R-RNO-8849932	9	-0.347305	-1.020435	0.400576	0.833454	1.000
608	GOBP_NEUROTRANSMITTER_SECRETION	GO:0007269	6	-0.409184	-1.025063	0.410811	0.828958	1.000
609	GOBP_PEPTIDYL_AMINO_ACID_MODIFICATION	GO:0018193	22	-0.271854	-1.029677	0.386503	0.824282	1.000
610	REACTOME_OPIOID_SIGNALLING	R-RNO-111885	12	-0.316767	-1.030936	0.371681	0.828131	1.000
611	REACTOME_G_ALPHA_I_SIGNALLING_EVENTS	R-RNO-418594	18	-0.285294	-1.040324	0.395137	0.811748	1.000
612	GOBP_HOMEOSTATIC_PROCESS	GO:0042592	31	-0.246966	-1.042359	0.401274	0.814144	1.000
613	REACTOME_SIGNALING_BY_GPCR	R-RNO-372790	22	-0.270965	-1.046192	0.403786	0.811893	1.000
614	GOBP_EPIDERMIS_DEVELOPMENT	GO:0008544	5	-0.450774	-1.059160	0.340852	0.785794	1.000
615	GOBP_MUSCLE_CONTRACTION	GO:0006936	5	-0.469880	-1.065673	0.361179	0.776254	1.000
616	GOBP_REGULATION_OF_CELL_CELL_ADHESION	GO:0022407	9	-0.366146	-1.069118	0.362117	0.774713	1.000
617	GOBP_CIRCULATORY_SYSTEM_DEVELOPMENT	GO:0072359	21	-0.279545	-1.071580	0.366883	0.775759	1.000
618	GOBP_EMBRYO_DEVELOPMENT	GO:0009790	14	-0.313255	-1.077867	0.348571	0.767392	1.000
619	GOBP_REGULATION_OF_CELL_ACTIVATION	GO:0050865	8	-0.393407	-1.082446	0.331461	0.763416	1.000
620	GOBP_NEURON_MIGRATION	GO:0001764	5	-0.469880	-1.083499	0.332524	0.768072	1.000
621	GOBP_POSITIVE_REGULATION_OF_CELLULAR_PROTEIN_L...	GO:1903829	7	-0.400618	-1.084911	0.338462	0.771478	1.000
622	GOBP_CHEMICAL_HOMEOSTASIS	GO:0048878	23	-0.273175	-1.085782	0.331190	0.776773	1.000
623	REACTOME_HEMOSTASIS	R-RNO-109582	14	-0.320999	-1.086846	0.336449	0.781740	1.000
624	GOBP_REGULATION_OF_SYSTEM_PROCESS	GO:0044057	18	-0.302034	-1.090419	0.305556	0.780171	1.000
625	GOBP_BLOOD_VESSEL_ENDOTHELIAL_CELL_MIGRATION	GO:0043534	8	-0.390442	-1.092668	0.320113	0.782170	1.000
626	GOBP_REPRODUCTIVE_SYSTEM_DEVELOPMENT	GO:0061458	6	-0.450453	-1.095566	0.338624	0.782525	1.000
627	GOBP_ORGANONITROGEN_COMPOUND_BIOSYNTHETIC_PROCESS	GO:1901566	13	-0.324641	-1.096678	0.299145	0.787230	1.000
628	GOBP_PHAGOCYTOSIS	GO:0006909	5	-0.467382	-1.097065	0.316832	0.794352	1.000
629	REACTOME_G_ALPHA_Z_SIGNALLING_EVENTS	R-RNO-418597	5	-0.471752	-1.099171	0.322251	0.797661	1.000
630	GOBP_ENDOTHELIAL_CELL_MIGRATION	GO:0043542	8	-0.390442	-1.100108	0.299728	0.803577	1.000
631	GOBP_POSITIVE_REGULATION_OF_APOPTOTIC_SIGNALIN...	GO:2001235	5	-0.468288	-1.100532	0.330864	0.810934	1.000
632	REACTOME_SIGNALING_BY_WNT	R-RNO-195721	7	-0.420337	-1.105882	0.290640	0.805106	1.000
633	GOBP_CALCIUM_ION_TRANSMEMBRANE_TRANSPORT	GO:0070588	7	-0.424147	-1.110710	0.280105	0.800752	1.000
634	GOBP_CALCIUM_ION_TRANSPORT_INTO_CYTOSOL	GO:0060402	7	-0.424147	-1.117466	0.304239	0.792073	1.000
635	GOBP_ENDOMEMBRANE_SYSTEM_ORGANIZATION	GO:0010256	6	-0.449637	-1.117554	0.293532	0.800688	1.000
636	GOBP_CYTOSOLIC_CALCIUM_ION_TRANSPORT	GO:0060401	7	-0.424147	-1.118174	0.289406	0.807864	1.000
637	GOBP_ERK1_AND_ERK2_CASCADE	GO:0070371	5	-0.488402	-1.121050	0.298969	0.808653	1.000
638	GOBP_BLOOD_VESSEL_MORPHOGENESIS	GO:0048514	14	-0.322893	-1.121898	0.268908	0.815701	1.000
639	GOBP_MUSCLE_CELL_DEVELOPMENT	GO:0055001	5	-0.469880	-1.125702	0.278772	0.814871	1.000
640	GOBP_SENSORY_PERCEPTION	GO:0007600	9	-0.387885	-1.130130	0.296296	0.811991	1.000
641	GOBP_GLUTAMATE_RECEPTOR_SIGNALING_PATHWAY	GO:0007215	17	-0.312382	-1.131526	0.258462	0.817675	1.000
642	GOBP_LIPID_LOCALIZATION	GO:0010876	10	-0.374061	-1.132740	0.260054	0.824113	1.000

Continuation of Table **S8**

	NAME	ID	SIZE	ES	NES	NOM.p.val	FDR.q.val	FWER.p.val
643	GOBP_ANIMAL_ORGAN_MORPHOGENESIS	GO:0009887	12	-0.350059	-1.133052	0.271084	0.833153	1.000
644	GOBP_INTRACELLULAR_PROTEIN_TRANSPORT	GO:0006886	8	-0.411156	-1.133831	0.279070	0.840925	1.000
645	GOBP_POSITIVE_REGULATION_OF_PROTEIN_METABOLIC_...	GO:0051247	27	-0.270975	-1.139933	0.242902	0.832962	1.000
646	GOBP_REGULATION_OF_SECRETION	GO:0051046	14	-0.339192	-1.141279	0.253731	0.839358	1.000
647	GOBP_EPITHELIUM_DEVELOPMENT	GO:0060429	15	-0.329365	-1.147356	0.266254	0.833476	1.000
648	GOBP_POSITIVE_REGULATION_OF_BIOSYNTHETIC_PROCESS	GO:0009891	27	-0.276951	-1.151029	0.224561	0.833618	1.000
649	REACTOME_NEUROTRANSMITTER_RECEPTORS_AND_POSTSY...	R-RNO-112314	22	-0.305125	-1.156845	0.245342	0.827267	1.000
650	GOBP_CELLULAR_RESPONSE_TO_DNA_DAMAGE_STIMULUS	GO:0006974	5	-0.492868	-1.157290	0.250000	0.836938	1.000
651	GOBP_REGULATION_OF_TRANSPORT	GO:0051049	44	-0.264398	-1.157381	0.242308	0.847915	1.000
652	REACTOME_NEGATIVE_REGULATION_OF_NMDA_RECEPTOR_...	R-HSA-961732	8	-0.425000	-1.165445	0.263587	0.835931	1.000
653	REACTOME_CELL_CYCLE_MITOTIC	R-RNO-69278	5	-0.512472	-1.169057	0.270208	0.836588	1.000
654	REACTOME_G_PROTEIN_MEDIATED_EVENTS	R-RNO-112040	9	-0.404509	-1.176073	0.247222	0.826860	1.000
655	REACTOME_CLASS_C_3_METABOTROPIC_GLUTAMATE_PHER...	R-RNO-420499	7	-0.438878	-1.181698	0.273196	0.822243	1.000
656	GOBP_SECOND_MESSENGER_MEDIATED_SIGNALING	GO:0019932	16	-0.335163	-1.181712	0.232416	0.834159	1.000
657	GOBP_RESPONSE_TO_HEAT	GO:0009408	7	-0.454547	-1.186666	0.230137	0.832000	1.000
658	GOBP_INORGANIC_ION_TRANSMEMBRANE_TRANSPORT	GO:0098660	9	-0.410062	-1.189560	0.220207	0.835034	1.000
659	GOBP_G_PROTEIN_COUPLED_GLUTAMATE_RECEPTOR_SIGN...	GO:0007216	8	-0.426943	-1.189989	0.257895	0.846391	1.000
660	GOBP_CELLULAR_MACROMOLECULE_CATABOLIC_PROCESS	GO:0044265	5	-0.528778	-1.194974	0.250000	0.844238	1.000
661	GOBP_REGULATION_OF_NMDA_RECEPTOR_ACTIVITY	GO:2000310	10	-0.392088	-1.197114	0.238606	0.850653	1.000
662	REACTOME_INTRACELLULAR_SIGNALING_BY_SECOND_MES...	R-RNO-9006925	15	-0.354260	-1.198532	0.206395	0.860017	1.000
663	REACTOME_ASSEMBLY_AND_CELL_SURFACE_PRESENTATIO...	R-RNO-9609736	8	-0.425000	-1.200109	0.211082	0.868937	1.000
664	REACTOME_PLATELET_ACTIVATION_SIGNALING_AND_AGG...	R-HSA-76002	7	-0.444800	-1.203356	0.240933	0.872798	1.000
665	GOBP_CELLULAR_ION_HOMEOSTASIS	GO:0006873	14	-0.353332	-1.203588	0.213855	0.886603	1.000
666	GOBP_TUBE_DEVELOPMENT	GO:0035295	18	-0.323425	-1.204573	0.223242	0.898713	1.000
667	REACTOME_CA_DEPENDENT_EVENTS	R-RNO-111996	8	-0.443835	-1.208104	0.223684	0.902534	1.000
668	REACTOME_CREB1_PHOSPHORYLATION_THROUGH_NMDA_RE...	R-RNO-442742	8	-0.441865	-1.213370	0.216285	0.901392	1.000
669	REACTOME_MAPK_FAMILY_SIGNALING_CASCADES	R-RNO-5683057	12	-0.368648	-1.216308	0.221239	0.907398	1.000
670	GOBP_RHYTHMIC_PROCESS	GO:0048511	15	-0.352485	-1.218955	0.182109	0.915032	1.000
671	GOBP_MUSCLE_ADAPTATION	GO:0043500	5	-0.530120	-1.223430	0.199482	0.916755	1.000
672	REACTOME_RHO_GTPASE_EFFECTORS	R-RNO-195258	9	-0.427928	-1.227901	0.188482	0.918985	1.000
673	GOBP_POSITIVE_REGULATION_OF_CATALYTIC_ACTIVITY	GO:0043085	29	-0.300557	-1.228735	0.189189	0.933457	1.000
674	GOBP_REGULATION_OF_METAL_ION_TRANSPORT	GO:0010959	6	-0.487805	-1.228846	0.219451	0.951338	1.000
675	GOBP_COAGULATION	GO:0050817	8	-0.435935	-1.230297	0.223404	0.964718	1.000
676	REACTOME_SIGNALING_BY_RHO_GTPASES_MIRO_GTPASES...	R-RNO-9716542	9	-0.427928	-1.231845	0.214076	0.979382	1.000
677	REACTOME_CELL_CYCLE	R-RNO-1640170	6	-0.503087	-1.235179	0.189744	0.987031	1.000
678	GOBP_REGULATION_OF_MUSCLE_ADAPTATION	GO:0043502	5	-0.530120	-1.249901	0.206235	0.954187	1.000
679	GOBP_NEGATIVE_REGULATION_OF_DEVELOPMENTAL_PROCESS	GO:0051093	13	-0.387444	-1.255958	0.180124	0.951670	1.000
680	REACTOME_TRAFFICKING_OF_GLUR2_CONTAINING_AMPA_...	R-RNO-416993	8	-0.448839	-1.261993	0.169863	0.950040	1.000
681	REACTOME_TRAFFICKING_OF_AMPA_RECEPTORS	R-RNO-399719	11	-0.407090	-1.273898	0.166227	0.930005	1.000
682	GOBP_TISSUE_MORPHOGENESIS	GO:0048729	7	-0.483438	-1.276356	0.171795	0.941928	1.000
683	GOBP_REGULATION_OF_NEUROTRANSMITTER_TRANSPORT	GO:0051588	6	-0.532052	-1.285005	0.157754	0.932300	1.000
684	GOBP_SKIN_DEVELOPMENT	GO:0043588	5	-0.538375	-1.285045	0.180412	0.954883	1.000
685	GOBP_NEGATIVE_REGULATION_OF_CELL_DIFFERENTIATION	GO:0045596	13	-0.387444	-1.291267	0.178082	0.955441	1.000

Continuation of Table **S8**

	NAME	ID	SIZE	ES	NES	NOM.p.val	FDR.q.val	FWER.p.val
686	REACTOME_PROTEIN_PROTEIN_INTERACTIONS_AT_SYNAPSES	R-RNO-6794362	12	-0.401472	-1.294725	0.150142	0.965416	1.000
687	REACTOME_RAS_ACTIVATION_UPON_CA2_INFLUX_THROUG...	R-HSA-442982	6	-0.524390	-1.296099	0.176623	0.986077	1.000
688	GOBP_VASCULATURE_DEVELOPMENT	GO:0001944	17	-0.347857	-1.301902	0.142395	0.989233	1.000
689	GOBP_REGULATION_OF_NEUROTRANSMITTER_LEVELS	GO:0001505	7	-0.500127	-1.309387	0.145553	0.988354	1.000
690	GOBP_RESPONSE_TO_TEMPERATURE_STIMULUS	GO:0009266	9	-0.453829	-1.309628	0.125000	1.000000	1.000
691	GOBP_MYOTUBE_DIFFERENTIATION	GO:0014902	5	-0.561243	-1.323961	0.145320	0.988590	1.000
692	GOBP_CELLULAR_HOMEOSTASIS	GO:0019725	18	-0.362388	-1.325645	0.116208	1.000000	1.000
693	REACTOME_TRANSMISSION_ACROSS_CHEMICAL_SYNAPSES	R-RNO-112315	23	-0.342572	-1.338900	0.106628	0.991694	1.000
694	GOBP_POSITIVE_REGULATION_OF_EPITHELIAL_CELL_PR...	GO:0050679	7	-0.509509	-1.340526	0.131054	1.000000	1.000
695	GOBP_ORGANIC_ANION_TRANSPORT	GO:0015711	6	-0.536585	-1.346933	0.134434	1.000000	1.000
696	GOBP_POSITIVE_REGULATION_OF_NUCLEOBASE_CONTAIN...	GO:0045935	28	-0.331436	-1.352039	0.095082	1.000000	1.000
697	REACTOME_BETA_CATENIN_INDEPENDENT_WNT_SIGNALING	R-RNO-3858494	5	-0.588614	-1.352726	0.165816	1.000000	1.000
698	GOBP_POSITIVE_REGULATION_OF_ENDOTHELIAL_CELL_P...	GO:0001938	6	-0.539942	-1.358840	0.118090	1.000000	1.000
699	GOBP_CARBOXYLIC_ACID_TRANSPORT	GO:0046942	6	-0.536585	-1.359162	0.121287	1.000000	1.000
700	GOBP_CALCIUM_ION_TRANSPORT	GO:0006816	11	-0.441558	-1.361395	0.140162	1.000000	1.000
701	GOBP_NEUROTRANSMITTER_TRANSPORT	GO:0006836	7	-0.500127	-1.363157	0.118132	1.000000	1.000
702	GOBP_AMINO_ACID_TRANSPORT	GO:0006865	6	-0.536585	-1.369402	0.116580	1.000000	1.000
703	GOBP_METAL_ION_TRANSPORT	GO:0030001	11	-0.441558	-1.370622	0.123494	1.000000	1.000
704	GOBP_SPROUTING_ANGIOGENESIS	GO:0002040	5	-0.593774	-1.381245	0.105911	1.000000	1.000
705	GOBP_PLATELET_ACTIVATION	GO:0030168	6	-0.561841	-1.399578	0.101604	1.000000	1.000
706	GOBP_REGULATION_OF_MUSCLE_SYSTEM_PROCESS	GO:0090257	8	-0.487500	-1.399604	0.090659	1.000000	1.000
707	GOBP_CHROMOSOME_ORGANIZATION	GO:0051276	6	-0.565826	-1.412235	0.100756	1.000000	1.000
708	GOBP_TAXIS	GO:0042330	14	-0.427532	-1.434595	0.092308	1.000000	1.000
709	GOBP_NEURON_PROJECTION_GUIDANCE	GO:0097485	9	-0.498428	-1.448268	0.093093	1.000000	1.000
710	GOBP_MUSCLE_SYSTEM_PROCESS	GO:0003012	9	-0.493671	-1.448345	0.086592	1.000000	1.000
711	GOBP_ENDOTHELIAL_CELL_PROLIFERATION	GO:0001935	8	-0.520087	-1.450953	0.087324	1.000000	1.000
712	REACTOME_G_ALPHA_Q_SIGNALLING_EVENTS	R-RNO-416476	6	-0.575778	-1.452381	0.097884	1.000000	1.000
713	GOBP_EPITHELIAL_CELL_PROLIFERATION	GO:0050673	12	-0.447118	-1.456253	0.078550	1.000000	1.000
714	REACTOME_NEURONAL_SYSTEM	R-RNO-112316	26	-0.372852	-1.458926	0.032468	1.000000	1.000
715	GOBP_MULTICELLULAR_ORGANISMAL_RESPONSE_TO_STRESS	GO:0033555	5	-0.629350	-1.469535	0.096774	1.000000	1.000
716	REACTOME_NEUREXINS_AND_NEUROLIGINS	R-RNO-6794361	9	-0.525013	-1.471839	0.086826	1.000000	1.000
717	GOBP_DICARBOXYLIC_ACID_TRANSPORT	GO:0006835	5	-0.650602	-1.490914	0.078385	1.000000	1.000
718	GOBP_REGULATION_OF_PROTEIN_TYROSINE_KINASE_ACT...	GO:0061097	5	-0.664016	-1.512291	0.083933	1.000000	1.000
719	GOBP_MONOCARBOXYLIC_ACID_TRANSPORT	GO:0015718	5	-0.650602	-1.521315	0.062331	1.000000	1.000
720	GOBP_ACIDIC_AMINO_ACID_TRANSPORT	GO:0015800	5	-0.650602	-1.528778	0.069767	1.000000	1.000
721	GOBP_POSITIVE_REGULATION_OF_PROTEIN_TYROSINE_K...	GO:0061098	5	-0.664016	-1.528883	0.058081	1.000000	1.000
722	GOBP_EMBRYONIC_MORPHOGENESIS	GO:0048598	6	-0.651750	-1.588475	0.056266	1.000000	1.000
723	GOBP_LIPID_EXPORT_FROM_CELL	GO:0140353	5	-0.732434	-1.707909	0.036765	1.000000	0.998
724	GOBP_ANATOMICAL_STRUCTURE_FORMATION_INVOLVED_I...	GO:0048646	20	-0.483673	-1.861030	0.010000	1.000000	0.903

End of Table

Supplementary Table S9: SC cervical segment early profile (8 DPL peak) GSEA results.

Begin of Table **S9**

	NAME	ID	SIZE	ES	NES	NOM.p.val	FDR.q.val	FWER.p.val
0	REACTOME_INTERLEUKIN_4_AND_INTERLEUKIN_13_SIGN...	R-RNO-6785807	8	0.767776	1.928067	0.003279	1.000000	0.626
1	GOBP_REGULATION_OF_DEFENSE_RESPONSE	GO:0031347	7	0.708593	1.789521	0.019830	1.000000	0.951
2	GOBP_EPITHELIAL_CELL_DIFFERENTIATION	GO:0030855	7	0.692776	1.758604	0.021538	1.000000	0.981
3	GOBP_MULTI_MULTICELLULAR_ORGANISM_PROCESS	GO:0044706	8	0.663654	1.756552	0.018576	1.000000	0.982
4	GOBP_REGULATION_OF_INFLAMMATORY_RESPONSE	GO:0050727	7	0.708593	1.685824	0.036810	1.000000	0.997
5	GOBP_INFLAMMATORY_RESPONSE	GO:0006954	12	0.555844	1.672846	0.010345	1.000000	0.999
6	GOBP_OSTEOCLAST_DIFFERENTIATION	GO:0030316	6	0.731707	1.671057	0.022727	1.000000	0.999
7	GOBP_GENERATION_OF_PRECURSOR_METABOLITES_AND_E...	GO:0006091	5	0.750803	1.661808	0.041436	1.000000	0.999
8	GOBP_POLYSACCHARIDE_METABOLIC_PROCESS	GO:0005976	5	0.750910	1.635348	0.038567	1.000000	0.999
9	REACTOME_SIGNALING_BY_INTERLEUKINS	R-RNO-449147	15	0.484726	1.621109	0.022059	1.000000	1.000
10	GOBP_REGULATION_OF_OSTEOCLAST_DIFFERENTIATION	GO:0045670	5	0.722892	1.596826	0.057065	1.000000	1.000
11	GOBP_OSTEOBLAST_DIFFERENTIATION	GO:0001649	5	0.734940	1.596129	0.056497	0.985193	1.000
12	GOBP_EXTRINSIC_APOPTOTIC_SIGNALING_PATHWAY	GO:0097191	7	0.634945	1.590985	0.047059	0.933322	1.000
13	GOBP_CYTOKINE_MEDIATED_SIGNALING_PATHWAY	GO:0019221	16	0.465831	1.569645	0.053232	0.960134	1.000
14	GOBP_OSSIFICATION	GO:0001503	9	0.559035	1.526269	0.053628	1.000000	1.000
15	GOBP_NEGATIVE_REGULATION_OF_PEPTIDASE_ACTIVITY	GO:0010466	6	0.653057	1.510690	0.076487	1.000000	1.000
16	GOBP_REGULATION_OF_PROTEIN_LOCALIZATION_TO_CEL...	GO:1904375	6	0.628164	1.497259	0.087324	1.000000	1.000
17	GOBP_RESPONSE_TO_MECHANICAL_STIMULUS	GO:0009612	6	0.616665	1.495942	0.086455	1.000000	1.000
18	GOBP_RESPONSE_TO_NUTRIENT	GO:0007584	5	0.667798	1.480369	0.120000	1.000000	1.000
19	GOBP_RAS_PROTEIN_SIGNAL_TRANSDUCTION	GO:0007265	5	0.674900	1.465923	0.115068	1.000000	1.000
20	GOBP_POSITIVE_REGULATION_OF_DEFENSE_RESPONSE	GO:0031349	5	0.658415	1.462696	0.089337	1.000000	1.000
21	GOBP_POSITIVE_REGULATION_OF_CELL_GROWTH	GO:0030307	6	0.605973	1.452327	0.090643	1.000000	1.000
22	GOBP_IN_UTERO_EMBRYONIC_DEVELOPMENT	GO:0001701	8	0.536433	1.440167	0.087948	1.000000	1.000
23	GOBP_REPRODUCTION	GO:0000003	18	0.405538	1.409598	0.092105	1.000000	1.000
24	GOBP_REGULATION_OF_BINDING	GO:0051098	11	0.484832	1.393095	0.097902	1.000000	1.000
25	GOBP_PEPTIDE_METABOLIC_PROCESS	GO:0006518	11	0.475329	1.390729	0.082508	1.000000	1.000
26	GOBP_LONG_TERM_SYNAPTIC_DEPRESSION	GO:0060292	5	0.632276	1.390187	0.139276	1.000000	1.000
27	REACTOME_SIGNALING_BY_RECEPTOR_TYROSINE_KINASES	R-RNO-9006934	26	0.367065	1.378060	0.057692	1.000000	1.000
28	GOBP_RESPONSE_TO_CYTOKINE	GO:0034097	23	0.372673	1.377423	0.069565	1.000000	1.000
29	GOBP_SMALL_GTPASE_MEDIATED_SIGNAL_TRANSDUCTION	GO:0007264	8	0.519251	1.348391	0.108696	1.000000	1.000
30	GOBP_DEVELOPMENTAL_CELL_GROWTH	GO:0048588	5	0.619809	1.346352	0.156069	1.000000	1.000
31	GOBP_REGULATION_OF_CARBOHYDRATE_BIOSYNTHETIC_P...	GO:0043255	5	0.594394	1.328837	0.168022	1.000000	1.000
32	GOBP_GLUCOSE_METABOLIC_PROCESS	GO:0006006	6	0.548583	1.312218	0.157738	1.000000	1.000
33	GOBP_CARBOHYDRATE_BIOSYNTHETIC_PROCESS	GO:0016051	5	0.594394	1.308581	0.200000	1.000000	1.000
34	GOBP_POSITIVE_REGULATION_OF_SMOOTH_MUSCLE_CELL...	GO:0048661	5	0.593711	1.304581	0.167614	1.000000	1.000
35	GOBP_POSITIVE_REGULATION_OF_TRANSMEMBRANE_TRAN...	GO:0034764	7	0.513621	1.298830	0.136232	1.000000	1.000
36	GOBP_MULTI_ORGANISM_PROCESS	GO:0051704	16	0.385276	1.296868	0.107807	1.000000	1.000
37	GOBP_CARBOHYDRATE_METABOLIC_PROCESS	GO:0005975	9	0.477514	1.294860	0.157556	1.000000	1.000
38	REACTOME_DEATH_RECEPTOR_SIGNALLING	R-RNO-73887	5	0.592523	1.286264	0.195402	1.000000	1.000
39	GOBP_CELLULAR_AMIDE_METABOLIC_PROCESS	GO:0043603	12	0.420567	1.285013	0.150538	1.000000	1.000
40	GOBP_REGULATION_OF_EXTRINSIC_APOPTOTIC_SIGNALI...	GO:2001236	6	0.544395	1.283536	0.178470	1.000000	1.000

Continuation of Table **S9**

	NAME	ID	SIZE	ES	NES	NOM.p.val	FDR.q.val	FWER.p.val
41	GOBP_REGULATION_OF_CARBOHYDRATE_METABOLIC_PROCESS	GO:0006109	5	0.594394	1.282522	0.172414	1.000000	1.000
42	GOBP_MONOSACCHARIDE_METABOLIC_PROCESS	GO:0005996	6	0.548583	1.282405	0.163793	1.000000	1.000
43	GOBP_POSITIVE_REGULATION_OF_GROWTH	GO:0045927	9	0.464107	1.278091	0.174757	0.998510	1.000
44	GOBP_POSITIVE_REGULATION_OF_CELL_CELL_ADHESION	GO:0022409	5	0.575633	1.276241	0.186667	0.983010	1.000
45	GOBP_NEGATIVE_REGULATION_OF_BINDING	GO:0051100	6	0.546279	1.275935	0.173789	0.962643	1.000
46	GOBP_POSITIVE_REGULATION_OF_CYTOKINE_PRODUCTION	GO:0001819	7	0.532139	1.271587	0.180473	0.956415	1.000
47	REACTOME_POST_TRANSLATIONAL_PROTEIN_MODIFICATION	R-RNO-597592	7	0.509963	1.270654	0.176966	0.939635	1.000
48	REACTOME_TRANSCRIPTIONAL_REGULATION_OF_WHITE_A...	R-RNO-381340	6	0.547506	1.267404	0.187683	0.931890	1.000
49	HALLMARK_KRAS_SIGNALING_UP	M5953	6	0.536219	1.262271	0.191950	0.930447	1.000
50	GOBP_MEMBRANE_PROTEIN_PROTEOLYSIS	GO:0033619	6	0.534891	1.254472	0.221212	0.937499	1.000
51	GOBP_REGULATION_OF_CELLULAR_COMPONENT_SIZE	GO:0032535	7	0.502345	1.252849	0.183544	0.925194	1.000
52	GOBP_PATTERN_SPECIFICATION_PROCESS	GO:0007389	6	0.534629	1.242617	0.205797	0.941839	1.000
53	GOBP_REGIONALIZATION	GO:0003002	6	0.534629	1.238482	0.183381	0.936612	1.000
54	GOBP_PROTEOLYSIS	GO:0006508	17	0.368615	1.236575	0.175781	0.925322	1.000
55	REACTOME_NGF_STIMULATED_TRANSCRIPTION	R-RNO-9031628	9	0.441279	1.236553	0.196078	0.908865	1.000
56	GOBP_POSITIVE_REGULATION_OF_LEUKOCYTE_CELL_CEL...	GO:1903039	5	0.575633	1.234217	0.223757	0.900205	1.000
57	GOBP_NEGATIVE_REGULATION_OF_CYSTEINE_TYPE_ENDO...	GO:2000117	5	0.573224	1.229107	0.228986	0.900715	1.000
58	GOBP_DEFENSE_RESPONSE	GO:0006952	19	0.361430	1.224563	0.155462	0.898626	1.000
59	HALLMARK_PI3K_AKT_MTOR_SIGNALING	M5923	6	0.523310	1.214547	0.253687	0.914960	1.000
60	GOBP_TEMPERATURE_HOMEOSTASIS	GO:0001659	5	0.564242	1.214039	0.221607	0.901204	1.000
61	REACTOME_CYTOKINE_SIGNALING_IN_IMMUNE_SYSTEM	R-RNO-1280215	21	0.356403	1.212873	0.199095	0.890125	1.000
62	GOBP_NEGATIVE_REGULATION_OF_MOLECULAR_FUNCTION	GO:0044092	24	0.324624	1.206548	0.182266	0.894735	1.000
63	GOBP_NEGATIVE_REGULATION_OF_PROTEOLYSIS	GO:0045861	8	0.464175	1.204284	0.236760	0.888220	1.000
64	GOBP_REGULATION_OF_PROTEIN_LOCALIZATION_TO_MEM...	GO:1905475	7	0.487920	1.202228	0.240356	0.881018	1.000
65	GOBP_RESPONSE_TO_CADMIUM_ION	GO:0046686	6	0.520517	1.202068	0.236025	0.868127	1.000
66	GOBP_REGULATION_OF_PROTEIN_BINDING	GO:0043393	7	0.479152	1.201862	0.216463	0.855745	1.000
67	GOBP_LEUKOCYTE_CELL_CELL_ADHESION	GO:0007159	6	0.507905	1.201333	0.230114	0.844824	1.000
68	GOBP_REGULATION_OF_CELL_DIFFERENTIATION	GO:0045595	36	0.290006	1.179317	0.181347	0.900187	1.000
69	GOBP_CELLULAR_COMPONENT_DISASSEMBLY	GO:0022411	6	0.498423	1.172343	0.259036	0.910011	1.000
70	GOBP_POSITIVE_REGULATION_OF_GENE_EXPRESSION	GO:0010628	16	0.360265	1.169380	0.214022	0.906714	1.000
71	GOBP_RESPONSE_TO_LIGHT_STIMULUS	GO:0009416	11	0.394086	1.168322	0.234043	0.897225	1.000
72	GOBP_CELLULAR_CARBOHYDRATE_METABOLIC_PROCESS	GO:0044262	5	0.528302	1.159699	0.262599	0.911457	1.000
73	GOBP_REGULATION_OF_DEVELOPMENTAL_GROWTH	GO:0048638	9	0.425354	1.156867	0.235880	0.907692	1.000
74	GOBP_EMBRYONIC_ORGAN_DEVELOPMENT	GO:0048568	7	0.458922	1.153741	0.265244	0.904990	1.000
75	GOBP_POSITIVE_REGULATION_OF_DEVELOPMENTAL_GROWTH	GO:0048639	8	0.439892	1.146351	0.248322	0.915290	1.000
76	REACTOME_ESTROGEN_DEPENDENT_NUCLEAR_EVENTS_DOW...	R-RNO-963463	5	0.514740	1.139324	0.267516	0.923706	1.000
77	GOBP_POSITIVE_REGULATION_OF_IMMUNE_SYSTEM_PROCESS	GO:0002684	18	0.330097	1.128884	0.273092	0.945679	1.000
78	GOBP_NEGATIVE_REGULATION_OF_SYNAPTIC_TRANSMISSION	GO:0050805	6	0.463415	1.121776	0.262346	0.956663	1.000
79	GOBP_RESPONSE_TO_RADIATION	GO:0009314	13	0.348463	1.120541	0.270073	0.948973	1.000
80	REACTOME_EXTRA_NUCLEAR_ESTROGEN_SIGNALING	R-RNO-900939	7	0.444811	1.118851	0.352201	0.942883	1.000
81	GOBP_POSITIVE_REGULATION_OF_CELL_DIFFERENTIATION	GO:0045597	27	0.296063	1.116553	0.288991	0.938608	1.000
82	GOBP_INOSITOL_LIPID_MEDIATED_SIGNALING	GO:0048017	8	0.415065	1.114769	0.303797	0.932846	1.000
83	GOBP_REGULATION_OF_NEURON_PROJECTION_DEVELOPMENT	GO:0010975	16	0.341137	1.110784	0.298387	0.933959	1.000

Continuation of Table **S9**

	NAME	ID	SIZE	ES	NES	NOM.p.val	FDR.q.val	FWER.p.val
84	GOBP_ORGAN_GROWTH	GO:0035265	5	0.500075	1.109664	0.287293	0.926466	1.000
85	GOBP_REGULATION_OF_CELL_DEVELOPMENT	GO:0060284	15	0.351812	1.108910	0.319703	0.917965	1.000
86	GOBP_AMYLOID_PRECURSOR_PROTEIN_CATABOLIC_PROCESS	GO:0042987	5	0.504594	1.107742	0.310924	0.910976	1.000
87	GOBP_AMYLOID_BETA_METABOLIC_PROCESS	GO:0050435	5	0.504594	1.098848	0.321127	0.927831	1.000
88	HALLMARK_APOPTOSIS	M5902	6	0.470030	1.097675	0.275964	0.920801	1.000
89	GOBP_NEGATIVE_REGULATION_OF_PROTEIN_METABOLIC_...	GO:0051248	20	0.309291	1.092760	0.296943	0.925408	1.000
90	GOBP_AMYLOID_PRECURSOR_PROTEIN_METABOLIC_PROCESS	GO:0042982	5	0.504594	1.091440	0.331288	0.919090	1.000
91	GOBP_GLIAL_CELL_DIFFERENTIATION	GO:0010001	10	0.381631	1.088551	0.302181	0.918071	1.000
92	GOBP_REGULATION_OF_NEURON_DIFFERENTIATION	GO:0045664	5	0.502021	1.081772	0.339474	0.928593	1.000
93	GOBP_ENZYME_LINKED_RECEPTOR_PROTEIN_SIGNALING_...	GO:0007167	24	0.294322	1.079026	0.357143	0.927382	1.000
94	GOBP_MATERNAL_PROCESS_INVOLVED_IN_FEMALE_PREGN...	GO:0060135	5	0.493982	1.076724	0.324022	0.925090	1.000
95	GOBP_POSITIVE_REGULATION_OF_BINDING	GO:0051099	6	0.462931	1.073787	0.355623	0.924552	1.000
96	GOBP_REGULATION_OF_ORGAN_GROWTH	GO:0046620	5	0.500075	1.073762	0.345946	0.915146	1.000
97	GOBP_GLIOGENESIS	GO:0042063	12	0.355263	1.070045	0.318033	0.917070	1.000
98	GOBP_MUSCLE_TISSUE_DEVELOPMENT	GO:0060537	11	0.355426	1.061522	0.385382	0.932426	1.000
99	GOBP_RESPONSE_TO_NERVE_GROWTH_FACTOR	GO:1990089	7	0.421744	1.057429	0.346749	0.935413	1.000
100	GOBP_POSITIVE_REGULATION_OF_PRI_MIRNA_TRANSCRI...	GO:1902895	7	0.419753	1.052141	0.375723	0.942053	1.000
101	GOBP_PROTEIN_KINASE_B_SIGNALING	GO:0043491	6	0.432191	1.049199	0.378698	0.941995	1.000
102	GOBP_PRI_MIRNA_TRANSCRIPTION_BY_RNA_POLYMERASE_II	GO:0061614	7	0.419753	1.042930	0.378698	0.952045	1.000
103	GOBP_EMBRYO_DEVELOPMENT	GO:0009790	14	0.330697	1.041867	0.402135	0.946044	1.000
104	GOBP_REGULATION_OF_NERVOUS_SYSTEM_DEVELOPMENT	GO:0051960	15	0.322037	1.034656	0.389286	0.959105	1.000
105	GOBP_EMBRYO_DEVELOPMENT_ENDING_IN_BIRTH_OR_EGG...	GO:0009792	9	0.386860	1.031723	0.385135	0.958757	1.000
106	GOBP_RESPONSE_TO_UV	GO:0009411	5	0.484419	1.030987	0.395543	0.952226	1.000
107	GOBP_DEVELOPMENTAL_PROCESS_INVOLVED_IN_REPRODU...	GO:0003006	11	0.353857	1.027703	0.411765	0.953442	1.000
108	GOBP_REGULATION_OF_ANION_TRANSMEMBRANE_TRANSPORT	GO:1903959	5	0.464476	1.027488	0.372928	0.945357	1.000
109	GOBP_POSTSYNAPSE_ORGANIZATION	GO:0099173	10	0.352042	1.027474	0.358108	0.936830	1.000
110	GOBP_DENDRITIC_SPINE_MORPHOGENESIS	GO:0060997	5	0.460004	1.026925	0.381215	0.930023	1.000
111	GOBP_FC_RECEPTOR_SIGNALING_PATHWAY	GO:0038093	8	0.383509	1.026480	0.410828	0.922961	1.000
112	GOBP_MULTICELLULAR_ORGANISM_REPRODUCTION	GO:0032504	12	0.345784	1.026158	0.390728	0.915632	1.000
113	GOBP_MONOCARBOXYLIC_ACID_METABOLIC_PROCESS	GO:0032787	6	0.430262	1.022932	0.389041	0.917172	1.000
114	GOBP_TRANSMEMBRANE_RECEPTOR_PROTEIN_TYROSINE_K...	GO:0007169	19	0.302160	1.021616	0.397541	0.913085	1.000
115	GOBP_POSITIVE_REGULATION_OF_ION_TRANSPORT	GO:0043270	17	0.303428	1.018016	0.430712	0.915224	1.000
116	GOBP_REGULATION_OF_CELLULAR_AMIDE_METABOLIC_PR...	GO:0034248	9	0.380688	1.012514	0.381579	0.923090	1.000
117	GOBP_DENDRITIC_SPINE_DEVELOPMENT	GO:0060996	6	0.437552	1.012193	0.408669	0.916264	1.000
118	GOBP_CELLULAR_RESPONSE_TO_CADMIUM_ION	GO:0071276	5	0.458766	1.009430	0.397101	0.916537	1.000
119	GOBP_NEGATIVE_REGULATION_OF_LOCOMOTION	GO:0040013	5	0.468553	1.007406	0.399417	0.914625	1.000
120	GOBP_NEGATIVE_REGULATION_OF_CELL_DEATH	GO:0060548	22	0.279246	1.007133	0.452000	0.907908	1.000
121	REACTOME_SIGNALING_BY_NTRKS	R-RNO-166520	18	0.300663	1.006880	0.429150	0.901166	1.000
122	GOBP_REGULATION_OF_CELL_CYCLE_PROCESS	GO:0010564	5	0.464060	1.005042	0.406162	0.898734	1.000
123	GOBP_GLAND_DEVELOPMENT	GO:0048732	7	0.392263	1.004778	0.436047	0.892251	1.000
124	GOBP_REGULATION_OF_POSTSYNAPSE_ORGANIZATION	GO:0099175	6	0.414950	1.002934	0.426332	0.890159	1.000
125	REACTOME_NUCLEAR_EVENTS_KINASE_AND_TRANSCRIPTI...	R-RNO-198725	11	0.336360	1.000049	0.441472	0.891347	1.000
126	GOBP_REGULATION_OF_CELL_CYCLE	GO:0051726	13	0.324016	0.998656	0.441860	0.887933	1.000

Continuation of Table **S9**

	NAME	ID	SIZE	ES	NES	NOM.p.val	FDR.q.val	FWER.p.val
127	GOBP_PERIPHERAL_NERVOUS_SYSTEM_DEVELOPMENT	GO:0007422	9	0.369745	0.995337	0.450658	0.890587	1.000
128	GOBP_APOPTOTIC_MITOCHONDRIAL_CHANGES	GO:0008637	6	0.417116	0.995061	0.426791	0.884472	1.000
129	GOBP_GAMETE_GENERATION	GO:0007276	9	0.353893	0.993465	0.457516	0.881802	1.000
130	REACTOME_EXTRACELLULAR_MATRIX_ORGANIZATION	R-RNO-1474244	5	0.449152	0.991652	0.428571	0.879964	1.000
131	GOBP_IMMUNE_RESPONSE_REGULATING_SIGNALING_PATHWAY	GO:000276	8	0.383509	0.989370	0.413907	0.879389	1.000
132	GOBP_HEART_DEVELOPMENT	GO:0007507	6	0.427288	0.986574	0.427245	0.879705	1.000
133	GOBP_REGULATION_OF_CELLULAR_CATABOLIC_PROCESS	GO:0031329	10	0.340760	0.977764	0.454545	0.895431	1.000
134	REACTOME_ESR_MEDIATED_SIGNALING	R-RNO-8939211	8	0.363962	0.977721	0.480519	0.888962	1.000
135	GOBP_CELL_MIGRATION	GO:0016477	22	0.265978	0.976290	0.489362	0.886158	1.000
136	GOBP_POSITIVE_REGULATION_OF_ANION_TRANSPORT	GO:1903793	9	0.354083	0.972301	0.438775	0.890388	1.000
137	GOBP_CARDIAC_MUSCLE_TISSUE_DEVELOPMENT	GO:0048738	6	0.427288	0.972158	0.454810	0.884296	1.000
138	REACTOME_SIGNALING_BY_NUCLEAR_RECEPTORS	R-RNO-9006931	8	0.363962	0.970168	0.462025	0.883506	1.000
139	GOBP_NEGATIVE_REGULATION_OF_RESPONSE_TO_EXTERN...	GO:0032102	5	0.440093	0.966406	0.449315	0.886825	1.000
140	GOBP_REGULATION_OF_GLIOGENESIS	GO:0014013	5	0.445783	0.965524	0.465116	0.882733	1.000
141	GOBP_RESPONSE_TO_ENDOGENOUS_STIMULUS	GO:0009719	34	0.248457	0.964668	0.550898	0.878647	1.000
142	GOBP_POSITIVE_REGULATION_OF_GLIOGENESIS	GO:0014015	5	0.445783	0.963776	0.469444	0.874774	1.000
143	GOBP_NEGATIVE_REGULATION_OF_MULTICELLULAR_ORGA...	GO:0051241	15	0.294671	0.962372	0.507634	0.872311	1.000
144	GOBP_NEGATIVE_REGULATION_OF_CATALYTIC_ACTIVITY	GO:0043086	17	0.277438	0.962310	0.500000	0.866442	1.000
145	GOBP_DEVELOPMENTAL_GROWTH	GO:0048589	10	0.344285	0.959631	0.531987	0.867501	1.000
146	GOBP_T_CELL_ACTIVATION	GO:0042110	8	0.374121	0.954632	0.495177	0.874260	1.000
147	GOBP_REGULATION_OF_INTRACELLULAR_SIGNAL_TRANSD...	GO:1902531	21	0.259226	0.943041	0.545852	0.896659	1.000
148	GOBP_NEGATIVE_REGULATION_OF_HYDROLASE_ACTIVITY	GO:0051346	11	0.318842	0.942035	0.526132	0.893067	1.000
149	GOBP_REGULATION_OF_CELL_SIZE	GO:0008361	5	0.440325	0.941803	0.492997	0.887749	1.000
150	GOBP_CARBOHYDRATE_DERIVATIVE_METABOLIC_PROCESS	GO:1901135	8	0.350714	0.931347	0.542587	0.909191	1.000
151	GOBP_REGULATION_OF_MULTICELLULAR_ORGANISMAL_DE...	GO:2000026	31	0.230075	0.922797	0.567416	0.925144	1.000
152	GOBP_RESPONSE_TO_NICOTINE	GO:0035094	7	0.370643	0.909068	0.545181	0.954898	1.000
153	GOBP_IMPORT_INTO_CELL	GO:0098657	6	0.379374	0.907303	0.532213	0.953187	1.000
154	GOBP_LOCOMOTION	GO:0040011	31	0.236258	0.906797	0.594872	0.948163	1.000
155	GOBP_ENDOCRINE_SYSTEM_DEVELOPMENT	GO:0035270	6	0.388898	0.902097	0.579288	0.954758	1.000
156	REACTOME_TOLL_LIKE_RECEPTOR_9_TLR9_CASCADE	R-RNO-168138	8	0.337500	0.896703	0.549689	0.962957	1.000
157	GOBP_POSITIVE_REGULATION_OF_INTRACELLULAR_SIGN...	GO:1902533	14	0.277580	0.891259	0.595420	0.970657	1.000
158	GOBP_POSITIVE_REGULATION_OF_NERVOUS_SYSTEM_DEV...	GO:0051962	12	0.291392	0.888685	0.615385	0.970968	1.000
159	GOBP_REGULATION_OF_LONG_TERM_SYNAPTIC_POTENTIA...	GO:1900271	6	0.395831	0.887837	0.569697	0.967162	1.000
160	GOBP_POSITIVE_REGULATION_OF_CELL_DEVELOPMENT	GO:0010720	12	0.291392	0.887195	0.577358	0.962792	1.000
161	GOBP_MORPHOGENESIS_OF_AN_EPITHELIUM	GO:0002009	6	0.377364	0.885672	0.546828	0.960547	1.000
162	GOBP_REGULATION_OF_CELL_DEATH	GO:0010941	34	0.218658	0.885106	0.702857	0.956140	1.000
163	GOBP_CELL_CYCLE	GO:0007049	21	0.240702	0.876333	0.660944	0.972277	1.000
164	REACTOME_TOLL_LIKE_RECEPTOR_TLR1_TLR2_CASCADE	R-RNO-168179	8	0.337500	0.876181	0.604317	0.966671	1.000
165	REACTOME_TOLL_LIKE_RECEPTOR_CASCADES	R-RNO-168898	8	0.337500	0.874003	0.595469	0.966368	1.000
166	GOBP_ACTIVATION_OF_PROTEIN_KINASE_ACTIVITY	GO:0032147	10	0.302661	0.866458	0.660131	0.979557	1.000
167	GOBP_POSITIVE_REGULATION_OF_DEVELOPMENTAL_PROCESS	GO:0051094	32	0.219987	0.866439	0.677596	0.973791	1.000
168	GOBP_EPITHELIUM_DEVELOPMENT	GO:0060429	15	0.268780	0.864150	0.656604	0.973216	1.000
169	REACTOME_MYD88_INDEPENDENT_TLR4_CASCADE	R-RNO-166166	8	0.337500	0.860872	0.634375	0.975466	1.000

Continuation of Table **S9**

	NAME	ID	SIZE	ES	NES	NOM.p.val	FDR.q.val	FWER.p.val
170	HALLMARK_UV_RESPONSE_UP	M5941	5	0.380834	0.857676	0.581538	0.977320	1.000
171	GOBP_REGULATION_OF_FAT_CELL_DIFFERENTIATION	GO:0045598	6	0.360765	0.857424	0.622590	0.972265	1.000
172	GOBP_POSITIVE_REGULATION_OF_NEUROGENESIS	GO:0050769	12	0.291392	0.857152	0.657895	0.967351	1.000
173	GOBP_REGULATION_OF_PEPTIDASE_ACTIVITY	GO:0052547	10	0.300129	0.856048	0.635783	0.964313	1.000
174	GOBP_REGULATION_OF_PROTEIN_LOCALIZATION	GO:0032880	18	0.241293	0.854419	0.687243	0.962496	1.000
175	GOBP_RESPONSE_TO_ACID_CHEMICAL	GO:0001101	6	0.353659	0.853122	0.615152	0.959845	1.000
176	GOBP_SMOOTH_MUSCLE_CELL_PROLIFERATION	GO:0048659	6	0.361882	0.849872	0.628895	0.962135	1.000
177	GOBP_CELL_GROWTH	GO:0016049	10	0.304418	0.844790	0.655063	0.968732	1.000
178	REACTOME_TRANSCRIPTIONAL_REGULATION_BY_TP53	R-RNO-3700989	6	0.362333	0.839633	0.634730	0.976080	1.000
179	GOBP_MUSCLE_ORGAN_DEVELOPMENT	GO:0007517	9	0.321487	0.837925	0.656463	0.974391	1.000
180	GOBP_REGULATION_OF_CELLULAR_PROTEIN_LOCALIZATION	GO:1903827	12	0.274621	0.835583	0.685512	0.974267	1.000
181	REACTOME_SENESCENCE_ASSOCIATED_SECRETORY_PHENO...	R-RNO-2559582	6	0.353990	0.830590	0.671429	0.980248	1.000
182	GOBP_REGULATION_OF_IMMUNE_SYSTEM_PROCESS	GO:0002682	21	0.236060	0.829946	0.733945	0.976614	1.000
183	GOBP_REGULATION_OF_NEUROGENESIS	GO:0050767	14	0.262496	0.828976	0.676976	0.973571	1.000
184	GOBP_AGING	GO:0007568	13	0.273130	0.828297	0.746032	0.969825	1.000
185	GOBP_ANION_TRANSMEMBRANE_TRANSPORT	GO:0098656	7	0.331314	0.827087	0.667598	0.967110	1.000
186	REACTOME_CELLULAR_SENESCENCE	R-RNO-2559583	6	0.353990	0.822443	0.696049	0.971927	1.000
187	GOBP_MUSCLE_CELL_DEVELOPMENT	GO:0055001	5	0.373862	0.818293	0.653959	0.976029	1.000
188	REACTOME_EPH_EPHRIN_SIGNALING	R-RNO-268233	6	0.344023	0.817183	0.664671	0.973255	1.000
189	GOBP_ACTIVATION_OF_IMMUNE_RESPONSE	GO:0002253	5	0.385484	0.816883	0.676471	0.968796	1.000
190	GOBP_REGULATION_OF_SMALL_MOLECULE_METABOLIC_PR...	GO:0062012	9	0.308172	0.814057	0.694245	0.969794	1.000
191	GOBP_NEGATIVE_REGULATION_OF_CELL_DEVELOPMENT	GO:0010721	6	0.343233	0.802302	0.676829	0.989877	1.000
192	GOBP_REGULATION_OF_PROTEIN_SERINE_THREONINE_KI...	GO:0071900	12	0.264284	0.798962	0.738182	0.991716	1.000
193	GOBP_LEUKOCYTE_MIGRATION	GO:0050900	6	0.334715	0.789060	0.725948	1.000000	1.000
194	GOBP_CELLULAR_RESPONSE_TO_ABIOTIC_STIMULUS	GO:0071214	7	0.318873	0.785546	0.737374	1.000000	1.000
195	GOBP_MEMORY	GO:0007613	13	0.256270	0.780718	0.776557	1.000000	1.000
196	GOBP_IMMUNE_EFFECTOR_PROCESS	GO:0002252	11	0.278438	0.780471	0.758278	1.000000	1.000
197	GOBP_RESPONSE_TO_CALCIUM_ION	GO:0051592	6	0.334890	0.778812	0.719547	1.000000	1.000
198	REACTOME_INTERLEUKIN_17_SIGNALING	R-RNO-448424	6	0.329268	0.778653	0.698225	1.000000	1.000
199	GOBP_CELL_CELL_JUNCTION_ORGANIZATION	GO:0045216	6	0.339838	0.774717	0.742857	1.000000	1.000
200	GOBP_POSITIVE_REGULATION_OF_CELL_DEATH	GO:0010942	19	0.225420	0.774498	0.814655	1.000000	1.000
201	GOBP_NEUROTROPHIN_SIGNALING_PATHWAY	GO:0038179	6	0.322305	0.772804	0.743516	0.998424	1.000
202	GOBP_DENDRITE_MORPHOGENESIS	GO:0048813	7	0.306055	0.770433	0.765766	0.998015	1.000
203	GOBP_NEUROTROPHIN_TRK_RECEPTOR_SIGNALING_PATHWAY	GO:0048011	6	0.322305	0.769894	0.761147	0.994084	1.000
204	GOBP_POSITIVE_REGULATION_OF_ESTABLISHMENT_OF_P...	GO:1904951	5	0.352768	0.764867	0.713018	0.999094	1.000
205	GOBP_ACTIN_FILAMENT_BASED_PROCESS	GO:0030029	7	0.312456	0.760639	0.776025	1.000000	1.000
206	REACTOME_VESICLE_MEDIATED_TRANSPORT	R-RNO-5653656	5	0.331088	0.758834	0.777473	1.000000	1.000
207	GOBP_RESPONSE_TO_OXIDATIVE_STRESS	GO:0006979	8	0.282711	0.757827	0.735385	0.997668	1.000
208	GOBP_REGULATION_OF_WNT_SIGNALING_PATHWAY	GO:0030111	5	0.337349	0.757000	0.783562	0.994470	1.000
209	GOBP_REGULATION_OF_PROTEOLYSIS	GO:0030162	12	0.253610	0.756228	0.819549	0.991016	1.000
210	GOBP_RESPONSE_TO_METAL_ION	GO:0010038	15	0.231584	0.751587	0.836957	0.994957	1.000
211	GOBP_CELLULAR_RESPONSE_TO_EXTERNAL_STIMULUS	GO:0071496	10	0.259658	0.749930	0.824324	0.992981	1.000
212	GOBP_RESPONSE_TO_NITROGEN_COMPOUND	GO:1901698	35	0.186702	0.748503	0.901099	0.990646	1.000

Continuation of Table **S9**

	NAME	ID	SIZE	ES	NES	NOM.p.val	FDR.q.val	FWER.p.val
213	GOBP_RESPONSE_TO_REACTIVE_OXYGEN_SPECIES	GO:0000302	8	0.282711	0.746249	0.809091	0.990023	1.000
214	GOBP_CELL_POPULATION_PROLIFERATION	GO:0008283	32	0.196266	0.741401	0.896552	0.993756	1.000
215	GOBP_POSITIVE_REGULATION_OF_MULTICELLULAR_ORGA...	GO:0051240	32	0.184770	0.737359	0.887097	0.996138	1.000
216	GOBP_REGULATION_OF_IMMUNE_RESPONSE	GO:0050776	12	0.244894	0.735095	0.851852	0.995413	1.000
217	GOBP_RESPONSE_TO_ABIOTIC_STIMULUS	GO:0009628	27	0.189258	0.731664	0.897297	0.996344	1.000
218	GOBP_REGULATION_OF_LYMPHOCYTE_ACTIVATION	GO:0051249	5	0.343593	0.731175	0.790831	0.992473	1.000
219	GOBP_REGULATION_OF_GROWTH	GO:0040008	13	0.233882	0.728184	0.868526	0.992754	1.000
220	REACTOME_MEMBRANE_TRAFFICKING	R-RNO-199991	5	0.331088	0.726834	0.767313	0.990679	1.000
221	REACTOME_PTEN_REGULATION	R-RNO-6807070	5	0.325301	0.724388	0.800539	0.990091	1.000
222	GOBP_ERK1_AND_ERK2_CASCADE	GO:0070371	5	0.323153	0.723091	0.831522	0.987811	1.000
223	GOBP_REGULATION_OF_AXONOGENESIS	GO:0050770	7	0.297853	0.719440	0.822430	0.989216	1.000
224	GOBP_PROTEIN_LOCALIZATION_TO_PLASMA_MEMBRANE	GO:0072659	7	0.294941	0.718299	0.834375	0.986582	1.000
225	GOBP_TISSUE_HOMEOSTASIS	GO:0001894	5	0.318826	0.717484	0.819945	0.983445	1.000
226	GOBP_REGULATION_OF_VASCULAR_ASSOCIATED_SMOOTH_...	GO:1904705	5	0.315843	0.715058	0.824713	0.982620	1.000
227	GOBP_NEURON_PROJECTION_ORGANIZATION	GO:0106027	8	0.271581	0.714967	0.846645	0.978442	1.000
228	GOBP_REGULATION_OF_CYTOSKELETON_ORGANIZATION	GO:0051493	5	0.325301	0.713799	0.830946	0.975974	1.000
229	GOBP_CELLULAR_RESPONSE_TO_REACTIVE_OXYGEN_SPECIES	GO:0034614	7	0.287537	0.711635	0.833333	0.974948	1.000
230	GOBP_PEPTIDE_BIOSYNTHETIC_PROCESS	GO:0043043	6	0.293030	0.706216	0.842541	0.978914	1.000
231	REACTOME_MAPK_TARGETS_NUCLEAR_EVENTS_MEDIATED_...	R-HSA-450282	5	0.325301	0.705175	0.854795	0.976224	1.000
232	GOBP_POSITIVE_REGULATION_OF_PHOSPHATIDYLINOSIT...	GO:0014068	5	0.329056	0.698898	0.806630	0.980674	1.000
233	GOBP_MUSCLE_CELL_PROLIFERATION	GO:0033002	8	0.271651	0.698791	0.864687	0.976715	1.000
234	GOBP_CELLULAR_RESPONSE_TO_CHEMICAL_STRESS	GO:0062197	7	0.287537	0.698525	0.855346	0.972939	1.000
235	GOBP_LYMPHOCYTE_ACTIVATION	GO:0046649	10	0.249493	0.697810	0.863192	0.969648	1.000
236	GOBP_AMIDE_BIOSYNTHETIC_PROCESS	GO:0043604	6	0.293030	0.696169	0.865103	0.967746	1.000
237	GOBP_PHOSPHATIDYLINOSITOL_3_KINASE_SIGNALING	GO:0014065	7	0.281953	0.691164	0.882522	0.970091	1.000
238	GOBP_POSITIVE_REGULATION_OF_NEURON_DEATH	GO:1901216	5	0.311193	0.686564	0.868715	0.971925	1.000
239	GOBP_ASSOCIATIVE_LEARNING	GO:0008306	7	0.280816	0.683952	0.876471	0.971023	1.000
240	GOBP_POSITIVE_REGULATION_OF_AXONOGENESIS	GO:0050772	5	0.315181	0.683099	0.849162	0.968048	1.000
241	HALLMARK_TNFA_SIGNALING_VIA_NFKB	M5890	14	0.221946	0.682721	0.892116	0.964521	1.000
242	GOBP_INTRINSIC_APOPTOTIC_SIGNALING_PATHWAY	GO:0097193	5	0.320011	0.682079	0.852410	0.961402	1.000
243	GOBP_MYELOID_LEUKOCYTE_ACTIVATION	GO:0002274	8	0.263431	0.673795	0.901899	0.967876	1.000
244	GOBP_MITOCHONDRION_ORGANIZATION	GO:0007005	7	0.270158	0.673448	0.859238	0.964376	1.000
245	REACTOME_DEVELOPMENTAL_BIOLOGY	R-RNO-1266738	22	0.182840	0.655850	0.972477	0.980329	1.000
246	GOBP_REGULATION_OF_PROTEIN_STABILITY	GO:0031647	5	0.295000	0.652711	0.929203	0.979515	1.000
247	GOBP_POSITIVE_REGULATION_OF_TRANSPORT	GO:0051050	26	0.173611	0.650317	0.945000	0.978124	1.000
248	GOBP_SKELETAL_MUSCLE_ORGAN_DEVELOPMENT	GO:0060538	5	0.297959	0.640016	0.912698	0.983968	1.000
249	GOBP_POSITIVE_REGULATION_OF_CELL_ADHESION	GO:0045785	7	0.261634	0.638870	0.921875	0.981153	1.000
250	GOBP_NEGATIVE_REGULATION_OF_TRANSMEMBRANE_TRAN...	GO:0034763	6	0.269747	0.632197	0.909366	0.983306	1.000
251	GOBP_REGULATION_OF_LIPID_METABOLIC_PROCESS	GO:0019216	7	0.242299	0.607653	0.961905	0.998616	1.000
252	GOBP_EPHRIN_RECEPTOR_SIGNALING_PATHWAY	GO:0048013	5	0.276744	0.605327	0.943662	0.996282	1.000
253	REACTOME_NEUTROPHIL_DEGRANULATION	R-RNO-6798695	6	0.247467	0.587872	0.948012	1.000000	1.000
254	GOBP_POSITIVE_REGULATION_OF_IMMUNE_RESPONSE	GO:0050778	9	0.208628	0.583028	0.958955	1.000000	1.000
255	GOBP_CELL_ACTIVATION_INVOLVED_IN_IMMUNE_RESPONSE	GO:0002263	6	0.247467	0.582866	0.975460	0.997674	1.000

Continuation of Table **S9**

	NAME	ID	SIZE	ES	NES	NOM.p.val	FDR.q.val	FWER.p.val
256	GOBP_MYELOID_LEUKOCYTE_MEDIATED_IMMUNITY	GO:0002444	6	0.247467	0.580452	0.972299	0.995074	1.000
257	GOBP_LEUKOCYTE_MEDIATED_IMMUNITY	GO:0002443	8	0.222188	0.572327	0.980519	0.995039	1.000
258	GOBP_PROTEIN_COMPLEX_OLIGOMERIZATION	GO:0051259	7	0.222222	0.552016	0.978593	0.999383	1.000
259	GOBP_ANATOMICAL_STRUCTURE_HOMEOSTASIS	GO:0060249	7	0.222656	0.548169	0.991404	0.996942	1.000
260	GOBP_CELLULAR_RESPONSE_TO_EXTRACELLULAR_STIMULUS	GO:0031668	8	0.202274	0.543000	0.993289	0.994646	1.000
261	REACTOME_ONCOGENIC_MAPK_SIGNALING	R-HSA-6802957	5	0.248600	0.535473	0.994429	0.992668	1.000
262	GOBP_LIPID_METABOLIC_PROCESS	GO:0006629	10	0.166667	0.470092	1.000000	0.998360	1.000
263	GOBP_BIOLOGICAL_PROCESS_INVOLVED_IN_SYMBIOTIC_...	GO:004440	10	-0.179329	-0.434554	0.997063	0.997894	1.000
264	REACTOME_DISEASES_OF_SIGNAL_TRANSDUCTION_BY_GR...	R-HSA-5663202	12	-0.178560	-0.448273	1.000000	0.999064	1.000
265	GOBP_POSITIVE_REGULATION_OF_NEURON_PROJECTION_...	GO:0010976	5	-0.240354	-0.463801	0.990385	0.999698	1.000
266	REACTOME_INNATE_IMMUNE_SYSTEM	R-RNO-168249	16	-0.178249	-0.477495	0.993270	0.999902	1.000
267	REACTOME_CIRCADIAN_CLOCK	R-HSA-400253	5	-0.255756	-0.495929	0.983508	0.998695	1.000
268	GOBP_PROTEIN_CONTAINING_COMPLEX_SUBUNIT_ORGANI...	GO:0043933	15	-0.185927	-0.496951	0.988205	1.000000	1.000
269	GOBP_CIRCULATORY_SYSTEM_DEVELOPMENT	GO:0072359	21	-0.170903	-0.497220	0.992126	1.000000	1.000
270	GOBP_CELL_CELL_SIGNALING_BY_WNT	GO:0198738	7	-0.229118	-0.498419	0.989521	1.000000	1.000
271	GOBP_POSITIVE_REGULATION_OF_HEMOPOIESIS	GO:1903708	7	-0.229569	-0.500749	0.990881	1.000000	1.000
272	GOBP_NEGATIVE_REGULATION_OF_NERVOUS_SYSTEM_DEV...	GO:0051961	5	-0.255329	-0.501313	0.992320	1.000000	1.000
273	GOBP_RESPONSE_TO_TUMOR_NECROSIS_FACTOR	GO:0034612	7	-0.233210	-0.505677	0.991018	1.000000	1.000
274	REACTOME_INFECTIOUS_DISEASE	R-HSA-5663205	12	-0.203185	-0.510681	0.982736	1.000000	1.000
275	GOBP_NEGATIVE_REGULATION_OF_INTRACELLULAR_SIGN...	GO:1902532	7	-0.239241	-0.521006	0.984849	1.000000	1.000
276	GOBP_IMMUNE_SYSTEM_DEVELOPMENT	GO:0002520	17	-0.199439	-0.544372	0.971698	1.000000	1.000
277	GOBP_CELLULAR_LIPID_METABOLIC_PROCESS	GO:0044255	7	-0.250086	-0.545572	0.979167	1.000000	1.000
278	GOBP_RESPONSE_TO_INORGANIC_SUBSTANCE	GO:0010035	19	-0.195007	-0.551509	0.984416	1.000000	1.000
279	GOBP_ANIMAL_ORGAN_MORPHOGENESIS	GO:0009887	12	-0.221411	-0.553618	0.964132	1.000000	1.000
280	GOBP_APOPTOTIC_PROCESS	GO:0006915	35	-0.180580	-0.558051	0.977025	1.000000	1.000
281	REACTOME_CELLULAR_RESPONSES_TO_EXTERNAL_STIMULI	R-RNO-8953897	11	-0.227507	-0.558368	0.956091	1.000000	1.000
282	GOBP_LIPID_BIOSYNTHETIC_PROCESS	GO:0008610	6	-0.270764	-0.560922	0.960177	1.000000	1.000
283	GOBP_REGULATION_OF_DNA_BINDING_TRANSCRIPTION_F...	GO:0051090	16	-0.204344	-0.562120	0.962517	1.000000	1.000
284	GOBP_POSITIVE_REGULATION_OF_LOCOMOTION	GO:0040017	11	-0.232819	-0.563489	0.968706	1.000000	1.000
285	GOBP_REGULATION_OF_LIPID_BIOSYNTHETIC_PROCESS	GO:0046890	6	-0.270764	-0.564983	0.963127	1.000000	1.000
286	GOBP_CYTOSKELETON_ORGANIZATION	GO:0007010	11	-0.228390	-0.565010	0.964626	1.000000	1.000
287	GOBP_NEURON_MIGRATION	GO:0001764	5	-0.300690	-0.581807	0.952756	1.000000	1.000
288	GOBP_NUCLEAR_TRANSPORT	GO:0051169	5	-0.293542	-0.581813	0.960784	1.000000	1.000
289	GGOBP_REGULATION_OF_PHOSPHATIDYLINOSITOL_3_KINA...	GO:0014066	6	-0.281220	-0.584534	0.954887	1.000000	1.000
290	GOBP_REGULATION_OF_PEPTIDE_TRANSPORT	GO:0090087	10	-0.243987	-0.585468	0.935714	1.000000	1.000
291	GOBP_REGULATION_OF_CYSTEINE_TYPE_ENDOPEPTIDASE...	GO:2000116	9	-0.250140	-0.586104	0.937053	1.000000	1.000
292	GOBP_RESPIRATORY_SYSTEM_DEVELOPMENT	GO:0060541	6	-0.283180	-0.589555	0.943164	1.000000	1.000
293	GOBP_MONONUCLEAR_CELL_DIFFERENTIATION	GO:1903131	7	-0.270395	-0.591864	0.956140	1.000000	1.000
294	GOBP_ORGANIC_HYDROXY_COMPOUND_METABOLIC_PROCESS	GO:1901615	8	-0.262872	-0.596172	0.953938	1.000000	1.000
295	GOBP_POSITIVE_REGULATION_OF_CELL_PROJECTION_OR...	GO:0031346	10	-0.247651	-0.596987	0.944915	1.000000	1.000
296	GOBP_NEGATIVE_REGULATION_OF_LIPID_METABOLIC_PR...	GO:0045833	5	-0.308315	-0.601860	0.937405	1.000000	1.000
297	REACTOME_NERVOUS_SYSTEM_DEVELOPMENT	R-RNO-9675108	14	-0.233211	-0.602211	0.956522	1.000000	1.000
298	GOBP_POSITIVE_REGULATION_OF_RESPONSE_TO_EXTERN...	GO:0032103	9	-0.261353	-0.603062	0.943503	1.000000	1.000

Continuation of Table **S9**

	NAME	ID	SIZE	ES	NES	NOM.p.val	FDR.q.val	FWER.p.val
299	REACTOME_INTERFERON_SIGNALING	R-RNO-913531	6	-0.288557	-0.603072	0.927673	1.000000	1.000
300	GOBP_TELENCEPHALON_DEVELOPMENT	GO:0021537	7	-0.278313	-0.603441	0.939063	1.000000	1.000
301	GOBP_POSITIVE_REGULATION_OF_MYELOID_LEUKOCYTE_...	GO:0002763	6	-0.287351	-0.605260	0.934150	1.000000	1.000
302	GOBP_CIRCADIAN_REGULATION_OF_GENE_EXPRESSION	GO:0032922	5	-0.308461	-0.607590	0.944870	1.000000	1.000
303	GOBP_NEGATIVE_REGULATION_OF_SIGNALING	GO:0023057	21	-0.215381	-0.608682	0.948914	1.000000	1.000
304	GOBP_DEFENSE_RESPONSE_TO_OTHER_ORGANISM	GO:0098542	9	-0.260768	-0.609968	0.930496	1.000000	1.000
305	GOBP_CELLULAR_RESPONSE_TO_MOLECULE_OF_BACTERIA...	GO:0071219	8	-0.270911	-0.610336	0.932836	1.000000	1.000
306	REACTOME_LEISHMANIA_INFECTION	R-HSA-9658195	10	-0.257436	-0.611593	0.948718	1.000000	1.000
307	GOBP_CELLULAR_RESPONSE_TO_BIOTIC_STIMULUS	GO:0071216	8	-0.270911	-0.611624	0.930595	1.000000	1.000
308	GOBP_ERBB_SIGNALING_PATHWAY	GO:0038127	5	-0.319923	-0.621744	0.922156	1.000000	1.000
309	GOBP_POSITIVE_REGULATION_OF_MAP_KINASE_ACTIVITY	GO:0043406	6	-0.306670	-0.625931	0.917933	1.000000	1.000
310	GOBP_RESPONSE_TO_EXTRACELLULAR_STIMULUS	GO:0009991	13	-0.246978	-0.634605	0.926129	1.000000	1.000
311	GOBP_NEURON_DEATH	GO:0070997	18	-0.227754	-0.635226	0.936693	1.000000	1.000
312	GOBP_CELLULAR_RESPONSE_TO_NITROGEN_COMPOUND	GO:1901699	20	-0.224352	-0.635442	0.929144	1.000000	1.000
313	GOBP_ACTIVATION_OF_MAPK_ACTIVITY	GO:0000187	6	-0.306670	-0.638037	0.920123	1.000000	1.000
314	HALLMARK_HYPOXIA	M5891	5	-0.322070	-0.639832	0.921212	1.000000	1.000
315	GOBP_POSITIVE_REGULATION_OF_CELLULAR_PROTEIN_L...	GO:1903829	7	-0.294354	-0.642755	0.911544	1.000000	1.000
316	GOBP_POSITIVE_REGULATION_OF_CELL_POPULATION_PR...	GO:0008284	21	-0.226068	-0.645987	0.919090	1.000000	1.000
317	GOBP_REGULATION_OF_PEPTIDE_SECRETION	GO:0002791	7	-0.294719	-0.646563	0.905744	1.000000	1.000
318	GOBP_REGULATION_OF_CELLULAR_COMPONENT_MOVEMENT	GO:0051270	15	-0.243574	-0.647458	0.920943	1.000000	1.000
319	GOBP_LEUKOCYTE_DIFFERENTIATION	GO:0002521	15	-0.244640	-0.647501	0.901596	1.000000	1.000
320	GOBP_NEURON_APOPTOTIC_PROCESS	GO:0051402	12	-0.257323	-0.649058	0.911323	1.000000	1.000
321	GOBP_RESPONSE_TO_INSULIN	GO:0032868	5	-0.334018	-0.649925	0.916272	1.000000	1.000
322	GOBP_NEGATIVE_REGULATION_OF_TRANSCRIPTION_BY_R...	GO:0000122	10	-0.269231	-0.650027	0.904300	1.000000	1.000
323	GOBP_MALE_GAMETE_GENERATION	GO:0048232	5	-0.335495	-0.650874	0.907975	1.000000	1.000
324	GOBP_REGULATION_OF_PHOSPHOLIPASE_ACTIVITY	GO:0010517	6	-0.320280	-0.651386	0.911532	1.000000	1.000
325	GOBP_REPRODUCTIVE_SYSTEM_DEVELOPMENT	GO:0061458	6	-0.308844	-0.651444	0.905063	1.000000	1.000
326	GOBP_ENDOCYTOSIS	GO:0006897	8	-0.287483	-0.651584	0.908046	1.000000	1.000
327	GOBP_ESTABLISHMENT_OF_PROTEIN_LOCALIZATION_TO_...	GO:0072594	5	-0.331139	-0.652206	0.899543	1.000000	1.000
328	GOBP_NEGATIVE_REGULATION_OF_IMMUNE_SYSTEM_PROCESS	GO:0002683	5	-0.329963	-0.655059	0.912420	1.000000	1.000
329	GOBP_REGULATION_OF_CELLULAR_LOCALIZATION	GO:0060341	17	-0.238582	-0.656158	0.915888	1.000000	1.000
330	GOBP_LEARNING	GO:0007612	13	-0.258925	-0.658551	0.910547	1.000000	1.000
331	REACTOME_FC_EPSILON_RECEPTOR_FCERI_SIGNALING	R-RNO-2454202	7	-0.302159	-0.661043	0.884674	1.000000	1.000
332	GOBP_NEGATIVE_REGULATION_OF_KINASE_ACTIVITY	GO:0033673	5	-0.337317	-0.661533	0.896000	1.000000	1.000
333	GOBP_POSITIVE_REGULATION_OF_PROTEOLYSIS	GO:0045862	8	-0.293849	-0.664012	0.866764	1.000000	1.000
334	GOBP_FC_EPSILON_RECEPTOR_SIGNALING_PATHWAY	GO:0038095	7	-0.302159	-0.665279	0.883929	1.000000	1.000
335	GOBP_NEGATIVE_REGULATION_OF_TRANSFERASE_ACTIVITY	GO:0051348	5	-0.337317	-0.667390	0.889937	1.000000	1.000
336	GOBP_REGULATION_OF_LIPASE_ACTIVITY	GO:0060191	6	-0.320280	-0.668285	0.874446	1.000000	1.000
337	GOBP_GROWTH	GO:0040007	14	-0.259607	-0.680803	0.889946	1.000000	1.000
338	GOBP_SENSORY_ORGAN_DEVELOPMENT	GO:0007423	8	-0.305097	-0.684870	0.877219	1.000000	1.000
339	GOBP_REGULATION_OF_SYNAPSE_STRUCTURE_OR_ACTIVITY	GO:0050803	11	-0.275554	-0.684977	0.893793	1.000000	1.000
340	GOBP_POSITIVE_REGULATION_OF_LIPASE_ACTIVITY	GO:0060193	5	-0.354080	-0.687393	0.878315	1.000000	1.000
341	GOBP_CENTRAL_NERVOUS_SYSTEM_NEURON_DIFFERENTIA...	GO:0021953	5	-0.353947	-0.690241	0.891239	1.000000	1.000

Continuation of Table **S9**

	NAME	ID	SIZE	ES	NES	NOM.p.val	FDR.q.val	FWER.p.val
342	GOBP_REGULATION_OF_PHOSPHOLIPASE_C_ACTIVITY	GO:1900274	5	-0.354080	-0.691164	0.866261	1.000000	1.000
343	REACTOME_ADAPTIVE_IMMUNE_SYSTEM	R-RNO-1280218	9	-0.299815	-0.694748	0.858616	1.000000	1.000
344	GOBP_CYTOKINE_PRODUCTION	GO:0001816	10	-0.285218	-0.697272	0.845714	1.000000	1.000
345	GOBP_EPIDERMIS_DEVELOPMENT	GO:0008544	5	-0.357238	-0.698458	0.856925	1.000000	1.000
346	GOBP_NEGATIVE_REGULATION_OF_RESPONSE_TO_STIMULUS	GO:0048585	16	-0.259808	-0.702723	0.866667	1.000000	1.000
347	GOBP_DEPHOSPHORYLATION	GO:0016311	7	-0.327075	-0.703754	0.841390	1.000000	1.000
348	GOBP_PROTEIN_DEPHOSPHORYLATION	GO:0006470	7	-0.327075	-0.704532	0.861446	1.000000	1.000
349	GOBP_PROTEIN_LOCALIZATION_TO_ORGANELLE	GO:0033365	8	-0.313187	-0.711604	0.820090	1.000000	1.000
350	GOBP_HOMEOSTASIS_OF_NUMBER_OF_CELLS	GO:0048872	5	-0.363671	-0.713413	0.834375	1.000000	1.000
351	GOBP_NEGATIVE_REGULATION_OF_APOPTOTIC_SIGNALIN...	GO:2001234	7	-0.330524	-0.716275	0.837446	1.000000	1.000
352	GOBP_NEGATIVE_REGULATION_OF_BIOSYNTHETIC_PROCESS	GO:0009890	13	-0.280000	-0.719923	0.833559	1.000000	1.000
353	GOBP_APOPTOTIC_SIGNALING_PATHWAY	GO:0097190	15	-0.275763	-0.721857	0.836927	1.000000	1.000
354	GOBP_POSITIVE_REGULATION_OF_BIOSYNTHETIC_PROCESS	GO:0009891	27	-0.242064	-0.727331	0.844697	1.000000	1.000
355	GOBP_RESPONSE_TO_PEPTIDE	GO:1901652	18	-0.263232	-0.729296	0.837057	1.000000	1.000
356	GOBP_REGULATION_OF_PEPTIDYL_SERINE_PHOSPHORYLA...	GO:0033135	8	-0.331590	-0.730538	0.831871	1.000000	1.000
357	GOBP_REGULATION_OF_HEMOPOIESIS	GO:1903706	10	-0.303603	-0.732527	0.821429	1.000000	1.000
358	GOBP_REGULATION_OF_RECEPTOR_MEDIATED_ENDOCYTOSIS	GO:0048259	5	-0.375341	-0.732614	0.827640	1.000000	1.000
359	GOBP_REGULATION_OF_ENDOCYTOSIS	GO:0030100	6	-0.353210	-0.733751	0.808157	1.000000	1.000
360	GOBP_EPITHELIAL_CELL_PROLIFERATION	GO:0050673	12	-0.289440	-0.735470	0.804677	1.000000	1.000
361	GOBP_DEVELOPMENTAL_MATURATION	GO:0021700	5	-0.375720	-0.736934	0.819005	1.000000	1.000
362	GOBP_REGULATION_OF_CATABOLIC_PROCESS	GO:0009894	13	-0.286075	-0.737480	0.831276	1.000000	1.000
363	GOBP_SPROUTING_ANGIOGENESIS	GO:0002040	5	-0.374279	-0.738143	0.808847	1.000000	1.000
364	GOBP_RECEPTOR_MEDIATED_ENDOCYTOSIS	GO:0006898	6	-0.363201	-0.739924	0.804665	1.000000	1.000
365	GOBP_REGULATION_OF_CELL_PROJECTION_ORGANIZATION	GO:0031344	20	-0.255907	-0.740703	0.835092	1.000000	1.000
366	REACTOME_PLATELET_ACTIVATION_SIGNALING_AND_AGG...	R-HSA-76002	7	-0.347031	-0.742576	0.813711	1.000000	1.000
367	GOBP_NEGATIVE_REGULATION_OF_CATABOLIC_PROCESS	GO:0009895	8	-0.330968	-0.746305	0.795588	1.000000	1.000
368	GOBP_DNA_METABOLIC_PROCESS	GO:0006259	6	-0.353444	-0.748005	0.799694	1.000000	1.000
369	GOBP_CARBOHYDRATE_HOMEOSTASIS	GO:0033500	7	-0.348267	-0.750478	0.789969	1.000000	1.000
370	GOBP_AMEBOIDAL_TYPE_CELL_MIGRATION	GO:0001667	11	-0.311825	-0.751778	0.820208	1.000000	1.000
371	GOBP_POSITIVE_REGULATION_OF_PEPTIDYL_SERINE_PH...	GO:0033138	8	-0.331590	-0.753854	0.782107	1.000000	1.000
372	GOBP_RESPONSE_TO_GROWTH_FACTOR	GO:0070848	18	-0.272020	-0.754047	0.789688	1.000000	1.000
373	REACTOME_L1CAM_INTERACTIONS	R-RNO-373760	5	-0.389227	-0.754826	0.791069	1.000000	1.000
374	GOBP_REGULATION_OF_MITOTIC_CELL_CYCLE	GO:0007346	5	-0.381893	-0.756447	0.808777	1.000000	1.000
375	GOBP_MYELOID_CELL_DIFFERENTIATION	GO:0030099	13	-0.293053	-0.758075	0.790859	1.000000	1.000
376	GOBP_CELLULAR_RESPONSE_TO_STARVATION	GO:0009267	5	-0.394031	-0.759200	0.800000	1.000000	1.000
377	GOBP_RESPONSE_TO_STARVATION	GO:0042594	5	-0.394031	-0.760552	0.809524	1.000000	1.000
378	GOBP_BEHAVIOR	GO:0007610	33	-0.247835	-0.760574	0.817633	1.000000	1.000
379	GOBP_REGULATION_OF_MAP_KINASE_ACTIVITY	GO:0043405	9	-0.323107	-0.762156	0.767806	1.000000	1.000
380	GOBP_REGULATION_OF_PROTEIN_CATABOLIC_PROCESS	GO:0042176	7	-0.356153	-0.764681	0.787106	1.000000	1.000
381	GOBP_PROTEIN_TETRAMERIZATION	GO:0051262	5	-0.379831	-0.764996	0.779014	1.000000	1.000
382	GOBP_POSITIVE_REGULATION_OF_DNA_BINDING_TRANSC...	GO:0051091	10	-0.320704	-0.768680	0.762712	1.000000	1.000
383	GOBP_POSITIVE_REGULATION_OF_CELLULAR_COMPONENT...	GO:0051130	24	-0.264989	-0.772238	0.786885	1.000000	1.000
384	GOBP_CELL_PART_MORPHOGENESIS	GO:0032990	25	-0.263400	-0.773750	0.782383	1.000000	1.000

Continuation of Table **S9**

	NAME	ID	SIZE	ES	NES	NOM.p.val	FDR.q.val	FWER.p.val
385	GOBP_FAT_CELL_DIFFERENTIATION	GO:0045444	9	-0.339851	-0.774667	0.783167	1.000000	1.000
386	GOBP_MUSCLE_STRUCTURE_DEVELOPMENT	GO:0061061	16	-0.289180	-0.775140	0.772606	1.000000	1.000
387	GOBP_REGULATION_OF_ORGANELLE_ORGANIZATION	GO:0033043	14	-0.298704	-0.777534	0.770805	1.000000	1.000
388	GOBP_CELL_MORPHOGENESIS	GO:0000902	25	-0.263400	-0.783049	0.786865	1.000000	1.000
389	REACTOME_CELL_CYCLE_MITOTIC	R-RNO-69278	5	-0.400386	-0.783532	0.766566	1.000000	1.000
390	GOBP_TUBE_MORPHOGENESIS	GO:0035239	15	-0.290677	-0.783915	0.748290	1.000000	1.000
391	GOBP_CELL_CELL_JUNCTION_ASSEMBLY	GO:0007043	5	-0.397898	-0.784814	0.754545	1.000000	1.000
392	GOBP_NEUROINFLAMMATORY_RESPONSE	GO:0150076	5	-0.399230	-0.785665	0.753012	1.000000	1.000
393	GOBP_CELLULAR_RESPONSE_TO_INORGANIC_SUBSTANCE	GO:0071241	11	-0.316262	-0.788489	0.759184	1.000000	1.000
394	GOBP_NEGATIVE_REGULATION_OF_CELL_POPULATION_PR...	GO:0008285	9	-0.340725	-0.791447	0.746398	1.000000	1.000
395	GOBP_CELLULAR_COMPONENT_MORPHOGENESIS	GO:0032989	25	-0.263400	-0.792434	0.769231	1.000000	1.000
396	GOBP_POSITIVE_REGULATION_OF_CATION_TRANSMEMBRA...	GO:1904064	5	-0.404117	-0.795291	0.767255	1.000000	1.000
397	GOBP_TRANSMEMBRANE_RECEPTOR_PROTEIN_SERINE_THR...	GO:0007178	6	-0.379655	-0.796434	0.735795	1.000000	1.000
398	GOBP_RESPONSE_TO_TEMPERATURE_STIMULUS	GO:0009266	9	-0.336293	-0.797530	0.729323	1.000000	1.000
399	GOBP_PEPTIDYL_TYROSINE_MODIFICATION	GO:0018212	10	-0.333938	-0.802633	0.725989	1.000000	1.000
400	GOBP_MULTICELLULAR_ORGANISMAL_HOMEOSTASIS	GO:0048871	12	-0.325111	-0.803995	0.725784	1.000000	1.000
401	GOBP_INNATE_IMMUNE_RESPONSE	GO:0045087	7	-0.368286	-0.804406	0.721068	1.000000	1.000
402	GOBP_REGULATION_OF_LEUKOCYTE_DIFFERENTIATION	GO:1902105	9	-0.345681	-0.804544	0.731214	1.000000	1.000
403	GOBP_REGULATION_OF_PEPTIDYL_TYROSINE_PHOSPHORY...	GO:0050730	9	-0.343501	-0.805350	0.741007	1.000000	1.000
404	GOBP_RESPONSE_TO_BIOTIC_STIMULUS	GO:0009607	17	-0.297343	-0.808397	0.736983	1.000000	1.000
405	GOBP_REGULATION_OF_MYELOID_CELL_DIFFERENTIATION	GO:0045637	9	-0.349627	-0.809240	0.739007	1.000000	1.000
406	GOBP_RESPONSE_TO_BACTERIUM	GO:0009617	13	-0.313414	-0.809316	0.723577	1.000000	1.000
407	GOBP_POSITIVE_REGULATION_OF_PEPTIDYL_TYROSINE_...	GO:0050731	8	-0.357239	-0.809377	0.729412	1.000000	1.000
408	GOBP_POSITIVE_REGULATION_OF_PEPTIDASE_ACTIVITY	GO:0010952	7	-0.382056	-0.811760	0.732475	1.000000	1.000
409	GOBP_NEGATIVE_REGULATION_OF_NEURON_APOPTOTIC_P...	GO:0043524	8	-0.364989	-0.812192	0.740371	1.000000	1.000
410	GOBP_RESPONSE_TO_MOLECULE_OF_BACTERIAL_ORIGIN	GO:0002237	13	-0.313414	-0.812485	0.722071	1.000000	1.000
411	GOBP_RESPONSE_TO_KETONE	GO:1901654	7	-0.371915	-0.812528	0.735649	1.000000	1.000
412	GOBP_POSITIVE_REGULATION_OF_EPITHELIAL_CELL_PR...	GO:0050679	7	-0.375596	-0.812759	0.723684	1.000000	1.000
413	GOBP_REGULATION_OF_TRANSMEMBRANE_TRANSPORT	GO:0034762	23	-0.279179	-0.812946	0.751948	1.000000	1.000
414	GOBP_MYELOID_LEUKOCYTE_DIFFERENTIATION	GO:0002573	11	-0.331706	-0.820860	0.716292	1.000000	1.000
415	GOBP_POSITIVE_REGULATION_OF_PROTEIN_SERINE_THR...	GO:0071902	9	-0.347650	-0.820937	0.686630	1.000000	1.000
416	GOBP_REGULATION_OF_APOPTOTIC_SIGNALING_PATHWAY	GO:2001233	10	-0.339242	-0.821410	0.713246	1.000000	1.000
417	GOBP_BIOLOGICAL_ADHESION	GO:0022610	19	-0.293850	-0.821840	0.709347	1.000000	1.000
418	GOBP_PHAGOCYTOSIS	GO:0006909	5	-0.412207	-0.822766	0.680000	1.000000	1.000
419	GOBP_NEGATIVE_REGULATION_OF_CELL_PROJECTION_OR...	GO:0031345	5	-0.425651	-0.824410	0.684533	1.000000	1.000
420	GOBP_POSITIVE_REGULATION_OF_ORGANELLE_ORGANIZA...	GO:0010638	10	-0.343888	-0.829104	0.704964	1.000000	1.000
421	GOBP_ORGANIC_ACID_METABOLIC_PROCESS	GO:0006082	7	-0.390387	-0.829716	0.728097	1.000000	1.000
422	GOBP_CELLULAR_RESPONSE_TO_OXYGEN_CONTAINING_CO...	GO:1901701	29	-0.276807	-0.829767	0.707347	1.000000	1.000
423	GOBP_POSITIVE_REGULATION_OF_CYSTEINE_TYPE_ENDO...	GO:2001056	7	-0.382056	-0.830499	0.699387	1.000000	1.000
424	GOBP_POSITIVE_REGULATION_OF_SMALL_MOLECULE_MET...	GO:0062013	5	-0.429592	-0.833493	0.687888	1.000000	1.000
425	GOBP_COGNITION	GO:0050890	28	-0.278408	-0.834299	0.711970	1.000000	1.000
426	GOBP_CELLULAR_RESPONSE_TO_ORGANIC_CYCLIC_COMPOUND	GO:0071407	13	-0.325760	-0.840052	0.687764	1.000000	1.000
427	GOBP_CELL_CYCLE_PROCESS	GO:0022402	11	-0.341129	-0.841574	0.684357	1.000000	1.000

Continuation of Table **S9**

	NAME	ID	SIZE	ES	NES	NOM.p.val	FDR.q.val	FWER.p.val
428	GOBP_FOREBRAIN_DEVELOPMENT	GO:0030900	10	-0.353807	-0.843280	0.658640	1.000000	1.000
429	GOBP_VASCULATURE_DEVELOPMENT	GO:0001944	17	-0.313169	-0.846862	0.667092	1.000000	1.000
430	GOBP_LONG_TERM_MEMORY	GO:0007616	6	-0.418945	-0.848965	0.677321	1.000000	1.000
431	GOBP_SMALL_MOLECULE_METABOLIC_PROCESS	GO:0044281	16	-0.315272	-0.849990	0.672000	1.000000	1.000
432	GOBP_REGULATION_OF_EPITHELIAL_CELL_MIGRATION	GO:0010632	6	-0.412437	-0.850164	0.669219	1.000000	1.000
433	GOBP_NEGATIVE_REGULATION_OF_NEURON_DEATH	GO:1901215	11	-0.346541	-0.851593	0.666195	1.000000	1.000
434	GOBP_POSITIVE_REGULATION_OF_TRANSCRIPTION_BY_R...	GO:0045944	24	-0.288382	-0.854771	0.661125	1.000000	1.000
435	GOBP_SEXUAL_REPRODUCTION	GO:0019953	10	-0.361216	-0.855437	0.651982	1.000000	1.000
436	HALLMARK_ALLOGRAFT_REJECTION	M5950	7	-0.398766	-0.856491	0.672464	1.000000	1.000
437	GOBP_POSITIVE_REGULATION_OF_SECRETION	GO:0051047	6	-0.417540	-0.856519	0.658574	1.000000	1.000
438	GOBP_RESPONSE_TO_ORGANIC_CYCLIC_COMPOUND	GO:0014070	20	-0.303278	-0.858286	0.683463	1.000000	1.000
439	GOBP_AXON_DEVELOPMENT	GO:0061564	18	-0.311245	-0.859383	0.660027	1.000000	1.000
440	GOBP_EXOCYTOSIS	GO:0006887	11	-0.341889	-0.861158	0.644719	1.000000	1.000
441	GOBP_POSITIVE_REGULATION_OF_EPITHELIAL_CELL_MI...	GO:0010634	5	-0.438783	-0.864717	0.674961	1.000000	1.000
442	GOBP_CELL_ACTIVATION	GO:0001775	22	-0.301399	-0.866613	0.667529	1.000000	1.000
443	REACTOME_SIGNALING_BY_NTRK2_TRKB	R-RNO-9006115	6	-0.425140	-0.866803	0.650915	1.000000	1.000
444	GOBP_INTRACELLULAR_PROTEIN_TRANSPORT	GO:0006886	8	-0.390332	-0.867577	0.636914	1.000000	1.000
445	GOBP_NEURON_DEVELOPMENT	GO:0048666	34	-0.286932	-0.868466	0.685680	1.000000	1.000
446	GOBP_REGULATION_OF_CELL_ADHESION	GO:0030155	13	-0.335171	-0.869829	0.671683	1.000000	1.000
447	GOBP_RECEPTOR_INTERNALIZATION	GO:0031623	5	-0.445387	-0.872766	0.641509	1.000000	1.000
448	GOBP_INTRACELLULAR_TRANSPORT	GO:0046907	10	-0.359076	-0.873243	0.622669	1.000000	1.000
449	GOBP_CARBOHYDRATE_DERIVATIVE_BIOSYNTHETIC_PROCESS	GO:1901137	5	-0.449552	-0.874398	0.663987	1.000000	1.000
450	GOBP_REGULATION_OF_MAPK_CASCADE	GO:0043408	13	-0.337748	-0.875682	0.616690	1.000000	1.000
451	GOBP_NEGATIVE_REGULATION_OF_PHOSPHORUS_METABOL...	GO:0010563	9	-0.372811	-0.876602	0.641135	1.000000	1.000
452	GOBP_ORGANIC_ACID_TRANSPORT	GO:0015849	7	-0.406930	-0.876939	0.648286	1.000000	1.000
453	GOBP_NEGATIVE_REGULATION_OF_CELLULAR_COMPONENT...	GO:0051129	11	-0.355887	-0.878647	0.639437	1.000000	1.000
454	GOBP_RESPONSE_TO_OXYGEN_CONTAINING_COMPOUND	GO:1901700	39	-0.279630	-0.879353	0.676792	1.000000	1.000
455	GOBP_NEGATIVE_REGULATION_OF_NUCLEOBASE_CONTAIN...	GO:0045934	13	-0.345992	-0.881489	0.628809	1.000000	1.000
456	GOBP_POSTTRANSCRIPTIONAL_REGULATION_OF_GENE_EX...	GO:0010608	8	-0.390883	-0.881649	0.615611	1.000000	1.000
457	GOBP_FATTY_ACID_TRANSPORT	GO:0015908	6	-0.430345	-0.881809	0.665680	1.000000	1.000
458	GOBP_RECEPTOR_METABOLIC_PROCESS	GO:0043112	5	-0.445387	-0.881980	0.647239	1.000000	1.000
459	HALLMARK_EPITHELIAL_MESENCHYMAL_TRANSITION	M5930	5	-0.454682	-0.884296	0.629630	1.000000	1.000
460	GOBP_NEGATIVE_REGULATION_OF_DEVELOPMENTAL_PROCESS	GO:0051093	13	-0.349337	-0.887796	0.610169	1.000000	1.000
461	GOBP_NEGATIVE_REGULATION_OF_PROTEIN_CATABOLIC_...	GO:0042177	5	-0.456622	-0.888480	0.630745	1.000000	1.000
462	GOBP_RESPONSE_TO_ALCOHOL	GO:0097305	11	-0.363953	-0.889245	0.638327	1.000000	1.000
463	GOBP_REGULATION_OF_PROTEIN_KINASE_ACTIVITY	GO:0045859	21	-0.318997	-0.893178	0.616883	1.000000	1.000
464	GOBP_TISSUE_MORPHOGENESIS	GO:0048729	7	-0.410229	-0.895797	0.595930	1.000000	1.000
465	GOBP_NEGATIVE_REGULATION_OF_PHOSPHORYLATION	GO:0042326	7	-0.420976	-0.896146	0.611963	1.000000	1.000
466	GOBP_PROTEIN_MODIFICATION_BY_SMALL_PROTEIN_CON...	GO:0070647	7	-0.416143	-0.896345	0.627422	1.000000	1.000
467	GOBP_CELL_MORPHOGENESIS_INVOLVED_IN_NEURON_DIF...	GO:0048667	22	-0.310800	-0.896685	0.604381	1.000000	1.000
468	GOBP_NERVE_DEVELOPMENT	GO:0021675	9	-0.385236	-0.899195	0.628040	1.000000	1.000
469	GOBP_NEGATIVE_REGULATION_OF_CELL_DIFFERENTIATION	GO:0045596	13	-0.349337	-0.900835	0.607789	1.000000	1.000
470	GOBP_CELL_PROJECTION_ORGANIZATION	GO:0030030	35	-0.291049	-0.902601	0.624526	1.000000	1.000

Continuation of Table **S9**

	NAME	ID	SIZE	ES	NES	NOM.p.val	FDR.q.val	FWER.p.val
471	GOBP_CENTRAL_NERVOUS_SYSTEM_DEVELOPMENT	GO:0007417	27	-0.306093	-0.903294	0.630065	1.000000	1.000
472	GOBP_REGULATION_OF_GTPASE_ACTIVITY	GO:0043087	5	-0.468821	-0.903542	0.618380	1.000000	1.000
473	GOBP_REGULATION_OF_MYELOID_LEUKOCYTE_DIFFERENT...	GO:0002761	8	-0.402165	-0.903545	0.596439	1.000000	1.000
474	GOBP_CELL_MORPHOGENESIS_INVOLVED_IN_DIFFERENTI...	GO:0000904	22	-0.310800	-0.906143	0.599179	1.000000	1.000
475	GOBP_NEUROGENESIS	GO:0022008	41	-0.289017	-0.906875	0.605583	1.000000	1.000
476	GOBP_MEMBRANE_ORGANIZATION	GO:0061024	12	-0.357829	-0.906955	0.587671	1.000000	1.000
477	GOBP_REGULATION_OF_HYDROLASE_ACTIVITY	GO:0051336	22	-0.316071	-0.907069	0.590226	1.000000	1.000
478	GOBP_REGULATION_OF_SYNAPTIC_TRANSMISSION_GLUTA...	GO:0051966	12	-0.359162	-0.908388	0.576976	1.000000	1.000
479	GOBP_CELL_SURFACE_RECEPTOR_SIGNALING_PATHWAY_I...	GO:1905114	15	-0.340396	-0.913454	0.575472	1.000000	1.000
480	GOBP_REGULATION_OF_ENDOTHELIAL_CELL_MIGRATION	GO:0010594	5	-0.472437	-0.913488	0.595777	1.000000	1.000
481	GOBP_POSITIVE_REGULATION_OF_LONG_TERM_SYNAPTIC...	GO:1900273	5	-0.464470	-0.914546	0.582181	1.000000	1.000
482	GOBP_REGULATION_OF_TRANSFERASE_ACTIVITY	GO:0051338	21	-0.318997	-0.914913	0.590164	1.000000	1.000
483	GOBP_REGULATION_OF_ANATOMICAL_STRUCTURE_MORPHO...	GO:0022603	17	-0.336747	-0.916877	0.591588	1.000000	1.000
484	GOBP_NEURON_PROJECTION_GUIDANCE	GO:0097485	9	-0.398901	-0.918431	0.580370	1.000000	1.000
485	GOBP_TAXIS	GO:0042330	14	-0.350577	-0.919103	0.594558	1.000000	1.000
486	GOBP_POSITIVE_REGULATION_OF_APOPTOTIC_SIGNALIN...	GO:2001235	5	-0.467780	-0.919288	0.587253	1.000000	1.000
487	GOBP_TUBE_DEVELOPMENT	GO:0035295	18	-0.335675	-0.921567	0.579710	1.000000	1.000
488	GOBP_RESPONSE_TO_PEPTIDE_HORMONE	GO:0043434	13	-0.353696	-0.923220	0.570248	1.000000	1.000
489	GOBP_SMALL_MOLECULE_BIOSYNTHETIC_PROCESS	GO:0044283	5	-0.469987	-0.924224	0.599379	1.000000	1.000
490	REACTOME_CELL_CYCLE	R-RNO-1640170	6	-0.450964	-0.924601	0.594880	1.000000	1.000
491	GOBP_STRIATED_MUSCLE_CELL_DIFFERENTIATION	GO:0051146	9	-0.395422	-0.924678	0.561119	1.000000	1.000
492	GOBP_PROTEIN_LOCALIZATION_TO_CELL_PERIPHERY	GO:1990778	12	-0.369396	-0.927523	0.570827	1.000000	1.000
493	REACTOME_HEMOSTASIS	R-RNO-109582	14	-0.353735	-0.927999	0.539597	1.000000	1.000
494	GOBP_RESPONSE_TO_ETHANOL	GO:0045471	5	-0.485894	-0.934515	0.562998	1.000000	1.000
495	GOBP_MACROMOLECULE_CATABOLIC_PROCESS	GO:0009057	11	-0.373989	-0.934689	0.564246	1.000000	1.000
496	GOBP_TRANSMEMBRANE_TRANSPORT	GO:0055085	27	-0.314495	-0.934914	0.575604	1.000000	1.000
497	REACTOME_ANTI_INFLAMMATORY_RESPONSE_FAVOURING_...	R-HSA-9662851	5	-0.479458	-0.939222	0.565831	1.000000	1.000
498	GOBP_POSITIVE_REGULATION_OF_MAPK_CASCADE	GO:0043410	9	-0.402317	-0.939257	0.558074	1.000000	1.000
499	GOBP_NEGATIVE_REGULATION_OF_GENE_EXPRESSION	GO:0010629	11	-0.386568	-0.940812	0.568182	1.000000	1.000
500	GOBP_POSITIVE_REGULATION_OF_SIGNALING	GO:0023056	34	-0.302186	-0.941259	0.555422	1.000000	1.000
501	GOBP_POSITIVE_REGULATION_OF_NUCLEOBASE_CONTAIN...	GO:0045935	28	-0.308099	-0.941843	0.532828	1.000000	1.000
502	GOBP_CELLULAR_MACROMOLECULE_LOCALIZATION	GO:0070727	20	-0.336918	-0.942973	0.562500	1.000000	1.000
503	GOBP_REGULATION_OF_RESPONSE_TO_STRESS	GO:0080134	19	-0.338504	-0.946213	0.533419	1.000000	1.000
504	GOBP_BLOOD_VESSEL_MORPHOGENESIS	GO:0048514	14	-0.358935	-0.946826	0.540184	1.000000	1.000
505	GOBP_POSITIVE_REGULATION_OF_HYDROLASE_ACTIVITY	GO:0051345	16	-0.349691	-0.947642	0.543742	1.000000	1.000
506	GOBP_REGULATION_OF_NERVOUS_SYSTEM_PROCESS	GO:0031644	8	-0.418656	-0.948201	0.534610	1.000000	1.000
507	GOBP_PROTEIN_CATABOLIC_PROCESS	GO:0030163	8	-0.422801	-0.949654	0.542692	1.000000	1.000
508	GOBP_WOUND_HEALING	GO:0042060	13	-0.377088	-0.952811	0.528671	1.000000	1.000
509	GOBP_POSITIVE_REGULATION_OF_MYELOID_CELL_DIFFE...	GO:0045639	7	-0.442972	-0.957806	0.533033	1.000000	1.000
510	GOBP_REGULATION_OF_SIGNALING_RECEPTOR_ACTIVITY	GO:0010469	14	-0.371588	-0.958910	0.541899	1.000000	1.000
511	GOBP_REGULATION_OF_TRANSPORTER_ACTIVITY	GO:0032409	17	-0.353068	-0.959125	0.543978	1.000000	1.000
512	GOBP_PROTEIN_LOCALIZATION_TO_CELL_JUNCTION	GO:1902414	6	-0.453162	-0.960169	0.519006	1.000000	1.000
513	GOBP_MONOAMINE_TRANSPORT	GO:0015844	5	-0.490798	-0.960898	0.528975	1.000000	1.000

Continuation of Table **S9**

	NAME	ID	SIZE	ES	NES	NOM.p.val	FDR.q.val	FWER.p.val
514	GOBP_NEGATIVE_REGULATION_OF_CELL_CYCLE	GO:0045786	5	-0.497122	-0.962495	0.556982	1.000000	1.000
515	GOBP_RESPONSE_TO_MONOSACCHARIDE	GO:0034284	5	-0.490588	-0.964714	0.532813	1.000000	1.000
516	GOBP_CELL_JUNCTION_ASSEMBLY	GO:0034329	8	-0.425645	-0.966178	0.521355	1.000000	1.000
517	GOBP_LONG_TERM_SYNAPTIC_POTENTIATION	GO:0060291	12	-0.391069	-0.966525	0.521862	1.000000	1.000
518	GOBP_MYOTUBE_DIFFERENTIATION	GO:0014902	5	-0.504609	-0.967307	0.511664	1.000000	1.000
519	GOBP_MITOTIC_CELL_CYCLE	GO:0000278	10	-0.401660	-0.967775	0.503597	1.000000	1.000
520	GOBP_RESPONSE_TO_CARBOHYDRATE	GO:0009743	5	-0.490588	-0.970067	0.526398	1.000000	1.000
521	GOBP_PROTEIN_AUTOPHOSPHORYLATION	GO:0046777	5	-0.493727	-0.972767	0.526081	1.000000	1.000
522	GOBP_REGULATION_OF_VASCULATURE_DEVELOPMENT	GO:1901342	5	-0.502763	-0.972944	0.530735	1.000000	1.000
523	GOBP_CELLULAR_RESPONSE_TO_DNA_DAMAGE_STIMULUS	GO:0006974	5	-0.501008	-0.977080	0.504870	1.000000	1.000
524	GOBP_REGULATION_OF_MUSCLE_CELL_DIFFERENTIATION	GO:0051147	6	-0.462630	-0.978013	0.500732	1.000000	1.000
525	GOBP_NEURON_DIFFERENTIATION	GO:0030182	37	-0.317397	-0.984567	0.492665	1.000000	1.000
526	GOBP_DENDRITE_DEVELOPMENT	GO:0016358	10	-0.408862	-0.987919	0.482507	1.000000	1.000
527	GOBP_TISSUE_MIGRATION	GO:0090130	9	-0.428119	-0.990055	0.500697	1.000000	1.000
528	GOBP_REGULATION_OF_CELL_ACTIVATION	GO:0050865	8	-0.437033	-0.990954	0.483642	1.000000	1.000
529	GOBP_REGULATION_OF_RESPONSE_TO_EXTERNAL_STIMULUS	GO:0032101	15	-0.373453	-0.991244	0.487705	1.000000	1.000
530	GOBP_RESPONSE_TO_HEAT	GO:0009408	7	-0.453312	-0.994283	0.498503	1.000000	1.000
531	GOBP_REGULATION_OF_PEPTIDE_HORMONE_SECRETION	GO:0090276	6	-0.477434	-0.995865	0.465774	1.000000	1.000
532	GOBP_REGULATION_OF_NEUROTRANSMITTER_RECEPTOR_A...	GO:0099601	13	-0.391507	-0.998831	0.472185	1.000000	1.000
533	GOBP_PROTEIN_LOCALIZATION_TO_MEMBRANE	GO:0072657	13	-0.395401	-1.005983	0.470667	1.000000	1.000
534	GOBP_NEGATIVE_REGULATION_OF_PROTEIN_MODIFICATI...	GO:0031400	10	-0.419755	-1.006891	0.483501	1.000000	1.000
535	GOBP_POSITIVE_REGULATION_OF_PROTEIN_TYROSINE_K...	GO:0061098	5	-0.523198	-1.011460	0.493066	1.000000	1.000
536	GOBP_ORGANONITROGEN_COMPOUND_BIOSYNTHETIC_PROCESS	GO:1901566	13	-0.396977	-1.013550	0.466851	1.000000	1.000
537	GOBP_MAINTENANCE_OF_LOCATION	GO:0051235	7	-0.468402	-1.017832	0.442308	1.000000	1.000
538	GOBP_ESTABLISHMENT_OF_PROTEIN_LOCALIZATION	GO:0045184	17	-0.371833	-1.022337	0.441489	1.000000	1.000
539	GOBP_REGULATION_OF_PROTEIN_TYROSINE_KINASE_ACT...	GO:0061097	5	-0.523198	-1.023947	0.455259	1.000000	1.000
540	GOBP_SKIN_DEVELOPMENT	GO:0043588	5	-0.521690	-1.024713	0.472403	1.000000	1.000
541	GOBP_REGULATION_OF_ANATOMICAL_STRUCTURE_SIZE	GO:0090066	10	-0.435948	-1.026202	0.444142	1.000000	1.000
542	GOBP_REGULATION_OF_PHOSPHORUS_METABOLIC_PROCESS	GO:0051174	26	-0.343276	-1.027083	0.426938	1.000000	1.000
543	REACTOME_SIGNALING_BY_RHO_GTPASES_MIRO_GTPASES...	R-RNO-9716542	9	-0.441325	-1.028024	0.441727	1.000000	1.000
544	REACTOME_RHO_GTPASE_EFFECTORS	R-RNO-195258	9	-0.441325	-1.029454	0.437768	1.000000	1.000
545	GOBP_REGULATION_OF_MUSCLE_ADAPTATION	GO:0043502	5	-0.531995	-1.030072	0.450237	1.000000	1.000
546	GOBP_ENDOMEMBRANE_SYSTEM_ORGANIZATION	GO:0010256	6	-0.483640	-1.030834	0.430493	1.000000	1.000
547	GOBP_SYNAPTIC_TRANSMISSION_GLUTAMATERGIC	GO:0035249	13	-0.403943	-1.033428	0.428962	1.000000	1.000
548	GOBP_RESPONSE_TO_WOUNDING	GO:0009611	15	-0.393523	-1.038053	0.405442	1.000000	1.000
549	GOBP_MUSCLE_ADAPTATION	GO:0043500	5	-0.531995	-1.039975	0.436937	1.000000	1.000
550	GOBP_POSITIVE_REGULATION_OF_SYNAPTIC_TRANSMISSION	GO:0050806	15	-0.393030	-1.040106	0.408840	1.000000	1.000
551	GOBP_REGULATION_OF_PROTEIN_PHOSPHORYLATION	GO:0001932	25	-0.353239	-1.043536	0.420724	1.000000	1.000
552	GOBP_LOCOMOTORY_BEHAVIOR	GO:0007626	8	-0.464910	-1.045775	0.428157	1.000000	1.000
553	GOBP_CHEMICAL_SYNAPTIC_TRANSMISSION_POSTSYNAPTIC	GO:0099565	9	-0.445176	-1.053956	0.400875	1.000000	1.000
554	GOBP_REGULATION_OF_CELL_CELL_ADHESION	GO:0022407	9	-0.454900	-1.055344	0.391679	1.000000	1.000
555	GOBP_CELLULAR_RESPONSE_TO_KETONE	GO:1901655	5	-0.527475	-1.055367	0.408951	1.000000	1.000
556	GOBP_SYNAPSE_ORGANIZATION	GO:0050808	17	-0.386293	-1.058257	0.399225	1.000000	1.000

Continuation of Table **S9**

	NAME	ID	SIZE	ES	NES	NOM.p.val	FDR.q.val	FWER.p.val
557	GOBP_POSITIVE_REGULATION_OF_TRANSFERASE_ACTIVITY	GO:0051347	19	-0.377536	-1.065566	0.371728	1.000000	1.000
558	GOBP_ENDOTHELIAL_CELL_MIGRATION	GO:0043542	8	-0.475854	-1.066189	0.385650	1.000000	1.000
559	GOBP_POSITIVE_REGULATION_OF_PROTEIN_KINASE_ACT...	GO:0045860	18	-0.384509	-1.068585	0.390564	1.000000	1.000
560	GOBP_ORGANIC_ANION_TRANSPORT	GO:0015711	6	-0.522709	-1.076434	0.411315	1.000000	1.000
561	GOBP_CELLULAR_RESPONSE_TO_PEPTIDE	GO:1901653	13	-0.417092	-1.078080	0.375848	1.000000	1.000
562	GOBP_REGULATION_OF_HORMONE_LEVELS	GO:0010817	12	-0.426909	-1.078515	0.367925	1.000000	1.000
563	GOBP_BLOOD_VESSEL_ENDOTHELIAL_CELL_MIGRATION	GO:0043534	8	-0.475854	-1.079772	0.389855	1.000000	1.000
564	GOBP_CARBOXYLIC_ACID_TRANSPORT	GO:0046942	6	-0.522709	-1.080217	0.377953	1.000000	1.000
565	GOBP_AMINO_ACID_TRANSPORT	GO:0006865	6	-0.522709	-1.081939	0.368421	1.000000	1.000
566	GOBP_CIRCADIAN_RHYTHM	GO:0007623	13	-0.418095	-1.082361	0.366897	1.000000	1.000
567	GOBP_POSITIVE_REGULATION_OF_ENDOTHELIAL_CELL_P...	GO:0001938	6	-0.513757	-1.084172	0.365031	1.000000	1.000
568	GOBP_NERVOUS_SYSTEM_PROCESS	GO:0050877	38	-0.347998	-1.086358	0.342201	1.000000	1.000
569	GOBP_ACIDIC_AMINO_ACID_TRANSPORT	GO:0015800	5	-0.552591	-1.086464	0.366819	1.000000	1.000
570	GOBP_RESPONSE_TO_OXYGEN_LEVELS	GO:0070482	8	-0.473609	-1.087998	0.358900	1.000000	1.000
571	GOBP_REGULATION_OF_CATION_CHANNEL_ACTIVITY	GO:2001257	14	-0.419704	-1.088703	0.344491	1.000000	1.000
572	GOBP_POSITIVE_REGULATION_OF_MOLECULAR_FUNCTION	GO:0044093	37	-0.347159	-1.093316	0.340992	1.000000	1.000
573	GOBP_CELLULAR_GLUCOSE_HOMEOSTASIS	GO:0001678	5	-0.563699	-1.094113	0.361281	1.000000	1.000
574	GOBP_MONOCARBOXYLIC_ACID_TRANSPORT	GO:0015718	5	-0.552591	-1.094331	0.372274	1.000000	1.000
575	GOBP_DICARBOXYLIC_ACID_TRANSPORT	GO:0006835	5	-0.552591	-1.095316	0.369969	1.000000	1.000
576	GOBP_REGULATION_OF_ANION_TRANSPORT	GO:0044070	20	-0.386324	-1.095837	0.325641	1.000000	1.000
577	GOBP_RESPONSE_TO_DRUG	GO:0042493	11	-0.444759	-1.098961	0.326360	1.000000	1.000
578	GOBP_ENDOTHELIAL_CELL_PROLIFERATION	GO:0001935	8	-0.491416	-1.101823	0.320513	1.000000	1.000
579	GOBP_MUSCLE_CELL_DIFFERENTIATION	GO:0042692	10	-0.464815	-1.102901	0.373372	1.000000	1.000
580	GOBP_CELL_CELL_ADHESION	GO:0098609	14	-0.419438	-1.110284	0.318436	1.000000	1.000
581	GOBP_CALCIUM_MEDIATED_SIGNALING	GO:0019722	10	-0.465824	-1.113162	0.318182	1.000000	1.000
582	GOBP_RESPONSE_TO_INTERLEUKIN_1	GO:0070555	5	-0.564244	-1.116376	0.329670	1.000000	1.000
583	GOBP_PEPTIDE_SECRETION	GO:0002790	9	-0.485818	-1.119999	0.326590	1.000000	1.000
584	GOBP_REGULATION_OF_LIPID_LOCALIZATION	GO:1905952	5	-0.575748	-1.127522	0.315625	1.000000	1.000
585	GOBP_PEPTIDYL_AMINO_ACID_MODIFICATION	GO:0018193	22	-0.394009	-1.129270	0.282946	1.000000	1.000
586	GOBP_G_PROTEIN_COUPLED_RECEPTOR_SIGNALING_PATHWAY	GO:0007186	18	-0.408097	-1.134236	0.308201	1.000000	1.000
587	REACTOME_RNA_POLYMERASE_II_TRANSCRIPTION	R-RNO-73857	20	-0.397488	-1.134652	0.318471	1.000000	1.000
588	GOBP_REGULATION_OF_CELLULAR_RESPONSE_TO_STRESS	GO:0080135	9	-0.481712	-1.134963	0.309278	1.000000	1.000
589	GOBP_REGULATION_OF_NMDA_RECEPTOR_ACTIVITY	GO:2000310	10	-0.473536	-1.139352	0.309722	1.000000	1.000
590	GOBP_REGULATION_OF_PROTEIN_MODIFICATION_PROCESS	GO:0031399	28	-0.378627	-1.139712	0.286064	1.000000	1.000
591	GOBP_CHROMOSOME_ORGANIZATION	GO:0051276	6	-0.557842	-1.141265	0.311526	1.000000	1.000
592	GOBP_CELLULAR_RESPONSE_TO_ALCOHOL	GO:0097306	5	-0.596216	-1.142470	0.283969	1.000000	1.000
593	GOBP_RHYTHMIC_PROCESS	GO:0048511	15	-0.427663	-1.142810	0.299724	1.000000	1.000
594	GOBP_REGULATION_OF_CATION_TRANSMEMBRANE_TRANSPORT	GO:1904062	16	-0.424573	-1.144105	0.298343	1.000000	1.000
595	GOBP_POSITIVE_REGULATION_OF_CELLULAR_COMPONENT...	GO:0044089	9	-0.489456	-1.145159	0.312769	1.000000	1.000
596	GOBP_CELLULAR_COMPONENT_MAINTENANCE	GO:0043954	5	-0.577035	-1.145598	0.308442	1.000000	1.000
597	GOBP_RESPONSE_TO_HORMONE	GO:0009725	20	-0.400516	-1.151542	0.272490	1.000000	1.000
598	GOBP_ANATOMICAL_STRUCTURE_FORMATION_INVOLVED_I...	GO:0048646	20	-0.411022	-1.158368	0.271208	1.000000	1.000
599	GOBP_SECRETION	GO:0046903	24	-0.397534	-1.161727	0.270130	1.000000	1.000

Continuation of Table **S9**

	NAME	ID	SIZE	ES	NES	NOM.p.val	FDR.q.val	FWER.p.val
600	GOBP_ORGANOPHOSPHATE_METABOLIC_PROCESS	GO:0019637	8	-0.511421	-1.161778	0.268012	1.000000	1.000
601	GOBP_POSITIVE_REGULATION_OF_PHOSPHORUS_METABOL...	GO:0010562	23	-0.402624	-1.164539	0.259446	1.000000	1.000
602	GOBP_POSITIVE_REGULATION_OF_PROTEIN_MODIFICATI...	GO:0031401	22	-0.409086	-1.173057	0.242967	0.998342	1.000
603	GOBP_ADENYLATE_CYCLASE_INHIBITING_G_PROTEIN_CO...	GO:0007196	5	-0.596236	-1.173152	0.261725	1.000000	1.000
604	GOBP_PROTEIN_PHOSPHORYLATION	GO:0006468	33	-0.381901	-1.173586	0.258724	1.000000	1.000
605	GOBP_MAPK_CASCADE	GO:0000165	18	-0.423257	-1.174522	0.242063	1.000000	1.000
606	GOBP_RESPONSE_TO_LIPID	GO:0033993	19	-0.424141	-1.174553	0.255629	1.000000	1.000
607	GOBP_NEGATIVE_REGULATION_OF_ION_TRANSPORT	GO:0043271	11	-0.473346	-1.176737	0.266943	1.000000	1.000
608	GOBP_CELLULAR_RESPONSE_TO_LIPID	GO:0071396	15	-0.451852	-1.178217	0.256831	1.000000	1.000
609	GOBP_ORGANONITROGEN_COMPOUND_CATABOLIC_PROCESS	GO:1901565	10	-0.493211	-1.179490	0.264069	1.000000	1.000
610	GOBP_CATION_TRANSPORT	GO:0006812	25	-0.405597	-1.181299	0.232617	1.000000	1.000
611	GOBP_REGULATION_OF_HORMONE_SECRETION	GO:0046883	8	-0.525095	-1.182016	0.266176	1.000000	1.000
612	GOBP_CIRCULATORY_SYSTEM_PROCESS	GO:0003013	5	-0.609112	-1.182520	0.233129	1.000000	1.000
613	GOBP_SENSORY_PERCEPTION_OF_PAIN	GO:0019233	5	-0.608226	-1.183996	0.242326	1.000000	1.000
614	GOBP_POSITIVE_REGULATION_OF_PROTEIN_PHOSPHORYL...	GO:0001934	22	-0.409086	-1.185452	0.256545	1.000000	1.000
615	GOBP_PROTEIN_MODIFICATION_BY_SMALL_PROTEIN_CON...	GO:0032446	6	-0.566924	-1.186729	0.263077	1.000000	1.000
616	GOBP_REGULATION_OF_ION_TRANSPORT	GO:0043269	36	-0.381749	-1.188255	0.223793	1.000000	1.000
617	GOBP_HEAD_DEVELOPMENT	GO:0060322	23	-0.415680	-1.192305	0.218629	1.000000	1.000
618	GOBP_RESPONSE_TO_ALKALOID	GO:0043279	5	-0.608198	-1.197047	0.265823	1.000000	1.000
619	REACTOME_MAPK_FAMILY_SIGNALING_CASCADES	R-RNO-5683057	12	-0.473506	-1.199269	0.240109	1.000000	1.000
620	GOBP_COAGULATION	GO:0050817	8	-0.537222	-1.201775	0.252252	1.000000	1.000
621	GOBP_CELL_JUNCTION_ORGANIZATION	GO:0034330	19	-0.433444	-1.202164	0.238095	1.000000	1.000
622	GOBP_LIPID_LOCALIZATION	GO:0010876	10	-0.496916	-1.210198	0.233658	1.000000	1.000
623	GOBP_CATION_TRANSMEMBRANE_TRANSPORT	GO:0098655	18	-0.441926	-1.215516	0.208820	1.000000	1.000
624	GOBP_NUCLEOBASE_CONTAINING_SMALL_MOLECULE_META...	GO:0055086	7	-0.566276	-1.216265	0.220264	1.000000	1.000
625	GOBP_CELLULAR_MACROMOLECULE_CATABOLIC_PROCESS	GO:0044265	5	-0.615464	-1.220344	0.218989	1.000000	1.000
626	GOBP_PEPTIDE_HORMONE_SECRETION	GO:0030072	7	-0.570427	-1.220394	0.209270	1.000000	1.000
627	GOBP_EMBRYONIC_MORPHOGENESIS	GO:0048598	6	-0.596193	-1.222666	0.227474	1.000000	1.000
628	GOBP_PEPTIDYL_SERINE_MODIFICATION	GO:0018209	12	-0.475743	-1.225924	0.206704	1.000000	1.000
629	GOBP_MULTICELLULAR_ORGANISMAL_RESPONSE_TO_STRESS	GO:0033555	5	-0.626071	-1.228291	0.225460	1.000000	1.000
630	REACTOME_INTEGRATION_OF_ENERGY_METABOLISM	R-RNO-163685	5	-0.627115	-1.233136	0.214734	0.994959	1.000
631	REACTOME_INTRACELLULAR_SIGNALING_BY_SECOND_MES...	R-RNO-9006925	15	-0.460083	-1.234268	0.216359	1.000000	1.000
632	GOBP_REGULATION_OF_SYNAPTIC_PLASTICITY	GO:0048167	21	-0.432408	-1.237526	0.193838	0.995584	1.000
633	GOBP_NEGATIVE_REGULATION_OF_ANION_TRANSPORT	GO:1903792	8	-0.547765	-1.240107	0.208999	0.994575	1.000
634	GOBP_ORGANIC_HYDROXY_COMPOUND_TRANSPORT	GO:0015850	6	-0.598071	-1.241865	0.214286	0.997390	1.000
635	GOBP_INSULIN_SECRETION	GO:0030073	7	-0.570427	-1.248338	0.220060	0.978718	1.000
636	GOBP_G_PROTEIN_COUPLED_GLUTAMATE_RECEPTOR_SIGN...	GO:0007216	8	-0.555863	-1.252400	0.206490	0.970793	1.000
637	GOBP_POSITIVE_REGULATION_OF_PROTEIN_METABOLIC_...	GO:0051247	27	-0.423352	-1.253732	0.179975	0.975219	1.000
638	GOBP_PROTEIN_LOCALIZATION_TO_POSTSYNAPSE	GO:0062237	5	-0.643746	-1.257259	0.176744	0.969323	1.000
639	GOBP_LIPID_EXPORT_FROM_CELL	GO:0140353	5	-0.635321	-1.258486	0.181269	0.974921	1.000
640	GOBP_PROTEIN_LOCALIZATION_TO_SYNAPSE	GO:0035418	5	-0.643746	-1.259984	0.176378	0.979005	1.000
641	REACTOME_G_ALPHA_Q_SIGNALLING_EVENTS	R-RNO-416476	6	-0.603556	-1.262308	0.178306	0.979645	1.000
642	REACTOME_GPCR_LIGAND_BINDING	R-RNO-500792	8	-0.565828	-1.262332	0.193922	0.991306	1.000

Continuation of Table **S9**

	NAME	ID	SIZE	ES	NES	NOM.p.val	FDR.q.val	FWER.p.val
643	GOBP_REGULATION_OF_NEURONAL_SYNAPTIC_PLASTICITY	GO:0048168	7	-0.585449	-1.263048	0.197309	0.999959	1.000
644	GOBP_REGULATION_OF_SYSTEM_PROCESS	GO:0044057	18	-0.463942	-1.265682	0.186577	0.999555	1.000
645	GOBP_CHROMATIN_ORGANIZATION	GO:0006325	5	-0.654717	-1.266646	0.163666	1.000000	1.000
646	GOBP_ADENYLATE_CYCLASE_INHIBITING_G_PROTEIN_CO...	GO:0007193	9	-0.546766	-1.268443	0.180115	1.000000	1.000
647	GOBP_REGULATION_OF_MEMBRANE_POTENTIAL	GO:0042391	17	-0.461381	-1.273254	0.169935	0.999643	1.000
648	GOBP_VESICLE_MEDIATED_TRANSPORT_IN_SYNAPSE	GO:0099003	7	-0.591483	-1.273622	0.173333	1.000000	1.000
649	GOBP_EXCITATORY_CHEMICAL_SYNAPTIC_TRANSMISSION	GO:0098976	5	-0.662498	-1.278628	0.158805	0.999542	1.000
650	GOBP_REGULATION_OF_METAL_ION_TRANSPORT	GO:0010959	6	-0.619213	-1.284928	0.143279	0.980427	1.000
651	GOBP_CELLULAR_RESPONSE_TO_HORMONE_STIMULUS	GO:0032870	11	-0.525615	-1.291158	0.161517	0.962047	1.000
652	GOBP_POSITIVE_REGULATION_OF_CATALYTIC_ACTIVITY	GO:0043085	29	-0.423040	-1.295109	0.156627	0.955319	1.000
653	GOBP_REGULATION_OF_VESICLE_MEDIATED_TRANSPORT	GO:0060627	12	-0.504986	-1.296152	0.157447	0.963406	1.000
654	REACTOME_SIGNALING_BY_WNT	R-RNO-195721	7	-0.602468	-1.298483	0.152012	0.965032	1.000
655	GOBP_PLATELET_ACTIVATION	GO:0030168	6	-0.616553	-1.299624	0.172107	0.972347	1.000
656	GOBP_REGULATION_OF_TRANS_SYNAPTIC_SIGNALING	GO:0099177	40	-0.415125	-1.301278	0.135553	0.978024	1.000
657	GOBP_INTRACELLULAR_RECEPTOR_SIGNALING_PATHWAY	GO:0030522	5	-0.670527	-1.315061	0.137405	0.920358	1.000
658	REACTOME_OPIOID_SIGNALLING	R-RNO-111885	12	-0.532007	-1.317205	0.126918	0.923636	1.000
659	REACTOME_RAS_ACTIVATION_UPON_CA2_INFLUX_THROUG...	R-HSA-442982	6	-0.645104	-1.324186	0.131268	0.901819	1.000
660	GOBP_REGULATION_OF_POSTSYNAPTIC_MEMBRANE_POTEN...	GO:0060078	14	-0.507939	-1.327337	0.114325	0.899018	1.000
661	GOBP_CELLULAR_RESPONSE_TO_PEPTIDE_HORMONE_STIM...	GO:0071375	9	-0.579565	-1.338287	0.131657	0.856880	1.000
662	REACTOME_CREB1_PHOSPHORYLATION_THROUGH_NMDA_RE...	R-RNO-442742	8	-0.603537	-1.343626	0.104956	0.842773	1.000
663	GOBP_PURINE_CONTAINING_COMPOUND_METABOLIC_PROCESS	GO:0072521	6	-0.653605	-1.351515	0.122137	0.817701	1.000
664	REACTOME_TRAFFICKING_OF_GLUR2_CONTAINING_AMPA_...	R-RNO-416993	8	-0.597618	-1.356767	0.120664	0.805597	1.000
665	GOBP_ADENYLATE_CYCLASE_MODULATING_G_PROTEIN_CO...	GO:0007188	11	-0.551606	-1.360440	0.111888	0.802109	1.000
666	GOBP_REGULATION_OF_CIRCADIAN_RHYTHM	GO:0042752	7	-0.632489	-1.364290	0.104046	0.796824	1.000
667	GOBP_ION_HOMEOSTASIS	GO:0050801	16	-0.509467	-1.365541	0.093878	0.804584	1.000
668	REACTOME_CLASS_C_3_METABOTROPIC_GLUTAMATE_PHER...	R-RNO-420499	7	-0.623198	-1.367355	0.110764	0.809659	1.000
669	GOBP_MUSCLE_CONTRACTION	GO:0006936	5	-0.702607	-1.368364	0.084375	0.819200	1.000
670	GOBP_REGULATION_OF_TRANSPORT	GO:0051049	44	-0.442158	-1.376852	0.075904	0.790028	1.000
671	GOBP_HORMONE_TRANSPORT	GO:0009914	9	-0.589728	-1.392303	0.094767	0.730630	1.000
672	GOBP_REGULATION_OF_CELLULAR_COMPONENT_BIOGENESIS	GO:0044087	10	-0.577488	-1.396875	0.098870	0.723042	1.000
673	GOBP_HOMEOSTATIC_PROCESS	GO:0042592	31	-0.452616	-1.399601	0.073885	0.722983	1.000
674	GOBP_REGULATION_OF_BODY_FLUID_LEVELS	GO:0050878	11	-0.576377	-1.403685	0.088359	0.716437	1.000
675	GOBP_SECOND_MESSENGER_MEDIATED_SIGNALING	GO:0019932	16	-0.532514	-1.407098	0.067024	0.714960	1.000
676	GOBP_NEUROTRANSMITTER_SECRETION	GO:0007269	6	-0.692969	-1.408347	0.061444	0.723541	1.000
677	GOBP_ORGANOPHOSPHATE_BIOSYNTHETIC_PROCESS	GO:0090407	5	-0.738159	-1.410495	0.061285	0.728433	1.000
678	GOBP_METAL_ION_HOMEOSTASIS	GO:0055065	14	-0.543855	-1.416322	0.078838	0.716275	1.000
679	GOBP_PEPTIDYL_LYSINE_MODIFICATION	GO:0018205	5	-0.718611	-1.425978	0.073244	0.684926	1.000
680	GOBP_IONOTROPIC_GLUTAMATE_RECEPTOR_SIGNALING_P...	GO:0035235	9	-0.615662	-1.428699	0.059002	0.687587	1.000
681	GOBP_CALCIUM_ION_TRANSPORT_INTO_CYTOSOL	GO:0060402	7	-0.654230	-1.429260	0.064897	0.701224	1.000
682	GOBP_CYTOSOLIC_CALCIUM_ION_TRANSPORT	GO:0060401	7	-0.654230	-1.432536	0.061285	0.701606	1.000
683	GOBP_MUSCLE_SYSTEM_PROCESS	GO:0003012	9	-0.598543	-1.433077	0.089021	0.715980	1.000
684	REACTOME_SYNAPTIC_ADHESION_LIKE_MOLECULES	R-RNO-8849932	9	-0.617106	-1.445398	0.066572	0.672724	1.000
685	GOBP_CALCIUM_ION_TRANSMEMBRANE_TRANSPORT	GO:0070588	7	-0.654230	-1.449090	0.077485	0.671131	1.000

Continuation of Table **S9**

	NAME	ID	SIZE	ES	NES	NOM.p.val	FDR.q.val	FWER.p.val
686	GOBP_NUCLEOSIDE_PHOSPHATE_BIOSYNTHETIC_PROCESS	GO:1901293	5	-0.738159	-1.454672	0.056162	0.661038	1.000
687	GOBP_NEGATIVE_REGULATION_OF_TRANSPORT	GO:0051051	13	-0.571344	-1.456044	0.061141	0.672112	1.000
688	REACTOME_G_ALPHA_I_SIGNALLING_EVENTS	R-RNO-418594	18	-0.528796	-1.457380	0.055851	0.683992	1.000
689	GOBP_REGULATION_OF_MUSCLE_SYSTEM_PROCESS	GO:0090257	8	-0.659383	-1.466323	0.049080	0.659484	1.000
690	REACTOME_ASSEMBLY_AND_CELL_SURFACE_PRESENTATIO...	R-RNO-9609736	8	-0.653444	-1.470223	0.042773	0.658543	1.000
691	REACTOME_NEGATIVE_REGULATION_OF_NMDA_RECEPTOR_...	R-HSA-961732	8	-0.653444	-1.477888	0.054492	0.640059	1.000
692	REACTOME_TRANSCRIPTIONAL_REGULATION_BY_MECP2	R-RNO-8986944	7	-0.688166	-1.488674	0.041667	0.605990	1.000
693	GOBP_INORGANIC_ION_TRANSMEMBRANE_TRANSPORT	GO:0098660	9	-0.643190	-1.505724	0.050279	0.547194	1.000
694	REACTOME_SIGNALING_BY_GPCR	R-RNO-372790	22	-0.524598	-1.525836	0.024516	0.480743	1.000
695	GOBP_SYNAPTIC_SIGNALING	GO:0099536	47	-0.478985	-1.532798	0.028674	0.468244	1.000
696	REACTOME_G_PROTEIN_MEDIATED_EVENTS	R-RNO-112040	9	-0.654350	-1.538890	0.036711	0.457880	1.000
697	GOBP_SENSORY_PERCEPTION	GO:0007600	9	-0.667809	-1.547433	0.036550	0.441207	1.000
698	GOBP_REGULATION_OF_CYTOSOLIC_CALCIUM_ION_CONCE...	GO:0051480	13	-0.602256	-1.554651	0.020690	0.430099	1.000
699	REACTOME_LONG_TERM_POTENTIATION	R-HSA-9620244	10	-0.646852	-1.556433	0.035813	0.439858	1.000
700	GOBP_DIVALENT_INORGANIC_CATION_HOMEOSTASIS	GO:0072507	13	-0.602256	-1.557471	0.030997	0.453915	1.000
701	GOBP_NEUROTRANSMITTER_TRANSPORT	GO:0006836	7	-0.722645	-1.560969	0.029586	0.458920	1.000
702	GOBP_REGULATION_OF_NEUROTRANSMITTER_LEVELS	GO:0001505	7	-0.722645	-1.562921	0.025148	0.471327	1.000
703	REACTOME_TRAFFICKING_OF_AMPA_RECEPTORS	R-RNO-399719	11	-0.643234	-1.569108	0.027816	0.465570	1.000
704	GOBP_CHEMICAL_HOMEOSTASIS	GO:0048878	23	-0.542770	-1.583397	0.017903	0.427749	1.000
705	REACTOME_CA_DEPENDENT_EVENTS	R-RNO-111996	8	-0.699311	-1.586428	0.023460	0.435859	1.000
706	REACTOME_UNBLOCKING_OF_NMDA_RECEPTORS_GLUTAMAT...	R-RNO-438066	12	-0.617424	-1.590209	0.024291	0.441943	1.000
707	GOBP_CELLULAR_ION_HOMEOSTASIS	GO:0006873	14	-0.615715	-1.595216	0.018945	0.445566	1.000
708	REACTOME_NEUREXINS_AND_NEUROLIGINS	R-RNO-6794361	9	-0.683473	-1.599594	0.029155	0.452451	1.000
709	REACTOME_BETA_CATENIN_INDEPENDENT_WNT_SIGNALING	R-RNO-3858494	5	-0.807129	-1.608761	0.010654	0.441505	0.999
710	REACTOME_ACTIVATION_OF_NMDA_RECEPTORS_AND_POST...	R-RNO-442755	16	-0.591208	-1.610997	0.017663	0.460898	0.999
711	REACTOME_DAG_AND_IP3_SIGNALING	R-RNO-1489509	8	-0.716803	-1.620917	0.012968	0.449983	0.998
712	GOBP_REGULATION_OF_SECRETION	GO:0051046	14	-0.618539	-1.622892	0.024324	0.474336	0.997
713	GOBP_CELL_CELL_SIGNALING	GO:0007267	56	-0.522178	-1.626755	0.007084	0.493022	0.997
714	GOBP_METAL_ION_TRANSPORT	GO:0030001	11	-0.660112	-1.631975	0.020776	0.510406	0.997
715	REACTOME_PROTEIN_PROTEIN_INTERACTIONS_AT_SYNAPSES	R-RNO-6794362	12	-0.646596	-1.632099	0.009472	0.560541	0.997
716	GOBP_CALCIUM_ION_TRANSPORT	GO:0006816	11	-0.660112	-1.644212	0.014045	0.550322	0.995
717	REACTOME_G_ALPHA_Z_SIGNALLING_EVENTS	R-RNO-418597	5	-0.845055	-1.661861	0.004710	0.514802	0.991
718	REACTOME_NEUROTRANSMITTER_RECEPTORS_AND_POSTSY...	R-RNO-112314	22	-0.585312	-1.672455	0.009162	0.528034	0.984
719	GOBP_GLUTAMATE_RECEPTOR_SIGNALING_PATHWAY	GO:0007215	17	-0.611666	-1.695879	0.014249	0.478738	0.960
720	GOBP_REGULATION_OF_NEUROTRANSMITTER_TRANSPORT	GO:0051588	6	-0.825433	-1.708039	0.001490	0.506180	0.940
721	GOBP_CELLULAR_HOMEOSTASIS	GO:0019725	18	-0.637441	-1.758646	0.003953	0.345479	0.772
722	REACTOME_TRANSMISSION_ACROSS_CHEMICAL_SYNAPSES	R-RNO-112315	23	-0.608925	-1.767434	0.001342	0.412871	0.737
723	GOBP_SIGNAL_RELEASE	GO:0023061	14	-0.677178	-1.799338	0.004065	0.417682	0.595
724	REACTOME_NEURONAL_SYSTEM	R-RNO-112316	26	-0.634937	-1.884670	0.000000	0.252430	0.240

End of Table

Supplementary Table S10: SC cervical segment late profile Profile (45 DPL peak) GSEA results.

Begin of Table **S10**

	NAME	ID	SIZE	ES	NES	NOM.p.val	FDR.q.val	FWER.p.val
0	REACTOME_G_PROTEIN_MEDIATED_EVENTS	R-RNO-112040	9	0.594937	2.096231	0.005464	0.321229	0.273
1	REACTOME_DAG_AND_IP3_SIGNALING	R-RNO-1489509	8	0.587500	2.038604	0.000000	0.240319	0.379
2	GOBP_NEUROINFLAMMATORY_RESPONSE	GO:0150076	5	0.746988	1.986881	0.000000	0.217888	0.478
3	REACTOME_CA_DEPENDENT_EVENTS	R-RNO-111996	8	0.587500	1.932299	0.005405	0.231253	0.603
4	GOBP_POSITIVE_REGULATION_OF_ESTABLISHMENT_OF_P...	GO:1904951	5	0.698795	1.873576	0.008032	0.253591	0.714
5	REACTOME_G_ALPHA_I_SIGNALLING_EVENTS	R-RNO-418594	18	0.371429	1.802278	0.000000	0.308596	0.838
6	REACTOME_G_ALPHA_Z_SIGNALLING_EVENTS	R-RNO-418597	5	0.590361	1.571350	0.027778	0.845724	0.998
7	REACTOME_ANTI_INFLAMMATORY_RESPONSE_FAVOURING_...	R-HSA-9662851	5	0.566265	1.558593	0.024691	0.788925	0.999
8	GOBP_VESICLE_MEDIATED_TRANSPORT_IN_SYNAPSE	GO:0099003	7	0.469136	1.483705	0.070270	0.980450	1.000
9	GOBP_ORGANOPHOSPHATE_BIOSYNTHETIC_PROCESS	GO:0090407	5	0.542169	1.436462	0.086066	1.000000	1.000
10	GOBP_REGULATION_OF_NEUROTRANSMITTER_TRANSPORT	GO:0051588	6	0.493152	1.423565	0.102326	1.000000	1.000
11	GOBP_NUCLEOSIDE_PHOSPHATE_BIOSYNTHETIC_PROCESS	GO:1901293	5	0.542169	1.423515	0.080645	0.945016	1.000
12	GOBP_REGULATION_OF_SECRETION	GO:0051046	14	0.324324	1.407917	0.083333	0.930927	1.000
13	REACTOME_OPIOID_SIGNALLING	R-RNO-111885	12	0.342105	1.397053	0.130769	0.904115	1.000
14	GOBP_SIGNAL_RELEASE	GO:0023061	14	0.324324	1.351769	0.120567	1.000000	1.000
15	GOBP_RESPONSE_TO_HEAT	GO:0009408	7	0.425440	1.335581	0.124378	1.000000	1.000
16	REACTOME_TRAFFICKING_OF_AMPA_RECEPTORS	R-RNO-399719	11	0.345258	1.332677	0.123188	0.974669	1.000
17	GOBP_REGULATION_OF_CELLULAR_RESPONSE_TO_STRESS	GO:0080135	9	0.386593	1.329130	0.160221	0.933027	1.000
18	GOBP_RESPONSE_TO_TEMPERATURE_STIMULUS	GO:0009266	9	0.370397	1.327962	0.147929	0.887156	1.000
19	GOBP_POSITIVE_REGULATION_OF_SECRETION	GO:0051047	6	0.463415	1.304201	0.133028	0.927399	1.000
20	GOBP_REGULATION_OF_VESICLE_MEDIATED_TRANSPORT	GO:0060627	12	0.315789	1.275096	0.164179	0.989246	1.000
21	HALLMARK_ALLOGRAFT_REJECTION	M5950	7	0.399785	1.241317	0.169591	1.000000	1.000
22	GOBP_POSITIVE_REGULATION_OF_ORGANELLE_ORGANIZA...	GO:0010638	10	0.330788	1.233393	0.194805	1.000000	1.000
23	REACTOME_INTERFERON_SIGNALING	R-RNO-913531	6	0.419293	1.219348	0.171806	1.000000	1.000
24	GOBP_ORGANOPHOSPHATE_METABOLIC_PROCESS	GO:0019637	8	0.362500	1.212824	0.210811	1.000000	1.000
25	REACTOME_TRANSCRIPTIONAL_REGULATION_BY_MECP2	R-RNO-8986944	7	0.370370	1.166843	0.235577	1.000000	1.000
26	GOBP_REGULATION_OF_METAL_ION_TRANSPORT	GO:0010959	6	0.396546	1.164011	0.219298	1.000000	1.000
27	REACTOME_NEURONAL_SYSTEM	R-RNO-112316	26	0.225807	1.154877	0.212121	1.000000	1.000
28	GOBP_ADENYLATE_CYCLASE_MODULATING_G_PROTEIN_CO...	GO:0007188	11	0.303015	1.153771	0.245399	1.000000	1.000
29	REACTOME_ONCOGENIC_MAPK_SIGNALING	R-HSA-6802957	5	0.434005	1.145050	0.270161	1.000000	1.000
30	REACTOME_TRANSMISSION_ACROSS_CHEMICAL_SYNAPSES	R-RNO-112315	23	0.215385	1.144123	0.259740	1.000000	1.000
31	GOBP_REGULATION_OF_PEPTIDE_TRANSPORT	GO:0090087	10	0.307692	1.123151	0.290323	1.000000	1.000
32	GOBP_EXOCYTOSIS	GO:0006887	11	0.286390	1.120433	0.290541	1.000000	1.000
33	GOBP_NEUROTRANSMITTER_TRANSPORT	GO:0006836	7	0.345679	1.119814	0.301435	1.000000	1.000
34	REACTOME_NEUROTRANSMITTER_RECEPTORS_AND_POSTSY...	R-RNO-112314	22	0.212121	1.110935	0.319149	1.000000	1.000
35	GOBP_REGULATION_OF_ORGANELLE_ORGANIZATION	GO:0033043	14	0.250672	1.100765	0.290323	1.000000	1.000
36	GOBP_ESTABLISHMENT_OF_PROTEIN_LOCALIZATION	GO:0045184	17	0.229681	1.097540	0.299065	1.000000	1.000
37	GOBP_SECRETION	GO:0046903	24	0.206223	1.094243	0.300000	1.000000	1.000
38	GOBP_POSITIVE_REGULATION_OF_APOPTOTIC_SIGNALIN...	GO:2001235	5	0.421687	1.093242	0.291188	1.000000	1.000
39	GOBP_POSITIVE_REGULATION_OF_TRANSPORT	GO:0051050	26	0.209677	1.089743	0.370370	1.000000	1.000
40	GOBP_REGULATION_OF_NEURON_DIFFERENTIATION	GO:0045664	5	0.433735	1.089111	0.327189	1.000000	1.000

Continuation of Table **S10**

	NAME	ID	SIZE	ES	NES	NOM.p.val	FDR.q.val	FWER.p.val
41	GOBP_REGULATION_OF_TRANS_SYNAPTIC_SIGNALING	GO:0099177	40	0.171324	1.083724	0.291667	0.999037	1.000
42	GOBP_NUCLEOBASE_CONTAINING_SMALL_MOLECULE_META...	GO:0055086	7	0.358025	1.082304	0.339207	0.980425	1.000
43	GOBP_HORMONE_TRANSPORT	GO:0009914	9	0.303797	1.076570	0.318182	0.977801	1.000
44	GOBP_REGULATION_OF_NEUROTRANSMITTER_LEVELS	GO:0001505	7	0.345679	1.056221	0.339806	1.000000	1.000
45	GOBP_POSITIVE_REGULATION_OF_PEPTIDYL_TYROSINE_...	GO:0050731	8	0.316544	1.053905	0.378238	1.000000	1.000
46	GOBP_PEPTIDE_SECRETION	GO:0002790	9	0.303797	1.036609	0.370166	1.000000	1.000
47	REACTOME_BETA_CATENIN_INDEPENDENT_WNT_SIGNALING	R-RNO-3858494	5	0.385542	1.036576	0.333333	1.000000	1.000
48	GOBP_PURINE_CONTAINING_COMPOUND_METABOLIC_PROCESS	GO:0072521	6	0.353659	1.004120	0.393939	1.000000	1.000
49	GOBP_ENDOCYTOSIS	GO:0006897	8	0.300000	0.998122	0.417526	1.000000	1.000
50	GOBP_MULTICELLULAR_ORGANISMAL_RESPONSE_TO_STRESS	GO:0033555	5	0.385542	0.991290	0.460000	1.000000	1.000
51	GOBP_NEUROTRANSMITTER_SECRETION	GO:0007269	6	0.341463	0.973863	0.480952	1.000000	1.000
52	GOBP_ENDOTHELIAL_CELL_PROLIFERATION	GO:0001935	8	0.288864	0.961263	0.448649	1.000000	1.000
53	GOBP_REGULATION_OF_HORMONE_SECRETION	GO:0046883	8	0.300000	0.955914	0.477273	1.000000	1.000
54	GOBP_REGULATION_OF_HEMOPOIESIS	GO:1903706	10	0.256410	0.937107	0.486667	1.000000	1.000
55	GOBP_PEPTIDE_HORMONE_SECRETION	GO:0030072	7	0.296296	0.933504	0.507614	1.000000	1.000
56	GOBP_INSULIN_SECRETION	GO:0030073	7	0.296296	0.932732	0.547619	1.000000	1.000
57	REACTOME_PLATELET_ACTIVATION_SIGNALING_AND_AGG...	R-HSA-76002	7	0.295410	0.932564	0.518518	1.000000	1.000
58	GOBP_CELL_CELL_SIGNALING	GO:0007267	56	0.161374	0.929590	0.600000	1.000000	1.000
59	GOBP_REGULATION_OF_ION_TRANSPORT	GO:0043269	36	0.161557	0.923654	0.531915	1.000000	1.000
60	GOBP_POSITIVE_REGULATION_OF_BINDING	GO:0051099	6	0.317073	0.915520	0.539535	1.000000	1.000
61	GOBP_ADENYLATE_CYCLASE_INHIBITING_G_PROTEIN_CO...	GO:0007196	5	0.337349	0.912058	0.583333	1.000000	1.000
62	REACTOME_GPCR_LIGAND_BINDING	R-RNO-500792	8	0.275000	0.908907	0.497238	1.000000	1.000
63	GOBP_REGULATION_OF_PEPTIDE_SECRETION	GO:0002791	7	0.296296	0.906814	0.511737	1.000000	1.000
64	GOBP_MEMORY	GO:0007613	13	0.208661	0.887545	0.627586	1.000000	1.000
65	REACTOME_RAS_ACTIVATION_UPON_CA2_INFLUX_THROUG...	R-HSA-442982	6	0.305898	0.885674	0.604348	1.000000	1.000
66	GOBP_EPITHELIAL_CELL_PROLIFERATION	GO:0050673	12	0.219438	0.880116	0.609589	1.000000	1.000
67	GOBP_REGULATION_OF_PROTEIN_LOCALIZATION	GO:0032880	18	0.185714	0.878188	0.616279	1.000000	1.000
68	REACTOME_ACTIVATION_OF_NMDA_RECEPTORS_AND_POST...	R-RNO-442755	16	0.194444	0.872191	0.610000	1.000000	1.000
69	GOBP_REGULATION_OF_ENDOCYTOSIS	GO:0030100	6	0.292683	0.871289	0.594203	1.000000	1.000
70	GOBP_ION_HOMEOSTASIS	GO:0050801	16	0.194444	0.869180	0.685714	1.000000	1.000
71	GOBP_CELLULAR_RESPONSE_TO_ALCOHOL	GO:0097306	5	0.318657	0.868549	0.617886	1.000000	1.000
72	GOBP_SYNAPTIC_TRANSMISSION_GLUTAMATERGIC	GO:0035249	13	0.200000	0.866284	0.602941	1.000000	1.000
73	GOBP_REGULATION_OF_PEPTIDE_HORMONE_SECRETION	GO:0090276	6	0.292683	0.865101	0.649038	1.000000	1.000
74	GOBP_RECEPTOR_MEDIATED_ENDOCYTOSIS	GO:0006898	6	0.292683	0.856334	0.614679	1.000000	1.000
75	GOBP_REGULATION_OF_TRANSPORT	GO:0051049	44	0.145711	0.855137	0.630435	1.000000	1.000
76	REACTOME_CLASS_C_3_METABOTROPIC_GLUTAMATE_PHER...	R-RNO-420499	7	0.271605	0.853978	0.602151	1.000000	1.000
77	GOBP_DENDRITE_DEVELOPMENT	GO:0016358	10	0.230769	0.847343	0.686391	1.000000	1.000
78	REACTOME_CREB1_PHOSPHORYLATION_THROUGH_NMDA_RE...	R-RNO-442742	8	0.260203	0.841317	0.698413	1.000000	1.000
79	GOBP_ADENYLATE_CYCLASE_INHIBITING_G_PROTEIN_CO...	GO:0007193	9	0.232374	0.834401	0.635870	1.000000	1.000
80	GOBP_SYNAPTIC_SIGNALING	GO:0099536	47	0.141383	0.830334	0.660000	1.000000	1.000
81	GOBP_PHAGOCYTOSIS	GO:0006909	5	0.316199	0.820870	0.655303	1.000000	1.000
82	GOBP_REGULATION_OF_VASCULAR_ASSOCIATED_SMOOTH_...	GO:1904705	5	0.313902	0.813714	0.712264	1.000000	1.000
83	REACTOME_INTEGRATION_OF_ENERGY_METABOLISM	R-RNO-163685	5	0.313253	0.811089	0.710526	1.000000	1.000

Continuation of Table **S10**

	NAME	ID	SIZE	ES	NES	NOM.p.val	FDR.q.val	FWER.p.val
84	GOBP_NEGATIVE_REGULATION_OF_CELL_DEVELOPMENT	GO:0010721	6	0.276136	0.810615	0.695652	1.000000	1.000
85	GOBP_NERVE_DEVELOPMENT	GO:0021675	9	0.227848	0.808345	0.751479	1.000000	1.000
86	GOBP_AMEBOIDAL_TYPE_CELL_MIGRATION	GO:0001667	11	0.211167	0.799571	0.722222	1.000000	1.000
87	GOBP_REGULATION_OF_RECEPTOR_MEDIATED_ENDOCYTOSIS	GO:0048259	5	0.289157	0.790245	0.761719	1.000000	1.000
88	GOBP_REGULATION_OF_SYNAPTIC_TRANSMISSION_GLUTA...	GO:0051966	12	0.197368	0.788995	0.726619	1.000000	1.000
89	GOBP_REGULATION_OF_CYTOSKELETON_ORGANIZATION	GO:0051493	5	0.289157	0.785252	0.736111	1.000000	1.000
90	GOBP_ACTIVATION_OF_MAPK_ACTIVITY	GO:0000187	6	0.268293	0.780053	0.795652	1.000000	1.000
91	GOBP_POSITIVE_REGULATION_OF_EPITHELIAL_CELL_PR...	GO:0050679	7	0.265510	0.779788	0.729282	1.000000	1.000
92	REACTOME_SIGNALING_BY_GPCR	R-RNO-372790	22	0.152712	0.777521	0.736842	1.000000	1.000
93	GOBP_POSITIVE_REGULATION_OF_HEMOPOIESIS	GO:1903708	7	0.246914	0.775330	0.799043	1.000000	1.000
94	GOBP_RECEPTOR_METABOLIC_PROCESS	GO:0043112	5	0.289157	0.765591	0.735043	1.000000	1.000
95	GOBP_PEPTIDYL_SERINE_MODIFICATION	GO:0018209	12	0.187444	0.764283	0.793333	1.000000	1.000
96	GOBP_RECEPTOR_INTERNALIZATION	GO:0031623	5	0.289157	0.763128	0.765217	1.000000	1.000
97	GOBP_POSITIVE_REGULATION_OF_MAP_KINASE_ACTIVITY	GO:0043406	6	0.268293	0.761989	0.772947	1.000000	1.000
98	GOBP_DEVELOPMENTAL_MATURATION	GO:0021700	5	0.289157	0.749860	0.807843	1.000000	1.000
99	GOBP_REGULATION_OF_LONG_TERM_SYNAPTIC_POTENTIA...	GO:1900271	6	0.255273	0.739757	0.815789	1.000000	1.000
100	GOBP_POSITIVE_REGULATION_OF_PHOSPHORUS_METABOL...	GO:0010562	23	0.138461	0.738561	0.782051	1.000000	1.000
250	GOBP_CELL_PART_MORPHOGENESIS	GO:0032990	25	-0.211248	-0.787343	0.734914	0.936496	1.000
251	GOBP_INFLAMMATORY_RESPONSE	GO:0006954	12	-0.256564	-0.790906	0.756912	0.932674	1.000
252	GOBP_MYELOID_LEUKOCYTE_MEDIATED_IMMUNITY	GO:0002444	6	-0.317299	-0.791671	0.734456	0.933406	1.000
253	GOBP_RESPIRATORY_SYSTEM_DEVELOPMENT	GO:0060541	6	-0.314022	-0.795203	0.726343	0.929373	1.000
254	GOBP_REGULATION_OF_RESPONSE_TO_STRESS	GO:0080134	19	-0.227322	-0.795848	0.726101	0.930325	1.000
255	GOBP_FOREBRAIN_DEVELOPMENT	GO:0030900	10	-0.269207	-0.796760	0.719660	0.930763	1.000
256	GOBP_CELL_ACTIVATION_INVOLVED_IN_IMMUNE_RESPONSE	GO:0002263	6	-0.317299	-0.798268	0.721716	0.930253	1.000
257	REACTOME_NEUTROPHIL_DEGRANULATION	R-RNO-6798695	6	-0.317299	-0.799810	0.715013	0.929658	1.000
258	GOBP_NEGATIVE_REGULATION_OF_APOPTOTIC_SIGNALIN...	GO:2001234	7	-0.301710	-0.800805	0.706853	0.929969	1.000
259	GOBP_DENDRITE_MORPHOGENESIS	GO:0048813	7	-0.300958	-0.804229	0.742749	0.926290	1.000
260	REACTOME_EXTRACELLULAR_MATRIX_ORGANIZATION	R-RNO-1474244	5	-0.347501	-0.813377	0.721536	0.913373	1.000
261	GOBP_CELL_CELL_JUNCTION_ASSEMBLY	GO:0007043	5	-0.343091	-0.813501	0.669811	0.915160	1.000
262	GOBP_BIOLOGICAL_PROCESS_INVOLVED_IN_SYMBIOTIC_...	GO:004440	10	-0.275517	-0.816560	0.697006	0.912089	1.000
263	GOBP_CELL_POPULATION_PROLIFERATION	GO:0008283	32	-0.214072	-0.818989	0.698821	0.909805	1.000
264	GOBP_REGULATION_OF_CELLULAR_PROTEIN_LOCALIZATION	GO:1903827	12	-0.265035	-0.820628	0.689157	0.908940	1.000
265	GOBP_REGULATION_OF_PROTEIN_PHOSPHORYLATION	GO:0001932	25	-0.225885	-0.821403	0.704423	0.909624	1.000
266	GOBP_LOCOMOTORY_BEHAVIOR	GO:0007626	8	-0.300000	-0.823014	0.696126	0.908631	1.000
267	GOBP_CELLULAR_LIPID_METABOLIC_PROCESS	GO:0044255	7	-0.310454	-0.823059	0.711929	0.910545	1.000
268	GOBP_NEGATIVE_REGULATION_OF_NEURON_DEATH	GO:1901215	11	-0.272538	-0.826276	0.684770	0.906912	1.000
269	GOBP_CHEMICAL_HOMEOSTASIS	GO:0048878	23	-0.228217	-0.828217	0.687836	0.905475	1.000
270	GOBP_MORPHOGENESIS_OF_AN_EPITHELIUM	GO:0002009	6	-0.335542	-0.829400	0.702945	0.905425	1.000
271	GOBP_NEGATIVE_REGULATION_OF_PROTEOLYSIS	GO:0045861	8	-0.308854	-0.834784	0.702703	0.897880	1.000
272	GOBP_INTRACELLULAR_PROTEIN_TRANSPORT	GO:0006886	8	-0.305823	-0.836534	0.684783	0.896815	1.000
273	GOBP_REGULATION_OF_OSTEOCLAST_DIFFERENTIATION	GO:0045670	5	-0.355416	-0.836542	0.674839	0.898770	1.000
274	GOBP_LOCOMOTION	GO:0040011	31	-0.222664	-0.844391	0.672359	0.887116	1.000
275	GOBP_ORGANONITROGEN_COMPOUND_BIOSYNTHETIC_PROCESS	GO:1901566	13	-0.274732	-0.845775	0.657534	0.886729	1.000

Continuation of Table **S10**

	NAME	ID	SIZE	ES	NES	NOM.p.val	FDR.q.val	FWER.p.val
276	GOBP_CHROMOSOME_ORGANIZATION	GO:0051276	6	-0.344368	-0.848940	0.675291	0.882959	1.000
277	GOBP_CELLULAR_RESPONSE_TO_PEPTIDE_HORMONE_STIM...	GO:0071375	9	-0.303289	-0.851775	0.669565	0.879967	1.000
278	GOBP_SENSORY_PERCEPTION_OF_PAIN	GO:0019233	5	-0.359701	-0.852870	0.648294	0.879989	1.000
279	GOBP_ORGANIC_ACID_METABOLIC_PROCESS	GO:0006082	7	-0.315379	-0.853015	0.648546	0.881692	1.000
280	GOBP_RESPONSE_TO_ALCOHOL	GO:0097305	11	-0.277153	-0.853224	0.657647	0.883256	1.000
281	REACTOME_MEMBRANE_TRAFFICKING	R-RNO-199991	5	-0.356914	-0.856123	0.649077	0.879910	1.000
282	REACTOME_VESICLE_MEDIATED_TRANSPORT	R-RNO-5653656	5	-0.356914	-0.858032	0.643236	0.878430	1.000
283	REACTOME_RHO_GTPASE_EFFECTORS	R-RNO-195258	9	-0.305686	-0.858675	0.653382	0.879296	1.000
284	GOBP_PROTEIN_PHOSPHORYLATION	GO:0006468	33	-0.224879	-0.860153	0.672287	0.878637	1.000
285	GOBP_PERIPHERAL_NERVOUS_SYSTEM_DEVELOPMENT	GO:0007422	9	-0.302556	-0.862009	0.659731	0.877243	1.000
286	GOBP_TISSUE_MORPHOGENESIS	GO:0048729	7	-0.330144	-0.871102	0.635787	0.862795	1.000
287	GOBP_REGULATION_OF_PROTEIN_BINDING	GO:0043393	7	-0.329703	-0.872666	0.622307	0.861885	1.000
288	GOBP_PROTEIN_LOCALIZATION_TO_PLASMA_MEMBRANE	GO:0072659	7	-0.334640	-0.873147	0.612296	0.863007	1.000
289	GOBP_PROTEIN_COMPLEX_OLIGOMERIZATION	GO:0051259	7	-0.326575	-0.874530	0.620779	0.862387	1.000
290	GOBP_REGULATION_OF_PEPTIDASE_ACTIVITY	GO:0052547	10	-0.297950	-0.875246	0.649939	0.862984	1.000
291	GOBP_PHOSPHATIDYLINOSITOL_3_KINASE_SIGNALING	GO:0014065	7	-0.334663	-0.875382	0.601258	0.864765	1.000
292	REACTOME_SIGNALING_BY_RHO_GTPASES_MIRO_GTPASES...	R-RNO-9716542	9	-0.305686	-0.875843	0.610849	0.865904	1.000
293	GOBP_REGULATION_OF_LIPID_LOCALIZATION	GO:1905952	5	-0.379489	-0.877565	0.609290	0.864694	1.000
294	GOBP_WOUND_HEALING	GO:0042060	13	-0.277601	-0.877816	0.625290	0.866209	1.000
295	GOBP_RESPONSE_TO_PEPTIDE_HORMONE	GO:0043434	13	-0.277589	-0.877975	0.611561	0.867869	1.000
296	GOBP_CELL_ACTIVATION	GO:0001775	22	-0.244754	-0.878597	0.643703	0.868849	1.000
297	GOBP_RESPONSE_TO_HORMONE	GO:0009725	20	-0.251645	-0.878697	0.634173	0.870679	1.000
298	GOBP_POSITIVE_REGULATION_OF_CATALYTIC_ACTIVITY	GO:0043085	29	-0.236773	-0.879058	0.658405	0.872074	1.000
299	GOBP_EPITHELIAL_CELL_DIFFERENTIATION	GO:0030855	7	-0.327550	-0.880365	0.610619	0.871719	1.000
300	GOBP_TUBE_DEVELOPMENT	GO:0035295	18	-0.254102	-0.885621	0.614016	0.863885	1.000
301	GOBP_GLUCOSE_METABOLIC_PROCESS	GO:0006006	6	-0.355225	-0.886008	0.613184	0.865267	1.000
302	REACTOME_SIGNALING_BY_NUCLEAR_RECEPTORS	R-RNO-9006931	8	-0.321798	-0.886343	0.620823	0.866703	1.000
303	GOBP_POSITIVE_REGULATION_OF_PROTEIN_SERINE_THR...	GO:0071902	9	-0.312283	-0.886363	0.630824	0.868723	1.000
304	REACTOME_ESR_MEDIATED_SIGNALING	R-RNO-8939211	8	-0.321798	-0.886525	0.597070	0.870478	1.000
305	GOBP_SMALL_GTPASE_MEDIATED_SIGNAL_TRANSDUCTION	GO:0007264	8	-0.323232	-0.886584	0.618644	0.872443	1.000
306	GOBP_MONOCARBOXYLIC_ACID_METABOLIC_PROCESS	GO:0032787	6	-0.355194	-0.886618	0.601504	0.874458	1.000
307	GOBP_MONOSACCHARIDE_METABOLIC_PROCESS	GO:0005996	6	-0.355225	-0.886936	0.605469	0.875981	1.000
308	GOBP_MONOAMINE_TRANSPORT	GO:0015844	5	-0.377950	-0.889683	0.576503	0.872823	1.000
309	GOBP_POSITIVE_REGULATION_OF_MYELOID_LEUKOCYTE_...	GO:0002763	6	-0.354979	-0.891500	0.607792	0.871390	1.000
310	GOBP_REGULATION_OF_SYNAPSE_STRUCTURE_OR_ACTIVITY	GO:0050803	11	-0.290626	-0.891631	0.595819	0.873204	1.000
311	REACTOME_SIGNALING_BY_NTRKS	R-RNO-166520	18	-0.258634	-0.894830	0.613611	0.869279	1.000
312	GOBP_SMALL_MOLECULE_METABOLIC_PROCESS	GO:0044281	16	-0.266292	-0.896214	0.590960	0.868646	1.000
313	GOBP_REGULATION_OF_MYELOID_CELL_DIFFERENTIATION	GO:0045637	9	-0.308249	-0.897610	0.596698	0.867983	1.000
314	GOBP_ANION_TRANSMEMBRANE_TRANSPORT	GO:0098656	7	-0.340022	-0.898773	0.599499	0.867716	1.000
315	GOBP_PEPTIDE_BIOSYNTHETIC_PROCESS	GO:0043043	6	-0.362184	-0.905454	0.608966	0.856808	1.000
316	GOBP_AMIDE_BIOSYNTHETIC_PROCESS	GO:0043604	6	-0.362184	-0.906868	0.580769	0.856085	1.000
317	GOBP_INTRACELLULAR_TRANSPORT	GO:0046907	10	-0.309368	-0.906913	0.574316	0.858110	1.000
318	GOBP_POSITIVE_REGULATION_OF_ION_TRANSPORT	GO:0043270	17	-0.268282	-0.912266	0.587500	0.849900	1.000

Continuation of Table **S10**

	NAME	ID	SIZE	ES	NES	NOM.p.val	FDR.q.val	FWER.p.val
319	GOBP_NERVOUS_SYSTEM_PROCESS	GO:0050877	38	-0.236307	-0.912821	0.577614	0.850850	1.000
320	GOBP_RAS_PROTEIN_SIGNAL_TRANSDUCTION	GO:0007265	5	-0.389252	-0.912947	0.588811	0.852673	1.000
321	GOBP_ORGANONITROGEN_COMPOUND_CATABOLIC_PROCESS	GO:1901565	10	-0.312859	-0.913405	0.556220	0.853854	1.000
322	GOBP_CELLULAR_HOMEOSTASIS	GO:0019725	18	-0.265920	-0.914289	0.570953	0.854267	1.000
323	GOBP_POSITIVE_REGULATION_OF_IMMUNE_SYSTEM_PROCESS	GO:0002684	18	-0.268208	-0.914390	0.574635	0.856182	1.000
324	GOBP_PROTEIN_KINASE_B_SIGNALING	GO:0043491	6	-0.371728	-0.915168	0.587071	0.856780	1.000
325	REACTOME_HEMOSTASIS	R-RNO-109582	14	-0.280338	-0.916834	0.546296	0.855469	1.000
326	GOBP_LONG_TERM_SYNAPTIC_POTENTIATION	GO:0060291	12	-0.293781	-0.919528	0.560414	0.852296	1.000
327	GOBP_CELLULAR_RESPONSE_TO_NITROGEN_COMPOUND	GO:1901699	20	-0.263656	-0.919554	0.575221	0.854382	1.000
328	GOBP_LEUKOCYTE_DIFFERENTIATION	GO:0002521	15	-0.284232	-0.920613	0.577227	0.854457	1.000
329	GOBP_CHROMATIN_ORGANIZATION	GO:0006325	5	-0.393927	-0.921961	0.568575	0.854036	1.000
330	GOBP_PROTEIN_CONTAINING_COMPLEX_SUBUNIT_ORGANI...	GO:0043933	15	-0.282135	-0.923272	0.572748	0.853597	1.000
331	GOBP_POSITIVE_REGULATION_OF_MYELOID_CELL_DIFFE...	GO:0045639	7	-0.347737	-0.925513	0.556544	0.851479	1.000
332	GOBP_PROTEIN_TETRAMERIZATION	GO:0051262	5	-0.405198	-0.930760	0.559367	0.842707	1.000
333	REACTOME_CELL_CYCLE_MITOTIC	R-RNO-69278	5	-0.396967	-0.932373	0.535533	0.841554	1.000
334	GOBP_POSTTRANSCRIPTIONAL_REGULATION_OF_GENE_EX...	GO:0010608	8	-0.347817	-0.933078	0.567436	0.842146	1.000
335	REACTOME_TRAFFICKING_OF_GLUR2_CONTAINING_AMPA_...	R-RNO-416993	8	-0.336724	-0.934288	0.545122	0.841766	1.000
336	GOBP_MUSCLE_SYSTEM_PROCESS	GO:0003012	9	-0.326958	-0.934652	0.545894	0.843078	1.000
337	GOBP_SMALL_MOLECULE_BIOSYNTHETIC_PROCESS	GO:0044283	5	-0.392995	-0.934734	0.545087	0.845052	1.000
338	GOBP_SYNAPSE_ORGANIZATION	GO:0050808	17	-0.273696	-0.934848	0.548206	0.846981	1.000
339	GOBP_PROTEIN_LOCALIZATION_TO_CELL_JUNCTION	GO:1902414	6	-0.373846	-0.935862	0.529948	0.847121	1.000
340	GOBP_POSITIVE_REGULATION_OF_SMOOTH_MUSCLE_CELL...	GO:0048661	5	-0.393275	-0.938059	0.525266	0.844911	1.000
341	GOBP_REGULATION_OF_CATABOLIC_PROCESS	GO:0009894	13	-0.298883	-0.938481	0.549020	0.846274	1.000
342	GOBP_FATTY_ACID_TRANSPORT	GO:0015908	6	-0.376106	-0.938692	0.535668	0.848048	1.000
343	GOBP_POSITIVE_REGULATION_OF_SIGNALING	GO:0023056	34	-0.241549	-0.939576	0.579894	0.848355	1.000
344	GOBP_REGULATION_OF_LYMPHOCYTE_ACTIVATION	GO:0051249	5	-0.392239	-0.942049	0.519308	0.845318	1.000
345	GOBP_NEGATIVE_REGULATION_OF_TRANSPORT	GO:0051051	13	-0.296902	-0.950803	0.518182	0.829692	1.000
346	GOBP_CELL_MORPHOGENESIS_INVOLVED_IN_NEURON_DIF...	GO:0048667	22	-0.265740	-0.951242	0.528078	0.830967	1.000
347	GOBP_REGULATION_OF_AXONOGENESIS	GO:0050770	7	-0.357561	-0.951508	0.528061	0.832660	1.000
348	GOBP_PLATELET_ACTIVATION	GO:0030168	6	-0.376801	-0.954033	0.515789	0.829469	1.000
349	REACTOME_INFECTIOUS_DISEASE	R-HSA-5663205	12	-0.305608	-0.955767	0.518900	0.827979	1.000
350	GOBP_NEURON_DEATH	GO:0070997	18	-0.280448	-0.957834	0.524554	0.825968	1.000
351	GOBP_PROTEIN_LOCALIZATION_TO_CELL_PERIPHERY	GO:1990778	12	-0.314959	-0.957958	0.524684	0.827915	1.000
352	GOBP_MUSCLE_ORGAN_DEVELOPMENT	GO:0007517	9	-0.338417	-0.958220	0.519952	0.829616	1.000
353	GOBP_CELL_MORPHOGENESIS_INVOLVED_IN_DIFFERENTI...	GO:0000904	22	-0.265740	-0.958284	0.527192	0.831730	1.000
354	HALLMARK_KRAS_SIGNALING_UP	M5953	6	-0.376214	-0.960802	0.506494	0.828613	1.000
355	GOBP_REGULATION_OF_PHOSPHORUS_METABOLIC_PROCESS	GO:0051174	26	-0.260514	-0.961582	0.527897	0.829218	1.000
356	GOBP_CELL_CELL_SIGNALING_BY_WNT	GO:0198738	7	-0.367762	-0.962011	0.505102	0.830594	1.000
357	GOBP_REGULATION_OF_CIRCADIAN_RHYTHM	GO:0042752	7	-0.362100	-0.963517	0.513043	0.829575	1.000
358	GOBP_CATION_TRANSPORT	GO:0006812	25	-0.264506	-0.964482	0.521413	0.829786	1.000
359	GOBP_METAL_ION_TRANSPORT	GO:0030001	11	-0.319596	-0.966319	0.504751	0.828161	1.000
360	GOBP_CALCIUM_ION_TRANSPORT	GO:0006816	11	-0.319596	-0.968776	0.508178	0.825217	1.000
361	GOBP_LIPID_LOCALIZATION	GO:0010876	10	-0.330697	-0.968894	0.508961	0.827196	1.000

Continuation of Table **S10**

	NAME	ID	SIZE	ES	NES	NOM.p.val	FDR.q.val	FWER.p.val
362	GOBP_LEUKOCYTE_MIGRATION	GO:0050900	6	-0.385138	-0.969430	0.487990	0.828289	1.000
363	GOBP_RESPONSE_TO_DRUG	GO:0042493	11	-0.324968	-0.970119	0.505364	0.829115	1.000
364	GOBP_REGULATION_OF_FAT_CELL_DIFFERENTIATION	GO:0045598	6	-0.389155	-0.970252	0.494297	0.831079	1.000
365	GOBP_BEHAVIOR	GO:0007610	33	-0.255876	-0.971366	0.520256	0.830883	1.000
366	REACTOME_MAPK_TARGETS_NUCLEAR_EVENTS_MEDIATED_...	R-HSA-450282	5	-0.420342	-0.971958	0.500652	0.831897	1.000
367	GOBP_REGULATION_OF_MUSCLE_SYSTEM_PROCESS	GO:0090257	8	-0.351782	-0.972163	0.484316	0.833827	1.000
368	GOBP_REGULATION_OF_CELL_SIZE	GO:0008361	5	-0.409639	-0.972250	0.484973	0.835983	1.000
369	GOBP_DEFENSE_RESPONSE	GO:0006952	19	-0.285875	-0.974380	0.512651	0.833542	1.000
370	GOBP_REGULATION_OF_CATION_CHANNEL_ACTIVITY	GO:2001257	14	-0.300767	-0.975014	0.501718	0.834455	1.000
371	GOBP_REGULATION_OF_NMDA_RECEPTOR_ACTIVITY	GO:2000310	10	-0.327141	-0.975625	0.498229	0.835486	1.000
372	GOBP_ORGANIC_HYDROXY_COMPOUND_TRANSPORT	GO:0015850	6	-0.386591	-0.975627	0.494297	0.837848	1.000
373	GOBP_NEGATIVE_REGULATION_OF_LIPID_METABOLIC_PR...	GO:0045833	5	-0.413736	-0.977581	0.456989	0.835975	1.000
374	REACTOME_CELLULAR_RESPONSES_TO_EXTERNAL_STIMULI	R-RNO-8953897	11	-0.320442	-0.977825	0.487945	0.837793	1.000
375	GOBP_AMINO_ACID_TRANSPORT	GO:0006865	6	-0.389695	-0.978924	0.493573	0.837872	1.000
376	GOBP_REGULATION_OF_TRANSMEMBRANE_TRANSPORT	GO:0034762	23	-0.274103	-0.980861	0.493003	0.836162	1.000
377	GOBP_RESPONSE_TO_WOUNDING	GO:0009611	15	-0.299312	-0.981384	0.499441	0.837509	1.000
378	GOBP_IMMUNE_SYSTEM_DEVELOPMENT	GO:0002520	17	-0.291260	-0.983641	0.479422	0.835024	1.000
379	GOBP_NEGATIVE_REGULATION_OF_LOCOMOTION	GO:0040013	5	-0.423978	-0.985637	0.473404	0.832823	1.000
380	GOBP_ORGANIC_ANION_TRANSPORT	GO:0015711	6	-0.389695	-0.985803	0.479896	0.834904	1.000
381	GOBP_CARBOXYLIC_ACID_TRANSPORT	GO:0046942	6	-0.389695	-0.990321	0.473548	0.827189	1.000
382	GOBP_REGULATION_OF_TRANSFERASE_ACTIVITY	GO:0051338	21	-0.283302	-0.990363	0.478735	0.829495	1.000
383	GOBP_RESPONSE_TO_NICOTINE	GO:0035094	7	-0.374343	-0.992663	0.467500	0.826919	1.000
384	GOBP_PROTEIN_LOCALIZATION_TO_MEMBRANE	GO:0072657	13	-0.306062	-0.992891	0.468499	0.828836	1.000
385	GOBP_NEGATIVE_REGULATION_OF_SIGNALING	GO:0023057	21	-0.276866	-0.992983	0.490545	0.831085	1.000
386	GOBP_SPROUTING_ANGIOGENESIS	GO:0002040	5	-0.421222	-0.994612	0.464332	0.830003	1.000
387	GOBP_REGULATION_OF_PROTEIN_KINASE_ACTIVITY	GO:0045859	21	-0.283302	-0.995622	0.472588	0.829999	1.000
388	GOBP_MUSCLE_STRUCTURE_DEVELOPMENT	GO:0061061	16	-0.294853	-0.996373	0.472222	0.830789	1.000
389	REACTOME_ESTROGEN_DEPENDENT_NUCLEAR_EVENTS_DOW...	R-RNO-963463	5	-0.423723	-0.997355	0.454784	0.830945	1.000
390	GOBP_REGULATION_OF_CELL_PROJECTION_ORGANIZATION	GO:0031344	20	-0.285703	-0.997825	0.468341	0.832361	1.000
391	REACTOME_POST_TRANSLATIONAL_PROTEIN_MODIFICATION	R-RNO-597592	7	-0.377869	-0.998508	0.490050	0.833302	1.000
392	GOBP_RESPONSE_TO_CADMIUM_ION	GO:0046686	6	-0.401690	-1.001733	0.473548	0.828313	1.000
393	GOBP_POSITIVE_REGULATION_OF_CELL_DEATH	GO:0010942	19	-0.289943	-1.001906	0.467778	0.830386	1.000
394	GOBP_POSITIVE_REGULATION_OF_MOLECULAR_FUNCTION	GO:0044093	37	-0.263296	-1.003181	0.453207	0.829905	1.000
395	GOBP_POSITIVE_REGULATION_OF_CELLULAR_COMPONENT...	GO:0044089	9	-0.358781	-1.004531	0.458937	0.829418	1.000
396	GOBP_NEGATIVE_REGULATION_OF_IMMUNE_SYSTEM_PROCESS	GO:0002683	5	-0.428853	-1.004846	0.451303	0.831201	1.000
397	GOBP_REGULATION_OF_CATION_TRANSMEMBRANE_TRANSPORT	GO:1904062	16	-0.302820	-1.005330	0.470990	0.832619	1.000
398	GOBP_RESPONSE_TO_UV	GO:0009411	5	-0.421474	-1.005371	0.454667	0.835070	1.000
399	GOBP_NEURON_MIGRATION	GO:0001764	5	-0.421617	-1.008002	0.442283	0.831338	1.000
400	REACTOME_RNA_POLYMERASE_II_TRANSCRIPTION	R-RNO-73857	20	-0.292694	-1.010033	0.463043	0.829555	1.000
401	GOBP_OSTEOCLAST_DIFFERENTIATION	GO:0030316	6	-0.400387	-1.010183	0.448635	0.831776	1.000
402	GOBP_ANATOMICAL_STRUCTURE_FORMATION_INVOLVED_I...	GO:0048646	20	-0.291298	-1.011654	0.463087	0.831140	1.000
403	GOBP_INORGANIC_ION_TRANSMEMBRANE_TRANSPORT	GO:0098660	9	-0.353803	-1.013285	0.430108	0.829812	1.000
404	GOBP_POSITIVE_REGULATION_OF_AXONOGENESIS	GO:0050772	5	-0.433603	-1.013886	0.441799	0.830928	1.000

Continuation of Table **S10**

	NAME	ID	SIZE	ES	NES	NOM.p.val	FDR.q.val	FWER.p.val
405	GOBP_CIRCADIAN_RHYTHM	GO:0007623	13	-0.326391	-1.014513	0.457901	0.832087	1.000
406	REACTOME_SIGNALING_BY_RECEPTOR_TYROSINE_KINASES	R-RNO-9006934	26	-0.278990	-1.018421	0.448387	0.825379	1.000
407	GOBP_NEGATIVE_REGULATION_OF_ION_TRANSPORT	GO:0043271	11	-0.342893	-1.022542	0.426714	0.818137	1.000
408	GOBP_REGULATION_OF_PROTEOLYSIS	GO:0030162	12	-0.331043	-1.022584	0.443662	0.820629	1.000
409	REACTOME_G_ALPHA_Q_SIGNALLING_EVENTS	R-RNO-416476	6	-0.411248	-1.023725	0.439735	0.820579	1.000
410	GOBP_ENDOMEMBRANE_SYSTEM_ORGANIZATION	GO:0010256	6	-0.410004	-1.025210	0.443850	0.819779	1.000
411	GOBP_MUSCLE_CELL_PROLIFERATION	GO:0033002	8	-0.374825	-1.025922	0.422794	0.820679	1.000
412	GOBP_REGULATION_OF_MYELOID_LEUKOCYTE_DIFFERENT...	GO:0002761	8	-0.370141	-1.027025	0.417800	0.820560	1.000
413	GOBP_LYMPHOCYTE_ACTIVATION	GO:0046649	10	-0.347701	-1.027032	0.447115	0.823187	1.000
414	GOBP_BIOLOGICAL_ADHESION	GO:0022610	19	-0.294813	-1.027039	0.423453	0.825820	1.000
415	GOBP_RESPONSE_TO_METAL_ION	GO:0010038	15	-0.313118	-1.027417	0.420814	0.827604	1.000
416	GOBP_REGULATION_OF_SYNAPTIC_PLASTICITY	GO:0048167	21	-0.287753	-1.028705	0.433880	0.827038	1.000
417	GOBP_POSITIVE_REGULATION_OF_CYSTEINE_TYPE_ENDO...	GO:2001056	7	-0.391886	-1.028824	0.423750	0.829438	1.000
418	GOBP_REGULATION_OF_INFLAMMATORY_RESPONSE	GO:0050727	7	-0.395809	-1.031123	0.418114	0.826517	1.000
419	GOBP_POSITIVE_REGULATION_OF_CELL_POPULATION_PR...	GO:0008284	21	-0.292261	-1.031908	0.420530	0.827259	1.000
420	GOBP_MACROMOLECULE_CATABOLIC_PROCESS	GO:0009057	11	-0.342871	-1.033545	0.416568	0.825952	1.000
421	GOBP_CELL_JUNCTION_ORGANIZATION	GO:0034330	19	-0.302127	-1.034770	0.440176	0.825495	1.000
422	GOBP_CELLULAR_MACROMOLECULE_LOCALIZATION	GO:0070727	20	-0.291044	-1.036016	0.413755	0.825030	1.000
423	GOBP_NEGATIVE_REGULATION_OF_CELL_PROJECTION_OR...	GO:0031345	5	-0.440213	-1.036708	0.404700	0.826184	1.000
424	GOBP_AGING	GO:0007568	13	-0.327160	-1.038466	0.407070	0.824577	1.000
425	GOBP_MULTI_ORGANISM_PROCESS	GO:0051704	16	-0.311217	-1.038827	0.397536	0.826482	1.000
426	GOBP_NEGATIVE_REGULATION_OF_PROTEIN_CATABOLIC_...	GO:0042177	5	-0.440124	-1.039431	0.399731	0.827774	1.000
427	GOBP_REGULATION_OF_CYSTEINE_TYPE_ENDOPEPTIDASE...	GO:2000116	9	-0.364639	-1.039458	0.416766	0.830485	1.000
428	GOBP_MYELOID_CELL_DIFFERENTIATION	GO:0030099	13	-0.324685	-1.041287	0.413242	0.828708	1.000
429	GOBP_NEGATIVE_REGULATION_OF_CELL_CYCLE	GO:0045786	5	-0.441291	-1.043211	0.410188	0.826708	1.000
430	GOBP_REGULATION_OF_ANION_TRANSMEMBRANE_TRANSPORT	GO:1903959	5	-0.446068	-1.044960	0.412316	0.825186	1.000
431	GOBP_EPIDERMIS_DEVELOPMENT	GO:0008544	5	-0.440136	-1.045830	0.399464	0.825862	1.000
432	REACTOME_DEATH_RECEPTOR_SIGNALLING	R-RNO-73887	5	-0.446982	-1.047353	0.398385	0.824791	1.000
433	GOBP_CARBOHYDRATE_DERIVATIVE_METABOLIC_PROCESS	GO:1901135	8	-0.379542	-1.047511	0.390511	0.827276	1.000
434	GOBP_CELLULAR_CARBOHYDRATE_METABOLIC_PROCESS	GO:0044262	5	-0.443114	-1.049487	0.397835	0.825011	1.000
435	GOBP_REGULATION_OF_DEFENSE_RESPONSE	GO:0031347	7	-0.395809	-1.049777	0.397727	0.827204	1.000
436	GOBP_REGULATION_OF_SMALL_MOLECULE_METABOLIC_PR...	GO:0062012	9	-0.367446	-1.051583	0.403423	0.825460	1.000
437	GOBP_CYTOKINE_PRODUCTION	GO:0001816	10	-0.358901	-1.052447	0.400984	0.826134	1.000
438	GOBP_RESPONSE_TO_ETHANOL	GO:0045471	5	-0.451259	-1.053275	0.384298	0.827046	1.000
439	GOBP_REGULATION_OF_CARBOHYDRATE_BIOSYNTHETIC_P...	GO:0043255	5	-0.447822	-1.053632	0.383798	0.828980	1.000
440	GOBP_CELLULAR_MACROMOLECULE_CATABOLIC_PROCESS	GO:0044265	5	-0.443521	-1.053650	0.370027	0.831833	1.000
441	GOBP_PROTEIN_CATABOLIC_PROCESS	GO:0030163	8	-0.383951	-1.054010	0.404674	0.833714	1.000
442	GOBP_REGULATION_OF_CARBOHYDRATE_METABOLIC_PROCESS	GO:0006109	5	-0.447822	-1.054522	0.382234	0.835360	1.000
443	GOBP_POSITIVE_REGULATION_OF_PEPTIDASE_ACTIVITY	GO:0010952	7	-0.391886	-1.055062	0.404551	0.836930	1.000
444	REACTOME_NGF_STIMULATED_TRANSCRIPTION	R-RNO-9031628	9	-0.373185	-1.057769	0.402895	0.833175	1.000
445	GOBP_POSITIVE_REGULATION_OF_CELL_GROWTH	GO:0030307	6	-0.413737	-1.058209	0.386305	0.835103	1.000
446	GOBP_REGULATION_OF_LIPASE_ACTIVITY	GO:0060191	6	-0.423051	-1.058339	0.374036	0.837746	1.000
447	GOBP_POSITIVE_REGULATION_OF_CELL_PROJECTION_OR...	GO:0031346	10	-0.357091	-1.063334	0.383353	0.827960	1.000

Continuation of Table **S10**

	NAME	ID	SIZE	ES	NES	NOM.p.val	FDR.q.val	FWER.p.val
448	GOBP_RESPONSE_TO_NITROGEN_COMPOUND	GO:1901698	35	-0.277884	-1.063930	0.397028	0.829442	1.000
449	GOBP_MYELOID_LEUKOCYTE_DIFFERENTIATION	GO:0002573	11	-0.346319	-1.065109	0.389773	0.829400	1.000
450	GOBP_POSITIVE_REGULATION_OF_LEUKOCYTE_CELL_CEL...	GO:1903039	5	-0.454164	-1.065268	0.378628	0.831966	1.000
451	GOBP_CARBOHYDRATE_BIOSYNTHETIC_PROCESS	GO:0016051	5	-0.447822	-1.065283	0.366534	0.834969	1.000
452	GOBP_CALCIUM_MEDIATED_SIGNALING	GO:0019722	10	-0.360061	-1.065963	0.365882	0.836185	1.000
453	GOBP_REGULATION_OF_EXTRINSIC_APOPTOTIC_SIGNALI...	GO:2001236	6	-0.421645	-1.066739	0.376486	0.837302	1.000
454	GOBP_REGULATION_OF_IMMUNE_SYSTEM_PROCESS	GO:0002682	21	-0.304706	-1.067738	0.384865	0.837741	1.000
455	GOBP_IMMUNE_EFFECTOR_PROCESS	GO:0002252	11	-0.358006	-1.068809	0.386555	0.837981	1.000
456	GOBP_POSITIVE_REGULATION_OF_CELL_CELL_ADHESION	GO:0022409	5	-0.454164	-1.069934	0.381770	0.838228	1.000
457	GOBP_POSITIVE_REGULATION_OF_IMMUNE_RESPONSE	GO:0050778	9	-0.372555	-1.070215	0.397375	0.840773	1.000
458	GOBP_CENTRAL_NERVOUS_SYSTEM_NEURON_DIFFERENTIA...	GO:0021953	5	-0.454860	-1.070765	0.362784	0.842428	1.000
459	GOBP_CELL_CELL_JUNCTION_ORGANIZATION	GO:0045216	6	-0.421231	-1.070949	0.374836	0.845100	1.000
460	GOBP_REGULATION_OF_INTRACELLULAR_SIGNAL_TRANSD...	GO:1902531	21	-0.301244	-1.072140	0.375965	0.844952	1.000
461	GOBP_HEAD_DEVELOPMENT	GO:0060322	23	-0.295157	-1.077526	0.375679	0.833706	1.000
462	GOBP_RESPONSE_TO_LIPID	GO:0033993	19	-0.308302	-1.077811	0.370130	0.836059	1.000
463	GOBP_LEUKOCYTE_CELL_CELL_ADHESION	GO:0007159	6	-0.425514	-1.078188	0.368906	0.838178	1.000
464	GOBP_REGULATION_OF_MUSCLE_CELL_DIFFERENTIATION	GO:0051147	6	-0.431095	-1.079195	0.343711	0.838850	1.000
465	GOBP_REGULATION_OF_PHOSPHOLIPASE_ACTIVITY	GO:0010517	6	-0.423051	-1.083234	0.355128	0.831018	1.000
466	GOBP_TRANSMEMBRANE_RECEPTOR_PROTEIN_TYROSINE_K...	GO:0007169	19	-0.313340	-1.083444	0.350593	0.833639	1.000
467	GOBP_REGULATION_OF_IMMUNE_RESPONSE	GO:0050776	12	-0.347654	-1.083774	0.354143	0.835929	1.000
468	GOBP_NEGATIVE_REGULATION_OF_RESPONSE_TO_EXTERN...	GO:0032102	5	-0.453184	-1.087272	0.371846	0.829648	1.000
469	GOBP_REGULATION_OF_CELLULAR_COMPONENT_SIZE	GO:0032535	7	-0.419753	-1.087560	0.338481	0.832049	1.000
470	GOBP_TUBE_MORPHOGENESIS	GO:0035239	15	-0.331361	-1.087687	0.371986	0.834947	1.000
471	GOBP_REGULATION_OF_NEURON_PROJECTION_DEVELOPMENT	GO:0010975	16	-0.323705	-1.089146	0.362528	0.834554	1.000
472	GOBP_REGULATION_OF_BODY_FLUID_LEVELS	GO:0050878	11	-0.363860	-1.089924	0.352381	0.835800	1.000
473	GOBP_REGULATION_OF_CELL_ADHESION	GO:0030155	13	-0.343324	-1.091970	0.336406	0.833430	1.000
474	GOBP_CIRCADIAN_REGULATION_OF_GENE_EXPRESSION	GO:0032922	5	-0.459417	-1.092222	0.348806	0.836075	1.000
475	GOBP_POSITIVE_REGULATION_OF_HYDROLASE_ACTIVITY	GO:0051345	16	-0.327479	-1.092782	0.361820	0.837935	1.000
476	GOBP_RESPONSE_TO_CALCIUM_ION	GO:0051592	6	-0.434908	-1.094373	0.354926	0.836920	1.000
477	GOBP_REGULATION_OF_WNT_SIGNALING_PATHWAY	GO:0030111	5	-0.462976	-1.097482	0.339572	0.831580	1.000
478	GOBP_REGULATION_OF_CELLULAR_COMPONENT_BIOGENESIS	GO:0044087	10	-0.370041	-1.099289	0.346062	0.829890	1.000
479	REACTOME_NERVOUS_SYSTEM_DEVELOPMENT	R-RNO-9675108	14	-0.348415	-1.099424	0.360505	0.832808	1.000
480	GOBP_EXTRINSIC_APOPTOTIC_SIGNALING_PATHWAY	GO:0097191	7	-0.417851	-1.100332	0.354381	0.833868	1.000
481	GOBP_RHYTHMIC_PROCESS	GO:0048511	15	-0.340945	-1.101998	0.350457	0.832794	1.000
482	GOBP_DEPHOSPHORYLATION	GO:0016311	7	-0.417454	-1.103901	0.344870	0.830715	1.000
483	GOBP_RESPONSE_TO_INORGANIC_SUBSTANCE	GO:0010035	19	-0.317384	-1.105189	0.332230	0.830651	1.000
484	REACTOME_LEISHMANIA_INFECTION	R-HSA-9658195	10	-0.372502	-1.105448	0.324096	0.833398	1.000
485	GOBP_REGULATION_OF_PROTEIN_MODIFICATION_PROCESS	GO:0031399	28	-0.293170	-1.108197	0.334398	0.828956	1.000
486	GOBP_REGULATION_OF_ANATOMICAL_STRUCTURE_SIZE	GO:0090066	10	-0.373628	-1.108788	0.324675	0.830747	1.000
487	HALLMARK_APOPTOSIS	M5902	6	-0.448852	-1.109981	0.332468	0.830973	1.000
488	REACTOME_INTERLEUKIN_4_AND_INTERLEUKIN_13_SIGN...	R-RNO-6785807	8	-0.411682	-1.115441	0.328377	0.819148	1.000
489	REACTOME_NUCLEAR_EVENTS_KINASE_AND_TRANSCRIPTI...	R-RNO-198725	11	-0.362142	-1.115987	0.310545	0.821066	1.000
490	GOBP_POSITIVE_REGULATION_OF_DEVELOPMENTAL_PROCESS	GO:0051094	32	-0.294911	-1.117229	0.325926	0.820882	1.000

Continuation of Table **S10**

	NAME	ID	SIZE	ES	NES	NOM.p.val	FDR.q.val	FWER.p.val
491	GOBP_POSITIVE_REGULATION_OF_DEVELOPMENTAL_GROWTH	GO:0048639	8	-0.402106	-1.121619	0.313119	0.811874	1.000
492	GOBP_PROTEIN_DEPHOSPHORYLATION	GO:0006470	7	-0.417454	-1.122295	0.319753	0.813589	1.000
493	GOBP_REGULATION_OF_SIGNALING_RECEPTOR_ACTIVITY	GO:0010469	14	-0.346317	-1.123498	0.323360	0.813942	1.000
494	GOBP_POSTSYNAPSE_ORGANIZATION	GO:0099173	10	-0.380815	-1.128150	0.300358	0.804695	1.000
495	GOBP_GLUTAMATE_RECEPTOR_SIGNALING_PATHWAY	GO:0007215	17	-0.332739	-1.131786	0.320542	0.798229	1.000
496	GOBP_POSITIVE_REGULATION_OF_RESPONSE_TO_EXTERN...	GO:0032103	9	-0.394845	-1.132017	0.311869	0.801034	1.000
497	GOBP_NEURON_PROJECTION_ORGANIZATION	GO:0106027	8	-0.405575	-1.134759	0.311111	0.796890	1.000
498	GOBP_NEGATIVE_REGULATION_OF_GENE_EXPRESSION	GO:0010629	11	-0.374993	-1.135714	0.290023	0.797817	1.000
499	GOBP_CELLULAR_RESPONSE_TO_CADMIUM_ION	GO:0071276	5	-0.482215	-1.138282	0.313394	0.794154	1.000
500	GOBP_CELLULAR_RESPONSE_TO_CHEMICAL_STRESS	GO:0062197	7	-0.435223	-1.139501	0.295311	0.793998	1.000
501	GOBP_NEGATIVE_REGULATION_OF_CATABOLIC_PROCESS	GO:0009895	8	-0.417907	-1.142143	0.296020	0.790359	1.000
502	GOBP_CIRCULATORY_SYSTEM_PROCESS	GO:0003013	5	-0.484362	-1.144898	0.284967	0.786259	1.000
503	REACTOME_CIRCADIAN_CLOCK	R-HSA-400253	5	-0.481928	-1.145495	0.283784	0.788206	1.000
504	GOBP_REGULATION_OF_POSTSYNAPTIC_MEMBRANE_POTEN...	GO:0060078	14	-0.354518	-1.147017	0.286517	0.787344	1.000
505	GOBP_CELLULAR_RESPONSE_TO_STARVATION	GO:0009267	5	-0.493556	-1.147262	0.276299	0.790246	1.000
506	GOBP_POSITIVE_REGULATION_OF_GROWTH	GO:0045927	9	-0.401078	-1.148445	0.293317	0.790565	1.000
507	GOBP_REGULATION_OF_LIPID_METABOLIC_PROCESS	GO:0019216	7	-0.437311	-1.148642	0.288010	0.793736	1.000
508	GOBP_CELLULAR_RESPONSE_TO_OXYGEN_CONTAINING_CO...	GO:1901701	29	-0.307794	-1.149442	0.292994	0.795135	1.000
509	GOBP_RESPONSE_TO_STARVATION	GO:0042594	5	-0.493556	-1.149999	0.292414	0.797249	1.000
510	GOBP_NEGATIVE_REGULATION_OF_ANION_TRANSPORT	GO:1903792	8	-0.420592	-1.153008	0.295261	0.792549	1.000
511	GOBP_RESPONSE_TO_ABIOTIC_STIMULUS	GO:0009628	27	-0.310952	-1.153310	0.285093	0.795547	1.000
512	GOBP_HOMEOSTATIC_PROCESS	GO:0042592	31	-0.307208	-1.153320	0.304162	0.799253	1.000
513	GOBP_FAT_CELL_DIFFERENTIATION	GO:0045444	9	-0.403793	-1.154499	0.285542	0.799826	1.000
514	GOBP_OSSIFICATION	GO:0001503	9	-0.409272	-1.156211	0.292121	0.798343	1.000
515	GOBP_CELLULAR_RESPONSE_TO_REACTIVE_OXYGEN_SPECIES	GO:0034614	7	-0.435223	-1.156250	0.283019	0.802035	1.000
516	GOBP_TELENCEPHALON_DEVELOPMENT	GO:0021537	7	-0.443837	-1.160992	0.291980	0.792124	1.000
517	GOBP_T_CELL_ACTIVATION	GO:0042110	8	-0.435015	-1.166351	0.271122	0.780241	1.000
518	GOBP_REGULATION_OF_GROWTH	GO:0040008	13	-0.362184	-1.167274	0.280138	0.781384	1.000
519	GOBP_MUSCLE_CELL_DIFFERENTIATION	GO:0042692	10	-0.397286	-1.168011	0.272175	0.783061	1.000
520	GOBP_ASSOCIATIVE_LEARNING	GO:0008306	7	-0.444444	-1.168120	0.263488	0.786570	1.000
521	GOBP_PEPTIDYL_LYSINE_MODIFICATION	GO:0018205	5	-0.494318	-1.173760	0.265971	0.774111	1.000
522	GOBP_POSITIVE_REGULATION_OF_PROTEOLYSIS	GO:0045862	8	-0.424733	-1.173811	0.251225	0.777776	1.000
523	GOBP_POSITIVE_REGULATION_OF_NEURON_PROJECTION_...	GO:0010976	5	-0.494436	-1.175628	0.253230	0.776401	1.000
524	GOBP_RESPONSE_TO_LIGHT_STIMULUS	GO:0009416	11	-0.390823	-1.179917	0.243757	0.767660	1.000
525	GOBP_REGULATION_OF_BINDING	GO:0051098	11	-0.389990	-1.182917	0.261176	0.763163	1.000
526	REACTOME_INTERLEUKIN_17_SIGNALING	R-RNO-448424	6	-0.468932	-1.184224	0.242188	0.763462	1.000
527	REACTOME_SYNAPTIC_ADHESION_LIKE_MOLECULES	R-RNO-8849932	9	-0.414349	-1.186236	0.250000	0.761257	1.000
528	GOBP_CENTRAL_NERVOUS_SYSTEM_DEVELOPMENT	GO:0007417	27	-0.323851	-1.191748	0.257895	0.749443	1.000
529	GOBP_TRANSMEMBRANE_TRANSPORT	GO:0055085	27	-0.319713	-1.192429	0.239872	0.751462	1.000
530	GOBP_ORGANIC_ACID_TRANSPORT	GO:0015849	7	-0.460695	-1.193345	0.250000	0.752860	1.000
531	GOBP_DEVELOPMENTAL_CELL_GROWTH	GO:0048588	5	-0.501668	-1.194557	0.253989	0.753089	1.000
532	GOBP_CYTOSOLIC_CALCIUM_ION_TRANSPORT	GO:0060401	7	-0.457574	-1.194649	0.252551	0.756768	1.000
533	GOBP_GROWTH	GO:0040007	14	-0.372564	-1.198095	0.242563	0.750263	1.000

Continuation of Table **S10**

	NAME	ID	SIZE	ES	NES	NOM.p.val	FDR.q.val	FWER.p.val
534	GOBP_CELL_GROWTH	GO:0016049	10	-0.415703	-1.198951	0.256691	0.751725	1.000
535	GOBP_TRANSMEMBRANE_RECEPTOR_PROTEIN_SERINE_THR...	GO:0007178	6	-0.475076	-1.198962	0.247706	0.755645	1.000
536	GOBP_CATION_TRANSMEMBRANE_TRANSPORT	GO:0098655	18	-0.352151	-1.199526	0.223451	0.758079	1.000
537	HALLMARK_HYPOXIA	M5891	5	-0.507555	-1.202818	0.232527	0.752501	1.000
538	GOBP_BLOOD_VESSEL_MORPHOGENESIS	GO:0048514	14	-0.376051	-1.205652	0.229807	0.748633	1.000
539	GOBP_GLIOGENESIS	GO:0042063	12	-0.398326	-1.206524	0.235916	0.750063	1.000
540	GOBP_CELL_JUNCTION_ASSEMBLY	GO:0034329	8	-0.435130	-1.206575	0.245169	0.753928	1.000
541	GOBP_CALCIUM_ION_TRANSMEMBRANE_TRANSPORT	GO:0070588	7	-0.457574	-1.208197	0.238095	0.753359	1.000
542	GOBP_REGULATION_OF_LIPID_BIOSYNTHETIC_PROCESS	GO:0046890	6	-0.488835	-1.209953	0.230971	0.752423	1.000
543	GOBP_REGULATION_OF_GTPASE_ACTIVITY	GO:0043087	5	-0.517147	-1.210823	0.237781	0.754081	1.000
544	GOBP_REGULATION_OF_NEUROTRANSMITTER_RECEPTOR_A...	GO:0099601	13	-0.391989	-1.217046	0.240319	0.739530	1.000
545	GOBP_LIPID_BIOSYNTHETIC_PROCESS	GO:0008610	6	-0.488835	-1.219674	0.221785	0.735834	1.000
546	GOBP_REGULATION_OF_SYSTEM_PROCESS	GO:0044057	18	-0.351110	-1.220016	0.232123	0.738880	1.000
547	GOBP_CALCIUM_ION_TRANSPORT_INTO_CYTOSOL	GO:0060402	7	-0.457574	-1.221395	0.214815	0.738616	1.000
548	GOBP_LIPID_METABOLIC_PROCESS	GO:0006629	10	-0.411482	-1.222056	0.195065	0.740835	1.000
549	GOBP_DNA_METABOLIC_PROCESS	GO:0006259	6	-0.486969	-1.224583	0.208440	0.737443	1.000
550	GOBP_GENERATION_OF_PRECURSOR_METABOLITES_AND_E...	GO:0006091	5	-0.518015	-1.227686	0.212435	0.732158	1.000
551	GOBP_CHEMICAL_SYNAPTIC_TRANSMISSION_POSTSYNAPTIC	GO:0099565	9	-0.434373	-1.229964	0.198565	0.729731	1.000
552	GOBP_REGULATION_OF_TRANSPORTER_ACTIVITY	GO:0032409	17	-0.360503	-1.233270	0.221477	0.724109	1.000
553	GOBP_REGULATION_OF_PROTEIN_CATABOLIC_PROCESS	GO:0042176	7	-0.472901	-1.233857	0.210263	0.726783	1.000
554	GOBP_POLYSACCHARIDE_METABOLIC_PROCESS	GO:0005976	5	-0.521907	-1.234258	0.206117	0.729835	1.000
555	GOBP_MULTI_MULTICELLULAR_ORGANISM_PROCESS	GO:0044706	8	-0.445664	-1.235027	0.205845	0.731834	1.000
556	HALLMARK_PI3K_AKT_MTOR_SIGNALING	M5923	6	-0.485913	-1.235116	0.207947	0.735857	1.000
557	GOBP_RESPONSE_TO_ALKALOID	GO:0043279	5	-0.524692	-1.235251	0.204216	0.739886	1.000
558	GOBP_REGULATION_OF_MEMBRANE_POTENTIAL	GO:0042391	17	-0.361744	-1.235976	0.210823	0.742088	1.000
559	GOBP_ENZYME_LINKED_RECEPTOR_PROTEIN_SIGNALING_...	GO:0007167	24	-0.335142	-1.241271	0.203587	0.730496	1.000
560	GOBP_EPITHELIUM_DEVELOPMENT	GO:0060429	15	-0.377603	-1.242585	0.205069	0.731008	1.000
561	GOBP_NEGATIVE_REGULATION_OF_TRANSMEMBRANE_TRAN...	GO:0034763	6	-0.487645	-1.243878	0.213351	0.731537	1.000
562	GOBP_RESPONSE_TO_REACTIVE_OXYGEN_SPECIES	GO:0000302	8	-0.457126	-1.244465	0.195062	0.734155	1.000
563	GOBP_PEPTIDE_METABOLIC_PROCESS	GO:0006518	11	-0.419616	-1.248576	0.188679	0.726292	1.000
564	GOBP_POSITIVE_REGULATION_OF_DEFENSE_RESPONSE	GO:0031349	5	-0.529570	-1.248618	0.189225	0.730684	1.000
565	GOBP_MALE_GAMETE_GENERATION	GO:0048232	5	-0.524187	-1.250750	0.187761	0.728758	1.000
566	GOBP_CARBOHYDRATE_METABOLIC_PROCESS	GO:0005975	9	-0.444401	-1.251268	0.197026	0.731789	1.000
567	GOBP_RESPONSE_TO_OXIDATIVE_STRESS	GO:0006979	8	-0.457126	-1.251360	0.195787	0.736142	1.000
568	GOBP_RESPONSE_TO_CARBOHYDRATE	GO:0009743	5	-0.534035	-1.254285	0.186508	0.731575	1.000
569	GOBP_ANATOMICAL_STRUCTURE_HOMEOSTASIS	GO:0060249	7	-0.467912	-1.254322	0.191595	0.736149	1.000
570	GOBP_CELL_SURFACE_RECEPTOR_SIGNALING_PATHWAY_I...	GO:1905114	15	-0.389498	-1.255783	0.198228	0.736566	1.000
571	GOBP_OSTEOBLAST_DIFFERENTIATION	GO:0001649	5	-0.536696	-1.257891	0.181586	0.734822	1.000
572	GOBP_NEGATIVE_REGULATION_OF_MOLECULAR_FUNCTION	GO:0044092	24	-0.339132	-1.257925	0.200662	0.739501	1.000
573	GOBP_REGULATION_OF_RESPONSE_TO_EXTERNAL_STIMULUS	GO:0032101	15	-0.378443	-1.259602	0.186020	0.739071	1.000
574	REACTOME_MAPK_FAMILY_SIGNALING_CASCADES	R-RNO-5683057	12	-0.408721	-1.260498	0.183432	0.740967	1.000
575	GOBP_CELLULAR_RESPONSE_TO_LIPID	GO:0071396	15	-0.380553	-1.261367	0.197714	0.743066	1.000
576	GOBP_TEMPERATURE_HOMEOSTASIS	GO:0001659	5	-0.533074	-1.261925	0.183727	0.746142	1.000

Continuation of Table **S10**

	NAME	ID	SIZE	ES	NES	NOM.p.val	FDR.q.val	FWER.p.val
577	GOBP_HOMEOSTASIS_OF_NUMBER_OF_CELLS	GO:0048872	5	-0.529772	-1.264395	0.169168	0.743679	1.000
578	GOBP_CELLULAR_RESPONSE_TO_ABIOTIC_STIMULUS	GO:0071214	7	-0.475198	-1.265220	0.167949	0.746010	1.000
579	GOBP_POSITIVE_REGULATION_OF_GLIOGENESIS	GO:0014015	5	-0.531087	-1.266455	0.169786	0.747328	1.000
580	GOBP_LEARNING	GO:0007612	13	-0.408207	-1.266534	0.175115	0.752185	1.000
581	REACTOME_NEUREXINS_AND_NEUROLIGINS	R-RNO-6794361	9	-0.450544	-1.267525	0.198529	0.754390	1.000
582	GOBP_POSITIVE_REGULATION_OF_DNA_BINDING_TRANSC...	GO:0051091	10	-0.421739	-1.267554	0.197115	0.759540	1.000
583	GOBP_REGULATION_OF_NERVOUS_SYSTEM_PROCESS	GO:0031644	8	-0.464109	-1.269537	0.170581	0.758236	1.000
584	GOBP_EMBRYONIC_MORPHOGENESIS	GO:0048598	6	-0.498796	-1.269686	0.168659	0.763139	1.000
585	GOBP_DEVELOPMENTAL_GROWTH	GO:0048589	10	-0.423749	-1.270105	0.184431	0.767241	1.000
586	GOBP_REGULATION_OF_GLIOGENESIS	GO:0014013	5	-0.531087	-1.271011	0.181217	0.769877	1.000
587	GOBP_RESPONSE_TO_OXYGEN_LEVELS	GO:0070482	8	-0.458119	-1.271251	0.199504	0.774609	1.000
588	GOBP_MEMBRANE_PROTEIN_PROTEOLYSIS	GO:0033619	6	-0.507726	-1.278903	0.167500	0.755035	1.000
589	GOBP_NEGATIVE_REGULATION_OF_PHOSPHORYLATION	GO:0042326	7	-0.484115	-1.280218	0.171795	0.756080	1.000
590	GOBP_RESPONSE_TO_MONOSACCHARIDE	GO:0034284	5	-0.534035	-1.284175	0.154248	0.748711	1.000
591	GOBP_REGULATION_OF_PROTEIN_LOCALIZATION_TO_MEM...	GO:1905475	7	-0.491644	-1.284911	0.169742	0.751987	1.000
592	GOBP_REPRODUCTIVE_SYSTEM_DEVELOPMENT	GO:0061458	6	-0.507860	-1.290239	0.160622	0.740680	1.000
593	GOBP_CELL_CYCLE	GO:0007049	21	-0.361266	-1.294390	0.162338	0.732485	1.000
594	GOBP_POSITIVE_REGULATION_OF_CYTOKINE_PRODUCTION	GO:0001819	7	-0.492671	-1.295938	0.155303	0.733084	1.000
595	GOBP_NEGATIVE_REGULATION_OF_PHOSPHORUS_METABOL...	GO:0010563	9	-0.459155	-1.298443	0.159420	0.730495	1.000
596	GOBP_REGULATION_OF_CELL_DEVELOPMENT	GO:0060284	15	-0.395378	-1.305644	0.162870	0.712249	1.000
597	GOBP_DEFENSE_RESPONSE_TO_OTHER_ORGANISM	GO:0098542	9	-0.458029	-1.306437	0.168246	0.715316	1.000
598	GOBP_STRIATED_MUSCLE_CELL_DIFFERENTIATION	GO:0051146	9	-0.457695	-1.310346	0.138012	0.707658	1.000
599	GOBP_POSITIVE_REGULATION_OF_NEURON_DEATH	GO:1901216	5	-0.558999	-1.313916	0.123684	0.701518	1.000
600	GOBP_SEXUAL_REPRODUCTION	GO:0019953	10	-0.443052	-1.318118	0.141119	0.693240	1.000
601	GOBP_REGULATION_OF_MAPK_CASCADE	GO:0043408	13	-0.417588	-1.321482	0.144330	0.688323	1.000
602	REACTOME_PROTEIN_PROTEIN_INTERACTIONS_AT_SYNAPSES	R-RNO-6794362	12	-0.426543	-1.322381	0.142191	0.691091	1.000
603	GOBP_POSITIVE_REGULATION_OF_NEUROGENESIS	GO:0050769	12	-0.435647	-1.325748	0.156398	0.686159	1.000
604	GOBP_REGULATION_OF_DNA_BINDING_TRANSCRIPTION_F...	GO:0051090	16	-0.400618	-1.331179	0.146893	0.674755	1.000
605	GOBP_CELLULAR_COMPONENT_MAINTENANCE	GO:0043954	5	-0.554217	-1.332403	0.127688	0.676482	1.000
606	GOBP_REGULATION_OF_MAP_KINASE_ACTIVITY	GO:0043405	9	-0.466486	-1.335335	0.123609	0.673049	1.000
607	GOBP_TISSUE_HOMEOSTASIS	GO:0001894	5	-0.569047	-1.336046	0.118170	0.676356	1.000
608	GOBP_EXCITATORY_CHEMICAL_SYNAPTIC_TRANSMISSION	GO:0098976	5	-0.566265	-1.338878	0.127027	0.672802	1.000
609	GOBP_PROTEOLYSIS	GO:0006508	17	-0.400677	-1.343693	0.132353	0.662858	1.000
610	GOBP_CELLULAR_AMIDE_METABOLIC_PROCESS	GO:0043603	12	-0.440920	-1.345630	0.118624	0.662289	1.000
611	GOBP_POSITIVE_REGULATION_OF_NERVOUS_SYSTEM_DEV...	GO:0051962	12	-0.435647	-1.347126	0.126874	0.663629	1.000
612	GOBP_POSITIVE_REGULATION_OF_CELL_DEVELOPMENT	GO:0010720	12	-0.435647	-1.348094	0.119266	0.666504	1.000
613	GOBP_NEGATIVE_REGULATION_OF_TRANSFERASE_ACTIVITY	GO:0051348	5	-0.567320	-1.348916	0.107374	0.669835	1.000
614	GOBP_RESPONSE_TO_ACID_CHEMICAL	GO:0001101	6	-0.540298	-1.350641	0.128827	0.670002	1.000
615	GOBP_NEGATIVE_REGULATION_OF_KINASE_ACTIVITY	GO:0033673	5	-0.567320	-1.352262	0.130319	0.671002	1.000
616	GOBP_ENDOCRINE_SYSTEM_DEVELOPMENT	GO:0035270	6	-0.544967	-1.353699	0.121019	0.672398	1.000
617	REACTOME_ADAPTIVE_IMMUNE_SYSTEM	R-RNO-1280218	9	-0.479020	-1.354940	0.125604	0.674344	1.000
618	GOBP_REGULATION_OF_CELL_DIFFERENTIATION	GO:0045595	36	-0.354908	-1.357724	0.109589	0.671399	1.000
619	GOBP_RESPONSE_TO_MOLECULE_OF_BACTERIAL_ORIGIN	GO:0002237	13	-0.426628	-1.359718	0.124717	0.671509	1.000

Continuation of Table **S10**

	NAME	ID	SIZE	ES	NES	NOM.p.val	FDR.q.val	FWER.p.val
620	GOBP_MUSCLE_CELL_DEVELOPMENT	GO:0055001	5	-0.575080	-1.360765	0.124021	0.674221	1.000
621	GOBP_MAINTENANCE_OF_LOCATION	GO:0051235	7	-0.518681	-1.363608	0.118987	0.671265	1.000
622	GOBP_EPHRIN_RECEPTOR_SIGNALING_PATHWAY	GO:0048013	5	-0.579619	-1.363899	0.106860	0.676852	1.000
623	GOBP_RESPONSE_TO_RADIATION	GO:0009314	13	-0.425259	-1.364248	0.114155	0.682350	1.000
624	GOBP_REGULATION_OF_CELL_CYCLE	GO:0051726	13	-0.431755	-1.364761	0.105324	0.687555	1.000
625	GOBP_NUCLEAR_TRANSPORT	GO:0051169	5	-0.579672	-1.365935	0.112299	0.690582	1.000
626	GOBP_NEGATIVE_REGULATION_OF_MULTICELLULAR_ORGA...	GO:0051241	15	-0.419828	-1.368762	0.108945	0.688183	1.000
627	GOBP_GLAND_DEVELOPMENT	GO:0048732	7	-0.516818	-1.369349	0.116688	0.693296	1.000
628	GOBP_RESPONSE_TO_INSULIN	GO:0032868	5	-0.580450	-1.370812	0.105882	0.695571	1.000
629	GOBP_CELLULAR_RESPONSE_TO_ORGANIC_CYCLIC_COMPOUND	GO:0071407	13	-0.429510	-1.374165	0.112903	0.691322	1.000
630	GOBP_AMYLOID_PRECURSOR_PROTEIN_METABOLIC_PROCESS	GO:0042982	5	-0.591906	-1.374238	0.115735	0.698307	1.000
631	GOBP_REGULATION_OF_NEUROGENESIS	GO:0050767	14	-0.427392	-1.374314	0.099429	0.705545	1.000
632	GOBP_NEGATIVE_REGULATION_OF_BINDING	GO:0051100	6	-0.553052	-1.377020	0.102302	0.704180	1.000
633	REACTOME_TRANSCRIPTIONAL_REGULATION_BY_TP53	R-RNO-3700989	6	-0.551622	-1.379881	0.105882	0.701299	1.000
634	REACTOME_TRANSCRIPTIONAL_REGULATION_OF_WHITE_A...	R-RNO-381340	6	-0.550990	-1.385444	0.100000	0.689177	1.000
635	GOBP_RESPONSE_TO_BACTERIUM	GO:0009617	13	-0.426628	-1.386959	0.098751	0.691787	1.000
636	GOBP_AMYLOID_BETA_METABOLIC_PROCESS	GO:0050435	5	-0.591906	-1.389481	0.085979	0.690987	1.000
637	GOBP_RESPONSE_TO_MECHANICAL_STIMULUS	GO:0009612	6	-0.549972	-1.390586	0.100128	0.694990	1.000
638	GOBP_POSITIVE_REGULATION_OF_TRANSCRIPTION_BY_R...	GO:0045944	24	-0.379912	-1.391704	0.090513	0.699176	1.000
639	GOBP_MULTICELLULAR_ORGANISMAL_HOMEOSTASIS	GO:0048871	12	-0.446302	-1.395349	0.111507	0.694320	1.000
640	GOBP_RESPONSE_TO_BIOTIC_STIMULUS	GO:0009607	17	-0.418454	-1.396798	0.094655	0.697301	1.000
641	GOBP_INTRACELLULAR_RECEPTOR_SIGNALING_PATHWAY	GO:0030522	5	-0.587159	-1.399064	0.104698	0.697198	1.000
642	GOBP_PRI_MIRNA_TRANSCRIPTION_BY_RNA_POLYMERASE_II	GO:0061614	7	-0.539743	-1.402169	0.086101	0.693956	1.000
643	GOBP_NEGATIVE_REGULATION_OF_RESPONSE_TO_STIMULUS	GO:0048585	16	-0.422233	-1.403473	0.096591	0.697519	1.000
644	REACTOME_INNATE_IMMUNE_SYSTEM	R-RNO-168249	16	-0.417786	-1.404493	0.093541	0.702132	1.000
645	GOBP_AMYLOID_PRECURSOR_PROTEIN_CATABOLIC_PROCESS	GO:0042987	5	-0.591906	-1.404896	0.098404	0.709548	1.000
646	GOBP_NEGATIVE_REGULATION_OF_NUCLEOBASE_CONTAIN...	GO:0045934	13	-0.445136	-1.418345	0.094907	0.669908	1.000
647	REACTOME_L1CAM_INTERACTIONS	R-RNO-373760	5	-0.594714	-1.419588	0.085752	0.674160	1.000
648	GOBP_REGULATION_OF_DEVELOPMENTAL_GROWTH	GO:0048638	9	-0.489745	-1.421006	0.082339	0.678127	1.000
649	GOBP_POSITIVE_REGULATION_OF_PRI_MIRNA_TRANSCRI...	GO:1902895	7	-0.539743	-1.429396	0.075738	0.657644	1.000
650	GOBP_IONOTROPIC_GLUTAMATE_RECEPTOR_SIGNALING_P...	GO:0035235	9	-0.504775	-1.438119	0.083233	0.636544	1.000
651	GOBP_PROTEIN_MODIFICATION_BY_SMALL_PROTEIN_CON...	GO:0032446	6	-0.575895	-1.438750	0.073491	0.643001	1.000
652	GOBP_GAMETE_GENERATION	GO:0007276	9	-0.513086	-1.438868	0.086480	0.651357	1.000
653	GOBP_CELLULAR_RESPONSE_TO_EXTRACELLULAR_STIMULUS	GO:0031668	8	-0.536060	-1.442127	0.088378	0.648002	1.000
654	GOBP_REGULATION_OF_PROTEIN_SERINE_THREONINE_KI...	GO:0071900	12	-0.467172	-1.443003	0.082654	0.654186	1.000
655	GOBP_REGULATION_OF_CELLULAR_AMIDE_METABOLIC_PR...	GO:0034248	9	-0.507164	-1.445152	0.078883	0.655805	1.000
656	GOBP_REGULATION_OF_MULTICELLULAR_ORGANISMAL_DE...	GO:2000026	31	-0.379564	-1.447050	0.069189	0.658550	1.000
657	GOBP_NEGATIVE_REGULATION_OF_INTRACELLULAR_SIGN...	GO:1902532	7	-0.548625	-1.448399	0.084054	0.663723	1.000
658	GOBP_IMPORT_INTO_CELL	GO:0098657	6	-0.584713	-1.451937	0.058278	0.660618	1.000
659	GOBP_POSITIVE_REGULATION_OF_CELL_DIFFERENTIATION	GO:0045597	27	-0.392753	-1.452230	0.069767	0.669521	1.000
660	GOBP_RESPONSE_TO_TUMOR_NECROSIS_FACTOR	GO:0034612	7	-0.555600	-1.463283	0.072822	0.638260	1.000
661	REACTOME_FC_EPSILON_RECEPTOR_FCERI_SIGNALING	R-RNO-2454202	7	-0.563379	-1.466777	0.073698	0.635173	1.000
662	GOBP_COAGULATION	GO:0050817	8	-0.538641	-1.468227	0.072319	0.640218	1.000

Continuation of Table **S10**

	NAME	ID	SIZE	ES	NES	NOM.p.val	FDR.q.val	FWER.p.val
663	GOBP_RESPONSE_TO_INTERLEUKIN_1	GO:0070555	5	-0.616610	-1.470030	0.067974	0.643565	1.000
664	GOBP_NEGATIVE_REGULATION_OF_PROTEIN_METABOLIC_...	GO:0051248	20	-0.413824	-1.470491	0.057175	0.652406	1.000
665	HALLMARK_TNFA_SIGNALING_VIA_NFKB	M5890	14	-0.461190	-1.475459	0.066069	0.644694	1.000
666	GOBP_MONONUCLEAR_CELL_DIFFERENTIATION	GO:1903131	7	-0.558046	-1.476975	0.086903	0.650004	1.000
667	GOBP_REPRODUCTION	GO:0000003	18	-0.430579	-1.481878	0.053333	0.642502	1.000
668	GOBP_RESPONSE_TO_OXYGEN_CONTAINING_COMPOUND	GO:1901700	39	-0.385835	-1.482274	0.055614	0.652491	1.000
669	GOBP_REGULATION_OF_NERVOUS_SYSTEM_DEVELOPMENT	GO:0051960	15	-0.452406	-1.485541	0.057303	0.651253	1.000
670	REACTOME_TOLL_LIKE_RECEPTOR_TLR1_TLR2_CASCADE	R-RNO-168179	8	-0.548139	-1.487205	0.060991	0.657311	1.000
671	GOBP_MUSCLE_CONTRACTION	GO:0006936	5	-0.634728	-1.494381	0.064603	0.642431	1.000
672	GOBP_FC_EPSILON_RECEPTOR_SIGNALING_PATHWAY	GO:0038095	7	-0.563379	-1.498453	0.039390	0.639404	1.000
673	GOBP_SENSORY_ORGAN_DEVELOPMENT	GO:0007423	8	-0.549151	-1.503507	0.049689	0.633504	1.000
674	GOBP_NEGATIVE_REGULATION_OF_PROTEIN_MODIFICATI...	GO:0031400	10	-0.504088	-1.503823	0.057075	0.644809	1.000
675	REACTOME_MYD88_INDEPENDENT_TLR4_CASCADE	R-RNO-166166	8	-0.548139	-1.505562	0.043956	0.651107	1.000
676	REACTOME_SIGNALING_BY_INTERLEUKINS	R-RNO-449147	15	-0.460942	-1.512339	0.060364	0.638254	1.000
677	GOBP_PROTEIN_AUTOPHOSPHORYLATION	GO:0046777	5	-0.651508	-1.512554	0.038513	0.650658	1.000
678	REACTOME_TOLL_LIKE_RECEPTOR_CASCADES	R-RNO-168898	8	-0.548139	-1.515488	0.058531	0.652903	1.000
679	REACTOME_TOLL_LIKE_RECEPTOR_9_TLR9_CASCADE	R-RNO-168138	8	-0.548139	-1.517552	0.056180	0.658529	1.000
680	GOBP_REGULATION_OF_PROTEIN_LOCALIZATION_TO_CEL...	GO:1904375	6	-0.589519	-1.520005	0.041184	0.663322	1.000
681	REACTOME_DEVELOPMENTAL_BIOLOGY	R-RNO-1266738	22	-0.431203	-1.527839	0.051780	0.648015	1.000
682	GOBP_VASCULATURE_DEVELOPMENT	GO:0001944	17	-0.454094	-1.544242	0.038889	0.601383	1.000
683	REACTOME_CYTOKINE_SIGNALING_IN_IMMUNE_SYSTEM	R-RNO-1280215	21	-0.445101	-1.544747	0.027233	0.613704	1.000
684	REACTOME_EPH_EPHRIN_SIGNALING	R-RNO-268233	6	-0.605914	-1.545544	0.051680	0.625601	1.000
685	GOBP_CELLULAR_RESPONSE_TO_MOLECULE_OF_BACTERIA...	GO:0071219	8	-0.564782	-1.546037	0.042787	0.639417	1.000
686	GOBP_POSITIVE_REGULATION_OF_MULTICELLULAR_ORGA...	GO:0051240	32	-0.402424	-1.551042	0.037473	0.635967	1.000
687	GOBP_RESPONSE_TO_CYTOKINE	GO:0034097	23	-0.427778	-1.552018	0.044565	0.648442	1.000
688	GOBP_POSITIVE_REGULATION_OF_NUCLEOBASE_CONTAIN...	GO:0045935	28	-0.417179	-1.555679	0.032258	0.650075	1.000
689	GOBP_CELLULAR_RESPONSE_TO_BIOTIC_STIMULUS	GO:0071216	8	-0.564782	-1.560310	0.035409	0.649493	1.000
690	GOBP_GLIAL_CELL_DIFFERENTIATION	GO:0010001	10	-0.533520	-1.560560	0.041225	0.666713	1.000
691	GOBP_POSITIVE_REGULATION_OF_GENE_EXPRESSION	GO:0010628	16	-0.466790	-1.564181	0.039282	0.671026	1.000
692	GOBP_INNATE_IMMUNE_RESPONSE	GO:0045087	7	-0.587317	-1.565076	0.033088	0.686940	1.000
693	GOBP_POSITIVE_REGULATION_OF_BIOSYNTHETIC_PROCESS	GO:0009891	27	-0.422646	-1.567580	0.034079	0.697514	1.000
694	GOBP_PROTEIN_MODIFICATION_BY_SMALL_PROTEIN_CON...	GO:0070647	7	-0.610735	-1.571455	0.020779	0.702460	1.000
695	GOBP_ANIMAL_ORGAN_MORPHOGENESIS	GO:0009887	12	-0.512549	-1.584745	0.034118	0.670030	1.000
696	GOBP_RESPONSE_TO_ORGANIC_CYCLIC_COMPOUND	GO:0014070	20	-0.450509	-1.591954	0.029412	0.662038	1.000
697	GOBP_RESPONSE_TO_NUTRIENT	GO:0007584	5	-0.685809	-1.592299	0.036096	0.683969	1.000
698	GOBP_SKELETAL_MUSCLE_ORGAN_DEVELOPMENT	GO:0060538	5	-0.662651	-1.592826	0.031496	0.707263	1.000
699	REACTOME_CELLULAR_SENESCENCE	R-RNO-2559583	6	-0.635771	-1.597409	0.029793	0.711672	1.000
700	GOBP_NEGATIVE_REGULATION_OF_BIOSYNTHETIC_PROCESS	GO:0009890	13	-0.501403	-1.597435	0.022910	0.740058	1.000
701	REACTOME_SENESCENCE_ASSOCIATED_SECRETORY_PHENO...	R-RNO-2559582	6	-0.635771	-1.604546	0.030303	0.734571	1.000
702	GOBP_FC_RECEPTOR_SIGNALING_PATHWAY	GO:0038093	8	-0.585707	-1.611141	0.024722	0.732085	1.000
703	GOBP_IMMUNE_RESPONSE_REGULATING_SIGNALING_PATHWAY	GO:000276	8	-0.585707	-1.615680	0.035714	0.743025	1.000
704	GOBP_MAPK_CASCADE	GO:0000165	18	-0.472144	-1.619633	0.031710	0.757535	1.000
705	GOBP_ACTIVATION_OF_IMMUNE_RESPONSE	GO:0002253	5	-0.686490	-1.622097	0.021136	0.782588	1.000

Continuation of Table **S10**

	NAME	ID	SIZE	ES	NES	NOM.p.val	FDR.q.val	FWER.p.val
706	GOBP_CELLULAR_RESPONSE_TO_EXTERNAL_STIMULUS	GO:0071496	10	-0.560067	-1.623904	0.023143	0.813685	1.000
707	GOBP_EMBRYO_DEVELOPMENT_ENDING_IN_BIRTH_OR_EGG...	GO:0009792	9	-0.575492	-1.633763	0.020408	0.803747	1.000
708	GOBP_CYTOKINE_MEDIATED_SIGNALING_PATHWAY	GO:0019221	16	-0.491688	-1.636183	0.022198	0.836990	1.000
709	GOBP_MUSCLE_TISSUE_DEVELOPMENT	GO:0060537	11	-0.541374	-1.639085	0.026590	0.873023	1.000
710	GOBP_NEGATIVE_REGULATION_OF_TRANSCRIPTION_BY_R...	GO:0000122	10	-0.564503	-1.639437	0.025362	0.929092	1.000
711	GOBP_DEVELOPMENTAL_PROCESS_INVOLVED_IN_REPRODU...	GO:0003006	11	-0.547095	-1.653222	0.019653	0.902553	1.000
712	GOBP_MATERNAL_PROCESS_INVOLVED_IN_FEMALE_PREGN...	GO:0060135	5	-0.710804	-1.665547	0.018692	0.892859	0.999
713	GOBP_SKIN_DEVELOPMENT	GO:0043588	5	-0.702984	-1.689212	0.015027	0.812773	0.998
714	GOBP_CIRCULATORY_SYSTEM_DEVELOPMENT	GO:0072359	21	-0.482465	-1.694184	0.015284	0.855002	0.998
715	GOBP_IN_UTERO_EMBRYONIC_DEVELOPMENT	GO:0001701	8	-0.618549	-1.697652	0.017566	0.916660	0.998
289	REACTOME_NGF_STIMULATED_TRANSCRIPTION	R-RNO-9031628	9	0.353930	0.942122	0.521880	0.901241	1.000
716	GOBP_RESPONSE_TO_EXTRACELLULAR_STIMULUS	GO:0009991	13	-0.531490	-1.703521	0.017281	0.976334	0.998
717	GOBP_REGULATION_OF_ORGAN_GROWTH	GO:0046620	5	-0.724434	-1.712719	0.009044	1.000000	0.996
718	GOBP_EMBRYONIC_ORGAN_DEVELOPMENT	GO:0048568	7	-0.659482	-1.715278	0.020000	1.000000	0.996
719	GOBP_ORGAN_GROWTH	GO:0035265	5	-0.724434	-1.718715	0.015424	1.000000	0.996
720	GOBP_EMBRYO_DEVELOPMENT	GO:0009790	14	-0.536858	-1.741192	0.021542	1.000000	0.996
721	GOBP_HEART_DEVELOPMENT	GO:0007507	6	-0.695437	-1.741786	0.010178	1.000000	0.996
722	GOBP_CARDIAC_MUSCLE_TISSUE_DEVELOPMENT	GO:0048738	6	-0.695437	-1.746402	0.010390	1.000000	0.996
723	GOBP_MULTICELLULAR_ORGANISM_REPRODUCTION	GO:0032504	12	-0.569524	-1.764001	0.014068	1.000000	0.992
724	REACTOME_PTEN_REGULATION	R-RNO-6807070	5	-0.754674	-1.777577	0.006402	1.000000	0.990

End of Table

Supplementary Table S11: CTX-M early profile (1 DPL peak) GSEA results.

Begin of Table **S11**

	NAME	ID	SIZE	ES	NES	NOM.p.val	FDR.q.val	FWER.p.val
0	GOBP_REGULATION_OF_NEURONAL_SYNAPTIC_PLASTICITY	GO:0048168	7	0.781099	1.916008	0.000000	0.512575	0.545
1	REACTOME_NEGATIVE_REGULATION_OF_NMDA_RECEPTOR_...	R-HSA-9617324	8	0.740255	1.915856	0.005226	0.257571	0.546
2	REACTOME_UNBLOCKING_OF_NMDA_RECEPTORS_GLUTAMAT...	R-RNO-438066	12	0.643641	1.885993	0.005367	0.231565	0.663
3	REACTOME_ASSEMBLY_AND_CELL_SURFACE_PRESENTATIO...	R-RNO-9609736	8	0.740255	1.870816	0.001812	0.201931	0.719
4	REACTOME_SYNAPTIC_ADHESION_LIKE_MOLECULES	R-RNO-8849932	9	0.652410	1.695269	0.013889	0.793753	1.000
5	REACTOME_RAS_ACTIVATION_UPON_CA2_INFLUX_THROUG...	R-HSA-442982	6	0.718701	1.666102	0.018416	0.828941	1.000
6	GOBP_CELL_SURFACE_RECEPTOR_SIGNALING_PATHWAY_I...	GO:1905114	15	0.526334	1.651526	0.024561	0.795491	1.000
7	REACTOME_LONG_TERM_POTENTIATION	R-HSA-9620244	10	0.591587	1.630903	0.031414	0.805733	1.000
8	REACTOME_TRANSMISSION_ACROSS_CHEMICAL_SYNAPSES	R-RNO-112315	23	0.468524	1.628466	0.026891	0.728449	1.000
9	REACTOME_ACTIVATION_OF_NMDA_RECEPTORS_AND_POST...	R-RNO-442755	16	0.513227	1.573137	0.028053	0.961150	1.000
10	GOBP_POSTSYNAPSE_ORGANIZATION	GO:0099173	10	0.566282	1.569294	0.043478	0.895008	1.000
11	REACTOME_NEURONAL_SYSTEM	R-RNO-112316	26	0.446338	1.561307	0.028862	0.866438	1.000
12	GOBP_RESPONSE_TO_ALCOHOL	GO:0097305	11	0.545978	1.559732	0.033748	0.806638	1.000
13	GOBP_REGULATION_OF_SYNAPTIC_PLASTICITY	GO:0048167	21	0.460294	1.558922	0.030822	0.753051	1.000
14	GOBP_RESPONSE_TO_ETHANOL	GO:0045471	5	0.718747	1.553164	0.033028	0.729094	1.000
15	REACTOME_MAPK_FAMILY_SIGNALING_CASCADES	R-RNO-5683057	12	0.526082	1.525754	0.049296	0.814406	1.000
16	GOBP_EXCITATORY_CHEMICAL_SYNAPTIC_TRANSMISSION	GO:0098976	5	0.703966	1.522089	0.049360	0.785421	1.000
17	REACTOME_NEUROTRANSMITTER_RECEPTORS_AND_POSTSY...	R-RNO-112314	22	0.438207	1.497067	0.043253	0.866216	1.000
18	REACTOME_PROTEIN_PROTEIN_INTERACTIONS_AT_SYNAPSES	R-RNO-6794362	12	0.523716	1.493978	0.066087	0.835384	1.000
19	GOBP_IONOTROPIC_GLUTAMATE_RECEPTOR_SIGNALING_P...	GO:0035235	9	0.556691	1.488562	0.084359	0.819220	1.000
20	GOBP_PROTEIN_LOCALIZATION_TO_CELL_JUNCTION	GO:1902414	6	0.646341	1.468410	0.068783	0.872393	1.000
21	GOBP_INORGANIC_ION_TRANSMEMBRANE_TRANSPORT	GO:0098660	9	0.550990	1.465765	0.069324	0.844717	1.000
22	REACTOME_ONCOGENIC_MAPK_SIGNALING	R-HSA-6802957	5	0.667239	1.454165	0.071813	0.862716	1.000
23	GOBP_REGULATION_OF_CELLULAR_COMPONENT_SIZE	GO:0032535	7	0.596441	1.441923	0.100000	0.883021	1.000
24	GOBP_REGULATION_OF_NMDA_RECEPTOR_ACTIVITY	GO:2000310	10	0.521728	1.434390	0.086799	0.882025	1.000
25	GOBP_PROTEIN_CONTAINING_COMPLEX_SUBUNIT_ORGANI...	GO:0043933	14	0.459858	1.409104	0.108202	0.971007	1.000
26	GOBP_LEARNING	GO:0007612	13	0.470597	1.401058	0.104167	0.972712	1.000
27	REACTOME_NEUREXINS_AND_NEUROLIGINS	R-RNO-6794361	9	0.532515	1.388430	0.125912	0.999461	1.000
28	GOBP_PROTEIN_COMPLEX_OLIGOMERIZATION	GO:0051259	6	0.590112	1.384438	0.110912	0.984565	1.000
29	GOBP_PROTEIN_LOCALIZATION_TO_CELL_PERIPHERY	GO:1990778	12	0.481015	1.382953	0.106618	0.958746	1.000
30	GOBP_PROTEIN_LOCALIZATION_TO_POSTSYNAPSE	GO:0062237	5	0.638554	1.380786	0.121528	0.937810	1.000
31	GOBP_PROTEIN_LOCALIZATION_TO_MEMBRANE	GO:0072657	14	0.451927	1.372858	0.116402	0.943251	1.000
32	GOBP_PROTEIN_LOCALIZATION_TO_SYNAPSE	GO:0035418	5	0.638554	1.368986	0.121324	0.931889	1.000
33	GOBP_CELLULAR_COMPONENT_MAINTENANCE	GO:0043954	5	0.626506	1.367283	0.128521	0.911429	1.000
34	GOBP_REGULATION_OF_POSTSYNAPTIC_MEMBRANE_POTEN...	GO:0060078	14	0.446026	1.359635	0.093379	0.918392	1.000
35	GOBP_REGULATION_OF_NEUROTRANSMITTER_RECEPTOR_A...	GO:0099601	13	0.453801	1.358894	0.108153	0.896114	1.000
36	GOBP_MONOAMINE_TRANSPORT	GO:0015844	5	0.615020	1.337279	0.155867	0.963628	1.000
37	GOBP_ASSOCIATIVE_LEARNING	GO:0008306	7	0.559081	1.327215	0.165517	0.982683	1.000
38	GOBP_CATION_TRANSPORT	GO:0006812	25	0.379935	1.326090	0.138514	0.962526	1.000
39	REACTOME_CREB1_PHOSPHORYLATION_THROUGH_NMDA_RE...	R-RNO-442742	8	0.520260	1.325481	0.149281	0.941185	1.000
40	GOBP_NEURON_PROJECTION_ORGANIZATION	GO:0106027	8	0.512422	1.322168	0.141869	0.932008	1.000

Continuation of Table **S11**

	NAME	ID	SIZE	ES	NES	NOM.p.val	FDR.q.val	FWER.p.val
41	GOBP_GENERATION_OF_PRECURSOR_METABOLITES_AND_E...	GO:0006091	5	0.608986	1.311754	0.157143	0.953841	1.000
42	GOBP_HOMEOSTASIS_OF_NUMBER_OF_CELLS	GO:0048872	5	0.594912	1.307946	0.160940	0.947470	1.000
43	GOBP_INNATE_IMMUNE_RESPONSE	GO:0045087	7	0.526377	1.283827	0.186125	1.000000	1.000
44	GOBP_GLUTAMATE_RECEPTOR_SIGNALING_PATHWAY	GO:0007215	17	0.399607	1.266181	0.174576	1.000000	1.000
45	REACTOME_CELLULAR_RESPONSES_TO_EXTERNAL_STIMULI	R-RNO-8953897	12	0.438862	1.252634	0.203327	1.000000	1.000
46	GOBP_RESPONSE_TO_NUTRIENT	GO:0007584	5	0.567904	1.247074	0.221223	1.000000	1.000
47	HALLMARK_PI3K_AKT_MTOR_SIGNALING	M5923	6	0.522204	1.227863	0.203936	1.000000	1.000
48	GOBP_RESPONSE_TO_ABIOTIC_STIMULUS	GO:0009628	27	0.336080	1.224115	0.190557	1.000000	1.000
49	GOBP_REGULATION_OF_ANATOMICAL_STRUCTURE_SIZE	GO:0090066	10	0.437734	1.207463	0.219684	1.000000	1.000
50	GOBP_REGULATION_OF_WNT_SIGNALING_PATHWAY	GO:0030111	5	0.548305	1.203603	0.254000	1.000000	1.000
51	GOBP_MALE_GAMETE_GENERATION	GO:0048232	5	0.548145	1.197118	0.248201	1.000000	1.000
52	GOBP_REGULATION_OF_POSTSYNAPSE_ORGANIZATION	GO:0099175	6	0.504298	1.181934	0.273205	1.000000	1.000
53	GOBP_REGULATION_OF_MEMBRANE_POTENTIAL	GO:0042391	17	0.363453	1.172667	0.249158	1.000000	1.000
54	GOBP_REGULATION_OF_METAL_ION_TRANSPORT	GO:0010959	6	0.511707	1.169342	0.274194	1.000000	1.000
55	GOBP_MEMBRANE_ORGANIZATION	GO:0061024	12	0.410460	1.167651	0.282098	1.000000	1.000
56	GOBP_NEUROTRANSMITTER_SECRETION	GO:0007269	6	0.502626	1.154663	0.288396	1.000000	1.000
57	GOBP_CATION_TRANSMEMBRANE_TRANSPORT	GO:0098655	18	0.354188	1.153508	0.273684	1.000000	1.000
58	GOBP_G_PROTEIN_COUPLED_RECEPTOR_SIGNALING_PATHWAY	GO:0007186	18	0.346863	1.151636	0.263934	1.000000	1.000
59	REACTOME_POST_TRANSLATIONAL_PROTEIN_MODIFICATION	R-RNO-597592	7	0.471931	1.147994	0.294872	1.000000	1.000
60	GOBP_DENDRITIC_SPINE_DEVELOPMENT	GO:0060996	6	0.511171	1.146298	0.329609	1.000000	1.000
61	REACTOME_INTERFERON_SIGNALING	R-RNO-913531	6	0.497862	1.141704	0.291367	1.000000	1.000
62	GOBP_DEVELOPMENTAL_MATURATION	GO:0021700	5	0.530720	1.131630	0.344178	1.000000	1.000
63	GOBP_REGULATION_OF_TRANSPORTER_ACTIVITY	GO:0032409	17	0.354141	1.131436	0.314721	1.000000	1.000
64	GOBP_ORGANIC_HYDROXY_COMPOUND_TRANSPORT	GO:0015850	6	0.495429	1.128347	0.326376	1.000000	1.000
65	GOBP_CHEMICAL_SYNAPTIC_TRANSMISSION_POSTSYNAPTIC	GO:0099565	9	0.430212	1.126542	0.306644	1.000000	1.000
66	REACTOME_MEMBRANE_TRAFFICKING	R-RNO-199991	5	0.516645	1.121580	0.341284	1.000000	1.000
67	GOBP_REGULATION_OF_CYTOSOLIC_CALCIUM_ION_CONCE...	GO:0051480	13	0.376914	1.118450	0.313406	1.000000	1.000
68	GOBP_REGULATION_OF_ION_TRANSPORT	GO:0043269	36	0.296374	1.116070	0.332149	1.000000	1.000
69	GOBP_DIVALENT_INORGANIC_CATION_HOMEOSTASIS	GO:0072507	13	0.376914	1.108401	0.326050	1.000000	1.000
70	GOBP_HEAD_DEVELOPMENT	GO:0060322	22	0.322022	1.107567	0.312187	1.000000	1.000
71	GOBP_CALCIUM_ION_TRANSPORT_INTO_CYTOSOL	GO:0060402	7	0.447161	1.107132	0.336237	1.000000	1.000
72	GOBP_METAL_ION_TRANSPORT	GO:0030001	11	0.380502	1.096673	0.332740	1.000000	1.000
73	GOBP_REGULATION_OF_SIGNALING_RECEPTOR_ACTIVITY	GO:0010469	14	0.365134	1.091687	0.351304	1.000000	1.000
74	REACTOME_TRANSCRIPTIONAL_REGULATION_BY_MECP2	R-RNO-8986944	7	0.449566	1.089911	0.353680	1.000000	1.000
75	REACTOME_INFECTIOUS_DISEASE	R-HSA-5663205	13	0.365399	1.088924	0.353041	1.000000	1.000
76	GOBP_CYTOSOLIC_CALCIUM_ION_TRANSPORT	GO:0060401	7	0.447161	1.087455	0.388785	1.000000	1.000
77	REACTOME_VESICLE_MEDIATED_TRANSPORT	R-RNO-5653656	5	0.516645	1.085773	0.372881	1.000000	1.000
78	GOBP_PROTEIN_LOCALIZATION_TO_PLASMA_MEMBRANE	GO:0072659	7	0.440613	1.084246	0.355240	1.000000	1.000
79	REACTOME_TRAFFICKING_OF_AMPA_RECEPTORS	R-RNO-399719	11	0.385754	1.084218	0.339789	1.000000	1.000
80	GOBP_RESPONSE_TO_DRUG	GO:0042493	11	0.379930	1.082146	0.361552	1.000000	1.000
81	GOBP_PEPTIDE_BIOSYNTHETIC_PROCESS	GO:0043043	7	0.440217	1.082037	0.350442	1.000000	1.000
82	GOBP_RESPIRATORY_SYSTEM_DEVELOPMENT	GO:0060541	6	0.460410	1.072992	0.379747	1.000000	1.000
83	GOBP_DEVELOPMENTAL_CELL_GROWTH	GO:0048588	5	0.499393	1.072022	0.386809	1.000000	1.000

Continuation of Table **S11**

	NAME	ID	SIZE	ES	NES	NOM.p.val	FDR.q.val	FWER.p.val
84	GOBP_CALCIUM_ION_TRANSPORT	GO:0006816	11	0.380502	1.071964	0.364791	1.000000	1.000
85	GOBP_SEXUAL_REPRODUCTION	GO:0019953	10	0.388175	1.070595	0.352740	1.000000	1.000
86	GOBP_LONG_TERM_SYNAPTIC_DEPRESSION	GO:0060292	5	0.496568	1.067658	0.385714	1.000000	1.000
87	GOBP_CALCIUM_ION_TRANSMEMBRANE_TRANSPORT	GO:0070588	7	0.447161	1.066191	0.366972	1.000000	1.000
88	GOBP_AMIDE_BIOSYNTHETIC_PROCESS	GO:0043604	7	0.440217	1.066106	0.403478	1.000000	1.000
89	REACTOME_DISEASES_OF_SIGNAL_TRANSDUCTION_BY_GR...	R-HSA-5663202	12	0.367294	1.066026	0.381944	1.000000	1.000
90	GOBP_NEGATIVE_REGULATION_OF_BINDING	GO:0051100	6	0.459380	1.061192	0.381125	1.000000	1.000
91	REACTOME_CIRCADIAN_CLOCK	R-HSA-400253	5	0.483531	1.058528	0.382784	1.000000	1.000
92	GOBP_ANION_TRANSMEMBRANE_TRANSPORT	GO:0098656	7	0.424702	1.053892	0.381282	1.000000	1.000
93	GOBP_REGULATION_OF_CELL_PROJECTION_ORGANIZATION	GO:0031344	20	0.320443	1.052005	0.384880	1.000000	1.000
94	GOBP_CELL_CELL_SIGNALING_BY_WNT	GO:0198738	7	0.432257	1.050591	0.418944	1.000000	1.000
95	GOBP_REGULATION_OF_CATION_TRANSMEMBRANE_TRANSPORT	GO:1904062	16	0.334915	1.044905	0.409241	1.000000	1.000
96	GOBP_NEGATIVE_REGULATION_OF_SYNAPTIC_TRANSMISSION	GO:0050805	6	0.442020	1.039826	0.433526	1.000000	1.000
97	GOBP_POSITIVE_REGULATION_OF_CELL_PROJECTION_OR...	GO:0031346	10	0.381644	1.033391	0.425225	1.000000	1.000
98	GOBP_REGULATION_OF_NEUROTRANSMITTER_LEVELS	GO:0001505	7	0.422952	1.031098	0.415301	1.000000	1.000
99	GOBP_REGULATION_OF_TRANSPORT	GO:0051049	44	0.267789	1.028635	0.411664	1.000000	1.000
100	GOBP_METAL_ION_HOMEOSTASIS	GO:0055065	14	0.344780	1.027953	0.427877	1.000000	1.000
101	GOBP_REGULATION_OF_CELL_SIZE	GO:0008361	5	0.481054	1.027845	0.433824	1.000000	1.000
102	GOBP_NEURON_DEVELOPMENT	GO:0048666	33	0.280429	1.026671	0.427336	1.000000	1.000
103	GOBP_NEUROTRANSMITTER_TRANSPORT	GO:0006836	7	0.422952	1.022985	0.428829	1.000000	1.000
104	GOBP_REGULATION_OF_NEUROTRANSMITTER_TRANSPORT	GO:0051588	6	0.441118	1.019756	0.440741	1.000000	1.000
105	GOBP_SENSORY_PERCEPTION	GO:0007600	9	0.380311	1.017152	0.441331	1.000000	1.000
106	GOBP_NEUROGENESIS	GO:0022008	40	0.267049	1.012745	0.444631	1.000000	1.000
107	GOBP_RESPONSE_TO_OXYGEN_LEVELS	GO:0070482	8	0.398507	1.004018	0.432916	1.000000	1.000
108	GOBP_CENTRAL_NERVOUS_SYSTEM_DEVELOPMENT	GO:0007417	26	0.279412	0.991034	0.466667	1.000000	1.000
109	GOBP_TRANSMEMBRANE_TRANSPORT	GO:0055085	27	0.276427	0.984567	0.480607	1.000000	1.000
110	GOBP_REGULATION_OF_PROTEIN_BINDING	GO:0043393	7	0.400157	0.979250	0.487455	1.000000	1.000
111	REACTOME_L1CAM_INTERACTIONS	R-RNO-373760	5	0.446396	0.977406	0.475564	1.000000	1.000
112	GOBP_IMPORT_INTO_CELL	GO:0098657	6	0.421627	0.973758	0.497345	1.000000	1.000
113	GOBP_DEFENSE_RESPONSE_TO_OTHER_ORGANISM	GO:0098542	9	0.352219	0.972414	0.470270	1.000000	1.000
114	GOBP_RESPONSE_TO_MECHANICAL_STIMULUS	GO:0009612	6	0.412262	0.968258	0.494624	1.000000	1.000
115	GOBP_CELLULAR_RESPONSE_TO_HORMONE_STIMULUS	GO:0032870	11	0.342638	0.965464	0.510714	1.000000	1.000
116	GOBP_EPHRIN_RECEPTOR_SIGNALING_PATHWAY	GO:0048013	5	0.440017	0.958411	0.491773	1.000000	1.000
117	GOBP_ADENYLATE_CYCLASE_INHIBITING_G_PROTEIN_CO...	GO:0007193	9	0.353721	0.957431	0.488496	1.000000	1.000
118	GOBP_NEURON_DIFFERENTIATION	GO:0030182	36	0.256198	0.953390	0.553366	1.000000	1.000
119	GOBP_NEGATIVE_REGULATION_OF_MOLECULAR_FUNCTION	GO:0044092	24	0.281609	0.950693	0.527919	1.000000	1.000
120	GOBP_CELLULAR_RESPONSE_TO_PEPTIDE_HORMONE_STIM...	GO:0071375	9	0.351531	0.950209	0.521515	1.000000	1.000
121	GOBP_MORPHOGENESIS_OF_AN_EPITHELIUM	GO:0002009	6	0.407845	0.949197	0.501742	1.000000	1.000
122	GOBP_ORGANONITROGEN_COMPOUND_BIOSYNTHETIC_PROCESS	GO:1901566	13	0.312304	0.944903	0.540587	1.000000	1.000
123	GOBP_VESICLE_MEDIATED_TRANSPORT_IN_SYNAPSE	GO:0099003	7	0.387115	0.934607	0.530797	1.000000	1.000
124	REACTOME_SIGNALING_BY_GPCR	R-RNO-372790	22	0.269010	0.919776	0.548231	1.000000	1.000
125	GOBP_REGULATION_OF_PROTEIN_LOCALIZATION_TO_CEL...	GO:1904375	6	0.397394	0.916372	0.553571	1.000000	1.000
126	GOBP_POSITIVE_REGULATION_OF_CELL_DEATH	GO:0010942	19	0.275996	0.911815	0.585366	1.000000	1.000

Continuation of Table **S11**

	NAME	ID	SIZE	ES	NES	NOM.p.val	FDR.q.val	FWER.p.val
127	GOBP_REGULATION_OF_CATION_CHANNEL_ACTIVITY	GO:2001257	14	0.306712	0.910644	0.568873	1.000000	1.000
128	GOBP_MULTICELLULAR_ORGANISMAL_HOMEOSTASIS	GO:0048871	12	0.317023	0.910180	0.575916	1.000000	1.000
129	GOBP_PEPTIDE_METABOLIC_PROCESS	GO:0006518	12	0.310859	0.903648	0.583190	1.000000	1.000
130	GOBP_TISSUE_HOMEOSTASIS	GO:0001894	5	0.423233	0.902167	0.578947	1.000000	1.000
131	GOBP_CELLULAR_ION_HOMEOSTASIS	GO:0006873	14	0.293939	0.901647	0.595745	1.000000	1.000
132	GOBP_RESPONSE_TO_MONOSACCHARIDE	GO:0034284	5	0.412327	0.900519	0.604736	1.000000	1.000
133	GOBP_CELL_PROJECTION_ORGANIZATION	GO:0030030	34	0.240126	0.893037	0.636852	1.000000	1.000
134	REACTOME_EPH_EPHRIN_SIGNALING	R-RNO-2682334	6	0.383213	0.886275	0.597450	1.000000	1.000
135	GOBP_RESPONSE_TO_CARBOHYDRATE	GO:0009743	5	0.412327	0.884967	0.610811	1.000000	1.000
136	GOBP_GAMETE_GENERATION	GO:0007276	9	0.332485	0.883397	0.607509	1.000000	1.000
137	GOBP_ENDOCRINE_SYSTEM_DEVELOPMENT	GO:0035270	6	0.384554	0.882929	0.629964	1.000000	1.000
138	GOBP_CELLULAR_AMIDE_METABOLIC_PROCESS	GO:0043603	13	0.295258	0.878717	0.612628	1.000000	1.000
139	GOBP_CARBOHYDRATE_METABOLIC_PROCESS	GO:0005975	9	0.334131	0.877108	0.614498	1.000000	1.000
140	GOBP_CELLULAR_RESPONSE_TO_ALCOHOL	GO:0097306	5	0.414001	0.875581	0.624329	1.000000	1.000
141	GOBP_LOCOMOTORY_BEHAVIOR	GO:0007626	7	0.360912	0.868117	0.625688	1.000000	1.000
142	GOBP_REGULATION_OF_ANION_TRANSMEMBRANE_TRANSPORT	GO:1903959	5	0.402034	0.862474	0.642322	1.000000	1.000
143	GOBP_TELENCEPHALON_DEVELOPMENT	GO:0021537	6	0.369488	0.856174	0.629565	1.000000	1.000
144	GOBP_POSITIVE_REGULATION_OF_PEPTIDASE_ACTIVITY	GO:0010952	7	0.344226	0.845646	0.646739	1.000000	1.000
145	GOBP_MONONUCLEAR_CELL_DIFFERENTIATION	GO:1903131	7	0.347866	0.844114	0.625862	1.000000	1.000
146	GOBP_TISSUE_MORPHOGENESIS	GO:0048729	7	0.344560	0.842446	0.642606	1.000000	1.000
147	GOBP_RESPONSE_TO_PEPTIDE_HORMONE	GO:0043434	13	0.280000	0.842190	0.662752	1.000000	1.000
148	GOBP_PROTEIN_MODIFICATION_BY_SMALL_PROTEIN_CON...	GO:0070647	7	0.345390	0.840351	0.661080	1.000000	1.000
149	GOBP_REGULATION_OF_LONG_TERM_SYNAPTIC_POTENTIA...	GO:1900271	6	0.365854	0.838525	0.646956	1.000000	1.000
150	GOBP_CELL_GROWTH	GO:0016049	10	0.300048	0.837426	0.689119	1.000000	1.000
151	GOBP_POSITIVE_REGULATION_OF_CYSTEINE_TYPE_ENDO...	GO:2001056	7	0.344226	0.835953	0.686238	1.000000	1.000
152	REACTOME_DEVELOPMENTAL_BIOLOGY	R-RNO-1266738	23	0.245577	0.833641	0.701493	1.000000	1.000
153	REACTOME_ANTI_INFLAMMATORY_RESPONSE_FAVOURING_...	R-HSA-9662851	5	0.385542	0.826516	0.678571	1.000000	1.000
154	GOBP_HOMEOSTATIC_PROCESS	GO:0042592	31	0.228752	0.825424	0.720588	1.000000	1.000
155	REACTOME_TRANSCRIPTIONAL_REGULATION_OF_WHITE_A...	R-RNO-381340	6	0.357542	0.822755	0.687713	1.000000	1.000
156	GOBP_REGULATION_OF_TRANSMEMBRANE_TRANSPORT	GO:0034762	23	0.238610	0.820490	0.722615	1.000000	1.000
157	GOBP_POLYSACCHARIDE_METABOLIC_PROCESS	GO:0005976	5	0.378159	0.813496	0.680702	1.000000	1.000
158	GOBP_POSITIVE_REGULATION_OF_PROTEOLYSIS	GO:0045862	8	0.315445	0.807562	0.705776	1.000000	1.000
159	GOBP_REGULATION_OF_HORMONE_LEVELS	GO:0010817	12	0.276045	0.805861	0.719595	1.000000	1.000
160	GOBP_CELLULAR_MACROMOLECULE_LOCALIZATION	GO:0070727	21	0.241402	0.805852	0.718519	1.000000	1.000
161	GOBP_CELLULAR_RESPONSE_TO_KETONE	GO:1901655	5	0.373494	0.800468	0.697133	1.000000	1.000
162	GOBP_DENDRITIC_SPINE_MORPHOGENESIS	GO:0060997	5	0.374724	0.800062	0.703704	1.000000	1.000
163	GOBP_MONOCARBOXYLIC_ACID_METABOLIC_PROCESS	GO:0032787	6	0.347766	0.796355	0.725314	1.000000	1.000
164	GOBP_PROTEOLYSIS	GO:0006508	17	0.249834	0.795647	0.730570	1.000000	1.000
165	REACTOME_NGF_STIMULATED_TRANSCRIPTION	R-RNO-9031628	9	0.303798	0.795264	0.717813	1.000000	1.000
166	GOBP_POSITIVE_REGULATION_OF_SECRETION	GO:0051047	6	0.342337	0.792974	0.719643	1.000000	1.000
167	GOBP_CELL_JUNCTION_ORGANIZATION	GO:0034330	19	0.237372	0.786197	0.767327	1.000000	1.000
168	GOBP_REGULATION_OF_TRANS_SYNAPTIC_SIGNALING	GO:0099177	40	0.205484	0.784573	0.801639	1.000000	1.000
169	GOBP_CELL_CELL_JUNCTION_ASSEMBLY	GO:0007043	5	0.365342	0.784135	0.723133	1.000000	1.000

Continuation of Table **S11**

	NAME	ID	SIZE	ES	NES	NOM.p.val	FDR.q.val	FWER.p.val
170	GOBP_CALCIUM_MEDIATED_SIGNALING	GO:0019722	10	0.279283	0.779990	0.737881	1.000000	1.000
171	GOBP_POSITIVE_REGULATION_OF_LONG_TERM_SYNAPTIC...	GO:1900273	5	0.361446	0.777931	0.726433	1.000000	1.000
172	GOBP_LIPID_EXPORT_FROM_CELL	GO:0140353	5	0.356493	0.772829	0.710383	1.000000	1.000
173	GOBP_SYNAPSE_ORGANIZATION	GO:0050808	17	0.239357	0.766052	0.786078	1.000000	1.000
174	GOBP_NEGATIVE_REGULATION_OF_KINASE_ACTIVITY	GO:0033673	5	0.345566	0.764877	0.732394	1.000000	1.000
175	GOBP_ACTIVATION_OF_IMMUNE_RESPONSE	GO:0002253	5	0.348754	0.759090	0.750439	1.000000	1.000
176	GOBP_IMMUNE_EFFECTOR_PROCESS	GO:0002252	10	0.274678	0.758344	0.756554	1.000000	1.000
177	GOBP_NEGATIVE_REGULATION_OF_TRANSFERASE_ACTIVITY	GO:0051348	5	0.345566	0.757334	0.769088	1.000000	1.000
178	GOBP_EPITHELIUM_DEVELOPMENT	GO:0060429	15	0.245003	0.757081	0.799325	1.000000	1.000
179	GOBP_CIRCADIAN_REGULATION_OF_GENE_EXPRESSION	GO:0032922	5	0.345256	0.754532	0.771325	1.000000	1.000
180	GOBP_ION_HOMEOSTASIS	GO:0050801	16	0.236438	0.749590	0.816949	1.000000	1.000
181	REACTOME_DAG_AND_IP3_SIGNALING	R-RNO-1489509	8	0.297147	0.749121	0.772487	1.000000	1.000
182	GOBP_CHROMATIN_ORGANIZATION	GO:0006325	5	0.351723	0.748818	0.794118	1.000000	1.000
183	GOBP_ORGANIC_ANION_TRANSPORT	GO:0015711	6	0.316344	0.732696	0.774194	1.000000	1.000
184	GOBP_EXTRINSIC_APOPTOTIC_SIGNALING_PATHWAY	GO:0097191	7	0.300851	0.731105	0.789931	1.000000	1.000
185	GOBP_ORGANIC_ACID_TRANSPORT	GO:0015849	7	0.297027	0.729627	0.798932	1.000000	1.000
186	GOBP_CARBOXYLIC_ACID_TRANSPORT	GO:0046942	6	0.316344	0.729463	0.819421	1.000000	1.000
187	GOBP_REGULATION_OF_NEURON_PROJECTION_DEVELOPMENT	GO:0010975	16	0.232202	0.729073	0.805604	1.000000	1.000
188	GOBP_DEFENSE_RESPONSE	GO:0006952	19	0.223639	0.727821	0.818182	1.000000	1.000
189	REACTOME_LEISHMANIA_INFECTION	R-HSA-9658195	10	0.257005	0.721314	0.812174	1.000000	1.000
190	GOBP_REGULATION_OF_RECEPTOR_MEDIATED_ENDOCYTOSIS	GO:0048259	5	0.327154	0.720272	0.807760	1.000000	1.000
191	GOBP_AMINO_ACID_TRANSPORT	GO:0006865	6	0.316344	0.719928	0.797891	1.000000	1.000
192	GOBP_REGULATION_OF_BINDING	GO:0051098	11	0.261227	0.717951	0.826087	1.000000	1.000
193	GOBP_REGULATION_OF_NERVOUS_SYSTEM_PROCESS	GO:0031644	8	0.277634	0.717705	0.802768	1.000000	1.000
194	GOBP_REGULATION_OF_MUSCLE_CELL_DIFFERENTIATION	GO:0051147	6	0.308707	0.717231	0.828671	1.000000	1.000
195	GOBP_AXON_DEVELOPMENT	GO:0061564	18	0.222157	0.715808	0.813067	1.000000	1.000
196	GOBP_REGULATION_OF_PROTEIN_TYROSINE_KINASE_ACT...	GO:0061097	5	0.331277	0.712493	0.815838	1.000000	1.000
197	GOBP_CELLULAR_HOMEOSTASIS	GO:0019725	18	0.212137	0.707958	0.855670	1.000000	1.000
198	GOBP_CELLULAR_CARBOHYDRATE_METABOLIC_PROCESS	GO:0044262	5	0.331203	0.705829	0.818182	1.000000	1.000
199	GOBP_POSITIVE_REGULATION_OF_PROTEIN_TYROSINE_K...	GO:0061098	5	0.331277	0.705551	0.825137	1.000000	1.000
200	GOBP_CELL_CELL_SIGNALING	GO:0007267	56	0.178555	0.705300	0.881034	1.000000	1.000
201	GOBP_POSITIVE_REGULATION_OF_DNA_BINDING_TRANSC...	GO:0051091	10	0.257131	0.699441	0.843310	1.000000	1.000
202	GOBP_RESPONSE_TO_LIGHT_STIMULUS	GO:0009416	11	0.246059	0.698442	0.842105	1.000000	1.000
203	GOBP_CELL_MORPHOGENESIS_INVOLVED_IN_NEURON_DIF...	GO:0048667	21	0.204781	0.688575	0.880471	1.000000	1.000
204	GOBP_CELL_MORPHOGENESIS_INVOLVED_IN_DIFFERENTI...	GO:0000904	21	0.204781	0.688382	0.899174	1.000000	1.000
205	GOBP_POSITIVE_REGULATION_OF_HYDROLASE_ACTIVITY	GO:0051345	16	0.218988	0.688054	0.893103	1.000000	1.000
206	GOBP_CELL_CELL_JUNCTION_ORGANIZATION	GO:0045216	6	0.300433	0.683021	0.849732	1.000000	1.000
207	GOBP_NERVOUS_SYSTEM_PROCESS	GO:0050877	38	0.177821	0.679960	0.915398	1.000000	1.000
208	GOBP_ORGANIC_ACID_METABOLIC_PROCESS	GO:0006082	7	0.271215	0.664879	0.862917	1.000000	1.000
209	GOBP_CHEMICAL_HOMEOSTASIS	GO:0048878	23	0.192896	0.659706	0.917763	1.000000	1.000
210	REACTOME_DEATH_RECEPTOR_SIGNALLING	R-RNO-73887	5	0.302565	0.655314	0.878049	1.000000	1.000
211	GOBP_POSITIVE_REGULATION_OF_CELL_GROWTH	GO:0030307	6	0.286138	0.654587	0.881119	1.000000	1.000
212	GOBP_REGULATION_OF_CARBOHYDRATE_METABOLIC_PROCESS	GO:0006109	5	0.303491	0.653217	0.888889	1.000000	1.000

Continuation of Table **S11**

	NAME	ID	SIZE	ES	NES	NOM.p.val	FDR.q.val	FWER.p.val
213	GOBP_RECEPTOR_INTERNALIZATION	GO:0031623	5	0.298956	0.652523	0.902256	1.000000	1.000
214	GOBP_REGULATION_OF_CARBOHYDRATE_BIOSYNTHETIC_P...	GO:0043255	5	0.303491	0.651037	0.876977	1.000000	1.000
215	GOBP_CARBOHYDRATE_BIOSYNTHETIC_PROCESS	GO:0016051	5	0.303491	0.649120	0.893382	1.000000	1.000
216	GOBP_ORGANIC_HYDROXY_COMPOUND_METABOLIC_PROCESS	GO:1901615	7	0.260367	0.640437	0.904425	1.000000	1.000
217	GOBP_RESPONSE_TO_ORGANIC_CYCLIC_COMPOUND	GO:0014070	20	0.188783	0.640182	0.934319	1.000000	1.000
218	GOBP_RECEPTOR_METABOLIC_PROCESS	GO:0043112	5	0.298956	0.639929	0.908425	1.000000	1.000
219	GOBP_PURINE_CONTAINING_COMPOUND_METABOLIC_PROCESS	GO:0072521	5	0.296816	0.636404	0.903108	1.000000	1.000
220	GOBP_REGULATION_OF_AXONOGENESIS	GO:0050770	7	0.262879	0.631115	0.916667	1.000000	1.000
221	GOBP_RESPONSE_TO_HORMONE	GO:0009725	20	0.187325	0.625584	0.928934	1.000000	1.000
222	HALLMARK_EPITHELIAL_MESENCHYMAL_TRANSITION	M5930	5	0.292480	0.625527	0.904315	1.000000	1.000
223	GOBP_COGNITION	GO:0050890	28	0.176986	0.624492	0.939597	1.000000	1.000
224	GOBP_CARBOHYDRATE_DERIVATIVE_METABOLIC_PROCESS	GO:1901135	7	0.254496	0.622185	0.933333	1.000000	1.000
225	GOBP_ADENYLATE_CYCLASE_MODULATING_G_PROTEIN_CO...	GO:0007188	11	0.220115	0.619251	0.908475	1.000000	1.000
226	GOBP_REGULATION_OF_PROTEIN_LOCALIZATION_TO_MEM...	GO:1905475	7	0.255202	0.617966	0.938849	1.000000	1.000
227	GOBP_LONG_TERM_MEMORY	GO:0007616	6	0.268293	0.616842	0.907749	1.000000	1.000
228	GOBP_MUSCLE_CONTRACTION	GO:0006936	5	0.274263	0.603266	0.932331	1.000000	1.000
229	GOBP_POSITIVE_REGULATION_OF_IMMUNE_RESPONSE	GO:0050778	9	0.224443	0.602341	0.941712	1.000000	1.000
230	GOBP_G_PROTEIN_COUPLED_GLUTAMATE_RECEPTOR_SIGN...	GO:0007216	8	0.233191	0.593848	0.961739	1.000000	1.000
231	HALLMARK_KRAS_SIGNALING_UP	M5953	6	0.256678	0.590334	0.950368	1.000000	1.000
232	GOBP_REGULATION_OF_CYSTEINE_TYPE_ENDOPEPTIDASE...	GO:2000116	9	0.222544	0.580748	0.942761	1.000000	1.000
233	GOBP_CELLULAR_RESPONSE_TO_PEPTIDE	GO:1901653	13	0.184918	0.563296	0.955556	1.000000	1.000
234	REACTOME_TOLL_LIKE_RECEPTOR_CASCADES	R-RNO-168898	8	0.214829	0.561910	0.965950	1.000000	1.000
235	REACTOME_TOLL_LIKE_RECEPTOR_TLR1_TLR2_CASCADE	R-RNO-168179	8	0.214829	0.560567	0.959664	1.000000	1.000
236	GOBP_REGULATION_OF_LIPID_LOCALIZATION	GO:1905952	5	0.262895	0.557740	0.958042	1.000000	1.000
237	GOBP_CYTOKINE_MEDIATED_SIGNALING_PATHWAY	GO:0019221	16	0.179934	0.555415	0.956294	1.000000	1.000
238	REACTOME_MYD88_INDEPENDENT_TLR4_CASCADE	R-RNO-166166	8	0.214829	0.554786	0.957895	1.000000	1.000
239	GOBP_REGULATION_OF_PEPTIDASE_ACTIVITY	GO:0052547	10	0.201975	0.553361	0.954466	1.000000	1.000
240	REACTOME_CYTOKINE_SIGNALING_IN_IMMUNE_SYSTEM	R-RNO-1280215	21	0.164324	0.549787	0.974705	1.000000	1.000
241	REACTOME_GPCR_LIGAND_BINDING	R-RNO-500792	8	0.214718	0.549163	0.968921	1.000000	1.000
242	REACTOME_TOLL_LIKE_RECEPTOR_9_TLR9_CASCADE	R-RNO-168138	8	0.214829	0.548355	0.974271	1.000000	1.000
243	HALLMARK_UV_RESPONSE_UP	M5941	5	0.253012	0.546216	0.964413	1.000000	1.000
244	REACTOME_RNA_POLYMERASE_II_TRANSCRIPTION	R-RNO-73857	20	0.164587	0.545866	0.978477	1.000000	1.000
245	GOBP_SYNAPTIC_SIGNALING	GO:0099536	47	0.143227	0.540769	0.978477	1.000000	1.000
246	GOBP_RESPONSE_TO_RADIATION	GO:0009314	13	0.178188	0.535104	0.968366	1.000000	1.000
247	REACTOME_CLASS_C_3_METABOTROPIC_GLUTAMATE_PHER...	R-RNO-420499	7	0.220433	0.534279	0.976991	1.000000	1.000
248	GOBP_REGULATION_OF_SYNAPTIC_TRANSMISSION_GLUTA...	GO:0051966	12	0.182611	0.529528	0.978078	1.000000	1.000
249	GOBP_SYNAPTIC_TRANSMISSION_GLUTAMATERGIC	GO:0035249	13	0.173333	0.520766	0.986014	1.000000	1.000
250	GOBP_RECEPTOR_MEDIATED_ENDOCYTOSIS	GO:0006898	6	0.227034	0.519119	0.983842	1.000000	1.000
251	GOBP_BEHAVIOR	GO:0007610	32	0.140793	0.518516	0.986820	1.000000	1.000
252	GOBP_MEMORY	GO:0007613	13	0.167040	0.497877	0.986755	1.000000	1.000
253	GOBP_POSITIVE_REGULATION_OF_APOPTOTIC_SIGNALIN...	GO:2001235	5	0.224829	0.492571	0.988743	1.000000	1.000
254	GOBP_CELLULAR_RESPONSE_TO_ORGANIC_CYCLIC_COMPOUND	GO:0071407	13	0.162668	0.484457	0.986159	1.000000	1.000
255	GOBP_NEGATIVE_REGULATION_OF_INTRACELLULAR_SIGN...	GO:1902532	7	0.194301	0.476022	0.990942	1.000000	1.000

Continuation of Table **S11**

	NAME	ID	SIZE	ES	NES	NOM.p.val	FDR.q.val	FWER.p.val
256	GOBP_BIOLOGICAL_PROCESS_INVOLVED_IN_SYMBIOTIC_...	GO:004440	11	0.162939	0.465491	0.991119	0.998179	1.000
257	GOBP_NEGATIVE_REGULATION_OF_CELL_CYCLE	GO:0045786	5	0.211429	0.458665	0.996344	0.995238	1.000
258	GOBP_NEGATIVE_REGULATION_OF_PHOSPHORYLATION	GO:0042326	7	-0.173870	-0.437660	1.000000	0.998710	1.000
259	GOBP_MAINTENANCE_OF_LOCATION	GO:0051235	7	-0.180946	-0.463343	1.000000	0.998949	1.000
260	GOBP_MULTICELLULAR_ORGANISM_REPRODUCTION	GO:0032504	12	-0.156481	-0.480749	0.995283	0.999093	1.000
261	GOBP_REPRODUCTION	GO:0000003	18	-0.155263	-0.535406	0.987469	0.988415	1.000
262	GOBP_SKIN_DEVELOPMENT	GO:0043588	5	-0.254108	-0.581444	0.962306	0.969275	1.000
263	GOBP_NEGATIVE_REGULATION_OF_LIPID_METABOLIC_PR...	GO:0045833	5	-0.253012	-0.582144	0.952703	0.971020	1.000
264	REACTOME_TRAFFICKING_OF_GLUR2_CONTAINING_AMPA_...	R-RNO-416993	8	-0.215549	-0.585660	0.938636	0.971191	1.000
265	GOBP_REGULATION_OF_CELL_CYCLE	GO:0051726	13	-0.184345	-0.587451	0.957143	0.972236	1.000
266	GOBP_TEMPERATURE_HOMEOSTASIS	GO:0001659	5	-0.254099	-0.591173	0.942350	0.971958	1.000
267	GOBP_RESPONSE_TO_INORGANIC_SUBSTANCE	GO:0010035	19	-0.170556	-0.606566	0.958025	0.963125	1.000
268	GOBP_REGULATION_OF_GROWTH	GO:0040008	13	-0.191088	-0.607055	0.939675	0.964928	1.000
269	GOBP_POSITIVE_REGULATION_OF_GLIOGENESIS	GO:0014015	5	-0.269114	-0.607879	0.937360	0.966565	1.000
270	GOBP_REGULATION_OF_GLIOGENESIS	GO:0014013	5	-0.269114	-0.609859	0.926339	0.967185	1.000
271	GOBP_LIPID_BIOSYNTHETIC_PROCESS	GO:0008610	6	-0.256098	-0.611202	0.921839	0.968196	1.000
272	GOBP_POSITIVE_REGULATION_OF_DEFENSE_RESPONSE	GO:0031349	5	-0.269493	-0.612292	0.944444	0.969623	1.000
273	GOBP_RESPONSE_TO_CYTOKINE	GO:0034097	23	-0.166121	-0.620586	0.950980	0.965125	1.000
274	GOBP_REGULATION_OF_LIPID_BIOSYNTHETIC_PROCESS	GO:0046890	6	-0.256098	-0.623806	0.943182	0.964544	1.000
275	GOBP_LIPID_LOCALIZATION	GO:0010876	10	-0.217944	-0.631622	0.932432	0.959707	1.000
276	GOBP_MEMBRANE_PROTEIN_PROTEOLYSIS	GO:0033619	6	-0.256098	-0.633327	0.913979	0.960213	1.000
277	GOBP_REGULATION_OF_NEURON_DIFFERENTIATION	GO:0045664	5	-0.284848	-0.643041	0.881944	0.952922	1.000
278	GOBP_DEVELOPMENTAL_PROCESS_INVOLVED_IN_REPRODU...	GO:0003006	11	-0.215800	-0.649973	0.912621	0.947936	1.000
279	GOBP_NEGATIVE_REGULATION_OF_TRANSCRIPTION_BY_R...	GO:0000122	10	-0.226968	-0.655576	0.882793	0.944404	1.000
280	GOBP_REGULATION_OF_PROTEOLYSIS	GO:0030162	12	-0.208271	-0.657235	0.916667	0.944770	1.000
281	GOBP_MYELOID_LEUKOCYTE_MEDIATED_IMMUNITY	GO:0002444	6	-0.269659	-0.660083	0.867882	0.944001	1.000
282	GOBP_POSITIVE_REGULATION_OF_CYTOKINE_PRODUCTION	GO:0001819	7	-0.259259	-0.665538	0.885906	0.940047	1.000
283	GOBP_CELL_ACTIVATION_INVOLVED_IN_IMMUNE_RESPONSE	GO:0002263	6	-0.269659	-0.667967	0.873239	0.939627	1.000
284	REACTOME_NEUTROPHIL_DEGRANULATION	R-RNO-6798695	6	-0.269659	-0.668550	0.895455	0.941036	1.000
285	GOBP_SENSORY_ORGAN_DEVELOPMENT	GO:0007423	8	-0.247295	-0.671180	0.877996	0.940182	1.000
286	GOBP_MACROMOLECULE_CATABOLIC_PROCESS	GO:0009057	12	-0.215408	-0.675545	0.890215	0.937450	1.000
287	GOBP_PROTEIN_CATABOLIC_PROCESS	GO:0030163	8	-0.256322	-0.677363	0.866051	0.937491	1.000
288	GOBP_REGULATION_OF_LIPID_METABOLIC_PROCESS	GO:0019216	7	-0.259259	-0.677878	0.841014	0.938894	1.000
289	REACTOME_NERVOUS_SYSTEM_DEVELOPMENT	R-RNO-9675108	15	-0.204562	-0.681852	0.871122	0.936345	1.000
290	GOBP_FATTY_ACID_TRANSPORT	GO:0015908	6	-0.283584	-0.684116	0.848233	0.935664	1.000
291	GOBP_REGULATION_OF_DNA_BINDING_TRANSCRIPTION_F...	GO:0051090	16	-0.203320	-0.688312	0.880488	0.932763	1.000
292	GOBP_REGULATION_OF_LYMPHOCYTE_ACTIVATION	GO:0051249	5	-0.301205	-0.688983	0.856818	0.934121	1.000
293	GOBP_RESPONSE_TO_PEPTIDE	GO:1901652	18	-0.193621	-0.690701	0.903890	0.934360	1.000
294	GOBP_RESPONSE_TO_BIOTIC_STIMULUS	GO:0009607	17	-0.204443	-0.692723	0.866828	0.934012	1.000
295	GOBP_NEGATIVE_REGULATION_OF_IMMUNE_SYSTEM_PROCESS	GO:0002683	5	-0.301205	-0.694311	0.842105	0.934121	1.000
296	GOBP_MONOCARBOXYLIC_ACID_TRANSPORT	GO:0015718	5	-0.308902	-0.695608	0.851598	0.934644	1.000
297	GOBP_RESPONSE_TO_INTERLEUKIN_1	GO:0070555	5	-0.307242	-0.695624	0.832589	0.936830	1.000
298	GOBP_GLUCOSE_METABOLIC_PROCESS	GO:0006006	6	-0.293253	-0.698087	0.828947	0.935812	1.000

Continuation of Table **S11**

	NAME	ID	SIZE	ES	NES	NOM.p.val	FDR.q.val	FWER.p.val
299	GOBP_ACIDIC_AMINO_ACID_TRANSPORT	GO:0015800	5	-0.308902	-0.701007	0.813901	0.934403	1.000
300	REACTOME_INNATE_IMMUNE_SYSTEM	R-RNO-168249	16	-0.204625	-0.702432	0.873418	0.934737	1.000
301	GOBP_DICARBOXYLIC_ACID_TRANSPORT	GO:0006835	5	-0.308902	-0.703640	0.827887	0.935347	1.000
302	GOBP_ANATOMICAL_STRUCTURE_HOMEOSTASIS	GO:0060249	7	-0.279928	-0.707145	0.836559	0.932640	1.000
303	GOBP_REGULATION_OF_EXTRINSIC_APOPTOTIC_SIGNALI...	GO:2001236	6	-0.294985	-0.712982	0.794702	0.927117	1.000
304	GOBP_MONOSACCHARIDE_METABOLIC_PROCESS	GO:0005996	6	-0.293253	-0.723077	0.816742	0.914954	1.000
305	REACTOME_INTERLEUKIN_17_SIGNALING	R-RNO-448424	6	-0.298419	-0.726614	0.811927	0.912056	1.000
306	GOBP_MUSCLE_CELL_DEVELOPMENT	GO:0055001	5	-0.320854	-0.728444	0.809313	0.911832	1.000
307	GOBP_LYMPHOCYTE_ACTIVATION	GO:0046649	9	-0.250399	-0.728843	0.814480	0.913422	1.000
308	GOBP_SMALL_MOLECULE_METABOLIC_PROCESS	GO:0044281	15	-0.224832	-0.731900	0.827586	0.911452	1.000
309	GOBP_SPROUTING_ANGIOGENESIS	GO:0002040	5	-0.317531	-0.734660	0.780911	0.909667	1.000
310	GOBP_FOREBRAIN_DEVELOPMENT	GO:0030900	9	-0.263111	-0.736853	0.808756	0.908416	1.000
311	GOBP_ADENYLATE_CYCLASE_INHIBITING_G_PROTEIN_CO...	GO:0007196	5	-0.325301	-0.737170	0.773365	0.910209	1.000
312	GOBP_FAT_CELL_DIFFERENTIATION	GO:0045444	9	-0.265600	-0.739438	0.801782	0.908874	1.000
313	GOBP_NEGATIVE_REGULATION_OF_MULTICELLULAR_ORGA...	GO:0051241	15	-0.222144	-0.740049	0.840095	0.910257	1.000
314	GOBP_LONG_TERM_SYNAPTIC_POTENTIATION	GO:0060291	12	-0.239148	-0.741261	0.821101	0.910670	1.000
315	GOBP_POSITIVE_REGULATION_OF_NEURON_DEATH	GO:1901216	5	-0.320817	-0.742290	0.791111	0.911553	1.000
316	GOBP_TRANSMEMBRANE_RECEPTOR_PROTEIN_SERINE_THR...	GO:0007178	6	-0.304878	-0.745276	0.765700	0.909174	1.000
317	GOBP_CELLULAR_RESPONSE_TO_ABIOTIC_STIMULUS	GO:0071214	7	-0.295278	-0.745740	0.772727	0.910701	1.000
318	REACTOME_PTEN_REGULATION	R-RNO-6807070	5	-0.323984	-0.746391	0.776190	0.911881	1.000
319	REACTOME_ESTROGEN_DEPENDENT_NUCLEAR_EVENTS_DOW...	R-RNO-9634638	5	-0.331565	-0.747471	0.774194	0.912348	1.000
320	HALLMARK_HYPOXIA	M5891	5	-0.325574	-0.749039	0.778539	0.912065	1.000
321	GOBP_NEGATIVE_REGULATION_OF_PROTEIN_CATABOLIC_...	GO:0042177	5	-0.326477	-0.749072	0.765086	0.914293	1.000
322	GOBP_POSITIVE_REGULATION_OF_SYNAPTIC_TRANSMISSION	GO:0050806	15	-0.220952	-0.749122	0.810127	0.916499	1.000
323	GOBP_EPITHELIAL_CELL_DIFFERENTIATION	GO:0030855	7	-0.288901	-0.755181	0.783550	0.909189	1.000
324	GOBP_ENDOTHELIAL_CELL_MIGRATION	GO:0043542	8	-0.283127	-0.757845	0.772627	0.907374	1.000
325	GOBP_CYTOKINE_PRODUCTION	GO:0001816	10	-0.269231	-0.758496	0.777778	0.908540	1.000
326	GOBP_RESPONSE_TO_ENDOGENOUS_STIMULUS	GO:0009719	34	-0.190516	-0.759401	0.806701	0.909379	1.000
327	GOBP_LIPID_METABOLIC_PROCESS	GO:0006629	10	-0.269231	-0.762689	0.749431	0.906802	1.000
328	GOBP_RAS_PROTEIN_SIGNAL_TRANSDUCTION	GO:0007265	5	-0.332501	-0.762930	0.745182	0.908649	1.000
329	GOBP_REPRODUCTIVE_SYSTEM_DEVELOPMENT	GO:0061458	6	-0.315536	-0.763075	0.749436	0.910766	1.000
330	GOBP_CELLULAR_RESPONSE_TO_EXTERNAL_STIMULUS	GO:0071496	10	-0.267887	-0.766684	0.763855	0.907215	1.000
331	GOBP_BLOOD_VESSEL_ENDOTHELIAL_CELL_MIGRATION	GO:0043534	8	-0.283127	-0.769042	0.774038	0.905680	1.000
332	GOBP_CELLULAR_RESPONSE_TO_INORGANIC_SUBSTANCE	GO:0071241	11	-0.259288	-0.773824	0.755294	0.900322	1.000
333	REACTOME_INTEGRATION_OF_ENERGY_METABOLISM	R-RNO-163685	5	-0.349398	-0.775081	0.707944	0.900636	1.000
334	GOBP_CELLULAR_GLUCOSE_HOMEOSTASIS	GO:0001678	5	-0.348620	-0.780082	0.736170	0.894328	1.000
335	GOBP_POSITIVE_REGULATION_OF_AXONOGENESIS	GO:0050772	5	-0.351033	-0.781171	0.704289	0.894677	1.000
336	REACTOME_TRANSCRIPTIONAL_REGULATION_BY_TP53	R-RNO-3700989	6	-0.324536	-0.782046	0.723595	0.895581	1.000
337	GOBP_REGULATION_OF_PROTEIN_CATABOLIC_PROCESS	GO:0042176	7	-0.307750	-0.784342	0.716312	0.894014	1.000
338	GOBP_NEGATIVE_REGULATION_OF_PHOSPHORUS_METABOL...	GO:0010563	9	-0.283184	-0.787856	0.744240	0.890415	1.000
339	GOBP_CELL_CYCLE	GO:0007049	21	-0.228462	-0.791204	0.729798	0.887067	1.000
340	GOBP_MUSCLE_CELL_DIFFERENTIATION	GO:0042692	10	-0.275539	-0.795760	0.725962	0.881622	1.000
341	GOBP_NEGATIVE_REGULATION_OF_CELL_PROJECTION_OR...	GO:0031345	5	-0.348812	-0.797818	0.718681	0.880271	1.000

Continuation of Table **S11**

	NAME	ID	SIZE	ES	NES	NOM.p.val	FDR.q.val	FWER.p.val
342	GOBP_REGIONALIZATION	GO:0003002	6	-0.334450	-0.801745	0.651316	0.876002	1.000
343	GOBP_TUBE_MORPHOGENESIS	GO:0035239	15	-0.242575	-0.804184	0.717540	0.874035	1.000
344	REACTOME_SENESCENCE_ASSOCIATED_SECRETORY_PHENO...	R-RNO-2559582	6	-0.335032	-0.804710	0.706897	0.875521	1.000
345	GOBP_NEGATIVE_REGULATION_OF_CELLULAR_COMPONENT...	GO:0051129	11	-0.268916	-0.805780	0.712941	0.876019	1.000
346	GOBP_RESPONSE_TO_HEAT	GO:0009408	7	-0.319900	-0.807277	0.720280	0.875623	1.000
347	GOBP_RESPONSE_TO_TUMOR_NECROSIS_FACTOR	GO:0034612	7	-0.311517	-0.807476	0.659722	0.877682	1.000
348	GOBP_PATTERN_SPECIFICATION_PROCESS	GO:0007389	6	-0.334450	-0.808249	0.699152	0.878653	1.000
349	GOBP_REGULATION_OF_SMALL_MOLECULE_METABOLIC_PR...	GO:0062012	9	-0.286530	-0.811637	0.725664	0.874773	1.000
350	GOBP_AMEBOIDAL_TYPE_CELL_MIGRATION	GO:0001667	11	-0.264891	-0.812548	0.704225	0.875468	1.000
351	GOBP_NEGATIVE_REGULATION_OF_CATABOLIC_PROCESS	GO:0009895	8	-0.304186	-0.815049	0.699580	0.873390	1.000
352	HALLMARK_TNFA_SIGNALING_VIA_NFKB	M5890	14	-0.256757	-0.818429	0.705736	0.869578	1.000
353	GOBP_LEUKOCYTE_MEDIATED_IMMUNITY	GO:0002443	7	-0.326653	-0.819113	0.690171	0.870697	1.000
354	GOBP_POSITIVE_REGULATION_OF_CATALYTIC_ACTIVITY	GO:0043085	30	-0.206873	-0.819280	0.734211	0.872781	1.000
355	GOBP_NEGATIVE_REGULATION_OF_CATALYTIC_ACTIVITY	GO:0043086	17	-0.233521	-0.821300	0.696833	0.871339	1.000
356	GOBP_CELL_CYCLE_PROCESS	GO:0022402	11	-0.271568	-0.826031	0.694915	0.864647	1.000
357	REACTOME_CELLULAR_SENESCENCE	R-RNO-2559583	6	-0.335032	-0.828571	0.663462	0.862387	1.000
358	REACTOME_INTRACELLULAR_SIGNALING_BY_SECOND_MES...	R-RNO-9006925	15	-0.252302	-0.830639	0.703529	0.860774	1.000
359	GOBP_POSITIVE_REGULATION_OF_NEURON_PROJECTION_...	GO:0010976	5	-0.372420	-0.832378	0.668919	0.860023	1.000
360	GOBP_REGULATION_OF_CELLULAR_RESPONSE_TO_STRESS	GO:0080135	9	-0.304897	-0.832561	0.660173	0.862113	1.000
361	GOBP_NEURON_MIGRATION	GO:0001764	5	-0.367985	-0.832772	0.647186	0.864229	1.000
362	GOBP_NEGATIVE_REGULATION_OF_HYDROLASE_ACTIVITY	GO:0051346	11	-0.285178	-0.837472	0.667513	0.858269	1.000
363	GOBP_SIGNAL_RELEASE	GO:0023061	14	-0.258677	-0.837683	0.702765	0.860287	1.000
364	GOBP_TUBE_DEVELOPMENT	GO:0035295	18	-0.241712	-0.839918	0.694789	0.858677	1.000
365	GOBP_CARBOHYDRATE_HOMEOSTASIS	GO:0033500	7	-0.324111	-0.843211	0.638767	0.854445	1.000
366	GOBP_NEGATIVE_REGULATION_OF_BIOSYNTHETIC_PROCESS	GO:0009890	13	-0.269100	-0.843599	0.642298	0.856217	1.000
367	GOBP_MATERNAL_PROCESS_INVOLVED_IN_FEMALE_PREGN...	GO:0060135	5	-0.378671	-0.849495	0.612245	0.846914	1.000
368	GOBP_T_CELL_ACTIVATION	GO:0042110	8	-0.313698	-0.851963	0.619450	0.844440	1.000
369	GOBP_REGULATION_OF_IMMUNE_RESPONSE	GO:0050776	12	-0.269079	-0.856588	0.646789	0.838087	1.000
370	GOBP_RESPONSE_TO_METAL_ION	GO:0010038	15	-0.259735	-0.856653	0.639798	0.840387	1.000
371	GOBP_RESPONSE_TO_TEMPERATURE_STIMULUS	GO:0009266	9	-0.306770	-0.857408	0.622768	0.841448	1.000
372	GOBP_POSITIVE_REGULATION_OF_NERVOUS_SYSTEM_DEV...	GO:0051962	12	-0.282567	-0.859499	0.630137	0.840010	1.000
373	GOBP_SECOND_MESSENGER_MEDIATED_SIGNALING	GO:0019932	16	-0.258510	-0.859891	0.641509	0.841717	1.000
374	GOBP_CELL_PART_MORPHOGENESIS	GO:0032990	24	-0.233945	-0.860018	0.668317	0.843864	1.000
375	GOBP_POSITIVE_REGULATION_OF_MOLECULAR_FUNCTION	GO:0044093	38	-0.208895	-0.861152	0.708543	0.844159	1.000
376	GOBP_SMALL_GTPASE_MEDIATED_SIGNAL_TRANSDUCTION	GO:0007264	8	-0.320989	-0.863386	0.598639	0.842199	1.000
377	GOBP_PROTEIN_DEPHOSPHORYLATION	GO:0006470	7	-0.341408	-0.864037	0.613839	0.843434	1.000
378	GOBP_POSITIVE_REGULATION_OF_CELL_DEVELOPMENT	GO:0010720	12	-0.282567	-0.864345	0.634091	0.845369	1.000
379	GOBP_MUSCLE_STRUCTURE_DEVELOPMENT	GO:0061061	16	-0.263372	-0.864734	0.631336	0.847185	1.000
380	GOBP_MYELOID_LEUKOCYTE_ACTIVATION	GO:0002274	8	-0.323697	-0.871687	0.583732	0.836091	1.000
381	GOBP_STRIATED_MUSCLE_CELL_DIFFERENTIATION	GO:0051146	9	-0.310895	-0.871731	0.615044	0.838494	1.000
382	GOBP_DEPHOSPHORYLATION	GO:0016311	7	-0.341408	-0.872111	0.606593	0.840197	1.000
383	GOBP_CELL_MORPHOGENESIS	GO:0000902	24	-0.233945	-0.878377	0.633417	0.830476	1.000
384	GOBP_CELLULAR_COMPONENT_MORPHOGENESIS	GO:0032989	24	-0.233945	-0.879280	0.618421	0.831209	1.000

Continuation of Table **S11**

	NAME	ID	SIZE	ES	NES	NOM.p.val	FDR.q.val	FWER.p.val
385	GOBP_REGULATION_OF_TRANSFERASE_ACTIVITY	GO:0051338	22	-0.242689	-0.879610	0.629108	0.833170	1.000
386	GOBP_POSITIVE_REGULATION_OF_NEUROGENESIS	GO:0050769	12	-0.282567	-0.880404	0.600000	0.834143	1.000
387	GOBP_RESPONSE_TO_REACTIVE_OXYGEN_SPECIES	GO:0000302	8	-0.328910	-0.880405	0.598684	0.836655	1.000
388	GOBP_AGING	GO:0007568	13	-0.274918	-0.881289	0.632850	0.837252	1.000
389	GOBP_REGULATION_OF_PROTEIN_KINASE_ACTIVITY	GO:0045859	22	-0.242689	-0.884060	0.629268	0.834164	1.000
390	REACTOME_SIGNALING_BY_INTERLEUKINS	R-RNO-449147	15	-0.275931	-0.886121	0.588372	0.832399	1.000
391	GOBP_IN_UTERO_EMBRYONIC_DEVELOPMENT	GO:0001701	8	-0.326012	-0.887219	0.578947	0.832700	1.000
392	GOBP_RESPONSE_TO_OXIDATIVE_STRESS	GO:0006979	8	-0.328910	-0.888782	0.612832	0.832228	1.000
393	GOBP_GROWTH	GO:0040007	14	-0.274933	-0.890512	0.589681	0.831507	1.000
394	GOBP_POSITIVE_REGULATION_OF_BINDING	GO:0051099	6	-0.375421	-0.902941	0.576200	0.809259	1.000
395	GOBP_NEGATIVE_REGULATION_OF_GENE_EXPRESSION	GO:0010629	12	-0.294195	-0.910059	0.580866	0.797277	1.000
396	GOBP_CELLULAR_RESPONSE_TO_LIPID	GO:0071396	15	-0.267680	-0.910147	0.544776	0.799546	1.000
397	GOBP_NEGATIVE_REGULATION_OF_CELL_POPULATION_PR...	GO:0008285	9	-0.327903	-0.910835	0.571101	0.800834	1.000
398	GOBP_DENDRITE_MORPHOGENESIS	GO:0048813	6	-0.376298	-0.911757	0.577273	0.801541	1.000
399	GOBP_REGULATION_OF_DEVELOPMENTAL_GROWTH	GO:0048638	9	-0.323231	-0.915726	0.561905	0.796034	1.000
400	GOBP_NEGATIVE_REGULATION_OF_ANION_TRANSPORT	GO:1903792	8	-0.337424	-0.918697	0.518779	0.792587	1.000
401	GOBP_RESPONSE_TO_KETONE	GO:1901654	7	-0.358025	-0.924728	0.509091	0.783122	1.000
402	GOBP_REGULATION_OF_CELL_CYCLE_PROCESS	GO:0010564	5	-0.409639	-0.925832	0.508584	0.783162	1.000
403	GOBP_CELLULAR_RESPONSE_TO_EXTRACELLULAR_STIMULUS	GO:0031668	8	-0.346803	-0.929313	0.538462	0.778878	1.000
404	GOBP_POSITIVE_REGULATION_OF_PRI_MIRNA_TRANSCRI...	GO:1902895	7	-0.357373	-0.929771	0.512987	0.780376	1.000
405	GOBP_GLIOGENESIS	GO:0042063	12	-0.308196	-0.932110	0.507576	0.777902	1.000
406	GOBP_REGULATION_OF_VASCULATURE_DEVELOPMENT	GO:1901342	5	-0.409639	-0.932877	0.527964	0.778805	1.000
407	GOBP_ORGANONITROGEN_COMPOUND_CATABOLIC_PROCESS	GO:1901565	9	-0.335179	-0.933142	0.529817	0.780749	1.000
408	GOBP_REGULATION_OF_CELL_DEATH	GO:0010941	34	-0.230783	-0.933885	0.593516	0.781664	1.000
409	GOBP_RESPONSE_TO_OXYGEN_CONTAINING_COMPOUND	GO:1901700	39	-0.223029	-0.938172	0.538265	0.775290	1.000
410	GOBP_CELL_JUNCTION_ASSEMBLY	GO:0034329	8	-0.347106	-0.940314	0.529284	0.773229	1.000
411	GOBP_PRI_MIRNA_TRANSCRIPTION_BY_RNA_POLYMERASE_II	GO:0061614	7	-0.357373	-0.940976	0.535354	0.774328	1.000
412	GOBP_POSITIVE_REGULATION_OF_SIGNALING	GO:0023056	34	-0.235395	-0.943714	0.540540	0.770991	1.000
413	GOBP_RESPONSE_TO_NICOTINE	GO:0035094	7	-0.372282	-0.945054	0.500000	0.770783	1.000
414	GOBP_REGULATION_OF_ANATOMICAL_STRUCTURE_MORPHO...	GO:0022603	17	-0.270747	-0.945691	0.513761	0.772012	1.000
415	GOBP_PEPTIDYL_LYSINE_MODIFICATION	GO:0018205	5	-0.421687	-0.947734	0.463636	0.770260	1.000
416	GOBP_MULTI_ORGANISM_PROCESS	GO:0051704	16	-0.268408	-0.947898	0.516908	0.772471	1.000
417	GOBP_NEGATIVE_REGULATION_OF_LOCOMOTION	GO:0040013	5	-0.411548	-0.950942	0.486486	0.769095	1.000
418	GOBP_RESPONSE_TO_CALCIUM_ION	GO:0051592	6	-0.397419	-0.952814	0.512472	0.767958	1.000
419	GOBP_RESPONSE_TO_INSULIN	GO:0032868	5	-0.421687	-0.954913	0.460385	0.766197	1.000
420	GOBP_RHYTHMIC_PROCESS	GO:0048511	15	-0.289539	-0.960088	0.502193	0.758422	1.000
421	GOBP_CARDIAC_MUSCLE_TISSUE_DEVELOPMENT	GO:0048738	6	-0.399065	-0.961195	0.473804	0.758543	1.000
422	GOBP_RESPONSE_TO_UV	GO:0009411	5	-0.421687	-0.962103	0.460215	0.759130	1.000
423	GOBP_HEART_DEVELOPMENT	GO:0007507	6	-0.399065	-0.966126	0.513825	0.753252	1.000
424	GOBP_INTRACELLULAR_RECEPTOR_SIGNALING_PATHWAY	GO:0030522	5	-0.421687	-0.974270	0.446390	0.738748	1.000
425	GOBP_REGULATION_OF_CATABOLIC_PROCESS	GO:0009894	13	-0.304496	-0.974811	0.479167	0.740086	1.000
426	HALLMARK_APOPTOSIS	M5902	6	-0.402439	-0.976512	0.442890	0.738801	1.000
427	GOBP_ORGANOPHOSPHATE_METABOLIC_PROCESS	GO:0019637	7	-0.383814	-0.976890	0.444196	0.740538	1.000

Continuation of Table **S11**

	NAME	ID	SIZE	ES	NES	NOM.p.val	FDR.q.val	FWER.p.val
428	GOBP_EPIDERMIS_DEVELOPMENT	GO:0008544	5	-0.429430	-0.981328	0.453901	0.733757	1.000
429	GOBP_RESPONSE_TO_NITROGEN_COMPOUND	GO:1901698	34	-0.242459	-0.984378	0.489189	0.729904	1.000
430	GOBP_FC_RECEPTOR_SIGNALING_PATHWAY	GO:0038093	8	-0.367628	-0.985707	0.466488	0.729748	1.000
431	GOBP_GLIAL_CELL_DIFFERENTIATION	GO:0010001	10	-0.328888	-0.986562	0.439636	0.730557	1.000
432	REACTOME_SIGNALING_BY_RHO_GTPASES_MIRO_GTPASES...	R-RNO-9716542	9	-0.358318	-0.987619	0.441704	0.730941	1.000
433	GOBP_NEGATIVE_REGULATION_OF_SIGNALING	GO:0023057	21	-0.271482	-0.991009	0.476886	0.726425	1.000
434	REACTOME_G_ALPHA_I_SIGNALLING_EVENTS	R-RNO-418594	18	-0.291099	-0.991084	0.469586	0.728795	1.000
435	GOBP_POSITIVE_REGULATION_OF_DEVELOPMENTAL_PROCESS	GO:0051094	32	-0.249293	-0.994182	0.467934	0.725033	1.000
436	GOBP_CIRCULATORY_SYSTEM_PROCESS	GO:0003013	5	-0.439660	-0.995168	0.439583	0.725659	1.000
437	GOBP_MITOCHONDRION_ORGANIZATION	GO:0007005	7	-0.392524	-0.998847	0.439462	0.720145	1.000
438	GOBP_POSITIVE_REGULATION_OF_GROWTH	GO:0045927	9	-0.362039	-1.000936	0.457275	0.718351	1.000
439	GOBP_IMMUNE_RESPONSE_REGULATING_SIGNALING_PATHWAY	GO:0002764	8	-0.367628	-1.002030	0.431718	0.718566	1.000
440	GOBP_POSITIVE_REGULATION_OF_ION_TRANSPORT	GO:0043270	17	-0.294705	-1.004698	0.452607	0.715351	1.000
441	GOBP_POSITIVE_REGULATION_OF_TRANSCRIPTION_BY_R...	GO:0045944	24	-0.267564	-1.005569	0.448276	0.715883	1.000
442	GOBP_RESPONSE_TO_LIPID	GO:0033993	19	-0.291581	-1.005700	0.431981	0.718279	1.000
443	GOBP_REGULATION_OF_ENDOCYTOSIS	GO:0030100	6	-0.393889	-1.007133	0.396963	0.717957	1.000
444	GOBP_NEGATIVE_REGULATION_OF_PEPTIDASE_ACTIVITY	GO:0010466	6	-0.403001	-1.008248	0.408791	0.718233	1.000
445	GOBP_REGULATION_OF_SYNAPSE_STRUCTURE_OR_ACTIVITY	GO:0050803	11	-0.344835	-1.008382	0.434053	0.720594	1.000
446	GOBP_NEGATIVE_REGULATION_OF_NEURON_DEATH	GO:1901215	11	-0.335921	-1.012184	0.419431	0.715298	1.000
447	GOBP_BLOOD_VESSEL_MORPHOGENESIS	GO:0048514	14	-0.314399	-1.012474	0.422604	0.717344	1.000
448	GOBP_HORMONE_TRANSPORT	GO:0009914	9	-0.367314	-1.013092	0.454746	0.718774	1.000
449	GOBP_REGULATION_OF_PHOSPHOLIPASE_ACTIVITY	GO:0010517	6	-0.421805	-1.013275	0.433260	0.721144	1.000
450	GOBP_POSITIVE_REGULATION_OF_TRANSPORT	GO:0051050	26	-0.260782	-1.013471	0.418421	0.723443	1.000
451	GOBP_EMBRYONIC_ORGAN_DEVELOPMENT	GO:0048568	7	-0.402272	-1.014816	0.407767	0.723365	1.000
452	GOBP_REGULATION_OF_NERVOUS_SYSTEM_DEVELOPMENT	GO:0051960	15	-0.308777	-1.020681	0.399072	0.713300	1.000
453	REACTOME_RHO_GTPASE_EFFECTORS	R-RNO-195258	9	-0.358318	-1.022186	0.400911	0.712687	1.000
454	GOBP_SKELETAL_MUSCLE_ORGAN_DEVELOPMENT	GO:0060538	5	-0.453676	-1.022777	0.414847	0.714123	1.000
455	GOBP_POSITIVE_REGULATION_OF_CATION_TRANSMEMBRA...	GO:1904064	5	-0.457831	-1.023281	0.405702	0.715794	1.000
456	GOBP_MULTI_MULTICELLULAR_ORGANISM_PROCESS	GO:0044706	8	-0.380294	-1.031012	0.392202	0.702392	1.000
457	REACTOME_ESR_MEDIATED_SIGNALING	R-RNO-8939211	8	-0.381377	-1.031156	0.417411	0.704776	1.000
458	REACTOME_SIGNALING_BY_NUCLEAR_RECEPTORS	R-RNO-9006931	8	-0.381377	-1.034182	0.402685	0.701087	1.000
459	GOBP_POSITIVE_REGULATION_OF_ENDOTHELIAL_CELL_P...	GO:0001938	6	-0.426829	-1.035582	0.397727	0.701009	1.000
460	GOBP_REGULATION_OF_FAT_CELL_DIFFERENTIATION	GO:0045598	6	-0.426829	-1.043922	0.413721	0.686487	1.000
461	GOBP_CELLULAR_MACROMOLECULE_CATABOLIC_PROCESS	GO:0044265	6	-0.434427	-1.044203	0.395034	0.688561	1.000
462	GOBP_DENDRITE_DEVELOPMENT	GO:0016358	9	-0.370671	-1.044955	0.377622	0.689636	1.000
463	GOBP_REGULATION_OF_LIPASE_ACTIVITY	GO:0060191	6	-0.421805	-1.045516	0.410314	0.691084	1.000
464	REACTOME_NUCLEAR_EVENTS_KINASE_AND_TRANSCRIPTI...	R-RNO-198725	11	-0.346103	-1.048037	0.387097	0.688389	1.000
465	GOBP_RESPONSE_TO_ACID_CHEMICAL	GO:0001101	6	-0.429247	-1.054035	0.362385	0.678548	1.000
466	GOBP_TISSUE_MIGRATION	GO:0090130	9	-0.375095	-1.056320	0.369412	0.676626	1.000
467	REACTOME_EXTRA_NUCLEAR_ESTROGEN_SIGNALING	R-RNO-9009391	7	-0.409420	-1.056554	0.375000	0.678885	1.000
468	GOBP_INFLAMMATORY_RESPONSE	GO:0006954	12	-0.340398	-1.057100	0.366162	0.680461	1.000
469	GOBP_NERVE_DEVELOPMENT	GO:0021675	9	-0.375723	-1.057863	0.380952	0.681604	1.000
470	GOBP_CELLULAR_COMPONENT_DISASSEMBLY	GO:0022411	6	-0.437583	-1.060260	0.353201	0.679334	1.000

Continuation of Table **S11**

	NAME	ID	SIZE	ES	NES	NOM.p.val	FDR.q.val	FWER.p.val
471	GOBP_REGULATION_OF_NEUROGENESIS	GO:0050767	14	-0.325453	-1.060387	0.376417	0.681794	1.000
472	GOBP_REGULATION_OF_CELLULAR_AMIDE_METABOLIC_PR...	GO:0034248	9	-0.390062	-1.060565	0.373550	0.684027	1.000
473	REACTOME_SIGNALING_BY_RECEPTOR_TYROSINE_KINASES	R-RNO-9006934	26	-0.280017	-1.062107	0.354460	0.683678	1.000
474	GOBP_ENDOMEMBRANE_SYSTEM_ORGANIZATION	GO:0010256	6	-0.430845	-1.069021	0.368664	0.671895	1.000
475	GOBP_GLAND_DEVELOPMENT	GO:0048732	7	-0.402462	-1.069662	0.362637	0.673228	1.000
476	GOBP_NUCLEOBASE_CONTAINING_SMALL_MOLECULE_META...	GO:0055086	6	-0.428977	-1.075120	0.352442	0.664571	1.000
477	REACTOME_INTERLEUKIN_4_AND_INTERLEUKIN_13_SIGN...	R-RNO-6785807	8	-0.404856	-1.077324	0.346491	0.663043	1.000
478	GOBP_NEGATIVE_REGULATION_OF_CYSTEINE_TYPE_ENDO...	GO:2000117	5	-0.468326	-1.078380	0.338144	0.663618	1.000
479	GOBP_RESPONSE_TO_NERVE_GROWTH_FACTOR	GO:1990089	7	-0.423809	-1.081504	0.356659	0.660073	1.000
480	GOBP_CIRCADIAN_RHYTHM	GO:0007623	13	-0.336001	-1.083242	0.346983	0.659120	1.000
481	GOBP_CELLULAR_LIPID_METABOLIC_PROCESS	GO:0044255	7	-0.432099	-1.085232	0.337995	0.657629	1.000
482	GOBP_REGULATION_OF_MUSCLE_ADAPTATION	GO:0043502	5	-0.485028	-1.085627	0.330377	0.659557	1.000
483	GOBP_POSITIVE_REGULATION_OF_CELL_DIFFERENTIATION	GO:0045597	27	-0.286857	-1.088229	0.328395	0.657100	1.000
484	GOBP_MUSCLE_ADAPTATION	GO:0043500	5	-0.485028	-1.090867	0.342553	0.654380	1.000
485	REACTOME_ADAPTIVE_IMMUNE_SYSTEM	R-RNO-1280218	9	-0.396149	-1.090980	0.334831	0.656990	1.000
486	GOBP_REGULATION_OF_ENDOTHELIAL_CELL_MIGRATION	GO:0010594	5	-0.469218	-1.092217	0.339956	0.657177	1.000
487	GOBP_MUSCLE_TISSUE_DEVELOPMENT	GO:0060537	11	-0.364630	-1.093953	0.310185	0.656247	1.000
488	GOBP_REGULATION_OF_SYSTEM_PROCESS	GO:0044057	18	-0.312299	-1.095575	0.315534	0.655949	1.000
489	GOBP_LEUKOCYTE_DIFFERENTIATION	GO:0002521	15	-0.333221	-1.096322	0.327059	0.657234	1.000
490	GOBP_CELL_CELL_ADHESION	GO:0098609	14	-0.336362	-1.097380	0.328090	0.657673	1.000
491	REACTOME_G_ALPHA_Q_SIGNALLING_EVENTS	R-RNO-416476	6	-0.441481	-1.097480	0.295872	0.660292	1.000
492	GOBP_ACTIN_FILAMENT_BASED_PROCESS	GO:0030029	7	-0.434950	-1.099979	0.324885	0.657698	1.000
493	GOBP_RESPONSE_TO_EXTRACELLULAR_STIMULUS	GO:0009991	13	-0.354342	-1.102728	0.314088	0.654722	1.000
494	GOBP_PROTEIN_MODIFICATION_BY_SMALL_PROTEIN_CON...	GO:0032446	6	-0.451260	-1.103399	0.322072	0.656258	1.000
495	GOBP_CHROMOSOME_ORGANIZATION	GO:0051276	6	-0.456582	-1.103499	0.332558	0.658982	1.000
496	GOBP_CELLULAR_RESPONSE_TO_NITROGEN_COMPOUND	GO:1901699	20	-0.304568	-1.105511	0.314496	0.657665	1.000
497	GOBP_CELLULAR_RESPONSE_TO_MOLECULE_OF_BACTERIA...	GO:0071219	8	-0.414255	-1.107187	0.338983	0.656965	1.000
498	GOBP_CELLULAR_RESPONSE_TO_BIOTIC_STIMULUS	GO:0071216	8	-0.414255	-1.108849	0.341743	0.656435	1.000
499	GOBP_VASCULATURE_DEVELOPMENT	GO:0001944	17	-0.329129	-1.109046	0.307107	0.658942	1.000
500	GOBP_NEGATIVE_REGULATION_OF_RESPONSE_TO_STIMULUS	GO:0048585	16	-0.329406	-1.110165	0.311778	0.659424	1.000
501	GOBP_POSTTRANSCRIPTIONAL_REGULATION_OF_GENE_EX...	GO:0010608	8	-0.413927	-1.110962	0.338747	0.660639	1.000
502	GOBP_POSITIVE_REGULATION_OF_EPITHELIAL_CELL_PR...	GO:0050679	7	-0.439593	-1.111480	0.312217	0.662666	1.000
503	GOBP_REGULATION_OF_PROTEIN_PHOSPHORYLATION	GO:0001932	26	-0.293559	-1.111877	0.312039	0.664775	1.000
504	REACTOME_G_PROTEIN_MEDIATED_EVENTS	R-RNO-112040	9	-0.405331	-1.113472	0.312896	0.664544	1.000
505	GOBP_RESPONSE_TO_BACTERIUM	GO:0009617	13	-0.358277	-1.114412	0.299766	0.665713	1.000
506	GOBP_ENZYME_LINKED_RECEPTOR_PROTEIN_SIGNALING_...	GO:0007167	24	-0.298400	-1.114720	0.280952	0.668198	1.000
507	GOBP_EXOCYTOSIS	GO:0006887	11	-0.373088	-1.118984	0.303738	0.662121	1.000
508	GOBP_REGULATION_OF_CELLULAR_LOCALIZATION	GO:0060341	17	-0.329739	-1.120243	0.276543	0.662469	1.000
509	GOBP_REGULATION_OF_HYDROLASE_ACTIVITY	GO:0051336	22	-0.304948	-1.120986	0.305000	0.664010	1.000
510	GOBP_REGULATION_OF_SECRETION	GO:0051046	14	-0.340897	-1.123877	0.288636	0.660600	1.000
511	GOBP_RESPONSE_TO_MOLECULE_OF_BACTERIAL_ORIGIN	GO:0002237	13	-0.358277	-1.126364	0.304850	0.658412	1.000
512	GOBP_PROTEIN_AUTOPHOSPHORYLATION	GO:0046777	5	-0.497262	-1.126946	0.299776	0.660240	1.000
513	GOBP_POSITIVE_REGULATION_OF_DEVELOPMENTAL_GROWTH	GO:0048639	8	-0.416174	-1.128899	0.291566	0.658917	1.000

Continuation of Table **S11**

	NAME	ID	SIZE	ES	NES	NOM.p.val	FDR.q.val	FWER.p.val
514	GOBP_NEGATIVE_REGULATION_OF_NEURON_APOPTOTIC_P...	GO:0043524	8	-0.414987	-1.130412	0.309051	0.658857	1.000
515	GOBP_PHAGOCYTOSIS	GO:0006909	5	-0.509836	-1.143983	0.313559	0.632695	1.000
516	REACTOME_CA_DEPENDENT_EVENTS	R-RNO-111996	8	-0.422217	-1.144941	0.280702	0.633807	1.000
517	GOBP_RESPONSE_TO_CADMIUM_ION	GO:0046686	6	-0.475866	-1.148759	0.287016	0.628795	1.000
518	GOBP_REGULATION_OF_CELLULAR_PROTEIN_LOCALIZATION	GO:1903827	12	-0.379526	-1.150157	0.275463	0.629160	1.000
519	GOBP_POSITIVE_REGULATION_OF_TRANSFERASE_ACTIVITY	GO:0051347	20	-0.322847	-1.153459	0.251908	0.625623	1.000
520	GOBP_POSITIVE_REGULATION_OF_BIOSYNTHETIC_PROCESS	GO:0009891	27	-0.300146	-1.153824	0.248101	0.628072	1.000
521	GOBP_DNA_METABOLIC_PROCESS	GO:0006259	6	-0.479290	-1.154001	0.262115	0.630914	1.000
522	GOBP_POSITIVE_REGULATION_OF_GENE_EXPRESSION	GO:0010628	16	-0.338283	-1.154576	0.258929	0.632854	1.000
523	GOBP_MUSCLE_ORGAN_DEVELOPMENT	GO:0007517	9	-0.405063	-1.157730	0.270455	0.629164	1.000
524	REACTOME_SIGNALING_BY_NTRKS	R-RNO-166520	18	-0.338492	-1.157962	0.263279	0.631988	1.000
525	GOBP_NEGATIVE_REGULATION_OF_PROTEOLYSIS	GO:0045861	8	-0.429925	-1.161640	0.260465	0.627181	1.000
526	GOBP_DEVELOPMENTAL_GROWTH	GO:0048589	10	-0.407473	-1.166236	0.265823	0.620987	1.000
527	REACTOME_SIGNALING_BY_NTRK2_TRKB	R-RNO-9006115	6	-0.470358	-1.166944	0.252252	0.622740	1.000
528	GOBP_INTRINSIC_APOPTOTIC_SIGNALING_PATHWAY	GO:0097193	5	-0.511537	-1.168036	0.265589	0.623889	1.000
529	GOBP_MYELOID_CELL_DIFFERENTIATION	GO:0030099	13	-0.374590	-1.169336	0.241218	0.624357	1.000
530	GOBP_ORGAN_GROWTH	GO:0035265	5	-0.520901	-1.171962	0.257143	0.621830	1.000
531	GOBP_REGULATION_OF_CELLULAR_COMPONENT_BIOGENESIS	GO:0044087	10	-0.407796	-1.172532	0.245033	0.623716	1.000
532	GOBP_ENDOCYTOSIS	GO:0006897	8	-0.451996	-1.174935	0.270089	0.621673	1.000
533	HALLMARK_ALLOGRAFT_REJECTION	M5950	7	-0.451705	-1.175717	0.242206	0.623391	1.000
534	GOBP_PEPTIDE_SECRETION	GO:0002790	9	-0.419296	-1.176778	0.269136	0.624595	1.000
535	GOBP_REGULATION_OF_HEMOPOIESIS	GO:1903706	10	-0.401519	-1.178265	0.226164	0.625035	1.000
536	GOBP_REGULATION_OF_ORGAN_GROWTH	GO:0046620	5	-0.520901	-1.178843	0.264192	0.627318	1.000
537	GOBP_ANIMAL_ORGAN_MORPHOGENESIS	GO:0009887	12	-0.384453	-1.179949	0.250608	0.628334	1.000
538	GOBP_PEPTIDE_HORMONE_SECRETION	GO:0030072	7	-0.462657	-1.180585	0.227373	0.630647	1.000
539	GOBP_NEGATIVE_REGULATION_OF_TRANSPORT	GO:0051051	13	-0.374087	-1.181478	0.245700	0.632317	1.000
540	GOBP_POSITIVE_REGULATION_OF_PROTEIN_KINASE_ACT...	GO:0045860	19	-0.329468	-1.182284	0.219388	0.634086	1.000
541	GOBP_NEURON_DEATH	GO:0070997	18	-0.332965	-1.182483	0.224344	0.637134	1.000
542	GOBP_POSITIVE_REGULATION_OF_PROTEIN_METABOLIC_...	GO:0051247	28	-0.305819	-1.185579	0.219277	0.633776	1.000
543	GOBP_POSITIVE_REGULATION_OF_EPITHELIAL_CELL_MI...	GO:0010634	5	-0.522545	-1.187202	0.257143	0.633882	1.000
544	GOBP_MITOTIC_CELL_CYCLE	GO:0000278	10	-0.408735	-1.196900	0.238095	0.616369	1.000
545	GOBP_REGULATION_OF_ANION_TRANSPORT	GO:0044070	20	-0.333087	-1.200152	0.199531	0.613302	1.000
546	GOBP_POSITIVE_REGULATION_OF_IMMUNE_SYSTEM_PROCESS	GO:0002684	18	-0.343170	-1.201359	0.231144	0.614200	1.000
547	REACTOME_MAPK_TARGETS_NUCLEAR_EVENTS_MEDIATED_...	R-HSA-450282	5	-0.531873	-1.202588	0.259794	0.615350	1.000
548	GOBP_REGULATION_OF_CELLULAR_CATABOLIC_PROCESS	GO:0031329	10	-0.417480	-1.206441	0.235033	0.610388	1.000
549	GOBP_PERIPHERAL_NERVOUS_SYSTEM_DEVELOPMENT	GO:0007422	9	-0.432383	-1.209099	0.224622	0.608281	1.000
550	GOBP_INSULIN_SECRETION	GO:0030073	7	-0.462657	-1.214204	0.220297	0.600374	1.000
551	GOBP_RESPONSE_TO_STARVATION	GO:0042594	5	-0.534429	-1.215167	0.233100	0.601724	1.000
552	GOBP_REGULATION_OF_MYELOID_CELL_DIFFERENTIATION	GO:0045637	9	-0.428584	-1.215325	0.185268	0.604938	1.000
553	GOBP_POSITIVE_REGULATION_OF_TRANSMEMBRANE_TRAN...	GO:0034764	7	-0.469136	-1.224432	0.209135	0.589792	1.000
554	GOBP_ESTABLISHMENT_OF_PROTEIN_LOCALIZATION	GO:0045184	18	-0.352385	-1.225421	0.185542	0.591255	1.000
555	GOBP_REGULATION_OF_CIRCADIAN_RHYTHM	GO:0042752	7	-0.485904	-1.229740	0.230769	0.585873	1.000
556	REACTOME_EXTRACELLULAR_MATRIX_ORGANIZATION	R-RNO-1474244	5	-0.528771	-1.232212	0.189655	0.583885	1.000

Continuation of Table **S11**

	NAME	ID	SIZE	ES	NES	NOM.p.val	FDR.q.val	FWER.p.val
557	GOBP_BIOLOGICAL_ADHESION	GO:0022610	19	-0.344879	-1.232526	0.179612	0.586803	1.000
558	GOBP_REGULATION_OF_APOPTOTIC_SIGNALING_PATHWAY	GO:2001233	10	-0.419575	-1.233713	0.209821	0.588060	1.000
559	GOBP_NEGATIVE_REGULATION_OF_RESPONSE_TO_EXTERN...	GO:0032102	5	-0.528865	-1.234965	0.216518	0.588902	1.000
560	GOBP_APOPTOTIC_MITOCHONDRIAL_CHANGES	GO:0008637	6	-0.523316	-1.237878	0.219457	0.586277	1.000
561	GOBP_INTRACELLULAR_TRANSPORT	GO:0046907	11	-0.407199	-1.238383	0.208738	0.588858	1.000
562	GOBP_CELLULAR_RESPONSE_TO_STARVATION	GO:0009267	5	-0.534429	-1.238448	0.211207	0.592492	1.000
563	GOBP_SENSORY_PERCEPTION_OF_PAIN	GO:0019233	5	-0.556438	-1.242430	0.225882	0.587266	1.000
564	REACTOME_OPIOID_SIGNALLING	R-RNO-111885	12	-0.405099	-1.246191	0.182898	0.583488	1.000
565	GOBP_NEGATIVE_REGULATION_OF_PROTEIN_METABOLIC_...	GO:0051248	20	-0.349207	-1.249851	0.180974	0.579234	1.000
566	GOBP_MULTICELLULAR_ORGANISMAL_RESPONSE_TO_STRESS	GO:0033555	5	-0.551579	-1.251002	0.212903	0.580755	1.000
567	GOBP_PROTEIN_LOCALIZATION_TO_ORGANELLE	GO:0033365	9	-0.459187	-1.257994	0.211905	0.569801	1.000
568	GOBP_CELLULAR_RESPONSE_TO_CHEMICAL_STRESS	GO:0062197	7	-0.493212	-1.259794	0.180365	0.569875	1.000
569	GOBP_EMBRYO_DEVELOPMENT_ENDING_IN_BIRTH_OR_EGG...	GO:0009792	9	-0.448569	-1.260423	0.206972	0.572279	1.000
570	GOBP_REGULATION_OF_CELL_DEVELOPMENT	GO:0060284	15	-0.382197	-1.264667	0.188073	0.566753	1.000
571	GOBP_CELLULAR_RESPONSE_TO_REACTIVE_OXYGEN_SPECIES	GO:0034614	7	-0.493212	-1.266365	0.179426	0.567096	1.000
572	GOBP_MUSCLE_SYSTEM_PROCESS	GO:0003012	9	-0.447964	-1.269166	0.170115	0.565017	1.000
573	GOBP_REGULATION_OF_PHOSPHORUS_METABOLIC_PROCESS	GO:0051174	27	-0.329132	-1.271459	0.154047	0.564183	1.000
574	GOBP_CELLULAR_RESPONSE_TO_OXYGEN_CONTAINING_CO...	GO:1901701	29	-0.322270	-1.272893	0.161376	0.565130	1.000
575	GOBP_CIRCULATORY_SYSTEM_DEVELOPMENT	GO:0072359	21	-0.346169	-1.279052	0.152542	0.556300	1.000
576	REACTOME_FC_EPSILON_RECEPTOR_FCERI_SIGNALING	R-RNO-2454202	7	-0.501189	-1.280601	0.167421	0.557180	1.000
577	GOBP_REGULATION_OF_VESICLE_MEDIATED_TRANSPORT	GO:0060627	12	-0.414013	-1.282753	0.154964	0.556506	1.000
578	GOBP_INTRACELLULAR_PROTEIN_TRANSPORT	GO:0006886	9	-0.452770	-1.284977	0.155660	0.555683	1.000
579	GOBP_REGULATION_OF_HORMONE_SECRETION	GO:0046883	8	-0.477997	-1.285158	0.190588	0.559274	1.000
580	GOBP_PROTEIN_PHOSPHORYLATION	GO:0006468	34	-0.319813	-1.285706	0.120643	0.562099	1.000
581	GOBP_NEUROTROPHIN_TRK_RECEPTOR_SIGNALING_PATHWAY	GO:0048011	6	-0.536785	-1.288560	0.179487	0.559957	1.000
582	GOBP_EMBRYONIC_MORPHOGENESIS	GO:0048598	6	-0.534977	-1.289572	0.171296	0.561904	1.000
583	GOBP_POSITIVE_REGULATION_OF_MULTICELLULAR_ORGA...	GO:0051240	32	-0.329443	-1.292199	0.132353	0.560299	1.000
584	GOBP_PEPTIDYL_TYROSINE_MODIFICATION	GO:0018212	10	-0.453646	-1.304735	0.136771	0.538046	1.000
585	GOBP_NEGATIVE_REGULATION_OF_PROTEIN_MODIFICATI...	GO:0031400	10	-0.451818	-1.306018	0.150685	0.539412	1.000
586	GOBP_SECRETION	GO:0046903	24	-0.350144	-1.308687	0.112782	0.537767	1.000
587	GOBP_POSITIVE_REGULATION_OF_NUCLEOBASE_CONTAIN...	GO:0045935	28	-0.340671	-1.314828	0.124352	0.529026	1.000
588	GOBP_NEGATIVE_REGULATION_OF_NERVOUS_SYSTEM_DEV...	GO:0051961	5	-0.578964	-1.315364	0.167800	0.531792	1.000
589	GOBP_ENDOTHELIAL_CELL_PROLIFERATION	GO:0001935	8	-0.478698	-1.316020	0.162291	0.534343	1.000
590	GOBP_NEUROTROPHIN_SIGNALING_PATHWAY	GO:0038179	6	-0.536785	-1.318328	0.177778	0.533873	1.000
591	GOBP_RESPONSE_TO_ALKALOID	GO:0043279	5	-0.588934	-1.318452	0.176734	0.537787	1.000
592	GOBP_REGULATION_OF_IMMUNE_SYSTEM_PROCESS	GO:0002682	21	-0.365909	-1.321638	0.125000	0.535601	1.000
593	GOBP_NEGATIVE_REGULATION_OF_TRANSMEMBRANE_TRAN...	GO:0034763	6	-0.542706	-1.321895	0.167442	0.539277	1.000
594	GOBP_FC_EPSILON_RECEPTOR_SIGNALING_PATHWAY	GO:0038095	7	-0.501189	-1.323492	0.166292	0.539976	1.000
595	GOBP_CYTOSKELETON_ORGANIZATION	GO:0007010	11	-0.439665	-1.324858	0.141026	0.541338	1.000
596	GOBP_REGULATION_OF_MUSCLE_SYSTEM_PROCESS	GO:0090257	8	-0.494203	-1.333279	0.138952	0.527858	1.000
597	GOBP_REGULATION_OF_LEUKOCYTE_DIFFERENTIATION	GO:1902105	9	-0.476719	-1.334868	0.128395	0.528801	1.000
598	GOBP_REGULATION_OF_PROTEIN_SERINE_THREONINE_KI...	GO:0071900	12	-0.434254	-1.340059	0.167920	0.521623	1.000
599	GOBP_APOPTOTIC_PROCESS	GO:0006915	35	-0.334089	-1.342518	0.105943	0.520980	1.000

Continuation of Table **S11**

	NAME	ID	SIZE	ES	NES	NOM.p.val	FDR.q.val	FWER.p.val
600	GOBP_REGULATION_OF_PEPTIDYL_TYROSINE_PHOSPHORY...	GO:0050730	9	-0.465386	-1.343222	0.150562	0.523966	1.000
601	GOBP_POSITIVE_REGULATION_OF_PEPTIDYL_TYROSINE_...	GO:0050731	8	-0.499029	-1.350519	0.127359	0.513184	1.000
602	GOBP_TRANSMEMBRANE_RECEPTOR_PROTEIN_TYROSINE_K...	GO:0007169	19	-0.375799	-1.353577	0.101990	0.511299	1.000
603	GOBP_EMBRYO_DEVELOPMENT	GO:0009790	14	-0.416274	-1.356255	0.103704	0.510021	1.000
604	GOBP_NEGATIVE_REGULATION_OF_NUCLEOBASE_CONTAIN...	GO:0045934	13	-0.427517	-1.357015	0.127359	0.512921	1.000
605	GOBP_POSITIVE_REGULATION_OF_PROTEIN_PHOSPHORYL...	GO:0001934	23	-0.370149	-1.362873	0.109865	0.505537	1.000
606	REACTOME_G_ALPHA_Z_SIGNALLING_EVENTS	R-RNO-418597	5	-0.601370	-1.364935	0.161702	0.505954	1.000
607	GOBP_CELLULAR_RESPONSE_TO_CADMIUM_ION	GO:0071276	5	-0.614021	-1.365182	0.140426	0.509935	1.000
608	GOBP_MAPK_CASCADE	GO:0000165	18	-0.404724	-1.384270	0.081081	0.475607	1.000
609	GOBP_LEUKOCYTE_MIGRATION	GO:0050900	6	-0.572415	-1.386692	0.127962	0.475008	1.000
610	GOBP_REGULATION_OF_OSTEOCLAST_DIFFERENTIATION	GO:0045670	5	-0.608122	-1.388514	0.128261	0.475292	1.000
611	GOBP_POSITIVE_REGULATION_OF_PROTEIN_MODIFICATI...	GO:0031401	23	-0.370149	-1.397436	0.086634	0.460417	1.000
612	GOBP_REGULATION_OF_MYELOID_LEUKOCYTE_DIFFERENT...	GO:0002761	8	-0.510990	-1.401103	0.126728	0.457852	1.000
613	GOBP_REGULATION_OF_PHOSPHOLIPASE_C_ACTIVITY	GO:1900274	5	-0.626951	-1.403147	0.131519	0.458186	1.000
614	GOBP_POSITIVE_REGULATION_OF_PHOSPHORUS_METABOL...	GO:0010562	24	-0.364279	-1.406203	0.084788	0.456868	1.000
615	GOBP_REGULATION_OF_EPITHELIAL_CELL_MIGRATION	GO:0010632	6	-0.572718	-1.408643	0.116331	0.456568	1.000
616	GOBP_POSITIVE_REGULATION_OF_MYELOID_CELL_DIFFE...	GO:0045639	7	-0.538151	-1.415053	0.110619	0.447727	1.000
617	GOBP_LEUKOCYTE_CELL_CELL_ADHESION	GO:0007159	6	-0.573299	-1.416138	0.113379	0.450191	1.000
618	GOBP_OSTEOCLAST_DIFFERENTIATION	GO:0030316	6	-0.585457	-1.417361	0.113586	0.452130	1.000
619	GOBP_POSITIVE_REGULATION_OF_LIPASE_ACTIVITY	GO:0060193	5	-0.626951	-1.419689	0.127155	0.452289	1.000
620	GOBP_POSITIVE_REGULATION_OF_SMALL_MOLECULE_MET...	GO:0062013	5	-0.617940	-1.420272	0.114486	0.455667	1.000
621	GOBP_COAGULATION	GO:0050817	8	-0.534606	-1.420283	0.108040	0.460287	1.000
622	GOBP_NEURON_APOPTOTIC_PROCESS	GO:0051402	12	-0.461134	-1.420525	0.109785	0.464664	1.000
623	GOBP_APOPTOTIC_SIGNALING_PATHWAY	GO:0097190	15	-0.434984	-1.425114	0.079646	0.459807	1.000
624	GOBP_PROTEIN_KINASE_B_SIGNALING	GO:0043491	6	-0.567364	-1.428146	0.106335	0.458683	1.000
625	GOBP_POSITIVE_REGULATION_OF_ANION_TRANSPORT	GO:1903793	9	-0.504673	-1.431539	0.097950	0.456758	1.000
626	GOBP_LOCOMOTION	GO:0040011	31	-0.364744	-1.435691	0.061008	0.453196	1.000
627	GOBP_OSTEOBLAST_DIFFERENTIATION	GO:0001649	5	-0.629492	-1.436988	0.116173	0.455562	1.000
628	GOBP_REGULATION_OF_BODY_FLUID_LEVELS	GO:0050878	11	-0.465844	-1.438244	0.087379	0.458236	1.000
629	GOBP_REGULATION_OF_MITOTIC_CELL_CYCLE	GO:0007346	5	-0.645340	-1.441063	0.101053	0.457130	1.000
630	GOBP_POSITIVE_REGULATION_OF_CELLULAR_COMPONENT...	GO:0044089	9	-0.514756	-1.443105	0.077626	0.458254	1.000
631	GOBP_REGULATION_OF_GTPASE_ACTIVITY	GO:0043087	5	-0.628153	-1.443191	0.097674	0.463328	1.000
632	GOBP_RESPONSE_TO_WOUNDING	GO:0009611	15	-0.440615	-1.444487	0.067757	0.465771	1.000
633	GOBP_REGULATION_OF_DEFENSE_RESPONSE	GO:0031347	7	-0.568663	-1.444987	0.095794	0.470247	1.000
634	GOBP_ACTIVATION_OF_PROTEIN_KINASE_ACTIVITY	GO:0032147	11	-0.482024	-1.446256	0.067470	0.473086	1.000
635	GOBP_PLATELET_ACTIVATION	GO:0030168	6	-0.597364	-1.448538	0.106195	0.474467	1.000
636	GOBP_REGULATION_OF_PROTEIN_MODIFICATION_PROCESS	GO:0031399	29	-0.370814	-1.459878	0.048593	0.457843	1.000
637	GOBP_REGULATION_OF_PROTEIN_STABILITY	GO:0031647	5	-0.640537	-1.460026	0.084862	0.463138	1.000
638	GOBP_POSITIVE_REGULATION_OF_CELL_CELL_ADHESION	GO:0022409	5	-0.647452	-1.462526	0.084112	0.463652	1.000
639	GOBP_REGULATION_OF_CELL_DIFFERENTIATION	GO:0045595	36	-0.360082	-1.470044	0.034392	0.453617	1.000
640	GOBP_REGULATION_OF_INFLAMMATORY_RESPONSE	GO:0050727	7	-0.568663	-1.471006	0.085253	0.457322	1.000
641	GOBP_NEGATIVE_REGULATION_OF_ION_TRANSPORT	GO:0043271	11	-0.486188	-1.479829	0.064815	0.445835	1.000
642	GOBP_REGULATION_OF_PEPTIDE_HORMONE_SECRETION	GO:0090276	6	-0.620792	-1.481370	0.068966	0.448876	1.000

Continuation of Table **S11**

	NAME	ID	SIZE	ES	NES	NOM.p.val	FDR.q.val	FWER.p.val
643	GOBP_NEUROINFLAMMATORY_RESPONSE	GO:0150076	5	-0.641995	-1.484543	0.082005	0.448162	1.000
644	GOBP_IMMUNE_SYSTEM_DEVELOPMENT	GO:0002520	17	-0.422402	-1.487282	0.060976	0.448288	1.000
645	GOBP_REGULATION_OF_PEPTIDYL_SERINE_PHOSPHORYLA...	GO:0033135	8	-0.560988	-1.488460	0.073563	0.451582	1.000
646	GOBP_POSITIVE_REGULATION_OF_LEUKOCYTE_CELL_CEL...	GO:1903039	5	-0.647452	-1.489033	0.082774	0.456418	1.000
647	GOBP_POSITIVE_REGULATION_OF_PEPTIDYL_SERINE_PH...	GO:0033138	8	-0.560988	-1.494309	0.075472	0.451005	1.000
648	REACTOME_HEMOSTASIS	R-RNO-109582	14	-0.457796	-1.498634	0.060533	0.447438	1.000
649	GOBP_CELLULAR_RESPONSE_TO_DNA_DAMAGE_STIMULUS	GO:0006974	5	-0.664264	-1.505843	0.080610	0.438424	1.000
650	GOBP_REGULATION_OF_CELL_ACTIVATION	GO:0050865	8	-0.564424	-1.507741	0.047414	0.440705	1.000
651	GOBP_MYOTUBE_DIFFERENTIATION	GO:0014902	5	-0.674591	-1.509458	0.080000	0.443495	1.000
652	GOBP_CELL_ACTIVATION	GO:0001775	21	-0.421811	-1.513681	0.058974	0.440404	1.000
653	GOBP_EPITHELIAL_CELL_PROLIFERATION	GO:0050673	12	-0.492856	-1.515965	0.059102	0.442164	1.000
654	GOBP_AMYLOID_BETA_METABOLIC_PROCESS	GO:0050435	5	-0.682707	-1.525907	0.059671	0.427671	1.000
655	GOBP_NEGATIVE_REGULATION_OF_DEVELOPMENTAL_PROCESS	GO:0051093	13	-0.484403	-1.528867	0.055556	0.428761	1.000
656	GOBP_PEPTIDYL_AMINO_ACID_MODIFICATION	GO:0018193	22	-0.419766	-1.532452	0.037879	0.427877	1.000
657	GOBP_MYELOID_LEUKOCYTE_DIFFERENTIATION	GO:0002573	11	-0.509890	-1.541709	0.064516	0.416116	1.000
658	GOBP_REGULATION_OF_CYTOSKELETON_ORGANIZATION	GO:0051493	5	-0.672583	-1.548426	0.050439	0.407886	1.000
659	GOBP_NEGATIVE_REGULATION_OF_CELL_DIFFERENTIATION	GO:0045596	13	-0.484403	-1.548510	0.056931	0.414387	1.000
660	GOBP_REGULATION_OF_MULTICELLULAR_ORGANISMAL_DE...	GO:2000026	31	-0.394403	-1.554820	0.025510	0.408844	1.000
661	REACTOME_CELL_CYCLE_MITOTIC	R-RNO-69278	5	-0.674765	-1.559186	0.063781	0.406821	1.000
662	GOBP_NEGATIVE_REGULATION_OF_CELL_DEVELOPMENT	GO:0010721	6	-0.633310	-1.568572	0.052521	0.394476	1.000
663	GOBP_AMYLOID_PRECURSOR_PROTEIN_CATABOLIC_PROCESS	GO:0042987	5	-0.682707	-1.583778	0.075055	0.369826	1.000
664	GOBP_POSITIVE_REGULATION_OF_HEMOPOIESIS	GO:1903708	7	-0.624195	-1.589717	0.055046	0.364446	1.000
665	GOBP_AMYLOID_PRECURSOR_PROTEIN_METABOLIC_PROCESS	GO:0042982	5	-0.682707	-1.597760	0.054374	0.354896	1.000
666	GOBP_POSITIVE_REGULATION_OF_RESPONSE_TO_EXTERN...	GO:0032103	9	-0.571632	-1.600836	0.042654	0.354805	1.000
667	REACTOME_BETA_CATENIN_INDEPENDENT_WNT_SIGNALING	R-RNO-3858494	5	-0.713583	-1.603333	0.046771	0.356887	1.000
668	GOBP_REGULATION_OF_RESPONSE_TO_STRESS	GO:0080134	19	-0.460005	-1.611166	0.035800	0.349061	1.000
669	GOBP_NEURON_PROJECTION_GUIDANCE	GO:0097485	9	-0.581613	-1.625345	0.039352	0.329714	1.000
670	GOBP_REGULATION_OF_PROTEIN_LOCALIZATION	GO:0032880	18	-0.472000	-1.633561	0.041667	0.322134	1.000
671	GOBP_CELL_POPULATION_PROLIFERATION	GO:0008283	31	-0.418403	-1.641530	0.017032	0.313427	1.000
672	GOBP_POSITIVE_REGULATION_OF_SMOOTH_MUSCLE_CELL...	GO:0048661	5	-0.718896	-1.641695	0.040632	0.319717	1.000
673	GOBP_NEGATIVE_REGULATION_OF_APOPTOTIC_SIGNALIN...	GO:2001234	7	-0.632371	-1.644545	0.034169	0.320978	1.000
674	GOBP_ESTABLISHMENT_OF_PROTEIN_LOCALIZATION_TO_...	GO:0072594	6	-0.691121	-1.649947	0.033860	0.319172	1.000
675	GOBP_ERBB_SIGNALING_PATHWAY	GO:0038127	5	-0.728548	-1.656747	0.049661	0.313216	1.000
676	GOBP_WOUND_HEALING	GO:0042060	13	-0.519775	-1.665462	0.019231	0.303139	1.000
677	GOBP_REGULATION_OF_INTRACELLULAR_SIGNAL_TRANSD...	GO:1902531	21	-0.463694	-1.666739	0.011933	0.308253	1.000
678	GOBP_POSITIVE_REGULATION_OF_MYELOID_LEUKOCYTE_...	GO:0002763	6	-0.675452	-1.671388	0.030501	0.306890	1.000
679	GOBP_RESPONSE_TO_GROWTH_FACTOR	GO:0070848	18	-0.481308	-1.681438	0.017370	0.294907	1.000
680	GOBP_TAXIS	GO:0042330	14	-0.515160	-1.683985	0.027335	0.298163	1.000
681	GOBP_POSITIVE_REGULATION_OF_PHOSPHATIDYLINOSIT...	GO:0014068	5	-0.749227	-1.684059	0.026906	0.305932	1.000
682	REACTOME_SIGNALING_BY_WNT	R-RNO-195721	7	-0.643566	-1.688209	0.029345	0.306713	1.000
683	GOBP_INOSITOL_LIPID_MEDIATED_SIGNALING	GO:0048017	8	-0.614242	-1.692759	0.026570	0.306614	1.000
684	GOBP_REGULATION_OF_MAP_KINASE_ACTIVITY	GO:0043405	9	-0.616295	-1.701887	0.013667	0.296605	0.999
685	GOBP_REGULATION_OF_PEPTIDE_SECRETION	GO:0002791	7	-0.674308	-1.708127	0.027842	0.292878	0.999

Continuation of Table **S11**

	NAME	ID	SIZE	ES	NES	NOM.p.val	FDR.q.val	FWER.p.val
686	GOBP_POSITIVE_REGULATION_OF_CELLULAR_PROTEIN_L...	GO:1903829	7	-0.677764	-1.721510	0.015625	0.278120	0.998
687	GOBP_ANATOMICAL_STRUCTURE_FORMATION_INVOLVED_I...	GO:0048646	20	-0.481020	-1.750632	0.009804	0.239676	0.994
688	GOBP_NEGATIVE_REGULATION_OF_CELL_DEATH	GO:0060548	22	-0.470940	-1.752967	0.014286	0.243146	0.994
689	GOBP_POSITIVE_REGULATION_OF_INTRACELLULAR_SIGN...	GO:1902533	14	-0.542037	-1.759478	0.008791	0.240231	0.993
690	GOBP_REGULATION_OF_ORGANELLE_ORGANIZATION	GO:0033043	14	-0.564031	-1.767300	0.006928	0.235417	0.991
691	GOBP_CELL_MIGRATION	GO:0016477	22	-0.486011	-1.784100	0.004975	0.218312	0.985
692	REACTOME_CELL_CYCLE	R-RNO-1640170	6	-0.754056	-1.798801	0.010438	0.203691	0.978
693	GOBP_PEPTIDYL_SERINE_MODIFICATION	GO:0018209	12	-0.586349	-1.806308	0.018561	0.200268	0.973
694	GOBP_NUCLEAR_TRANSPORT	GO:0051169	5	-0.792643	-1.824440	0.006608	0.180546	0.957
695	GOBP_SMOOTH_MUSCLE_CELL_PROLIFERATION	GO:0048659	6	-0.766386	-1.824954	0.011338	0.187454	0.957
696	GOBP_REGULATION_OF_VASCULAR_ASSOCIATED_SMOOTH_...	GO:1904705	5	-0.817806	-1.829163	0.008529	0.188675	0.953
697	GOBP_REGULATION_OF_CELLULAR_COMPONENT_MOVEMENT	GO:0051270	15	-0.566820	-1.834603	0.005076	0.189225	0.939
698	GOBP_POSITIVE_REGULATION_OF_CELLULAR_COMPONENT...	GO:0051130	24	-0.493686	-1.856780	0.004988	0.166144	0.911
699	GOBP_POSITIVE_REGULATION_OF_PROTEIN_SERINE_THR...	GO:0071902	9	-0.664176	-1.886095	0.004762	0.138093	0.852
700	GOBP_REGULATION_OF_CELL_ADHESION	GO:0030155	13	-0.598240	-1.886961	0.014151	0.144055	0.851
701	GOBP_ERK1_AND_ERK2_CASCADE	GO:0070371	5	-0.841075	-1.891822	0.004338	0.145961	0.836
702	REACTOME_PLATELET_ACTIVATION_SIGNALING_AND_AGG...	R-HSA-76002	7	-0.725904	-1.901326	0.004484	0.141143	0.815
703	GOBP_PHOSPHATIDYLINOSITOL_3_KINASE_SIGNALING	GO:0014065	7	-0.745647	-1.911279	0.006849	0.137642	0.777
704	GOBP_POSITIVE_REGULATION_OF_ESTABLISHMENT_OF_P...	GO:1904951	5	-0.856813	-1.936586	0.004535	0.116525	0.692
705	GOBP_REGULATION_OF_MAPK_CASCADE	GO:0043408	13	-0.609127	-1.948559	0.002481	0.111934	0.650
706	GOBP_REGULATION_OF_PHOSPHATIDYLINOSITOL_3_KINA...	GO:0014066	6	-0.783878	-1.963295	0.004237	0.102936	0.599
707	GOBP_POSITIVE_REGULATION_OF_CELL_ADHESION	GO:0045785	7	-0.752015	-1.973063	0.004695	0.101850	0.566
708	GOBP_POSITIVE_REGULATION_OF_CELL_POPULATION_PR...	GO:0008284	21	-0.557474	-2.015513	0.000000	0.074913	0.441
709	GOBP_ACTIVATION_OF_MAPK_ACTIVITY	GO:0000187	6	-0.838398	-2.018368	0.000000	0.080351	0.431
710	GOBP_REGULATION_OF_CELL_CELL_ADHESION	GO:0022407	9	-0.756126	-2.065082	0.004202	0.055305	0.294
711	GOBP_POSITIVE_REGULATION_OF_LOCOMOTION	GO:0040017	11	-0.707142	-2.087959	0.000000	0.049878	0.249
712	GOBP_POSITIVE_REGULATION_OF_MAP_KINASE_ACTIVITY	GO:0043406	6	-0.838398	-2.089277	0.000000	0.056779	0.248
713	GOBP_OSSIFICATION	GO:0001503	9	-0.771636	-2.111545	0.000000	0.050400	0.192
714	GOBP_POSITIVE_REGULATION_OF_MAPK_CASCADE	GO:0043410	9	-0.768611	-2.153635	0.000000	0.039986	0.130
715	GOBP_POSITIVE_REGULATION_OF_ORGANELLE_ORGANIZA...	GO:0010638	10	-0.747409	-2.157120	0.002299	0.047046	0.122
716	GOBP_REGULATION_OF_PEPTIDE_TRANSPORT	GO:0090087	10	-0.738994	-2.159328	0.000000	0.060828	0.118
717	GOBP_MUSCLE_CELL_PROLIFERATION	GO:0033002	8	-0.792860	-2.163419	0.000000	0.089101	0.115
718	GOBP_REGULATION_OF_RESPONSE_TO_EXTERNAL_STIMULUS	GO:0032101	15	-0.686656	-2.283672	0.000000	0.037286	0.025

End of Table

Supplementary Table S12: CTX-M early profile (8 DPL peak) GSEA results.

Begin of Table **S12**

	NAME	ID	SIZE	ES	NES	NOM.p.val	FDR.q.val	FWER.p.val
0	REACTOME_UNBLOCKING_OF_NMDA_RECEPTORS_GLUTAMAT...	R-RNO-438066	12	0.599549	1.767262	0.007737	1.000000	0.986
1	REACTOME_NEURONAL_SYSTEM	R-RNO-112316	26	0.481141	1.766692	0.009091	1.000000	0.987
2	REACTOME_TRANSMISSION_ACROSS_CHEMICAL_SYNAPSES	R-RNO-112315	23	0.489211	1.753888	0.005576	1.000000	0.992
3	GOBP_G_PROTEIN_COUPLED_RECEPTOR_SIGNALING_PATHWAY	GO:0007186	18	0.520962	1.715385	0.024299	1.000000	0.998
4	REACTOME_POST_TRANSLATIONAL_PROTEIN_MODIFICATION	R-RNO-597592	7	0.670988	1.667953	0.015625	1.000000	1.000
5	GOBP_PROTEIN_LOCALIZATION_TO_CELL_PERIPHERY	GO:1990778	12	0.571855	1.664676	0.019891	1.000000	1.000
6	REACTOME_NEGATIVE_REGULATION_OF_NMDA_RECEPTOR_...	R-HSA-961732	8	0.625000	1.654605	0.025180	1.000000	1.000
7	REACTOME_NEUROTRANSMITTER_RECEPTORS_AND_POSTSY...	R-RNO-112314	22	0.467556	1.650605	0.016981	1.000000	1.000
8	GOBP_CELLULAR_RESPONSE_TO_ALCOHOL	GO:0097306	5	0.733359	1.646519	0.015534	0.958915	1.000
9	REACTOME_ASSEMBLY_AND_CELL_SURFACE_PRESENTATIO...	R-RNO-9609736	8	0.625000	1.636965	0.033663	0.921557	1.000
10	REACTOME_ACTIVATION_OF_NMDA_RECEPTORS_AND_POST...	R-RNO-442755	16	0.499603	1.635245	0.039106	0.850281	1.000
11	GOBP_PROTEIN_LOCALIZATION_TO_CELL_JUNCTION	GO:1902414	6	0.682927	1.614915	0.025145	0.895684	1.000
12	GOBP_CELLULAR_RESPONSE_TO_KETONE	GO:1901655	5	0.746988	1.600881	0.037951	0.904788	1.000
13	GOBP_GLUTAMATE_RECEPTOR_SIGNALING_PATHWAY	GO:0007215	17	0.479191	1.584430	0.038610	0.931415	1.000
14	REACTOME_LONG_TERM_POTENTIATION	R-HSA-9620244	10	0.565748	1.570278	0.047714	0.945878	1.000
15	GOBP_ADENYLATE_CYCLASE_INHIBITING_G_PROTEIN_CO...	GO:0007193	9	0.566426	1.544682	0.062147	1.000000	1.000
16	GOBP_PROTEIN_LOCALIZATION_TO_MEMBRANE	GO:0072657	14	0.497549	1.540480	0.049702	0.995066	1.000
17	REACTOME_SYNAPTIC_ADHESION_LIKE_MOLECULES	R-RNO-8849932	9	0.558248	1.538382	0.062030	0.951029	1.000
18	GOBP_ORGANIC_HYDROXY_COMPOUND_TRANSPORT	GO:0015850	6	0.649641	1.522391	0.077821	0.990228	1.000
19	HALLMARK_PI3K_AKT_MTOR_SIGNALING	M5923	6	0.638770	1.513777	0.073705	0.987905	1.000
20	GOBP_POSITIVE_REGULATION_OF_CELL_DEATH	GO:0010942	19	0.447558	1.512260	0.047348	0.948185	1.000
21	GOBP_REGULATION_OF_NMDA_RECEPTOR_ACTIVITY	GO:2000310	10	0.531271	1.505432	0.068053	0.940561	1.000
22	GOBP_POSITIVE_REGULATION_OF_IMMUNE_RESPONSE	GO:0050778	9	0.554897	1.502780	0.056075	0.912790	1.000
23	GOBP_PROTEIN_LOCALIZATION_TO_POSTSYNAPSE	GO:0062237	5	0.674699	1.501587	0.059730	0.880402	1.000
24	GOBP_PROTEIN_LOCALIZATION_TO_SYNAPSE	GO:0035418	5	0.674699	1.493818	0.069388	0.881773	1.000
25	GOBP_IONOTROPIC_GLUTAMATE_RECEPTOR_SIGNALING_P...	GO:0035235	9	0.556379	1.486816	0.063116	0.882940	1.000
26	GOBP_SMALL_MOLECULE_METABOLIC_PROCESS	GO:0044281	15	0.450474	1.467918	0.072937	0.938840	1.000
27	REACTOME_PROTEIN_PROTEIN_INTERACTIONS_AT_SYNAPSES	R-RNO-6794362	12	0.501960	1.467711	0.062264	0.906280	1.000
28	GOBP_CARBOHYDRATE_METABOLIC_PROCESS	GO:0005975	9	0.526143	1.443969	0.098456	0.987862	1.000
29	GOBP_CELL_SURFACE_RECEPTOR_SIGNALING_PATHWAY_I...	GO:1905114	15	0.466804	1.443516	0.077799	0.957145	1.000
30	REACTOME_RAS_ACTIVATION_UPON_CA2_INFLUX_THROUG...	R-HSA-442982	6	0.609756	1.441100	0.103448	0.937064	1.000
31	GOBP_ACTIVATION_OF_IMMUNE_RESPONSE	GO:0002253	5	0.649649	1.440250	0.108499	0.911991	1.000
32	GOBP_MAINTENANCE_OF_LOCATION	GO:0051235	7	0.572121	1.417574	0.095506	0.989674	1.000
33	REACTOME_LEISHMANIA_INFECTION	R-HSA-9658195	10	0.500275	1.416263	0.096045	0.966601	1.000
34	GOBP_REGULATION_OF_POSTSYNAPTIC_MEMBRANE_POTEN...	GO:0060078	14	0.442009	1.402884	0.098113	1.000000	1.000
35	GOBP_IMMUNE_EFFECTOR_PROCESS	GO:0002252	10	0.497732	1.398479	0.105364	0.996011	1.000
36	REACTOME_ANTI_INFLAMMATORY_RESPONSE_FAVOURING_...	R-HSA-9662851	5	0.622568	1.393101	0.127953	0.993792	1.000
37	GOBP_VESICLE_MEDIATED_TRANSPORT_IN_SYNAPSE	GO:0099003	7	0.555577	1.391908	0.122000	0.973296	1.000
38	GOBP_NEGATIVE_REGULATION_OF_MOLECULAR_FUNCTION	GO:0044092	24	0.392892	1.389408	0.087657	0.959330	1.000
39	GOBP_CATION_TRANSPORT	GO:0006812	25	0.385385	1.387241	0.103774	0.944523	1.000
40	REACTOME_CIRCADIAN_CLOCK	R-HSA-400253	5	0.618496	1.386783	0.122841	0.923209	1.000

Continuation of Table **S12**

	NAME	ID	SIZE	ES	NES	NOM.p.val	FDR.q.val	FWER.p.val
41	GOBP_POSTSYNAPSE_ORGANIZATION	GO:0099173	10	0.480596	1.386317	0.124521	0.903493	1.000
42	GOBP_PROTEOLYSIS	GO:0006508	17	0.424658	1.385751	0.112132	0.885159	1.000
43	GOBP_RESPONSE_TO_ALCOHOL	GO:0097305	11	0.471336	1.369583	0.119565	0.930747	1.000
44	GOBP_SEXUAL_REPRODUCTION	GO:0019953	10	0.483559	1.369220	0.130515	0.911607	1.000
45	GOBP_CELLULAR_RESPONSE_TO_PEPTIDE_HORMONE_STIM...	GO:0071375	9	0.505884	1.362149	0.110887	0.920825	1.000
46	REACTOME_NEUREXINS_AND_NEUROLIGINS	R-RNO-6794361	9	0.503978	1.360998	0.122807	0.905795	1.000
47	REACTOME_INFECTIOUS_DISEASE	R-HSA-5663205	13	0.441916	1.359202	0.113949	0.894054	1.000
48	GOBP_CIRCADIAN_REGULATION_OF_GENE_EXPRESSION	GO:0032922	5	0.609411	1.355397	0.164234	0.890780	1.000
49	GOBP_MEMBRANE_PROTEIN_PROTEOLYSIS	GO:0033619	6	0.570490	1.342228	0.120459	0.924599	1.000
50	GOBP_REGULATION_OF_CARBOHYDRATE_METABOLIC_PROCESS	GO:0006109	5	0.600490	1.338495	0.163306	0.920831	1.000
51	GOBP_REGULATION_OF_CARBOHYDRATE_BIOSYNTHETIC_P...	GO:0043255	5	0.600490	1.328573	0.166667	0.941821	1.000
52	GOBP_CELLULAR_COMPONENT_MAINTENANCE	GO:0043954	5	0.581473	1.328249	0.156701	0.925489	1.000
53	GOBP_INORGANIC_ION_TRANSMEMBRANE_TRANSPORT	GO:0098660	9	0.490646	1.328124	0.153696	0.908666	1.000
54	GOBP_CARBOHYDRATE_BIOSYNTHETIC_PROCESS	GO:0016051	5	0.600490	1.324532	0.180233	0.905341	1.000
55	GOBP_EXCITATORY_CHEMICAL_SYNAPTIC_TRANSMISSION	GO:0098976	5	0.602410	1.319737	0.164329	0.907662	1.000
56	GOBP_RESPONSE_TO_DRUG	GO:0042493	11	0.457964	1.316027	0.160517	0.905523	1.000
57	GOBP_ORGANIC_HYDROXY_COMPOUND_METABOLIC_PROCESS	GO:1901615	7	0.534261	1.314287	0.156190	0.896015	1.000
58	GOBP_PROTEIN_LOCALIZATION_TO_PLASMA_MEMBRANE	GO:0072659	7	0.530609	1.302524	0.169524	0.925840	1.000
59	REACTOME_INNATE_IMMUNE_SYSTEM	R-RNO-168249	16	0.397738	1.291572	0.165049	0.951549	1.000
60	GOBP_EXOCYTOSIS	GO:0006887	11	0.447674	1.290058	0.160305	0.941658	1.000
61	GOBP_CELL_ACTIVATION_INVOLVED_IN_IMMUNE_RESPONSE	GO:0002263	6	0.534412	1.287372	0.186992	0.935826	1.000
62	GOBP_CELL_CELL_SIGNALING_BY_WNT	GO:0198738	7	0.504783	1.282608	0.186792	0.938801	1.000
63	GOBP_MYELOID_LEUKOCYTE_MEDIATED_IMMUNITY	GO:0002444	6	0.534412	1.273605	0.201887	0.958142	1.000
64	GOBP_REGULATION_OF_ION_TRANSPORT	GO:0043269	36	0.332705	1.270922	0.154696	0.952589	1.000
65	REACTOME_EPH_EPHRIN_SIGNALING	R-RNO-2682334	6	0.536661	1.269958	0.219608	0.941435	1.000
66	GOBP_REGULATION_OF_WNT_SIGNALING_PATHWAY	GO:0030111	5	0.579201	1.267367	0.201550	0.936521	1.000
67	GOBP_MALE_GAMETE_GENERATION	GO:0048232	5	0.582629	1.258748	0.227898	0.954460	1.000
68	GOBP_CATION_TRANSMEMBRANE_TRANSPORT	GO:0098655	18	0.375942	1.251294	0.176692	0.967766	1.000
69	REACTOME_NEUTROPHIL_DEGRANULATION	R-RNO-6798695	6	0.534412	1.247381	0.210821	0.967722	1.000
70	GOBP_NEGATIVE_REGULATION_OF_KINASE_ACTIVITY	GO:0033673	5	0.556317	1.243092	0.215190	0.970109	1.000
71	GOBP_RESPONSE_TO_KETONE	GO:1901654	7	0.495849	1.236282	0.204771	0.981859	1.000
72	GOBP_CARBOHYDRATE_DERIVATIVE_METABOLIC_PROCESS	GO:1901135	7	0.491393	1.234215	0.220561	0.976181	1.000
73	GOBP_POSITIVE_REGULATION_OF_APOPTOTIC_SIGNALIN...	GO:2001235	5	0.552899	1.227370	0.229167	0.987272	1.000
74	GOBP_NEGATIVE_REGULATION_OF_TRANSFERASE_ACTIVITY	GO:0051348	5	0.556317	1.225645	0.258189	0.980182	1.000
75	GOBP_DIVALENT_INORGANIC_CATION_HOMEOSTASIS	GO:0072507	13	0.396348	1.221757	0.223529	0.980908	1.000
76	GOBP_CELLULAR_RESPONSE_TO_PEPTIDE	GO:1901653	13	0.402694	1.221236	0.214552	0.969922	1.000
77	GOBP_METAL_ION_HOMEOSTASIS	GO:0055065	14	0.382044	1.217116	0.217472	0.972269	1.000
78	GOBP_REGULATION_OF_CYTOSOLIC_CALCIUM_ION_CONCE...	GO:0051480	13	0.396348	1.215116	0.209016	0.966724	1.000
79	REACTOME_SIGNALING_BY_GPCR	R-RNO-372790	22	0.344419	1.212778	0.212406	0.963190	1.000
80	GOBP_RESPONSE_TO_ETHANOL	GO:0045471	5	0.560562	1.212334	0.248555	0.952796	1.000
81	REACTOME_DAG_AND_IP3_SIGNALING	R-RNO-1489509	8	0.460017	1.202414	0.219282	0.974958	1.000
82	REACTOME_TRAFFICKING_OF_AMPA_RECEPTORS	R-RNO-399719	11	0.412893	1.198867	0.248148	0.975097	1.000
83	GOBP_GAMETE_GENERATION	GO:0007276	9	0.437644	1.198554	0.227687	0.964709	1.000

Continuation of Table **S12**

	NAME	ID	SIZE	ES	NES	NOM.p.val	FDR.q.val	FWER.p.val
84	GOBP_REGULATION_OF_PROTEIN_BINDING	GO:0043393	7	0.467713	1.197843	0.249035	0.955764	1.000
85	GOBP_CELLULAR_RESPONSE_TO_HORMONE_STIMULUS	GO:0032870	11	0.414508	1.194315	0.250454	0.956450	1.000
86	GOBP_ADENYLATE_CYCLASE_MODULATING_G_PROTEIN_CO...	GO:0007188	11	0.404822	1.188431	0.258964	0.964689	1.000
87	GOBP_CELL_GROWTH	GO:0016049	10	0.418957	1.187323	0.243446	0.957192	1.000
88	GOBP_RESPONSE_TO_PEPTIDE_HORMONE	GO:0043434	13	0.382065	1.179166	0.258491	0.972906	1.000
89	GOBP_POLYSACCHARIDE_METABOLIC_PROCESS	GO:0005976	5	0.538739	1.174383	0.302804	0.977399	1.000
90	GOBP_GENERATION_OF_PRECURSOR_METABOLITES_AND_E...	GO:0006091	5	0.531151	1.174191	0.261364	0.967326	1.000
91	GOBP_REGULATION_OF_TRANSPORT	GO:0051049	44	0.297782	1.173940	0.246454	0.957650	1.000
92	GOBP_RESPONSE_TO_TUMOR_NECROSIS_FACTOR	GO:0034612	7	0.468438	1.170961	0.264325	0.956953	1.000
93	REACTOME_ONCOGENIC_MAPK_SIGNALING	R-HSA-6802957	5	0.525484	1.167804	0.291913	0.957541	1.000
94	REACTOME_TOLL_LIKE_RECEPTOR_CASCADES	R-RNO-168898	8	0.443908	1.167086	0.257692	0.949763	1.000
95	GOBP_NEUROTRANSMITTER_SECRETION	GO:0007269	6	0.495026	1.166082	0.257194	0.942995	1.000
96	REACTOME_TOLL_LIKE_RECEPTOR_9_TLR9_CASCADE	R-RNO-168138	8	0.443908	1.164425	0.264815	0.938456	1.000
97	GOBP_ORGANONITROGEN_COMPOUND_BIOSYNTHETIC_PROCESS	GO:1901566	13	0.376284	1.155559	0.291045	0.957207	1.000
98	REACTOME_MYD88_INDEPENDENT_TLR4_CASCADE	R-RNO-166166	8	0.443908	1.152916	0.299228	0.955563	1.000
99	GOBP_REGULATION_OF_IMMUNE_RESPONSE	GO:0050776	12	0.388875	1.152083	0.285981	0.948296	1.000
100	GOBP_HOMEOSTASIS_OF_NUMBER_OF_CELLS	GO:0048872	5	0.516576	1.151661	0.297619	0.940228	1.000
101	GOBP_REGULATION_OF_VASCULATURE_DEVELOPMENT	GO:1901342	5	0.521127	1.145698	0.310277	0.949348	1.000
102	GOBP_NEURON_DEVELOPMENT	GO:0048666	33	0.301410	1.144035	0.295956	0.945291	1.000
103	GOBP_REGULATION_OF_CATION_TRANSMEMBRANE_TRANSPORT	GO:1904062	16	0.350693	1.137787	0.304991	0.955541	1.000
104	REACTOME_TOLL_LIKE_RECEPTOR_TLR1_TLR2_CASCADE	R-RNO-168179	8	0.443908	1.135608	0.312871	0.953069	1.000
105	GOBP_CELL_PART_MORPHOGENESIS	GO:0032990	24	0.307266	1.121845	0.293333	0.987063	1.000
106	GOBP_CHEMICAL_HOMEOSTASIS	GO:0048878	23	0.314824	1.120183	0.317739	0.982921	1.000
107	GOBP_CHEMICAL_SYNAPTIC_TRANSMISSION_POSTSYNAPTIC	GO:0099565	9	0.416186	1.117581	0.312500	0.981564	1.000
108	GOBP_REGULATION_OF_CELL_CYCLE_PROCESS	GO:0010564	5	0.504774	1.114742	0.315582	0.981262	1.000
109	GOBP_CELLULAR_COMPONENT_MORPHOGENESIS	GO:0032989	24	0.307266	1.112686	0.300000	0.978967	1.000
110	GOBP_CELL_MORPHOGENESIS	GO:0000902	24	0.307266	1.104890	0.308571	0.993441	1.000
111	GOBP_POSITIVE_REGULATION_OF_PROTEOLYSIS	GO:0045862	8	0.417302	1.100775	0.316479	0.997177	1.000
112	GOBP_SENSORY_PERCEPTION	GO:0007600	9	0.409659	1.100690	0.343396	0.988610	1.000
113	GOBP_AXON_DEVELOPMENT	GO:0061564	18	0.326472	1.100681	0.340909	0.980000	1.000
114	REACTOME_ADAPTIVE_IMMUNE_SYSTEM	R-RNO-1280218	9	0.409772	1.096708	0.345865	0.983661	1.000
115	GOBP_REGULATION_OF_NEUROTRANSMITTER_RECEPTOR_A...	GO:0099601	13	0.353560	1.094960	0.312977	0.980572	1.000
116	GOBP_ORGANOPHOSPHATE_METABOLIC_PROCESS	GO:0019637	7	0.434539	1.090255	0.362173	0.985966	1.000
117	GOBP_RESPONSE_TO_ORGANIC_CYCLIC_COMPOUND	GO:0014070	20	0.314935	1.089081	0.324428	0.981338	1.000
118	GOBP_REGULATION_OF_CELLULAR_COMPONENT_SIZE	GO:0032535	7	0.435292	1.087705	0.350294	0.977140	1.000
119	GOBP_REGULATION_OF_NEUROTRANSMITTER_TRANSPORT	GO:0051588	6	0.459410	1.085058	0.354709	0.976954	1.000
120	GOBP_REGULATION_OF_ANATOMICAL_STRUCTURE_MORPHO...	GO:0022603	17	0.325554	1.080761	0.369231	0.981494	1.000
121	GOBP_CELL_MORPHOGENESIS_INVOLVED_IN_DIFFERENTI...	GO:0000904	21	0.307997	1.078785	0.360687	0.979547	1.000
122	GOBP_DENDRITIC_SPINE_DEVELOPMENT	GO:0060996	6	0.458716	1.077025	0.360000	0.976507	1.000
123	GOBP_CALCIUM_ION_TRANSMEMBRANE_TRANSPORT	GO:0070588	7	0.419594	1.074369	0.366279	0.976179	1.000
124	GOBP_REGULATION_OF_PROTEIN_LOCALIZATION_TO_CEL...	GO:1904375	6	0.459751	1.068439	0.383364	0.985589	1.000
125	GOBP_CELLULAR_RESPONSE_TO_ABIOTIC_STIMULUS	GO:0071214	7	0.424712	1.068338	0.379747	0.978040	1.000
126	GOBP_REGULATION_OF_LIPID_LOCALIZATION	GO:1905952	5	0.471522	1.062273	0.355899	0.987476	1.000

Continuation of Table **S12**

	NAME	ID	SIZE	ES	NES	NOM.p.val	FDR.q.val	FWER.p.val
127	GOBP_NEURON_DIFFERENTIATION	GO:0030182	36	0.270440	1.061561	0.376426	0.981945	1.000
128	GOBP_TISSUE_MORPHOGENESIS	GO:0048729	7	0.433176	1.057567	0.389299	0.986120	1.000
129	REACTOME_MAPK_FAMILY_SIGNALING_CASCADES	R-RNO-5683057	12	0.363356	1.057383	0.360000	0.979067	1.000
130	GOBP_CELLULAR_CARBOHYDRATE_METABOLIC_PROCESS	GO:0044262	5	0.479705	1.055874	0.394286	0.975939	1.000
131	REACTOME_INTERFERON_SIGNALING	R-RNO-913531	6	0.462584	1.054918	0.388031	0.971249	1.000
132	GOBP_NEGATIVE_REGULATION_OF_CATALYTIC_ACTIVITY	GO:0043086	17	0.321296	1.054675	0.394636	0.964675	1.000
133	GOBP_MEMBRANE_ORGANIZATION	GO:0061024	12	0.348778	1.054186	0.394052	0.958887	1.000
134	GOBP_REGULATION_OF_CELL_DEATH	GO:0010941	34	0.271458	1.050272	0.382716	0.962473	1.000
135	GOBP_INNATE_IMMUNE_RESPONSE	GO:0045087	7	0.416964	1.046135	0.389925	0.967067	1.000
136	GOBP_CELL_MORPHOGENESIS_INVOLVED_IN_NEURON_DIF...	GO:0048667	21	0.307997	1.044866	0.400000	0.963384	1.000
137	GOBP_CALCIUM_ION_TRANSPORT_INTO_CYTOSOL	GO:0060402	7	0.419594	1.044473	0.393881	0.957474	1.000
138	GOBP_ION_HOMEOSTASIS	GO:0050801	16	0.323956	1.043940	0.391386	0.951886	1.000
139	GOBP_NEGATIVE_REGULATION_OF_BINDING	GO:0051100	6	0.450813	1.042911	0.399209	0.947883	1.000
140	GOBP_CELLULAR_RESPONSE_TO_ORGANIC_CYCLIC_COMPOUND	GO:0071407	13	0.339044	1.042247	0.375234	0.943052	1.000
141	GOBP_REGULATION_OF_CIRCADIAN_RHYTHM	GO:0042752	7	0.424438	1.040647	0.389210	0.940827	1.000
142	GOBP_CYTOSOLIC_CALCIUM_ION_TRANSPORT	GO:0060401	7	0.419594	1.039804	0.397032	0.936633	1.000
143	GOBP_BIOLOGICAL_PROCESS_INVOLVED_IN_SYMBIOTIC_...	GO:004440	11	0.355021	1.039016	0.407045	0.932487	1.000
144	GOBP_RESPONSE_TO_ABIOTIC_STIMULUS	GO:0009628	27	0.282635	1.037845	0.395221	0.929101	1.000
145	REACTOME_DISEASES_OF_SIGNAL_TRANSDUCTION_BY_GR...	R-HSA-5663202	12	0.343512	1.037206	0.387879	0.924519	1.000
146	GOBP_NEURON_PROJECTION_ORGANIZATION	GO:0106027	8	0.388486	1.033247	0.400756	0.928633	1.000
147	GOBP_REGULATION_OF_DNA_BINDING_TRANSCRIPTION_F...	GO:0051090	16	0.323758	1.033039	0.419729	0.922774	1.000
148	GOBP_IMMUNE_RESPONSE_REGULATING_SIGNALING_PATHWAY	GO:000276	8	0.392711	1.029455	0.404990	0.925761	1.000
149	GOBP_REGULATION_OF_MEMBRANE_POTENTIAL	GO:0042391	17	0.307845	1.029203	0.414313	0.920301	1.000
150	GOBP_CELLULAR_MACROMOLECULE_LOCALIZATION	GO:0070727	21	0.291373	1.028264	0.415385	0.916554	1.000
151	GOBP_NEGATIVE_REGULATION_OF_INTRACELLULAR_SIGN...	GO:1902532	7	0.407904	1.028053	0.408318	0.911033	1.000
152	GOBP_PURINE_CONTAINING_COMPOUND_METABOLIC_PROCESS	GO:0072521	5	0.457319	1.027097	0.413793	0.907393	1.000
153	GOBP_NEGATIVE_REGULATION_OF_HYDROLASE_ACTIVITY	GO:0051346	11	0.348570	1.024390	0.425182	0.908427	1.000
154	GOBP_FC_RECEPTOR_SIGNALING_PATHWAY	GO:0038093	8	0.392711	1.024169	0.420233	0.903077	1.000
155	GOBP_REGULATION_OF_SYNAPTIC_PLASTICITY	GO:0048167	21	0.289887	1.022758	0.427798	0.900573	1.000
156	GOBP_NEGATIVE_REGULATION_OF_LIPID_METABOLIC_PR...	GO:0045833	5	0.467277	1.022428	0.430528	0.895637	1.000
157	GOBP_MORPHOGENESIS_OF_AN_EPITHELIUM	GO:0002009	6	0.434657	1.014973	0.419776	0.908418	1.000
158	GOBP_POSITIVE_REGULATION_OF_LONG_TERM_SYNAPTIC...	GO:1900273	5	0.451589	1.006449	0.435845	0.923892	1.000
159	GOBP_CELLULAR_COMPONENT_DISASSEMBLY	GO:0022411	6	0.419650	1.006000	0.428571	0.919305	1.000
160	GOBP_CELLULAR_RESPONSE_TO_NITROGEN_COMPOUND	GO:1901699	20	0.292910	1.005229	0.440454	0.915517	1.000
161	GOBP_LIPID_METABOLIC_PROCESS	GO:0006629	10	0.370471	1.000882	0.422311	0.920492	1.000
162	GOBP_PROTEIN_CONTAINING_COMPLEX_SUBUNIT_ORGANI...	GO:0043933	14	0.317513	0.999257	0.474806	0.918982	1.000
163	GOBP_MYELOID_LEUKOCYTE_ACTIVATION	GO:0002274	8	0.384669	0.996918	0.433712	0.919149	1.000
164	GOBP_REGULATION_OF_POSTSYNAPSE_ORGANIZATION	GO:0099175	6	0.414724	0.996597	0.450943	0.914387	1.000
165	GOBP_LEUKOCYTE_MEDIATED_IMMUNITY	GO:0002443	7	0.396953	0.996004	0.450192	0.910261	1.000
166	GOBP_MONOCARBOXYLIC_ACID_METABOLIC_PROCESS	GO:0032787	6	0.414994	0.995828	0.471664	0.905301	1.000
167	GOBP_CELLULAR_RESPONSE_TO_LIPID	GO:0071396	15	0.312188	0.995270	0.479705	0.901291	1.000
168	GOBP_METAL_ION_TRANSPORT	GO:0030001	11	0.332734	0.991903	0.457249	0.904456	1.000
169	GOBP_REGULATION_OF_METAL_ION_TRANSPORT	GO:0010959	6	0.418273	0.984740	0.457594	0.916178	1.000

Continuation of Table **S12**

	NAME	ID	SIZE	ES	NES	NOM.p.val	FDR.q.val	FWER.p.val
170	GOBP_REGULATION_OF_PEPTIDASE_ACTIVITY	GO:0052547	10	0.347059	0.984723	0.465160	0.910851	1.000
171	GOBP_NEUROGENESIS	GO:0022008	40	0.250693	0.982420	0.466179	0.911267	1.000
172	GOBP_REGULATION_OF_CYSTEINE_TYPE_ENDOPEPTIDASE...	GO:2000116	9	0.357373	0.981355	0.485714	0.908361	1.000
173	GOBP_NEUROTRANSMITTER_TRANSPORT	GO:0006836	7	0.390018	0.979530	0.495088	0.907485	1.000
174	GOBP_CELLULAR_ION_HOMEOSTASIS	GO:0006873	14	0.312609	0.979095	0.479769	0.903351	1.000
175	GOBP_RESPONSE_TO_INORGANIC_SUBSTANCE	GO:0010035	19	0.283453	0.977421	0.488189	0.902277	1.000
176	GOBP_REGULATION_OF_CELL_PROJECTION_ORGANIZATION	GO:0031344	20	0.282854	0.975901	0.454714	0.900691	1.000
177	REACTOME_DEATH_RECEPTOR_SIGNALLING	R-RNO-73887	5	0.443503	0.974998	0.474187	0.897617	1.000
178	GOBP_POSITIVE_REGULATION_OF_PEPTIDASE_ACTIVITY	GO:0010952	7	0.382506	0.973969	0.486486	0.895059	1.000
179	GOBP_CELL_PROJECTION_ORGANIZATION	GO:0030030	34	0.247858	0.973406	0.498127	0.891501	1.000
180	GOBP_MONOAMINE_TRANSPORT	GO:0015844	5	0.436190	0.972685	0.470588	0.888112	1.000
181	REACTOME_CLASS_C_3_METABOTROPIC_GLUTAMATE_PHER...	R-RNO-420499	7	0.388363	0.970466	0.489066	0.888528	1.000
182	GOBP_CELL_CELL_SIGNALING	GO:0007267	56	0.246844	0.970390	0.516729	0.883836	1.000
183	GOBP_CELLULAR_RESPONSE_TO_BIOTIC_STIMULUS	GO:0071216	8	0.372045	0.968683	0.446565	0.882977	1.000
184	GOBP_REGULATION_OF_NEUROTRANSMITTER_LEVELS	GO:0001505	7	0.390018	0.963483	0.492000	0.889946	1.000
185	GOBP_REGULATION_OF_SMALL_MOLECULE_METABOLIC_PR...	GO:0062012	9	0.352176	0.962927	0.497186	0.886396	1.000
186	GOBP_RESPONSE_TO_PEPTIDE	GO:1901652	18	0.290173	0.962011	0.504817	0.883828	1.000
187	GOBP_POSITIVE_REGULATION_OF_DNA_BINDING_TRANSC...	GO:0051091	10	0.342296	0.961944	0.491651	0.879249	1.000
188	GOBP_CALCIUM_ION_TRANSPORT	GO:0006816	11	0.332734	0.957760	0.501010	0.883825	1.000
189	GOBP_CELL_CELL_JUNCTION_ORGANIZATION	GO:0045216	6	0.402150	0.954384	0.485149	0.886741	1.000
190	GOBP_NUCLEOBASE_CONTAINING_SMALL_MOLECULE_META...	GO:0055086	6	0.390479	0.951806	0.497248	0.887984	1.000
191	GOBP_POSITIVE_REGULATION_OF_CYSTEINE_TYPE_ENDO...	GO:2001056	7	0.382506	0.949186	0.504836	0.889052	1.000
192	GOBP_CELLULAR_RESPONSE_TO_MOLECULE_OF_BACTERIA...	GO:0071219	8	0.372045	0.946386	0.512195	0.890450	1.000
193	GOBP_REGULATION_OF_CELLULAR_RESPONSE_TO_STRESS	GO:0080135	9	0.347656	0.942644	0.478846	0.893936	1.000
194	GOBP_RESPONSE_TO_HORMONE	GO:0009725	20	0.269218	0.941289	0.533203	0.892206	1.000
195	GOBP_G_PROTEIN_COUPLED_GLUTAMATE_RECEPTOR_SIGN...	GO:0007216	8	0.355481	0.937343	0.504854	0.896167	1.000
196	GOBP_AMIDE_BIOSYNTHETIC_PROCESS	GO:0043604	7	0.370359	0.925960	0.520599	0.917174	1.000
197	REACTOME_TRANSCRIPTIONAL_REGULATION_BY_MECP2	R-RNO-8986944	7	0.367854	0.923250	0.558608	0.918499	1.000
198	GOBP_POSITIVE_REGULATION_OF_EPITHELIAL_CELL_MI...	GO:0010634	5	0.411448	0.922268	0.532673	0.916225	1.000
199	GOBP_PEPTIDE_BIOSYNTHETIC_PROCESS	GO:0043043	7	0.370359	0.919648	0.545977	0.917395	1.000
200	GOBP_RESPONSE_TO_UV	GO:0009411	5	0.418289	0.919499	0.544304	0.913182	1.000
201	REACTOME_TRAFFICKING_OF_GLUR2_CONTAINING_AMPA_...	R-RNO-416993	8	0.356793	0.916066	0.574510	0.916193	1.000
202	GOBP_NEGATIVE_REGULATION_OF_PHOSPHORYLATION	GO:0042326	7	0.358529	0.914931	0.565619	0.914247	1.000
203	GOBP_REGULATION_OF_NEURONAL_SYNAPTIC_PLASTICITY	GO:0048168	7	0.368715	0.912572	0.581262	0.914885	1.000
204	GOBP_CHROMATIN_ORGANIZATION	GO:0006325	5	0.412014	0.911626	0.558767	0.912375	1.000
205	GOBP_LIPID_LOCALIZATION	GO:0010876	10	0.321276	0.904167	0.568932	0.924083	1.000
206	REACTOME_GPCR_LIGAND_BINDING	R-RNO-500792	8	0.350766	0.903521	0.565056	0.921107	1.000
207	GOBP_REGULATION_OF_PROTEIN_LOCALIZATION_TO_MEM...	GO:1905475	7	0.350512	0.899644	0.544423	0.925018	1.000
208	GOBP_DENDRITE_DEVELOPMENT	GO:0016358	9	0.337296	0.899244	0.590164	0.921416	1.000
209	GOBP_REGULATION_OF_TRANSPORTER_ACTIVITY	GO:0032409	17	0.269605	0.897677	0.588583	0.920326	1.000
210	GOBP_TRANSMEMBRANE_TRANSPORT	GO:0055085	27	0.239632	0.895454	0.608133	0.920583	1.000
211	GOBP_PEPTIDE_METABOLIC_PROCESS	GO:0006518	12	0.291689	0.893916	0.579737	0.919553	1.000
212	GOBP_ORGANIC_ACID_TRANSPORT	GO:0015849	7	0.357413	0.891554	0.586207	0.920520	1.000

Continuation of Table **S12**

	NAME	ID	SIZE	ES	NES	NOM.p.val	FDR.q.val	FWER.p.val
213	REACTOME_INTEGRATION_OF_ENERGY_METABOLISM	R-RNO-163685	5	0.396270	0.888936	0.583658	0.921664	1.000
214	GOBP_DEFENSE_RESPONSE	GO:0006952	19	0.260661	0.883292	0.617375	0.929224	1.000
215	REACTOME_OPIOID_SIGNALLING	R-RNO-111885	12	0.292389	0.877554	0.619329	0.936954	1.000
216	GOBP_POSITIVE_REGULATION_OF_HYDROLASE_ACTIVITY	GO:0051345	16	0.268775	0.876779	0.609524	0.934229	1.000
217	REACTOME_G_PROTEIN_MEDIATED_EVENTS	R-RNO-112040	9	0.321144	0.874675	0.609195	0.934320	1.000
218	GOBP_CELLULAR_LIPID_METABOLIC_PROCESS	GO:0044255	7	0.343862	0.874139	0.606403	0.931096	1.000
219	GOBP_POSITIVE_REGULATION_OF_CELL_PROJECTION_OR...	GO:0031346	10	0.312916	0.871929	0.598425	0.931311	1.000
220	REACTOME_G_ALPHA_I_SIGNALLING_EVENTS	R-RNO-418594	18	0.259510	0.865754	0.654611	0.940146	1.000
221	GOBP_CIRCADIAN_RHYTHM	GO:0007623	13	0.287040	0.865731	0.642293	0.935966	1.000
222	GOBP_RESPONSE_TO_LIGHT_STIMULUS	GO:0009416	11	0.302633	0.865727	0.635659	0.931781	1.000
223	GOBP_ADENYLATE_CYCLASE_INHIBITING_G_PROTEIN_CO...	GO:0007196	5	0.380858	0.865708	0.603622	0.927653	1.000
224	GOBP_ORGANIC_ACID_METABOLIC_PROCESS	GO:0006082	7	0.337435	0.861323	0.651515	0.932241	1.000
225	GOBP_CARBOHYDRATE_HOMEOSTASIS	GO:0033500	7	0.340805	0.853592	0.611321	0.943670	1.000
226	GOBP_RESPONSE_TO_LIPID	GO:0033993	19	0.245778	0.850103	0.686567	0.946427	1.000
227	GOBP_REGULATION_OF_NEURON_PROJECTION_DEVELOPMENT	GO:0010975	16	0.256981	0.847185	0.640884	0.947871	1.000
228	GOBP_CELL_CYCLE_PROCESS	GO:0022402	11	0.289247	0.844740	0.644841	0.948574	1.000
229	GOBP_REGULATION_OF_PROTEIN_KINASE_ACTIVITY	GO:0045859	22	0.238338	0.843939	0.680529	0.945904	1.000
230	REACTOME_MEMBRANE_TRAFFICKING	R-RNO-199991	5	0.378263	0.841714	0.626667	0.946037	1.000
231	GOBP_DENDRITIC_SPINE_MORPHOGENESIS	GO:0060997	5	0.383785	0.840892	0.615830	0.943584	1.000
232	REACTOME_VESICLE_MEDIATED_TRANSPORT	R-RNO-5653656	5	0.378263	0.840365	0.605210	0.940561	1.000
233	REACTOME_CELLULAR_RESPONSES_TO_EXTERNAL_STIMULI	R-RNO-8953897	12	0.282566	0.837989	0.660952	0.941259	1.000
234	GOBP_REGULATION_OF_CELLULAR_LOCALIZATION	GO:0060341	17	0.253392	0.836788	0.683919	0.939613	1.000
235	GOBP_REGULATION_OF_CATION_CHANNEL_ACTIVITY	GO:2001257	14	0.271212	0.836063	0.687140	0.937045	1.000
236	GOBP_REGULATION_OF_TRANSFERASE_ACTIVITY	GO:0051338	22	0.238338	0.832550	0.675325	0.939704	1.000
237	GOBP_REGULATION_OF_SYNAPTIC_TRANSMISSION_GLUTA...	GO:0051966	12	0.277533	0.831553	0.662942	0.937683	1.000
238	GOBP_RESPONSE_TO_MECHANICAL_STIMULUS	GO:0009612	6	0.343800	0.830438	0.663620	0.935989	1.000
239	GOBP_REGULATION_OF_LIPID_BIOSYNTHETIC_PROCESS	GO:0046890	6	0.348824	0.828937	0.631179	0.934991	1.000
240	GOBP_EPHRIN_RECEPTOR_SIGNALING_PATHWAY	GO:0048013	5	0.373630	0.827878	0.667276	0.933158	1.000
241	GOBP_REGULATION_OF_LIPID_METABOLIC_PROCESS	GO:0019216	7	0.334597	0.825843	0.680970	0.933070	1.000
242	GOBP_REGULATION_OF_BINDING	GO:0051098	11	0.282300	0.825405	0.677485	0.930081	1.000
243	GOBP_DEVELOPMENTAL_MATURATION	GO:0021700	5	0.371699	0.824958	0.689788	0.927111	1.000
244	GOBP_CELLULAR_AMIDE_METABOLIC_PROCESS	GO:0043603	13	0.271327	0.822135	0.669903	0.928731	1.000
245	REACTOME_EXTRACELLULAR_MATRIX_ORGANIZATION	R-RNO-1474244	5	0.373111	0.813596	0.674374	0.940641	1.000
246	REACTOME_CREB1_PHOSPHORYLATION_THROUGH_NMDA_RE...	R-RNO-442742	8	0.317774	0.812689	0.706865	0.938528	1.000
247	GOBP_RESPONSE_TO_OXYGEN_LEVELS	GO:0070482	8	0.308629	0.810585	0.686992	0.938419	1.000
248	GOBP_CELLULAR_HOMEOSTASIS	GO:0019725	18	0.241028	0.807897	0.719536	0.939391	1.000
249	GOBP_LIPID_BIOSYNTHETIC_PROCESS	GO:0008610	6	0.348824	0.807835	0.699422	0.935790	1.000
250	HALLMARK_KRAS_SIGNALING_UP	M5953	6	0.353659	0.806476	0.695327	0.934730	1.000
251	REACTOME_CA_DEPENDENT_EVENTS	R-RNO-111996	8	0.312355	0.806235	0.706320	0.931387	1.000
252	GOBP_CELLULAR_GLUCOSE_HOMEOSTASIS	GO:0001678	5	0.367711	0.804152	0.706667	0.931676	1.000
253	GOBP_REGULATION_OF_TRANS_SYNAPTIC_SIGNALING	GO:0099177	40	0.206981	0.801417	0.777365	0.933142	1.000
254	GOBP_HOMEOSTATIC_PROCESS	GO:0042592	31	0.207828	0.794460	0.780669	0.942172	1.000
255	GOBP_SYNAPTIC_SIGNALING	GO:0099536	47	0.199763	0.792993	0.768482	0.941205	1.000

Continuation of Table **S12**

	NAME	ID	SIZE	ES	NES	NOM.p.val	FDR.q.val	FWER.p.val
256	GOBP_RESPONSE_TO_OXIDATIVE_STRESS	GO:0006979	8	0.293935	0.791877	0.707865	0.939705	1.000
257	REACTOME_DEVELOPMENTAL_BIOLOGY	R-RNO-1266738	23	0.213141	0.788358	0.751402	0.942282	1.000
258	GOBP_POSITIVE_REGULATION_OF_DEFENSE_RESPONSE	GO:0031349	5	0.354899	0.782766	0.714008	0.948455	1.000
259	GOBP_REGULATION_OF_LONG_TERM_SYNAPTIC_POTENTIA...	GO:1900271	6	0.322412	0.781691	0.721190	0.946756	1.000
260	GOBP_SYNAPTIC_TRANSMISSION_GLUTAMATERGIC	GO:0035249	13	0.251809	0.779644	0.729242	0.946475	1.000
261	GOBP_LEUKOCYTE_MIGRATION	GO:0050900	6	0.324711	0.775101	0.728846	0.950337	1.000
262	REACTOME_NERVOUS_SYSTEM_DEVELOPMENT	R-RNO-9675108	15	0.239258	0.773632	0.774067	0.949345	1.000
263	GOBP_POSITIVE_REGULATION_OF_CELL_GROWTH	GO:0030307	6	0.328895	0.772978	0.718310	0.946797	1.000
264	GOBP_CHROMOSOME_ORGANIZATION	GO:0051276	6	0.330331	0.771235	0.743396	0.946142	1.000
265	GOBP_REGULATION_OF_MUSCLE_CELL_DIFFERENTIATION	GO:0051147	6	0.327717	0.767821	0.738140	0.948031	1.000
266	GOBP_NEGATIVE_REGULATION_OF_SYNAPTIC_TRANSMISSION	GO:0050805	6	0.319437	0.767107	0.744639	0.945664	1.000
267	GOBP_TISSUE_HOMEOSTASIS	GO:0001894	5	0.346281	0.763209	0.765799	0.948525	1.000
268	GOBP_REGULATION_OF_SIGNALING_RECEPTOR_ACTIVITY	GO:0010469	14	0.243745	0.755460	0.788151	0.957130	1.000
269	GOBP_PHAGOCYTOSIS	GO:0006909	5	0.343375	0.754432	0.777164	0.955363	1.000
270	GOBP_REGULATION_OF_ENDOTHELIAL_CELL_MIGRATION	GO:0010594	5	0.337736	0.752240	0.741445	0.955273	1.000
271	GOBP_RESPONSE_TO_REACTIVE_OXYGEN_SPECIES	GO:0000302	8	0.293935	0.748928	0.793103	0.956977	1.000
272	REACTOME_G_ALPHA_Z_SIGNALLING_EVENTS	R-RNO-418597	5	0.330284	0.746144	0.779026	0.957829	1.000
273	GOBP_REGULATION_OF_ANATOMICAL_STRUCTURE_SIZE	GO:0090066	10	0.264047	0.742440	0.778672	0.960302	1.000
274	GOBP_RESPONSE_TO_HEAT	GO:0009408	7	0.295050	0.741020	0.749012	0.958969	1.000
275	GOBP_IMPORT_INTO_CELL	GO:0098657	6	0.307442	0.736290	0.788497	0.962602	1.000
276	GOBP_POSITIVE_REGULATION_OF_CATALYTIC_ACTIVITY	GO:0043085	30	0.197456	0.735547	0.841418	0.960231	1.000
277	GOBP_RESPONSE_TO_NICOTINE	GO:0035094	7	0.291804	0.733417	0.777778	0.959910	1.000
278	GOBP_PROTEIN_MODIFICATION_BY_SMALL_PROTEIN_CON...	GO:0070647	7	0.296117	0.731456	0.789077	0.959198	1.000
279	GOBP_REGULATION_OF_PROTEOLYSIS	GO:0030162	12	0.247674	0.731135	0.822857	0.956246	1.000
280	GOBP_POSITIVE_REGULATION_OF_MOLECULAR_FUNCTION	GO:0044093	38	0.185061	0.727425	0.870270	0.958267	1.000
281	GOBP_CARBOXYLIC_ACID_TRANSPORT	GO:0046942	6	0.301914	0.721422	0.813462	0.963427	1.000
282	GOBP_MACROMOLECULE_CATABOLIC_PROCESS	GO:0009057	12	0.242237	0.719259	0.828846	0.962974	1.000
283	GOBP_FATTY_ACID_TRANSPORT	GO:0015908	6	0.297320	0.715959	0.875000	0.964024	1.000
284	GOBP_AMINO_ACID_TRANSPORT	GO:0006865	6	0.301914	0.715152	0.808300	0.961654	1.000
285	REACTOME_CYTOKINE_SIGNALING_IN_IMMUNE_SYSTEM	R-RNO-1280215	21	0.208564	0.714243	0.853229	0.959638	1.000
286	GOBP_MEMORY	GO:0007613	13	0.233206	0.712980	0.862934	0.958073	1.000
287	GOBP_REGULATION_OF_HYDROLASE_ACTIVITY	GO:0051336	22	0.199388	0.703611	0.867993	0.967384	1.000
288	GOBP_SYNAPSE_ORGANIZATION	GO:0050808	17	0.211668	0.702442	0.838951	0.965781	1.000
289	GOBP_REGULATION_OF_EPITHELIAL_CELL_MIGRATION	GO:0010632	6	0.300762	0.700556	0.819066	0.964929	1.000
290	GOBP_ORGANIC_ANION_TRANSPORT	GO:0015711	6	0.301914	0.700302	0.843100	0.961976	1.000
291	REACTOME_INTERLEUKIN_17_SIGNALING	R-RNO-448424	6	0.295063	0.693681	0.854685	0.967384	1.000
292	GOBP_REGULATION_OF_CELL_SIZE	GO:0008361	5	0.313253	0.689142	0.839695	0.970118	1.000
293	REACTOME_L1CAM_INTERACTIONS	R-RNO-373760	5	0.309009	0.687405	0.833333	0.968941	1.000
294	GOBP_POSITIVE_REGULATION_OF_SECRETION	GO:0051047	6	0.290187	0.686810	0.834368	0.966388	1.000
295	HALLMARK_EPITHELIAL_MESENCHYMAL_TRANSITION	M5930	5	0.313253	0.686411	0.856631	0.963662	1.000
296	GOBP_SIGNAL_RELEASE	GO:0023061	14	0.218155	0.686170	0.861272	0.960739	1.000
297	GOBP_MULTICELLULAR_ORGANISMAL_HOMEOSTASIS	GO:0048871	12	0.231059	0.684004	0.859922	0.960114	1.000
298	REACTOME_INTRACELLULAR_SIGNALING_BY_SECOND_MES...	R-RNO-9006925	15	0.215209	0.680585	0.875000	0.960998	1.000

Continuation of Table **S12**

	NAME	ID	SIZE	ES	NES	NOM.p.val	FDR.q.val	FWER.p.val
299	GOBP_LONG_TERM_MEMORY	GO:0007616	6	0.293141	0.680347	0.856016	0.958036	1.000
300	GOBP_REGULATION_OF_GROWTH	GO:0040008	13	0.220873	0.679830	0.884990	0.955506	1.000
301	GOBP_REGULATION_OF_AXONOGENESIS	GO:0050770	7	0.275751	0.679046	0.850860	0.953338	1.000
302	GOBP_DEFENSE_RESPONSE_TO_OTHER_ORGANISM	GO:0098542	9	0.242620	0.669537	0.868668	0.961543	1.000
303	GOBP_PROTEIN_COMPLEX_OLIGOMERIZATION	GO:0051259	6	0.280488	0.660281	0.898467	0.968989	1.000
304	GOBP_PROTEIN_CATABOLIC_PROCESS	GO:0030163	8	0.252928	0.652824	0.863281	0.973925	1.000
305	GOBP_REGULATION_OF_CELLULAR_PROTEIN_LOCALIZATION	GO:1903827	12	0.216407	0.641644	0.899431	0.982249	1.000
306	GOBP_MULTICELLULAR_ORGANISM_REPRODUCTION	GO:0032504	12	0.215359	0.635015	0.915851	0.985680	1.000
307	GOBP_RESPONSE_TO_MONOSACCHARIDE	GO:0034284	5	0.285028	0.634057	0.889943	0.983380	1.000
308	GOBP_RESPONSE_TO_CARBOHYDRATE	GO:0009743	5	0.285028	0.633948	0.920849	0.980312	1.000
309	GOBP_POSITIVE_REGULATION_OF_SIGNALING	GO:0023056	34	0.162188	0.629597	0.933573	0.981168	1.000
310	GOBP_LIPID_EXPORT_FROM_CELL	GO:0140353	5	0.286001	0.629435	0.886100	0.978110	1.000
311	GOBP_RESPONSE_TO_RADIATION	GO:0009314	13	0.199795	0.614111	0.924797	0.988360	1.000
312	GOBP_NEGATIVE_REGULATION_OF_PROTEIN_CATABOLIC_...	GO:0042177	5	0.283785	0.608440	0.902622	0.990070	1.000
313	GOBP_DENDRITE_MORPHOGENESIS	GO:0048813	6	0.246336	0.594799	0.938462	0.996975	1.000
314	GOBP_LONG_TERM_SYNAPTIC_DEPRESSION	GO:0060292	5	0.277108	0.594548	0.953488	0.993990	1.000
315	GOBP_DEVELOPMENTAL_PROCESS_INVOLVED_IN_REPRODU...	GO:0003006	11	0.207086	0.585594	0.948718	0.997271	1.000
316	GOBP_LONG_TERM_SYNAPTIC_POTENTIATION	GO:0060291	12	0.185252	0.559760	0.959770	1.000000	1.000
317	GOBP_RESPIRATORY_SYSTEM_DEVELOPMENT	GO:0060541	6	0.233123	0.557378	0.968064	1.000000	1.000
318	GOBP_EPITHELIUM_DEVELOPMENT	GO:0060429	15	0.175139	0.555795	0.979284	1.000000	1.000
319	GOBP_RESPONSE_TO_METAL_ION	GO:0010038	15	0.176868	0.553491	0.966403	1.000000	1.000
320	GOBP_RESPONSE_TO_ALKALOID	GO:0043279	5	0.253012	0.552635	0.971429	1.000000	1.000
321	GOBP_NEGATIVE_REGULATION_OF_PROTEIN_METABOLIC_...	GO:0051248	20	0.154904	0.541200	0.975096	1.000000	1.000
322	GOBP_LOCOMOTORY_BEHAVIOR	GO:0007626	7	0.209876	0.534543	0.972167	1.000000	1.000
323	GOBP_REGULATION_OF_PROTEIN_TYROSINE_KINASE_ACT...	GO:0061097	5	0.226692	0.513598	0.990892	1.000000	1.000
324	GOBP_POSITIVE_REGULATION_OF_PROTEIN_TYROSINE_K...	GO:0061098	5	0.226692	0.494297	0.990272	1.000000	1.000
325	GOBP_CELLULAR_MACROMOLECULE_CATABOLIC_PROCESS	GO:0044265	6	0.205525	0.492125	0.988000	1.000000	1.000
326	GOBP_MONOCARBOXYLIC_ACID_TRANSPORT	GO:0015718	5	0.221969	0.490913	0.990138	1.000000	1.000
327	GOBP_DICARBOXYLIC_ACID_TRANSPORT	GO:0006835	5	0.221969	0.488723	0.983806	1.000000	1.000
328	GOBP_ACIDIC_AMINO_ACID_TRANSPORT	GO:0015800	5	0.221969	0.488624	0.990758	0.999477	1.000
329	GOBP_POSITIVE_REGULATION_OF_CATION_TRANSMEMBRA...	GO:1904064	5	0.216867	0.483912	0.984526	0.997421	1.000
330	GOBP_ENDOCRINE_SYSTEM_DEVELOPMENT	GO:0035270	6	0.195122	0.459736	0.998073	0.998173	1.000
331	GOBP_POSITIVE_REGULATION_OF_SYNAPTIC_TRANSMISSION	GO:0050806	15	0.123646	0.387066	1.000000	0.999577	1.000
332	GOBP_ASSOCIATIVE_LEARNING	GO:0008306	7	-0.172840	-0.437457	0.997925	0.997725	1.000
333	GOBP_LEARNING	GO:0007612	13	-0.160000	-0.509510	0.989407	0.988764	1.000
334	GOBP_DEVELOPMENTAL_CELL_GROWTH	GO:0048588	5	-0.231032	-0.511076	0.984704	0.990831	1.000
335	GOBP_SENSORY_PERCEPTION_OF_PAIN	GO:0019233	5	-0.228916	-0.511214	0.988327	0.993362	1.000
336	GOBP_POSITIVE_REGULATION_OF_AXONOGENESIS	GO:0050772	5	-0.228058	-0.515626	0.985972	0.994722	1.000
337	GOBP_INTRINSIC_APOPTOTIC_SIGNALING_PATHWAY	GO:0097193	5	-0.238854	-0.536096	0.979798	0.990148	1.000
338	GOBP_TELENCEPHALON_DEVELOPMENT	GO:0021537	6	-0.225076	-0.538504	0.968685	0.991723	1.000
339	GOBP_ORGANONITROGEN_COMPOUND_CATABOLIC_PROCESS	GO:1901565	9	-0.196260	-0.542240	0.976143	0.992776	1.000
340	GOBP_CELLULAR_RESPONSE_TO_CHEMICAL_STRESS	GO:0062197	7	-0.213626	-0.543730	0.966457	0.994763	1.000
341	GOBP_NEGATIVE_REGULATION_OF_PROTEIN_MODIFICATI...	GO:0031400	10	-0.192595	-0.548186	0.969290	0.995445	1.000

Continuation of Table **S12**

	NAME	ID	SIZE	ES	NES	NOM.p.val	FDR.q.val	FWER.p.val
342	GOBP_CELLULAR_RESPONSE_TO_REACTIVE_OXYGEN_SPECIES	GO:0034614	7	-0.213626	-0.556955	0.958606	0.993755	1.000
343	GOBP_NEGATIVE_REGULATION_OF_PHOSPHORUS_METABOL...	GO:0010563	9	-0.209941	-0.578375	0.962882	0.984608	1.000
344	GOBP_RESPONSE_TO_INSULIN	GO:0032868	5	-0.258670	-0.582876	0.963855	0.984424	1.000
345	GOBP_NEGATIVE_REGULATION_OF_PROTEOLYSIS	GO:0045861	8	-0.221846	-0.594173	0.954048	0.979692	1.000
346	GOBP_MUSCLE_CELL_DIFFERENTIATION	GO:0042692	10	-0.206411	-0.597244	0.928279	0.980259	1.000
347	REACTOME_G_ALPHA_Q_SIGNALLING_EVENTS	R-RNO-416476	6	-0.250671	-0.602232	0.943633	0.979159	1.000
348	GOBP_NEGATIVE_REGULATION_OF_SIGNALING	GO:0023057	21	-0.168192	-0.603420	0.951965	0.980816	1.000
349	GOBP_CELLULAR_RESPONSE_TO_INORGANIC_SUBSTANCE	GO:0071241	11	-0.207337	-0.608424	0.936403	0.979745	1.000
350	GOBP_REGULATION_OF_PROTEIN_CATABOLIC_PROCESS	GO:0042176	7	-0.234568	-0.611932	0.926829	0.979685	1.000
351	GOBP_POSITIVE_REGULATION_OF_NEURON_PROJECTION_...	GO:0010976	5	-0.277108	-0.612155	0.933472	0.982158	1.000
352	REACTOME_RNA_POLYMERASE_II_TRANSCRIPTION	R-RNO-73857	20	-0.174087	-0.612674	0.931818	0.984429	1.000
353	GOBP_RESPONSE_TO_STARVATION	GO:0042594	5	-0.279034	-0.620718	0.919840	0.980280	1.000
354	GOBP_ENDOMEMBRANE_SYSTEM_ORGANIZATION	GO:0010256	6	-0.264239	-0.623202	0.929936	0.980791	1.000
355	GOBP_NEGATIVE_REGULATION_OF_CATABOLIC_PROCESS	GO:0009895	8	-0.237500	-0.625109	0.880478	0.981720	1.000
356	GOBP_CELLULAR_RESPONSE_TO_STARVATION	GO:0009267	5	-0.279034	-0.626345	0.910256	0.983227	1.000
357	GOBP_ERBB_SIGNALING_PATHWAY	GO:0038127	5	-0.282080	-0.645640	0.904471	0.967977	1.000
358	GOBP_POSITIVE_REGULATION_OF_TRANSMEMBRANE_TRAN...	GO:0034764	7	-0.252738	-0.649519	0.909474	0.966782	1.000
359	GOBP_IN_UTERO_EMBRYONIC_DEVELOPMENT	GO:0001701	8	-0.245971	-0.650473	0.897796	0.968449	1.000
360	GOBP_POSITIVE_REGULATION_OF_BINDING	GO:0051099	6	-0.269324	-0.654575	0.884368	0.966870	1.000
361	GOBP_AGING	GO:0007568	13	-0.208250	-0.654924	0.887202	0.969244	1.000
362	GOBP_MATERNAL_PROCESS_INVOLVED_IN_FEMALE_PREGN...	GO:0060135	5	-0.299468	-0.658233	0.888000	0.968572	1.000
363	GOBP_REPRODUCTION	GO:0000003	18	-0.192079	-0.660973	0.902954	0.968215	1.000
364	REACTOME_SENESCENCE_ASSOCIATED_SECRETORY_PHENO...	R-RNO-2559582	6	-0.285057	-0.672682	0.854911	0.957863	1.000
365	REACTOME_CELLULAR_SENESCENCE	R-RNO-2559583	6	-0.285057	-0.679519	0.858012	0.952438	1.000
366	GOBP_NEGATIVE_REGULATION_OF_CYSTEINE_TYPE_ENDO...	GO:2000117	5	-0.300394	-0.682399	0.884536	0.951614	1.000
367	GOBP_NERVOUS_SYSTEM_PROCESS	GO:0050877	38	-0.168442	-0.687657	0.900222	0.947851	1.000
368	GOBP_REGULATION_OF_BODY_FLUID_LEVELS	GO:0050878	11	-0.232167	-0.692414	0.855422	0.944853	1.000
369	GOBP_REPRODUCTIVE_SYSTEM_DEVELOPMENT	GO:0061458	6	-0.297362	-0.702570	0.836401	0.934618	1.000
370	GOBP_NEGATIVE_REGULATION_OF_PEPTIDASE_ACTIVITY	GO:0010466	6	-0.294806	-0.702609	0.845188	0.937247	1.000
371	GOBP_POSITIVE_REGULATION_OF_TRANSPORT	GO:0051050	26	-0.189778	-0.705069	0.877419	0.936640	1.000
372	GOBP_REGULATION_OF_RECEPTOR_MEDIATED_ENDOCYTOSIS	GO:0048259	5	-0.313253	-0.706368	0.845098	0.937608	1.000
373	GOBP_POSITIVE_REGULATION_OF_PROTEIN_METABOLIC_...	GO:0051247	28	-0.188501	-0.706767	0.856566	0.939750	1.000
374	GOBP_MULTI_ORGANISM_PROCESS	GO:0051704	16	-0.212949	-0.709658	0.810526	0.938615	1.000
375	GOBP_RECEPTOR_INTERNALIZATION	GO:0031623	5	-0.313253	-0.710046	0.816284	0.940840	1.000
376	GOBP_CELL_JUNCTION_ORGANIZATION	GO:0034330	19	-0.202071	-0.711488	0.853763	0.941722	1.000
377	GOBP_RAS_PROTEIN_SIGNAL_TRANSDUCTION	GO:0007265	5	-0.321363	-0.711882	0.820866	0.943860	1.000
378	GOBP_NEGATIVE_REGULATION_OF_TRANSCRIPTION_BY_R...	GO:0000122	10	-0.245314	-0.711927	0.841365	0.946566	1.000
379	GOBP_SECRETION	GO:0046903	24	-0.193467	-0.717904	0.871397	0.941127	1.000
380	GOBP_RECEPTOR_METABOLIC_PROCESS	GO:0043112	5	-0.313253	-0.720599	0.817814	0.940374	1.000
381	GOBP_REGULATION_OF_PHOSPHORUS_METABOLIC_PROCESS	GO:0051174	27	-0.189590	-0.721419	0.852083	0.941932	1.000
382	GOBP_RESPONSE_TO_INTERLEUKIN_1	GO:0070555	5	-0.324362	-0.722393	0.820202	0.943300	1.000
383	GOBP_REGULATION_OF_PROTEIN_MODIFICATION_PROCESS	GO:0031399	29	-0.184584	-0.723281	0.838565	0.944847	1.000
384	REACTOME_TRANSCRIPTIONAL_REGULATION_OF_WHITE_A...	R-RNO-381340	6	-0.301403	-0.724765	0.819473	0.945415	1.000

Continuation of Table **S12**

	NAME	ID	SIZE	ES	NES	NOM.p.val	FDR.q.val	FWER.p.val
385	GOBP_NEGATIVE_REGULATION_OF_ANION_TRANSPORT	GO:1903792	8	-0.276102	-0.734156	0.797040	0.934775	1.000
386	GOBP_DNA_METABOLIC_PROCESS	GO:0006259	6	-0.301284	-0.734569	0.831622	0.936849	1.000
387	GOBP_CYTOKINE_MEDIATED_SIGNALING_PATHWAY	GO:0019221	16	-0.229538	-0.742451	0.807440	0.927766	1.000
388	GOBP_POSITIVE_REGULATION_OF_ION_TRANSPORT	GO:0043270	17	-0.216652	-0.742717	0.801282	0.930198	1.000
389	GOBP_POSITIVE_REGULATION_OF_TRANSFERASE_ACTIVITY	GO:0051347	20	-0.208386	-0.743377	0.829374	0.931964	1.000
390	GOBP_POSITIVE_REGULATION_OF_PROTEIN_KINASE_ACT...	GO:0045860	19	-0.216495	-0.744362	0.812641	0.933228	1.000
391	GOBP_PROTEIN_MODIFICATION_BY_SMALL_PROTEIN_CON...	GO:0032446	6	-0.308346	-0.750415	0.746362	0.926497	1.000
392	GOBP_REGULATION_OF_PROTEIN_PHOSPHORYLATION	GO:0001932	26	-0.198132	-0.751383	0.806981	0.927796	1.000
393	GOBP_REGULATION_OF_CATABOLIC_PROCESS	GO:0009894	13	-0.236574	-0.754200	0.790456	0.926377	1.000
394	GOBP_REGULATION_OF_SYNAPSE_STRUCTURE_OR_ACTIVITY	GO:0050803	11	-0.258287	-0.755567	0.781457	0.927117	1.000
395	GOBP_RECEPTOR_MEDIATED_ENDOCYTOSIS	GO:0006898	6	-0.317073	-0.757030	0.755144	0.927644	1.000
396	GOBP_EPITHELIAL_CELL_DIFFERENTIATION	GO:0030855	7	-0.293164	-0.757752	0.746753	0.929292	1.000
397	GOBP_TRANSMEMBRANE_RECEPTOR_PROTEIN_TYROSINE_K...	GO:0007169	19	-0.220571	-0.767063	0.804721	0.917088	1.000
398	GOBP_SENSORY_ORGAN_DEVELOPMENT	GO:0007423	8	-0.285768	-0.772060	0.762575	0.911530	1.000
399	GOBP_POSITIVE_REGULATION_OF_GROWTH	GO:0045927	9	-0.285464	-0.780914	0.758763	0.899665	1.000
400	GOBP_STRIATED_MUSCLE_CELL_DIFFERENTIATION	GO:0051146	9	-0.283263	-0.781312	0.730435	0.901885	1.000
401	GOBP_REGULATION_OF_APOPTOTIC_SIGNALING_PATHWAY	GO:2001233	10	-0.270555	-0.785243	0.726115	0.898120	1.000
402	GOBP_REGULATION_OF_HORMONE_LEVELS	GO:0010817	12	-0.259475	-0.786651	0.750552	0.898658	1.000
403	GOBP_FC_EPSILON_RECEPTOR_SIGNALING_PATHWAY	GO:0038095	7	-0.308626	-0.787454	0.734440	0.900117	1.000
404	GOBP_ACTIVATION_OF_PROTEIN_KINASE_ACTIVITY	GO:0032147	11	-0.268280	-0.788453	0.725051	0.901213	1.000
405	REACTOME_FC_EPSILON_RECEPTOR_FCERI_SIGNALING	R-RNO-2454202	7	-0.308626	-0.793560	0.700405	0.895308	1.000
406	GOBP_GLUCOSE_METABOLIC_PROCESS	GO:0006006	6	-0.335856	-0.795741	0.710145	0.894369	1.000
407	GOBP_GROWTH	GO:0040007	14	-0.246004	-0.797152	0.737705	0.894606	1.000
408	GOBP_MONOSACCHARIDE_METABOLIC_PROCESS	GO:0005996	6	-0.335856	-0.799464	0.696703	0.893437	1.000
409	GOBP_ANATOMICAL_STRUCTURE_HOMEOSTASIS	GO:0060249	7	-0.314748	-0.805320	0.706114	0.886145	1.000
410	GOBP_CELL_CELL_JUNCTION_ASSEMBLY	GO:0007043	5	-0.358965	-0.816731	0.688985	0.869493	1.000
411	GOBP_RESPONSE_TO_CYTOKINE	GO:0034097	23	-0.225677	-0.817631	0.723077	0.870743	1.000
412	GOBP_CYTOKINE_PRODUCTION	GO:0001816	10	-0.291006	-0.821314	0.683406	0.867254	1.000
413	GOBP_RESPONSE_TO_CALCIUM_ION	GO:0051592	6	-0.345653	-0.821789	0.671610	0.869237	1.000
414	GOBP_PROTEIN_PHOSPHORYLATION	GO:0006468	34	-0.205306	-0.823090	0.728051	0.869606	1.000
415	GOBP_EMBRYO_DEVELOPMENT_ENDING_IN_BIRTH_OR_EGG...	GO:0009792	9	-0.304509	-0.827766	0.654167	0.864222	1.000
416	GOBP_PROTEIN_LOCALIZATION_TO_ORGANELLE	GO:0033365	9	-0.300453	-0.828968	0.678005	0.864973	1.000
417	GOBP_ANION_TRANSMEMBRANE_TRANSPORT	GO:0098656	7	-0.324898	-0.829963	0.677485	0.866037	1.000
418	REACTOME_SIGNALING_BY_INTERLEUKINS	R-RNO-449147	15	-0.264002	-0.831652	0.687631	0.865939	1.000
419	GOBP_NEGATIVE_REGULATION_OF_RESPONSE_TO_STIMULUS	GO:0048585	16	-0.255234	-0.833663	0.681917	0.865198	1.000
420	GOBP_INFLAMMATORY_RESPONSE	GO:0006954	12	-0.270697	-0.835236	0.665975	0.865216	1.000
421	GOBP_REGULATION_OF_PROTEIN_SERINE_THREONINE_KI...	GO:0071900	12	-0.270946	-0.836699	0.658281	0.865487	1.000
422	GOBP_RESPONSE_TO_NUTRIENT	GO:0007584	5	-0.369227	-0.839967	0.615385	0.862237	1.000
423	GOBP_POSITIVE_REGULATION_OF_CYTOKINE_PRODUCTION	GO:0001819	7	-0.333789	-0.843292	0.630522	0.858849	1.000
424	GOBP_CELLULAR_RESPONSE_TO_DNA_DAMAGE_STIMULUS	GO:0006974	5	-0.381317	-0.844896	0.634888	0.858719	1.000
425	GOBP_REGULATION_OF_TRANSMEMBRANE_TRANSPORT	GO:0034762	23	-0.229275	-0.848327	0.688000	0.855293	1.000
426	GOBP_CIRCULATORY_SYSTEM_PROCESS	GO:0003013	5	-0.378825	-0.856179	0.640177	0.843392	1.000
427	REACTOME_EXTRA_NUCLEAR_ESTROGEN_SIGNALING	R-RNO-9009391	7	-0.327997	-0.856661	0.637500	0.845514	1.000

Continuation of Table **S12**

	NAME	ID	SIZE	ES	NES	NOM.p.val	FDR.q.val	FWER.p.val
428	GOBP_RESPONSE_TO_EXTRACELLULAR_STIMULUS	GO:0009991	13	-0.276838	-0.858729	0.657143	0.844451	1.000
429	GOBP_MITOCHONDRION_ORGANIZATION	GO:0007005	7	-0.341089	-0.861891	0.590717	0.841679	1.000
430	GOBP_HEAD_DEVELOPMENT	GO:0060322	22	-0.240355	-0.864015	0.665948	0.840813	1.000
431	GOBP_REGULATION_OF_NEURON_DIFFERENTIATION	GO:0045664	5	-0.388307	-0.872358	0.587992	0.828038	1.000
432	REACTOME_ESR_MEDIATED_SIGNALING	R-RNO-8939211	8	-0.338501	-0.872965	0.610638	0.829632	1.000
433	GOBP_REGULATION_OF_PHOSPHOLIPASE_ACTIVITY	GO:0010517	6	-0.366609	-0.875161	0.589011	0.828298	1.000
434	GOBP_TRANSMEMBRANE_RECEPTOR_PROTEIN_SERINE_THR...	GO:0007178	6	-0.366676	-0.880524	0.573171	0.821016	1.000
435	GOBP_REGULATION_OF_ANION_TRANSPORT	GO:0044070	20	-0.250368	-0.881046	0.614583	0.822922	1.000
436	GOBP_NEUROINFLAMMATORY_RESPONSE	GO:0150076	5	-0.406765	-0.885663	0.592593	0.817032	1.000
437	GOBP_BEHAVIOR	GO:0007610	32	-0.228647	-0.888361	0.649462	0.814760	1.000
438	GOBP_NEGATIVE_REGULATION_OF_BIOSYNTHETIC_PROCESS	GO:0009890	13	-0.288653	-0.889027	0.616667	0.816342	1.000
439	GOBP_REGULATION_OF_RESPONSE_TO_STRESS	GO:0080134	19	-0.254154	-0.889199	0.621444	0.818915	1.000
440	REACTOME_PTEN_REGULATION	R-RNO-6807070	5	-0.394737	-0.891453	0.573441	0.817562	1.000
441	GOBP_REGULATION_OF_LIPASE_ACTIVITY	GO:0060191	6	-0.366609	-0.892741	0.602851	0.818033	1.000
442	GOBP_EXTRINSIC_APOPTOTIC_SIGNALING_PATHWAY	GO:0097191	7	-0.349639	-0.896040	0.576763	0.814410	1.000
443	GOBP_POSITIVE_REGULATION_OF_PHOSPHORUS_METABOL...	GO:0010562	24	-0.245896	-0.903667	0.599129	0.802835	1.000
444	GOBP_EMBRYO_DEVELOPMENT	GO:0009790	14	-0.282021	-0.904685	0.588595	0.803546	1.000
445	REACTOME_SIGNALING_BY_NUCLEAR_RECEPTORS	R-RNO-9006931	8	-0.338501	-0.904953	0.548902	0.806065	1.000
446	GOBP_NEGATIVE_REGULATION_OF_NEURON_DEATH	GO:1901215	11	-0.311375	-0.909173	0.559748	0.800613	1.000
447	GOBP_NEURON_PROJECTION_GUIDANCE	GO:0097485	9	-0.330302	-0.910156	0.561122	0.801634	1.000
448	GOBP_REGULATION_OF_INFLAMMATORY_RESPONSE	GO:0050727	7	-0.363485	-0.913370	0.532328	0.798312	1.000
449	GOBP_REGULATION_OF_INTRACELLULAR_SIGNAL_TRANSD...	GO:1902531	21	-0.254182	-0.914834	0.591880	0.798571	1.000
450	GOBP_REGULATION_OF_DEVELOPMENTAL_GROWTH	GO:0048638	9	-0.333629	-0.919956	0.552521	0.791288	1.000
451	GOBP_REGULATION_OF_CELLULAR_CATABOLIC_PROCESS	GO:0031329	10	-0.323984	-0.920713	0.537500	0.792908	1.000
452	GOBP_RESPONSE_TO_TEMPERATURE_STIMULUS	GO:0009266	9	-0.329560	-0.922647	0.560729	0.791843	1.000
453	GOBP_SECOND_MESSENGER_MEDIATED_SIGNALING	GO:0019932	16	-0.282695	-0.923255	0.570281	0.793607	1.000
454	GOBP_NEGATIVE_REGULATION_OF_APOPTOTIC_SIGNALIN...	GO:2001234	7	-0.355149	-0.923526	0.544330	0.796167	1.000
455	GOBP_RESPONSE_TO_BIOTIC_STIMULUS	GO:0009607	17	-0.269934	-0.923730	0.553015	0.798786	1.000
456	GOBP_CELLULAR_RESPONSE_TO_EXTERNAL_STIMULUS	GO:0071496	10	-0.321061	-0.926882	0.527668	0.795473	1.000
457	GOBP_POSITIVE_REGULATION_OF_PROTEIN_PHOSPHORYL...	GO:0001934	23	-0.254269	-0.929932	0.547672	0.792449	1.000
458	GOBP_POSITIVE_REGULATION_OF_ENDOTHELIAL_CELL_P...	GO:0001938	6	-0.392052	-0.932804	0.536481	0.789779	1.000
459	GOBP_POSITIVE_REGULATION_OF_RESPONSE_TO_EXTERN...	GO:0032103	9	-0.340538	-0.934166	0.517103	0.790093	1.000
460	GOBP_REGULATION_OF_DEFENSE_RESPONSE	GO:0031347	7	-0.363485	-0.934217	0.518518	0.793053	1.000
461	GOBP_POSITIVE_REGULATION_OF_PROTEIN_MODIFICATI...	GO:0031401	23	-0.254269	-0.935823	0.545249	0.793057	1.000
462	GOBP_REGULATION_OF_SECRETION	GO:0051046	14	-0.290654	-0.937945	0.515789	0.792118	1.000
463	REACTOME_ESTROGEN_DEPENDENT_NUCLEAR_EVENTS_DOW...	R-RNO-9634638	5	-0.422580	-0.940690	0.501976	0.789525	1.000
464	GOBP_PROTEIN_AUTOPHOSPHORYLATION	GO:0046777	5	-0.427721	-0.942062	0.516807	0.789606	1.000
465	GOBP_POSITIVE_REGULATION_OF_ANION_TRANSPORT	GO:1903793	9	-0.339402	-0.943960	0.506383	0.788921	1.000
466	GOBP_REGULATION_OF_ANION_TRANSMEMBRANE_TRANSPORT	GO:1903959	5	-0.433974	-0.944357	0.494759	0.791342	1.000
467	GOBP_NEGATIVE_REGULATION_OF_IMMUNE_SYSTEM_PROCESS	GO:0002683	5	-0.416878	-0.944591	0.510000	0.794030	1.000
468	GOBP_MULTICELLULAR_ORGANISMAL_RESPONSE_TO_STRESS	GO:0033555	5	-0.418725	-0.948150	0.490196	0.790156	1.000
469	GOBP_MULTI_MULTICELLULAR_ORGANISM_PROCESS	GO:0044706	8	-0.356735	-0.950113	0.519669	0.789266	1.000
470	REACTOME_SIGNALING_BY_NTRK2_TRKB	R-RNO-9006115	6	-0.397650	-0.955251	0.480851	0.781583	1.000

Continuation of Table **S12**

	NAME	ID	SIZE	ES	NES	NOM.p.val	FDR.q.val	FWER.p.val
471	GOBP_RESPONSE_TO_BACTERIUM	GO:0009617	13	-0.305742	-0.956488	0.468619	0.782260	1.000
472	GOBP_TUBE_DEVELOPMENT	GO:0035295	18	-0.288885	-0.957790	0.537209	0.782733	1.000
473	GOBP_APOPTOTIC_SIGNALING_PATHWAY	GO:0097190	15	-0.295560	-0.961525	0.480885	0.778411	1.000
474	GOBP_REGULATION_OF_NERVOUS_SYSTEM_PROCESS	GO:0031644	8	-0.362500	-0.964128	0.515030	0.776098	1.000
475	GOBP_RESPONSE_TO_MOLECULE_OF_BACTERIAL_ORIGIN	GO:0002237	13	-0.305742	-0.964169	0.492569	0.779182	1.000
476	GOBP_DEPHOSPHORYLATION	GO:0016311	7	-0.386051	-0.965168	0.494071	0.780208	1.000
477	GOBP_NEGATIVE_REGULATION_OF_GENE_EXPRESSION	GO:0010629	12	-0.318014	-0.965273	0.491262	0.783182	1.000
478	GOBP_RESPONSE_TO_NITROGEN_COMPOUND	GO:1901698	34	-0.238680	-0.965556	0.515528	0.785829	1.000
479	GOBP_NEGATIVE_REGULATION_OF_TRANSPORT	GO:0051051	13	-0.305741	-0.966728	0.508097	0.786711	1.000
480	GOBP_PROTEIN_DEPHOSPHORYLATION	GO:0006470	7	-0.386051	-0.974787	0.483193	0.772767	1.000
481	GOBP_CALCIUM_MEDIATED_SIGNALING	GO:0019722	10	-0.339575	-0.975222	0.461009	0.775091	1.000
482	GOBP_POSITIVE_REGULATION_OF_SMALL_MOLECULE_MET...	GO:0062013	5	-0.442234	-0.979207	0.460922	0.769641	1.000
483	GOBP_REGULATION_OF_CELLULAR_AMIDE_METABOLIC_PR...	GO:0034248	9	-0.349749	-0.982362	0.482546	0.766080	1.000
484	GOBP_PEPTIDYL_LYSINE_MODIFICATION	GO:0018205	5	-0.437258	-0.984175	0.457023	0.765644	1.000
485	GOBP_NEURON_MIGRATION	GO:0001764	5	-0.446074	-0.984552	0.458599	0.768061	1.000
486	REACTOME_SIGNALING_BY_RHO_GTPASES_MIRO_GTPASES...	R-RNO-9716542	9	-0.361939	-0.984646	0.462185	0.771139	1.000
487	GOBP_POSITIVE_REGULATION_OF_NERVOUS_SYSTEM_DEV...	GO:0051962	12	-0.327832	-0.985401	0.471338	0.772930	1.000
488	GOBP_REGULATION_OF_CELL_CYCLE	GO:0051726	13	-0.314983	-0.990465	0.472917	0.765862	1.000
489	GOBP_NERVE_DEVELOPMENT	GO:0021675	9	-0.357722	-0.990604	0.469828	0.768935	1.000
490	GOBP_MUSCLE_CONTRACTION	GO:0006936	5	-0.443507	-0.990908	0.432203	0.771607	1.000
491	GOBP_CENTRAL_NERVOUS_SYSTEM_DEVELOPMENT	GO:0007417	26	-0.262922	-0.995004	0.452915	0.766192	1.000
492	REACTOME_RHO_GTPASE_EFFECTORS	R-RNO-195258	9	-0.361939	-0.995536	0.466258	0.768398	1.000
493	GOBP_FOREBRAIN_DEVELOPMENT	GO:0030900	9	-0.360003	-0.995656	0.442797	0.771578	1.000
494	GOBP_NEGATIVE_REGULATION_OF_ION_TRANSPORT	GO:0043271	11	-0.332248	-1.003890	0.448578	0.757417	1.000
495	GOBP_APOPTOTIC_MITOCHONDRIAL_CHANGES	GO:0008637	6	-0.418457	-1.004043	0.448413	0.760487	1.000
496	GOBP_POSITIVE_REGULATION_OF_CELL_DEVELOPMENT	GO:0010720	12	-0.327832	-1.004247	0.427686	0.763535	1.000
497	GOBP_POSITIVE_REGULATION_OF_NEUROGENESIS	GO:0050769	12	-0.327832	-1.008814	0.463675	0.757201	1.000
498	GOBP_REGULATION_OF_FAT_CELL_DIFFERENTIATION	GO:0045598	6	-0.422965	-1.009266	0.433684	0.759644	1.000
499	GOBP_GLAND_DEVELOPMENT	GO:0048732	7	-0.410313	-1.013658	0.441118	0.753776	1.000
500	GOBP_REGULATION_OF_PROTEIN_LOCALIZATION	GO:0032880	18	-0.294564	-1.015817	0.434599	0.752548	1.000
501	GOBP_TUBE_MORPHOGENESIS	GO:0035239	15	-0.319601	-1.018500	0.417391	0.750149	1.000
502	GOBP_POSTTRANSCRIPTIONAL_REGULATION_OF_GENE_EX...	GO:0010608	8	-0.377077	-1.023671	0.431250	0.742483	1.000
503	GOBP_REGULATION_OF_PHOSPHOLIPASE_C_ACTIVITY	GO:1900274	5	-0.469837	-1.024230	0.405010	0.744666	1.000
504	GOBP_NEGATIVE_REGULATION_OF_CELL_PROJECTION_OR...	GO:0031345	5	-0.459349	-1.027290	0.415800	0.741512	1.000
505	GOBP_ENZYME_LINKED_RECEPTOR_PROTEIN_SIGNALING_...	GO:0007167	24	-0.282056	-1.033670	0.405738	0.731122	1.000
506	GOBP_POSITIVE_REGULATION_OF_LIPASE_ACTIVITY	GO:0060193	5	-0.469837	-1.034701	0.418327	0.732352	1.000
507	GOBP_GLIOGENESIS	GO:0042063	12	-0.344391	-1.038106	0.404711	0.728380	1.000
508	GOBP_REGULATION_OF_ENDOCYTOSIS	GO:0030100	6	-0.432507	-1.042375	0.390756	0.722614	1.000
509	GOBP_CELL_JUNCTION_ASSEMBLY	GO:0034329	8	-0.393436	-1.045495	0.382883	0.719494	1.000
510	GOBP_RHYTHMIC_PROCESS	GO:0048511	15	-0.325814	-1.051108	0.371795	0.710839	1.000
511	GOBP_REGULATION_OF_EXTRINSIC_APOPTOTIC_SIGNALI...	GO:2001236	6	-0.440920	-1.060859	0.367220	0.694258	1.000
512	GOBP_AMEBOIDAL_TYPE_CELL_MIGRATION	GO:0001667	11	-0.358920	-1.070693	0.332645	0.677125	1.000
513	GOBP_CELL_CELL_ADHESION	GO:0098609	14	-0.333064	-1.072490	0.352818	0.676714	1.000

Continuation of Table **S12**

	NAME	ID	SIZE	ES	NES	NOM.p.val	FDR.q.val	FWER.p.val
514	REACTOME_HEMOSTASIS	R-RNO-109582	14	-0.340238	-1.072648	0.341253	0.679734	1.000
515	GOBP_CELLULAR_RESPONSE_TO_CADMIUM_ION	GO:0071276	5	-0.476646	-1.073487	0.341948	0.681347	1.000
516	GOBP_COGNITION	GO:0050890	28	-0.277684	-1.074058	0.369803	0.683485	1.000
517	GOBP_RESPONSE_TO_NERVE_GROWTH_FACTOR	GO:1990089	7	-0.416604	-1.074307	0.351410	0.686446	1.000
518	GOBP_POSITIVE_REGULATION_OF_DEVELOPMENTAL_GROWTH	GO:0048639	8	-0.405376	-1.075322	0.349398	0.687802	1.000
519	REACTOME_CELL_CYCLE_MITOTIC	R-RNO-69278	5	-0.481637	-1.080460	0.354077	0.680278	1.000
520	HALLMARK_ALLOGRAFT_REJECTION	M5950	7	-0.420106	-1.081981	0.354701	0.680350	1.000
521	GOBP_BIOLOGICAL_ADHESION	GO:0022610	19	-0.308902	-1.082776	0.334719	0.682193	1.000
522	GOBP_MAPK_CASCADE	GO:0000165	18	-0.319594	-1.097376	0.336000	0.654962	1.000
523	GOBP_NEUROTROPHIN_TRK_RECEPTOR_SIGNALING_PATHWAY	GO:0048011	6	-0.469326	-1.098035	0.348837	0.656868	1.000
524	GOBP_CELL_CYCLE	GO:0007049	21	-0.313180	-1.101047	0.334021	0.653889	1.000
525	GOBP_RESPONSE_TO_CADMIUM_ION	GO:0046686	6	-0.451277	-1.102935	0.324544	0.653196	1.000
526	GOBP_NEUROTROPHIN_SIGNALING_PATHWAY	GO:0038179	6	-0.469326	-1.105882	0.319920	0.650732	1.000
527	GOBP_INTRACELLULAR_PROTEIN_TRANSPORT	GO:0006886	9	-0.401980	-1.106531	0.304979	0.652766	1.000
528	GOBP_INTRACELLULAR_TRANSPORT	GO:0046907	11	-0.370596	-1.109246	0.314465	0.650503	1.000
529	GOBP_POSITIVE_REGULATION_OF_IMMUNE_SYSTEM_PROCESS	GO:0002684	18	-0.319970	-1.114180	0.329140	0.643943	1.000
530	GOBP_SMALL_GTPASE_MEDIATED_SIGNAL_TRANSDUCTION	GO:0007264	8	-0.419915	-1.117525	0.311850	0.640153	1.000
531	GOBP_REGULATION_OF_VESICLE_MEDIATED_TRANSPORT	GO:0060627	12	-0.368537	-1.120440	0.313853	0.637676	1.000
532	GOBP_GLIAL_CELL_DIFFERENTIATION	GO:0010001	10	-0.384941	-1.123095	0.295359	0.635543	1.000
533	GOBP_POSITIVE_REGULATION_OF_GENE_EXPRESSION	GO:0010628	16	-0.335354	-1.127036	0.304255	0.630940	1.000
534	GOBP_DEVELOPMENTAL_GROWTH	GO:0048589	10	-0.393514	-1.128503	0.308642	0.631287	1.000
535	GOBP_APOPTOTIC_PROCESS	GO:0006915	35	-0.283163	-1.133320	0.282328	0.624915	1.000
536	GOBP_NEGATIVE_REGULATION_OF_DEVELOPMENTAL_PROCESS	GO:0051093	13	-0.368151	-1.143135	0.268344	0.607482	1.000
537	GOBP_TISSUE_MIGRATION	GO:0090130	9	-0.419638	-1.145282	0.296460	0.606683	1.000
538	GOBP_REGULATION_OF_PROTEIN_STABILITY	GO:0031647	5	-0.498665	-1.148561	0.276210	0.603212	1.000
539	GOBP_POSITIVE_REGULATION_OF_PROTEIN_SERINE_THR...	GO:0071902	9	-0.408232	-1.152179	0.273834	0.599364	1.000
540	GOBP_NEGATIVE_REGULATION_OF_NEURON_APOPTOTIC_P...	GO:0043524	8	-0.447016	-1.153905	0.275154	0.598805	1.000
541	GOBP_COAGULATION	GO:0050817	8	-0.455158	-1.160078	0.265351	0.590287	1.000
542	GOBP_CYTOSKELETON_ORGANIZATION	GO:0007010	11	-0.396997	-1.160675	0.274059	0.592441	1.000
543	GOBP_NEGATIVE_REGULATION_OF_CELL_DIFFERENTIATION	GO:0045596	13	-0.368151	-1.161903	0.252796	0.593427	1.000
544	REACTOME_SIGNALING_BY_WNT	R-RNO-195721	7	-0.455899	-1.167902	0.259958	0.585046	1.000
545	GOBP_NEGATIVE_REGULATION_OF_CELLULAR_COMPONENT...	GO:0051129	11	-0.392823	-1.168719	0.260128	0.586714	1.000
546	GOBP_REGULATION_OF_NERVOUS_SYSTEM_DEVELOPMENT	GO:0051960	15	-0.360378	-1.171842	0.262821	0.583974	1.000
547	REACTOME_TRANSCRIPTIONAL_REGULATION_BY_TP53	R-RNO-3700989	6	-0.496894	-1.174781	0.279116	0.581606	1.000
548	GOBP_ENDOCYTOSIS	GO:0006897	8	-0.445022	-1.175419	0.269311	0.583694	1.000
549	GOBP_SPROUTING_ANGIOGENESIS	GO:0002040	5	-0.533935	-1.176640	0.281720	0.584706	1.000
550	GOBP_POSITIVE_REGULATION_OF_EPITHELIAL_CELL_PR...	GO:0050679	7	-0.474771	-1.178516	0.267241	0.584150	1.000
551	GOBP_BLOOD_VESSEL_MORPHOGENESIS	GO:0048514	14	-0.370220	-1.180105	0.235294	0.584479	1.000
552	GOBP_FAT_CELL_DIFFERENTIATION	GO:0045444	9	-0.427304	-1.180434	0.239837	0.587250	1.000
553	GOBP_NEGATIVE_REGULATION_OF_TRANSMEMBRANE_TRAN...	GO:0034763	6	-0.491642	-1.183266	0.251037	0.584939	1.000
554	GOBP_ORGAN_GROWTH	GO:0035265	5	-0.546573	-1.188716	0.272366	0.577403	1.000
555	GOBP_PROTEIN_KINASE_B_SIGNALING	GO:0043491	6	-0.495492	-1.191238	0.254620	0.576117	1.000
556	GOBP_REGULATION_OF_LYMPHOCYTE_ACTIVATION	GO:0051249	5	-0.544297	-1.197027	0.247917	0.568054	1.000

Continuation of Table **S12**

	NAME	ID	SIZE	ES	NES	NOM.p.val	FDR.q.val	FWER.p.val
557	GOBP_PLATELET_ACTIVATION	GO:0030168	6	-0.504833	-1.197210	0.245726	0.571165	1.000
558	GOBP_INSULIN_SECRETION	GO:0030073	7	-0.475066	-1.199055	0.235294	0.571310	1.000
559	GOBP_NEGATIVE_REGULATION_OF_NUCLEOBASE_CONTAIN...	GO:0045934	13	-0.382797	-1.202841	0.210526	0.566992	1.000
560	GOBP_EMBRYONIC_MORPHOGENESIS	GO:0048598	6	-0.491263	-1.207787	0.239754	0.560649	1.000
561	GOBP_HORMONE_TRANSPORT	GO:0009914	9	-0.424347	-1.210269	0.217822	0.559266	1.000
562	GOBP_BLOOD_VESSEL_ENDOTHELIAL_CELL_MIGRATION	GO:0043534	8	-0.453042	-1.213456	0.245690	0.556420	1.000
563	GOBP_REGULATION_OF_PEPTIDYL_TYROSINE_PHOSPHORY...	GO:0050730	9	-0.439054	-1.215781	0.239175	0.555681	1.000
564	GOBP_PEPTIDYL_TYROSINE_MODIFICATION	GO:0018212	10	-0.419442	-1.216641	0.204124	0.557402	1.000
565	GOBP_ENDOTHELIAL_CELL_MIGRATION	GO:0043542	8	-0.453042	-1.217064	0.220044	0.560197	1.000
566	GOBP_REGULATION_OF_MITOTIC_CELL_CYCLE	GO:0007346	5	-0.539917	-1.218885	0.237903	0.559966	1.000
567	GOBP_INTRACELLULAR_RECEPTOR_SIGNALING_PATHWAY	GO:0030522	5	-0.555262	-1.222920	0.239919	0.555663	1.000
568	GOBP_EMBRYONIC_ORGAN_DEVELOPMENT	GO:0048568	7	-0.493073	-1.227143	0.225610	0.551115	1.000
569	GOBP_RESPONSE_TO_ACID_CHEMICAL	GO:0001101	6	-0.505889	-1.232070	0.210421	0.544766	1.000
570	GOBP_HEART_DEVELOPMENT	GO:0007507	6	-0.520285	-1.232219	0.224101	0.548187	1.000
571	REACTOME_MAPK_TARGETS_NUCLEAR_EVENTS_MEDIATED_...	R-HSA-450282	5	-0.561187	-1.233441	0.231092	0.549400	1.000
572	GOBP_SKIN_DEVELOPMENT	GO:0043588	5	-0.542083	-1.238939	0.223195	0.542384	1.000
573	GOBP_POSITIVE_REGULATION_OF_LOCOMOTION	GO:0040017	11	-0.428609	-1.239449	0.197959	0.545164	1.000
574	GOBP_REGULATION_OF_IMMUNE_SYSTEM_PROCESS	GO:0002682	21	-0.344463	-1.240053	0.169133	0.547757	1.000
575	GOBP_REGULATION_OF_SYSTEM_PROCESS	GO:0044057	18	-0.365548	-1.240926	0.200846	0.549736	1.000
576	GOBP_REGULATION_OF_CYTOSKELETON_ORGANIZATION	GO:0051493	5	-0.554605	-1.240950	0.217391	0.553548	1.000
577	GOBP_CARDIAC_MUSCLE_TISSUE_DEVELOPMENT	GO:0048738	6	-0.520285	-1.243663	0.200418	0.551720	1.000
578	GOBP_POSITIVE_REGULATION_OF_PEPTIDYL_TYROSINE_...	GO:0050731	8	-0.468586	-1.246005	0.197034	0.550952	1.000
579	GOBP_AMYLOID_PRECURSOR_PROTEIN_METABOLIC_PROCESS	GO:0042982	5	-0.569151	-1.247118	0.212058	0.552460	1.000
580	GOBP_LEUKOCYTE_CELL_CELL_ADHESION	GO:0007159	6	-0.534680	-1.247267	0.198783	0.556044	1.000
581	GOBP_REGULATION_OF_ORGAN_GROWTH	GO:0046620	5	-0.546573	-1.248735	0.200820	0.557220	1.000
582	GOBP_POSITIVE_REGULATION_OF_SMOOTH_MUSCLE_CELL...	GO:0048661	5	-0.550295	-1.251361	0.220859	0.556179	1.000
583	GOBP_PEPTIDE_HORMONE_SECRETION	GO:0030072	7	-0.475066	-1.252879	0.212982	0.557599	1.000
584	GOBP_RESPONSE_TO_ENDOGENOUS_STIMULUS	GO:0009719	34	-0.321686	-1.253451	0.167391	0.560453	1.000
585	GOBP_PERIPHERAL_NERVOUS_SYSTEM_DEVELOPMENT	GO:0007422	9	-0.453690	-1.254995	0.209016	0.561007	1.000
586	GOBP_RESPONSE_TO_OXYGEN_CONTAINING_COMPOUND	GO:1901700	39	-0.314459	-1.256246	0.169772	0.562651	1.000
587	GOBP_ENDOTHELIAL_CELL_PROLIFERATION	GO:0001935	8	-0.467869	-1.256616	0.198381	0.566195	1.000
588	GOBP_ESTABLISHMENT_OF_PROTEIN_LOCALIZATION_TO_...	GO:0072594	6	-0.522297	-1.262512	0.193416	0.557938	1.000
589	GOBP_CELLULAR_RESPONSE_TO_EXTRACELLULAR_STIMULUS	GO:0031668	8	-0.473513	-1.264480	0.179959	0.558265	1.000
590	GOBP_AMYLOID_PRECURSOR_PROTEIN_CATABOLIC_PROCESS	GO:0042987	5	-0.569151	-1.266016	0.212724	0.559536	1.000
591	GOBP_POSITIVE_REGULATION_OF_CELLULAR_PROTEIN_L...	GO:1903829	7	-0.494507	-1.271144	0.170732	0.553254	1.000
592	GOBP_ESTABLISHMENT_OF_PROTEIN_LOCALIZATION	GO:0045184	18	-0.372201	-1.275383	0.168399	0.548454	1.000
593	GOBP_MITOTIC_CELL_CYCLE	GO:0000278	10	-0.436571	-1.275402	0.190476	0.552771	1.000
594	HALLMARK_HYPOXIA	M5891	5	-0.564267	-1.277061	0.182203	0.553482	1.000
595	GOBP_POSITIVE_REGULATION_OF_INTRACELLULAR_SIGN...	GO:1902533	14	-0.397074	-1.279130	0.169197	0.553648	1.000
596	GOBP_NEGATIVE_REGULATION_OF_CELL_DEATH	GO:0060548	22	-0.361069	-1.287355	0.183932	0.540643	1.000
597	HALLMARK_APOPTOSIS	M5902	6	-0.544245	-1.287596	0.198198	0.544585	1.000
598	GOBP_REGULATION_OF_NEUROGENESIS	GO:0050767	14	-0.404806	-1.294264	0.148559	0.535449	1.000
599	GOBP_INOSITOL_LIPID_MEDIATED_SIGNALING	GO:0048017	8	-0.495881	-1.294940	0.168399	0.538567	1.000

Continuation of Table **S12**

	NAME	ID	SIZE	ES	NES	NOM.p.val	FDR.q.val	FWER.p.val
600	HALLMARK_UV_RESPONSE_UP	M5941	5	-0.572518	-1.295872	0.186441	0.541404	1.000
601	GOBP_MUSCLE_CELL_DEVELOPMENT	GO:0055001	5	-0.575117	-1.297733	0.179435	0.541769	1.000
602	GOBP_AMYLOID_BETA_METABOLIC_PROCESS	GO:0050435	5	-0.569151	-1.300247	0.178357	0.540693	1.000
603	GOBP_VASCULATURE_DEVELOPMENT	GO:0001944	17	-0.384114	-1.302199	0.159915	0.541130	1.000
604	GOBP_REGIONALIZATION	GO:0003002	6	-0.550943	-1.302645	0.154506	0.544892	1.000
605	GOBP_POSITIVE_REGULATION_OF_DEVELOPMENTAL_PROCESS	GO:0051094	32	-0.325929	-1.312977	0.121277	0.528227	1.000
606	GOBP_NEGATIVE_REGULATION_OF_CELL_POPULATION_PR...	GO:0008285	9	-0.480222	-1.315295	0.146751	0.527680	1.000
607	REACTOME_PLATELET_ACTIVATION_SIGNALING_AND_AGG...	R-HSA-76002	7	-0.519365	-1.315499	0.134956	0.531967	1.000
608	GOBP_REGULATION_OF_HORMONE_SECRETION	GO:0046883	8	-0.495165	-1.319630	0.154959	0.527597	1.000
609	GOBP_EPIDERMIS_DEVELOPMENT	GO:0008544	5	-0.579498	-1.320089	0.169422	0.531184	1.000
610	GOBP_NEGATIVE_REGULATION_OF_CELL_CYCLE	GO:0045786	5	-0.585152	-1.324033	0.142276	0.527542	1.000
611	GOBP_PATTERN_SPECIFICATION_PROCESS	GO:0007389	6	-0.550943	-1.329192	0.160083	0.521782	1.000
612	GOBP_REGULATION_OF_PEPTIDE_HORMONE_SECRETION	GO:0090276	6	-0.559672	-1.330337	0.148374	0.524241	1.000
613	GOBP_CELLULAR_RESPONSE_TO_OXYGEN_CONTAINING_CO...	GO:1901701	29	-0.349899	-1.334255	0.114407	0.520829	1.000
614	GOBP_PEPTIDE_SECRETION	GO:0002790	9	-0.475666	-1.338868	0.131915	0.515499	1.000
615	GOBP_REGULATION_OF_PEPTIDYL_SERINE_PHOSPHORYLA...	GO:0033135	8	-0.503369	-1.339895	0.140725	0.518143	1.000
616	GOBP_REGULATION_OF_MUSCLE_ADAPTATION	GO:0043502	5	-0.591301	-1.342875	0.159091	0.516677	1.000
617	REACTOME_BETA_CATENIN_INDEPENDENT_WNT_SIGNALING	R-RNO-3858494	5	-0.586736	-1.345371	0.150826	0.516470	1.000
618	GOBP_CELL_ACTIVATION	GO:0001775	21	-0.378232	-1.345901	0.124197	0.520503	1.000
619	GOBP_POSITIVE_REGULATION_OF_PEPTIDYL_SERINE_PH...	GO:0033138	8	-0.503369	-1.348521	0.132383	0.520212	1.000
620	GOBP_REGULATION_OF_ORGANELLE_ORGANIZATION	GO:0033043	14	-0.425096	-1.361664	0.124731	0.497228	1.000
621	GOBP_TAXIS	GO:0042330	14	-0.420183	-1.363002	0.117391	0.499503	1.000
622	GOBP_REGULATION_OF_CELLULAR_COMPONENT_MOVEMENT	GO:0051270	15	-0.416302	-1.364720	0.123318	0.500953	1.000
623	GOBP_REGULATION_OF_GTPASE_ACTIVITY	GO:0043087	5	-0.594421	-1.367121	0.150101	0.501364	1.000
624	GOBP_MUSCLE_ADAPTATION	GO:0043500	5	-0.591301	-1.369894	0.131631	0.500891	1.000
625	GOBP_PRI_MIRNA_TRANSCRIPTION_BY_RNA_POLYMERASE_II	GO:0061614	7	-0.540994	-1.376152	0.128514	0.492995	1.000
626	GOBP_POSITIVE_REGULATION_OF_PRI_MIRNA_TRANSCRI...	GO:1902895	7	-0.540994	-1.377712	0.130081	0.495068	1.000
627	GOBP_MONONUCLEAR_CELL_DIFFERENTIATION	GO:1903131	7	-0.531582	-1.377879	0.121399	0.499973	1.000
628	GOBP_REGULATION_OF_MAP_KINASE_ACTIVITY	GO:0043405	9	-0.490243	-1.378515	0.129103	0.503858	1.000
629	GOBP_POSITIVE_REGULATION_OF_NEURON_DEATH	GO:1901216	5	-0.610837	-1.383073	0.107071	0.500295	1.000
630	GOBP_TEMPERATURE_HOMEOSTASIS	GO:0001659	5	-0.610729	-1.387734	0.144269	0.496130	1.000
631	GOBP_POSITIVE_REGULATION_OF_LEUKOCYTE_CELL_CEL...	GO:1903039	5	-0.625227	-1.389115	0.119748	0.498816	1.000
632	GOBP_ACTIN_FILAMENT_BASED_PROCESS	GO:0030029	7	-0.547046	-1.390985	0.120582	0.500557	1.000
633	GOBP_OSTEOBLAST_DIFFERENTIATION	GO:0001649	5	-0.602279	-1.396642	0.128205	0.494447	1.000
634	GOBP_POSITIVE_REGULATION_OF_GLIOGENESIS	GO:0014015	5	-0.615425	-1.397355	0.117886	0.498841	1.000
635	GOBP_RESPONSE_TO_WOUNDING	GO:0009611	15	-0.431944	-1.398397	0.108051	0.502279	1.000
636	GOBP_REGULATION_OF_RESPONSE_TO_EXTERNAL_STIMULUS	GO:0032101	15	-0.425832	-1.399342	0.076923	0.505897	1.000
637	GOBP_REGULATION_OF_GLIOGENESIS	GO:0014013	5	-0.615425	-1.399512	0.124473	0.511528	1.000
638	GOBP_NEURON_DEATH	GO:0070997	18	-0.414330	-1.403058	0.094595	0.509347	1.000
639	GOBP_POSITIVE_REGULATION_OF_CELL_CELL_ADHESION	GO:0022409	5	-0.625227	-1.421438	0.117886	0.475040	1.000
640	GOBP_REGULATION_OF_CELLULAR_COMPONENT_BIOGENESIS	GO:0044087	10	-0.501060	-1.439004	0.069721	0.443309	1.000
641	GOBP_POSITIVE_REGULATION_OF_CELLULAR_COMPONENT...	GO:0051130	24	-0.386394	-1.439997	0.088421	0.447206	1.000
642	REACTOME_CELL_CYCLE	R-RNO-1640170	6	-0.589117	-1.440993	0.110647	0.451208	1.000

Continuation of Table **S12**

	NAME	ID	SIZE	ES	NES	NOM.p.val	FDR.q.val	FWER.p.val
643	GOBP_POSITIVE_REGULATION_OF_PHOSPHATIDYLINOSIT...	GO:0014068	5	-0.640037	-1.441014	0.113636	0.457072	1.000
644	GOBP_NUCLEAR_TRANSPORT	GO:0051169	5	-0.648141	-1.444187	0.106996	0.456106	1.000
645	REACTOME_INTERLEUKIN_4_AND_INTERLEUKIN_13_SIGN...	R-RNO-6785807	8	-0.542144	-1.450218	0.096579	0.449526	1.000
646	GOBP_NEGATIVE_REGULATION_OF_RESPONSE_TO_EXTERN...	GO:0032102	5	-0.649576	-1.450960	0.104895	0.453997	1.000
647	GOBP_RESPONSE_TO_GROWTH_FACTOR	GO:0070848	18	-0.439938	-1.473195	0.074786	0.413154	1.000
648	GOBP_PHOSPHATIDYLINOSITOL_3_KINASE_SIGNALING	GO:0014065	7	-0.591342	-1.474078	0.082803	0.417188	1.000
649	GOBP_NEURON_APOPTOTIC_PROCESS	GO:0051402	12	-0.478088	-1.474587	0.086242	0.422006	1.000
650	GOBP_REGULATION_OF_MAPK_CASCADE	GO:0043408	13	-0.478367	-1.476210	0.057377	0.424326	1.000
651	GOBP_NEGATIVE_REGULATION_OF_LOCOMOTION	GO:0040013	5	-0.674416	-1.490691	0.086316	0.400576	1.000
652	GOBP_EPITHELIAL_CELL_PROLIFERATION	GO:0050673	12	-0.482710	-1.492066	0.072435	0.403646	1.000
653	GOBP_T_CELL_ACTIVATION	GO:0042110	8	-0.569826	-1.501333	0.081933	0.391856	1.000
654	GOBP_LOCOMOTION	GO:0040011	31	-0.378983	-1.510029	0.046256	0.379981	1.000
655	GOBP_PEPTIDYL_AMINO_ACID_MODIFICATION	GO:0018193	22	-0.418669	-1.510622	0.036017	0.384932	1.000
656	GOBP_POSITIVE_REGULATION_OF_CELL_DIFFERENTIATION	GO:0045597	27	-0.397351	-1.514642	0.043912	0.382852	1.000
657	GOBP_POSITIVE_REGULATION_OF_ESTABLISHMENT_OF_P...	GO:1904951	5	-0.677115	-1.518990	0.072495	0.380272	1.000
658	GOBP_MUSCLE_SYSTEM_PROCESS	GO:0003012	9	-0.566462	-1.519516	0.047826	0.385371	1.000
659	GOBP_REGULATION_OF_MUSCLE_SYSTEM_PROCESS	GO:0090257	8	-0.584220	-1.522674	0.045652	0.385200	1.000
660	GOBP_MYOTUBE_DIFFERENTIATION	GO:0014902	5	-0.683719	-1.529059	0.060345	0.379134	1.000
661	GOBP_CIRCULATORY_SYSTEM_DEVELOPMENT	GO:0072359	21	-0.425040	-1.531168	0.052747	0.381561	1.000
662	GOBP_MUSCLE_STRUCTURE_DEVELOPMENT	GO:0061061	16	-0.469936	-1.536619	0.040632	0.377089	1.000
663	GOBP_ANIMAL_ORGAN_MORPHOGENESIS	GO:0009887	12	-0.503698	-1.539273	0.044625	0.378050	1.000
664	GOBP_REGULATION_OF_CELL_DEVELOPMENT	GO:0060284	15	-0.472826	-1.544749	0.037657	0.373583	1.000
665	GOBP_SMOOTH_MUSCLE_CELL_PROLIFERATION	GO:0048659	6	-0.659462	-1.565843	0.055066	0.337845	1.000
666	GOBP_REGULATION_OF_CELL_ADHESION	GO:0030155	13	-0.506298	-1.573046	0.038055	0.329712	1.000
667	GOBP_LYMPHOCYTE_ACTIVATION	GO:0046649	9	-0.565885	-1.589436	0.054167	0.306512	1.000
668	GOBP_PEPTIDYL_SERINE_MODIFICATION	GO:0018209	12	-0.515823	-1.596325	0.037895	0.299959	1.000
669	GOBP_ACTIVATION_OF_MAPK_ACTIVITY	GO:0000187	6	-0.677561	-1.609641	0.024145	0.281506	1.000
670	GOBP_POSITIVE_REGULATION_OF_MAP_KINASE_ACTIVITY	GO:0043406	6	-0.677561	-1.610984	0.032990	0.284697	1.000
671	GOBP_MYELOID_CELL_DIFFERENTIATION	GO:0030099	13	-0.511716	-1.618745	0.030501	0.277068	1.000
672	GOBP_WOUND_HEALING	GO:0042060	13	-0.523043	-1.619145	0.036559	0.282313	1.000
673	GOBP_REGULATION_OF_PHOSPHATIDYLINOSITOL_3_KINA...	GO:0014066	6	-0.668211	-1.620133	0.029528	0.286553	1.000
674	GOBP_POSITIVE_REGULATION_OF_CELLULAR_COMPONENT...	GO:0044089	9	-0.581902	-1.621444	0.047131	0.290513	1.000
675	GOBP_NEGATIVE_REGULATION_OF_MULTICELLULAR_ORGA...	GO:0051241	15	-0.498699	-1.625225	0.030905	0.289950	1.000
676	GOBP_REGULATION_OF_PEPTIDE_SECRETION	GO:0002791	7	-0.631246	-1.635431	0.033058	0.277872	1.000
677	REACTOME_SIGNALING_BY_RECEPTOR_TYROSINE_KINASES	R-RNO-9006934	26	-0.437481	-1.644474	0.027197	0.267731	1.000
678	GOBP_CELL_MIGRATION	GO:0016477	22	-0.458771	-1.651563	0.013158	0.262281	1.000
679	GOBP_REGULATION_OF_MULTICELLULAR_ORGANISMAL_DE...	GO:2000026	31	-0.423982	-1.652043	0.010917	0.268200	1.000
680	GOBP_REGULATION_OF_OSTEOCLAST_DIFFERENTIATION	GO:0045670	5	-0.722892	-1.652381	0.032922	0.274207	1.000
681	GOBP_REGULATION_OF_CELL_ACTIVATION	GO:0050865	8	-0.619836	-1.658298	0.041580	0.270636	1.000
682	GOBP_MYELOID_LEUKOCYTE_DIFFERENTIATION	GO:0002573	11	-0.559032	-1.674263	0.024283	0.250379	1.000
683	GOBP_NEGATIVE_REGULATION_OF_NERVOUS_SYSTEM_DEV...	GO:0051961	5	-0.758339	-1.689847	0.015054	0.231535	0.999
684	GOBP_POSITIVE_REGULATION_OF_MYELOID_LEUKOCYTE_...	GO:0002763	6	-0.720226	-1.691009	0.025478	0.236108	0.999
685	GOBP_MUSCLE_ORGAN_DEVELOPMENT	GO:0007517	9	-0.618178	-1.697899	0.041580	0.231359	0.999

Continuation of Table **S12**

	NAME	ID	SIZE	ES	NES	NOM.p.val	FDR.q.val	FWER.p.val
686	GOBP_REGULATION_OF_PEPTIDE_TRANSPORT	GO:0090087	10	-0.595757	-1.717912	0.020450	0.205472	0.997
687	GOBP_REGULATION_OF_VASCULAR_ASSOCIATED_SMOOTH_...	GO:1904705	5	-0.763553	-1.724440	0.012793	0.201777	0.996
688	GOBP_MUSCLE_CELL_PROLIFERATION	GO:0033002	8	-0.648073	-1.731200	0.016563	0.198255	0.994
689	GOBP_POSITIVE_REGULATION_OF_MULTICELLULAR_ORGA...	GO:0051240	32	-0.443673	-1.737259	0.008197	0.196567	0.994
690	GOBP_POSITIVE_REGULATION_OF_MAPK_CASCADE	GO:0043410	9	-0.632339	-1.748022	0.015217	0.188448	0.991
691	GOBP_REGULATION_OF_CELL_CELL_ADHESION	GO:0022407	9	-0.642770	-1.755669	0.019190	0.184707	0.990
692	GOBP_REGULATION_OF_CELL_DIFFERENTIATION	GO:0045595	36	-0.431519	-1.766512	0.008715	0.177398	0.985
693	GOBP_POSITIVE_REGULATION_OF_ORGANELLE_ORGANIZA...	GO:0010638	10	-0.619184	-1.778978	0.014644	0.167682	0.978
694	HALLMARK_TNFA_SIGNALING_VIA_NFKB	M5890	14	-0.562234	-1.783902	0.010142	0.167690	0.975
695	GOBP_OSTEOCLAST_DIFFERENTIATION	GO:0030316	6	-0.731707	-1.787862	0.017893	0.168708	0.970
696	GOBP_OSSIFICATION	GO:0001503	9	-0.637738	-1.790477	0.010142	0.172434	0.969
697	GOBP_ERK1_AND_ERK2_CASCADE	GO:0070371	5	-0.793286	-1.797290	0.008299	0.171282	0.958
698	GOBP_POSITIVE_REGULATION_OF_MYELOID_CELL_DIFFE...	GO:0045639	7	-0.717991	-1.799217	0.006237	0.176799	0.957
699	GOBP_LEUKOCYTE_DIFFERENTIATION	GO:0002521	15	-0.562342	-1.802932	0.013100	0.180780	0.955
700	GOBP_SKELETAL_MUSCLE_ORGAN_DEVELOPMENT	GO:0060538	5	-0.795857	-1.827798	0.012685	0.156117	0.929
701	GOBP_REGULATION_OF_MYELOID_CELL_DIFFERENTIATION	GO:0045637	9	-0.667040	-1.841018	0.004132	0.147869	0.905
702	GOBP_REGULATION_OF_MYELOID_LEUKOCYTE_DIFFERENT...	GO:0002761	8	-0.686563	-1.842793	0.010460	0.154857	0.903
703	GOBP_POSITIVE_REGULATION_OF_CELL_ADHESION	GO:0045785	7	-0.727441	-1.876811	0.008032	0.122151	0.825
704	GOBP_REGULATION_OF_LEUKOCYTE_DIFFERENTIATION	GO:1902105	9	-0.695397	-1.895522	0.012712	0.109228	0.765
705	GOBP_POSITIVE_REGULATION_OF_TRANSCRIPTION_BY_R...	GO:0045944	24	-0.518103	-1.897927	0.002105	0.114690	0.757
706	GOBP_ANATOMICAL_STRUCTURE_FORMATION_INVOLVED_I...	GO:0048646	20	-0.534976	-1.904357	0.002070	0.116432	0.729
707	GOBP_POSITIVE_REGULATION_OF_HEMOPOIESIS	GO:1903708	7	-0.745360	-1.909096	0.003953	0.120339	0.717
708	REACTOME_SIGNALING_BY_NTRKS	R-RNO-166520	18	-0.562111	-1.922023	0.004283	0.115332	0.658
709	GOBP_REGULATION_OF_HEMOPOIESIS	GO:1903706	10	-0.678086	-1.926710	0.004090	0.121362	0.643
710	GOBP_NEGATIVE_REGULATION_OF_CELL_DEVELOPMENT	GO:0010721	6	-0.815338	-1.940928	0.002096	0.117181	0.591
711	REACTOME_NGF_STIMULATED_TRANSCRIPTION	R-RNO-9031628	9	-0.704926	-1.968362	0.006316	0.096106	0.478
712	GOBP_CELL_POPULATION_PROLIFERATION	GO:0008283	31	-0.496633	-1.970494	0.000000	0.108387	0.476
713	REACTOME_NUCLEAR_EVENTS_KINASE_AND_TRANSCRIPTI...	R-RNO-198725	11	-0.679255	-1.990635	0.002179	0.103686	0.427
714	GOBP_MUSCLE_TISSUE_DEVELOPMENT	GO:0060537	11	-0.674644	-2.018202	0.002304	0.091098	0.334
715	GOBP_POSITIVE_REGULATION_OF_CELL_POPULATION_PR...	GO:0008284	21	-0.571767	-2.033686	0.000000	0.096320	0.289
716	GOBP_POSITIVE_REGULATION_OF_BIOSYNTHETIC_PROCESS	GO:0009891	27	-0.535520	-2.059733	0.002088	0.099952	0.235
717	GOBP_POSITIVE_REGULATION_OF_NUCLEOBASE_CONTAIN...	GO:0045935	28	-0.545353	-2.087747	0.000000	0.116790	0.185
718	GOBP_IMMUNE_SYSTEM_DEVELOPMENT	GO:0002520	17	-0.608753	-2.088167	0.002212	0.233580	0.185

End of Table

Supplementary Table S13: CTX-M late profile (45 DPL peak) GSEA results.

Begin of Table **S13**

	NAME	ID	SIZE	ES	NES	NOM.p.val	FDR.q.val	FWER.p.val
0	GOBP_TEMPERATURE_HOMEOSTASIS	GO:0001659	5	0.814246	1.904456	0.003205	1.000000	0.724
1	GOBP_LONG_TERM_MEMORY	GO:0007616	6	0.692177	1.737028	0.034843	1.000000	0.976
2	GOBP_NEGATIVE_REGULATION_OF_LIPID_METABOLIC_PR...	GO:0045833	5	0.724414	1.727147	0.046053	1.000000	0.978
3	GOBP_REGULATION_OF_LIPID_METABOLIC_PROCESS	GO:0019216	7	0.624385	1.719359	0.010753	1.000000	0.979
4	GOBP_CELLULAR_RESPONSE_TO_ORGANIC_CYCLIC_COMPOUND	GO:0071407	13	0.480000	1.695269	0.021552	1.000000	0.987
5	GOBP_CARBOHYDRATE_HOMEOSTASIS	GO:0033500	7	0.608837	1.655799	0.025735	1.000000	0.994
6	GOBP_REGULATION_OF_SMALL_MOLECULE_METABOLIC_PR...	GO:0062012	9	0.528680	1.650303	0.029167	1.000000	0.995
7	GOBP_MULTICELLULAR_ORGANISMAL_HOMEOSTASIS	GO:0048871	12	0.471564	1.632236	0.043269	1.000000	0.997
8	GOBP_MORPHOGENESIS_OF_AN_EPITHELIUM	GO:0002009	6	0.621951	1.594586	0.050955	1.000000	0.999
9	GOBP_CELLULAR_RESPONSE_TO_KETONE	GO:1901655	5	0.612336	1.432981	0.078261	1.000000	1.000
10	GOBP_ENDOTHELIAL_CELL_PROLIFERATION	GO:0001935	8	0.492299	1.411948	0.098540	1.000000	1.000
11	GOBP_NEGATIVE_REGULATION_OF_CATALYTIC_ACTIVITY	GO:0043086	17	0.363209	1.404382	0.091398	1.000000	1.000
12	GOBP_POSITIVE_REGULATION_OF_APOPTOTIC_SIGNALIN...	GO:2001235	5	0.579000	1.402481	0.108280	1.000000	1.000
13	GOBP_REGULATION_OF_VASCULATURE_DEVELOPMENT	GO:1901342	5	0.571971	1.393704	0.116208	1.000000	1.000
14	GOBP_SMALL_MOLECULE_METABOLIC_PROCESS	GO:0044281	15	0.391764	1.390275	0.055814	1.000000	1.000
15	GOBP_MALE_GAMETE_GENERATION	GO:0048232	5	0.577344	1.387334	0.110063	1.000000	1.000
16	GOBP_RESPONSE_TO_CARBOHYDRATE	GO:0009743	5	0.580980	1.380092	0.130000	1.000000	1.000
17	GOBP_MYELOID_LEUKOCYTE_ACTIVATION	GO:0002274	8	0.475974	1.375632	0.108949	1.000000	1.000
18	GOBP_NEGATIVE_REGULATION_OF_IMMUNE_SYSTEM_PROCESS	GO:0002683	5	0.566800	1.368483	0.109756	1.000000	1.000
19	GOBP_LIPID_METABOLIC_PROCESS	GO:0006629	10	0.438158	1.357959	0.120370	1.000000	1.000
20	GOBP_ESTABLISHMENT_OF_PROTEIN_LOCALIZATION_TO_...	GO:0072594	6	0.524390	1.340301	0.115152	1.000000	1.000
21	GOBP_CELLULAR_RESPONSE_TO_DNA_DAMAGE_STIMULUS	GO:0006974	5	0.568726	1.338634	0.138554	1.000000	1.000
22	GOBP_LEUKOCYTE_MIGRATION	GO:0050900	6	0.524390	1.335666	0.119205	1.000000	1.000
23	GOBP_POSITIVE_REGULATION_OF_LOCOMOTION	GO:0040017	11	0.428572	1.334863	0.130952	1.000000	1.000
24	GOBP_EPITHELIAL_CELL_PROLIFERATION	GO:0050673	12	0.381579	1.322804	0.103960	1.000000	1.000
25	GOBP_RESPONSE_TO_MONOSACCHARIDE	GO:0034284	5	0.580980	1.321477	0.128125	1.000000	1.000
26	GOBP_BLOOD_VESSEL_ENDOTHELIAL_CELL_MIGRATION	GO:0043534	8	0.444610	1.317215	0.142857	1.000000	1.000
27	GOBP_REGULATION_OF_ENDOTHELIAL_CELL_MIGRATION	GO:0010594	5	0.565265	1.314722	0.166667	1.000000	1.000
28	GOBP_REGULATION_OF_EPITHELIAL_CELL_MIGRATION	GO:0010632	6	0.507243	1.312504	0.151515	1.000000	1.000
29	GOBP_REGULATION_OF_LIPID_BIOSYNTHETIC_PROCESS	GO:0046890	6	0.515207	1.297160	0.153846	1.000000	1.000
30	GOBP_ENDOTHELIAL_CELL_MIGRATION	GO:0043542	8	0.444610	1.292866	0.153846	1.000000	1.000
31	GOBP_POSITIVE_REGULATION_OF_IMMUNE_RESPONSE	GO:0050778	9	0.421582	1.278661	0.181818	1.000000	1.000
32	GOBP_LIPID_BIOSYNTHETIC_PROCESS	GO:0008610	6	0.515207	1.277405	0.155709	1.000000	1.000
33	GOBP_CELLULAR_GLUCOSE_HOMEOSTASIS	GO:0001678	5	0.554217	1.270988	0.166163	1.000000	1.000
34	GOBP_RESPONSE_TO_ORGANIC_CYCLIC_COMPOUND	GO:0014070	20	0.314057	1.260219	0.128655	1.000000	1.000
35	GOBP_CELL_ACTIVATION	GO:0001775	21	0.298530	1.251973	0.154696	1.000000	1.000
36	GOBP_PHAGOCYTOSIS	GO:0006909	5	0.518072	1.250350	0.170732	1.000000	1.000
37	GOBP_CELLULAR_LIPID_METABOLIC_PROCESS	GO:0044255	7	0.471653	1.248008	0.194946	1.000000	1.000
38	GOBP_PATTERN_SPECIFICATION_PROCESS	GO:0007389	6	0.475610	1.246578	0.170492	1.000000	1.000
39	GOBP_NEGATIVE_REGULATION_OF_GENE_EXPRESSION	GO:0010629	12	0.368421	1.240564	0.188285	1.000000	1.000
40	GOBP_RESPONSE_TO_NITROGEN_COMPOUND	GO:1901698	34	0.264033	1.238171	0.116279	1.000000	1.000

Continuation of Table **S13**

	NAME	ID	SIZE	ES	NES	NOM.p.val	FDR.q.val	FWER.p.val
41	GOBP_PEPTIDE_BIOSYNTHETIC_PROCESS	GO:0043043	7	0.444321	1.238102	0.184906	1.000000	1.000
42	GOBP_POSITIVE_REGULATION_OF_ANION_TRANSPORT	GO:1903793	9	0.417721	1.232201	0.199134	1.000000	1.000
43	GOBP_INTRINSIC_APOPTOTIC_SIGNALING_PATHWAY	GO:0097193	5	0.503992	1.223392	0.199357	1.000000	1.000
44	GOBP_CELLULAR_RESPONSE_TO_NITROGEN_COMPOUND	GO:1901699	20	0.302673	1.223322	0.193939	1.000000	1.000
45	GOBP_TISSUE_MORPHOGENESIS	GO:0048729	7	0.444444	1.211666	0.189189	1.000000	1.000
46	GOBP_MEMORY	GO:0007613	13	0.348991	1.210904	0.181395	1.000000	1.000
47	GOBP_REGULATION_OF_CELLULAR_CATABOLIC_PROCESS	GO:0031329	10	0.378560	1.207050	0.195833	1.000000	1.000
48	GOBP_CELL_CYCLE_PROCESS	GO:0022402	11	0.363636	1.200359	0.194805	1.000000	1.000
49	GOBP_REGIONALIZATION	GO:0003002	6	0.475610	1.196240	0.231317	1.000000	1.000
50	GOBP_AMIDE_BIOSYNTHETIC_PROCESS	GO:0043604	7	0.444321	1.188910	0.246269	1.000000	1.000
51	GOBP_REGULATION_OF_PEPTIDE_HORMONE_SECRETION	GO:0090276	6	0.457240	1.187195	0.228374	1.000000	1.000
52	GOBP_PLATELET_ACTIVATION	GO:0030168	6	0.451219	1.179721	0.228669	1.000000	1.000
53	GOBP_TISSUE_MIGRATION	GO:0090130	9	0.408387	1.178161	0.240506	1.000000	1.000
54	GOBP_NEGATIVE_REGULATION_OF_CELL_CYCLE	GO:0045786	5	0.492080	1.160302	0.259016	1.000000	1.000
55	GOBP_POSITIVE_REGULATION_OF_DEFENSE_RESPONSE	GO:0031349	5	0.474561	1.148284	0.249147	1.000000	1.000
56	GOBP_FAT_CELL_DIFFERENTIATION	GO:0045444	9	0.367277	1.133323	0.248062	1.000000	1.000
57	GOBP_NEGATIVE_REGULATION_OF_MOLECULAR_FUNCTION	GO:0044092	24	0.263408	1.127556	0.262069	1.000000	1.000
58	GOBP_DEVELOPMENTAL_PROCESS_INVOLVED_IN_REPRODU...	GO:0003006	11	0.337662	1.122976	0.244898	1.000000	1.000
59	GOBP_CARBOHYDRATE_DERIVATIVE_METABOLIC_PROCESS	GO:1901135	7	0.407407	1.117982	0.285714	1.000000	1.000
60	GOBP_MITOTIC_CELL_CYCLE	GO:0000278	10	0.358974	1.117073	0.296943	1.000000	1.000
61	GOBP_PHOSPHATIDYLINOSITOL_3_KINASE_SIGNALING	GO:0014065	7	0.407407	1.114405	0.283088	1.000000	1.000
62	GOBP_NEGATIVE_REGULATION_OF_HYDROLASE_ACTIVITY	GO:0051346	11	0.341251	1.104337	0.287500	1.000000	1.000
63	GOBP_ANATOMICAL_STRUCTURE_HOMEOSTASIS	GO:0060249	7	0.395062	1.091505	0.309859	1.000000	1.000
64	GOBP_LYMPHOCYTE_ACTIVATION	GO:0046649	9	0.367089	1.089079	0.324786	1.000000	1.000
65	REACTOME_MEMBRANE_TRAFFICKING	R-RNO-199991	5	0.454434	1.084972	0.299094	1.000000	1.000
66	REACTOME_G_ALPHA_Z_SIGNALLING_EVENTS	R-RNO-418597	5	0.445783	1.073439	0.346479	1.000000	1.000
67	REACTOME_VESICLE_MEDIATED_TRANSPORT	R-RNO-5653656	5	0.454434	1.066071	0.345794	1.000000	1.000
68	GOBP_RESPONSE_TO_NUTRIENT	GO:0007584	5	0.453874	1.064207	0.361635	1.000000	1.000
69	GOBP_REGULATION_OF_IMMUNE_RESPONSE	GO:0050776	12	0.305695	1.060265	0.355769	1.000000	1.000
70	GOBP_NEGATIVE_REGULATION_OF_PHOSPHORUS_METABOL...	GO:0010563	9	0.348214	1.058991	0.371429	1.000000	1.000
71	GOBP_REGULATION_OF_INFLAMMATORY_RESPONSE	GO:0050727	7	0.384692	1.057949	0.328720	1.000000	1.000
72	GOBP_REGULATION_OF_DEFENSE_RESPONSE	GO:0031347	7	0.384692	1.056955	0.340000	1.000000	1.000
73	GOBP_REGULATION_OF_CELL_ACTIVATION	GO:0050865	8	0.362500	1.055096	0.368231	1.000000	1.000
74	GOBP_MONOCARBOXYLIC_ACID_METABOLIC_PROCESS	GO:0032787	6	0.408672	1.054570	0.378571	1.000000	1.000
75	GOBP_POSITIVE_REGULATION_OF_EPITHELIAL_CELL_MI...	GO:0010634	5	0.445783	1.054308	0.369637	1.000000	1.000
76	GOBP_REGULATION_OF_HORMONE_LEVELS	GO:0010817	12	0.304942	1.054018	0.393443	1.000000	1.000
77	GOBP_EPIDERMIS_DEVELOPMENT	GO:0008544	5	0.431328	1.044782	0.403614	1.000000	1.000
78	GOBP_LEUKOCYTE_MEDIATED_IMMUNITY	GO:0002443	7	0.382716	1.042437	0.349265	1.000000	1.000
79	GOBP_T_CELL_ACTIVATION	GO:0042110	8	0.362500	1.041906	0.378788	1.000000	1.000
80	GOBP_OSSIFICATION	GO:0001503	9	0.341772	1.041450	0.389830	1.000000	1.000
81	GOBP_ERBB_SIGNALING_PATHWAY	GO:0038127	5	0.445783	1.039690	0.374150	1.000000	1.000
82	REACTOME_BETA_CATENIN_INDEPENDENT_WNT_SIGNALING	R-RNO-3858494	5	0.445783	1.037556	0.372014	1.000000	1.000
83	GOBP_REGULATION_OF_PEPTIDE_SECRETION	GO:0002791	7	0.390455	1.035456	0.394366	1.000000	1.000

Continuation of Table **S13**

	NAME	ID	SIZE	ES	NES	NOM.p.val	FDR.q.val	FWER.p.val
84	GOBP_PROTEIN_KINASE_B_SIGNALING	GO:0043491	6	0.406964	1.032593	0.381119	1.000000	1.000
85	GOBP_REGULATION_OF_PHOSPHATIDYLINOSITOL_3_KINA...	GO:0014066	6	0.402439	1.030972	0.391304	1.000000	1.000
86	GOBP_CYTOSKELETON_ORGANIZATION	GO:0007010	11	0.308775	1.021986	0.383966	1.000000	1.000
87	GOBP_EMBRYO_DEVELOPMENT_ENDING_IN_BIRTH_OR_EGG...	GO:0009792	9	0.341772	1.020099	0.403101	1.000000	1.000
88	GOBP_CIRCADIAN_REGULATION_OF_GENE_EXPRESSION	GO:0032922	5	0.420377	1.019278	0.396875	1.000000	1.000
89	GOBP_CELLULAR_RESPONSE_TO_ALCOHOL	GO:0097306	5	0.433735	1.016363	0.372483	1.000000	1.000
90	GOBP_NEGATIVE_REGULATION_OF_BIOSYNTHETIC_PROCESS	GO:0009890	13	0.279729	1.011303	0.403361	1.000000	1.000
91	GOBP_NEGATIVE_REGULATION_OF_SYNAPTIC_TRANSMISSION	GO:0050805	6	0.398952	1.009713	0.438538	1.000000	1.000
92	GOBP_NEGATIVE_REGULATION_OF_ANION_TRANSPORT	GO:1903792	8	0.348817	1.007726	0.453061	1.000000	1.000
93	GOBP_ORGANIC_HYDROXY_COMPOUND_TRANSPORT	GO:0015850	6	0.378203	1.000933	0.425087	1.000000	1.000
94	GOBP_REGULATION_OF_PEPTIDE_TRANSPORT	GO:0090087	10	0.313833	0.999715	0.457831	1.000000	1.000
95	GOBP_NEGATIVE_REGULATION_OF_RESPONSE_TO_EXTERN...	GO:0032102	5	0.418326	0.999598	0.421569	1.000000	1.000
96	GOBP_REGULATION_OF_PHOSPHORUS_METABOLIC_PROCESS	GO:0051174	27	0.228089	0.994008	0.475862	1.000000	1.000
97	GOBP_CIRCULATORY_SYSTEM_PROCESS	GO:0003013	5	0.426402	0.993006	0.405844	1.000000	1.000
98	GOBP_INSULIN_SECRETION	GO:0030073	7	0.365790	0.989077	0.417476	1.000000	1.000
99	REACTOME_NEUTROPHIL_DEGRANULATION	R-RNO-6798695	6	0.378049	0.988374	0.451389	1.000000	1.000
100	GOBP_POSITIVE_REGULATION_OF_EPITHELIAL_CELL_PR...	GO:0050679	7	0.358025	0.985677	0.407692	1.000000	1.000
101	GOBP_PEPTIDE_HORMONE_SECRETION	GO:0030072	7	0.365790	0.984920	0.444840	1.000000	1.000
102	GOBP_REGULATION_OF_CELL_CYCLE_PROCESS	GO:0010564	5	0.399378	0.983288	0.448276	1.000000	1.000
103	GOBP_MULTICELLULAR_ORGANISM_REPRODUCTION	GO:0032504	12	0.285784	0.980875	0.468402	1.000000	1.000
104	GOBP_NEGATIVE_REGULATION_OF_TRANSCRIPTION_BY_R...	GO:0000122	10	0.326612	0.976803	0.456522	1.000000	1.000
105	GOBP_RESPONSE_TO_NICOTINE	GO:0035094	7	0.361004	0.976342	0.469751	1.000000	1.000
106	GOBP_REGULATION_OF_NEUROTRANSMITTER_TRANSPORT	GO:0051588	6	0.381196	0.976023	0.480000	1.000000	1.000
107	GOBP_POSITIVE_REGULATION_OF_RESPONSE_TO_EXTERN...	GO:0032103	9	0.327050	0.975590	0.444915	1.000000	1.000
108	GOBP_PROTEIN_AUTOPHOSPHORYLATION	GO:0046777	5	0.409639	0.973133	0.453642	1.000000	1.000
109	GOBP_CELL_ACTIVATION_INVOLVED_IN_IMMUNE_RESPONSE	GO:0002263	6	0.378049	0.972605	0.490260	1.000000	1.000
110	GOBP_NEURON_MIGRATION	GO:0001764	5	0.409639	0.969626	0.437700	1.000000	1.000
111	GOBP_MYELOID_LEUKOCYTE_MEDIATED_IMMUNITY	GO:0002444	6	0.378049	0.968809	0.489286	1.000000	1.000
112	GOBP_NEGATIVE_REGULATION_OF_PROTEIN_MODIFICATI...	GO:0031400	10	0.304006	0.964258	0.496154	1.000000	1.000
113	GOBP_NEGATIVE_REGULATION_OF_SIGNALING	GO:0023057	21	0.240151	0.961587	0.509554	1.000000	1.000
114	GOBP_MATERNAL_PROCESS_INVOLVED_IN_FEMALE_PREGN...	GO:0060135	5	0.408584	0.960171	0.470968	1.000000	1.000
115	GOBP_REGULATION_OF_ORGAN_GROWTH	GO:0046620	5	0.397590	0.957730	0.478395	1.000000	1.000
116	GOBP_IN_UTERO_EMBRYONIC_DEVELOPMENT	GO:0001701	8	0.337500	0.957276	0.505618	1.000000	1.000
117	GOBP_MAINTENANCE_OF_LOCATION	GO:0051235	7	0.351857	0.954443	0.510949	1.000000	1.000
118	GOBP_REGULATION_OF_CARBOHYDRATE_BIOSYNTHETIC_P...	GO:0043255	5	0.397590	0.952087	0.467320	1.000000	1.000
119	REACTOME_SIGNALING_BY_WNT	R-RNO-195721	7	0.345679	0.946157	0.503521	1.000000	1.000
120	GOBP_REGULATION_OF_MITOTIC_CELL_CYCLE	GO:0007346	5	0.398390	0.942650	0.500000	1.000000	1.000
121	GOBP_REGULATION_OF_CARBOHYDRATE_METABOLIC_PROCESS	GO:0006109	5	0.397590	0.941609	0.507886	1.000000	1.000
122	GOBP_POSITIVE_REGULATION_OF_ESTABLISHMENT_OF_P...	GO:1904951	5	0.397590	0.936090	0.512739	1.000000	1.000
123	GOBP_NEUROINFLAMMATORY_RESPONSE	GO:0150076	5	0.397590	0.931265	0.529231	1.000000	1.000
124	GOBP_CELLULAR_RESPONSE_TO_OXYGEN_CONTAINING_CO...	GO:1901701	29	0.206252	0.930143	0.559055	1.000000	1.000
125	GOBP_ORGANONITROGEN_COMPOUND_BIOSYNTHETIC_PROCESS	GO:1901566	13	0.266667	0.927968	0.566667	1.000000	1.000
126	GOBP_POSITIVE_REGULATION_OF_PHOSPHATIDYLINOSIT...	GO:0014068	5	0.397590	0.927255	0.520767	1.000000	1.000

Continuation of Table **S13**

	NAME	ID	SIZE	ES	NES	NOM.p.val	FDR.q.val	FWER.p.val
127	GOBP_ORGAN_GROWTH	GO:0035265	5	0.397590	0.917706	0.539855	1.000000	1.000
128	GOBP_MUSCLE_CELL_DEVELOPMENT	GO:0055001	5	0.385542	0.916988	0.545150	1.000000	1.000
129	GOBP_LONG_TERM_SYNAPTIC_DEPRESSION	GO:0060292	5	0.385556	0.915172	0.510972	1.000000	1.000
130	GOBP_PROTEIN_MODIFICATION_BY_SMALL_PROTEIN_CON...	GO:0032446	6	0.360075	0.913811	0.571885	1.000000	1.000
131	GOBP_PROTEIN_MODIFICATION_BY_SMALL_PROTEIN_CON...	GO:0070647	7	0.332776	0.913194	0.588235	1.000000	1.000
132	GOBP_SPROUTING_ANGIOGENESIS	GO:0002040	5	0.394763	0.911516	0.535503	1.000000	1.000
133	GOBP_REGULATION_OF_PROTEIN_MODIFICATION_PROCESS	GO:0031399	29	0.209465	0.910855	0.605839	1.000000	1.000
134	GOBP_REGULATION_OF_CELLULAR_COMPONENT_MOVEMENT	GO:0051270	15	0.245556	0.909407	0.630769	1.000000	1.000
135	GOBP_PRI_MIRNA_TRANSCRIPTION_BY_RNA_POLYMERASE_II	GO:0061614	7	0.323428	0.906663	0.581132	1.000000	1.000
136	GOBP_GLUCOSE_METABOLIC_PROCESS	GO:0006006	6	0.351847	0.902609	0.600000	1.000000	1.000
137	GOBP_TISSUE_HOMEOSTASIS	GO:0001894	5	0.385542	0.902144	0.574074	1.000000	1.000
138	GOBP_CARBOHYDRATE_BIOSYNTHETIC_PROCESS	GO:0016051	5	0.397590	0.899090	0.571031	1.000000	1.000
139	GOBP_REGULATION_OF_HORMONE_SECRETION	GO:0046883	8	0.321656	0.898972	0.575758	1.000000	1.000
140	GOBP_POSITIVE_REGULATION_OF_PRI_MIRNA_TRANSCRI...	GO:1902895	7	0.323428	0.894710	0.562044	1.000000	1.000
141	GOBP_POSITIVE_REGULATION_OF_ENDOTHELIAL_CELL_P...	GO:0001938	6	0.353658	0.893448	0.605960	1.000000	1.000
142	GOBP_SKELETAL_MUSCLE_ORGAN_DEVELOPMENT	GO:0060538	5	0.385542	0.892829	0.575949	1.000000	1.000
143	GOBP_NEUROTRANSMITTER_SECRETION	GO:0007269	6	0.351443	0.890271	0.612245	1.000000	1.000
144	GOBP_CYTOKINE_PRODUCTION	GO:0001816	10	0.275555	0.884713	0.608696	1.000000	1.000
145	GOBP_MONOSACCHARIDE_METABOLIC_PROCESS	GO:0005996	6	0.351847	0.882583	0.565693	1.000000	1.000
146	GOBP_POSITIVE_REGULATION_OF_CYTOKINE_PRODUCTION	GO:0001819	7	0.318705	0.882047	0.580882	1.000000	1.000
147	GOBP_NEGATIVE_REGULATION_OF_RESPONSE_TO_STIMULUS	GO:0048585	16	0.226430	0.880522	0.646707	1.000000	1.000
148	GOBP_CELLULAR_MACROMOLECULE_CATABOLIC_PROCESS	GO:0044265	6	0.341463	0.874619	0.559871	1.000000	1.000
149	GOBP_RESPONSE_TO_BIOTIC_STIMULUS	GO:0009607	17	0.228665	0.874089	0.650538	1.000000	1.000
150	GOBP_NEGATIVE_REGULATION_OF_NUCLEOBASE_CONTAIN...	GO:0045934	13	0.254257	0.871554	0.654545	1.000000	1.000
151	GOBP_RESPONSE_TO_INSULIN	GO:0032868	5	0.368194	0.870799	0.589595	1.000000	1.000
152	GOBP_RESPONSE_TO_ALKALOID	GO:0043279	5	0.368372	0.870473	0.608434	1.000000	1.000
153	GOBP_REGULATION_OF_CELLULAR_RESPONSE_TO_STRESS	GO:0080135	9	0.286117	0.869576	0.660517	1.000000	1.000
154	GOBP_PROTEIN_DEPHOSPHORYLATION	GO:0006470	7	0.315974	0.869000	0.642857	1.000000	1.000
155	GOBP_PEPTIDE_SECRETION	GO:0002790	9	0.281645	0.866491	0.628352	1.000000	1.000
156	GOBP_POSITIVE_REGULATION_OF_PROTEIN_PHOSPHORYL...	GO:0001934	23	0.201080	0.862527	0.720000	1.000000	1.000
157	GOBP_POSITIVE_REGULATION_OF_NEURON_DEATH	GO:1901216	5	0.362233	0.862181	0.588757	1.000000	1.000
158	GOBP_REGULATION_OF_VESICLE_MEDIATED_TRANSPORT	GO:0060627	12	0.255489	0.860303	0.699074	1.000000	1.000
159	GOBP_RESPONSE_TO_CALCIUM_ION	GO:0051592	6	0.329268	0.859991	0.653430	1.000000	1.000
160	GOBP_ORGANIC_ACID_METABOLIC_PROCESS	GO:0006082	7	0.313544	0.857757	0.648855	1.000000	1.000
161	GOBP_LEUKOCYTE_DIFFERENTIATION	GO:0002521	15	0.226083	0.855127	0.664865	1.000000	1.000
162	REACTOME_OPIOID_SIGNALLING	R-RNO-111885	12	0.250000	0.848256	0.652381	1.000000	1.000
163	GOBP_REGULATION_OF_PHOSPHOLIPASE_ACTIVITY	GO:0010517	6	0.320891	0.847567	0.651007	1.000000	1.000
164	GOBP_REGULATION_OF_FAT_CELL_DIFFERENTIATION	GO:0045598	6	0.326866	0.847358	0.645270	1.000000	1.000
165	GOBP_ORGANOPHOSPHATE_METABOLIC_PROCESS	GO:0019637	7	0.308642	0.839576	0.642857	1.000000	1.000
166	GOBP_DEPHOSPHORYLATION	GO:0016311	7	0.315974	0.837657	0.656489	1.000000	1.000
167	GOBP_REGULATION_OF_LIPASE_ACTIVITY	GO:0060191	6	0.320891	0.834725	0.694545	1.000000	1.000
168	GOBP_POSITIVE_REGULATION_OF_SMALL_MOLECULE_MET...	GO:0062013	5	0.353938	0.833634	0.672956	1.000000	1.000
169	GOBP_REPRODUCTIVE_SYSTEM_DEVELOPMENT	GO:0061458	6	0.329268	0.828429	0.667797	1.000000	1.000

Continuation of Table **S13**

	NAME	ID	SIZE	ES	NES	NOM.p.val	FDR.q.val	FWER.p.val
170	GOBP_POSITIVE_REGULATION_OF_PROTEIN_MODIFICATI...	GO:0031401	23	0.201080	0.826885	0.711656	1.000000	1.000
171	GOBP_REGULATION_OF_LIPID_LOCALIZATION	GO:1905952	5	0.342119	0.826247	0.706485	1.000000	1.000
172	REACTOME_NUCLEAR_EVENTS_KINASE_AND_TRANSCRIPTI...	R-RNO-198725	11	0.246753	0.818523	0.786290	1.000000	1.000
173	GOBP_RESPONSE_TO_TEMPERATURE_STIMULUS	GO:0009266	9	0.265823	0.815903	0.732000	1.000000	1.000
174	GOBP_POSITIVE_REGULATION_OF_NEURON_PROJECTION_...	GO:0010976	5	0.340989	0.815362	0.669670	1.000000	1.000
175	GOBP_REGULATION_OF_RESPONSE_TO_STRESS	GO:0080134	19	0.202853	0.815264	0.758242	1.000000	1.000
176	GOBP_POSITIVE_REGULATION_OF_CELLULAR_PROTEIN_L...	GO:1903829	7	0.299543	0.810529	0.688172	1.000000	1.000
177	GOBP_MUSCLE_TISSUE_DEVELOPMENT	GO:0060537	11	0.246753	0.808128	0.758475	1.000000	1.000
178	GOBP_HORMONE_TRANSPORT	GO:0009914	9	0.269563	0.806984	0.722846	1.000000	1.000
179	GOBP_HOMEOSTATIC_PROCESS	GO:0042592	31	0.181896	0.806767	0.762712	1.000000	1.000
180	GOBP_PEPTIDYL_SERINE_MODIFICATION	GO:0018209	12	0.236842	0.804434	0.754167	1.000000	1.000
181	GOBP_DEFENSE_RESPONSE	GO:0006952	19	0.204479	0.802198	0.805031	1.000000	1.000
182	GOBP_RESPONSE_TO_TUMOR_NECROSIS_FACTOR	GO:0034612	7	0.293054	0.801823	0.763441	1.000000	1.000
183	GOBP_IMMUNE_SYSTEM_DEVELOPMENT	GO:0002520	17	0.211268	0.799547	0.754717	1.000000	1.000
184	GOBP_ORGANIC_HYDROXY_COMPOUND_METABOLIC_PROCESS	GO:1901615	7	0.293523	0.797391	0.738351	1.000000	1.000
185	GOBP_NEGATIVE_REGULATION_OF_CYSTEINE_TYPE_ENDO...	GO:2000117	5	0.333518	0.794337	0.710884	1.000000	1.000
186	GOBP_VASCULATURE_DEVELOPMENT	GO:0001944	17	0.201569	0.792881	0.789744	1.000000	1.000
187	GOBP_POSITIVE_REGULATION_OF_IMMUNE_SYSTEM_PROCESS	GO:0002684	18	0.195830	0.789344	0.801242	1.000000	1.000
188	REACTOME_INTEGRATION_OF_ENERGY_METABOLISM	R-RNO-163685	5	0.337349	0.787045	0.699690	1.000000	1.000
189	GOBP_NEGATIVE_REGULATION_OF_CELL_POPULATION_PR...	GO:0008285	9	0.259077	0.786441	0.782443	1.000000	1.000
190	GOBP_VESICLE_MEDIATED_TRANSPORT_IN_SYNAPSE	GO:0099003	7	0.280546	0.782036	0.740072	1.000000	1.000
191	GOBP_IMPORT_INTO_CELL	GO:0098657	6	0.304878	0.776063	0.786408	1.000000	1.000
192	GOBP_REGULATION_OF_CELL_CYCLE	GO:0051726	13	0.219995	0.774998	0.798030	1.000000	1.000
193	GOBP_INFLAMMATORY_RESPONSE	GO:0006954	12	0.231622	0.774764	0.764940	1.000000	1.000
194	GOBP_CELLULAR_RESPONSE_TO_ABIOTIC_STIMULUS	GO:0071214	7	0.289581	0.774728	0.756944	1.000000	1.000
195	GOBP_REGULATION_OF_NEUROTRANSMITTER_LEVELS	GO:0001505	7	0.283994	0.774473	0.762542	1.000000	1.000
196	GOBP_CELL_MIGRATION	GO:0016477	22	0.186293	0.771530	0.788889	1.000000	1.000
197	GOBP_REGULATION_OF_PROTEIN_BINDING	GO:0043393	7	0.283951	0.769649	0.742537	1.000000	1.000
198	GOBP_ESTABLISHMENT_OF_PROTEIN_LOCALIZATION	GO:0045184	18	0.186380	0.767593	0.848485	1.000000	1.000
199	GOBP_GAMETE_GENERATION	GO:0007276	9	0.256660	0.766181	0.717949	1.000000	1.000
200	GOBP_RESPONSE_TO_LIPID	GO:0033993	19	0.189719	0.763951	0.787356	1.000000	1.000
201	GOBP_OSTEOBLAST_DIFFERENTIATION	GO:0001649	5	0.325301	0.761783	0.764706	1.000000	1.000
202	GOBP_REGULATION_OF_DNA_BINDING_TRANSCRIPTION_F...	GO:0051090	16	0.205669	0.757254	0.837696	1.000000	1.000
203	GOBP_PROTEIN_LOCALIZATION_TO_ORGANELLE	GO:0033365	9	0.252003	0.757238	0.815574	1.000000	1.000
204	REACTOME_INTRACELLULAR_SIGNALING_BY_SECOND_MES...	R-RNO-9006925	15	0.205479	0.756747	0.839024	1.000000	1.000
205	GOBP_EXOCYTOSIS	GO:0006887	11	0.230617	0.754101	0.798246	1.000000	1.000
206	GOBP_NEUROTRANSMITTER_TRANSPORT	GO:0006836	7	0.283994	0.750662	0.818182	1.000000	1.000
207	GOBP_CIRCULATORY_SYSTEM_DEVELOPMENT	GO:0072359	21	0.180914	0.747816	0.840491	1.000000	1.000
208	GOBP_POSITIVE_REGULATION_OF_PEPTIDYL_SERINE_PH...	GO:0033138	8	0.262348	0.745399	0.799228	1.000000	1.000
209	GOBP_REGULATION_OF_PEPTIDYL_SERINE_PHOSPHORYLA...	GO:0033135	8	0.262348	0.744444	0.832714	1.000000	1.000
210	GOBP_RESPONSE_TO_PEPTIDE	GO:1901652	18	0.192842	0.737911	0.856322	1.000000	1.000
211	GOBP_POSITIVE_REGULATION_OF_SECRETION	GO:0051047	6	0.285577	0.735848	0.844884	1.000000	1.000
212	GOBP_PEPTIDYL_LYSINE_MODIFICATION	GO:0018205	5	0.306102	0.734276	0.818182	1.000000	1.000

Continuation of Table **S13**

	NAME	ID	SIZE	ES	NES	NOM.p.val	FDR.q.val	FWER.p.val
213	GOBP_MULTI_MULTICELLULAR_ORGANISM_PROCESS	GO:0044706	8	0.254816	0.734001	0.797753	1.000000	1.000
214	GOBP_POSTTRANSCRIPTIONAL_REGULATION_OF_GENE_EX...	GO:0010608	8	0.262097	0.732178	0.836957	1.000000	1.000
215	GOBP_RESPONSE_TO_BACTERIUM	GO:0009617	13	0.208473	0.731638	0.841346	1.000000	1.000
216	GOBP_REGULATION_OF_OSTEOCLAST_DIFFERENTIATION	GO:0045670	5	0.316704	0.731299	0.819079	1.000000	1.000
217	GOBP_REGULATION_OF_RESPONSE_TO_EXTERNAL_STIMULUS	GO:0032101	15	0.198920	0.729445	0.888350	1.000000	1.000
218	GOBP_MONOAMINE_TRANSPORT	GO:0015844	5	0.309087	0.727351	0.796178	1.000000	1.000
219	GOBP_ANATOMICAL_STRUCTURE_FORMATION_INVOLVED_I...	GO:0048646	20	0.182319	0.726442	0.883333	1.000000	1.000
220	REACTOME_NGF_STIMULATED_TRANSCRIPTION	R-RNO-9031628	9	0.240506	0.725770	0.841202	1.000000	1.000
221	GOBP_RESPONSE_TO_MOLECULE_OF_BACTERIAL_ORIGIN	GO:0002237	13	0.208473	0.721411	0.861472	1.000000	1.000
222	REACTOME_ANTI_INFLAMMATORY_RESPONSE_FAVOURING_...	R-HSA-9662851	5	0.295695	0.720439	0.864112	1.000000	1.000
223	GOBP_MUSCLE_ORGAN_DEVELOPMENT	GO:0007517	9	0.240506	0.717366	0.835878	1.000000	1.000
224	GOBP_MYELOID_CELL_DIFFERENTIATION	GO:0030099	13	0.200000	0.716302	0.868020	1.000000	1.000
225	GOBP_POSITIVE_REGULATION_OF_ION_TRANSPORT	GO:0043270	17	0.184707	0.713772	0.835979	1.000000	1.000
226	REACTOME_G_PROTEIN_MEDIATED_EVENTS	R-RNO-112040	9	0.240506	0.712580	0.851711	1.000000	1.000
227	GOBP_CELLULAR_RESPONSE_TO_LIPID	GO:0071396	15	0.196834	0.711133	0.867299	1.000000	1.000
228	GOBP_RESPONSE_TO_HORMONE	GO:0009725	20	0.178921	0.710022	0.875598	1.000000	1.000
229	GOBP_PURINE_CONTAINING_COMPOUND_METABOLIC_PROCESS	GO:0072521	5	0.301205	0.709762	0.823529	1.000000	1.000
230	GOBP_POSITIVE_REGULATION_OF_NUCLEOBASE_CONTAIN...	GO:0045935	28	0.156521	0.708527	0.928571	1.000000	1.000
231	GOBP_INTRACELLULAR_TRANSPORT	GO:0046907	11	0.205048	0.700831	0.896396	1.000000	1.000
232	GOBP_REGULATION_OF_IMMUNE_SYSTEM_PROCESS	GO:0002682	21	0.162734	0.691734	0.926471	1.000000	1.000
233	REACTOME_PTEN_REGULATION	R-RNO-6807070	5	0.292803	0.688775	0.849315	1.000000	1.000
234	GOBP_POSITIVE_REGULATION_OF_DNA_BINDING_TRANSC...	GO:0051091	10	0.220355	0.685448	0.902542	1.000000	1.000
235	GOBP_ENDOCYTOSIS	GO:0006897	8	0.238350	0.684700	0.884615	1.000000	1.000
236	GOBP_SEXUAL_REPRODUCTION	GO:0019953	10	0.216397	0.682760	0.911017	1.000000	1.000
237	GOBP_RESPONSE_TO_ENDOGENOUS_STIMULUS	GO:0009719	34	0.148491	0.682056	0.923077	1.000000	1.000
238	GOBP_RESPONSE_TO_HEAT	GO:0009408	7	0.259259	0.681657	0.855634	1.000000	1.000
239	GOBP_REGULATION_OF_ANION_TRANSMEMBRANE_TRANSPORT	GO:1903959	5	0.277308	0.678854	0.888217	1.000000	1.000
240	GOBP_REGULATION_OF_PHOSPHOLIPASE_C_ACTIVITY	GO:1900274	5	0.277108	0.672640	0.910891	1.000000	1.000
241	GOBP_POSITIVE_REGULATION_OF_BIOSYNTHETIC_PROCESS	GO:0009891	27	0.152424	0.670750	0.937500	1.000000	1.000
242	GOBP_POSITIVE_REGULATION_OF_LIPASE_ACTIVITY	GO:0060193	5	0.277108	0.661533	0.892857	1.000000	1.000
243	GOBP_RESPONSE_TO_KETONE	GO:1901654	7	0.246020	0.660559	0.930909	1.000000	1.000
244	GOBP_ACTIN_FILAMENT_BASED_PROCESS	GO:0030029	7	0.233478	0.655700	0.936090	1.000000	1.000
245	REACTOME_EXTRA_NUCLEAR_ESTROGEN_SIGNALING	R-RNO-9009391	7	0.234568	0.652697	0.922535	1.000000	1.000
246	GOBP_ANION_TRANSMEMBRANE_TRANSPORT	GO:0098656	7	0.246914	0.650432	0.913534	1.000000	1.000
247	GOBP_INOSITOL_LIPID_MEDIATED_SIGNALING	GO:0048017	8	0.225000	0.650068	0.937743	1.000000	1.000
248	GOBP_RESPONSE_TO_OXYGEN_CONTAINING_COMPOUND	GO:1901700	39	0.138749	0.649540	0.973684	1.000000	1.000
249	GOBP_TRANSMEMBRANE_RECEPTOR_PROTEIN_SERINE_THR...	GO:0007178	6	0.246771	0.646403	0.922819	1.000000	1.000
250	GOBP_SKIN_DEVELOPMENT	GO:0043588	5	0.261893	0.643392	0.946844	1.000000	1.000
251	HALLMARK_TNFA_SIGNALING_VIA_NFKB	M5890	14	0.175742	0.614026	0.938967	1.000000	1.000
252	GOBP_RESPIRATORY_SYSTEM_DEVELOPMENT	GO:0060541	6	0.231707	0.606982	0.949833	1.000000	1.000
253	GOBP_OSTEOCLAST_DIFFERENTIATION	GO:0030316	6	0.235081	0.595391	0.966777	1.000000	1.000
254	GOBP_REGULATION_OF_APOPTOTIC_SIGNALING_PATHWAY	GO:2001233	10	0.189368	0.587928	0.972763	1.000000	1.000
255	GOBP_NEGATIVE_REGULATION_OF_CELL_DIFFERENTIATION	GO:0045596	13	0.165930	0.586858	0.986607	1.000000	1.000

Continuation of Table **S13**

	NAME	ID	SIZE	ES	NES	NOM.p.val	FDR.q.val	FWER.p.val
256	GOBP_CELL_CYCLE	GO:0007049	21	0.145012	0.586369	0.994253	1.000000	1.000
257	GOBP_CELL_CELL_SIGNALING_BY_WNT	GO:0198738	7	0.208614	0.583451	0.982456	1.000000	1.000
258	GOBP_NEGATIVE_REGULATION_OF_DEVELOPMENTAL_PROCESS	GO:0051093	13	0.165930	0.583417	0.995305	1.000000	1.000
259	GOBP_POSITIVE_REGULATION_OF_TRANSCRIPTION_BY_R...	GO:0045944	24	0.140625	0.578241	1.000000	1.000000	1.000
260	GOBP_NEGATIVE_REGULATION_OF_APOPTOTIC_SIGNALIN...	GO:2001234	7	0.220223	0.577453	0.961538	1.000000	1.000
261	GOBP_REGULATION_OF_VASCULAR_ASSOCIATED_SMOOTH_...	GO:1904705	5	0.239620	0.573663	0.983051	1.000000	1.000
262	HALLMARK_PI3K_AKT_MTOR_SIGNALING	M5923	6	0.219512	0.559363	0.983819	1.000000	1.000
263	GOBP_MUSCLE_CELL_PROLIFERATION	GO:0033002	8	0.187500	0.557557	0.975207	1.000000	1.000
264	GOBP_LIPID_LOCALIZATION	GO:0010876	10	0.175669	0.550018	0.996032	1.000000	1.000
265	GOBP_POSITIVE_REGULATION_OF_ORGANELLE_ORGANIZA...	GO:0010638	10	0.173917	0.548175	0.982759	1.000000	1.000
266	GOBP_REGULATION_OF_WNT_SIGNALING_PATHWAY	GO:0030111	5	0.228027	0.546103	0.996678	1.000000	1.000
267	GOBP_SMOOTH_MUSCLE_CELL_PROLIFERATION	GO:0048659	6	0.209084	0.545528	0.980328	1.000000	1.000
268	GOBP_REGULATION_OF_ANION_TRANSPORT	GO:0044070	20	0.136901	0.544324	0.994048	0.998654	1.000
269	GOBP_POSITIVE_REGULATION_OF_SMOOTH_MUSCLE_CELL...	GO:0048661	5	0.223978	0.535401	0.993671	0.997532	1.000
270	GOBP_ACTIVATION_OF_PROTEIN_KINASE_ACTIVITY	GO:0032147	11	0.155590	0.516693	1.000000	0.997329	1.000
271	GOBP_INTRACELLULAR_PROTEIN_TRANSPORT	GO:0006886	9	0.164557	0.498538	0.988550	0.996400	1.000
272	GOBP_IMMUNE_EFFECTOR_PROCESS	GO:0002252	10	-0.134766	-0.345584	1.000000	0.999880	1.000
273	GOBP_CELLULAR_RESPONSE_TO_HORMONE_STIMULUS	GO:0032870	11	-0.150731	-0.392427	1.000000	1.000000	1.000
274	GOBP_REGULATION_OF_EXTRINSIC_APOPTOTIC_SIGNALI...	GO:2001236	6	-0.186094	-0.403957	0.998613	1.000000	1.000
275	REACTOME_TRANSCRIPTIONAL_REGULATION_OF_WHITE_A...	R-RNO-381340	6	-0.212786	-0.462341	0.997097	1.000000	1.000
276	GOBP_REGULATION_OF_CELLULAR_AMIDE_METABOLIC_PR...	GO:0034248	9	-0.189715	-0.467366	0.992147	1.000000	1.000
277	GOBP_REGULATION_OF_ORGANELLE_ORGANIZATION	GO:0033043	14	-0.168282	-0.467599	0.995000	1.000000	1.000
278	GOBP_CELLULAR_RESPONSE_TO_BIOTIC_STIMULUS	GO:0071216	8	-0.198581	-0.468590	0.993216	1.000000	1.000
279	REACTOME_ADAPTIVE_IMMUNE_SYSTEM	R-RNO-1280218	9	-0.191698	-0.471214	0.989348	1.000000	1.000
280	GOBP_RESPONSE_TO_INTERLEUKIN_1	GO:0070555	5	-0.229479	-0.473619	0.994444	1.000000	1.000
281	GOBP_CELLULAR_RESPONSE_TO_MOLECULE_OF_BACTERIA...	GO:0071219	8	-0.198581	-0.474250	0.990305	1.000000	1.000
282	GOBP_PEPTIDE_METABOLIC_PROCESS	GO:0006518	12	-0.183110	-0.486386	0.978205	1.000000	1.000
283	GOBP_REGULATION_OF_PROTEIN_LOCALIZATION	GO:0032880	18	-0.167688	-0.489118	0.983503	1.000000	1.000
284	GOBP_POSITIVE_REGULATION_OF_PROTEIN_SERINE_THR...	GO:0071902	9	-0.201980	-0.498164	0.985155	1.000000	1.000
285	GOBP_MONONUCLEAR_CELL_DIFFERENTIATION	GO:1903131	7	-0.224790	-0.505386	0.976257	1.000000	1.000
286	REACTOME_INTERLEUKIN_4_AND_INTERLEUKIN_13_SIGN...	R-RNO-6785807	8	-0.214935	-0.510316	0.983095	1.000000	1.000
287	GOBP_REGULATION_OF_PROTEIN_STABILITY	GO:0031647	5	-0.255184	-0.519954	0.990085	1.000000	1.000
288	GOBP_DNA_METABOLIC_PROCESS	GO:0006259	6	-0.239383	-0.526487	0.990028	1.000000	1.000
289	GOBP_CELL_CELL_JUNCTION_ASSEMBLY	GO:0007043	5	-0.256930	-0.526981	0.994092	1.000000	1.000
290	GOBP_CELLULAR_RESPONSE_TO_PEPTIDE	GO:1901653	13	-0.196494	-0.529136	0.975124	1.000000	1.000
291	GOBP_CARDIAC_MUSCLE_TISSUE_DEVELOPMENT	GO:0048738	6	-0.242804	-0.530971	0.982906	1.000000	1.000
292	GOBP_REGULATION_OF_NEURON_DIFFERENTIATION	GO:0045664	5	-0.251916	-0.532045	0.990085	1.000000	1.000
293	GOBP_CELL_CELL_JUNCTION_ORGANIZATION	GO:0045216	6	-0.239747	-0.533305	0.977077	1.000000	1.000
294	GOBP_HEART_DEVELOPMENT	GO:0007507	6	-0.242804	-0.535297	0.988506	1.000000	1.000
295	GOBP_REGULATION_OF_PROTEIN_SERINE_THREONINE_KI...	GO:0071900	12	-0.203842	-0.544512	0.959688	1.000000	1.000
296	GOBP_POSITIVE_REGULATION_OF_MULTICELLULAR_ORGA...	GO:0051240	32	-0.169276	-0.545413	0.965792	1.000000	1.000
297	GOBP_LEUKOCYTE_CELL_CELL_ADHESION	GO:0007159	6	-0.255921	-0.546946	0.974648	1.000000	1.000
298	REACTOME_RHO_GTPASE_EFFECTORS	R-RNO-195258	9	-0.222492	-0.548346	0.959239	1.000000	1.000

Continuation of Table **S13**

	NAME	ID	SIZE	ES	NES	NOM.p.val	FDR.q.val	FWER.p.val
299	GOBP_NEUROTROPHIN_SIGNALING_PATHWAY	GO:0038179	6	-0.252671	-0.552026	0.965116	1.000000	1.000
300	GOBP_NEUROTROPHIN_TRK_RECEPTOR_SIGNALING_PATHWAY	GO:0048011	6	-0.252671	-0.552258	0.965418	1.000000	1.000
301	REACTOME_SIGNALING_BY_RHO_GTPASES_MIRO_GTPASES...	R-RNO-9716542	9	-0.222492	-0.552469	0.967213	1.000000	1.000
302	GOBP_POSITIVE_REGULATION_OF_MYELOID_LEUKOCYTE_...	GO:0002763	6	-0.253089	-0.552531	0.964789	1.000000	1.000
303	GOBP_EMBRYO_DEVELOPMENT	GO:0009790	14	-0.203764	-0.557204	0.965736	1.000000	1.000
304	GOBP_REPRODUCTION	GO:0000003	18	-0.195183	-0.561298	0.949559	1.000000	1.000
305	GOBP_POSITIVE_REGULATION_OF_CELL_POPULATION_PR...	GO:0008284	21	-0.188474	-0.568377	0.957473	1.000000	1.000
306	GOBP_RESPONSE_TO_PEPTIDE_HORMONE	GO:0043434	13	-0.213034	-0.577342	0.947301	1.000000	1.000
307	GOBP_REGULATION_OF_CELLULAR_PROTEIN_LOCALIZATION	GO:1903827	12	-0.222388	-0.580566	0.945806	1.000000	1.000
308	REACTOME_SIGNALING_BY_NTRK2_TRKB	R-RNO-9006115	6	-0.269203	-0.581761	0.956091	1.000000	1.000
309	GOBP_EPITHELIUM_DEVELOPMENT	GO:0060429	15	-0.210201	-0.582500	0.950980	1.000000	1.000
310	GOBP_APOPTOTIC_MITOCHONDRIAL_CHANGES	GO:0008637	6	-0.268293	-0.583587	0.942623	1.000000	1.000
311	GOBP_CELLULAR_RESPONSE_TO_PEPTIDE_HORMONE_STIM...	GO:0071375	9	-0.235502	-0.584194	0.953020	1.000000	1.000
312	GOBP_CARBOHYDRATE_METABOLIC_PROCESS	GO:0005975	9	-0.231172	-0.584253	0.947226	1.000000	1.000
313	GOBP_CELLULAR_RESPONSE_TO_CADMIUM_ION	GO:0071276	5	-0.282311	-0.584815	0.959762	1.000000	1.000
314	GOBP_ORGANIC_ACID_TRANSPORT	GO:0015849	7	-0.258741	-0.586746	0.954930	1.000000	1.000
315	GOBP_REGULATION_OF_PROTEIN_PHOSPHORYLATION	GO:0001932	26	-0.190821	-0.588820	0.946449	1.000000	1.000
316	GOBP_RESPONSE_TO_ALCOHOL	GO:0097305	11	-0.228698	-0.590459	0.951031	1.000000	1.000
317	GOBP_TUBE_MORPHOGENESIS	GO:0035239	15	-0.212796	-0.594081	0.942118	1.000000	1.000
318	GOBP_MUSCLE_STRUCTURE_DEVELOPMENT	GO:0061061	16	-0.212337	-0.594303	0.931336	1.000000	1.000
319	GOBP_AMEBOIDAL_TYPE_CELL_MIGRATION	GO:0001667	11	-0.231325	-0.594378	0.924084	1.000000	1.000
320	GOBP_REGULATION_OF_CELL_CELL_ADHESION	GO:0022407	9	-0.242315	-0.599360	0.933333	1.000000	1.000
321	GOBP_POSITIVE_REGULATION_OF_MYELOID_CELL_DIFFE...	GO:0045639	7	-0.266941	-0.599893	0.942008	1.000000	1.000
322	GOBP_RESPONSE_TO_GROWTH_FACTOR	GO:0070848	18	-0.205535	-0.600109	0.944030	1.000000	1.000
323	GOBP_RESPONSE_TO_CADMIUM_ION	GO:0046686	6	-0.268293	-0.600749	0.951591	1.000000	1.000
324	GOBP_REGULATION_OF_BODY_FLUID_LEVELS	GO:0050878	11	-0.233867	-0.602086	0.916991	1.000000	1.000
325	GOBP_RECEPTOR_MEDIATED_ENDOCYTOSIS	GO:0006898	6	-0.273939	-0.602892	0.930736	1.000000	1.000
326	GOBP_RESPONSE_TO_INORGANIC_SUBSTANCE	GO:0010035	19	-0.208591	-0.606924	0.926380	1.000000	1.000
327	GOBP_POSITIVE_REGULATION_OF_CELL_PROJECTION_OR...	GO:0031346	10	-0.242937	-0.607580	0.912698	1.000000	1.000
328	GOBP_POSITIVE_REGULATION_OF_CELL_CELL_ADHESION	GO:0022409	5	-0.296853	-0.608212	0.943396	1.000000	1.000
329	REACTOME_PLATELET_ACTIVATION_SIGNALING_AND_AGG...	R-HSA-76002	7	-0.269794	-0.609963	0.926491	1.000000	1.000
330	GOBP_POSITIVE_REGULATION_OF_LEUKOCYTE_CELL_CEL...	GO:1903039	5	-0.296853	-0.612453	0.931983	1.000000	1.000
331	GOBP_REGULATION_OF_CELL_ADHESION	GO:0030155	13	-0.226880	-0.613021	0.930719	1.000000	1.000
332	GOBP_RECEPTOR_METABOLIC_PROCESS	GO:0043112	5	-0.301951	-0.614015	0.928994	1.000000	1.000
333	GOBP_CELLULAR_AMIDE_METABOLIC_PROCESS	GO:0043603	13	-0.227821	-0.617642	0.938383	1.000000	1.000
334	GOBP_RECEPTOR_INTERNALIZATION	GO:0031623	5	-0.301951	-0.620130	0.933921	1.000000	1.000
335	REACTOME_ESTROGEN_DEPENDENT_NUCLEAR_EVENTS_DOW...	R-RNO-9634638	5	-0.306896	-0.620840	0.940388	1.000000	1.000
336	GOBP_REGULATION_OF_SECRETION	GO:0051046	14	-0.226643	-0.622799	0.910112	1.000000	1.000
337	GOBP_REGULATION_OF_CYTOSKELETON_ORGANIZATION	GO:0051493	5	-0.304682	-0.624357	0.937213	1.000000	1.000
338	GOBP_APOPTOTIC_PROCESS	GO:0006915	35	-0.191656	-0.624722	0.929465	1.000000	1.000
339	GOBP_POSITIVE_REGULATION_OF_CELL_ADHESION	GO:0045785	7	-0.273998	-0.626934	0.916667	1.000000	1.000
340	GOBP_NEGATIVE_REGULATION_OF_BINDING	GO:0051100	6	-0.283987	-0.628967	0.925287	1.000000	1.000
341	REACTOME_DAG_AND_IP3_SIGNALING	R-RNO-1489509	8	-0.264178	-0.629021	0.908108	1.000000	1.000

Continuation of Table **S13**

	NAME	ID	SIZE	ES	NES	NOM.p.val	FDR.q.val	FWER.p.val
342	GOBP_POSITIVE_REGULATION_OF_HEMOPOIESIS	GO:1903708	7	-0.274618	-0.629421	0.933803	1.000000	1.000
343	GOBP_POSITIVE_REGULATION_OF_TRANSPORT	GO:0051050	26	-0.201958	-0.629931	0.924617	1.000000	1.000
344	GOBP_NEGATIVE_REGULATION_OF_PHOSPHORYLATION	GO:0042326	7	-0.269673	-0.630261	0.918056	1.000000	1.000
345	GOBP_REGULATION_OF_PROTEIN_KINASE_ACTIVITY	GO:0045859	22	-0.210358	-0.630322	0.924821	1.000000	1.000
346	GOBP_SENSORY_ORGAN_DEVELOPMENT	GO:0007423	8	-0.261451	-0.630941	0.910448	1.000000	1.000
347	GOBP_MYELOID_LEUKOCYTE_DIFFERENTIATION	GO:0002573	11	-0.240745	-0.633576	0.899619	1.000000	1.000
348	GOBP_REGULATION_OF_MYELOID_CELL_DIFFERENTIATION	GO:0045637	9	-0.260595	-0.634610	0.893793	1.000000	1.000
349	GOBP_RHYTHMIC_PROCESS	GO:0048511	15	-0.223861	-0.635339	0.909091	1.000000	1.000
350	GOBP_REGULATION_OF_TRANSFERASE_ACTIVITY	GO:0051338	22	-0.210358	-0.636603	0.892944	1.000000	1.000
351	REACTOME_INFECTIOUS_DISEASE	R-HSA-5663205	13	-0.233286	-0.637487	0.910141	1.000000	1.000
352	GOBP_GLAND_DEVELOPMENT	GO:0048732	7	-0.273537	-0.637524	0.904564	1.000000	1.000
353	GOBP_EMBRYONIC_ORGAN_DEVELOPMENT	GO:0048568	7	-0.278018	-0.637648	0.916782	1.000000	1.000
354	GOBP_EXTRINSIC_APOPTOTIC_SIGNALING_PATHWAY	GO:0097191	7	-0.275159	-0.639215	0.924016	1.000000	1.000
355	GOBP_CARBOXYLIC_ACID_TRANSPORT	GO:0046942	6	-0.297865	-0.640700	0.911444	1.000000	1.000
356	GOBP_AMYLOID_PRECURSOR_PROTEIN_CATABOLIC_PROCESS	GO:0042987	5	-0.313635	-0.641880	0.911047	1.000000	1.000
357	GOBP_APOPTOTIC_SIGNALING_PATHWAY	GO:0097190	15	-0.230736	-0.642381	0.895466	1.000000	1.000
358	GOBP_POSITIVE_REGULATION_OF_PROTEIN_KINASE_ACT...	GO:0045860	19	-0.219418	-0.642542	0.915357	1.000000	1.000
359	GOBP_MUSCLE_CONTRACTION	GO:0006936	5	-0.303538	-0.642748	0.931034	1.000000	1.000
360	GOBP_NEGATIVE_REGULATION_OF_PROTEOLYSIS	GO:0045861	8	-0.273370	-0.645770	0.900415	1.000000	1.000
361	GOBP_CELLULAR_RESPONSE_TO_EXTERNAL_STIMULUS	GO:0071496	10	-0.258692	-0.645791	0.904110	1.000000	1.000
362	GOBP_AMYLOID_BETA_METABOLIC_PROCESS	GO:0050435	5	-0.313635	-0.646333	0.926901	1.000000	1.000
363	REACTOME_HEMOSTASIS	R-RNO-109582	14	-0.239259	-0.646844	0.897277	1.000000	1.000
364	GOBP_ORGANIC_ANION_TRANSPORT	GO:0015711	6	-0.297865	-0.647119	0.906340	1.000000	1.000
365	GOBP_SIGNAL_RELEASE	GO:0023061	14	-0.234367	-0.648667	0.887626	1.000000	1.000
366	GOBP_ACTIVATION_OF_MAPK_ACTIVITY	GO:0000187	6	-0.298718	-0.648827	0.887784	1.000000	1.000
367	GOBP_POSITIVE_REGULATION_OF_MAP_KINASE_ACTIVITY	GO:0043406	6	-0.298718	-0.652095	0.890625	1.000000	1.000
368	GOBP_ERK1_AND_ERK2_CASCADE	GO:0070371	5	-0.315006	-0.652474	0.902299	1.000000	1.000
369	GOBP_AMYLOID_PRECURSOR_PROTEIN_METABOLIC_PROCESS	GO:0042982	5	-0.313635	-0.654672	0.919762	1.000000	1.000
370	REACTOME_SIGNALING_BY_RECEPTOR_TYROSINE_KINASES	R-RNO-9006934	26	-0.208386	-0.655216	0.913753	1.000000	1.000
371	GOBP_AMINO_ACID_TRANSPORT	GO:0006865	6	-0.297865	-0.656011	0.882192	1.000000	1.000
372	GOBP_NEGATIVE_REGULATION_OF_TRANSPORT	GO:0051051	13	-0.243735	-0.658247	0.891551	1.000000	1.000
373	GOBP_CELLULAR_COMPONENT_DISASSEMBLY	GO:0022411	6	-0.304863	-0.659169	0.897210	1.000000	1.000
374	HALLMARK_ALLOGRAFT_REJECTION	M5950	7	-0.287153	-0.662770	0.866571	1.000000	1.000
375	GOBP_INTRACELLULAR_RECEPTOR_SIGNALING_PATHWAY	GO:0030522	5	-0.319670	-0.663450	0.907378	1.000000	1.000
376	REACTOME_TRANSCRIPTIONAL_REGULATION_BY_TP53	R-RNO-3700989	6	-0.305988	-0.665919	0.889685	1.000000	1.000
377	GOBP_CIRCADIAN_RHYTHM	GO:0007623	13	-0.249158	-0.669401	0.883605	1.000000	1.000
378	GOBP_BLOOD_VESSEL_MORPHOGENESIS	GO:0048514	14	-0.242010	-0.672604	0.875792	1.000000	1.000
379	GOBP_MULTI_ORGANISM_PROCESS	GO:0051704	16	-0.238143	-0.676448	0.885856	1.000000	1.000
380	GOBP_EPITHELIAL_CELL_DIFFERENTIATION	GO:0030855	7	-0.294644	-0.676880	0.867332	1.000000	1.000
381	REACTOME_G_ALPHA_I_SIGNALLING_EVENTS	R-RNO-418594	18	-0.236661	-0.681996	0.867629	1.000000	1.000
382	GOBP_POSITIVE_REGULATION_OF_GENE_EXPRESSION	GO:0010628	16	-0.241829	-0.684043	0.875940	1.000000	1.000
383	GOBP_CHROMOSOME_ORGANIZATION	GO:0051276	6	-0.310535	-0.686243	0.882857	1.000000	1.000
384	GOBP_DEFENSE_RESPONSE_TO_OTHER_ORGANISM	GO:0098542	9	-0.282711	-0.689018	0.848728	1.000000	1.000

Continuation of Table **S13**

	NAME	ID	SIZE	ES	NES	NOM.p.val	FDR.q.val	FWER.p.val
385	REACTOME_INTERFERON_SIGNALING	R-RNO-913531	6	-0.316956	-0.694171	0.862937	1.000000	1.000
386	GOBP_LOCOMOTORY_BEHAVIOR	GO:0007626	7	-0.313139	-0.695674	0.856738	1.000000	1.000
387	GOBP_REGULATION_OF_MYELOID_LEUKOCYTE_DIFFERENT...	GO:0002761	8	-0.297615	-0.699103	0.840382	1.000000	1.000
388	REACTOME_SIGNALING_BY_NUCLEAR_RECEPTORS	R-RNO-9006931	8	-0.296105	-0.700032	0.847645	1.000000	1.000
389	REACTOME_ESR_MEDIATED_SIGNALING	R-RNO-8939211	8	-0.296105	-0.703331	0.850283	1.000000	1.000
390	REACTOME_SIGNALING_BY_GPCR	R-RNO-372790	22	-0.233039	-0.705576	0.849169	1.000000	1.000
391	GOBP_POSITIVE_REGULATION_OF_TRANSMEMBRANE_TRAN...	GO:0034764	7	-0.305749	-0.705832	0.833563	1.000000	1.000
392	GOBP_HOMEOSTASIS_OF_NUMBER_OF_CELLS	GO:0048872	5	-0.346055	-0.706306	0.882436	1.000000	1.000
393	GOBP_POSITIVE_REGULATION_OF_CELL_DEATH	GO:0010942	19	-0.237071	-0.706332	0.828125	1.000000	1.000
394	GOBP_FATTY_ACID_TRANSPORT	GO:0015908	6	-0.322993	-0.710433	0.835425	1.000000	1.000
395	GOBP_CELL_POPULATION_PROLIFERATION	GO:0008283	31	-0.217964	-0.710488	0.834313	1.000000	1.000
396	GOBP_POSITIVE_REGULATION_OF_CATION_TRANSMEMBRA...	GO:1904064	5	-0.345956	-0.710602	0.853727	1.000000	1.000
397	GOBP_NUCLEOBASE_CONTAINING_SMALL_MOLECULE_META...	GO:0055086	6	-0.327393	-0.714940	0.829167	1.000000	1.000
398	GOBP_POSITIVE_REGULATION_OF_PHOSPHORUS_METABOL...	GO:0010562	24	-0.231216	-0.716746	0.849882	1.000000	1.000
399	GOBP_REGULATION_OF_MUSCLE_CELL_DIFFERENTIATION	GO:0051147	6	-0.333756	-0.717497	0.842486	1.000000	1.000
400	GOBP_NEGATIVE_REGULATION_OF_CELL_DEVELOPMENT	GO:0010721	6	-0.330506	-0.719270	0.835674	1.000000	1.000
401	GOBP_REGULATION_OF_MULTICELLULAR_ORGANISMAL_DE...	GO:2000026	31	-0.227293	-0.722435	0.830080	1.000000	1.000
402	GOBP_POSITIVE_REGULATION_OF_HYDROLASE_ACTIVITY	GO:0051345	16	-0.255473	-0.724038	0.820859	1.000000	1.000
403	GOBP_CHROMATIN_ORGANIZATION	GO:0006325	5	-0.353719	-0.725093	0.815138	1.000000	1.000
404	GOBP_GROWTH	GO:0040007	14	-0.259608	-0.726812	0.822194	1.000000	1.000
405	GOBP_CELL_CELL_ADHESION	GO:0098609	14	-0.263346	-0.730242	0.824691	1.000000	1.000
406	GOBP_POSITIVE_REGULATION_OF_CELLULAR_COMPONENT...	GO:0051130	24	-0.238189	-0.730897	0.817647	1.000000	1.000
407	GOBP_REGULATION_OF_GTPASE_ACTIVITY	GO:0043087	5	-0.348025	-0.731125	0.839763	1.000000	1.000
408	GOBP_RESPONSE_TO_CYTOKINE	GO:0034097	23	-0.239776	-0.731706	0.825581	1.000000	1.000
409	GOBP_NEGATIVE_REGULATION_OF_TRANSFERASE_ACTIVITY	GO:0051348	5	-0.361759	-0.733221	0.833581	1.000000	1.000
410	GOBP_ACTIVATION_OF_IMMUNE_RESPONSE	GO:0002253	5	-0.354224	-0.733408	0.819648	1.000000	1.000
411	GOBP_NEGATIVE_REGULATION_OF_PEPTIDASE_ACTIVITY	GO:0010466	6	-0.340694	-0.734390	0.833568	1.000000	1.000
412	REACTOME_SIGNALING_BY_NTRKS	R-RNO-166520	18	-0.248057	-0.739366	0.813107	1.000000	1.000
413	GOBP_SECRETION	GO:0046903	24	-0.243405	-0.742188	0.820574	1.000000	1.000
414	GOBP_NEGATIVE_REGULATION_OF_MULTICELLULAR_ORGA...	GO:0051241	15	-0.268702	-0.747280	0.808132	1.000000	1.000
415	GOBP_NEGATIVE_REGULATION_OF_KINASE_ACTIVITY	GO:0033673	5	-0.361759	-0.749036	0.812772	1.000000	1.000
416	REACTOME_DEATH_RECEPTOR_SIGNALLING	R-RNO-73887	5	-0.359991	-0.750335	0.823034	1.000000	1.000
417	GOBP_LOCOMOTION	GO:0040011	31	-0.234846	-0.751881	0.796275	1.000000	1.000
418	GOBP_RESPONSE_TO_OXYGEN_LEVELS	GO:0070482	8	-0.314410	-0.754234	0.766074	1.000000	1.000
419	GOBP_POSITIVE_REGULATION_OF_MOLECULAR_FUNCTION	GO:0044093	38	-0.231138	-0.754824	0.828442	1.000000	1.000
420	GOBP_REGULATION_OF_HEMOPOIESIS	GO:1903706	10	-0.298854	-0.755286	0.783290	1.000000	1.000
421	GOBP_MUSCLE_ADAPTATION	GO:0043500	5	-0.368963	-0.755514	0.810198	1.000000	1.000
422	GOBP_DEVELOPMENTAL_GROWTH	GO:0048589	10	-0.300573	-0.756066	0.773263	1.000000	1.000
423	GOBP_NEGATIVE_REGULATION_OF_TRANSMEMBRANE_TRAN...	GO:0034763	6	-0.349388	-0.759644	0.776824	1.000000	1.000
424	GOBP_RESPONSE_TO_NERVE_GROWTH_FACTOR	GO:1990089	7	-0.329110	-0.760620	0.778976	1.000000	1.000
425	GOBP_REGULATION_OF_GROWTH	GO:0040008	13	-0.276903	-0.761201	0.789541	1.000000	1.000
426	GOBP_NEGATIVE_REGULATION_OF_NEURON_DEATH	GO:1901215	11	-0.295422	-0.763768	0.804491	1.000000	1.000
427	GOBP_REGULATION_OF_MUSCLE_ADAPTATION	GO:0043502	5	-0.368963	-0.764256	0.793353	1.000000	1.000

Continuation of Table **S13**

	NAME	ID	SIZE	ES	NES	NOM.p.val	FDR.q.val	FWER.p.val
428	GOBP_TUBE_DEVELOPMENT	GO:0035295	18	-0.254150	-0.765548	0.774074	1.000000	1.000
429	GOBP_POSITIVE_REGULATION_OF_TRANSFERASE_ACTIVITY	GO:0051347	20	-0.255747	-0.765790	0.796569	1.000000	1.000
430	GOBP_PROTEIN_PHOSPHORYLATION	GO:0006468	34	-0.237587	-0.767455	0.782066	1.000000	1.000
431	GOBP_REGULATION_OF_CELL_DEATH	GO:0010941	34	-0.232024	-0.767660	0.783784	1.000000	1.000
432	GOBP_CYTOKINE_MEDIATED_SIGNALING_PATHWAY	GO:0019221	16	-0.267380	-0.772549	0.771887	1.000000	1.000
433	GOBP_MITOCHONDRION_ORGANIZATION	GO:0007005	7	-0.340541	-0.774785	0.769126	1.000000	1.000
434	GOBP_NEGATIVE_REGULATION_OF_LOCOMOTION	GO:0040013	5	-0.388531	-0.775967	0.777937	1.000000	1.000
435	GOBP_REGULATION_OF_NEURON_PROJECTION_DEVELOPMENT	GO:0010975	16	-0.273408	-0.776840	0.781595	1.000000	1.000
436	GOBP_REGULATION_OF_HYDROLASE_ACTIVITY	GO:0051336	22	-0.259044	-0.779545	0.760241	1.000000	1.000
437	REACTOME_CA_DEPENDENT_EVENTS	R-RNO-111996	8	-0.328887	-0.782301	0.761714	1.000000	1.000
438	GOBP_REGULATION_OF_PROTEIN_LOCALIZATION_TO_CEL...	GO:1904375	6	-0.357154	-0.783576	0.736990	1.000000	1.000
439	GOBP_NEGATIVE_REGULATION_OF_CELL_DEATH	GO:0060548	22	-0.259547	-0.784844	0.774818	1.000000	1.000
440	GOBP_G_PROTEIN_COUPLED_RECEPTOR_SIGNALING_PATHWAY	GO:0007186	18	-0.272829	-0.787235	0.746377	1.000000	1.000
441	GOBP_CELL_GROWTH	GO:0016049	10	-0.303582	-0.788422	0.735799	1.000000	1.000
442	GOBP_TRANSMEMBRANE_RECEPTOR_PROTEIN_TYROSINE_K...	GO:0007169	19	-0.268968	-0.790665	0.745476	1.000000	1.000
443	GOBP_CELLULAR_RESPONSE_TO_EXTRACELLULAR_STIMULUS	GO:0031668	8	-0.333081	-0.791764	0.721622	1.000000	1.000
444	GOBP_NEGATIVE_REGULATION_OF_ION_TRANSPORT	GO:0043271	11	-0.306981	-0.794594	0.728232	1.000000	1.000
445	GOBP_RESPONSE_TO_EXTRACELLULAR_STIMULUS	GO:0009991	13	-0.295187	-0.799165	0.750000	1.000000	1.000
446	GOBP_CHEMICAL_HOMEOSTASIS	GO:0048878	23	-0.259722	-0.799658	0.736780	1.000000	1.000
447	GOBP_REGULATION_OF_LONG_TERM_SYNAPTIC_POTENTIA...	GO:1900271	6	-0.365355	-0.801993	0.727532	1.000000	1.000
448	GOBP_PROTEIN_CONTAINING_COMPLEX_SUBUNIT_ORGANI...	GO:0043933	14	-0.295101	-0.802851	0.742058	1.000000	1.000
449	GOBP_REGULATION_OF_MAP_KINASE_ACTIVITY	GO:0043405	9	-0.321961	-0.806139	0.719321	1.000000	1.000
450	GOBP_REGULATION_OF_ANATOMICAL_STRUCTURE_MORPHO...	GO:0022603	17	-0.285184	-0.808593	0.720960	1.000000	1.000
451	GOBP_RESPONSE_TO_METAL_ION	GO:0010038	15	-0.290037	-0.808813	0.724816	1.000000	1.000
452	GOBP_REGULATION_OF_DEVELOPMENTAL_GROWTH	GO:0048638	9	-0.321767	-0.809462	0.722444	1.000000	1.000
453	REACTOME_DISEASES_OF_SIGNAL_TRANSDUCTION_BY_GR...	R-HSA-5663202	12	-0.306526	-0.810784	0.738095	1.000000	1.000
454	GOBP_RESPONSE_TO_ETHANOL	GO:0045471	5	-0.393956	-0.811577	0.737991	1.000000	1.000
455	REACTOME_CELL_CYCLE	R-RNO-1640170	6	-0.369360	-0.812255	0.723435	1.000000	1.000
456	GOBP_SENSORY_PERCEPTION_OF_PAIN	GO:0019233	5	-0.397003	-0.812557	0.746627	1.000000	1.000
457	GOBP_CELLULAR_CARBOHYDRATE_METABOLIC_PROCESS	GO:0044262	5	-0.397722	-0.814037	0.734421	1.000000	1.000
458	GOBP_PERIPHERAL_NERVOUS_SYSTEM_DEVELOPMENT	GO:0007422	9	-0.325904	-0.816645	0.704453	1.000000	1.000
459	GOBP_PROTEIN_COMPLEX_OLIGOMERIZATION	GO:0051259	6	-0.377942	-0.818744	0.729844	1.000000	1.000
460	GOBP_RESPONSE_TO_ACID_CHEMICAL	GO:0001101	6	-0.379902	-0.820568	0.719149	1.000000	1.000
461	GOBP_CELLULAR_RESPONSE_TO_INORGANIC_SUBSTANCE	GO:0071241	11	-0.321863	-0.820611	0.727632	1.000000	1.000
462	GOBP_NEURON_APOPTOTIC_PROCESS	GO:0051402	12	-0.311935	-0.828918	0.684685	1.000000	1.000
463	GOBP_REGULATION_OF_LEUKOCYTE_DIFFERENTIATION	GO:1902105	9	-0.333481	-0.830614	0.679947	1.000000	1.000
464	GOBP_ANIMAL_ORGAN_MORPHOGENESIS	GO:0009887	12	-0.314526	-0.831447	0.701665	1.000000	1.000
465	GOBP_REGULATION_OF_MUSCLE_SYSTEM_PROCESS	GO:0090257	8	-0.356456	-0.837017	0.714479	1.000000	1.000
466	HALLMARK_UV_RESPONSE_UP	M5941	5	-0.403116	-0.837776	0.705968	1.000000	1.000
467	GOBP_DICARBOXYLIC_ACID_TRANSPORT	GO:0006835	5	-0.410381	-0.839722	0.680973	1.000000	1.000
468	GOBP_DENDRITE_DEVELOPMENT	GO:0016358	9	-0.337469	-0.842253	0.688568	1.000000	1.000
469	GOBP_ACIDIC_AMINO_ACID_TRANSPORT	GO:0015800	5	-0.410381	-0.846052	0.677892	1.000000	1.000
470	GOBP_NEGATIVE_REGULATION_OF_NERVOUS_SYSTEM_DEV...	GO:0051961	5	-0.408714	-0.847596	0.693114	1.000000	1.000

Continuation of Table **S13**

	NAME	ID	SIZE	ES	NES	NOM.p.val	FDR.q.val	FWER.p.val
471	HALLMARK_KRAS_SIGNALING_UP	M5953	6	-0.395184	-0.852253	0.669916	1.000000	1.000
472	GOBP_ADENYLATE_CYCLASE_MODULATING_G_PROTEIN_CO...	GO:0007188	11	-0.328870	-0.853764	0.667110	1.000000	1.000
473	GOBP_REGULATION_OF_INTRACELLULAR_SIGNAL_TRANSD...	GO:1902531	21	-0.285087	-0.853801	0.659498	1.000000	1.000
474	GOBP_ENZYME_LINKED_RECEPTOR_PROTEIN_SIGNALING_...	GO:0007167	24	-0.275870	-0.859539	0.659880	1.000000	1.000
475	GOBP_WOUND_HEALING	GO:0042060	13	-0.315768	-0.861277	0.630295	1.000000	1.000
476	GOBP_NEURON_DEATH	GO:0070997	18	-0.297878	-0.861295	0.665874	1.000000	1.000
477	REACTOME_CELL_CYCLE_MITOTIC	R-RNO-69278	5	-0.416731	-0.861429	0.672059	1.000000	1.000
478	GOBP_MONOCARBOXYLIC_ACID_TRANSPORT	GO:0015718	5	-0.410381	-0.861549	0.660237	1.000000	1.000
479	GOBP_POLYSACCHARIDE_METABOLIC_PROCESS	GO:0005976	5	-0.421869	-0.862686	0.689655	1.000000	1.000
480	GOBP_NEURON_DEVELOPMENT	GO:0048666	33	-0.261170	-0.864704	0.661346	1.000000	1.000
481	REACTOME_LEISHMANIA_INFECTION	R-HSA-9658195	10	-0.342506	-0.865841	0.643709	1.000000	1.000
482	GOBP_REGULATION_OF_ION_TRANSPORT	GO:0043269	36	-0.268419	-0.869786	0.652174	1.000000	1.000
483	GOBP_PROTEIN_LOCALIZATION_TO_PLASMA_MEMBRANE	GO:0072659	7	-0.375941	-0.871323	0.645658	1.000000	1.000
484	GOBP_PEPTIDYL_AMINO_ACID_MODIFICATION	GO:0018193	22	-0.289376	-0.871391	0.654126	1.000000	1.000
485	GOBP_CELLULAR_RESPONSE_TO_REACTIVE_OXYGEN_SPECIES	GO:0034614	7	-0.376777	-0.872004	0.650815	1.000000	1.000
486	GOBP_POSITIVE_REGULATION_OF_LONG_TERM_SYNAPTIC...	GO:1900273	5	-0.431655	-0.873566	0.654179	1.000000	1.000
487	GOBP_AXON_DEVELOPMENT	GO:0061564	18	-0.291397	-0.874527	0.642236	1.000000	1.000
488	GOBP_CELL_JUNCTION_ASSEMBLY	GO:0034329	8	-0.372419	-0.875121	0.642663	1.000000	1.000
489	REACTOME_G_ALPHA_Q_SIGNALLING_EVENTS	R-RNO-416476	6	-0.404208	-0.877174	0.654232	1.000000	1.000
490	GOBP_REGULATION_OF_CATABOLIC_PROCESS	GO:0009894	13	-0.321200	-0.878636	0.622930	1.000000	1.000
491	GOBP_COAGULATION	GO:0050817	8	-0.370974	-0.879443	0.629921	1.000000	1.000
492	GOBP_IMMUNE_RESPONSE_REGULATING_SIGNALING_PATHWAY	GO:000276	8	-0.372394	-0.881626	0.646492	1.000000	1.000
493	GOBP_CELLULAR_RESPONSE_TO_CHEMICAL_STRESS	GO:0062197	7	-0.376777	-0.882384	0.635117	1.000000	1.000
494	GOBP_NEGATIVE_REGULATION_OF_PROTEIN_METABOLIC_...	GO:0051248	20	-0.297064	-0.882813	0.649940	1.000000	1.000
495	GOBP_NEGATIVE_REGULATION_OF_NEURON_APOPTOTIC_P...	GO:0043524	8	-0.367909	-0.884537	0.636115	1.000000	1.000
496	GOBP_RESPONSE_TO_OXIDATIVE_STRESS	GO:0006979	8	-0.372121	-0.885925	0.620548	1.000000	1.000
497	GOBP_REGULATION_OF_LYMPHOCYTE_ACTIVATION	GO:0051249	5	-0.431955	-0.891683	0.660322	1.000000	1.000
498	GOBP_FC_RECEPTOR_SIGNALING_PATHWAY	GO:0038093	8	-0.372394	-0.892107	0.596974	1.000000	1.000
499	GOBP_RESPONSE_TO_REACTIVE_OXYGEN_SPECIES	GO:0000302	8	-0.372121	-0.895040	0.599469	1.000000	1.000
500	REACTOME_CELLULAR_RESPONSES_TO_EXTERNAL_STIMULI	R-RNO-8953897	12	-0.344571	-0.896131	0.618090	1.000000	1.000
501	REACTOME_SIGNALING_BY_INTERLEUKINS	R-RNO-449147	15	-0.315050	-0.897001	0.606516	1.000000	1.000
502	HALLMARK_HYPOXIA	M5891	5	-0.437716	-0.897564	0.624460	1.000000	1.000
503	REACTOME_CYTOKINE_SIGNALING_IN_IMMUNE_SYSTEM	R-RNO-1280215	21	-0.296233	-0.898123	0.595849	1.000000	1.000
504	GOBP_REGULATION_OF_SYNAPTIC_TRANSMISSION_GLUTA...	GO:0051966	12	-0.338009	-0.900185	0.600249	1.000000	1.000
505	GOBP_CELL_PROJECTION_ORGANIZATION	GO:0030030	34	-0.275215	-0.904872	0.610660	1.000000	1.000
506	GOBP_POSITIVE_REGULATION_OF_GROWTH	GO:0045927	9	-0.369929	-0.906332	0.603518	1.000000	1.000
507	GOBP_POSITIVE_REGULATION_OF_PROTEIN_METABOLIC_...	GO:0051247	28	-0.285922	-0.909707	0.603995	1.000000	1.000
508	GOBP_MEMBRANE_ORGANIZATION	GO:0061024	12	-0.340580	-0.912819	0.583867	1.000000	1.000
509	GOBP_MUSCLE_CELL_DIFFERENTIATION	GO:0042692	10	-0.359719	-0.914700	0.580563	1.000000	1.000
510	REACTOME_INNATE_IMMUNE_SYSTEM	R-RNO-168249	16	-0.324015	-0.916022	0.588235	1.000000	1.000
511	GOBP_CELLULAR_RESPONSE_TO_STARVATION	GO:0009267	5	-0.445614	-0.917221	0.606607	1.000000	1.000
512	GOBP_POSITIVE_REGULATION_OF_AXONOGENESIS	GO:0050772	5	-0.442360	-0.918098	0.589080	1.000000	1.000
513	GOBP_FC_EPSILON_RECEPTOR_SIGNALING_PATHWAY	GO:0038095	7	-0.404406	-0.919451	0.610335	1.000000	1.000

Continuation of Table **S13**

	NAME	ID	SIZE	ES	NES	NOM.p.val	FDR.q.val	FWER.p.val
514	GOBP_RESPONSE_TO_STARVATION	GO:0042594	5	-0.445614	-0.920998	0.588674	1.000000	1.000
515	GOBP_POSITIVE_REGULATION_OF_CATALYTIC_ACTIVITY	GO:0043085	30	-0.288219	-0.921532	0.581158	1.000000	1.000
516	GOBP_REGULATION_OF_CELLULAR_LOCALIZATION	GO:0060341	17	-0.319435	-0.922197	0.585784	1.000000	1.000
517	REACTOME_FC_EPSILON_RECEPTOR_FCERI_SIGNALING	R-RNO-2454202	7	-0.404406	-0.923777	0.576056	1.000000	1.000
518	GOBP_BIOLOGICAL_PROCESS_INVOLVED_IN_SYMBIOTIC_...	GO:004440	11	-0.358994	-0.928067	0.572005	1.000000	1.000
519	GOBP_DEVELOPMENTAL_CELL_GROWTH	GO:0048588	5	-0.449049	-0.930773	0.568513	1.000000	1.000
520	GOBP_REGULATION_OF_CELL_DIFFERENTIATION	GO:0045595	36	-0.287615	-0.932209	0.588432	1.000000	1.000
521	GOBP_REGULATION_OF_CYSTEINE_TYPE_ENDOPEPTIDASE...	GO:2000116	9	-0.376841	-0.932613	0.548139	1.000000	1.000
522	GOBP_POSITIVE_REGULATION_OF_DEVELOPMENTAL_GROWTH	GO:0048639	8	-0.394318	-0.936242	0.572414	1.000000	1.000
523	GOBP_REGULATION_OF_ENDOCYTOSIS	GO:0030100	6	-0.431767	-0.941092	0.541787	1.000000	1.000
524	REACTOME_GPCR_LIGAND_BINDING	R-RNO-500792	8	-0.398844	-0.943625	0.549724	1.000000	1.000
525	GOBP_REGULATION_OF_RECEPTOR_MEDIATED_ENDOCYTOSIS	GO:0048259	5	-0.457062	-0.943758	0.562942	1.000000	1.000
526	GOBP_NERVE_DEVELOPMENT	GO:0021675	9	-0.383326	-0.944485	0.549587	1.000000	1.000
527	GOBP_ENDOMEMBRANE_SYSTEM_ORGANIZATION	GO:0010256	6	-0.433298	-0.945618	0.565460	1.000000	1.000
528	GOBP_REGULATION_OF_CELL_DEVELOPMENT	GO:0060284	15	-0.332473	-0.947404	0.543532	1.000000	1.000
529	GOBP_MACROMOLECULE_CATABOLIC_PROCESS	GO:0009057	12	-0.359068	-0.949170	0.539052	1.000000	1.000
530	GOBP_POSITIVE_REGULATION_OF_CELL_GROWTH	GO:0030307	6	-0.443117	-0.954214	0.532578	1.000000	1.000
531	GOBP_REGULATION_OF_CIRCADIAN_RHYTHM	GO:0042752	7	-0.422127	-0.954991	0.546593	1.000000	1.000
532	GOBP_GLIAL_CELL_DIFFERENTIATION	GO:0010001	10	-0.379435	-0.955364	0.541724	1.000000	1.000
533	GOBP_BIOLOGICAL_ADHESION	GO:0022610	19	-0.322720	-0.956929	0.519573	1.000000	1.000
534	GOBP_POSITIVE_REGULATION_OF_PROTEOLYSIS	GO:0045862	8	-0.400377	-0.960244	0.512855	1.000000	1.000
535	GOBP_RESPONSE_TO_WOUNDING	GO:0009611	15	-0.347811	-0.963425	0.517767	1.000000	1.000
536	GOBP_CELLULAR_MACROMOLECULE_LOCALIZATION	GO:0070727	21	-0.318958	-0.963815	0.523077	1.000000	1.000
537	GOBP_REGULATION_OF_BINDING	GO:0051098	11	-0.375072	-0.970026	0.501323	1.000000	1.000
538	GOBP_NEURON_PROJECTION_GUIDANCE	GO:0097485	9	-0.394243	-0.970742	0.529491	1.000000	1.000
539	GOBP_REGULATION_OF_SYNAPSE_STRUCTURE_OR_ACTIVITY	GO:0050803	11	-0.375352	-0.972750	0.504551	1.000000	1.000
540	GOBP_NEGATIVE_REGULATION_OF_INTRACELLULAR_SIGN...	GO:1902532	7	-0.422518	-0.974353	0.504132	1.000000	1.000
541	GOBP_REGULATION_OF_MAPK_CASCADE	GO:0043408	13	-0.360615	-0.975328	0.492366	1.000000	1.000
542	GOBP_REGULATION_OF_NERVOUS_SYSTEM_DEVELOPMENT	GO:0051960	15	-0.347593	-0.976842	0.508642	1.000000	1.000
543	GOBP_INNATE_IMMUNE_RESPONSE	GO:0045087	7	-0.430172	-0.977855	0.509642	1.000000	1.000
544	GOBP_NEURON_DIFFERENTIATION	GO:0030182	36	-0.296160	-0.978842	0.516968	1.000000	1.000
545	GOBP_POSITIVE_REGULATION_OF_MAPK_CASCADE	GO:0043410	9	-0.400377	-0.980287	0.503285	1.000000	1.000
546	GOBP_GENERATION_OF_PRECURSOR_METABOLITES_AND_E...	GO:0006091	5	-0.478377	-0.984062	0.509299	1.000000	1.000
547	GOBP_REGULATION_OF_CELL_PROJECTION_ORGANIZATION	GO:0031344	20	-0.335810	-0.989216	0.497549	1.000000	1.000
548	GOBP_MULTICELLULAR_ORGANISMAL_RESPONSE_TO_STRESS	GO:0033555	5	-0.484750	-0.992234	0.469208	1.000000	1.000
549	GOBP_TAXIS	GO:0042330	14	-0.359202	-0.995285	0.477387	1.000000	1.000
550	GOBP_LIPID_EXPORT_FROM_CELL	GO:0140353	5	-0.488924	-1.002163	0.482014	1.000000	1.000
551	GOBP_CELLULAR_COMPONENT_MORPHOGENESIS	GO:0032989	24	-0.321972	-1.003070	0.464576	1.000000	1.000
552	GOBP_CELL_PART_MORPHOGENESIS	GO:0032990	24	-0.321972	-1.005885	0.468160	1.000000	1.000
553	GOBP_MUSCLE_SYSTEM_PROCESS	GO:0003012	9	-0.395253	-1.007126	0.457143	1.000000	1.000
554	REACTOME_CIRCADIAN_CLOCK	R-HSA-400253	5	-0.492923	-1.007630	0.476401	1.000000	1.000
555	GOBP_POSITIVE_REGULATION_OF_CYSTEINE_TYPE_ENDO...	GO:2001056	7	-0.439456	-1.009106	0.492517	1.000000	1.000
556	GOBP_CELL_MORPHOGENESIS	GO:0000902	24	-0.321972	-1.010261	0.465476	1.000000	1.000

Continuation of Table **S13**

	NAME	ID	SIZE	ES	NES	NOM.p.val	FDR.q.val	FWER.p.val
557	GOBP_EMBRYONIC_MORPHOGENESIS	GO:0048598	6	-0.455930	-1.010781	0.452211	1.000000	1.000
558	HALLMARK_APOPTOSIS	M5902	6	-0.467373	-1.011378	0.464849	1.000000	1.000
559	GOBP_REGULATION_OF_TRANSPORT	GO:0051049	44	-0.308809	-1.016790	0.448889	1.000000	1.000
560	GOBP_REGULATION_OF_POSTSYNAPSE_ORGANIZATION	GO:0099175	6	-0.460119	-1.017007	0.456311	1.000000	1.000
561	GOBP_SMALL_GTPASE_MEDIATED_SIGNAL_TRANSDUCTION	GO:0007264	8	-0.436041	-1.018066	0.469880	1.000000	1.000
562	GOBP_REGULATION_OF_NEUROGENESIS	GO:0050767	14	-0.373634	-1.020277	0.443742	1.000000	1.000
563	GOBP_DENDRITIC_SPINE_MORPHOGENESIS	GO:0060997	5	-0.497036	-1.021181	0.453102	1.000000	1.000
564	GOBP_CELL_MORPHOGENESIS_INVOLVED_IN_DIFFERENTI...	GO:0000904	21	-0.346012	-1.024785	0.452607	1.000000	1.000
565	REACTOME_TRAFFICKING_OF_GLUR2_CONTAINING_AMPA_...	R-RNO-416993	8	-0.429855	-1.025037	0.470509	1.000000	1.000
566	GOBP_COGNITION	GO:0050890	28	-0.322019	-1.025425	0.438279	1.000000	1.000
567	GOBP_POSITIVE_REGULATION_OF_PEPTIDASE_ACTIVITY	GO:0010952	7	-0.439456	-1.025468	0.449376	1.000000	1.000
568	GOBP_CELL_MORPHOGENESIS_INVOLVED_IN_NEURON_DIF...	GO:0048667	21	-0.346012	-1.025485	0.438107	1.000000	1.000
569	GOBP_DENDRITIC_SPINE_DEVELOPMENT	GO:0060996	6	-0.469296	-1.028298	0.422438	1.000000	1.000
570	GOBP_DENDRITE_MORPHOGENESIS	GO:0048813	6	-0.470610	-1.029367	0.443526	1.000000	1.000
571	GOBP_CELLULAR_HOMEOSTASIS	GO:0019725	18	-0.357835	-1.029437	0.438788	1.000000	1.000
572	GOBP_NEUROGENESIS	GO:0022008	40	-0.313680	-1.036672	0.415919	1.000000	1.000
573	GOBP_REGULATION_OF_PROTEOLYSIS	GO:0030162	12	-0.391617	-1.037270	0.443456	1.000000	1.000
574	GOBP_REGULATION_OF_PROTEIN_LOCALIZATION_TO_MEM...	GO:1905475	7	-0.446443	-1.039867	0.428571	1.000000	1.000
575	GOBP_POSITIVE_REGULATION_OF_PEPTIDYL_TYROSINE_...	GO:0050731	8	-0.439800	-1.041256	0.409029	1.000000	1.000
576	GOBP_MEMBRANE_PROTEIN_PROTEOLYSIS	GO:0033619	6	-0.472809	-1.041374	0.425626	1.000000	1.000
577	GOBP_PROTEOLYSIS	GO:0006508	17	-0.357102	-1.046264	0.415648	1.000000	1.000
578	GOBP_REGULATION_OF_TRANSMEMBRANE_TRANSPORT	GO:0034762	23	-0.340909	-1.051141	0.413998	1.000000	1.000
579	GOBP_BEHAVIOR	GO:0007610	32	-0.328696	-1.053493	0.420118	1.000000	1.000
580	GOBP_POSITIVE_REGULATION_OF_NERVOUS_SYSTEM_DEV...	GO:0051962	12	-0.406712	-1.056167	0.404732	1.000000	1.000
581	GOBP_G_PROTEIN_COUPLED_GLUTAMATE_RECEPTOR_SIGN...	GO:0007216	8	-0.454723	-1.060345	0.395561	1.000000	1.000
582	REACTOME_ONCOGENIC_MAPK_SIGNALING	R-HSA-6802957	5	-0.515237	-1.063095	0.400281	1.000000	1.000
583	GOBP_STRIATED_MUSCLE_CELL_DIFFERENTIATION	GO:0051146	9	-0.439544	-1.063622	0.378729	1.000000	1.000
584	GOBP_ENDOCRINE_SYSTEM_DEVELOPMENT	GO:0035270	6	-0.483186	-1.064178	0.406685	1.000000	1.000
585	GOBP_REGULATION_OF_NEURONAL_SYNAPTIC_PLASTICITY	GO:0048168	7	-0.477437	-1.072065	0.391365	1.000000	1.000
586	GOBP_NUCLEAR_TRANSPORT	GO:0051169	5	-0.512201	-1.073673	0.387697	1.000000	1.000
587	GOBP_POSITIVE_REGULATION_OF_NEUROGENESIS	GO:0050769	12	-0.406712	-1.074596	0.375979	1.000000	1.000
588	GOBP_CELLULAR_COMPONENT_MAINTENANCE	GO:0043954	5	-0.522502	-1.080046	0.374456	1.000000	1.000
589	GOBP_POSITIVE_REGULATION_OF_CELL_DEVELOPMENT	GO:0010720	12	-0.406712	-1.080283	0.371681	1.000000	1.000
590	GOBP_NEGATIVE_REGULATION_OF_CELLULAR_COMPONENT...	GO:0051129	11	-0.411096	-1.082345	0.374194	1.000000	1.000
591	GOBP_POSITIVE_REGULATION_OF_CELLULAR_COMPONENT...	GO:0044089	9	-0.436868	-1.083633	0.386179	1.000000	1.000
592	REACTOME_MAPK_TARGETS_NUCLEAR_EVENTS_MEDIATED_...	R-HSA-450282	5	-0.530120	-1.084974	0.364943	1.000000	1.000
593	GOBP_AGING	GO:0007568	13	-0.403173	-1.087372	0.368758	1.000000	1.000
594	GOBP_POSITIVE_REGULATION_OF_INTRACELLULAR_SIGN...	GO:1902533	14	-0.395490	-1.095511	0.340452	1.000000	1.000
595	GOBP_REGULATION_OF_PEPTIDASE_ACTIVITY	GO:0052547	10	-0.441739	-1.101688	0.356307	1.000000	1.000
596	GOBP_SENSORY_PERCEPTION	GO:0007600	9	-0.452981	-1.111169	0.349933	1.000000	1.000
597	GOBP_ION_HOMEOSTASIS	GO:0050801	16	-0.390991	-1.112541	0.341404	1.000000	1.000
598	GOBP_SYNAPTIC_TRANSMISSION_GLUTAMATERGIC	GO:0035249	13	-0.410925	-1.112704	0.341837	1.000000	1.000
599	GOBP_REGULATION_OF_PEPTIDYL_TYROSINE_PHOSPHORY...	GO:0050730	9	-0.462756	-1.122932	0.316778	1.000000	1.000

Continuation of Table **S13**

	NAME	ID	SIZE	ES	NES	NOM.p.val	FDR.q.val	FWER.p.val
600	REACTOME_POST_TRANSLATIONAL_PROTEIN_MODIFICATION	R-RNO-597592	7	-0.496854	-1.123051	0.334752	1.000000	1.000
601	GOBP_ADENYLATE_CYCLASE_INHIBITING_G_PROTEIN_CO...	GO:0007193	9	-0.453729	-1.126028	0.335142	1.000000	1.000
602	GOBP_REGULATION_OF_SIGNALING_RECEPTOR_ACTIVITY	GO:0010469	14	-0.416229	-1.126545	0.320203	1.000000	1.000
603	GOBP_CENTRAL_NERVOUS_SYSTEM_DEVELOPMENT	GO:0007417	26	-0.361100	-1.127087	0.304551	1.000000	1.000
604	GOBP_POSITIVE_REGULATION_OF_GLIOGENESIS	GO:0014015	5	-0.552516	-1.127870	0.306428	1.000000	1.000
605	GOBP_REGULATION_OF_METAL_ION_TRANSPORT	GO:0010959	6	-0.511182	-1.129126	0.315049	1.000000	1.000
606	GOBP_SECOND_MESSENGER_MEDIATED_SIGNALING	GO:0007196	16	-0.401719	-1.132291	0.327500	1.000000	1.000
607	REACTOME_NERVOUS_SYSTEM_DEVELOPMENT	R-RNO-9675108	15	-0.401931	-1.138058	0.299007	1.000000	1.000
608	GOBP_POSITIVE_REGULATION_OF_BINDING	GO:0051099	6	-0.515832	-1.138606	0.314706	1.000000	1.000
609	REACTOME_CELLULAR_SENESCENCE	R-RNO-2559583	6	-0.524390	-1.139635	0.333809	1.000000	1.000
610	GOBP_REGULATION_OF_GLIOGENESIS	GO:0014013	5	-0.552516	-1.144229	0.285075	1.000000	1.000
611	REACTOME_SENESCENCE_ASSOCIATED_SECRETORY_PHENO...	R-RNO-2559582	6	-0.524390	-1.146157	0.281831	1.000000	1.000
612	GOBP_NEGATIVE_REGULATION_OF_CATABOLIC_PROCESS	GO:0009895	8	-0.481073	-1.149288	0.306383	1.000000	1.000
613	REACTOME_TRAFFICKING_OF_AMPA_RECEPTORS	R-RNO-399719	11	-0.451166	-1.149474	0.295699	1.000000	1.000
614	REACTOME_INTERLEUKIN_17_SIGNALING	R-RNO-448424	6	-0.524390	-1.152525	0.290323	1.000000	1.000
615	GOBP_GLIOGENESIS	GO:0042063	12	-0.446941	-1.160225	0.269821	1.000000	1.000
616	GOBP_REGULATION_OF_CELLULAR_COMPONENT_BIOGENESIS	GO:0044087	10	-0.459190	-1.161174	0.296296	1.000000	1.000
617	GOBP_DEVELOPMENTAL_MATURATION	GO:0021700	5	-0.578313	-1.170570	0.284875	1.000000	1.000
618	GOBP_RAS_PROTEIN_SIGNAL_TRANSDUCTION	GO:0007265	5	-0.568525	-1.174403	0.265625	1.000000	1.000
619	GOBP_FOREBRAIN_DEVELOPMENT	GO:0030900	9	-0.478198	-1.175461	0.277778	1.000000	1.000
620	GOBP_REGULATION_OF_SYSTEM_PROCESS	GO:0044057	18	-0.414025	-1.176605	0.273515	1.000000	1.000
621	REACTOME_CLASS_C_3_METABOTROPIC_GLUTAMATE_PHER...	R-RNO-420499	7	-0.527205	-1.181464	0.258250	1.000000	1.000
622	GOBP_ORGANONITROGEN_COMPOUND_CATABOLIC_PROCESS	GO:1901565	9	-0.484991	-1.188145	0.265823	1.000000	1.000
623	GOBP_REGULATION_OF_PROTEIN_TYROSINE_KINASE_ACT...	GO:0061097	5	-0.578313	-1.191033	0.244666	1.000000	1.000
624	REACTOME_MYD88_INDEPENDENT_TLR4_CASCADE	R-RNO-166166	8	-0.500000	-1.193399	0.251001	1.000000	1.000
625	GOBP_POSITIVE_REGULATION_OF_PROTEIN_TYROSINE_K...	GO:0061098	5	-0.578313	-1.193493	0.228814	1.000000	1.000
626	GOBP_PROTEIN_LOCALIZATION_TO_CELL_PERIPHERY	GO:1990778	12	-0.454596	-1.197825	0.246173	1.000000	1.000
627	REACTOME_DEVELOPMENTAL_BIOLOGY	R-RNO-1266738	23	-0.392781	-1.198501	0.243013	1.000000	1.000
628	REACTOME_TOLL_LIKE_RECEPTOR_9_TLR9_CASCADE	R-RNO-168138	8	-0.500000	-1.199451	0.242991	1.000000	1.000
629	GOBP_METAL_ION_HOMEOSTASIS	GO:0055065	14	-0.438007	-1.200006	0.241730	1.000000	1.000
630	GOBP_CELLULAR_ION_HOMEOSTASIS	GO:0006873	14	-0.430252	-1.200130	0.234414	1.000000	1.000
631	REACTOME_TOLL_LIKE_RECEPTOR_CASCADES	R-RNO-168898	8	-0.500000	-1.200143	0.261224	1.000000	1.000
632	GOBP_POSITIVE_REGULATION_OF_CELL_DIFFERENTIATION	GO:0045597	27	-0.383984	-1.201938	0.238754	1.000000	1.000
633	GOBP_TELENCEPHALON_DEVELOPMENT	GO:0021537	6	-0.547686	-1.202760	0.232295	1.000000	1.000
634	REACTOME_TOLL_LIKE_RECEPTOR_TLR1_TLR2_CASCADE	R-RNO-168179	8	-0.500000	-1.206976	0.209809	1.000000	1.000
635	GOBP_DIVALENT_INORGANIC_CATION_HOMEOSTASIS	GO:0072507	13	-0.454779	-1.210221	0.230089	1.000000	1.000
636	GOBP_REGULATION_OF_NERVOUS_SYSTEM_PROCESS	GO:0031644	8	-0.525000	-1.217776	0.216714	1.000000	1.000
637	GOBP_REGULATION_OF_CYTOSOLIC_CALCIUM_ION_CONCE...	GO:0051480	13	-0.454779	-1.218932	0.224439	1.000000	1.000
638	GOBP_MYOTUBE_DIFFERENTIATION	GO:0014902	5	-0.600574	-1.220344	0.191176	1.000000	1.000
639	GOBP_ASSOCIATIVE_LEARNING	GO:0008306	7	-0.534403	-1.222552	0.209538	1.000000	1.000
640	GOBP_RESPONSE_TO_ABIOTIC_STIMULUS	GO:0009628	27	-0.390779	-1.223687	0.220554	1.000000	1.000
641	GOBP_RESPONSE_TO_UV	GO:0009411	5	-0.593740	-1.232479	0.194074	1.000000	1.000
642	GOBP_POSITIVE_REGULATION_OF_SIGNALING	GO:0023056	34	-0.381787	-1.237294	0.201835	1.000000	1.000

Continuation of Table **S13**

	NAME	ID	SIZE	ES	NES	NOM.p.val	FDR.q.val	FWER.p.val
643	GOBP_POSITIVE_REGULATION_OF_SYNAPTIC_TRANSMISSION	GO:0050806	15	-0.446310	-1.243693	0.197270	1.000000	1.000
644	GOBP_REGULATION_OF_NEUROTRANSMITTER_RECEPTOR_A...	GO:0099601	13	-0.464089	-1.244266	0.198980	1.000000	1.000
645	GOBP_NERVOUS_SYSTEM_PROCESS	GO:0050877	38	-0.383299	-1.246184	0.170843	1.000000	1.000
646	GOBP_REGULATION_OF_CELL_SIZE	GO:0008361	5	-0.616342	-1.249027	0.193303	1.000000	1.000
647	GOBP_RESPONSE_TO_DRUG	GO:0042493	11	-0.483898	-1.254085	0.183448	1.000000	1.000
648	GOBP_PEPTIDYL_TYROSINE_MODIFICATION	GO:0018212	10	-0.491220	-1.260758	0.195187	1.000000	1.000
649	GOBP_CATION_TRANSPORT	GO:0006812	25	-0.405444	-1.262656	0.174323	1.000000	1.000
650	GOBP_POSITIVE_REGULATION_OF_DEVELOPMENTAL_PROCESS	GO:0051094	32	-0.390252	-1.269152	0.186421	1.000000	1.000
651	REACTOME_EPH_EPHRIN_SIGNALING	R-RNO-2682334	6	-0.594419	-1.278423	0.161572	1.000000	1.000
652	GOBP_REGULATION_OF_TRANS_SYNAPTIC_SIGNALING	GO:0099177	40	-0.391732	-1.280379	0.162556	1.000000	1.000
653	GOBP_PROTEIN_LOCALIZATION_TO_CELL_JUNCTION	GO:1902414	6	-0.592862	-1.283175	0.165242	1.000000	1.000
654	GOBP_REGULATION_OF_CATION_TRANSMEMBRANE_TRANSPORT	GO:1904062	16	-0.445148	-1.284100	0.167920	1.000000	1.000
655	GOBP_EPHRIN_RECEPTOR_SIGNALING_PATHWAY	GO:0048013	5	-0.625294	-1.284540	0.142433	1.000000	1.000
656	GOBP_RESPONSE_TO_MECHANICAL_STIMULUS	GO:0009612	6	-0.598820	-1.286244	0.154728	1.000000	1.000
657	GOBP_CALCIUM_MEDIATED_SIGNALING	GO:0019722	10	-0.513248	-1.289832	0.170077	1.000000	1.000
658	GOBP_REGULATION_OF_AXONOGENESIS	GO:0050770	7	-0.551931	-1.291324	0.155705	1.000000	1.000
659	GOBP_CELL_SURFACE_RECEPTOR_SIGNALING_PATHWAY_I...	GO:1905114	15	-0.461716	-1.291678	0.141800	1.000000	1.000
660	GOBP_REGULATION_OF_MEMBRANE_POTENTIAL	GO:0042391	17	-0.452883	-1.296827	0.168130	1.000000	1.000
661	GOBP_SYNAPSE_ORGANIZATION	GO:0050808	17	-0.453899	-1.298745	0.161491	1.000000	1.000
662	GOBP_REGULATION_OF_ANATOMICAL_STRUCTURE_SIZE	GO:0090066	10	-0.509417	-1.301084	0.156836	1.000000	1.000
663	GOBP_PROTEIN_LOCALIZATION_TO_MEMBRANE	GO:0072657	14	-0.478424	-1.314003	0.129111	1.000000	1.000
664	GOBP_PROTEIN_CATABOLIC_PROCESS	GO:0030163	8	-0.554996	-1.324773	0.151070	1.000000	1.000
665	GOBP_MAPK_CASCADE	GO:0000165	18	-0.449601	-1.325786	0.135135	1.000000	1.000
666	GOBP_NEURON_PROJECTION_ORGANIZATION	GO:0106027	8	-0.558306	-1.327725	0.139785	1.000000	1.000
667	GOBP_CELL_JUNCTION_ORGANIZATION	GO:0034330	19	-0.444941	-1.330638	0.133333	1.000000	1.000
668	GOBP_ADENYLATE_CYCLASE_INHIBITING_G_PROTEIN_CO...	GO:0007193	5	-0.646547	-1.334125	0.113128	1.000000	1.000
669	GOBP_PROTEIN_LOCALIZATION_TO_POSTSYNAPSE	GO:0062237	5	-0.644871	-1.338003	0.110951	1.000000	1.000
670	GOBP_TRANSMEMBRANE_TRANSPORT	GO:0055085	27	-0.423912	-1.346033	0.116359	1.000000	1.000
671	GOBP_PROTEIN_LOCALIZATION_TO_SYNAPSE	GO:0035418	5	-0.644871	-1.347787	0.104634	1.000000	1.000
672	GOBP_REGULATION_OF_CELLULAR_COMPONENT_SIZE	GO:0032535	7	-0.591471	-1.351600	0.114168	1.000000	1.000
673	GOBP_RESPONSE_TO_RADIATION	GO:0009314	13	-0.497857	-1.353789	0.116751	1.000000	1.000
674	HALLMARK_EPITHELIAL_MESENCHYMAL_TRANSITION	M5930	5	-0.644960	-1.357589	0.098240	1.000000	1.000
675	GOBP_REGULATION_OF_SYNAPTIC_PLASTICITY	GO:0048167	21	-0.457507	-1.362874	0.110843	1.000000	1.000
676	GOBP_HEAD_DEVELOPMENT	GO:0060322	22	-0.452719	-1.367406	0.091667	1.000000	1.000
677	GOBP_REGULATION_OF_CATION_CHANNEL_ACTIVITY	GO:2001257	14	-0.500998	-1.371778	0.115854	1.000000	1.000
678	REACTOME_L1CAM_INTERACTIONS	R-RNO-373760	5	-0.677759	-1.394307	0.071736	0.940606	1.000
679	GOBP_REGULATION_OF_PROTEIN_CATABOLIC_PROCESS	GO:0042176	7	-0.621936	-1.407330	0.067511	0.886952	1.000
680	REACTOME_RNA_POLYMERASE_II_TRANSCRIPTION	R-RNO-73857	20	-0.473125	-1.408702	0.083933	0.902358	1.000
681	GOBP_CELL_CELL_SIGNALING	GO:0007267	56	-0.425812	-1.412175	0.081839	0.904939	1.000
682	REACTOME_EXTRACELLULAR_MATRIX_ORGANIZATION	R-RNO-1474244	5	-0.690200	-1.419354	0.070100	0.887782	1.000
683	GOBP_REGULATION_OF_TRANSPORTER_ACTIVITY	GO:0032409	17	-0.497382	-1.421711	0.073983	0.898037	1.000
684	GOBP_RESPONSE_TO_LIGHT_STIMULUS	GO:0009416	11	-0.541968	-1.425348	0.083979	0.902774	1.000
685	GOBP_NEGATIVE_REGULATION_OF_CELL_PROJECTION_OR...	GO:0031345	5	-0.691376	-1.430457	0.057863	0.897435	1.000

Continuation of Table **S13**

	NAME	ID	SIZE	ES	NES	NOM.p.val	FDR.q.val	FWER.p.val
686	GOBP_REGULATION_OF_POSTSYNAPTIC_MEMBRANE_POTEN...	GO:0060078	14	-0.530373	-1.442613	0.058752	0.849494	1.000
687	GOBP_LONG_TERM_SYNAPTIC_POTENTIATION	GO:0060291	12	-0.543404	-1.448000	0.067639	0.846073	1.000
688	GOBP_CATION_TRANSMEMBRANE_TRANSPORT	GO:0098655	18	-0.511180	-1.448633	0.063246	0.869335	1.000
689	GOBP_POSTSYNAPSE_ORGANIZATION	GO:0099173	10	-0.585837	-1.474440	0.049096	0.747872	1.000
690	GOBP_REGULATION_OF_NMDA_RECEPTOR_ACTIVITY	GO:2000310	10	-0.584558	-1.492679	0.040682	0.679055	1.000
691	REACTOME_PROTEIN_PROTEIN_INTERACTIONS_AT_SYNAPSES	R-RNO-6794362	12	-0.563934	-1.502185	0.038911	0.655744	1.000
692	GOBP_CHEMICAL_SYNAPTIC_TRANSMISSION_POSTSYNAPTIC	GO:0099565	9	-0.627280	-1.508233	0.034993	0.647702	1.000
693	GOBP_SYNAPTIC_SIGNALING	GO:0099536	47	-0.464335	-1.525744	0.041989	0.586923	1.000
694	REACTOME_SYNAPTIC_ADHESION_LIKE_MOLECULES	R-RNO-8849932	9	-0.628279	-1.530006	0.038564	0.589731	1.000
695	GOBP_CALCIUM_ION_TRANSPORT	GO:0006816	11	-0.602221	-1.551766	0.019815	0.516079	1.000
696	GOBP_IONOTROPIC_GLUTAMATE_RECEPTOR_SIGNALING_P...	GO:0035235	9	-0.632724	-1.552929	0.021769	0.534304	1.000
697	GOBP_CALCIUM_ION_TRANSMEMBRANE_TRANSPORT	GO:0070588	7	-0.694495	-1.568769	0.022191	0.487994	1.000
698	GOBP_EXCITATORY_CHEMICAL_SYNAPTIC_TRANSMISSION	GO:0098976	5	-0.759036	-1.569967	0.013043	0.506471	1.000
699	GOBP_METAL_ION_TRANSPORT	GO:0030001	11	-0.602221	-1.570468	0.025572	0.529265	1.000
700	REACTOME_NEUREXINS_AND_NEUROLIGINS	R-RNO-6794361	9	-0.650585	-1.572629	0.019417	0.547126	1.000
701	REACTOME_TRANSMISSION_ACROSS_CHEMICAL_SYNAPSES	R-RNO-112315	23	-0.523620	-1.576184	0.024882	0.560184	1.000
702	GOBP_CALCIUM_ION_TRANSPORT_INTO_CYTOSOL	GO:0060402	7	-0.694495	-1.577596	0.016925	0.584850	1.000
703	GOBP_CYTOSOLIC_CALCIUM_ION_TRANSPORT	GO:0060401	7	-0.694495	-1.582640	0.021614	0.596277	1.000
704	GOBP_LEARNING	GO:0007612	13	-0.578894	-1.593336	0.026515	0.579069	1.000
705	REACTOME_RAS_ACTIVATION_UPON_CA2_INFLUX_THROUG...	R-HSA-442982	6	-0.732040	-1.609258	0.012676	0.536089	1.000
706	REACTOME_CREB1_PHOSPHORYLATION_THROUGH_NMDA_RE...	R-RNO-442742	8	-0.686836	-1.613974	0.011189	0.554203	1.000
707	REACTOME_LONG_TERM_POTENTIATION	R-HSA-9620244	10	-0.643855	-1.640353	0.018494	0.468409	1.000
708	REACTOME_NEURONAL_SYSTEM	R-RNO-112316	26	-0.525283	-1.645742	0.016336	0.484998	0.997
709	GOBP_GLUTAMATE_RECEPTOR_SIGNALING_PATHWAY	GO:0007215	17	-0.580634	-1.668039	0.010000	0.428699	0.995
710	REACTOME_NEUROTRANSMITTER_RECEPTORS_AND_POSTSY...	R-RNO-112314	22	-0.554506	-1.686760	0.006002	0.394026	0.981
711	REACTOME_TRANSCRIPTIONAL_REGULATION_BY_MECP2	R-RNO-8986944	7	-0.758617	-1.706046	0.009749	0.356542	0.960
712	REACTOME_ACTIVATION_OF_NMDA_RECEPTORS_AND_POST...	R-RNO-442755	16	-0.605770	-1.712185	0.008883	0.382714	0.953
713	GOBP_NEGATIVE_REGULATION_OF_PROTEIN_CATABOLIC_...	GO:0042177	5	-0.832030	-1.712568	0.005848	0.444565	0.952
714	REACTOME_MAPK_FAMILY_SIGNALING_CASCADES	R-RNO-5683057	12	-0.666907	-1.760786	0.005141	0.306394	0.839
715	REACTOME_ASSEMBLY_AND_CELL_SURFACE_PRESENTATIO...	R-RNO-9609736	8	-0.758077	-1.772551	0.001376	0.335735	0.798
716	REACTOME_UNBLOCKING_OF_NMDA_RECEPTORS_GLUTAMAT...	R-RNO-438066	12	-0.664723	-1.772840	0.003916	0.444888	0.797
717	GOBP_INORGANIC_ION_TRANSMEMBRANE_TRANSPORT	GO:0098660	9	-0.726479	-1.794163	0.000000	0.520146	0.716
718	REACTOME_NEGATIVE_REGULATION_OF_NMDA_RECEPTOR_...	R-HSA-961732	8	-0.758077	-1.820415	0.005420	0.749830	0.596

End of Table

Supplementary Table S14: BG early profile (1 DPL peak) GSEA results.

Begin of Table **S14**

	NAME	ID	SIZE	ES	NES	NOM.p.val	FDR.q.val	FWER.p.val
0	GOBP_REGIONALIZATION	GO:0003002	6	0.795552	2.033180	0.000000	0.374573	0.380
1	GOBP_PATTERN_SPECIFICATION_PROCESS	GO:0007389	6	0.795552	1.997639	0.000000	0.254615	0.471
2	GOBP_REGULATION_OF_NEUROTRANSMITTER_RECEPTOR_A...	GO:0099601	13	0.556547	1.873164	0.000000	0.476056	0.826
3	GOBP_MEMORY	GO:0007613	13	0.560787	1.822978	0.006780	0.505561	0.916
4	GOBP_POSITIVE_REGULATION_OF_NEURON_PROJECTION_...	GO:0010976	5	0.776988	1.820985	0.014793	0.410562	0.923
5	REACTOME_SIGNALING_BY_NTRK2_TRKB	R-RNO-9006115	6	0.697617	1.806138	0.017391	0.381213	0.945
6	GOBP_CATION_TRANSPORT	GO:0006812	25	0.440951	1.797742	0.004425	0.343217	0.952
7	GOBP_REGULATION_OF_CATION_CHANNEL_ACTIVITY	GO:2001257	14	0.517522	1.784610	0.003546	0.327694	0.965
8	GOBP_REGULATION_OF_CATION_TRANSMEMBRANE_TRANSPORT	GO:1904062	16	0.493500	1.756228	0.007117	0.353100	0.985
9	GOBP_REGULATION_OF_NMDA_RECEPTOR_ACTIVITY	GO:2000310	10	0.567724	1.740969	0.009901	0.347295	0.990
10	GOBP_LONG_TERM_MEMORY	GO:0007616	6	0.650395	1.617003	0.056548	0.625724	1.000
11	GOBP_REGULATION_OF_SIGNALING_RECEPTOR_ACTIVITY	GO:0010469	14	0.472601	1.602173	0.045752	0.618898	1.000
12	GOBP_RESPONSE_TO_NERVE_GROWTH_FACTOR	GO:1990089	7	0.589754	1.595750	0.050746	0.589619	1.000
13	GOBP_CATION_TRANSMEMBRANE_TRANSPORT	GO:0098655	18	0.446054	1.588654	0.034188	0.566391	1.000
14	GOBP_REGULATION_OF_CELL_PROJECTION_ORGANIZATION	GO:0031344	20	0.418111	1.583646	0.022901	0.541412	1.000
15	GOBP_DEVELOPMENTAL_CELL_GROWTH	GO:0048588	5	0.683493	1.551274	0.060150	0.588788	1.000
16	GOBP_REGULATION_OF_PHOSPHOLIPASE_ACTIVITY	GO:0010517	6	0.609756	1.549827	0.051672	0.558037	1.000
17	GOBP_REGULATION_OF_MEMBRANE_POTENTIAL	GO:0042391	17	0.436748	1.549519	0.011450	0.527735	1.000
18	GOBP_POSITIVE_REGULATION_OF_CELL_PROJECTION_OR...	GO:0031346	10	0.509386	1.535480	0.055363	0.533615	1.000
19	GOBP_REGULATION_OF_POSTSYNAPTIC_MEMBRANE_POTEN...	GO:0060078	14	0.453953	1.516340	0.062044	0.554028	1.000
20	GOBP_NEGATIVE_REGULATION_OF_NEURON_APOPTOTIC_P...	GO:0043524	8	0.522920	1.497864	0.082822	0.572828	1.000
21	GOBP_REGULATION_OF_LIPASE_ACTIVITY	GO:0060191	6	0.609756	1.495094	0.072674	0.553403	1.000
22	REACTOME_UNBLOCKING_OF_NMDA_RECEPTORS_GLUTAMAT...	R-RNO-438066	12	0.464444	1.490851	0.063694	0.539180	1.000
23	REACTOME_PROTEIN_PROTEIN_INTERACTIONS_AT_SYNAPSES	R-RNO-6794362	12	0.459785	1.480677	0.078231	0.539346	1.000
24	GOBP_BEHAVIOR	GO:0007610	32	0.335125	1.448239	0.042857	0.595613	1.000
25	GOBP_REGULATION_OF_PHOSPHOLIPASE_C_ACTIVITY	GO:1900274	5	0.617969	1.423842	0.118980	0.634326	1.000
26	GOBP_POSITIVE_REGULATION_OF_LIPASE_ACTIVITY	GO:0060193	5	0.617969	1.423718	0.119444	0.611125	1.000
27	GOBP_IONOTROPIC_GLUTAMATE_RECEPTOR_SIGNALING_P...	GO:0035235	9	0.474758	1.412292	0.085366	0.617777	1.000
28	GOBP_PERIPHERAL_NERVOUS_SYSTEM_DEVELOPMENT	GO:0007422	9	0.495494	1.396987	0.102719	0.634366	1.000
29	GOBP_NEURON_PROJECTION_ORGANIZATION	GO:0106027	8	0.492915	1.389429	0.103030	0.632393	1.000
30	GOBP_REGULATION_OF_TRANSPORTER_ACTIVITY	GO:0032409	17	0.382473	1.386217	0.064777	0.619928	1.000
31	REACTOME_SYNAPTIC_ADHESION_LIKE_MOLECULES	R-RNO-8849932	9	0.480187	1.384744	0.119632	0.604215	1.000
32	GOBP_NEUROTROPHIN_SIGNALING_PATHWAY	GO:0038179	6	0.538930	1.380173	0.116071	0.596165	1.000
33	GOBP_RECEPTOR_METABOLIC_PROCESS	GO:0043112	5	0.595182	1.379532	0.126582	0.580186	1.000
34	GOBP_POSITIVE_REGULATION_OF_PROTEIN_TYROSINE_K...	GO:0061098	5	0.578313	1.368779	0.117493	0.590172	1.000
35	GOBP_REGULATION_OF_SYNAPSE_STRUCTURE_OR_ACTIVITY	GO:0050803	11	0.435506	1.367590	0.134948	0.576764	1.000
36	GOBP_RECEPTOR_INTERNALIZATION	GO:0031623	5	0.595182	1.367022	0.111979	0.562235	1.000
37	GOBP_REGULATION_OF_PEPTIDYL_SERINE_PHOSPHORYLA...	GO:0033135	8	0.485175	1.366892	0.089286	0.547740	1.000
38	GOBP_SENSORY_PERCEPTION_OF_PAIN	GO:0019233	5	0.573679	1.339308	0.137755	0.597092	1.000
39	GOBP_REGULATION_OF_NEURON_PROJECTION_DEVELOPMENT	GO:0010975	16	0.370848	1.337354	0.116981	0.586709	1.000
40	GOBP_SENSORY_PERCEPTION	GO:0007600	9	0.455696	1.333043	0.128028	0.581937	1.000

Continuation of Table **S14**

	NAME	ID	SIZE	ES	NES	NOM.p.val	FDR.q.val	FWER.p.val
41	GOBP_REGULATION_OF_NEURON_DIFFERENTIATION	GO:0045664	5	0.580022	1.330974	0.151596	0.572494	1.000
42	REACTOME_ACTIVATION_OF_NMDA_RECEPTORS_AND_POST...	R-RNO-442755	16	0.365657	1.330818	0.122744	0.559507	1.000
43	GOBP_REGULATION_OF_PROTEIN_TYROSINE_KINASE_ACT...	GO:0061097	5	0.578313	1.328704	0.122016	0.551767	1.000
44	GOBP_LOCOMOTORY_BEHAVIOR	GO:0007626	7	0.494400	1.323526	0.146199	0.550826	1.000
45	GOBP_LONG_TERM_SYNAPTIC_DEPRESSION	GO:0060292	5	0.563514	1.320130	0.142857	0.546482	1.000
46	GOBP_NEUROTROPHIN_TRK_RECEPTOR_SIGNALING_PATHWAY	GO:0048011	6	0.538930	1.319499	0.126471	0.536203	1.000
47	GOBP_REGULATION_OF_POSTSYNAPSE_ORGANIZATION	GO:0099175	6	0.545098	1.317964	0.144847	0.528018	1.000
48	GOBP_NUCLEAR_TRANSPORT	GO:0051169	5	0.554217	1.313552	0.144022	0.525729	1.000
49	GOBP_POSITIVE_REGULATION_OF_PEPTIDYL_SERINE_PH...	GO:0033138	8	0.485175	1.307605	0.151057	0.526956	1.000
50	REACTOME_LONG_TERM_POTENTIATION	R-HSA-9620244	10	0.426928	1.295503	0.141892	0.542745	1.000
51	REACTOME_SIGNALING_BY_NTRKS	R-RNO-166520	18	0.336214	1.273990	0.140288	0.577693	1.000
52	GOBP_RESPONSE_TO_ETHANOL	GO:0045471	5	0.538866	1.272913	0.182306	0.569154	1.000
53	GOBP_COGNITION	GO:0050890	28	0.307498	1.256976	0.123348	0.593327	1.000
54	GOBP_DIVALENT_INORGANIC_CATION_HOMEOSTASIS	GO:0072507	13	0.360895	1.247641	0.153846	0.604446	1.000
55	GOBP_CELLULAR_ION_HOMEOSTASIS	GO:0006873	14	0.355879	1.232970	0.163121	0.628052	1.000
56	GOBP_MUSCLE_CELL_DEVELOPMENT	GO:0055001	5	0.542169	1.224567	0.192612	0.637110	1.000
57	GOBP_DEVELOPMENTAL_MATURATION	GO:0021700	5	0.518072	1.205502	0.241935	0.673235	1.000
58	GOBP_NERVOUS_SYSTEM_PROCESS	GO:0050877	38	0.270208	1.204162	0.211957	0.664721	1.000
59	GOBP_REGULATION_OF_TRANS_SYNAPTIC_SIGNALING	GO:0099177	40	0.264572	1.199557	0.196532	0.665228	1.000
60	GOBP_POSITIVE_REGULATION_OF_CELL_GROWTH	GO:0030307	6	0.487905	1.197148	0.238095	0.660755	1.000
61	GOBP_REGULATION_OF_CYTOSOLIC_CALCIUM_ION_CONCE...	GO:0051480	13	0.360895	1.193457	0.240678	0.659144	1.000
62	GOBP_REGULATION_OF_CELL_SIZE	GO:0008361	5	0.512112	1.183697	0.248021	0.672083	1.000
63	GOBP_ORGANIC_ACID_TRANSPORT	GO:0015849	7	0.444444	1.173247	0.236923	0.686786	1.000
64	REACTOME_RAS_ACTIVATION_UPON_CA2_INFLUX_THROUG...	R-HSA-442982	6	0.477844	1.172527	0.224377	0.677850	1.000
65	GOBP_POSITIVE_REGULATION_OF_HYDROLASE_ACTIVITY	GO:0051345	16	0.327144	1.167971	0.217082	0.679120	1.000
66	GOBP_GLUTAMATE_RECEPTOR_SIGNALING_PATHWAY	GO:0007215	17	0.315300	1.164302	0.195572	0.678056	1.000
67	GOBP_SYNAPTIC_SIGNALING	GO:0099536	47	0.255185	1.150386	0.262032	0.703371	1.000
68	GOBP_POSITIVE_REGULATION_OF_SYNAPTIC_TRANSMISSION	GO:0050806	15	0.329092	1.138220	0.278985	0.724065	1.000
69	GOBP_REGULATION_OF_DEVELOPMENTAL_GROWTH	GO:0048638	9	0.384908	1.132101	0.293919	0.729817	1.000
70	GOBP_ORGANIC_ANION_TRANSPORT	GO:0015711	6	0.439024	1.102555	0.308333	0.797133	1.000
71	GOBP_AMINO_ACID_TRANSPORT	GO:0006865	6	0.439024	1.101150	0.305476	0.789939	1.000
72	GOBP_CELL_PROJECTION_ORGANIZATION	GO:0030030	34	0.253033	1.097412	0.305000	0.789395	1.000
73	GOBP_REGULATION_OF_NEURONAL_SYNAPTIC_PLASTICITY	GO:0048168	7	0.407407	1.096331	0.293255	0.781742	1.000
74	HALLMARK_KRAS_SIGNALING_UP	M5953	6	0.440225	1.094307	0.327635	0.776511	1.000
75	GOBP_CARBOXYLIC_ACID_TRANSPORT	GO:0046942	6	0.439024	1.090292	0.336158	0.777030	1.000
76	GOBP_DEVELOPMENTAL_GROWTH	GO:0048589	10	0.359641	1.086984	0.326797	0.774748	1.000
77	REACTOME_CREB1_PHOSPHORYLATION_THROUGH_NMDA_RE...	R-RNO-442742	8	0.385350	1.080723	0.354232	0.780394	1.000
78	GOBP_FATTY_ACID_TRANSPORT	GO:0015908	6	0.439024	1.076681	0.314103	0.780704	1.000
79	GOBP_NEGATIVE_REGULATION_OF_SYNAPTIC_TRANSMISSION	GO:0050805	6	0.430383	1.076296	0.356948	0.771920	1.000
80	REACTOME_TRANSCRIPTIONAL_REGULATION_BY_MECP2	R-RNO-8986944	7	0.415662	1.074945	0.342342	0.765758	1.000
81	GOBP_POSITIVE_REGULATION_OF_DEVELOPMENTAL_GROWTH	GO:0048639	8	0.391848	1.074624	0.345345	0.757375	1.000
82	GOBP_NEGATIVE_REGULATION_OF_NEURON_DEATH	GO:1901215	11	0.350828	1.071086	0.335526	0.757026	1.000
83	GOBP_REGULATION_OF_ION_TRANSPORT	GO:0043269	36	0.241413	1.069443	0.376190	0.751694	1.000

Continuation of Table **S14**

	NAME	ID	SIZE	ES	NES	NOM.p.val	FDR.q.val	FWER.p.val
84	GOBP_METAL_ION_HOMEOSTASIS	GO:0055065	14	0.311357	1.069291	0.342373	0.743143	1.000
85	GOBP_CHEMICAL_SYNAPTIC_TRANSMISSION_POSTSYNAPTIC	GO:0099565	9	0.368604	1.069141	0.344322	0.734907	1.000
86	GOBP_REGULATION_OF_NERVOUS_SYSTEM_PROCESS	GO:0031644	8	0.370539	1.065195	0.364458	0.735540	1.000
87	GOBP_CALCIUM_ION_TRANSPORT	GO:0006816	11	0.326203	1.057797	0.363636	0.744813	1.000
88	GOBP_REGULATION_OF_TRANSMEMBRANE_TRANSPORT	GO:0034762	23	0.272029	1.055202	0.404255	0.742345	1.000
89	GOBP_ION_HOMEOSTASIS	GO:0050801	16	0.291622	1.040765	0.412451	0.768703	1.000
90	GOBP_NERVE_DEVELOPMENT	GO:0021675	9	0.360256	1.038763	0.366154	0.764999	1.000
91	GOBP_LONG_TERM_SYNAPTIC_POTENTIATION	GO:0060291	12	0.316767	1.038123	0.382550	0.758268	1.000
92	GOBP_TRANSMEMBRANE_TRANSPORT	GO:0055085	27	0.255763	1.037034	0.401914	0.752712	1.000
93	GOBP_CELL_CELL_SIGNALING	GO:0007267	56	0.228038	1.032624	0.461538	0.755162	1.000
94	GOBP_MONOCARBOXYLIC_ACID_TRANSPORT	GO:0015718	5	0.433735	1.023149	0.398844	0.769170	1.000
95	GOBP_REGULATION_OF_SYNAPTIC_PLASTICITY	GO:0048167	21	0.259426	1.006589	0.420863	0.801096	1.000
96	GOBP_CELLULAR_COMPONENT_MAINTENANCE	GO:0043954	5	0.432154	1.005735	0.398396	0.795378	1.000
97	GOBP_DICARBOXYLIC_ACID_TRANSPORT	GO:0006835	5	0.433735	1.005512	0.422977	0.787825	1.000
98	GOBP_ACIDIC_AMINO_ACID_TRANSPORT	GO:0015800	5	0.433735	1.005445	0.427778	0.780020	1.000
99	GOBP_METAL_ION_TRANSPORT	GO:0030001	11	0.326203	0.996820	0.425532	0.792439	1.000
100	GOBP_SYNAPSE_ORGANIZATION	GO:0050808	17	0.276010	0.990080	0.460076	0.801108	1.000
101	GOBP_STRIATED_MUSCLE_CELL_DIFFERENTIATION	GO:0051146	9	0.341772	0.989157	0.428571	0.795412	1.000
102	GOBP_RESPONSE_TO_MONOSACCHARIDE	GO:0034284	5	0.409639	0.973442	0.441096	0.824746	1.000
103	GOBP_POSITIVE_REGULATION_OF_TRANSMEMBRANE_TRAN...	GO:0034764	7	0.364674	0.973000	0.429429	0.817939	1.000
104	GOBP_POSITIVE_REGULATION_OF_AXONOGENESIS	GO:0050772	5	0.421498	0.963786	0.497175	0.832291	1.000
105	GOBP_REGULATION_OF_CELLULAR_COMPONENT_SIZE	GO:0032535	7	0.361878	0.960598	0.461538	0.832096	1.000
106	REACTOME_NEGATIVE_REGULATION_OF_NMDA_RECEPTOR_...	R-HSA-961732	8	0.340328	0.959167	0.486567	0.827729	1.000
107	GOBP_POSITIVE_REGULATION_OF_GROWTH	GO:0045927	9	0.317078	0.958318	0.485981	0.822102	1.000
108	REACTOME_NEURONAL_SYSTEM	R-RNO-112316	26	0.237341	0.958216	0.515021	0.814918	1.000
109	HALLMARK_PI3K_AKT_MTOR_SIGNALING	M5923	6	0.376224	0.953599	0.497297	0.818473	1.000
110	GOBP_POSITIVE_REGULATION_OF_TRANSFERASE_ACTIVITY	GO:0051347	20	0.250000	0.951431	0.514925	0.816169	1.000
111	REACTOME_ASSEMBLY_AND_CELL_SURFACE_PRESENTATIO...	R-RNO-9609736	8	0.340328	0.941593	0.530675	0.830928	1.000
112	GOBP_RESPONSE_TO_CARBOHYDRATE	GO:0009743	5	0.409639	0.940428	0.464986	0.826241	1.000
113	GOBP_POSITIVE_REGULATION_OF_PROTEIN_KINASE_ACT...	GO:0045860	19	0.246377	0.937982	0.569853	0.824475	1.000
114	GOBP_POSITIVE_REGULATION_OF_BINDING	GO:0051099	6	0.381388	0.935662	0.510204	0.822769	1.000
115	GOBP_POSITIVE_REGULATION_OF_PHOSPHORUS_METABOL...	GO:0010562	24	0.234375	0.935318	0.549783	0.816495	1.000
116	REACTOME_TRAFFICKING_OF_AMPA_RECEPTORS	R-RNO-399719	11	0.302856	0.934727	0.529032	0.810934	1.000
117	GOBP_PEPTIDYL_SERINE_MODIFICATION	GO:0018209	12	0.289577	0.927907	0.539519	0.819104	1.000
118	GOBP_POSITIVE_REGULATION_OF_CATALYTIC_ACTIVITY	GO:0043085	30	0.216781	0.915958	0.561947	0.840105	1.000
119	GOBP_MATERNAL_PROCESS_INVOLVED_IN_FEMALE_PREGN...	GO:0060135	5	0.394862	0.908630	0.530120	0.849826	1.000
120	GOBP_POSITIVE_REGULATION_OF_PROTEIN_PHOSPHORYL...	GO:0001934	23	0.230769	0.904863	0.584746	0.851065	1.000
121	GOBP_POSTSYNAPSE_ORGANIZATION	GO:0099173	10	0.301895	0.904744	0.590476	0.844335	1.000
122	GOBP_RECEPTOR_MEDIATED_ENDOCYTOSIS	GO:0006898	6	0.363253	0.904330	0.582386	0.838338	1.000
123	GOBP_POSITIVE_REGULATION_OF_PROTEIN_MODIFICATI...	GO:0031401	23	0.230769	0.903878	0.597744	0.832607	1.000
124	GOBP_REGULATION_OF_HORMONE_LEVELS	GO:0010817	12	0.280914	0.902472	0.586441	0.829060	1.000
125	GOBP_CARBOHYDRATE_DERIVATIVE_METABOLIC_PROCESS	GO:1901135	7	0.345679	0.901310	0.542773	0.825067	1.000
126	GOBP_CELLULAR_HOMEOSTASIS	GO:0019725	18	0.248577	0.896771	0.564854	0.828038	1.000

Continuation of Table **S14**

	NAME	ID	SIZE	ES	NES	NOM.p.val	FDR.q.val	FWER.p.val
127	GOBP_POSITIVE_REGULATION_OF_CATION_TRANSMEMBRA...	GO:1904064	5	0.388807	0.896107	0.580381	0.822969	1.000
128	GOBP_INOSITOL_LIPID_MEDIATED_SIGNALING	GO:0048017	8	0.316281	0.893039	0.585443	0.822919	1.000
129	GOBP_ORGANOPHOSPHATE_METABOLIC_PROCESS	GO:0019637	7	0.333333	0.891909	0.549206	0.818998	1.000
130	GOBP_REGULATION_OF_ORGAN_GROWTH	GO:0046620	5	0.373494	0.890604	0.586301	0.815375	1.000
131	GOBP_ENDOCYTOSIS	GO:0006897	8	0.321347	0.883073	0.585586	0.825358	1.000
132	GOBP_ORGAN_GROWTH	GO:0035265	5	0.373494	0.867635	0.620596	0.851432	1.000
133	GOBP_CHEMICAL_HOMEOSTASIS	GO:0048878	23	0.217691	0.866808	0.692982	0.846693	1.000
134	GOBP_REGULATION_OF_AXONOGENESIS	GO:0050770	7	0.323434	0.866770	0.587692	0.840499	1.000
135	GOBP_REGULATION_OF_TRANSPORT	GO:0051049	44	0.187517	0.861945	0.700535	0.843909	1.000
136	GOBP_NEURON_DEVELOPMENT	GO:0048666	33	0.197215	0.840987	0.730594	0.881311	1.000
137	GOBP_POSITIVE_REGULATION_OF_PEPTIDYL_TYROSINE_...	GO:0050731	8	0.296511	0.813904	0.685294	0.928365	1.000
138	GOBP_CELL_JUNCTION_ORGANIZATION	GO:0034330	19	0.217074	0.807625	0.755102	0.934495	1.000
139	GOBP_POSITIVE_REGULATION_OF_NEUROGENESIS	GO:0050769	12	0.248249	0.798608	0.731707	0.946356	1.000
140	GOBP_REGULATION_OF_CYTOSKELETON_ORGANIZATION	GO:0051493	5	0.349398	0.798413	0.728022	0.940125	1.000
141	GOBP_POLYSACCHARIDE_METABOLIC_PROCESS	GO:0005976	5	0.339283	0.793756	0.731507	0.942519	1.000
142	GOBP_POSITIVE_REGULATION_OF_NERVOUS_SYSTEM_DEV...	GO:0051962	12	0.248249	0.793264	0.761745	0.936779	1.000
143	GOBP_POSITIVE_REGULATION_OF_CELL_DEVELOPMENT	GO:0010720	12	0.248249	0.792632	0.781351	0.931519	1.000
144	GOBP_MYOTUBE_DIFFERENTIATION	GO:0014902	5	0.325301	0.746720	0.809659	1.000000	1.000
145	GOBP_NEURON_DIFFERENTIATION	GO:0030182	36	0.167530	0.740298	0.897059	1.000000	1.000
146	GOBP_SENSORY_ORGAN_DEVELOPMENT	GO:0007423	8	0.259238	0.733515	0.780186	1.000000	1.000
147	GOBP_DENDRITE_MORPHOGENESIS	GO:0048813	6	0.292683	0.727726	0.820581	1.000000	1.000
148	GOBP_DENDRITIC_SPINE_DEVELOPMENT	GO:0060996	6	0.292683	0.727455	0.788162	1.000000	1.000
149	GOBP_REGULATION_OF_DEFENSE_RESPONSE	GO:0031347	7	0.271769	0.727136	0.835694	1.000000	1.000
150	GOBP_GENERATION_OF_PRECURSOR_METABOLITES_AND_E...	GO:0006091	5	0.313253	0.725343	0.835227	1.000000	1.000
151	GOBP_MUSCLE_CELL_DIFFERENTIATION	GO:0042692	10	0.243590	0.723570	0.848765	0.998903	1.000
152	GOBP_INSULIN_SECRETION	GO:0030073	7	0.272025	0.722257	0.809231	0.994467	1.000
153	GOBP_DENDRITIC_SPINE_MORPHOGENESIS	GO:0060997	5	0.315435	0.721876	0.846821	0.988669	1.000
154	GOBP_REGULATION_OF_PROTEIN_PHOSPHORYLATION	GO:0001932	26	0.179999	0.721078	0.848485	0.983493	1.000
155	GOBP_GLIOGENESIS	GO:0042063	12	0.223684	0.716417	0.845614	0.984438	1.000
156	GOBP_REGULATION_OF_INFLAMMATORY_RESPONSE	GO:0050727	7	0.271769	0.714030	0.842105	0.981635	1.000
157	GOBP_PEPTIDE_SECRETION	GO:0002790	9	0.245444	0.713969	0.810056	0.975498	1.000
158	GOBP_CELL_SURFACE_RECEPTOR_SIGNALING_PATHWAY_I...	GO:1905114	15	0.197042	0.705349	0.888136	0.981056	1.000
159	GOBP_POSITIVE_REGULATION_OF_MOLECULAR_FUNCTION	GO:0044093	38	0.163384	0.701977	0.911765	0.979315	1.000
160	GOBP_POSITIVE_REGULATION_OF_DEFENSE_RESPONSE	GO:0031349	5	0.295127	0.691171	0.872396	0.987825	1.000
161	GOBP_REGULATION_OF_LONG_TERM_SYNAPTIC_POTENTIA...	GO:1900271	6	0.280488	0.688337	0.861963	0.985042	1.000
162	GOBP_ACTIVATION_OF_PROTEIN_KINASE_ACTIVITY	GO:0032147	11	0.225829	0.685366	0.858025	0.982414	1.000
163	GOBP_PEPTIDE_HORMONE_SECRETION	GO:0030072	7	0.272025	0.677376	0.888889	0.986013	1.000
164	GOBP_EMBRYO_DEVELOPMENT_ENDING_IN_BIRTH_OR_EGG...	GO:0009792	9	0.227848	0.664860	0.860759	0.994652	1.000
165	GOBP_REGULATION_OF_SYNAPTIC_TRANSMISSION_GLUTA...	GO:0051966	12	0.199810	0.657028	0.936842	0.997048	1.000
166	GOBP_PEPTIDYL_TYROSINE_MODIFICATION	GO:0018212	10	0.217949	0.652299	0.905363	0.995832	1.000
167	GOBP_GLIAL_CELL_DIFFERENTIATION	GO:0010001	10	0.217949	0.648116	0.915825	0.993686	1.000
168	GOBP_REGULATION_OF_PEPTIDYL_TYROSINE_PHOSPHORY...	GO:0050730	9	0.215190	0.642636	0.914191	0.992961	1.000
169	GOBP_POSITIVE_REGULATION_OF_LONG_TERM_SYNAPTIC...	GO:1900273	5	0.277109	0.642364	0.901370	0.987418	1.000

Continuation of Table **S14**

	NAME	ID	SIZE	ES	NES	NOM.p.val	FDR.q.val	FWER.p.val
170	GOBP_POSITIVE_REGULATION_OF_DNA_BINDING_TRANSC...	GO:0051091	10	0.217949	0.637609	0.909091	0.985752	1.000
171	GOBP_EMBRYONIC_MORPHOGENESIS	GO:0048598	6	0.251273	0.621357	0.943953	0.993251	1.000
172	GOBP_CELLULAR_RESPONSE_TO_ABIOTIC_STIMULUS	GO:0071214	7	0.234568	0.612392	0.945289	0.994035	1.000
173	GOBP_SYNAPTIC_TRANSMISSION_GLUTAMATERGIC	GO:0035249	13	0.186968	0.610930	0.963415	0.989322	1.000
174	GOBP_TEMPERATURE_HOMEOSTASIS	GO:0001659	5	0.264083	0.603791	0.935484	0.988146	1.000
175	GOBP_NEUROGENESIS	GO:0022008	40	0.135842	0.598825	0.985849	0.985561	1.000
176	GOBP_NEGATIVE_REGULATION_OF_ANION_TRANSPORT	GO:1903792	8	0.212500	0.593632	0.969419	0.983221	1.000
177	GOBP_RESPONSE_TO_NUTRIENT	GO:0007584	5	0.240964	0.557474	0.975000	0.994878	1.000
178	GOBP_REGULATION_OF_METAL_ION_TRANSPORT	GO:0010959	6	0.222248	0.555794	0.972678	0.989955	1.000
179	GOBP_ANION_TRANSMEMBRANE_TRANSPORT	GO:0098656	7	0.209877	0.538815	0.982609	0.990215	1.000
180	GOBP_TELENCEPHALON_DEVELOPMENT	GO:0021537	6	0.200215	0.513294	1.000000	0.991088	1.000
181	GOBP_DENDRITE_DEVELOPMENT	GO:0016358	9	-0.169825	-0.421612	0.995792	0.997950	1.000
182	REACTOME_GPCR_LIGAND_BINDING	R-RNO-500792	8	-0.191630	-0.456364	0.993865	0.996325	1.000
183	GOBP_REGULATION_OF_PROTEIN_KINASE_ACTIVITY	GO:0045859	22	-0.152553	-0.476431	0.992042	0.995066	1.000
184	GOBP_REGULATION_OF_TRANSFERASE_ACTIVITY	GO:0051338	22	-0.152553	-0.480209	0.997358	0.996202	1.000
185	REACTOME_INTERFERON_SIGNALING	R-RNO-913531	6	-0.249880	-0.546301	0.979782	0.977704	1.000
186	GOBP_NEURON_MIGRATION	GO:0001764	5	-0.258937	-0.547467	0.968504	0.979031	1.000
187	GOBP_MUSCLE_TISSUE_DEVELOPMENT	GO:0060537	11	-0.212192	-0.554019	0.962910	0.977843	1.000
188	GOBP_G_PROTEIN_COUPLED_GLUTAMATE_RECEPTOR_SIGN...	GO:0007216	8	-0.231319	-0.558337	0.962853	0.977504	1.000
189	GOBP_NEGATIVE_REGULATION_OF_ION_TRANSPORT	GO:0043271	11	-0.216081	-0.566890	0.948468	0.975020	1.000
190	GOBP_LIPID_EXPORT_FROM_CELL	GO:0140353	5	-0.271597	-0.569057	0.959016	0.975739	1.000
191	GOBP_T_CELL_ACTIVATION	GO:0042110	8	-0.242287	-0.585993	0.940666	0.967468	1.000
192	GOBP_POSITIVE_REGULATION_OF_SECRETION	GO:0051047	6	-0.271901	-0.586346	0.944954	0.969097	1.000
193	GOBP_G_PROTEIN_COUPLED_RECEPTOR_SIGNALING_PATHWAY	GO:0007186	18	-0.200370	-0.594394	0.934051	0.965623	1.000
194	GOBP_INFLAMMATORY_RESPONSE	GO:0006954	12	-0.231952	-0.616344	0.922554	0.952071	1.000
195	GOBP_POSITIVE_REGULATION_OF_CELLULAR_COMPONENT...	GO:0051130	24	-0.203035	-0.636922	0.908740	0.937042	1.000
196	GOBP_PEPTIDYL_AMINO_ACID_MODIFICATION	GO:0018193	22	-0.206343	-0.652997	0.898123	0.924371	1.000
197	GOBP_REGULATION_OF_ENDOCYTOSIS	GO:0030100	6	-0.300046	-0.661145	0.894009	0.918418	1.000
198	GOBP_REGULATION_OF_CELL_DEVELOPMENT	GO:0060284	15	-0.230942	-0.666005	0.889675	0.915462	1.000
199	GOBP_REGULATION_OF_NERVOUS_SYSTEM_DEVELOPMENT	GO:0051960	15	-0.235938	-0.672473	0.875342	0.910643	1.000
200	GOBP_SKELETAL_MUSCLE_ORGAN_DEVELOPMENT	GO:0060538	5	-0.334096	-0.679968	0.896147	0.904833	1.000
201	GOBP_TAXIS	GO:0042330	14	-0.243338	-0.681463	0.876902	0.905071	1.000
202	GOBP_RESPONSE_TO_HEAT	GO:0009408	7	-0.290321	-0.682955	0.859873	0.905257	1.000
203	GOBP_INNATE_IMMUNE_RESPONSE	GO:0045087	7	-0.298775	-0.685053	0.869894	0.904760	1.000
204	REACTOME_POST_TRANSLATIONAL_PROTEIN_MODIFICATION	R-RNO-597592	7	-0.302892	-0.687271	0.870207	0.904123	1.000
205	GOBP_CELLULAR_COMPONENT_MORPHOGENESIS	GO:0032989	24	-0.220795	-0.688067	0.853137	0.904996	1.000
206	GOBP_DEFENSE_RESPONSE	GO:0006952	19	-0.225312	-0.689072	0.859395	0.905689	1.000
207	GOBP_REGULATION_OF_NEUROGENESIS	GO:0050767	14	-0.241254	-0.690108	0.863388	0.906352	1.000
208	GOBP_CIRCULATORY_SYSTEM_PROCESS	GO:0003013	5	-0.328266	-0.690497	0.867200	0.907709	1.000
209	GOBP_RESPONSE_TO_ALKALOID	GO:0043279	5	-0.333665	-0.692290	0.874433	0.907645	1.000
210	GOBP_REGULATION_OF_ANATOMICAL_STRUCTURE_MORPHO...	GO:0022603	17	-0.232897	-0.695752	0.860027	0.905634	1.000
211	GOBP_CELL_MORPHOGENESIS	GO:0000902	24	-0.220795	-0.698983	0.863937	0.903715	1.000
212	GOBP_CELL_PART_MORPHOGENESIS	GO:0032990	24	-0.220795	-0.700919	0.853529	0.903406	1.000

Continuation of Table **S14**

	NAME	ID	SIZE	ES	NES	NOM.p.val	FDR.q.val	FWER.p.val
213	GOBP_MUSCLE_CONTRACTION	GO:0006936	5	-0.336882	-0.703906	0.882258	0.901710	1.000
214	GOBP_REGULATION_OF_RECEPTOR_MEDIATED_ENDOCYTOSIS	GO:0048259	5	-0.348446	-0.713329	0.851974	0.892453	1.000
215	GOBP_MONOCARBOXYLIC_ACID_METABOLIC_PROCESS	GO:0032787	6	-0.322380	-0.715135	0.848159	0.892147	1.000
216	GOBP_PEPTIDE_METABOLIC_PROCESS	GO:0006518	12	-0.266934	-0.724419	0.811403	0.882653	1.000
217	GOBP_POSITIVE_REGULATION_OF_INTRACELLULAR_SIGN...	GO:1902533	14	-0.262033	-0.731756	0.850214	0.875542	1.000
218	GOBP_NEGATIVE_REGULATION_OF_PEPTIDASE_ACTIVITY	GO:0010466	6	-0.335684	-0.735434	0.818040	0.872770	1.000
219	GOBP_ACTIVATION_OF_MAPK_ACTIVITY	GO:0000187	6	-0.336967	-0.740621	0.801562	0.867809	1.000
220	GOBP_IN_UTERO_EMBRYONIC_DEVELOPMENT	GO:0001701	8	-0.305708	-0.740752	0.800298	0.869386	1.000
221	GOBP_VESICLE_MEDIATED_TRANSPORT_IN_SYNAPSE	GO:0099003	7	-0.320928	-0.740795	0.781818	0.871075	1.000
222	GOBP_POSITIVE_REGULATION_OF_MAP_KINASE_ACTIVITY	GO:0043406	6	-0.336967	-0.743018	0.810345	0.869985	1.000
223	GOBP_NEGATIVE_REGULATION_OF_LIPID_METABOLIC_PR...	GO:0045833	5	-0.359910	-0.744028	0.811232	0.870377	1.000
224	GOBP_POSITIVE_REGULATION_OF_PROTEIN_METABOLIC_...	GO:0051247	28	-0.226118	-0.746772	0.801556	0.868671	1.000
225	GOBP_REGULATION_OF_PHOSPHORUS_METABOLIC_PROCESS	GO:0051174	27	-0.226993	-0.748995	0.810323	0.867606	1.000
226	GOBP_REGULATION_OF_INTRACELLULAR_SIGNAL_TRANSD...	GO:1902531	21	-0.236480	-0.749125	0.816406	0.869188	1.000
227	REACTOME_TRANSCRIPTIONAL_REGULATION_OF_WHITE_A...	R-RNO-381340	6	-0.340009	-0.753964	0.786482	0.864581	1.000
228	GOBP_CARBOHYDRATE_HOMEOSTASIS	GO:0033500	7	-0.327619	-0.755096	0.786237	0.864889	1.000
229	GOBP_POSITIVE_REGULATION_OF_GLIOGENESIS	GO:0014015	5	-0.367355	-0.756854	0.794913	0.864336	1.000
230	GOBP_REGULATION_OF_LYMPHOCYTE_ACTIVATION	GO:0051249	5	-0.363501	-0.759099	0.797030	0.863120	1.000
231	GOBP_MULTICELLULAR_ORGANISMAL_RESPONSE_TO_STRESS	GO:0033555	5	-0.368926	-0.763752	0.768852	0.858766	1.000
232	GOBP_DEFENSE_RESPONSE_TO_OTHER_ORGANISM	GO:0098542	9	-0.306646	-0.763813	0.777299	0.860439	1.000
233	GOBP_ORGANIC_HYDROXY_COMPOUND_METABOLIC_PROCESS	GO:1901615	7	-0.329471	-0.768195	0.767516	0.856178	1.000
234	GOBP_REGULATION_OF_GLIOGENESIS	GO:0014013	5	-0.367355	-0.769565	0.771160	0.856067	1.000
235	GOBP_HEART_DEVELOPMENT	GO:0007507	6	-0.347948	-0.775517	0.751903	0.849694	1.000
236	GOBP_CARDIAC_MUSCLE_TISSUE_DEVELOPMENT	GO:0048738	6	-0.347948	-0.778112	0.762557	0.847880	1.000
237	GOBP_ORGANIC_HYDROXY_COMPOUND_TRANSPORT	GO:0015850	6	-0.357129	-0.781629	0.725637	0.844799	1.000
238	GOBP_MAPK_CASCADE	GO:0000165	18	-0.262669	-0.782731	0.777933	0.845085	1.000
239	GOBP_REGULATION_OF_MAP_KINASE_ACTIVITY	GO:0043405	9	-0.310818	-0.782838	0.726172	0.846683	1.000
240	GOBP_REGULATION_OF_ANATOMICAL_STRUCTURE_SIZE	GO:0090066	10	-0.299178	-0.782893	0.745714	0.848366	1.000
241	GOBP_CELLULAR_CARBOHYDRATE_METABOLIC_PROCESS	GO:0044262	5	-0.375523	-0.785391	0.773148	0.846500	1.000
242	GOBP_REGULATION_OF_MUSCLE_CELL_DIFFERENTIATION	GO:0051147	6	-0.355428	-0.785527	0.743511	0.848105	1.000
243	GOBP_NEGATIVE_REGULATION_OF_TRANSPORT	GO:0051051	13	-0.286187	-0.785537	0.734551	0.849859	1.000
244	GOBP_EXOCYTOSIS	GO:0006887	11	-0.300909	-0.790332	0.742938	0.844853	1.000
245	GOBP_MULTI_MULTICELLULAR_ORGANISM_PROCESS	GO:0044706	8	-0.325989	-0.790866	0.742560	0.845854	1.000
246	GOBP_REGULATION_OF_PEPTIDE_HORMONE_SECRETION	GO:0090276	6	-0.363780	-0.793880	0.748068	0.843151	1.000
247	GOBP_REGULATION_OF_PEPTIDE_SECRETION	GO:0002791	7	-0.343115	-0.803564	0.745038	0.830685	1.000
248	GOBP_INORGANIC_ION_TRANSMEMBRANE_TRANSPORT	GO:0098660	9	-0.322584	-0.803706	0.715774	0.832272	1.000
249	GOBP_PROTEIN_CONTAINING_COMPLEX_SUBUNIT_ORGANI...	GO:0043933	14	-0.289326	-0.806501	0.701513	0.829799	1.000
250	GOBP_REGULATION_OF_SYSTEM_PROCESS	GO:0044057	18	-0.271833	-0.809292	0.730028	0.827443	1.000
251	GOBP_ADENYLATE_CYCLASE_INHIBITING_G_PROTEIN_CO...	GO:0007196	5	-0.386404	-0.809356	0.734158	0.829136	1.000
252	REACTOME_NEUREXINS_AND_NEUROLIGINS	R-RNO-6794361	9	-0.324146	-0.809974	0.726471	0.830048	1.000
253	GOBP_POSITIVE_REGULATION_OF_SIGNALING	GO:0023056	34	-0.237600	-0.813645	0.738966	0.826352	1.000
254	GOBP_EMBRYO_DEVELOPMENT	GO:0009790	14	-0.288469	-0.825061	0.682652	0.811049	1.000
255	REACTOME_CLASS_C_3_METABOTROPIC_GLUTAMATE_PHER...	R-RNO-420499	7	-0.349545	-0.825956	0.676737	0.811449	1.000

Continuation of Table **S14**

	NAME	ID	SIZE	ES	NES	NOM.p.val	FDR.q.val	FWER.p.val
256	GOBP_PROTEIN_PHOSPHORYLATION	GO:0006468	34	-0.244965	-0.826224	0.701149	0.812819	1.000
257	GOBP_RESPONSE_TO_ALCOHOL	GO:0097305	11	-0.312598	-0.830026	0.688218	0.808631	1.000
258	GOBP_ORGANIC_ACID_METABOLIC_PROCESS	GO:0006082	7	-0.361574	-0.830306	0.710879	0.809981	1.000
259	GOBP_RESPONSE_TO_CALCIUM_ION	GO:0051592	6	-0.380301	-0.832963	0.689708	0.807619	1.000
260	GOBP_CELLULAR_RESPONSE_TO_NITROGEN_COMPOUND	GO:1901699	20	-0.277458	-0.837828	0.690958	0.802128	1.000
261	GOBP_MONOAMINE_TRANSPORT	GO:0015844	5	-0.396070	-0.838617	0.677083	0.802647	1.000
262	GOBP_NEGATIVE_REGULATION_OF_IMMUNE_SYSTEM_PROCESS	GO:0002683	5	-0.403248	-0.840231	0.722311	0.802037	1.000
263	GOBP_PEPTIDE_BIOSYNTHETIC_PROCESS	GO:0043043	7	-0.368201	-0.842160	0.676971	0.800936	1.000
264	GOBP_NEUROTRANSMITTER_SECRETION	GO:0007269	6	-0.387671	-0.842863	0.662461	0.801633	1.000
265	REACTOME_TRANSMISSION_ACROSS_CHEMICAL_SYNAPSES	R-RNO-112315	23	-0.265954	-0.845244	0.685601	0.799709	1.000
266	REACTOME_ONCOGENIC_MAPK_SIGNALING	R-HSA-6802957	5	-0.411024	-0.845937	0.675719	0.800374	1.000
267	GOBP_AMIDE_BIOSYNTHETIC_PROCESS	GO:0043604	7	-0.368201	-0.846261	0.685759	0.801679	1.000
268	GOBP_REGULATION_OF_NEUROTRANSMITTER_TRANSPORT	GO:0051588	6	-0.381977	-0.850134	0.646154	0.797467	1.000
269	REACTOME_NEUROTRANSMITTER_RECEPTORS_AND_POSTSY...	R-RNO-112314	22	-0.271442	-0.851369	0.666229	0.797441	1.000
270	REACTOME_EXTRACELLULAR_MATRIX_ORGANIZATION	R-RNO-1474244	5	-0.414832	-0.854713	0.678627	0.793887	1.000
271	GOBP_RESPONSE_TO_GROWTH_FACTOR	GO:0070848	18	-0.285305	-0.857323	0.648322	0.791391	1.000
272	GOBP_CALCIUM_ION_TRANSPORT_INTO_CYTOSOL	GO:0060402	7	-0.375595	-0.857672	0.666170	0.792570	1.000
273	GOBP_CELL_MORPHOGENESIS_INVOLVED_IN_NEURON_DIF...	GO:0048667	21	-0.276004	-0.857895	0.648074	0.793975	1.000
274	GOBP_MYELOID_LEUKOCYTE_ACTIVATION	GO:0002274	8	-0.359600	-0.860698	0.630277	0.791256	1.000
275	GOBP_CELL_MORPHOGENESIS_INVOLVED_IN_DIFFERENTI...	GO:0000904	21	-0.276004	-0.862922	0.673307	0.789473	1.000
276	GOBP_AXON_DEVELOPMENT	GO:0061564	18	-0.289943	-0.863928	0.655827	0.789675	1.000
277	REACTOME_DEATH_RECEPTOR_SIGNALLING	R-RNO-73887	5	-0.419493	-0.865334	0.670017	0.789222	1.000
278	GOBP_MUSCLE_SYSTEM_PROCESS	GO:0003012	9	-0.350214	-0.867375	0.643895	0.787891	1.000
279	GOBP_CYTOSOLIC_CALCIUM_ION_TRANSPORT	GO:0060401	7	-0.375595	-0.868712	0.635693	0.787531	1.000
280	GOBP_REGULATION_OF_VESICLE_MEDIATED_TRANSPORT	GO:0060627	12	-0.315591	-0.869931	0.620934	0.787388	1.000
281	GOBP_PROTEIN_COMPLEX_OLIGOMERIZATION	GO:0051259	6	-0.397790	-0.874762	0.635258	0.781468	1.000
282	GOBP_RESPONSE_TO_PEPTIDE	GO:1901652	18	-0.292727	-0.877093	0.627717	0.779537	1.000
283	GOBP_CALCIUM_ION_TRANSMEMBRANE_TRANSPORT	GO:0070588	7	-0.375595	-0.877148	0.628788	0.781237	1.000
284	GOBP_ADENYLATE_CYCLASE_MODULATING_G_PROTEIN_CO...	GO:0007188	11	-0.335552	-0.879306	0.595652	0.779339	1.000
285	GOBP_NEGATIVE_REGULATION_OF_CYSTEINE_TYPE_ENDO...	GO:2000117	5	-0.425797	-0.880236	0.641654	0.779557	1.000
286	GOBP_MUSCLE_ORGAN_DEVELOPMENT	GO:0007517	9	-0.355412	-0.880239	0.603976	0.781350	1.000
287	GOBP_CARBOHYDRATE_BIOSYNTHETIC_PROCESS	GO:0016051	5	-0.430286	-0.883736	0.650382	0.777398	1.000
288	GOBP_CALCIUM_MEDIATED_SIGNALING	GO:0019722	10	-0.344400	-0.884372	0.616783	0.778263	1.000
289	GOBP_NEGATIVE_REGULATION_OF_CATALYTIC_ACTIVITY	GO:0043086	17	-0.299231	-0.888683	0.623790	0.772936	1.000
290	REACTOME_DISEASES_OF_SIGNAL_TRANSDUCTION_BY_GR...	R-HSA-5663202	12	-0.328506	-0.890767	0.600559	0.771091	1.000
291	GOBP_RESPONSE_TO_PEPTIDE_HORMONE	GO:0043434	13	-0.320200	-0.891461	0.603878	0.771743	1.000
292	GOBP_HORMONE_TRANSPORT	GO:0009914	9	-0.357943	-0.893159	0.601783	0.770589	1.000
293	GOBP_POSITIVE_REGULATION_OF_RESPONSE_TO_EXTERN...	GO:0032103	9	-0.355374	-0.893227	0.599402	0.772277	1.000
294	GOBP_LIPID_LOCALIZATION	GO:0010876	10	-0.349426	-0.898180	0.584962	0.765836	1.000
295	GOBP_NUCLEOBASE_CONTAINING_SMALL_MOLECULE_META...	GO:0055086	6	-0.409846	-0.903127	0.597201	0.759328	1.000
296	GOBP_ANIMAL_ORGAN_MORPHOGENESIS	GO:0009887	12	-0.333184	-0.903343	0.572028	0.760775	1.000
297	GOBP_REGULATION_OF_CARBOHYDRATE_BIOSYNTHETIC_P...	GO:0043255	5	-0.430286	-0.903514	0.608108	0.762280	1.000
298	GOBP_REGULATION_OF_SMALL_MOLECULE_METABOLIC_PR...	GO:0062012	9	-0.370130	-0.904961	0.608889	0.761663	1.000

Continuation of Table **S14**

	NAME	ID	SIZE	ES	NES	NOM.p.val	FDR.q.val	FWER.p.val
299	REACTOME_TRAFFICKING_OF_GLUR2_CONTAINING_AMPA_...	R-RNO-416993	8	-0.375613	-0.905719	0.578550	0.762253	1.000
300	GOBP_REGULATION_OF_LIPID_METABOLIC_PROCESS	GO:0019216	7	-0.386184	-0.908596	0.583072	0.759201	1.000
301	GOBP_REGULATION_OF_CARBOHYDRATE_METABOLIC_PROCESS	GO:0006109	5	-0.430286	-0.910658	0.595506	0.757263	1.000
302	GOBP_CELLULAR_AMIDE_METABOLIC_PROCESS	GO:0043603	13	-0.327409	-0.910707	0.575676	0.759000	1.000
303	GOBP_PURINE_CONTAINING_COMPOUND_METABOLIC_PROCESS	GO:0072521	5	-0.440350	-0.915421	0.572123	0.753035	1.000
304	GOBP_NEGATIVE_REGULATION_OF_CELL_PROJECTION_OR...	GO:0031345	5	-0.445173	-0.917350	0.607085	0.751543	1.000
305	GOBP_INTRACELLULAR_TRANSPORT	GO:0046907	11	-0.348456	-0.918302	0.574409	0.751681	1.000
306	GOBP_LYMPHOCYTE_ACTIVATION	GO:0046649	9	-0.365535	-0.918410	0.560761	0.753320	1.000
307	GOBP_OSSIFICATION	GO:0001503	9	-0.379966	-0.920649	0.547724	0.751289	1.000
308	GOBP_REGULATION_OF_PEPTIDASE_ACTIVITY	GO:0052547	10	-0.363103	-0.923294	0.574405	0.748487	1.000
309	GOBP_NEGATIVE_REGULATION_OF_CELL_DEATH	GO:0060548	22	-0.298039	-0.924676	0.568493	0.747958	1.000
310	GOBP_DEPHOSPHORYLATION	GO:0016311	7	-0.405771	-0.928483	0.572755	0.742973	1.000
311	GOBP_CELL_GROWTH	GO:0016049	10	-0.357757	-0.930615	0.564470	0.741130	1.000
312	GOBP_NEURON_APOPTOTIC_PROCESS	GO:0051402	12	-0.340113	-0.932211	0.556309	0.740223	1.000
313	GOBP_REGULATION_OF_CELL_DIFFERENTIATION	GO:0045595	36	-0.276246	-0.934746	0.545918	0.737302	1.000
314	GOBP_POSITIVE_REGULATION_OF_EPITHELIAL_CELL_PR...	GO:0050679	7	-0.409642	-0.938258	0.541667	0.733102	1.000
315	GOBP_REGULATION_OF_ANION_TRANSMEMBRANE_TRANSPORT	GO:1903959	5	-0.448869	-0.938745	0.582400	0.734056	1.000
316	GOBP_REGULATION_OF_LIPID_LOCALIZATION	GO:1905952	5	-0.450405	-0.939823	0.551672	0.734068	1.000
317	GOBP_CELL_CELL_SIGNALING_BY_WNT	GO:0198738	7	-0.408384	-0.940435	0.542042	0.734786	1.000
318	GOBP_RESPONSE_TO_ENDOGENOUS_STIMULUS	GO:0009719	34	-0.275295	-0.940452	0.528809	0.736565	1.000
319	GOBP_CELL_MIGRATION	GO:0016477	22	-0.295742	-0.940588	0.537333	0.738165	1.000
320	GOBP_MUSCLE_ADAPTATION	GO:0043500	5	-0.454840	-0.941714	0.567901	0.738049	1.000
321	GOBP_PROTEIN_LOCALIZATION_TO_SYNAPSE	GO:0035418	5	-0.451705	-0.942334	0.576531	0.738776	1.000
322	GOBP_GROWTH	GO:0040007	14	-0.329649	-0.946533	0.535211	0.732944	1.000
323	GOBP_PROTEIN_LOCALIZATION_TO_PLASMA_MEMBRANE	GO:0072659	7	-0.406902	-0.946864	0.557576	0.734171	1.000
324	REACTOME_CELL_CYCLE_MITOTIC	R-RNO-69278	5	-0.450702	-0.949090	0.541085	0.732129	1.000
325	GOBP_PROTEIN_LOCALIZATION_TO_POSTSYNAPSE	GO:0062237	5	-0.451705	-0.949144	0.554662	0.733893	1.000
326	GOBP_SMALL_MOLECULE_METABOLIC_PROCESS	GO:0044281	15	-0.332213	-0.949978	0.527817	0.734314	1.000
327	GOBP_POSITIVE_REGULATION_OF_TRANSPORT	GO:0051050	26	-0.291410	-0.952397	0.530105	0.731819	1.000
328	GOBP_PROTEIN_DEPHOSPHORYLATION	GO:0006470	7	-0.405771	-0.952424	0.510294	0.733648	1.000
329	GOBP_REGULATION_OF_MUSCLE_ADAPTATION	GO:0043502	5	-0.454840	-0.954144	0.543131	0.732505	1.000
330	GOBP_REGULATION_OF_WNT_SIGNALING_PATHWAY	GO:0030111	5	-0.465355	-0.954902	0.559271	0.733030	1.000
331	GOBP_EXTRINSIC_APOPTOTIC_SIGNALING_PATHWAY	GO:0097191	7	-0.420125	-0.955076	0.524088	0.734636	1.000
332	GOBP_EPITHELIAL_CELL_DIFFERENTIATION	GO:0030855	7	-0.409356	-0.955216	0.524024	0.736281	1.000
333	GOBP_EMBRYONIC_ORGAN_DEVELOPMENT	GO:0048568	7	-0.412491	-0.955405	0.540458	0.737877	1.000
334	GOBP_SECRETION	GO:0046903	24	-0.299420	-0.959709	0.542969	0.731860	1.000
335	GOBP_ENDOCRINE_SYSTEM_DEVELOPMENT	GO:0035270	6	-0.435930	-0.960273	0.503852	0.732717	1.000
336	GOBP_RESPONSE_TO_NITROGEN_COMPOUND	GO:1901698	34	-0.286851	-0.963127	0.526510	0.729262	1.000
337	REACTOME_MAPK_FAMILY_SIGNALING_CASCADES	R-RNO-5683057	12	-0.349705	-0.964209	0.504348	0.729165	1.000
338	GOBP_POSITIVE_REGULATION_OF_PHOSPHATIDYLINOSIT...	GO:0014068	5	-0.472075	-0.965485	0.536392	0.728790	1.000
339	REACTOME_CELLULAR_RESPONSES_TO_EXTERNAL_STIMULI	R-RNO-8953897	12	-0.355016	-0.965697	0.510086	0.730313	1.000
340	HALLMARK_EPITHELIAL_MESENCHYMAL_TRANSITION	M5930	5	-0.464885	-0.966558	0.532925	0.730588	1.000
341	REACTOME_NERVOUS_SYSTEM_DEVELOPMENT	R-RNO-9675108	15	-0.337498	-0.968958	0.504881	0.728042	1.000

Continuation of Table **S14**

	NAME	ID	SIZE	ES	NES	NOM.p.val	FDR.q.val	FWER.p.val
342	REACTOME_NEUTROPHIL_DEGRANULATION	R-RNO-6798695	6	-0.440796	-0.969933	0.511905	0.728158	1.000
343	GOBP_SPROUTING_ANGIOGENESIS	GO:0002040	5	-0.477801	-0.971365	0.515823	0.727496	1.000
344	GOBP_CELL_ACTIVATION_INVOLVED_IN_IMMUNE_RESPONSE	GO:0002263	6	-0.440796	-0.973448	0.512938	0.725539	1.000
345	GOBP_NEUROTRANSMITTER_TRANSPORT	GO:0006836	7	-0.428602	-0.976511	0.499232	0.721888	1.000
346	GOBP_REGULATION_OF_NEUROTRANSMITTER_LEVELS	GO:0001505	7	-0.428602	-0.978564	0.496229	0.719929	1.000
347	GOBP_POSITIVE_REGULATION_OF_CYSTEINE_TYPE_ENDO...	GO:2001056	7	-0.422371	-0.979975	0.494012	0.719123	1.000
348	GOBP_EXCITATORY_CHEMICAL_SYNAPTIC_TRANSMISSION	GO:0098976	5	-0.472269	-0.980034	0.494506	0.720956	1.000
349	GOBP_FOREBRAIN_DEVELOPMENT	GO:0030900	9	-0.398592	-0.980358	0.490674	0.722309	1.000
350	GOBP_POSITIVE_REGULATION_OF_PEPTIDASE_ACTIVITY	GO:0010952	7	-0.422371	-0.980670	0.495342	0.723748	1.000
351	GOBP_LEUKOCYTE_CELL_CELL_ADHESION	GO:0007159	6	-0.443084	-0.980710	0.514821	0.725644	1.000
352	GOBP_POSITIVE_REGULATION_OF_ENDOTHELIAL_CELL_P...	GO:0001938	6	-0.450245	-0.983842	0.484472	0.721678	1.000
353	GOBP_ADENYLATE_CYCLASE_INHIBITING_G_PROTEIN_CO...	GO:0007196	9	-0.384718	-0.983851	0.485714	0.723635	1.000
354	REACTOME_SIGNALING_BY_RECEPTOR_TYROSINE_KINASES	R-RNO-9006934	26	-0.303982	-0.984868	0.469309	0.723837	1.000
355	HALLMARK_UV_RESPONSE_UP	M5941	5	-0.482279	-0.986752	0.506536	0.722246	1.000
356	GOBP_NEUROINFLAMMATORY_RESPONSE	GO:0150076	5	-0.475460	-0.987031	0.483819	0.723670	1.000
357	GOBP_RESPONSE_TO_INTERLEUKIN_1	GO:0070555	5	-0.469547	-0.987733	0.501538	0.724349	1.000
358	GOBP_MYELOID_LEUKOCYTE_MEDIATED_IMMUNITY	GO:0002444	6	-0.440796	-0.988536	0.489764	0.724887	1.000
359	GOBP_MALE_GAMETE_GENERATION	GO:0048232	5	-0.470730	-0.988875	0.489130	0.726218	1.000
360	GOBP_NEGATIVE_REGULATION_OF_HYDROLASE_ACTIVITY	GO:0051346	11	-0.370726	-0.990923	0.473609	0.724406	1.000
361	GOBP_REGULATION_OF_GROWTH	GO:0040008	13	-0.358004	-0.992030	0.473397	0.724205	1.000
362	REACTOME_G_ALPHA_Q_SIGNALLING_EVENTS	R-RNO-416476	6	-0.446040	-0.994336	0.483821	0.721409	1.000
363	GOBP_AMEBOIDAL_TYPE_CELL_MIGRATION	GO:0001667	11	-0.371903	-0.995528	0.461201	0.721094	1.000
364	GOBP_REGULATION_OF_CELLULAR_COMPONENT_BIOGENESIS	GO:0044087	10	-0.387436	-0.997655	0.484050	0.719261	1.000
365	GOBP_REGULATION_OF_MUSCLE_SYSTEM_PROCESS	GO:0090257	8	-0.413910	-0.998384	0.468702	0.719752	1.000
366	GOBP_POSITIVE_REGULATION_OF_MULTICELLULAR_ORGA...	GO:0051240	32	-0.295986	-0.998781	0.467949	0.721042	1.000
367	REACTOME_ANTI_INFLAMMATORY_RESPONSE_FAVOURING_...	R-HSA-9662851	5	-0.478060	-0.999069	0.462366	0.722576	1.000
368	GOBP_PHOSPHATIDYLINOSITOL_3_KINASE_SIGNALING	GO:0014065	7	-0.438727	-1.002825	0.468606	0.717320	1.000
369	GOBP_CARBOHYDRATE_METABOLIC_PROCESS	GO:0005975	9	-0.396140	-1.004090	0.466851	0.716757	1.000
370	GOBP_POSITIVE_REGULATION_OF_PROTEOLYSIS	GO:0045862	8	-0.416338	-1.004554	0.467391	0.717921	1.000
371	GOBP_CELLULAR_RESPONSE_TO_STARVATION	GO:0009267	5	-0.493329	-1.004799	0.490015	0.719528	1.000
372	GOBP_MULTICELLULAR_ORGANISMAL_HOMEOSTASIS	GO:0048871	12	-0.376595	-1.005207	0.470109	0.720827	1.000
373	GOBP_FAT_CELL_DIFFERENTIATION	GO:0045444	9	-0.404516	-1.005938	0.478582	0.721497	1.000
374	GOBP_REGULATION_OF_PHOSPHATIDYLINOSITOL_3_KINA...	GO:0014066	6	-0.450967	-1.006929	0.481651	0.721637	1.000
375	REACTOME_EPH_EPHRIN_SIGNALING	R-RNO-2682334	6	-0.459712	-1.008983	0.469419	0.719632	1.000
376	REACTOME_DAG_AND_IP3_SIGNALING	R-RNO-1489509	8	-0.416769	-1.010299	0.467262	0.719058	1.000
377	GOBP_REGULATION_OF_HYDROLASE_ACTIVITY	GO:0051336	22	-0.325901	-1.012859	0.443857	0.716150	1.000
378	GOBP_CELLULAR_RESPONSE_TO_PEPTIDE_HORMONE_STIM...	GO:0071375	9	-0.406157	-1.015340	0.449405	0.713284	1.000
379	GOBP_REGULATION_OF_PROTEIN_MODIFICATION_PROCESS	GO:0031399	29	-0.306673	-1.019740	0.428756	0.706491	1.000
380	GOBP_HOMEOSTATIC_PROCESS	GO:0042592	31	-0.307377	-1.022093	0.444159	0.703629	1.000
381	GOBP_REGULATION_OF_MAPK_CASCADE	GO:0043408	13	-0.367067	-1.024035	0.436700	0.701903	1.000
382	GOBP_NEGATIVE_REGULATION_OF_KINASE_ACTIVITY	GO:0033673	5	-0.495056	-1.026687	0.451078	0.698963	1.000
383	GOBP_CELLULAR_RESPONSE_TO_EXTERNAL_STIMULUS	GO:0071496	10	-0.400279	-1.027209	0.419214	0.699968	1.000
384	GOBP_NEGATIVE_REGULATION_OF_TRANSFERASE_ACTIVITY	GO:0051348	5	-0.495056	-1.027819	0.441130	0.700780	1.000

Continuation of Table **S14**

	NAME	ID	SIZE	ES	NES	NOM.p.val	FDR.q.val	FWER.p.val
385	GOBP_RESPONSE_TO_UV	GO:0009411	5	-0.503461	-1.028990	0.445652	0.700442	1.000
386	GOBP_RESPONSE_TO_STARVATION	GO:0042594	5	-0.493329	-1.029565	0.437908	0.701271	1.000
387	GOBP_NEGATIVE_REGULATION_OF_PHOSPHORYLATION	GO:0042326	7	-0.441962	-1.029846	0.431008	0.702814	1.000
388	GOBP_REGULATION_OF_SECRETION	GO:0051046	14	-0.366493	-1.034277	0.422652	0.695971	1.000
389	GOBP_REGULATION_OF_CYSTEINE_TYPE_ENDOPEPTIDASE...	GO:2000116	9	-0.413344	-1.043637	0.402077	0.678820	1.000
390	REACTOME_DEVELOPMENTAL_BIOLOGY	R-RNO-1266738	23	-0.328959	-1.044384	0.412550	0.679374	1.000
391	GOBP_NEGATIVE_REGULATION_OF_SIGNALING	GO:0023057	21	-0.336964	-1.048748	0.402850	0.672597	1.000
392	GOBP_RAS_PROTEIN_SIGNAL_TRANSDUCTION	GO:0007265	5	-0.517023	-1.055691	0.405864	0.660812	1.000
393	GOBP_ORGANONITROGEN_COMPOUND_BIOSYNTHETIC_PROCESS	GO:1901566	13	-0.382275	-1.057446	0.395184	0.659325	1.000
394	GOBP_POSITIVE_REGULATION_OF_ION_TRANSPORT	GO:0043270	17	-0.358662	-1.057859	0.390647	0.660433	1.000
395	GOBP_REGULATION_OF_ANION_TRANSPORT	GO:0044070	20	-0.349890	-1.059001	0.380886	0.660163	1.000
396	GOBP_NEGATIVE_REGULATION_OF_LOCOMOTION	GO:0040013	5	-0.502628	-1.060607	0.386435	0.659060	1.000
397	GOBP_POSITIVE_REGULATION_OF_MAPK_CASCADE	GO:0043410	9	-0.412827	-1.061161	0.384389	0.660041	1.000
398	GOBP_POSITIVE_REGULATION_OF_CELLULAR_COMPONENT...	GO:0044089	9	-0.423034	-1.061309	0.406755	0.661829	1.000
399	GOBP_ESTABLISHMENT_OF_PROTEIN_LOCALIZATION	GO:0045184	18	-0.359840	-1.061989	0.396783	0.662564	1.000
400	GOBP_SIGNAL_RELEASE	GO:0023061	14	-0.377679	-1.062816	0.374302	0.662972	1.000
401	GOBP_ASSOCIATIVE_LEARNING	GO:0008306	7	-0.460200	-1.064057	0.406480	0.662524	1.000
402	REACTOME_RNA_POLYMERASE_II_TRANSCRIPTION	R-RNO-73857	20	-0.351475	-1.066125	0.374507	0.660340	1.000
403	GOBP_CELLULAR_GLUCOSE_HOMEOSTASIS	GO:0001678	5	-0.512427	-1.068103	0.395210	0.658267	1.000
404	GOBP_CELLULAR_RESPONSE_TO_PEPTIDE	GO:1901653	13	-0.382318	-1.069068	0.395862	0.658262	1.000
405	GOBP_CIRCADIAN_REGULATION_OF_GENE_EXPRESSION	GO:0032922	5	-0.515160	-1.069613	0.384498	0.659201	1.000
406	REACTOME_INTERLEUKIN_4_AND_INTERLEUKIN_13_SIGN...	R-RNO-6785807	8	-0.449741	-1.071826	0.398571	0.656595	1.000
407	GOBP_SEXUAL_REPRODUCTION	GO:0019953	10	-0.416722	-1.072864	0.365682	0.656649	1.000
408	GOBP_REGULATION_OF_CELLULAR_COMPONENT_MOVEMENT	GO:0051270	15	-0.373971	-1.073419	0.374294	0.657593	1.000
409	GOBP_REGULATION_OF_OSTEOCLAST_DIFFERENTIATION	GO:0045670	5	-0.513404	-1.073717	0.382848	0.659065	1.000
410	GOBP_REGULATION_OF_CELL_DEATH	GO:0010941	34	-0.322989	-1.084159	0.343150	0.638977	1.000
411	GOBP_REGULATION_OF_HORMONE_SECRETION	GO:0046883	8	-0.446121	-1.084608	0.373494	0.640138	1.000
412	REACTOME_CA_DEPENDENT_EVENTS	R-RNO-111996	8	-0.440478	-1.085465	0.333333	0.640376	1.000
413	GOBP_ENDOTHELIAL_CELL_MIGRATION	GO:0043542	8	-0.461052	-1.086193	0.372635	0.640887	1.000
414	GOBP_ACTIVATION_OF_IMMUNE_RESPONSE	GO:0002253	5	-0.521884	-1.088918	0.354267	0.637460	1.000
415	GOBP_REGULATION_OF_CELL_ACTIVATION	GO:0050865	8	-0.459134	-1.099515	0.342342	0.617923	1.000
416	GOBP_RESPONSE_TO_NICOTINE	GO:0035094	7	-0.475707	-1.103661	0.358548	0.611465	1.000
417	GOBP_NEURON_PROJECTION_GUIDANCE	GO:0097485	9	-0.442951	-1.105987	0.343612	0.608811	1.000
418	GOBP_NEGATIVE_REGULATION_OF_CELLULAR_COMPONENT...	GO:0051129	11	-0.418384	-1.108764	0.338912	0.605050	1.000
419	GOBP_RESPONSE_TO_TEMPERATURE_STIMULUS	GO:0009266	9	-0.441108	-1.112314	0.324168	0.599623	1.000
420	GOBP_GAMETE_GENERATION	GO:0007276	9	-0.448546	-1.112449	0.329078	0.601329	1.000
421	GOBP_PROTEIN_KINASE_B_SIGNALING	GO:0043491	6	-0.507879	-1.113993	0.335866	0.600006	1.000
422	GOBP_REGULATION_OF_CELLULAR_CATABOLIC_PROCESS	GO:0031329	10	-0.430138	-1.114546	0.331420	0.600801	1.000
423	GOBP_LEARNING	GO:0007612	13	-0.400810	-1.115254	0.325352	0.601471	1.000
424	GOBP_BLOOD_VESSEL_ENDOTHELIAL_CELL_MIGRATION	GO:0043534	8	-0.461052	-1.116691	0.319749	0.600587	1.000
425	GOBP_CELLULAR_LIPID_METABOLIC_PROCESS	GO:0044255	7	-0.481714	-1.117480	0.327300	0.601067	1.000
426	GOBP_RESPONSE_TO_EXTRACELLULAR_STIMULUS	GO:0009991	13	-0.413583	-1.118410	0.324750	0.601075	1.000
427	REACTOME_SIGNALING_BY_GPCR	R-RNO-372790	22	-0.354932	-1.120183	0.302865	0.599164	1.000

Continuation of Table **S14**

	NAME	ID	SIZE	ES	NES	NOM.p.val	FDR.q.val	FWER.p.val
428	REACTOME_L1CAM_INTERACTIONS	R-RNO-373760	5	-0.546893	-1.120938	0.325914	0.599627	1.000
429	GOBP_POSITIVE_REGULATION_OF_NEURON_DEATH	GO:1901216	5	-0.541925	-1.122610	0.326923	0.598241	1.000
430	GOBP_RESPONSE_TO_OXYGEN_LEVELS	GO:0070482	8	-0.464156	-1.123583	0.311047	0.598175	1.000
431	REACTOME_INTRACELLULAR_SIGNALING_BY_SECOND_MES...	R-RNO-9006925	15	-0.388713	-1.123719	0.311522	0.599949	1.000
432	GOBP_TRANSMEMBRANE_RECEPTOR_PROTEIN_TYROSINE_K...	GO:0007169	19	-0.369271	-1.126988	0.315364	0.595051	1.000
433	GOBP_CYTOKINE_MEDIATED_SIGNALING_PATHWAY	GO:0019221	16	-0.385985	-1.127792	0.297260	0.595450	1.000
434	GOBP_POSITIVE_REGULATION_OF_CELL_DEATH	GO:0010942	19	-0.372147	-1.131475	0.311634	0.589656	1.000
435	GOBP_NEURON_DEATH	GO:0070997	18	-0.376074	-1.133688	0.301061	0.587087	1.000
436	GOBP_POSITIVE_REGULATION_OF_PROTEIN_SERINE_THR...	GO:0071902	9	-0.446523	-1.135837	0.298682	0.584469	1.000
437	GOBP_POSITIVE_REGULATION_OF_DEVELOPMENTAL_PROCESS	GO:0051094	32	-0.338772	-1.139227	0.293520	0.579437	1.000
438	GOBP_MORPHOGENESIS_OF_AN_EPITHELIUM	GO:0002009	6	-0.509450	-1.139361	0.314815	0.581188	1.000
439	GOBP_PROTEIN_AUTOPHOSPHORYLATION	GO:0046777	5	-0.553332	-1.140026	0.314425	0.581823	1.000
440	GOBP_MUSCLE_STRUCTURE_DEVELOPMENT	GO:0061061	16	-0.396315	-1.142965	0.306849	0.577695	1.000
441	GOBP_NEGATIVE_REGULATION_OF_PROTEOLYSIS	GO:0045861	8	-0.469777	-1.145393	0.288084	0.574484	1.000
442	GOBP_REGULATION_OF_CELL_CELL_ADHESION	GO:0022407	9	-0.459300	-1.145529	0.292467	0.576294	1.000
443	REACTOME_G_ALPHA_I_SIGNALLING_EVENTS	R-RNO-418594	18	-0.379797	-1.148312	0.280822	0.572578	1.000
444	GOBP_NEGATIVE_REGULATION_OF_CELL_CYCLE	GO:0045786	5	-0.554217	-1.148394	0.297078	0.574457	1.000
445	GOBP_CELLULAR_RESPONSE_TO_KETONE	GO:1901655	5	-0.552973	-1.149409	0.293461	0.574417	1.000
446	GOBP_POSITIVE_REGULATION_OF_CELL_DIFFERENTIATION	GO:0045597	27	-0.344946	-1.150488	0.284967	0.574196	1.000
447	GOBP_REGULATION_OF_PROTEIN_STABILITY	GO:0031647	5	-0.566259	-1.150598	0.293564	0.576102	1.000
448	GOBP_TISSUE_MIGRATION	GO:0090130	9	-0.457598	-1.154792	0.279104	0.569072	1.000
449	GOBP_CELLULAR_RESPONSE_TO_ALCOHOL	GO:0097306	5	-0.557662	-1.154854	0.278135	0.571038	1.000
450	REACTOME_ESTROGEN_DEPENDENT_NUCLEAR_EVENTS_DOW...	R-RNO-9634638	5	-0.557976	-1.155832	0.285943	0.571301	1.000
451	GOBP_RESPIRATORY_SYSTEM_DEVELOPMENT	GO:0060541	6	-0.521203	-1.158269	0.283742	0.568111	1.000
452	GOBP_REGULATION_OF_CELLULAR_AMIDE_METABOLIC_PR...	GO:0034248	9	-0.467688	-1.159762	0.282421	0.566966	1.000
453	GOBP_EPIDERMIS_DEVELOPMENT	GO:0008544	5	-0.561536	-1.159794	0.278207	0.569021	1.000
454	GOBP_EPHRIN_RECEPTOR_SIGNALING_PATHWAY	GO:0048013	5	-0.561362	-1.164966	0.277512	0.560548	1.000
455	GOBP_POSITIVE_REGULATION_OF_CELL_CELL_ADHESION	GO:0022409	5	-0.570060	-1.168124	0.293185	0.555695	1.000
456	GOBP_REGULATION_OF_PEPTIDE_TRANSPORT	GO:0090087	10	-0.442469	-1.169897	0.278499	0.553887	1.000
457	GOBP_ANATOMICAL_STRUCTURE_FORMATION_INVOLVED_I...	GO:0048646	20	-0.385197	-1.171160	0.259591	0.553202	1.000
458	GOBP_IMPORT_INTO_CELL	GO:0098657	6	-0.531559	-1.174518	0.275969	0.548020	1.000
459	GOBP_REGULATION_OF_BINDING	GO:0051098	11	-0.452476	-1.174642	0.265335	0.549847	1.000
460	GOBP_HOMEOSTASIS_OF_NUMBER_OF_CELLS	GO:0048872	5	-0.554608	-1.176729	0.266254	0.547597	1.000
461	GOBP_INTRACELLULAR_RECEPTOR_SIGNALING_PATHWAY	GO:0030522	5	-0.562297	-1.177173	0.248788	0.548710	1.000
462	GOBP_REGULATION_OF_CELL_ADHESION	GO:0030155	13	-0.427447	-1.177257	0.264828	0.550667	1.000
463	GOBP_MEMBRANE_ORGANIZATION	GO:0061024	12	-0.449054	-1.177756	0.267525	0.551749	1.000
464	GOBP_SECOND_MESSENGER_MEDIATED_SIGNALING	GO:0019932	16	-0.402811	-1.179236	0.267983	0.550574	1.000
465	GOBP_GLAND_DEVELOPMENT	GO:0048732	7	-0.509580	-1.179953	0.264122	0.551194	1.000
466	GOBP_POSITIVE_REGULATION_OF_ORGANELLE_ORGANIZA...	GO:0010638	10	-0.455895	-1.180425	0.263533	0.552337	1.000
467	GOBP_REGULATION_OF_FAT_CELL_DIFFERENTIATION	GO:0045598	6	-0.542294	-1.180577	0.257576	0.554212	1.000
468	REACTOME_G_PROTEIN_MEDIATED_EVENTS	R-RNO-112040	9	-0.468667	-1.181526	0.248889	0.554314	1.000
469	GOBP_LOCOMOTION	GO:0040011	31	-0.354203	-1.182814	0.253827	0.553695	1.000
470	GOBP_LIPID_METABOLIC_PROCESS	GO:0006629	10	-0.453245	-1.183579	0.249288	0.554258	1.000

Continuation of Table **S14**

	NAME	ID	SIZE	ES	NES	NOM.p.val	FDR.q.val	FWER.p.val
471	GOBP_MEMBRANE_PROTEIN_PROTEOLYSIS	GO:0033619	6	-0.534829	-1.183702	0.272586	0.556206	1.000
472	GOBP_POSITIVE_REGULATION_OF_GENE_EXPRESSION	GO:0010628	16	-0.404110	-1.184729	0.248032	0.556217	1.000
473	GOBP_SMALL_GTPASE_MEDIATED_SIGNAL_TRANSDUCTION	GO:0007264	8	-0.493019	-1.185563	0.261516	0.556509	1.000
474	GOBP_POSITIVE_REGULATION_OF_LEUKOCYTE_CELL_CEL...	GO:1903039	5	-0.570060	-1.185918	0.230887	0.558019	1.000
475	GOBP_CELLULAR_RESPONSE_TO_EXTRACELLULAR_STIMULUS	GO:0031668	8	-0.497305	-1.186036	0.279001	0.560033	1.000
476	GOBP_OSTEOBLAST_DIFFERENTIATION	GO:0001649	5	-0.566061	-1.190067	0.242376	0.553522	1.000
477	GOBP_REGULATION_OF_CIRCADIAN_RHYTHM	GO:0042752	7	-0.514972	-1.192325	0.260212	0.551068	1.000
478	REACTOME_CIRCADIAN_CLOCK	R-HSA-400253	5	-0.573930	-1.195935	0.244019	0.545312	1.000
479	GOBP_REGULATION_OF_MULTICELLULAR_ORGANISMAL_DE...	GO:2000026	31	-0.362303	-1.205084	0.236709	0.527647	1.000
480	HALLMARK_APOPTOSIS	M5902	6	-0.550328	-1.209423	0.215873	0.520616	1.000
481	GOBP_BIOLOGICAL_ADHESION	GO:0022610	19	-0.394708	-1.210534	0.226519	0.520402	1.000
482	GOBP_LEUKOCYTE_MIGRATION	GO:0050900	6	-0.544886	-1.213434	0.214391	0.516189	1.000
483	REACTOME_CELLULAR_SENESCENCE	R-RNO-2559583	6	-0.559810	-1.218086	0.222910	0.508285	1.000
484	REACTOME_EXTRA_NUCLEAR_ESTROGEN_SIGNALING	R-RNO-9009391	7	-0.528607	-1.220095	0.223077	0.506211	1.000
485	GOBP_CYTOSKELETON_ORGANIZATION	GO:0007010	11	-0.474431	-1.220989	0.231760	0.506650	1.000
486	REACTOME_PTEN_REGULATION	R-RNO-6807070	5	-0.594614	-1.221061	0.226415	0.508713	1.000
487	REACTOME_SENESCENCE_ASSOCIATED_SECRETORY_PHENO...	R-RNO-2559582	6	-0.559810	-1.227801	0.200000	0.496446	1.000
488	GOBP_RESPONSE_TO_ABIOTIC_STIMULUS	GO:0009628	27	-0.381607	-1.230990	0.198925	0.491983	1.000
489	GOBP_LEUKOCYTE_MEDIATED_IMMUNITY	GO:0002443	7	-0.526107	-1.233912	0.214176	0.488042	1.000
490	GOBP_OSTEOCLAST_DIFFERENTIATION	GO:0030316	6	-0.569164	-1.234265	0.206731	0.489500	1.000
491	GOBP_POSTTRANSCRIPTIONAL_REGULATION_OF_GENE_EX...	GO:0010608	8	-0.509474	-1.234846	0.217066	0.490295	1.000
492	GOBP_NEGATIVE_REGULATION_OF_CATABOLIC_PROCESS	GO:0009895	8	-0.509810	-1.237152	0.202050	0.487432	1.000
493	GOBP_RESPONSE_TO_MECHANICAL_STIMULUS	GO:0009612	6	-0.559612	-1.238363	0.210687	0.487117	1.000
494	GOBP_REGULATION_OF_LIPID_BIOSYNTHETIC_PROCESS	GO:0046890	6	-0.571877	-1.238642	0.216718	0.488716	1.000
495	GOBP_POSITIVE_REGULATION_OF_CELL_ADHESION	GO:0045785	7	-0.539602	-1.239507	0.209199	0.489233	1.000
496	GOBP_REGULATION_OF_RESPONSE_TO_STRESS	GO:0080134	19	-0.410329	-1.239849	0.202514	0.490625	1.000
497	GOBP_INTRINSIC_APOPTOTIC_SIGNALING_PATHWAY	GO:0097193	5	-0.595652	-1.240722	0.183359	0.490984	1.000
498	REACTOME_INTEGRATION_OF_ENERGY_METABOLISM	R-RNO-163685	5	-0.612785	-1.243963	0.197390	0.485938	1.000
499	GOBP_CELL_JUNCTION_ASSEMBLY	GO:0034329	8	-0.519218	-1.244396	0.200295	0.487276	1.000
500	GOBP_NEGATIVE_REGULATION_OF_PROTEIN_CATABOLIC_...	GO:0042177	5	-0.603428	-1.245600	0.193199	0.486963	1.000
501	GOBP_REGULATION_OF_PROTEIN_SERINE_THREONINE_KI...	GO:0071900	12	-0.461404	-1.247432	0.204769	0.485419	1.000
502	GOBP_RESPONSE_TO_OXYGEN_CONTAINING_COMPOUND	GO:1901700	39	-0.359915	-1.248847	0.188045	0.484644	1.000
503	GOBP_DEVELOPMENTAL_PROCESS_INVOLVED_IN_REPRODU...	GO:0003006	11	-0.482261	-1.249592	0.202899	0.485306	1.000
504	REACTOME_FC_EPSILON_RECEPTOR_FCERI_SIGNALING	R-RNO-2454202	7	-0.537202	-1.250647	0.191063	0.485288	1.000
505	GOBP_FC_EPSILON_RECEPTOR_SIGNALING_PATHWAY	GO:0038095	7	-0.537202	-1.250977	0.189024	0.486745	1.000
506	GOBP_PROTEIN_LOCALIZATION_TO_ORGANELLE	GO:0033365	9	-0.510995	-1.251775	0.183381	0.487145	1.000
507	GOBP_REGULATION_OF_PROTEOLYSIS	GO:0030162	12	-0.455525	-1.253173	0.195225	0.486534	1.000
508	GOBP_MAINTENANCE_OF_LOCATION	GO:0051235	7	-0.540365	-1.253625	0.207143	0.487841	1.000
509	GOBP_RESPONSE_TO_BIOTIC_STIMULUS	GO:0009607	17	-0.426189	-1.255092	0.199158	0.486850	1.000
510	GOBP_PEPTIDYL_LYSINE_MODIFICATION	GO:0018205	5	-0.601123	-1.255610	0.186520	0.488046	1.000
511	GOBP_REPRODUCTION	GO:0000003	18	-0.422724	-1.258602	0.163265	0.483948	1.000
512	GOBP_ERK1_AND_ERK2_CASCADE	GO:0070371	5	-0.602525	-1.259114	0.175115	0.485174	1.000
513	GOBP_REGULATION_OF_CELL_CYCLE_PROCESS	GO:0010564	5	-0.605539	-1.261307	0.173313	0.482861	1.000

Continuation of Table **S14**

	NAME	ID	SIZE	ES	NES	NOM.p.val	FDR.q.val	FWER.p.val
514	GOBP_REGULATION_OF_ORGANELLE_ORGANIZATION	GO:0033043	14	-0.440816	-1.261566	0.195122	0.484709	1.000
515	GOBP_LIPID_BIOSYNTHETIC_PROCESS	GO:0008610	6	-0.571877	-1.263558	0.176563	0.482898	1.000
516	GOBP_NEGATIVE_REGULATION_OF_RESPONSE_TO_EXTERN...	GO:0032102	5	-0.610088	-1.264972	0.181126	0.482005	1.000
517	GOBP_REGULATION_OF_DNA_BINDING_TRANSCRIPTION_F...	GO:0051090	16	-0.432672	-1.269080	0.176144	0.474919	1.000
518	GOBP_PROTEIN_LOCALIZATION_TO_MEMBRANE	GO:0072657	14	-0.454465	-1.270282	0.178121	0.474634	1.000
519	GOBP_CELLULAR_COMPONENT_DISASSEMBLY	GO:0022411	6	-0.582656	-1.275201	0.156105	0.465813	1.000
520	GOBP_ESTABLISHMENT_OF_PROTEIN_LOCALIZATION_TO_...	GO:0072594	6	-0.575273	-1.278013	0.165635	0.461857	1.000
521	GOBP_MULTICELLULAR_ORGANISM_REPRODUCTION	GO:0032504	12	-0.473916	-1.280665	0.169188	0.458971	1.000
522	GOBP_REGULATION_OF_CELLULAR_RESPONSE_TO_STRESS	GO:0080135	9	-0.510234	-1.281282	0.176558	0.460050	1.000
523	GOBP_RESPONSE_TO_INSULIN	GO:0032868	5	-0.621597	-1.282353	0.159420	0.460180	1.000
524	GOBP_ANATOMICAL_STRUCTURE_HOMEOSTASIS	GO:0060249	7	-0.552246	-1.282803	0.174528	0.461561	1.000
525	REACTOME_G_ALPHA_Z_SIGNALLING_EVENTS	R-RNO-418597	5	-0.623425	-1.284219	0.172414	0.461037	1.000
526	GOBP_POSITIVE_REGULATION_OF_CYTOKINE_PRODUCTION	GO:0001819	7	-0.556004	-1.285835	0.175150	0.459874	1.000
527	GOBP_POSITIVE_REGULATION_OF_LOCOMOTION	GO:0040017	11	-0.478343	-1.286289	0.173104	0.461199	1.000
528	GOBP_PROTEIN_LOCALIZATION_TO_CELL_PERIPHERY	GO:1990778	12	-0.475630	-1.286669	0.171388	0.462808	1.000
529	GOBP_MONONUCLEAR_CELL_DIFFERENTIATION	GO:1903131	7	-0.553067	-1.288037	0.182371	0.462345	1.000
530	GOBP_APOPTOTIC_PROCESS	GO:0006915	35	-0.381447	-1.289892	0.153186	0.460708	1.000
531	GOBP_ACTIN_FILAMENT_BASED_PROCESS	GO:0030029	7	-0.549885	-1.290410	0.159940	0.462110	1.000
532	GOBP_CELLULAR_RESPONSE_TO_HORMONE_STIMULUS	GO:0032870	11	-0.489732	-1.292907	0.165975	0.458502	1.000
533	GOBP_HEAD_DEVELOPMENT	GO:0060322	22	-0.414989	-1.294014	0.167726	0.458470	1.000
534	GOBP_CELL_CELL_ADHESION	GO:0098609	14	-0.456416	-1.297615	0.170765	0.453007	1.000
535	REACTOME_MEMBRANE_TRAFFICKING	R-RNO-199991	5	-0.627173	-1.300739	0.158654	0.448624	1.000
536	GOBP_AGING	GO:0007568	13	-0.469068	-1.303006	0.171788	0.446118	1.000
537	GOBP_NEGATIVE_REGULATION_OF_CELL_POPULATION_PR...	GO:0008285	9	-0.522323	-1.303332	0.151825	0.447952	1.000
538	GOBP_TISSUE_MORPHOGENESIS	GO:0048729	7	-0.550675	-1.304957	0.156923	0.447002	1.000
539	GOBP_AMYLOID_BETA_METABOLIC_PROCESS	GO:0050435	5	-0.633039	-1.306881	0.125786	0.444996	1.000
540	GOBP_REGULATION_OF_PROTEIN_LOCALIZATION_TO_CEL...	GO:1904375	6	-0.587960	-1.306930	0.145062	0.447421	1.000
541	GOBP_POSITIVE_REGULATION_OF_ESTABLISHMENT_OF_P...	GO:1904951	5	-0.631771	-1.312334	0.147673	0.437844	1.000
542	GOBP_CELLULAR_MACROMOLECULE_LOCALIZATION	GO:0070727	21	-0.425897	-1.312821	0.134349	0.439223	1.000
543	GOBP_POSITIVE_REGULATION_OF_CELL_POPULATION_PR...	GO:0008284	21	-0.421622	-1.312956	0.138441	0.441427	1.000
544	GOBP_PROTEOLYSIS	GO:0006508	17	-0.450445	-1.312972	0.128065	0.443932	1.000
545	GOBP_NEGATIVE_REGULATION_OF_INTRACELLULAR_SIGN...	GO:1902532	7	-0.559023	-1.312992	0.139466	0.446414	1.000
546	GOBP_CELL_CELL_JUNCTION_ASSEMBLY	GO:0007043	5	-0.628631	-1.314178	0.127055	0.446192	1.000
547	REACTOME_INFECTIOUS_DISEASE	R-HSA-5663205	13	-0.477951	-1.315433	0.144044	0.445762	1.000
548	GOBP_POSITIVE_REGULATION_OF_SMALL_MOLECULE_MET...	GO:0062013	5	-0.635402	-1.318008	0.122417	0.442287	1.000
549	REACTOME_NGF_STIMULATED_TRANSCRIPTION	R-RNO-9031628	9	-0.524113	-1.322209	0.145115	0.435186	1.000
550	GOBP_PROTEIN_LOCALIZATION_TO_CELL_JUNCTION	GO:1902414	6	-0.609480	-1.322591	0.144216	0.436764	1.000
551	GOBP_AMYLOID_PRECURSOR_PROTEIN_METABOLIC_PROCESS	GO:0042982	5	-0.633039	-1.324007	0.124595	0.436316	1.000
552	REACTOME_VESICLE_MEDIATED_TRANSPORT	R-RNO-5653656	5	-0.627173	-1.324319	0.137600	0.438319	1.000
553	GOBP_AMYLOID_PRECURSOR_PROTEIN_CATABOLIC_PROCESS	GO:0042987	5	-0.633039	-1.326422	0.127451	0.435923	1.000
554	REACTOME_CELL_CYCLE	R-RNO-1640170	6	-0.590729	-1.328338	0.135542	0.434269	1.000
555	REACTOME_OPIOID_SIGNALLING	R-RNO-111885	12	-0.489571	-1.333008	0.122963	0.426280	1.000
556	GOBP_REGULATION_OF_RESPONSE_TO_EXTERNAL_STIMULUS	GO:0032101	15	-0.469180	-1.335375	0.122253	0.423528	1.000

Continuation of Table **S14**

	NAME	ID	SIZE	ES	NES	NOM.p.val	FDR.q.val	FWER.p.val
557	REACTOME_CYTOKINE_SIGNALING_IN_IMMUNE_SYSTEM	R-RNO-1280215	21	-0.438737	-1.336157	0.136126	0.424438	1.000
558	HALLMARK_HYPOXIA	M5891	5	-0.640660	-1.341054	0.124807	0.416019	1.000
559	GOBP_REGULATION_OF_PROTEIN_LOCALIZATION	GO:0032880	18	-0.439813	-1.341594	0.120805	0.417429	1.000
560	GOBP_POSITIVE_REGULATION_OF_CELLULAR_PROTEIN_L...	GO:1903829	7	-0.575381	-1.341643	0.125000	0.419883	1.000
561	GOBP_EPITHELIUM_DEVELOPMENT	GO:0060429	15	-0.466605	-1.344429	0.125176	0.416549	1.000
562	GOBP_INTRACELLULAR_PROTEIN_TRANSPORT	GO:0006886	9	-0.534929	-1.346239	0.120715	0.414884	1.000
563	HALLMARK_TNFA_SIGNALING_VIA_NFKB	M5890	14	-0.475107	-1.353748	0.116901	0.400574	1.000
564	REACTOME_INTERLEUKIN_17_SIGNALING	R-RNO-448424	6	-0.617888	-1.362088	0.119335	0.385255	1.000
565	GOBP_RESPONSE_TO_HORMONE	GO:0009725	20	-0.436957	-1.364745	0.123835	0.381856	1.000
566	GOBP_POSITIVE_REGULATION_OF_EPITHELIAL_CELL_MI...	GO:0010634	5	-0.661651	-1.365557	0.092332	0.382734	1.000
567	GOBP_ENDOMEMBRANE_SYSTEM_ORGANIZATION	GO:0010256	6	-0.615238	-1.368580	0.109940	0.378576	1.000
568	GOBP_REGULATION_OF_GTPASE_ACTIVITY	GO:0043087	5	-0.656120	-1.372505	0.106725	0.372952	1.000
569	GOBP_REGULATION_OF_CATABOLIC_PROCESS	GO:0009894	13	-0.494918	-1.373650	0.105644	0.372994	1.000
570	GOBP_NEGATIVE_REGULATION_OF_NERVOUS_SYSTEM_DEV...	GO:0051961	5	-0.663511	-1.373919	0.075710	0.374979	1.000
571	GOBP_RESPONSE_TO_INORGANIC_SUBSTANCE	GO:0010035	19	-0.457066	-1.373941	0.116248	0.377476	1.000
572	GOBP_NEGATIVE_REGULATION_OF_MULTICELLULAR_ORGA...	GO:0051241	15	-0.473331	-1.374968	0.106534	0.377747	1.000
573	GOBP_CELL_CYCLE_PROCESS	GO:0022402	11	-0.522879	-1.380349	0.101156	0.368932	1.000
574	GOBP_POSITIVE_REGULATION_OF_HEMOPOIESIS	GO:1903708	7	-0.604938	-1.382346	0.103048	0.367235	1.000
575	GOBP_CELLULAR_RESPONSE_TO_ORGANIC_CYCLIC_COMPOUND	GO:0071407	13	-0.495187	-1.383210	0.103550	0.367858	1.000
576	REACTOME_TRANSCRIPTIONAL_REGULATION_BY_TP53	R-RNO-3700989	6	-0.630828	-1.385165	0.087500	0.366304	1.000
577	REACTOME_SIGNALING_BY_NUCLEAR_RECEPTORS	R-RNO-9006931	8	-0.578316	-1.386378	0.102453	0.366225	1.000
578	GOBP_CELLULAR_MACROMOLECULE_CATABOLIC_PROCESS	GO:0044265	6	-0.630397	-1.388491	0.089202	0.363972	1.000
579	GOBP_RESPONSE_TO_ACID_CHEMICAL	GO:0001101	6	-0.630962	-1.389425	0.085139	0.364565	1.000
580	GOBP_RESPONSE_TO_CYTOKINE	GO:0034097	23	-0.444123	-1.391782	0.095872	0.361911	1.000
581	GOBP_NEGATIVE_REGULATION_OF_PROTEIN_METABOLIC_...	GO:0051248	20	-0.460963	-1.399774	0.083799	0.348249	1.000
582	GOBP_NEGATIVE_REGULATION_OF_TRANSMEMBRANE_TRAN...	GO:0034763	6	-0.632308	-1.399997	0.088550	0.350289	1.000
583	GOBP_CELL_CELL_JUNCTION_ORGANIZATION	GO:0045216	6	-0.629966	-1.400035	0.094595	0.352784	1.000
584	GOBP_NEGATIVE_REGULATION_OF_MOLECULAR_FUNCTION	GO:0044092	24	-0.445276	-1.400230	0.084918	0.354855	1.000
585	GOBP_NEGATIVE_REGULATION_OF_CELL_DEVELOPMENT	GO:0010721	6	-0.632957	-1.400496	0.089202	0.357000	1.000
586	GOBP_REPRODUCTIVE_SYSTEM_DEVELOPMENT	GO:0061458	6	-0.641572	-1.401005	0.082792	0.358474	1.000
587	GOBP_RESPONSE_TO_TUMOR_NECROSIS_FACTOR	GO:0034612	7	-0.607462	-1.403199	0.094815	0.356791	1.000
588	REACTOME_NUCLEAR_EVENTS_KINASE_AND_TRANSCRIPTI...	R-RNO-198725	11	-0.533065	-1.403523	0.093484	0.358863	1.000
589	GOBP_NEGATIVE_REGULATION_OF_PHOSPHORUS_METABOL...	GO:0010563	9	-0.560604	-1.406121	0.089419	0.355892	1.000
590	REACTOME_ESR_MEDIATED_SIGNALING	R-RNO-8939211	8	-0.578316	-1.406476	0.083458	0.357889	1.000
591	GOBP_REGULATION_OF_PROTEIN_LOCALIZATION_TO_MEM...	GO:1905475	7	-0.604870	-1.406846	0.080871	0.359883	1.000
592	GOBP_RESPONSE_TO_LIGHT_STIMULUS	GO:0009416	11	-0.523387	-1.410242	0.095729	0.355214	1.000
593	GOBP_REGULATION_OF_VASCULATURE_DEVELOPMENT	GO:1901342	5	-0.686454	-1.413411	0.073600	0.351240	1.000
594	REACTOME_SIGNALING_BY_WNT	R-RNO-195721	7	-0.611373	-1.414512	0.085139	0.351682	1.000
595	REACTOME_LEISHMANIA_INFECTION	R-HSA-9658195	10	-0.539120	-1.415090	0.091298	0.353179	1.000
596	GOBP_NEGATIVE_REGULATION_OF_TRANSCRIPTION_BY_R...	GO:0000122	10	-0.545247	-1.417694	0.087770	0.350286	1.000
597	GOBP_RESPONSE_TO_KETONE	GO:1901654	7	-0.621300	-1.418721	0.102832	0.350822	1.000
598	GOBP_POSITIVE_REGULATION_OF_IMMUNE_RESPONSE	GO:0050778	9	-0.555320	-1.419267	0.093220	0.352513	1.000
599	GOBP_RESPONSE_TO_ORGANIC_CYCLIC_COMPOUND	GO:0014070	20	-0.460948	-1.423189	0.074728	0.347424	1.000

Continuation of Table **S14**

	NAME	ID	SIZE	ES	NES	NOM.p.val	FDR.q.val	FWER.p.val
600	GOBP_CENTRAL_NERVOUS_SYSTEM_DEVELOPMENT	GO:0007417	26	-0.446402	-1.423451	0.081117	0.349762	1.000
601	REACTOME_PLATELET_ACTIVATION_SIGNALING_AND_AGG...	R-HSA-76002	7	-0.601663	-1.434621	0.082831	0.329465	1.000
602	GOBP_CELL_ACTIVATION	GO:0001775	21	-0.469663	-1.436794	0.074667	0.327892	1.000
603	GOBP_ENDOTHELIAL_CELL_PROLIFERATION	GO:0001935	8	-0.579062	-1.437413	0.077160	0.329297	1.000
604	GOBP_TISSUE_HOMEOSTASIS	GO:0001894	5	-0.695341	-1.442242	0.068182	0.322665	1.000
605	GOBP_MULTI_ORGANISM_PROCESS	GO:0051704	16	-0.495280	-1.443253	0.080711	0.323192	1.000
606	GOBP_IMMUNE_RESPONSE_REGULATING_SIGNALING_PATHWAY	GO:000276	8	-0.608852	-1.445589	0.076358	0.321128	1.000
607	REACTOME_ADAPTIVE_IMMUNE_SYSTEM	R-RNO-1280218	9	-0.570356	-1.455088	0.073906	0.304840	1.000
608	GOBP_VASCULATURE_DEVELOPMENT	GO:0001944	17	-0.499892	-1.458490	0.065826	0.301290	1.000
609	GOBP_GLUCOSE_METABOLIC_PROCESS	GO:0006006	6	-0.673422	-1.466457	0.053042	0.288099	1.000
610	GOBP_NEGATIVE_REGULATION_OF_GENE_EXPRESSION	GO:0010629	12	-0.545970	-1.467629	0.065248	0.288487	1.000
611	GOBP_CELL_POPULATION_PROLIFERATION	GO:0008283	31	-0.439369	-1.469941	0.051572	0.286800	1.000
612	GOBP_FC_RECEPTOR_SIGNALING_PATHWAY	GO:0038093	8	-0.608852	-1.470034	0.074242	0.289307	1.000
613	GOBP_BLOOD_VESSEL_MORPHOGENESIS	GO:0048514	14	-0.524079	-1.471157	0.064156	0.289922	1.000
614	GOBP_CELLULAR_RESPONSE_TO_BIOTIC_STIMULUS	GO:0071216	8	-0.604991	-1.471758	0.061605	0.291454	1.000
615	GOBP_DNA_METABOLIC_PROCESS	GO:0006259	6	-0.673749	-1.478918	0.044961	0.280553	1.000
616	GOBP_REGULATION_OF_LEUKOCYTE_DIFFERENTIATION	GO:1902105	9	-0.587632	-1.479242	0.063470	0.282481	1.000
617	GOBP_CELLULAR_RESPONSE_TO_MOLECULE_OF_BACTERIA...	GO:0071219	8	-0.604991	-1.481979	0.061728	0.280022	1.000
618	GOBP_ORGANONITROGEN_COMPOUND_CATABOLIC_PROCESS	GO:1901565	9	-0.604426	-1.482138	0.051750	0.282513	1.000
619	GOBP_REGULATION_OF_EXTRINSIC_APOPTOTIC_SIGNALI...	GO:2001236	6	-0.672396	-1.482403	0.046948	0.284975	1.000
620	GOBP_TUBE_DEVELOPMENT	GO:0035295	18	-0.510793	-1.484679	0.052561	0.283516	1.000
621	GOBP_MUSCLE_CELL_PROLIFERATION	GO:0033002	8	-0.612194	-1.484760	0.043541	0.286353	1.000
622	GOBP_REGULATION_OF_PROTEIN_CATABOLIC_PROCESS	GO:0042176	7	-0.637297	-1.488548	0.056923	0.282305	1.000
623	GOBP_ERBB_SIGNALING_PATHWAY	GO:0038127	5	-0.722630	-1.489523	0.034215	0.283106	1.000
624	GOBP_CHROMATIN_ORGANIZATION	GO:0006325	5	-0.722892	-1.490524	0.036866	0.284118	1.000
625	GOBP_REGULATION_OF_ENDOTHELIAL_CELL_MIGRATION	GO:0010594	5	-0.709613	-1.491129	0.042276	0.285952	1.000
626	GOBP_MONOSACCHARIDE_METABOLIC_PROCESS	GO:0005996	6	-0.673422	-1.491129	0.051128	0.289027	1.000
627	GOBP_EPITHELIAL_CELL_PROLIFERATION	GO:0050673	12	-0.550209	-1.491385	0.069672	0.291564	1.000
628	GOBP_POSITIVE_REGULATION_OF_APOPTOTIC_SIGNALIN...	GO:2001235	5	-0.734940	-1.493987	0.043339	0.289385	1.000
629	GOBP_IMMUNE_EFFECTOR_PROCESS	GO:0002252	10	-0.583485	-1.494707	0.048780	0.291249	1.000
630	GOBP_TUBE_MORPHOGENESIS	GO:0035239	15	-0.522309	-1.505098	0.053908	0.273409	1.000
631	GOBP_PROTEIN_MODIFICATION_BY_SMALL_PROTEIN_CON...	GO:0032446	6	-0.691855	-1.508328	0.033233	0.270355	1.000
632	GOBP_POSITIVE_REGULATION_OF_SMOOTH_MUSCLE_CELL...	GO:0048661	5	-0.732086	-1.509184	0.037736	0.271924	1.000
633	GOBP_RESPONSE_TO_BACTERIUM	GO:0009617	13	-0.559844	-1.510477	0.042877	0.272539	1.000
634	GOBP_NEGATIVE_REGULATION_OF_PROTEIN_MODIFICATI...	GO:0031400	10	-0.600181	-1.514599	0.044863	0.268169	1.000
635	GOBP_POSITIVE_REGULATION_OF_MYELOID_LEUKOCYTE_...	GO:0002763	6	-0.686842	-1.515990	0.048465	0.268528	1.000
636	REACTOME_BETA_CATENIN_INDEPENDENT_WNT_SIGNALING	R-RNO-3858494	5	-0.740174	-1.519006	0.022013	0.265945	1.000
637	GOBP_CIRCADIAN_RHYTHM	GO:0007623	13	-0.545876	-1.519849	0.043185	0.267258	1.000
638	GOBP_MITOTIC_CELL_CYCLE	GO:0000278	10	-0.590661	-1.519872	0.042336	0.270544	1.000
639	GOBP_RESPONSE_TO_RADIATION	GO:0009314	13	-0.551247	-1.523121	0.049226	0.266780	1.000
640	GOBP_NEGATIVE_REGULATION_OF_BINDING	GO:0051100	6	-0.678112	-1.523159	0.046225	0.270076	1.000
641	GOBP_REGULATION_OF_CELLULAR_LOCALIZATION	GO:0060341	17	-0.522516	-1.524919	0.031464	0.270129	1.000
642	REACTOME_MAPK_TARGETS_NUCLEAR_EVENTS_MEDIATED_...	R-HSA-450282	5	-0.715967	-1.526846	0.035427	0.270064	1.000

Continuation of Table **S14**

	NAME	ID	SIZE	ES	NES	NOM.p.val	FDR.q.val	FWER.p.val
643	GOBP_BIOLOGICAL_PROCESS_INVOLVED_IN_SYMBIOTIC_...	GO:004440	11	-0.574124	-1.527849	0.046784	0.271718	1.000
644	GOBP_ENZYME_LINKED_RECEPTOR_PROTEIN_SIGNALING_...	GO:0007167	24	-0.478180	-1.538891	0.034076	0.255122	1.000
645	GOBP_CHROMOSOME_ORGANIZATION	GO:0051276	6	-0.705104	-1.540686	0.027287	0.255059	1.000
646	GOBP_REGULATION_OF_MITOTIC_CELL_CYCLE	GO:0007346	5	-0.742239	-1.540790	0.033333	0.258328	1.000
647	GOBP_SKIN_DEVELOPMENT	GO:0043588	5	-0.730149	-1.541176	0.040310	0.261079	1.000
648	GOBP_MITOCHONDRION_ORGANIZATION	GO:0007005	7	-0.675904	-1.548931	0.036220	0.250316	1.000
649	GOBP_RESPONSE_TO_REACTIVE_OXYGEN_SPECIES	GO:0000302	8	-0.647348	-1.550406	0.031019	0.251207	1.000
650	REACTOME_HEMOSTASIS	R-RNO-109582	14	-0.554022	-1.551307	0.031989	0.253327	1.000
651	GOBP_PHAGOCYTOSIS	GO:0006909	5	-0.739949	-1.551887	0.024316	0.256039	1.000
652	GOBP_RHYTHMIC_PROCESS	GO:0048511	15	-0.549462	-1.555519	0.028302	0.252716	1.000
653	GOBP_NEGATIVE_REGULATION_OF_NUCLEOBASE_CONTAIN...	GO:0045934	13	-0.561585	-1.557017	0.036212	0.253808	1.000
654	REACTOME_SIGNALING_BY_INTERLEUKINS	R-RNO-449147	15	-0.540274	-1.558723	0.036932	0.253987	1.000
655	GOBP_RESPONSE_TO_MOLECULE_OF_BACTERIAL_ORIGIN	GO:0002237	13	-0.559844	-1.562099	0.028050	0.251192	1.000
656	GOBP_REGULATION_OF_EPITHELIAL_CELL_MIGRATION	GO:0010632	6	-0.705708	-1.565336	0.018293	0.249027	1.000
657	GOBP_RESPONSE_TO_OXIDATIVE_STRESS	GO:0006979	8	-0.647348	-1.566597	0.031157	0.250695	1.000
658	GOBP_NEGATIVE_REGULATION_OF_APOPTOTIC_SIGNALIN...	GO:2001234	7	-0.674534	-1.569100	0.028860	0.249839	1.000
659	GOBP_REGULATION_OF_PROTEIN_BINDING	GO:0043393	7	-0.673716	-1.569365	0.033537	0.253602	1.000
660	GOBP_REGULATION_OF_BODY_FLUID_LEVELS	GO:0050878	11	-0.597467	-1.570443	0.024965	0.255950	1.000
661	GOBP_REGULATION_OF_HEMOPOIESIS	GO:1903706	10	-0.619904	-1.572032	0.031884	0.257611	1.000
662	GOBP_POSITIVE_REGULATION_OF_ANION_TRANSPORT	GO:1903793	9	-0.632214	-1.573492	0.027616	0.259134	1.000
663	GOBP_WOUND_HEALING	GO:0042060	13	-0.573044	-1.582511	0.028610	0.244817	1.000
664	GOBP_CELLULAR_RESPONSE_TO_INORGANIC_SUBSTANCE	GO:0071241	11	-0.605967	-1.582897	0.021645	0.248496	1.000
665	GOBP_CELLULAR_RESPONSE_TO_DNA_DAMAGE_STIMULUS	GO:0006974	5	-0.761192	-1.584056	0.016975	0.250800	1.000
666	GOBP_REGULATION_OF_MYELOID_LEUKOCYTE_DIFFERENT...	GO:0002761	8	-0.655406	-1.584343	0.025875	0.254975	1.000
667	GOBP_APOPTOTIC_MITOCHONDRIAL_CHANGES	GO:0008637	6	-0.724333	-1.585737	0.014354	0.256772	1.000
668	GOBP_CYTOKINE_PRODUCTION	GO:0001816	10	-0.612547	-1.586598	0.028571	0.260261	1.000
669	GOBP_NEGATIVE_REGULATION_OF_RESPONSE_TO_STIMULUS	GO:0048585	16	-0.533139	-1.586816	0.037657	0.264960	1.000
670	GOBP_NEGATIVE_REGULATION_OF_BIOSYNTHETIC_PROCESS	GO:0009890	13	-0.577697	-1.591550	0.019417	0.260685	1.000
671	REACTOME_TOLL_LIKE_RECEPTOR_TLR1_TLR2_CASCADE	R-RNO-168179	8	-0.670845	-1.592570	0.029806	0.264088	1.000
672	GOBP_RESPONSE_TO_METAL_ION	GO:0010038	15	-0.543059	-1.598558	0.015850	0.256662	1.000
673	REACTOME_TOLL_LIKE_RECEPTOR_CASCADES	R-RNO-168898	8	-0.670845	-1.599909	0.020313	0.259409	1.000
674	GOBP_REGULATION_OF_VASCULAR_ASSOCIATED_SMOOTH_...	GO:1904705	5	-0.778860	-1.602224	0.014331	0.260437	1.000
675	REACTOME_TOLL_LIKE_RECEPTOR_9_TLR9_CASCADE	R-RNO-168138	8	-0.670845	-1.607773	0.033528	0.254186	1.000
676	GOBP_NEGATIVE_REGULATION_OF_CELL_DIFFERENTIATION	GO:0045596	13	-0.588441	-1.609863	0.012894	0.255832	1.000
677	GOBP_REGULATION_OF_CELLULAR_PROTEIN_LOCALIZATION	GO:1903827	12	-0.597250	-1.609962	0.024148	0.261616	1.000
678	GOBP_CIRCULATORY_SYSTEM_DEVELOPMENT	GO:0072359	21	-0.514645	-1.612949	0.030749	0.260931	1.000
679	GOBP_PROTEIN_CATABOLIC_PROCESS	GO:0030163	8	-0.669457	-1.617114	0.022189	0.258427	1.000
680	HALLMARK_ALLOGRAFT_REJECTION	M5950	7	-0.702358	-1.625750	0.016743	0.247502	1.000
681	REACTOME_MYD88_INDEPENDENT_TLR4_CASCADE	R-RNO-166166	8	-0.670845	-1.626224	0.024206	0.253095	1.000
682	GOBP_TRANSMEMBRANE_RECEPTOR_PROTEIN_SERINE_THR...	GO:0007178	6	-0.750179	-1.626701	0.016248	0.258933	1.000
683	GOBP_POSITIVE_REGULATION_OF_MYELOID_CELL_DIFFE...	GO:0045639	7	-0.708362	-1.635383	0.020000	0.246571	1.000
684	REACTOME_SIGNALING_BY_RHO_GTPASES_MIRO_GTPASES...	R-RNO-9716542	9	-0.660284	-1.636720	0.010219	0.250339	1.000
685	GOBP_CELLULAR_RESPONSE_TO_LIPID	GO:0071396	15	-0.578584	-1.646026	0.013793	0.237044	1.000

Continuation of Table **S14**

	NAME	ID	SIZE	ES	NES	NOM.p.val	FDR.q.val	FWER.p.val
686	GOBP_CELLULAR_RESPONSE_TO_OXYGEN_CONTAINING_CO...	GO:1901701	29	-0.493290	-1.646801	0.019330	0.242403	1.000
687	GOBP_APOPTOTIC_SIGNALING_PATHWAY	GO:0097190	15	-0.582939	-1.654206	0.017833	0.233949	0.999
688	GOBP_NEGATIVE_REGULATION_OF_DEVELOPMENTAL_PROCESS	GO:0051093	13	-0.588441	-1.660823	0.017143	0.226673	0.999
689	GOBP_REGULATION_OF_IMMUNE_RESPONSE	GO:0050776	12	-0.613339	-1.665371	0.015471	0.224716	0.999
690	GOBP_PROTEIN_MODIFICATION_BY_SMALL_PROTEIN_CON...	GO:0070647	7	-0.727459	-1.669098	0.014948	0.225311	0.999
691	GOBP_POSITIVE_REGULATION_OF_TRANSCRIPTION_BY_R...	GO:0045944	24	-0.520024	-1.670979	0.019048	0.229584	0.999
692	GOBP_POSITIVE_REGULATION_OF_BIOSYNTHETIC_PROCESS	GO:0009891	27	-0.526428	-1.675706	0.011827	0.228155	0.999
693	GOBP_MACROMOLECULE_CATABOLIC_PROCESS	GO:0009057	12	-0.616347	-1.675857	0.015737	0.236765	0.999
694	GOBP_RESPONSE_TO_WOUNDING	GO:0009611	15	-0.593490	-1.679823	0.009790	0.236967	0.998
695	GOBP_POSITIVE_REGULATION_OF_PRI_MIRNA_TRANSCRI...	GO:1902895	7	-0.737014	-1.686360	0.011994	0.231239	0.996
696	GOBP_COAGULATION	GO:0050817	8	-0.695155	-1.687644	0.014948	0.238675	0.996
697	GOBP_CELL_CYCLE	GO:0007049	21	-0.543600	-1.689326	0.014825	0.245422	0.995
698	REACTOME_RHO_GTPASE_EFFECTORS	R-RNO-195258	9	-0.660284	-1.692465	0.011628	0.249449	0.994
699	GOBP_REGULATION_OF_MYELOID_CELL_DIFFERENTIATION	GO:0045637	9	-0.680721	-1.693223	0.008511	0.259851	0.994
700	GOBP_CELLULAR_RESPONSE_TO_CADMIUM_ION	GO:0071276	5	-0.817470	-1.699882	0.001577	0.256770	0.988
701	GOBP_SMOOTH_MUSCLE_CELL_PROLIFERATION	GO:0048659	6	-0.765608	-1.704261	0.004615	0.260477	0.987
702	GOBP_PRI_MIRNA_TRANSCRIPTION_BY_RNA_POLYMERASE_II	GO:0061614	7	-0.737014	-1.712164	0.003035	0.257147	0.983
703	GOBP_IMMUNE_SYSTEM_DEVELOPMENT	GO:0002520	17	-0.577877	-1.712215	0.008392	0.273084	0.983
704	GOBP_REGULATION_OF_CELL_CYCLE	GO:0051726	13	-0.622145	-1.717631	0.011817	0.277643	0.978
705	GOBP_RESPONSE_TO_DRUG	GO:0042493	11	-0.659907	-1.719809	0.010309	0.291391	0.978
706	GOBP_POSITIVE_REGULATION_OF_IMMUNE_SYSTEM_PROCESS	GO:0002684	18	-0.567760	-1.720327	0.006748	0.312281	0.978
707	GOBP_REGULATION_OF_IMMUNE_SYSTEM_PROCESS	GO:0002682	21	-0.554817	-1.721168	0.005479	0.335933	0.978
708	GOBP_RESPONSE_TO_LIPID	GO:0033993	19	-0.572650	-1.725202	0.011111	0.351913	0.975
709	GOBP_CELLULAR_RESPONSE_TO_CHEMICAL_STRESS	GO:0062197	7	-0.761052	-1.753678	0.001536	0.285094	0.938
710	GOBP_REGULATION_OF_APOPTOTIC_SIGNALING_PATHWAY	GO:2001233	10	-0.678609	-1.761606	0.004392	0.289781	0.911
711	GOBP_CELLULAR_RESPONSE_TO_REACTIVE_OXYGEN_SPECIES	GO:0034614	7	-0.761052	-1.780140	0.007452	0.265944	0.864
712	GOBP_LEUKOCYTE_DIFFERENTIATION	GO:0002521	15	-0.630362	-1.813362	0.002721	0.204134	0.724
713	GOBP_RESPONSE_TO_CADMIUM_ION	GO:0046686	6	-0.843411	-1.826616	0.000000	0.206032	0.673
714	GOBP_POSITIVE_REGULATION_OF_NUCLEOBASE_CONTAIN...	GO:0045935	28	-0.551661	-1.838182	0.003861	0.209858	0.614
715	GOBP_MYELOID_LEUKOCYTE_DIFFERENTIATION	GO:0002573	11	-0.696257	-1.846048	0.001406	0.237667	0.583
716	GOBP_MYELOID_CELL_DIFFERENTIATION	GO:0030099	13	-0.689421	-1.893598	0.001379	0.180309	0.396
717	REACTOME_INNATE_IMMUNE_SYSTEM	R-RNO-168249	16	-0.639921	-1.899566	0.001372	0.253494	0.376
718	GOBP_PLATELET_ACTIVATION	GO:0030168	6	-0.862601	-1.917365	0.000000	0.399732	0.305

End of Table

Supplementary Table S15: BG early profile (8 DPL peak) GSEA results.

Begin of Table **S15**

	NAME	ID	SIZE	ES	NES	NOM.p.val	FDR.q.val	FWER.p.val
0	GOBP_MEMORY	GO:0007613	13	0.586962	2.102377	0.000000	0.625162	0.373
1	GOBP_CATION_TRANSPORT	GO:0006812	25	0.421588	1.803097	0.007067	1.000000	0.973
2	GOBP_NERVE_DEVELOPMENT	GO:0021675	9	0.565701	1.733075	0.014925	1.000000	0.993
3	REACTOME_SIGNALING_BY_NTRK2_TRKB	R-RNO-9006115	6	0.662655	1.713326	0.037037	1.000000	0.996
4	GOBP_POSITIVE_REGULATION_OF_LIPASE_ACTIVITY	GO:0060193	5	0.698339	1.702445	0.029326	1.000000	0.998
5	GOBP_NEGATIVE_REGULATION_OF_NEURON_APOPTOTIC_P...	GO:0043524	8	0.565633	1.682155	0.031447	1.000000	0.999
6	GOBP_REGULATION_OF_PHOSPHOLIPASE_C_ACTIVITY	GO:1900274	5	0.698339	1.677069	0.027778	1.000000	0.999
7	GOBP_SYNAPTIC_SIGNALING	GO:0099536	47	0.337414	1.628808	0.020101	1.000000	1.000
8	GOBP_BEHAVIOR	GO:0007610	32	0.353545	1.625855	0.013636	1.000000	1.000
9	GOBP_REGULATION_OF_NEURON_PROJECTION_DEVELOPMENT	GO:0010975	16	0.431813	1.603620	0.042105	1.000000	1.000
10	GOBP_REGULATION_OF_PHOSPHOLIPASE_ACTIVITY	GO:0010517	6	0.613173	1.591696	0.040984	1.000000	1.000
11	GOBP_RESPONSE_TO_NERVE_GROWTH_FACTOR	GO:1990089	7	0.592249	1.590288	0.050000	1.000000	1.000
12	GOBP_REGULATION_OF_LIPASE_ACTIVITY	GO:0060191	6	0.613173	1.582726	0.063768	1.000000	1.000
13	GOBP_NEUROTROPHIN_SIGNALING_PATHWAY	GO:0038179	6	0.594194	1.574801	0.048991	1.000000	1.000
14	GOBP_NEURON_DEVELOPMENT	GO:0048666	33	0.339039	1.563936	0.026217	1.000000	1.000
15	GOBP_CELL_CELL_SIGNALING	GO:0007267	56	0.331361	1.553680	0.009174	1.000000	1.000
16	GOBP_NEUROTROPHIN_TRK_RECEPTOR_SIGNALING_PATHWAY	GO:0048011	6	0.594194	1.553286	0.055556	0.946455	1.000
17	GOBP_REGULATION_OF_TRANS_SYNAPTIC_SIGNALING	GO:0099177	40	0.327914	1.544139	0.021186	0.929174	1.000
18	GOBP_NERVOUS_SYSTEM_PROCESS	GO:0050877	38	0.317309	1.542059	0.013216	0.887685	1.000
19	GOBP_REGULATION_OF_PEPTIDYL_SERINE_PHOSPHORYLA...	GO:0033135	8	0.519312	1.541639	0.072555	0.844781	1.000
20	GOBP_REGULATION_OF_NMDA_RECEPTOR_ACTIVITY	GO:2000310	10	0.475723	1.522818	0.057692	0.874604	1.000
21	GOBP_LONG_TERM_MEMORY	GO:0007616	6	0.564900	1.520606	0.065903	0.841472	1.000
22	GOBP_POSITIVE_REGULATION_OF_PEPTIDYL_SERINE_PH...	GO:0033138	8	0.519312	1.519085	0.095238	0.809561	1.000
23	GOBP_REGULATION_OF_NEURON_DIFFERENTIATION	GO:0045664	5	0.626381	1.508076	0.077957	0.814428	1.000
24	GOBP_NEURON_DIFFERENTIATION	GO:0030182	36	0.324184	1.501531	0.048673	0.802127	1.000
25	GOBP_POSITIVE_REGULATION_OF_LONG_TERM_SYNAPTIC...	GO:1900273	5	0.631234	1.500513	0.082888	0.775103	1.000
26	GOBP_NEGATIVE_REGULATION_OF_NEURON_DEATH	GO:1901215	11	0.450046	1.496745	0.049342	0.758595	1.000
27	GOBP_REGULATION_OF_SYNAPSE_STRUCTURE_OR_ACTIVITY	GO:0050803	11	0.454545	1.488265	0.055728	0.760855	1.000
28	GOBP_REGULATION_OF_ION_TRANSPORT	GO:0043269	36	0.313378	1.475224	0.049020	0.778360	1.000
29	GOBP_POSITIVE_REGULATION_OF_PHOSPHORUS_METABOL...	GO:0010562	24	0.342758	1.469525	0.044715	0.770544	1.000
30	GOBP_COGNITION	GO:0050890	28	0.328435	1.443552	0.047101	0.836379	1.000
31	HALLMARK_KRAS_SIGNALING_UP	M5953	6	0.530501	1.436866	0.075581	0.834213	1.000
32	GOBP_POSITIVE_REGULATION_OF_TRANSMEMBRANE_TRAN...	GO:0034764	7	0.515390	1.435338	0.089385	0.814059	1.000
33	GOBP_REGULATION_OF_TRANSPORT	GO:0051049	44	0.293922	1.413607	0.053922	0.867050	1.000
34	GOBP_REGULATION_OF_CATION_TRANSMEMBRANE_TRANSPORT	GO:1904062	16	0.376954	1.395064	0.111511	0.910023	1.000
35	GOBP_POSITIVE_REGULATION_OF_NEURON_PROJECTION_...	GO:0010976	5	0.573435	1.394029	0.107817	0.888373	1.000
36	GOBP_REGULATION_OF_CELL_PROJECTION_ORGANIZATION	GO:0031344	20	0.350048	1.393083	0.082353	0.868397	1.000
37	GOBP_REGULATION_OF_PROTEIN_PHOSPHORYLATION	GO:0001932	26	0.308394	1.381989	0.084677	0.887928	1.000
38	GOBP_TRANSMEMBRANE_RECEPTOR_PROTEIN_TYROSINE_K...	GO:0007169	19	0.336086	1.365265	0.091549	0.927280	1.000
39	GOBP_POSITIVE_REGULATION_OF_PEPTIDYL_TYROSINE_...	GO:0050731	8	0.462500	1.363957	0.133127	0.908630	1.000
40	GOBP_NEGATIVE_REGULATION_OF_CATALYTIC_ACTIVITY	GO:0043086	17	0.336174	1.349111	0.107807	0.943980	1.000

Continuation of Table **S15**

	NAME	ID	SIZE	ES	NES	NOM.p.val	FDR.q.val	FWER.p.val
41	GOBP_REGULATION_OF_LONG_TERM_SYNAPTIC_POTENTIA...	GO:1900271	6	0.526804	1.345409	0.151335	0.935476	1.000
42	GOBP_POSITIVE_REGULATION_OF_TRANSFERASE_ACTIVITY	GO:0051347	20	0.320449	1.317525	0.124481	1.000000	1.000
43	GOBP_POSITIVE_REGULATION_OF_SYNAPTIC_TRANSMISSION	GO:0050806	15	0.346662	1.315701	0.138047	1.000000	1.000
44	GOBP_CELL_PROJECTION_ORGANIZATION	GO:0030030	34	0.288877	1.313954	0.097778	0.994272	1.000
45	REACTOME_ACTIVATION_OF_NMDA_RECEPTORS_AND_POST...	R-RNO-442755	16	0.344284	1.307321	0.108696	0.998971	1.000
46	GOBP_POSITIVE_REGULATION_OF_HYDROLASE_ACTIVITY	GO:0051345	16	0.343225	1.306930	0.131034	0.978962	1.000
47	GOBP_NEGATIVE_REGULATION_OF_PEPTIDASE_ACTIVITY	GO:0010466	6	0.502978	1.299203	0.158358	0.988251	1.000
48	GOBP_POSITIVE_REGULATION_OF_CATALYTIC_ACTIVITY	GO:0043085	30	0.283846	1.295197	0.116183	0.982978	1.000
49	GOBP_NEUROGENESIS	GO:0022008	40	0.276123	1.287505	0.106481	0.990969	1.000
50	HALLMARK_PI3K_AKT_MTOR_SIGNALING	M5923	6	0.490942	1.280965	0.184438	0.996258	1.000
51	GOBP_CATION_TRANSMEMBRANE_TRANSPORT	GO:0098655	18	0.331029	1.279110	0.162630	0.984469	1.000
52	GOBP_REGULATION_OF_CATION_CHANNEL_ACTIVITY	GO:2001257	14	0.353197	1.270461	0.175862	0.998630	1.000
53	GOBP_PERIPHERAL_NERVOUS_SYSTEM_DEVELOPMENT	GO:0007422	9	0.411433	1.264759	0.170213	1.000000	1.000
54	GOBP_NEGATIVE_REGULATION_OF_CYSTEINE_TYPE_ENDO...	GO:2000117	5	0.513106	1.261071	0.165746	0.998356	1.000
55	GOBP_POSITIVE_REGULATION_OF_CELL_PROJECTION_OR...	GO:0031346	10	0.398549	1.260058	0.179710	0.984178	1.000
56	GOBP_REGULATION_OF_NEUROTRANSMITTER_RECEPTOR_A...	GO:0099601	13	0.356768	1.255446	0.160839	0.983494	1.000
57	GOBP_POSITIVE_REGULATION_OF_PROTEIN_MODIFICATI...	GO:0031401	23	0.298172	1.254352	0.167347	0.970161	1.000
58	GOBP_SYNAPSE_ORGANIZATION	GO:0050808	17	0.323054	1.251861	0.185714	0.963410	1.000
59	GOBP_REGULATION_OF_HORMONE_LEVELS	GO:0010817	12	0.358843	1.241763	0.188679	0.985164	1.000
60	REACTOME_UNBLOCKING_OF_NMDA_RECEPTORS_GLUTAMAT...	R-RNO-438066	12	0.348542	1.235293	0.192308	0.993437	1.000
61	GOBP_DEFENSE_RESPONSE	GO:0006952	19	0.307890	1.233504	0.179931	0.984065	1.000
62	GOBP_POSITIVE_REGULATION_OF_CATION_TRANSMEMBRA...	GO:1904064	5	0.502387	1.227369	0.184282	0.991750	1.000
63	REACTOME_LONG_TERM_POTENTIATION	R-HSA-9620244	10	0.384855	1.226857	0.195783	0.978042	1.000
64	GOBP_AXON_DEVELOPMENT	GO:0061564	18	0.312077	1.225383	0.184502	0.967772	1.000
65	GOBP_REGULATION_OF_PHOSPHORUS_METABOLIC_PROCESS	GO:0051174	27	0.285357	1.225307	0.185520	0.953309	1.000
66	GOBP_POSITIVE_REGULATION_OF_PROTEIN_PHOSPHORYL...	GO:0001934	23	0.298172	1.221312	0.205761	0.953216	1.000
67	GOBP_REGULATION_OF_MEMBRANE_POTENTIAL	GO:0042391	17	0.318722	1.216002	0.219231	0.956842	1.000
68	GOBP_PEPTIDYL_TYROSINE_MODIFICATION	GO:0018212	10	0.385097	1.214244	0.210031	0.949391	1.000
69	GOBP_RECEPTOR_METABOLIC_PROCESS	GO:0043112	5	0.482779	1.204479	0.190981	0.968869	1.000
70	GOBP_SENSORY_PERCEPTION	GO:0007600	9	0.397368	1.200400	0.216617	0.969000	1.000
71	GOBP_SMALL_GTPASE_MEDIATED_SIGNAL_TRANSDUCTION	GO:0007264	8	0.398566	1.192449	0.234568	0.985311	1.000
72	GOBP_REGULATION_OF_TRANSFERASE_ACTIVITY	GO:0051338	22	0.278565	1.189361	0.210970	0.982882	1.000
73	REACTOME_TRANSCRIPTIONAL_REGULATION_BY_MECP2	R-RNO-8986944	7	0.422698	1.188313	0.255014	0.973254	1.000
74	GOBP_REGULATION_OF_SIGNALING_RECEPTOR_ACTIVITY	GO:0010469	14	0.323774	1.188187	0.243506	0.960856	1.000
75	GOBP_REGULATION_OF_POSTSYNAPTIC_MEMBRANE_POTEN...	GO:0060078	14	0.332216	1.185434	0.227891	0.958073	1.000
76	GOBP_ION_HOMEOSTASIS	GO:0050801	16	0.319886	1.183774	0.250784	0.951084	1.000
77	GOBP_INSULIN_SECRETION	GO:0030073	7	0.430593	1.180256	0.267606	0.951314	1.000
78	GOBP_PROTEIN_PHOSPHORYLATION	GO:0006468	34	0.251425	1.179764	0.239669	0.940795	1.000
79	GOBP_PEPTIDE_HORMONE_SECRETION	GO:0030072	7	0.430593	1.178769	0.271053	0.932536	1.000
80	GOBP_REGULATION_OF_INTRACELLULAR_SIGNAL_TRANSD...	GO:1902531	21	0.289288	1.176046	0.219331	0.930137	1.000
81	GOBP_NEURON_PROJECTION_ORGANIZATION	GO:0106027	8	0.387500	1.173090	0.254777	0.927808	1.000
82	GOBP_ACTIVATION_OF_PROTEIN_KINASE_ACTIVITY	GO:0032147	11	0.345323	1.166073	0.264407	0.939484	1.000
83	GOBP_ORGANOPHOSPHATE_METABOLIC_PROCESS	GO:0019637	7	0.414705	1.165045	0.279279	0.931595	1.000

Continuation of Table **S15**

	NAME	ID	SIZE	ES	NES	NOM.p.val	FDR.q.val	FWER.p.val
84	GOBP_NEUROINFLAMMATORY_RESPONSE	GO:0150076	5	0.479492	1.164680	0.275660	0.921762	1.000
85	GOBP_REGULATION_OF_PROTEIN_KINASE_ACTIVITY	GO:0045859	22	0.278565	1.158781	0.260700	0.931319	1.000
86	GOBP_TRANSMEMBRANE_TRANSPORT	GO:0055085	27	0.263346	1.158768	0.250000	0.920647	1.000
87	GOBP_INOSITOL_LIPID_MEDIATED_SIGNALING	GO:0048017	8	0.398535	1.157872	0.264797	0.912807	1.000
88	GOBP_REGULATION_OF_ANION_TRANSMEMBRANE_TRANSPORT	GO:1903959	5	0.457831	1.156713	0.255495	0.906300	1.000
89	GOBP_RECEPTOR_INTERNALIZATION	GO:0031623	5	0.482779	1.154286	0.253561	0.904133	1.000
90	GOBP_POSITIVE_REGULATION_OF_CELL_GROWTH	GO:0030307	6	0.443102	1.147331	0.270195	0.917229	1.000
91	GOBP_LEUKOCYTE_MIGRATION	GO:0050900	6	0.439024	1.143018	0.287390	0.921876	1.000
92	GOBP_REGULATION_OF_TRANSMEMBRANE_TRANSPORT	GO:0034762	23	0.270006	1.142541	0.256098	0.913708	1.000
93	GOBP_CARBOHYDRATE_DERIVATIVE_METABOLIC_PROCESS	GO:1901135	7	0.416946	1.136472	0.275281	0.923102	1.000
94	GOBP_ERBB_SIGNALING_PATHWAY	GO:0038127	5	0.464265	1.133795	0.277922	0.921966	1.000
95	GOBP_PEPTIDE_SECRETION	GO:0002790	9	0.371115	1.131023	0.265244	0.921237	1.000
96	GOBP_DENDRITE_MORPHOGENESIS	GO:0048813	6	0.426829	1.130645	0.273481	0.912858	1.000
97	REACTOME_ONCOGENIC_MAPK_SIGNALING	R-HSA-6802957	5	0.471383	1.124776	0.291444	0.922290	1.000
98	GOBP_INFLAMMATORY_RESPONSE	GO:0006954	12	0.331357	1.122780	0.283077	0.919544	1.000
99	GOBP_REGULATION_OF_CYTOSOLIC_CALCIUM_ION_CONCE...	GO:0051480	13	0.318126	1.115304	0.306931	0.934062	1.000
100	GOBP_SYNAPTIC_TRANSMISSION_GLUTAMATERGIC	GO:0035249	13	0.311801	1.113963	0.305031	0.929455	1.000
101	GOBP_CELLULAR_MACROMOLECULE_LOCALIZATION	GO:0070727	21	0.268657	1.107370	0.324042	0.941316	1.000
102	GOBP_DIVALENT_INORGANIC_CATION_HOMEOSTASIS	GO:0072507	13	0.318126	1.102203	0.300353	0.948280	1.000
103	GOBP_REGULATION_OF_POSTSYNAPSE_ORGANIZATION	GO:0099175	6	0.426829	1.101668	0.315493	0.941231	1.000
104	GOBP_PEPTIDYL_SERINE_MODIFICATION	GO:0018209	12	0.321562	1.095198	0.328720	0.953425	1.000
105	GOBP_CELL_MORPHOGENESIS	GO:0000902	24	0.252174	1.095061	0.321970	0.944877	1.000
106	GOBP_CELLULAR_RESPONSE_TO_KETONE	GO:1901655	5	0.450174	1.094522	0.332454	0.937640	1.000
107	GOBP_REGULATION_OF_PROTEIN_TYROSINE_KINASE_ACT...	GO:0061097	5	0.445783	1.092863	0.309524	0.934014	1.000
108	GOBP_CELLULAR_COMPONENT_MORPHOGENESIS	GO:0032989	24	0.252174	1.091624	0.318519	0.929815	1.000
109	GOBP_REGULATION_OF_AXONOGENESIS	GO:0050770	7	0.395062	1.085877	0.331429	0.938819	1.000
110	GOBP_REGULATION_OF_PROTEIN_MODIFICATION_PROCESS	GO:0031399	29	0.244284	1.085133	0.331967	0.932642	1.000
111	GOBP_POSITIVE_REGULATION_OF_TRANSPORT	GO:0051050	26	0.248082	1.084306	0.323144	0.926742	1.000
112	GOBP_CELLULAR_ION_HOMEOSTASIS	GO:0006873	14	0.302520	1.083749	0.335463	0.920401	1.000
113	GOBP_DENDRITIC_SPINE_DEVELOPMENT	GO:0060996	6	0.426829	1.081383	0.352025	0.919669	1.000
114	GOBP_REGULATION_OF_SYNAPTIC_TRANSMISSION_GLUTA...	GO:0051966	12	0.313965	1.079286	0.343653	0.917671	1.000
115	GOBP_POSITIVE_REGULATION_OF_SIGNALING	GO:0023056	34	0.227816	1.078752	0.337719	0.911041	1.000
116	REACTOME_ANTI_INFLAMMATORY_RESPONSE_FAVOURING_...	R-HSA-9662851	5	0.442468	1.078693	0.371274	0.903369	1.000
117	GOBP_POSITIVE_REGULATION_OF_MOLECULAR_FUNCTION	GO:0044093	38	0.227800	1.077548	0.384977	0.899165	1.000
118	GOBP_CELL_PART_MORPHOGENESIS	GO:0032990	24	0.252174	1.076980	0.347985	0.893424	1.000
119	GOBP_LONG_TERM_SYNAPTIC_POTENTIATION	GO:0060291	12	0.315452	1.076076	0.316993	0.889058	1.000
120	GOBP_DENDRITIC_SPINE_MORPHOGENESIS	GO:0060997	5	0.442226	1.069899	0.344444	0.899395	1.000
121	GOBP_POSITIVE_REGULATION_OF_PROTEIN_TYROSINE_K...	GO:0061098	5	0.445783	1.068143	0.352770	0.896931	1.000
122	GOBP_G_PROTEIN_COUPLED_RECEPTOR_SIGNALING_PATHWAY	GO:0007186	18	0.267645	1.066953	0.361940	0.892892	1.000
123	GOBP_POSITIVE_REGULATION_OF_PROTEIN_KINASE_ACT...	GO:0045860	19	0.269296	1.066930	0.309701	0.885755	1.000
124	GOBP_CHEMICAL_HOMEOSTASIS	GO:0048878	23	0.254702	1.064461	0.348276	0.885741	1.000
125	GOBP_NEURON_APOPTOTIC_PROCESS	GO:0051402	12	0.314215	1.063549	0.338762	0.881176	1.000
126	GOBP_NEGATIVE_REGULATION_OF_TRANSMEMBRANE_TRAN...	GO:0034763	6	0.415659	1.062069	0.371274	0.878158	1.000

Continuation of Table **S15**

	NAME	ID	SIZE	ES	NES	NOM.p.val	FDR.q.val	FWER.p.val
127	GOBP_REGULATION_OF_HYDROLASE_ACTIVITY	GO:0051336	22	0.254058	1.057424	0.364662	0.885089	1.000
128	GOBP_SENSORY_ORGAN_DEVELOPMENT	GO:0007423	8	0.352899	1.052645	0.386997	0.892513	1.000
129	GOBP_REGULATION_OF_RESPONSE_TO_STRESS	GO:0080134	19	0.264390	1.046480	0.385185	0.902969	1.000
130	GOBP_CELLULAR_RESPONSE_TO_ALCOHOL	GO:0097306	5	0.450355	1.045421	0.373595	0.899036	1.000
131	GOBP_REGULATION_OF_CELL_SIZE	GO:0008361	5	0.433987	1.045017	0.346253	0.893491	1.000
132	GOBP_PEPTIDYL_AMINO_ACID_MODIFICATION	GO:0018193	22	0.252041	1.042614	0.418605	0.893309	1.000
133	GOBP_PROTEIN_LOCALIZATION_TO_ORGANELLE	GO:0033365	9	0.341772	1.036763	0.380645	0.903611	1.000
134	GOBP_CARBOHYDRATE_HOMEOSTASIS	GO:0033500	7	0.375534	1.033026	0.400000	0.907923	1.000
135	GOBP_NEURON_PROJECTION_GUIDANCE	GO:0097485	9	0.338754	1.031171	0.381098	0.906215	1.000
136	GOBP_RAS_PROTEIN_SIGNAL_TRANSDUCTION	GO:0007265	5	0.425330	1.030746	0.394256	0.900849	1.000
137	GOBP_POSITIVE_REGULATION_OF_CELLULAR_COMPONENT...	GO:0051130	24	0.240645	1.028609	0.406716	0.900403	1.000
138	GOBP_RESPONSE_TO_HEAT	GO:0009408	7	0.359033	1.018019	0.406332	0.923290	1.000
139	GOBP_POSITIVE_REGULATION_OF_DEVELOPMENTAL_GROWTH	GO:0048639	8	0.340391	1.008511	0.414474	0.943312	1.000
140	GOBP_POSITIVE_REGULATION_OF_CELLULAR_PROTEIN_L...	GO:1903829	7	0.358025	0.999044	0.428962	0.964263	1.000
141	GOBP_NEGATIVE_REGULATION_OF_ION_TRANSPORT	GO:0043271	11	0.303419	0.994363	0.442953	0.970867	1.000
142	GOBP_METAL_ION_HOMEOSTASIS	GO:0055065	14	0.269796	0.987987	0.467320	0.982499	1.000
143	REACTOME_VESICLE_MEDIATED_TRANSPORT	R-RNO-5653656	5	0.407940	0.985816	0.428177	0.981701	1.000
144	GOBP_POSITIVE_REGULATION_OF_DNA_BINDING_TRANSC...	GO:0051091	10	0.308924	0.984774	0.440972	0.978320	1.000
145	GOBP_POSITIVE_REGULATION_OF_PROTEIN_METABOLIC_...	GO:0051247	28	0.220289	0.982415	0.466667	0.978120	1.000
146	GOBP_POSITIVE_REGULATION_OF_ION_TRANSPORT	GO:0043270	17	0.256057	0.978768	0.454545	0.982328	1.000
147	GOBP_NEGATIVE_REGULATION_OF_SYNAPTIC_TRANSMISSION	GO:0050805	6	0.378630	0.977581	0.444759	0.978852	1.000
148	REACTOME_TRANSMISSION_ACROSS_CHEMICAL_SYNAPSES	R-RNO-112315	23	0.231832	0.974077	0.513410	0.982237	1.000
149	GOBP_DEVELOPMENTAL_CELL_GROWTH	GO:0048588	5	0.410743	0.973896	0.451351	0.976205	1.000
150	GOBP_POSITIVE_REGULATION_OF_SECRETION	GO:0051047	6	0.370498	0.973735	0.498623	0.970138	1.000
151	GOBP_POLYSACCHARIDE_METABOLIC_PROCESS	GO:0005976	5	0.410612	0.972679	0.500000	0.966567	1.000
152	GOBP_SENSORY_PERCEPTION_OF_PAIN	GO:0019233	5	0.404815	0.971314	0.452778	0.964141	1.000
153	GOBP_NEGATIVE_REGULATION_OF_CELL_DEATH	GO:0060548	22	0.235332	0.970019	0.452632	0.961146	1.000
154	GOBP_REGULATION_OF_TRANSPORTER_ACTIVITY	GO:0032409	17	0.254705	0.969205	0.506211	0.957279	1.000
155	GOBP_TELENCEPHALON_DEVELOPMENT	GO:0021537	6	0.373313	0.968221	0.460177	0.953511	1.000
156	GOBP_ORGAN_GROWTH	GO:0035265	5	0.397590	0.964999	0.488372	0.956633	1.000
157	REACTOME_MEMBRANE_TRAFFICKING	R-RNO-199991	5	0.407940	0.964058	0.479564	0.953360	1.000
158	GOBP_RECEPTOR_MEDIATED_ENDOCYTOSIS	GO:0006898	6	0.365437	0.961877	0.513889	0.953675	1.000
159	GOBP_HORMONE_TRANSPORT	GO:0009914	9	0.304349	0.961550	0.469697	0.948517	1.000
160	GOBP_NEGATIVE_REGULATION_OF_PHOSPHORYLATION	GO:0042326	7	0.335472	0.955856	0.511299	0.957641	1.000
161	GOBP_REGULATION_OF_NEURONAL_SYNAPTIC_PLASTICITY	GO:0048168	7	0.345679	0.955195	0.488372	0.953786	1.000
162	GOBP_REGULATION_OF_ORGAN_GROWTH	GO:0046620	5	0.397590	0.952337	0.510870	0.955254	1.000
163	GOBP_REGULATION_OF_PEPTIDYL_TYROSINE_PHOSPHORY...	GO:0050730	9	0.307995	0.948712	0.506536	0.959750	1.000
164	GOBP_ADENYLATE_CYCLASE_MODULATING_G_PROTEIN_CO...	GO:0007188	11	0.281481	0.948564	0.503425	0.954287	1.000
165	GOBP_GENERATION_OF_PRECURSOR_METABOLITES_AND_E...	GO:0006091	5	0.396848	0.947667	0.491979	0.950660	1.000
166	GOBP_POSITIVE_REGULATION_OF_AXONOGENESIS	GO:0050772	5	0.385542	0.945480	0.492837	0.950859	1.000
167	GOBP_REGULATION_OF_DEVELOPMENTAL_GROWTH	GO:0048638	9	0.297021	0.938085	0.521173	0.964712	1.000
168	GOBP_MITOCHONDRION_ORGANIZATION	GO:0007005	7	0.339085	0.937404	0.497024	0.960953	1.000
169	GOBP_CELL_JUNCTION_ORGANIZATION	GO:0034330	19	0.234019	0.934708	0.547101	0.962515	1.000

Continuation of Table **S15**

	NAME	ID	SIZE	ES	NES	NOM.p.val	FDR.q.val	FWER.p.val
170	GOBP_NEGATIVE_REGULATION_OF_LIPID_METABOLIC_PR...	GO:0045833	5	0.394411	0.934026	0.518841	0.958576	1.000
171	GOBP_POSITIVE_REGULATION_OF_BINDING	GO:0051099	6	0.355724	0.933076	0.507246	0.955281	1.000
172	GOBP_POSITIVE_REGULATION_OF_LOCOMOTION	GO:0040017	11	0.282560	0.932979	0.527687	0.950026	1.000
173	GOBP_SMALL_MOLECULE_METABOLIC_PROCESS	GO:0044281	15	0.255727	0.932883	0.546358	0.944808	1.000
174	GOBP_AMIDE_BIOSYNTHETIC_PROCESS	GO:0043604	7	0.333333	0.932368	0.543103	0.940606	1.000
175	GOBP_PEPTIDE_BIOSYNTHETIC_PROCESS	GO:0043043	7	0.333333	0.926905	0.498599	0.949502	1.000
176	GOBP_REGULATION_OF_SYNAPTIC_PLASTICITY	GO:0048167	21	0.218710	0.925628	0.616279	0.947459	1.000
177	GOBP_LONG_TERM_SYNAPTIC_DEPRESSION	GO:0060292	5	0.395899	0.924260	0.541114	0.945615	1.000
178	GOBP_MUSCLE_CELL_DEVELOPMENT	GO:0055001	5	0.385542	0.915145	0.531856	0.961820	1.000
179	GOBP_RESPONSE_TO_CARBOHYDRATE	GO:0009743	5	0.385542	0.913011	0.542936	0.961964	1.000
180	GOBP_DEVELOPMENTAL_MATURATION	GO:0021700	5	0.373494	0.908876	0.564033	0.967152	1.000
181	GOBP_REGULATION_OF_CYTOSKELETON_ORGANIZATION	GO:0051493	5	0.373494	0.907331	0.557692	0.965531	1.000
182	GOBP_RESPONSE_TO_MONOSACCHARIDE	GO:0034284	5	0.385542	0.905847	0.536458	0.963827	1.000
183	GOBP_APOPTOTIC_PROCESS	GO:0006915	35	0.193760	0.903012	0.636752	0.965401	1.000
184	GOBP_CELLULAR_RESPONSE_TO_ORGANIC_CYCLIC_COMPOUND	GO:0071407	13	0.253333	0.902028	0.573333	0.962511	1.000
185	GOBP_MATERNAL_PROCESS_INVOLVED_IN_FEMALE_PREGN...	GO:0060135	5	0.360141	0.899742	0.568783	0.963086	1.000
186	GOBP_POSITIVE_REGULATION_OF_CELL_DEVELOPMENT	GO:0010720	12	0.259269	0.899731	0.557522	0.957958	1.000
187	GOBP_POSITIVE_REGULATION_OF_NEUROGENESIS	GO:0050769	12	0.259269	0.899265	0.590361	0.953830	1.000
188	GOBP_POSITIVE_REGULATION_OF_NERVOUS_SYSTEM_DEV...	GO:0051962	12	0.259269	0.898636	0.615132	0.950226	1.000
189	GOBP_REGULATION_OF_NERVOUS_SYSTEM_DEVELOPMENT	GO:0051960	15	0.241474	0.898195	0.590580	0.946213	1.000
190	GOBP_LOCOMOTORY_BEHAVIOR	GO:0007626	7	0.318211	0.895599	0.605341	0.947725	1.000
191	GOBP_ENDOCYTOSIS	GO:0006897	8	0.311423	0.890978	0.579251	0.954650	1.000
192	GOBP_RESPONSE_TO_ALKALOID	GO:0043279	5	0.367966	0.890768	0.578947	0.950124	1.000
193	GOBP_NEGATIVE_REGULATION_OF_SIGNALING	GO:0023057	21	0.216297	0.887056	0.641975	0.953204	1.000
194	GOBP_REGIONALIZATION	GO:0003002	6	0.347957	0.885320	0.555556	0.952450	1.000
195	GOBP_CELLULAR_LIPID_METABOLIC_PROCESS	GO:0044255	7	0.315690	0.882531	0.586826	0.954271	1.000
196	REACTOME_RAS_ACTIVATION_UPON_CA2_INFLUX_THROUG...	R-HSA-442982	6	0.338946	0.880258	0.603352	0.954814	1.000
197	GOBP_POSITIVE_REGULATION_OF_EPITHELIAL_CELL_MI...	GO:0010634	5	0.365226	0.877313	0.592208	0.957150	1.000
198	REACTOME_TRAFFICKING_OF_AMPA_RECEPTORS	R-RNO-399719	11	0.266731	0.874100	0.604027	0.959434	1.000
199	GOBP_TAXIS	GO:0042330	14	0.236919	0.873412	0.649123	0.956033	1.000
200	GOBP_POSITIVE_REGULATION_OF_ESTABLISHMENT_OF_P...	GO:1904951	5	0.349398	0.862803	0.634006	0.975384	1.000
201	GOBP_NEGATIVE_REGULATION_OF_IMMUNE_SYSTEM_PROCESS	GO:0002683	5	0.361673	0.858712	0.601626	0.979823	1.000
202	GOBP_EXOCYTOSIS	GO:0006887	11	0.257296	0.858165	0.665493	0.976274	1.000
203	GOBP_CELL_GROWTH	GO:0016049	10	0.269231	0.857107	0.663664	0.974019	1.000
204	GOBP_PATTERN_SPECIFICATION_PROCESS	GO:0007389	6	0.347957	0.855514	0.587896	0.973106	1.000
205	GOBP_MONOCARBOXYLIC_ACID_METABOLIC_PROCESS	GO:0032787	6	0.318527	0.854511	0.625000	0.970685	1.000
206	REACTOME_NEUROTRANSMITTER_RECEPTORS_AND_POSTSY...	R-RNO-112314	22	0.207947	0.850462	0.752852	0.975242	1.000
207	REACTOME_G_ALPHA_I_SIGNALLING_EVENTS	R-RNO-418594	18	0.221461	0.850117	0.684397	0.971298	1.000
208	GOBP_MYELOID_LEUKOCYTE_ACTIVATION	GO:0002274	8	0.286177	0.846631	0.662614	0.974352	1.000
209	GOBP_SEXUAL_REPRODUCTION	GO:0019953	10	0.269231	0.843937	0.670554	0.975632	1.000
210	GOBP_MULTICELLULAR_ORGANISMAL_RESPONSE_TO_STRESS	GO:0033555	5	0.350514	0.842098	0.645946	0.975001	1.000
211	GOBP_REGULATION_OF_PROTEIN_LOCALIZATION	GO:0032880	18	0.212244	0.838529	0.687747	0.978205	1.000
212	GOBP_RESPONSE_TO_ALCOHOL	GO:0097305	11	0.252925	0.835988	0.677419	0.978964	1.000

Continuation of Table **S15**

	NAME	ID	SIZE	ES	NES	NOM.p.val	FDR.q.val	FWER.p.val
213	GOBP_POSITIVE_REGULATION_OF_GROWTH	GO:0045927	9	0.276808	0.835767	0.683386	0.974867	1.000
214	GOBP_ESTABLISHMENT_OF_PROTEIN_LOCALIZATION_TO_...	GO:0072594	6	0.329268	0.835500	0.660969	0.970988	1.000
215	GOBP_REGULATION_OF_CELLULAR_PROTEIN_LOCALIZATION	GO:1903827	12	0.250000	0.832545	0.688136	0.972744	1.000
216	GOBP_DEVELOPMENTAL_GROWTH	GO:0048589	10	0.258477	0.832207	0.723810	0.968875	1.000
217	GOBP_CELL_CELL_SIGNALING_BY_WNT	GO:0198738	7	0.296296	0.823581	0.656151	0.981719	1.000
218	GOBP_NEGATIVE_REGULATION_OF_TRANSPORT	GO:0051051	13	0.233166	0.822824	0.692053	0.978871	1.000
219	GOBP_CARBOHYDRATE_METABOLIC_PROCESS	GO:0005975	9	0.265823	0.822407	0.669697	0.975404	1.000
220	GOBP_EMBRYONIC_MORPHOGENESIS	GO:0048598	6	0.305481	0.821845	0.647383	0.972196	1.000
221	GOBP_PROTEIN_LOCALIZATION_TO_CELL_PERIPHERY	GO:1990778	12	0.236842	0.814754	0.720000	0.983078	1.000
222	GOBP_NUCLEAR_TRANSPORT	GO:0051169	5	0.337349	0.813810	0.685792	0.980459	1.000
223	GOBP_IONOTROPIC_GLUTAMATE_RECEPTOR_SIGNALING_P...	GO:0035235	9	0.267109	0.807978	0.730897	0.988804	1.000
224	GOBP_REGULATION_OF_ANATOMICAL_STRUCTURE_MORPHO...	GO:0022603	17	0.209805	0.807023	0.719425	0.986574	1.000
225	GOBP_REGULATION_OF_PEPTIDE_HORMONE_SECRETION	GO:0090276	6	0.308128	0.802247	0.701705	0.991283	1.000
226	GOBP_GAMETE_GENERATION	GO:0007276	9	0.265823	0.801765	0.718310	0.987654	1.000
227	GOBP_CELLULAR_HOMEOSTASIS	GO:0019725	18	0.204987	0.800822	0.756554	0.985030	1.000
228	GOBP_REGULATION_OF_CELLULAR_RESPONSE_TO_STRESS	GO:0080135	9	0.260908	0.800268	0.758730	0.981711	1.000
229	GOBP_PHOSPHATIDYLINOSITOL_3_KINASE_SIGNALING	GO:0014065	7	0.291107	0.799913	0.715100	0.978006	1.000
230	GOBP_NEGATIVE_REGULATION_OF_PROTEOLYSIS	GO:0045861	8	0.271355	0.793693	0.699088	0.985918	1.000
231	GOBP_REGULATION_OF_PEPTIDE_SECRETION	GO:0002791	7	0.284243	0.792518	0.698925	0.984037	1.000
232	GOBP_REGULATION_OF_CELLULAR_LOCALIZATION	GO:0060341	17	0.205774	0.791055	0.773381	0.982720	1.000
233	GOBP_REGULATION_OF_MUSCLE_ADAPTATION	GO:0043502	5	0.330002	0.790020	0.730245	0.980536	1.000
234	GOBP_RESPONSE_TO_ETHANOL	GO:0045471	5	0.333807	0.786552	0.716180	0.983302	1.000
235	GOBP_REGULATION_OF_CYSTEINE_TYPE_ENDOPEPTIDASE...	GO:2000116	9	0.248969	0.781194	0.766355	0.989686	1.000
236	REACTOME_INTERFERON_SIGNALING	R-RNO-913531	6	0.304565	0.780894	0.692308	0.986150	1.000
237	REACTOME_ASSEMBLY_AND_CELL_SURFACE_PRESENTATIO...	R-RNO-9609736	8	0.262346	0.780622	0.715493	0.982460	1.000
238	GOBP_INTRACELLULAR_TRANSPORT	GO:0046907	11	0.233766	0.778058	0.781759	0.983076	1.000
239	GOBP_NUCLEOBASE_CONTAINING_SMALL_MOLECULE_META...	GO:0055086	6	0.307594	0.776835	0.732733	0.981080	1.000
240	GOBP_REGULATION_OF_EPITHELIAL_CELL_MIGRATION	GO:0010632	6	0.295882	0.774116	0.736559	0.982007	1.000
241	GOBP_ESTABLISHMENT_OF_PROTEIN_LOCALIZATION	GO:0045184	18	0.195018	0.772842	0.816254	0.980412	1.000
242	GOBP_NEGATIVE_REGULATION_OF_MOLECULAR_FUNCTION	GO:0044092	24	0.181228	0.772735	0.789272	0.976602	1.000
243	REACTOME_NEGATIVE_REGULATION_OF_NMDA_RECEPTOR_...	R-HSA-961732	8	0.262346	0.771380	0.791277	0.974756	1.000
244	GOBP_REGULATION_OF_CELLULAR_COMPONENT_SIZE	GO:0032535	7	0.280910	0.770440	0.749235	0.972834	1.000
245	GOBP_MUSCLE_ADAPTATION	GO:0043500	5	0.330002	0.769423	0.735135	0.970818	1.000
246	GOBP_REGULATION_OF_PEPTIDASE_ACTIVITY	GO:0052547	10	0.244091	0.769163	0.784257	0.967374	1.000
247	GOBP_REGULATION_OF_NEUROGENESIS	GO:0050767	14	0.209302	0.762407	0.795775	0.975525	1.000
248	GOBP_TEMPERATURE_HOMEOSTASIS	GO:0001659	5	0.313253	0.757293	0.752187	0.979907	1.000
249	GOBP_POSITIVE_REGULATION_OF_CELL_DEATH	GO:0010942	19	0.185679	0.743625	0.851406	0.999491	1.000
250	GOBP_REGULATION_OF_DEFENSE_RESPONSE	GO:0031347	7	0.262837	0.741803	0.821429	0.998800	1.000
251	GOBP_ORGANIC_HYDROXY_COMPOUND_METABOLIC_PROCESS	GO:1901615	7	0.259259	0.739034	0.771015	0.999180	1.000
252	GOBP_POSITIVE_REGULATION_OF_ANION_TRANSPORT	GO:1903793	9	0.240506	0.735512	0.844720	1.000000	1.000
253	GOBP_POSTSYNAPSE_ORGANIZATION	GO:0099173	10	0.243590	0.735128	0.803125	0.997910	1.000
254	GOBP_REGULATION_OF_VESICLE_MEDIATED_TRANSPORT	GO:0060627	12	0.209818	0.727286	0.846875	1.000000	1.000
255	GOBP_REGULATION_OF_CELL_DEVELOPMENT	GO:0060284	15	0.201262	0.726365	0.862295	1.000000	1.000

Continuation of Table **S15**

	NAME	ID	SIZE	ES	NES	NOM.p.val	FDR.q.val	FWER.p.val
256	GOBP_EMBRYO_DEVELOPMENT	GO:0009790	14	0.204587	0.725824	0.830935	1.000000	1.000
257	GOBP_CELL_MORPHOGENESIS_INVOLVED_IN_NEURON_DIF...	GO:0048667	21	0.175457	0.720842	0.888430	1.000000	1.000
258	GOBP_REGULATION_OF_CELLULAR_COMPONENT_MOVEMENT	GO:0051270	15	0.191562	0.715050	0.848592	1.000000	1.000
259	GOBP_APOPTOTIC_MITOCHONDRIAL_CHANGES	GO:0008637	6	0.273523	0.714223	0.849003	1.000000	1.000
260	GOBP_REGULATION_OF_INFLAMMATORY_RESPONSE	GO:0050727	7	0.262837	0.713966	0.785124	1.000000	1.000
261	GOBP_CELL_MORPHOGENESIS_INVOLVED_IN_DIFFERENTI...	GO:0000904	21	0.175457	0.713868	0.846715	1.000000	1.000
262	GOBP_NEGATIVE_REGULATION_OF_KINASE_ACTIVITY	GO:0033673	5	0.289044	0.709549	0.861878	1.000000	1.000
263	GOBP_PROTEIN_LOCALIZATION_TO_PLASMA_MEMBRANE	GO:0072659	7	0.257121	0.707852	0.871166	1.000000	1.000
264	GOBP_NEGATIVE_REGULATION_OF_INTRACELLULAR_SIGN...	GO:1902532	7	0.261763	0.704334	0.835294	1.000000	1.000
265	GOBP_DENDRITE_DEVELOPMENT	GO:0016358	9	0.223438	0.699453	0.870432	1.000000	1.000
266	GOBP_NEGATIVE_REGULATION_OF_TRANSFERASE_ACTIVITY	GO:0051348	5	0.289044	0.694647	0.844327	1.000000	1.000
267	GOBP_REGULATION_OF_ORGANELLE_ORGANIZATION	GO:0033043	14	0.188248	0.693270	0.903448	1.000000	1.000
268	GOBP_MEMBRANE_ORGANIZATION	GO:0061024	12	0.200978	0.692565	0.871473	1.000000	1.000
269	REACTOME_DEATH_RECEPTOR_SIGNALLING	R-RNO-73887	5	0.293522	0.690907	0.845960	1.000000	1.000
270	GOBP_ADENYLATE_CYCLASE_INHIBITING_G_PROTEIN_CO...	GO:0007193	9	0.222140	0.684025	0.890282	1.000000	1.000
271	GOBP_PROTEIN_KINASE_B_SIGNALING	GO:0043491	6	0.257489	0.681750	0.876081	1.000000	1.000
272	GOBP_FATTY_ACID_TRANSPORT	GO:0015908	6	0.268396	0.681750	0.853521	1.000000	1.000
273	GOBP_REGULATION_OF_MAP_KINASE_ACTIVITY	GO:0043405	9	0.214761	0.676153	0.906832	1.000000	1.000
274	GOBP_REGULATION_OF_PHOSPHATIDYLINOSITOL_3_KINA...	GO:0014066	6	0.256098	0.675583	0.881720	1.000000	1.000
275	GOBP_CELL_SURFACE_RECEPTOR_SIGNALING_PATHWAY_I...	GO:1905114	15	0.177210	0.675576	0.924460	1.000000	1.000
276	GOBP_NEGATIVE_REGULATION_OF_APOPTOTIC_SIGNALIN...	GO:2001234	7	0.235976	0.670109	0.887240	1.000000	1.000
277	GOBP_ANION_TRANSMEMBRANE_TRANSPORT	GO:0098656	7	0.240082	0.663266	0.911504	1.000000	1.000
278	GOBP_REGULATION_OF_MUSCLE_CELL_DIFFERENTIATION	GO:0051147	6	0.256098	0.662156	0.888889	1.000000	1.000
279	GOBP_REGULATION_OF_PROTEIN_LOCALIZATION_TO_CEL...	GO:1904375	6	0.265603	0.662001	0.898072	1.000000	1.000
280	GOBP_INTRINSIC_APOPTOTIC_SIGNALING_PATHWAY	GO:0097193	5	0.266292	0.659204	0.887671	1.000000	1.000
281	GOBP_PEPTIDE_METABOLIC_PROCESS	GO:0006518	12	0.193260	0.653903	0.914754	1.000000	1.000
282	REACTOME_DISEASES_OF_SIGNAL_TRANSDUCTION_BY_GR...	R-HSA-5663202	12	0.190461	0.653163	0.906832	1.000000	1.000
283	REACTOME_SYNAPTIC_ADHESION_LIKE_MOLECULES	R-RNO-8849932	9	0.216571	0.649236	0.900000	1.000000	1.000
284	GOBP_ORGANIC_ACID_TRANSPORT	GO:0015849	7	0.229544	0.646966	0.913947	1.000000	1.000
285	GOBP_MALE_GAMETE_GENERATION	GO:0048232	5	0.258920	0.637455	0.910053	1.000000	1.000
286	GOBP_REGULATION_OF_PROTEIN_LOCALIZATION_TO_MEM...	GO:1905475	7	0.234568	0.633212	0.915989	1.000000	1.000
287	GOBP_REGULATION_OF_PEPTIDE_TRANSPORT	GO:0090087	10	0.192271	0.627037	0.948718	1.000000	1.000
288	GOBP_REGULATION_OF_LIPID_METABOLIC_PROCESS	GO:0019216	7	0.224911	0.624483	0.933908	1.000000	1.000
289	GOBP_POSITIVE_REGULATION_OF_PHOSPHATIDYLINOSIT...	GO:0014068	5	0.253012	0.624423	0.951031	1.000000	1.000
290	GOBP_REGULATION_OF_CELL_CYCLE_PROCESS	GO:0010564	5	0.253012	0.616766	0.936464	1.000000	1.000
291	GOBP_POSITIVE_REGULATION_OF_SMOOTH_MUSCLE_CELL...	GO:0048661	5	0.249521	0.613950	0.931579	1.000000	1.000
292	GOBP_CELLULAR_GLUCOSE_HOMEOSTASIS	GO:0001678	5	0.253012	0.610753	0.943978	1.000000	1.000
293	GOBP_CHEMICAL_SYNAPTIC_TRANSMISSION_POSTSYNAPTIC	GO:0099565	9	0.198286	0.608456	0.962428	1.000000	1.000
294	GOBP_CELLULAR_CARBOHYDRATE_METABOLIC_PROCESS	GO:0044262	5	0.253012	0.599794	0.939726	1.000000	1.000
295	GOBP_PROTEIN_DEPHOSPHORYLATION	GO:0006470	7	0.214328	0.593257	0.965714	1.000000	1.000
296	GOBP_NEGATIVE_REGULATION_OF_HYDROLASE_ACTIVITY	GO:0051346	11	0.179643	0.586794	0.938710	1.000000	1.000
297	GOBP_DEPHOSPHORYLATION	GO:0016311	7	0.214328	0.583958	0.946429	1.000000	1.000
298	GOBP_EMBRYO_DEVELOPMENT_ENDING_IN_BIRTH_OR_EGG...	GO:0009792	9	0.186571	0.576436	0.964602	1.000000	1.000

Continuation of Table **S15**

	NAME	ID	SIZE	ES	NES	NOM.p.val	FDR.q.val	FWER.p.val
299	GOBP_EMBRYONIC_ORGAN_DEVELOPMENT	GO:0048568	7	0.203897	0.573003	0.985955	1.000000	1.000
300	GOBP_NEGATIVE_REGULATION_OF_ANION_TRANSPORT	GO:1903792	8	0.195135	0.572101	0.977654	1.000000	1.000
301	GOBP_IN_UTERO_EMBRYONIC_DEVELOPMENT	GO:0001701	8	0.196000	0.570899	0.959877	1.000000	1.000
302	GOBP_NEURON_MIGRATION	GO:0001764	5	0.232317	0.558362	0.983146	1.000000	1.000
303	REACTOME_L1CAM_INTERACTIONS	R-RNO-373760	5	0.228916	0.555463	0.975207	1.000000	1.000
304	GOBP_ORGANONITROGEN_COMPOUND_BIOSYNTHETIC_PROCESS	GO:1901566	13	0.160000	0.550076	0.980645	0.999474	1.000
305	GOBP_POSITIVE_REGULATION_OF_PROTEOLYSIS	GO:0045862	8	0.188316	0.545840	0.982301	0.997882	1.000
306	GOBP_OSSIFICATION	GO:0001503	9	0.173241	0.539555	0.985673	0.996778	1.000
307	GOBP_EXTRINSIC_APOPTOTIC_SIGNALING_PATHWAY	GO:0097191	7	0.192200	0.527174	0.981651	0.997181	1.000
308	GOBP_STRIATED_MUSCLE_CELL_DIFFERENTIATION	GO:0051146	9	0.167890	0.520091	0.984849	0.995529	1.000
309	GOBP_EPITHELIAL_CELL_DIFFERENTIATION	GO:0030855	7	0.172840	0.479206	0.993711	0.999106	1.000
310	GOBP_REGULATION_OF_SMALL_MOLECULE_METABOLIC_PR...	GO:0062012	9	0.161147	0.475423	0.990683	0.996274	1.000
311	GOBP_CALCIUM_MEDIATED_SIGNALING	GO:0019722	10	-0.141026	-0.383831	1.000000	0.999366	1.000
312	GOBP_MUSCLE_CELL_DIFFERENTIATION	GO:0042692	10	-0.153846	-0.422492	0.997054	1.000000	1.000
313	GOBP_NEGATIVE_REGULATION_OF_DEVELOPMENTAL_PROCESS	GO:0051093	13	-0.155610	-0.454574	0.993026	0.999088	1.000
314	GOBP_NEGATIVE_REGULATION_OF_CELL_DIFFERENTIATION	GO:0045596	13	-0.155610	-0.455983	0.985975	1.000000	1.000
315	REACTOME_PROTEIN_PROTEIN_INTERACTIONS_AT_SYNAPSES	R-RNO-6794362	12	-0.163838	-0.473252	0.988795	1.000000	1.000
316	GOBP_NEGATIVE_REGULATION_OF_CELLULAR_COMPONENT...	GO:0051129	11	-0.170382	-0.479859	0.982353	1.000000	1.000
317	GOBP_LIPID_EXPORT_FROM_CELL	GO:0140353	5	-0.225316	-0.484504	0.984472	1.000000	1.000
318	GOBP_MAINTENANCE_OF_LOCATION	GO:0051235	7	-0.207377	-0.500630	0.970326	1.000000	1.000
319	GOBP_RESPONSE_TO_PEPTIDE	GO:1901652	18	-0.158643	-0.507489	0.984148	1.000000	1.000
320	GOBP_REGULATION_OF_ANION_TRANSPORT	GO:0044070	20	-0.154479	-0.508653	0.983193	1.000000	1.000
321	REACTOME_EPH_EPHRIN_SIGNALING	R-RNO-2682334	6	-0.220944	-0.515362	0.979719	1.000000	1.000
322	GOBP_REGULATION_OF_CELLULAR_AMIDE_METABOLIC_PR...	GO:0034248	9	-0.199357	-0.517716	0.976574	1.000000	1.000
323	GOBP_NEGATIVE_REGULATION_OF_CELL_DEVELOPMENT	GO:0010721	6	-0.227567	-0.525068	0.982732	1.000000	1.000
324	GOBP_NEGATIVE_REGULATION_OF_NERVOUS_SYSTEM_DEV...	GO:0051961	5	-0.249492	-0.538065	0.981337	1.000000	1.000
325	GOBP_CELLULAR_RESPONSE_TO_NITROGEN_COMPOUND	GO:1901699	20	-0.161836	-0.538748	0.960396	1.000000	1.000
326	GOBP_REGULATION_OF_CARBOHYDRATE_BIOSYNTHETIC_P...	GO:0043255	5	-0.255061	-0.545114	0.972561	1.000000	1.000
327	GOBP_REGULATION_OF_CARBOHYDRATE_METABOLIC_PROCESS	GO:0006109	5	-0.255061	-0.549076	0.970874	1.000000	1.000
328	REACTOME_PTEN_REGULATION	R-RNO-6807070	5	-0.252572	-0.550394	0.979133	1.000000	1.000
329	GOBP_REGULATION_OF_RECEPTOR_MEDIATED_ENDOCYTOSIS	GO:0048259	5	-0.254355	-0.552087	0.976708	1.000000	1.000
330	GOBP_CARBOHYDRATE_BIOSYNTHETIC_PROCESS	GO:0016051	5	-0.255061	-0.553159	0.958194	1.000000	1.000
331	GOBP_VESICLE_MEDIATED_TRANSPORT_IN_SYNAPSE	GO:0099003	7	-0.227890	-0.554455	0.961821	1.000000	1.000
332	GOBP_CALCIUM_ION_TRANSPORT	GO:0006816	11	-0.198673	-0.555909	0.953587	1.000000	1.000
333	GOBP_METAL_ION_TRANSPORT	GO:0030001	11	-0.198673	-0.556515	0.960584	1.000000	1.000
334	REACTOME_POST_TRANSLATIONAL_PROTEIN_MODIFICATION	R-RNO-597592	7	-0.232667	-0.560671	0.957227	1.000000	1.000
335	GOBP_MULTICELLULAR_ORGANISM_REPRODUCTION	GO:0032504	12	-0.195390	-0.563693	0.954802	1.000000	1.000
336	GOBP_REGULATION_OF_HORMONE_SECRETION	GO:0046883	8	-0.219294	-0.570924	0.958841	1.000000	1.000
337	GOBP_POSITIVE_REGULATION_OF_PEPTIDASE_ACTIVITY	GO:0010952	7	-0.234846	-0.571187	0.957252	1.000000	1.000
338	GOBP_CELLULAR_COMPONENT_MAINTENANCE	GO:0043954	5	-0.261672	-0.573965	0.958730	1.000000	1.000
339	REACTOME_NERVOUS_SYSTEM_DEVELOPMENT	R-RNO-9675108	15	-0.187591	-0.575109	0.963432	1.000000	1.000
340	GOBP_POSITIVE_REGULATION_OF_CYSTEINE_TYPE_ENDO...	GO:2001056	7	-0.234846	-0.577513	0.945227	1.000000	1.000
341	GOBP_AMINO_ACID_TRANSPORT	GO:0006865	6	-0.251394	-0.577904	0.957317	1.000000	1.000

Continuation of Table **S15**

	NAME	ID	SIZE	ES	NES	NOM.p.val	FDR.q.val	FWER.p.val
342	GOBP_CARBOXYLIC_ACID_TRANSPORT	GO:0046942	6	-0.251394	-0.580232	0.940092	1.000000	1.000
343	GOBP_GROWTH	GO:0040007	14	-0.192353	-0.580407	0.960469	1.000000	1.000
344	GOBP_MAPK_CASCADE	GO:0000165	18	-0.181914	-0.580996	0.954240	1.000000	1.000
345	GOBP_ORGANIC_ANION_TRANSPORT	GO:0015711	6	-0.251394	-0.590596	0.946792	1.000000	1.000
346	GOBP_CELLULAR_RESPONSE_TO_ABIOTIC_STIMULUS	GO:0071214	7	-0.245403	-0.591865	0.946237	1.000000	1.000
347	GOBP_REGULATION_OF_ENDOTHELIAL_CELL_MIGRATION	GO:0010594	5	-0.269864	-0.594629	0.949765	1.000000	1.000
348	GOBP_LEUKOCYTE_MEDIATED_IMMUNITY	GO:0002443	7	-0.239585	-0.596431	0.940549	1.000000	1.000
349	GOBP_POSTTRANSCRIPTIONAL_REGULATION_OF_GENE_EX...	GO:0010608	8	-0.238569	-0.600247	0.937879	1.000000	1.000
350	GOBP_LIPID_METABOLIC_PROCESS	GO:0006629	10	-0.219572	-0.602721	0.925501	1.000000	1.000
351	GOBP_REGULATION_OF_ENDOCYTOSIS	GO:0030100	6	-0.260772	-0.604500	0.934959	1.000000	1.000
352	GOBP_MONOAMINE_TRANSPORT	GO:0015844	5	-0.279081	-0.609293	0.921753	1.000000	1.000
353	GOBP_MYOTUBE_DIFFERENTIATION	GO:0014902	5	-0.278270	-0.609567	0.928105	1.000000	1.000
354	GOBP_NEGATIVE_REGULATION_OF_CELL_PROJECTION_OR...	GO:0031345	5	-0.272983	-0.610160	0.948136	1.000000	1.000
355	GOBP_PURINE_CONTAINING_COMPOUND_METABOLIC_PROCESS	GO:0072521	5	-0.282557	-0.614695	0.918712	1.000000	1.000
356	GOBP_NEGATIVE_REGULATION_OF_CATABOLIC_PROCESS	GO:0009895	8	-0.245632	-0.620396	0.906793	1.000000	1.000
357	GOBP_NEGATIVE_REGULATION_OF_PHOSPHORUS_METABOL...	GO:0010563	9	-0.236030	-0.624501	0.922628	1.000000	1.000
358	GOBP_REGULATION_OF_BINDING	GO:0051098	11	-0.222590	-0.624824	0.896652	1.000000	1.000
359	GOBP_RESPONSE_TO_NUTRIENT	GO:0007584	5	-0.293930	-0.627922	0.924194	1.000000	1.000
360	REACTOME_DEVELOPMENTAL_BIOLOGY	R-RNO-1266738	23	-0.186103	-0.631221	0.924528	1.000000	1.000
361	GOBP_RESPONSE_TO_TEMPERATURE_STIMULUS	GO:0009266	9	-0.241522	-0.632063	0.891655	1.000000	1.000
362	GOBP_CELLULAR_AMIDE_METABOLIC_PROCESS	GO:0043603	13	-0.214767	-0.632609	0.914454	1.000000	1.000
363	GOBP_REGULATION_OF_NERVOUS_SYSTEM_PROCESS	GO:0031644	8	-0.249814	-0.632875	0.905488	1.000000	1.000
364	GOBP_PROTEIN_AUTOPHOSPHORYLATION	GO:0046777	5	-0.293641	-0.641719	0.908082	1.000000	1.000
365	GOBP_CELLULAR_RESPONSE_TO_DNA_DAMAGE_STIMULUS	GO:0006974	5	-0.299925	-0.643431	0.914242	1.000000	1.000
366	GOBP_HOMEOSTATIC_PROCESS	GO:0042592	31	-0.177620	-0.643605	0.916880	1.000000	1.000
367	GOBP_EPHRIN_RECEPTOR_SIGNALING_PATHWAY	GO:0048013	5	-0.299163	-0.645748	0.894737	1.000000	1.000
368	GOBP_REGULATION_OF_MAPK_CASCADE	GO:0043408	13	-0.220998	-0.652356	0.899713	1.000000	1.000
369	GOBP_POSITIVE_REGULATION_OF_ORGANELLE_ORGANIZA...	GO:0010638	10	-0.239262	-0.652751	0.893275	1.000000	1.000
370	GOBP_DICARBOXYLIC_ACID_TRANSPORT	GO:0006835	5	-0.301410	-0.654380	0.895223	1.000000	1.000
371	GOBP_MONOCARBOXYLIC_ACID_TRANSPORT	GO:0015718	5	-0.301410	-0.655807	0.887439	1.000000	1.000
372	GOBP_ACIDIC_AMINO_ACID_TRANSPORT	GO:0015800	5	-0.301410	-0.656609	0.906396	1.000000	1.000
373	GOBP_REGULATION_OF_GROWTH	GO:0040008	13	-0.226395	-0.659025	0.877874	1.000000	1.000
374	GOBP_POSITIVE_REGULATION_OF_SMALL_MOLECULE_MET...	GO:0062013	5	-0.304800	-0.661614	0.879365	1.000000	1.000
375	GOBP_T_CELL_ACTIVATION	GO:0042110	8	-0.260073	-0.666083	0.870343	1.000000	1.000
376	GOBP_DEVELOPMENTAL_PROCESS_INVOLVED_IN_REPRODU...	GO:0003006	11	-0.236683	-0.667634	0.869048	1.000000	1.000
377	GOBP_OSTEOBLAST_DIFFERENTIATION	GO:0001649	5	-0.308052	-0.668621	0.882927	1.000000	1.000
378	GOBP_POSITIVE_REGULATION_OF_GENE_EXPRESSION	GO:0010628	16	-0.212132	-0.669254	0.889037	1.000000	1.000
379	GOBP_CELL_MIGRATION	GO:0016477	22	-0.202540	-0.678299	0.878415	1.000000	1.000
380	GOBP_CELL_ACTIVATION_INVOLVED_IN_IMMUNE_RESPONSE	GO:0002263	6	-0.296951	-0.682961	0.873846	1.000000	1.000
381	GOBP_REGULATION_OF_MITOTIC_CELL_CYCLE	GO:0007346	5	-0.318508	-0.683943	0.872441	1.000000	1.000
382	GOBP_MULTICELLULAR_ORGANISMAL_HOMEOSTASIS	GO:0048871	12	-0.231107	-0.684411	0.861386	1.000000	1.000
383	GOBP_REPRODUCTION	GO:0000003	18	-0.213401	-0.686385	0.878492	1.000000	1.000
384	GOBP_SECRETION	GO:0046903	24	-0.200204	-0.688006	0.841379	1.000000	1.000

Continuation of Table **S15**

	NAME	ID	SIZE	ES	NES	NOM.p.val	FDR.q.val	FWER.p.val
385	REACTOME_NEUTROPHIL_DEGRANULATION	R-RNO-6798695	6	-0.296951	-0.688892	0.842788	1.000000	1.000
386	GOBP_MYELOID_LEUKOCYTE_MEDIATED_IMMUNITY	GO:0002444	6	-0.296951	-0.688918	0.840874	1.000000	1.000
387	GOBP_NEGATIVE_REGULATION_OF_PROTEIN_METABOLIC_...	GO:0051248	20	-0.206313	-0.689445	0.860846	1.000000	1.000
388	GOBP_INORGANIC_ION_TRANSMEMBRANE_TRANSPORT	GO:0098660	9	-0.259364	-0.694213	0.848440	1.000000	1.000
389	GOBP_PROTEIN_CONTAINING_COMPLEX_SUBUNIT_ORGANI...	GO:0043933	14	-0.237787	-0.696493	0.852113	1.000000	1.000
390	REACTOME_CELL_CYCLE_MITOTIC	R-RNO-69278	5	-0.321875	-0.697897	0.825954	1.000000	1.000
391	GOBP_ACTIVATION_OF_MAPK_ACTIVITY	GO:0000187	6	-0.304350	-0.700099	0.847962	1.000000	1.000
392	GOBP_PHAGOCYTOSIS	GO:0006909	5	-0.319697	-0.701582	0.835404	1.000000	1.000
393	REACTOME_MAPK_FAMILY_SIGNALING_CASCADES	R-RNO-5683057	12	-0.241086	-0.702155	0.848617	1.000000	1.000
394	GOBP_NEGATIVE_REGULATION_OF_RESPONSE_TO_STIMULUS	GO:0048585	16	-0.226152	-0.702886	0.858921	1.000000	1.000
395	REACTOME_NEURONAL_SYSTEM	R-RNO-112316	26	-0.197839	-0.706650	0.853175	1.000000	1.000
396	GOBP_POSITIVE_REGULATION_OF_MAP_KINASE_ACTIVITY	GO:0043406	6	-0.304350	-0.710201	0.829630	1.000000	1.000
397	GOBP_POSITIVE_REGULATION_OF_DEFENSE_RESPONSE	GO:0031349	5	-0.322895	-0.711115	0.844828	1.000000	1.000
398	GOBP_CELL_JUNCTION_ASSEMBLY	GO:0034329	8	-0.283964	-0.712001	0.828006	1.000000	1.000
399	GOBP_REGULATION_OF_PROTEOLYSIS	GO:0030162	12	-0.249696	-0.715038	0.839763	1.000000	1.000
400	REACTOME_SIGNALING_BY_NTRKS	R-RNO-166520	18	-0.223378	-0.715060	0.820690	1.000000	1.000
401	GOBP_REGULATION_OF_LIPID_BIOSYNTHETIC_PROCESS	GO:0046890	6	-0.315189	-0.715415	0.828869	1.000000	1.000
402	GOBP_GLIAL_CELL_DIFFERENTIATION	GO:0010001	10	-0.258211	-0.715725	0.857576	1.000000	1.000
403	GOBP_POSITIVE_REGULATION_OF_MULTICELLULAR_ORGA...	GO:0051240	32	-0.198697	-0.717295	0.844208	1.000000	1.000
404	REACTOME_OPIOID_SIGNALLING	R-RNO-111885	12	-0.253079	-0.719531	0.829662	1.000000	1.000
405	GOBP_GLAND_DEVELOPMENT	GO:0048732	7	-0.297555	-0.727632	0.796043	1.000000	1.000
406	GOBP_REGULATION_OF_LIPID_LOCALIZATION	GO:1905952	5	-0.332834	-0.728137	0.830745	1.000000	1.000
407	REACTOME_CREB1_PHOSPHORYLATION_THROUGH_NMDA_RE...	R-RNO-442742	8	-0.284086	-0.730558	0.823976	1.000000	1.000
408	GOBP_ADENYLATE_CYCLASE_INHIBITING_G_PROTEIN_CO...	GO:0007196	5	-0.332218	-0.730829	0.816746	1.000000	1.000
409	GOBP_LIPID_BIOSYNTHETIC_PROCESS	GO:0008610	6	-0.315189	-0.731892	0.818325	1.000000	1.000
410	GOBP_ORGANIC_ACID_METABOLIC_PROCESS	GO:0006082	7	-0.297588	-0.732509	0.796712	1.000000	1.000
411	GOBP_CARDIAC_MUSCLE_TISSUE_DEVELOPMENT	GO:0048738	6	-0.321305	-0.734381	0.777778	1.000000	1.000
412	GOBP_GLUTAMATE_RECEPTOR_SIGNALING_PATHWAY	GO:0007215	17	-0.230791	-0.735311	0.797844	1.000000	1.000
413	GOBP_NEGATIVE_REGULATION_OF_LOCOMOTION	GO:0040013	5	-0.334751	-0.735552	0.796825	1.000000	1.000
414	GOBP_ERK1_AND_ERK2_CASCADE	GO:0070371	5	-0.337477	-0.736978	0.799051	1.000000	1.000
415	GOBP_ENDOCRINE_SYSTEM_DEVELOPMENT	GO:0035270	6	-0.316227	-0.737751	0.801964	1.000000	1.000
416	GOBP_HEART_DEVELOPMENT	GO:0007507	6	-0.321305	-0.738306	0.794643	1.000000	1.000
417	GOBP_ANIMAL_ORGAN_MORPHOGENESIS	GO:0009887	12	-0.254598	-0.738699	0.802038	1.000000	1.000
418	GOBP_REGULATION_OF_LYMPHOCYTE_ACTIVATION	GO:0051249	5	-0.346482	-0.742661	0.797488	1.000000	1.000
419	REACTOME_CELL_CYCLE	R-RNO-1640170	6	-0.324282	-0.747242	0.789147	1.000000	1.000
420	GOBP_NEURON_DEATH	GO:0070997	18	-0.230725	-0.748150	0.816804	1.000000	1.000
421	GOBP_SIGNAL_RELEASE	GO:0023061	14	-0.248176	-0.754013	0.801688	1.000000	1.000
422	GOBP_GLIOGENESIS	GO:0042063	12	-0.261970	-0.756692	0.775298	1.000000	1.000
423	GOBP_REGULATION_OF_APOPTOTIC_SIGNALING_PATHWAY	GO:2001233	10	-0.275270	-0.758591	0.777941	1.000000	1.000
424	GOBP_LIPID_LOCALIZATION	GO:0010876	10	-0.279097	-0.762122	0.754491	1.000000	1.000
425	GOBP_REGULATION_OF_PROTEIN_SERINE_THREONINE_KI...	GO:0071900	12	-0.267358	-0.762288	0.767544	1.000000	1.000
426	GOBP_LYMPHOCYTE_ACTIVATION	GO:0046649	9	-0.293889	-0.764500	0.761905	1.000000	1.000
427	GOBP_GLUCOSE_METABOLIC_PROCESS	GO:0006006	6	-0.335603	-0.765267	0.755385	1.000000	1.000

Continuation of Table **S15**

	NAME	ID	SIZE	ES	NES	NOM.p.val	FDR.q.val	FWER.p.val
428	REACTOME_GPCR_LIGAND_BINDING	R-RNO-500792	8	-0.300888	-0.770134	0.769120	1.000000	1.000
429	REACTOME_SIGNALING_BY_GPCR	R-RNO-372790	22	-0.225838	-0.772090	0.782967	1.000000	1.000
430	GOBP_MONOSACCHARIDE_METABOLIC_PROCESS	GO:0005996	6	-0.335603	-0.772918	0.725962	1.000000	1.000
431	GOBP_REGULATION_OF_DNA_BINDING_TRANSCRIPTION_F...	GO:0051090	16	-0.250585	-0.775492	0.762500	1.000000	1.000
432	GOBP_CIRCULATORY_SYSTEM_PROCESS	GO:0003013	5	-0.356188	-0.780847	0.775157	1.000000	1.000
433	GOBP_REGULATION_OF_CELL_DEATH	GO:0010941	34	-0.213025	-0.784339	0.760052	1.000000	1.000
434	GOBP_REGULATION_OF_FAT_CELL_DIFFERENTIATION	GO:0045598	6	-0.339784	-0.785009	0.752252	1.000000	1.000
435	GOBP_RESPONSE_TO_TUMOR_NECROSIS_FACTOR	GO:0034612	7	-0.324557	-0.785861	0.754123	1.000000	1.000
436	REACTOME_EXTRA_NUCLEAR_ESTROGEN_SIGNALING	R-RNO-9009391	7	-0.318658	-0.786100	0.748126	1.000000	1.000
437	REACTOME_EXTRACELLULAR_MATRIX_ORGANIZATION	R-RNO-1474244	5	-0.363523	-0.790234	0.755556	1.000000	1.000
438	GOBP_REGULATION_OF_PROTEIN_BINDING	GO:0043393	7	-0.329582	-0.790254	0.734158	1.000000	1.000
439	GOBP_RESPONSE_TO_NITROGEN_COMPOUND	GO:1901698	34	-0.216616	-0.790364	0.750639	1.000000	1.000
440	REACTOME_INTEGRATION_OF_ENERGY_METABOLISM	R-RNO-163685	5	-0.366499	-0.791501	0.730645	1.000000	1.000
441	GOBP_RESPONSE_TO_UV	GO:0009411	5	-0.363129	-0.792886	0.741486	1.000000	1.000
442	GOBP_RESPONSE_TO_ENDOGENOUS_STIMULUS	GO:0009719	34	-0.217058	-0.799391	0.735369	1.000000	1.000
443	GOBP_REGULATION_OF_CELL_DIFFERENTIATION	GO:0045595	36	-0.219299	-0.800329	0.747736	1.000000	1.000
444	GOBP_REGULATION_OF_EXTRINSIC_APOPTOTIC_SIGNALI...	GO:2001236	6	-0.351206	-0.805878	0.738583	1.000000	1.000
445	GOBP_REGULATION_OF_CELLULAR_CATABOLIC_PROCESS	GO:0031329	10	-0.290981	-0.808215	0.724812	1.000000	1.000
446	GOBP_REGULATION_OF_SYSTEM_PROCESS	GO:0044057	18	-0.245200	-0.808749	0.704708	1.000000	1.000
447	GOBP_POSITIVE_REGULATION_OF_PROTEIN_SERINE_THR...	GO:0071902	9	-0.304207	-0.810455	0.732169	1.000000	1.000
448	GOBP_REGULATION_OF_SECRETION	GO:0051046	14	-0.265804	-0.810918	0.701556	1.000000	1.000
449	GOBP_PROTEIN_LOCALIZATION_TO_MEMBRANE	GO:0072657	14	-0.264422	-0.812529	0.709632	1.000000	1.000
450	GOBP_REGULATION_OF_METAL_ION_TRANSPORT	GO:0010959	6	-0.347358	-0.812577	0.701219	1.000000	1.000
451	GOBP_AMEBOIDAL_TYPE_CELL_MIGRATION	GO:0001667	11	-0.292051	-0.816310	0.679831	1.000000	1.000
452	GOBP_MUSCLE_CELL_PROLIFERATION	GO:0033002	8	-0.326586	-0.817419	0.702128	1.000000	1.000
453	GOBP_IMMUNE_EFFECTOR_PROCESS	GO:0002252	10	-0.299667	-0.819964	0.708029	1.000000	1.000
454	GOBP_TISSUE_MIGRATION	GO:0090130	9	-0.315495	-0.822786	0.700752	1.000000	1.000
455	GOBP_FOREBRAIN_DEVELOPMENT	GO:0030900	9	-0.312941	-0.825249	0.710526	1.000000	1.000
456	GOBP_LEUKOCYTE_CELL_CELL_ADHESION	GO:0007159	6	-0.360315	-0.829140	0.689441	1.000000	1.000
457	GOBP_CIRCADIAN_REGULATION_OF_GENE_EXPRESSION	GO:0032922	5	-0.377575	-0.834692	0.672000	1.000000	1.000
458	GOBP_RESPONSE_TO_INSULIN	GO:0032868	5	-0.387578	-0.834944	0.713141	1.000000	1.000
459	GOBP_EPIDERMIS_DEVELOPMENT	GO:0008544	5	-0.375858	-0.835332	0.698113	1.000000	1.000
460	GOBP_REGULATION_OF_WNT_SIGNALING_PATHWAY	GO:0030111	5	-0.389317	-0.836626	0.697205	1.000000	1.000
461	GOBP_REGULATION_OF_NEUROTRANSMITTER_TRANSPORT	GO:0051588	6	-0.370947	-0.846788	0.677019	1.000000	1.000
462	GOBP_REGULATION_OF_ANATOMICAL_STRUCTURE_SIZE	GO:0090066	10	-0.307604	-0.847605	0.668175	1.000000	1.000
463	REACTOME_SIGNALING_BY_RECEPTOR_TYROSINE_KINASES	R-RNO-9006934	26	-0.243320	-0.848284	0.682561	1.000000	1.000
464	GOBP_APOPTOTIC_SIGNALING_PATHWAY	GO:0097190	15	-0.277939	-0.853598	0.662393	1.000000	1.000
465	GOBP_RESPIRATORY_SYSTEM_DEVELOPMENT	GO:0060541	6	-0.371669	-0.858307	0.655763	1.000000	1.000
466	GOBP_POSITIVE_REGULATION_OF_INTRACELLULAR_SIGN...	GO:1902533	14	-0.287822	-0.860357	0.643994	1.000000	1.000
467	GOBP_HOMEOSTASIS_OF_NUMBER_OF_CELLS	GO:0048872	5	-0.394896	-0.860543	0.649612	1.000000	1.000
468	GOBP_LOCOMOTION	GO:0040011	31	-0.243054	-0.863587	0.671018	1.000000	1.000
469	GOBP_REGULATION_OF_VASCULATURE_DEVELOPMENT	GO:1901342	5	-0.396738	-0.864099	0.662903	1.000000	1.000
470	GOBP_NEGATIVE_REGULATION_OF_PROTEIN_MODIFICATI...	GO:0031400	10	-0.320006	-0.865652	0.645926	1.000000	1.000

Continuation of Table **S15**

	NAME	ID	SIZE	ES	NES	NOM.p.val	FDR.q.val	FWER.p.val
471	GOBP_PROTEOLYSIS	GO:0006508	17	-0.277090	-0.873182	0.609929	1.000000	1.000
472	GOBP_CELLULAR_RESPONSE_TO_PEPTIDE	GO:1901653	13	-0.295253	-0.874776	0.617111	1.000000	1.000
473	GOBP_NEGATIVE_REGULATION_OF_CELL_POPULATION_PR...	GO:0008285	9	-0.333921	-0.875372	0.622419	1.000000	1.000
474	GOBP_MULTI_ORGANISM_PROCESS	GO:0051704	16	-0.284382	-0.877456	0.629477	1.000000	1.000
475	REACTOME_TRAFFICKING_OF_GLUR2_CONTAINING_AMPA_...	R-RNO-416993	8	-0.341685	-0.879027	0.628866	1.000000	1.000
476	GOBP_POSITIVE_REGULATION_OF_CELL_CELL_ADHESION	GO:0022409	5	-0.406004	-0.879239	0.654664	1.000000	1.000
477	HALLMARK_ALLOGRAFT_REJECTION	M5950	7	-0.360129	-0.879627	0.627871	1.000000	1.000
478	GOBP_REGULATION_OF_CELLULAR_COMPONENT_BIOGENESIS	GO:0044087	10	-0.315922	-0.879857	0.611026	1.000000	1.000
479	GOBP_POSITIVE_REGULATION_OF_LEUKOCYTE_CELL_CEL...	GO:1903039	5	-0.406004	-0.884296	0.618012	1.000000	1.000
480	GOBP_NEUROTRANSMITTER_SECRETION	GO:0007269	6	-0.381670	-0.885794	0.634969	1.000000	1.000
481	GOBP_POSITIVE_REGULATION_OF_MAPK_CASCADE	GO:0043410	9	-0.338713	-0.896571	0.589260	1.000000	1.000
482	GOBP_LEARNING	GO:0007612	13	-0.313048	-0.901216	0.585366	1.000000	1.000
483	GOBP_RESPONSE_TO_OXYGEN_CONTAINING_COMPOUND	GO:1901700	39	-0.236732	-0.901216	0.590377	1.000000	1.000
484	GOBP_CELL_ACTIVATION	GO:0001775	21	-0.273791	-0.906303	0.586486	1.000000	1.000
485	REACTOME_ESTROGEN_DEPENDENT_NUCLEAR_EVENTS_DOW...	R-RNO-9634638	5	-0.413713	-0.906913	0.601926	1.000000	1.000
486	GOBP_CELLULAR_RESPONSE_TO_LIPID	GO:0071396	15	-0.291263	-0.910595	0.587021	1.000000	1.000
487	GOBP_SECOND_MESSENGER_MEDIATED_SIGNALING	GO:0019932	16	-0.287964	-0.910788	0.559441	1.000000	1.000
488	GOBP_MUSCLE_STRUCTURE_DEVELOPMENT	GO:0061061	16	-0.296298	-0.915626	0.558533	1.000000	1.000
489	GOBP_MITOTIC_CELL_CYCLE	GO:0000278	10	-0.338358	-0.918131	0.589443	1.000000	1.000
490	REACTOME_DAG_AND_IP3_SIGNALING	R-RNO-1489509	8	-0.357960	-0.918524	0.570370	1.000000	1.000
491	GOBP_POSITIVE_REGULATION_OF_RESPONSE_TO_EXTERN...	GO:0032103	9	-0.344439	-0.926390	0.545727	1.000000	1.000
492	GOBP_HEAD_DEVELOPMENT	GO:0060322	22	-0.277441	-0.927152	0.539095	1.000000	1.000
493	GOBP_DEFENSE_RESPONSE_TO_OTHER_ORGANISM	GO:0098542	9	-0.350618	-0.930056	0.582335	1.000000	1.000
494	GOBP_CELLULAR_COMPONENT_DISASSEMBLY	GO:0022411	6	-0.402313	-0.937126	0.551669	0.997346	1.000
495	GOBP_POSITIVE_REGULATION_OF_IMMUNE_RESPONSE	GO:0050778	9	-0.357252	-0.938338	0.558209	0.998924	1.000
496	GOBP_ANATOMICAL_STRUCTURE_HOMEOSTASIS	GO:0060249	7	-0.380477	-0.939679	0.549467	1.000000	1.000
497	GOBP_MULTI_MULTICELLULAR_ORGANISM_PROCESS	GO:0044706	8	-0.370361	-0.940234	0.517804	1.000000	1.000
498	GOBP_REGULATION_OF_MULTICELLULAR_ORGANISMAL_DE...	GO:2000026	31	-0.264240	-0.941307	0.540365	1.000000	1.000
499	GOBP_ORGANIC_HYDROXY_COMPOUND_TRANSPORT	GO:0015850	6	-0.400176	-0.941840	0.543307	1.000000	1.000
500	REACTOME_INTERLEUKIN_4_AND_INTERLEUKIN_13_SIGN...	R-RNO-6785807	8	-0.378643	-0.944547	0.533626	1.000000	1.000
501	GOBP_PROTEIN_COMPLEX_OLIGOMERIZATION	GO:0051259	6	-0.407538	-0.945818	0.556904	1.000000	1.000
502	GOBP_REGULATION_OF_PROTEIN_CATABOLIC_PROCESS	GO:0042176	7	-0.381075	-0.945906	0.519637	1.000000	1.000
503	GOBP_ENDOTHELIAL_CELL_PROLIFERATION	GO:0001935	8	-0.376708	-0.946219	0.524540	1.000000	1.000
504	GOBP_RESPONSE_TO_PEPTIDE_HORMONE	GO:0043434	13	-0.317643	-0.946855	0.533898	1.000000	1.000
505	GOBP_ACTIVATION_OF_IMMUNE_RESPONSE	GO:0002253	5	-0.428592	-0.948476	0.541411	1.000000	1.000
506	GOBP_FAT_CELL_DIFFERENTIATION	GO:0045444	9	-0.362500	-0.949317	0.563798	1.000000	1.000
507	GOBP_REGULATION_OF_GTPASE_ACTIVITY	GO:0043087	5	-0.436050	-0.950546	0.540582	1.000000	1.000
508	GOBP_IMPORT_INTO_CELL	GO:0098657	6	-0.420317	-0.950650	0.565149	1.000000	1.000
509	GOBP_ENDOTHELIAL_CELL_MIGRATION	GO:0043542	8	-0.377053	-0.952699	0.523006	1.000000	1.000
510	GOBP_ENDOMEMBRANE_SYSTEM_ORGANIZATION	GO:0010256	6	-0.408568	-0.953531	0.541213	1.000000	1.000
511	GOBP_REGULATION_OF_PROTEIN_STABILITY	GO:0031647	5	-0.437451	-0.953783	0.545598	1.000000	1.000
512	GOBP_BLOOD_VESSEL_ENDOTHELIAL_CELL_MIGRATION	GO:0043534	8	-0.377053	-0.958857	0.532012	1.000000	1.000
513	GOBP_SKELETAL_MUSCLE_ORGAN_DEVELOPMENT	GO:0060538	5	-0.439272	-0.967618	0.510870	1.000000	1.000

Continuation of Table **S15**

	NAME	ID	SIZE	ES	NES	NOM.p.val	FDR.q.val	FWER.p.val
514	GOBP_NEGATIVE_REGULATION_OF_GENE_EXPRESSION	GO:0010629	12	-0.339042	-0.971567	0.489362	1.000000	1.000
515	REACTOME_CLASS_C_3_METABOTROPIC_GLUTAMATE_PHER...	R-RNO-420499	7	-0.407264	-0.973462	0.510736	1.000000	1.000
516	GOBP_RESPONSE_TO_ABIOTIC_STIMULUS	GO:0009628	27	-0.274885	-0.975755	0.503320	1.000000	1.000
517	GOBP_POSITIVE_REGULATION_OF_CELLULAR_COMPONENT...	GO:0044089	9	-0.370638	-0.976899	0.489943	1.000000	1.000
518	REACTOME_INTRACELLULAR_SIGNALING_BY_SECOND_MES...	R-RNO-9006925	15	-0.323305	-0.979541	0.485836	1.000000	1.000
519	GOBP_ACTIN_FILAMENT_BASED_PROCESS	GO:0030029	7	-0.402114	-0.979852	0.513514	1.000000	1.000
520	GOBP_PROTEIN_LOCALIZATION_TO_SYNAPSE	GO:0035418	5	-0.457618	-0.980945	0.529687	1.000000	1.000
521	GOBP_POSITIVE_REGULATION_OF_CELL_POPULATION_PR...	GO:0008284	21	-0.290149	-0.982022	0.491252	1.000000	1.000
522	HALLMARK_EPITHELIAL_MESENCHYMAL_TRANSITION	M5930	5	-0.454135	-0.984350	0.477707	1.000000	1.000
523	GOBP_SMOOTH_MUSCLE_CELL_PROLIFERATION	GO:0048659	6	-0.419735	-0.985087	0.507042	1.000000	1.000
524	GOBP_SPROUTING_ANGIOGENESIS	GO:0002040	5	-0.457282	-0.986906	0.505017	1.000000	1.000
525	GOBP_CELLULAR_RESPONSE_TO_OXYGEN_CONTAINING_CO...	GO:1901701	29	-0.274257	-0.987229	0.471910	1.000000	1.000
526	GOBP_RESPONSE_TO_WOUNDING	GO:0009611	15	-0.333577	-0.992935	0.484286	1.000000	1.000
527	GOBP_PROTEIN_LOCALIZATION_TO_POSTSYNAPSE	GO:0062237	5	-0.457618	-0.994452	0.477419	1.000000	1.000
528	GOBP_CENTRAL_NERVOUS_SYSTEM_DEVELOPMENT	GO:0007417	26	-0.281200	-0.997138	0.454301	1.000000	1.000
529	GOBP_REGULATION_OF_CELL_ACTIVATION	GO:0050865	8	-0.396144	-1.001272	0.465569	1.000000	1.000
530	GOBP_BIOLOGICAL_ADHESION	GO:0022610	19	-0.308483	-1.002934	0.464088	1.000000	1.000
531	GOBP_REGULATION_OF_GLIOGENESIS	GO:0014013	5	-0.459194	-1.003377	0.474441	1.000000	1.000
532	GOBP_CALCIUM_ION_TRANSMEMBRANE_TRANSPORT	GO:0070588	7	-0.413700	-1.003565	0.471842	1.000000	1.000
533	GOBP_MUSCLE_TISSUE_DEVELOPMENT	GO:0060537	11	-0.354671	-1.004038	0.450581	1.000000	1.000
534	REACTOME_G_PROTEIN_MEDIATED_EVENTS	R-RNO-112040	9	-0.380591	-1.005494	0.485673	1.000000	1.000
535	REACTOME_NEUREXINS_AND_NEUROLIGINS	R-RNO-6794361	9	-0.382585	-1.006864	0.453102	1.000000	1.000
536	GOBP_POSITIVE_REGULATION_OF_GLIOGENESIS	GO:0014015	5	-0.459194	-1.007796	0.455975	1.000000	1.000
537	REACTOME_TRANSCRIPTIONAL_REGULATION_OF_WHITE_A...	R-RNO-381340	6	-0.426829	-1.008355	0.451163	1.000000	1.000
538	GOBP_CALCIUM_ION_TRANSPORT_INTO_CYTOSOL	GO:0060402	7	-0.413700	-1.009157	0.455752	1.000000	1.000
539	G...	GO:0007216	8	-0.390362	-1.009738	0.457143	1.000000	1.000
540	GOBP_CYTOSOLIC_CALCIUM_ION_TRANSPORT	GO:0060401	7	-0.413700	-1.010712	0.470229	1.000000	1.000
541	REACTOME_CA_DEPENDENT_EVENTS	R-RNO-111996	8	-0.394839	-1.013624	0.445802	1.000000	1.000
542	GOBP_REGULATION_OF_MUSCLE_SYSTEM_PROCESS	GO:0090257	8	-0.398763	-1.016763	0.482655	1.000000	1.000
543	GOBP_MUSCLE_SYSTEM_PROCESS	GO:0003012	9	-0.386318	-1.019082	0.448680	1.000000	1.000
544	GOBP_REPRODUCTIVE_SYSTEM_DEVELOPMENT	GO:0061458	6	-0.445970	-1.019978	0.440367	1.000000	1.000
545	GOBP_CELL_CYCLE_PROCESS	GO:0022402	11	-0.365200	-1.022490	0.429178	1.000000	1.000
546	GOBP_RESPONSE_TO_STARVATION	GO:0042594	5	-0.475357	-1.033415	0.419558	1.000000	1.000
547	GOBP_INNATE_IMMUNE_RESPONSE	GO:0045087	7	-0.427049	-1.035124	0.431517	1.000000	1.000
548	GOBP_EPITHELIAL_CELL_PROLIFERATION	GO:0050673	12	-0.359808	-1.039745	0.424965	1.000000	1.000
549	GOBP_CELLULAR_RESPONSE_TO_STARVATION	GO:0009267	5	-0.475357	-1.042028	0.406400	1.000000	1.000
550	GOBP_MUSCLE_CONTRACTION	GO:0006936	5	-0.497188	-1.048822	0.402174	0.986314	1.000
551	GOBP_DNA_METABOLIC_PROCESS	GO:0006259	6	-0.452777	-1.050817	0.417533	0.986631	1.000
552	GOBP_NEGATIVE_REGULATION_OF_BINDING	GO:0051100	6	-0.447037	-1.053793	0.441221	0.983605	1.000
553	GOBP_RESPONSE_TO_OXYGEN_LEVELS	GO:0070482	8	-0.414000	-1.059415	0.409836	0.972756	1.000
554	GOBP_AMYLOID_PRECURSOR_PROTEIN_METABOLIC_PROCESS	GO:0042982	5	-0.492010	-1.060547	0.388060	0.975387	1.000
555	GOBP_REGULATION_OF_OSTEOCLAST_DIFFERENTIATION	GO:0045670	5	-0.492541	-1.063147	0.404025	0.973855	1.000
556	GOBP_RESPONSE_TO_ORGANIC_CYCLIC_COMPOUND	GO:0014070	20	-0.319441	-1.064552	0.392351	0.975593	1.000

Continuation of Table **S15**

	NAME	ID	SIZE	ES	NES	NOM.p.val	FDR.q.val	FWER.p.val
557	GOBP_AMYLOID_BETA_METABOLIC_PROCESS	GO:0050435	5	-0.492010	-1.064621	0.415000	0.981502	1.000
558	GOBP_PROTEIN_MODIFICATION_BY_SMALL_PROTEIN_CON...	GO:0032446	6	-0.456732	-1.069884	0.383915	0.971669	1.000
559	GOBP_AMYLOID_PRECURSOR_PROTEIN_CATABOLIC_PROCESS	GO:0042987	5	-0.492010	-1.070152	0.396923	0.977022	1.000
560	REACTOME_ESR_MEDIATED_SIGNALING	R-RNO-8939211	8	-0.426994	-1.072066	0.376190	0.977608	1.000
561	GOBP_CELL_CELL_ADHESION	GO:0098609	14	-0.363485	-1.074994	0.379404	0.974460	1.000
562	GOBP_INTRACELLULAR_PROTEIN_TRANSPORT	GO:0006886	9	-0.399135	-1.075824	0.380466	0.978055	1.000
563	GOBP_EXCITATORY_CHEMICAL_SYNAPTIC_TRANSMISSION	GO:0098976	5	-0.491521	-1.077748	0.395899	0.978250	1.000
564	GOBP_REGULATION_OF_RESPONSE_TO_EXTERNAL_STIMULUS	GO:0032101	15	-0.356463	-1.085414	0.370739	0.960645	1.000
565	GOBP_PROTEIN_LOCALIZATION_TO_CELL_JUNCTION	GO:1902414	6	-0.474984	-1.088023	0.355385	0.958550	1.000
566	GOBP_RESPONSE_TO_NICOTINE	GO:0035094	7	-0.456882	-1.089531	0.366771	0.960144	1.000
567	GOBP_VASCULATURE_DEVELOPMENT	GO:0001944	17	-0.343906	-1.089783	0.347709	0.965642	1.000
568	GOBP_RESPONSE_TO_KETONE	GO:1901654	7	-0.454154	-1.089983	0.368343	0.971484	1.000
569	REACTOME_PLATELET_ACTIVATION_SIGNALING_AND_AGG...	R-HSA-76002	7	-0.450459	-1.100777	0.348367	0.944502	1.000
570	GOBP_REGULATION_OF_CATABOLIC_PROCESS	GO:0009894	13	-0.372462	-1.100988	0.349138	0.950167	1.000
571	REACTOME_SIGNALING_BY_NUCLEAR_RECEPTORS	R-RNO-9006931	8	-0.426994	-1.104251	0.365152	0.946103	1.000
572	GOBP_ASSOCIATIVE_LEARNING	GO:0008306	7	-0.454096	-1.110026	0.340062	0.934589	1.000
573	GOBP_WOUND_HEALING	GO:0042060	13	-0.381623	-1.114408	0.319502	0.927445	1.000
574	GOBP_RESPONSE_TO_GROWTH_FACTOR	GO:0070848	18	-0.338391	-1.115007	0.330111	0.931911	1.000
575	GOBP_POSITIVE_REGULATION_OF_CELL_ADHESION	GO:0045785	7	-0.459129	-1.123231	0.322239	0.912577	1.000
576	GOBP_CYTOSKELETON_ORGANIZATION	GO:0007010	11	-0.394125	-1.124160	0.315407	0.916161	1.000
577	GOBP_RESPONSE_TO_HORMONE	GO:0009725	20	-0.340209	-1.126390	0.308921	0.915289	1.000
578	GOBP_ENZYME_LINKED_RECEPTOR_PROTEIN_SIGNALING_...	GO:0007167	24	-0.325712	-1.127490	0.323529	0.918189	1.000
579	GOBP_EPITHELIUM_DEVELOPMENT	GO:0060429	15	-0.365037	-1.130119	0.304721	0.916337	1.000
580	GOBP_NEGATIVE_REGULATION_OF_MULTICELLULAR_ORGA...	GO:0051241	15	-0.370502	-1.132096	0.310830	0.916429	1.000
581	GOBP_PROTEIN_CATABOLIC_PROCESS	GO:0030163	8	-0.444902	-1.132385	0.327354	0.922130	1.000
582	GOBP_POSITIVE_REGULATION_OF_DEVELOPMENTAL_PROCESS	GO:0051094	32	-0.314084	-1.134654	0.297789	0.921511	1.000
583	REACTOME_CYTOKINE_SIGNALING_IN_IMMUNE_SYSTEM	R-RNO-1280215	21	-0.335608	-1.135350	0.309917	0.926036	1.000
584	GOBP_REGULATION_OF_CELL_ADHESION	GO:0030155	13	-0.388785	-1.138985	0.326180	0.920555	1.000
585	GOBP_CELL_CELL_JUNCTION_ASSEMBLY	GO:0007043	5	-0.524304	-1.140321	0.314103	0.923106	1.000
586	GOBP_CIRCULATORY_SYSTEM_DEVELOPMENT	GO:0072359	21	-0.344811	-1.140686	0.294430	0.928942	1.000
587	GOBP_NEGATIVE_REGULATION_OF_TRANSCRIPTION_BY_R...	GO:0000122	10	-0.425685	-1.140895	0.298422	0.935235	1.000
588	REACTOME_G_ALPHA_Z_SIGNALLING_EVENTS	R-RNO-418597	5	-0.518144	-1.142769	0.281834	0.935980	1.000
589	GOBP_REGULATION_OF_VASCULAR_ASSOCIATED_SMOOTH_...	GO:1904705	5	-0.510798	-1.143030	0.304478	0.942366	1.000
590	GOBP_CELLULAR_RESPONSE_TO_CADMIUM_ION	GO:0071276	5	-0.515070	-1.143813	0.309562	0.946963	1.000
591	GOBP_POSITIVE_REGULATION_OF_NEURON_DEATH	GO:1901216	5	-0.523392	-1.145828	0.301858	0.947563	1.000
592	GOBP_BLOOD_VESSEL_MORPHOGENESIS	GO:0048514	14	-0.386338	-1.152489	0.278184	0.932296	1.000
593	GOBP_RESPONSE_TO_METAL_ION	GO:0010038	15	-0.378169	-1.152894	0.292887	0.938324	1.000
594	GOBP_CELLULAR_RESPONSE_TO_PEPTIDE_HORMONE_STIM...	GO:0071375	9	-0.437952	-1.160068	0.306092	0.920952	1.000
595	GOBP_CELLULAR_RESPONSE_TO_MOLECULE_OF_BACTERIA...	GO:0071219	8	-0.457646	-1.167378	0.274032	0.903951	1.000
596	GOBP_POSITIVE_REGULATION_OF_CELL_DIFFERENTIATION	GO:0045597	27	-0.333192	-1.171701	0.237838	0.896409	1.000
597	REACTOME_ADAPTIVE_IMMUNE_SYSTEM	R-RNO-1280218	9	-0.448359	-1.171790	0.253776	0.903447	1.000
598	GOBP_RESPONSE_TO_INORGANIC_SUBSTANCE	GO:0010035	19	-0.359472	-1.175542	0.267956	0.898297	1.000
599	GOBP_POSITIVE_REGULATION_OF_EPITHELIAL_CELL_PR...	GO:0050679	7	-0.477754	-1.176447	0.272162	0.902646	1.000

Continuation of Table **S15**

	NAME	ID	SIZE	ES	NES	NOM.p.val	FDR.q.val	FWER.p.val
600	GOBP_CELLULAR_RESPONSE_TO_BIOTIC_STIMULUS	GO:0071216	8	-0.457646	-1.182256	0.282090	0.890172	1.000
601	GOBP_RESPONSE_TO_EXTRACELLULAR_STIMULUS	GO:0009991	13	-0.401407	-1.182627	0.255618	0.896368	1.000
602	GOBP_NEUROTRANSMITTER_TRANSPORT	GO:0006836	7	-0.487949	-1.184139	0.266667	0.898454	1.000
603	REACTOME_INFECTIOUS_DISEASE	R-HSA-5663205	13	-0.402949	-1.186614	0.262272	0.897612	1.000
604	GOBP_REGULATION_OF_CIRCADIAN_RHYTHM	GO:0042752	7	-0.485345	-1.187146	0.267584	0.903607	1.000
605	GOBP_ANATOMICAL_STRUCTURE_FORMATION_INVOLVED_I...	GO:0048646	20	-0.356987	-1.189714	0.251058	0.901997	1.000
606	GOBP_CYTOKINE_MEDIATED_SIGNALING_PATHWAY	GO:0019221	16	-0.382404	-1.195623	0.247326	0.889620	1.000
607	GOBP_POSITIVE_REGULATION_OF_ENDOTHELIAL_CELL_P...	GO:0001938	6	-0.518302	-1.198118	0.239940	0.888312	1.000
608	REACTOME_CIRCADIAN_CLOCK	R-HSA-400253	5	-0.558157	-1.198494	0.230047	0.895175	1.000
609	GOBP_REGULATION_OF_NEUROTRANSMITTER_LEVELS	GO:0001505	7	-0.487949	-1.207694	0.241641	0.871023	1.000
610	GOBP_CELLULAR_RESPONSE_TO_EXTERNAL_STIMULUS	GO:0071496	10	-0.438739	-1.209200	0.233859	0.873588	1.000
611	REACTOME_LEISHMANIA_INFECTION	R-HSA-9658195	10	-0.444701	-1.211175	0.244868	0.874624	1.000
612	GOBP_PROTEIN_MODIFICATION_BY_SMALL_PROTEIN_CON...	GO:0070647	7	-0.502210	-1.213291	0.242613	0.875561	1.000
613	HALLMARK_UV_RESPONSE_UP	M5941	5	-0.555993	-1.213373	0.218354	0.883537	1.000
614	GOBP_MONONUCLEAR_CELL_DIFFERENTIATION	GO:1903131	7	-0.504386	-1.217453	0.230089	0.877573	1.000
615	REACTOME_SIGNALING_BY_INTERLEUKINS	R-RNO-449147	15	-0.394507	-1.225899	0.220630	0.855973	1.000
616	GOBP_RESPONSE_TO_ACID_CHEMICAL	GO:0001101	6	-0.523940	-1.228774	0.205970	0.854344	1.000
617	GOBP_RESPONSE_TO_LIGHT_STIMULUS	GO:0009416	11	-0.445851	-1.231855	0.206442	0.851560	1.000
618	REACTOME_RHO_GTPASE_EFFECTORS	R-RNO-195258	9	-0.467014	-1.233821	0.204748	0.852782	1.000
619	GOBP_CELLULAR_RESPONSE_TO_CHEMICAL_STRESS	GO:0062197	7	-0.517462	-1.235686	0.229970	0.854351	1.000
620	GOBP_TUBE_DEVELOPMENT	GO:0035295	18	-0.376918	-1.236840	0.208564	0.858781	1.000
621	GOBP_MEMBRANE_PROTEIN_PROTEOLYSIS	GO:0033619	6	-0.532106	-1.237013	0.200603	0.866961	1.000
622	REACTOME_SIGNALING_BY_RHO_GTPASES_MIRO_GTPASES...	R-RNO-9716542	9	-0.467014	-1.237437	0.228614	0.874390	1.000
623	GOBP_CELL_CELL_JUNCTION_ORGANIZATION	GO:0045216	6	-0.539751	-1.244711	0.224060	0.855990	1.000
624	REACTOME_SIGNALING_BY_WNT	R-RNO-195721	7	-0.490777	-1.245724	0.202808	0.861006	1.000
625	GOBP_TUBE_MORPHOGENESIS	GO:0035239	15	-0.407913	-1.248990	0.203081	0.857874	1.000
626	GOBP_RESPONSE_TO_CALCIUM_ION	GO:0051592	6	-0.550809	-1.249243	0.196172	0.866307	1.000
627	GOBP_PEPTIDYL_LYSINE_MODIFICATION	GO:0018205	5	-0.577399	-1.256788	0.195618	0.847889	1.000
628	GOBP_RESPONSE_TO_LIPID	GO:0033993	19	-0.387268	-1.258514	0.188782	0.850135	1.000
629	GOBP_CELL_POPULATION_PROLIFERATION	GO:0008283	31	-0.349394	-1.259985	0.189857	0.854376	1.000
630	GOBP_AGING	GO:0007568	13	-0.434099	-1.269062	0.172943	0.830071	1.000
631	GOBP_RESPONSE_TO_OXIDATIVE_STRESS	GO:0006979	8	-0.503877	-1.273106	0.175150	0.824349	1.000
632	GOBP_MUSCLE_ORGAN_DEVELOPMENT	GO:0007517	9	-0.476076	-1.276722	0.189349	0.820506	1.000
633	GOBP_RESPONSE_TO_REACTIVE_OXYGEN_SPECIES	GO:0000302	8	-0.503877	-1.280173	0.185759	0.817224	1.000
634	GOBP_NEGATIVE_REGULATION_OF_RESPONSE_TO_EXTERN...	GO:0032102	5	-0.594458	-1.282683	0.143082	0.817937	1.000
635	GOBP_CELLULAR_RESPONSE_TO_REACTIVE_OXYGEN_SPECIES	GO:0034614	7	-0.517462	-1.284035	0.176901	0.822688	1.000
636	GOBP_NEGATIVE_REGULATION_OF_CELL_CYCLE	GO:0045786	5	-0.590361	-1.293442	0.153094	0.798017	1.000
637	GOBP_SKIN_DEVELOPMENT	GO:0043588	5	-0.590361	-1.296129	0.162939	0.797520	1.000
638	GOBP_PRI_MIRNA_TRANSCRIPTION_BY_RNA_POLYMERASE_II	GO:0061614	7	-0.543210	-1.301003	0.155172	0.789622	1.000
639	GOBP_NEGATIVE_REGULATION_OF_BIOSYNTHETIC_PROCESS	GO:0009890	13	-0.441104	-1.301772	0.145049	0.796712	1.000
640	GOBP_REGULATION_OF_IMMUNE_RESPONSE	GO:0050776	12	-0.445444	-1.303244	0.156338	0.801388	1.000
641	GOBP_POSITIVE_REGULATION_OF_APOPTOTIC_SIGNALIN...	GO:2001235	5	-0.590361	-1.303845	0.153846	0.809322	1.000
642	GOBP_NEGATIVE_REGULATION_OF_NUCLEOBASE_CONTAIN...	GO:0045934	13	-0.441144	-1.305200	0.154381	0.814562	1.000

Continuation of Table **S15**

	NAME	ID	SIZE	ES	NES	NOM.p.val	FDR.q.val	FWER.p.val
643	GOBP_POSITIVE_REGULATION_OF_PRI_MIRNA_TRANSCRI...	GO:1902895	7	-0.543210	-1.308474	0.147761	0.812417	1.000
644	GOBP_BIOLOGICAL_PROCESS_INVOLVED_IN_SYMBIOTIC_...	GO:004440	11	-0.468308	-1.313195	0.130564	0.804651	1.000
645	GOBP_POSITIVE_REGULATION_OF_CYTOKINE_PRODUCTION	GO:0001819	7	-0.529457	-1.315668	0.137558	0.806027	1.000
646	GOBP_TISSUE_HOMEOSTASIS	GO:0001894	5	-0.617752	-1.316657	0.154446	0.812683	1.000
647	REACTOME_TRANSCRIPTIONAL_REGULATION_BY_TP53	R-RNO-3700989	6	-0.568724	-1.320485	0.140060	0.808267	1.000
648	GOBP_MORPHOGENESIS_OF_AN_EPITHELIUM	GO:0002009	6	-0.564938	-1.320495	0.121396	0.819616	1.000
649	GOBP_RESPONSE_TO_CYTOKINE	GO:0034097	23	-0.384359	-1.321209	0.121751	0.828558	1.000
650	GOBP_RESPONSE_TO_RADIATION	GO:0009314	13	-0.453353	-1.324540	0.135135	0.826742	1.000
651	GOBP_RESPONSE_TO_BIOTIC_STIMULUS	GO:0009607	17	-0.420353	-1.324562	0.125683	0.838863	1.000
652	GOBP_ORGANONITROGEN_COMPOUND_CATABOLIC_PROCESS	GO:1901565	9	-0.499458	-1.328261	0.141384	0.836013	1.000
653	GOBP_CELL_CYCLE	GO:0007049	21	-0.402671	-1.334929	0.128137	0.821869	1.000
654	GOBP_OSTEOCLAST_DIFFERENTIATION	GO:0030316	6	-0.589089	-1.354193	0.121951	0.755060	1.000
655	GOBP_FC_RECEPTOR_SIGNALING_PATHWAY	GO:0038093	8	-0.533909	-1.357554	0.105797	0.753613	1.000
656	GOBP_IMMUNE_RESPONSE_REGULATING_SIGNALING_PATHWAY	GO:000276	8	-0.533909	-1.358258	0.126506	0.762686	1.000
657	GOBP_CIRCADIAN_RHYTHM	GO:0007623	13	-0.468835	-1.358722	0.122628	0.773043	1.000
658	REACTOME_RNA_POLYMERASE_II_TRANSCRIPTION	R-RNO-73857	20	-0.420654	-1.380226	0.098202	0.702127	1.000
659	REACTOME_G_ALPHA_Q_SIGNALLING_EVENTS	R-RNO-416476	6	-0.595291	-1.380667	0.117363	0.711960	1.000
660	REACTOME_CELLULAR_RESPONSES_TO_EXTERNAL_STIMULI	R-RNO-8953897	12	-0.491161	-1.392040	0.102817	0.681195	1.000
661	GOBP_CELLULAR_MACROMOLECULE_CATABOLIC_PROCESS	GO:0044265	6	-0.594837	-1.396994	0.096519	0.673791	1.000
662	GOBP_CYTOKINE_PRODUCTION	GO:0001816	10	-0.500590	-1.397545	0.098257	0.683218	1.000
663	GOBP_INTRACELLULAR_RECEPTOR_SIGNALING_PATHWAY	GO:0030522	5	-0.650602	-1.401082	0.076443	0.682430	1.000
664	GOBP_CELLULAR_RESPONSE_TO_EXTRACELLULAR_STIMULUS	GO:0031668	8	-0.552303	-1.404357	0.089419	0.682470	1.000
665	GOBP_COAGULATION	GO:0050817	8	-0.546685	-1.406119	0.104012	0.688634	1.000
666	REACTOME_INNATE_IMMUNE_SYSTEM	R-RNO-168249	16	-0.448105	-1.406603	0.111898	0.699762	1.000
667	GOBP_TISSUE_MORPHOGENESIS	GO:0048729	7	-0.576078	-1.420323	0.090484	0.659447	1.000
668	GOBP_PLATELET_ACTIVATION	GO:0030168	6	-0.612069	-1.423467	0.078025	0.660328	1.000
669	GOBP_REGULATION_OF_BODY_FLUID_LEVELS	GO:0050878	11	-0.504163	-1.428257	0.088496	0.655637	1.000
670	GOBP_CELLULAR_RESPONSE_TO_INORGANIC_SUBSTANCE	GO:0071241	11	-0.513412	-1.429643	0.089574	0.663382	1.000
671	GOBP_NEGATIVE_REGULATION_OF_PROTEIN_CATABOLIC_...	GO:0042177	5	-0.660691	-1.429925	0.082298	0.676168	1.000
672	GOBP_RESPONSE_TO_CADMIUM_ION	GO:0046686	6	-0.619557	-1.434778	0.056146	0.671078	1.000
673	REACTOME_HEMOSTASIS	R-RNO-109582	14	-0.467906	-1.442918	0.086131	0.655561	1.000
674	GOBP_REGULATION_OF_CELL_CELL_ADHESION	GO:0022407	9	-0.536418	-1.446826	0.076700	0.654301	1.000
675	GOBP_CELLULAR_RESPONSE_TO_HORMONE_STIMULUS	GO:0032870	11	-0.531242	-1.475629	0.063328	0.563856	1.000
676	REACTOME_MAPK_TARGETS_NUCLEAR_EVENTS_MEDIATED_...	R-HSA-450282	5	-0.676909	-1.478375	0.057692	0.567292	1.000
677	HALLMARK_TNFA_SIGNALING_VIA_NFKB	M5890	14	-0.499837	-1.492754	0.056277	0.532340	1.000
678	GOBP_REGULATION_OF_CELL_CYCLE	GO:0051726	13	-0.510416	-1.500639	0.052709	0.519832	1.000
679	REACTOME_BETA_CATENIN_INDEPENDENT_WNT_SIGNALING	R-RNO-3858494	5	-0.691304	-1.501532	0.046774	0.529601	1.000
680	GOBP_FC_EPSILON_RECEPTOR_SIGNALING_PATHWAY	GO:0038095	7	-0.622534	-1.509061	0.047041	0.517593	1.000
681	GOBP_REGULATION_OF_IMMUNE_SYSTEM_PROCESS	GO:0002682	21	-0.460122	-1.509232	0.049731	0.530836	1.000
682	GOBP_MACROMOLECULE_CATABOLIC_PROCESS	GO:0009057	12	-0.528122	-1.509751	0.056548	0.543332	1.000
683	REACTOME_MYD88_INDEPENDENT_TLR4_CASCADE	R-RNO-166166	8	-0.595824	-1.510770	0.049347	0.554601	1.000
684	REACTOME_INTERLEUKIN_17_SIGNALING	R-RNO-448424	6	-0.656629	-1.515415	0.041475	0.554572	1.000
685	GOBP_REGULATION_OF_MYELOID_LEUKOCYTE_DIFFERENT...	GO:0002761	8	-0.591163	-1.515754	0.067485	0.569509	1.000

Continuation of Table **S15**

	NAME	ID	SIZE	ES	NES	NOM.p.val	FDR.q.val	FWER.p.val
686	REACTOME_TOLL_LIKE_RECEPTOR_CASCADES	R-RNO-168898	8	-0.595824	-1.521964	0.047913	0.564319	1.000
687	REACTOME_TOLL_LIKE_RECEPTOR_9_TLR9_CASCADE	R-RNO-168138	8	-0.595824	-1.527133	0.040000	0.562309	1.000
688	REACTOME_FC_EPSILON_RECEPTOR_FCERI_SIGNALING	R-RNO-2454202	7	-0.622534	-1.530240	0.042105	0.567781	1.000
689	GOBP_RHYTHMIC_PROCESS	GO:0048511	15	-0.507901	-1.547678	0.038516	0.522763	1.000
690	REACTOME_TOLL_LIKE_RECEPTOR_TLR1_TLR2_CASCADE	R-RNO-168179	8	-0.595824	-1.557066	0.039394	0.506852	1.000
691	REACTOME_NGF_STIMULATED_TRANSCRIPTION	R-RNO-9031628	9	-0.579671	-1.564125	0.038348	0.497564	1.000
692	GOBP_POSITIVE_REGULATION_OF_IMMUNE_SYSTEM_PROCESS	GO:0002684	18	-0.481099	-1.572991	0.027510	0.485703	1.000
693	GOBP_RESPONSE_TO_INTERLEUKIN_1	GO:0070555	5	-0.699716	-1.575267	0.028892	0.496384	1.000
694	GOBP_POSITIVE_REGULATION_OF_MYELOID_CELL_DIFFE...	GO:0045639	7	-0.634592	-1.575442	0.039809	0.515600	1.000
695	REACTOME_NUCLEAR_EVENTS_KINASE_AND_TRANSCRIPTI...	R-RNO-198725	11	-0.558085	-1.589192	0.031161	0.486285	1.000
696	GOBP_IMMUNE_SYSTEM_DEVELOPMENT	GO:0002520	17	-0.505115	-1.592543	0.026426	0.495009	1.000
697	GOBP_POSITIVE_REGULATION_OF_MYELOID_LEUKOCYTE_...	GO:0002763	6	-0.698977	-1.609914	0.019118	0.455885	1.000
698	GOBP_RESPONSE_TO_BACTERIUM	GO:0009617	13	-0.551643	-1.614482	0.031474	0.461098	1.000
699	GOBP_POSITIVE_REGULATION_OF_BIOSYNTHETIC_PROCESS	GO:0009891	27	-0.460307	-1.616333	0.030914	0.477278	1.000
700	GOBP_TRANSMEMBRANE_RECEPTOR_PROTEIN_SERINE_THR...	GO:0007178	6	-0.684808	-1.618317	0.012480	0.494704	1.000
701	GOBP_RESPONSE_TO_MOLECULE_OF_BACTERIAL_ORIGIN	GO:0002237	13	-0.551643	-1.619766	0.031609	0.516284	1.000
702	HALLMARK_HYPOXIA	M5891	5	-0.747985	-1.623891	0.021104	0.529960	1.000
703	HALLMARK_APOPTOSIS	M5902	6	-0.715914	-1.643744	0.014218	0.483331	0.999
704	GOBP_REGULATION_OF_LEUKOCYTE_DIFFERENTIATION	GO:1902105	9	-0.625920	-1.666078	0.011799	0.433024	0.998
705	GOBP_REGULATION_OF_MYELOID_CELL_DIFFERENTIATION	GO:0045637	9	-0.627973	-1.667471	0.018547	0.459465	0.998
706	GOBP_MYELOID_LEUKOCYTE_DIFFERENTIATION	GO:0002573	11	-0.594605	-1.667495	0.017518	0.494679	0.998
707	GOBP_MYELOID_CELL_DIFFERENTIATION	GO:0030099	13	-0.572303	-1.675075	0.018651	0.503931	0.998
708	GOBP_CHROMOSOME_ORGANIZATION	GO:0051276	6	-0.735472	-1.679460	0.020093	0.528623	0.997
709	GOBP_POSITIVE_REGULATION_OF_NUCLEOBASE_CONTAIN...	GO:0045935	28	-0.482639	-1.682217	0.019894	0.569421	0.997
710	GOBP_RESPONSE_TO_MECHANICAL_STIMULUS	GO:0009612	6	-0.720544	-1.687154	0.008157	0.611013	0.997
711	GOBP_CHROMATIN_ORGANIZATION	GO:0006325	5	-0.795181	-1.712097	0.007899	0.562914	0.994
712	REACTOME_SENESCENCE_ASSOCIATED_SECRETORY_PHENO...	R-RNO-2559582	6	-0.767245	-1.745132	0.003150	0.481880	0.984
713	GOBP_POSITIVE_REGULATION_OF_TRANSCRIPTION_BY_R...	GO:0045944	24	-0.509287	-1.748578	0.020747	0.545625	0.983
714	REACTOME_CELLULAR_SENESCENCE	R-RNO-2559583	6	-0.767245	-1.780211	0.003175	0.492311	0.940
715	GOBP_LEUKOCYTE_DIFFERENTIATION	GO:0002521	15	-0.566125	-1.785010	0.008287	0.590950	0.931
716	GOBP_POSITIVE_REGULATION_OF_HEMOPOIESIS	GO:1903708	7	-0.724212	-1.787789	0.001582	0.768016	0.927
717	GOBP_REGULATION_OF_HEMOPOIESIS	GO:1903706	10	-0.656561	-1.814804	0.010606	0.887978	0.874
718	GOBP_RESPONSE_TO_DRUG	GO:0042493	11	-0.670402	-1.898578	0.004373	0.731224	0.574

End of Table

Supplementary Table S16: BG late profile (45 DPL peak) GSEA results.

Begin of Table **S16**

	NAME	ID	SIZE	ES	NES	NOM.p.val	FDR.q.val	FWER.p.val
0	GOBP_CELL_CYCLE	GO:0007049	21	0.688508	2.187956	0.000000	0.008712	0.007
1	GOBP_NEGATIVE_REGULATION_OF_GENE_EXPRESSION	GO:0010629	12	0.747671	2.080675	0.000000	0.027282	0.043
2	GOBP_POSTTRANSCRIPTIONAL_REGULATION_OF_GENE_EX...	GO:0010608	8	0.837801	2.059416	0.000000	0.022322	0.052
3	GOBP_CELLULAR_AMIDE_METABOLIC_PROCESS	GO:0043603	13	0.704689	2.000565	0.000000	0.042499	0.130
4	GOBP_GROWTH	GO:0040007	14	0.678726	1.941892	0.000000	0.075044	0.264
5	GOBP_GAMETE_GENERATION	GO:0007276	9	0.749268	1.908591	0.000000	0.091444	0.364
6	GOBP_PEPTIDE_METABOLIC_PROCESS	GO:0006518	12	0.689573	1.903534	0.001361	0.082782	0.383
7	GOBP_REGULATION_OF_CELLULAR_AMIDE_METABOLIC_PR...	GO:0034248	9	0.734005	1.870772	0.001397	0.112932	0.529
8	GOBP_MULTICELLULAR_ORGANISM_REPRODUCTION	GO:0032504	12	0.666189	1.852039	0.005369	0.127062	0.607
9	GOBP_ORGANONITROGEN_COMPOUND_BIOSYNTHETIC_PROCESS	GO:1901566	13	0.654662	1.849304	0.000000	0.117583	0.615
10	GOBP_NEGATIVE_REGULATION_OF_MULTICELLULAR_ORGA...	GO:0051241	15	0.627710	1.826315	0.005236	0.140529	0.730
11	GOBP_REGULATION_OF_GROWTH	GO:0040008	13	0.651571	1.811658	0.002714	0.150821	0.774
12	GOBP_POSITIVE_REGULATION_OF_MAP_KINASE_ACTIVITY	GO:0043406	6	0.798853	1.810989	0.004373	0.140642	0.779
13	GOBP_REGULATION_OF_MAP_KINASE_ACTIVITY	GO:0043405	9	0.701610	1.800464	0.002837	0.146364	0.812
14	GOBP_SEXUAL_REPRODUCTION	GO:0019953	10	0.690190	1.799481	0.002878	0.138514	0.815
15	GOBP_POSITIVE_REGULATION_OF_PROTEIN_SERINE_THR...	GO:0071902	9	0.686880	1.784689	0.008475	0.151177	0.861
16	GOBP_ACTIVATION_OF_MAPK_ACTIVITY	GO:0000187	6	0.798853	1.784683	0.001427	0.142284	0.861
17	GOBP_POSITIVE_REGULATION_OF_CELL_POPULATION_PR...	GO:0008284	21	0.565608	1.784369	0.005249	0.134584	0.861
18	GOBP_POSITIVE_REGULATION_OF_ESTABLISHMENT_OF_P...	GO:1904951	5	0.843560	1.766732	0.000000	0.154477	0.900
19	GOBP_REGULATION_OF_PROTEIN_SERINE_THREONINE_KI...	GO:0071900	12	0.639657	1.766392	0.001395	0.146994	0.900
20	GOBP_ERK1_AND_ERK2_CASCADE	GO:0070371	5	0.827784	1.765826	0.002981	0.140700	0.901
21	GOBP_MITOTIC_CELL_CYCLE	GO:0000278	10	0.676616	1.763109	0.008523	0.138416	0.906
22	GOBP_MUSCLE_ADAPTATION	GO:0043500	5	0.823221	1.760237	0.001618	0.137139	0.909
23	GOBP_MONOSACCHARIDE_METABOLIC_PROCESS	GO:0005996	6	0.775158	1.755633	0.003012	0.137359	0.922
24	GOBP_GLUCOSE_METABOLIC_PROCESS	GO:0006006	6	0.775158	1.750625	0.001484	0.138716	0.933
25	GOBP_POSITIVE_REGULATION_OF_CELL_ADHESION	GO:0045785	7	0.729700	1.743435	0.004348	0.143252	0.948
26	GOBP_CELL_GROWTH	GO:0016049	10	0.650283	1.734641	0.005772	0.149353	0.959
27	GOBP_REGULATION_OF_DNA_BINDING_TRANSCRIPTION_F...	GO:0051090	16	0.575079	1.725752	0.003937	0.156378	0.974
28	GOBP_REGULATION_OF_ANION_TRANSMEMBRANE_TRANSPORT	GO:1903959	5	0.801351	1.724875	0.009174	0.152392	0.975
29	GOBP_REGULATION_OF_CELLULAR_COMPONENT_MOVEMENT	GO:0051270	15	0.585625	1.718063	0.010430	0.158724	0.979
30	GOBP_AMIDE_BIOSYNTHETIC_PROCESS	GO:0043604	7	0.712430	1.710088	0.012640	0.166351	0.986
31	GOBP_DEVELOPMENTAL_PROCESS_INVOLVED_IN_REPRODU...	GO:0003006	11	0.631560	1.708764	0.014205	0.163182	0.987
32	GOBP_NEGATIVE_REGULATION_OF_MOLECULAR_FUNCTION	GO:0044092	24	0.527897	1.705909	0.008816	0.162268	0.987
33	GOBP_PROTEIN_DEPHOSPHORYLATION	GO:0006470	7	0.719714	1.705505	0.007267	0.157965	0.987
34	GOBP_ORGAN_GROWTH	GO:0035265	5	0.801419	1.705058	0.006192	0.153948	0.988
35	GOBP_PEPTIDE_BIOSYNTHETIC_PROCESS	GO:0043043	7	0.712430	1.702800	0.008451	0.152984	0.988
36	GOBP_CARBOHYDRATE_METABOLIC_PROCESS	GO:0005975	9	0.666849	1.700850	0.016997	0.151199	0.989
37	GOBP_REGULATION_OF_MUSCLE_ADAPTATION	GO:0043502	5	0.823221	1.693276	0.001563	0.158753	0.993
38	GOBP_DEPHOSPHORYLATION	GO:0016311	7	0.719714	1.688806	0.010057	0.161593	0.994
39	GOBP_MULTI_ORGANISM_PROCESS	GO:0051704	16	0.572056	1.688659	0.014925	0.157862	0.994
40	GOBP_POSITIVE_REGULATION_OF_LOCOMOTION	GO:0040017	11	0.630808	1.683650	0.012766	0.161349	0.994

Continuation of Table **S16**

	NAME	ID	SIZE	ES	NES	NOM.p.val	FDR.q.val	FWER.p.val
41	GOBP_REGULATION_OF_ORGAN_GROWTH	GO:0046620	5	0.801419	1.680083	0.013255	0.162600	0.996
42	GOBP_REGULATION_OF_PEPTIDE_SECRETION	GO:0002791	7	0.701645	1.661094	0.013699	0.188761	0.999
43	GOBP_NEGATIVE_REGULATION_OF_CELL_DIFFERENTIATION	GO:0045596	13	0.595946	1.658601	0.016086	0.188547	0.999
44	GOBP_NEGATIVE_REGULATION_OF_DEVELOPMENTAL_PROCESS	GO:0051093	13	0.595946	1.655921	0.017380	0.188221	0.999
45	GOBP_REGULATION_OF_PEPTIDE_TRANSPORT	GO:0090087	10	0.631603	1.655710	0.016506	0.184481	0.999
46	GOBP_NEGATIVE_REGULATION_OF_CELLULAR_COMPONENT...	GO:0051129	11	0.617579	1.655543	0.025352	0.180818	0.999
47	GOBP_RESPONSE_TO_TUMOR_NECROSIS_FACTOR	GO:0034612	7	0.707604	1.651983	0.012987	0.182869	0.999
48	GOBP_CELL_CYCLE_PROCESS	GO:0022402	11	0.599334	1.650543	0.019310	0.181391	0.999
49	GOBP_POSITIVE_REGULATION_OF_APOPTOTIC_SIGNALIN...	GO:2001235	5	0.783133	1.646733	0.010870	0.184106	1.000
50	GOBP_REGULATION_OF_ANION_TRANSPORT	GO:0044070	20	0.526105	1.645768	0.015645	0.182027	1.000
51	GOBP_EXTRINSIC_APOPTOTIC_SIGNALING_PATHWAY	GO:0097191	7	0.691442	1.638405	0.011799	0.189770	1.000
52	GOBP_REGULATION_OF_MULTICELLULAR_ORGANISMAL_DE...	GO:2000026	31	0.477615	1.632374	0.013514	0.195428	1.000
53	GOBP_MEMBRANE_PROTEIN_PROTEOLYSIS	GO:0033619	6	0.721138	1.631770	0.010903	0.193118	1.000
54	GOBP_MALE_GAMETE_GENERATION	GO:0048232	5	0.764139	1.620189	0.010989	0.209931	1.000
55	GOBP_CIRCULATORY_SYSTEM_DEVELOPMENT	GO:0072359	21	0.499822	1.617551	0.022078	0.210402	1.000
56	GOBP_CELLULAR_CARBOHYDRATE_METABOLIC_PROCESS	GO:0044262	5	0.749962	1.608577	0.018663	0.223117	1.000
57	GOBP_CELLULAR_RESPONSE_TO_EXTERNAL_STIMULUS	GO:0071496	10	0.614118	1.604973	0.021739	0.226382	1.000
58	GOBP_MUSCLE_CELL_PROLIFERATION	GO:0033002	8	0.656806	1.602217	0.011494	0.227589	1.000
59	GOBP_REGULATION_OF_CELLULAR_CATABOLIC_PROCESS	GO:0031329	10	0.612452	1.596786	0.022378	0.233894	1.000
60	GOBP_CELLULAR_GLUCOSE_HOMEOSTASIS	GO:0001678	5	0.739652	1.592208	0.019727	0.238339	1.000
61	GOBP_CELL_POPULATION_PROLIFERATION	GO:0008283	31	0.469021	1.590572	0.013189	0.237552	1.000
62	GOBP_REGULATION_OF_EXTRINSIC_APOPTOTIC_SIGNALI...	GO:2001236	6	0.704620	1.580704	0.023881	0.253528	1.000
63	GOBP_CELLULAR_RESPONSE_TO_OXYGEN_CONTAINING_CO...	GO:1901701	29	0.463362	1.580182	0.021303	0.250319	1.000
64	GOBP_ANION_TRANSMEMBRANE_TRANSPORT	GO:0098656	7	0.668632	1.579817	0.026826	0.247288	1.000
65	GOBP_REPRODUCTION	GO:0000003	18	0.510132	1.564785	0.032425	0.274049	1.000
66	GOBP_SKIN_DEVELOPMENT	GO:0043588	5	0.727062	1.555852	0.027950	0.288245	1.000
67	GOBP_CELLULAR_RESPONSE_TO_LIPID	GO:0071396	15	0.523995	1.548531	0.038889	0.300991	1.000
68	GOBP_CELLULAR_RESPONSE_TO_EXTRACELLULAR_STIMULUS	GO:0031668	8	0.632456	1.547673	0.026462	0.298607	1.000
69	GOBP_AMYLOID_BETA_METABOLIC_PROCESS	GO:0050435	5	0.722308	1.545967	0.015576	0.297773	1.000
70	GOBP_CELLULAR_COMPONENT_DISASSEMBLY	GO:0022411	6	0.694200	1.540547	0.025335	0.305784	1.000
71	GOBP_AMYLOID_PRECURSOR_PROTEIN_METABOLIC_PROCESS	GO:0042982	5	0.722308	1.539824	0.024206	0.303123	1.000
72	GOBP_AMYLOID_PRECURSOR_PROTEIN_CATABOLIC_PROCESS	GO:0042987	5	0.722308	1.534315	0.025797	0.311598	1.000
73	GOBP_REGULATION_OF_CARBOHYDRATE_METABOLIC_PROCESS	GO:0006109	5	0.711265	1.528994	0.027650	0.318735	1.000
74	GOBP_CELL_MIGRATION	GO:0016477	22	0.472978	1.526626	0.047739	0.320484	1.000
75	GOBP_PROTEIN_PHOSPHORYLATION	GO:0006468	34	0.446659	1.522357	0.035496	0.326567	1.000
76	GOBP_POSITIVE_REGULATION_OF_MAPK_CASCADE	GO:0043410	9	0.600959	1.520716	0.050992	0.325895	1.000
77	GOBP_ESTABLISHMENT_OF_PROTEIN_LOCALIZATION	GO:0045184	18	0.502325	1.517711	0.040682	0.329100	1.000
78	GOBP_PEPTIDE_SECRETION	GO:0002790	9	0.595202	1.516385	0.038028	0.327883	1.000
79	GOBP_LIPID_METABOLIC_PROCESS	GO:0006629	10	0.564921	1.516164	0.043988	0.324205	1.000
80	GOBP_HEART_DEVELOPMENT	GO:0007507	6	0.682015	1.515066	0.030257	0.322826	1.000
81	GOBP_NUCLEAR_TRANSPORT	GO:0051169	5	0.719320	1.511851	0.041481	0.326353	1.000
82	GOBP_POSITIVE_REGULATION_OF_MULTICELLULAR_ORGA...	GO:0051240	32	0.444260	1.511406	0.040964	0.323502	1.000
83	GOBP_PEPTIDE_HORMONE_SECRETION	GO:0030072	7	0.635005	1.511232	0.035211	0.319931	1.000

Continuation of Table **S16**

	NAME	ID	SIZE	ES	NES	NOM.p.val	FDR.q.val	FWER.p.val
84	GOBP_NEGATIVE_REGULATION_OF_PROTEIN_METABOLIC_...	GO:0051248	20	0.493293	1.511107	0.049682	0.316400	1.000
85	GOBP_CARDIAC_MUSCLE_TISSUE_DEVELOPMENT	GO:0048738	6	0.682015	1.507559	0.042587	0.320904	1.000
86	GOBP_POLYSACCHARIDE_METABOLIC_PROCESS	GO:0005976	5	0.712255	1.506432	0.034429	0.319805	1.000
87	GOBP_NEGATIVE_REGULATION_OF_SIGNALING	GO:0023057	21	0.472943	1.505065	0.046572	0.318868	1.000
88	GOBP_PROTEIN_LOCALIZATION_TO_ORGANELLE	GO:0033365	9	0.590261	1.504442	0.038905	0.316575	1.000
89	GOBP_NEGATIVE_REGULATION_OF_CATALYTIC_ACTIVITY	GO:0043086	17	0.499422	1.502805	0.044595	0.316882	1.000
90	GOBP_MUSCLE_TISSUE_DEVELOPMENT	GO:0060537	11	0.555288	1.501256	0.047026	0.316783	1.000
91	GOBP_CARBOHYDRATE_BIOSYNTHETIC_PROCESS	GO:0016051	5	0.711265	1.500809	0.042122	0.314355	1.000
92	GOBP_REGULATION_OF_MITOTIC_CELL_CYCLE	GO:0007346	5	0.699075	1.499104	0.029687	0.314417	1.000
93	REACTOME_CELL_CYCLE_MITOTIC	R-RNO-69278	5	0.708654	1.497430	0.035008	0.315158	1.000
94	GOBP_INSULIN_SECRETION	GO:0030073	7	0.635005	1.494897	0.046784	0.317424	1.000
95	GOBP_REGULATION_OF_MAPK_CASCADE	GO:0043408	13	0.525589	1.492548	0.049333	0.319076	1.000
96	GOBP_REGULATION_OF_CARBOHYDRATE_BIOSYNTHETIC_P...	GO:0043255	5	0.711265	1.491633	0.036641	0.317667	1.000
97	GOBP_REGULATION_OF_PHOSPHATIDYLINOSITOL_3_KINA...	GO:0014066	6	0.664193	1.488207	0.039514	0.322096	1.000
98	GOBP_REGULATION_OF_HORMONE_LEVELS	GO:0010817	12	0.539114	1.487776	0.062927	0.319780	1.000
99	GOBP_REGULATION_OF_MUSCLE_SYSTEM_PROCESS	GO:0090257	8	0.593085	1.487711	0.049635	0.316706	1.000
100	REACTOME_SIGNALING_BY_RHO_GTPASES_MIRO_GTPASES...	R-RNO-9716542	9	0.586295	1.486456	0.048951	0.316065	1.000
101	GOBP_SECRETION	GO:0046903	24	0.455603	1.483955	0.063452	0.318480	1.000
102	REACTOME_RHO_GTPASE_EFFECTORS	R-RNO-195258	9	0.586295	1.483700	0.050776	0.315982	1.000
103	GOBP_MUSCLE_CELL_DEVELOPMENT	GO:0055001	5	0.702409	1.483187	0.053731	0.314149	1.000
104	GOBP_CELLULAR_RESPONSE_TO_DNA_DAMAGE_STIMULUS	GO:0006974	5	0.700093	1.482426	0.041359	0.312940	1.000
105	REACTOME_SIGNALING_BY_GPCR	R-RNO-372790	22	0.459041	1.481280	0.051414	0.312404	1.000
106	GOBP_POSITIVE_REGULATION_OF_CELLULAR_COMPONENT...	GO:0051130	24	0.449022	1.474650	0.051546	0.323955	1.000
107	GOBP_DEVELOPMENTAL_GROWTH	GO:0048589	10	0.559522	1.473146	0.059219	0.324163	1.000
108	GOBP_REGULATION_OF_PROTEIN_STABILITY	GO:0031647	5	0.701171	1.470555	0.048632	0.326888	1.000
109	REACTOME_CELL_CYCLE	R-RNO-1640170	6	0.650876	1.467244	0.046407	0.331125	1.000
110	GOBP_REGULATION_OF_PEPTIDE_HORMONE_SECRETION	GO:0090276	6	0.658570	1.466014	0.049275	0.330835	1.000
111	GOBP_REGULATION_OF_PROTEIN_KINASE_ACTIVITY	GO:0045859	22	0.449097	1.463482	0.063325	0.333354	1.000
112	REACTOME_CIRCADIAN_CLOCK	R-HSA-400253	5	0.691443	1.461657	0.046656	0.334293	1.000
113	GOBP_ACTIVATION_OF_PROTEIN_KINASE_ACTIVITY	GO:0032147	11	0.535297	1.457646	0.067227	0.340153	1.000
114	GOBP_MACROMOLECULE_CATABOLIC_PROCESS	GO:0009057	12	0.522189	1.457064	0.077562	0.338406	1.000
115	GOBP_INOSITOL_LIPID_MEDIATED_SIGNALING	GO:0048017	8	0.587361	1.453691	0.079096	0.342817	1.000
116	REACTOME_DEATH_RECEPTOR_SIGNALLING	R-RNO-73887	5	0.682140	1.452914	0.049401	0.341342	1.000
117	REACTOME_G_ALPHA_Q_SIGNALLING_EVENTS	R-RNO-416476	6	0.645659	1.449127	0.078550	0.347137	1.000
118	GOBP_PHOSPHATIDYLINOSITOL_3_KINASE_SIGNALING	GO:0014065	7	0.599973	1.448985	0.078148	0.344482	1.000
119	GOBP_REGULATION_OF_TRANSFERASE_ACTIVITY	GO:0051338	22	0.449097	1.444248	0.082051	0.352067	1.000
120	GOBP_INTRACELLULAR_TRANSPORT	GO:0046907	11	0.532943	1.440905	0.083562	0.356632	1.000
121	GOBP_INTRACELLULAR_RECEPTOR_SIGNALING_PATHWAY	GO:0030522	5	0.682014	1.439067	0.079755	0.357722	1.000
122	REACTOME_DISEASES_OF_SIGNAL_TRANSDUCTION_BY_GR...	R-HSA-5663202	12	0.515641	1.438080	0.096454	0.357144	1.000
123	GOBP_REGULATION_OF_PHOSPHORUS_METABOLIC_PROCESS	GO:0051174	27	0.423754	1.437162	0.062344	0.356425	1.000
124	GOBP_REGULATION_OF_SMALL_MOLECULE_METABOLIC_PR...	GO:0062012	9	0.555652	1.434624	0.073171	0.359152	1.000
125	GOBP_NEGATIVE_REGULATION_OF_PHOSPHORYLATION	GO:0042326	7	0.602882	1.432820	0.064140	0.360320	1.000
126	GOBP_REGULATION_OF_PROTEIN_LOCALIZATION	GO:0032880	18	0.467685	1.431893	0.086050	0.359342	1.000

Continuation of Table **S16**

	NAME	ID	SIZE	ES	NES	NOM.p.val	FDR.q.val	FWER.p.val
127	GOBP_POSITIVE_REGULATION_OF_PROTEIN_PHOSPHORYL...	GO:0001934	23	0.442270	1.428026	0.091837	0.365005	1.000
128	GOBP_REGULATION_OF_APOPTOTIC_SIGNALING_PATHWAY	GO:2001233	10	0.545515	1.426399	0.070126	0.365903	1.000
129	GOBP_REGULATION_OF_VASCULATURE_DEVELOPMENT	GO:1901342	5	0.670521	1.424845	0.065282	0.366506	1.000
130	GOBP_POSITIVE_REGULATION_OF_ORGANELLE_ORGANIZA...	GO:0010638	10	0.535680	1.424758	0.077465	0.363830	1.000
131	GOBP_REGULATION_OF_CELL_DIFFERENTIATION	GO:0045595	36	0.412612	1.423751	0.075243	0.363213	1.000
132	GOBP_REGULATION_OF_PROTEIN_PHOSPHORYLATION	GO:0001932	26	0.432652	1.422958	0.075031	0.362126	1.000
133	GOBP_POSITIVE_REGULATION_OF_NUCLEOBASE_CONTAIN...	GO:0045935	28	0.420682	1.421636	0.075871	0.362526	1.000
134	GOBP_INTRACELLULAR_PROTEIN_TRANSPORT	GO:0006886	9	0.565899	1.420945	0.093931	0.361380	1.000
135	GOBP_REGULATION_OF_HORMONE_SECRETION	GO:0046883	8	0.581110	1.419628	0.085554	0.361742	1.000
136	REACTOME_PLATELET_ACTIVATION_SIGNALING_AND_AGG...	R-HSA-76002	7	0.602485	1.417533	0.071839	0.363887	1.000
137	GOBP_POSITIVE_REGULATION_OF_PROTEIN_MODIFICATI...	GO:0031401	23	0.442270	1.416368	0.082687	0.363833	1.000
138	GOBP_TISSUE_MORPHOGENESIS	GO:0048729	7	0.611578	1.415876	0.066474	0.362201	1.000
139	GOBP_DNA_METABOLIC_PROCESS	GO:0006259	6	0.624629	1.411682	0.096070	0.368744	1.000
140	GOBP_NEGATIVE_REGULATION_OF_PHOSPHORUS_METABOL...	GO:0010563	9	0.561169	1.411395	0.089655	0.366827	1.000
141	GOBP_HORMONE_TRANSPORT	GO:0009914	9	0.565425	1.410075	0.092287	0.367036	1.000
142	GOBP_POSITIVE_REGULATION_OF_GROWTH	GO:0045927	9	0.554940	1.408940	0.084626	0.366708	1.000
143	GOBP_CARBOHYDRATE_HOMEOSTASIS	GO:0033500	7	0.591453	1.408855	0.085014	0.364359	1.000
144	REACTOME_INNATE_IMMUNE_SYSTEM	R-RNO-168249	16	0.470422	1.408168	0.091146	0.363221	1.000
145	GOBP_POSITIVE_REGULATION_OF_BIOSYNTHETIC_PROCESS	GO:0009891	27	0.428590	1.406929	0.085242	0.363299	1.000
146	GOBP_EMBRYO_DEVELOPMENT	GO:0009790	14	0.479642	1.402893	0.090066	0.370051	1.000
147	GOBP_NEGATIVE_REGULATION_OF_HYDROLASE_ACTIVITY	GO:0051346	11	0.512405	1.402540	0.076596	0.368365	1.000
148	REACTOME_OPIOID_SIGNALLING	R-RNO-111885	12	0.505661	1.401353	0.106648	0.368777	1.000
149	GOBP_REGULATION_OF_CELL_CYCLE_PROCESS	GO:0010564	5	0.659436	1.400113	0.081325	0.369255	1.000
150	REACTOME_SIGNALING_BY_INTERLEUKINS	R-RNO-449147	15	0.475314	1.396138	0.088591	0.375485	1.000
151	GOBP_REGULATION_OF_INTRACELLULAR_SIGNAL_TRANSD...	GO:1902531	21	0.443092	1.390477	0.101430	0.385886	1.000
152	GOBP_WOUND_HEALING	GO:0042060	13	0.484829	1.389330	0.087379	0.385880	1.000
153	GOBP_POSITIVE_REGULATION_OF_PROTEIN_KINASE_ACT...	GO:0045860	19	0.451097	1.386529	0.093298	0.389640	1.000
154	GOBP_LEUKOCYTE_MIGRATION	GO:0050900	6	0.623666	1.386375	0.074405	0.387382	1.000
155	GOBP_NEGATIVE_REGULATION_OF_CELL_CYCLE	GO:0045786	5	0.650330	1.383859	0.090016	0.390544	1.000
156	GOBP_POSITIVE_REGULATION_OF_PEPTIDYL_TYROSINE_...	GO:0050731	8	0.558929	1.379317	0.109705	0.398812	1.000
157	GOBP_POSITIVE_REGULATION_OF_LEUKOCYTE_CELL_CEL...	GO:1903039	5	0.659362	1.375481	0.080425	0.405416	1.000
158	GOBP_REGULATION_OF_PEPTIDYL_TYROSINE_PHOSPHORY...	GO:0050730	9	0.537944	1.375472	0.117898	0.402897	1.000
159	GOBP_POSITIVE_REGULATION_OF_CELL_CELL_ADHESION	GO:0022409	5	0.659362	1.372807	0.099248	0.406428	1.000
160	GOBP_SMALL_MOLECULE_METABOLIC_PROCESS	GO:0044281	15	0.464769	1.372731	0.097497	0.404128	1.000
161	GOBP_POSITIVE_REGULATION_OF_MOLECULAR_FUNCTION	GO:0044093	38	0.391160	1.372464	0.099515	0.402239	1.000
162	GOBP_REGULATION_OF_CELL_ADHESION	GO:0030155	13	0.485208	1.372309	0.113855	0.400022	1.000
163	GOBP_REGULATION_OF_CATABOLIC_PROCESS	GO:0009894	13	0.485129	1.370629	0.103356	0.401187	1.000
164	GOBP_CYTOKINE_PRODUCTION	GO:0001816	10	0.515139	1.368169	0.111111	0.404171	1.000
165	GOBP_REGULATION_OF_ANATOMICAL_STRUCTURE_MORPHO...	GO:0022603	17	0.456246	1.367727	0.103811	0.402754	1.000
166	GOBP_NEGATIVE_REGULATION_OF_TRANSPORT	GO:0051051	13	0.487307	1.366904	0.095890	0.402385	1.000
167	GOBP_REGULATION_OF_PROTEIN_MODIFICATION_PROCESS	GO:0031399	29	0.404457	1.366818	0.101759	0.400154	1.000
168	GOBP_POSITIVE_REGULATION_OF_PHOSPHORUS_METABOL...	GO:0010562	24	0.420828	1.366376	0.094645	0.398889	1.000
169	GOBP_RHYTHMIC_PROCESS	GO:0048511	15	0.466049	1.364993	0.104497	0.399738	1.000

Continuation of Table **S16**

	NAME	ID	SIZE	ES	NES	NOM.p.val	FDR.q.val	FWER.p.val
170	GOBP_POSITIVE_REGULATION_OF_INTRACELLULAR_SIGN...	GO:1902533	14	0.482014	1.362877	0.114911	0.402024	1.000
171	REACTOME_TOLL_LIKE_RECEPTOR_TLR1_TLR2_CASCADE	R-RNO-168179	8	0.548196	1.361252	0.119829	0.403425	1.000
172	GOBP_RESPONSE_TO_LIPID	GO:0033993	19	0.440622	1.359410	0.102394	0.405305	1.000
173	REACTOME_SIGNALING_BY_RECEPTOR_TYROSINE_KINASES	R-RNO-9006934	26	0.411548	1.357700	0.105721	0.406869	1.000
174	GOBP_POSITIVE_REGULATION_OF_TRANSFERASE_ACTIVITY	GO:0051347	20	0.426411	1.357673	0.110115	0.404586	1.000
175	REACTOME_CYTOKINE_SIGNALING_IN_IMMUNE_SYSTEM	R-RNO-1280215	21	0.418228	1.357347	0.095541	0.402929	1.000
176	GOBP_REGULATION_OF_SYSTEM_PROCESS	GO:0044057	18	0.445123	1.355734	0.107527	0.404441	1.000
177	GOBP_BIOLOGICAL_ADHESION	GO:0022610	19	0.431715	1.355368	0.114213	0.403013	1.000
178	REACTOME_TOLL_LIKE_RECEPTOR_9_TLR9_CASCADE	R-RNO-168138	8	0.548196	1.354986	0.118477	0.401524	1.000
179	GOBP_REGULATION_OF_IMMUNE_SYSTEM_PROCESS	GO:0002682	21	0.425944	1.354439	0.122581	0.400468	1.000
180	GOBP_CELL_ACTIVATION	GO:0001775	21	0.423276	1.350968	0.115183	0.405431	1.000
181	GOBP_MUSCLE_SYSTEM_PROCESS	GO:0003012	9	0.535406	1.346895	0.127168	0.411836	1.000
182	REACTOME_TOLL_LIKE_RECEPTOR_CASCADES	R-RNO-168898	8	0.548196	1.345482	0.123769	0.412702	1.000
183	GOBP_CELLULAR_RESPONSE_TO_ORGANIC_CYCLIC_COMPOUND	GO:0071407	13	0.483889	1.345478	0.119522	0.410459	1.000
184	GOBP_CELLULAR_LIPID_METABOLIC_PROCESS	GO:0044255	7	0.567552	1.343670	0.132267	0.412495	1.000
185	GOBP_ANIMAL_ORGAN_MORPHOGENESIS	GO:0009887	12	0.485941	1.342009	0.127717	0.413986	1.000
186	GOBP_POSITIVE_REGULATION_OF_ANION_TRANSPORT	GO:1903793	9	0.523845	1.339686	0.124128	0.417229	1.000
187	GOBP_DENDRITE_MORPHOGENESIS	GO:0048813	6	0.609136	1.339594	0.116864	0.415200	1.000
188	GOBP_NEGATIVE_REGULATION_OF_CELL_DEVELOPMENT	GO:0010721	6	0.597567	1.338643	0.142857	0.415158	1.000
189	GOBP_AGING	GO:0007568	13	0.478849	1.338337	0.098886	0.413548	1.000
190	GOBP_POSITIVE_REGULATION_OF_SMALL_MOLECULE_MET...	GO:0062013	5	0.626517	1.337757	0.117647	0.412663	1.000
191	GOBP_POSITIVE_REGULATION_OF_PHOSPHATIDYLINOSIT...	GO:0014068	5	0.640491	1.335813	0.106481	0.414744	1.000
192	GOBP_REGULATION_OF_CELL_DEVELOPMENT	GO:0060284	15	0.457587	1.333042	0.124332	0.418799	1.000
193	GOBP_POSITIVE_REGULATION_OF_GENE_EXPRESSION	GO:0010628	16	0.449029	1.328050	0.131613	0.427672	1.000
194	GOBP_PEPTIDYL_TYROSINE_MODIFICATION	GO:0018212	10	0.505245	1.327831	0.139535	0.426020	1.000
195	REACTOME_MYD88_INDEPENDENT_TLR4_CASCADE	R-RNO-166166	8	0.548196	1.326410	0.127479	0.427070	1.000
196	GOBP_CELL_CELL_JUNCTION_ORGANIZATION	GO:0045216	6	0.578863	1.326052	0.140741	0.425630	1.000
197	GOBP_CYTOSKELETON_ORGANIZATION	GO:0007010	11	0.486544	1.325712	0.139860	0.424272	1.000
198	GOBP_CIRCADIAN_REGULATION_OF_GENE_EXPRESSION	GO:0032922	5	0.638993	1.323538	0.125387	0.426849	1.000
199	GOBP_NEGATIVE_REGULATION_OF_PEPTIDASE_ACTIVITY	GO:0010466	6	0.583218	1.322887	0.135878	0.426422	1.000
200	GOBP_REGULATION_OF_DEVELOPMENTAL_GROWTH	GO:0048638	9	0.512722	1.317824	0.154596	0.436173	1.000
201	GOBP_REGULATION_OF_CELL_CYCLE	GO:0051726	13	0.466994	1.317583	0.153215	0.434634	1.000
202	GOBP_REGULATION_OF_VESICLE_MEDIATED_TRANSPORT	GO:0060627	12	0.472274	1.317058	0.144847	0.433619	1.000
203	REACTOME_NUCLEAR_EVENTS_KINASE_AND_TRANSCRIPTI...	R-RNO-198725	11	0.490012	1.316712	0.160443	0.432249	1.000
204	GOBP_IMMUNE_EFFECTOR_PROCESS	GO:0002252	10	0.491873	1.313193	0.153619	0.437955	1.000
205	GOBP_PEPTIDYL_AMINO_ACID_MODIFICATION	GO:0018193	22	0.405121	1.311419	0.150524	0.439718	1.000
206	GOBP_NEGATIVE_REGULATION_OF_ANION_TRANSPORT	GO:1903792	8	0.531564	1.308292	0.145533	0.444595	1.000
207	GOBP_NEGATIVE_REGULATION_OF_KINASE_ACTIVITY	GO:0033673	5	0.616901	1.307733	0.142410	0.443731	1.000
208	GOBP_POSITIVE_REGULATION_OF_RESPONSE_TO_EXTERN...	GO:0032103	9	0.509486	1.305695	0.130682	0.446492	1.000
209	GOBP_FC_EPSILON_RECEPTOR_SIGNALING_PATHWAY	GO:0038095	7	0.546725	1.304911	0.150585	0.446127	1.000
210	GOBP_RESPONSE_TO_ABIOTIC_STIMULUS	GO:0009628	27	0.391948	1.303733	0.150127	0.446530	1.000
211	GOBP_CIRCADIAN_RHYTHM	GO:0007623	13	0.467047	1.302695	0.151099	0.446720	1.000
212	GOBP_POSITIVE_REGULATION_OF_DEVELOPMENTAL_PROCESS	GO:0051094	32	0.378183	1.301815	0.144623	0.446595	1.000

Continuation of Table **S16**

	NAME	ID	SIZE	ES	NES	NOM.p.val	FDR.q.val	FWER.p.val
213	GOBP_AMEBOIDAL_TYPE_CELL_MIGRATION	GO:0001667	11	0.479249	1.301809	0.155492	0.444520	1.000
214	GOBP_CELLULAR_RESPONSE_TO_BIOTIC_STIMULUS	GO:0071216	8	0.528909	1.300663	0.155340	0.445004	1.000
215	GOBP_RESPONSE_TO_METAL_ION	GO:0010038	15	0.438684	1.300163	0.146405	0.444211	1.000
216	GOBP_MAPK_CASCADE	GO:0000165	18	0.427742	1.296140	0.150633	0.451464	1.000
217	GOBP_MAINTENANCE_OF_LOCATION	GO:0051235	7	0.549026	1.295737	0.182891	0.450090	1.000
218	GOBP_REGULATION_OF_EPITHELIAL_CELL_MIGRATION	GO:0010632	6	0.572369	1.292844	0.151376	0.454628	1.000
219	GOBP_MORPHOGENESIS_OF_AN_EPITHELIUM	GO:0002009	6	0.568842	1.290065	0.156923	0.458745	1.000
220	GOBP_CELLULAR_RESPONSE_TO_MOLECULE_OF_BACTERIA...	GO:0071219	8	0.528909	1.287259	0.159884	0.462789	1.000
221	REACTOME_FC_EPSILON_RECEPTOR_FCERI_SIGNALING	R-RNO-2454202	7	0.546725	1.285485	0.175809	0.465018	1.000
222	HALLMARK_PI3K_AKT_MTOR_SIGNALING	M5923	6	0.584171	1.284133	0.162003	0.466143	1.000
223	GOBP_NEGATIVE_REGULATION_OF_TRANSFERASE_ACTIVITY	GO:0051348	5	0.616901	1.279802	0.155146	0.474391	1.000
224	GOBP_NEGATIVE_REGULATION_OF_LIPID_METABOLIC_PR...	GO:0045833	5	0.596416	1.277023	0.151033	0.478635	1.000
225	GOBP_LEUKOCYTE_MEDIATED_IMMUNITY	GO:0002443	7	0.544984	1.276300	0.186289	0.478114	1.000
226	GOBP_BIOLOGICAL_PROCESS_INVOLVED_IN_SYMBIOTIC_...	GO:004440	11	0.468690	1.276127	0.187151	0.476431	1.000
227	GOBP_NEGATIVE_REGULATION_OF_PROTEIN_MODIFICATI...	GO:0031400	10	0.488954	1.275801	0.167147	0.475111	1.000
228	GOBP_RESPIRATORY_SYSTEM_DEVELOPMENT	GO:0060541	6	0.562539	1.275423	0.155750	0.473829	1.000
229	GOBP_REGULATION_OF_RESPONSE_TO_EXTERNAL_STIMULUS	GO:0032101	15	0.440306	1.274191	0.166223	0.474702	1.000
230	GOBP_PHAGOCYTOSIS	GO:0006909	5	0.596194	1.272247	0.178102	0.477093	1.000
231	GOBP_REGULATION_OF_IMMUNE_RESPONSE	GO:0050776	12	0.463542	1.269733	0.178025	0.480603	1.000
232	GOBP_CELLULAR_MACROMOLECULE_CATABOLIC_PROCESS	GO:0044265	6	0.565084	1.266547	0.186930	0.485381	1.000
233	GOBP_POSITIVE_REGULATION_OF_IMMUNE_RESPONSE	GO:0050778	9	0.494630	1.265115	0.183616	0.486473	1.000
234	GOBP_IMMUNE_SYSTEM_DEVELOPMENT	GO:0002520	17	0.421271	1.261054	0.198693	0.493671	1.000
235	GOBP_RESPONSE_TO_OXYGEN_CONTAINING_COMPOUND	GO:1901700	39	0.363294	1.259069	0.182578	0.496209	1.000
236	GOBP_EPIDERMIS_DEVELOPMENT	GO:0008544	5	0.592191	1.255675	0.186414	0.501873	1.000
237	GOBP_RESPONSE_TO_STARVATION	GO:0042594	5	0.599538	1.255592	0.167428	0.499952	1.000
238	REACTOME_ONCOGENIC_MAPK_SIGNALING	R-HSA-6802957	5	0.579567	1.255411	0.160247	0.498307	1.000
239	GOBP_REGULATION_OF_SECRETION	GO:0051046	14	0.440896	1.254781	0.198639	0.497608	1.000
240	GOBP_REGULATION_OF_PROTEIN_LOCALIZATION_TO_CEL...	GO:1904375	6	0.556410	1.252942	0.175150	0.499745	1.000
241	GOBP_PROTEIN_KINASE_B_SIGNALING	GO:0043491	6	0.553920	1.249983	0.198519	0.504670	1.000
242	GOBP_LIPID_BIOSYNTHETIC_PROCESS	GO:0008610	6	0.551339	1.248703	0.177273	0.505417	1.000
243	GOBP_GENERATION_OF_PRECURSOR_METABOLITES_AND_E...	GO:0006091	5	0.591884	1.247717	0.175655	0.505510	1.000
244	GOBP_EXOCYTOSIS	GO:0006887	11	0.467386	1.247404	0.194602	0.504197	1.000
245	GOBP_SYNAPTIC_TRANSMISSION_GLUTAMATERGIC	GO:0035249	13	0.443172	1.245997	0.199450	0.505260	1.000
246	GOBP_CELL_CELL_SIGNALING_BY_WNT	GO:0198738	7	0.532574	1.245715	0.199702	0.503792	1.000
247	GOBP_TISSUE_MIGRATION	GO:0090130	9	0.492764	1.245689	0.192253	0.501841	1.000
248	GOBP_CELLULAR_RESPONSE_TO_STARVATION	GO:0009267	5	0.599538	1.245307	0.197550	0.500686	1.000
249	HALLMARK_ALLOGRAFT_REJECTION	M5950	7	0.523029	1.244982	0.190407	0.499321	1.000
250	GOBP_RESPONSE_TO_NICOTINE	GO:0035094	7	0.520972	1.244914	0.196221	0.497497	1.000
251	GOBP_RESPONSE_TO_HEAT	GO:0009408	7	0.526650	1.244487	0.207101	0.496383	1.000
252	GOBP_REGULATION_OF_LIPID_BIOSYNTHETIC_PROCESS	GO:0046890	6	0.551339	1.240934	0.206186	0.502223	1.000
253	GOBP_NUCLEOBASE_CONTAINING_SMALL_MOLECULE_META...	GO:0055086	6	0.562170	1.240793	0.171303	0.500524	1.000
254	GOBP_DENDRITE_DEVELOPMENT	GO:0016358	9	0.485680	1.239314	0.200577	0.501961	1.000
255	GOBP_NEGATIVE_REGULATION_OF_PROTEOLYSIS	GO:0045861	8	0.498687	1.238821	0.198839	0.501059	1.000

Continuation of Table **S16**

	NAME	ID	SIZE	ES	NES	NOM.p.val	FDR.q.val	FWER.p.val
256	GOBP_REGULATION_OF_MUSCLE_CELL_DIFFERENTIATION	GO:0051147	6	0.546523	1.237255	0.196839	0.502591	1.000
257	GOBP_RESPONSE_TO_TEMPERATURE_STIMULUS	GO:0009266	9	0.491301	1.235251	0.219686	0.505296	1.000
258	GOBP_RESPONSE_TO_WOUNDING	GO:0009611	15	0.419366	1.232915	0.213406	0.508311	1.000
259	GOBP_SENSORY_ORGAN_DEVELOPMENT	GO:0007423	8	0.497150	1.231893	0.208824	0.508529	1.000
260	HALLMARK_UV_RESPONSE_UP	M5941	5	0.582492	1.231775	0.184664	0.506828	1.000
261	GOBP_REGULATION_OF_LIPID_LOCALIZATION	GO:1905952	5	0.585014	1.230809	0.209302	0.507033	1.000
262	GOBP_CELLULAR_HOMEOSTASIS	GO:0019725	18	0.399758	1.230119	0.207124	0.506487	1.000
263	GOBP_CALCIUM_MEDIATED_SIGNALING	GO:0019722	10	0.472657	1.229017	0.231760	0.507055	1.000
264	GOBP_GLIOGENESIS	GO:0042063	12	0.443824	1.226090	0.215924	0.511621	1.000
265	GOBP_MUSCLE_CELL_DIFFERENTIATION	GO:0042692	10	0.467184	1.225729	0.216939	0.510547	1.000
266	GOBP_NEGATIVE_REGULATION_OF_NUCLEOBASE_CONTAIN...	GO:0045934	13	0.432858	1.222027	0.236209	0.516921	1.000
267	GOBP_EPITHELIUM_DEVELOPMENT	GO:0060429	15	0.411775	1.221909	0.215405	0.515219	1.000
268	GOBP_NEGATIVE_REGULATION_OF_BINDING	GO:0051100	6	0.533769	1.220617	0.219178	0.516132	1.000
269	GOBP_NEGATIVE_REGULATION_OF_CELL_DEATH	GO:0060548	22	0.382921	1.220571	0.209476	0.514344	1.000
270	GOBP_ENDOCYTOSIS	GO:0006897	8	0.485744	1.219781	0.245763	0.514211	1.000
271	REACTOME_CREB1_PHOSPHORYLATION_THROUGH_NMDA_RE...	R-RNO-442742	8	0.492374	1.218431	0.221264	0.515310	1.000
272	GOBP_MYELOID_LEUKOCYTE_ACTIVATION	GO:0002274	8	0.498349	1.217270	0.229885	0.515757	1.000
273	GOBP_POSITIVE_REGULATION_OF_GLIOGENESIS	GO:0014015	5	0.581865	1.216961	0.208889	0.514500	1.000
274	GOBP_REGULATION_OF_GLIOGENESIS	GO:0014013	5	0.581865	1.216936	0.210031	0.512683	1.000
275	GOBP_VASCULATURE_DEVELOPMENT	GO:0001944	17	0.403699	1.214326	0.218016	0.516391	1.000
276	GOBP_REGULATION_OF_CIRCADIAN_RHYTHM	GO:0042752	7	0.515882	1.212905	0.229607	0.517650	1.000
277	GOBP_PROTEIN_CONTAINING_COMPLEX_SUBUNIT_ORGANI...	GO:0043933	14	0.422913	1.211492	0.206199	0.518871	1.000
278	GOBP_IMMUNE_RESPONSE_REGULATING_SIGNALING_PATHWAY	GO:0002764	8	0.486627	1.209947	0.232353	0.520420	1.000
279	GOBP_NEGATIVE_REGULATION_OF_TRANSCRIPTION_BY_R...	GO:0000122	10	0.453957	1.208252	0.224688	0.522156	1.000
280	GOBP_FC_RECEPTOR_SIGNALING_PATHWAY	GO:0038093	8	0.486627	1.205783	0.235123	0.526148	1.000
281	GOBP_CELL_CELL_ADHESION	GO:0098609	14	0.415307	1.204427	0.242991	0.527179	1.000
282	GOBP_NEUROINFLAMMATORY_RESPONSE	GO:0150076	5	0.573692	1.203893	0.228482	0.526408	1.000
283	REACTOME_G_ALPHA_I_SIGNALLING_EVENTS	R-RNO-418594	18	0.389164	1.203531	0.228000	0.525298	1.000
284	GOBP_POSITIVE_REGULATION_OF_IMMUNE_SYSTEM_PROCESS	GO:0002684	18	0.391003	1.203228	0.245235	0.524112	1.000
285	GOBP_POSITIVE_REGULATION_OF_PROTEIN_METABOLIC_...	GO:0051247	28	0.361105	1.201013	0.245211	0.527087	1.000
286	GOBP_REGULATION_OF_TRANSPORT	GO:0051049	44	0.341687	1.200031	0.233129	0.527383	1.000
287	GOBP_NEGATIVE_REGULATION_OF_RESPONSE_TO_STIMULUS	GO:0048585	16	0.403143	1.196681	0.252964	0.532740	1.000
288	GOBP_NEGATIVE_REGULATION_OF_BIOSYNTHETIC_PROCESS	GO:0009890	13	0.427791	1.196112	0.240163	0.532252	1.000
289	GOBP_REGULATION_OF_CELLULAR_RESPONSE_TO_STRESS	GO:0080135	9	0.464100	1.195211	0.243243	0.532273	1.000
290	GOBP_RESPONSE_TO_EXTRACELLULAR_STIMULUS	GO:0009991	13	0.415206	1.194894	0.252604	0.531049	1.000
291	GOBP_REGULATION_OF_PROTEIN_LOCALIZATION_TO_MEM...	GO:1905475	7	0.500774	1.194333	0.247344	0.530510	1.000
292	REACTOME_ADAPTIVE_IMMUNE_SYSTEM	R-RNO-1280218	9	0.471049	1.192328	0.250350	0.532769	1.000
293	REACTOME_INTERLEUKIN_17_SIGNALING	R-RNO-448424	6	0.529888	1.188046	0.240713	0.540449	1.000
294	GOBP_ANATOMICAL_STRUCTURE_FORMATION_INVOLVED_I...	GO:0048646	20	0.376960	1.187418	0.251664	0.539973	1.000
295	GOBP_REGULATION_OF_NEUROGENESIS	GO:0050767	14	0.413505	1.186522	0.236559	0.540083	1.000
296	GOBP_REGULATION_OF_ENDOCYTOSIS	GO:0030100	6	0.529622	1.186454	0.256757	0.538483	1.000
297	GOBP_NEGATIVE_REGULATION_OF_RESPONSE_TO_EXTERN...	GO:0032102	5	0.550816	1.186100	0.243865	0.537413	1.000
298	GOBP_REGULATION_OF_WNT_SIGNALING_PATHWAY	GO:0030111	5	0.542501	1.183165	0.259601	0.541996	1.000

Continuation of Table **S16**

	NAME	ID	SIZE	ES	NES	NOM.p.val	FDR.q.val	FWER.p.val
299	GOBP_NEGATIVE_REGULATION_OF_INTRACELLULAR_SIGN...	GO:1902532	7	0.492611	1.180187	0.253201	0.546695	1.000
300	GOBP_OSSIFICATION	GO:0001503	9	0.455032	1.179942	0.266003	0.545307	1.000
301	GOBP_RESPONSE_TO_CYTOKINE	GO:0034097	23	0.359814	1.177830	0.272050	0.547783	1.000
302	GOBP_APOPTOTIC_SIGNALING_PATHWAY	GO:0097190	15	0.403423	1.176062	0.248340	0.549870	1.000
303	GOBP_CELL_SURFACE_RECEPTOR_SIGNALING_PATHWAY_I...	GO:1905114	15	0.396832	1.174149	0.253012	0.551920	1.000
304	GOBP_APOPTOTIC_PROCESS	GO:0006915	35	0.339034	1.173946	0.268382	0.550526	1.000
305	GOBP_IN_UTERO_EMBRYONIC_DEVELOPMENT	GO:0001701	8	0.476621	1.171853	0.279346	0.553468	1.000
306	GOBP_CELLULAR_MACROMOLECULE_LOCALIZATION	GO:0070727	21	0.367995	1.169685	0.260638	0.556296	1.000
307	GOBP_GLIAL_CELL_DIFFERENTIATION	GO:0010001	10	0.447063	1.165763	0.301653	0.563125	1.000
308	GOBP_POSITIVE_REGULATION_OF_DEVELOPMENTAL_GROWTH	GO:0048639	8	0.474140	1.165329	0.288160	0.562248	1.000
309	GOBP_MYELOID_LEUKOCYTE_MEDIATED_IMMUNITY	GO:0002444	6	0.519028	1.164971	0.282511	0.561272	1.000
310	GOBP_TUBE_MORPHOGENESIS	GO:0035239	15	0.401487	1.164474	0.294355	0.560555	1.000
311	GOBP_CELLULAR_RESPONSE_TO_ABIOTIC_STIMULUS	GO:0071214	7	0.499442	1.164237	0.277126	0.559294	1.000
312	GOBP_CELL_ACTIVATION_INVOLVED_IN_IMMUNE_RESPONSE	GO:0002263	6	0.519028	1.163116	0.275510	0.559941	1.000
313	GOBP_POSITIVE_REGULATION_OF_CELL_DIFFERENTIATION	GO:0045597	27	0.348091	1.156402	0.286076	0.572871	1.000
314	GOBP_MYOTUBE_DIFFERENTIATION	GO:0014902	5	0.549092	1.155885	0.280063	0.572102	1.000
315	GOBP_FAT_CELL_DIFFERENTIATION	GO:0045444	9	0.453397	1.155280	0.279137	0.571640	1.000
316	GOBP_SECOND_MESSENGER_MEDIATED_SIGNALING	GO:0019932	16	0.393316	1.154762	0.289438	0.570992	1.000
317	GOBP_POSITIVE_REGULATION_OF_SMOOTH_MUSCLE_CELL...	GO:0048661	5	0.548366	1.154630	0.276074	0.569493	1.000
318	GOBP_DEVELOPMENTAL_MATURATION	GO:0021700	5	0.550425	1.153933	0.267296	0.569121	1.000
319	GOBP_NEGATIVE_REGULATION_OF_CYSTEINE_TYPE_ENDO...	GO:2000117	5	0.545750	1.153016	0.271772	0.569349	1.000
320	GOBP_TUBE_DEVELOPMENT	GO:0035295	18	0.372632	1.152265	0.294584	0.569122	1.000
321	GOBP_TRANSMEMBRANE_RECEPTOR_PROTEIN_SERINE_THR...	GO:0007178	6	0.503166	1.147195	0.294210	0.578417	1.000
322	GOBP_REGULATION_OF_ORGANELLE_ORGANIZATION	GO:0033043	14	0.401040	1.145773	0.302778	0.579823	1.000
323	GOBP_REGULATION_OF_VASCULAR_ASSOCIATED_SMOOTH_...	GO:1904705	5	0.548016	1.144649	0.284211	0.580348	1.000
324	GOBP_REGULATION_OF_LIPID_METABOLIC_PROCESS	GO:0019216	7	0.491135	1.144137	0.309838	0.579666	1.000
325	GOBP_DEVELOPMENTAL_CELL_GROWTH	GO:0048588	5	0.534123	1.143479	0.296703	0.579301	1.000
326	GOBP_POSITIVE_REGULATION_OF_NEUROGENESIS	GO:0050769	12	0.415343	1.141462	0.300135	0.581749	1.000
327	GOBP_POSITIVE_REGULATION_OF_TRANSPORT	GO:0051050	26	0.340548	1.139583	0.312339	0.584002	1.000
328	GOBP_POSITIVE_REGULATION_OF_NERVOUS_SYSTEM_DEV...	GO:0051962	12	0.415343	1.138847	0.310014	0.583898	1.000
329	GOBP_ORGANOPHOSPHATE_METABOLIC_PROCESS	GO:0019637	7	0.477045	1.135761	0.293605	0.588831	1.000
330	REACTOME_NEUTROPHIL_DEGRANULATION	R-RNO-6798695	6	0.519028	1.132963	0.316788	0.593051	1.000
331	GOBP_POSITIVE_REGULATION_OF_TRANSCRIPTION_BY_R...	GO:0045944	24	0.344203	1.128876	0.334180	0.600161	1.000
332	GOBP_POSITIVE_REGULATION_OF_CELL_DEVELOPMENT	GO:0010720	12	0.415343	1.128726	0.312329	0.598649	1.000
333	GOBP_LEUKOCYTE_CELL_CELL_ADHESION	GO:0007159	6	0.505302	1.128426	0.308908	0.597555	1.000
334	GOBP_REGULATION_OF_ENDOTHELIAL_CELL_MIGRATION	GO:0010594	5	0.538282	1.128186	0.310811	0.596252	1.000
335	GOBP_ENDOTHELIAL_CELL_MIGRATION	GO:0043542	8	0.453348	1.125762	0.318248	0.599534	1.000
336	GOBP_EPITHELIAL_CELL_PROLIFERATION	GO:0050673	12	0.397316	1.122931	0.318560	0.603601	1.000
337	GOBP_POSITIVE_REGULATION_OF_PRI_MIRNA_TRANSCRI...	GO:1902895	7	0.465476	1.122829	0.317578	0.602025	1.000
338	GOBP_ESTABLISHMENT_OF_PROTEIN_LOCALIZATION_TO_...	GO:0072594	6	0.506250	1.122642	0.330838	0.600648	1.000
339	GOBP_REGULATION_OF_NERVOUS_SYSTEM_DEVELOPMENT	GO:0051960	15	0.382404	1.122603	0.321990	0.598947	1.000
340	GOBP_LIPID_LOCALIZATION	GO:0010876	10	0.425351	1.122448	0.337589	0.597504	1.000
341	GOBP_ENDOTHELIAL_CELL_PROLIFERATION	GO:0001935	8	0.452199	1.122329	0.305233	0.596061	1.000

Continuation of Table **S16**

	NAME	ID	SIZE	ES	NES	NOM.p.val	FDR.q.val	FWER.p.val
342	GOBP_ACTIVATION_OF_IMMUNE_RESPONSE	GO:0002253	5	0.527031	1.119666	0.318797	0.599965	1.000
343	GOBP_RESPONSE_TO_PEPTIDE	GO:1901652	18	0.364172	1.114022	0.318003	0.610077	1.000
344	GOBP_CELLULAR_RESPONSE_TO_PEPTIDE	GO:1901653	13	0.388033	1.112072	0.346829	0.612526	1.000
345	GOBP_POSITIVE_REGULATION_OF_DNA_BINDING_TRANSC...	GO:0051091	10	0.431486	1.112050	0.351801	0.610813	1.000
346	GOBP_REGULATION_OF_BINDING	GO:0051098	11	0.407816	1.111845	0.338889	0.609479	1.000
347	GOBP_BLOOD_VESSEL_ENDOTHELIAL_CELL_MIGRATION	GO:0043534	8	0.453348	1.111832	0.334290	0.607756	1.000
348	GOBP_CHROMOSOME_ORGANIZATION	GO:0051276	6	0.498133	1.111392	0.323881	0.607034	1.000
349	GOBP_ENZYME_LINKED_RECEPTOR_PROTEIN_SIGNALING_...	GO:0007167	24	0.344563	1.111224	0.319588	0.605622	1.000
350	GOBP_POSITIVE_REGULATION_OF_EPITHELIAL_CELL_MI...	GO:0010634	5	0.522857	1.110860	0.347894	0.604663	1.000
351	GOBP_MUSCLE_ORGAN_DEVELOPMENT	GO:0007517	9	0.430380	1.107472	0.336606	0.610093	1.000
352	REACTOME_HEMOSTASIS	R-RNO-109582	14	0.381172	1.106388	0.326693	0.610564	1.000
353	GOBP_ERBB_SIGNALING_PATHWAY	GO:0038127	5	0.533063	1.106259	0.336350	0.609103	1.000
354	GOBP_NEGATIVE_REGULATION_OF_NERVOUS_SYSTEM_DEV...	GO:0051961	5	0.522341	1.103815	0.336907	0.612493	1.000
355	REACTOME_NGF_STIMULATED_TRANSCRIPTION	R-RNO-9031628	9	0.430380	1.103376	0.342291	0.611719	1.000
356	GOBP_EMBRYO_DEVELOPMENT_ENDING_IN_BIRTH_OR_EGG...	GO:0009792	9	0.431500	1.100904	0.354749	0.615180	1.000
357	GOBP_NEGATIVE_REGULATION_OF_LOCOMOTION	GO:0040013	5	0.518707	1.099563	0.356391	0.616141	1.000
358	GOBP_SMOOTH_MUSCLE_CELL_PROLIFERATION	GO:0048659	6	0.489239	1.098565	0.349558	0.616509	1.000
359	GOBP_PROTEOLYSIS	GO:0006508	17	0.357695	1.097549	0.343791	0.616739	1.000
360	GOBP_SIGNAL_RELEASE	GO:0023061	14	0.385764	1.097515	0.335655	0.615123	1.000
361	GOBP_RESPONSE_TO_RADIATION	GO:0009314	13	0.386176	1.095649	0.356383	0.617285	1.000
362	GOBP_MUSCLE_STRUCTURE_DEVELOPMENT	GO:0061061	16	0.367559	1.095438	0.341623	0.616122	1.000
363	GOBP_RESPONSE_TO_INORGANIC_SUBSTANCE	GO:0010035	19	0.348756	1.088134	0.337317	0.629541	1.000
364	GOBP_POSITIVE_REGULATION_OF_SIGNALING	GO:0023056	34	0.312732	1.087711	0.367470	0.628748	1.000
365	GOBP_POSITIVE_REGULATION_OF_CELLULAR_PROTEIN_L...	GO:1903829	7	0.466446	1.086685	0.396501	0.629095	1.000
366	GOBP_POSITIVE_REGULATION_OF_CATALYTIC_ACTIVITY	GO:0043085	30	0.313789	1.081676	0.357500	0.637711	1.000
367	GOBP_POSITIVE_REGULATION_OF_CELL_PROJECTION_OR...	GO:0031346	10	0.416232	1.079991	0.389830	0.639317	1.000
368	GOBP_RESPONSE_TO_LIGHT_STIMULUS	GO:0009416	11	0.400435	1.078758	0.349933	0.640086	1.000
369	GOBP_REGULATION_OF_TRANSMEMBRANE_TRANSPORT	GO:0034762	23	0.336479	1.077926	0.359335	0.640068	1.000
370	GOBP_PRI_MIRNA_TRANSCRIPTION_BY_RNA_POLYMERASE_II	GO:0061614	7	0.465476	1.077596	0.384844	0.638940	1.000
371	GOBP_EMBRYONIC_MORPHOGENESIS	GO:0048598	6	0.481537	1.077177	0.364329	0.638069	1.000
372	GOBP_MULTI_MULTICELLULAR_ORGANISM_PROCESS	GO:0044706	8	0.440811	1.073254	0.385475	0.644691	1.000
373	GOBP_LOCOMOTORY_BEHAVIOR	GO:0007626	7	0.445776	1.072978	0.389630	0.643446	1.000
374	GOBP_MONOCARBOXYLIC_ACID_METABOLIC_PROCESS	GO:0032787	6	0.475344	1.072290	0.370753	0.643122	1.000
375	GOBP_ACTIN_FILAMENT_BASED_PROCESS	GO:0030029	7	0.452026	1.064971	0.400000	0.656219	1.000
376	GOBP_RESPONSE_TO_UV	GO:0009411	5	0.508481	1.064011	0.373846	0.656435	1.000
377	GOBP_DENDRITIC_SPINE_DEVELOPMENT	GO:0060996	6	0.469681	1.062871	0.408511	0.657092	1.000
378	GOBP_LOCOMOTION	GO:0040011	31	0.314593	1.062725	0.387137	0.655611	1.000
379	GOBP_REGULATION_OF_RECEPTOR_MEDIATED_ENDOCYTOSIS	GO:0048259	5	0.499246	1.060962	0.396947	0.657474	1.000
380	GOBP_ENDOCRINE_SYSTEM_DEVELOPMENT	GO:0035270	6	0.462989	1.060483	0.402108	0.656745	1.000
381	GOBP_POSITIVE_REGULATION_OF_CELL_GROWTH	GO:0030307	6	0.473949	1.059006	0.389985	0.658255	1.000
382	GOBP_CYTOKINE_MEDIATED_SIGNALING_PATHWAY	GO:0019221	16	0.357563	1.058510	0.408488	0.657512	1.000
383	REACTOME_NERVOUS_SYSTEM_DEVELOPMENT	R-RNO-9675108	15	0.366370	1.058065	0.400804	0.656659	1.000
384	GOBP_DENDRITIC_SPINE_MORPHOGENESIS	GO:0060997	5	0.504773	1.055511	0.407108	0.659997	1.000

Continuation of Table **S16**

	NAME	ID	SIZE	ES	NES	NOM.p.val	FDR.q.val	FWER.p.val
385	GOBP_REGULATION_OF_CELLULAR_PROTEIN_LOCALIZATION	GO:1903827	12	0.386367	1.054114	0.424791	0.661222	1.000
386	REACTOME_MAPK_FAMILY_SIGNALING_CASCADES	R-RNO-5683057	12	0.376086	1.053462	0.385773	0.660904	1.000
387	GOBP_NEGATIVE_REGULATION_OF_ION_TRANSPORT	GO:0043271	11	0.394235	1.051641	0.395512	0.662787	1.000
388	REACTOME_BETA_CATENIN_INDEPENDENT_WNT_SIGNALING	R-RNO-3858494	5	0.487389	1.050013	0.387334	0.664455	1.000
389	GOBP_LEUKOCYTE_DIFFERENTIATION	GO:0002521	15	0.358574	1.049430	0.400273	0.664020	1.000
390	GOBP_REGULATION_OF_CYTOSKELETON_ORGANIZATION	GO:0051493	5	0.492740	1.048578	0.411326	0.663915	1.000
391	GOBP_STRIATED_MUSCLE_CELL_DIFFERENTIATION	GO:0051146	9	0.410926	1.047798	0.422190	0.663912	1.000
392	GOBP_REGULATION_OF_INFLAMMATORY_RESPONSE	GO:0050727	7	0.443346	1.047142	0.394074	0.663428	1.000
393	GOBP_REGULATION_OF_RESPONSE_TO_STRESS	GO:0080134	19	0.341173	1.047023	0.410923	0.662021	1.000
394	GOBP_ANATOMICAL_STRUCTURE_HOMEOSTASIS	GO:0060249	7	0.451283	1.045383	0.433681	0.663678	1.000
395	GOBP_MATERNAL_PROCESS_INVOLVED_IN_FEMALE_PREGN...	GO:0060135	5	0.504715	1.045235	0.424806	0.662300	1.000
396	GOBP_MYELOID_CELL_DIFFERENTIATION	GO:0030099	13	0.366349	1.043979	0.401628	0.663160	1.000
397	GOBP_OSTEOBLAST_DIFFERENTIATION	GO:0001649	5	0.498963	1.042697	0.416413	0.664087	1.000
398	GOBP_RAS_PROTEIN_SIGNAL_TRANSDUCTION	GO:0007265	5	0.492870	1.042666	0.402496	0.662497	1.000
399	GOBP_PLATELET_ACTIVATION	GO:0030168	6	0.468289	1.041768	0.430250	0.662638	1.000
400	GOBP_CELL_CELL_JUNCTION_ASSEMBLY	GO:0007043	5	0.488509	1.040883	0.429217	0.662671	1.000
401	GOBP_TRANSMEMBRANE_RECEPTOR_PROTEIN_TYROSINE_K...	GO:0007169	19	0.332104	1.037489	0.404066	0.667992	1.000
402	GOBP_LYMPHOCYTE_ACTIVATION	GO:0046649	9	0.401397	1.037332	0.430727	0.666633	1.000
403	GOBP_PROTEIN_AUTOPHOSPHORYLATION	GO:0046777	5	0.496904	1.037102	0.427300	0.665426	1.000
404	GOBP_REGULATION_OF_DEFENSE_RESPONSE	GO:0031347	7	0.443346	1.034927	0.429608	0.668034	1.000
405	REACTOME_EXTRA_NUCLEAR_ESTROGEN_SIGNALING	R-RNO-9009391	7	0.441011	1.033165	0.437500	0.669833	1.000
406	GOBP_CELLULAR_RESPONSE_TO_NITROGEN_COMPOUND	GO:1901699	20	0.331766	1.030252	0.439024	0.674103	1.000
407	GOBP_HOMEOSTATIC_PROCESS	GO:0042592	31	0.304893	1.029128	0.442260	0.674585	1.000
408	REACTOME_SIGNALING_BY_NTRKS	R-RNO-166520	18	0.331083	1.027240	0.450382	0.676653	1.000
409	REACTOME_MAPK_TARGETS_NUCLEAR_EVENTS_MEDIATED_...	R-HSA-450282	5	0.494016	1.023955	0.441221	0.681471	1.000
410	GOBP_REGULATION_OF_NERVOUS_SYSTEM_PROCESS	GO:0031644	8	0.410960	1.021646	0.436496	0.684285	1.000
411	REACTOME_INTERLEUKIN_4_AND_INTERLEUKIN_13_SIGN...	R-RNO-6785807	8	0.416372	1.020253	0.439706	0.685488	1.000
412	GOBP_RESPONSE_TO_ORGANIC_CYCLIC_COMPOUND	GO:0014070	20	0.325243	1.016115	0.443734	0.692034	1.000
413	REACTOME_INTRACELLULAR_SIGNALING_BY_SECOND_MES...	R-RNO-9006925	15	0.344252	1.015788	0.453939	0.690962	1.000
414	GOBP_BLOOD_VESSEL_MORPHOGENESIS	GO:0048514	14	0.352240	1.014332	0.451436	0.692131	1.000
415	GOBP_CELLULAR_RESPONSE_TO_REACTIVE_OXYGEN_SPECIES	GO:0034614	7	0.426616	1.011869	0.478261	0.695409	1.000
416	GOBP_PROTEIN_LOCALIZATION_TO_MEMBRANE	GO:0072657	14	0.351339	1.008080	0.469003	0.701420	1.000
417	GOBP_NEGATIVE_REGULATION_OF_APOPTOTIC_SIGNALIN...	GO:2001234	7	0.427422	1.007577	0.456973	0.700713	1.000
418	GOBP_TISSUE_HOMEOSTASIS	GO:0001894	5	0.464039	1.004884	0.465544	0.704206	1.000
419	GOBP_REGULATION_OF_PEPTIDASE_ACTIVITY	GO:0052547	10	0.384852	1.004185	0.470027	0.703931	1.000
420	GOBP_POSITIVE_REGULATION_OF_EPITHELIAL_CELL_PR...	GO:0050679	7	0.427957	1.003570	0.466476	0.703414	1.000
421	REACTOME_CELLULAR_RESPONSES_TO_EXTERNAL_STIMULI	R-RNO-8953897	12	0.356991	1.001096	0.455540	0.706588	1.000
422	REACTOME_RAS_ACTIVATION_UPON_CA2_INFLUX_THROUG...	R-HSA-442982	6	0.443347	0.998714	0.474926	0.709540	1.000
423	GOBP_CELLULAR_RESPONSE_TO_INORGANIC_SUBSTANCE	GO:0071241	11	0.367213	0.997991	0.471467	0.709387	1.000
424	GOBP_REPRODUCTIVE_SYSTEM_DEVELOPMENT	GO:0061458	6	0.449761	0.995980	0.511799	0.711593	1.000
425	GOBP_ORGANIC_ACID_METABOLIC_PROCESS	GO:0006082	7	0.422551	0.992550	0.482759	0.716673	1.000
426	GOBP_NEURON_PROJECTION_ORGANIZATION	GO:0106027	8	0.401315	0.992347	0.494876	0.715365	1.000
427	GOBP_REGULATION_OF_FAT_CELL_DIFFERENTIATION	GO:0045598	6	0.444888	0.992321	0.477134	0.713743	1.000

Continuation of Table **S16**

	NAME	ID	SIZE	ES	NES	NOM.p.val	FDR.q.val	FWER.p.val
428	REACTOME_SIGNALING_BY_WNT	R-RNO-195721	7	0.422309	0.991450	0.485163	0.713639	1.000
429	GOBP_POSITIVE_REGULATION_OF_CELL_DEATH	GO:0010942	19	0.323513	0.991026	0.487646	0.712826	1.000
430	GOBP_INFLAMMATORY_RESPONSE	GO:0006954	12	0.353663	0.988831	0.488340	0.715356	1.000
431	GOBP_CELLULAR_RESPONSE_TO_CHEMICAL_STRESS	GO:0062197	7	0.426616	0.988541	0.496296	0.714223	1.000
432	GOBP_REGULATION_OF_SYNAPTIC_TRANSMISSION_GLUTA...	GO:0051966	12	0.360982	0.986172	0.484006	0.716996	1.000
433	GOBP_POSITIVE_REGULATION_OF_ION_TRANSPORT	GO:0043270	17	0.332098	0.984433	0.498694	0.718805	1.000
434	GOBP_POSITIVE_REGULATION_OF_CYTOKINE_PRODUCTION	GO:0001819	7	0.409537	0.983649	0.488987	0.718721	1.000
435	GOBP_REGULATION_OF_PROTEOLYSIS	GO:0030162	12	0.350844	0.980225	0.504155	0.723611	1.000
436	GOBP_CARBOHYDRATE_DERIVATIVE_METABOLIC_PROCESS	GO:1901135	7	0.411593	0.979137	0.505344	0.724137	1.000
437	GOBP_REGULATION_OF_CELL_PROJECTION_ORGANIZATION	GO:0031344	20	0.311545	0.977057	0.493438	0.726396	1.000
438	GOBP_CENTRAL_NERVOUS_SYSTEM_DEVELOPMENT	GO:0007417	26	0.298163	0.973574	0.508035	0.731471	1.000
439	GOBP_PROTEIN_CATABOLIC_PROCESS	GO:0030163	8	0.400432	0.973231	0.510294	0.730471	1.000
440	REACTOME_ESTROGEN_DEPENDENT_NUCLEAR_EVENTS_DOW...	R-RNO-9634638	5	0.464124	0.970563	0.515432	0.733706	1.000
441	REACTOME_L1CAM_INTERACTIONS	R-RNO-373760	5	0.459885	0.968841	0.520129	0.735343	1.000
442	GOBP_REGULATION_OF_CELL_CELL_ADHESION	GO:0022407	9	0.376863	0.965184	0.521739	0.740525	1.000
443	GOBP_SPROUTING_ANGIOGENESIS	GO:0002040	5	0.445783	0.964818	0.526237	0.739547	1.000
444	GOBP_REGULATION_OF_ION_TRANSPORT	GO:0043269	36	0.281308	0.962658	0.504717	0.741861	1.000
445	HALLMARK_KRAS_SIGNALING_UP	M5953	6	0.427254	0.962357	0.511144	0.740738	1.000
446	GOBP_FOREBRAIN_DEVELOPMENT	GO:0030900	9	0.368201	0.959535	0.523605	0.744157	1.000
447	GOBP_RESPONSE_TO_HORMONE	GO:0009725	20	0.303155	0.958693	0.498094	0.744002	1.000
448	REACTOME_ESR_MEDIATED_SIGNALING	R-RNO-8939211	8	0.390477	0.952048	0.552941	0.754856	1.000
449	GOBP_RESPONSE_TO_MOLECULE_OF_BACTERIAL_ORIGIN	GO:0002237	13	0.341481	0.948940	0.533512	0.759038	1.000
450	GOBP_REGULATION_OF_SYNAPTIC_PLASTICITY	GO:0048167	21	0.301222	0.948194	0.530612	0.758826	1.000
451	GOBP_REGULATION_OF_NEURON_PROJECTION_DEVELOPMENT	GO:0010975	16	0.321198	0.947812	0.539267	0.757873	1.000
452	GOBP_NEGATIVE_REGULATION_OF_CELL_POPULATION_PR...	GO:0008285	9	0.375509	0.946639	0.538889	0.758281	1.000
453	GOBP_NEGATIVE_REGULATION_OF_TRANSMEMBRANE_TRAN...	GO:0034763	6	0.425084	0.945885	0.547511	0.757964	1.000
454	GOBP_RESPONSE_TO_BACTERIUM	GO:0009617	13	0.341481	0.943250	0.522161	0.761138	1.000
455	GOBP_TRANSMEMBRANE_TRANSPORT	GO:0055085	27	0.281659	0.939829	0.551151	0.766197	1.000
456	REACTOME_PTEN_REGULATION	R-RNO-6807070	5	0.438356	0.939664	0.543344	0.764852	1.000
457	REACTOME_SIGNALING_BY_NUCLEAR_RECEPTORS	R-RNO-9006931	8	0.390477	0.939314	0.551429	0.763744	1.000
458	GOBP_REGULATION_OF_NEURONAL_SYNAPTIC_PLASTICITY	GO:0048168	7	0.397562	0.934431	0.579942	0.770800	1.000
459	GOBP_REGULATION_OF_CELL_DEATH	GO:0010941	34	0.269086	0.934338	0.558458	0.769342	1.000
460	GOBP_POSITIVE_REGULATION_OF_ENDOTHELIAL_CELL_P...	GO:0001938	6	0.415427	0.932238	0.566717	0.771359	1.000
461	GOBP_POSITIVE_REGULATION_OF_CELLULAR_COMPONENT...	GO:0044089	9	0.358511	0.929561	0.546610	0.774723	1.000
462	REACTOME_CA_DEPENDENT_EVENTS	R-RNO-111996	8	0.376383	0.927259	0.554412	0.777243	1.000
463	GOBP_PROTEIN_MODIFICATION_BY_SMALL_PROTEIN_CON...	GO:0070647	7	0.382716	0.926243	0.548246	0.777492	1.000
464	REACTOME_CLASS_C_3_METABOTROPIC_GLUTAMATE_PHER...	R-RNO-420499	7	0.391143	0.926022	0.575284	0.776209	1.000
465	GOBP_SYNAPSE_ORGANIZATION	GO:0050808	17	0.304132	0.922526	0.578947	0.781022	1.000
466	GOBP_CHEMICAL_HOMEOSTASIS	GO:0048878	23	0.287481	0.921428	0.563975	0.781299	1.000
467	GOBP_PROTEIN_LOCALIZATION_TO_SYNAPSE	GO:0035418	5	0.435596	0.921200	0.574400	0.780051	1.000
468	GOBP_CELL_CELL_SIGNALING	GO:0007267	56	0.267935	0.917385	0.605882	0.785098	1.000
469	GOBP_REGULATION_OF_ANATOMICAL_STRUCTURE_SIZE	GO:0090066	10	0.351096	0.916205	0.583215	0.785377	1.000
470	REACTOME_INFECTIOUS_DISEASE	R-HSA-5663205	13	0.322038	0.911119	0.572238	0.792527	1.000

Continuation of Table **S16**

	NAME	ID	SIZE	ES	NES	NOM.p.val	FDR.q.val	FWER.p.val
471	GOBP_ORGANONITROGEN_COMPOUND_CATABOLIC_PROCESS	GO:1901565	9	0.352892	0.909317	0.589563	0.794082	1.000
472	GOBP_ORGANIC_HYDROXY_COMPOUND_METABOLIC_PROCESS	GO:1901615	7	0.378620	0.906487	0.587706	0.797436	1.000
473	GOBP_RESPONSE_TO_NITROGEN_COMPOUND	GO:1901698	34	0.264833	0.906443	0.589461	0.795827	1.000
474	GOBP_PROTEIN_LOCALIZATION_TO_POSTSYNAPSE	GO:0062237	5	0.435596	0.903073	0.608696	0.800167	1.000
475	GOBP_LEARNING	GO:0007612	13	0.323243	0.902751	0.576407	0.799033	1.000
476	GOBP_NEURON_MIGRATION	GO:0001764	5	0.426890	0.898373	0.625373	0.805023	1.000
477	GOBP_POSTSYNAPSE_ORGANIZATION	GO:0099173	10	0.341655	0.896550	0.594142	0.806755	1.000
478	GOBP_CELLULAR_RESPONSE_TO_HORMONE_STIMULUS	GO:0032870	11	0.328024	0.895276	0.623580	0.807270	1.000
479	GOBP_CELL_JUNCTION_ORGANIZATION	GO:0034330	19	0.285241	0.894956	0.600519	0.806116	1.000
480	GOBP_SMALL_GTPASE_MEDIATED_SIGNAL_TRANSDUCTION	GO:0007264	8	0.362990	0.892702	0.610719	0.808371	1.000
481	GOBP_PROTEIN_LOCALIZATION_TO_CELL_PERIPHERY	GO:1990778	12	0.325203	0.889001	0.603001	0.813071	1.000
482	GOBP_ENDOMEMBRANE_SYSTEM_ORGANIZATION	GO:0010256	6	0.386855	0.886390	0.630792	0.815749	1.000
483	GOBP_NEGATIVE_REGULATION_OF_CELL_PROJECTION_OR...	GO:0031345	5	0.422335	0.885512	0.632504	0.815587	1.000
484	GOBP_REGULATION_OF_CYSTEINE_TYPE_ENDOPEPTIDASE...	GO:2000116	9	0.341495	0.885374	0.619392	0.814149	1.000
485	GOBP_POSITIVE_REGULATION_OF_BINDING	GO:0051099	6	0.396872	0.881922	0.632308	0.818334	1.000
486	REACTOME_GPCR_LIGAND_BINDING	R-RNO-500792	8	0.356439	0.881907	0.615385	0.816674	1.000
487	GOBP_RESPONSE_TO_ACID_CHEMICAL	GO:0001101	6	0.397203	0.881807	0.626973	0.815166	1.000
488	GOBP_POSITIVE_REGULATION_OF_AXONOGENESIS	GO:0050772	5	0.420261	0.880661	0.658462	0.815480	1.000
489	GOBP_SKELETAL_MUSCLE_ORGAN_DEVELOPMENT	GO:0060538	5	0.409639	0.879974	0.625564	0.815026	1.000
490	REACTOME_G_PROTEIN_MEDIATED_EVENTS	R-RNO-112040	9	0.346721	0.879284	0.627424	0.814445	1.000
491	GOBP_CELLULAR_RESPONSE_TO_CADMIUM_ION	GO:0071276	5	0.416524	0.876469	0.640000	0.817565	1.000
492	GOBP_GLAND_DEVELOPMENT	GO:0048732	7	0.370188	0.876077	0.654572	0.816530	1.000
493	GOBP_REGULATION_OF_CELLULAR_LOCALIZATION	GO:0060341	17	0.294597	0.874694	0.649551	0.817175	1.000
494	GOBP_T_CELL_ACTIVATION	GO:0042110	8	0.361342	0.874094	0.625869	0.816503	1.000
495	REACTOME_DEVELOPMENTAL_BIOLOGY	R-RNO-1266738	23	0.270858	0.870693	0.650773	0.820653	1.000
496	GOBP_CELLULAR_COMPONENT_MAINTENANCE	GO:0043954	5	0.416096	0.869140	0.650078	0.821553	1.000
497	REACTOME_EPH_EPHRIN_SIGNALING	R-RNO-2682334	6	0.381367	0.866242	0.660633	0.824697	1.000
498	GOBP_HOMEOSTASIS_OF_NUMBER_OF_CELLS	GO:0048872	5	0.408684	0.862973	0.630468	0.828557	1.000
499	GOBP_RESPONSE_TO_CADMIUM_ION	GO:0046686	6	0.382837	0.861826	0.665726	0.828908	1.000
500	GOBP_PROTEIN_LOCALIZATION_TO_CELL_JUNCTION	GO:1902414	6	0.386283	0.861748	0.647416	0.827358	1.000
501	REACTOME_EXTRACELLULAR_MATRIX_ORGANIZATION	R-RNO-1474244	5	0.402740	0.859492	0.673252	0.829330	1.000
502	GOBP_G_PROTEIN_COUPLED_GLUTAMATE_RECEPTOR_SIGN...	GO:0007216	8	0.348932	0.856184	0.655882	0.833119	1.000
503	GOBP_PROTEIN_MODIFICATION_BY_SMALL_PROTEIN_CON...	GO:0032446	6	0.378049	0.851239	0.665685	0.839549	1.000
504	GOBP_CELLULAR_RESPONSE_TO_PEPTIDE_HORMONE_STIM...	GO:0071375	9	0.328738	0.850864	0.666207	0.838610	1.000
505	GOBP_TELENCEPHALON_DEVELOPMENT	GO:0021537	6	0.377617	0.848078	0.676602	0.841544	1.000
506	GOBP_EMBRYONIC_ORGAN_DEVELOPMENT	GO:0048568	7	0.364950	0.846729	0.688478	0.842041	1.000
507	GOBP_REGULATION_OF_CELLULAR_COMPONENT_SIZE	GO:0032535	7	0.354772	0.845475	0.672439	0.842496	1.000
508	REACTOME_LEISHMANIA_INFECTION	R-HSA-9658195	10	0.316437	0.844331	0.652463	0.842573	1.000
509	GOBP_REGULATION_OF_PROTEIN_TYROSINE_KINASE_ACT...	GO:0061097	5	0.399069	0.841215	0.693098	0.845928	1.000
510	GOBP_RESPONSE_TO_CALCIUM_ION	GO:0051592	6	0.376664	0.840017	0.685714	0.846329	1.000
511	GOBP_POSITIVE_REGULATION_OF_MYELOID_CELL_DIFFE...	GO:0045639	7	0.352811	0.838458	0.693908	0.847360	1.000
512	GOBP_RESPONSE_TO_INSULIN	GO:0032868	5	0.397590	0.835759	0.712782	0.850035	1.000
513	GOBP_POSITIVE_REGULATION_OF_PROTEIN_TYROSINE_K...	GO:0061098	5	0.399069	0.833736	0.718110	0.851572	1.000

Continuation of Table **S16**

	NAME	ID	SIZE	ES	NES	NOM.p.val	FDR.q.val	FWER.p.val
514	GOBP_PURINE_CONTAINING_COMPOUND_METABOLIC_PROCESS	GO:0072521	5	0.392307	0.832345	0.722741	0.852079	1.000
515	GOBP_POSITIVE_REGULATION_OF_SECRETION	GO:0051047	6	0.372137	0.830466	0.696319	0.853451	1.000
516	GOBP_MYELOID_LEUKOCYTE_DIFFERENTIATION	GO:0002573	11	0.304565	0.829793	0.687943	0.852964	1.000
517	GOBP_INTRINSIC_APOPTOTIC_SIGNALING_PATHWAY	GO:0097193	5	0.387377	0.829400	0.694228	0.851870	1.000
518	GOBP_CELL_PROJECTION_ORGANIZATION	GO:0030030	34	0.238263	0.828379	0.708892	0.851821	1.000
519	GOBP_RECEPTOR_MEDIATED_ENDOCYTOSIS	GO:0006898	6	0.365151	0.828134	0.713056	0.850616	1.000
520	GOBP_CELL_MORPHOGENESIS_INVOLVED_IN_NEURON_DIF...	GO:0048667	21	0.258517	0.827948	0.716837	0.849258	1.000
521	GOBP_RESPONSE_TO_PEPTIDE_HORMONE	GO:0043434	13	0.293289	0.825155	0.712912	0.852002	1.000
522	GOBP_CELL_MORPHOGENESIS_INVOLVED_IN_DIFFERENTI...	GO:0000904	21	0.258517	0.824600	0.692695	0.851306	1.000
523	HALLMARK_APOPTOSIS	M5902	6	0.361849	0.817405	0.694084	0.860792	1.000
524	GOBP_POSITIVE_REGULATION_OF_CYSTEINE_TYPE_ENDO...	GO:2001056	7	0.344234	0.816348	0.715152	0.860803	1.000
525	GOBP_POSITIVE_REGULATION_OF_NEURON_DEATH	GO:1901216	5	0.389248	0.815837	0.718894	0.859933	1.000
526	GOBP_PEPTIDYL_SERINE_MODIFICATION	GO:0018209	12	0.293566	0.814723	0.707483	0.859935	1.000
527	GOBP_POSITIVE_REGULATION_OF_PEPTIDASE_ACTIVITY	GO:0010952	7	0.344234	0.814641	0.705185	0.858420	1.000
528	GOBP_APOPTOTIC_MITOCHONDRIAL_CHANGES	GO:0008637	6	0.361234	0.814500	0.714495	0.857025	1.000
529	GOBP_MULTICELLULAR_ORGANISMAL_RESPONSE_TO_STRESS	GO:0033555	5	0.380015	0.813928	0.746154	0.856258	1.000
530	GOBP_REGULATION_OF_HYDROLASE_ACTIVITY	GO:0051336	22	0.254758	0.811522	0.713342	0.858315	1.000
531	REACTOME_CELLULAR_SENESCENCE	R-RNO-2559583	6	0.358464	0.810048	0.743161	0.858980	1.000
532	GOBP_SYNAPTIC_SIGNALING	GO:0099536	47	0.227103	0.808653	0.742690	0.859521	1.000
533	REACTOME_SENESCENCE_ASSOCIATED_SECRETORY_PHENO...	R-RNO-2559582	6	0.358464	0.807668	0.704918	0.859329	1.000
534	GOBP_POSITIVE_REGULATION_OF_SYNAPTIC_TRANSMISSION	GO:0050806	15	0.278566	0.805956	0.732713	0.860339	1.000
535	GOBP_COGNITION	GO:0050890	28	0.239326	0.804769	0.720497	0.860569	1.000
536	GOBP_NEGATIVE_REGULATION_OF_IMMUNE_SYSTEM_PROCESS	GO:0002683	5	0.373534	0.804194	0.739968	0.859799	1.000
537	GOBP_REGULATION_OF_LYMPHOCYTE_ACTIVATION	GO:0051249	5	0.386456	0.802546	0.753776	0.860670	1.000
538	GOBP_RESPONSE_TO_ENDOGENOUS_STIMULUS	GO:0009719	34	0.234127	0.800723	0.734055	0.861736	1.000
539	GOBP_RESPONSE_TO_OXYGEN_LEVELS	GO:0070482	8	0.320802	0.800698	0.733612	0.860177	1.000
540	GOBP_MUSCLE_CONTRACTION	GO:0006936	5	0.378086	0.794110	0.757079	0.868107	1.000
541	GOBP_CHEMICAL_SYNAPTIC_TRANSMISSION_POSTSYNAPTIC	GO:0099565	9	0.308284	0.793965	0.733894	0.866716	1.000
542	GOBP_POSITIVE_REGULATION_OF_DEFENSE_RESPONSE	GO:0031349	5	0.374790	0.792777	0.753870	0.866856	1.000
543	GOBP_RECEPTOR_INTERNALIZATION	GO:0031623	5	0.368751	0.785952	0.783489	0.874925	1.000
544	GOBP_RECEPTOR_METABOLIC_PROCESS	GO:0043112	5	0.368751	0.779191	0.792109	0.883164	1.000
545	GOBP_REGULATION_OF_CELLULAR_COMPONENT_BIOGENESIS	GO:0044087	10	0.294901	0.778720	0.742075	0.882233	1.000
546	GOBP_POSITIVE_REGULATION_OF_TRANSMEMBRANE_TRAN...	GO:0034764	7	0.331291	0.776100	0.764368	0.884304	1.000
547	REACTOME_INTEGRATION_OF_ENERGY_METABOLISM	R-RNO-163685	5	0.355879	0.775341	0.782071	0.883763	1.000
548	GOBP_REGULATION_OF_PROTEIN_BINDING	GO:0043393	7	0.328518	0.772513	0.752907	0.885987	1.000
549	GOBP_REGULATION_OF_TRANS_SYNAPTIC_SIGNALING	GO:0099177	40	0.223399	0.765029	0.798788	0.894640	1.000
550	GOBP_RESPONSE_TO_GROWTH_FACTOR	GO:0070848	18	0.250419	0.764327	0.782956	0.893940	1.000
551	GOBP_REGULATION_OF_PROTEIN_CATABOLIC_PROCESS	GO:0042176	7	0.320324	0.759465	0.765832	0.898977	1.000
552	GOBP_HEAD_DEVELOPMENT	GO:0060322	22	0.236883	0.751412	0.816195	0.908441	1.000
553	GOBP_LONG_TERM_SYNAPTIC_DEPRESSION	GO:0060292	5	0.352038	0.748006	0.819165	0.911247	1.000
554	GOBP_CELLULAR_ION_HOMEOSTASIS	GO:0006873	14	0.263148	0.746604	0.794078	0.911592	1.000
555	GOBP_MITOCHONDRION_ORGANIZATION	GO:0007005	7	0.310399	0.742492	0.806967	0.915269	1.000
556	GOBP_NEURON_DEVELOPMENT	GO:0048666	33	0.209590	0.735766	0.809002	0.922335	1.000

Continuation of Table **S16**

	NAME	ID	SIZE	ES	NES	NOM.p.val	FDR.q.val	FWER.p.val
557	GOBP_NEURON_DIFFERENTIATION	GO:0030182	36	0.213715	0.726414	0.828916	0.932489	1.000
558	GOBP_COAGULATION	GO:0050817	8	0.297789	0.725442	0.826761	0.932029	1.000
559	GOBP_EPITHELIAL_CELL_DIFFERENTIATION	GO:0030855	7	0.311233	0.725118	0.821429	0.930727	1.000
560	GOBP_REGULATION_OF_POSTSYNAPSE_ORGANIZATION	GO:0099175	6	0.326741	0.723270	0.824806	0.931485	1.000
561	GOBP_POSITIVE_REGULATION_OF_PROTEOLYSIS	GO:0045862	8	0.291015	0.722199	0.820550	0.931140	1.000
562	GOBP_REGULATION_OF_GTPASE_ACTIVITY	GO:0043087	5	0.342363	0.718798	0.855118	0.933698	1.000
563	GOBP_RESPONSE_TO_NUTRIENT	GO:0007584	5	0.335713	0.713776	0.852146	0.938057	1.000
564	GOBP_NEUROGENESIS	GO:0022008	40	0.201882	0.713425	0.837545	0.936812	1.000
565	REACTOME_TRANSCRIPTIONAL_REGULATION_BY_TP53	R-RNO-3700989	6	0.321822	0.713096	0.847458	0.935600	1.000
566	GOBP_POSITIVE_REGULATION_OF_MYELOID_LEUKOCYTE_...	GO:0002763	6	0.314213	0.708836	0.858824	0.939093	1.000
567	GOBP_ORGANIC_HYDROXY_COMPOUND_TRANSPORT	GO:0015850	6	0.315467	0.707041	0.826023	0.939513	1.000
568	GOBP_NEGATIVE_REGULATION_OF_CATABOLIC_PROCESS	GO:0009895	8	0.281105	0.703266	0.834734	0.942368	1.000
569	HALLMARK_HYPOXIA	M5891	5	0.332456	0.702769	0.839637	0.941340	1.000
570	REACTOME_TRANSCRIPTIONAL_REGULATION_OF_WHITE_A...	R-RNO-381340	6	0.313055	0.702469	0.846847	0.940026	1.000
571	GOBP_REGULATION_OF_CELL_ACTIVATION	GO:0050865	8	0.278881	0.692941	0.861386	0.949342	1.000
572	GOBP_NERVOUS_SYSTEM_PROCESS	GO:0050877	38	0.201346	0.690816	0.854897	0.949999	1.000
573	GOBP_MEMBRANE_ORGANIZATION	GO:0061024	12	0.247880	0.682772	0.862797	0.957545	1.000
574	GOBP_RESPONSE_TO_OXIDATIVE_STRESS	GO:0006979	8	0.274812	0.682431	0.865248	0.956217	1.000
575	GOBP_RESPONSE_TO_REACTIVE_OXYGEN_SPECIES	GO:0000302	8	0.274812	0.680847	0.856330	0.956232	1.000
576	GOBP_NEGATIVE_REGULATION_OF_SYNAPTIC_TRANSMISSION	GO:0050805	6	0.302797	0.677124	0.895833	0.958410	1.000
577	GOBP_RESPONSE_TO_MONOSACCHARIDE	GO:0034284	5	0.319893	0.677009	0.874433	0.956879	1.000
578	GOBP_POSITIVE_REGULATION_OF_HYDROLASE_ACTIVITY	GO:0051345	16	0.227822	0.676636	0.878113	0.955644	1.000
579	GOBP_REGULATION_OF_AXONOGENESIS	GO:0050770	7	0.288068	0.676066	0.868644	0.954620	1.000
580	GOBP_G_PROTEIN_COUPLED_RECEPTOR_SIGNALING_PATHWAY	GO:0007186	18	0.223313	0.676056	0.878828	0.952994	1.000
581	GOBP_MONONUCLEAR_CELL_DIFFERENTIATION	GO:1903131	7	0.291129	0.674017	0.887240	0.953485	1.000
582	GOBP_RESPONSE_TO_CARBOHYDRATE	GO:0009743	5	0.319893	0.671858	0.909375	0.954195	1.000
583	GOBP_ION_HOMEOSTASIS	GO:0050801	16	0.226014	0.667336	0.878378	0.957281	1.000
584	GOBP_CELL_PART_MORPHOGENESIS	GO:0032990	24	0.204069	0.664174	0.905897	0.958852	1.000
585	GOBP_CELLULAR_COMPONENT_MORPHOGENESIS	GO:0032989	24	0.204069	0.663606	0.894147	0.957788	1.000
586	GOBP_CELL_MORPHOGENESIS	GO:0000902	24	0.204069	0.659993	0.902378	0.959807	1.000
587	GOBP_MULTICELLULAR_ORGANISMAL_HOMEOSTASIS	GO:0048871	12	0.240227	0.659317	0.887671	0.958837	1.000
588	GOBP_REGULATION_OF_SYNAPSE_STRUCTURE_OR_ACTIVITY	GO:0050803	11	0.248887	0.657875	0.894366	0.958703	1.000
589	GOBP_CELL_JUNCTION_ASSEMBLY	GO:0034329	8	0.266507	0.653921	0.901288	0.960963	1.000
590	GOBP_POSITIVE_REGULATION_OF_HEMOPOIESIS	GO:1903708	7	0.275255	0.652232	0.904192	0.960964	1.000
591	GOBP_REGULATION_OF_PHOSPHOLIPASE_ACTIVITY	GO:0010517	6	0.284849	0.640894	0.917647	0.969926	1.000
592	GOBP_CHROMATIN_ORGANIZATION	GO:0006325	5	0.299886	0.638827	0.929003	0.970120	1.000
593	GOBP_TEMPERATURE_HOMEOSTASIS	GO:0001659	5	0.303063	0.638534	0.918836	0.968740	1.000
594	GOBP_REGULATION_OF_BODY_FLUID_LEVELS	GO:0050878	11	0.231723	0.637373	0.888738	0.968126	1.000
595	GOBP_REGULATION_OF_LIPASE_ACTIVITY	GO:0060191	6	0.284849	0.635814	0.910470	0.967887	1.000
596	GOBP_DEFENSE_RESPONSE	GO:0006952	19	0.198859	0.628519	0.922380	0.972454	1.000
597	GOBP_TAXIS	GO:0042330	14	0.217005	0.620908	0.910714	0.977097	1.000
598	GOBP_REGULATION_OF_MYELOID_CELL_DIFFERENTIATION	GO:0045637	9	0.241323	0.620662	0.907143	0.975657	1.000
599	GOBP_LONG_TERM_SYNAPTIC_POTENTIATION	GO:0060291	12	0.220013	0.619716	0.923611	0.974821	1.000

Continuation of Table **S16**

	NAME	ID	SIZE	ES	NES	NOM.p.val	FDR.q.val	FWER.p.val
600	REACTOME_NEUREXINS_AND_NEUROLIGINS	R-RNO-6794361	9	0.242981	0.616868	0.926491	0.975482	1.000
601	GOBP_IMPORT_INTO_CELL	GO:0098657	6	0.270001	0.614051	0.916176	0.976052	1.000
602	GOBP_PROTEIN_LOCALIZATION_TO_PLASMA_MEMBRANE	GO:0072659	7	0.264032	0.612996	0.918759	0.975287	1.000
603	GOBP_BEHAVIOR	GO:0007610	32	0.180228	0.609970	0.945679	0.975952	1.000
604	GOBP_REGULATION_OF_MYELOID_LEUKOCYTE_DIFFERENT...	GO:0002761	8	0.242378	0.604517	0.917012	0.978470	1.000
605	GOBP_RESPONSE_TO_BIOTIC_STIMULUS	GO:0009607	17	0.197730	0.603226	0.946738	0.977794	1.000
606	REACTOME_LONG_TERM_POTENTIATION	R-HSA-9620244	10	0.225161	0.594295	0.945302	0.982453	1.000
607	GOBP_ASSOCIATIVE_LEARNING	GO:0008306	7	0.255620	0.592860	0.935484	0.981834	1.000
608	GOBP_METAL_ION_TRANSPORT	GO:0030001	11	0.215412	0.587824	0.935441	0.983504	1.000
609	GOBP_LIPID_EXPORT_FROM_CELL	GO:0140353	5	0.275638	0.584391	0.945312	0.984160	1.000
610	HALLMARK_EPITHELIAL_MESENCHYMAL_TRANSITION	M5930	5	0.268031	0.583003	0.952160	0.983478	1.000
611	GOBP_CALCIUM_ION_TRANSPORT	GO:0006816	11	0.215412	0.577884	0.941909	0.985009	1.000
612	REACTOME_RNA_POLYMERASE_II_TRANSCRIPTION	R-RNO-73857	20	0.176889	0.561027	0.953905	0.992801	1.000
613	GOBP_CELLULAR_RESPONSE_TO_KETONE	GO:1901655	5	0.259139	0.550715	0.975904	0.996523	1.000
614	GOBP_RESPONSE_TO_MECHANICAL_STIMULUS	GO:0009612	6	0.243902	0.550654	0.973479	0.994921	1.000
615	REACTOME_POST_TRANSLATIONAL_PROTEIN_MODIFICATION	R-RNO-597592	7	0.234188	0.550396	0.969914	0.993431	1.000
616	GOBP_PEPTIDYL_LYSINE_MODIFICATION	GO:0018205	5	0.256173	0.549842	0.981482	0.992080	1.000
617	GOBP_REGULATION_OF_LEUKOCYTE_DIFFERENTIATION	GO:1902105	9	0.212615	0.540235	0.969609	0.994837	1.000
618	GOBP_CIRCULATORY_SYSTEM_PROCESS	GO:0003013	5	0.254032	0.539799	0.980798	0.993428	1.000
619	GOBP_METAL_ION_HOMEOSTASIS	GO:0055065	14	0.183963	0.535566	0.971698	0.993513	1.000
620	GOBP_AMINO_ACID_TRANSPORT	GO:0006865	6	0.237175	0.534135	0.981846	0.992465	1.000
621	GOBP_REGULATION_OF_HEMOPOIESIS	GO:1903706	10	0.201782	0.534026	0.967742	0.990893	1.000
622	GOBP_CARBOXYLIC_ACID_TRANSPORT	GO:0046942	6	0.237175	0.533323	0.971976	0.989580	1.000
623	GOBP_REGULATION_OF_CYTOSOLIC_CALCIUM_ION_CONCE...	GO:0051480	13	0.186371	0.532482	0.970549	0.988294	1.000
624	GOBP_ORGANIC_ANION_TRANSPORT	GO:0015711	6	0.237175	0.532296	0.980031	0.986794	1.000
625	GOBP_DIVALENT_INORGANIC_CATION_HOMEOSTASIS	GO:0072507	13	0.186371	0.522581	0.972490	0.988855	1.000
626	GOBP_CELLULAR_RESPONSE_TO_ALCOHOL	GO:0097306	5	0.246986	0.511750	0.987988	0.990736	1.000
627	HALLMARK_TNFA_SIGNALING_VIA_NFKB	M5890	14	0.164738	0.473874	0.989390	0.997981	1.000
628	GOBP_POSITIVE_REGULATION_OF_NEURON_PROJECTION_...	GO:0010976	5	0.213312	0.450531	0.998466	0.999848	1.000
629	GOBP_ORGANIC_ACID_TRANSPORT	GO:0015849	7	0.175111	0.420216	1.000000	1.000000	1.000
630	GOBP_POSITIVE_REGULATION_OF_PEPTIDYL_SERINE_PH...	GO:0033138	8	0.166069	0.413800	0.995726	0.999859	1.000
631	GOBP_REGULATION_OF_PEPTIDYL_SERINE_PHOSPHORYLA...	GO:0033135	8	0.166069	0.407824	1.000000	0.998574	1.000
632	GOBP_MONOAMINE_TRANSPORT	GO:0015844	5	-0.234939	-0.539969	0.983389	0.986805	1.000
633	GOBP_OSTEOCLAST_DIFFERENTIATION	GO:0030316	6	-0.215261	-0.551849	0.976676	0.994314	1.000
634	GOBP_RESPONSE_TO_KETONE	GO:1901654	7	-0.209877	-0.557894	0.980892	1.000000	1.000
635	GOBP_RESPONSE_TO_INTERLEUKIN_1	GO:0070555	5	-0.241161	-0.568188	0.985549	1.000000	1.000
636	GOBP_NEURON_DEATH	GO:0070997	18	-0.151896	-0.580561	0.958678	1.000000	1.000
637	GOBP_REGULATION_OF_CELL_SIZE	GO:0008361	5	-0.253012	-0.605023	0.964770	1.000000	1.000
638	GOBP_INORGANIC_ION_TRANSMEMBRANE_TRANSPORT	GO:0098660	9	-0.214062	-0.641498	0.927336	1.000000	1.000
639	GOBP_REGULATION_OF_OSTEOCLAST_DIFFERENTIATION	GO:0045670	5	-0.271720	-0.644746	0.929179	1.000000	1.000
640	GOBP_REGIONALIZATION	GO:0003002	6	-0.256098	-0.661413	0.928144	1.000000	1.000
641	GOBP_PATTERN_SPECIFICATION_PROCESS	GO:0007389	6	-0.256098	-0.664408	0.915584	1.000000	1.000
642	GOBP_AXON_DEVELOPMENT	GO:0061564	18	-0.168375	-0.665138	0.944186	1.000000	1.000

Continuation of Table **S16**

	NAME	ID	SIZE	ES	NES	NOM.p.val	FDR.q.val	FWER.p.val
643	GOBP_INNATE_IMMUNE_RESPONSE	GO:0045087	7	-0.246914	-0.669381	0.867069	1.000000	1.000
644	GOBP_NEUROTROPHIN_SIGNALING_PATHWAY	GO:0038179	6	-0.268293	-0.685256	0.880126	1.000000	1.000
645	GOBP_NEUROTROPHIN_TRK_RECEPTOR_SIGNALING_PATHWAY	GO:0048011	6	-0.268293	-0.694379	0.877976	1.000000	1.000
646	GOBP_LONG_TERM_MEMORY	GO:0007616	6	-0.256098	-0.694480	0.891374	1.000000	1.000
647	REACTOME_ASSEMBLY_AND_CELL_SURFACE_PRESENTATIO...	R-RNO-9609736	8	-0.249903	-0.714602	0.852941	1.000000	1.000
648	REACTOME_NEGATIVE_REGULATION_OF_NMDA_RECEPTOR_...	R-HSA-9617324	8	-0.249903	-0.732055	0.843949	1.000000	1.000
649	GOBP_RESPONSE_TO_ALCOHOL	GO:0097305	11	-0.221654	-0.742545	0.812950	1.000000	1.000
650	GOBP_DEFENSE_RESPONSE_TO_OTHER_ORGANISM	GO:0098542	9	-0.244763	-0.763849	0.775578	0.994076	1.000
651	GOBP_REGULATION_OF_PHOSPHOLIPASE_C_ACTIVITY	GO:1900274	5	-0.325301	-0.765502	0.789174	1.000000	1.000
652	GOBP_POSITIVE_REGULATION_OF_LIPASE_ACTIVITY	GO:0060193	5	-0.325301	-0.772834	0.753086	1.000000	1.000
653	GOBP_REGULATION_OF_MEMBRANE_POTENTIAL	GO:0042391	17	-0.205273	-0.774434	0.793860	1.000000	1.000
654	GOBP_CALCIUM_ION_TRANSMEMBRANE_TRANSPORT	GO:0070588	7	-0.283951	-0.777547	0.744548	1.000000	1.000
655	GOBP_CYTOSOLIC_CALCIUM_ION_TRANSPORT	GO:0060401	7	-0.283951	-0.779044	0.738853	1.000000	1.000
656	GOBP_NEURON_PROJECTION_GUIDANCE	GO:0097485	9	-0.261453	-0.785441	0.746875	1.000000	1.000
657	GOBP_CALCIUM_ION_TRANSPORT_INTO_CYTOSOL	GO:0060402	7	-0.283951	-0.788305	0.765060	1.000000	1.000
658	GOBP_EPHRIN_RECEPTOR_SIGNALING_PATHWAY	GO:0048013	5	-0.337349	-0.795211	0.719888	1.000000	1.000
659	REACTOME_ANTI_INFLAMMATORY_RESPONSE_FAVOURING_...	R-HSA-9662851	5	-0.349398	-0.811318	0.699717	1.000000	1.000
660	GOBP_ADENYLATE_CYCLASE_MODULATING_G_PROTEIN_CO...	GO:0007188	11	-0.253295	-0.835694	0.668990	1.000000	1.000
661	REACTOME_SIGNALING_BY_NTRK2_TRKB	R-RNO-9006115	6	-0.329268	-0.837158	0.658147	1.000000	1.000
662	GOBP_POSITIVE_REGULATION_OF_CATION_TRANSMEMBRA...	GO:1904064	5	-0.349398	-0.847774	0.675439	1.000000	1.000
663	GOBP_CATION_TRANSPORT	GO:0006812	25	-0.200800	-0.854463	0.697561	1.000000	1.000
664	GOBP_EXCITATORY_CHEMICAL_SYNAPTIC_TRANSMISSION	GO:0098976	5	-0.368844	-0.866533	0.633846	0.996438	1.000
665	GOBP_FATTY_ACID_TRANSPORT	GO:0015908	6	-0.329268	-0.870402	0.591429	1.000000	1.000
666	GOBP_RESPONSE_TO_ETHANOL	GO:0045471	5	-0.367185	-0.874586	0.601173	1.000000	1.000
667	GOBP_RESPONSE_TO_DRUG	GO:0042493	11	-0.268354	-0.883165	0.641221	1.000000	1.000
668	GOBP_SENSORY_PERCEPTION_OF_PAIN	GO:0019233	5	-0.367579	-0.887802	0.531609	1.000000	1.000
669	GOBP_RESPONSE_TO_NERVE_GROWTH_FACTOR	GO:1990089	7	-0.333333	-0.889620	0.557276	1.000000	1.000
670	GOBP_ADENYLATE_CYCLASE_INHIBITING_G_PROTEIN_CO...	GO:0007193	9	-0.297020	-0.890131	0.593333	1.000000	1.000
671	REACTOME_INTERFERON_SIGNALING	R-RNO-913531	6	-0.353659	-0.898999	0.571429	1.000000	1.000
672	GOBP_REGULATION_OF_METAL_ION_TRANSPORT	GO:0010959	6	-0.353659	-0.907339	0.563798	1.000000	1.000
673	GOBP_REGULATION_OF_POSTSYNAPTIC_MEMBRANE_POTEN...	GO:0060078	14	-0.249274	-0.909562	0.581590	1.000000	1.000
674	GOBP_DICARBOXYLIC_ACID_TRANSPORT	GO:0006835	5	-0.396002	-0.925038	0.508065	1.000000	1.000
675	GOBP_NERVE_DEVELOPMENT	GO:0021675	9	-0.311409	-0.932972	0.517915	1.000000	1.000
676	GOBP_POSITIVE_REGULATION_OF_LONG_TERM_SYNAPTIC...	GO:1900273	5	-0.397590	-0.941043	0.462908	1.000000	1.000
677	GOBP_NEGATIVE_REGULATION_OF_PROTEIN_CATABOLIC_...	GO:0042177	5	-0.404920	-0.946734	0.501577	1.000000	1.000
678	GOBP_ACIDIC_AMINO_ACID_TRANSPORT	GO:0015800	5	-0.396002	-0.951964	0.462567	1.000000	1.000
679	GOBP_MONOCARBOXYLIC_ACID_TRANSPORT	GO:0015718	5	-0.396002	-0.953832	0.510324	1.000000	1.000
680	GOBP_NEURON_APOPTOTIC_PROCESS	GO:0051402	12	-0.282176	-0.969210	0.488722	1.000000	1.000
681	GOBP_PROTEIN_COMPLEX_OLIGOMERIZATION	GO:0051259	6	-0.390244	-0.984699	0.444444	1.000000	1.000
682	GOBP_NEGATIVE_REGULATION_OF_NEURON_DEATH	GO:1901215	11	-0.296224	-0.993928	0.460993	1.000000	1.000
683	GOBP_REGULATION_OF_NEURON_DIFFERENTIATION	GO:0045664	5	-0.421687	-0.995958	0.404624	1.000000	1.000
684	GOBP_ADENYLATE_CYCLASE_INHIBITING_G_PROTEIN_CO...	GO:0007196	5	-0.424679	-1.019082	0.368732	1.000000	1.000
685	GOBP_REGULATION_OF_LONG_TERM_SYNAPTIC_POTENTIA...	GO:1900271	6	-0.402439	-1.025188	0.393082	1.000000	1.000

Continuation of Table **S16**

	NAME	ID	SIZE	ES	NES	NOM.p.val	FDR.q.val	FWER.p.val
686	GOBP_CATION_TRANSMEMBRANE_TRANSPORT	GO:0098655	18	-0.259978	-1.025837	0.378855	1.000000	1.000
687	GOBP_REGULATION_OF_TRANSPORTER_ACTIVITY	GO:0032409	17	-0.270550	-1.027270	0.386555	1.000000	1.000
688	REACTOME_NEURONAL_SYSTEM	R-RNO-112316	26	-0.240174	-1.029561	0.390110	1.000000	1.000
689	REACTOME_TRAFFICKING_OF_AMPA_RECEPTORS	R-RNO-399719	11	-0.326846	-1.044494	0.355072	1.000000	1.000
690	REACTOME_DAG_AND_IP3_SIGNALING	R-RNO-1489509	8	-0.362500	-1.054230	0.368601	1.000000	1.000
691	GOBP_PERIPHERAL_NERVOUS_SYSTEM_DEVELOPMENT	GO:0007422	9	-0.341772	-1.072286	0.341297	1.000000	1.000
692	REACTOME_NEUROTRANSMITTER_RECEPTORS_AND_POSTSY...	R-RNO-112314	22	-0.264780	-1.074473	0.324074	1.000000	1.000
693	GOBP_REGULATION_OF_CATION_TRANSMEMBRANE_TRANSPORT	GO:1904062	16	-0.290334	-1.111868	0.321429	1.000000	1.000
694	REACTOME_ACTIVATION_OF_NMDA_RECEPTORS_AND_POST...	R-RNO-442755	16	-0.303180	-1.115232	0.330709	1.000000	1.000
695	GOBP_REGULATION_OF_SIGNALING_RECEPTOR_ACTIVITY	GO:0010469	14	-0.308697	-1.115484	0.270270	1.000000	1.000
696	REACTOME_TRANSCRIPTIONAL_REGULATION_BY_MECP2	R-RNO-8986944	7	-0.400512	-1.118382	0.273927	1.000000	1.000
697	REACTOME_TRANSMISSION_ACROSS_CHEMICAL_SYNAPSES	R-RNO-112315	23	-0.287669	-1.194233	0.216667	0.879095	1.000
698	GOBP_REGULATION_OF_NEUROTRANSMITTER_RECEPTOR_A...	GO:0099601	13	-0.343780	-1.197952	0.253012	0.908238	1.000
699	REACTOME_VESICLE_MEDIATED_TRANSPORT	R-RNO-5653656	5	-0.518072	-1.218409	0.198347	0.880527	1.000
700	REACTOME_MEMBRANE_TRAFFICKING	R-RNO-199991	5	-0.518072	-1.226903	0.201149	0.895837	1.000
701	GOBP_RESPONSE_TO_ALKALOID	GO:0043279	5	-0.530120	-1.242053	0.184358	0.892776	1.000
702	GOBP_REGULATION_OF_CATION_CHANNEL_ACTIVITY	GO:2001257	14	-0.344012	-1.242065	0.188679	0.945244	1.000
703	GOBP_REGULATION_OF_NEUROTRANSMITTER_TRANSPORT	GO:0051588	6	-0.487805	-1.248661	0.193651	0.978972	1.000
704	GOBP_VESICLE_MEDIATED_TRANSPORT_IN_SYNAPSE	GO:0099003	7	-0.456790	-1.250071	0.201220	1.000000	1.000
705	GOBP_NEUROTRANSMITTER_SECRETION	GO:0007269	6	-0.487805	-1.257625	0.179331	1.000000	1.000
706	REACTOME_UNBLOCKING_OF_NMDA_RECEPTORS_GLUTAMAT...	R-RNO-438066	12	-0.385784	-1.301319	0.125000	0.976863	1.000
707	REACTOME_PROTEIN_PROTEIN_INTERACTIONS_AT_SYNAPSES	R-RNO-6794362	12	-0.380925	-1.314834	0.132353	1.000000	1.000
708	REACTOME_TRAFFICKING_OF_GLUR2_CONTAINING_AMPA_...	R-RNO-416993	8	-0.468963	-1.343074	0.132492	0.972490	1.000
709	GOBP_NEUROTRANSMITTER_TRANSPORT	GO:0006836	7	-0.493827	-1.352076	0.149123	1.000000	1.000
710	GOBP_REGULATION_OF_NEUROTRANSMITTER_LEVELS	GO:0001505	7	-0.493827	-1.356853	0.103896	1.000000	1.000
711	GOBP_REGULATION_OF_NMDA_RECEPTOR_ACTIVITY	GO:2000310	10	-0.435820	-1.373401	0.104918	1.000000	1.000
712	GOBP_MEMORY	GO:0007613	13	-0.402633	-1.382369	0.108787	1.000000	1.000
713	REACTOME_SYNAPTIC_ADHESION_LIKE_MOLECULES	R-RNO-8849932	9	-0.475681	-1.442328	0.064286	1.000000	1.000
714	GOBP_GLUTAMATE_RECEPTOR_SIGNALING_PATHWAY	GO:0007215	17	-0.392060	-1.442749	0.074074	1.000000	1.000
715	GOBP_IONOTROPIC_GLUTAMATE_RECEPTOR_SIGNALING_P...	GO:0035235	9	-0.474252	-1.448275	0.088328	1.000000	1.000
716	GOBP_NEGATIVE_REGULATION_OF_NEURON_APOPTOTIC_P...	GO:0043524	8	-0.499217	-1.468829	0.057239	1.000000	1.000
717	GOBP_SENSORY_PERCEPTION	GO:0007600	9	-0.478823	-1.484719	0.071429	1.000000	1.000
718	REACTOME_G_ALPHA_Z_SIGNALLING_EVENTS	R-RNO-418597	5	-0.674699	-1.613486	0.043353	1.000000	0.999

End of Table

Supplementary Table S17: CB early profile (1 DPL peak) GSEA results.

Begin of Table **S17**

	NAME	ID	SIZE	ES	NES	NOM.p.val	FDR.q.val	FWER.p.val
0	GOBP_REGULATION_OF_SIGNALING_RECEPTOR_ACTIVITY	GO:0010469	14	0.553427	1.737270	0.016667	1.000000	0.981
1	GOBP_PEPTIDYL_TYROSINE_MODIFICATION	GO:0018212	10	0.598815	1.641833	0.050439	1.000000	0.999
2	GOBP_POSITIVE_REGULATION_OF_TRANSMEMBRANE_TRAN...	GO:0034764	7	0.673470	1.640401	0.036559	1.000000	0.999
3	GOBP_REGULATION_OF_PEPTIDYL_TYROSINE_PHOSPHORY...	GO:0050730	9	0.619031	1.612860	0.030702	1.000000	0.999
4	GOBP_AMYLOID_PRECURSOR_PROTEIN_METABOLIC_PROCESS	GO:0042982	5	0.737072	1.606316	0.044872	1.000000	1.000
5	GOBP_REGULATION_OF_SYNAPSE_STRUCTURE_OR_ACTIVITY	GO:0050803	11	0.557700	1.594614	0.043384	1.000000	1.000
6	GOBP_AMYLOID_PRECURSOR_PROTEIN_CATABOLIC_PROCESS	GO:0042987	5	0.737072	1.591621	0.031683	1.000000	1.000
7	GOBP_DENDRITIC_SPINE_MORPHOGENESIS	GO:0060997	5	0.740500	1.580148	0.035644	1.000000	1.000
8	GOBP_REGULATION_OF_NEUROTRANSMITTER_RECEPTOR_A...	GO:0099601	13	0.515914	1.572800	0.046154	1.000000	1.000
9	GOBP_AMYLOID_BETA_METABOLIC_PROCESS	GO:0050435	5	0.737072	1.559440	0.042827	1.000000	1.000
10	GOBP_POSITIVE_REGULATION_OF_PEPTIDYL_TYROSINE_...	GO:0050731	8	0.597194	1.522464	0.062921	1.000000	1.000
11	GOBP_REGULATION_OF_NMDA_RECEPTOR_ACTIVITY	GO:2000310	10	0.541527	1.520396	0.060215	1.000000	1.000
12	GOBP_DENDRITE_MORPHOGENESIS	GO:0048813	6	0.663187	1.509494	0.055556	1.000000	1.000
13	GOBP_PROTEIN_KINASE_B_SIGNALING	GO:0043491	6	0.641143	1.497409	0.069444	1.000000	1.000
14	GOBP_POSITIVE_REGULATION_OF_CATION_TRANSMEMBRA...	GO:1904064	5	0.679893	1.475966	0.050998	1.000000	1.000
15	GOBP_DENDRITIC_SPINE_DEVELOPMENT	GO:0060996	6	0.633509	1.461829	0.093275	1.000000	1.000
16	GOBP_REGULATION_OF_PHOSPHOLIPASE_ACTIVITY	GO:0010517	6	0.631052	1.457953	0.080087	1.000000	1.000
17	GOBP_REGULATION_OF_LIPASE_ACTIVITY	GO:0060191	6	0.631052	1.453733	0.079060	1.000000	1.000
18	REACTOME_SIGNALING_BY_NTRK2_TRKB	R-RNO-9006115	6	0.618735	1.449941	0.096360	1.000000	1.000
19	REACTOME_TRANSCRIPTIONAL_REGULATION_BY_MECP2	R-RNO-8986944	7	0.599361	1.448450	0.088745	1.000000	1.000
20	GOBP_POSITIVE_REGULATION_OF_NEURON_PROJECTION_...	GO:0010976	5	0.670605	1.446736	0.092050	1.000000	1.000
21	GOBP_POSITIVE_REGULATION_OF_LIPASE_ACTIVITY	GO:0060193	5	0.648726	1.424224	0.098121	1.000000	1.000
22	GOBP_REGULATION_OF_PHOSPHOLIPASE_C_ACTIVITY	GO:1900274	5	0.648726	1.409200	0.103672	1.000000	1.000
23	GOBP_REGULATION_OF_CATION_TRANSMEMBRANE_TRANSPORT	GO:1904062	16	0.452929	1.406942	0.077103	1.000000	1.000
24	GOBP_DEVELOPMENTAL_CELL_GROWTH	GO:0048588	5	0.646533	1.391884	0.096774	1.000000	1.000
25	GOBP_TRANSMEMBRANE_TRANSPORT	GO:0055085	27	0.377326	1.377706	0.079012	1.000000	1.000
26	GOBP_REGULATION_OF_ANATOMICAL_STRUCTURE_MORPHO...	GO:0022603	17	0.414523	1.370964	0.097130	1.000000	1.000
27	REACTOME_RAS_ACTIVATION_UPON_CA2_INFLUX_THROUG...	R-HSA-442982	6	0.585494	1.326908	0.148760	1.000000	1.000
28	GOBP_NEURON_PROJECTION_ORGANIZATION	GO:0106027	8	0.509534	1.323843	0.148069	1.000000	1.000
29	REACTOME_SIGNALING_BY_INTERLEUKINS	R-RNO-449147	15	0.414773	1.307124	0.137472	1.000000	1.000
30	GOBP_CATION_TRANSMEMBRANE_TRANSPORT	GO:0098655	18	0.395074	1.304301	0.154589	1.000000	1.000
31	GOBP_REGULATION_OF_LONG_TERM_SYNAPTIC_POTENTIA...	GO:1900271	6	0.557423	1.302489	0.182008	1.000000	1.000
32	GOBP_POSTSYNAPSE_ORGANIZATION	GO:0099173	10	0.472396	1.300126	0.167391	1.000000	1.000
33	GOBP_CELLULAR_LIPID_METABOLIC_PROCESS	GO:0044255	7	0.508846	1.275120	0.177384	1.000000	1.000
34	GOBP_REGULATION_OF_CELLULAR_COMPONENT_SIZE	GO:0032535	7	0.518077	1.273581	0.181624	1.000000	1.000
35	GOBP_ACTIVATION_OF_PROTEIN_KINASE_ACTIVITY	GO:0032147	11	0.441929	1.261416	0.193478	1.000000	1.000
36	REACTOME_UNBLOCKING_OF_NMDA_RECEPTORS_GLUTAMAT...	R-RNO-438066	12	0.428290	1.258085	0.175115	1.000000	1.000
37	GOBP_REGULATION_OF_TRANSMEMBRANE_TRANSPORT	GO:0034762	23	0.364441	1.254415	0.179487	1.000000	1.000
38	GOBP_ORGANIC_HYDROXY_COMPOUND_METABOLIC_PROCESS	GO:1901615	7	0.508793	1.237507	0.223451	1.000000	1.000
39	GOBP_POSITIVE_REGULATION_OF_LEUKOCYTE_CELL_CEL...	GO:1903039	5	0.566699	1.222577	0.238494	1.000000	1.000
40	REACTOME_INTERLEUKIN_4_AND_INTERLEUKIN_13_SIGN...	R-RNO-6785807	8	0.478101	1.221130	0.230937	1.000000	1.000

Continuation of Table **S17**

	NAME	ID	SIZE	ES	NES	NOM.p.val	FDR.q.val	FWER.p.val
41	GOBP_SYNAPSE_ORGANIZATION	GO:0050808	17	0.367498	1.206755	0.193995	1.000000	1.000
42	GOBP_ANION_TRANSMEMBRANE_TRANSPORT	GO:0098656	7	0.484816	1.202431	0.245119	1.000000	1.000
43	GOBP_POSITIVE_REGULATION_OF_CELL_CELL_ADHESION	GO:0022409	5	0.566699	1.199950	0.258621	1.000000	1.000
44	GOBP_POSITIVE_REGULATION_OF_INTRACELLULAR_SIGN...	GO:1902533	14	0.379754	1.193349	0.252294	1.000000	1.000
45	GOBP_POSITIVE_REGULATION_OF_CELL_PROJECTION_OR...	GO:0031346	10	0.427649	1.184902	0.231626	1.000000	1.000
46	GOBP_RESPONSE_TO_ETHANOL	GO:0045471	5	0.542169	1.183501	0.296460	1.000000	1.000
47	HALLMARK_APOPTOSIS	M5902	6	0.510464	1.178983	0.266385	1.000000	1.000
48	GOBP_POSITIVE_REGULATION_OF_CELLULAR_COMPONENT...	GO:0044089	9	0.434788	1.169869	0.261641	1.000000	1.000
49	GOBP_REGULATION_OF_CELL_DEVELOPMENT	GO:0060284	15	0.370353	1.157085	0.290179	1.000000	1.000
50	GOBP_POSITIVE_REGULATION_OF_MYELOID_LEUKOCYTE_...	GO:0002763	6	0.503249	1.156960	0.303226	1.000000	1.000
51	GOBP_SYNAPTIC_TRANSMISSION_GLUTAMATERGIC	GO:0035249	13	0.384734	1.156334	0.260090	1.000000	1.000
52	GOBP_REGULATION_OF_CELLULAR_CATABOLIC_PROCESS	GO:0031329	10	0.416026	1.156097	0.261574	1.000000	1.000
53	GOBP_POSITIVE_REGULATION_OF_PHOSPHATIDYLINOSIT...	GO:0014068	5	0.538615	1.152584	0.316929	1.000000	1.000
54	GOBP_POSITIVE_REGULATION_OF_CELL_DEVELOPMENT	GO:0010720	12	0.379869	1.150691	0.272517	1.000000	1.000
55	GOBP_POSITIVE_REGULATION_OF_SYNAPTIC_TRANSMISSION	GO:0050806	15	0.365869	1.148001	0.274882	1.000000	1.000
56	GOBP_REGULATION_OF_SYNAPTIC_TRANSMISSION_GLUTA...	GO:0051966	12	0.390195	1.145650	0.281250	1.000000	1.000
57	GOBP_POSITIVE_REGULATION_OF_PROTEIN_KINASE_ACT...	GO:0045860	19	0.337902	1.141511	0.274827	1.000000	1.000
58	GOBP_REGULATION_OF_POSTSYNAPSE_ORGANIZATION	GO:0099175	6	0.498165	1.137474	0.307036	1.000000	1.000
59	GOBP_POSITIVE_REGULATION_OF_LONG_TERM_SYNAPTIC...	GO:1900273	5	0.526397	1.134293	0.326489	1.000000	1.000
60	GOBP_POSITIVE_REGULATION_OF_CELL_GROWTH	GO:0030307	6	0.477160	1.131145	0.321962	1.000000	1.000
61	GOBP_REGULATION_OF_CATION_CHANNEL_ACTIVITY	GO:2001257	14	0.360711	1.129517	0.286667	1.000000	1.000
62	REACTOME_ASSEMBLY_AND_CELL_SURFACE_PRESENTATIO...	R-RNO-9609736	8	0.437876	1.128746	0.292411	1.000000	1.000
63	GOBP_POSITIVE_REGULATION_OF_SMOOTH_MUSCLE_CELL...	GO:0048661	5	0.518698	1.128432	0.347107	1.000000	1.000
64	GOBP_POSITIVE_REGULATION_OF_TRANSFERASE_ACTIVITY	GO:0051347	20	0.329227	1.125665	0.286976	1.000000	1.000
65	GOBP_MEMBRANE_PROTEIN_PROTEOLYSIS	GO:0033619	6	0.488537	1.122872	0.318275	1.000000	1.000
66	GOBP_REGULATION_OF_INTRACELLULAR_SIGNAL_TRANSD...	GO:1902531	21	0.325240	1.121250	0.291866	1.000000	1.000
67	REACTOME_NEGATIVE_REGULATION_OF_NMDA_RECEPTOR_...	R-HSA-961732	8	0.437876	1.115430	0.317181	1.000000	1.000
68	GOBP_POSITIVE_REGULATION_OF_NEUROGENESIS	GO:0050769	12	0.379869	1.113779	0.310421	1.000000	1.000
69	REACTOME_CYTOKINE_SIGNALING_IN_IMMUNE_SYSTEM	R-RNO-1280215	21	0.323308	1.109734	0.294664	1.000000	1.000
70	GOBP_REGIONALIZATION	GO:0003002	6	0.471138	1.108806	0.321962	1.000000	1.000
71	GOBP_NEUROINFLAMMATORY_RESPONSE	GO:0150076	5	0.505053	1.107442	0.344262	1.000000	1.000
72	GOBP_RECEPTOR_INTERNALIZATION	GO:0031623	5	0.504324	1.107137	0.343313	1.000000	1.000
73	GOBP_POSITIVE_REGULATION_OF_NERVOUS_SYSTEM_DEV...	GO:0051962	12	0.379869	1.099315	0.350000	1.000000	1.000
74	GOBP_PATTERN_SPECIFICATION_PROCESS	GO:0007389	6	0.471138	1.096640	0.330595	1.000000	1.000
75	GOBP_CALCIUM_MEDIATED_SIGNALING	GO:0019722	10	0.400984	1.095929	0.334047	1.000000	1.000
76	GOBP_POSITIVE_REGULATION_OF_DNA_BINDING_TRANSC...	GO:0051091	10	0.398091	1.095271	0.327434	1.000000	1.000
77	GOBP_CHEMICAL_SYNAPTIC_TRANSMISSION_POSTSYNAPTIC	GO:0099565	9	0.406591	1.088896	0.361419	1.000000	1.000
78	GOBP_REGULATION_OF_CATABOLIC_PROCESS	GO:0009894	13	0.358236	1.086171	0.345416	1.000000	1.000
79	GOBP_RECEPTOR_METABOLIC_PROCESS	GO:0043112	5	0.504324	1.082956	0.364017	1.000000	1.000
80	REACTOME_DEVELOPMENTAL_BIOLOGY	R-RNO-1266738	23	0.311160	1.081602	0.340090	1.000000	1.000
81	REACTOME_LONG_TERM_POTENTIATION	R-HSA-9620244	10	0.393010	1.081486	0.359140	1.000000	1.000
82	REACTOME_SYNAPTIC_ADHESION_LIKE_MOLECULES	R-RNO-8849932	9	0.402539	1.079910	0.350731	1.000000	1.000
83	GOBP_NEGATIVE_REGULATION_OF_CATABOLIC_PROCESS	GO:0009895	8	0.416918	1.079067	0.354626	1.000000	1.000

Continuation of Table **S17**

	NAME	ID	SIZE	ES	NES	NOM.p.val	FDR.q.val	FWER.p.val
84	GOBP_NEGATIVE_REGULATION_OF_PEPTIDASE_ACTIVITY	GO:0010466	6	0.471463	1.077746	0.371179	1.000000	1.000
85	GOBP_BIOLOGICAL_PROCESS_INVOLVED_IN_SYMBIOTIC_...	GO:004440	11	0.381215	1.077044	0.371495	1.000000	1.000
86	GOBP_REGULATION_OF_PROTEIN_CATABOLIC_PROCESS	GO:0042176	7	0.434353	1.076275	0.362869	1.000000	1.000
87	REACTOME_ACTIVATION_OF_NMDA_RECEPTORS_AND_POST...	R-RNO-442755	16	0.337730	1.075855	0.350515	1.000000	1.000
88	GOBP_DEVELOPMENTAL_MATURATION	GO:0021700	5	0.501132	1.075507	0.395299	1.000000	1.000
89	GOBP_LEARNING	GO:0007612	13	0.355124	1.075018	0.343750	1.000000	1.000
90	GOBP_NEGATIVE_REGULATION_OF_LOCOMOTION	GO:0040013	5	0.490676	1.070620	0.403614	1.000000	1.000
91	GOBP_SMALL_GTPASE_MEDIATED_SIGNAL_TRANSDUCTION	GO:0007264	8	0.407477	1.066816	0.364583	1.000000	1.000
92	GOBP_AMEBOIDAL_TYPE_CELL_MIGRATION	GO:0001667	11	0.371425	1.061049	0.374449	1.000000	1.000
93	GOBP_POSITIVE_REGULATION_OF_PROTEIN_TYROSINE_K...	GO:0061098	5	0.489330	1.059815	0.388664	1.000000	1.000
94	GOBP_REGULATION_OF_VASCULATURE_DEVELOPMENT	GO:1901342	5	0.494091	1.059164	0.397727	1.000000	1.000
95	GOBP_REGULATION_OF_MEMBRANE_POTENTIAL	GO:0042391	17	0.325084	1.058898	0.383178	1.000000	1.000
96	GOBP_GLIAL_CELL_DIFFERENTIATION	GO:0010001	10	0.386239	1.058745	0.344754	1.000000	1.000
97	GOBP_CELL_MIGRATION	GO:0016477	22	0.299695	1.058637	0.373239	1.000000	1.000
98	GOBP_MAINTENANCE_OF_LOCATION	GO:0051235	7	0.445555	1.058433	0.375271	1.000000	1.000
99	GOBP_REGULATION_OF_PROTEIN_TYROSINE_KINASE_ACT...	GO:0061097	5	0.489330	1.055188	0.407834	1.000000	1.000
100	GOBP_REGULATION_OF_PEPTIDE_SECRETION	GO:0002791	7	0.418152	1.049450	0.406393	1.000000	1.000
101	GOBP_OSTEOBLAST_DIFFERENTIATION	GO:0001649	5	0.476929	1.045568	0.405063	1.000000	1.000
102	GOBP_POSITIVE_REGULATION_OF_ION_TRANSPORT	GO:0043270	17	0.310992	1.044829	0.378076	1.000000	1.000
103	GOBP_REGULATION_OF_ENDOTHELIAL_CELL_MIGRATION	GO:0010594	5	0.476726	1.044273	0.411628	1.000000	1.000
104	GOBP_ASSOCIATIVE_LEARNING	GO:0008306	7	0.418025	1.041709	0.405579	1.000000	1.000
105	GOBP_REGULATION_OF_MAP_KINASE_ACTIVITY	GO:0043405	9	0.389900	1.041089	0.409692	1.000000	1.000
106	GOBP_CELLULAR_COMPONENT_DISASSEMBLY	GO:0022411	6	0.451399	1.039386	0.405830	1.000000	1.000
107	GOBP_POSITIVE_REGULATION_OF_MAP_KINASE_ACTIVITY	GO:0043406	6	0.437450	1.038454	0.412903	1.000000	1.000
108	GOBP_INFLAMMATORY_RESPONSE	GO:0006954	12	0.359969	1.037183	0.396514	1.000000	1.000
109	GOBP_GLUCOSE_METABOLIC_PROCESS	GO:0006006	6	0.442840	1.032139	0.415778	1.000000	1.000
110	GOBP_MACROMOLECULE_CATABOLIC_PROCESS	GO:0009057	12	0.349866	1.031642	0.404922	1.000000	1.000
111	GOBP_REGULATION_OF_PROTEIN_KINASE_ACTIVITY	GO:0045859	22	0.293744	1.028128	0.401478	1.000000	1.000
112	GOBP_MONOSACCHARIDE_METABOLIC_PROCESS	GO:0005996	6	0.442840	1.026317	0.422078	1.000000	1.000
113	GOBP_POSITIVE_REGULATION_OF_CATALYTIC_ACTIVITY	GO:0043085	30	0.272058	1.026071	0.427184	1.000000	1.000
114	GOBP_REGULATION_OF_CELLULAR_COMPONENT_MOVEMENT	GO:0051270	15	0.327632	1.024178	0.433036	1.000000	1.000
115	GOBP_LEUKOCYTE_MIGRATION	GO:0050900	6	0.439851	1.016347	0.451745	1.000000	1.000
116	GOBP_PROTEIN_CONTAINING_COMPLEX_SUBUNIT_ORGANI...	GO:0043933	14	0.323505	1.012129	0.432314	1.000000	1.000
117	GOBP_CELL_JUNCTION_ORGANIZATION	GO:0034330	19	0.303180	1.012069	0.422062	1.000000	1.000
118	GOBP_REGULATION_OF_TRANSFERASE_ACTIVITY	GO:0051338	22	0.293744	1.009868	0.415423	1.000000	1.000
119	REACTOME_NERVOUS_SYSTEM_DEVELOPMENT	R-RNO-9675108	15	0.321819	1.008948	0.463470	1.000000	1.000
120	GOBP_POSITIVE_REGULATION_OF_HEMOPOIESIS	GO:1903708	7	0.420502	1.002097	0.445148	1.000000	1.000
121	GOBP_PEPTIDE_METABOLIC_PROCESS	GO:0006518	12	0.338941	0.995506	0.436242	1.000000	1.000
122	GOBP_ACTIVATION_OF_MAPK_ACTIVITY	GO:0000187	6	0.437450	0.991307	0.470982	1.000000	1.000
123	GOBP_EXTRINSIC_APOPTOTIC_SIGNALING_PATHWAY	GO:0097191	7	0.407933	0.989224	0.441176	1.000000	1.000
124	GOBP_LONG_TERM_SYNAPTIC_DEPRESSION	GO:0060292	5	0.448846	0.987093	0.477974	1.000000	1.000
125	GOBP_REGULATION_OF_CELL_SIZE	GO:0008361	5	0.455062	0.986457	0.465217	1.000000	1.000
126	GOBP_REGULATION_OF_POSTSYNAPTIC_MEMBRANE_POTEN...	GO:0060078	14	0.322480	0.983107	0.475059	1.000000	1.000

Continuation of Table **S17**

	NAME	ID	SIZE	ES	NES	NOM.p.val	FDR.q.val	FWER.p.val
127	GOBP_REGULATION_OF_GLIOGENESIS	GO:0014013	5	0.450503	0.977214	0.484009	1.000000	1.000
128	GOBP_RESPONSE_TO_NERVE_GROWTH_FACTOR	GO:1990089	7	0.407407	0.972910	0.474012	1.000000	1.000
129	GOBP_CELLULAR_AMIDE_METABOLIC_PROCESS	GO:0043603	13	0.324687	0.969786	0.484234	1.000000	1.000
130	GOBP_REGULATION_OF_EXTRINSIC_APOPTOTIC_SIGNALI...	GO:2001236	6	0.417758	0.967784	0.511677	1.000000	1.000
131	GOBP_MULTICELLULAR_ORGANISMAL_RESPONSE_TO_STRESS	GO:0033555	5	0.445724	0.966886	0.479570	1.000000	1.000
132	GOBP_REGULATION_OF_DEVELOPMENTAL_GROWTH	GO:0048638	9	0.364100	0.965483	0.516269	1.000000	1.000
133	GOBP_POSITIVE_REGULATION_OF_BINDING	GO:0051099	6	0.413650	0.963924	0.484979	1.000000	1.000
134	GOBP_POSITIVE_REGULATION_OF_GLIOGENESIS	GO:0014015	5	0.450503	0.963718	0.495868	1.000000	1.000
135	GOBP_REGULATION_OF_CELL_PROJECTION_ORGANIZATION	GO:0031344	20	0.283550	0.957109	0.502222	1.000000	1.000
136	HALLMARK_EPITHELIAL_MESENCHYMAL_TRANSITION	M5930	5	0.438653	0.955454	0.524590	1.000000	1.000
137	GOBP_DENDRITE_DEVELOPMENT	GO:0016358	9	0.364871	0.953309	0.531959	1.000000	1.000
138	GOBP_NEGATIVE_REGULATION_OF_APOPTOTIC_SIGNALIN...	GO:2001234	7	0.387210	0.947575	0.505643	1.000000	1.000
139	GOBP_LEUKOCYTE_CELL_CELL_ADHESION	GO:0007159	6	0.415721	0.946043	0.516340	1.000000	1.000
140	GOBP_LIPID_METABOLIC_PROCESS	GO:0006629	10	0.351092	0.945994	0.489177	1.000000	1.000
141	GOBP_REGULATION_OF_OSTEOCLAST_DIFFERENTIATION	GO:0045670	5	0.443696	0.944510	0.548387	1.000000	1.000
142	GOBP_REGULATION_OF_EPITHELIAL_CELL_MIGRATION	GO:0010632	6	0.402629	0.939678	0.521839	1.000000	1.000
143	GOBP_NEGATIVE_REGULATION_OF_CELL_DEVELOPMENT	GO:0010721	6	0.398058	0.937006	0.520971	1.000000	1.000
144	GOBP_POSITIVE_REGULATION_OF_PEPTIDYL_SERINE_PH...	GO:0033138	8	0.365276	0.936772	0.534216	1.000000	1.000
145	GOBP_POSITIVE_REGULATION_OF_MYELOID_CELL_DIFFE...	GO:0045639	7	0.371227	0.935273	0.557576	1.000000	1.000
146	GOBP_POSITIVE_REGULATION_OF_DEVELOPMENTAL_PROCESS	GO:0051094	32	0.249967	0.934314	0.563380	1.000000	1.000
147	REACTOME_INFECTIOUS_DISEASE	R-HSA-5663205	13	0.303754	0.932967	0.498824	1.000000	1.000
148	GOBP_CELL_SURFACE_RECEPTOR_SIGNALING_PATHWAY_I...	GO:1905114	15	0.298042	0.932932	0.544643	1.000000	1.000
149	GOBP_POSITIVE_REGULATION_OF_CELL_ADHESION	GO:0045785	7	0.382311	0.928428	0.551148	1.000000	1.000
150	GOBP_POSITIVE_REGULATION_OF_LOCOMOTION	GO:0040017	11	0.328671	0.927256	0.548753	1.000000	1.000
151	GOBP_POSITIVE_REGULATION_OF_HYDROLASE_ACTIVITY	GO:0051345	16	0.282051	0.925516	0.557920	1.000000	1.000
152	GOBP_REGULATION_OF_PEPTIDYL_SERINE_PHOSPHORYLA...	GO:0033135	8	0.365276	0.922288	0.520607	1.000000	1.000
153	GOBP_REGULATION_OF_NEUROGENESIS	GO:0050767	14	0.304957	0.921722	0.562637	1.000000	1.000
154	GOBP_POSITIVE_REGULATION_OF_SMALL_MOLECULE_MET...	GO:0062013	5	0.431685	0.917948	0.566160	1.000000	1.000
155	GOBP_LONG_TERM_MEMORY	GO:0007616	6	0.403833	0.916711	0.556769	1.000000	1.000
156	GOBP_DICARBOXYLIC_ACID_TRANSPORT	GO:0006835	5	0.425305	0.914108	0.551579	1.000000	1.000
157	GOBP_PROTEOLYSIS	GO:0006508	17	0.277918	0.911478	0.580645	1.000000	1.000
158	GOBP_ACIDIC_AMINO_ACID_TRANSPORT	GO:0015800	5	0.425305	0.909964	0.567194	1.000000	1.000
159	GOBP_MONOCARBOXYLIC_ACID_TRANSPORT	GO:0015718	5	0.425305	0.906268	0.567265	1.000000	1.000
160	GOBP_PHAGOCYTOSIS	GO:0006909	5	0.422278	0.905543	0.590810	1.000000	1.000
161	GOBP_REGULATION_OF_NERVOUS_SYSTEM_DEVELOPMENT	GO:0051960	15	0.283040	0.902900	0.584245	1.000000	1.000
162	GOBP_REGULATION_OF_NERVOUS_SYSTEM_PROCESS	GO:0031644	8	0.360080	0.902207	0.548246	1.000000	1.000
163	GOBP_PROTEIN_CATABOLIC_PROCESS	GO:0030163	8	0.356101	0.896435	0.588636	1.000000	1.000
164	GOBP_OSTEOCLAST_DIFFERENTIATION	GO:0030316	6	0.393397	0.896094	0.577825	1.000000	1.000
165	GOBP_DEFENSE_RESPONSE	GO:0006952	19	0.267423	0.894638	0.623810	1.000000	1.000
166	GOBP_REGULATION_OF_BINDING	GO:0051098	11	0.306993	0.889018	0.598699	1.000000	1.000
167	GOBP_DEVELOPMENTAL_GROWTH	GO:0048589	10	0.327520	0.887204	0.594714	1.000000	1.000
168	GOBP_FATTY_ACID_TRANSPORT	GO:0015908	6	0.384886	0.884425	0.614256	1.000000	1.000
169	REACTOME_CREB1_PHOSPHORYLATION_THROUGH_NMDA_RE...	R-RNO-442742	8	0.357587	0.883979	0.609244	1.000000	1.000

Continuation of Table **S17**

	NAME	ID	SIZE	ES	NES	NOM.p.val	FDR.q.val	FWER.p.val
170	GOBP_GLIOGENESIS	GO:0042063	12	0.294140	0.876877	0.619835	1.000000	1.000
171	GOBP_IONOTROPIC_GLUTAMATE_RECEPTOR_SIGNALING_P...	GO:0035235	9	0.325609	0.874038	0.633640	1.000000	1.000
172	GOBP_CARBOHYDRATE_DERIVATIVE_METABOLIC_PROCESS	GO:1901135	7	0.358171	0.868516	0.614512	1.000000	1.000
173	GOBP_REGULATION_OF_ANION_TRANSMEMBRANE_TRANSPORT	GO:1903959	5	0.396508	0.861733	0.640000	1.000000	1.000
174	GOBP_REGULATION_OF_MITOTIC_CELL_CYCLE	GO:0007346	5	0.398101	0.856412	0.605603	1.000000	1.000
175	GOBP_REGULATION_OF_RECEPTOR_MEDIATED_ENDOCYTOSIS	GO:0048259	5	0.389182	0.856377	0.642105	1.000000	1.000
176	HALLMARK_PI3K_AKT_MTOR_SIGNALING	M5923	6	0.379046	0.856030	0.623950	1.000000	1.000
177	GOBP_REGULATION_OF_DEFENSE_RESPONSE	GO:0031347	7	0.344986	0.855838	0.621681	1.000000	1.000
178	GOBP_REGULATION_OF_PHOSPHATIDYLINOSITOL_3_KINA...	GO:0014066	6	0.365162	0.853868	0.645570	1.000000	1.000
179	GOBP_POSITIVE_REGULATION_OF_IMMUNE_SYSTEM_PROCESS	GO:0002684	18	0.259599	0.853585	0.695175	1.000000	1.000
180	GOBP_RESPONSE_TO_ACID_CHEMICAL	GO:0001101	6	0.356119	0.849036	0.612159	1.000000	1.000
181	GOBP_CATION_TRANSPORT	GO:0006812	25	0.239421	0.847218	0.696471	1.000000	1.000
182	GOBP_NEURON_PROJECTION_GUIDANCE	GO:0097485	9	0.315733	0.846694	0.646018	1.000000	1.000
183	GOBP_ORGANONITROGEN_COMPOUND_CATABOLIC_PROCESS	GO:1901565	9	0.314072	0.845971	0.654945	1.000000	1.000
184	GOBP_REGULATION_OF_CELLULAR_COMPONENT_BIOGENESIS	GO:0044087	10	0.311204	0.845318	0.634146	1.000000	1.000
185	GOBP_REGULATION_OF_INFLAMMATORY_RESPONSE	GO:0050727	7	0.344986	0.844846	0.639013	1.000000	1.000
186	GOBP_OSSIFICATION	GO:0001503	9	0.321536	0.843701	0.673709	1.000000	1.000
187	GOBP_NEGATIVE_REGULATION_OF_CATALYTIC_ACTIVITY	GO:0043086	17	0.265127	0.843126	0.688940	1.000000	1.000
188	GOBP_INOSITOL_LIPID_MEDIATED_SIGNALING	GO:0048017	8	0.325680	0.840450	0.664516	1.000000	1.000
189	GOBP_REGULATION_OF_METAL_ION_TRANSPORT	GO:0010959	6	0.366070	0.839500	0.649378	1.000000	1.000
190	GOBP_BLOOD_VESSEL_ENDOTHELIAL_CELL_MIGRATION	GO:0043534	8	0.324666	0.839001	0.629956	1.000000	1.000
191	GOBP_EMBRYO_DEVELOPMENT	GO:0009790	14	0.271175	0.838341	0.697674	1.000000	1.000
192	GOBP_NEGATIVE_REGULATION_OF_PROTEOLYSIS	GO:0045861	8	0.329330	0.838054	0.672234	1.000000	1.000
193	GOBP_ORGANIC_ANION_TRANSPORT	GO:0015711	6	0.359263	0.837877	0.612903	1.000000	1.000
194	REACTOME_ANTI_INFLAMMATORY_RESPONSE_FAVOURING_...	R-HSA-9662851	5	0.392749	0.837121	0.676349	1.000000	1.000
195	GOBP_ENDOTHELIAL_CELL_MIGRATION	GO:0043542	8	0.324666	0.836888	0.658174	1.000000	1.000
196	GOBP_AMINO_ACID_TRANSPORT	GO:0006865	6	0.359263	0.833728	0.651163	1.000000	1.000
197	GOBP_REGULATION_OF_TRANSPORTER_ACTIVITY	GO:0032409	17	0.257673	0.833500	0.704104	1.000000	1.000
198	GOBP_POSITIVE_REGULATION_OF_CELLULAR_COMPONENT...	GO:0051130	24	0.228588	0.830867	0.719626	1.000000	1.000
199	GOBP_ORGANOPHOSPHATE_METABOLIC_PROCESS	GO:0019637	7	0.336993	0.829930	0.659836	1.000000	1.000
200	REACTOME_TOLL_LIKE_RECEPTOR_TLR1_TLR2_CASCADE	R-RNO-168179	8	0.318183	0.828919	0.639130	1.000000	1.000
201	GOBP_NEGATIVE_REGULATION_OF_MULTICELLULAR_ORGA...	GO:0051241	15	0.263888	0.825812	0.722222	1.000000	1.000
202	GOBP_ERK1_AND_ERK2_CASCADE	GO:0070371	5	0.380414	0.824145	0.696099	1.000000	1.000
203	GOBP_POSITIVE_REGULATION_OF_MOLECULAR_FUNCTION	GO:0044093	38	0.212001	0.823991	0.741206	1.000000	1.000
204	REACTOME_TOLL_LIKE_RECEPTOR_CASCADES	R-RNO-168898	8	0.318183	0.822216	0.686147	1.000000	1.000
205	GOBP_POSITIVE_REGULATION_OF_DEVELOPMENTAL_GROWTH	GO:0048639	8	0.322491	0.821554	0.699317	1.000000	1.000
206	GOBP_PHOSPHATIDYLINOSITOL_3_KINASE_SIGNALING	GO:0014065	7	0.332870	0.820578	0.653445	1.000000	1.000
207	GOBP_NEGATIVE_REGULATION_OF_GENE_EXPRESSION	GO:0010629	12	0.281832	0.820019	0.672414	1.000000	1.000
208	GOBP_ION_HOMEOSTASIS	GO:0050801	16	0.260396	0.814869	0.709890	1.000000	1.000
209	GOBP_CARBOXYLIC_ACID_TRANSPORT	GO:0046942	6	0.359263	0.811294	0.675676	1.000000	1.000
210	GOBP_MYOTUBE_DIFFERENTIATION	GO:0014902	5	0.371388	0.810892	0.697425	1.000000	1.000
211	GOBP_REGULATION_OF_MUSCLE_CELL_DIFFERENTIATION	GO:0051147	6	0.354563	0.810393	0.699789	1.000000	1.000
212	GOBP_HEART_DEVELOPMENT	GO:0007507	6	0.347103	0.809820	0.704782	1.000000	1.000

Continuation of Table **S17**

	NAME	ID	SIZE	ES	NES	NOM.p.val	FDR.q.val	FWER.p.val
213	GOBP_ORGANIC_ACID_TRANSPORT	GO:0015849	7	0.329990	0.808744	0.664336	1.000000	1.000
214	GOBP_ADENYLATE_CYCLASE_INHIBITING_G_PROTEIN_CO...	GO:0007193	5	0.367498	0.805808	0.684322	1.000000	1.000
215	REACTOME_TOLL_LIKE_RECEPTOR_9_TLR9_CASCADE	R-RNO-168138	8	0.318183	0.805366	0.695279	1.000000	1.000
216	GOBP_IN_UTERO_EMBRYONIC_DEVELOPMENT	GO:0001701	8	0.317241	0.803982	0.709607	1.000000	1.000
217	GOBP_RESPONSE_TO_NUTRIENT	GO:0007584	5	0.373494	0.803456	0.706383	1.000000	1.000
218	GOBP_MUSCLE_CELL_DEVELOPMENT	GO:0055001	5	0.380880	0.802668	0.712153	1.000000	1.000
219	REACTOME_MYD88_INDEPENDENT_TLR4_CASCADE	R-RNO-166166	8	0.318183	0.801749	0.700214	1.000000	1.000
220	GOBP_RECEPTOR_MEDIATED_ENDOCYTOSIS	GO:0006898	6	0.349180	0.800684	0.692771	1.000000	1.000
221	GOBP_REGULATION_OF_PEPTIDASE_ACTIVITY	GO:0052547	10	0.292289	0.796440	0.712389	1.000000	1.000
222	GOBP_CARDIAC_MUSCLE_TISSUE_DEVELOPMENT	GO:0048738	6	0.347103	0.795046	0.702643	1.000000	1.000
223	GOBP_INORGANIC_ION_TRANSMEMBRANE_TRANSPORT	GO:0098660	9	0.302462	0.789109	0.737443	1.000000	1.000
224	GOBP_REGULATION_OF_ENDOCYTOSIS	GO:0030100	6	0.337786	0.783627	0.740506	1.000000	1.000
225	GOBP_POSITIVE_REGULATION_OF_MAPK_CASCADE	GO:0043410	9	0.290787	0.781524	0.746204	1.000000	1.000
226	GOBP_REGULATION_OF_PROTEIN_LOCALIZATION_TO_CEL...	GO:1904375	6	0.343587	0.780996	0.723577	1.000000	1.000
227	REACTOME_EXTRACELLULAR_MATRIX_ORGANIZATION	R-RNO-1474244	5	0.355376	0.780906	0.754132	1.000000	1.000
228	GOBP_RAS_PROTEIN_SIGNAL_TRANSDUCTION	GO:0007265	5	0.354766	0.778564	0.719123	1.000000	1.000
229	GOBP_MAPK_CASCADE	GO:0000165	18	0.237574	0.778521	0.791011	1.000000	1.000
230	GOBP_POSITIVE_REGULATION_OF_GROWTH	GO:0045927	9	0.296827	0.777671	0.740000	1.000000	1.000
231	GOBP_IMPORT_INTO_CELL	GO:0098657	6	0.347569	0.777326	0.753275	1.000000	1.000
232	GOBP_GENERATION_OF_PRECURSOR_METABOLITES_AND_E...	GO:0006091	5	0.358022	0.773973	0.758621	1.000000	1.000
233	GOBP_REGULATION_OF_MUSCLE_ADAPTATION	GO:0043502	5	0.355997	0.770206	0.752083	1.000000	1.000
234	GOBP_NEGATIVE_REGULATION_OF_RESPONSE_TO_EXTERN...	GO:0032102	5	0.357464	0.768025	0.752119	1.000000	1.000
235	GOBP_CALCIUM_ION_TRANSPORT	GO:0006816	11	0.259740	0.767802	0.783158	1.000000	1.000
236	GOBP_CELL_GROWTH	GO:0016049	10	0.273934	0.764706	0.776824	1.000000	1.000
237	GOBP_TELENCEPHALON_DEVELOPMENT	GO:0021537	6	0.329295	0.763871	0.776000	1.000000	1.000
238	REACTOME_MAPK_FAMILY_SIGNALING_CASCADES	R-RNO-5683057	12	0.259837	0.760389	0.804989	1.000000	1.000
239	GOBP_REGULATION_OF_VASCULAR_ASSOCIATED_SMOOTH_...	GO:1904705	5	0.360374	0.760343	0.742739	1.000000	1.000
240	REACTOME_POST_TRANSLATIONAL_PROTEIN_MODIFICATION	R-RNO-597592	7	0.305997	0.757408	0.772727	1.000000	1.000
241	GOBP_EMBRYO_DEVELOPMENT_ENDING_IN_BIRTH_OR_EGG...	GO:0009792	9	0.282313	0.751503	0.780702	1.000000	1.000
242	GOBP_SMOOTH_MUSCLE_CELL_PROLIFERATION	GO:0048659	6	0.322037	0.749119	0.790948	1.000000	1.000
243	GOBP_CELL_MORPHOGENESIS_INVOLVED_IN_DIFFERENTI...	GO:0000904	21	0.215358	0.748977	0.803097	1.000000	1.000
244	GOBP_CYTOSOLIC_CALCIUM_ION_TRANSPORT	GO:0060401	7	0.310150	0.745409	0.761388	1.000000	1.000
245	GOBP_ACTIVATION_OF_IMMUNE_RESPONSE	GO:0002253	5	0.343390	0.744703	0.766004	1.000000	1.000
246	GOBP_CALCIUM_ION_TRANSPORT_INTO_CYTOSOL	GO:0060402	7	0.310150	0.744586	0.773389	1.000000	1.000
247	GOBP_METAL_ION_TRANSPORT	GO:0030001	11	0.259740	0.740979	0.781116	1.000000	1.000
248	GOBP_MUSCLE_ADAPTATION	GO:0043500	5	0.355997	0.740367	0.808791	1.000000	1.000
249	GOBP_CALCIUM_ION_TRANSMEMBRANE_TRANSPORT	GO:0070588	7	0.310150	0.739938	0.780172	1.000000	1.000
250	GOBP_REGULATION_OF_IMMUNE_RESPONSE	GO:0050776	12	0.249068	0.738205	0.820000	1.000000	1.000
251	GOBP_PURINE_CONTAINING_COMPOUND_METABOLIC_PROCESS	GO:0072521	5	0.349788	0.737375	0.792123	1.000000	1.000
252	GOBP_CARBOHYDRATE_METABOLIC_PROCESS	GO:0005975	9	0.274287	0.735193	0.812095	1.000000	1.000
253	REACTOME_TRANSCRIPTIONAL_REGULATION_BY_TP53	R-RNO-3700989	6	0.319635	0.734475	0.797040	1.000000	1.000
254	GOBP_STRIATED_MUSCLE_CELL_DIFFERENTIATION	GO:0051146	9	0.276853	0.734353	0.803738	1.000000	1.000
255	GOBP_CELL_MORPHOGENESIS_INVOLVED_IN_NEURON_DIF...	GO:0048667	21	0.215358	0.734223	0.849462	1.000000	1.000

Continuation of Table **S17**

	NAME	ID	SIZE	ES	NES	NOM.p.val	FDR.q.val	FWER.p.val
256	GOBP_LONG_TERM_SYNAPTIC_POTENTIATION	GO:0060291	12	0.247401	0.731094	0.805621	1.000000	1.000
257	GOBP_RESPONSE_TO_UV	GO:0009411	5	0.337143	0.725460	0.792812	1.000000	1.000
258	GOBP_NEGATIVE_REGULATION_OF_PROTEIN_CATABOLIC_...	GO:0042177	5	0.337883	0.725332	0.808102	1.000000	1.000
259	GOBP_POSITIVE_REGULATION_OF_PROTEIN_SERINE_THR...	GO:0071902	9	0.272946	0.722368	0.813901	1.000000	1.000
260	GOBP_REGULATION_OF_NEURON_PROJECTION_DEVELOPMENT	GO:0010975	16	0.223822	0.722330	0.827354	1.000000	1.000
261	GOBP_EPITHELIAL_CELL_DIFFERENTIATION	GO:0030855	7	0.300703	0.719321	0.794372	1.000000	1.000
262	GOBP_PEPTIDE_SECRETION	GO:0002790	9	0.268923	0.718667	0.823529	1.000000	1.000
263	GOBP_BIOLOGICAL_ADHESION	GO:0022610	19	0.211763	0.715531	0.853881	1.000000	1.000
264	GOBP_PERIPHERAL_NERVOUS_SYSTEM_DEVELOPMENT	GO:0007422	9	0.265823	0.712362	0.815287	1.000000	1.000
265	GOBP_SMALL_MOLECULE_METABOLIC_PROCESS	GO:0044281	15	0.225565	0.710981	0.852495	1.000000	1.000
266	GOBP_POSITIVE_REGULATION_OF_NEURON_DEATH	GO:1901216	5	0.326391	0.708261	0.819302	1.000000	1.000
267	GOBP_MORPHOGENESIS_OF_AN_EPITHELIUM	GO:0002009	6	0.296444	0.703586	0.804671	1.000000	1.000
268	GOBP_PROTEIN_LOCALIZATION_TO_MEMBRANE	GO:0072657	14	0.223389	0.701269	0.855011	1.000000	1.000
269	GOBP_CELL_JUNCTION_ASSEMBLY	GO:0034329	8	0.268824	0.700649	0.843096	1.000000	1.000
270	GOBP_REGULATION_OF_PROTEIN_SERINE_THREONINE_KI...	GO:0071900	12	0.241542	0.700541	0.866667	1.000000	1.000
271	GOBP_SECOND_MESSENGER_MEDIATED_SIGNALING	GO:0019932	16	0.217178	0.697857	0.874419	1.000000	1.000
272	GOBP_SYNAPTIC_SIGNALING	GO:0099536	47	0.177273	0.696971	0.919668	1.000000	1.000
273	REACTOME_MAPK_TARGETS_NUCLEAR_EVENTS_MEDIATED_...	R-HSA-450282	5	0.320955	0.695698	0.859756	1.000000	1.000
274	REACTOME_TRANSCRIPTIONAL_REGULATION_OF_WHITE_A...	R-RNO-381340	6	0.298753	0.694437	0.852174	1.000000	1.000
275	GOBP_MONOCARBOXYLIC_ACID_METABOLIC_PROCESS	GO:0032787	6	0.301281	0.693398	0.853002	1.000000	1.000
276	GOBP_NEGATIVE_REGULATION_OF_MOLECULAR_FUNCTION	GO:0044092	24	0.194352	0.693343	0.891473	1.000000	1.000
277	GOBP_CELL_PART_MORPHOGENESIS	GO:0032990	24	0.192448	0.690071	0.895981	1.000000	1.000
278	GOBP_MEMORY	GO:0007613	13	0.233218	0.686438	0.854031	1.000000	1.000
279	GOBP_CELLULAR_COMPONENT_MORPHOGENESIS	GO:0032989	24	0.192448	0.685958	0.907767	1.000000	1.000
280	GOBP_PROTEIN_COMPLEX_OLIGOMERIZATION	GO:0051259	6	0.291032	0.683836	0.860927	1.000000	1.000
281	GOBP_REGULATION_OF_PEPTIDE_HORMONE_SECRETION	GO:0090276	6	0.296185	0.679374	0.876380	1.000000	1.000
282	GOBP_CELL_MORPHOGENESIS	GO:0000902	24	0.192448	0.678776	0.898058	1.000000	1.000
283	REACTOME_SIGNALING_BY_NTRKS	R-RNO-166520	18	0.211639	0.678661	0.899772	1.000000	1.000
284	GOBP_CELL_CELL_JUNCTION_ASSEMBLY	GO:0007043	5	0.308489	0.674775	0.847390	1.000000	1.000
285	REACTOME_SIGNALING_BY_RECEPTOR_TYROSINE_KINASES	R-RNO-9006934	26	0.184079	0.672300	0.923645	1.000000	1.000
286	REACTOME_NEUROTRANSMITTER_RECEPTORS_AND_POSTSY...	R-RNO-112314	22	0.190123	0.664551	0.923991	1.000000	1.000
287	GOBP_NEGATIVE_REGULATION_OF_CELLULAR_COMPONENT...	GO:0051129	11	0.233055	0.663433	0.899533	1.000000	1.000
288	GOBP_REGULATION_OF_DNA_BINDING_TRANSCRIPTION_F...	GO:0051090	16	0.211128	0.662605	0.901149	1.000000	1.000
289	GOBP_REGULATION_OF_CIRCADIAN_RHYTHM	GO:0042752	7	0.280875	0.662429	0.874715	1.000000	1.000
290	GOBP_CELL_CELL_JUNCTION_ORGANIZATION	GO:0045216	6	0.285936	0.657428	0.875789	1.000000	1.000
291	GOBP_REGULATION_OF_MAPK_CASCADE	GO:0043408	13	0.220571	0.656707	0.897092	0.999632	1.000
292	GOBP_ADENYLATE_CYCLASE_INHIBITING_G_PROTEIN_CO...	GO:0007193	9	0.242444	0.649845	0.903930	1.000000	1.000
293	GOBP_FC_EPSILON_RECEPTOR_SIGNALING_PATHWAY	GO:0038095	7	0.260042	0.648375	0.907757	1.000000	1.000
294	REACTOME_INTERFERON_SIGNALING	R-RNO-913531	6	0.286098	0.644584	0.899782	1.000000	1.000
295	GOBP_PROTEIN_LOCALIZATION_TO_CELL_JUNCTION	GO:1902414	6	0.280488	0.639035	0.900212	1.000000	1.000
296	REACTOME_ADAPTIVE_IMMUNE_SYSTEM	R-RNO-1280218	9	0.246677	0.637809	0.902335	1.000000	1.000
297	GOBP_IMMUNE_RESPONSE_REGULATING_SIGNALING_PATHWAY	GO:000276	8	0.244512	0.630564	0.924211	1.000000	1.000
298	REACTOME_FC_EPSILON_RECEPTOR_FCERI_SIGNALING	R-RNO-2454202	7	0.260042	0.625683	0.920705	1.000000	1.000

Continuation of Table **S17**

	NAME	ID	SIZE	ES	NES	NOM.p.val	FDR.q.val	FWER.p.val
299	GOBP_FC_RECEPTOR_SIGNALING_PATHWAY	GO:0038093	8	0.244512	0.623948	0.920705	1.000000	1.000
300	GOBP_PROTEIN_LOCALIZATION_TO_PLASMA_MEMBRANE	GO:0072659	7	0.245205	0.621636	0.935982	1.000000	1.000
301	GOBP_PROTEIN_LOCALIZATION_TO_POSTSYNAPSE	GO:0062237	5	0.277108	0.612834	0.927536	1.000000	1.000
302	GOBP_INSULIN_SECRETION	GO:0030073	7	0.247778	0.611511	0.920091	1.000000	1.000
303	GOBP_PEPTIDE_HORMONE_SECRETION	GO:0030072	7	0.247778	0.608272	0.942350	1.000000	1.000
304	GOBP_EXCITATORY_CHEMICAL_SYNAPTIC_TRANSMISSION	GO:0098976	5	0.277108	0.599552	0.934599	1.000000	1.000
305	REACTOME_DAG_AND_IP3_SIGNALING	R-RNO-1489509	8	0.234189	0.598181	0.946467	1.000000	1.000
306	GOBP_PROTEIN_LOCALIZATION_TO_SYNAPSE	GO:0035418	5	0.277108	0.594815	0.926882	1.000000	1.000
307	GOBP_REGULATION_OF_LIPID_LOCALIZATION	GO:1905952	5	0.266685	0.568277	0.941685	1.000000	1.000
308	GOBP_POSITIVE_REGULATION_OF_SIGNALING	GO:0023056	34	0.145338	0.563750	0.988263	1.000000	1.000
309	GOBP_REGULATION_OF_PROTEIN_BINDING	GO:0043393	7	0.229171	0.562036	0.956236	1.000000	1.000
310	GOBP_NEGATIVE_REGULATION_OF_ANION_TRANSPORT	GO:1903792	8	0.225000	0.557854	0.966316	1.000000	1.000
311	GOBP_NEGATIVE_REGULATION_OF_SYNAPTIC_TRANSMISSION	GO:0050805	6	0.231707	0.548428	0.981777	1.000000	1.000
312	GOBP_COGNITION	GO:0050890	28	0.146929	0.545219	0.990453	1.000000	1.000
313	REACTOME_LEISHMANIA_INFECTION	R-HSA-9658195	10	0.195544	0.537774	0.974359	1.000000	1.000
314	GOBP_NEGATIVE_REGULATION_OF_BINDING	GO:0051100	6	0.227526	0.529328	0.986900	1.000000	1.000
315	GOBP_DIVALENT_INORGANIC_CATION_HOMEOSTASIS	GO:0072507	13	0.175471	0.525989	0.974194	1.000000	1.000
316	GOBP_REGULATION_OF_CYTOSOLIC_CALCIUM_ION_CONCE...	GO:0051480	13	0.175471	0.524212	0.982456	1.000000	1.000
317	GOBP_RESPONSE_TO_MECHANICAL_STIMULUS	GO:0009612	6	0.219512	0.500502	0.993377	1.000000	1.000
318	REACTOME_PROTEIN_PROTEIN_INTERACTIONS_AT_SYNAPSES	R-RNO-6794362	12	0.167516	0.494912	0.988426	1.000000	1.000
319	GOBP_MUSCLE_CELL_DIFFERENTIATION	GO:0042692	10	0.179487	0.487547	0.989107	1.000000	1.000
320	GOBP_NEGATIVE_REGULATION_OF_KINASE_ACTIVITY	GO:0033673	5	0.223847	0.481315	0.991091	1.000000	1.000
321	GOBP_METAL_ION_HOMEOSTASIS	GO:0055065	14	0.157335	0.478386	0.993228	1.000000	1.000
322	GOBP_NEGATIVE_REGULATION_OF_TRANSFERASE_ACTIVITY	GO:0051348	5	0.223847	0.465631	0.993711	1.000000	1.000
323	GOBP_ORGANIC_ACID_METABOLIC_PROCESS	GO:0006082	7	0.187750	0.454007	0.995283	1.000000	1.000
324	GOBP_REGULATION_OF_NEURON_DIFFERENTIATION	GO:0045664	5	0.204819	0.433908	1.000000	1.000000	1.000
325	GOBP_PROTEIN_LOCALIZATION_TO_CELL_PERIPHERY	GO:1990778	12	0.139492	0.411755	1.000000	0.999098	1.000
326	GOBP_MALE_GAMETE_GENERATION	GO:0048232	5	-0.240964	-0.510617	0.992095	0.984988	1.000
327	GOBP_NEURON_DEVELOPMENT	GO:0048666	33	-0.145683	-0.515500	0.986087	0.986139	1.000
328	GOBP_CELL_PROJECTION_ORGANIZATION	GO:0030030	34	-0.143257	-0.519058	0.987784	0.987463	1.000
329	GOBP_POLYSACCHARIDE_METABOLIC_PROCESS	GO:0005976	5	-0.256169	-0.542382	0.969981	0.981309	1.000
330	HALLMARK_HYPOXIA	M5891	5	-0.257328	-0.545221	0.974903	0.982559	1.000
331	GOBP_RESPONSE_TO_LIGHT_STIMULUS	GO:0009416	11	-0.196439	-0.545893	0.970480	0.984803	1.000
332	GOBP_MULTI_MULTICELLULAR_ORGANISM_PROCESS	GO:0044706	8	-0.221115	-0.548046	0.967308	0.986452	1.000
333	GOBP_REGULATION_OF_SYSTEM_PROCESS	GO:0044057	18	-0.174105	-0.548478	0.962393	0.988816	1.000
334	GOBP_REGULATION_OF_AXONOGENESIS	GO:0050770	7	-0.227215	-0.550781	0.965116	0.990367	1.000
335	GOBP_ENDOCYTOSIS	GO:0006897	8	-0.223243	-0.552687	0.968045	0.992057	1.000
336	HALLMARK_UV_RESPONSE_UP	M5941	5	-0.267794	-0.564542	0.960073	0.988595	1.000
337	REACTOME_NEUREXINS_AND_NEUROLIGINS	R-RNO-6794361	9	-0.219053	-0.567618	0.954710	0.989538	1.000
338	GOBP_EPHRIN_RECEPTOR_SIGNALING_PATHWAY	GO:0048013	5	-0.265060	-0.570726	0.955473	0.990339	1.000
339	GOBP_NUCLEOBASE_CONTAINING_SMALL_MOLECULE_META...	GO:0055086	6	-0.254952	-0.573318	0.966797	0.991559	1.000
340	GOBP_RESPONSE_TO_METAL_ION	GO:0010038	15	-0.191781	-0.574399	0.939068	0.993505	1.000
341	GOBP_GAMETE_GENERATION	GO:0007276	9	-0.217731	-0.575077	0.939068	0.995754	1.000

Continuation of Table **S17**

	NAME	ID	SIZE	ES	NES	NOM.p.val	FDR.q.val	FWER.p.val
342	GOBP_REGULATION_OF_CARBOHYDRATE_BIOSYNTHETIC_P...	GO:0043255	5	-0.275296	-0.575165	0.948718	0.998348	1.000
343	GOBP_HORMONE_TRANSPORT	GO:0009914	9	-0.224620	-0.578500	0.957295	0.999134	1.000
344	GOBP_ADENYLATE_CYCLASE_MODULATING_G_PROTEIN_CO...	GO:0007188	11	-0.214345	-0.581683	0.945355	0.999870	1.000
345	GOBP_POSITIVE_REGULATION_OF_PROTEOLYSIS	GO:0045862	8	-0.234908	-0.587582	0.940972	0.998535	1.000
346	GOBP_CARBOHYDRATE_BIOSYNTHETIC_PROCESS	GO:0016051	5	-0.275296	-0.589868	0.960967	0.999773	1.000
347	GOBP_REGULATION_OF_CARBOHYDRATE_METABOLIC_PROCESS	GO:0006109	5	-0.275296	-0.590619	0.945055	1.000000	1.000
348	GOBP_REGULATION_OF_PROTEIN_LOCALIZATION_TO_MEM...	GO:1905475	7	-0.246606	-0.604782	0.927619	0.995013	1.000
349	GOBP_CELLULAR_RESPONSE_TO_INORGANIC_SUBSTANCE	GO:0071241	11	-0.220897	-0.604914	0.940270	0.997609	1.000
350	REACTOME_TRAFFICKING_OF_AMPA_RECEPTORS	R-RNO-399719	11	-0.222052	-0.607773	0.927239	0.998209	1.000
351	GOBP_APOPTOTIC_MITOCHONDRIAL_CHANGES	GO:0008637	6	-0.272970	-0.609014	0.942272	0.999959	1.000
352	GOBP_CELL_CELL_SIGNALING	GO:0007267	56	-0.167222	-0.615451	0.950931	0.997605	1.000
353	GOBP_ENDOCRINE_SYSTEM_DEVELOPMENT	GO:0035270	6	-0.273646	-0.615759	0.932961	1.000000	1.000
354	REACTOME_EPH_EPHRIN_SIGNALING	R-RNO-2682334	6	-0.268293	-0.616092	0.939394	1.000000	1.000
355	GOBP_NEGATIVE_REGULATION_OF_LIPID_METABOLIC_PR...	GO:0045833	5	-0.291155	-0.620533	0.907197	1.000000	1.000
356	REACTOME_INTERLEUKIN_17_SIGNALING	R-RNO-448424	6	-0.275947	-0.623455	0.913556	1.000000	1.000
357	GOBP_CELL_CELL_ADHESION	GO:0098609	14	-0.210742	-0.629030	0.931699	0.999636	1.000
358	GOBP_POSITIVE_REGULATION_OF_CYSTEINE_TYPE_ENDO...	GO:2001056	7	-0.266783	-0.630817	0.918330	1.000000	1.000
359	GOBP_G_PROTEIN_COUPLED_RECEPTOR_SIGNALING_PATHWAY	GO:0007186	18	-0.200818	-0.638090	0.924028	0.997283	1.000
360	GOBP_REGULATION_OF_CELL_CYCLE_PROCESS	GO:0010564	5	-0.303711	-0.638338	0.936210	0.999801	1.000
361	GOBP_REGULATION_OF_ANATOMICAL_STRUCTURE_SIZE	GO:0090066	10	-0.244465	-0.640823	0.897727	1.000000	1.000
362	GOBP_POSITIVE_REGULATION_OF_PEPTIDASE_ACTIVITY	GO:0010952	7	-0.266783	-0.644383	0.902574	0.999815	1.000
363	GOBP_MUSCLE_STRUCTURE_DEVELOPMENT	GO:0061061	16	-0.216015	-0.648015	0.911032	0.999087	1.000
364	GOBP_NEGATIVE_REGULATION_OF_TRANSMEMBRANE_TRAN...	GO:0034763	6	-0.285851	-0.649824	0.903808	1.000000	1.000
365	GOBP_CHEMICAL_HOMEOSTASIS	GO:0048878	23	-0.198177	-0.652738	0.922414	0.999773	1.000
366	GOBP_RESPONSE_TO_DRUG	GO:0042493	11	-0.233766	-0.652795	0.906643	1.000000	1.000
367	GOBP_RESPONSE_TO_INORGANIC_SUBSTANCE	GO:0010035	19	-0.202899	-0.653263	0.908612	1.000000	1.000
368	REACTOME_TRANSMISSION_ACROSS_CHEMICAL_SYNAPSES	R-RNO-112315	23	-0.193982	-0.653524	0.922242	1.000000	1.000
369	GOBP_CELL_CELL_SIGNALING_BY_WNT	GO:0198738	7	-0.271004	-0.656767	0.904936	1.000000	1.000
370	GOBP_REGULATION_OF_HORMONE_SECRETION	GO:0046883	8	-0.264162	-0.658798	0.893657	1.000000	1.000
371	GOBP_PROTEIN_DEPHOSPHORYLATION	GO:0006470	7	-0.279892	-0.665068	0.878277	1.000000	1.000
372	GOBP_BEHAVIOR	GO:0007610	32	-0.188070	-0.667340	0.911519	1.000000	1.000
373	REACTOME_DISEASES_OF_SIGNAL_TRANSDUCTION_BY_GR...	R-HSA-5663202	12	-0.241488	-0.669008	0.882662	1.000000	1.000
374	GOBP_DEPHOSPHORYLATION	GO:0016311	7	-0.279892	-0.678107	0.869403	0.997637	1.000
375	GOBP_POSITIVE_REGULATION_OF_DEFENSE_RESPONSE	GO:0031349	5	-0.323706	-0.679219	0.890411	0.999183	1.000
376	GOBP_REGULATION_OF_TRANS_SYNAPTIC_SIGNALING	GO:0099177	40	-0.187856	-0.680931	0.925203	0.999987	1.000
377	GOBP_NEGATIVE_REGULATION_OF_INTRACELLULAR_SIGN...	GO:1902532	7	-0.283354	-0.684168	0.856884	0.999094	1.000
378	GOBP_REGULATION_OF_FAT_CELL_DIFFERENTIATION	GO:0045598	6	-0.307407	-0.694871	0.860735	0.988479	1.000
379	REACTOME_CELLULAR_RESPONSES_TO_EXTERNAL_STIMULI	R-RNO-8953897	12	-0.244958	-0.699014	0.864865	0.985939	1.000
380	GOBP_CELLULAR_CARBOHYDRATE_METABOLIC_PROCESS	GO:0044262	5	-0.330969	-0.699395	0.852612	0.988299	1.000
381	REACTOME_INTEGRATION_OF_ENERGY_METABOLISM	R-RNO-163685	5	-0.328368	-0.704236	0.835644	0.984873	1.000
382	GOBP_CELLULAR_RESPONSE_TO_EXTRACELLULAR_STIMULUS	GO:0031668	8	-0.285972	-0.704882	0.850000	0.986901	1.000
383	GOBP_GROWTH	GO:0040007	14	-0.245664	-0.708034	0.840951	0.985658	1.000
384	GOBP_POSITIVE_REGULATION_OF_AXONOGENESIS	GO:0050772	5	-0.335168	-0.712317	0.825832	0.982795	1.000

Continuation of Table **S17**

	NAME	ID	SIZE	ES	NES	NOM.p.val	FDR.q.val	FWER.p.val
385	GOBP_ORGAN_GROWTH	GO:0035265	5	-0.335429	-0.715125	0.859073	0.981899	1.000
386	GOBP_CELLULAR_MACROMOLECULE_LOCALIZATION	GO:0070727	21	-0.224746	-0.720497	0.846939	0.977051	1.000
387	GOBP_NEGATIVE_REGULATION_OF_NERVOUS_SYSTEM_DEV...	GO:0051961	5	-0.329935	-0.721797	0.834601	0.978258	1.000
388	GOBP_NEUROTROPHIN_TRK_RECEPTOR_SIGNALING_PATHWAY	GO:0048011	6	-0.319117	-0.728551	0.843212	0.971647	1.000
389	GOBP_NEUROTROPHIN_SIGNALING_PATHWAY	GO:0038179	6	-0.319117	-0.728908	0.833984	0.974096	1.000
390	GOBP_REGULATION_OF_CELL_ADHESION	GO:0030155	13	-0.249013	-0.731353	0.829907	0.973608	1.000
391	REACTOME_CIRCADIAN_CLOCK	R-HSA-400253	5	-0.337444	-0.736192	0.796610	0.969516	1.000
392	GOBP_REGULATION_OF_ORGAN_GROWTH	GO:0046620	5	-0.335429	-0.736608	0.805714	0.971860	1.000
393	GOBP_REGULATION_OF_PROTEIN_PHOSPHORYLATION	GO:0001932	26	-0.217357	-0.739541	0.819700	0.970385	1.000
394	GOBP_REGULATION_OF_LEUKOCYTE_DIFFERENTIATION	GO:1902105	9	-0.286388	-0.741212	0.789773	0.970786	1.000
395	GOBP_NEGATIVE_REGULATION_OF_SIGNALING	GO:0023057	21	-0.229914	-0.741306	0.808970	0.973633	1.000
396	GOBP_ACTIN_FILAMENT_BASED_PROCESS	GO:0030029	7	-0.307528	-0.743940	0.790566	0.972400	1.000
397	GOBP_POSITIVE_REGULATION_OF_CELL_DEATH	GO:0010942	19	-0.237814	-0.744352	0.799648	0.974813	1.000
398	GOBP_NEUROGENESIS	GO:0022008	40	-0.201593	-0.745815	0.848185	0.975510	1.000
399	GOBP_TUBE_MORPHOGENESIS	GO:0035239	15	-0.250059	-0.749656	0.781818	0.972355	1.000
400	GOBP_NERVOUS_SYSTEM_PROCESS	GO:0050877	38	-0.204811	-0.752788	0.831034	0.970425	1.000
401	GOBP_CELLULAR_ION_HOMEOSTASIS	GO:0006873	14	-0.255505	-0.753119	0.809074	0.972966	1.000
402	REACTOME_DEATH_RECEPTOR_SIGNALLING	R-RNO-73887	5	-0.350561	-0.753531	0.774436	0.975423	1.000
403	GOBP_CELLULAR_RESPONSE_TO_EXTERNAL_STIMULUS	GO:0071496	10	-0.284928	-0.755885	0.766727	0.974839	1.000
404	GOBP_MITOTIC_CELL_CYCLE	GO:0000278	10	-0.283106	-0.757317	0.779130	0.975492	1.000
405	GOBP_REGULATION_OF_WNT_SIGNALING_PATHWAY	GO:0030111	5	-0.355168	-0.757418	0.756007	0.978465	1.000
406	GOBP_LOCOMOTION	GO:0040011	31	-0.214758	-0.758474	0.810858	0.979906	1.000
407	GOBP_POSITIVE_REGULATION_OF_CELL_DIFFERENTIATION	GO:0045597	27	-0.218191	-0.758597	0.812825	0.982837	1.000
408	GOBP_REGULATION_OF_GROWTH	GO:0040008	13	-0.263334	-0.758766	0.788194	0.985694	1.000
409	GOBP_RESPONSE_TO_ALCOHOL	GO:0097305	11	-0.272727	-0.759184	0.763251	0.988147	1.000
410	GOBP_CYTOKINE_MEDIATED_SIGNALING_PATHWAY	GO:0019221	16	-0.250105	-0.766793	0.769674	0.978708	1.000
411	GOBP_GLUTAMATE_RECEPTOR_SIGNALING_PATHWAY	GO:0007215	17	-0.249207	-0.766885	0.801038	0.981711	1.000
412	GOBP_NEGATIVE_REGULATION_OF_CYSTEINE_TYPE_ENDO...	GO:2000117	5	-0.353347	-0.767754	0.760536	0.983480	1.000
413	GOBP_TISSUE_MORPHOGENESIS	GO:0048729	7	-0.316556	-0.768656	0.802607	0.985115	1.000
414	GOBP_ORGANONITROGEN_COMPOUND_BIOSYNTHETIC_PROCESS	GO:1901566	13	-0.270174	-0.768825	0.779751	0.988051	1.000
415	GOBP_CELL_CYCLE_PROCESS	GO:0022402	11	-0.285995	-0.769204	0.756708	0.990583	1.000
416	REACTOME_ESTROGEN_DEPENDENT_NUCLEAR_EVENTS_DOW...	R-RNO-9634638	5	-0.367007	-0.769814	0.754128	0.992726	1.000
417	GOBP_REGULATION_OF_SYNAPTIC_PLASTICITY	GO:0048167	21	-0.235194	-0.772035	0.754685	0.992286	1.000
418	GOBP_REGULATION_OF_MYELOID_LEUKOCYTE_DIFFERENT...	GO:0002761	8	-0.302660	-0.775109	0.759013	0.990186	1.000
419	GOBP_RESPONSE_TO_RADIATION	GO:0009314	13	-0.272304	-0.777141	0.740143	0.989798	1.000
420	GOBP_REGULATION_OF_CYTOSKELETON_ORGANIZATION	GO:0051493	5	-0.366054	-0.777897	0.749035	0.991889	1.000
421	GOBP_REGULATION_OF_CYSTEINE_TYPE_ENDOPEPTIDASE...	GO:2000116	9	-0.301265	-0.781742	0.757634	0.988311	1.000
422	GOBP_REGULATION_OF_MUSCLE_SYSTEM_PROCESS	GO:0090257	8	-0.316917	-0.787403	0.714795	0.981270	1.000
423	GOBP_NERVE_DEVELOPMENT	GO:0021675	9	-0.305370	-0.791670	0.724907	0.976929	1.000
424	GOBP_BLOOD_VESSEL_MORPHOGENESIS	GO:0048514	14	-0.272567	-0.792021	0.739895	0.979529	1.000
425	GOBP_REGULATION_OF_PROTEIN_LOCALIZATION	GO:0032880	18	-0.251046	-0.792461	0.732472	0.982133	1.000
426	GOBP_RESPONSE_TO_STARVATION	GO:0042594	5	-0.377560	-0.794471	0.736842	0.981754	1.000
427	GOBP_POSITIVE_REGULATION_OF_PRI_MIRNA_TRANSCRI...	GO:1902895	7	-0.344199	-0.796278	0.705996	0.981868	1.000

Continuation of Table **S17**

	NAME	ID	SIZE	ES	NES	NOM.p.val	FDR.q.val	FWER.p.val
428	GOBP_TEMPERATURE_HOMEOSTASIS	GO:0001659	5	-0.381414	-0.797433	0.706204	0.983237	1.000
429	GOBP_CENTRAL_NERVOUS_SYSTEM_DEVELOPMENT	GO:0007417	26	-0.235665	-0.797927	0.746219	0.985702	1.000
430	HALLMARK_KRAS_SIGNALING_UP	M5953	6	-0.351981	-0.799536	0.729679	0.986129	1.000
431	GOBP_GLAND_DEVELOPMENT	GO:0048732	7	-0.334184	-0.805072	0.684615	0.979153	1.000
432	GOBP_REGULATION_OF_PROTEOLYSIS	GO:0030162	12	-0.283622	-0.806262	0.740608	0.980184	1.000
433	GOBP_RESPONSE_TO_CALCIUM_ION	GO:0051592	6	-0.365854	-0.806709	0.667300	0.982781	1.000
434	GOBP_FAT_CELL_DIFFERENTIATION	GO:0045444	9	-0.317881	-0.810416	0.705882	0.979299	1.000
435	GOBP_RESPONSE_TO_HEAT	GO:0009408	7	-0.344344	-0.810717	0.696798	0.982136	1.000
436	GOBP_POSITIVE_REGULATION_OF_TRANSPORT	GO:0051050	26	-0.239038	-0.811073	0.747826	0.984882	1.000
437	GOBP_REGULATION_OF_CELL_DEATH	GO:0010941	34	-0.226950	-0.811999	0.752212	0.986512	1.000
438	GOBP_NEGATIVE_REGULATION_OF_CELL_CYCLE	GO:0045786	5	-0.381268	-0.819739	0.669246	0.975192	1.000
439	GOBP_REGULATION_OF_CELLULAR_LOCALIZATION	GO:0060341	17	-0.266993	-0.820239	0.719852	0.977604	1.000
440	GOBP_NEGATIVE_REGULATION_OF_PHOSPHORYLATION	GO:0042326	7	-0.342878	-0.821338	0.659615	0.978906	1.000
441	GOBP_POSITIVE_REGULATION_OF_PROTEIN_MODIFICATI...	GO:0031401	23	-0.251991	-0.821441	0.746317	0.982211	1.000
442	GOBP_POSITIVE_REGULATION_OF_PHOSPHORUS_METABOL...	GO:0010562	24	-0.244858	-0.821718	0.731922	0.985244	1.000
443	GOBP_PRI_MIRNA_TRANSCRIPTION_BY_RNA_POLYMERASE_II	GO:0061614	7	-0.344199	-0.822491	0.661654	0.987379	1.000
444	GOBP_AXON_DEVELOPMENT	GO:0061564	18	-0.259866	-0.822546	0.737030	0.990842	1.000
445	GOBP_REGULATION_OF_MYELOID_CELL_DIFFERENTIATION	GO:0045637	9	-0.318119	-0.826042	0.675373	0.987472	1.000
446	GOBP_CELLULAR_RESPONSE_TO_STARVATION	GO:0009267	5	-0.377560	-0.826344	0.670565	0.990525	1.000
447	GOBP_NEGATIVE_REGULATION_OF_ION_TRANSPORT	GO:0043271	11	-0.298889	-0.828319	0.703846	0.990259	1.000
448	GOBP_NEURON_APOPTOTIC_PROCESS	GO:0051402	12	-0.296602	-0.829549	0.680851	0.991302	1.000
449	GOBP_CELLULAR_RESPONSE_TO_KETONE	GO:1901655	5	-0.393422	-0.833599	0.656075	0.986883	1.000
450	REACTOME_G_ALPHA_I_SIGNALLING_EVENTS	R-RNO-418594	18	-0.266325	-0.833792	0.696270	0.990121	1.000
451	GOBP_REGULATION_OF_SMALL_MOLECULE_METABOLIC_PR...	GO:0062012	9	-0.326279	-0.834213	0.681481	0.993030	1.000
452	REACTOME_NEURONAL_SYSTEM	R-RNO-112316	26	-0.246910	-0.834586	0.700717	0.996046	1.000
453	GOBP_AMIDE_BIOSYNTHETIC_PROCESS	GO:0043604	7	-0.359650	-0.836378	0.651837	0.996060	1.000
454	GOBP_POSITIVE_REGULATION_OF_EPITHELIAL_CELL_PR...	GO:0050679	7	-0.351124	-0.837435	0.657356	0.997702	1.000
455	REACTOME_INNATE_IMMUNE_SYSTEM	R-RNO-168249	16	-0.282102	-0.839177	0.661202	0.997616	1.000
456	GOBP_NEGATIVE_REGULATION_OF_TRANSCRIPTION_BY_R...	GO:0000122	10	-0.323154	-0.850224	0.677596	0.978910	1.000
457	GOBP_REGULATION_OF_CELL_CYCLE	GO:0051726	13	-0.295029	-0.852917	0.648746	0.976855	1.000
458	GOBP_POSITIVE_REGULATION_OF_PROTEIN_PHOSPHORYL...	GO:0001934	23	-0.251991	-0.852972	0.678261	0.980478	1.000
459	GOBP_REGULATION_OF_PEPTIDE_TRANSPORT	GO:0090087	10	-0.321012	-0.856704	0.625709	0.976337	1.000
460	GOBP_REGULATION_OF_CELL_CELL_ADHESION	GO:0022407	9	-0.335140	-0.862699	0.622430	0.967519	1.000
461	GOBP_CELLULAR_HOMEOSTASIS	GO:0019725	18	-0.268978	-0.863256	0.642218	0.970059	1.000
462	GOBP_REGULATION_OF_CELLULAR_PROTEIN_LOCALIZATION	GO:1903827	12	-0.303139	-0.864231	0.631776	0.971709	1.000
463	GOBP_PEPTIDE_BIOSYNTHETIC_PROCESS	GO:0043043	7	-0.359650	-0.866282	0.632114	0.971149	1.000
464	REACTOME_SIGNALING_BY_NUCLEAR_RECEPTORS	R-RNO-9006931	8	-0.354072	-0.869335	0.626894	0.968392	1.000
465	GOBP_NEURON_MIGRATION	GO:0001764	5	-0.410807	-0.871054	0.614943	0.968471	1.000
466	GOBP_CELLULAR_RESPONSE_TO_PEPTIDE_HORMONE_STIM...	GO:0071375	9	-0.338317	-0.871283	0.627737	0.971786	1.000
467	GOBP_HEAD_DEVELOPMENT	GO:0060322	22	-0.268655	-0.872931	0.641667	0.971999	1.000
468	GOBP_POSITIVE_REGULATION_OF_PROTEIN_METABOLIC_...	GO:0051247	28	-0.250585	-0.873654	0.650602	0.974098	1.000
469	GOBP_RESPONSE_TO_NICOTINE	GO:0035094	7	-0.365317	-0.875416	0.629496	0.974039	1.000
470	GOBP_PROTEIN_PHOSPHORYLATION	GO:0006468	34	-0.248383	-0.876064	0.666667	0.976588	1.000

Continuation of Table **S17**

	NAME	ID	SIZE	ES	NES	NOM.p.val	FDR.q.val	FWER.p.val
471	REACTOME_ESR_MEDIATED_SIGNALING	R-RNO-8939211	8	-0.354072	-0.876575	0.631485	0.979360	1.000
472	GOBP_NEGATIVE_REGULATION_OF_NEURON_APOPTOTIC_P...	GO:0043524	8	-0.351938	-0.877826	0.637383	0.980623	1.000
473	GOBP_EMBRYONIC_ORGAN_DEVELOPMENT	GO:0048568	7	-0.365075	-0.877952	0.618147	0.984399	1.000
474	GOBP_CYTOSKELETON_ORGANIZATION	GO:0007010	11	-0.317989	-0.878412	0.634409	0.987372	1.000
475	GOBP_RESPONSE_TO_KETONE	GO:1901654	7	-0.370370	-0.879927	0.605948	0.988111	1.000
476	GOBP_TRANSMEMBRANE_RECEPTOR_PROTEIN_TYROSINE_K...	GO:0007169	19	-0.276588	-0.880858	0.626736	0.990137	1.000
477	GOBP_REGULATION_OF_HYDROLASE_ACTIVITY	GO:0051336	22	-0.265347	-0.881264	0.639860	0.993278	1.000
478	GOBP_MATERNAL_PROCESS_INVOLVED_IN_FEMALE_PREGN...	GO:0060135	5	-0.408336	-0.881512	0.608949	0.996902	1.000
479	GOBP_CELLULAR_RESPONSE_TO_ALCOHOL	GO:0097306	5	-0.412911	-0.881996	0.605948	1.000000	1.000
480	REACTOME_L1CAM_INTERACTIONS	R-RNO-373760	5	-0.419935	-0.882346	0.609037	1.000000	1.000
481	GOBP_MUSCLE_SYSTEM_PROCESS	GO:0003012	9	-0.333340	-0.882634	0.592593	1.000000	1.000
482	GOBP_FOREBRAIN_DEVELOPMENT	GO:0030900	9	-0.348172	-0.882935	0.603571	1.000000	1.000
483	GOBP_NEGATIVE_REGULATION_OF_IMMUNE_SYSTEM_PROCESS	GO:0002683	5	-0.418955	-0.885651	0.634387	1.000000	1.000
484	GOBP_LIPID_LOCALIZATION	GO:0010876	10	-0.332354	-0.888362	0.608939	1.000000	1.000
485	GOBP_LIPID_BIOSYNTHETIC_PROCESS	GO:0008610	6	-0.388824	-0.889669	0.585687	1.000000	1.000
486	REACTOME_OPIOID_SIGNALLING	R-RNO-111885	12	-0.316882	-0.890202	0.615809	1.000000	1.000
487	GOBP_REGULATION_OF_LIPID_BIOSYNTHETIC_PROCESS	GO:0046890	6	-0.388824	-0.891199	0.608696	1.000000	1.000
488	GOBP_REGULATION_OF_LIPID_METABOLIC_PROCESS	GO:0019216	7	-0.366724	-0.891576	0.606589	1.000000	1.000
489	GOBP_RESPONSE_TO_EXTRACELLULAR_STIMULUS	GO:0009991	13	-0.308974	-0.899458	0.571691	1.000000	1.000
490	GOBP_POSITIVE_REGULATION_OF_IMMUNE_RESPONSE	GO:0050778	9	-0.351547	-0.899844	0.578067	1.000000	1.000
491	GOBP_REGULATION_OF_APOPTOTIC_SIGNALING_PATHWAY	GO:2001233	10	-0.334615	-0.901610	0.589416	1.000000	1.000
492	GOBP_REGULATION_OF_PROTEIN_STABILITY	GO:0031647	5	-0.418211	-0.902545	0.574040	1.000000	1.000
493	GOBP_REGULATION_OF_MULTICELLULAR_ORGANISMAL_DE...	GO:2000026	31	-0.257397	-0.906882	0.599343	1.000000	1.000
494	GOBP_REGULATION_OF_CELL_DIFFERENTIATION	GO:0045595	36	-0.251371	-0.906986	0.611667	1.000000	1.000
495	GOBP_REGULATION_OF_ION_TRANSPORT	GO:0043269	36	-0.256301	-0.911642	0.585586	0.999507	1.000
496	GOBP_CIRCULATORY_SYSTEM_PROCESS	GO:0003013	5	-0.434575	-0.912002	0.579048	1.000000	1.000
497	REACTOME_RNA_POLYMERASE_II_TRANSCRIPTION	R-RNO-73857	20	-0.281754	-0.912620	0.588441	1.000000	1.000
498	GOBP_HOMEOSTASIS_OF_NUMBER_OF_CELLS	GO:0048872	5	-0.441927	-0.912791	0.541267	1.000000	1.000
499	GOBP_RESPONSE_TO_OXYGEN_LEVELS	GO:0070482	8	-0.359091	-0.913025	0.593284	1.000000	1.000
500	GOBP_NEGATIVE_REGULATION_OF_NEURON_DEATH	GO:1901215	11	-0.333774	-0.913181	0.566794	1.000000	1.000
501	GOBP_REGULATION_OF_HEMOPOIESIS	GO:1903706	10	-0.336589	-0.913780	0.573386	1.000000	1.000
502	GOBP_MEMBRANE_ORGANIZATION	GO:0061024	12	-0.328512	-0.916532	0.559322	1.000000	1.000
503	GOBP_AGING	GO:0007568	13	-0.314324	-0.917559	0.572183	1.000000	1.000
504	GOBP_REGULATION_OF_IMMUNE_SYSTEM_PROCESS	GO:0002682	21	-0.281532	-0.918504	0.568995	1.000000	1.000
505	GOBP_MUSCLE_CELL_PROLIFERATION	GO:0033002	8	-0.360822	-0.919022	0.530055	1.000000	1.000
506	GOBP_CELL_CYCLE	GO:0007049	21	-0.287419	-0.923409	0.592721	1.000000	1.000
507	GOBP_SEXUAL_REPRODUCTION	GO:0019953	10	-0.343185	-0.923677	0.549719	1.000000	1.000
508	GOBP_NEGATIVE_REGULATION_OF_CELL_PROJECTION_OR...	GO:0031345	5	-0.429093	-0.926137	0.536680	1.000000	1.000
509	GOBP_NEGATIVE_REGULATION_OF_TRANSPORT	GO:0051051	13	-0.319679	-0.926790	0.555957	1.000000	1.000
510	GOBP_TAXIS	GO:0042330	14	-0.313669	-0.930418	0.528029	1.000000	1.000
511	GOBP_NEGATIVE_REGULATION_OF_PROTEIN_METABOLIC_...	GO:0051248	20	-0.292818	-0.932154	0.570690	1.000000	1.000
512	GOBP_REGULATION_OF_LYMPHOCYTE_ACTIVATION	GO:0051249	5	-0.443989	-0.933993	0.523232	1.000000	1.000
513	GOBP_TISSUE_MIGRATION	GO:0090130	9	-0.368271	-0.938072	0.529740	1.000000	1.000

Continuation of Table **S17**

	NAME	ID	SIZE	ES	NES	NOM.p.val	FDR.q.val	FWER.p.val
514	GOBP_NEGATIVE_REGULATION_OF_BIOSYNTHETIC_PROCESS	GO:0009890	13	-0.324267	-0.938104	0.522769	1.000000	1.000
515	GOBP_REGULATION_OF_CELLULAR_AMIDE_METABOLIC_PR...	GO:0034248	9	-0.356078	-0.939209	0.539823	1.000000	1.000
516	GOBP_NEURON_DIFFERENTIATION	GO:0030182	36	-0.260207	-0.941202	0.553691	1.000000	1.000
517	GOBP_MULTICELLULAR_ORGANISMAL_HOMEOSTASIS	GO:0048871	12	-0.328038	-0.945827	0.526882	1.000000	1.000
518	GOBP_CELLULAR_RESPONSE_TO_CADMIUM_ION	GO:0071276	5	-0.439672	-0.946908	0.525714	1.000000	1.000
519	GOBP_MITOCHONDRION_ORGANIZATION	GO:0007005	7	-0.400982	-0.947426	0.493433	1.000000	1.000
520	GOBP_ESTABLISHMENT_OF_PROTEIN_LOCALIZATION	GO:0045184	18	-0.302140	-0.949733	0.520761	1.000000	1.000
521	REACTOME_HEMOSTASIS	R-RNO-109582	14	-0.319070	-0.953966	0.509338	1.000000	1.000
522	GOBP_REGULATION_OF_NEURONAL_SYNAPTIC_PLASTICITY	GO:0048168	7	-0.396569	-0.955004	0.491651	1.000000	1.000
523	REACTOME_SIGNALING_BY_GPCR	R-RNO-372790	22	-0.293603	-0.959340	0.514580	1.000000	1.000
524	GOBP_PEPTIDYL_SERINE_MODIFICATION	GO:0018209	12	-0.343046	-0.960068	0.477860	1.000000	1.000
525	GOBP_RESPONSE_TO_ABIOTIC_STIMULUS	GO:0009628	27	-0.279535	-0.962076	0.505357	1.000000	1.000
526	GOBP_POSITIVE_REGULATION_OF_ESTABLISHMENT_OF_P...	GO:1904951	5	-0.459867	-0.965862	0.500000	1.000000	1.000
527	GOBP_RESPONSE_TO_ALKALOID	GO:0043279	5	-0.461792	-0.966374	0.501859	1.000000	1.000
528	GOBP_POSITIVE_REGULATION_OF_EPITHELIAL_CELL_MI...	GO:0010634	5	-0.444285	-0.969285	0.482176	1.000000	1.000
529	GOBP_REGULATION_OF_PHOSPHORUS_METABOLIC_PROCESS	GO:0051174	27	-0.280212	-0.969288	0.494585	1.000000	1.000
530	GOBP_RESPIRATORY_SYSTEM_DEVELOPMENT	GO:0060541	6	-0.425662	-0.970452	0.480151	1.000000	1.000
531	REACTOME_TRAFFICKING_OF_GLUR2_CONTAINING_AMPA_...	R-RNO-416993	8	-0.389211	-0.970595	0.503846	1.000000	1.000
532	GOBP_RESPONSE_TO_TUMOR_NECROSIS_FACTOR	GO:0034612	7	-0.416564	-0.971300	0.485437	1.000000	1.000
533	GOBP_MUSCLE_TISSUE_DEVELOPMENT	GO:0060537	11	-0.349242	-0.971709	0.488055	1.000000	1.000
534	GOBP_REGULATION_OF_HORMONE_LEVELS	GO:0010817	12	-0.344315	-0.971828	0.486289	1.000000	1.000
535	HALLMARK_ALLOGRAFT_REJECTION	M5950	7	-0.408881	-0.972166	0.495238	1.000000	1.000
536	GOBP_CELLULAR_COMPONENT_MAINTENANCE	GO:0043954	5	-0.450586	-0.973630	0.486339	1.000000	1.000
537	GOBP_MONOAMINE_TRANSPORT	GO:0015844	5	-0.455045	-0.976931	0.490458	1.000000	1.000
538	GOBP_REGULATION_OF_VESICLE_MEDIATED_TRANSPORT	GO:0060627	12	-0.343768	-0.978932	0.489484	1.000000	1.000
539	GOBP_NEGATIVE_REGULATION_OF_PHOSPHORUS_METABOL...	GO:0010563	9	-0.378151	-0.983265	0.450835	1.000000	1.000
540	GOBP_ESTABLISHMENT_OF_PROTEIN_LOCALIZATION_TO_...	GO:0072594	6	-0.424945	-0.983634	0.445087	1.000000	1.000
541	GOBP_NEGATIVE_REGULATION_OF_CELL_DEATH	GO:0060548	22	-0.293635	-0.984654	0.498246	1.000000	1.000
542	GOBP_SKIN_DEVELOPMENT	GO:0043588	5	-0.455807	-0.985693	0.464627	1.000000	1.000
543	GOBP_INTRINSIC_APOPTOTIC_SIGNALING_PATHWAY	GO:0097193	5	-0.474233	-0.986493	0.480151	1.000000	1.000
544	GOBP_NEGATIVE_REGULATION_OF_HYDROLASE_ACTIVITY	GO:0051346	11	-0.353201	-0.986570	0.468806	1.000000	1.000
545	GOBP_REGULATION_OF_SECRETION	GO:0051046	14	-0.332096	-0.987147	0.473776	1.000000	1.000
546	GOBP_POSITIVE_REGULATION_OF_SECRETION	GO:0051047	6	-0.437681	-0.988090	0.462121	1.000000	1.000
547	GOBP_PROTEIN_AUTOPHOSPHORYLATION	GO:0046777	5	-0.466222	-0.988456	0.459259	1.000000	1.000
548	GOBP_LIPID_EXPORT_FROM_CELL	GO:0140353	5	-0.455096	-0.989409	0.452865	1.000000	1.000
549	GOBP_NEGATIVE_REGULATION_OF_NUCLEOBASE_CONTAIN...	GO:0045934	13	-0.340262	-0.989511	0.448276	1.000000	1.000
550	GOBP_PROTEIN_LOCALIZATION_TO_ORGANELLE	GO:0033365	9	-0.378395	-0.990193	0.449458	1.000000	1.000
551	GOBP_NEGATIVE_REGULATION_OF_PROTEIN_MODIFICATI...	GO:0031400	10	-0.372822	-0.990643	0.480874	1.000000	1.000
552	REACTOME_ONCOGENIC_MAPK_SIGNALING	R-HSA-6802957	5	-0.465631	-1.000876	0.457295	1.000000	1.000
553	GOBP_EPITHELIUM_DEVELOPMENT	GO:0060429	15	-0.334743	-1.001599	0.456637	1.000000	1.000
554	REACTOME_PLATELET_ACTIVATION_SIGNALING_AND_AGG...	R-HSA-76002	7	-0.420169	-1.002076	0.448399	1.000000	1.000
555	GOBP_ERBB_SIGNALING_PATHWAY	GO:0038127	5	-0.472223	-1.003205	0.453668	1.000000	1.000
556	GOBP_CELLULAR_MACROMOLECULE_CATABOLIC_PROCESS	GO:0044265	6	-0.440747	-1.005959	0.434109	1.000000	1.000

Continuation of Table **S17**

	NAME	ID	SIZE	ES	NES	NOM.p.val	FDR.q.val	FWER.p.val
557	GOBP_MUSCLE_CONTRACTION	GO:0006936	5	-0.483260	-1.010656	0.444444	1.000000	1.000
558	GOBP_REGULATION_OF_GTPASE_ACTIVITY	GO:0043087	5	-0.466401	-1.012724	0.432971	1.000000	1.000
559	GOBP_NEGATIVE_REGULATION_OF_RESPONSE_TO_STIMULUS	GO:0048585	16	-0.340426	-1.015809	0.436330	1.000000	1.000
560	GOBP_SECRETION	GO:0046903	24	-0.304797	-1.016794	0.442341	1.000000	1.000
561	GOBP_RESPONSE_TO_OXIDATIVE_STRESS	GO:0006979	8	-0.411000	-1.017841	0.436090	1.000000	1.000
562	GOBP_ENDOTHELIAL_CELL_PROLIFERATION	GO:0001935	8	-0.404972	-1.020263	0.408318	1.000000	1.000
563	GOBP_NEURON_DEATH	GO:0070997	18	-0.321066	-1.020649	0.408273	1.000000	1.000
564	GOBP_POSITIVE_REGULATION_OF_ORGANELLE_ORGANIZA...	GO:0010638	10	-0.378709	-1.021307	0.425243	1.000000	1.000
565	GOBP_EPIDERMIS_DEVELOPMENT	GO:0008544	5	-0.479728	-1.022036	0.448718	1.000000	1.000
566	GOBP_MYELOID_LEUKOCYTE_DIFFERENTIATION	GO:0002573	11	-0.376473	-1.023769	0.428846	1.000000	1.000
567	GOBP_RESPONSE_TO_MONOSACCHARIDE	GO:0034284	5	-0.481747	-1.024687	0.410646	1.000000	1.000
568	GOBP_RESPONSE_TO_CARBOHYDRATE	GO:0009743	5	-0.481747	-1.024895	0.425612	1.000000	1.000
569	GOBP_CELLULAR_RESPONSE_TO_PEPTIDE	GO:1901653	13	-0.362164	-1.030840	0.425725	1.000000	1.000
570	GOBP_RESPONSE_TO_PEPTIDE_HORMONE	GO:0043434	13	-0.373743	-1.032592	0.434028	1.000000	1.000
571	GOBP_RESPONSE_TO_TEMPERATURE_STIMULUS	GO:0009266	9	-0.399247	-1.032598	0.392336	1.000000	1.000
572	GOBP_RESPONSE_TO_REACTIVE_OXYGEN_SPECIES	GO:0000302	8	-0.411000	-1.039949	0.395872	1.000000	1.000
573	REACTOME_EXTRA_NUCLEAR_ESTROGEN_SIGNALING	R-RNO-9009391	7	-0.450473	-1.044871	0.395221	1.000000	1.000
574	GOBP_CIRCULATORY_SYSTEM_DEVELOPMENT	GO:0072359	21	-0.320841	-1.046599	0.382143	1.000000	1.000
575	GOBP_POSITIVE_REGULATION_OF_ENDOTHELIAL_CELL_P...	GO:0001938	6	-0.458149	-1.047039	0.403013	1.000000	1.000
576	GOBP_POSTTRANSCRIPTIONAL_REGULATION_OF_GENE_EX...	GO:0010608	8	-0.415152	-1.047486	0.395307	1.000000	1.000
577	GOBP_CELLULAR_RESPONSE_TO_MOLECULE_OF_BACTERIA...	GO:0071219	8	-0.421684	-1.050226	0.403704	1.000000	1.000
578	REACTOME_NGF_STIMULATED_TRANSCRIPTION	R-RNO-9031628	9	-0.413370	-1.053783	0.397790	1.000000	1.000
579	GOBP_HOMEOSTATIC_PROCESS	GO:0042592	31	-0.294474	-1.054194	0.364407	1.000000	1.000
580	GOBP_CARBOHYDRATE_HOMEOSTASIS	GO:0033500	7	-0.441105	-1.054769	0.381982	1.000000	1.000
581	GOBP_PEPTIDYL_AMINO_ACID_MODIFICATION	GO:0018193	22	-0.321413	-1.055029	0.403571	1.000000	1.000
582	GOBP_CELLULAR_RESPONSE_TO_BIOTIC_STIMULUS	GO:0071216	8	-0.421684	-1.055460	0.361624	1.000000	1.000
583	GOBP_G_PROTEIN_COUPLED_GLUTAMATE_RECEPTOR_SIGN...	GO:0007216	8	-0.432013	-1.055966	0.387097	1.000000	1.000
584	GOBP_POSITIVE_REGULATION_OF_MULTICELLULAR_ORGA...	GO:0051240	32	-0.302216	-1.057806	0.376491	1.000000	1.000
585	GOBP_CELLULAR_RESPONSE_TO_HORMONE_STIMULUS	GO:0032870	11	-0.382360	-1.058641	0.377163	1.000000	1.000
586	GOBP_POSITIVE_REGULATION_OF_APOPTOTIC_SIGNALIN...	GO:2001235	5	-0.508850	-1.069576	0.364662	1.000000	1.000
587	REACTOME_NUCLEAR_EVENTS_KINASE_AND_TRANSCRIPTI...	R-RNO-198725	11	-0.399388	-1.082255	0.373473	1.000000	1.000
588	REACTOME_INTRACELLULAR_SIGNALING_BY_SECOND_MES...	R-RNO-9006925	15	-0.363970	-1.082823	0.343525	1.000000	1.000
589	GOBP_SENSORY_PERCEPTION_OF_PAIN	GO:0019233	5	-0.514541	-1.097756	0.337864	1.000000	1.000
590	GOBP_VESICLE_MEDIATED_TRANSPORT_IN_SYNAPSE	GO:0099003	7	-0.457735	-1.099625	0.335849	1.000000	1.000
591	GOBP_ENZYME_LINKED_RECEPTOR_PROTEIN_SIGNALING_...	GO:0007167	24	-0.329189	-1.103545	0.340619	1.000000	1.000
592	GOBP_RESPONSE_TO_MOLECULE_OF_BACTERIAL_ORIGIN	GO:0002237	13	-0.381479	-1.108690	0.310219	1.000000	1.000
593	GOBP_REGULATION_OF_PROTEIN_MODIFICATION_PROCESS	GO:0031399	29	-0.321889	-1.111505	0.362500	1.000000	1.000
594	GOBP_SIGNAL_RELEASE	GO:0023061	14	-0.364823	-1.112872	0.325000	1.000000	1.000
595	REACTOME_CLASS_C_3_METABOTROPIC_GLUTAMATE_PHER...	R-RNO-420499	7	-0.467421	-1.113107	0.323917	1.000000	1.000
596	GOBP_RESPONSE_TO_BACTERIUM	GO:0009617	13	-0.381479	-1.114098	0.300725	1.000000	1.000
597	GOBP_DEFENSE_RESPONSE_TO_OTHER_ORGANISM	GO:0098542	9	-0.424023	-1.116243	0.317940	1.000000	1.000
598	GOBP_T_CELL_ACTIVATION	GO:0042110	8	-0.440402	-1.116846	0.311808	1.000000	1.000
599	GOBP_IMMUNE_EFFECTOR_PROCESS	GO:0002252	10	-0.421477	-1.118004	0.319312	1.000000	1.000

Continuation of Table **S17**

	NAME	ID	SIZE	ES	NES	NOM.p.val	FDR.q.val	FWER.p.val
600	GOBP_POSITIVE_REGULATION_OF_CYTOKINE_PRODUCTION	GO:0001819	7	-0.473974	-1.122191	0.323793	1.000000	1.000
601	GOBP_MYELOID_CELL_DIFFERENTIATION	GO:0030099	13	-0.394082	-1.124852	0.323636	1.000000	1.000
602	GOBP_INTRACELLULAR_PROTEIN_TRANSPORT	GO:0006886	9	-0.434450	-1.125901	0.304348	1.000000	1.000
603	GOBP_REGULATION_OF_CELLULAR_RESPONSE_TO_STRESS	GO:0080135	9	-0.436322	-1.133599	0.282908	1.000000	1.000
604	GOBP_CIRCADIAN_RHYTHM	GO:0007623	13	-0.398569	-1.135474	0.289617	1.000000	1.000
605	GOBP_RESPONSE_TO_CADMIUM_ION	GO:0046686	6	-0.497680	-1.135741	0.298279	1.000000	1.000
606	GOBP_NEGATIVE_REGULATION_OF_DEVELOPMENTAL_PROCESS	GO:0051093	13	-0.401756	-1.136608	0.284710	1.000000	1.000
607	REACTOME_GPCR_LIGAND_BINDING	R-RNO-500792	8	-0.450624	-1.137968	0.302632	1.000000	1.000
608	GOBP_TISSUE_HOMEOSTASIS	GO:0001894	5	-0.551256	-1.138624	0.306358	1.000000	1.000
609	GOBP_TRANSMEMBRANE_RECEPTOR_PROTEIN_SERINE_THR...	GO:0007178	6	-0.508179	-1.140026	0.322097	1.000000	1.000
610	REACTOME_PTEN_REGULATION	R-RNO-6807070	5	-0.536815	-1.140867	0.321839	1.000000	1.000
611	GOBP_MUSCLE_ORGAN_DEVELOPMENT	GO:0007517	9	-0.443124	-1.141710	0.296429	1.000000	1.000
612	GOBP_RESPONSE_TO_CYTOKINE	GO:0034097	23	-0.341815	-1.147188	0.290844	1.000000	1.000
613	REACTOME_G_PROTEIN_MEDIATED_EVENTS	R-RNO-112040	9	-0.444302	-1.148263	0.292776	1.000000	1.000
614	HALLMARK_TNFA_SIGNALING_VIA_NFKB	M5890	14	-0.388945	-1.149273	0.281250	1.000000	1.000
615	GOBP_CELLULAR_RESPONSE_TO_CHEMICAL_STRESS	GO:0062197	7	-0.478848	-1.152781	0.312741	1.000000	1.000
616	GOBP_CELLULAR_RESPONSE_TO_REACTIVE_OXYGEN_SPECIES	GO:0034614	7	-0.478848	-1.157208	0.262477	1.000000	1.000
617	GOBP_SENSORY_ORGAN_DEVELOPMENT	GO:0007423	8	-0.459658	-1.157982	0.275605	1.000000	1.000
618	GOBP_RESPONSE_TO_NITROGEN_COMPOUND	GO:1901698	34	-0.324602	-1.158801	0.260274	1.000000	1.000
619	GOBP_TUBE_DEVELOPMENT	GO:0035295	18	-0.367834	-1.159369	0.270945	1.000000	1.000
620	GOBP_RESPONSE_TO_BIOTIC_STIMULUS	GO:0009607	17	-0.379776	-1.161181	0.275000	1.000000	1.000
621	GOBP_REGULATION_OF_NEUROTRANSMITTER_LEVELS	GO:0001505	7	-0.487229	-1.168534	0.258589	1.000000	1.000
622	GOBP_INTRACELLULAR_TRANSPORT	GO:0046907	11	-0.427955	-1.168588	0.284895	1.000000	1.000
623	GOBP_MULTI_ORGANISM_PROCESS	GO:0051704	16	-0.385973	-1.169561	0.252951	1.000000	1.000
624	GOBP_INTRACELLULAR_RECEPTOR_SIGNALING_PATHWAY	GO:0030522	5	-0.547279	-1.171722	0.279029	1.000000	1.000
625	GOBP_DEVELOPMENTAL_PROCESS_INVOLVED_IN_REPRODU...	GO:0003006	11	-0.428170	-1.172364	0.273874	1.000000	1.000
626	GOBP_RESPONSE_TO_GROWTH_FACTOR	GO:0070848	18	-0.373102	-1.172486	0.270650	1.000000	1.000
627	REACTOME_CA_DEPENDENT_EVENTS	R-RNO-111996	8	-0.467645	-1.173623	0.262570	1.000000	1.000
628	GOBP_RESPONSE_TO_WOUNDING	GO:0009611	15	-0.394074	-1.174360	0.239604	1.000000	1.000
629	GOBP_REGULATION_OF_TRANSPORT	GO:0051049	44	-0.320616	-1.174566	0.230118	1.000000	1.000
630	GOBP_INNATE_IMMUNE_RESPONSE	GO:0045087	7	-0.498767	-1.176941	0.246094	1.000000	1.000
631	GOBP_CELLULAR_RESPONSE_TO_DNA_DAMAGE_STIMULUS	GO:0006974	5	-0.553815	-1.179150	0.274336	1.000000	1.000
632	GOBP_NEUROTRANSMITTER_TRANSPORT	GO:0006836	7	-0.487229	-1.179804	0.255083	1.000000	1.000
633	GOBP_CELLULAR_RESPONSE_TO_ABIOTIC_STIMULUS	GO:0071214	7	-0.489387	-1.184321	0.254335	1.000000	1.000
634	GOBP_NEGATIVE_REGULATION_OF_CELL_DIFFERENTIATION	GO:0045596	13	-0.401756	-1.184568	0.237660	1.000000	1.000
635	GOBP_POSITIVE_REGULATION_OF_ANION_TRANSPORT	GO:1903793	9	-0.459818	-1.185317	0.264815	1.000000	1.000
636	GOBP_APOPTOTIC_PROCESS	GO:0006915	35	-0.330345	-1.187828	0.229730	1.000000	1.000
637	GOBP_REGULATION_OF_NEUROTRANSMITTER_TRANSPORT	GO:0051588	6	-0.523714	-1.204312	0.240076	1.000000	1.000
638	GOBP_RESPONSE_TO_ENDOGENOUS_STIMULUS	GO:0009719	34	-0.336124	-1.209668	0.186047	1.000000	1.000
639	GOBP_MULTICELLULAR_ORGANISM_REPRODUCTION	GO:0032504	12	-0.418760	-1.210626	0.223636	1.000000	1.000
640	GOBP_RESPONSE_TO_OXYGEN_CONTAINING_COMPOUND	GO:1901700	39	-0.330835	-1.210829	0.198413	1.000000	1.000
641	GOBP_CELLULAR_GLUCOSE_HOMEOSTASIS	GO:0001678	5	-0.579450	-1.220342	0.224206	1.000000	1.000
642	GOBP_POSITIVE_REGULATION_OF_CELLULAR_PROTEIN_L...	GO:1903829	7	-0.519609	-1.222117	0.210630	1.000000	1.000

Continuation of Table **S17**

	NAME	ID	SIZE	ES	NES	NOM.p.val	FDR.q.val	FWER.p.val
643	REACTOME_CELLULAR_SENESCENCE	R-RNO-2559583	6	-0.539751	-1.231341	0.213611	1.000000	1.000
644	GOBP_RESPONSE_TO_HORMONE	GO:0009725	20	-0.385429	-1.234662	0.175347	1.000000	1.000
645	REACTOME_SENESCENCE_ASSOCIATED_SECRETORY_PHENO...	R-RNO-2559582	6	-0.539751	-1.237579	0.227778	1.000000	1.000
646	GOBP_POSITIVE_REGULATION_OF_TRANSCRIPTION_BY_R...	GO:0045944	24	-0.373162	-1.238477	0.179715	1.000000	1.000
647	GOBP_NUCLEAR_TRANSPORT	GO:0051169	5	-0.571574	-1.239763	0.208647	1.000000	1.000
648	GOBP_WOUND_HEALING	GO:0042060	13	-0.437100	-1.240211	0.209220	1.000000	1.000
649	GOBP_ENDOMEMBRANE_SYSTEM_ORGANIZATION	GO:0010256	6	-0.542079	-1.241413	0.216822	1.000000	1.000
650	GOBP_RESPONSE_TO_LIPID	GO:0033993	19	-0.394147	-1.247149	0.195164	1.000000	1.000
651	GOBP_POSITIVE_REGULATION_OF_RESPONSE_TO_EXTERN...	GO:0032103	9	-0.483751	-1.254024	0.199275	1.000000	1.000
652	GOBP_POSITIVE_REGULATION_OF_CELL_POPULATION_PR...	GO:0008284	21	-0.390006	-1.254825	0.169611	1.000000	1.000
653	GOBP_SPROUTING_ANGIOGENESIS	GO:0002040	5	-0.587800	-1.255246	0.217143	1.000000	1.000
654	GOBP_REGULATION_OF_ORGANELLE_ORGANIZATION	GO:0033043	14	-0.423197	-1.255777	0.177331	1.000000	1.000
655	REACTOME_CELL_CYCLE_MITOTIC	R-RNO-69278	5	-0.594870	-1.262422	0.215534	1.000000	1.000
656	REACTOME_SIGNALING_BY_WNT	R-RNO-195721	7	-0.539098	-1.268229	0.215470	1.000000	1.000
657	GOBP_POSITIVE_REGULATION_OF_GENE_EXPRESSION	GO:0010628	16	-0.413721	-1.273607	0.174863	1.000000	1.000
658	GOBP_RESPONSE_TO_PEPTIDE	GO:1901652	18	-0.409999	-1.276927	0.149228	1.000000	1.000
659	GOBP_POSITIVE_REGULATION_OF_BIOSYNTHETIC_PROCESS	GO:0009891	27	-0.370181	-1.280812	0.142373	1.000000	1.000
660	GOBP_ANATOMICAL_STRUCTURE_HOMEOSTASIS	GO:0060249	7	-0.551538	-1.293833	0.180113	1.000000	1.000
661	GOBP_NEUROTRANSMITTER_SECRETION	GO:0007269	6	-0.571402	-1.298017	0.167293	1.000000	1.000
662	GOBP_REPRODUCTION	GO:0000003	18	-0.415411	-1.309239	0.139746	1.000000	1.000
663	GOBP_RESPONSE_TO_INSULIN	GO:0032868	5	-0.611207	-1.309444	0.177449	1.000000	1.000
664	GOBP_LEUKOCYTE_DIFFERENTIATION	GO:0002521	15	-0.436468	-1.311407	0.144366	1.000000	1.000
665	GOBP_LYMPHOCYTE_ACTIVATION	GO:0046649	9	-0.505247	-1.313235	0.168880	1.000000	1.000
666	GOBP_VASCULATURE_DEVELOPMENT	GO:0001944	17	-0.412708	-1.314231	0.134513	1.000000	1.000
667	REACTOME_G_ALPHA_Z_SIGNALLING_EVENTS	R-RNO-418597	5	-0.599928	-1.314658	0.153846	1.000000	1.000
668	GOBP_DNA_METABOLIC_PROCESS	GO:0006259	6	-0.590536	-1.316252	0.146947	1.000000	1.000
669	REACTOME_G_ALPHA_Q_SIGNALLING_EVENTS	R-RNO-416476	6	-0.574715	-1.324297	0.145422	1.000000	1.000
670	GOBP_EPITHELIAL_CELL_PROLIFERATION	GO:0050673	12	-0.482462	-1.332953	0.123574	1.000000	1.000
671	GOBP_NEGATIVE_REGULATION_OF_CELL_POPULATION_PR...	GO:0008285	9	-0.526182	-1.336109	0.164021	1.000000	1.000
672	GOBP_EXOCYTOSIS	GO:0006887	11	-0.491934	-1.338361	0.134752	1.000000	1.000
673	GOBP_CHROMATIN_ORGANIZATION	GO:0006325	5	-0.634096	-1.350145	0.144665	1.000000	1.000
674	GOBP_CYTOKINE_PRODUCTION	GO:0001816	10	-0.500136	-1.355593	0.123616	1.000000	1.000
675	GOBP_CELLULAR_RESPONSE_TO_LIPID	GO:0071396	15	-0.451310	-1.356311	0.128521	1.000000	1.000
676	GOBP_EMBRYONIC_MORPHOGENESIS	GO:0048598	6	-0.598975	-1.369116	0.123260	1.000000	1.000
677	GOBP_POSITIVE_REGULATION_OF_NUCLEOBASE_CONTAIN...	GO:0045935	28	-0.404568	-1.369721	0.093537	1.000000	1.000
678	GOBP_RHYTHMIC_PROCESS	GO:0048511	15	-0.460344	-1.369945	0.123862	1.000000	1.000
679	GOBP_CELLULAR_RESPONSE_TO_NITROGEN_COMPOUND	GO:1901699	20	-0.432242	-1.375963	0.094033	1.000000	1.000
680	GOBP_REGULATION_OF_CELL_ACTIVATION	GO:0050865	8	-0.562184	-1.391066	0.088448	1.000000	1.000
681	GOBP_APOPTOTIC_SIGNALING_PATHWAY	GO:0097190	15	-0.468701	-1.395557	0.104452	1.000000	1.000
682	REACTOME_MEMBRANE_TRAFFICKING	R-RNO-199991	5	-0.666381	-1.422593	0.118421	0.967296	1.000
683	GOBP_SENSORY_PERCEPTION	GO:0007600	9	-0.559274	-1.426705	0.100569	0.973322	1.000
684	GOBP_PROTEIN_MODIFICATION_BY_SMALL_PROTEIN_CON...	GO:0070647	7	-0.603033	-1.427123	0.095149	0.998888	1.000
685	GOBP_MYELOID_LEUKOCYTE_ACTIVATION	GO:0002274	8	-0.581523	-1.428807	0.087199	1.000000	1.000

Continuation of Table **S17**

	NAME	ID	SIZE	ES	NES	NOM.p.val	FDR.q.val	FWER.p.val
686	REACTOME_VESICLE_MEDIATED_TRANSPORT	R-RNO-5653656	5	-0.666381	-1.432497	0.104046	1.000000	1.000
687	GOBP_LEUKOCYTE_MEDIATED_IMMUNITY	GO:0002443	7	-0.606019	-1.450108	0.084149	0.965792	1.000
688	GOBP_LOCOMOTORY_BEHAVIOR	GO:0007626	7	-0.593397	-1.450393	0.090559	0.995552	1.000
689	GOBP_RESPONSE_TO_ORGANIC_CYCLIC_COMPOUND	GO:0014070	20	-0.450526	-1.458316	0.051002	0.983416	1.000
690	GOBP_REGULATION_OF_ANION_TRANSPORT	GO:0044070	20	-0.444662	-1.459106	0.066318	1.000000	1.000
691	GOBP_ANIMAL_ORGAN_MORPHOGENESIS	GO:0009887	12	-0.521575	-1.460099	0.073741	1.000000	1.000
692	GOBP_IMMUNE_SYSTEM_DEVELOPMENT	GO:0002520	17	-0.478511	-1.461233	0.066038	1.000000	1.000
693	GOBP_REGULATION_OF_RESPONSE_TO_STRESS	GO:0080134	19	-0.462979	-1.467809	0.066308	1.000000	1.000
694	GOBP_REGULATION_OF_BODY_FLUID_LEVELS	GO:0050878	11	-0.528423	-1.475043	0.086556	1.000000	1.000
695	REACTOME_CELL_CYCLE	R-RNO-1640170	6	-0.662755	-1.476919	0.055856	1.000000	1.000
696	GOBP_SKELETAL_MUSCLE_ORGAN_DEVELOPMENT	GO:0060538	5	-0.686747	-1.479346	0.055118	1.000000	1.000
697	GOBP_PROTEIN_MODIFICATION_BY_SMALL_PROTEIN_CON...	GO:0032446	6	-0.659361	-1.491438	0.069943	1.000000	1.000
698	REACTOME_BETA_CATENIN_INDEPENDENT_WNT_SIGNALING	R-RNO-3858494	5	-0.699678	-1.493149	0.058712	1.000000	1.000
699	GOBP_ANATOMICAL_STRUCTURE_FORMATION_INVOLVED_I...	GO:0048646	20	-0.454745	-1.495917	0.052158	1.000000	1.000
700	GOBP_REGULATION_OF_RESPONSE_TO_EXTERNAL_STIMULUS	GO:0032101	15	-0.511835	-1.507414	0.042403	1.000000	1.000
701	GOBP_MONONUCLEAR_CELL_DIFFERENTIATION	GO:1903131	7	-0.629639	-1.525069	0.064912	1.000000	1.000
702	GOBP_ORGANIC_HYDROXY_COMPOUND_TRANSPORT	GO:0015850	6	-0.674419	-1.528868	0.054820	1.000000	1.000
703	REACTOME_RHO_GTPASE_EFFECTORS	R-RNO-195258	9	-0.604415	-1.539969	0.054206	1.000000	1.000
704	GOBP_PEPTIDYL_LYSINE_MODIFICATION	GO:0018205	5	-0.729186	-1.545917	0.047445	1.000000	1.000
705	GOBP_CHROMOSOME_ORGANIZATION	GO:0051276	6	-0.688163	-1.585605	0.025440	0.963933	1.000
706	GOBP_CELLULAR_RESPONSE_TO_OXYGEN_CONTAINING_CO...	GO:1901701	29	-0.458270	-1.586052	0.020443	1.000000	1.000
707	GOBP_CIRCADIAN_REGULATION_OF_GENE_EXPRESSION	GO:0032922	5	-0.751861	-1.587849	0.027290	1.000000	1.000
708	REACTOME_SIGNALING_BY_RHO_GTPASES_MIRO_GTPASES...	R-RNO-9716542	9	-0.604415	-1.588145	0.038168	1.000000	1.000
709	GOBP_CELL_POPULATION_PROLIFERATION	GO:0008283	31	-0.447355	-1.599630	0.022034	1.000000	1.000
710	GOBP_CELLULAR_RESPONSE_TO_ORGANIC_CYCLIC_COMPOUND	GO:0071407	13	-0.560914	-1.630957	0.026502	1.000000	1.000
711	GOBP_REPRODUCTIVE_SYSTEM_DEVELOPMENT	GO:0061458	6	-0.729849	-1.661377	0.009174	0.965187	1.000
712	GOBP_RESPONSE_TO_INTERLEUKIN_1	GO:0070555	5	-0.781354	-1.682932	0.017208	0.940891	1.000
713	GOBP_COAGULATION	GO:0050817	8	-0.676126	-1.709760	0.020794	0.878536	0.995
714	GOBP_PLATELET_ACTIVATION	GO:0030168	6	-0.777872	-1.756444	0.007692	0.706238	0.974
715	REACTOME_NEUTROPHIL_DEGRANULATION	R-RNO-6798695	6	-0.789539	-1.767587	0.003466	0.807992	0.964
716	GOBP_MYELOID_LEUKOCYTE_MEDIATED_IMMUNITY	GO:0002444	6	-0.789539	-1.787366	0.003906	0.897560	0.935
717	GOBP_CELL_ACTIVATION_INVOLVED_IN_IMMUNE_RESPONSE	GO:0002263	6	-0.789539	-1.810870	0.005386	1.000000	0.892
718	GOBP_CELL_ACTIVATION	GO:0001775	21	-0.566059	-1.827684	0.003503	1.000000	0.844

End of Table

Supplementary Table S18: CB early profile (8 DPL peak) GSEA results.

Begin of Table **S18**

	NAME	ID	SIZE	ES	NES	NOM.p.val	FDR.q.val	FWER.p.val
0	GOBP_POSITIVE_REGULATION_OF_TRANSMEMBRANE_TRAN...	GO:0034764	7	0.634079	1.681567	0.018100	1.000000	0.949
1	GOBP_POSITIVE_REGULATION_OF_CATION_TRANSMEMBRA...	GO:1904064	5	0.685165	1.601183	0.050000	1.000000	0.989
2	HALLMARK_PI3K_AKT_MTOR_SIGNALING	M5923	6	0.595609	1.559698	0.052209	1.000000	0.994
3	GOBP_MAINTENANCE_OF_LOCATION	GO:0051235	7	0.548383	1.487546	0.053812	1.000000	1.000
4	GOBP_POSITIVE_REGULATION_OF_SMOOTH_MUSCLE_CELL...	GO:0048661	5	0.626506	1.450866	0.073913	1.000000	1.000
5	GOBP_MULTICELLULAR_ORGANISMAL_RESPONSE_TO_STRESS	GO:0033555	5	0.590239	1.400324	0.090909	1.000000	1.000
6	GOBP_REGULATION_OF_CELL_SIZE	GO:0008361	5	0.602410	1.398462	0.099617	1.000000	1.000
7	GOBP_DEVELOPMENTAL_CELL_GROWTH	GO:0048588	5	0.578313	1.350037	0.105660	1.000000	1.000
8	GOBP_REGULATION_OF_CELLULAR_COMPONENT_SIZE	GO:0032535	7	0.481481	1.291670	0.116279	1.000000	1.000
9	HALLMARK_EPITHELIAL_MESENCHYMAL_TRANSITION	M5930	5	0.542169	1.279244	0.150376	1.000000	1.000
10	GOBP_POSITIVE_REGULATION_OF_CELL_GROWTH	GO:0030307	6	0.500000	1.255753	0.162879	1.000000	1.000
11	GOBP_CELLULAR_LIPID_METABOLIC_PROCESS	GO:0044255	7	0.463166	1.249504	0.181373	1.000000	1.000
12	GOBP_IMPORT_INTO_CELL	GO:0098657	6	0.487805	1.239003	0.163180	1.000000	1.000
13	GOBP_RESPONSE_TO_ETHANOL	GO:0045471	5	0.530120	1.235441	0.189003	1.000000	1.000
14	GOBP_LEUKOCYTE_MIGRATION	GO:0050900	6	0.473379	1.183697	0.247826	1.000000	1.000
15	GOBP_NEUROTRANSMITTER_TRANSPORT	GO:0006836	7	0.419753	1.175854	0.240000	1.000000	1.000
16	GOBP_NEUROINFLAMMATORY_RESPONSE	GO:0150076	5	0.506024	1.175312	0.216867	1.000000	1.000
17	GOBP_REGULATION_OF_PEPTIDYL_TYROSINE_PHOSPHORY...	GO:0050730	9	0.379754	1.169050	0.251337	1.000000	1.000
18	GOBP_PURINE_CONTAINING_COMPOUND_METABOLIC_PROCESS	GO:0072521	5	0.506024	1.158525	0.248120	1.000000	1.000
19	GOBP_REGULATION_OF_CIRCADIAN_RHYTHM	GO:0042752	7	0.419753	1.141470	0.231405	1.000000	1.000
20	REACTOME_ADAPTIVE_IMMUNE_SYSTEM	R-RNO-1280218	9	0.378689	1.124814	0.238806	1.000000	1.000
21	GOBP_CALCIUM_MEDIATED_SIGNALING	GO:0019722	10	0.344439	1.124603	0.255556	1.000000	1.000
22	GOBP_REGULATION_OF_NEUROTRANSMITTER_LEVELS	GO:0001505	7	0.419753	1.120681	0.261044	1.000000	1.000
23	GOBP_POSITIVE_REGULATION_OF_DNA_BINDING_TRANSC...	GO:0051091	10	0.355452	1.098615	0.320442	1.000000	1.000
24	GOBP_PHAGOCYTOSIS	GO:0006909	5	0.474411	1.097960	0.307407	1.000000	1.000
25	GOBP_SYNAPTIC_SIGNALING	GO:0099536	47	0.213272	1.085643	0.319149	1.000000	1.000
26	GOBP_POSITIVE_REGULATION_OF_PEPTIDYL_TYROSINE_...	GO:0050731	8	0.387488	1.074784	0.347222	1.000000	1.000
27	GOBP_POSITIVE_REGULATION_OF_SECRETION	GO:0051047	6	0.426829	1.074168	0.329218	1.000000	1.000
28	GOBP_REGULATION_OF_NEUROTRANSMITTER_TRANSPORT	GO:0051588	6	0.414634	1.070534	0.334677	1.000000	1.000
29	GOBP_REGULATION_OF_METAL_ION_TRANSPORT	GO:0010959	6	0.418551	1.069529	0.330645	1.000000	1.000
30	GOBP_ANION_TRANSMEMBRANE_TRANSPORT	GO:0098656	7	0.380083	1.061942	0.352423	1.000000	1.000
31	GOBP_NEUROTRANSMITTER_SECRETION	GO:0007269	6	0.414634	1.054983	0.356364	1.000000	1.000
32	GOBP_LONG_TERM_SYNAPTIC_DEPRESSION	GO:0060292	5	0.445783	1.053680	0.371901	1.000000	1.000
33	GOBP_GENERATION_OF_PRECURSOR_METABOLITES_AND_E...	GO:0006091	5	0.457831	1.052033	0.335766	1.000000	1.000
34	GOBP_REGULATION_OF_CATION_TRANSMEMBRANE_TRANSPORT	GO:1904062	16	0.276959	1.049990	0.356164	1.000000	1.000
35	GOBP_NEGATIVE_REGULATION_OF_PEPTIDASE_ACTIVITY	GO:0010466	6	0.413066	1.045760	0.354086	1.000000	1.000
36	GOBP_EXTRINSIC_APOPTOTIC_SIGNALING_PATHWAY	GO:0097191	7	0.382716	1.045181	0.392405	1.000000	1.000
37	GOBP_REGULATION_OF_SYNAPTIC_TRANSMISSION_GLUTA...	GO:0051966	12	0.312473	1.040453	0.383648	1.000000	1.000
38	GOBP_REGULATION_OF_TRANSMEMBRANE_TRANSPORT	GO:0034762	23	0.253381	1.036645	0.390805	1.000000	1.000
39	GOBP_ORGANIC_HYDROXY_COMPOUND_METABOLIC_PROCESS	GO:1901615	7	0.376333	1.033767	0.400000	1.000000	1.000
40	GOBP_REGULATION_OF_SYNAPSE_STRUCTURE_OR_ACTIVITY	GO:0050803	11	0.315907	1.031784	0.366013	1.000000	1.000

Continuation of Table **S18**

	NAME	ID	SIZE	ES	NES	NOM.p.val	FDR.q.val	FWER.p.val
41	REACTOME_POST_TRANSLATIONAL_PROTEIN_MODIFICATION	R-RNO-597592	7	0.382716	1.029434	0.403509	1.000000	1.000
42	GOBP_NEGATIVE_REGULATION_OF_LOCOMOTION	GO:0040013	5	0.437111	1.027291	0.364341	1.000000	1.000
43	GOBP_PEPTIDYL_TYROSINE_MODIFICATION	GO:0018212	10	0.323728	1.026020	0.421875	1.000000	1.000
44	GOBP_MONOAMINE_TRANSPORT	GO:0015844	5	0.421687	0.999500	0.422481	1.000000	1.000
45	GOBP_PROTEIN_KINASE_B_SIGNALING	GO:0043491	6	0.386692	0.990706	0.437247	1.000000	1.000
46	GOBP_TRANSMEMBRANE_TRANSPORT	GO:0055085	27	0.226163	0.988514	0.500000	1.000000	1.000
47	GOBP_DEVELOPMENTAL_MATURATION	GO:0021700	5	0.424817	0.975783	0.447876	1.000000	1.000
48	GOBP_SYNAPTIC_TRANSMISSION_GLUTAMATERGIC	GO:0035249	13	0.281411	0.971804	0.522988	1.000000	1.000
49	GOBP_DENDRITIC_SPINE_DEVELOPMENT	GO:0060996	6	0.388227	0.964698	0.500000	1.000000	1.000
50	GOBP_REGULATION_OF_EXTRINSIC_APOPTOTIC_SIGNALI...	GO:2001236	6	0.378049	0.947307	0.504202	1.000000	1.000
51	GOBP_DENDRITE_MORPHOGENESIS	GO:0048813	6	0.374035	0.941670	0.523810	1.000000	1.000
52	GOBP_DENDRITIC_SPINE_MORPHOGENESIS	GO:0060997	5	0.403918	0.925754	0.516605	1.000000	1.000
53	GOBP_REGULATION_OF_MEMBRANE_POTENTIAL	GO:0042391	17	0.239437	0.916956	0.571429	1.000000	1.000
54	GOBP_PATTERN_SPECIFICATION_PROCESS	GO:0007389	6	0.361926	0.909083	0.537778	1.000000	1.000
55	GOBP_INORGANIC_ION_TRANSMEMBRANE_TRANSPORT	GO:0098660	9	0.291581	0.904032	0.601010	1.000000	1.000
56	REACTOME_ANTI_INFLAMMATORY_RESPONSE_FAVOURING_...	R-HSA-9662851	5	0.383721	0.897969	0.608163	1.000000	1.000
57	GOBP_MORPHOGENESIS_OF_AN_EPITHELIUM	GO:0002009	6	0.348872	0.896994	0.628459	1.000000	1.000
58	GOBP_REGIONALIZATION	GO:0003002	6	0.361926	0.895205	0.560484	1.000000	1.000
59	GOBP_CATION_TRANSMEMBRANE_TRANSPORT	GO:0098655	18	0.224483	0.893480	0.661157	1.000000	1.000
60	GOBP_REGULATION_OF_NMDA_RECEPTOR_ACTIVITY	GO:2000310	10	0.282051	0.888327	0.632979	1.000000	1.000
61	GOBP_REGULATION_OF_LONG_TERM_SYNAPTIC_POTENTIA...	GO:1900271	6	0.351370	0.879746	0.597561	1.000000	1.000
62	GOBP_POSITIVE_REGULATION_OF_GLIOGENESIS	GO:0014015	5	0.373494	0.872370	0.637631	1.000000	1.000
63	GOBP_REGULATION_OF_GLIOGENESIS	GO:0014013	5	0.373494	0.868551	0.607930	1.000000	1.000
64	GOBP_CALCIUM_ION_TRANSMEMBRANE_TRANSPORT	GO:0070588	7	0.311632	0.865025	0.653509	1.000000	1.000
65	GOBP_TISSUE_MORPHOGENESIS	GO:0048729	7	0.315186	0.864055	0.646091	1.000000	1.000
66	GOBP_REGULATION_OF_SIGNALING_RECEPTOR_ACTIVITY	GO:0010469	14	0.243939	0.862282	0.680000	1.000000	1.000
67	HALLMARK_ALLOGRAFT_REJECTION	M5950	7	0.315448	0.854894	0.621005	1.000000	1.000
68	GOBP_CYTOSOLIC_CALCIUM_ION_TRANSPORT	GO:0060401	7	0.311632	0.849554	0.656652	1.000000	1.000
69	GOBP_GLIAL_CELL_DIFFERENTIATION	GO:0010001	10	0.264346	0.841469	0.668639	1.000000	1.000
70	GOBP_SMOOTH_MUSCLE_CELL_PROLIFERATION	GO:0048659	6	0.326512	0.834000	0.693023	1.000000	1.000
71	GOBP_CALCIUM_ION_TRANSPORT_INTO_CYTOSOL	GO:0060402	7	0.311632	0.830171	0.652000	1.000000	1.000
72	REACTOME_RAS_ACTIVATION_UPON_CA2_INFLUX_THROUG...	R-HSA-442982	6	0.317073	0.825116	0.681034	1.000000	1.000
73	GOBP_PROTEIN_LOCALIZATION_TO_PLASMA_MEMBRANE	GO:0072659	7	0.308642	0.825014	0.709544	1.000000	1.000
74	GOBP_EMBRYO_DEVELOPMENT	GO:0009790	14	0.228986	0.824119	0.762431	1.000000	1.000
75	GOBP_POSITIVE_REGULATION_OF_NERVOUS_SYSTEM_DEV...	GO:0051962	12	0.239554	0.824112	0.718563	1.000000	1.000
76	GOBP_REGULATION_OF_VASCULAR_ASSOCIATED_SMOOTH_...	GO:1904705	5	0.347794	0.821874	0.705454	1.000000	1.000
77	GOBP_POSITIVE_REGULATION_OF_LONG_TERM_SYNAPTIC...	GO:1900273	5	0.359789	0.814904	0.689139	1.000000	1.000
78	GOBP_GLIOGENESIS	GO:0042063	12	0.235990	0.802063	0.760234	1.000000	1.000
79	GOBP_REGULATION_OF_POSTSYNAPTIC_MEMBRANE_POTEN...	GO:0060078	14	0.229730	0.801394	0.760479	1.000000	1.000
80	GOBP_POSITIVE_REGULATION_OF_NEUROGENESIS	GO:0050769	12	0.239554	0.800818	0.722628	1.000000	1.000
81	GOBP_EPITHELIAL_CELL_DIFFERENTIATION	GO:0030855	7	0.298946	0.799980	0.746835	1.000000	1.000
82	GOBP_POSITIVE_REGULATION_OF_CELL_DEVELOPMENT	GO:0010720	12	0.239554	0.799867	0.737143	1.000000	1.000
83	GOBP_REGULATION_OF_ANION_TRANSMEMBRANE_TRANSPORT	GO:1903959	5	0.337733	0.799748	0.759398	1.000000	1.000

Continuation of Table **S18**

	NAME	ID	SIZE	ES	NES	NOM.p.val	FDR.q.val	FWER.p.val
84	GOBP_POSITIVE_REGULATION_OF_NEURON_PROJECTION_...	GO:0010976	5	0.348753	0.794252	0.796296	1.000000	1.000
85	GOBP_POSITIVE_REGULATION_OF_LEUKOCYTE_CELL_CEL...	GO:1903039	5	0.337349	0.784630	0.805447	1.000000	1.000
86	GOBP_POSITIVE_REGULATION_OF_CELL_CELL_ADHESION	GO:0022409	5	0.337349	0.784565	0.810606	1.000000	1.000
87	GOBP_REGULATION_OF_PROTEIN_CATABOLIC_PROCESS	GO:0042176	7	0.281888	0.782932	0.778261	1.000000	1.000
88	GOBP_SIGNAL_RELEASE	GO:0023061	14	0.212320	0.775929	0.848485	1.000000	1.000
89	GOBP_REGULATION_OF_POSTSYNAPSE_ORGANIZATION	GO:0099175	6	0.297679	0.775893	0.838057	1.000000	1.000
90	GOBP_DIVALENT_INORGANIC_CATION_HOMEOSTASIS	GO:0072507	13	0.226667	0.765988	0.764706	1.000000	1.000
91	GOBP_REGULATION_OF_CYTOSOLIC_CALCIUM_ION_CONCE...	GO:0051480	13	0.226667	0.764472	0.826667	1.000000	1.000
92	REACTOME_EPH_EPHRIN_SIGNALING	R-RNO-2682334	6	0.304878	0.763666	0.828947	1.000000	1.000
93	GOBP_REGULATION_OF_PHOSPHOLIPASE_ACTIVITY	GO:0010517	6	0.291749	0.761602	0.832618	1.000000	1.000
94	REACTOME_SIGNALING_BY_NTRK2_TRKB	R-RNO-9006115	6	0.301961	0.760647	0.797571	1.000000	1.000
95	GOBP_SYNAPSE_ORGANIZATION	GO:0050808	17	0.204680	0.756041	0.842520	1.000000	1.000
96	GOBP_METAL_ION_TRANSPORT	GO:0030001	11	0.222583	0.755444	0.808989	1.000000	1.000
97	GOBP_REGULATION_OF_PHOSPHOLIPASE_C_ACTIVITY	GO:1900274	5	0.318083	0.754633	0.829545	1.000000	1.000
98	GOBP_POSITIVE_REGULATION_OF_LIPASE_ACTIVITY	GO:0060193	5	0.318083	0.752016	0.809160	1.000000	1.000
99	GOBP_CATION_TRANSPORT	GO:0006812	25	0.169638	0.746098	0.855422	1.000000	1.000
100	GOBP_REGULATION_OF_PROTEIN_TYROSINE_KINASE_ACT...	GO:0061097	5	0.316145	0.739822	0.873077	1.000000	1.000
101	GOBP_TELENCEPHALON_DEVELOPMENT	GO:0021537	6	0.292683	0.737821	0.836134	1.000000	1.000
102	GOBP_CALCIUM_ION_TRANSPORT	GO:0006816	11	0.222583	0.733632	0.852041	1.000000	1.000
103	HALLMARK_KRAS_SIGNALING_UP	M5953	6	0.292720	0.729363	0.825726	1.000000	1.000
104	GOBP_REGULATION_OF_LIPASE_ACTIVITY	GO:0060191	6	0.291749	0.726278	0.859575	1.000000	1.000
105	GOBP_POSITIVE_REGULATION_OF_PROTEIN_TYROSINE_K...	GO:0061098	5	0.316145	0.717369	0.877863	1.000000	1.000
106	GOBP_REGULATION_OF_ANATOMICAL_STRUCTURE_SIZE	GO:0090066	10	0.230769	0.716076	0.865285	1.000000	1.000
107	GOBP_CHEMICAL_SYNAPTIC_TRANSMISSION_POSTSYNAPTIC	GO:0099565	9	0.240506	0.715221	0.861386	1.000000	1.000
108	HALLMARK_APOPTOSIS	M5902	6	0.287659	0.713471	0.861905	1.000000	1.000
109	GOBP_NEGATIVE_REGULATION_OF_CATABOLIC_PROCESS	GO:0009895	8	0.248459	0.701080	0.873096	1.000000	1.000
110	GOBP_NEURON_MIGRATION	GO:0001764	5	0.298133	0.699933	0.875000	1.000000	1.000
111	GOBP_AMYLOID_BETA_METABOLIC_PROCESS	GO:0050435	5	0.289157	0.693705	0.902985	1.000000	1.000
112	GOBP_ORGANIC_ACID_TRANSPORT	GO:0015849	7	0.259259	0.693472	0.918803	1.000000	1.000
113	GOBP_EPHRIN_RECEPTOR_SIGNALING_PATHWAY	GO:0048013	5	0.301205	0.692274	0.870722	1.000000	1.000
114	GOBP_NEGATIVE_REGULATION_OF_IMMUNE_SYSTEM_PROCESS	GO:0002683	5	0.292511	0.686598	0.910156	1.000000	1.000
115	GOBP_AMYLOID_PRECURSOR_PROTEIN_CATABOLIC_PROCESS	GO:0042987	5	0.289157	0.674389	0.921162	1.000000	1.000
116	GOBP_AMYLOID_PRECURSOR_PROTEIN_METABOLIC_PROCESS	GO:0042982	5	0.289157	0.671919	0.912281	1.000000	1.000
117	GOBP_REGULATION_OF_NEUROTRANSMITTER_RECEPTOR_A...	GO:0099601	13	0.186667	0.670323	0.946746	1.000000	1.000
118	GOBP_REGULATION_OF_CATION_CHANNEL_ACTIVITY	GO:2001257	14	0.189189	0.662374	0.935065	1.000000	1.000
119	GOBP_AMINO_ACID_TRANSPORT	GO:0006865	6	0.256098	0.648466	0.946154	1.000000	1.000
120	GOBP_CARBOXYLIC_ACID_TRANSPORT	GO:0046942	6	0.256098	0.635214	0.956863	1.000000	1.000
121	GOBP_NEURON_PROJECTION_ORGANIZATION	GO:0106027	8	0.221662	0.634519	0.965812	1.000000	1.000
122	GOBP_BIOLOGICAL_ADHESION	GO:0022610	19	0.166682	0.627984	0.982301	1.000000	1.000
123	GOBP_ORGANIC_ANION_TRANSPORT	GO:0015711	6	0.256098	0.617411	0.968000	1.000000	1.000
124	GOBP_ACTIN_FILAMENT_BASED_PROCESS	GO:0030029	7	0.222222	0.612260	0.942478	1.000000	1.000
125	GOBP_POSTSYNAPSE_ORGANIZATION	GO:0099173	10	0.191677	0.603822	0.972527	0.996679	1.000
126	GOBP_REGULATION_OF_NERVOUS_SYSTEM_PROCESS	GO:0031644	8	0.201793	0.577702	0.985222	0.999113	1.000

Continuation of Table **S18**

	NAME	ID	SIZE	ES	NES	NOM.p.val	FDR.q.val	FWER.p.val
127	GOBP_REGULATION_OF_PROTEIN_LOCALIZATION_TO_CEL...	GO:1904375	6	0.222314	0.552862	0.991803	0.998223	1.000
128	GOBP_REGULATION_OF_PROTEIN_LOCALIZATION_TO_MEM...	GO:1905475	7	0.198703	0.531375	0.995745	0.994638	1.000
129	GOBP_STRIATED_MUSCLE_CELL_DIFFERENTIATION	GO:0051146	9	-0.193886	-0.450544	0.993703	0.996086	1.000
130	GOBP_REGULATION_OF_SYSTEM_PROCESS	GO:0044057	18	-0.172399	-0.467421	0.991842	0.995836	1.000
131	GOBP_CELL_JUNCTION_ORGANIZATION	GO:0034330	19	-0.174643	-0.476901	0.982778	0.996045	1.000
132	GOBP_REGULATION_OF_SECRETION	GO:0051046	14	-0.197071	-0.507269	0.980347	0.991733	1.000
133	GOBP_NEGATIVE_REGULATION_OF_TRANSMEMBRANE_TRAN...	GO:0034763	6	-0.247119	-0.508387	0.985526	0.993150	1.000
134	GOBP_NEGATIVE_REGULATION_OF_ANION_TRANSPORT	GO:1903792	8	-0.229170	-0.516100	0.982301	0.992868	1.000
135	GOBP_NEUROGENESIS	GO:0022008	40	-0.172650	-0.525990	0.969116	0.991707	1.000
136	GOBP_POSITIVE_REGULATION_OF_CELL_PROJECTION_OR...	GO:0031346	10	-0.223183	-0.532551	0.974201	0.991232	1.000
137	GOBP_LIPID_EXPORT_FROM_CELL	GO:0140353	5	-0.277850	-0.543986	0.991946	0.988855	1.000
138	GOBP_REGULATION_OF_NERVOUS_SYSTEM_DEVELOPMENT	GO:0051960	15	-0.209369	-0.555603	0.954076	0.985927	1.000
139	GOBP_REGULATION_OF_CELL_DEVELOPMENT	GO:0060284	15	-0.210868	-0.556547	0.963006	0.987243	1.000
140	GOBP_ORGANONITROGEN_COMPOUND_CATABOLIC_PROCESS	GO:1901565	9	-0.240705	-0.557480	0.974328	0.988560	1.000
141	GOBP_CELLULAR_AMIDE_METABOLIC_PROCESS	GO:0043603	13	-0.219976	-0.561018	0.949701	0.988799	1.000
142	GOBP_PROTEIN_DEPHOSPHORYLATION	GO:0006470	7	-0.259362	-0.561054	0.971286	0.990497	1.000
143	GOBP_DEPHOSPHORYLATION	GO:0016311	7	-0.259362	-0.566792	0.967363	0.989674	1.000
144	GOBP_ION_HOMEOSTASIS	GO:0050801	16	-0.220759	-0.578842	0.949425	0.985556	1.000
145	GOBP_PEPTIDE_METABOLIC_PROCESS	GO:0006518	12	-0.228444	-0.579699	0.945783	0.986823	1.000
146	GOBP_REGULATION_OF_NEUROGENESIS	GO:0050767	14	-0.225653	-0.581275	0.950178	0.987651	1.000
147	GOBP_POSITIVE_REGULATION_OF_HYDROLASE_ACTIVITY	GO:0051345	16	-0.220654	-0.581678	0.957176	0.989182	1.000
148	GOBP_CIRCULATORY_SYSTEM_PROCESS	GO:0003013	5	-0.293884	-0.586455	0.961379	0.988360	1.000
149	GOBP_ADENYLATE_CYCLASE_INHIBITING_G_PROTEIN_CO...	GO:0007196	5	-0.296749	-0.586690	0.975477	0.989960	1.000
150	GOBP_FATTY_ACID_TRANSPORT	GO:0015908	6	-0.286086	-0.588928	0.953333	0.990332	1.000
151	GOBP_DENDRITE_DEVELOPMENT	GO:0016358	9	-0.253643	-0.590114	0.948466	0.991353	1.000
152	GOBP_REGULATION_OF_CELLULAR_COMPONENT_BIOGENESIS	GO:0044087	10	-0.254006	-0.593447	0.941919	0.991163	1.000
153	REACTOME_TRANSCRIPTIONAL_REGULATION_BY_MECP2	R-RNO-8986944	7	-0.271605	-0.596366	0.951838	0.991160	1.000
154	GOBP_SKIN_DEVELOPMENT	GO:0043588	5	-0.306130	-0.599186	0.965326	0.991159	1.000
155	GOBP_CELL_CELL_SIGNALING	GO:0007267	56	-0.200858	-0.605848	0.929240	0.988675	1.000
156	GOBP_PROTEIN_CATABOLIC_PROCESS	GO:0030163	8	-0.265234	-0.607355	0.944374	0.989458	1.000
157	GOBP_ASSOCIATIVE_LEARNING	GO:0008306	7	-0.282554	-0.611691	0.958442	0.988355	1.000
158	GOBP_SECOND_MESSENGER_MEDIATED_SIGNALING	GO:0019932	16	-0.230918	-0.613348	0.931634	0.989010	1.000
159	GOBP_MITOCHONDRION_ORGANIZATION	GO:0007005	7	-0.283342	-0.614331	0.954068	0.990113	1.000
160	GOBP_PROTEIN_CONTAINING_COMPLEX_SUBUNIT_ORGANI...	GO:0043933	14	-0.237607	-0.620384	0.928489	0.987466	1.000
161	GOBP_DICARBOXYLIC_ACID_TRANSPORT	GO:0006835	5	-0.320166	-0.622395	0.931081	0.987843	1.000
162	GOBP_MONOCARBOXYLIC_ACID_TRANSPORT	GO:0015718	5	-0.320166	-0.628881	0.952957	0.984885	1.000
163	GOBP_PEPTIDE_SECRETION	GO:0002790	9	-0.268755	-0.629794	0.916250	0.985992	1.000
164	GOBP_RESPONSE_TO_ACID_CHEMICAL	GO:0001101	6	-0.301644	-0.632268	0.918314	0.986058	1.000
165	GOBP_ADENYLATE_CYCLASE_INHIBITING_G_PROTEIN_CO...	GO:0007193	9	-0.275620	-0.636440	0.915276	0.984598	1.000
166	GOBP_ACIDIC_AMINO_ACID_TRANSPORT	GO:0015800	5	-0.320166	-0.637038	0.940781	0.985917	1.000
167	GOBP_POSITIVE_REGULATION_OF_SYNAPTIC_TRANSMISSION	GO:0050806	15	-0.244579	-0.640077	0.914520	0.985308	1.000
168	GOBP_REGULATION_OF_TRANSPORTER_ACTIVITY	GO:0032409	17	-0.239102	-0.642434	0.905512	0.985183	1.000
169	GOBP_CARBOHYDRATE_DERIVATIVE_METABOLIC_PROCESS	GO:1901135	7	-0.298604	-0.644311	0.924552	0.985458	1.000

Continuation of Table **S18**

	NAME	ID	SIZE	ES	NES	NOM.p.val	FDR.q.val	FWER.p.val
170	REACTOME_INTERLEUKIN_4_AND_INTERLEUKIN_13_SIGN...	R-RNO-6785807	8	-0.283977	-0.645245	0.922597	0.986452	1.000
171	GOBP_METAL_ION_HOMEOSTASIS	GO:0055065	14	-0.250432	-0.646316	0.902074	0.987338	1.000
172	GOBP_POSITIVE_REGULATION_OF_CELLULAR_COMPONENT...	GO:0044089	9	-0.280358	-0.650148	0.911250	0.985935	1.000
173	GOBP_REGULATION_OF_HYDROLASE_ACTIVITY	GO:0051336	22	-0.229194	-0.651949	0.885301	0.986164	1.000
174	GOBP_REGULATION_OF_PEPTIDYL_SERINE_PHOSPHORYLA...	GO:0033135	8	-0.289440	-0.655576	0.912769	0.984907	1.000
175	GOBP_POSITIVE_REGULATION_OF_PEPTIDYL_SERINE_PH...	GO:0033138	8	-0.289440	-0.657043	0.908000	0.985366	1.000
176	GOBP_REGULATION_OF_NEURON_PROJECTION_DEVELOPMENT	GO:0010975	16	-0.249055	-0.663219	0.892236	0.981600	1.000
177	GOBP_HORMONE_TRANSPORT	GO:0009914	9	-0.286605	-0.665599	0.878079	0.981199	1.000
178	REACTOME_DAG_AND_IP3_SIGNALING	R-RNO-1489509	8	-0.295281	-0.665896	0.908755	0.982772	1.000
179	GOBP_CELLULAR_ION_HOMEOSTASIS	GO:0006873	14	-0.257362	-0.665943	0.878394	0.984546	1.000
180	GOBP_NEURON_DIFFERENTIATION	GO:0030182	36	-0.225736	-0.666950	0.905782	0.985438	1.000
181	GOBP_NEURON_DEVELOPMENT	GO:0048666	33	-0.225949	-0.670011	0.896368	0.984431	1.000
182	GOBP_LEUKOCYTE_CELL_CELL_ADHESION	GO:0007159	6	-0.319552	-0.675286	0.911023	0.981105	1.000
183	GOBP_NEGATIVE_REGULATION_OF_PROTEIN_CATABOLIC_...	GO:0042177	5	-0.340745	-0.675607	0.909934	0.982634	1.000
184	GOBP_POSITIVE_REGULATION_OF_CELL_ADHESION	GO:0045785	7	-0.313810	-0.677726	0.902622	0.982364	1.000
185	REACTOME_ASSEMBLY_AND_CELL_SURFACE_PRESENTATIO...	R-RNO-9609736	8	-0.306355	-0.679803	0.883895	0.982143	1.000
186	GOBP_REGULATION_OF_HORMONE_SECRETION	GO:0046883	8	-0.297974	-0.681255	0.886650	0.982428	1.000
187	GOBP_PROTEIN_LOCALIZATION_TO_MEMBRANE	GO:0072657	14	-0.260992	-0.683930	0.867731	0.981563	1.000
188	GOBP_INSULIN_SECRETION	GO:0030073	7	-0.319996	-0.688753	0.866841	0.978463	1.000
189	GOBP_REGULATION_OF_PEPTIDE_HORMONE_SECRETION	GO:0090276	6	-0.333844	-0.690467	0.865591	0.978424	1.000
190	GOBP_MUSCLE_CELL_PROLIFERATION	GO:0033002	8	-0.307317	-0.691654	0.861935	0.979046	1.000
191	GOBP_LIPID_LOCALIZATION	GO:0010876	10	-0.293993	-0.691669	0.869148	0.980880	1.000
192	GOBP_CELL_JUNCTION_ASSEMBLY	GO:0034329	8	-0.309910	-0.692878	0.888889	0.981458	1.000
193	GOBP_CELLULAR_RESPONSE_TO_ALCOHOL	GO:0097306	5	-0.355466	-0.694041	0.889972	0.982078	1.000
194	REACTOME_NEGATIVE_REGULATION_OF_NMDA_RECEPTOR_...	R-HSA-961732	8	-0.306355	-0.694702	0.875312	0.983265	1.000
195	GOBP_CELL_CELL_ADHESION	GO:0098609	14	-0.271572	-0.695653	0.861111	0.984088	1.000
196	GOBP_REGULATION_OF_TRANS_SYNAPTIC_SIGNALING	GO:0099177	40	-0.228364	-0.698575	0.864693	0.982972	1.000
197	GOBP_REGULATION_OF_MAP_KINASE_ACTIVITY	GO:0043405	9	-0.294355	-0.699447	0.851485	0.983886	1.000
198	REACTOME_SYNAPTIC_ADHESION_LIKE_MOLECULES	R-RNO-8849932	9	-0.309940	-0.704381	0.864030	0.980168	1.000
199	GOBP_EPIDERMIS_DEVELOPMENT	GO:0008544	5	-0.357499	-0.705539	0.878146	0.980728	1.000
200	GOBP_PEPTIDE_HORMONE_SECRETION	GO:0030072	7	-0.319996	-0.706593	0.865359	0.981415	1.000
201	GOBP_MUSCLE_CELL_DEVELOPMENT	GO:0055001	5	-0.358724	-0.707120	0.872801	0.982723	1.000
202	GOBP_REGULATION_OF_PEPTIDE_SECRETION	GO:0002791	7	-0.322934	-0.710476	0.868594	0.980845	1.000
203	REACTOME_DEVELOPMENTAL_BIOLOGY	R-RNO-1266738	23	-0.248339	-0.711282	0.846847	0.981836	1.000
204	GOBP_REGULATION_OF_CELL_ADHESION	GO:0030155	13	-0.281693	-0.715923	0.861575	0.978361	1.000
205	GOBP_CELL_SURFACE_RECEPTOR_SIGNALING_PATHWAY_I...	GO:1905114	15	-0.280164	-0.716640	0.842410	0.979387	1.000
206	GOBP_PROTEIN_LOCALIZATION_TO_CELL_PERIPHERY	GO:1990778	12	-0.287609	-0.717736	0.817073	0.979912	1.000
207	GOBP_ACTIVATION_OF_PROTEIN_KINASE_ACTIVITY	GO:0032147	11	-0.292900	-0.721006	0.819036	0.978002	1.000
208	GOBP_NEGATIVE_REGULATION_OF_PROTEOLYSIS	GO:0045861	8	-0.319944	-0.721914	0.841495	0.978841	1.000
209	REACTOME_ACTIVATION_OF_NMDA_RECEPTORS_AND_POST...	R-RNO-442755	16	-0.277294	-0.728393	0.819840	0.973131	1.000
210	REACTOME_CLASS_C_3_METABOTROPIC_GLUTAMATE_PHER...	R-RNO-420499	7	-0.335924	-0.729184	0.856579	0.974083	1.000
211	GOBP_CELL_PROJECTION_ORGANIZATION	GO:0030030	34	-0.245045	-0.730975	0.841202	0.973709	1.000
212	GOBP_REGULATION_OF_TRANSPORT	GO:0051049	44	-0.243744	-0.732220	0.834547	0.974001	1.000

Continuation of Table **S18**

	NAME	ID	SIZE	ES	NES	NOM.p.val	FDR.q.val	FWER.p.val
213	GOBP_NEGATIVE_REGULATION_OF_APOPTOTIC_SIGNALIN...	GO:2001234	7	-0.336986	-0.735430	0.849333	0.971962	1.000
214	GOBP_CELL_CELL_JUNCTION_ORGANIZATION	GO:0045216	6	-0.353998	-0.739752	0.856369	0.968332	1.000
215	GOBP_CELLULAR_COMPONENT_DISASSEMBLY	GO:0022411	6	-0.358087	-0.740828	0.822517	0.968903	1.000
216	REACTOME_LONG_TERM_POTENTIATION	R-HSA-9620244	10	-0.308734	-0.741850	0.817848	0.969468	1.000
217	GOBP_NEGATIVE_REGULATION_OF_CELL_CYCLE	GO:0045786	5	-0.371225	-0.746387	0.843490	0.965306	1.000
218	GOBP_NEGATIVE_REGULATION_OF_TRANSPORT	GO:0051051	13	-0.299604	-0.755241	0.831982	0.955444	1.000
219	GOBP_COGNITION	GO:0050890	28	-0.257377	-0.755861	0.797400	0.956504	1.000
220	REACTOME_GPCR_LIGAND_BINDING	R-RNO-500792	8	-0.335623	-0.756539	0.807789	0.957473	1.000
221	GOBP_LONG_TERM_MEMORY	GO:0007616	6	-0.367677	-0.756970	0.831117	0.958827	1.000
222	GOBP_MEMBRANE_PROTEIN_PROTEOLYSIS	GO:0033619	6	-0.370276	-0.757663	0.822997	0.959841	1.000
223	GOBP_AMIDE_BIOSYNTHETIC_PROCESS	GO:0043604	7	-0.351409	-0.760924	0.809403	0.957247	1.000
224	REACTOME_MAPK_FAMILY_SIGNALING_CASCADES	R-RNO-5683057	12	-0.309979	-0.765655	0.792683	0.952570	1.000
225	GOBP_REGULATION_OF_CELL_PROJECTION_ORGANIZATION	GO:0031344	20	-0.274160	-0.766607	0.789354	0.953140	1.000
226	GOBP_ORGANOPHOSPHATE_METABOLIC_PROCESS	GO:0019637	7	-0.357044	-0.767682	0.803364	0.953624	1.000
227	REACTOME_UNBLOCKING_OF_NMDA_RECEPTORS_GLUTAMAT...	R-RNO-438066	12	-0.312925	-0.767774	0.801921	0.955432	1.000
228	GOBP_PEPTIDE_BIOSYNTHETIC_PROCESS	GO:0043043	7	-0.351409	-0.775069	0.794308	0.946976	1.000
229	GOBP_SECRETION	GO:0046903	24	-0.271511	-0.776448	0.790466	0.946980	1.000
230	GOBP_REGULATION_OF_CELLULAR_AMIDE_METABOLIC_PR...	GO:0034248	9	-0.335781	-0.780293	0.790932	0.943343	1.000
231	REACTOME_NERVOUS_SYSTEM_DEVELOPMENT	R-RNO-9675108	15	-0.301423	-0.783244	0.796296	0.941063	1.000
232	GOBP_EXCITATORY_CHEMICAL_SYNAPTIC_TRANSMISSION	GO:0098976	5	-0.390909	-0.783407	0.790368	0.942759	1.000
233	GOBP_CELL_MORPHOGENESIS	GO:0000902	24	-0.277341	-0.785549	0.789130	0.941600	1.000
234	GOBP_REGULATION_OF_SYNAPTIC_PLASTICITY	GO:0048167	21	-0.276833	-0.786668	0.771271	0.941844	1.000
235	GOBP_MEMORY	GO:0007613	13	-0.308508	-0.786725	0.750296	0.943711	1.000
236	GOBP_REGULATION_OF_INFLAMMATORY_RESPONSE	GO:0050727	7	-0.365516	-0.789266	0.755442	0.941935	1.000
237	GOBP_REGULATION_OF_PROTEIN_MODIFICATION_PROCESS	GO:0031399	29	-0.266413	-0.789312	0.760087	0.943819	1.000
238	GOBP_SMALL_GTPASE_MEDIATED_SIGNAL_TRANSDUCTION	GO:0007264	8	-0.350595	-0.789430	0.772953	0.945597	1.000
239	GOBP_GAMETE_GENERATION	GO:0007276	9	-0.339026	-0.790113	0.764115	0.946562	1.000
240	GOBP_CELL_PART_MORPHOGENESIS	GO:0032990	24	-0.277341	-0.790308	0.766703	0.948243	1.000
241	GOBP_CELLULAR_COMPONENT_MORPHOGENESIS	GO:0032989	24	-0.277341	-0.791355	0.783814	0.948707	1.000
242	GOBP_REGULATION_OF_DEFENSE_RESPONSE	GO:0031347	7	-0.365516	-0.792119	0.761528	0.949491	1.000
243	GOBP_REGULATION_OF_CATABOLIC_PROCESS	GO:0009894	13	-0.308806	-0.792138	0.751790	0.951452	1.000
244	GOBP_LEARNING	GO:0007612	13	-0.315778	-0.793315	0.757757	0.951690	1.000
245	GOBP_REGULATION_OF_VESICLE_MEDIATED_TRANSPORT	GO:0060627	12	-0.317547	-0.795673	0.755924	0.949953	1.000
246	REACTOME_TRANSCRIPTIONAL_REGULATION_OF_WHITE_A...	R-RNO-381340	6	-0.379745	-0.796041	0.764398	0.951452	1.000
247	GOBP_POSITIVE_REGULATION_OF_DEFENSE_RESPONSE	GO:0031349	5	-0.401263	-0.798473	0.799180	0.949814	1.000
248	GOBP_POSITIVE_REGULATION_OF_PHOSPHATIDYLINOSIT...	GO:0014068	5	-0.404513	-0.799418	0.769022	0.950426	1.000
249	REACTOME_SIGNALING_BY_INTERLEUKINS	R-RNO-449147	15	-0.308748	-0.801534	0.762791	0.949333	1.000
250	GOBP_POSITIVE_REGULATION_OF_MAP_KINASE_ACTIVITY	GO:0043406	6	-0.389175	-0.802827	0.768246	0.949300	1.000
251	GOBP_PROTEIN_LOCALIZATION_TO_CELL_JUNCTION	GO:1902414	6	-0.384987	-0.803630	0.764554	0.950121	1.000
252	GOBP_POLYSACCHARIDE_METABOLIC_PROCESS	GO:0005976	5	-0.407785	-0.804296	0.759891	0.951130	1.000
253	GOBP_PROTEOLYSIS	GO:0006508	17	-0.296938	-0.805342	0.739380	0.951605	1.000
254	GOBP_CELL_CELL_JUNCTION_ASSEMBLY	GO:0007043	5	-0.411711	-0.805352	0.763305	0.953634	1.000
255	GOBP_ACTIVATION_OF_MAPK_ACTIVITY	GO:0000187	6	-0.389175	-0.807223	0.751989	0.952631	1.000

Continuation of Table **S18**

	NAME	ID	SIZE	ES	NES	NOM.p.val	FDR.q.val	FWER.p.val
256	GOBP_REGULATION_OF_PROTEIN_PHOSPHORYLATION	GO:0001932	26	-0.278752	-0.812441	0.740331	0.946535	1.000
257	GOBP_REGULATION_OF_INTRACELLULAR_SIGNAL_TRANSD...	GO:1902531	21	-0.293087	-0.817928	0.728285	0.939690	1.000
258	GOBP_NEGATIVE_REGULATION_OF_ION_TRANSPORT	GO:0043271	11	-0.335574	-0.819094	0.709070	0.939871	1.000
259	GOBP_MACROMOLECULE_CATABOLIC_PROCESS	GO:0009057	12	-0.329430	-0.823257	0.719812	0.935144	1.000
260	GOBP_ORGANONITROGEN_COMPOUND_BIOSYNTHETIC_PROCESS	GO:1901566	13	-0.323550	-0.826726	0.713951	0.931513	1.000
261	GOBP_RESPIRATORY_SYSTEM_DEVELOPMENT	GO:0060541	6	-0.393648	-0.827205	0.752604	0.932752	1.000
262	GOBP_CELL_MORPHOGENESIS_INVOLVED_IN_NEURON_DIF...	GO:0048667	21	-0.295704	-0.827959	0.709030	0.933445	1.000
263	GOBP_POSITIVE_REGULATION_OF_CATALYTIC_ACTIVITY	GO:0043085	30	-0.285548	-0.831598	0.726681	0.929525	1.000
264	GOBP_FOREBRAIN_DEVELOPMENT	GO:0030900	9	-0.357521	-0.832151	0.706897	0.930642	1.000
265	GOBP_SEXUAL_REPRODUCTION	GO:0019953	10	-0.352724	-0.834845	0.713759	0.928004	1.000
266	GOBP_RESPONSE_TO_ALCOHOL	GO:0097305	11	-0.347850	-0.838036	0.715651	0.924741	1.000
267	GOBP_POSITIVE_REGULATION_OF_MOLECULAR_FUNCTION	GO:0044093	38	-0.279474	-0.839183	0.722103	0.924795	1.000
268	GOBP_GLUCOSE_METABOLIC_PROCESS	GO:0006006	6	-0.402487	-0.840461	0.730924	0.924688	1.000
269	GOBP_CELL_MORPHOGENESIS_INVOLVED_IN_DIFFERENTI...	GO:0000904	21	-0.295704	-0.841079	0.713969	0.925803	1.000
270	GOBP_POSITIVE_REGULATION_OF_SIGNALING	GO:0023056	34	-0.285893	-0.843108	0.714133	0.924295	1.000
271	GOBP_MAPK_CASCADE	GO:0000165	18	-0.312752	-0.843483	0.701814	0.925699	1.000
272	GOBP_MONOSACCHARIDE_METABOLIC_PROCESS	GO:0005996	6	-0.402487	-0.843875	0.713731	0.927048	1.000
273	GOBP_POSITIVE_REGULATION_OF_PHOSPHORUS_METABOL...	GO:0010562	24	-0.297093	-0.844084	0.727273	0.928755	1.000
274	REACTOME_EXTRACELLULAR_MATRIX_ORGANIZATION	R-RNO-1474244	5	-0.416565	-0.845655	0.700269	0.927979	1.000
275	GOBP_REGULATION_OF_ANATOMICAL_STRUCTURE_MORPHO...	GO:0022603	17	-0.316613	-0.846091	0.703072	0.929297	1.000
276	GOBP_REGULATION_OF_CELLULAR_LOCALIZATION	GO:0060341	17	-0.314426	-0.846493	0.696552	0.930758	1.000
277	REACTOME_SIGNALING_BY_GPCR	R-RNO-372790	22	-0.301464	-0.847945	0.698009	0.930243	1.000
278	GOBP_ADENYLATE_CYCLASE_MODULATING_G_PROTEIN_CO...	GO:0007188	11	-0.340793	-0.848593	0.655422	0.931207	1.000
279	GOBP_CELLULAR_HOMEOSTASIS	GO:0019725	18	-0.312006	-0.848644	0.686164	0.933219	1.000
280	GOBP_AXON_DEVELOPMENT	GO:0061564	18	-0.307699	-0.848666	0.690722	0.935322	1.000
281	GOBP_CENTRAL_NERVOUS_SYSTEM_DEVELOPMENT	GO:0007417	26	-0.297615	-0.852865	0.691548	0.929815	1.000
282	GOBP_NEGATIVE_REGULATION_OF_PHOSPHORUS_METABOL...	GO:0010563	9	-0.367892	-0.855815	0.680798	0.926597	1.000
283	GOBP_VESICLE_MEDIATED_TRANSPORT_IN_SYNAPSE	GO:0099003	7	-0.397977	-0.857149	0.704427	0.926325	1.000
284	GOBP_IMMUNE_EFFECTOR_PROCESS	GO:0002252	10	-0.362676	-0.857198	0.678441	0.928356	1.000
285	GOBP_G_PROTEIN_COUPLED_RECEPTOR_SIGNALING_PATHWAY	GO:0007186	18	-0.315908	-0.858409	0.678330	0.928190	1.000
286	GOBP_MALE_GAMETE_GENERATION	GO:0048232	5	-0.433735	-0.859674	0.660403	0.927937	1.000
287	GOBP_NEURON_APOPTOTIC_PROCESS	GO:0051402	12	-0.344081	-0.862356	0.658019	0.925077	1.000
288	GOBP_ACTIVATION_OF_IMMUNE_RESPONSE	GO:0002253	5	-0.435676	-0.867597	0.684931	0.917538	1.000
289	GOBP_NEGATIVE_REGULATION_OF_CYSTEINE_TYPE_ENDO...	GO:2000117	5	-0.438609	-0.874406	0.682256	0.906974	1.000
290	REACTOME_VESICLE_MEDIATED_TRANSPORT	R-RNO-5653656	5	-0.443327	-0.874984	0.678238	0.907972	1.000
291	REACTOME_G_ALPHA_I_SIGNALLING_EVENTS	R-RNO-418594	18	-0.317548	-0.875680	0.649718	0.908745	1.000
292	GOBP_CELL_GROWTH	GO:0016049	10	-0.367305	-0.876368	0.655340	0.909546	1.000
293	GOBP_NEGATIVE_REGULATION_OF_SYNAPTIC_TRANSMISSION	GO:0050805	6	-0.419560	-0.877738	0.646597	0.909204	1.000
294	GOBP_GLAND_DEVELOPMENT	GO:0048732	7	-0.408347	-0.878649	0.648825	0.909678	1.000
295	GOBP_REGULATION_OF_BINDING	GO:0051098	11	-0.359697	-0.879989	0.644150	0.909223	1.000
296	GOBP_POSITIVE_REGULATION_OF_PROTEIN_MODIFICATI...	GO:0031401	23	-0.313212	-0.880207	0.656319	0.910960	1.000
297	GOBP_RESPONSE_TO_NUTRIENT	GO:0007584	5	-0.445783	-0.881715	0.665757	0.910143	1.000
298	GOBP_EMBRYO_DEVELOPMENT_ENDING_IN_BIRTH_OR_EGG...	GO:0009792	9	-0.382137	-0.884617	0.657500	0.906398	1.000

Continuation of Table **S18**

	NAME	ID	SIZE	ES	NES	NOM.p.val	FDR.q.val	FWER.p.val
299	GOBP_CARBOHYDRATE_METABOLIC_PROCESS	GO:0005975	9	-0.380630	-0.884891	0.647131	0.908019	1.000
300	GOBP_IONOTROPIC_GLUTAMATE_RECEPTOR_SIGNALING_P...	GO:0035235	9	-0.376037	-0.885641	0.656289	0.908661	1.000
301	REACTOME_MEMBRANE_TRAFFICKING	R-RNO-199991	5	-0.443327	-0.886122	0.659459	0.909926	1.000
302	GOBP_REGULATION_OF_PHOSPHORUS_METABOLIC_PROCESS	GO:0051174	27	-0.304471	-0.886521	0.660870	0.911319	1.000
303	GOBP_RESPONSE_TO_UV	GO:0009411	5	-0.442483	-0.886539	0.661517	0.913488	1.000
304	GOBP_OSTEOBLAST_DIFFERENTIATION	GO:0001649	5	-0.454280	-0.888900	0.657754	0.911010	1.000
305	GOBP_APOPTOTIC_MITOCHONDRIAL_CHANGES	GO:0008637	6	-0.425430	-0.891543	0.656417	0.908019	1.000
306	GOBP_REGULATION_OF_TRANSFERASE_ACTIVITY	GO:0051338	22	-0.319744	-0.892851	0.634938	0.907729	1.000
307	GOBP_EMBRYONIC_MORPHOGENESIS	GO:0048598	6	-0.427338	-0.893156	0.650190	0.909242	1.000
308	GOBP_MYOTUBE_DIFFERENTIATION	GO:0014902	5	-0.452618	-0.894736	0.633609	0.908288	1.000
309	GOBP_POSITIVE_REGULATION_OF_TRANSPORT	GO:0051050	26	-0.311186	-0.895129	0.648291	0.909733	1.000
310	GOBP_POSITIVE_REGULATION_OF_SMALL_MOLECULE_MET...	GO:0062013	5	-0.454852	-0.898450	0.620321	0.905173	1.000
311	GOBP_LONG_TERM_SYNAPTIC_POTENTIATION	GO:0060291	12	-0.363630	-0.899305	0.626478	0.905751	1.000
312	GOBP_NEGATIVE_REGULATION_OF_SIGNALING	GO:0023057	21	-0.324175	-0.900817	0.622047	0.904842	1.000
313	GOBP_PROTEIN_PHOSPHORYLATION	GO:0006468	34	-0.299829	-0.901227	0.620022	0.906226	1.000
314	GOBP_POSITIVE_REGULATION_OF_PROTEIN_PHOSPHORYL...	GO:0001934	23	-0.313212	-0.903069	0.615128	0.904766	1.000
315	GOBP_REGULATION_OF_PROTEIN_KINASE_ACTIVITY	GO:0045859	22	-0.319744	-0.906602	0.607821	0.899690	1.000
316	GOBP_HOMEOSTASIS_OF_NUMBER_OF_CELLS	GO:0048872	5	-0.457813	-0.907640	0.617766	0.899772	1.000
317	GOBP_PROTEIN_LOCALIZATION_TO_POSTSYNAPSE	GO:0062237	5	-0.462052	-0.909168	0.604336	0.898876	1.000
318	GOBP_CELLULAR_MACROMOLECULE_LOCALIZATION	GO:0070727	21	-0.320447	-0.909519	0.600660	0.900366	1.000
319	GOBP_NERVOUS_SYSTEM_PROCESS	GO:0050877	38	-0.303719	-0.910068	0.605042	0.901471	1.000
320	GOBP_REGULATION_OF_LYMPHOCYTE_ACTIVATION	GO:0051249	5	-0.456349	-0.910253	0.613636	0.903377	1.000
321	GOBP_REGULATION_OF_FAT_CELL_DIFFERENTIATION	GO:0045598	6	-0.438605	-0.912003	0.613423	0.901912	1.000
322	GOBP_REGULATION_OF_MAPK_CASCADE	GO:0043408	13	-0.361865	-0.912646	0.598566	0.902844	1.000
323	GOBP_RESPONSE_TO_DRUG	GO:0042493	11	-0.370977	-0.913092	0.583738	0.904112	1.000
324	GOBP_INFLAMMATORY_RESPONSE	GO:0006954	12	-0.365702	-0.913767	0.616505	0.904903	1.000
325	GOBP_PROTEIN_LOCALIZATION_TO_SYNAPSE	GO:0035418	5	-0.462052	-0.917417	0.587169	0.899595	1.000
326	GOBP_EPITHELIUM_DEVELOPMENT	GO:0060429	15	-0.351674	-0.919425	0.586247	0.897721	1.000
327	GOBP_WOUND_HEALING	GO:0042060	13	-0.365414	-0.921845	0.586729	0.894894	1.000
328	GOBP_HEAD_DEVELOPMENT	GO:0060322	22	-0.324855	-0.926472	0.583058	0.887190	1.000
329	GOBP_REGULATION_OF_VASCULATURE_DEVELOPMENT	GO:1901342	5	-0.471428	-0.926616	0.560876	0.889206	1.000
330	GOBP_POSITIVE_REGULATION_OF_TRANSFERASE_ACTIVITY	GO:0051347	20	-0.336846	-0.928507	0.570304	0.887589	1.000
331	GOBP_REGULATION_OF_ENDOCYTOSIS	GO:0030100	6	-0.441263	-0.929737	0.600522	0.887242	1.000
332	GOBP_REGULATION_OF_DNA_BINDING_TRANSCRIPTION_F...	GO:0051090	16	-0.349400	-0.931666	0.582367	0.885333	1.000
333	GOBP_LOCOMOTION	GO:0040011	31	-0.316732	-0.934462	0.589071	0.881361	1.000
334	GOBP_BEHAVIOR	GO:0007610	32	-0.313088	-0.935927	0.567308	0.880217	1.000
335	GOBP_REGULATION_OF_PROTEOLYSIS	GO:0030162	12	-0.371230	-0.940270	0.552311	0.872786	1.000
336	GOBP_TEMPERATURE_HOMEOSTASIS	GO:0001659	5	-0.475103	-0.941418	0.575305	0.872600	1.000
337	GOBP_REGULATION_OF_CELL_CELL_ADHESION	GO:0022407	9	-0.406290	-0.941579	0.556391	0.874613	1.000
338	GOBP_POSITIVE_REGULATION_OF_ESTABLISHMENT_OF_P...	GO:1904951	5	-0.479260	-0.942112	0.557952	0.875715	1.000
339	REACTOME_CIRCADIAN_CLOCK	R-HSA-400253	5	-0.487314	-0.949023	0.570442	0.862342	1.000
340	GOBP_RESPONSE_TO_LIGHT_STIMULUS	GO:0009416	11	-0.389385	-0.949427	0.539683	0.863704	1.000
341	GOBP_RESPONSE_TO_WOUNDING	GO:0009611	15	-0.365622	-0.950174	0.546620	0.864447	1.000

Continuation of Table **S18**

	NAME	ID	SIZE	ES	NES	NOM.p.val	FDR.q.val	FWER.p.val
342	GOBP_POSITIVE_REGULATION_OF_GROWTH	GO:0045927	9	-0.404314	-0.950258	0.559343	0.866564	1.000
343	GOBP_ESTABLISHMENT_OF_PROTEIN_LOCALIZATION	GO:0045184	18	-0.349232	-0.951107	0.553022	0.867128	1.000
344	GOBP_DEVELOPMENTAL_GROWTH	GO:0048589	10	-0.401727	-0.951248	0.567308	0.869177	1.000
345	GOBP_REGULATION_OF_GTPASE_ACTIVITY	GO:0043087	5	-0.480972	-0.951959	0.557123	0.869931	1.000
346	REACTOME_LEISHMANIA_INFECTION	R-HSA-9658195	10	-0.398238	-0.952150	0.563659	0.871791	1.000
347	GOBP_IN_UTERO_EMBRYONIC_DEVELOPMENT	GO:0001701	8	-0.425529	-0.952958	0.568702	0.872337	1.000
348	GOBP_NEGATIVE_REGULATION_OF_PROTEIN_MODIFICATI...	GO:0031400	10	-0.406245	-0.954104	0.539855	0.872071	1.000
349	REACTOME_DEATH_RECEPTOR_SIGNALLING	R-RNO-73887	5	-0.486466	-0.955720	0.544952	0.870727	1.000
350	GOBP_NEGATIVE_REGULATION_OF_INTRACELLULAR_SIGN...	GO:1902532	7	-0.437106	-0.958242	0.554140	0.867389	1.000
351	GOBP_RECEPTOR_INTERNALIZATION	GO:0031623	5	-0.494890	-0.958871	0.546961	0.868421	1.000
352	GOBP_G_PROTEIN_COUPLED_GLUTAMATE_RECEPTOR_SIGN...	GO:0007216	8	-0.422644	-0.959570	0.538657	0.869213	1.000
353	GOBP_ORGANIC_ACID_METABOLIC_PROCESS	GO:0006082	7	-0.433558	-0.960023	0.529936	0.870558	1.000
354	GOBP_REGULATION_OF_MUSCLE_CELL_DIFFERENTIATION	GO:0051147	6	-0.465580	-0.961155	0.520750	0.870326	1.000
355	GOBP_GLUTAMATE_RECEPTOR_SIGNALING_PATHWAY	GO:0007215	17	-0.362039	-0.964349	0.534025	0.865538	1.000
356	REACTOME_NEUROTRANSMITTER_RECEPTORS_AND_POSTSY...	R-RNO-112314	22	-0.343138	-0.965251	0.540000	0.865637	1.000
357	GOBP_REGULATION_OF_MUSCLE_SYSTEM_PROCESS	GO:0090257	8	-0.438128	-0.965628	0.540676	0.867176	1.000
358	GOBP_POSITIVE_REGULATION_OF_PROTEIN_METABOLIC_...	GO:0051247	28	-0.332455	-0.965983	0.544858	0.868812	1.000
359	GOBP_REGULATION_OF_DEVELOPMENTAL_GROWTH	GO:0048638	9	-0.422696	-0.967731	0.543226	0.867259	1.000
360	REACTOME_PROTEIN_PROTEIN_INTERACTIONS_AT_SYNAPSES	R-RNO-6794362	12	-0.394165	-0.970692	0.506634	0.862815	1.000
361	GOBP_HEART_DEVELOPMENT	GO:0007507	6	-0.467978	-0.970929	0.529032	0.864687	1.000
362	REACTOME_CYTOKINE_SIGNALING_IN_IMMUNE_SYSTEM	R-RNO-1280215	21	-0.349148	-0.971337	0.547778	0.866156	1.000
363	GOBP_MEMBRANE_ORGANIZATION	GO:0061024	12	-0.392707	-0.971816	0.523298	0.867394	1.000
364	GOBP_SENSORY_ORGAN_DEVELOPMENT	GO:0007423	8	-0.427625	-0.972444	0.508728	0.868306	1.000
365	GOBP_SENSORY_PERCEPTION	GO:0007600	9	-0.409585	-0.972546	0.522670	0.870523	1.000
366	GOBP_POSITIVE_REGULATION_OF_CELLULAR_COMPONENT...	GO:0051130	24	-0.337240	-0.973445	0.524698	0.870984	1.000
367	GOBP_POSITIVE_REGULATION_OF_HEMOPOIESIS	GO:1903708	7	-0.450708	-0.974867	0.538847	0.870171	1.000
368	GOBP_REGULATION_OF_PROTEIN_SERINE_THREONINE_KI...	GO:0071900	12	-0.386944	-0.977065	0.512315	0.867286	1.000
369	GOBP_NEGATIVE_REGULATION_OF_PHOSPHORYLATION	GO:0042326	7	-0.445823	-0.977728	0.512041	0.868159	1.000
370	GOBP_NEGATIVE_REGULATION_OF_TRANSFERASE_ACTIVITY	GO:0051348	5	-0.504454	-0.981067	0.516438	0.862534	1.000
371	GOBP_CARDIAC_MUSCLE_TISSUE_DEVELOPMENT	GO:0048738	6	-0.467978	-0.982297	0.492848	0.862042	1.000
372	GOBP_POSITIVE_REGULATION_OF_PROTEIN_KINASE_ACT...	GO:0045860	19	-0.363393	-0.982850	0.521691	0.863090	1.000
373	GOBP_MONOCARBOXYLIC_ACID_METABOLIC_PROCESS	GO:0032787	6	-0.464644	-0.983229	0.499331	0.864703	1.000
374	GOBP_RECEPTOR_METABOLIC_PROCESS	GO:0043112	5	-0.494890	-0.984874	0.503448	0.863198	1.000
375	GOBP_REGULATION_OF_ION_TRANSPORT	GO:0043269	36	-0.328275	-0.987730	0.494612	0.859025	1.000
376	GOBP_NUCLEAR_TRANSPORT	GO:0051169	5	-0.503324	-0.988208	0.503383	0.860423	1.000
377	REACTOME_INNATE_IMMUNE_SYSTEM	R-RNO-168249	16	-0.370581	-0.989422	0.515789	0.859854	1.000
378	GOBP_CELL_MIGRATION	GO:0016477	22	-0.349330	-0.990111	0.488294	0.860717	1.000
379	GOBP_MUSCLE_CELL_DIFFERENTIATION	GO:0042692	10	-0.409796	-0.991929	0.523694	0.858847	1.000
380	GOBP_NEGATIVE_REGULATION_OF_BINDING	GO:0051100	6	-0.477434	-0.992192	0.494148	0.860729	1.000
381	GOBP_MUSCLE_CONTRACTION	GO:0006936	5	-0.506732	-0.992936	0.487110	0.861403	1.000
382	GOBP_REGULATION_OF_OSTEOCLAST_DIFFERENTIATION	GO:0045670	5	-0.502493	-0.993573	0.487738	0.862413	1.000
383	GOBP_SENSORY_PERCEPTION_OF_PAIN	GO:0019233	5	-0.493976	-0.995700	0.494595	0.859704	1.000
384	GOBP_REGULATION_OF_CELL_DEATH	GO:0010941	34	-0.336609	-0.995738	0.492600	0.862166	1.000

Continuation of Table **S18**

	NAME	ID	SIZE	ES	NES	NOM.p.val	FDR.q.val	FWER.p.val
385	GOBP_REGULATION_OF_ENDOTHELIAL_CELL_MIGRATION	GO:0010594	5	-0.505829	-0.995910	0.496011	0.864317	1.000
386	REACTOME_DISEASES_OF_SIGNAL_TRANSDUCTION_BY_GR...	R-HSA-5663202	12	-0.397706	-0.996564	0.505455	0.865259	1.000
387	GOBP_NEGATIVE_REGULATION_OF_KINASE_ACTIVITY	GO:0033673	5	-0.504454	-0.997571	0.480170	0.865427	1.000
388	GOBP_NEGATIVE_REGULATION_OF_LIPID_METABOLIC_PR...	GO:0045833	5	-0.514950	-0.998249	0.467940	0.866417	1.000
389	GOBP_NEGATIVE_REGULATION_OF_MOLECULAR_FUNCTION	GO:0044092	24	-0.345614	-0.999845	0.481195	0.864976	1.000
390	GOBP_REGULATION_OF_RECEPTOR_MEDIATED_ENDOCYTOSIS	GO:0048259	5	-0.510252	-1.003910	0.487614	0.857114	1.000
391	GOBP_PROTEIN_AUTOPHOSPHORYLATION	GO:0046777	5	-0.513834	-1.005372	0.467480	0.855944	1.000
392	GOBP_RESPONSE_TO_CARBOHYDRATE	GO:0009743	5	-0.512255	-1.007988	0.483266	0.851775	1.000
393	GOBP_PEPTIDYL_SERINE_MODIFICATION	GO:0018209	12	-0.411258	-1.010005	0.460890	0.849207	1.000
394	GOBP_NEGATIVE_REGULATION_OF_CELL_DEVELOPMENT	GO:0010721	6	-0.488130	-1.013867	0.452349	0.841992	1.000
395	GOBP_NEGATIVE_REGULATION_OF_NERVOUS_SYSTEM_DEV...	GO:0051961	5	-0.504016	-1.014013	0.462500	0.844197	1.000
396	GOBP_CHEMICAL_HOMEOSTASIS	GO:0048878	23	-0.352719	-1.014069	0.468397	0.846634	1.000
397	GOBP_RESPONSE_TO_MONOSACCHARIDE	GO:0034284	5	-0.512255	-1.015044	0.466572	0.846628	1.000
398	GOBP_CELLULAR_COMPONENT_MAINTENANCE	GO:0043954	5	-0.513453	-1.017411	0.451268	0.843086	1.000
399	GOBP_NEURON_PROJECTION_GUIDANCE	GO:0097485	9	-0.439338	-1.018113	0.453184	0.843989	1.000
400	GOBP_REGULATION_OF_CELLULAR_CATABOLIC_PROCESS	GO:0031329	10	-0.424600	-1.019401	0.470096	0.843123	1.000
401	REACTOME_INTERFERON_SIGNALING	R-RNO-913531	6	-0.485531	-1.019997	0.451268	0.844221	1.000
402	GOBP_RESPONSE_TO_ALKALOID	GO:0043279	5	-0.518072	-1.022121	0.435247	0.841342	1.000
403	GOBP_REGULATION_OF_EPITHELIAL_CELL_MIGRATION	GO:0010632	6	-0.488591	-1.022388	0.428016	0.843257	1.000
404	GOBP_REGULATION_OF_CELLULAR_COMPONENT_MOVEMENT	GO:0051270	15	-0.392554	-1.022894	0.464692	0.844514	1.000
405	GOBP_POSITIVE_REGULATION_OF_CELL_DIFFERENTIATION	GO:0045597	27	-0.359060	-1.023380	0.454345	0.845900	1.000
406	GOBP_BIOLOGICAL_PROCESS_INVOLVED_IN_SYMBIOTIC_...	GO:004440	11	-0.417840	-1.025857	0.436019	0.841956	1.000
407	GOBP_REGULATION_OF_PHOSPHATIDYLINOSITOL_3_KINA...	GO:0014066	6	-0.491266	-1.026606	0.453595	0.842593	1.000
408	REACTOME_SIGNALING_BY_WNT	R-RNO-195721	7	-0.477680	-1.027590	0.450392	0.842767	1.000
409	GOBP_POSITIVE_REGULATION_OF_ION_TRANSPORT	GO:0043270	17	-0.384523	-1.028080	0.444319	0.844098	1.000
410	GOBP_NEGATIVE_REGULATION_OF_NEURON_APOPTOTIC_P...	GO:0043524	8	-0.460376	-1.029150	0.441890	0.844021	1.000
411	REACTOME_TRANSMISSION_ACROSS_CHEMICAL_SYNAPSES	R-RNO-112315	23	-0.359738	-1.029511	0.448430	0.845740	1.000
412	GOBP_OSSIFICATION	GO:0001503	9	-0.439058	-1.030010	0.434080	0.847156	1.000
413	GOBP_REGULATION_OF_AXONOGENESIS	GO:0050770	7	-0.480227	-1.030538	0.412739	0.848520	1.000
414	GOBP_POSITIVE_REGULATION_OF_MAPK_CASCADE	GO:0043410	9	-0.442603	-1.030539	0.431181	0.851302	1.000
415	REACTOME_CREB1_PHOSPHORYLATION_THROUGH_NMDA_RE...	R-RNO-442742	8	-0.464570	-1.034443	0.424051	0.843484	1.000
416	GOBP_REGULATION_OF_PROTEIN_BINDING	GO:0043393	7	-0.476756	-1.038274	0.439791	0.835977	1.000
417	GOBP_NEGATIVE_REGULATION_OF_MULTICELLULAR_ORGA...	GO:0051241	15	-0.401193	-1.038663	0.422170	0.837663	1.000
418	GOBP_REGULATION_OF_ORGAN_GROWTH	GO:0046620	5	-0.529944	-1.039577	0.425474	0.838028	1.000
419	GOBP_POSITIVE_REGULATION_OF_AXONOGENESIS	GO:0050772	5	-0.522941	-1.040028	0.430556	0.839466	1.000
420	REACTOME_G_ALPHA_Z_SIGNALLING_EVENTS	R-RNO-418597	5	-0.528100	-1.040966	0.423611	0.839653	1.000
421	GOBP_MUSCLE_STRUCTURE_DEVELOPMENT	GO:0061061	16	-0.393444	-1.041659	0.451835	0.840629	1.000
422	GOBP_REGULATION_OF_LIPID_LOCALIZATION	GO:1905952	5	-0.530397	-1.041910	0.423868	0.842783	1.000
423	GOBP_POSITIVE_REGULATION_OF_DEVELOPMENTAL_GROWTH	GO:0048639	8	-0.468508	-1.043629	0.422879	0.841062	1.000
424	GOBP_PHOSPHATIDYLINOSITOL_3_KINASE_SIGNALING	GO:0014065	7	-0.477351	-1.045551	0.417344	0.838712	1.000
425	GOBP_LIPID_METABOLIC_PROCESS	GO:0006629	10	-0.437588	-1.046918	0.436518	0.837895	1.000
426	REACTOME_NEUREXINS_AND_NEUROLIGINS	R-RNO-6794361	9	-0.449711	-1.047289	0.440594	0.839715	1.000
427	GOBP_RESPONSE_TO_MECHANICAL_STIMULUS	GO:0009612	6	-0.509930	-1.049657	0.397117	0.835702	1.000

Continuation of Table **S18**

	NAME	ID	SIZE	ES	NES	NOM.p.val	FDR.q.val	FWER.p.val
428	GOBP_POSITIVE_REGULATION_OF_INTRACELLULAR_SIGN...	GO:1902533	14	-0.410030	-1.054073	0.418033	0.826174	1.000
429	GOBP_INTRACELLULAR_PROTEIN_TRANSPORT	GO:0006886	9	-0.459869	-1.056107	0.413580	0.823356	1.000
430	GOBP_CYTOSKELETON_ORGANIZATION	GO:0007010	11	-0.439503	-1.057694	0.409976	0.822015	1.000
431	GOBP_INOSITOL_LIPID_MEDIATED_SIGNALING	GO:0048017	8	-0.475753	-1.058219	0.409439	0.823317	1.000
432	GOBP_CELLULAR_CARBOHYDRATE_METABOLIC_PROCESS	GO:0044262	5	-0.533794	-1.059430	0.398929	0.822864	1.000
433	GOBP_EMBRYONIC_ORGAN_DEVELOPMENT	GO:0048568	7	-0.483291	-1.059645	0.385101	0.825207	1.000
434	GOBP_ORGAN_GROWTH	GO:0035265	5	-0.529944	-1.059876	0.404167	0.827399	1.000
435	REACTOME_BETA_CATENIN_INDEPENDENT_WNT_SIGNALING	R-RNO-3858494	5	-0.531400	-1.063301	0.370421	0.820622	1.000
436	GOBP_NEGATIVE_REGULATION_OF_CATALYTIC_ACTIVITY	GO:0043086	17	-0.401790	-1.065959	0.405780	0.815934	1.000
437	GOBP_REGULATION_OF_PEPTIDE_TRANSPORT	GO:0090087	10	-0.451905	-1.066714	0.390036	0.816556	1.000
438	GOBP_APOPTOTIC_PROCESS	GO:0006915	35	-0.353009	-1.067269	0.404482	0.817778	1.000
439	GOBP_REGULATION_OF_APOPTOTIC_SIGNALING_PATHWAY	GO:2001233	10	-0.447476	-1.067539	0.397546	0.819883	1.000
440	REACTOME_L1CAM_INTERACTIONS	R-RNO-373760	5	-0.544547	-1.067551	0.399171	0.822785	1.000
441	GOBP_POSITIVE_REGULATION_OF_ORGANELLE_ORGANIZA...	GO:0010638	10	-0.451521	-1.067589	0.394673	0.825649	1.000
442	GOBP_REGULATION_OF_MULTICELLULAR_ORGANISMAL_DE...	GO:2000026	31	-0.357134	-1.075588	0.380851	0.805237	1.000
443	GOBP_REGULATION_OF_CYTOSKELETON_ORGANIZATION	GO:0051493	5	-0.540803	-1.075615	0.349933	0.808092	1.000
444	GOBP_RESPONSE_TO_NERVE_GROWTH_FACTOR	GO:1990089	7	-0.491605	-1.076152	0.372215	0.809546	1.000
445	GOBP_NERVE_DEVELOPMENT	GO:0021675	9	-0.463842	-1.076488	0.401741	0.811619	1.000
446	GOBP_RHYTHMIC_PROCESS	GO:0048511	15	-0.415136	-1.080386	0.377598	0.803425	1.000
447	GOBP_ENDOCRINE_SYSTEM_DEVELOPMENT	GO:0035270	6	-0.517580	-1.080770	0.356784	0.805173	1.000
448	GOBP_RESPONSE_TO_EXTRACELLULAR_STIMULUS	GO:0009991	13	-0.423274	-1.081271	0.371328	0.806764	1.000
449	GOBP_POSITIVE_REGULATION_OF_MYELOID_CELL_DIFFE...	GO:0045639	7	-0.505257	-1.081700	0.374359	0.808540	1.000
450	GOBP_FAT_CELL_DIFFERENTIATION	GO:0045444	9	-0.461689	-1.086858	0.354061	0.796433	1.000
451	GOBP_NEGATIVE_REGULATION_OF_RESPONSE_TO_EXTERN...	GO:0032102	5	-0.552747	-1.092012	0.363874	0.784385	1.000
452	GOBP_REGULATION_OF_PEPTIDASE_ACTIVITY	GO:0052547	10	-0.461979	-1.092486	0.366748	0.785910	1.000
453	GOBP_INTRINSIC_APOPTOTIC_SIGNALING_PATHWAY	GO:0097193	5	-0.546028	-1.092903	0.337802	0.787705	1.000
454	GOBP_NUCLEOBASE_CONTAINING_SMALL_MOLECULE_META...	GO:0055086	6	-0.522361	-1.093808	0.342704	0.787886	1.000
455	GOBP_MATERNAL_PROCESS_INVOLVED_IN_FEMALE_PREGN...	GO:0060135	5	-0.547650	-1.094254	0.360811	0.789575	1.000
456	GOBP_RECEPTOR_MEDIATED_ENDOCYTOSIS	GO:0006898	6	-0.524928	-1.094730	0.341927	0.791123	1.000
457	GOBP_EXOCYTOSIS	GO:0006887	11	-0.445940	-1.097064	0.361474	0.787356	1.000
458	GOBP_REGULATION_OF_LEUKOCYTE_DIFFERENTIATION	GO:1902105	9	-0.473565	-1.098939	0.354321	0.784823	1.000
459	GOBP_AMEBOIDAL_TYPE_CELL_MIGRATION	GO:0001667	11	-0.462080	-1.102169	0.352246	0.778427	1.000
460	GOBP_OSTEOCLAST_DIFFERENTIATION	GO:0030316	6	-0.532135	-1.102181	0.347712	0.781394	1.000
461	GOBP_RAS_PROTEIN_SIGNAL_TRANSDUCTION	GO:0007265	5	-0.551180	-1.103545	0.343324	0.780224	1.000
462	REACTOME_HEMOSTASIS	R-RNO-109582	14	-0.434580	-1.105133	0.349112	0.778446	1.000
463	GOBP_POSTTRANSCRIPTIONAL_REGULATION_OF_GENE_EX...	GO:0010608	8	-0.494436	-1.105477	0.341740	0.780452	1.000
464	GOBP_IMMUNE_RESPONSE_REGULATING_SIGNALING_PATHWAY	GO:000276	8	-0.492427	-1.107423	0.338832	0.777720	1.000
465	REACTOME_TOLL_LIKE_RECEPTOR_9_TLR9_CASCADE	R-RNO-168138	8	-0.494573	-1.108818	0.336683	0.776540	1.000
466	REACTOME_MYD88_INDEPENDENT_TLR4_CASCADE	R-RNO-166166	8	-0.494573	-1.109328	0.341371	0.778194	1.000
467	GOBP_TAXIS	GO:0042330	14	-0.428495	-1.110798	0.326744	0.776863	1.000
468	GOBP_MYELOID_LEUKOCYTE_ACTIVATION	GO:0002274	8	-0.490411	-1.111388	0.319481	0.778058	1.000
469	GOBP_TUBE_MORPHOGENESIS	GO:0035239	15	-0.425619	-1.112571	0.340278	0.777468	1.000
470	GOBP_REGULATION_OF_HORMONE_LEVELS	GO:0010817	12	-0.453927	-1.113239	0.328622	0.778494	1.000

Continuation of Table **S18**

	NAME	ID	SIZE	ES	NES	NOM.p.val	FDR.q.val	FWER.p.val
471	GOBP_NEGATIVE_REGULATION_OF_NEURON_DEATH	GO:1901215	11	-0.452437	-1.114802	0.333741	0.776651	1.000
472	REACTOME_TOLL_LIKE_RECEPTOR_CASCADES	R-RNO-168898	8	-0.494573	-1.116775	0.317781	0.774171	1.000
473	REACTOME_TOLL_LIKE_RECEPTOR_TLR1_TLR2_CASCADE	R-RNO-168179	8	-0.494573	-1.118786	0.335045	0.771512	1.000
474	GOBP_ORGANIC_HYDROXY_COMPOUND_TRANSPORT	GO:0015850	6	-0.533197	-1.121222	0.320051	0.767263	1.000
475	GOBP_FC_RECEPTOR_SIGNALING_PATHWAY	GO:0038093	8	-0.492427	-1.121832	0.302564	0.768629	1.000
476	GOBP_REGULATION_OF_ORGANELLE_ORGANIZATION	GO:0033043	14	-0.434467	-1.124089	0.336105	0.764993	1.000
477	GOBP_FC_EPSILON_RECEPTOR_SIGNALING_PATHWAY	GO:0038095	7	-0.513467	-1.127053	0.312581	0.759210	1.000
478	GOBP_POSITIVE_REGULATION_OF_MYELOID_LEUKOCYTE_...	GO:0002763	6	-0.539832	-1.130451	0.288512	0.752033	1.000
479	REACTOME_FC_EPSILON_RECEPTOR_FCERI_SIGNALING	R-RNO-2454202	7	-0.513467	-1.132265	0.305520	0.749660	1.000
480	GOBP_GROWTH	GO:0040007	14	-0.447146	-1.135248	0.313860	0.744224	1.000
481	GOBP_BLOOD_VESSEL_MORPHOGENESIS	GO:0048514	14	-0.441572	-1.136421	0.309327	0.743980	1.000
482	GOBP_REGULATION_OF_CELL_ACTIVATION	GO:0050865	8	-0.504492	-1.137817	0.321429	0.742810	1.000
483	GOBP_REGULATION_OF_NEURONAL_SYNAPTIC_PLASTICITY	GO:0048168	7	-0.528319	-1.139618	0.325611	0.740528	1.000
484	GOBP_REGULATION_OF_BODY_FLUID_LEVELS	GO:0050878	11	-0.466218	-1.140047	0.299031	0.742402	1.000
485	GOBP_POSITIVE_REGULATION_OF_PROTEIN_SERINE_THR...	GO:0071902	9	-0.493555	-1.141359	0.296798	0.741461	1.000
486	GOBP_SMALL_MOLECULE_METABOLIC_PROCESS	GO:0044281	15	-0.442990	-1.141393	0.310983	0.744539	1.000
487	REACTOME_INFECTIOUS_DISEASE	R-HSA-5663205	13	-0.448255	-1.143323	0.314079	0.741997	1.000
488	GOBP_ENDOCYTOSIS	GO:0006897	8	-0.502546	-1.144852	0.288804	0.740235	1.000
489	GOBP_POSITIVE_REGULATION_OF_NEURON_DEATH	GO:1901216	5	-0.580665	-1.147928	0.293478	0.734172	1.000
490	GOBP_POSITIVE_REGULATION_OF_DEVELOPMENTAL_PROCESS	GO:0051094	32	-0.390242	-1.148293	0.280553	0.736120	1.000
491	GOBP_PROTEIN_COMPLEX_OLIGOMERIZATION	GO:0051259	6	-0.549081	-1.149312	0.280000	0.736151	1.000
492	GOBP_CELLULAR_RESPONSE_TO_MOLECULE_OF_BACTERIA...	GO:0071219	8	-0.519658	-1.151403	0.265789	0.732997	1.000
493	GOBP_RESPONSE_TO_NICOTINE	GO:0035094	7	-0.530603	-1.151682	0.272152	0.735404	1.000
494	GOBP_REGULATION_OF_GROWTH	GO:0040008	13	-0.463149	-1.154998	0.286047	0.728615	1.000
495	GOBP_PROTEIN_MODIFICATION_BY_SMALL_PROTEIN_CON...	GO:0070647	7	-0.535659	-1.157397	0.278859	0.724745	1.000
496	GOBP_LOCOMOTORY_BEHAVIOR	GO:0007626	7	-0.533040	-1.158419	0.253947	0.724770	1.000
497	GOBP_CIRCADIAN_RHYTHM	GO:0007623	13	-0.462430	-1.161839	0.270463	0.717091	1.000
498	GOBP_DEFENSE_RESPONSE	GO:0006952	19	-0.426618	-1.164526	0.260771	0.711494	1.000
499	HALLMARK_HYPOXIA	M5891	5	-0.597419	-1.167531	0.266297	0.705531	1.000
500	REACTOME_ONCOGENIC_MAPK_SIGNALING	R-HSA-6802957	5	-0.595493	-1.167960	0.257576	0.707449	1.000
501	GOBP_LEUKOCYTE_MEDIATED_IMMUNITY	GO:0002443	7	-0.534614	-1.169171	0.259843	0.706984	1.000
502	GOBP_CELLULAR_RESPONSE_TO_BIOTIC_STIMULUS	GO:0071216	8	-0.519658	-1.170445	0.248724	0.706499	1.000
503	GOBP_NEGATIVE_REGULATION_OF_GENE_EXPRESSION	GO:0010629	12	-0.467989	-1.171800	0.271166	0.705439	1.000
504	GOBP_INTRACELLULAR_RECEPTOR_SIGNALING_PATHWAY	GO:0030522	5	-0.598187	-1.171837	0.237964	0.708621	1.000
505	GOBP_REGULATION_OF_PROTEIN_LOCALIZATION	GO:0032880	18	-0.435204	-1.176950	0.258701	0.695876	1.000
506	GOBP_CELLULAR_RESPONSE_TO_DNA_DAMAGE_STIMULUS	GO:0006974	5	-0.593801	-1.177345	0.240766	0.697846	1.000
507	GOBP_MULTI_MULTICELLULAR_ORGANISM_PROCESS	GO:0044706	8	-0.525535	-1.180214	0.256281	0.692455	1.000
508	GOBP_BLOOD_VESSEL_ENDOTHELIAL_CELL_MIGRATION	GO:0043534	8	-0.527351	-1.180496	0.250318	0.694796	1.000
509	GOBP_POSITIVE_REGULATION_OF_IMMUNE_RESPONSE	GO:0050778	9	-0.505468	-1.180894	0.263094	0.696809	1.000
510	GOBP_ENDOTHELIAL_CELL_MIGRATION	GO:0043542	8	-0.527351	-1.183422	0.227895	0.691892	1.000
511	GOBP_INTRACELLULAR_TRANSPORT	GO:0046907	11	-0.490728	-1.183502	0.256442	0.694954	1.000
512	GOBP_NEURON_DEATH	GO:0070997	18	-0.433687	-1.186376	0.258427	0.689069	1.000
513	REACTOME_NEURONAL_SYSTEM	R-RNO-112316	26	-0.415133	-1.186667	0.232533	0.691378	1.000

Continuation of Table **S18**

	NAME	ID	SIZE	ES	NES	NOM.p.val	FDR.q.val	FWER.p.val
514	GOBP_REGULATION_OF_CELLULAR_PROTEIN_LOCALIZATION	GO:1903827	12	-0.475343	-1.186710	0.257414	0.694621	1.000
515	GOBP_POSITIVE_REGULATION_OF_EPITHELIAL_CELL_MI...	GO:0010634	5	-0.601667	-1.189394	0.227901	0.689628	1.000
516	GOBP_POSITIVE_REGULATION_OF_LOCOMOTION	GO:0040017	11	-0.488367	-1.189772	0.262470	0.691892	1.000
517	REACTOME_TRAFFICKING_OF_AMPA_RECEPTORS	R-RNO-399719	11	-0.484541	-1.190284	0.245466	0.693518	1.000
518	GOBP_POSITIVE_REGULATION_OF_APOPTOTIC_SIGNALIN...	GO:2001235	5	-0.607085	-1.193152	0.219945	0.687550	1.000
519	GOBP_RESPONSE_TO_TUMOR_NECROSIS_FACTOR	GO:0034612	7	-0.540302	-1.193449	0.221790	0.690117	1.000
520	GOBP_POSITIVE_REGULATION_OF_RESPONSE_TO_EXTERN...	GO:0032103	9	-0.510972	-1.195092	0.250000	0.688196	1.000
521	GOBP_REGULATION_OF_NEURON_DIFFERENTIATION	GO:0045664	5	-0.611769	-1.196472	0.209524	0.687116	1.000
522	GOBP_POSITIVE_REGULATION_OF_PROTEOLYSIS	GO:0045862	8	-0.536159	-1.196725	0.237726	0.689679	1.000
523	GOBP_NEGATIVE_REGULATION_OF_CELL_DEATH	GO:0060548	22	-0.424765	-1.200169	0.239824	0.682420	1.000
524	GOBP_ANATOMICAL_STRUCTURE_FORMATION_INVOLVED_I...	GO:0048646	20	-0.428956	-1.201419	0.223799	0.681931	1.000
525	GOBP_POSITIVE_REGULATION_OF_CELL_DEATH	GO:0010942	19	-0.438592	-1.203814	0.236486	0.677882	1.000
526	GOBP_PLATELET_ACTIVATION	GO:0030168	6	-0.580264	-1.204079	0.206675	0.680490	1.000
527	GOBP_HOMEOSTATIC_PROCESS	GO:0042592	31	-0.405974	-1.204617	0.214208	0.682204	1.000
528	GOBP_POSITIVE_REGULATION_OF_PEPTIDASE_ACTIVITY	GO:0010952	7	-0.555656	-1.205996	0.197659	0.681377	1.000
529	GOBP_PROTEIN_MODIFICATION_BY_SMALL_PROTEIN_CON...	GO:0032446	6	-0.580344	-1.208274	0.204515	0.677691	1.000
530	GOBP_TISSUE_MIGRATION	GO:0090130	9	-0.515267	-1.208624	0.224335	0.680283	1.000
531	GOBP_NEGATIVE_REGULATION_OF_CELL_POPULATION_PR...	GO:0008285	9	-0.528002	-1.212078	0.219603	0.672566	1.000
532	GOBP_REGULATION_OF_CYSTEINE_TYPE_ENDOPEPTIDASE...	GO:2000116	9	-0.514813	-1.212329	0.213936	0.675292	1.000
533	GOBP_ANIMAL_ORGAN_MORPHOGENESIS	GO:0009887	12	-0.488471	-1.213169	0.210094	0.676180	1.000
534	GOBP_CELLULAR_RESPONSE_TO_KETONE	GO:1901655	5	-0.614852	-1.213389	0.202740	0.679147	1.000
535	GOBP_ERK1_AND_ERK2_CASCADE	GO:0070371	5	-0.596592	-1.214516	0.201058	0.678838	1.000
536	GOBP_MUSCLE_SYSTEM_PROCESS	GO:0003012	9	-0.526770	-1.215073	0.223844	0.680489	1.000
537	GOBP_PEPTIDYL_AMINO_ACID_MODIFICATION	GO:0018193	22	-0.426557	-1.218703	0.215664	0.672063	1.000
538	GOBP_REGULATION_OF_MUSCLE_ADAPTATION	GO:0043502	5	-0.617149	-1.220319	0.191257	0.670538	1.000
539	GOBP_NEGATIVE_REGULATION_OF_CELL_PROJECTION_OR...	GO:0031345	5	-0.620292	-1.223452	0.195973	0.663858	1.000
540	REACTOME_INTEGRATION_OF_ENERGY_METABOLISM	R-RNO-163685	5	-0.628517	-1.223640	0.195739	0.666816	1.000
541	GOBP_POSITIVE_REGULATION_OF_CYSTEINE_TYPE_ENDO...	GO:2001056	7	-0.555656	-1.225671	0.190039	0.663673	1.000
542	GOBP_MUSCLE_ADAPTATION	GO:0043500	5	-0.617149	-1.228946	0.171034	0.656684	1.000
543	REACTOME_TRANSCRIPTIONAL_REGULATION_BY_TP53	R-RNO-3700989	6	-0.585918	-1.229184	0.205298	0.659622	1.000
544	GOBP_REGULATION_OF_MYELOID_LEUKOCYTE_DIFFERENT...	GO:0002761	8	-0.549896	-1.229196	0.200260	0.663368	1.000
545	GOBP_REGULATION_OF_RESPONSE_TO_EXTERNAL_STIMULUS	GO:0032101	15	-0.477336	-1.231059	0.194866	0.660977	1.000
546	GOBP_ERBB_SIGNALING_PATHWAY	GO:0038127	5	-0.630523	-1.232083	0.181579	0.661175	1.000
547	REACTOME_TRAFFICKING_OF_GLUR2_CONTAINING_AMPA_...	R-RNO-416993	8	-0.549668	-1.234836	0.188535	0.656042	1.000
548	GOBP_NEGATIVE_REGULATION_OF_PROTEIN_METABOLIC_...	GO:0051248	20	-0.445174	-1.235514	0.198864	0.657596	1.000
549	GOBP_REGULATION_OF_CELL_DIFFERENTIATION	GO:0045595	36	-0.414412	-1.245504	0.173220	0.628594	1.000
550	REACTOME_SIGNALING_BY_NTRKS	R-RNO-166520	18	-0.463043	-1.246975	0.189655	0.627649	1.000
551	GOBP_RESPONSE_TO_RADIATION	GO:0009314	13	-0.490165	-1.251703	0.193357	0.616164	1.000
552	GOBP_PERIPHERAL_NERVOUS_SYSTEM_DEVELOPMENT	GO:0007422	9	-0.537156	-1.251859	0.190898	0.619397	1.000
553	GOBP_NEGATIVE_REGULATION_OF_RESPONSE_TO_STIMULUS	GO:0048585	16	-0.475600	-1.253112	0.178322	0.619324	1.000
554	GOBP_POSITIVE_REGULATION_OF_BINDING	GO:0051099	6	-0.607608	-1.253645	0.163073	0.621480	1.000
555	REACTOME_G_ALPHA_Q_SIGNALLING_EVENTS	R-RNO-416476	6	-0.607757	-1.254597	0.171123	0.621983	1.000
556	GOBP_TUBE_DEVELOPMENT	GO:0035295	18	-0.463594	-1.256568	0.164948	0.619430	1.000

Continuation of Table **S18**

	NAME	ID	SIZE	ES	NES	NOM.p.val	FDR.q.val	FWER.p.val
557	REACTOME_OPIOID_SIGNALLING	R-RNO-111885	12	-0.512486	-1.256768	0.163070	0.622511	1.000
558	GOBP_SPROUTING_ANGIOGENESIS	GO:0002040	5	-0.644819	-1.258721	0.154891	0.619889	1.000
559	GOBP_POSITIVE_REGULATION_OF_PRI_MIRNA_TRANSCRI...	GO:1902895	7	-0.578642	-1.259399	0.178893	0.621653	1.000
560	GOBP_PRI_MIRNA_TRANSCRIPTION_BY_RNA_POLYMERASE_II	GO:0061614	7	-0.578642	-1.259705	0.163636	0.624529	1.000
561	GOBP_PEPTIDYL_LYSINE_MODIFICATION	GO:0018205	5	-0.631095	-1.260173	0.151915	0.626985	1.000
562	REACTOME_SIGNALING_BY_RECEPTOR_TYROSINE_KINASES	R-RNO-9006934	26	-0.436353	-1.260465	0.177560	0.630053	1.000
563	GOBP_RESPONSE_TO_INSULIN	GO:0032868	5	-0.635883	-1.261164	0.154263	0.631720	1.000
564	GOBP_REGULATION_OF_CELL_CYCLE_PROCESS	GO:0010564	5	-0.634288	-1.261231	0.166893	0.635565	1.000
565	GOBP_CELL_CYCLE	GO:0007049	21	-0.449087	-1.267321	0.149490	0.619114	1.000
566	GOBP_REGULATION_OF_RESPONSE_TO_STRESS	GO:0080134	19	-0.462761	-1.271287	0.164798	0.609774	1.000
567	GOBP_NEGATIVE_REGULATION_OF_CELLULAR_COMPONENT...	GO:0051129	11	-0.516756	-1.271363	0.167651	0.613599	1.000
568	GOBP_CELL_ACTIVATION	GO:0001775	21	-0.450516	-1.274195	0.162042	0.608086	1.000
569	GOBP_RESPONSE_TO_ABIOTIC_STIMULUS	GO:0009628	27	-0.436362	-1.278149	0.145394	0.598101	1.000
570	GOBP_RESPONSE_TO_KETONE	GO:1901654	7	-0.586229	-1.278803	0.160804	0.599806	1.000
571	GOBP_CARBOHYDRATE_HOMEOSTASIS	GO:0033500	7	-0.583299	-1.281675	0.141772	0.594322	1.000
572	GOBP_RESPONSE_TO_CALCIUM_ION	GO:0051592	6	-0.616889	-1.282772	0.146405	0.594890	1.000
573	GOBP_MULTI_ORGANISM_PROCESS	GO:0051704	16	-0.476899	-1.283422	0.154913	0.596759	1.000
574	GOBP_COAGULATION	GO:0050817	8	-0.572784	-1.287836	0.143392	0.585801	1.000
575	GOBP_POSITIVE_REGULATION_OF_CELLULAR_PROTEIN_L...	GO:1903829	7	-0.600093	-1.290053	0.136483	0.582333	1.000
576	GOBP_RESPONSE_TO_ENDOGENOUS_STIMULUS	GO:0009719	34	-0.428934	-1.290251	0.136752	0.585741	1.000
577	REACTOME_NGF_STIMULATED_TRANSCRIPTION	R-RNO-9031628	9	-0.554841	-1.291909	0.137717	0.584166	1.000
578	GOBP_MUSCLE_TISSUE_DEVELOPMENT	GO:0060537	11	-0.538445	-1.297069	0.118873	0.570823	1.000
579	GOBP_DEVELOPMENTAL_PROCESS_INVOLVED_IN_REPRODU...	GO:0003006	11	-0.537393	-1.299761	0.132616	0.566497	1.000
580	GOBP_DEFENSE_RESPONSE_TO_OTHER_ORGANISM	GO:0098542	9	-0.558386	-1.301892	0.118812	0.563558	1.000
581	GOBP_REGULATION_OF_SMALL_MOLECULE_METABOLIC_PR...	GO:0062012	9	-0.561030	-1.302998	0.118306	0.563899	1.000
582	GOBP_REGULATION_OF_LIPID_METABOLIC_PROCESS	GO:0019216	7	-0.600028	-1.304611	0.124679	0.562481	1.000
583	GOBP_MONONUCLEAR_CELL_DIFFERENTIATION	GO:1903131	7	-0.598281	-1.304773	0.149874	0.565999	1.000
584	GOBP_INNATE_IMMUNE_RESPONSE	GO:0045087	7	-0.607080	-1.312189	0.129887	0.546105	1.000
585	GOBP_REGULATION_OF_MITOTIC_CELL_CYCLE	GO:0007346	5	-0.663435	-1.313064	0.125000	0.547133	1.000
586	GOBP_ESTABLISHMENT_OF_PROTEIN_LOCALIZATION_TO_...	GO:0072594	6	-0.624114	-1.313382	0.106299	0.550329	1.000
587	GOBP_MITOTIC_CELL_CYCLE	GO:0000278	10	-0.554408	-1.314412	0.114673	0.551153	1.000
588	GOBP_MULTICELLULAR_ORGANISM_REPRODUCTION	GO:0032504	12	-0.519882	-1.315770	0.148681	0.550967	1.000
589	GOBP_NEUROTROPHIN_SIGNALING_PATHWAY	GO:0038179	6	-0.633634	-1.317171	0.111111	0.550374	1.000
590	GOBP_REGULATION_OF_HEMOPOIESIS	GO:1903706	10	-0.551038	-1.320645	0.135802	0.543056	1.000
591	GOBP_TRANSMEMBRANE_RECEPTOR_PROTEIN_TYROSINE_K...	GO:0007169	19	-0.485845	-1.322687	0.144495	0.540418	1.000
592	GOBP_CYTOKINE_MEDIATED_SIGNALING_PATHWAY	GO:0019221	16	-0.493639	-1.322800	0.119590	0.544288	1.000
593	GOBP_LYMPHOCYTE_ACTIVATION	GO:0046649	9	-0.571244	-1.323303	0.138650	0.546848	1.000
594	GOBP_REGULATION_OF_IMMUNE_RESPONSE	GO:0050776	12	-0.536988	-1.323476	0.124088	0.550627	1.000
595	GOBP_NEUROTROPHIN_TRK_RECEPTOR_SIGNALING_PATHWAY	GO:0048011	6	-0.633634	-1.323949	0.115538	0.553294	1.000
596	REACTOME_G_PROTEIN_MEDIATED_EVENTS	R-RNO-112040	9	-0.571028	-1.324025	0.120988	0.557562	1.000
597	GOBP_NEGATIVE_REGULATION_OF_TRANSCRIPTION_BY_R...	GO:0000122	10	-0.546933	-1.325030	0.125945	0.558490	1.000
598	GOBP_POSITIVE_REGULATION_OF_ENDOTHELIAL_CELL_P...	GO:0001938	6	-0.637985	-1.326998	0.130890	0.556233	1.000
599	GOBP_POSITIVE_REGULATION_OF_GENE_EXPRESSION	GO:0010628	16	-0.499931	-1.329373	0.124857	0.552649	1.000

Continuation of Table **S18**

	NAME	ID	SIZE	ES	NES	NOM.p.val	FDR.q.val	FWER.p.val
600	GOBP_REGULATION_OF_CELLULAR_RESPONSE_TO_STRESS	GO:0080135	9	-0.579178	-1.333126	0.120585	0.544455	1.000
601	GOBP_RESPONSE_TO_OXYGEN_LEVELS	GO:0070482	8	-0.591888	-1.335357	0.114103	0.541322	1.000
602	HALLMARK_TNFA_SIGNALING_VIA_NFKB	M5890	14	-0.513846	-1.335793	0.122970	0.544412	1.000
603	GOBP_NEGATIVE_REGULATION_OF_HYDROLASE_ACTIVITY	GO:0051346	11	-0.542857	-1.339425	0.108935	0.536785	1.000
604	GOBP_SKELETAL_MUSCLE_ORGAN_DEVELOPMENT	GO:0060538	5	-0.685491	-1.340974	0.094415	0.536130	1.000
605	GOBP_POSITIVE_REGULATION_OF_IMMUNE_SYSTEM_PROCESS	GO:0002684	18	-0.505520	-1.346063	0.090498	0.523633	1.000
606	GOBP_REGULATION_OF_WNT_SIGNALING_PATHWAY	GO:0030111	5	-0.688480	-1.346639	0.085754	0.526443	1.000
607	GOBP_CELLULAR_RESPONSE_TO_PEPTIDE_HORMONE_STIM...	GO:0071375	9	-0.586567	-1.347261	0.103362	0.529159	1.000
608	GOBP_CELL_ACTIVATION_INVOLVED_IN_IMMUNE_RESPONSE	GO:0002263	6	-0.654873	-1.347995	0.087075	0.531460	1.000
609	GOBP_CELLULAR_RESPONSE_TO_LIPID	GO:0071396	15	-0.509074	-1.349005	0.104953	0.533025	1.000
610	GOBP_NEGATIVE_REGULATION_OF_NUCLEOBASE_CONTAIN...	GO:0045934	13	-0.545054	-1.349650	0.109027	0.535637	1.000
611	REACTOME_NEUTROPHIL_DEGRANULATION	R-RNO-6798695	6	-0.654873	-1.349777	0.098667	0.540153	1.000
612	GOBP_CELLULAR_GLUCOSE_HOMEOSTASIS	GO:0001678	5	-0.685186	-1.350977	0.084337	0.540837	1.000
613	GOBP_POSITIVE_REGULATION_OF_MULTICELLULAR_ORGA...	GO:0051240	32	-0.458485	-1.351387	0.114754	0.544371	1.000
614	GOBP_REGULATION_OF_CELL_CYCLE	GO:0051726	13	-0.535257	-1.351941	0.109606	0.547388	1.000
615	GOBP_LIPID_BIOSYNTHETIC_PROCESS	GO:0008610	6	-0.659353	-1.353785	0.083879	0.545991	1.000
616	GOBP_CELL_CELL_SIGNALING_BY_WNT	GO:0198738	7	-0.624074	-1.356213	0.090561	0.542985	1.000
617	GOBP_NEGATIVE_REGULATION_OF_BIOSYNTHETIC_PROCESS	GO:0009890	13	-0.533960	-1.357306	0.093859	0.544293	1.000
618	GOBP_CELLULAR_RESPONSE_TO_EXTRACELLULAR_STIMULUS	GO:0031668	8	-0.602057	-1.361504	0.091925	0.534450	1.000
619	GOBP_T_CELL_ACTIVATION	GO:0042110	8	-0.605059	-1.361838	0.094697	0.538744	1.000
620	GOBP_CELLULAR_RESPONSE_TO_STARVATION	GO:0009267	5	-0.698734	-1.363189	0.076398	0.539616	1.000
621	GOBP_REGULATION_OF_LIPID_BIOSYNTHETIC_PROCESS	GO:0046890	6	-0.659353	-1.364065	0.093176	0.541956	1.000
622	GOBP_MULTICELLULAR_ORGANISMAL_HOMEOSTASIS	GO:0048871	12	-0.547096	-1.365463	0.098321	0.542740	1.000
623	GOBP_POSITIVE_REGULATION_OF_CYTOKINE_PRODUCTION	GO:0001819	7	-0.632302	-1.365757	0.097345	0.547173	1.000
624	GOBP_REPRODUCTION	GO:0000003	18	-0.503871	-1.366529	0.091954	0.550180	1.000
625	GOBP_ENDOTHELIAL_CELL_PROLIFERATION	GO:0001935	8	-0.613882	-1.366962	0.093710	0.554124	1.000
626	GOBP_AGING	GO:0007568	13	-0.537121	-1.367624	0.098301	0.557470	1.000
627	REACTOME_CA_DEPENDENT_EVENTS	R-RNO-111996	8	-0.613456	-1.367630	0.077419	0.563507	1.000
628	GOBP_MYELOID_LEUKOCYTE_MEDIATED_IMMUNITY	GO:0002444	6	-0.654873	-1.368569	0.095816	0.566194	1.000
629	GOBP_CELL_CYCLE_PROCESS	GO:0022402	11	-0.565366	-1.369923	0.094645	0.566818	1.000
630	GOBP_CELLULAR_MACROMOLECULE_CATABOLIC_PROCESS	GO:0044265	6	-0.665301	-1.373403	0.099355	0.560339	1.000
631	REACTOME_PLATELET_ACTIVATION_SIGNALING_AND_AGG...	R-HSA-76002	7	-0.625394	-1.373762	0.096306	0.565522	1.000
632	GOBP_CELL_POPULATION_PROLIFERATION	GO:0008283	31	-0.468222	-1.381003	0.088267	0.544315	1.000
633	GOBP_REGULATION_OF_ANION_TRANSPORT	GO:0044070	20	-0.489206	-1.382665	0.085106	0.544417	1.000
634	GOBP_CIRCULATORY_SYSTEM_DEVELOPMENT	GO:0072359	21	-0.498398	-1.384517	0.084081	0.544142	1.000
635	GOBP_POSITIVE_REGULATION_OF_EPITHELIAL_CELL_PR...	GO:0050679	7	-0.625349	-1.386790	0.079324	0.542269	1.000
636	GOBP_CHROMATIN_ORGANIZATION	GO:0006325	5	-0.693507	-1.387774	0.055935	0.545083	1.000
637	GOBP_POSITIVE_REGULATION_OF_CELL_POPULATION_PR...	GO:0008284	21	-0.498657	-1.389268	0.075028	0.546130	1.000
638	GOBP_PROTEIN_LOCALIZATION_TO_ORGANELLE	GO:0033365	9	-0.605475	-1.390180	0.070263	0.549206	1.000
639	GOBP_MUSCLE_ORGAN_DEVELOPMENT	GO:0007517	9	-0.602148	-1.390990	0.073658	0.553119	1.000
640	GOBP_ENZYME_LINKED_RECEPTOR_PROTEIN_SIGNALING_...	GO:0007167	24	-0.489849	-1.398259	0.064978	0.531454	1.000
641	GOBP_POSITIVE_REGULATION_OF_ANION_TRANSPORT	GO:1903793	9	-0.602035	-1.398876	0.087763	0.535511	1.000
642	GOBP_RESPONSE_TO_STARVATION	GO:0042594	5	-0.698734	-1.405055	0.064171	0.519721	1.000

Continuation of Table **S18**

	NAME	ID	SIZE	ES	NES	NOM.p.val	FDR.q.val	FWER.p.val
643	GOBP_REGULATION_OF_PROTEIN_STABILITY	GO:0031647	5	-0.704817	-1.411878	0.068966	0.502191	1.000
644	GOBP_RESPONSE_TO_OXYGEN_CONTAINING_COMPOUND	GO:1901700	39	-0.467609	-1.426152	0.051447	0.457789	1.000
645	GOBP_RESPONSE_TO_LIPID	GO:0033993	19	-0.514598	-1.429730	0.061017	0.451814	1.000
646	GOBP_CELLULAR_RESPONSE_TO_ABIOTIC_STIMULUS	GO:0071214	7	-0.656175	-1.434210	0.059627	0.442607	1.000
647	GOBP_CELLULAR_RESPONSE_TO_PEPTIDE	GO:1901653	13	-0.567004	-1.434478	0.051435	0.447567	1.000
648	GOBP_MYELOID_LEUKOCYTE_DIFFERENTIATION	GO:0002573	11	-0.591863	-1.435353	0.056931	0.451116	1.000
649	GOBP_REGULATION_OF_MYELOID_CELL_DIFFERENTIATION	GO:0045637	9	-0.620915	-1.436069	0.065854	0.455236	1.000
650	REACTOME_ESTROGEN_DEPENDENT_NUCLEAR_EVENTS_DOW...	R-RNO-9634638	5	-0.731514	-1.437364	0.042553	0.457334	1.000
651	GOBP_RESPONSE_TO_INORGANIC_SUBSTANCE	GO:0010035	19	-0.515211	-1.438111	0.057175	0.461411	1.000
652	GOBP_RESPONSE_TO_NITROGEN_COMPOUND	GO:1901698	34	-0.481730	-1.438718	0.048077	0.466002	1.000
653	GOBP_VASCULATURE_DEVELOPMENT	GO:0001944	17	-0.541411	-1.447895	0.052692	0.441139	1.000
654	GOBP_REGULATION_OF_IMMUNE_SYSTEM_PROCESS	GO:0002682	21	-0.517109	-1.448162	0.060502	0.446920	1.000
655	GOBP_TISSUE_HOMEOSTASIS	GO:0001894	5	-0.730595	-1.449404	0.034483	0.449333	1.000
656	GOBP_CARBOHYDRATE_BIOSYNTHETIC_PROCESS	GO:0016051	5	-0.735446	-1.450579	0.046703	0.452508	1.000
657	GOBP_CELLULAR_RESPONSE_TO_INORGANIC_SUBSTANCE	GO:0071241	11	-0.595297	-1.453256	0.038694	0.449702	1.000
658	GOBP_REGULATION_OF_CARBOHYDRATE_METABOLIC_PROCESS	GO:0006109	5	-0.735446	-1.455973	0.037241	0.447052	1.000
659	GOBP_CELLULAR_RESPONSE_TO_EXTERNAL_STIMULUS	GO:0071496	10	-0.613285	-1.456497	0.041262	0.452565	1.000
660	GOBP_RESPONSE_TO_MOLECULE_OF_BACTERIAL_ORIGIN	GO:0002237	13	-0.572657	-1.457839	0.049763	0.455531	1.000
661	GOBP_APOPTOTIC_SIGNALING_PATHWAY	GO:0097190	15	-0.557619	-1.458684	0.039261	0.459883	1.000
662	GOBP_REGULATION_OF_CARBOHYDRATE_BIOSYNTHETIC_P...	GO:0043255	5	-0.735446	-1.459505	0.047490	0.464199	1.000
663	HALLMARK_UV_RESPONSE_UP	M5941	5	-0.733690	-1.464476	0.037344	0.454873	1.000
664	REACTOME_ESR_MEDIATED_SIGNALING	R-RNO-8939211	8	-0.652815	-1.469482	0.049869	0.445088	1.000
665	REACTOME_CELL_CYCLE	R-RNO-1640170	6	-0.703607	-1.470180	0.042553	0.451373	1.000
666	GOBP_RESPONSE_TO_OXIDATIVE_STRESS	GO:0006979	8	-0.658290	-1.471982	0.051414	0.453490	1.000
667	REACTOME_SIGNALING_BY_NUCLEAR_RECEPTORS	R-RNO-9006931	8	-0.652815	-1.473035	0.048969	0.457839	1.000
668	REACTOME_NUCLEAR_EVENTS_KINASE_AND_TRANSCRIPTI...	R-RNO-198725	11	-0.605835	-1.475356	0.042908	0.458328	1.000
669	GOBP_RESPONSE_TO_BACTERIUM	GO:0009617	13	-0.572657	-1.480237	0.035294	0.448426	1.000
670	GOBP_DNA_METABOLIC_PROCESS	GO:0006259	6	-0.704260	-1.483121	0.036896	0.446954	1.000
671	GOBP_NEGATIVE_REGULATION_OF_CELL_DIFFERENTIATION	GO:0045596	13	-0.596055	-1.485279	0.042503	0.448219	1.000
672	GOBP_CELLULAR_RESPONSE_TO_CADMIUM_ION	GO:0071276	5	-0.752648	-1.486642	0.036697	0.452443	1.000
673	GOBP_RESPONSE_TO_GROWTH_FACTOR	GO:0070848	18	-0.545097	-1.487974	0.038117	0.456843	1.000
674	GOBP_RESPONSE_TO_REACTIVE_OXYGEN_SPECIES	GO:0000302	8	-0.658290	-1.488636	0.049808	0.464979	1.000
675	GOBP_CELLULAR_RESPONSE_TO_NITROGEN_COMPOUND	GO:1901699	20	-0.545764	-1.502901	0.030067	0.420395	1.000
676	GOBP_NEGATIVE_REGULATION_OF_DEVELOPMENTAL_PROCESS	GO:0051093	13	-0.596055	-1.505536	0.027187	0.419574	1.000
677	GOBP_CELLULAR_RESPONSE_TO_HORMONE_STIMULUS	GO:0032870	11	-0.625261	-1.505621	0.028916	0.429250	1.000
678	GOBP_ENDOMEMBRANE_SYSTEM_ORGANIZATION	GO:0010256	6	-0.729473	-1.507025	0.028461	0.434377	1.000
679	REACTOME_MAPK_TARGETS_NUCLEAR_EVENTS_MEDIATED_...	R-HSA-450282	5	-0.761795	-1.511425	0.023448	0.427747	1.000
680	GOBP_POSITIVE_REGULATION_OF_NUCLEOBASE_CONTAIN...	GO:0045935	28	-0.519870	-1.513458	0.029001	0.430795	1.000
681	GOBP_CELLULAR_RESPONSE_TO_REACTIVE_OXYGEN_SPECIES	GO:0034614	7	-0.705586	-1.517514	0.029040	0.426558	1.000
682	REACTOME_EXTRA_NUCLEAR_ESTROGEN_SIGNALING	R-RNO-9009391	7	-0.697905	-1.518169	0.025990	0.435436	1.000
683	GOBP_RESPONSE_TO_PEPTIDE_HORMONE	GO:0043434	13	-0.587908	-1.524988	0.027091	0.421098	1.000
684	GOBP_RESPONSE_TO_PEPTIDE	GO:1901652	18	-0.562621	-1.528581	0.018931	0.419428	1.000
685	GOBP_MYELOID_CELL_DIFFERENTIATION	GO:0030099	13	-0.607730	-1.529383	0.023952	0.428292	1.000

Continuation of Table **S18**

	NAME	ID	SIZE	ES	NES	NOM.p.val	FDR.q.val	FWER.p.val
686	GOBP_ANATOMICAL_STRUCTURE_HOMEOSTASIS	GO:0060249	7	-0.706786	-1.536390	0.028169	0.414164	1.000
687	GOBP_RESPONSE_TO_ORGANIC_CYCLIC_COMPOUND	GO:0014070	20	-0.551466	-1.538216	0.019473	0.420615	1.000
688	REACTOME_SENESCENCE_ASSOCIATED_SECRETORY_PHENO...	R-RNO-2559582	6	-0.743931	-1.544775	0.022457	0.408269	1.000
689	GOBP_RESPONSE_TO_BIOTIC_STIMULUS	GO:0009607	17	-0.575603	-1.548995	0.022599	0.406303	1.000
690	GOBP_RESPONSE_TO_HORMONE	GO:0009725	20	-0.563460	-1.551259	0.015730	0.412053	1.000
691	GOBP_POSITIVE_REGULATION_OF_BIOSYNTHETIC_PROCESS	GO:0009891	27	-0.537777	-1.551505	0.022801	0.425528	1.000
692	GOBP_RESPONSE_TO_METAL_ION	GO:0010038	15	-0.586003	-1.552706	0.022353	0.436035	1.000
693	REACTOME_CELLULAR_SENESCENCE	R-RNO-2559583	6	-0.743931	-1.555659	0.018494	0.440793	1.000
694	REACTOME_CELL_CYCLE_MITOTIC	R-RNO-69278	5	-0.777316	-1.560863	0.014865	0.435689	1.000
695	GOBP_CYTOKINE_PRODUCTION	GO:0001816	10	-0.653944	-1.564655	0.023002	0.436099	1.000
696	GOBP_LEUKOCYTE_DIFFERENTIATION	GO:0002521	15	-0.601688	-1.565265	0.026775	0.453241	1.000
697	GOBP_CELLULAR_RESPONSE_TO_CHEMICAL_STRESS	GO:0062197	7	-0.705586	-1.568984	0.022930	0.457875	1.000
698	REACTOME_RHO_GTPASE_EFFECTORS	R-RNO-195258	9	-0.683420	-1.572556	0.018916	0.462620	1.000
699	REACTOME_INTRACELLULAR_SIGNALING_BY_SECOND_MES...	R-RNO-9006925	15	-0.597985	-1.573132	0.015012	0.482392	1.000
700	REACTOME_SIGNALING_BY_RHO_GTPASES_MIRO_GTPASES...	R-RNO-9716542	9	-0.683420	-1.573596	0.019876	0.504761	1.000
701	REACTOME_PTEN_REGULATION	R-RNO-6807070	5	-0.795312	-1.574377	0.017287	0.528511	1.000
702	GOBP_CIRCADIAN_REGULATION_OF_GENE_EXPRESSION	GO:0032922	5	-0.810652	-1.582998	0.009615	0.511561	1.000
703	GOBP_RESPONSE_TO_CYTOKINE	GO:0034097	23	-0.561041	-1.590455	0.012182	0.503463	1.000
704	GOBP_RESPONSE_TO_INTERLEUKIN_1	GO:0070555	5	-0.788071	-1.592308	0.011080	0.527145	1.000
705	GOBP_CELLULAR_RESPONSE_TO_ORGANIC_CYCLIC_COMPOUND	GO:0071407	13	-0.628618	-1.605673	0.017815	0.492604	0.999
706	GOBP_CHROMOSOME_ORGANIZATION	GO:0051276	6	-0.779348	-1.616309	0.011749	0.474459	0.998
707	GOBP_IMMUNE_SYSTEM_DEVELOPMENT	GO:0002520	17	-0.602560	-1.621369	0.005855	0.484531	0.997
708	REACTOME_INTERLEUKIN_17_SIGNALING	R-RNO-448424	6	-0.779880	-1.624243	0.010540	0.511357	0.997
709	REACTOME_CELLULAR_RESPONSES_TO_EXTERNAL_STIMULI	R-RNO-8953897	12	-0.659393	-1.624315	0.003563	0.561680	0.997
710	GOBP_REPRODUCTIVE_SYSTEM_DEVELOPMENT	GO:0061458	6	-0.768679	-1.630165	0.004098	0.580990	0.995
711	GOBP_CELLULAR_RESPONSE_TO_OXYGEN_CONTAINING_CO...	GO:1901701	29	-0.556350	-1.633406	0.007503	0.630095	0.995
712	GOBP_POSITIVE_REGULATION_OF_TRANSCRIPTION_BY_R...	GO:0045944	24	-0.562724	-1.634757	0.005525	0.709635	0.995
713	GOBP_TRANSMEMBRANE_RECEPTOR_PROTEIN_SERINE_THR...	GO:0007178	6	-0.793485	-1.642650	0.009272	0.760634	0.992
714	GOBP_RESPONSE_TO_CADMIUM_ION	GO:0046686	6	-0.811818	-1.668194	0.005249	0.687236	0.959
715	REACTOME_RNA_POLYMERASE_II_TRANSCRIPTION	R-RNO-73857	20	-0.603682	-1.670752	0.006787	0.833580	0.954
716	GOBP_RESPONSE_TO_HEAT	GO:0009408	7	-0.770551	-1.697763	0.005141	0.786915	0.885
717	GOBP_EPITHELIAL_CELL_PROLIFERATION	GO:0050673	12	-0.676235	-1.702127	0.003550	1.000000	0.876
718	GOBP_RESPONSE_TO_TEMPERATURE_STIMULUS	GO:0009266	9	-0.835824	-1.940079	0.000000	0.059114	0.056

End of Table

Supplementary Table S19: CB late profile (45 DPL peak) GSEA results.

Begin of Table **S19**

	NAME	ID	SIZE	ES	NES	NOM.p.val	FDR.q.val	FWER.p.val
0	GOBP_NERVE_DEVELOPMENT	GO:0021675	9	0.716460	2.134525	0.000000	0.229673	0.297
1	GOBP_NEUROTROPHIN_TRK_RECEPTOR_SIGNALING_PATHWAY	GO:0048011	6	0.756005	2.012518	0.000000	0.294539	0.603
2	GOBP_NEUROTROPHIN_SIGNALING_PATHWAY	GO:0038179	6	0.756005	1.964451	0.005666	0.274723	0.720
3	GOBP_NEGATIVE_REGULATION_OF_NEURON_APOPTOTIC_P...	GO:0043524	8	0.644055	1.874099	0.014085	0.364420	0.896
4	GOBP_MULTICELLULAR_ORGANISMAL_RESPONSE_TO_STRESS	GO:0033555	5	0.747005	1.776908	0.032432	0.498949	0.973
5	GOBP_NUCLEAR_TRANSPORT	GO:0051169	5	0.728041	1.751098	0.021390	0.476310	0.986
6	GOBP_SKELETAL_MUSCLE_ORGAN_DEVELOPMENT	GO:0060538	5	0.732227	1.747755	0.022059	0.416709	0.986
7	GOBP_REGULATION_OF_NEUROGENESIS	GO:0050767	14	0.468034	1.694843	0.018462	0.478156	0.997
8	GOBP_POSITIVE_REGULATION_OF_BINDING	GO:0051099	6	0.631168	1.659829	0.028490	0.507211	0.998
9	GOBP_REGULATION_OF_CELL_DEVELOPMENT	GO:0060284	15	0.457693	1.647894	0.038095	0.483624	0.998
10	GOBP_REGULATION_OF_CELL_DIFFERENTIATION	GO:0045595	36	0.330436	1.616250	0.013575	0.514549	1.000
11	GOBP_REGULATION_OF_NERVOUS_SYSTEM_DEVELOPMENT	GO:0051960	15	0.417206	1.588721	0.055738	0.540900	1.000
12	GOBP_REGULATION_OF_PHOSPHOLIPASE_ACTIVITY	GO:0010517	6	0.597561	1.579981	0.057221	0.521679	1.000
13	GOBP_PEPTIDYL_AMINO_ACID_MODIFICATION	GO:0018193	22	0.363230	1.538866	0.047619	0.592602	1.000
14	GOBP_REGULATION_OF_LIPASE_ACTIVITY	GO:0060191	6	0.597561	1.533080	0.086705	0.568456	1.000
15	GOBP_INTRACELLULAR_TRANSPORT	GO:0046907	11	0.452115	1.530977	0.057927	0.538756	1.000
16	GOBP_REGULATION_OF_NEURON_DIFFERENTIATION	GO:0045664	5	0.617818	1.514529	0.068493	0.547635	1.000
17	GOBP_POSITIVE_REGULATION_OF_CELL_DEVELOPMENT	GO:0010720	12	0.432380	1.514442	0.062706	0.517392	1.000
18	GOBP_NEGATIVE_REGULATION_OF_CELL_DIFFERENTIATION	GO:0045596	13	0.411971	1.484162	0.070513	0.565311	1.000
19	GOBP_NEGATIVE_REGULATION_OF_DEVELOPMENTAL_PROCESS	GO:0051093	13	0.411971	1.481328	0.083871	0.543793	1.000
20	GOBP_POSITIVE_REGULATION_OF_NEUROGENESIS	GO:0050769	12	0.432380	1.480072	0.072131	0.520629	1.000
21	GOBP_POSITIVE_REGULATION_OF_NERVOUS_SYSTEM_DEV...	GO:0051962	12	0.432380	1.475271	0.084416	0.507155	1.000
22	GOBP_NEURON_APOPTOTIC_PROCESS	GO:0051402	12	0.425131	1.462701	0.070968	0.515005	1.000
23	GOBP_PERIPHERAL_NERVOUS_SYSTEM_DEVELOPMENT	GO:0007422	9	0.457535	1.455716	0.080357	0.509114	1.000
24	GOBP_REGULATION_OF_PHOSPHOLIPASE_C_ACTIVITY	GO:1900274	5	0.590361	1.441845	0.114362	0.520327	1.000
25	GOBP_REGULATION_OF_MULTICELLULAR_ORGANISMAL_DE...	GO:2000026	31	0.306068	1.431152	0.047619	0.524655	1.000
26	GOBP_POSITIVE_REGULATION_OF_LIPASE_ACTIVITY	GO:0060193	5	0.590361	1.424417	0.085873	0.520287	1.000
27	GOBP_RESPONSE_TO_STARVATION	GO:0042594	5	0.578313	1.423891	0.083102	0.502754	1.000
28	GOBP_CELLULAR_RESPONSE_TO_STARVATION	GO:0009267	5	0.578313	1.413315	0.108992	0.508393	1.000
29	GOBP_REGULATION_OF_GTPASE_ACTIVITY	GO:0043087	5	0.578313	1.375816	0.133880	0.577743	1.000
30	GOBP_FC_EPSILON_RECEPTOR_SIGNALING_PATHWAY	GO:0038095	7	0.481482	1.369556	0.111782	0.572921	1.000
31	GOBP_NEGATIVE_REGULATION_OF_PROTEIN_METABOLIC_...	GO:0051248	20	0.333283	1.354928	0.111913	0.589906	1.000
32	GOBP_NEGATIVE_REGULATION_OF_PROTEOLYSIS	GO:0045861	8	0.456930	1.341875	0.133333	0.604494	1.000
33	GOBP_NEGATIVE_REGULATION_OF_NEURON_DEATH	GO:1901215	11	0.400801	1.335697	0.141538	0.602383	1.000
34	GOBP_POSITIVE_REGULATION_OF_GLIOGENESIS	GO:0014015	5	0.546907	1.332623	0.136816	0.592521	1.000
35	GOBP_POSITIVE_REGULATION_OF_LOCOMOTION	GO:0040017	11	0.395448	1.332378	0.133779	0.576815	1.000
36	GOBP_SMALL_GTPASE_MEDIATED_SIGNAL_TRANSDUCTION	GO:0007264	8	0.453414	1.332291	0.153425	0.561364	1.000
37	REACTOME_FC_EPSILON_RECEPTOR_FCERI_SIGNALING	R-RNO-2454202	7	0.481481	1.329276	0.150000	0.553650	1.000
38	GOBP_REGULATION_OF_GLIOGENESIS	GO:0014013	5	0.546907	1.324237	0.135977	0.550937	1.000
39	GOBP_MUSCLE_ORGAN_DEVELOPMENT	GO:0007517	9	0.429393	1.323356	0.150769	0.539286	1.000
40	GOBP_INTRACELLULAR_PROTEIN_TRANSPORT	GO:0006886	9	0.423097	1.318148	0.132743	0.537549	1.000

Continuation of Table **S19**

	NAME	ID	SIZE	ES	NES	NOM.p.val	FDR.q.val	FWER.p.val
41	REACTOME_BETA_CATENIN_INDEPENDENT_WNT_SIGNALING	R-RNO-3858494	5	0.527452	1.309498	0.156522	0.543938	1.000
42	GOBP_MYOTUBE_DIFFERENTIATION	GO:0014902	5	0.542169	1.304991	0.170455	0.540888	1.000
43	GOBP_RHYTHMIC_PROCESS	GO:0048511	15	0.341645	1.283914	0.138047	0.572486	1.000
44	GOBP_REGULATION_OF_BINDING	GO:0051098	11	0.381002	1.278581	0.179878	0.572397	1.000
45	REACTOME_SIGNALING_BY_WNT	R-RNO-195721	7	0.440998	1.267772	0.173410	0.583030	1.000
46	GOBP_MUSCLE_TISSUE_DEVELOPMENT	GO:0060537	11	0.355989	1.234762	0.205607	0.647616	1.000
47	REACTOME_CELLULAR_SENESCENCE	R-RNO-2559583	6	0.463415	1.229137	0.202740	0.647220	1.000
48	REACTOME_SIGNALING_BY_NTRKS	R-RNO-166520	18	0.309306	1.225833	0.179856	0.641921	1.000
49	REACTOME_SENESCENCE_ASSOCIATED_SECRETORY_PHENO...	R-RNO-2559582	6	0.463415	1.221107	0.218289	0.639940	1.000
50	HALLMARK_KRAS_SIGNALING_UP	M5953	6	0.469763	1.220152	0.217514	0.629797	1.000
51	GOBP_PEPTIDYL_LYSINE_MODIFICATION	GO:0018205	5	0.481928	1.215751	0.220472	0.628005	1.000
52	REACTOME_CELL_CYCLE_MITOTIC	R-RNO-69278	5	0.493718	1.205332	0.248603	0.641023	1.000
53	GOBP_REGULATION_OF_PEPTIDYL_TYROSINE_PHOSPHORY...	GO:0050730	9	0.383853	1.201798	0.224377	0.638281	1.000
54	GOBP_NEGATIVE_REGULATION_OF_PEPTIDASE_ACTIVITY	GO:0010466	6	0.451545	1.200791	0.209945	0.629241	1.000
55	GOBP_CIRCADIAN_REGULATION_OF_GENE_EXPRESSION	GO:0032922	5	0.486814	1.193968	0.240113	0.633790	1.000
56	REACTOME_MYD88_INDEPENDENT_TLR4_CASCADE	R-RNO-166166	8	0.387500	1.184682	0.219020	0.643827	1.000
57	GOBP_NEGATIVE_REGULATION_OF_CYSTEINE_TYPE_ENDO...	GO:2000117	5	0.489926	1.180252	0.230769	0.642828	1.000
58	REACTOME_TOLL_LIKE_RECEPTOR_TLR1_TLR2_CASCADE	R-RNO-168179	8	0.387500	1.178623	0.250765	0.635857	1.000
59	GOBP_DEPHOSPHORYLATION	GO:0016311	7	0.409268	1.169015	0.278515	0.647711	1.000
60	REACTOME_TOLL_LIKE_RECEPTOR_CASCADES	R-RNO-168898	8	0.387500	1.155161	0.249275	0.670074	1.000
61	GOBP_RESPONSE_TO_NERVE_GROWTH_FACTOR	GO:1990089	7	0.413277	1.147483	0.285714	0.677009	1.000
62	GOBP_PROTEIN_DEPHOSPHORYLATION	GO:0006470	7	0.409268	1.144085	0.298851	0.674264	1.000
63	GOBP_NEURON_PROJECTION_GUIDANCE	GO:0097485	9	0.363208	1.120672	0.303681	0.719431	1.000
64	GOBP_RESPONSE_TO_INTERLEUKIN_1	GO:0070555	5	0.457831	1.116054	0.293814	0.719585	1.000
65	REACTOME_TOLL_LIKE_RECEPTOR_9_TLR9_CASCADE	R-RNO-168138	8	0.387500	1.113771	0.280347	0.714299	1.000
66	GOBP_REGULATION_OF_MUSCLE_ADAPTATION	GO:0043502	5	0.452378	1.104137	0.301047	0.726482	1.000
67	GOBP_INOSITOL_LIPID_MEDIATED_SIGNALING	GO:0048017	8	0.377988	1.103656	0.318885	0.716931	1.000
68	GOBP_RAS_PROTEIN_SIGNAL_TRANSDUCTION	GO:0007265	5	0.455482	1.093220	0.321244	0.732153	1.000
69	GOBP_POSITIVE_REGULATION_OF_PEPTIDYL_SERINE_PH...	GO:0033138	8	0.359703	1.084723	0.361979	0.743248	1.000
70	GOBP_POSITIVE_REGULATION_OF_MULTICELLULAR_ORGA...	GO:0051240	32	0.240193	1.084691	0.327465	0.732898	1.000
71	REACTOME_CELL_CYCLE	R-RNO-1640170	6	0.415120	1.083348	0.352632	0.725709	1.000
72	GOBP_POSITIVE_REGULATION_OF_AXONOGENESIS	GO:0050772	5	0.438849	1.078785	0.334239	0.726323	1.000
73	GOBP_MUSCLE_ADAPTATION	GO:0043500	5	0.452378	1.077121	0.330275	0.720460	1.000
74	GOBP_REGULATION_OF_PEPTIDYL_SERINE_PHOSPHORYLA...	GO:0033135	8	0.359703	1.076031	0.335329	0.713469	1.000
75	GOBP_NEGATIVE_REGULATION_OF_CELL_DEATH	GO:0060548	22	0.268185	1.068055	0.332090	0.722301	1.000
76	GOBP_DEVELOPMENTAL_GROWTH	GO:0048589	10	0.333508	1.045291	0.373771	0.768877	1.000
77	GOBP_PROTEIN_MODIFICATION_BY_SMALL_PROTEIN_CON...	GO:0032446	6	0.388088	1.041826	0.388406	0.766668	1.000
78	GOBP_NEURON_DEATH	GO:0070997	18	0.263215	1.032896	0.410256	0.777486	1.000
79	REACTOME_SIGNALING_BY_RECEPTOR_TYROSINE_KINASES	R-RNO-9006934	26	0.229682	1.024655	0.429688	0.788181	1.000
80	GOBP_POSITIVE_REGULATION_OF_CELLULAR_COMPONENT...	GO:0051130	24	0.238158	1.023835	0.423611	0.780463	1.000
81	GOBP_CELLULAR_COMPONENT_MORPHOGENESIS	GO:0032989	24	0.235420	1.022987	0.444444	0.772778	1.000
82	GOBP_CELL_MORPHOGENESIS	GO:0000902	24	0.235420	1.019011	0.422053	0.772606	1.000
83	GOBP_REGULATION_OF_ENDOCYTOSIS	GO:0030100	6	0.389262	1.015818	0.437888	0.770661	1.000

Continuation of Table **S19**

	NAME	ID	SIZE	ES	NES	NOM.p.val	FDR.q.val	FWER.p.val
84	GOBP_NEGATIVE_REGULATION_OF_CELL_POPULATION_PR...	GO:0008285	9	0.329339	1.014069	0.428144	0.765887	1.000
85	GOBP_CELL_MORPHOGENESIS_INVOLVED_IN_DIFFERENTI...	GO:0000904	21	0.241925	1.014042	0.455830	0.757020	1.000
86	GOBP_PEPTIDYL_TYROSINE_MODIFICATION	GO:0018212	10	0.309895	1.013318	0.419753	0.749825	1.000
87	GOBP_CELL_PART_MORPHOGENESIS	GO:0032990	24	0.235420	1.006619	0.415094	0.756027	1.000
88	GOBP_TRANSMEMBRANE_RECEPTOR_PROTEIN_SERINE_THR...	GO:0007178	6	0.378049	1.005526	0.389535	0.750183	1.000
89	GOBP_CELL_MORPHOGENESIS_INVOLVED_IN_NEURON_DIF...	GO:0048667	21	0.241925	0.998708	0.448630	0.757084	1.000
90	GOBP_REGULATION_OF_PROTEOLYSIS	GO:0030162	12	0.288387	0.990185	0.448276	0.768204	1.000
91	GOBP_REGULATION_OF_INTRACELLULAR_SIGNAL_TRANSD...	GO:1902531	21	0.233492	0.988595	0.487719	0.763245	1.000
92	REACTOME_INTERLEUKIN_17_SIGNALING	R-RNO-448424	6	0.378049	0.987267	0.475138	0.758124	1.000
93	GOBP_REGULATION_OF_PHOSPHATIDYLINOSITOL_3_KINA...	GO:0014066	6	0.370835	0.986633	0.431267	0.751468	1.000
94	GOBP_GROWTH	GO:0040007	14	0.262635	0.983833	0.468227	0.749883	1.000
95	GOBP_NEGATIVE_REGULATION_OF_MULTICELLULAR_ORGA...	GO:0051241	15	0.264120	0.975911	0.450658	0.758765	1.000
96	GOBP_POSITIVE_REGULATION_OF_CELL_POPULATION_PR...	GO:0008284	21	0.232286	0.975493	0.473186	0.751821	1.000
97	GOBP_TRANSMEMBRANE_RECEPTOR_PROTEIN_TYROSINE_K...	GO:0007169	19	0.234887	0.960266	0.531773	0.776809	1.000
98	GOBP_POSITIVE_REGULATION_OF_INTRACELLULAR_SIGN...	GO:1902533	14	0.258148	0.944790	0.535928	0.802576	1.000
99	GOBP_POSITIVE_REGULATION_OF_PHOSPHATIDYLINOSIT...	GO:0014068	5	0.378161	0.934229	0.522310	0.817339	1.000
100	REACTOME_MAPK_TARGETS_NUCLEAR_EVENTS_MEDIATED_...	R-HSA-450282	5	0.373494	0.929190	0.524638	0.820399	1.000
101	GOBP_ENDOCYTOSIS	GO:0006897	8	0.317761	0.926144	0.546828	0.818990	1.000
102	GOBP_RESPONSE_TO_GROWTH_FACTOR	GO:0070848	18	0.235347	0.924477	0.568690	0.814326	1.000
103	GOBP_POSITIVE_REGULATION_OF_EPITHELIAL_CELL_MI...	GO:0010634	5	0.375650	0.918646	0.522078	0.819024	1.000
104	GOBP_MUSCLE_CELL_DEVELOPMENT	GO:0055001	5	0.372794	0.915466	0.562857	0.818056	1.000
105	GOBP_CIRCADIAN_RHYTHM	GO:0007623	13	0.256841	0.909546	0.560137	0.822730	1.000
106	GOBP_POSITIVE_REGULATION_OF_PEPTIDYL_TYROSINE_...	GO:0050731	8	0.305413	0.907952	0.557103	0.818416	1.000
107	GOBP_CELLULAR_MACROMOLECULE_CATABOLIC_PROCESS	GO:0044265	6	0.340300	0.901461	0.576819	0.824088	1.000
108	GOBP_OSSIFICATION	GO:0001503	9	0.292325	0.897862	0.587349	0.823741	1.000
109	GOBP_DEVELOPMENTAL_CELL_GROWTH	GO:0048588	5	0.363172	0.893180	0.569892	0.826537	1.000
110	REACTOME_ADAPTIVE_IMMUNE_SYSTEM	R-RNO-1280218	9	0.295496	0.889328	0.573529	0.826876	1.000
111	GOBP_REGULATION_OF_VESICLE_MEDIATED_TRANSPORT	GO:0060627	12	0.251160	0.889098	0.593443	0.820013	1.000
112	GOBP_NEGATIVE_REGULATION_OF_CELL_PROJECTION_OR...	GO:0031345	5	0.368189	0.885217	0.598425	0.820328	1.000
113	GOBP_POSITIVE_REGULATION_OF_ORGANELLE_ORGANIZA...	GO:0010638	10	0.275196	0.883026	0.612613	0.817529	1.000
114	GOBP_CELL_POPULATION_PROLIFERATION	GO:0008283	31	0.194700	0.882057	0.637860	0.812175	1.000
115	GOBP_NEGATIVE_REGULATION_OF_PROTEIN_MODIFICATI...	GO:0031400	10	0.272593	0.876698	0.629179	0.815539	1.000
116	GOBP_GLIOGENESIS	GO:0042063	12	0.256193	0.874804	0.638978	0.812294	1.000
117	GOBP_NEGATIVE_REGULATION_OF_CELL_DEVELOPMENT	GO:0010721	6	0.327420	0.872096	0.610959	0.810770	1.000
118	REACTOME_INNATE_IMMUNE_SYSTEM	R-RNO-168249	16	0.223922	0.864512	0.638095	0.818424	1.000
119	GOBP_RESPONSE_TO_PEPTIDE	GO:1901652	18	0.225209	0.863760	0.621053	0.813164	1.000
120	REACTOME_DEATH_RECEPTOR_SIGNALLING	R-RNO-73887	5	0.356092	0.850111	0.635389	0.832587	1.000
121	GOBP_NEGATIVE_REGULATION_OF_NERVOUS_SYSTEM_DEV...	GO:0051961	5	0.348451	0.847495	0.655353	0.830251	1.000
122	GOBP_POSITIVE_REGULATION_OF_CELL_DIFFERENTIATION	GO:0045597	27	0.196802	0.839909	0.694444	0.837437	1.000
123	GOBP_NEGATIVE_REGULATION_OF_PHOSPHORYLATION	GO:0042326	7	0.301912	0.839595	0.652709	0.831210	1.000
124	GOBP_REGULATION_OF_CIRCADIAN_RHYTHM	GO:0042752	7	0.304121	0.836531	0.663044	0.830127	1.000
125	GOBP_POSITIVE_REGULATION_OF_DEVELOPMENTAL_PROCESS	GO:0051094	32	0.176184	0.832391	0.740458	0.831071	1.000
126	GOBP_TAXIS	GO:0042330	14	0.231693	0.832133	0.674267	0.825044	1.000

Continuation of Table **S19**

	NAME	ID	SIZE	ES	NES	NOM.p.val	FDR.q.val	FWER.p.val
127	GOBP_CELLULAR_GLUCOSE_HOMEOSTASIS	GO:0001678	5	0.346271	0.829617	0.650273	0.823230	1.000
128	GOBP_ENZYME_LINKED_RECEPTOR_PROTEIN_SIGNALING_...	GO:0007167	24	0.187124	0.820410	0.715867	0.833635	1.000
129	REACTOME_SIGNALING_BY_NTRK2_TRKB	R-RNO-9006115	6	0.304272	0.799453	0.712329	0.863987	1.000
130	GOBP_REGULATION_OF_VASCULAR_ASSOCIATED_SMOOTH_...	GO:1904705	5	0.332702	0.791959	0.724868	0.870263	1.000
131	GOBP_POSITIVE_REGULATION_OF_PROTEIN_TYROSINE_K...	GO:0061098	5	0.323331	0.784913	0.731904	0.875942	1.000
132	GOBP_RESPONSE_TO_INSULIN	GO:0032868	5	0.326827	0.781643	0.735897	0.874723	1.000
133	GOBP_REGULATION_OF_PROTEIN_TYROSINE_KINASE_ACT...	GO:0061097	5	0.323331	0.779706	0.740053	0.871251	1.000
134	GOBP_FC_RECEPTOR_SIGNALING_PATHWAY	GO:0038093	8	0.258225	0.766793	0.807580	0.886239	1.000
135	GOBP_REGULATION_OF_AXONOGENESIS	GO:0050770	7	0.267458	0.761439	0.755043	0.888616	1.000
136	GOBP_ESTABLISHMENT_OF_PROTEIN_LOCALIZATION_TO_...	GO:0072594	6	0.290600	0.759529	0.744565	0.885148	1.000
137	GOBP_IMMUNE_RESPONSE_REGULATING_SIGNALING_PATHWAY	GO:0002764	8	0.258225	0.759285	0.735736	0.879044	1.000
138	GOBP_NEGATIVE_REGULATION_OF_INTRACELLULAR_SIGN...	GO:1902532	7	0.264403	0.749274	0.750000	0.887674	1.000
139	GOBP_RESPONSE_TO_CALCIUM_ION	GO:0051592	6	0.279673	0.737742	0.810959	0.899069	1.000
140	GOBP_MYELOID_LEUKOCYTE_DIFFERENTIATION	GO:0002573	11	0.218297	0.717416	0.853035	0.922017	1.000
141	REACTOME_EXTRACELLULAR_MATRIX_ORGANIZATION	R-RNO-1474244	5	0.279941	0.710499	0.818182	0.925219	1.000
142	REACTOME_TRANSCRIPTIONAL_REGULATION_BY_TP53	R-RNO-3700989	6	0.269051	0.708610	0.835165	0.921394	1.000
143	GOBP_REGULATION_OF_RESPONSE_TO_EXTERNAL_STIMULUS	GO:0032101	15	0.191644	0.699205	0.875000	0.927876	1.000
144	REACTOME_CIRCADIAN_CLOCK	R-HSA-400253	5	0.273243	0.671574	0.890710	0.954912	1.000
145	GOBP_CELLULAR_COMPONENT_MAINTENANCE	GO:0043954	5	0.277108	0.653934	0.899244	0.967523	1.000
146	GOBP_REPRODUCTION	GO:0000003	18	0.160220	0.653657	0.908127	0.961271	1.000
147	REACTOME_G_ALPHA_Q_SIGNALLING_EVENTS	R-RNO-416476	6	0.243902	0.649381	0.924471	0.958618	1.000
148	GOBP_MYELOID_LEUKOCYTE_MEDIATED_IMMUNITY	GO:0002444	6	0.230647	0.628173	0.945409	0.971514	1.000
149	REACTOME_NEUTROPHIL_DEGRANULATION	R-RNO-6798695	6	0.230647	0.621796	0.942779	0.970507	1.000
150	GOBP_CELL_ACTIVATION_INVOLVED_IN_IMMUNE_RESPONSE	GO:0002263	6	0.230647	0.608382	0.958904	0.974498	1.000
151	GOBP_NEGATIVE_REGULATION_OF_RESPONSE_TO_STIMULUS	GO:0048585	16	0.154738	0.588399	0.957929	0.980415	1.000
152	GOBP_REGULATION_OF_CYTOSKELETON_ORGANIZATION	GO:0051493	5	0.224575	0.554040	0.984127	0.990694	1.000
153	GOBP_DEVELOPMENTAL_MATURATION	GO:0021700	5	0.218154	0.551313	0.972900	0.985373	1.000
154	GOBP_REGULATION_OF_RECEPTOR_MEDIATED_ENDOCYTOSIS	GO:0048259	5	0.216867	0.538108	0.977143	0.983573	1.000
155	GOBP_NEGATIVE_REGULATION_OF_PROTEIN_CATABOLIC_...	GO:0042177	5	-0.216810	-0.478337	0.988449	0.989492	1.000
156	GOBP_RESPONSE_TO_OXIDATIVE_STRESS	GO:0006979	8	-0.193124	-0.492650	0.983824	0.987941	1.000
157	GOBP_RESPONSE_TO_REACTIVE_OXYGEN_SPECIES	GO:0000302	8	-0.193124	-0.494061	0.992296	0.989300	1.000
158	GOBP_POSITIVE_REGULATION_OF_MYELOID_LEUKOCYTE_...	GO:0002763	6	-0.221299	-0.514193	0.984102	0.984878	1.000
159	GOBP_REGULATION_OF_MYELOID_LEUKOCYTE_DIFFERENT...	GO:0002761	8	-0.203522	-0.523400	0.974852	0.983415	1.000
160	GOBP_POSITIVE_REGULATION_OF_MYELOID_CELL_DIFFE...	GO:0045639	7	-0.217987	-0.524860	0.969088	0.984618	1.000
161	GOBP_CELLULAR_RESPONSE_TO_ABIOTIC_STIMULUS	GO:0071214	7	-0.215440	-0.545773	0.964451	0.977746	1.000
162	HALLMARK_TNFA_SIGNALING_VIA_NFKB	M5890	14	-0.180369	-0.550461	0.970845	0.977218	1.000
163	GOBP_RECEPTOR_MEDIATED_ENDOCYTOSIS	GO:0006898	6	-0.243525	-0.565002	0.959055	0.971348	1.000
164	GOBP_G_PROTEIN_COUPLED_GLUTAMATE_RECEPTOR_SIGN...	GO:0007216	8	-0.219274	-0.569855	0.950151	0.970416	1.000
165	GOBP_LEUKOCYTE_MEDIATED_IMMUNITY	GO:0002443	7	-0.231359	-0.571788	0.957878	0.971020	1.000
166	GOBP_MYELOID_CELL_DIFFERENTIATION	GO:0030099	13	-0.194892	-0.573897	0.944776	0.971565	1.000
167	GOBP_CELLULAR_RESPONSE_TO_CHEMICAL_STRESS	GO:0062197	7	-0.241171	-0.593668	0.925117	0.960420	1.000
168	GOBP_NEGATIVE_REGULATION_OF_SIGNALING	GO:0023057	21	-0.172317	-0.596654	0.931507	0.960159	1.000
169	GOBP_CELLULAR_RESPONSE_TO_REACTIVE_OXYGEN_SPECIES	GO:0034614	7	-0.241171	-0.601966	0.937042	0.958073	1.000

Continuation of Table **S19**

	NAME	ID	SIZE	ES	NES	NOM.p.val	FDR.q.val	FWER.p.val
170	REACTOME_NUCLEAR_EVENTS_KINASE_AND_TRANSCRIPTI...	R-RNO-198725	11	-0.213670	-0.606468	0.928465	0.956486	1.000
171	GOBP_REGULATION_OF_ORGANELLE_ORGANIZATION	GO:0033043	14	-0.203183	-0.607879	0.924638	0.957175	1.000
172	GOBP_NEGATIVE_REGULATION_OF_NUCLEOBASE_CONTAIN...	GO:0045934	13	-0.203541	-0.617184	0.906021	0.951579	1.000
173	GOBP_RESPONSE_TO_UV	GO:0009411	5	-0.280211	-0.621026	0.935430	0.950225	1.000
174	GOBP_CELLULAR_RESPONSE_TO_EXTRACELLULAR_STIMULUS	GO:0031668	8	-0.238400	-0.622128	0.904687	0.951089	1.000
175	REACTOME_TRAFFICKING_OF_GLUR2_CONTAINING_AMPA_...	R-RNO-416993	8	-0.244391	-0.628898	0.917933	0.947131	1.000
176	GOBP_REGULATION_OF_WNT_SIGNALING_PATHWAY	GO:0030111	5	-0.283753	-0.631496	0.932496	0.946580	1.000
177	GOBP_CELL_CELL_SIGNALING_BY_WNT	GO:0198738	7	-0.261444	-0.642730	0.905054	0.937927	1.000
178	GOBP_RESPONSE_TO_CADMIUM_ION	GO:0046686	6	-0.276102	-0.647506	0.905132	0.935300	1.000
179	GOBP_GLIAL_CELL_DIFFERENTIATION	GO:0010001	10	-0.234361	-0.650355	0.896296	0.934345	1.000
180	GOBP_REGULATION_OF_MYELOID_CELL_DIFFERENTIATION	GO:0045637	9	-0.237986	-0.651613	0.889222	0.934854	1.000
181	GOBP_POSITIVE_REGULATION_OF_NUCLEOBASE_CONTAIN...	GO:0045935	28	-0.178292	-0.655201	0.884097	0.933197	1.000
182	REACTOME_CLASS_C_3_METABOTROPIC_GLUTAMATE_PHER...	R-RNO-420499	7	-0.270473	-0.665859	0.862442	0.924447	1.000
183	GOBP_INTRACELLULAR_RECEPTOR_SIGNALING_PATHWAY	GO:0030522	5	-0.311048	-0.670695	0.889984	0.921375	1.000
184	GOBP_POSITIVE_REGULATION_OF_CATION_TRANSMEMBRA...	GO:1904064	5	-0.302928	-0.672167	0.889968	0.921559	1.000
185	GOBP_APOPTOTIC_SIGNALING_PATHWAY	GO:0097190	15	-0.218418	-0.674857	0.880597	0.920520	1.000
186	GOBP_MEMBRANE_PROTEIN_PROTEOLYSIS	GO:0033619	6	-0.294101	-0.681947	0.870253	0.914634	1.000
187	GOBP_EXOCYTOSIS	GO:0006887	11	-0.236962	-0.685883	0.854197	0.912216	1.000
188	GOBP_MULTI_ORGANISM_PROCESS	GO:0051704	16	-0.212412	-0.686118	0.860465	0.913661	1.000
189	GOBP_EPIDERMIS_DEVELOPMENT	GO:0008544	5	-0.312094	-0.695811	0.862237	0.904629	1.000
190	GOBP_NEGATIVE_REGULATION_OF_RESPONSE_TO_EXTERN...	GO:0032102	5	-0.312990	-0.695979	0.857820	0.906159	1.000
191	REACTOME_SIGNALING_BY_INTERLEUKINS	R-RNO-449147	15	-0.218145	-0.696064	0.853557	0.907777	1.000
192	GOBP_RESPONSE_TO_INORGANIC_SUBSTANCE	GO:0010035	19	-0.208576	-0.698735	0.841160	0.906495	1.000
193	GOBP_EPITHELIAL_CELL_DIFFERENTIATION	GO:0030855	7	-0.289977	-0.700842	0.838473	0.905845	1.000
194	REACTOME_INTERLEUKIN_4_AND_INTERLEUKIN_13_SIGN...	R-RNO-6785807	8	-0.271211	-0.705093	0.845921	0.902560	1.000
195	GOBP_POSITIVE_REGULATION_OF_BIOSYNTHETIC_PROCESS	GO:0009891	27	-0.195950	-0.705130	0.855014	0.904228	1.000
196	GOBP_REGULATION_OF_OSTEOCLAST_DIFFERENTIATION	GO:0045670	5	-0.318916	-0.711721	0.824121	0.897970	1.000
197	GOBP_RECEPTOR_METABOLIC_PROCESS	GO:0043112	5	-0.324880	-0.711824	0.849693	0.899572	1.000
198	GOBP_RECEPTOR_INTERNALIZATION	GO:0031623	5	-0.324880	-0.712408	0.854200	0.900732	1.000
199	GOBP_ADENYLATE_CYCLASE_INHIBITING_G_PROTEIN_CO...	GO:0007196	5	-0.325373	-0.716630	0.834356	0.897477	1.000
200	GOBP_REGULATION_OF_ANATOMICAL_STRUCTURE_MORPHO...	GO:0022603	17	-0.218761	-0.720172	0.812325	0.894997	1.000
201	REACTOME_PLATELET_ACTIVATION_SIGNALING_AND_AGG...	R-HSA-76002	7	-0.289228	-0.721285	0.824534	0.895440	1.000
202	GOBP_RESPONSE_TO_ALKALOID	GO:0043279	5	-0.326817	-0.724384	0.827974	0.893429	1.000
203	GOBP_FAT_CELL_DIFFERENTIATION	GO:0045444	9	-0.267182	-0.724990	0.802711	0.894405	1.000
204	GOBP_NEGATIVE_REGULATION_OF_GENE_EXPRESSION	GO:0010629	12	-0.249044	-0.725765	0.818689	0.895138	1.000
205	GOBP_PROTEIN_LOCALIZATION_TO_ORGANELLE	GO:0033365	9	-0.268830	-0.727100	0.813846	0.895210	1.000
206	GOBP_CELLULAR_RESPONSE_TO_CADMIUM_ION	GO:0071276	5	-0.334615	-0.729147	0.843849	0.894329	1.000
207	GOBP_ENDOMEMBRANE_SYSTEM_ORGANIZATION	GO:0010256	6	-0.312383	-0.730533	0.797468	0.894425	1.000
208	REACTOME_TRANSCRIPTIONAL_REGULATION_OF_WHITE_A...	R-RNO-381340	6	-0.315745	-0.730972	0.819149	0.895643	1.000
209	GOBP_IMMUNE_SYSTEM_DEVELOPMENT	GO:0002520	17	-0.228748	-0.749271	0.771709	0.874415	1.000
210	GOBP_POSITIVE_REGULATION_OF_CELL_PROJECTION_OR...	GO:0031346	10	-0.267422	-0.750112	0.773529	0.875089	1.000
211	GOBP_CELL_CYCLE_PROCESS	GO:0022402	11	-0.262715	-0.751268	0.796160	0.875304	1.000
212	GOBP_RESPONSE_TO_CARBOHYDRATE	GO:0009743	5	-0.349136	-0.759058	0.775244	0.866819	1.000

Continuation of Table **S19**

	NAME	ID	SIZE	ES	NES	NOM.p.val	FDR.q.val	FWER.p.val
213	REACTOME_NGF_STIMULATED_TRANSCRIPTION	R-RNO-9031628	9	-0.277320	-0.759388	0.797317	0.868114	1.000
214	GOBP_RESPONSE_TO_MONOSACCHARIDE	GO:0034284	5	-0.349136	-0.763212	0.794992	0.864620	1.000
215	GOBP_NEGATIVE_REGULATION_OF_TRANSFERASE_ACTIVITY	GO:0051348	5	-0.352325	-0.764850	0.801262	0.864059	1.000
216	GOBP_POSITIVE_REGULATION_OF_GENE_EXPRESSION	GO:0010628	16	-0.240542	-0.766466	0.739766	0.863703	1.000
217	GOBP_REGULATION_OF_HEMOPOIESIS	GO:1903706	10	-0.281724	-0.766568	0.777280	0.865292	1.000
218	GOBP_NEGATIVE_REGULATION_OF_KINASE_ACTIVITY	GO:0033673	5	-0.352325	-0.770150	0.783654	0.862024	1.000
219	GOBP_RESPONSE_TO_METAL_ION	GO:0010038	15	-0.245883	-0.770387	0.737752	0.863382	1.000
220	HALLMARK_HYPOXIA	M5891	5	-0.350418	-0.772066	0.802817	0.862738	1.000
221	GOBP_DNA_METABOLIC_PROCESS	GO:0006259	6	-0.329523	-0.772090	0.752280	0.864452	1.000
222	GOBP_REGULATION_OF_MUSCLE_CELL_DIFFERENTIATION	GO:0051147	6	-0.334677	-0.774887	0.754977	0.862413	1.000
223	GOBP_OSTEOCLAST_DIFFERENTIATION	GO:0030316	6	-0.329268	-0.780695	0.770291	0.855920	1.000
224	GOBP_SYNAPTIC_TRANSMISSION_GLUTAMATERGIC	GO:0035249	13	-0.262932	-0.784731	0.756637	0.851929	1.000
225	GOBP_REGULATION_OF_PROTEIN_STABILITY	GO:0031647	5	-0.360329	-0.785335	0.764706	0.852799	1.000
226	GOBP_REGULATION_OF_GROWTH	GO:0040008	13	-0.259519	-0.785883	0.742358	0.853817	1.000
227	GOBP_REGULATION_OF_DEFENSE_RESPONSE	GO:0031347	7	-0.316721	-0.785921	0.735669	0.855502	1.000
228	GOBP_NEGATIVE_REGULATION_OF_PHOSPHORUS_METABOL...	GO:0010563	9	-0.299500	-0.788221	0.724458	0.854087	1.000
229	GOBP_POSITIVE_REGULATION_OF_TRANSCRIPTION_BY_R...	GO:0045944	24	-0.224299	-0.788419	0.737748	0.855545	1.000
230	GOBP_REGULATION_OF_INFLAMMATORY_RESPONSE	GO:0050727	7	-0.316721	-0.790661	0.714286	0.854050	1.000
231	GOBP_AXON_DEVELOPMENT	GO:0061564	18	-0.239499	-0.795630	0.737796	0.848623	1.000
232	GOBP_LIPID_EXPORT_FROM_CELL	GO:0140353	5	-0.361199	-0.796504	0.770000	0.849207	1.000
233	GOBP_POSITIVE_REGULATION_OF_NEURON_PROJECTION_...	GO:0010976	5	-0.362405	-0.807342	0.717156	0.835262	1.000
234	GOBP_IMMUNE_EFFECTOR_PROCESS	GO:0002252	10	-0.290836	-0.810351	0.723781	0.832654	1.000
235	REACTOME_CELLULAR_RESPONSES_TO_EXTERNAL_STIMULI	R-RNO-8953897	12	-0.272210	-0.815441	0.705185	0.826919	1.000
236	GOBP_NEGATIVE_REGULATION_OF_TRANSCRIPTION_BY_R...	GO:0000122	10	-0.294302	-0.819400	0.695586	0.822517	1.000
237	GOBP_NEGATIVE_REGULATION_OF_ION_TRANSPORT	GO:0043271	11	-0.284096	-0.819880	0.706408	0.823522	1.000
238	GOBP_POSITIVE_REGULATION_OF_HYDROLASE_ACTIVITY	GO:0051345	16	-0.257597	-0.820083	0.686630	0.824902	1.000
239	GOBP_REGULATION_OF_HYDROLASE_ACTIVITY	GO:0051336	22	-0.231034	-0.822310	0.690608	0.823318	1.000
240	GOBP_REGULATION_OF_LEUKOCYTE_DIFFERENTIATION	GO:1902105	9	-0.312046	-0.827982	0.688550	0.816727	1.000
241	REACTOME_G_ALPHA_Z_SIGNALLING_EVENTS	R-RNO-418597	5	-0.377357	-0.828000	0.725240	0.818406	1.000
242	GOBP_MUSCLE_CELL_PROLIFERATION	GO:0033002	8	-0.319296	-0.832926	0.677130	0.812693	1.000
243	GOBP_MUSCLE_CONTRACTION	GO:0006936	5	-0.377676	-0.833988	0.678333	0.812861	1.000
244	GOBP_REGULATION_OF_NEURONAL_SYNAPTIC_PLASTICITY	GO:0048168	7	-0.338107	-0.834045	0.682109	0.814505	1.000
245	GOBP_REGULATION_OF_SYNAPTIC_TRANSMISSION_GLUTA...	GO:0051966	12	-0.287141	-0.836542	0.662558	0.812395	1.000
246	REACTOME_INTEGRATION_OF_ENERGY_METABOLISM	R-RNO-163685	5	-0.382286	-0.844165	0.682770	0.802633	1.000
247	GOBP_ERBB_SIGNALING_PATHWAY	GO:0038127	5	-0.387135	-0.845756	0.683465	0.801862	1.000
248	REACTOME_PTEN_REGULATION	R-RNO-6807070	5	-0.392361	-0.847346	0.688119	0.800929	1.000
249	GOBP_REGULATION_OF_PEPTIDE_TRANSPORT	GO:0090087	10	-0.306555	-0.848186	0.655275	0.801412	1.000
250	GOBP_REPRODUCTIVE_SYSTEM_DEVELOPMENT	GO:0061458	6	-0.366360	-0.849486	0.663974	0.801059	1.000
251	REACTOME_HEMOSTASIS	R-RNO-109582	14	-0.270109	-0.853170	0.638849	0.796822	1.000
252	GOBP_POSITIVE_REGULATION_OF_CELLULAR_PROTEIN_L...	GO:1903829	7	-0.344320	-0.853286	0.663522	0.798319	1.000
253	GOBP_LEUKOCYTE_DIFFERENTIATION	GO:0002521	15	-0.269955	-0.858602	0.660511	0.791633	1.000
254	HALLMARK_UV_RESPONSE_UP	M5941	5	-0.386158	-0.866742	0.644817	0.780346	1.000
255	REACTOME_NERVOUS_SYSTEM_DEVELOPMENT	R-RNO-9675108	15	-0.276845	-0.868774	0.649783	0.778747	1.000

Continuation of Table **S19**

	NAME	ID	SIZE	ES	NES	NOM.p.val	FDR.q.val	FWER.p.val
256	GOBP_AMINO_ACID_TRANSPORT	GO:0006865	6	-0.377787	-0.869132	0.643518	0.779782	1.000
257	GOBP_GENERATION_OF_PRECURSOR_METABOLITES_AND_E...	GO:0006091	5	-0.391059	-0.869932	0.660626	0.780172	1.000
258	HALLMARK_EPITHELIAL_MESENCHYMAL_TRANSITION	M5930	5	-0.399774	-0.872622	0.641694	0.777565	1.000
259	GOBP_ACIDIC_AMINO_ACID_TRANSPORT	GO:0015800	5	-0.400030	-0.874559	0.640063	0.776048	1.000
260	GOBP_FOREBRAIN_DEVELOPMENT	GO:0030900	9	-0.327152	-0.876378	0.620214	0.774754	1.000
261	GOBP_MONOCARBOXYLIC_ACID_TRANSPORT	GO:0015718	5	-0.400030	-0.877846	0.647152	0.773952	1.000
262	GOBP_NEURON_DIFFERENTIATION	GO:0030182	36	-0.230265	-0.878739	0.625806	0.774156	1.000
263	REACTOME_CYTOKINE_SIGNALING_IN_IMMUNE_SYSTEM	R-RNO-1280215	21	-0.253180	-0.880151	0.616848	0.773503	1.000
264	GOBP_NEGATIVE_REGULATION_OF_APOPTOTIC_SIGNALIN...	GO:2001234	7	-0.358084	-0.883371	0.612598	0.769735	1.000
265	GOBP_CELL_GROWTH	GO:0016049	10	-0.320079	-0.884379	0.620061	0.769689	1.000
266	GOBP_SECOND_MESSENGER_MEDIATED_SIGNALING	GO:0019932	16	-0.275405	-0.884474	0.602837	0.771188	1.000
267	GOBP_POLYSACCHARIDE_METABOLIC_PROCESS	GO:0005976	5	-0.404792	-0.884751	0.626959	0.772424	1.000
268	GOBP_RESPONSE_TO_RADIATION	GO:0009314	13	-0.296204	-0.886404	0.608069	0.771471	1.000
269	HALLMARK_ALLOGRAFT_REJECTION	M5950	7	-0.350936	-0.889334	0.594679	0.768374	1.000
270	GOBP_NEGATIVE_REGULATION_OF_TRANSMEMBRANE_TRAN...	GO:0034763	6	-0.384651	-0.889589	0.623211	0.769641	1.000
271	GOBP_DICARBOXYLIC_ACID_TRANSPORT	GO:0006835	5	-0.400030	-0.889811	0.617323	0.770939	1.000
272	GOBP_REGULATION_OF_ANION_TRANSPORT	GO:0044070	20	-0.267639	-0.891794	0.593620	0.769456	1.000
273	GOBP_MYELOID_LEUKOCYTE_ACTIVATION	GO:0002274	8	-0.346707	-0.892671	0.609984	0.769696	1.000
274	GOBP_MUSCLE_CELL_DIFFERENTIATION	GO:0042692	10	-0.322350	-0.894323	0.602446	0.768631	1.000
275	GOBP_CARBOXYLIC_ACID_TRANSPORT	GO:0046942	6	-0.377787	-0.894904	0.599686	0.769262	1.000
276	GOBP_ORGANIC_ANION_TRANSPORT	GO:0015711	6	-0.377787	-0.894918	0.624801	0.770978	1.000
277	GOBP_REGULATION_OF_HORMONE_LEVELS	GO:0010817	12	-0.304395	-0.899804	0.603107	0.764228	1.000
278	GOBP_REGULATION_OF_DEVELOPMENTAL_GROWTH	GO:0048638	9	-0.333877	-0.900555	0.600299	0.764750	1.000
279	GOBP_POSITIVE_REGULATION_OF_DEVELOPMENTAL_GROWTH	GO:0048639	8	-0.348332	-0.902195	0.600000	0.763601	1.000
280	GOBP_CELLULAR_AMIDE_METABOLIC_PROCESS	GO:0043603	13	-0.298001	-0.902775	0.567208	0.764326	1.000
281	GOBP_REGULATION_OF_NEURON_PROJECTION_DEVELOPMENT	GO:0010975	16	-0.286580	-0.906522	0.556369	0.759731	1.000
282	GOBP_LOCOMOTION	GO:0040011	31	-0.245498	-0.908095	0.574324	0.758782	1.000
283	GOBP_RESPONSE_TO_WOUNDING	GO:0009611	15	-0.290837	-0.908517	0.570213	0.759803	1.000
284	REACTOME_GPCR_LIGAND_BINDING	R-RNO-500792	8	-0.351378	-0.911322	0.585404	0.756749	1.000
285	GOBP_POSITIVE_REGULATION_OF_PEPTIDASE_ACTIVITY	GO:0010952	7	-0.370545	-0.911348	0.598425	0.758431	1.000
286	GOBP_NEGATIVE_REGULATION_OF_BIOSYNTHETIC_PROCESS	GO:0009890	13	-0.302366	-0.913161	0.585477	0.757014	1.000
287	GOBP_ORGANONITROGEN_COMPOUND_CATABOLIC_PROCESS	GO:1901565	9	-0.334709	-0.913310	0.569925	0.758465	1.000
288	GOBP_ORGANIC_HYDROXY_COMPOUND_METABOLIC_PROCESS	GO:1901615	7	-0.371510	-0.913924	0.571865	0.759191	1.000
289	GOBP_CELLULAR_RESPONSE_TO_PEPTIDE	GO:1901653	13	-0.297408	-0.916727	0.549708	0.756209	1.000
290	GOBP_NEUROGENESIS	GO:0022008	40	-0.236953	-0.916868	0.568063	0.757728	1.000
291	GOBP_POSITIVE_REGULATION_OF_NEURON_DEATH	GO:1901216	5	-0.425528	-0.919531	0.574713	0.754904	1.000
292	GOBP_DENDRITE_MORPHOGENESIS	GO:0048813	6	-0.389871	-0.922603	0.579200	0.751296	1.000
293	GOBP_CELL_PROJECTION_ORGANIZATION	GO:0030030	34	-0.245128	-0.927658	0.576000	0.744303	1.000
294	GOBP_PROTEOLYSIS	GO:0006508	17	-0.286877	-0.928002	0.555862	0.745388	1.000
295	GOBP_NEGATIVE_REGULATION_OF_CATALYTIC_ACTIVITY	GO:0043086	17	-0.282907	-0.928426	0.545455	0.746474	1.000
296	GOBP_REGULATION_OF_PEPTIDASE_ACTIVITY	GO:0052547	10	-0.332458	-0.930334	0.564829	0.744843	1.000
297	GOBP_POSITIVE_REGULATION_OF_CYSTEINE_TYPE_ENDO...	GO:2001056	7	-0.370545	-0.930947	0.546154	0.745555	1.000
298	GOBP_CELLULAR_RESPONSE_TO_INORGANIC_SUBSTANCE	GO:0071241	11	-0.315615	-0.932828	0.526786	0.744035	1.000

Continuation of Table **S19**

	NAME	ID	SIZE	ES	NES	NOM.p.val	FDR.q.val	FWER.p.val
299	GOBP_SMOOTH_MUSCLE_CELL_PROLIFERATION	GO:0048659	6	-0.398447	-0.934553	0.576299	0.742700	1.000
300	GOBP_STRIATED_MUSCLE_CELL_DIFFERENTIATION	GO:0051146	9	-0.341317	-0.937127	0.564897	0.739773	1.000
301	GOBP_NEGATIVE_REGULATION_OF_CELLULAR_COMPONENT...	GO:0051129	11	-0.325939	-0.938715	0.547865	0.738658	1.000
302	GOBP_ACTIVATION_OF_IMMUNE_RESPONSE	GO:0002253	5	-0.428702	-0.941001	0.565284	0.736407	1.000
303	GOBP_PROTEIN_CATABOLIC_PROCESS	GO:0030163	8	-0.364916	-0.941266	0.526946	0.737698	1.000
304	GOBP_RESPONSE_TO_LIGHT_STIMULUS	GO:0009416	11	-0.331621	-0.942911	0.542522	0.736741	1.000
305	GOBP_RESPONSE_TO_EXTRACELLULAR_STIMULUS	GO:0009991	13	-0.319965	-0.943742	0.546742	0.736956	1.000
306	GOBP_ADENYLATE_CYCLASE_INHIBITING_G_PROTEIN_CO...	GO:0007193	9	-0.339965	-0.945407	0.537178	0.735814	1.000
307	GOBP_MUSCLE_SYSTEM_PROCESS	GO:0003012	9	-0.345063	-0.946457	0.531531	0.735615	1.000
308	GOBP_POSITIVE_REGULATION_OF_PROTEIN_MODIFICATI...	GO:0031401	23	-0.268879	-0.946773	0.540136	0.736772	1.000
309	GOBP_AGING	GO:0007568	13	-0.309497	-0.948457	0.546529	0.735278	1.000
310	GOBP_POSITIVE_REGULATION_OF_HEMOPOIESIS	GO:1903708	7	-0.384439	-0.949407	0.543413	0.735218	1.000
311	GOBP_WOUND_HEALING	GO:0042060	13	-0.314736	-0.950013	0.532394	0.735951	1.000
312	GOBP_REGULATION_OF_PROTEIN_MODIFICATION_PROCESS	GO:0031399	29	-0.255617	-0.950032	0.535763	0.737736	1.000
313	GOBP_INTRINSIC_APOPTOTIC_SIGNALING_PATHWAY	GO:0097193	5	-0.428665	-0.950756	0.541735	0.738263	1.000
314	GOBP_POSITIVE_REGULATION_OF_PROTEIN_PHOSPHORYL...	GO:0001934	23	-0.268879	-0.951544	0.537271	0.738719	1.000
315	GOBP_REGULATION_OF_CYSTEINE_TYPE_ENDOPEPTIDASE...	GO:2000116	9	-0.354468	-0.952869	0.531758	0.738182	1.000
316	GOBP_REGULATION_OF_HORMONE_SECRETION	GO:0046883	8	-0.369613	-0.954904	0.510355	0.736154	1.000
317	GOBP_ANION_TRANSMEMBRANE_TRANSPORT	GO:0098656	7	-0.388139	-0.959733	0.529595	0.729021	1.000
318	GOBP_CELLULAR_RESPONSE_TO_EXTERNAL_STIMULUS	GO:0071496	10	-0.345361	-0.960914	0.517291	0.728628	1.000
319	REACTOME_ESR_MEDIATED_SIGNALING	R-RNO-8939211	8	-0.373438	-0.962505	0.503771	0.727455	1.000
320	GOBP_RESPONSE_TO_MECHANICAL_STIMULUS	GO:0009612	6	-0.412773	-0.962693	0.512618	0.728910	1.000
321	GOBP_POSITIVE_REGULATION_OF_PHOSPHORUS_METABOL...	GO:0010562	24	-0.278428	-0.964034	0.533145	0.728296	1.000
322	REACTOME_SIGNALING_BY_NUCLEAR_RECEPTORS	R-RNO-9006931	8	-0.373438	-0.964499	0.504532	0.729301	1.000
323	GOBP_CELLULAR_MACROMOLECULE_LOCALIZATION	GO:0070727	21	-0.278806	-0.968014	0.476124	0.724269	1.000
324	GOBP_APOPTOTIC_PROCESS	GO:0006915	35	-0.257820	-0.968184	0.516689	0.725827	1.000
325	GOBP_REGULATION_OF_ANATOMICAL_STRUCTURE_SIZE	GO:0090066	10	-0.345788	-0.968624	0.485755	0.726905	1.000
326	GOBP_NEURON_DEVELOPMENT	GO:0048666	33	-0.253922	-0.970567	0.506089	0.725102	1.000
327	GOBP_VESICLE_MEDIATED_TRANSPORT_IN_SYNAPSE	GO:0099003	7	-0.398980	-0.972332	0.503077	0.723649	1.000
328	GOBP_CARBOHYDRATE_METABOLIC_PROCESS	GO:0005975	9	-0.359253	-0.973079	0.506647	0.724113	1.000
329	GOBP_RESPONSE_TO_PEPTIDE_HORMONE	GO:0043434	13	-0.320752	-0.973571	0.486956	0.725045	1.000
330	GOBP_REGULATION_OF_NERVOUS_SYSTEM_PROCESS	GO:0031644	8	-0.373448	-0.974197	0.525926	0.725663	1.000
331	GOBP_REGULATION_OF_CELL_SIZE	GO:0008361	5	-0.446919	-0.976029	0.512945	0.724062	1.000
332	HALLMARK_APOPTOSIS	M5902	6	-0.426829	-0.979121	0.497653	0.719819	1.000
333	GOBP_REGULATION_OF_CELL_CYCLE_PROCESS	GO:0010564	5	-0.449546	-0.980811	0.487220	0.718478	1.000
334	GOBP_CELLULAR_CARBOHYDRATE_METABOLIC_PROCESS	GO:0044262	5	-0.451188	-0.981542	0.484520	0.719017	1.000
335	GOBP_CELLULAR_RESPONSE_TO_OXYGEN_CONTAINING_CO...	GO:1901701	29	-0.265757	-0.982574	0.485830	0.718942	1.000
336	REACTOME_RNA_POLYMERASE_II_TRANSCRIPTION	R-RNO-73857	20	-0.290317	-0.982988	0.495105	0.720062	1.000
337	GOBP_POSITIVE_REGULATION_OF_TRANSMEMBRANE_TRAN...	GO:0034764	7	-0.393066	-0.984528	0.493865	0.718955	1.000
338	GOBP_POSITIVE_REGULATION_OF_TRANSPORT	GO:0051050	26	-0.276812	-0.985001	0.497997	0.720000	1.000
339	GOBP_NEGATIVE_REGULATION_OF_TRANSPORT	GO:0051051	13	-0.331129	-0.986447	0.475714	0.718978	1.000
340	GOBP_POSITIVE_REGULATION_OF_GROWTH	GO:0045927	9	-0.367439	-0.986500	0.483434	0.720763	1.000
341	GOBP_PROTEIN_MODIFICATION_BY_SMALL_PROTEIN_CON...	GO:0070647	7	-0.401263	-0.986768	0.481833	0.722124	1.000

Continuation of Table **S19**

	NAME	ID	SIZE	ES	NES	NOM.p.val	FDR.q.val	FWER.p.val
342	REACTOME_SIGNALING_BY_RHO_GTPASES_MIRO_GTPASES...	R-RNO-9716542	9	-0.365638	-0.988068	0.488954	0.721460	1.000
343	GOBP_NEGATIVE_REGULATION_OF_HYDROLASE_ACTIVITY	GO:0051346	11	-0.342233	-0.988672	0.466872	0.722172	1.000
344	GOBP_REGULATION_OF_PROTEIN_CATABOLIC_PROCESS	GO:0042176	7	-0.400422	-0.989379	0.490712	0.722715	1.000
345	GOBP_POSITIVE_REGULATION_OF_RESPONSE_TO_EXTERN...	GO:0032103	9	-0.367555	-0.989817	0.483871	0.723755	1.000
346	GOBP_ESTABLISHMENT_OF_PROTEIN_LOCALIZATION	GO:0045184	18	-0.299860	-0.991287	0.473118	0.722888	1.000
347	REACTOME_RHO_GTPASE_EFFECTORS	R-RNO-195258	9	-0.365638	-0.994973	0.473354	0.717316	1.000
348	GOBP_RESPONSE_TO_NITROGEN_COMPOUND	GO:1901698	34	-0.266885	-0.996718	0.472703	0.715862	1.000
349	GOBP_REGULATION_OF_POSTSYNAPSE_ORGANIZATION	GO:0099175	6	-0.425630	-0.997498	0.487692	0.716241	1.000
350	GOBP_POSITIVE_REGULATION_OF_PRI_MIRNA_TRANSCRI...	GO:1902895	7	-0.409414	-0.998037	0.465732	0.717158	1.000
351	GOBP_MULTI_MULTICELLULAR_ORGANISM_PROCESS	GO:0044706	8	-0.381563	-0.999175	0.482707	0.716811	1.000
352	GOBP_DENDRITE_DEVELOPMENT	GO:0016358	9	-0.378092	-1.001311	0.478807	0.714550	1.000
353	GOBP_REGULATION_OF_IMMUNE_RESPONSE	GO:0050776	12	-0.342849	-1.005521	0.462304	0.708107	1.000
354	GOBP_MUSCLE_STRUCTURE_DEVELOPMENT	GO:0061061	16	-0.318472	-1.005947	0.454810	0.709117	1.000
355	GOBP_REGULATION_OF_CELL_DEATH	GO:0010941	34	-0.267575	-1.009335	0.450875	0.704441	1.000
356	GOBP_RESPONSE_TO_ENDOGENOUS_STIMULUS	GO:0009719	34	-0.263912	-1.010844	0.442257	0.703465	1.000
357	GOBP_DEVELOPMENTAL_PROCESS_INVOLVED_IN_REPRODU...	GO:0003006	11	-0.354516	-1.015562	0.431884	0.695824	1.000
358	GOBP_REGULATION_OF_RESPONSE_TO_STRESS	GO:0080134	19	-0.305656	-1.016273	0.432815	0.696386	1.000
359	GOBP_MALE_GAMETE_GENERATION	GO:0048232	5	-0.457950	-1.016966	0.431008	0.697009	1.000
360	GOBP_CYTOKINE_PRODUCTION	GO:0001816	10	-0.366651	-1.017102	0.440184	0.698654	1.000
361	GOBP_ORGANOPHOSPHATE_METABOLIC_PROCESS	GO:0019637	7	-0.410218	-1.019518	0.443932	0.695785	1.000
362	GOBP_REGULATION_OF_MUSCLE_SYSTEM_PROCESS	GO:0090257	8	-0.390017	-1.021426	0.436252	0.693852	1.000
363	GOBP_PRI_MIRNA_TRANSCRIPTION_BY_RNA_POLYMERASE_II	GO:0061614	7	-0.409414	-1.023804	0.435858	0.691031	1.000
364	GOBP_MACROMOLECULE_CATABOLIC_PROCESS	GO:0009057	12	-0.345243	-1.024631	0.421439	0.691285	1.000
365	GOBP_POSITIVE_REGULATION_OF_SMOOTH_MUSCLE_CELL...	GO:0048661	5	-0.468740	-1.024964	0.451664	0.692561	1.000
366	GOBP_PEPTIDE_METABOLIC_PROCESS	GO:0006518	12	-0.346609	-1.025600	0.433333	0.693190	1.000
367	GOBP_LIPID_BIOSYNTHETIC_PROCESS	GO:0008610	6	-0.451636	-1.026332	0.454839	0.693733	1.000
368	GOBP_REGULATION_OF_APOPTOTIC_SIGNALING_PATHWAY	GO:2001233	10	-0.373453	-1.030180	0.437117	0.687802	1.000
369	GOBP_NEGATIVE_REGULATION_OF_MOLECULAR_FUNCTION	GO:0044092	24	-0.293879	-1.036402	0.414734	0.677503	1.000
370	REACTOME_POST_TRANSLATIONAL_PROTEIN_MODIFICATION	R-RNO-597592	7	-0.424254	-1.038582	0.413961	0.675090	1.000
371	GOBP_CHROMOSOME_ORGANIZATION	GO:0051276	6	-0.455757	-1.040464	0.432000	0.673184	1.000
372	GOBP_CELL_CYCLE	GO:0007049	21	-0.298989	-1.041823	0.408392	0.672266	1.000
373	GOBP_CHROMATIN_ORGANIZATION	GO:0006325	5	-0.477464	-1.042114	0.429892	0.673624	1.000
374	GOBP_BIOLOGICAL_PROCESS_INVOLVED_IN_SYMBIOTIC_...	GO:004440	11	-0.358102	-1.043259	0.389706	0.673307	1.000
375	REACTOME_EXTRA_NUCLEAR_ESTROGEN_SIGNALING	R-RNO-9009391	7	-0.412535	-1.043692	0.392638	0.674439	1.000
376	GOBP_REGULATION_OF_MAP_KINASE_ACTIVITY	GO:0043405	9	-0.396884	-1.049881	0.399390	0.663867	1.000
377	GOBP_NEGATIVE_REGULATION_OF_ANION_TRANSPORT	GO:1903792	8	-0.413731	-1.050368	0.425373	0.664755	1.000
378	GOBP_REGULATION_OF_LIPID_BIOSYNTHETIC_PROCESS	GO:0046890	6	-0.451636	-1.050932	0.413399	0.665590	1.000
379	GOBP_CELL_MIGRATION	GO:0016477	22	-0.299230	-1.053247	0.391365	0.662799	1.000
380	GOBP_POSITIVE_REGULATION_OF_PROTEIN_METABOLIC_...	GO:0051247	28	-0.290077	-1.053387	0.383064	0.664460	1.000
381	GOBP_ORGANIC_ACID_METABOLIC_PROCESS	GO:0006082	7	-0.423125	-1.054399	0.418429	0.664417	1.000
382	GOBP_REGIONALIZATION	GO:0003002	6	-0.457696	-1.061866	0.388802	0.651247	1.000
383	GOBP_MULTICELLULAR_ORGANISM_REPRODUCTION	GO:0032504	12	-0.354383	-1.061933	0.391176	0.653073	1.000
384	GOBP_MONOCARBOXYLIC_ACID_METABOLIC_PROCESS	GO:0032787	6	-0.467408	-1.065208	0.407407	0.648238	1.000

Continuation of Table **S19**

	NAME	ID	SIZE	ES	NES	NOM.p.val	FDR.q.val	FWER.p.val
385	GOBP_TEMPERATURE_HOMEOSTASIS	GO:0001659	5	-0.483173	-1.065225	0.393365	0.650154	1.000
386	GOBP_REGULATION_OF_CELLULAR_COMPONENT_SIZE	GO:0032535	7	-0.430674	-1.065392	0.382540	0.651744	1.000
387	GOBP_POSITIVE_REGULATION_OF_MOLECULAR_FUNCTION	GO:0044093	38	-0.281602	-1.066679	0.374339	0.651023	1.000
388	GOBP_MITOCHONDRION_ORGANIZATION	GO:0007005	7	-0.424789	-1.070054	0.379786	0.646119	1.000
389	GOBP_REGULATION_OF_LIPID_LOCALIZATION	GO:1905952	5	-0.487070	-1.070534	0.372287	0.647149	1.000
390	GOBP_INORGANIC_ION_TRANSMEMBRANE_TRANSPORT	GO:0098660	9	-0.398262	-1.071592	0.373874	0.646927	1.000
391	GOBP_MONONUCLEAR_CELL_DIFFERENTIATION	GO:1903131	7	-0.440983	-1.073382	0.381395	0.645323	1.000
392	GOBP_REGULATION_OF_PEPTIDE_SECRETION	GO:0002791	7	-0.435257	-1.074379	0.396605	0.645276	1.000
393	GOBP_BEHAVIOR	GO:0007610	32	-0.287017	-1.074908	0.363995	0.646104	1.000
394	GOBP_RESPONSE_TO_DRUG	GO:0042493	11	-0.382544	-1.075417	0.383459	0.646951	1.000
395	GOBP_PATTERN_SPECIFICATION_PROCESS	GO:0007389	6	-0.457696	-1.077303	0.367852	0.645178	1.000
396	HALLMARK_PI3K_AKT_MTOR_SIGNALING	M5923	6	-0.458100	-1.078250	0.367314	0.645297	1.000
397	GOBP_COAGULATION	GO:0050817	8	-0.412018	-1.078993	0.355039	0.645687	1.000
398	GOBP_POSITIVE_REGULATION_OF_CELL_GROWTH	GO:0030307	6	-0.457609	-1.081262	0.371560	0.642900	1.000
399	GOBP_APOPTOTIC_MITOCHONDRIAL_CHANGES	GO:0008637	6	-0.457707	-1.083253	0.373089	0.640820	1.000
400	GOBP_PEPTIDYL_SERINE_MODIFICATION	GO:0018209	12	-0.369179	-1.083884	0.359352	0.641730	1.000
401	GOBP_PURINE_CONTAINING_COMPOUND_METABOLIC_PROCESS	GO:0072521	5	-0.504696	-1.087283	0.379147	0.637014	1.000
402	GOBP_NEGATIVE_REGULATION_OF_CELL_CYCLE	GO:0045786	5	-0.489833	-1.088109	0.352025	0.637399	1.000
403	GOBP_REGULATION_OF_PEPTIDE_HORMONE_SECRETION	GO:0090276	6	-0.462238	-1.090499	0.348800	0.634457	1.000
404	GOBP_REGULATION_OF_PROTEIN_BINDING	GO:0043393	7	-0.446570	-1.091029	0.359014	0.635339	1.000
405	GOBP_RESPONSE_TO_OXYGEN_LEVELS	GO:0070482	8	-0.417329	-1.093067	0.361801	0.633135	1.000
406	GOBP_POSITIVE_REGULATION_OF_MAPK_CASCADE	GO:0043410	9	-0.401785	-1.097210	0.355725	0.626592	1.000
407	GOBP_CYTOKINE_MEDIATED_SIGNALING_PATHWAY	GO:0019221	16	-0.344914	-1.103342	0.350852	0.615894	1.000
408	GOBP_ORGANIC_ACID_TRANSPORT	GO:0015849	7	-0.442124	-1.104464	0.344186	0.615520	1.000
409	GOBP_REGULATION_OF_CELL_PROJECTION_ORGANIZATION	GO:0031344	20	-0.320244	-1.104696	0.345481	0.616962	1.000
410	GOBP_REGULATION_OF_CELLULAR_COMPONENT_MOVEMENT	GO:0051270	15	-0.355715	-1.106169	0.330893	0.615865	1.000
411	GOBP_REGULATION_OF_PHOSPHORUS_METABOLIC_PROCESS	GO:0051174	27	-0.304770	-1.106267	0.318783	0.617643	1.000
412	GOBP_NUCLEOBASE_CONTAINING_SMALL_MOLECULE_META...	GO:0055086	6	-0.469581	-1.111326	0.310680	0.609554	1.000
413	GOBP_ADENYLATE_CYCLASE_MODULATING_G_PROTEIN_CO...	GO:0007188	11	-0.392753	-1.113992	0.321888	0.606260	1.000
414	GOBP_POSITIVE_REGULATION_OF_PROTEIN_KINASE_ACT...	GO:0045860	19	-0.334578	-1.115983	0.330544	0.604244	1.000
415	GOBP_REGULATION_OF_EPITHELIAL_CELL_MIGRATION	GO:0010632	6	-0.474950	-1.116804	0.328051	0.604596	1.000
416	GOBP_OSTEOBLAST_DIFFERENTIATION	GO:0001649	5	-0.505072	-1.119887	0.325434	0.600151	1.000
417	GOBP_MEMORY	GO:0007613	13	-0.366294	-1.121579	0.318314	0.598368	1.000
418	GOBP_AMYLOID_PRECURSOR_PROTEIN_METABOLIC_PROCESS	GO:0042982	5	-0.513831	-1.122059	0.323102	0.599330	1.000
419	GOBP_ENDOCRINE_SYSTEM_DEVELOPMENT	GO:0035270	6	-0.477092	-1.123713	0.304085	0.598122	1.000
420	GOBP_CELLULAR_COMPONENT_DISASSEMBLY	GO:0022411	6	-0.492575	-1.123913	0.328125	0.599714	1.000
421	GOBP_COGNITION	GO:0050890	28	-0.306297	-1.124522	0.297151	0.600522	1.000
422	GOBP_LIPID_METABOLIC_PROCESS	GO:0006629	10	-0.401989	-1.126219	0.302766	0.599110	1.000
423	REACTOME_OPIOID_SIGNALLING	R-RNO-111885	12	-0.384285	-1.128213	0.302147	0.597167	1.000
424	GOBP_REGULATION_OF_SECRETION	GO:0051046	14	-0.369306	-1.130722	0.306452	0.593834	1.000
425	GOBP_REGULATION_OF_PROTEIN_PHOSPHORYLATION	GO:0001932	26	-0.316306	-1.131548	0.319559	0.594035	1.000
426	GOBP_AMYLOID_PRECURSOR_PROTEIN_CATABOLIC_PROCESS	GO:0042987	5	-0.513831	-1.131756	0.312893	0.595621	1.000
427	GOBP_HEAD_DEVELOPMENT	GO:0060322	22	-0.325822	-1.132129	0.294444	0.596817	1.000

Continuation of Table **S19**

	NAME	ID	SIZE	ES	NES	NOM.p.val	FDR.q.val	FWER.p.val
428	GOBP_RESPONSE_TO_KETONE	GO:1901654	7	-0.460814	-1.132589	0.321212	0.597937	1.000
429	GOBP_RESPONSE_TO_OXYGEN_CONTAINING_COMPOUND	GO:1901700	39	-0.292916	-1.133877	0.307798	0.597428	1.000
430	GOBP_REGULATION_OF_CELLULAR_LOCALIZATION	GO:0060341	17	-0.356823	-1.134481	0.296719	0.598225	1.000
431	GOBP_TELENCEPHALON_DEVELOPMENT	GO:0021537	6	-0.489302	-1.136469	0.306154	0.596305	1.000
432	GOBP_REGULATION_OF_PROTEIN_LOCALIZATION	GO:0032880	18	-0.342539	-1.136729	0.299572	0.597847	1.000
433	GOBP_POSITIVE_REGULATION_OF_ESTABLISHMENT_OF_P...	GO:1904951	5	-0.531465	-1.136807	0.292994	0.599756	1.000
434	GOBP_REGULATION_OF_CATION_TRANSMEMBRANE_TRANSPORT	GO:1904062	16	-0.350513	-1.136874	0.300429	0.601767	1.000
435	GOBP_POSITIVE_REGULATION_OF_SECRETION	GO:0051047	6	-0.483004	-1.137199	0.307573	0.603158	1.000
436	GOBP_RESPONSE_TO_BACTERIUM	GO:0009617	13	-0.376744	-1.139488	0.303892	0.600476	1.000
437	GOBP_LIPID_LOCALIZATION	GO:0010876	10	-0.413902	-1.139589	0.291982	0.602350	1.000
438	GOBP_SKIN_DEVELOPMENT	GO:0043588	5	-0.520756	-1.139811	0.300161	0.604112	1.000
439	GOBP_CALCIUM_MEDIATED_SIGNALING	GO:0019722	10	-0.410246	-1.140862	0.298682	0.603952	1.000
440	REACTOME_TRAFFICKING_OF_AMPA_RECEPTORS	R-RNO-399719	11	-0.398962	-1.140884	0.318043	0.606067	1.000
441	GOBP_RESPONSE_TO_TUMOR_NECROSIS_FACTOR	GO:0034612	7	-0.464982	-1.141812	0.323752	0.606015	1.000
442	GOBP_RESPONSE_TO_MOLECULE_OF_BACTERIAL_ORIGIN	GO:0002237	13	-0.376744	-1.142116	0.278393	0.607536	1.000
443	GOBP_HEART_DEVELOPMENT	GO:0007507	6	-0.483281	-1.142589	0.305556	0.608743	1.000
444	GOBP_ACTIVATION_OF_MAPK_ACTIVITY	GO:0000187	6	-0.491622	-1.143003	0.297420	0.610011	1.000
445	GOBP_CARDIAC_MUSCLE_TISSUE_DEVELOPMENT	GO:0048738	6	-0.483281	-1.144317	0.292259	0.609397	1.000
446	GOBP_AMYLOID_BETA_METABOLIC_PROCESS	GO:0050435	5	-0.513831	-1.146426	0.301092	0.607154	1.000
447	GOBP_CYTOSKELETON_ORGANIZATION	GO:0007010	11	-0.398312	-1.147816	0.280802	0.606504	1.000
448	GOBP_POSITIVE_REGULATION_OF_PROTEOLYSIS	GO:0045862	8	-0.441417	-1.156338	0.284639	0.590180	1.000
449	GOBP_FATTY_ACID_TRANSPORT	GO:0015908	6	-0.488282	-1.156757	0.282012	0.591446	1.000
450	GOBP_NEGATIVE_REGULATION_OF_BINDING	GO:0051100	6	-0.500753	-1.158003	0.302652	0.590846	1.000
451	GOBP_RESPONSE_TO_HORMONE	GO:0009725	20	-0.349848	-1.160979	0.276243	0.586734	1.000
452	GOBP_REGULATION_OF_CARBOHYDRATE_METABOLIC_PROCESS	GO:0006109	5	-0.538005	-1.161470	0.269663	0.587870	1.000
453	GOBP_REGULATION_OF_SYSTEM_PROCESS	GO:0044057	18	-0.349048	-1.161691	0.270156	0.589577	1.000
454	GOBP_POSITIVE_REGULATION_OF_LONG_TERM_SYNAPTIC...	GO:1900273	5	-0.528088	-1.162740	0.265244	0.589441	1.000
455	GOBP_POSITIVE_REGULATION_OF_TRANSFERASE_ACTIVITY	GO:0051347	20	-0.345124	-1.162971	0.277008	0.591233	1.000
456	GOBP_REGULATION_OF_ANION_TRANSMEMBRANE_TRANSPORT	GO:1903959	5	-0.543331	-1.166724	0.273322	0.585584	1.000
457	GOBP_REGULATION_OF_MAPK_CASCADE	GO:0043408	13	-0.383372	-1.167423	0.272597	0.586178	1.000
458	GOBP_REGULATION_OF_IMMUNE_SYSTEM_PROCESS	GO:0002682	21	-0.337135	-1.167719	0.262319	0.587701	1.000
459	GOBP_LEUKOCYTE_MIGRATION	GO:0050900	6	-0.512104	-1.169182	0.278317	0.586481	1.000
460	GOBP_NEUROINFLAMMATORY_RESPONSE	GO:0150076	5	-0.541722	-1.169645	0.270313	0.587819	1.000
461	GOBP_POSITIVE_REGULATION_OF_MAP_KINASE_ACTIVITY	GO:0043406	6	-0.491622	-1.169869	0.267188	0.589556	1.000
462	GOBP_POSTTRANSCRIPTIONAL_REGULATION_OF_GENE_EX...	GO:0010608	8	-0.453214	-1.172598	0.272152	0.585820	1.000
463	GOBP_CENTRAL_NERVOUS_SYSTEM_DEVELOPMENT	GO:0007417	26	-0.327970	-1.172702	0.271093	0.587926	1.000
464	GOBP_SEXUAL_REPRODUCTION	GO:0019953	10	-0.418138	-1.172721	0.264012	0.590185	1.000
465	GOBP_POSITIVE_REGULATION_OF_PROTEIN_SERINE_THR...	GO:0071902	9	-0.434921	-1.177397	0.256966	0.581964	1.000
466	GOBP_CARBOHYDRATE_BIOSYNTHETIC_PROCESS	GO:0016051	5	-0.538005	-1.178939	0.276295	0.580951	1.000
467	GOBP_MITOTIC_CELL_CYCLE	GO:0000278	10	-0.418891	-1.181548	0.246246	0.577580	1.000
468	GOBP_POSITIVE_REGULATION_OF_APOPTOTIC_SIGNALIN...	GO:2001235	5	-0.538350	-1.184676	0.242378	0.573012	1.000
469	GOBP_CELLULAR_RESPONSE_TO_NITROGEN_COMPOUND	GO:1901699	20	-0.344172	-1.184750	0.259722	0.575144	1.000
470	GOBP_PHOSPHATIDYLINOSITOL_3_KINASE_SIGNALING	GO:0014065	7	-0.488598	-1.193283	0.243750	0.558675	1.000

Continuation of Table **S19**

	NAME	ID	SIZE	ES	NES	NOM.p.val	FDR.q.val	FWER.p.val
471	GOBP_T_CELL_ACTIVATION	GO:0042110	8	-0.455090	-1.195040	0.255887	0.557188	1.000
472	GOBP_REGULATION_OF_CARBOHYDRATE_BIOSYNTHETIC_P...	GO:0043255	5	-0.538005	-1.199603	0.233385	0.549619	1.000
473	GOBP_IN_UTERO_EMBRYONIC_DEVELOPMENT	GO:0001701	8	-0.459415	-1.199870	0.229358	0.551284	1.000
474	GOBP_MAINTENANCE_OF_LOCATION	GO:0051235	7	-0.489072	-1.203225	0.241379	0.546513	1.000
475	GOBP_EPHRIN_RECEPTOR_SIGNALING_PATHWAY	GO:0048013	5	-0.546727	-1.204412	0.234921	0.546165	1.000
476	GOBP_REGULATION_OF_ORGAN_GROWTH	GO:0046620	5	-0.546824	-1.204461	0.234528	0.548326	1.000
477	GOBP_ORGANIC_HYDROXY_COMPOUND_TRANSPORT	GO:0015850	6	-0.508624	-1.204714	0.237421	0.550031	1.000
478	GOBP_REGULATION_OF_CELL_CYCLE	GO:0051726	13	-0.391102	-1.204743	0.240469	0.552240	1.000
479	GOBP_CARBOHYDRATE_HOMEOSTASIS	GO:0033500	7	-0.499021	-1.204775	0.236392	0.554472	1.000
480	GOBP_EPITHELIAL_CELL_PROLIFERATION	GO:0050673	12	-0.410303	-1.204933	0.249645	0.556434	1.000
481	REACTOME_ANTI_INFLAMMATORY_RESPONSE_FAVOURING_...	R-HSA-9662851	5	-0.545676	-1.205220	0.226006	0.557963	1.000
482	GOBP_ORGAN_GROWTH	GO:0035265	5	-0.546824	-1.206975	0.232372	0.556468	1.000
483	GOBP_PROTEIN_COMPLEX_OLIGOMERIZATION	GO:0051259	6	-0.512195	-1.207555	0.233282	0.557508	1.000
484	GOBP_NEURON_PROJECTION_ORGANIZATION	GO:0106027	8	-0.474623	-1.209028	0.234472	0.556660	1.000
485	GOBP_REGULATION_OF_CATION_CHANNEL_ACTIVITY	GO:2001257	14	-0.393446	-1.211686	0.236575	0.552824	1.000
486	GOBP_ANATOMICAL_STRUCTURE_FORMATION_INVOLVED_I...	GO:0048646	20	-0.361323	-1.212468	0.234890	0.553507	1.000
487	GOBP_G_PROTEIN_COUPLED_RECEPTOR_SIGNALING_PATHWAY	GO:0007186	18	-0.370194	-1.212754	0.222069	0.555236	1.000
488	GOBP_INNATE_IMMUNE_RESPONSE	GO:0045087	7	-0.492671	-1.213076	0.223054	0.556841	1.000
489	GOBP_CELLULAR_RESPONSE_TO_DNA_DAMAGE_STIMULUS	GO:0006974	5	-0.549407	-1.213623	0.217391	0.557993	1.000
490	GOBP_EMBRYONIC_MORPHOGENESIS	GO:0048598	6	-0.515498	-1.213743	0.224490	0.560115	1.000
491	GOBP_HORMONE_TRANSPORT	GO:0009914	9	-0.453956	-1.214581	0.220521	0.560711	1.000
492	REACTOME_LEISHMANIA_INFECTION	R-HSA-9658195	10	-0.437418	-1.217557	0.218182	0.556523	1.000
493	GOBP_RESPONSE_TO_NICOTINE	GO:0035094	7	-0.492001	-1.217577	0.223765	0.558949	1.000
494	GOBP_REGULATION_OF_TRANSPORT	GO:0051049	44	-0.312415	-1.218465	0.203974	0.559528	1.000
495	GOBP_POSITIVE_REGULATION_OF_CELL_DEATH	GO:0010942	19	-0.368148	-1.219931	0.213353	0.558773	1.000
496	GOBP_REGULATION_OF_POSTSYNAPTIC_MEMBRANE_POTEN...	GO:0060078	14	-0.398170	-1.220125	0.248521	0.560843	1.000
497	GOBP_RESPONSE_TO_ABIOTIC_STIMULUS	GO:0009628	27	-0.332079	-1.221049	0.216552	0.561226	1.000
498	GOBP_REGULATION_OF_CELLULAR_COMPONENT_BIOGENESIS	GO:0044087	10	-0.436790	-1.221276	0.236232	0.563217	1.000
499	GOBP_NEGATIVE_REGULATION_OF_SYNAPTIC_TRANSMISSION	GO:0050805	6	-0.527455	-1.221517	0.216906	0.565200	1.000
500	GOBP_GAMETE_GENERATION	GO:0007276	9	-0.458359	-1.222527	0.239819	0.565570	1.000
501	GOBP_LYMPHOCYTE_ACTIVATION	GO:0046649	9	-0.452148	-1.222930	0.224961	0.567174	1.000
502	REACTOME_INTERFERON_SIGNALING	R-RNO-913531	6	-0.513568	-1.225899	0.199690	0.562677	1.000
503	GOBP_POSITIVE_REGULATION_OF_CYTOKINE_PRODUCTION	GO:0001819	7	-0.490150	-1.226258	0.229904	0.564623	1.000
504	REACTOME_L1CAM_INTERACTIONS	R-RNO-373760	5	-0.569629	-1.226452	0.185304	0.566697	1.000
505	GOBP_REGULATION_OF_FAT_CELL_DIFFERENTIATION	GO:0045598	6	-0.519832	-1.226634	0.215071	0.568850	1.000
506	REACTOME_MEMBRANE_TRAFFICKING	R-RNO-199991	5	-0.567644	-1.231422	0.199336	0.559949	1.000
507	REACTOME_VESICLE_MEDIATED_TRANSPORT	R-RNO-5653656	5	-0.567644	-1.234194	0.207120	0.556313	1.000
508	GOBP_RESPONSE_TO_NUTRIENT	GO:0007584	5	-0.566265	-1.236501	0.206557	0.553570	1.000
509	GOBP_EXTRINSIC_APOPTOTIC_SIGNALING_PATHWAY	GO:0097191	7	-0.499437	-1.237725	0.218009	0.553337	1.000
510	GOBP_POSITIVE_REGULATION_OF_DEFENSE_RESPONSE	GO:0031349	5	-0.566265	-1.240009	0.205329	0.550652	1.000
511	GOBP_POSITIVE_REGULATION_OF_CATALYTIC_ACTIVITY	GO:0043085	30	-0.332754	-1.241417	0.190728	0.550066	1.000
512	GOBP_NEGATIVE_REGULATION_OF_CATABOLIC_PROCESS	GO:0009895	8	-0.474679	-1.243397	0.213961	0.548192	1.000
513	REACTOME_DEVELOPMENTAL_BIOLOGY	R-RNO-1266738	23	-0.351760	-1.243875	0.191781	0.549716	1.000

Continuation of Table **S19**

	NAME	ID	SIZE	ES	NES	NOM.p.val	FDR.q.val	FWER.p.val
514	GOBP_REGULATION_OF_VASCULATURE_DEVELOPMENT	GO:1901342	5	-0.570549	-1.246922	0.196694	0.545302	1.000
515	GOBP_ASSOCIATIVE_LEARNING	GO:0008306	7	-0.509752	-1.249283	0.197869	0.542141	1.000
516	GOBP_RESPONSE_TO_CYTOKINE	GO:0034097	23	-0.361301	-1.249424	0.188105	0.544495	1.000
517	GOBP_VASCULATURE_DEVELOPMENT	GO:0001944	17	-0.382477	-1.250226	0.174785	0.545221	1.000
518	GOBP_ANIMAL_ORGAN_MORPHOGENESIS	GO:0009887	12	-0.419316	-1.256274	0.210604	0.533583	1.000
519	REACTOME_SIGNALING_BY_GPCR	R-RNO-372790	22	-0.365841	-1.256903	0.206327	0.534907	1.000
520	GOBP_PHAGOCYTOSIS	GO:0006909	5	-0.572634	-1.258110	0.179650	0.534639	1.000
521	REACTOME_G_ALPHA_I_SIGNALLING_EVENTS	R-RNO-418594	18	-0.384200	-1.260236	0.175714	0.532272	1.000
522	GOBP_PROTEIN_AUTOPHOSPHORYLATION	GO:0046777	5	-0.569624	-1.260647	0.176287	0.534069	1.000
523	GOBP_POSITIVE_REGULATION_OF_CELL_ADHESION	GO:0045785	7	-0.504424	-1.261099	0.204082	0.535748	1.000
524	GOBP_ERK1_AND_ERK2_CASCADE	GO:0070371	5	-0.571016	-1.263284	0.189189	0.533315	1.000
525	GOBP_SENSORY_PERCEPTION	GO:0007600	9	-0.474957	-1.264574	0.183486	0.533108	1.000
526	REACTOME_INTRACELLULAR_SIGNALING_BY_SECOND_MES...	R-RNO-9006925	15	-0.396819	-1.264710	0.184319	0.535607	1.000
527	GOBP_RESPONSE_TO_ORGANIC_CYCLIC_COMPOUND	GO:0014070	20	-0.377711	-1.264952	0.178218	0.537722	1.000
528	GOBP_INFLAMMATORY_RESPONSE	GO:0006954	12	-0.427786	-1.270050	0.186047	0.528770	1.000
529	REACTOME_G_PROTEIN_MEDIATED_EVENTS	R-RNO-112040	9	-0.470248	-1.272588	0.188707	0.525519	1.000
530	GOBP_CIRCULATORY_SYSTEM_DEVELOPMENT	GO:0072359	21	-0.373626	-1.279667	0.189373	0.511963	1.000
531	REACTOME_TRANSCRIPTIONAL_REGULATION_BY_MECP2	R-RNO-8986944	7	-0.523311	-1.281195	0.161481	0.511060	1.000
532	GOBP_POSITIVE_REGULATION_OF_CELLULAR_COMPONENT...	GO:0044089	9	-0.483154	-1.281937	0.167656	0.512010	1.000
533	GOBP_LONG_TERM_SYNAPTIC_DEPRESSION	GO:0060292	5	-0.588178	-1.282561	0.161695	0.513440	1.000
534	GOBP_CIRCULATORY_SYSTEM_PROCESS	GO:0003013	5	-0.584403	-1.283104	0.154560	0.514891	1.000
535	GOBP_BLOOD_VESSEL_MORPHOGENESIS	GO:0048514	14	-0.419025	-1.284355	0.168103	0.514604	1.000
536	GOBP_CELLULAR_RESPONSE_TO_KETONE	GO:1901655	5	-0.593023	-1.288191	0.164110	0.508849	1.000
537	GOBP_CELLULAR_RESPONSE_TO_MOLECULE_OF_BACTERIA...	GO:0071219	8	-0.501819	-1.289122	0.165615	0.509438	1.000
538	GOBP_REGULATION_OF_CELLULAR_PROTEIN_LOCALIZATION	GO:1903827	12	-0.433045	-1.293586	0.150972	0.501685	1.000
539	GOBP_PEPTIDE_SECRETION	GO:0002790	9	-0.481040	-1.295741	0.186364	0.499634	1.000
540	GOBP_SECRETION	GO:0046903	24	-0.365882	-1.299166	0.157025	0.494901	1.000
541	GOBP_EMBRYO_DEVELOPMENT	GO:0009790	14	-0.421062	-1.301056	0.164575	0.493515	1.000
542	GOBP_CELLULAR_RESPONSE_TO_BIOTIC_STIMULUS	GO:0071216	8	-0.501819	-1.301787	0.162003	0.494651	1.000
543	GOBP_MONOAMINE_TRANSPORT	GO:0015844	5	-0.598248	-1.307422	0.144026	0.484616	1.000
544	GOBP_REGULATION_OF_TRANSPORTER_ACTIVITY	GO:0032409	17	-0.393676	-1.309685	0.166667	0.482440	1.000
545	GOBP_CELLULAR_RESPONSE_TO_HORMONE_STIMULUS	GO:0032870	11	-0.454816	-1.310839	0.146747	0.482734	1.000
546	GOBP_DENDRITIC_SPINE_MORPHOGENESIS	GO:0060997	5	-0.599045	-1.311494	0.159869	0.484130	1.000
547	GOBP_POSITIVE_REGULATION_OF_IMMUNE_RESPONSE	GO:0050778	9	-0.487429	-1.311855	0.148710	0.486172	1.000
548	GOBP_REGULATION_OF_CATABOLIC_PROCESS	GO:0009894	13	-0.432216	-1.313775	0.163324	0.484715	1.000
549	GOBP_REGULATION_OF_PROTEIN_KINASE_ACTIVITY	GO:0045859	22	-0.380570	-1.319553	0.143875	0.474411	1.000
550	GOBP_MEMBRANE_ORGANIZATION	GO:0061024	12	-0.449024	-1.321334	0.157083	0.473365	1.000
551	GOBP_REGULATION_OF_TRANSFERASE_ACTIVITY	GO:0051338	22	-0.380570	-1.322856	0.142857	0.472733	1.000
552	GOBP_INSULIN_SECRETION	GO:0030073	7	-0.540570	-1.323464	0.158400	0.474060	1.000
553	GOBP_REGULATION_OF_MEMBRANE_POTENTIAL	GO:0042391	17	-0.401550	-1.323477	0.149931	0.476888	1.000
554	GOBP_REGULATION_OF_LONG_TERM_SYNAPTIC_POTENTIA...	GO:1900271	6	-0.558430	-1.326098	0.147152	0.473977	1.000
555	GOBP_CELLULAR_RESPONSE_TO_PEPTIDE_HORMONE_STIM...	GO:0071375	9	-0.494963	-1.326687	0.149701	0.475469	1.000
556	GOBP_REGULATION_OF_BODY_FLUID_LEVELS	GO:0050878	11	-0.460312	-1.326765	0.130631	0.478170	1.000

Continuation of Table **S19**

	NAME	ID	SIZE	ES	NES	NOM.p.val	FDR.q.val	FWER.p.val
557	GOBP_EMBRYONIC_ORGAN_DEVELOPMENT	GO:0048568	7	-0.533119	-1.328820	0.135671	0.476362	1.000
558	GOBP_RESPONSE_TO_ACID_CHEMICAL	GO:0001101	6	-0.560792	-1.329695	0.142420	0.477103	1.000
559	REACTOME_DISEASES_OF_SIGNAL_TRANSDUCTION_BY_GR...	R-HSA-5663202	12	-0.450596	-1.331092	0.133523	0.476550	1.000
560	GOBP_CATION_TRANSMEMBRANE_TRANSPORT	GO:0098655	18	-0.401962	-1.332976	0.146067	0.475067	1.000
561	GOBP_GLUTAMATE_RECEPTOR_SIGNALING_PATHWAY	GO:0007215	17	-0.409920	-1.335294	0.136691	0.472916	1.000
562	GOBP_REGULATION_OF_LYMPHOCYTE_ACTIVATION	GO:0051249	5	-0.609746	-1.337698	0.115445	0.470481	1.000
563	GOBP_PEPTIDE_BIOSYNTHETIC_PROCESS	GO:0043043	7	-0.550471	-1.338581	0.114062	0.471559	1.000
564	GOBP_PROTEIN_KINASE_B_SIGNALING	GO:0043491	6	-0.571452	-1.340615	0.117371	0.469880	1.000
565	GOBP_NERVOUS_SYSTEM_PROCESS	GO:0050877	38	-0.353650	-1.343867	0.131649	0.465254	1.000
566	GOBP_REGULATION_OF_NEUROTRANSMITTER_TRANSPORT	GO:0051588	6	-0.571740	-1.345454	0.130631	0.464630	1.000
567	GOBP_REGULATION_OF_ION_TRANSPORT	GO:0043269	36	-0.355551	-1.345570	0.128415	0.467416	1.000
568	REACTOME_INFECTIOUS_DISEASE	R-HSA-5663205	13	-0.449653	-1.346975	0.147710	0.467423	1.000
569	REACTOME_MAPK_FAMILY_SIGNALING_CASCADES	R-RNO-5683057	12	-0.459249	-1.347573	0.135258	0.469139	1.000
570	GOBP_PEPTIDE_HORMONE_SECRETION	GO:0030072	7	-0.540570	-1.349861	0.125758	0.467347	1.000
571	GOBP_MATERNAL_PROCESS_INVOLVED_IN_FEMALE_PREGN...	GO:0060135	5	-0.616936	-1.350344	0.115016	0.469452	1.000
572	GOBP_LEARNING	GO:0007612	13	-0.446031	-1.351147	0.119708	0.470804	1.000
573	GOBP_REGULATION_OF_DNA_BINDING_TRANSCRIPTION_F...	GO:0051090	16	-0.422064	-1.353097	0.131653	0.469540	1.000
574	GOBP_TUBE_DEVELOPMENT	GO:0035295	18	-0.404631	-1.353511	0.120433	0.471772	1.000
575	GOBP_REGULATION_OF_CELLULAR_AMIDE_METABOLIC_PR...	GO:0034248	9	-0.504229	-1.353689	0.120611	0.474597	1.000
576	GOBP_METAL_ION_TRANSPORT	GO:0030001	11	-0.474680	-1.354806	0.123907	0.475138	1.000
577	GOBP_POSITIVE_REGULATION_OF_IMMUNE_SYSTEM_PROCESS	GO:0002684	18	-0.412335	-1.355853	0.131728	0.476100	1.000
578	GOBP_HOMEOSTASIS_OF_NUMBER_OF_CELLS	GO:0048872	5	-0.600912	-1.356267	0.118859	0.478422	1.000
579	GOBP_MAPK_CASCADE	GO:0000165	18	-0.408262	-1.360107	0.130560	0.472406	1.000
580	GOBP_GLAND_DEVELOPMENT	GO:0048732	7	-0.545173	-1.364767	0.087500	0.465332	1.000
581	GOBP_REGULATION_OF_LIPID_METABOLIC_PROCESS	GO:0019216	7	-0.554776	-1.369639	0.111614	0.456912	1.000
582	GOBP_SENSORY_ORGAN_DEVELOPMENT	GO:0007423	8	-0.528165	-1.373030	0.126657	0.452446	1.000
583	GOBP_POSITIVE_REGULATION_OF_SMALL_MOLECULE_MET...	GO:0062013	5	-0.629364	-1.375097	0.098592	0.450887	1.000
584	GOBP_NEURON_MIGRATION	GO:0001764	5	-0.628224	-1.375136	0.109635	0.454174	1.000
585	GOBP_CALCIUM_ION_TRANSPORT	GO:0006816	11	-0.474680	-1.376588	0.121037	0.454185	1.000
586	REACTOME_EPH_EPHRIN_SIGNALING	R-RNO-2682334	6	-0.589306	-1.378982	0.111111	0.452091	1.000
587	GOBP_LEUKOCYTE_CELL_CELL_ADHESION	GO:0007159	6	-0.589032	-1.380513	0.093991	0.452025	1.000
588	GOBP_AMIDE_BIOSYNTHETIC_PROCESS	GO:0043604	7	-0.550471	-1.382719	0.109063	0.450463	1.000
589	GOBP_REGULATION_OF_CELL_ADHESION	GO:0030155	13	-0.461229	-1.382864	0.107143	0.453542	1.000
590	GOBP_ACTIN_FILAMENT_BASED_PROCESS	GO:0030029	7	-0.567215	-1.386808	0.104651	0.447770	1.000
591	GOBP_CARBOHYDRATE_DERIVATIVE_METABOLIC_PROCESS	GO:1901135	7	-0.544695	-1.387697	0.107765	0.449276	1.000
592	GOBP_ACTIVATION_OF_PROTEIN_KINASE_ACTIVITY	GO:0032147	11	-0.477842	-1.387876	0.110795	0.452460	1.000
593	GOBP_CELL_SURFACE_RECEPTOR_SIGNALING_PATHWAY_I...	GO:1905114	15	-0.448187	-1.388299	0.104499	0.454937	1.000
594	GOBP_RESPONSE_TO_LIPID	GO:0033993	19	-0.421497	-1.389190	0.110169	0.456226	1.000
595	GOBP_PLATELET_ACTIVATION	GO:0030168	6	-0.585410	-1.390205	0.100154	0.457457	1.000
596	GOBP_LOCOMOTORY_BEHAVIOR	GO:0007626	7	-0.573009	-1.391185	0.082803	0.458931	1.000
597	GOBP_DEFENSE_RESPONSE_TO_OTHER_ORGANISM	GO:0098542	9	-0.512433	-1.391623	0.089706	0.461525	1.000
598	GOBP_CELLULAR_LIPID_METABOLIC_PROCESS	GO:0044255	7	-0.552009	-1.392030	0.099071	0.464183	1.000
599	GOBP_CELL_ACTIVATION	GO:0001775	21	-0.402232	-1.394754	0.102703	0.461494	1.000

Continuation of Table **S19**

	NAME	ID	SIZE	ES	NES	NOM.p.val	FDR.q.val	FWER.p.val
600	REACTOME_DAG_AND_IP3_SIGNALING	R-RNO-1489509	8	-0.532998	-1.395594	0.091757	0.463483	1.000
601	REACTOME_ESTROGEN_DEPENDENT_NUCLEAR_EVENTS_DOW...	R-RNO-9634638	5	-0.644819	-1.398937	0.092800	0.458940	1.000
602	GOBP_REGULATION_OF_SYNAPSE_STRUCTURE_OR_ACTIVITY	GO:0050803	11	-0.493107	-1.399041	0.100149	0.462513	1.000
603	GOBP_REGULATION_OF_TRANSMEMBRANE_TRANSPORT	GO:0034762	23	-0.395083	-1.399124	0.108635	0.466299	1.000
604	GOBP_IMPORT_INTO_CELL	GO:0098657	6	-0.608864	-1.416063	0.083462	0.429288	1.000
605	GOBP_DEFENSE_RESPONSE	GO:0006952	19	-0.423416	-1.416133	0.093750	0.432970	1.000
606	GOBP_POSITIVE_REGULATION_OF_EPITHELIAL_CELL_PR...	GO:0050679	7	-0.578217	-1.417640	0.096367	0.433237	1.000
607	GOBP_POSTSYNAPSE_ORGANIZATION	GO:0099173	10	-0.497259	-1.420517	0.109774	0.429686	1.000
608	GOBP_REGULATION_OF_PROTEIN_SERINE_THREONINE_KI...	GO:0071900	12	-0.488818	-1.422382	0.105030	0.428992	1.000
609	GOBP_CELLULAR_RESPONSE_TO_ALCOHOL	GO:0097306	5	-0.651170	-1.425066	0.068643	0.426362	1.000
610	REACTOME_CA_DEPENDENT_EVENTS	R-RNO-111996	8	-0.547443	-1.428880	0.079193	0.421109	1.000
611	GOBP_REGULATION_OF_METAL_ION_TRANSPORT	GO:0010959	6	-0.614436	-1.432021	0.091054	0.417668	1.000
612	GOBP_CHEMICAL_SYNAPTIC_TRANSMISSION_POSTSYNAPTIC	GO:0099565	9	-0.528614	-1.432541	0.094453	0.420176	1.000
613	GOBP_TISSUE_HOMEOSTASIS	GO:0001894	5	-0.657539	-1.433148	0.064516	0.422534	1.000
614	GOBP_POSITIVE_REGULATION_OF_ENDOTHELIAL_CELL_P...	GO:0001938	6	-0.624595	-1.442753	0.072231	0.404969	1.000
615	GOBP_AMEBOIDAL_TYPE_CELL_MIGRATION	GO:0001667	11	-0.512086	-1.444559	0.083333	0.404701	1.000
616	GOBP_ORGANONITROGEN_COMPOUND_BIOSYNTHETIC_PROCESS	GO:1901566	13	-0.486044	-1.444572	0.071953	0.408585	1.000
617	GOBP_POSITIVE_REGULATION_OF_ION_TRANSPORT	GO:0043270	17	-0.446042	-1.451748	0.071625	0.396230	1.000
618	GOBP_TUBE_MORPHOGENESIS	GO:0035239	15	-0.469390	-1.454345	0.064378	0.394332	1.000
619	GOBP_EMBRYO_DEVELOPMENT_ENDING_IN_BIRTH_OR_EGG...	GO:0009792	9	-0.541175	-1.455118	0.071964	0.396393	1.000
620	GOBP_PROTEIN_LOCALIZATION_TO_PLASMA_MEMBRANE	GO:0072659	7	-0.588796	-1.457089	0.069291	0.395854	1.000
621	GOBP_SYNAPSE_ORGANIZATION	GO:0050808	17	-0.454425	-1.466549	0.067659	0.378477	1.000
622	GOBP_PROTEIN_PHOSPHORYLATION	GO:0006468	34	-0.386669	-1.467295	0.071332	0.380543	1.000
623	GOBP_PROTEIN_LOCALIZATION_TO_POSTSYNAPSE	GO:0062237	5	-0.672227	-1.470965	0.060897	0.376563	1.000
624	GOBP_POSITIVE_REGULATION_OF_ANION_TRANSPORT	GO:1903793	9	-0.553478	-1.477057	0.066566	0.367605	1.000
625	GOBP_REGULATION_OF_CELLULAR_CATABOLIC_PROCESS	GO:0031329	10	-0.537397	-1.479244	0.051593	0.366640	1.000
626	GOBP_RESPIRATORY_SYSTEM_DEVELOPMENT	GO:0060541	6	-0.626841	-1.482244	0.056782	0.364355	1.000
627	GOBP_CYTOSOLIC_CALCIUM_ION_TRANSPORT	GO:0060401	7	-0.609325	-1.482586	0.061947	0.367604	1.000
628	GOBP_PROTEIN_LOCALIZATION_TO_SYNAPSE	GO:0035418	5	-0.672227	-1.483414	0.047468	0.369518	1.000
629	GOBP_CELL_JUNCTION_ASSEMBLY	GO:0034329	8	-0.576809	-1.484034	0.066874	0.372058	1.000
630	REACTOME_ONCOGENIC_MAPK_SIGNALING	R-HSA-6802957	5	-0.676784	-1.489062	0.041401	0.364767	1.000
631	GOBP_ENDOTHELIAL_CELL_PROLIFERATION	GO:0001935	8	-0.578586	-1.490484	0.056662	0.365591	1.000
632	GOBP_DENDRITIC_SPINE_DEVELOPMENT	GO:0060996	6	-0.637936	-1.495950	0.049844	0.358155	1.000
633	GOBP_CALCIUM_ION_TRANSPORT_INTO_CYTOSOL	GO:0060402	7	-0.609325	-1.497642	0.059724	0.358664	1.000
634	GOBP_CELLULAR_HOMEOSTASIS	GO:0019725	18	-0.464489	-1.499157	0.055398	0.359548	1.000
635	GOBP_REGULATION_OF_ENDOTHELIAL_CELL_MIGRATION	GO:0010594	5	-0.687709	-1.506538	0.044481	0.347189	1.000
636	GOBP_RESPONSE_TO_ETHANOL	GO:0045471	5	-0.701938	-1.513302	0.032915	0.338450	1.000
637	GOBP_REGULATION_OF_CELL_ACTIVATION	GO:0050865	8	-0.587446	-1.514668	0.054773	0.339581	1.000
638	GOBP_NEGATIVE_REGULATION_OF_LIPID_METABOLIC_PR...	GO:0045833	5	-0.692033	-1.514924	0.036050	0.343250	1.000
639	GOBP_SPROUTING_ANGIOGENESIS	GO:0002040	5	-0.694896	-1.523025	0.042879	0.329679	1.000
640	GOBP_CATION_TRANSPORT	GO:0006812	25	-0.430202	-1.523611	0.045330	0.332702	1.000
641	REACTOME_NEUREXINS_AND_NEUROLIGINS	R-RNO-6794361	9	-0.565686	-1.524936	0.044709	0.334261	1.000
642	GOBP_POSITIVE_REGULATION_OF_DNA_BINDING_TRANSC...	GO:0051091	10	-0.543097	-1.527402	0.043091	0.333168	1.000

Continuation of Table **S19**

	NAME	ID	SIZE	ES	NES	NOM.p.val	FDR.q.val	FWER.p.val
643	GOBP_RESPONSE_TO_TEMPERATURE_STIMULUS	GO:0009266	9	-0.581502	-1.527997	0.026393	0.336107	1.000
644	GOBP_HOMEOSTATIC_PROCESS	GO:0042592	31	-0.416435	-1.528865	0.038822	0.338393	1.000
645	GOBP_EPITHELIUM_DEVELOPMENT	GO:0060429	15	-0.483532	-1.533457	0.053672	0.333036	1.000
646	GOBP_MONOSACCHARIDE_METABOLIC_PROCESS	GO:0005996	6	-0.648945	-1.535324	0.046584	0.333685	1.000
647	GOBP_POSITIVE_REGULATION_OF_LEUKOCYTE_CELL_CEL...	GO:1903039	5	-0.704221	-1.536566	0.038400	0.335698	1.000
648	GOBP_REGULATION_OF_PROTEIN_LOCALIZATION_TO_MEM...	GO:1905475	7	-0.614175	-1.536597	0.039002	0.340377	1.000
649	GOBP_REGULATION_OF_MITOTIC_CELL_CYCLE	GO:0007346	5	-0.702046	-1.537972	0.036107	0.342601	1.000
650	GOBP_REGULATION_OF_TRANS_SYNAPTIC_SIGNALING	GO:0099177	40	-0.399298	-1.540052	0.034574	0.342755	1.000
651	GOBP_BIOLOGICAL_ADHESION	GO:0022610	19	-0.463767	-1.542025	0.042936	0.343676	1.000
652	REACTOME_PROTEIN_PROTEIN_INTERACTIONS_AT_SYNAPSES	R-RNO-6794362	12	-0.533407	-1.543097	0.046899	0.346338	1.000
653	GOBP_RESPONSE_TO_BIOTIC_STIMULUS	GO:0009607	17	-0.481567	-1.545354	0.043228	0.346602	1.000
654	GOBP_POSITIVE_REGULATION_OF_SIGNALING	GO:0023056	34	-0.408622	-1.547248	0.035480	0.347697	1.000
655	GOBP_CALCIUM_ION_TRANSMEMBRANE_TRANSPORT	GO:0070588	7	-0.609325	-1.548876	0.036741	0.349279	1.000
656	GOBP_GLUCOSE_METABOLIC_PROCESS	GO:0006006	6	-0.648945	-1.551068	0.043614	0.349803	1.000
657	GOBP_REGULATION_OF_PROTEIN_LOCALIZATION_TO_CEL...	GO:1904375	6	-0.660698	-1.558760	0.035484	0.338178	1.000
658	GOBP_CHEMICAL_HOMEOSTASIS	GO:0048878	23	-0.440941	-1.566298	0.045961	0.327065	1.000
659	GOBP_POSITIVE_REGULATION_OF_CELL_CELL_ADHESION	GO:0022409	5	-0.704221	-1.569203	0.020833	0.326644	1.000
660	GOBP_CELL_CELL_JUNCTION_ASSEMBLY	GO:0007043	5	-0.731175	-1.574314	0.020701	0.321685	1.000
661	GOBP_CELLULAR_RESPONSE_TO_ORGANIC_CYCLIC_COMPOUND	GO:0071407	13	-0.520332	-1.584871	0.044978	0.304318	1.000
662	GOBP_SMALL_MOLECULE_METABOLIC_PROCESS	GO:0044281	15	-0.506576	-1.585161	0.031343	0.308917	1.000
663	GOBP_REGULATION_OF_NEUROTRANSMITTER_RECEPTOR_A...	GO:0099601	13	-0.535129	-1.585931	0.027818	0.313133	1.000
664	GOBP_REGULATION_OF_CELL_CELL_ADHESION	GO:0022407	9	-0.595497	-1.587014	0.032305	0.316746	1.000
665	GOBP_ION_HOMEOSTASIS	GO:0050801	16	-0.502610	-1.587068	0.023876	0.322501	1.000
666	GOBP_PROTEIN_LOCALIZATION_TO_CELL_JUNCTION	GO:1902414	6	-0.693762	-1.591490	0.014241	0.319366	1.000
667	GOBP_CELL_JUNCTION_ORGANIZATION	GO:0034330	19	-0.480441	-1.593919	0.031073	0.320600	1.000
668	GOBP_REGULATION_OF_NMDA_RECEPTOR_ACTIVITY	GO:2000310	10	-0.560693	-1.594472	0.025037	0.325689	1.000
669	GOBP_NEGATIVE_REGULATION_OF_LOCOMOTION	GO:0040013	5	-0.712905	-1.597060	0.030945	0.326241	1.000
670	GOBP_NEUROTRANSMITTER_TRANSPORT	GO:0006836	7	-0.650850	-1.607798	0.033846	0.307398	1.000
671	GOBP_LONG_TERM_MEMORY	GO:0007616	6	-0.701639	-1.610295	0.017134	0.307980	1.000
672	GOBP_TRANSMEMBRANE_TRANSPORT	GO:0055085	27	-0.449229	-1.610499	0.019894	0.313933	1.000
673	GOBP_PROTEIN_LOCALIZATION_TO_MEMBRANE	GO:0072657	14	-0.518001	-1.619715	0.032164	0.300818	1.000
674	GOBP_REGULATION_OF_EXTRINSIC_APOPTOTIC_SIGNALI...	GO:2001236	6	-0.686812	-1.622524	0.019449	0.300929	1.000
675	GOBP_CELL_CELL_JUNCTION_ORGANIZATION	GO:0045216	6	-0.679886	-1.622891	0.018349	0.306918	1.000
676	REACTOME_NEURONAL_SYSTEM	R-RNO-112316	26	-0.449025	-1.625147	0.025210	0.308451	1.000
677	GOBP_REGULATION_OF_CELLULAR_RESPONSE_TO_STRESS	GO:0080135	9	-0.607338	-1.626709	0.024818	0.312643	1.000
678	GOBP_NEUROTRANSMITTER_SECRETION	GO:0007269	6	-0.699857	-1.629409	0.021309	0.313798	1.000
679	GOBP_EXCITATORY_CHEMICAL_SYNAPTIC_TRANSMISSION	GO:0098976	5	-0.734940	-1.629668	0.014516	0.321232	1.000
680	GOBP_PROTEIN_CONTAINING_COMPLEX_SUBUNIT_ORGANI...	GO:0043933	14	-0.531547	-1.632389	0.021930	0.323768	1.000
681	GOBP_CELLULAR_RESPONSE_TO_LIPID	GO:0071396	15	-0.514470	-1.634339	0.024390	0.327604	1.000
682	GOBP_ANATOMICAL_STRUCTURE_HOMEOSTASIS	GO:0060249	7	-0.670165	-1.634582	0.013333	0.335888	1.000
683	GOBP_REGULATION_OF_NEUROTRANSMITTER_LEVELS	GO:0001505	7	-0.650850	-1.646717	0.017799	0.315420	1.000
684	GOBP_NEGATIVE_REGULATION_OF_IMMUNE_SYSTEM_PROCESS	GO:0002683	5	-0.747473	-1.654530	0.013932	0.305515	1.000
685	GOBP_MULTICELLULAR_ORGANISMAL_HOMEOSTASIS	GO:0048871	12	-0.572448	-1.669085	0.023529	0.281698	1.000

Continuation of Table **S19**

	NAME	ID	SIZE	ES	NES	NOM.p.val	FDR.q.val	FWER.p.val
686	GOBP_TISSUE_MIGRATION	GO:0090130	9	-0.620679	-1.675839	0.015576	0.276290	1.000
687	GOBP_SENSORY_PERCEPTION_OF_PAIN	GO:0019233	5	-0.762718	-1.684219	0.013997	0.267451	1.000
688	GOBP_REGULATION_OF_SMALL_MOLECULE_METABOLIC_PR...	GO:0062012	9	-0.627242	-1.685356	0.010370	0.273429	1.000
689	GOBP_SYNAPTIC_SIGNALING	GO:0099536	47	-0.427485	-1.689640	0.012870	0.272643	1.000
690	GOBP_SIGNAL_RELEASE	GO:0023061	14	-0.543418	-1.698342	0.012931	0.263249	1.000
691	GOBP_IONOTROPIC_GLUTAMATE_RECEPTOR_SIGNALING_P...	GO:0035235	9	-0.632911	-1.710785	0.017161	0.249509	0.998
692	GOBP_REGULATION_OF_SIGNALING_RECEPTOR_ACTIVITY	GO:0010469	14	-0.553057	-1.724086	0.011869	0.231932	0.997
693	REACTOME_TRANSMISSION_ACROSS_CHEMICAL_SYNAPSES	R-RNO-112315	23	-0.503234	-1.734796	0.005517	0.220369	0.996
694	REACTOME_CREB1_PHOSPHORYLATION_THROUGH_NMDA_RE...	R-RNO-442742	8	-0.668566	-1.735813	0.009119	0.226884	0.995
695	GOBP_METAL_ION_HOMEOSTASIS	GO:0055065	14	-0.561354	-1.742089	0.011445	0.223768	0.989
696	GOBP_REGULATION_OF_SYNAPTIC_PLASTICITY	GO:0048167	21	-0.515871	-1.746472	0.016484	0.224581	0.988
697	GOBP_CELL_CELL_SIGNALING	GO:0007267	56	-0.459364	-1.765695	0.003886	0.200745	0.983
698	GOBP_RESPONSE_TO_HEAT	GO:0009408	7	-0.721531	-1.772382	0.004651	0.199109	0.979
699	GOBP_LONG_TERM_SYNAPTIC_POTENTIATION	GO:0060291	12	-0.617680	-1.776981	0.005780	0.201518	0.976
700	GOBP_PROTEIN_LOCALIZATION_TO_CELL_PERIPHERY	GO:1990778	12	-0.607005	-1.784797	0.004451	0.197299	0.968
701	REACTOME_SYNAPTIC_ADHESION_LIKE_MOLECULES	R-RNO-8849932	9	-0.670886	-1.792105	0.006107	0.194969	0.961
702	GOBP_DIVALENT_INORGANIC_CATION_HOMEOSTASIS	GO:0072507	13	-0.607158	-1.815839	0.009777	0.167554	0.923
703	GOBP_CELLULAR_ION_HOMEOSTASIS	GO:0006873	14	-0.593443	-1.816342	0.011817	0.177216	0.921
704	REACTOME_NEUROTRANSMITTER_RECEPTORS_AND_POSTSY...	R-RNO-112314	22	-0.524333	-1.818207	0.001377	0.186140	0.917
705	REACTOME_RAS_ACTIVATION_UPON_CA2_INFLUX_THROUG...	R-HSA-442982	6	-0.780488	-1.823158	0.006483	0.191050	0.901
706	GOBP_RESPONSE_TO_ALCOHOL	GO:0097305	11	-0.634361	-1.825040	0.004405	0.203475	0.899
707	GOBP_REGULATION_OF_CYTOSOLIC_CALCIUM_ION_CONCE...	GO:0051480	13	-0.607158	-1.830605	0.005857	0.206569	0.883
708	REACTOME_LONG_TERM_POTENTIATION	R-HSA-9620244	10	-0.667578	-1.834206	0.007321	0.217874	0.876
709	GOBP_CELL_CELL_ADHESION	GO:0098609	14	-0.618002	-1.904023	0.004219	0.117604	0.624
710	GOBP_MORPHOGENESIS_OF_AN_EPITHELIUM	GO:0002009	6	-0.809941	-1.907440	0.001605	0.126101	0.611
711	REACTOME_UNBLOCKING_OF_NMDA_RECEPTORS_GLUTAMAT...	R-RNO-438066	12	-0.657895	-1.949656	0.004431	0.091273	0.444
712	REACTOME_NEGATIVE_REGULATION_OF_NMDA_RECEPTOR_...	R-HSA-9617324	8	-0.762500	-1.955284	0.003063	0.097942	0.429
713	GOBP_ENDOTHELIAL_CELL_MIGRATION	GO:0043542	8	-0.764257	-1.963357	0.000000	0.104155	0.401
714	REACTOME_ASSEMBLY_AND_CELL_SURFACE_PRESENTATIO...	R-RNO-9609736	8	-0.762500	-1.964867	0.000000	0.123102	0.396
715	GOBP_TISSUE_MORPHOGENESIS	GO:0048729	7	-0.806186	-1.988465	0.001515	0.116012	0.326
716	GOBP_BLOOD_VESSEL_ENDOTHELIAL_CELL_MIGRATION	GO:0043534	8	-0.764257	-1.989083	0.000000	0.154295	0.325
717	GOBP_POSITIVE_REGULATION_OF_SYNAPTIC_TRANSMISSION	GO:0050806	15	-0.666694	-2.062194	0.000000	0.088657	0.138
718	REACTOME_ACTIVATION_OF_NMDA_RECEPTORS_AND_POST...	R-RNO-442755	16	-0.671154	-2.150442	0.000000	0.044580	0.035

End of Table

2 Supplementary Figures

2.1 Pearson Correlation Plots

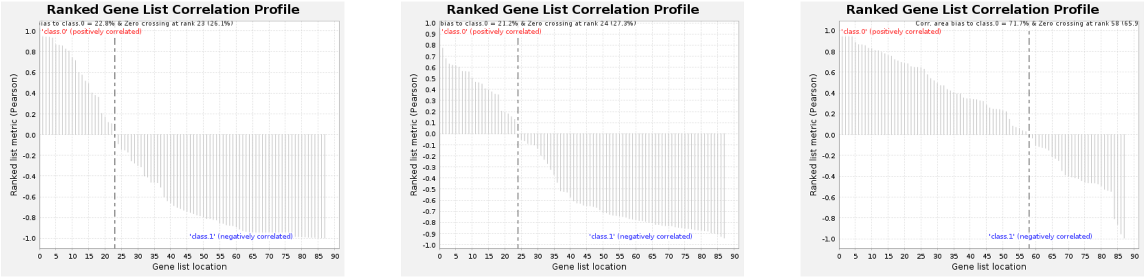

1 DPL Peak | 8 DPL Peak | 45 DPL Peak

Figure S1: Bar plots of SC rostral segment 1, 8 and 45 DPL, Ranked Gene List Pearson Correlation Profile.

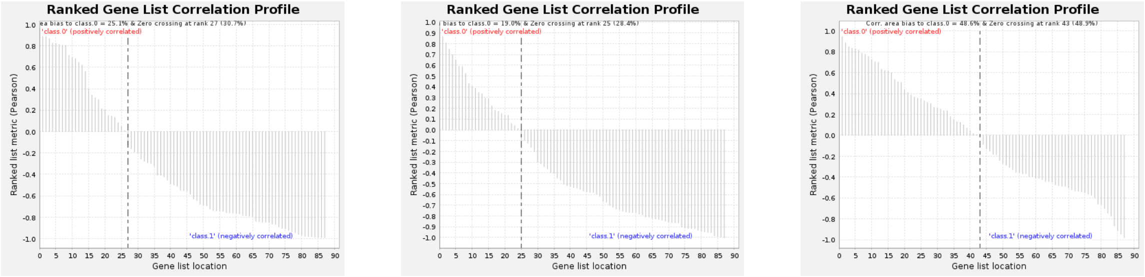

1 DPL Peak | 8 DPL Peak | 45 DPL Peak

Figure S2: Bar plots of SC caudal segment 1, 8 and 45 DPL, Ranked Gene List Pearson Correlation Profile.

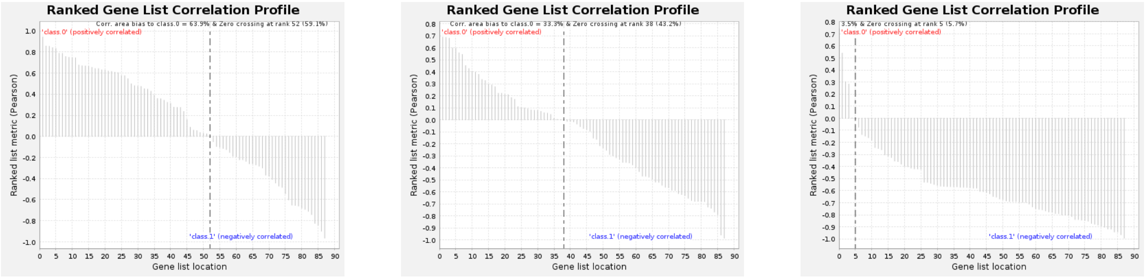

1 DPL Peak | 8 DPL Peak | 45 DPL Peak

Figure S3: Bar plots of SC cervical segment 1, 8 and 45 DPL, Ranked Gene List Pearson Correlation Profile.

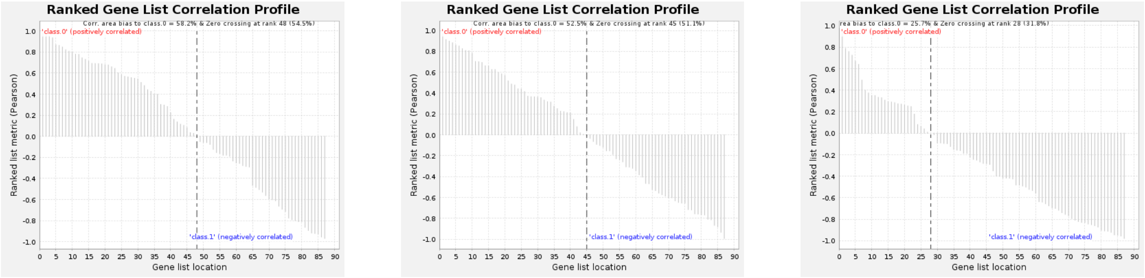

1 DPL Peak 8 DPL Peak 45 DPL Peak

Figure S4: Bar plots of CTX-M 1, 8 and 45 DPL, Ranked Gene List Pearson Correlation Profile.

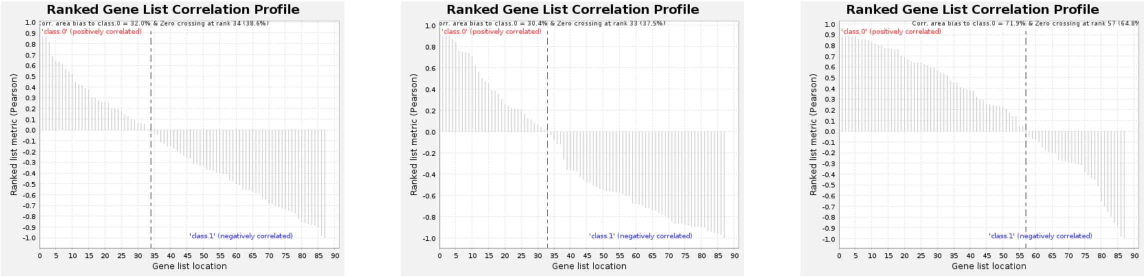

1 DPL Peak 8 DPL Peak 45 DPL Peak

Figure S5: Bar plots of BG 1, 8 and 45 DPL, Ranked Gene List Pearson Correlation Profile.

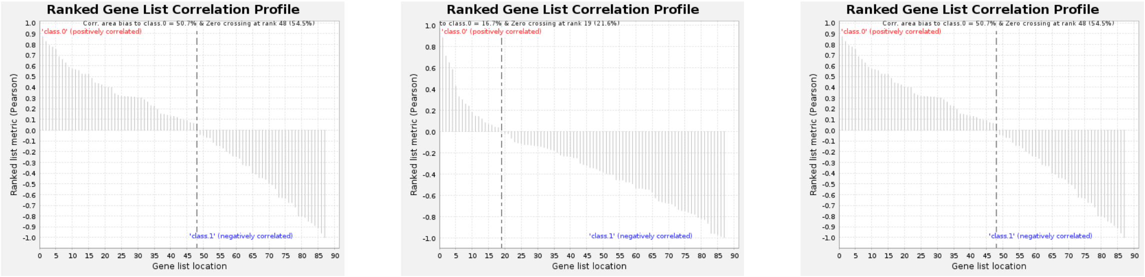

1 DPL Peak 8 DPL Peak 45 DPL Peak

Figure S6: Bar plots of CB 1, 8 and 45 DPL, Ranked Gene List Pearson Correlation Profile.

2.2 NES vs Significance Plots

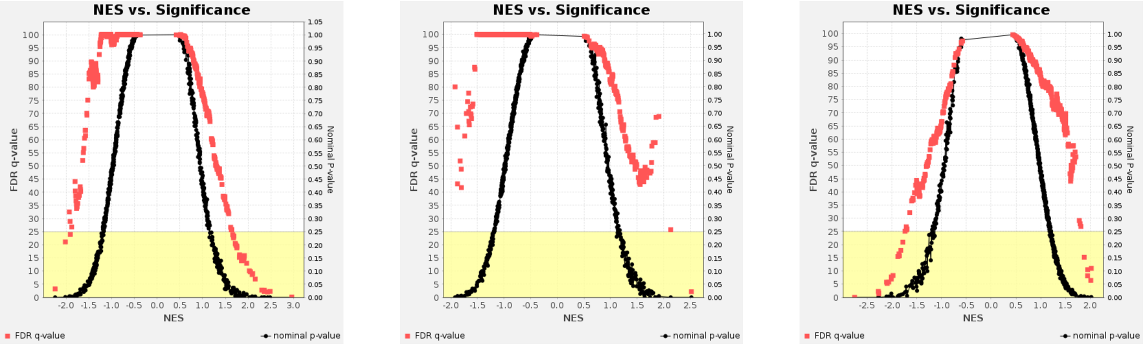

1 DPL Peak   8 DPL Peak   45 DPL Peak

Figure S7: Plots of the distribution of SC rostral segment 1, 8 and 45 DPL, NES values. Significant pathways, FDR ≤ 0.25, are highlighted in yellow.

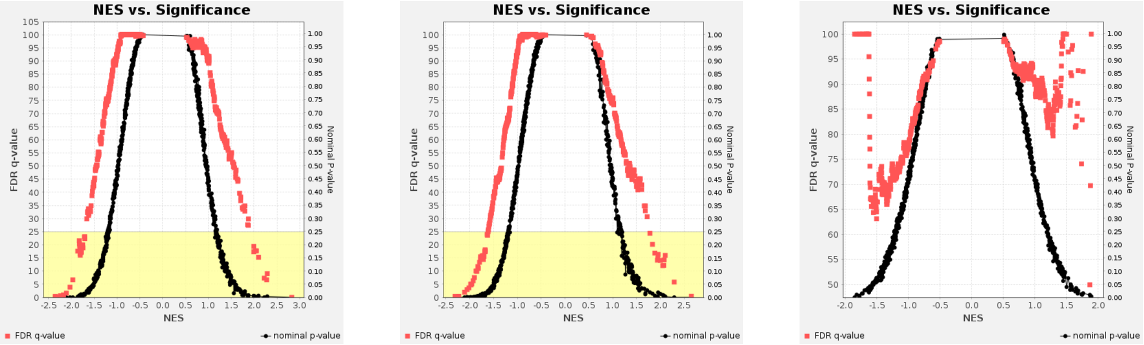

1 DPL Peak   8 DPL Peak   45 DPL Peak

Figure S8: Plots of the distributions of SC caudal section's 1, 8 and 45 DPL, NES values. Significant pathways, FDR ≤ 0.25, are highlighted in yellow.

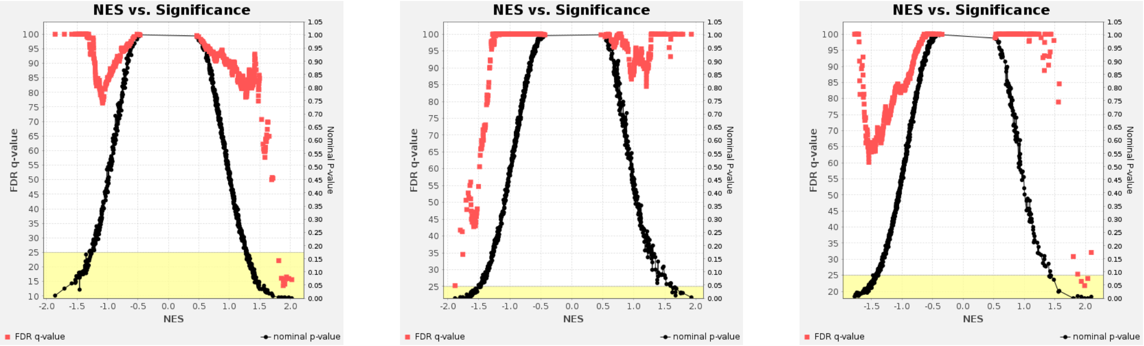

1 DPL Peak　　8 DPL Peak　　45 DPL Peak

Figure S9: Plots of the distributions of SC cervical section's 1, 8 and 45 DPL, NES values. Significant pathways, FDR ≤ 0.25, are highlighted in yellow.

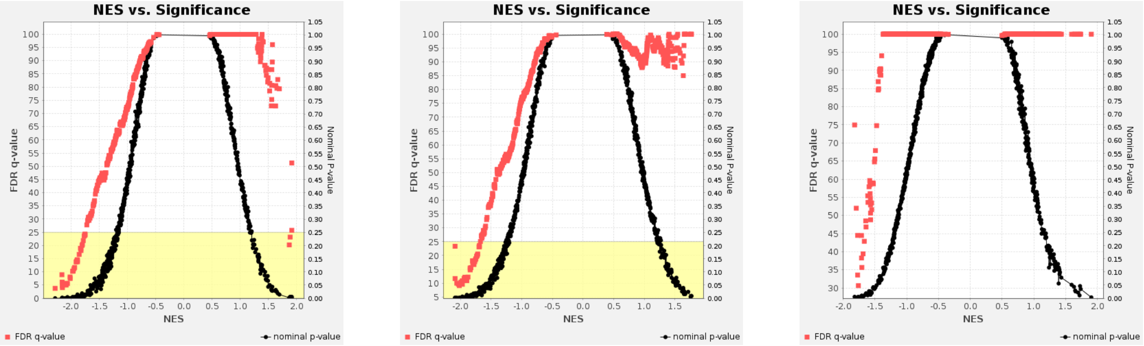

1 DPL Peak　　8 DPL Peak　　45 DPL Peak

Figure S10: Plots of the distributions of CTX-M 1, 8 and 45 DPL, NES values. Significant pathways, FDR ≤ 0.25, are highlighted in yellow.

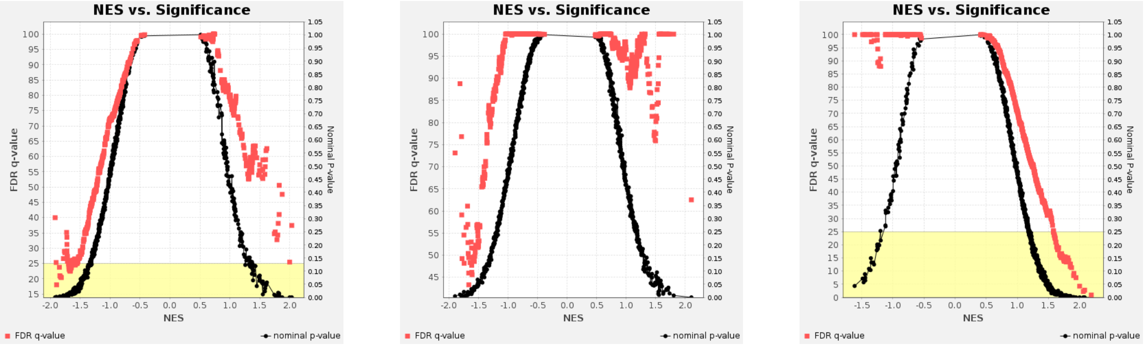

1 DPL Peak　　8 DPL Peak　　45 DPL Peak

Figure S11: Plots of the distributions of BG 1, 8 and 45 DPL, NES values. Significant pathways, FDR ≤ 0.25, are highlighted in yellow.

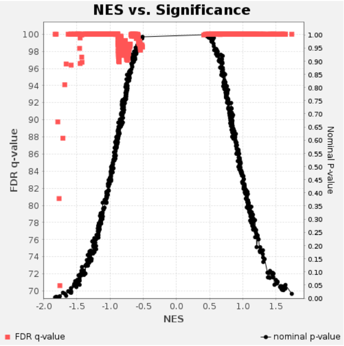

1 DPL Peak

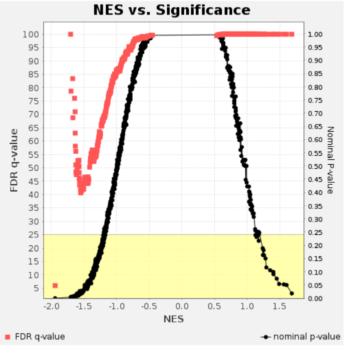

8 DPL Peak

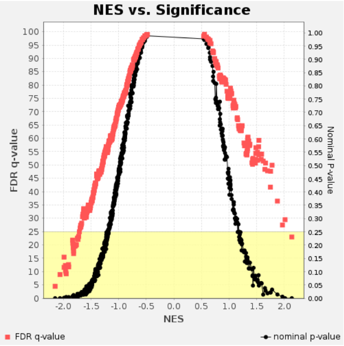

45 DPL Peak

Figure S12: Plots of the distributions of CB 1, 8 and 45 DPL, NES values. Significant pathways, FDR ≤ 0.25, are highlighted in yellow.

2.3 Leading Edge Analysis

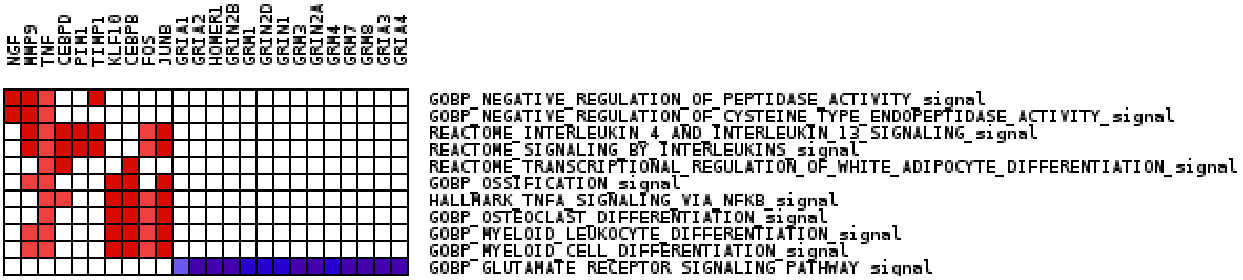

Figure S13: SC rostral 1 DPL Leading Edge Analysis results

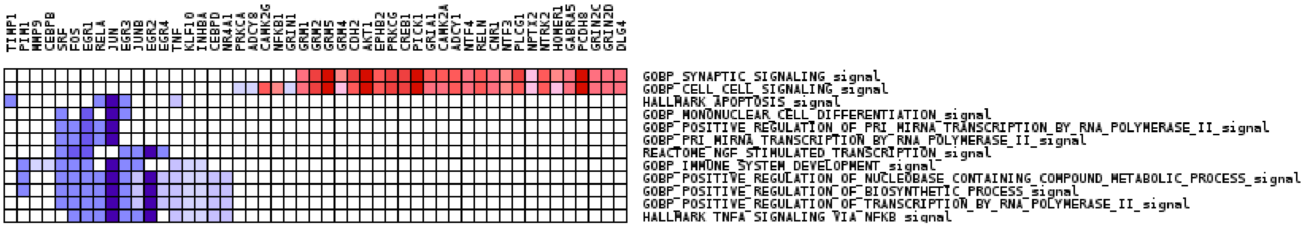

Figure S14: SC rostral 45 DPL Leading Edge Analysis results

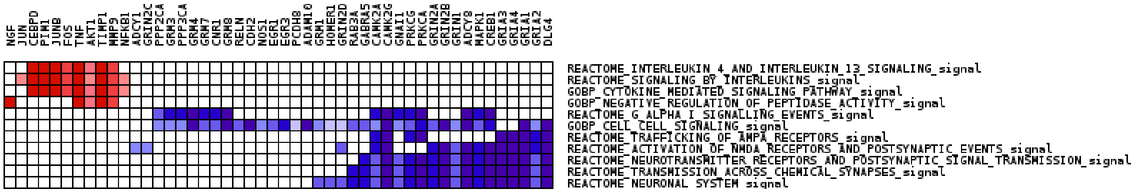

Figure S15: SC caudal 1 DPL Leading Edge Analysis results

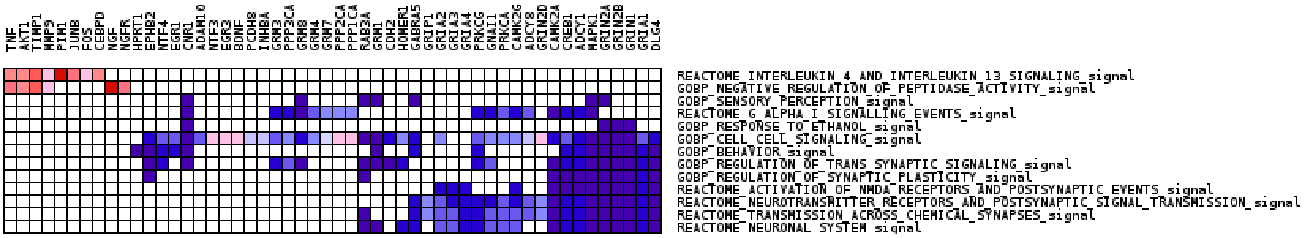

Figure S16: SC caudal 8 DPL Leading Edge Analysis results

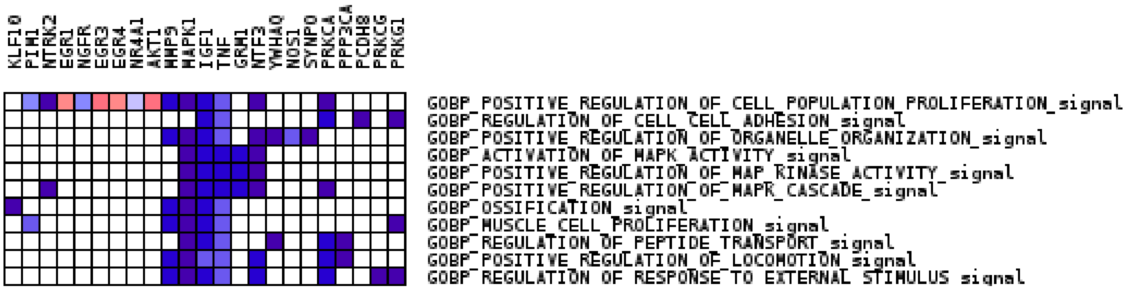

Figure S17: CTX-M 1 DPL Leading Edge Analysis results

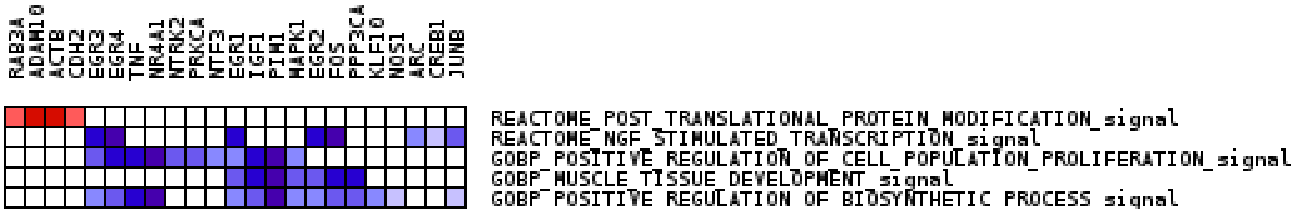

Figure S18: CTX-M 8 DPL Leading Edge Analysis results

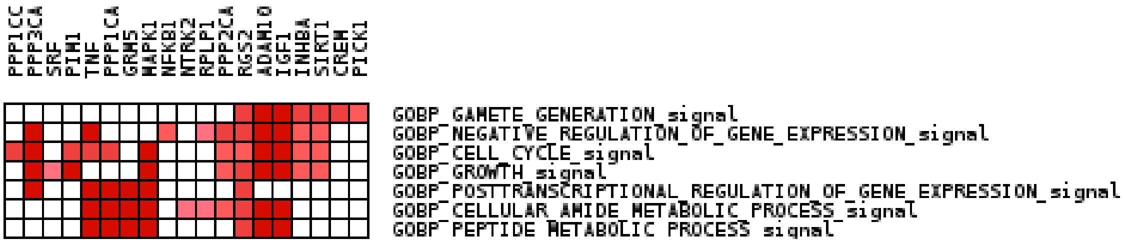

Figure S19: BG 45 DPL Leading Edge Analysis results

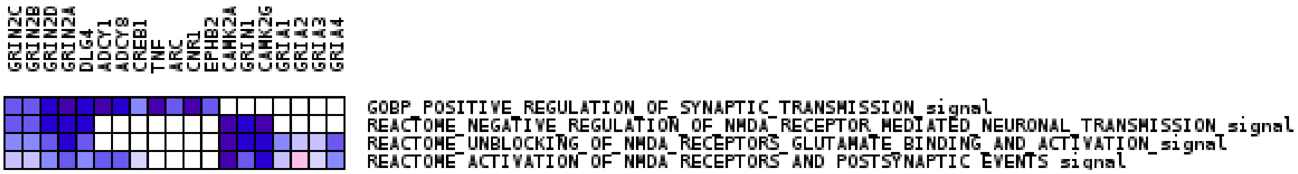

Figure S20: CB 45 DPL Leading Edge Analysis results

2.4 Deregulated Pathways EN Plots and Heatmaps

2.4.1 SC rostral 1 DPL Peak Pathways

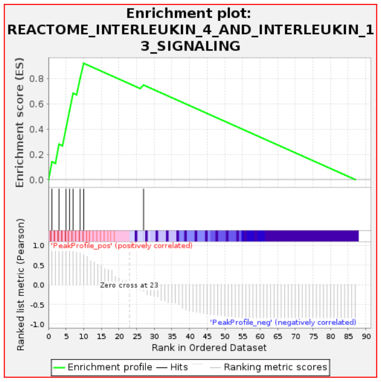

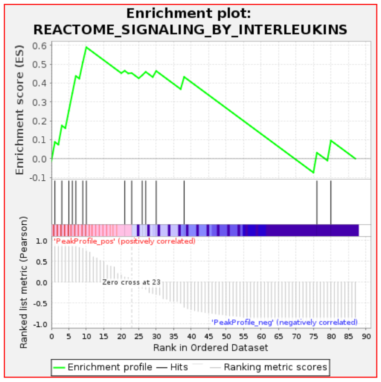

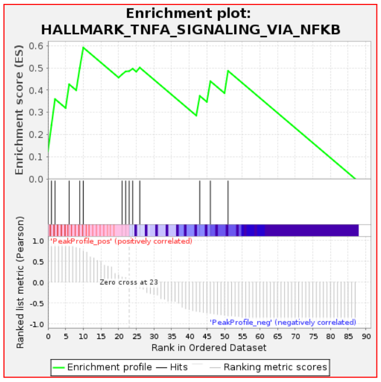

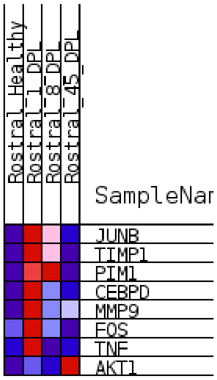

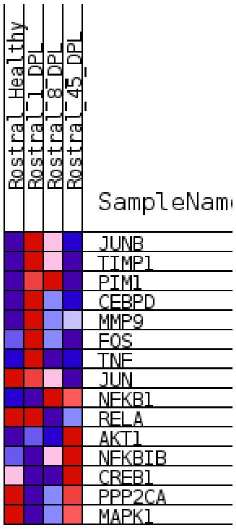

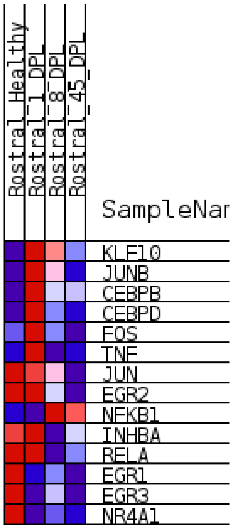

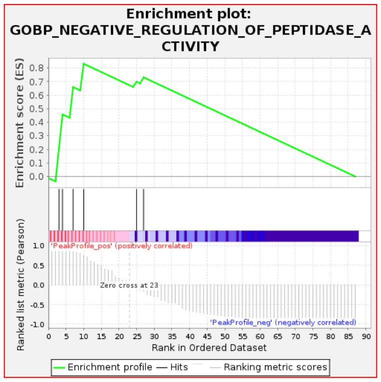
Enrichment plot:
GOBP_NEGATIVE_REGULATION_OF_PEPTIDASE_A
CTIVITY
Enrichment score (ES)
Ranked list metric (Pearson)
'PeakProfile_pos' (positively correlated)
Zero cross at 23
'PeakProfile_neg' (negatively correlated)
Rank in Ordered Dataset
Enrichment profile — Hits — Ranking metric scores

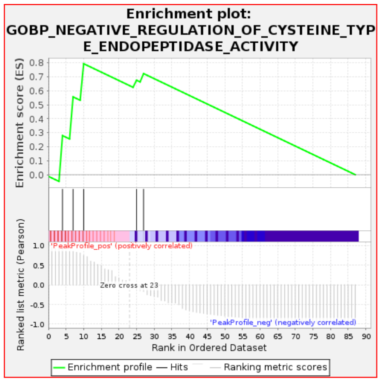
Enrichment plot:
GOBP_NEGATIVE_REGULATION_OF_CYSTEINE_TYP
E_ENDOPEPTIDASE_ACTIVITY
Enrichment score (ES)
Ranked list metric (Pearson)
'PeakProfile_pos' (positively correlated)
Zero cross at 23
'PeakProfile_neg' (negatively correlated)
Rank in Ordered Dataset
Enrichment profile — Hits — Ranking metric scores

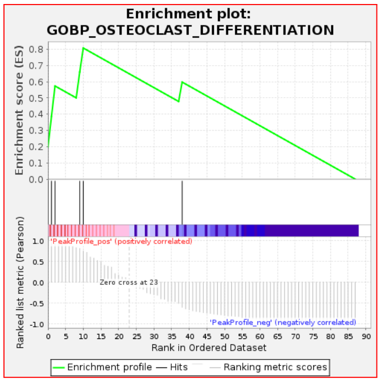
Enrichment plot:
GOBP_OSTEOCLAST_DIFFERENTIATION
Enrichment score (ES)
Ranked list metric (Pearson)
'PeakProfile_pos' (positively correlated)
Zero cross at 23
'PeakProfile_neg' (negatively correlated)
Rank in Ordered Dataset
Enrichment profile — Hits — Ranking metric scores

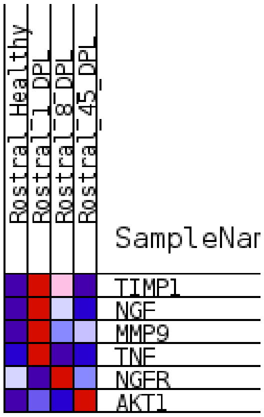
Rostral_Healthy
Rostral_1_DPI
Rostral_8_DPI
Rostral_45_DPI
SampleNar
TIMP1
NGF
MMP9
TNF
NGFR
AKT1

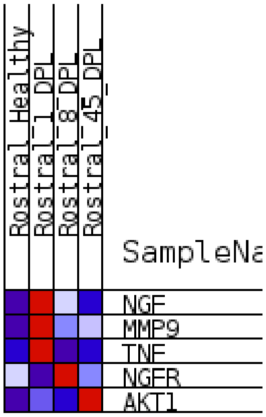
Rostral_Healthy
Rostral_1_DPI
Rostral_8_DPI
Rostral_45_DPI
SampleNa
NGF
MMP9
TNF
NGFR
AKT1

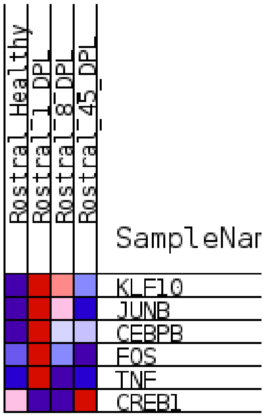
Rostral_Healthy
Rostral_1_DPI
Rostral_8_DPI
Rostral_45_DPI
SampleNar
KLF10
JUNB
CEBPB
FOS
TNF
CREB1

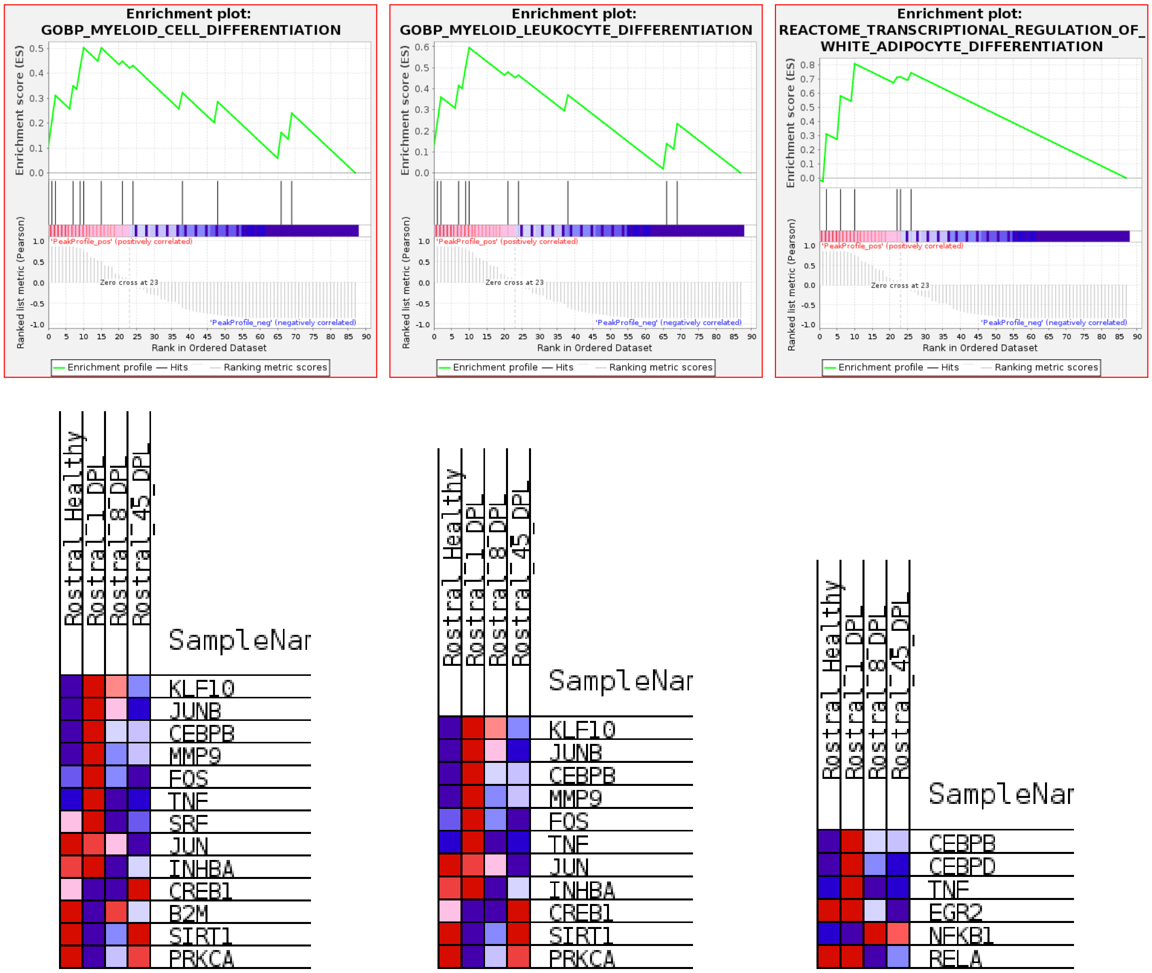
Enrichment plot:
GOBP_MYELOID_CELL_DIFFERENTIATION
Enrichment plot:
GOBP_MYELOID_LEUKOCYTE_DIFFERENTIATION
Enrichment plot:
REACTOME_TRANSCRIPTIONAL_REGULATION_OF_
WHITE_ADIPOCYTE_DIFFERENTIATION
Enrichment score (ES)
Ranked list metric (Pearson)
'PeakProfile_pos' (positively correlated)
Zero cross at 23
'PeakProfile_neg' (negatively correlated)
Rank in Ordered Dataset
Enrichment profile — Hits
Ranking metric scores
Rostral_Healthy
Rostral_1_DPL
Rostral_8_DPL
Rostral_45_DPL_
SampleNar
KLF10
JUNB
CEBPB
MMP9
FOS
TNF
SRF
JUN
INHBA
CREB1
B2M
SIRT1
PRKCA
CEBPD
EGR2
NFKB1
RELA

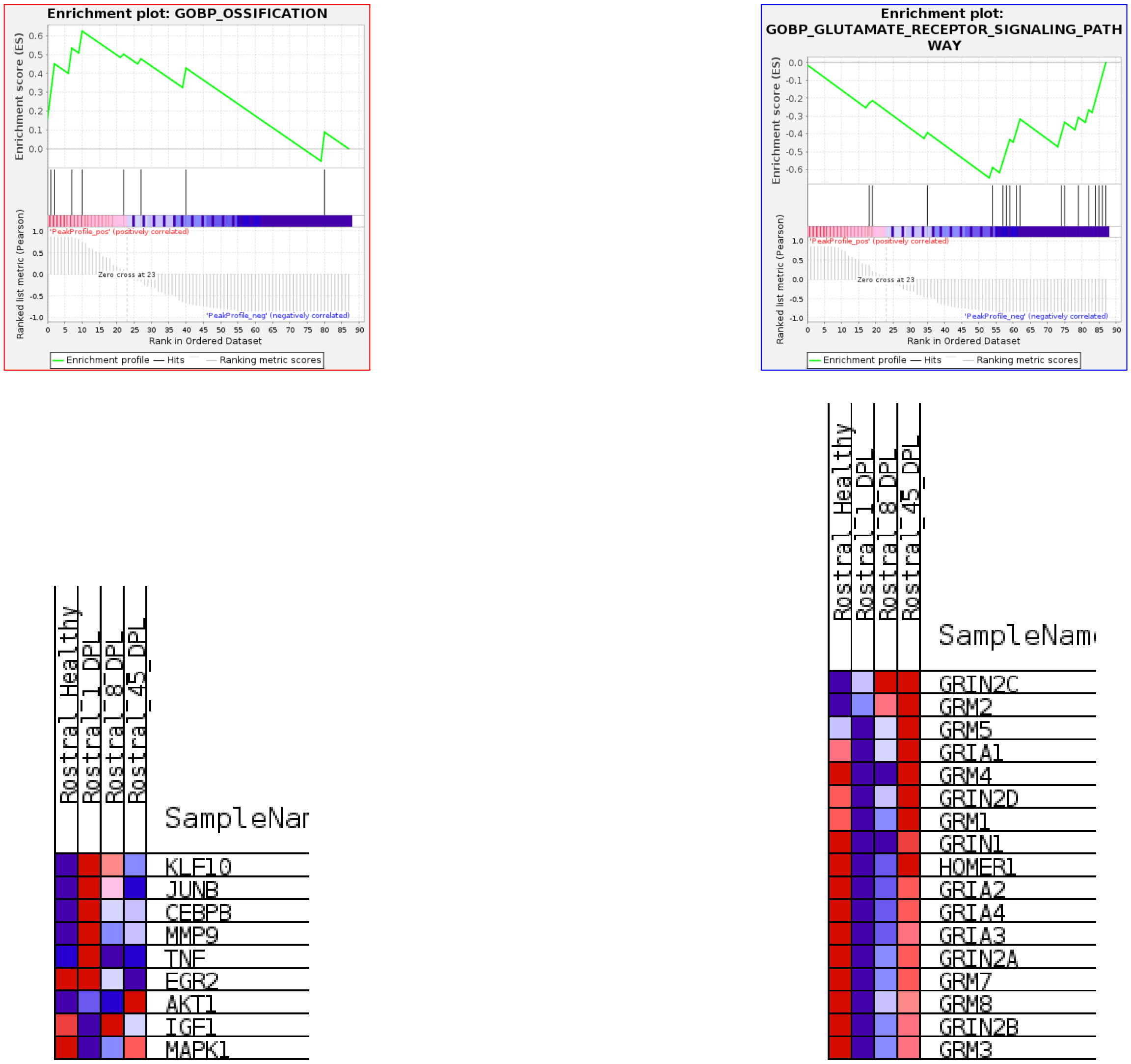

Figure S21: Enrichment plots (upper) and corresponding gene set Heatmaps (lower) of SC rostral 1 DPL peak deregulated pathways (FDR ≤ 0.1). Up-regulated pathways are framed in red, down-regulated in blue.

2.4.2 SC rostral 8 DPL Peak Pathways

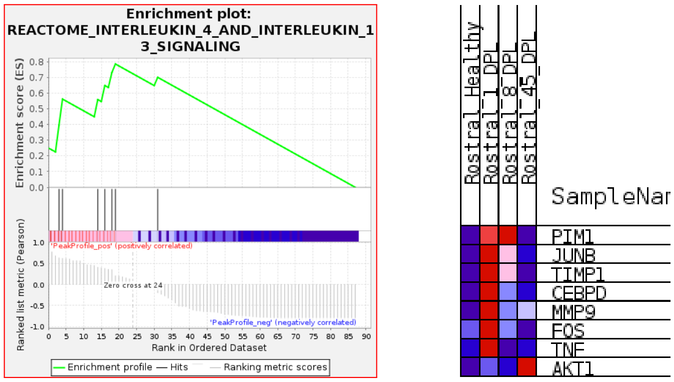

Figure S22: Enrichment plots (upper) and corresponding gene set Heatmaps (lower) of SC rostral 8 DPL peak deregulated pathways (FDR ≤ 0.1). Up-regulated pathways are framed in red, down-regulated in blue.

2.4.3 SC rostral 45 DPL Peak Pathways

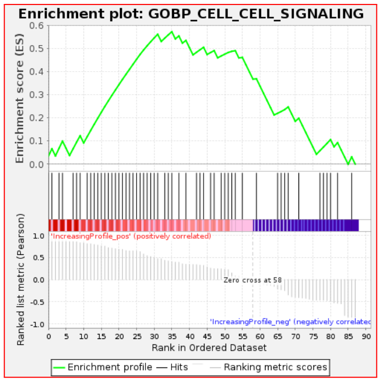
Enrichment plot: GOBP_CELL_CELL_SIGNALING
Enrichment score (ES)
Ranked list metric (Pearson)
'IncreasingProfile_pos' (positively correlated)
Zero cross at 58
'IncreasingProfile_neg' (negatively correlated)
Rank in Ordered Dataset
Enrichment profile — Hits — Ranking metric scores

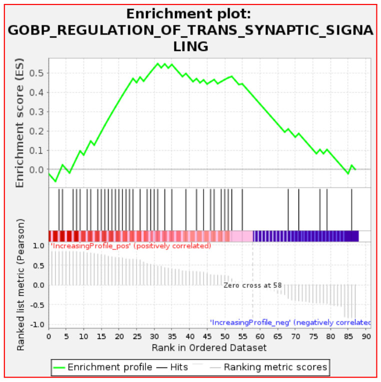
Enrichment plot: GOBP_REGULATION_OF_TRANS_SYNAPTIC_SIGNALING
Enrichment score (ES)
Ranked list metric (Pearson)
'IncreasingProfile_pos' (positively correlated)
Zero cross at 58
'IncreasingProfile_neg' (negatively correlated)
Rank in Ordered Dataset
Enrichment profile — Hits — Ranking metric scores

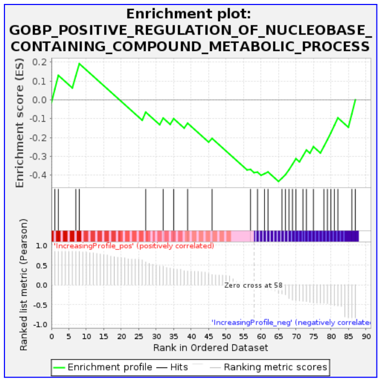
Enrichment plot: GOBP_POSITIVE_REGULATION_OF_NUCLEOBASE_CONTAINING_COMPOUND_METABOLIC_PROCESS
Enrichment score (ES)
Ranked list metric (Pearson)
'IncreasingProfile_pos' (positively correlated)
Zero cross at 58
'IncreasingProfile_neg' (negatively correlated)
Rank in Ordered Dataset
Enrichment profile — Hits — Ranking metric scores

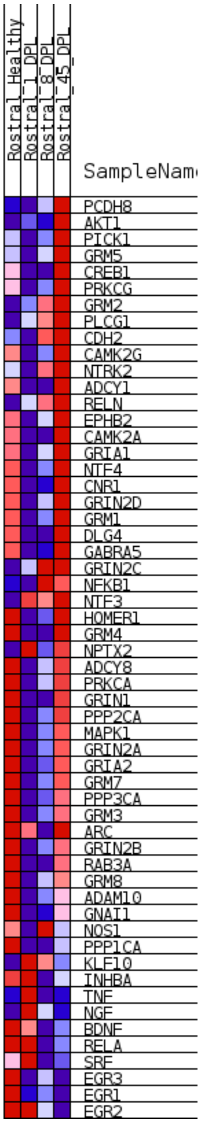
Rostral_Healthy
Rostral_1_DPL
Rostral_8_DPL
Rostral_45_DPL
SampleNam
PCDH8
AKT1
PICK1
GRM5
CREB1
PRKCG
GRM2
PLCG1
CDH2
CAMK2G
NTRK2
ADCY1
RELN
EPHB2
CAMK2A
GRIA1
NTF4
CNR1
GRIN2D
GRM1
DLG4
GABRA5
GRIN2C
NFKB1
NTF3
HOMER1
GRM4
NPTX2
ADCY8
PRKCA
GRIN1
PPP2CA
MAPK1
GRIN2A
GRIA2
GRM7
PPP3CA
GRM3
ARC
GRIN2B
RAB3A
GRM8
ADAM10
GNAI1
NOS1
PPP1CA
KLF10
INHBA
TNF
NGF
BDNF
RELA
SRF
EGR3
EGR1
EGR2

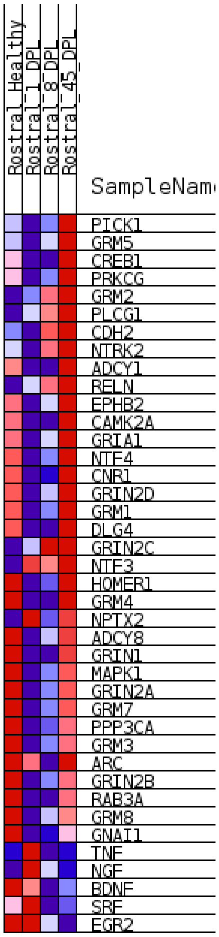
Rostral_Healthy
Rostral_1_DPL
Rostral_8_DPL
Rostral_45_DPL
SampleNam
PICK1
GRM5
CREB1
PRKCG
GRM2
PLCG1
CDH2
NTRK2
ADCY1
RELN
EPHB2
CAMK2A
GRIA1
NTF4
CNR1
GRIN2D
GRM1
DLG4
GRIN2C
NTF3
HOMER1
GRM4
NPTX2
ADCY8
GRIN1
MAPK1
GRIN2A
GRM7
PPP3CA
GRM3
ARC
GRIN2B
RAB3A
GRM8
GNAI1
TNF
NGF
BDNF
SRF
EGR2

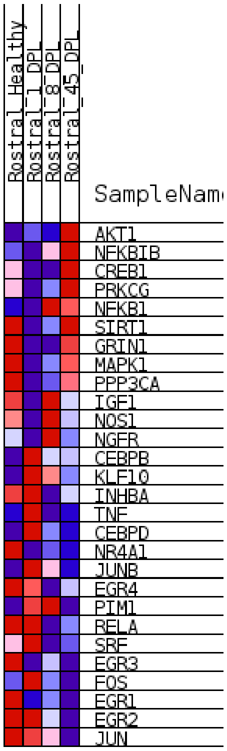
Rostral_Healthy
Rostral_1_DPL
Rostral_8_DPL
Rostral_45_DPL
SampleNam
AKT1
NFKBIB
CREB1
PRKCG
NFKB1
SIRT1
GRIN1
MAPK1
PPP3CA
IGF1
NOS1
NGFR
CEBPB
KLF10
INHBA
TNF
CEBPD
NR4A1
JUNB
EGR4
PIM1
RELA
SRF
EGR3
FOS
EGR1
EGR2
JUN

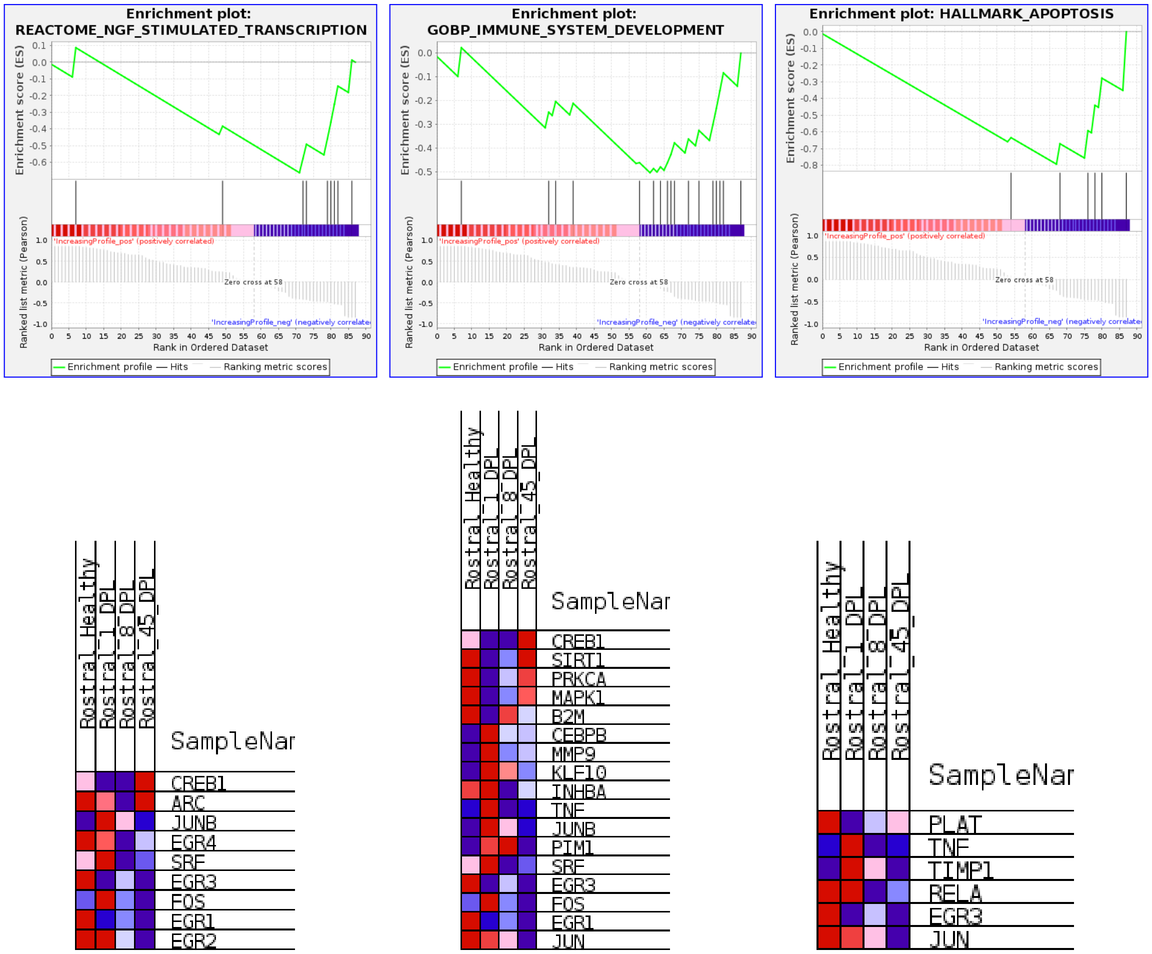

Enrichment plot: REACTOME_NGF_STIMULATED_TRANSCRIPTION
Enrichment plot: GOBP_IMMUNE_SYSTEM_DEVELOPMENT
Enrichment plot: HALLMARK_APOPTOSIS
Enrichment score (ES)
Ranked list metric (Pearson)
Rank in Ordered Dataset
Zero cross at 58
Enrichment profile — Hits — Ranking metric scores
Rostral_Healthy
Rostral_1_DPL
Rostral_8_DPL
Rostral_45_DPL
SampleNar
CREB1
ARC
JUNB
EGR4
SRF
EGR3
FOS
EGR1
EGR2
CREB1
SIRT1
PRKCA
MAPK1
B2M
CEBPB
MMP9
KLF10
INHBA
TNF
JUNB
PIM1
SRF
EGR3
FOS
EGR1
JUN
PLAT
TNF
TIMP1
RELA
EGR3
JUN

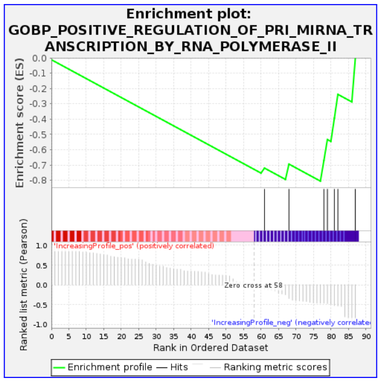
Enrichment plot:
GOBP_POSITIVE_REGULATION_OF_PRI_MIRNA_TRANSCRIPTION_BY_RNA_POLYMERASE_II
Enrichment score (ES)
Ranked list metric (Pearson)
'IncreasingProfile_pos' (positively correlated)
Zero cross at 58
'IncreasingProfile_neg' (negatively correlated)
Rank in Ordered Dataset
Enrichment profile — Hits — Ranking metric scores

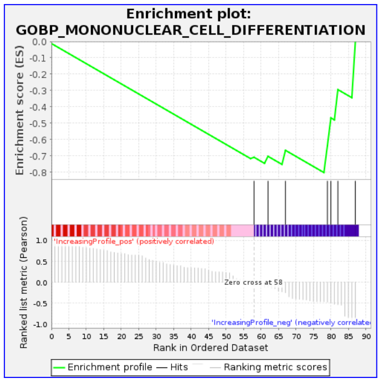
Enrichment plot:
GOBP_MONONUCLEAR_CELL_DIFFERENTIATION
Enrichment score (ES)
Ranked list metric (Pearson)
'IncreasingProfile_pos' (positively correlated)
Zero cross at 58
'IncreasingProfile_neg' (negatively correlated)
Rank in Ordered Dataset
Enrichment profile — Hits — Ranking metric scores

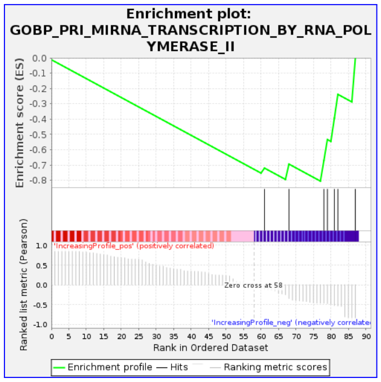
Enrichment plot:
GOBP_PRI_MIRNA_TRANSCRIPTION_BY_RNA_POLYMERASE_II
Enrichment score (ES)
Ranked list metric (Pearson)
'IncreasingProfile_pos' (positively correlated)
Zero cross at 58
'IncreasingProfile_neg' (negatively correlated)
Rank in Ordered Dataset
Enrichment profile — Hits — Ranking metric scores

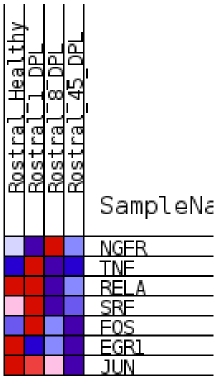
Rostral_Healthy
Rostral_1_DPL
Rostral_8_DPL
Rostral_45_DPL
SampleNa
NGFR
TNF
RELA
SRF
FOS
EGR1
JUN

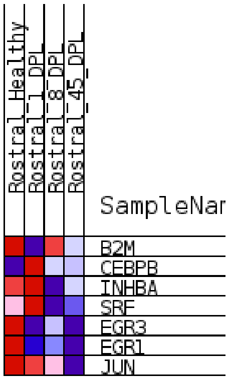
Rostral_Healthy
Rostral_1_DPL
Rostral_8_DPL
Rostral_45_DPL
SampleNar
B2M
CEBPB
INHBA
SRF
EGR3
EGR1
JUN

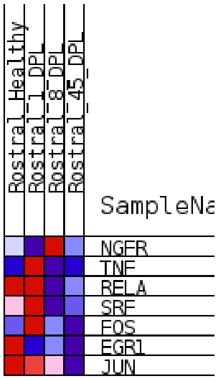
Rostral_Healthy
Rostral_1_DPL
Rostral_8_DPL
Rostral_45_DPL
SampleNa
NGFR
TNF
RELA
SRF
FOS
EGR1
JUN

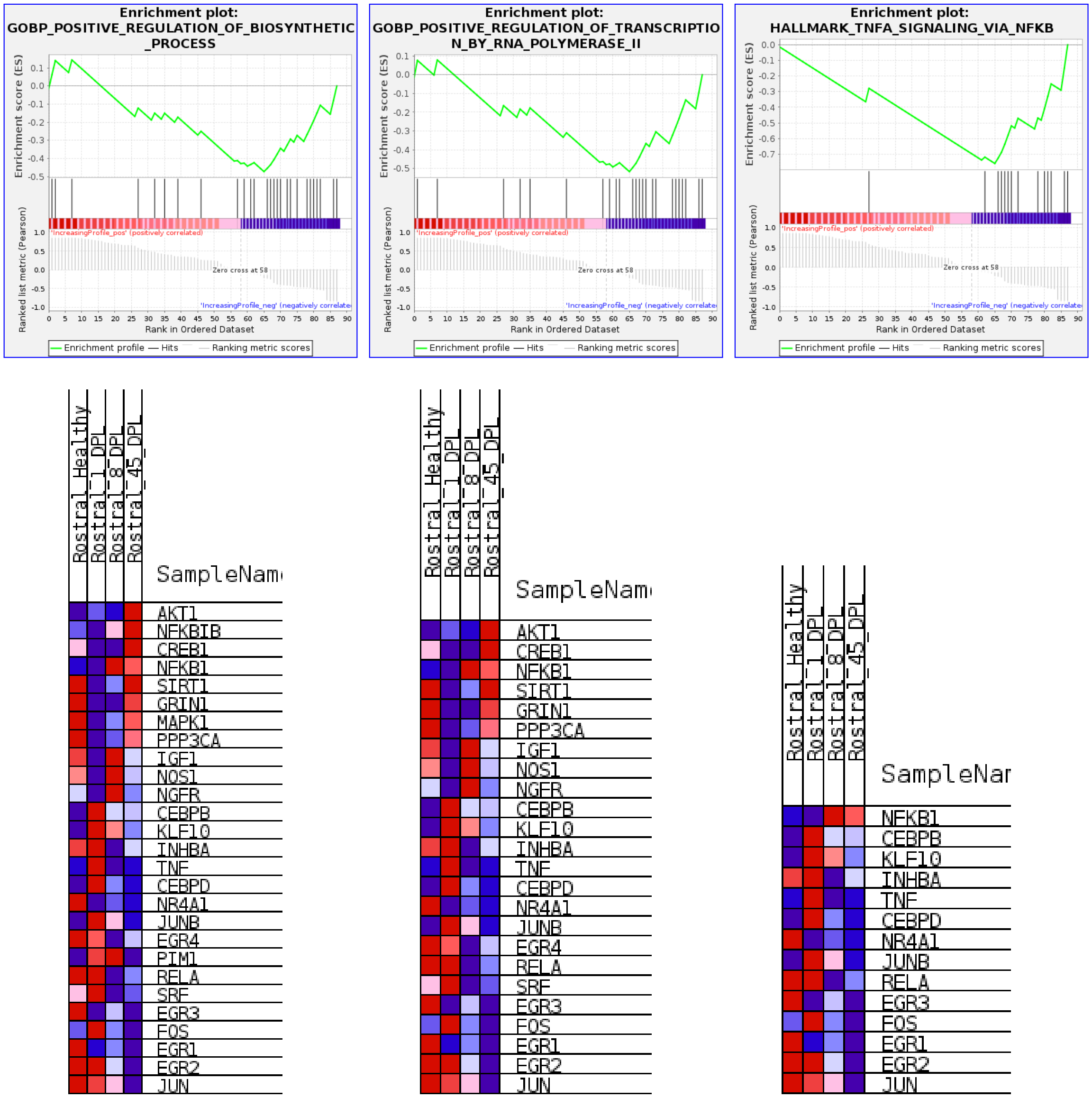

Figure S23: Enrichment plots (upper) and corresponding gene set Heatmaps (lower) of SC rostral 45 DPL peak deregulated pathways (FDR ≤ 0.1). Up-regulated pathways are framed in red, down-regulated in blue.

2.4.4 SC caudal 1 DPL Peak Pathways

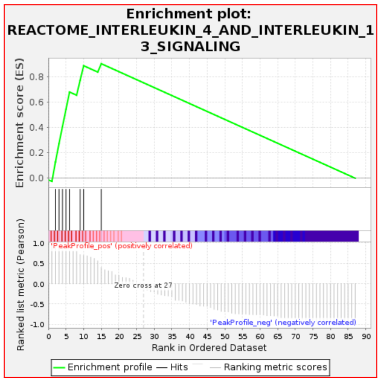

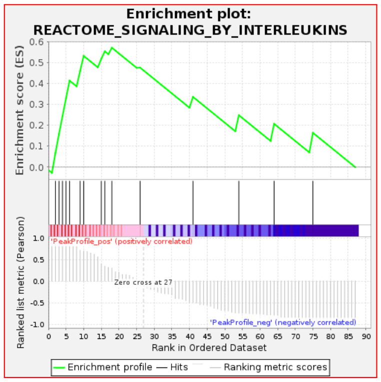

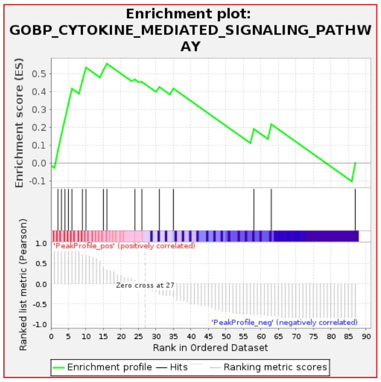

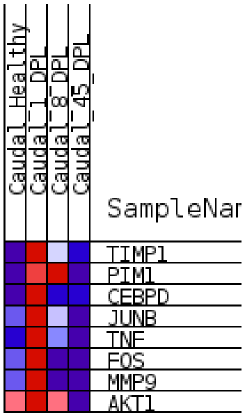

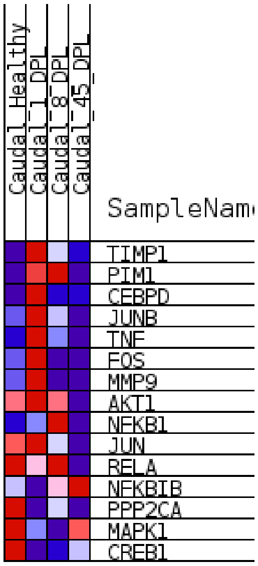

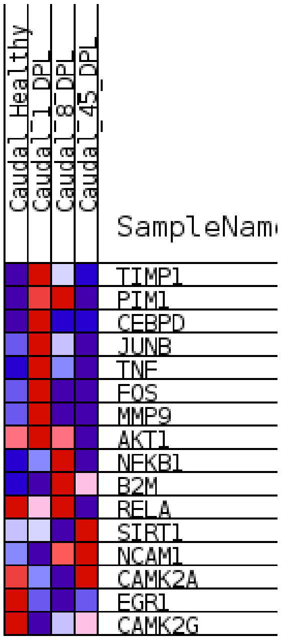

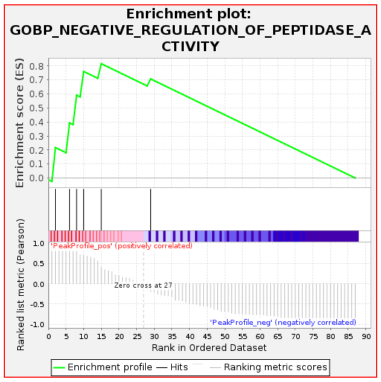
Enrichment plot:
GOBP_NEGATIVE_REGULATION_OF_PEPTIDASE_ACTIVITY
Enrichment score (ES)
Ranked list metric (Pearson)
'PeakProfile_pos' (positively correlated)
Zero cross at 27
'PeakProfile_neg' (negatively correlated)
Rank in Ordered Dataset
Enrichment profile — Hits — Ranking metric scores

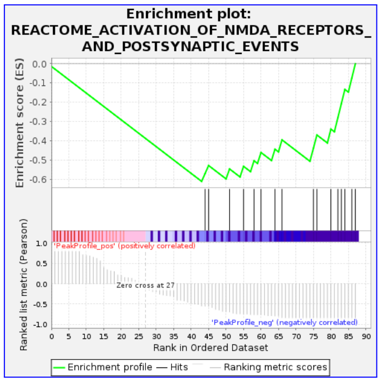
Enrichment plot:
REACTOME_ACTIVATION_OF_NMDA_RECEPTORS_AND_POSTSYNAPTIC_EVENTS
Enrichment score (ES)
Ranked list metric (Pearson)
'PeakProfile_pos' (positively correlated)
Zero cross at 27
'PeakProfile_neg' (negatively correlated)
Rank in Ordered Dataset
Enrichment profile — Hits — Ranking metric scores

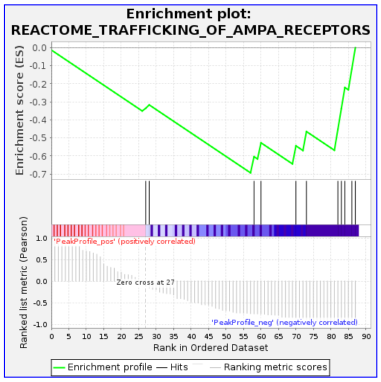
Enrichment plot:
REACTOME_TRAFFICKING_OF_AMPA_RECEPTORS
Enrichment score (ES)
Ranked list metric (Pearson)
'PeakProfile_pos' (positively correlated)
Zero cross at 27
'PeakProfile_neg' (negatively correlated)
Rank in Ordered Dataset
Enrichment profile — Hits — Ranking metric scores

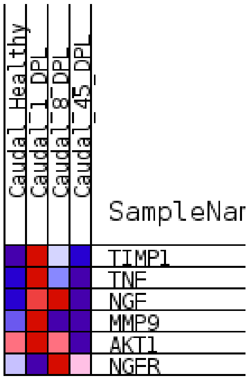
Caudal_Healthy
Caudal_1_DPL
Caudal_8_DPL
Caudal_45_DPL
SampleNar
TIMP1
TNF
NGF
MMP9
AKT1
NGFR

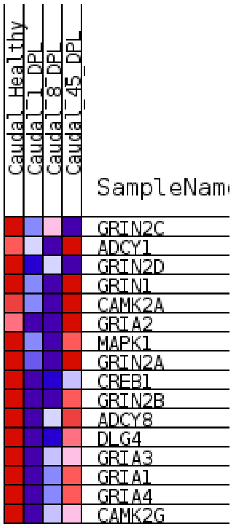
Caudal_Healthy
Caudal_1_DPL
Caudal_8_DPL
Caudal_45_DPL
SampleNam
GRIN2C
ADCY1
GRIN2D
GRIN1
CAMK2A
GRIA2
MAPK1
GRIN2A
CREB1
GRIN2B
ADCY8
DLG4
GRIA3
GRIA1
GRIA4
CAMK2G

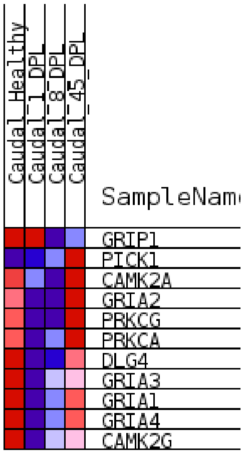
Caudal_Healthy
Caudal_1_DPL
Caudal_8_DPL
Caudal_45_DPL
SampleNam
GRIP1
PICK1
CAMK2A
GRIA2
PRKCG
PRKCA
DLG4
GRIA3
GRIA1
GRIA4
CAMK2G

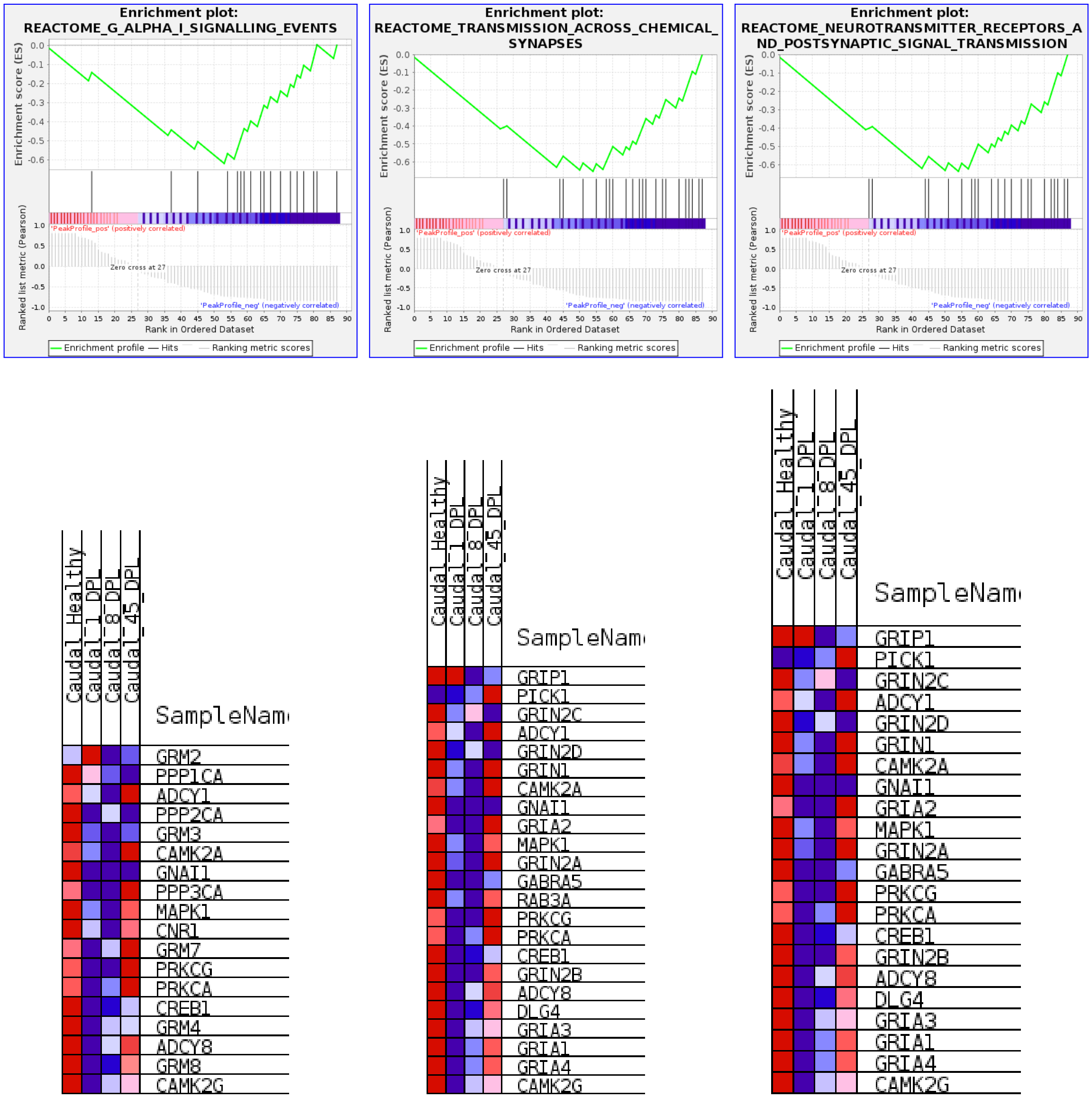
Enrichment plot:
REACTOME_G_ALPHA_I_SIGNALLING_EVENTS
Enrichment plot:
REACTOME_TRANSMISSION_ACROSS_CHEMICAL_SYNAPSES
Enrichment plot:
REACTOME_NEUROTRANSMITTER_RECEPTORS_AND_POSTSYNAPTIC_SIGNAL_TRANSMISSION
Enrichment score (ES)
Ranked list metric (Pearson)
'PeakProfile_pos' (positively correlated)
Zero cross at 27
'PeakProfile_neg' (negatively correlated)
Rank in Ordered Dataset
Enrichment profile — Hits — Ranking metric scores
Caudal_Healthy
Caudal_1_DPL
Caudal_8_DPL
Caudal_45_DPL
SampleNam
GRM2
PPP1CA
ADCY1
PPP2CA
GRM3
CAMK2A
GNAI1
PPP3CA
MAPK1
CNR1
GRM7
PRKCG
PRKCA
CREB1
GRM4
ADCY8
GRM8
CAMK2G
GRIP1
PICK1
GRIN2C
ADCY1
GRIN2D
GRIN1
CAMK2A
GNAI1
GRIA2
MAPK1
GRIN2A
GABRA5
RAB3A
PRKCG
PRKCA
CREB1
GRIN2B
ADCY8
DLG4
GRIA3
GRIA1
GRIA4
CAMK2G
GRIP1
PICK1
GRIN2C
ADCY1
GRIN2D
GRIN1
CAMK2A
GNAI1
GRIA2
MAPK1
GRIN2A
GABRA5
PRKCG
PRKCA
CREB1
GRIN2B
ADCY8
DLG4
GRIA3
GRIA1
GRIA4
CAMK2G

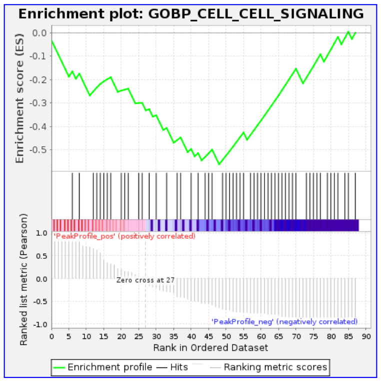

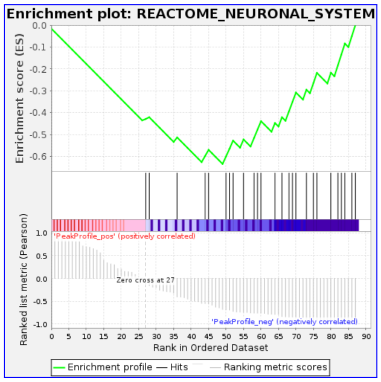

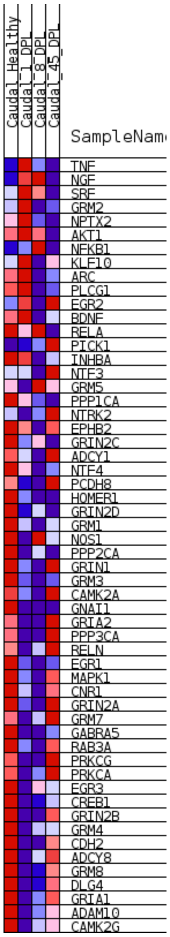

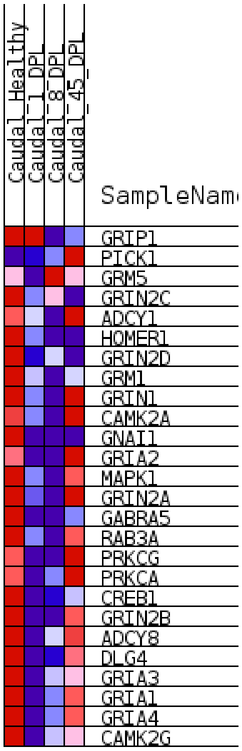

Figure S24: Enrichment plots (upper) and corresponding gene set Heatmaps (lower) of SC caudal 1 DPL peak deregulated pathways (FDR ≤ 0.1). Up-regulated pathways are framed in red, down-regulated in blue.

2.4.5 SC caudal 8 DPL Peak Pathways

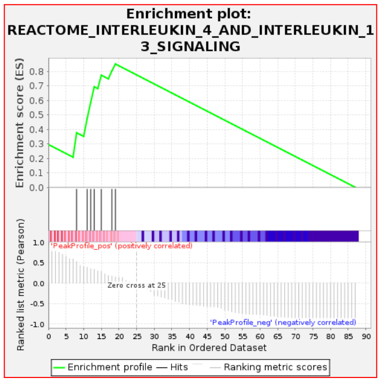

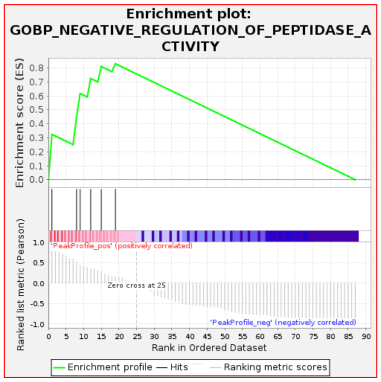

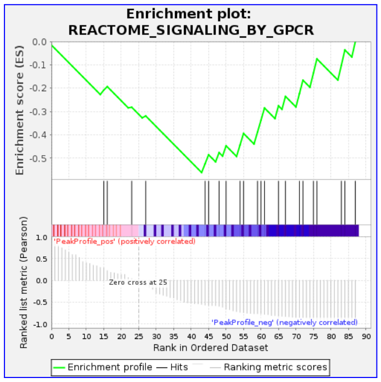

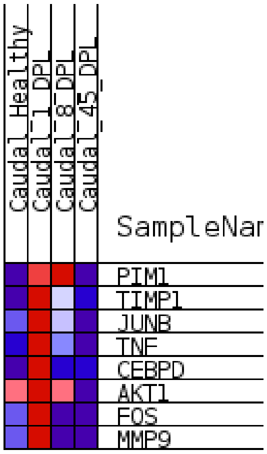

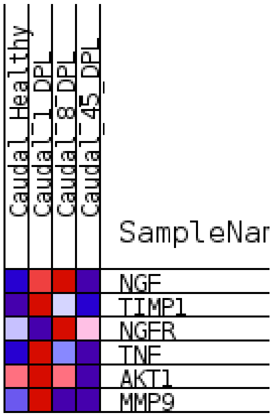

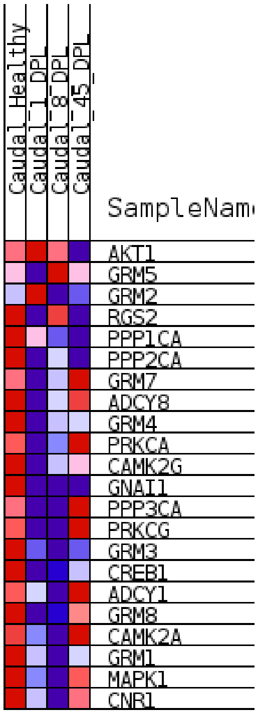

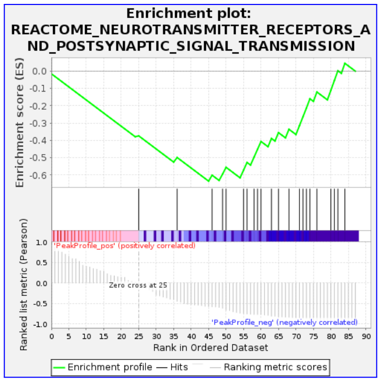
Enrichment plot:
REACTOME_NEUROTRANSMITTER_RECEPTORS_AND_POSTSYNAPTIC_SIGNAL_TRANSMISSION
Enrichment score (ES)
Ranked list metric (Pearson)
'PeakProfile_pos' (positively correlated)
Zero cross at 25
'PeakProfile_neg' (negatively correlated)
Rank in Ordered Dataset
Enrichment profile — Hits — Ranking metric scores

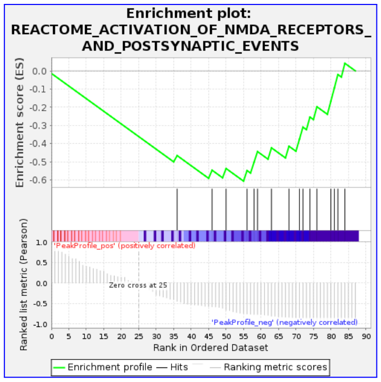
Enrichment plot:
REACTOME_ACTIVATION_OF_NMDA_RECEPTORS_AND_POSTSYNAPTIC_EVENTS
Enrichment score (ES)
Ranked list metric (Pearson)
'PeakProfile_pos' (positively correlated)
Zero cross at 25
'PeakProfile_neg' (negatively correlated)
Rank in Ordered Dataset
Enrichment profile — Hits — Ranking metric scores

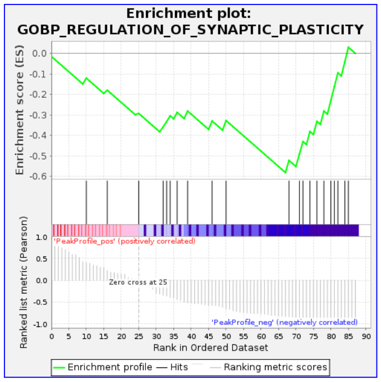
Enrichment plot:
GOBP_REGULATION_OF_SYNAPTIC_PLASTICITY
Enrichment score (ES)
Ranked list metric (Pearson)
'PeakProfile_pos' (positively correlated)
Zero cross at 25
'PeakProfile_neg' (negatively correlated)
Rank in Ordered Dataset
Enrichment profile — Hits — Ranking metric scores

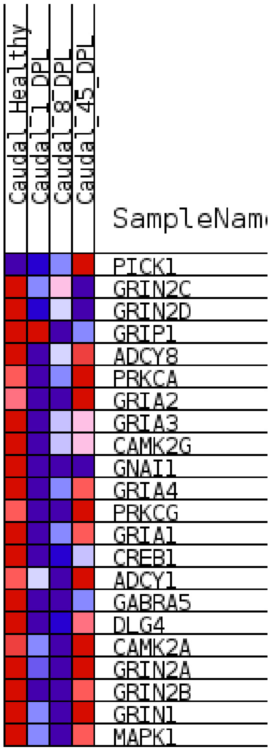
Caudal_Healthy
Caudal_1_DPL
Caudal_8_DPL
Caudal_45_DPL
SampleNam
PICK1
GRIN2C
GRIN2D
GRIP1
ADCY8
PRKCA
GRIA2
GRIA3
CAMK2G
GNAI1
GRIA4
PRKCG
GRIA1
CREB1
ADCY1
GABRA5
DLG4
CAMK2A
GRIN2A
GRIN2B
GRIN1
MAPK1

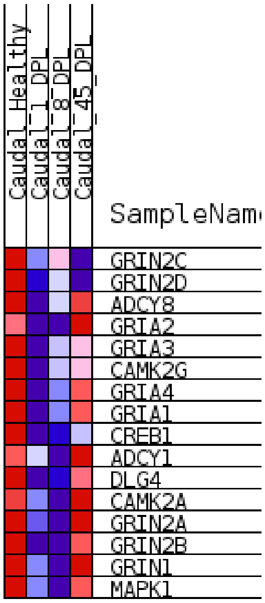
Caudal_Healthy
Caudal_1_DPL
Caudal_8_DPL
Caudal_45_DPL
SampleNam
GRIN2C
GRIN2D
ADCY8
GRIA2
GRIA3
CAMK2G
GRIA4
GRIA1
CREB1
ADCY1
DLG4
CAMK2A
GRIN2A
GRIN2B
GRIN1
MAPK1

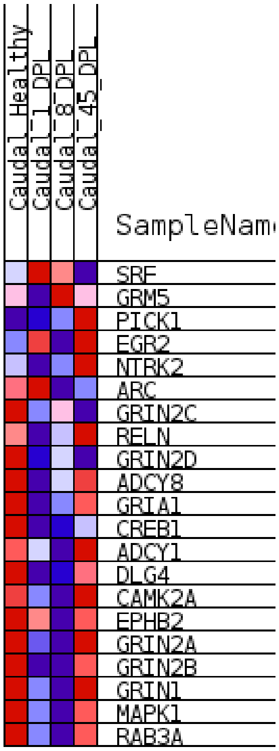
Caudal_Healthy
Caudal_1_DPL
Caudal_8_DPL
Caudal_45_DPL
SampleNam
SRF
GRM5
PICK1
EGR2
NTRK2
ARC
GRIN2C
RELN
GRIN2D
ADCY8
GRIA1
CREB1
ADCY1
DLG4
CAMK2A
EPHB2
GRIN2A
GRIN2B
GRIN1
MAPK1
RAB3A

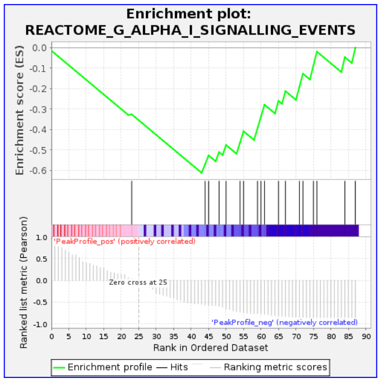
Enrichment plot:
REACTOME_G_ALPHA_I_SIGNALLING_EVENTS
Enrichment score (ES)
'PeakProfile_pos' (positively correlated)
Zero cross at 25
'PeakProfile_neg' (negatively correlated)
Ranked list metric (Pearson)
Rank in Ordered Dataset
Enrichment profile — Hits — Ranking metric scores

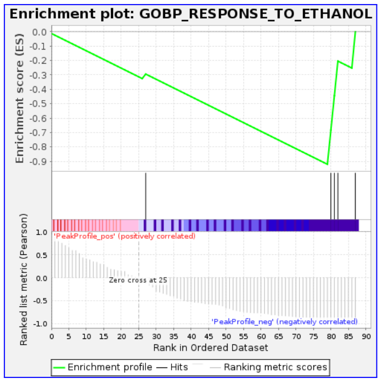
Enrichment plot: GOBP_RESPONSE_TO_ETHANOL
Enrichment score (ES)
'PeakProfile_pos' (positively correlated)
Zero cross at 25
'PeakProfile_neg' (negatively correlated)
Ranked list metric (Pearson)
Rank in Ordered Dataset
Enrichment profile — Hits — Ranking metric scores

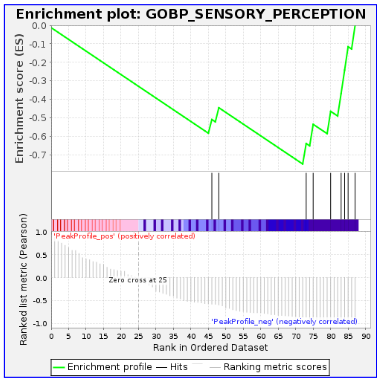
Enrichment plot: GOBP_SENSORY_PERCEPTION
Enrichment score (ES)
'PeakProfile_pos' (positively correlated)
Zero cross at 25
'PeakProfile_neg' (negatively correlated)
Ranked list metric (Pearson)
Rank in Ordered Dataset
Enrichment profile — Hits — Ranking metric scores

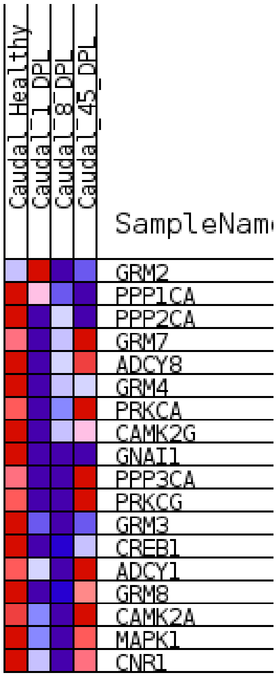
Caudal_Healthy
Caudal_1_DPL
Caudal_8_DPL
Caudal_45_DPL
SampleNam
GRM2
PPP1CA
PPP2CA
GRM7
ADCY8
GRM4
PRKCA
CAMK2G
GNAI1
PPP3CA
PRKCG
GRM3
CREB1
ADCY1
GRM8
CAMK2A
MAPK1
CNR1

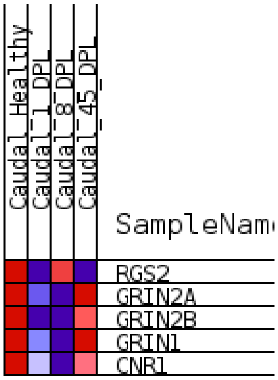
Caudal_Healthy
Caudal_1_DPL
Caudal_8_DPL
Caudal_45_DPL
SampleNam
RGS2
GRIN2A
GRIN2B
GRIN1
CNR1

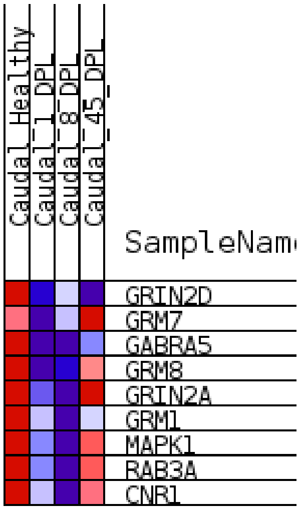
Caudal_Healthy
Caudal_1_DPL
Caudal_8_DPL
Caudal_45_DPL
SampleNam
GRIN2D
GRM7
GABRA5
GRM8
GRIN2A
GRM1
MAPK1
RAB3A
CNR1

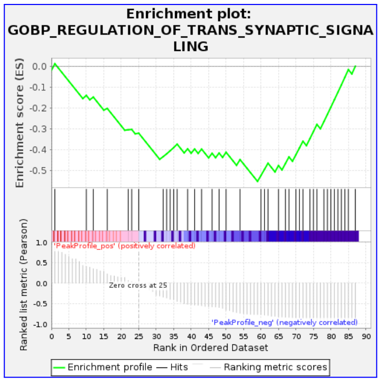
Enrichment plot:
GOBP_REGULATION_OF_TRANS_SYNAPTIC_SIGNALING
Enrichment score (ES)
Ranked list metric (Pearson)
'PeakProfile_pos' (positively correlated)
Zero cross at 25
'PeakProfile_neg' (negatively correlated)
Rank in Ordered Dataset
Enrichment profile — Hits — Ranking metric scores

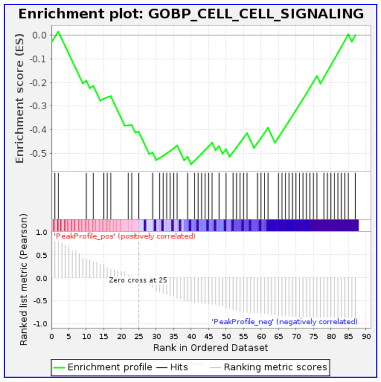
Enrichment plot: GOBP_CELL_CELL_SIGNALING
Enrichment score (ES)
Ranked list metric (Pearson)
'PeakProfile_pos' (positively correlated)
Zero cross at 25
'PeakProfile_neg' (negatively correlated)
Rank in Ordered Dataset
Enrichment profile — Hits — Ranking metric scores

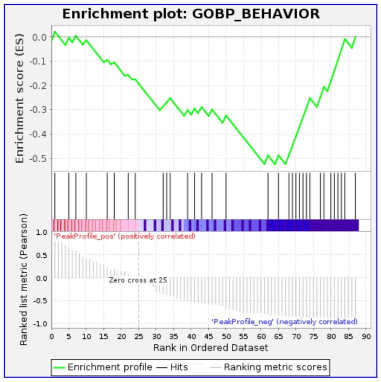
Enrichment plot: GOBP_BEHAVIOR
Enrichment score (ES)
Ranked list metric (Pearson)
'PeakProfile_pos' (positively correlated)
Zero cross at 25
'PeakProfile_neg' (negatively correlated)
Rank in Ordered Dataset
Enrichment profile — Hits — Ranking metric scores

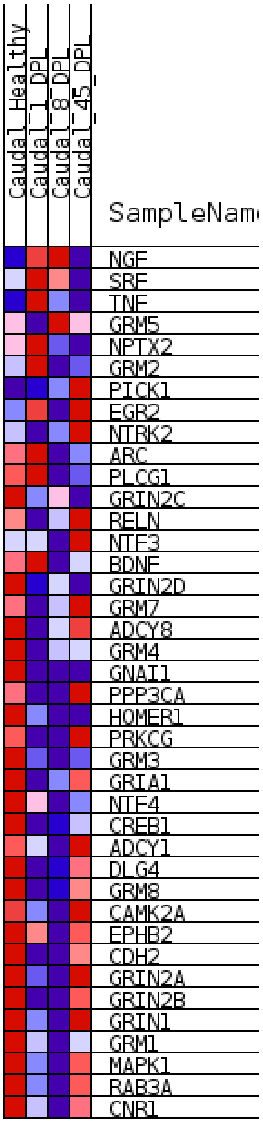
Caudal_Healthy
Caudal_1_DPL
Caudal_8_DPL
Caudal_45_DPL
NGF
SRF
TNF
GRM5
NPTX2
GRM2
PICK1
EGR2
NTRK2
ARC
PLCG1
GRIN2C
RELN
NTF3
BDNF
GRIN2D
GRM7
ADCY8
GRM4
GNAI1
PPP3CA
HOMER1
PRKCG
GRM3
GRIA1
NTF4
CREB1
ADCY1
DLG4
GRM8
CAMK2A
EPHB2
CDH2
GRIN2A
GRIN2B
GRIN1
GRM1
MAPK1
RAB3A
CNR1

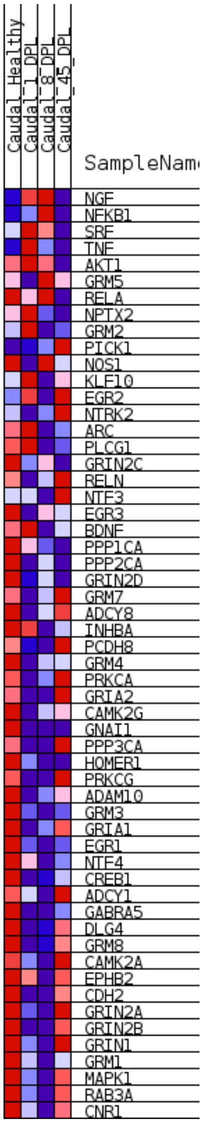
Caudal_Healthy
Caudal_1_DPL
Caudal_8_DPL
Caudal_45_DPL
NGF
NFKB1
SRF
TNF
AKT1
GRM5
RELA
NPTX2
GRM2
PICK1
NOS1
KLF10
EGR2
NTRK2
ARC
PLCG1
GRIN2C
RELN
NTF3
EGR3
BDNF
PPP1CA
PPP2CA
GRIN2D
GRM7
ADCY8
INHBA
PCDH8
GRM4
PRKCA
GRIA2
CAMK2G
GNAI1
PPP3CA
HOMER1
PRKCG
ADAM10
GRM3
GRIA1
EGR1
NTF4
CREB1
ADCY1
GABRA5
DLG4
GRM8
CAMK2A
EPHB2
CDH2
GRIN2A
GRIN2B
GRIN1
GRM1
MAPK1
RAB3A
CNR1

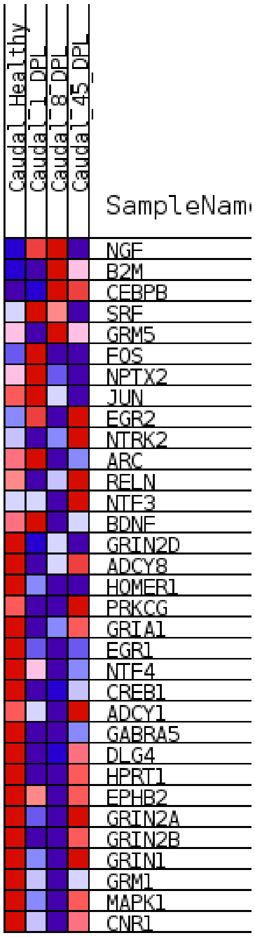
Caudal_Healthy
Caudal_1_DPL
Caudal_8_DPL
Caudal_45_DPL
NGF
B2M
CEBPB
SRF
GRM5
FOS
NPTX2
JUN
EGR2
NTRK2
ARC
RELN
NTF3
BDNF
GRIN2D
ADCY8
HOMER1
PRKCG
GRIA1
EGR1
NTF4
CREB1
ADCY1
GABRA5
DLG4
HPRT1
EPHB2
GRIN2A
GRIN2B
GRIN1
GRM1
MAPK1
CNR1

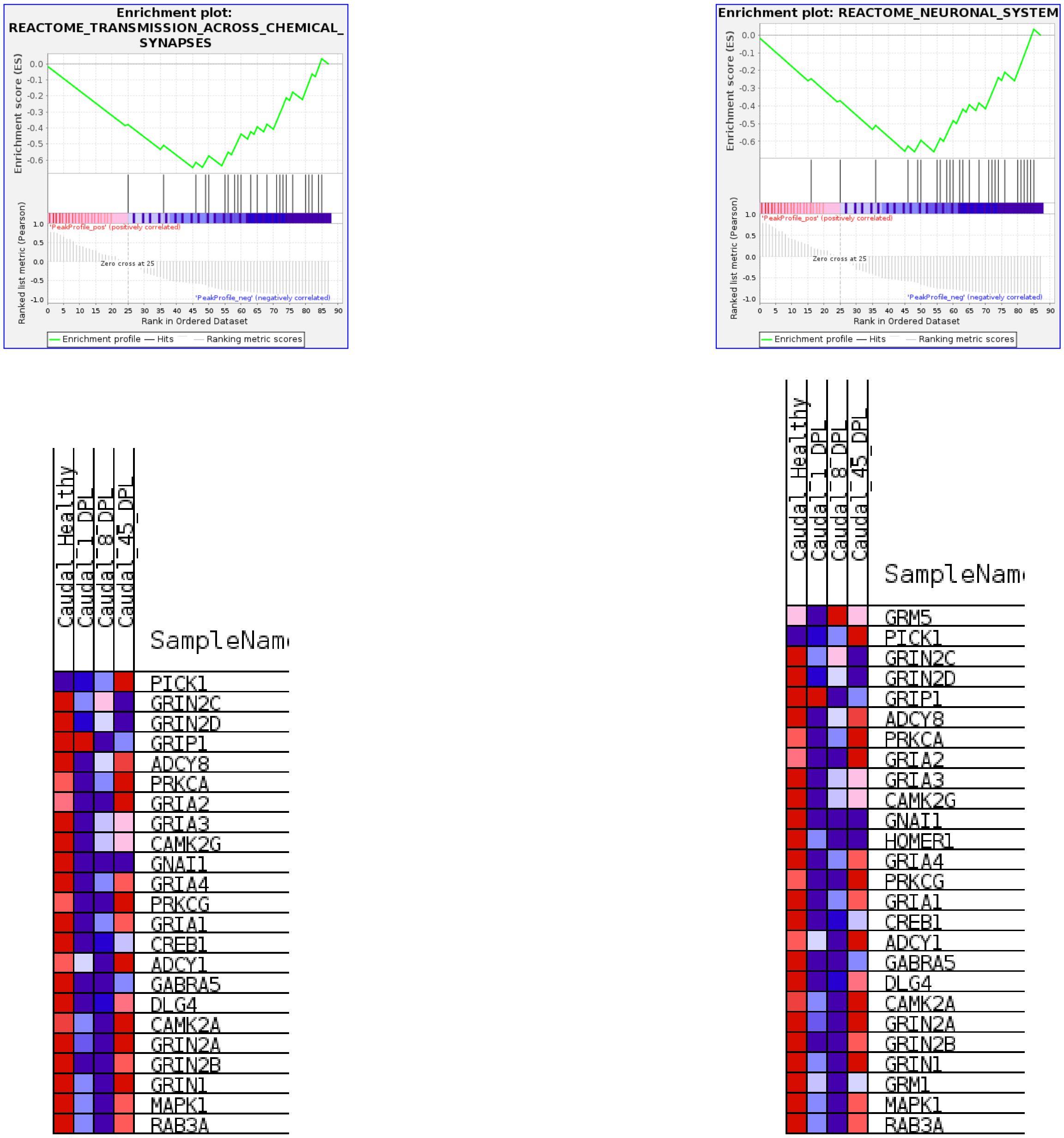

Figure S25: Enrichment plots (upper) and corresponding gene set Heatmaps (lower) of SC caudal 8 DPL peak deregulated pathways (FDR ≤ 0.1). Up-regulated pathways are framed in red, down-regulated in blue.

2.4.6 CTX-M 1 DPL Peak Pathways

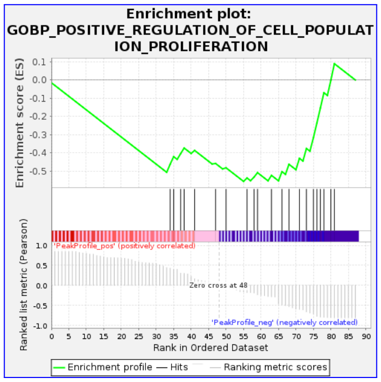

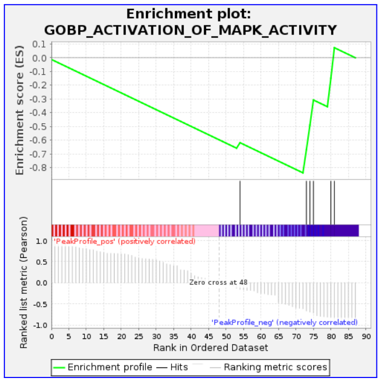

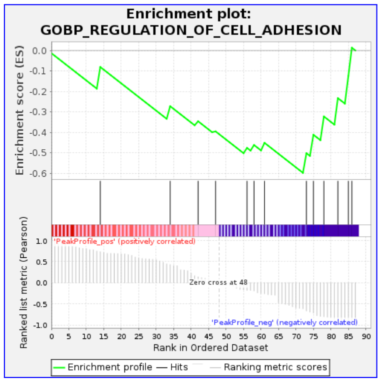

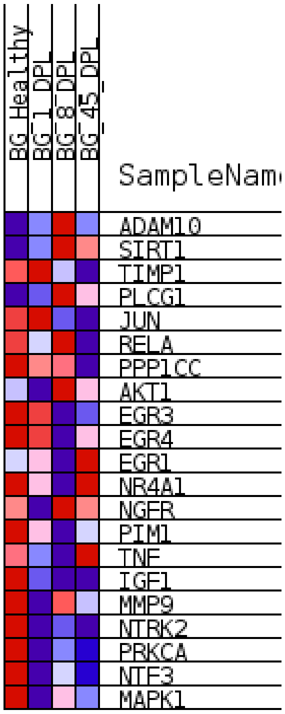

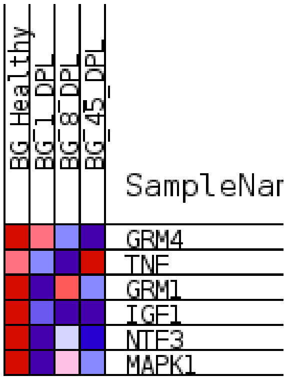

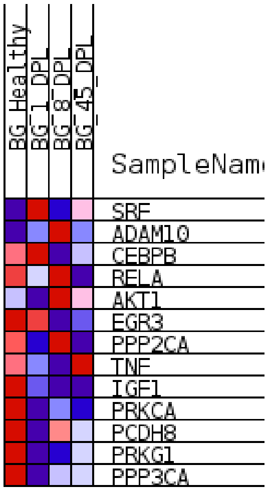

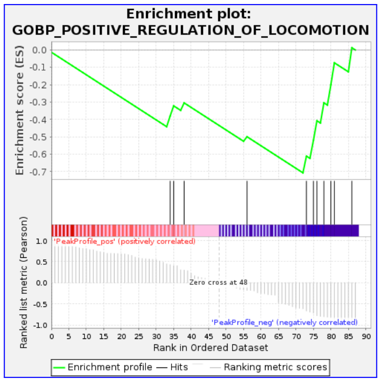
Enrichment plot:
GOBP_POSITIVE_REGULATION_OF_LOCOMOTION
Enrichment score (ES)
Ranked list metric (Pearson)
'PeakProfile_pos' (positively correlated)
Zero cross at 48
'PeakProfile_neg' (negatively correlated)
Rank in Ordered Dataset
Enrichment profile — Hits — Ranking metric scores

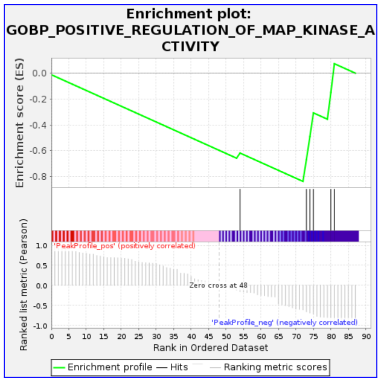
Enrichment plot:
GOBP_POSITIVE_REGULATION_OF_MAP_KINASE_A
CTIVITY
Enrichment score (ES)
Ranked list metric (Pearson)
'PeakProfile_pos' (positively correlated)
Zero cross at 48
'PeakProfile_neg' (negatively correlated)
Rank in Ordered Dataset
Enrichment profile — Hits — Ranking metric scores

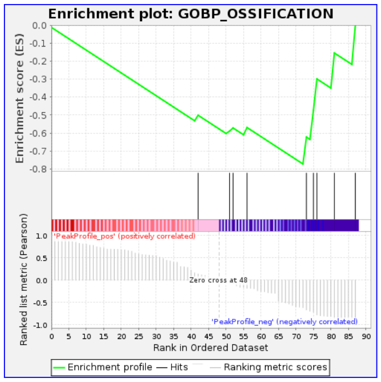
Enrichment plot: GOBP_OSSIFICATION
Enrichment score (ES)
Ranked list metric (Pearson)
'PeakProfile_pos' (positively correlated)
Zero cross at 48
'PeakProfile_neg' (negatively correlated)
Rank in Ordered Dataset
Enrichment profile — Hits — Ranking metric scores

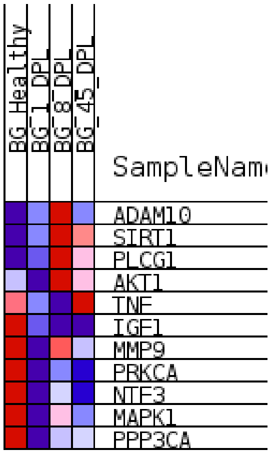
BG_Healthy
BG_1_DPL
BG_8_DPL
BG_45_DPL
SampleNam
ADAM10
SIRT1
PLCG1
AKT1
TNF
IGF1
MMP9
PRKCA
NTF3
MAPK1
PPP3CA

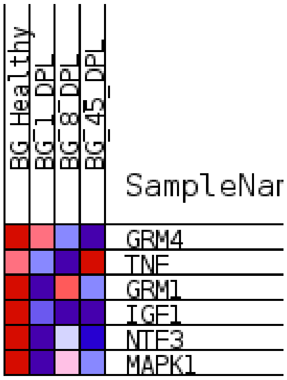
BG_Healthy
BG_1_DPL
BG_8_DPL
BG_45_DPL
SampleNar
GRM4
TNF
GRM1
IGF1
NTF3
MAPK1

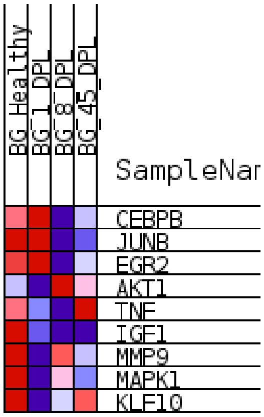
BG_Healthy
BG_1_DPL
BG_8_DPL
BG_45_DPL
SampleNar
CEBPB
JUNB
EGR2
AKT1
TNF
IGF1
MMP9
MAPK1
KLF10

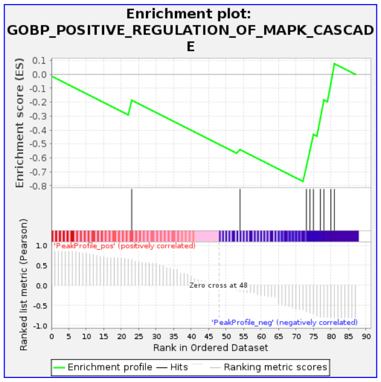
Enrichment plot:
GOBP_POSITIVE_REGULATION_OF_MAPK_CASCAD
E
Enrichment score (ES)
Ranked list metric (Pearson)
'PeakProfile_pos' (positively correlated)
Zero cross at 48
'PeakProfile_neg' (negatively correlated)
Rank in Ordered Dataset
Enrichment profile — Hits — Ranking metric scores

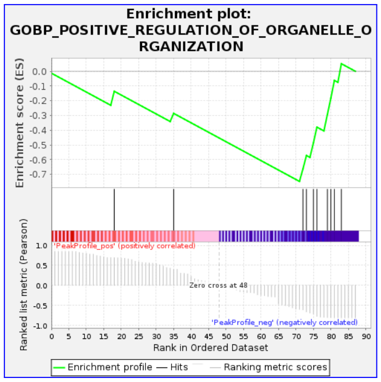
Enrichment plot:
GOBP_POSITIVE_REGULATION_OF_ORGANELLE_O
RGANIZATION
Enrichment score (ES)
Ranked list metric (Pearson)
'PeakProfile_pos' (positively correlated)
Zero cross at 48
'PeakProfile_neg' (negatively correlated)
Rank in Ordered Dataset
Enrichment profile — Hits — Ranking metric scores

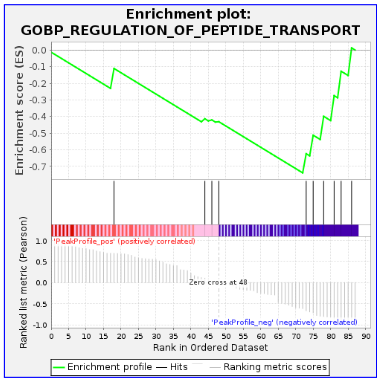
Enrichment plot:
GOBP_REGULATION_OF_PEPTIDE_TRANSPORT
Enrichment score (ES)
Ranked list metric (Pearson)
'PeakProfile_pos' (positively correlated)
Zero cross at 48
'PeakProfile_neg' (negatively correlated)
Rank in Ordered Dataset
Enrichment profile — Hits — Ranking metric scores

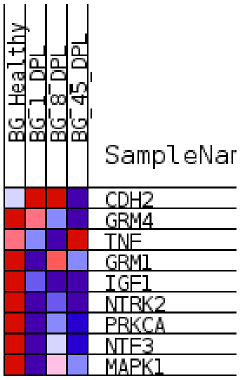
BG_Healthy
BG_1_DPL
BG_8_DPL
BG_45_DPL
SampleNar
CDH2
GRM4
TNF
GRM1
IGF1
NTRK2
PRKCA
NTF3
MAPK1

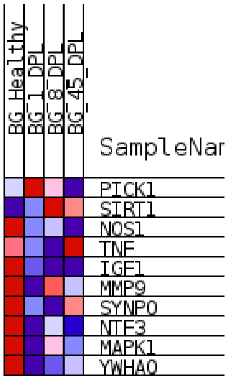
BG_Healthy
BG_1_DPL
BG_8_DPL
BG_45_DPL
SampleNar
PICK1
SIRT1
NOS1
TNF
IGF1
MMP9
SYNPO
NTF3
MAPK1
YWHAQ

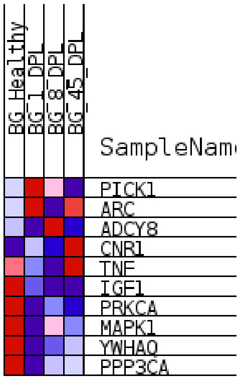
BG_Healthy
BG_1_DPL
BG_8_DPL
BG_45_DPL
SampleNam
PICK1
ARC
ADCY8
CNR1
TNF
IGF1
PRKCA
MAPK1
YWHAQ
PPP3CA

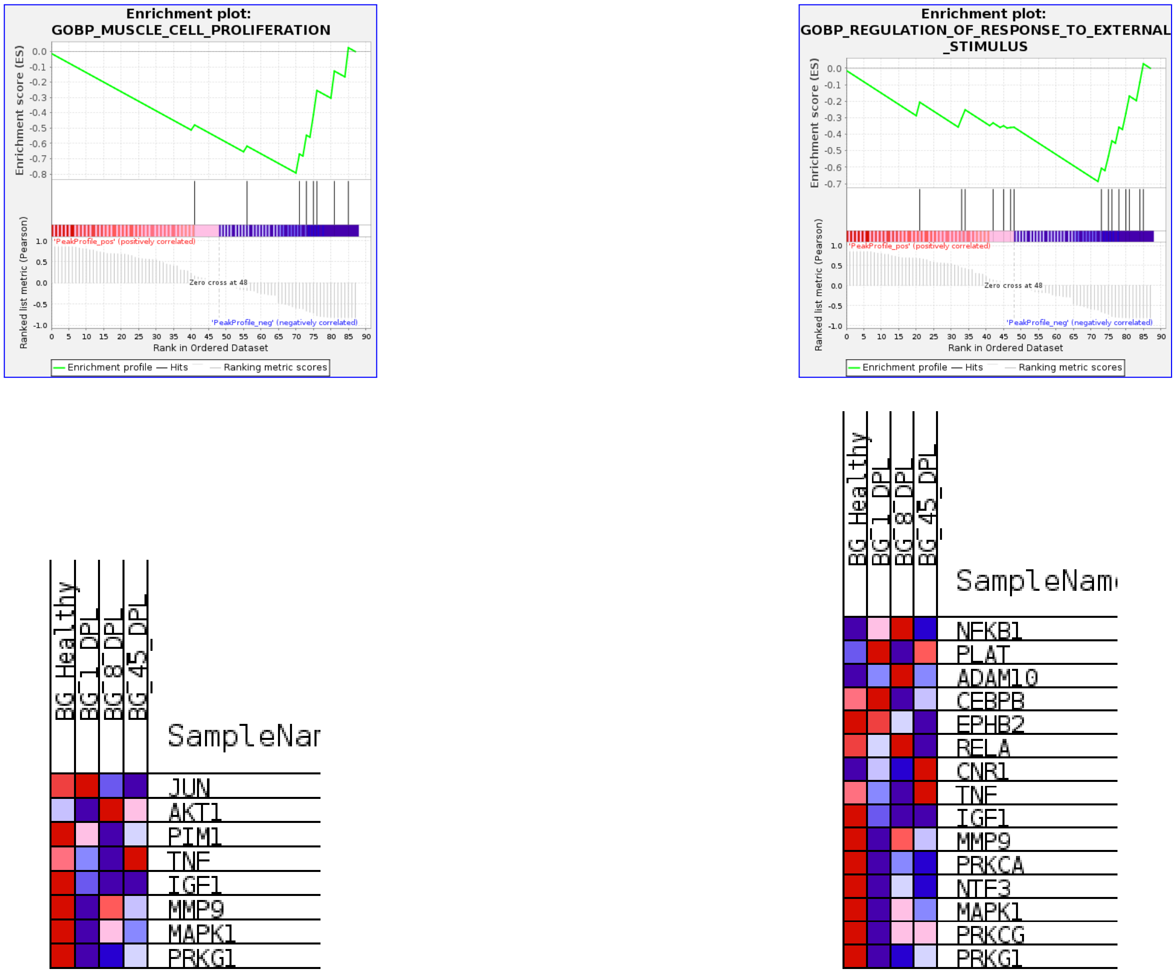

Figure S26: Enrichment plots (upper) and corresponding gene set Heatmaps (lower) of CTX-M 1 DPL peak deregulated pathways (FDR ≤ 0.1). Up-regulated pathways are framed in red, down-regulated in blue.

2.4.7 CTX-M 8 DPL Peak Pathways

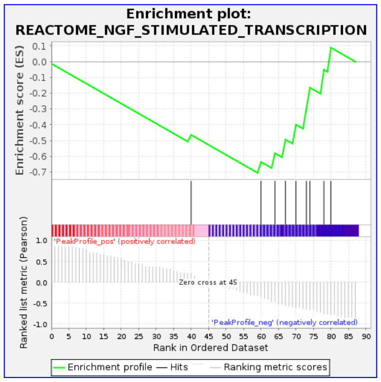

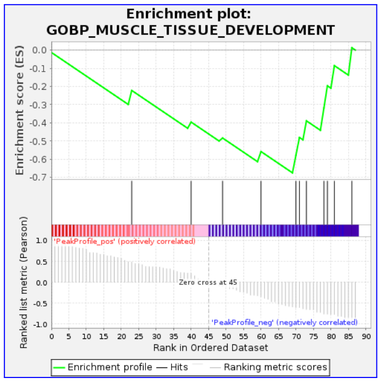

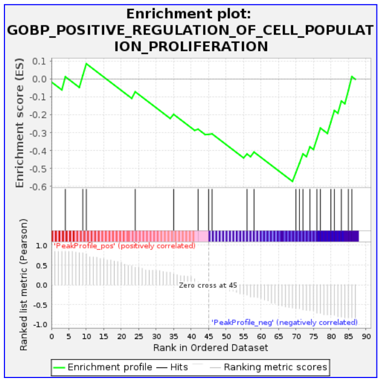

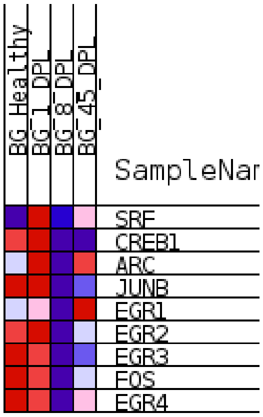

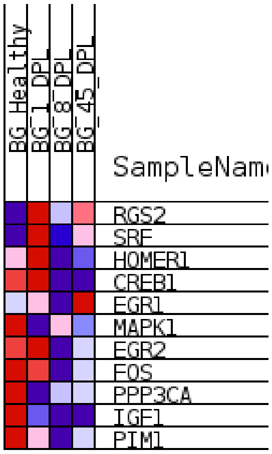

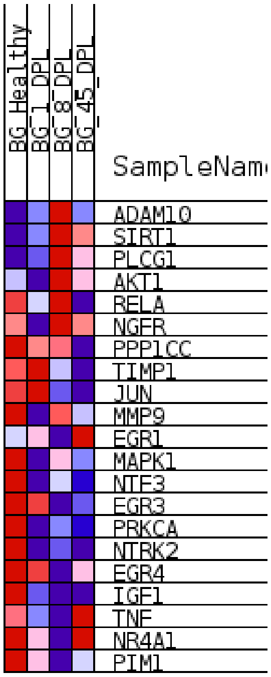

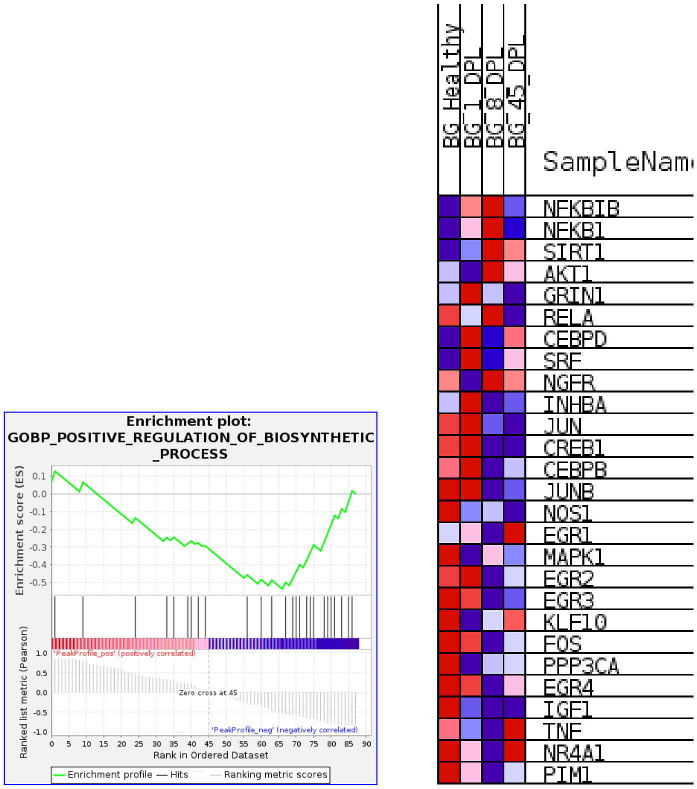

Figure S27: Enrichment plots (upper) and corresponding gene set Heatmaps (lower) of CTX-M 8 DPL peak deregulated pathways (FDR ≤ 0.1). Up-regulated pathways are framed in red, down-regulated in blue.

2.4.8 BG 45 DPL Peak Pathways

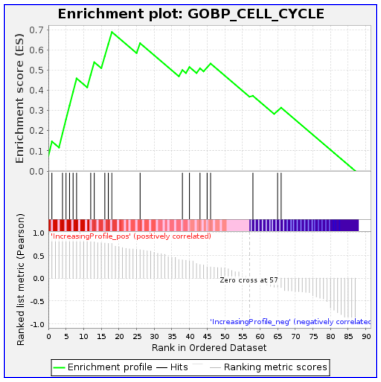

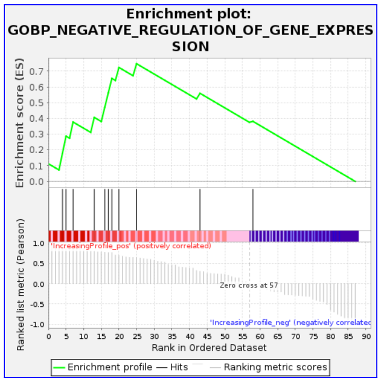

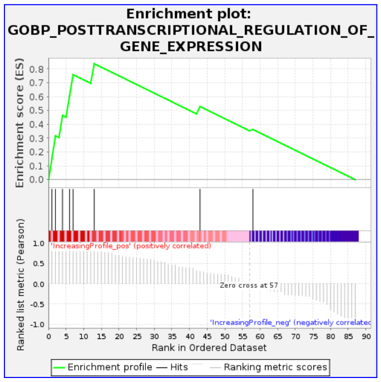

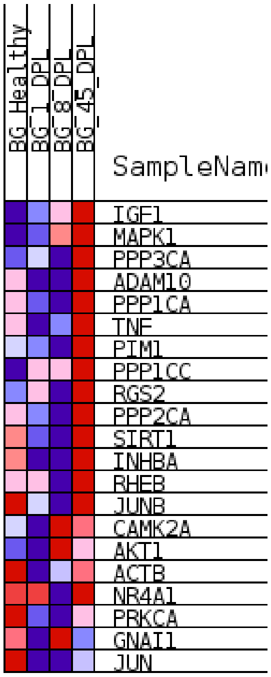

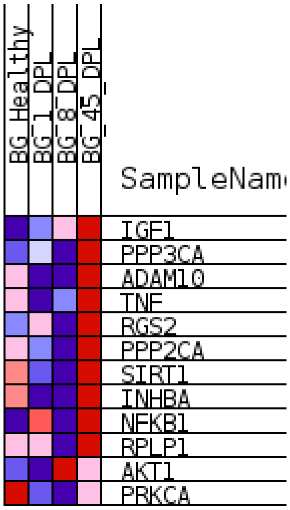

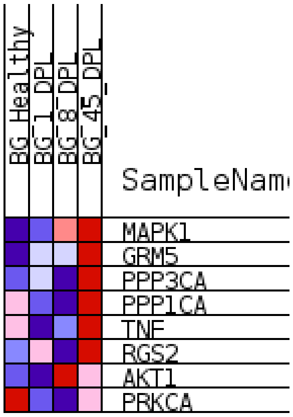

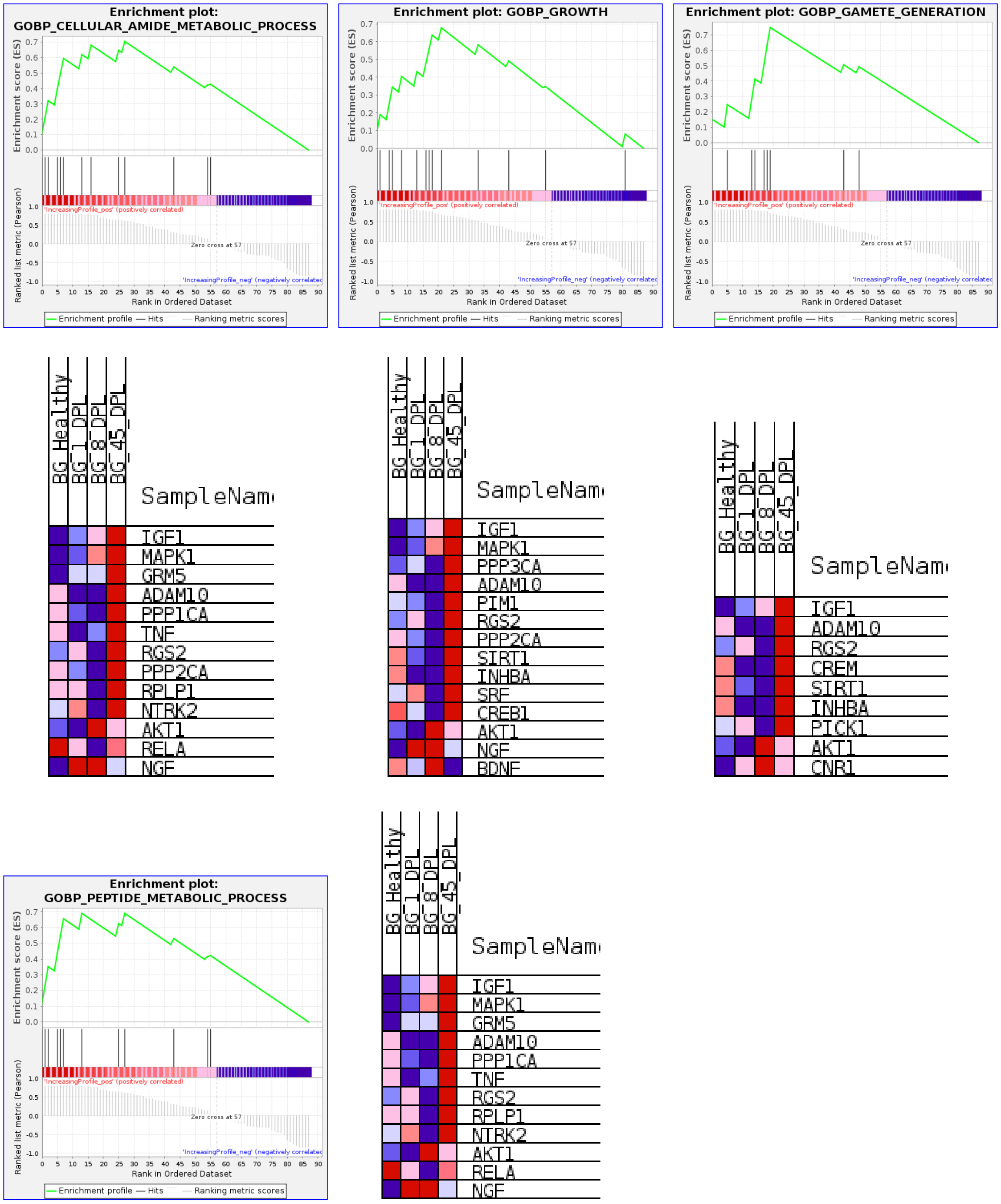

Figure S28: Enrichment plots (upper) and corresponding gene set Heatmaps (lower) of BG 45 DPL peak deregulated pathways (FDR ≤ 0.1). Up-regulated pathways are framed in red, down-regulated in blue.

2.4.9 CB 8 DPL Pathways

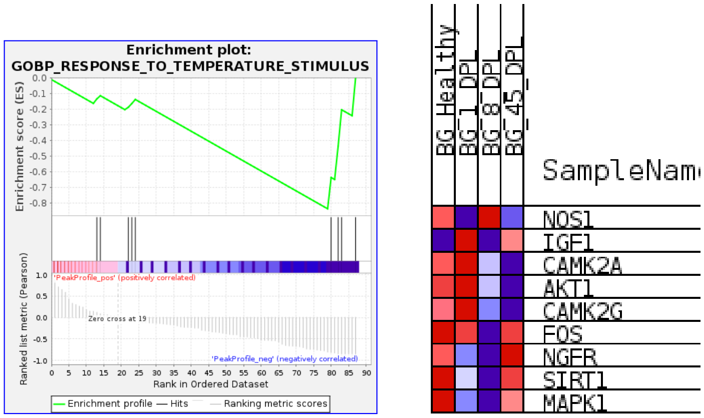

Figure S29: Enrichment plots (upper) and corresponding gene set Heatmaps (lower) of CB 8 DPL peak deregulated pathways (FDR $\leq$ 0.1). Up-regulated pathways are framed in red, down-regulated in blue.

2.4.10 CB 45 DPL Peak Pathways

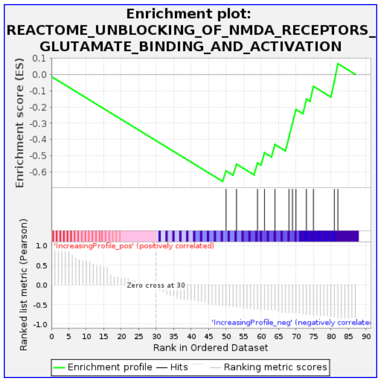

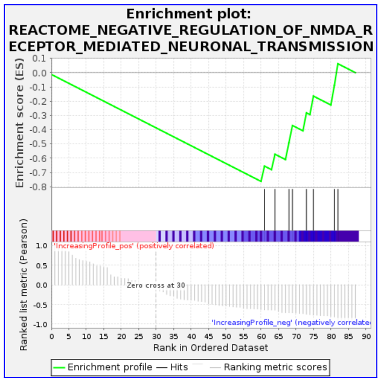

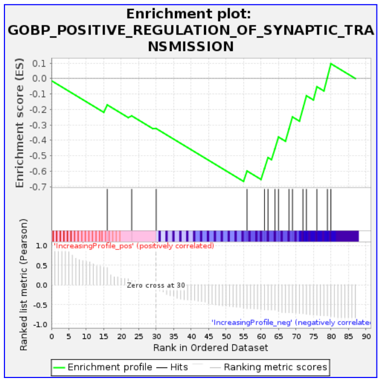

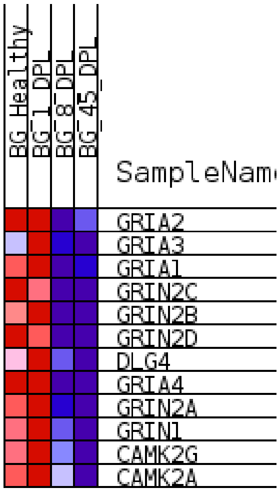

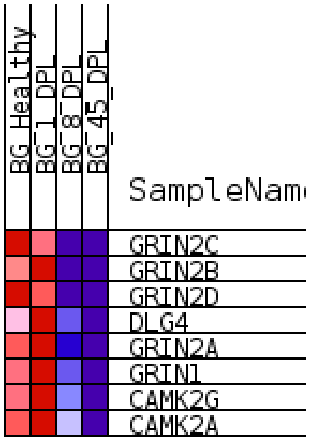

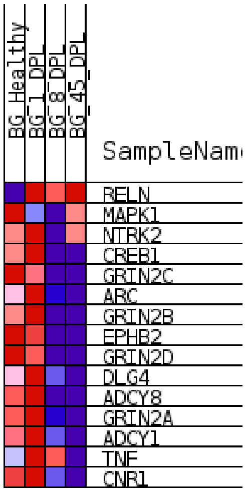

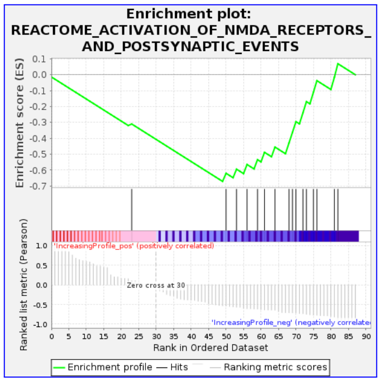

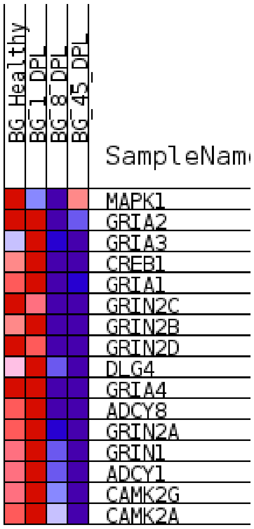

Figure S30: Enrichment plots (upper) and corresponding gene set Heatmaps (lower) of CB 45 DPL peak deregulated pathways (FDR ≤ 0.1). Up-regulated pathways are framed in red, down-regulated in blue.

List of Figures

S1 Bar plots of SC rostral segment 1, 8 and 45 DPL, Ranked Gene List Pearson Correlation Profile. . . . 310
S2 Bar plots of SC caudal segment 1, 8 and 45 DPL, Ranked Gene List Pearson Correlation Profile. . . . 310
S3 Bar plots of SC cervical segment 1, 8 and 45 DPL, Ranked Gene List Pearson Correlation Profile. . . 310
S4 Bar plots of CTX-M 1, 8 and 45 DPL, Ranked Gene List Pearson Correlation Profile. 311
S5 Bar plots of BG 1, 8 and 45 DPL, Ranked Gene List Pearson Correlation Profile. 311
S6 Bar plots of CB 1, 8 and 45 DPL, Ranked Gene List Pearson Correlation Profile. 311
S7 Plots of the distribution of SC rostral segment 1, 8 and 45 DPL, NES values. Significant pathways, FDR ≤ 0.25, are highlighted in yellow. 312
S8 Plots of the distributions of SC caudal section's 1, 8 and 45 DPL, NES values. Significant pathways, FDR ≤ 0.25, are highlighted in yellow. 312
S9 Plots of the distributions of SC cervical section's 1, 8 and 45 DPL, NES values. Significant pathways, FDR ≤ 0.25, are highlighted in yellow. 313
S10 Plots of the distributions of CTX-M 1, 8 and 45 DPL, NES values. Significant pathways, FDR ≤ 0.25, are highlighted in yellow. 313
S11 Plots of the distributions of BG 1, 8 and 45 DPL, NES values. Significant pathways, FDR ≤ 0.25, are highlighted in yellow. 313
S12 Plots of the distributions of CB 1, 8 and 45 DPL, NES values. Significant pathways, FDR ≤ 0.25, are highlighted in yellow. 314
S13 SC rostral 1 DPL Leading Edge Analysis results 315
S14 SC rostral 45 DPL Leading Edge Analysis results 315
S15 SC caudal 1 DPL Leading Edge Analysis results 315
S16 SC caudal 8 DPL Leading Edge Analysis results 315
S17 CTX-M 1 DPL Leading Edge Analysis results 316
S18 CTX-M 8 DPL Leading Edge Analysis results 316
S19 BG 45 DPL Leading Edge Analysis results 316
S20 CB 45 DPL Leading Edge Analysis results 316
S21 Enrichment plots (upper) and corresponding gene set Heatmaps (lower) of SC rostral 1 DPL peak deregulated pathways (FDR ≤ 0.1). Up-regulated pathways are framed in red, down-regulated in blue. 320
S22 Enrichment plots (upper) and corresponding gene set Heatmaps (lower) of SC rostral 8 DPL peak deregulated pathways (FDR ≤ 0.1). Up-regulated pathways are framed in red, down-regulated in blue. 321
S23 Enrichment plots (upper) and corresponding gene set Heatmaps (lower) of SC rostral 45 DPL peak deregulated pathways (FDR ≤ 0.1). Up-regulated pathways are framed in red, down-regulated in blue. 326
S24 Enrichment plots (upper) and corresponding gene set Heatmaps (lower) of SC caudal 1 DPL peak deregulated pathways (FDR ≤ 0.1). Up-regulated pathways are framed in red, down-regulated in blue. 330
S25 Enrichment plots (upper) and corresponding gene set Heatmaps (lower) of SC caudal 8 DPL peak deregulated pathways (FDR ≤ 0.1). Up-regulated pathways are framed in red, down-regulated in blue. 335
S26 Enrichment plots (upper) and corresponding gene set Heatmaps (lower) of CTX-M 1 DPL peak deregulated pathways (FDR ≤ 0.1). Up-regulated pathways are framed in red, down-regulated in blue. . . . 339
S27 Enrichment plots (upper) and corresponding gene set Heatmaps (lower) of CTX-M 8 DPL peak deregulated pathways (FDR ≤ 0.1). Up-regulated pathways are framed in red, down-regulated in blue. . . . 341
S28 Enrichment plots (upper) and corresponding gene set Heatmaps (lower) of BG 45 DPL peak deregulated pathways (FDR ≤ 0.1). Up-regulated pathways are framed in red, down-regulated in blue. . . . 343
S29 Enrichment plots (upper) and corresponding gene set Heatmaps (lower) of CB 8 DPL peak deregulated pathways (FDR ≤ 0.1). Up-regulated pathways are framed in red, down-regulated in blue. 344
S30 Enrichment plots (upper) and corresponding gene set Heatmaps (lower) of CB 45 DPL peak deregulated pathways (FDR ≤ 0.1). Up-regulated pathways are framed in red, down-regulated in blue. . . . 346

List of Tables

S1 PARN-126ZA Genes Array Table. 3
S2 SC rostral segment early (1 DPL peak) profile GSEA results. 7
S3 SC rostral segment early (8 DPL peak) profile GSEA results. 24
S4 SC rostral segment late (45 DPL peak) profile GSEA results. 41
S5 SC caudal segment early (1 DPL peak) profile GSEA results. 58

S6 SC caudal segment early (8 DPL peak) profile GSEA results. 75
S7 SC caudal segment late (45 DPL peak) profile GSEA results. 92
S8 SC cervical segment early profile (1 DPL peak) GSEA results. 109
S9 SC cervical segment early profile (8 DPL peak) GSEA results. 126
S10 SC cervical segment late profile Profile (45 DPL peak) GSEA results. 143
S11 CTX-M early profile (1 DPL peak) GSEA results. 157
S12 CTX-M early profile (8 DPL peak) GSEA results. 174
S13 CTX-M late profile (45 DPL peak) GSEA results. 191
S14 BG early profile (1 DPL peak) GSEA results. 208
S15 BG early profile (8 DPL peak) GSEA results. 225
S16 BG late profile (45 DPL peak) GSEA results. 242
S17 CB early profile (1 DPL peak) GSEA results. 259
S18 CB early profile (8 DPL peak) GSEA results. 276
S19 CB late profile (45 DPL peak) GSEA results. 293
